# *Parabacteroides distasonis* uses dietary inulin to suppress NASH via its metabolite pentadecanoic acid

**DOI:** 10.1038/s41564-023-01418-7

**Published:** 2023-06-29

**Authors:** Wenchao Wei, Chi Chun Wong, Zhongjun Jia, Weixin Liu, Changan Liu, Fenfen Ji, Yasi Pan, Feixue Wang, Guoping Wang, Liuyang Zhao, Eagle S. H. Chu, Xiang Zhang, Joseph J. Y. Sung, Jun Yu

**Affiliations:** 1 Institute of Digestive Disease and Department of Medicine and Therapeutics, State Key Laboratory of Digestive Disease, Li Ka Shing Institute of Health Sciences, CUHK Shenzhen Research Institute, The Chinese University of Hong Kong, Hong Kong SAR, China; 2 State Key Laboratory of Black Soils Conservation and Utilization, Northeast Institute of Geography and Agroecology, Chinese Academy of Sciences, Changchun, China; 3 Institute of Soil Science, Chinese Academy of Science, Nanjing, China; 4 Lee Kong Chian School of Medicine, Nanyang Technological University, Singapore, Singapore

**Keywords:** Bacterial systems biology, Metagenomics

## Abstract

Non-alcoholic steatohepatitis (NASH) is the severe form of non-alcoholic fatty liver disease, and is characterized by liver inflammation and fat accumulation. Dietary interventions, such as fibre, have been shown to alleviate this metabolic disorder in mice via the gut microbiota. Here, we investigated the mechanistic role of the gut microbiota in ameliorating NASH via dietary fibre in mice. Soluble fibre inulin was found to be more effective than insoluble fibre cellulose to suppress NASH progression in mice, as shown by reduced hepatic steatosis, necro-inflammation, ballooning and fibrosis. We employed stable isotope probing to trace the incorporation of ^13^C-inulin into gut bacterial genomes and metabolites during NASH progression. Shotgun metagenome sequencing revealed that the commensal *Parabacteroides distasonis* was enriched by ^13^C-inulin. Integration of ^13^C-inulin metagenomes and metabolomes suggested that *P. distasonis* used inulin to produce pentadecanoic acid, an odd-chain fatty acid, which was confirmed in vitro and in germ-free mice. *P. distasonis* or pentadecanoic acid was protective against NASH in mice. Mechanistically, inulin, *P. distasonis* or pentadecanoic acid restored gut barrier function in NASH models, which reduced serum lipopolysaccharide and liver pro-inflammatory cytokine expression. Overall this shows that gut microbiota members can use dietary fibre to generate beneficial metabolites to suppress metabolic disease.

## Abstract (web summary)

The gut commensal *Parabacteroides distasonis* uses inulin to produce the odd-chain fatty acid pentadecanoic acid, which alleviates non-alcoholic steatohepatitis via improved barrier function in mice.

## Main

Non-alcoholic fatty liver disease (NAFLD) is a global health burden. Non-alcoholic steatohepatitis (NASH), the severe form of NAFLD, is a pathogenic condition with inflammation and hepatocyte damage in addition to steatosis. Without intervention, NASH would progress to fibrosis, cirrhosis and even hepatocellular carcinoma. It has been predicted that the prevalence of NASH and its associated mortality will double by 2030 (ref. ^1^). Strategies to prevent NAFLD or progression to NASH are urgently needed. Currently, there is no Food and Drug Administration-approved pharmacological agent for NASH. Because NASH is strongly associated with metabolic disorder and obesity, dietary modification has been suggested as one of the main strategies to prevent NASH. Supplementation of dietary fibre has been proposed for the alleviation of NAFLD owing to its beneficial effects on reducing serum triglycerides (TG), cholesterol (CHOL) and obesity^2^. Nevertheless, the mechanism underlying the action of dietary fibre remains poorly understood.

Considering that fibre is largely indigestible by host enzymes, gut microbes play a crucial role in fibre fermentation and utilization in humans. A dietary fibre-modulated gut microbiota has demonstrated beneficial effects through faecal microbiota transplantation in germ-free mice^3^. However, it remains unclear how fibre modulates gut microbiota. Fundamentally, fibre serves as a carbon source for gut microbes. Microbes either directly degrade fibre or indirectly utilize fibre-derived products released by other microbes. Previous studies have indicated that dietary fibre is beneficial for NAFLD by modulating the gut microbiota and enriching short-chain fatty acid producers^4–6^. For example, the acetate producers *Bacteroides acidifaciens* and *Blautia* were found to be increased by inulin and could prevent NAFLD/NASH progression^7^. Although studies have reported the influence of different fibres on the gut microbiota at the species level, the effects of fibre could vary depending on the type of fibre and the gut microbiota. Hence, more in-depth elucidation of fibre-induced microbiota changes and the consequential effect on gut metabolites is urgently needed. Current metagenomic strategies only profile changes in overall microbiome composition, and it is unclear which gut microbial species are fostered by fibre and which bacterial metabolites are derived from fibre metabolism.

In this study, we investigated the effect of dietary fibre on the evolution of NASH. We demonstrated that fibre, in particular inulin, alleviates NASH development in different mouse models. To track dietary fibre-derived gut microbiota and metabolite alterations in NASH, we applied a stable isotope ^13^C-labelling approach for dietary fibre. DNA-stable isotope probing, a technique used to label metabolically active microbes in environmental samples, was combined with metagenomic sequencing of stool to enable quantitative capture of ^13^C fibre-utilizing, potentially beneficial gut bacteria. In addition, we tacked the flow of ^13^C-labelled metabolites synthesized directly via the microbial fermentation of fibre. We discovered that inulin protects against NASH development by promoting the enrichment of potential beneficial bacteria and the depletion of potential pathogenic bacteria. Inulin could also contribute to the biosynthesis of beneficial metabolites and the restoration of gut barrier function, thereby suppressing pro-inflammatory signalling in the liver. Collectively, we revealed that the soluble fibre inulin ameliorates NASH by directly modulating gut microbiota and metabolites.

## Results

### Inulin ameliorates NASH in two mouse models of disease

**Fig. 1:**
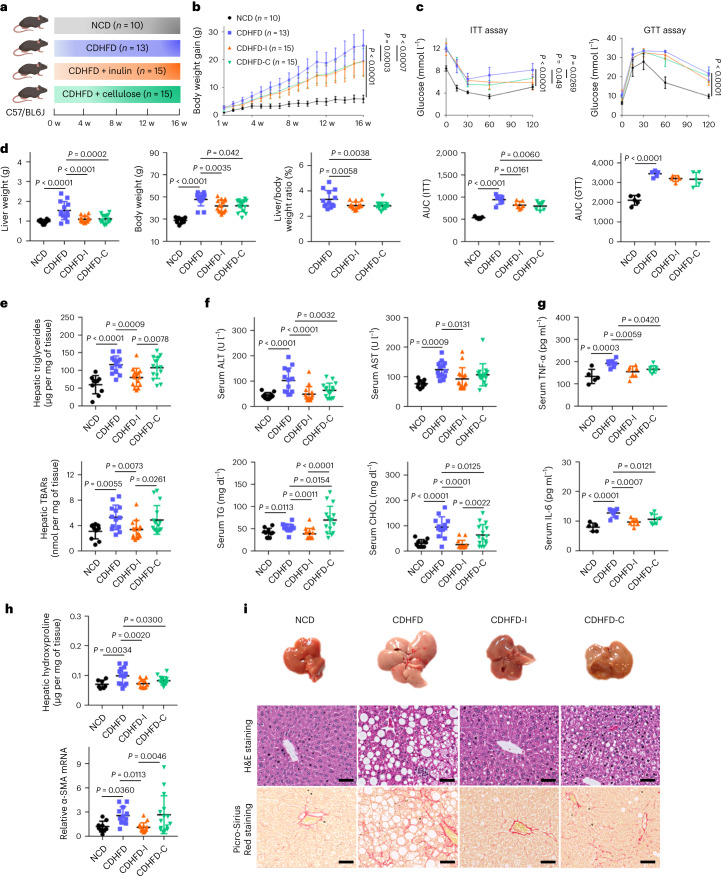
Inulin ameliorated CDHFD-induced NASH in mice. **a**, Study design of the CDHFD-induced NASH model. Created with BioRender.com. **b**, Body weight curve under different treatments. Data are presented as the mean of biological replicates ± s.d. *P* value obtained by two-way ANOVA with Fisher’s LSD test. **c**, Insulin tolerance and glucose tolerance tests. Between five and seven mice were used in each group in the insulin tolerance test (ITT): NCD (*n* = 5), CDHFD (*n* = 6), CDHFD-I (*n* = 7) and CDHFD-C (*n* = 7). Five mice were used in each group in the glucose tolerance test (GTT). Data are presented as the mean of biological replicates ± s.d. *P* value obtained by two-way ANOVA with Fisher’s LSD test for the growth curve, or one-way ANOVA with Fisher’s LSD test for area under the curve. **d**, Liver weight, body weight and liver-to-body weight ratio. **e**, Hepatic TG and TBARs. **f**, Serum ALT, AST, TG and CHOL. **d**–**f**, Between 10 and 15 mice were used in each group: NCD (*n* = 10), CDHFD (*n* = 13), CDHFD-I (*n* = 15) and CDHFD-C (*n* = 15). **g**, Serum TNF-α and IL-6. Between five and seven mice were used in each group: NCD (*n* = 5), CDHFD (*n* = 7), CDHFD-I (*n* = 7) and CDHFD-C (*n* = 7). **h**, Hepatic hydroxyproline and α-SMA mRNA. Between 7 and 15 mice were used in each group for the hepatic hydroxyproline assay: NCD (*n* = 7), CDHFD (*n* = 13), CDHFD-I (*n* = 11) and CDHFD-C (*n* = 15). Between 9 and 15 mice were used in each group for α-SMA mRNA expression: NCD (*n* = 9), CDHFD (*n* = 12), CDHFD-I (*n* = 15) and CDHFD-C (*n* = 15). **i**, Representative morphology, haematoxylin and eosin (H&E) staining, and Picro-Sirius Red staining of the liver from mice fed NCD, CDHFD, CDHFD-I and CDHFD-C. Scale bar, 50 µm. One slide per mouse was stained. **d**–**h**, Data are presented as the mean of biological replicates ± s.d. *P* value obtained by one-way ANOVA with Fisher’s LSD method. Source data

**Extended Data Fig. 1:**
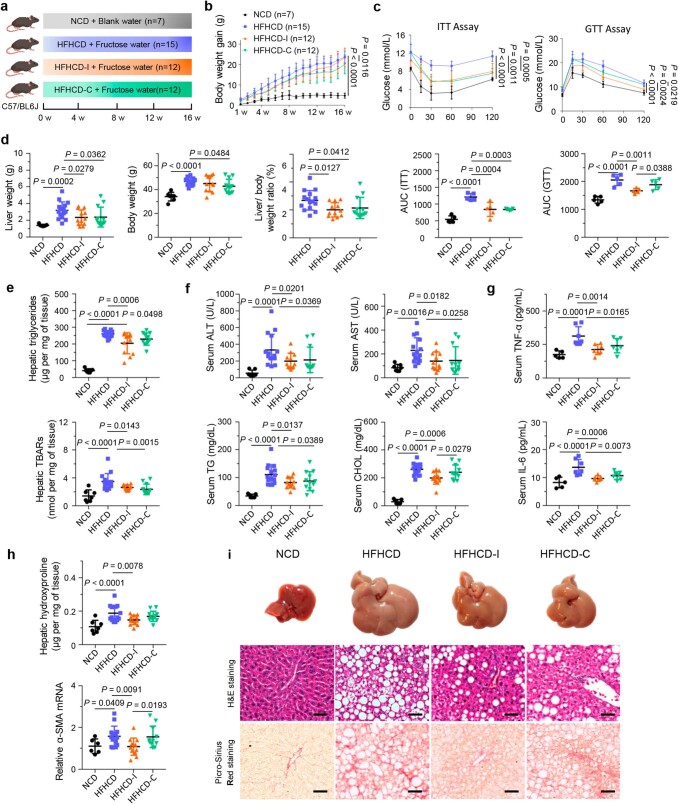
Inulin ameliorated HFHCD induced NASH in mice. **a** Study design of high fat high cholesterol diet (HFHCD) + fructose water induced NASH model (Created with BioRender.com). **b** Body weight curve under different treatments. Data are presented as mean of biological replicates ± s.d. *P* value obtained with two-way ANOVA Fisher’s LSD test. **c** Insulin tolerance and glucose tolerance tests. 5 mice were used in each group. Data are presented as mean of biological replicates ± s.d. *P* value obtained with two-way ANOVA Fisher’s LSD test for growth curve or one-way ANOVA Fisher’s LSD test for area under curve. **d** Liver weight, body weight, and the liver-to-body weight ratio. **e** Hepatic triglyceride and hepatic TBARs level. **f** Serum ALT, AST, triglyceride (TG), and cholesterol (CHOL). **d-f** 7–15 mice were used in each group, including NCD (n = 7), HFHCD (n = 15), HFHCD-I (n = 12), and HFHCD-C (n = 12). **g** Serum tumour necrosis factor alpha (TNF-α) and interleukin 6 (IL-6). 5–7 mice were used in each group, including NCD (n = 5), HFHCD (n = 7), HFHCD-I (n = 7), and HFHCD-C (n = 7). **h** Hepatic hydroxyproline and α-SMA mRNA. 7–15 mice were used in each group for hepatic hydroxyproline assay, including NCD (n = 7), HFHCD (n = 15), HFHCD-I (n = 12), and HFHCD-C (n = 12); 6–14 mice were used in each group for α-SMA mRNA expression, including NCD (n = 6), HFHCD (n = 14), HFHCD-I (n = 12), and HFHCD-C (n = 11). **i** Representative morphology, H&E staining, and Picro-Sirius Red staining of the liver from mice fed NCD, HFHCD, HFHCD-I, and HFHCD-C. scale bars, 50 µm. One slide per mouse was stained. **d-h** Data are presented as mean of biological replicates ± s.d. *P* value obtained with one-way ANOVA using Fisher’s LSD. Source data

Choline is required for lipoprotein secretion by the liver. Choline deficiency thus hinders lipid export from the liver to peripheral tissues and exacerbates NAFLD/NASH^8^. We first used a choline-deficient high-fat diet (CDHFD) to establish a NASH model in mice. Dietary fibre can be classified into soluble fibre and insoluble fibre, which are differentially utilized by gut microbes^9^. To comprehensively understand the role of fibre in NASH, we mixed CDHFD with one representative soluble fibre, inulin (CDHFD-I), and one representative insoluble fibre, cellulose (CDHFD-C) (Fig. 1a and Supplementary Tables 1 and 2). Compared with mice fed CDHFD, inulin and cellulose attenuated body weight gain (Fig. 1b), improved insulin resistance (Fig. 1c) and reduced liver weight (all *P* < 0.001), body weight (all *P* < 0.05) and liver-to-body weight ratio (all *P* < 0.01) (Fig. 1d). Inulin largely alleviated CDHFD-induced hepatic steatosis and oxidative stress, as evidenced by reduced hepatic TG (*P* = 0.0009), thiobarbituric acid-reactive substances (TBARs) (*P* = 0.0073) and Oil Red O scores (*P* < 0.0001), and was more effective than cellulose (Fig. 1e and Supplementary Fig. 1a). In addition, inulin significantly reduced serum alanine transaminase (ALT; *P* < 0.0001), aspartate transferase (AST; *P* = 0.0131), TG (*P* = 0.0011), CHOL (*P* < 0.0001) and the pro-inflammatory cytokines tumour necrosis factor-α (TNF-α; *P* = 0.0059) and interleukin-6 (IL-6; *P* = 0.0007) (Fig. 1f,g). Moreover, inulin suppressed hepatic hydroxyproline (*P* = 0.0020) and α-smooth muscle actin (α-SMA) messenger RNA levels (*P* = 0.0113) (Fig. 1h). Consistent with this, histological evaluation showed that inulin ameliorated CDHFD-induced hepatic steatosis (*P* < 0.0001), necro-inflammation (*P* < 0.0001), ballooning (*P* < 0.001) and fibrosis (*P* < 0.0001) (Fig. 1i and Supplementary Table 3). Although cellulose displayed some protective effect against NASH, it was significantly less effective than inulin in suppressing hepatic steatosis and fibrosis (*P* < 0.05) (Fig. 1i and Supplementary Table 3). This was further verified with a second NASH model induced by a high-fat, high-cholesterol diet (HFHCD) plus high-fructose drinking water (Extended Data Fig. 1, Supplementary Fig. 1 and Supplementary Tables 4 and 5). Together, inulin ameliorated diet-induced NASH in mice and its associated metabolic disorders with greater effectiveness than cellulose.

### Inulin alters the gut microbiota and is assimilated by specific bacteria

**Fig. 2:**
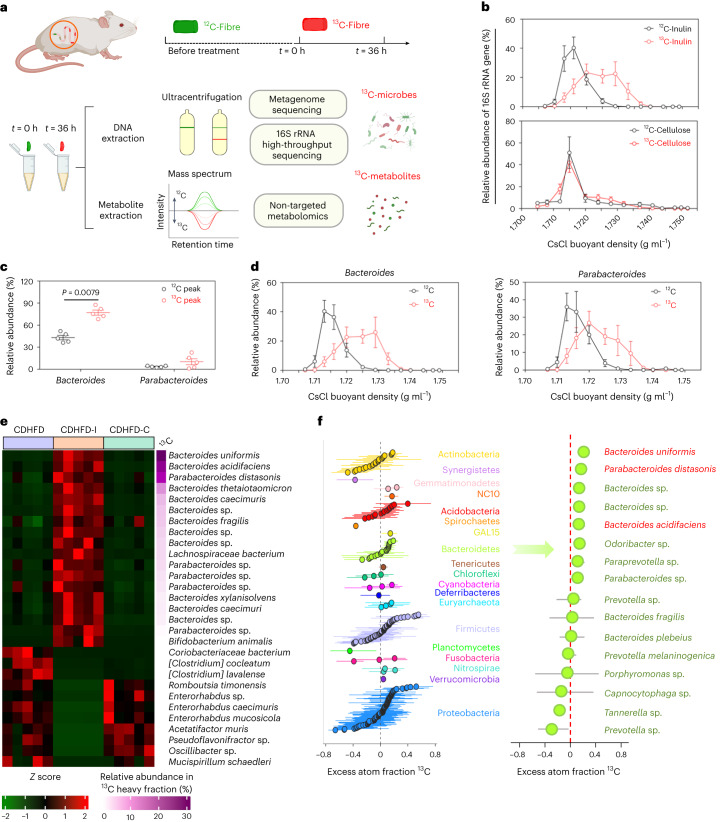
Inulin altered gut microbiota. **a**, Design and workflow of the ^13^C-labelling experiment. Mice from the CDHFD-I and CDHFD-C groups received ^13^C-fibre (^13^C-inulin or ^13^C-cellulose) in the diet for 36 h. Mouse stools were collected at 0 and 36 h. The extracted faecal DNA was fractionated by gradient density ultracentrifugation to separate ^13^C-labelled ‘heavy’ DNA, and was analysed by metagenomic and 16S rRNA sequencing. Faecal metabolites were profiled by non-targeted metabolomics analysis. Created with BioRender.com. **b**, Quantitative distribution of 16S rRNA gene from mice receiving ^13^C-inulin or ^13^C-cellulose for 36 h compared with non-labelled samples (0 h), *n* = 5 per group. **c**, Relative abundance of *Bacteroides* and *Parabacteroides* from mice fed CDHFD-^13^C-inulin for 0 h (^12^C) and 36 h (^13^C) (*n* = 5 per group), respectively. *P* value determined by two-tailed Mann–Whitney *U*-test. **d**, Relative distribution of *Bacteroides* or *Parabacteroides* across gradient densities, based on 16S rRNA sequencing abundance, normalized to total 16S rRNA expression by qPCR, *n* = 5 per group. **e**, Heatmap of different bacterial species in mice fed CDHFD, CDHFD-I or CDHFD-C. **f**, q-SIP showing the excess atom fraction of ^13^C in individual OTUs. Points are coloured according to their phylum category. Error bars represent 90% confidence interval. Key OTUs belonging to *Bacteroidetes* are presented on the right, *n* = 5 per group. **b**–**d**, Data are presented as the mean of biological replicates ± s.e.m. Source data

**Extended Data Fig. 2:**
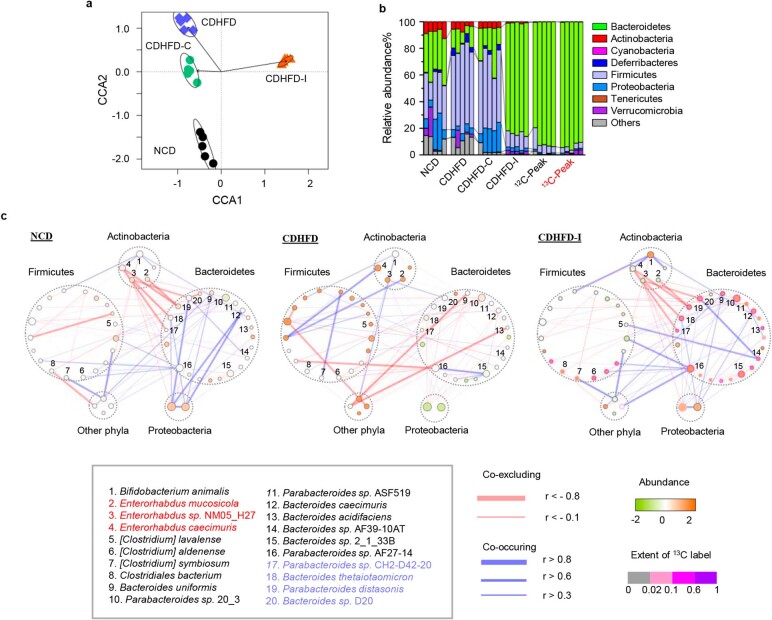
Inulin altered gut microbiota composition and ecological network. **a** Beta diversity (Constrained Correspondence Analysis) in the stool of mice fed NCD, CDHFD, CDHFD-I and CDHFD-C, respectively. **b** Phylum composition of gut microbiota from mice fed NCD, CDHFD, CDHFD-C, CDHFD-I, and peak fractions of un-labelled and labelled faecal DNA (^12^C-peak, 0 h; ^13^C-peak, 36 h). **c** Ecological network analysis among differentially abundant bacteria in NCD, CDHFD, and CDHFD-I group. Correlations were measured by SparCC. **a-c** 5 mice were used in each group.

Compared with mice fed CDHFD only, in mice administered inulin, not cellulose, gut microbiota composition was largely differentiated by highly enriched *Bacteroidetes* (Extended Data Fig. 2a,b). Utilization of dietary fibre depends on the gut microbiota^10^. To unravel the direct link between fibre and gut microbiota, we performed stable isotope labelling using ^13^C-inulin and ^13^C-cellulose in a CDHFD model (Fig. 2a). To determine the incorporation of ^13^C label into microbial genomic DNA, we performed quantitative polymerase chain reaction (qPCR) analysis of the 16S ribosomal RNA gene from DNA fractionated by density gradient ultracentrifugation (Supplementary Table 6). As shown in Fig. 2b, the majority of faecal DNA from mice that received ^13^C-inulin accumulated in the heavier gradients, whereas that from the unlabelled inulin group was mainly distributed in the lighter fractions, suggesting incorporation of ^13^C from inulin into microbial DNA. By contrast, there was no peak shift after ^13^C-cellulose labelling (Fig. 2b), consistent with the notion that cellulose could not be utilized by murine microbiota.

To identify microbes labelled by ^13^C-inulin, metagenome sequencing was performed with DNA isolated from the respective peak fractions from ^13^C-inulin and unlabelled inulin, where ultracentrifugation would separate the ^13^C-labelled ‘heavier’ DNA from ‘lighter’ DNA (Supplementary Fig. 2). *Bacteroidetes* accounted for ~90% of ^13^C-labelled bacteria (Extended Data Fig. 2b). Most species in the ^13^C heavy fraction were affiliated to *Bacteroides* (80%) and *Parabacteroides* (10%), which were enriched twofold compared with unlabelled samples (Fig. 2c), and this was further confirmed by the 16S rRNA sequencing results (Fig. 2d), implying that these genera were enriched by inulin. At a species level, *Bacteroides uniformis*, *Bacteroides acidifaciens* and *Parabacteroides distasonis* were the top three species enriched by ^13^C-inulin, but were depleted in both the CDHFD and CDHFD-C groups (Fig. 2e). Interestingly, they have been reported as depleted bacteria in patients with diabetes and could ameliorate metabolic syndrome and obesity^11–13^, implying a potential role in suppressing NASH. To consolidate our identification, the excess atom fraction of ^13^C for each operational taxonomic unit (OTU) was measured using quantitate-SIP (q-SIP) (Fig. 2f). Consistent results were obtained in which approximately half of the OTUs in the *Bacteroidetes* phylum had a positive excess atom fraction of ^13^C, mainly contributed by *Bacteroides* and *Parabacteroides* (Fig. 2f), including the top three species enriched in the ^13^C peak fraction. Although ^13^C enrichment was observed in other phyla, including *Acidobacteria*, *Firmicutes* and *Proteobacteria* (Fig. 2f), their relative abundances were reduced owing to the expansion of *Bacteroidetes*.

To investigate the impact of inulin-enriched *Bacteroides* and *Parabacteroides* on the gut microbial communities, ecological network analysis was performed. Compared with mice fed a normal chow diet (NCD), microbial co-occurring and co-excluding interactions were altered in mice fed CDHFD, but were partly rescued by inulin. Inulin also promoted new bacterial interactions compared with NCD or CDHFD groups (Extended Data Fig. 2c). In particular, we found that ^13^C-inulin-derived species, including *Parabacteroides distasonis*, *Parabacteroides* sp., *Bacteroides thetaiotaomicron* and *Bacteroides* sp., displayed strong co-excluding interactions with a cluster of depleted bacteria in the inulin group, including *Enterorhabdus mucosicola*, *Enterorhabdus* sp. and *Enterorhabdus caecimuris*, potential pathogens that are overrepresented in inflammatory conditions^14,15^. Consistent co-excluding interactions were observed in the NCD group but disrupted in the CDHFD group, suggesting that inulin-derived *Bacteroides* and *Parabacteroides* may suppress the expansion of intestinal pathogens.

### Inulin-enriched *Parabacteroides distasonis* suppresses NASH development

**Fig. 3:**
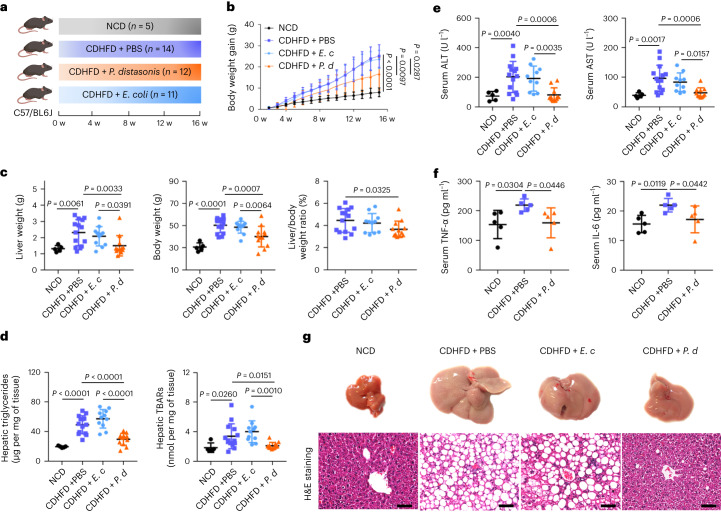
*P. distasonis* ameliorated CDHFD-induced NASH in mice. **a**, Study design of the CDHFD-induced NASH model with bacteria treatment. Created with BioRender.com. **b**, Body weight curve. **c**, Liver weight, body weight and liver-to-body weight ratio. **d**, Hepatic TG and hepatic TBARs. **b**–**d**, Between 5 and 14 mice were used in each group: NCD (*n* = 5), CDHFD + PBS (*n* = 14), CDHFD + *E. coli* (*E. c*) (n = 11) and CDHFD + *P. distasonis* (*P. d*) (*n* = 12). **e**, Serum ALT and AST. Between 5 and 14 mice were used in each group: NCD (*n* = 5), CDHFD + PBS (*n* = 14), CDHFD + *E. c* (*n* = 10) and CDHFD + *P. d* (*n* = 11). **f**, Serum TNF-α and IL-6, *n* = 5 per group. **g**, Representative morphology, haematoxylin and eosin staining of liver from mice fed NCD, CDHFD + PBS, CDHFD + *E. c* and CDHFD + *P. d*. Scale bars, 50 µm. One slide per mouse was stained. **b**–**f**, Data are presented as the mean of biological replicates ± s.d. *P* value obtained by one-way ANOVA with Fisher’s LSD. Source data

We next selected the inulin-enriched bacteria *P. distasonis* (DSM 108218) (Fig. 3a), *B. uniformis* (DSM 108148) and *B. acidifaciens* (DSM 15896) (Supplementary Fig. 3a) for validation in a CDHFD model. Administration of *P. distasonis* (Fig. 3b) reduced body weight gain (*P* = 0.0097). *P. distasonis* also reduced liver weight (*P* = 0.0033), body weight (*P* = 0.0007), liver-to-body weight ratio (*P* = 0.0325) (Fig. 3c), hepatic TG (*P* < 0.0001), TBARs (*P* = 0.0151), Oil Red O score (*P* = 0.0009) (Fig. 3d and Supplementary Fig. 1c), serum ALT (*P* = 0.0006) and AST (*P* = 0.0006), as well as the pro-inflammatory cytokines TNF-α (*P* = 0.0446) and IL-6 (*P* = 0.0442) (Fig. 3e,f). Notably, mice gavaged with *P. distasonis* demonstrated a significant reduction in hepatic steatosis (*P* < 0.0001) and necro-inflammation (*P* < 0.05), indicating that *P. distasonis* suppressed NASH (Fig. 3g and Supplementary Table 7). *B. uniformis* and *B. acidifaciens* showed beneficial trends without statistical significance (Supplementary Fig. 3b–f and Supplementary Table 7). *Escherichia coli*, as a negative control, had no effect. All three anaerobes administered to mice were viable (71.58%–90.07%) compared with *E. coli* (94.86%), as determined by a bacterial viability kit (Supplementary Fig. 4). Concordantly, *P. distasonis* was significantly enriched in healthy subjects compared with those that had type 2 diabetes from our previous study, although this cohort is limited by the low number of patients with type 2 diabetes (*n* = 14) and healthy controls (*n* = 91) (Supplementary Fig. 5)^16^, consistent with its role as a beneficial commensal bacterium. Thus, inulin-derived *P. distasonis* exerts a beneficial effect in the prevention of NASH.

### Inulin is bio-transformed by gut microbiota into beneficial metabolites

**Extended Data Fig. 3:**
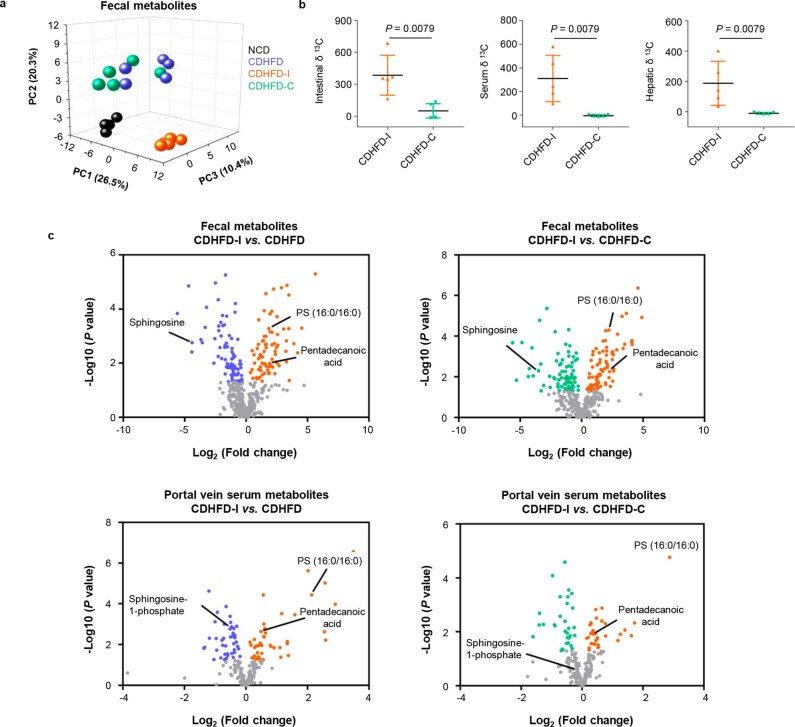
Inulin altered gut metabolomics. **a** Principal component analysis (PCA) of faecal metabolites from mice fed NCD, CDHFD, CDHFD-I, and CDHFD-C, respectively.5 mice were included in each group. **b** δ^13^C value in the intestinal tissue, serum and liver tissue of mice received ^13^C-inulin or ^13^C-cellulose (n = 5 per group) for 36 h. Data are presented as mean of biological replicates ± s.d. Statistical significance was determined by two tailed Mann-Whitney U test. **c** Volcano plots showing altered faecal or portal vein serum metabolites in CDHFD-I group compared to CDHFD group or CDHFD-C group. 5–7 mice were used in each group. *P* value was obtained by two tailed students’ *t* test.

To profile the metabolic changes, non-targeted metabolomics was conducted with mouse stool. Principal component analysis of the overall metabolite composition revealed that the CDHFD-I group was clearly separated from the CDHFD group (Extended Data Fig. 3a). By contrast, the CDHFD-C group overlapped with the CDHFD group, indicating that inulin, but not cellulose, altered gut metabolite profiles (Extended Data Fig. 3a). This was also supported by direct measurement of the ^13^C ratio using an isotope ratio mass spectrometer combined with an elemental analyser. δ^13^C levels in colon tissue, serum and liver tissue in mice that received ^13^C-inulin were substantially higher than in mice fed ^13^C-cellulose (Extended Data Fig. 3b), suggesting that inulin, but not cellulose, was metabolized in vivo. Hence, inulin might modulate gut microbiota and metabolites to suppress NASH.

**Fig. 4:**
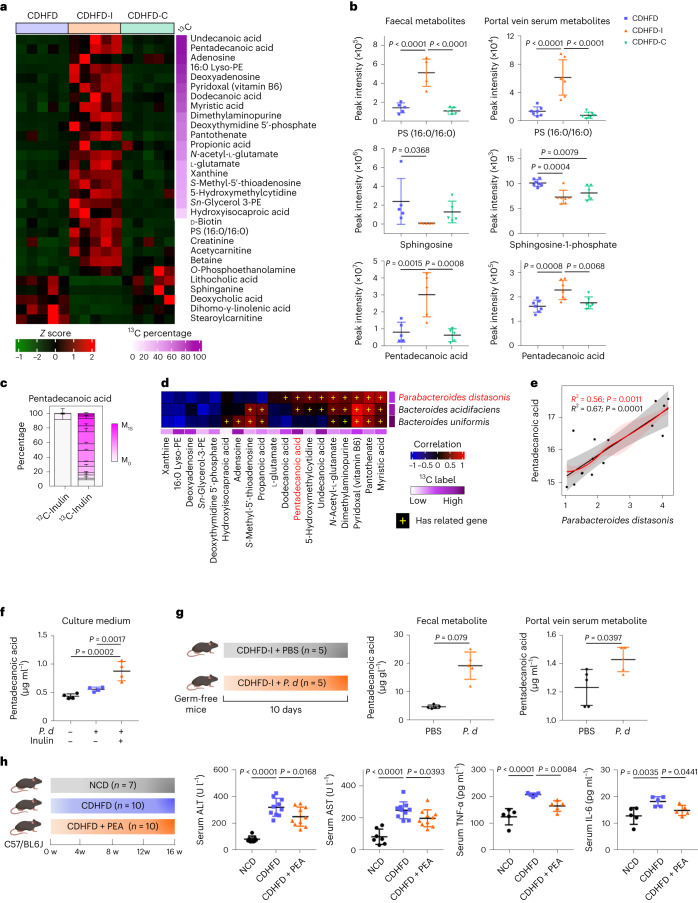
Inulin modulated gut metabolites. **a**, Heatmap of representative faecal metabolites in mice fed CDHFD, CDHFD-I and CDHFD-C. The right-hand side represents the percentage of ^13^C-labelling for each metabolite. Lyso-PE, lysophosphatidylethanolamine; PS, phosphatidylserine; *sn*-glycerol 3-PE, *sn*-glycerol 3-phosphoethanolamine. **b**, Quantification of representative metabolites in faecal (*n* = 5 per group) and portal vein serum (*n* = 6–7 per group). **c**, Isotopologue distribution of labelled pentadecanoic acid in stool from mice treated with CDHFD-I (*n* = 3), data are presented as the mean of biological replicates ± s.e.m. **d**, Correlation analysis between ^13^C-labelled bacteria and metabolites by partial Spearman’s correlation. Plus signs indicate that the bacteria expressed genes necessary for the biosynthesis of the respective metabolites. **e**, Correlation between *P. distasonis* and pentadecanoic acid abundance with (red) and without (grey) correction. Error bands indicate the 95% confidence interval. **f**, Targeted metabolomic analysis of pentadecanoic acid in blank medium or *P. distasonis* conditioned medium with or without the addition of inulin, *n* = 4 per group. **g**, Experimental design of *P. distasonis* (*P. d*) gavage in germ-free mice fed with CDHFD-I. Created with BioRender.com. Pentadecanoic acid was detected in faeces and portal vein serum, *n* = 5 per group. **h**, Effect of pentadecanoic acid (0.4%) on CDHFD diet-induced liver damage. Created with BioRender.com. Between 7 and 10 mice were used in the serum ALT and AST tests: NCD (*n* = 7), CDHFD (*n* = 10), and CDHFD + pentadecanoic acid (PEA) (*n* = 10). Five mice from each group were used for serum TNF-α and IL-6 tests. **b**, **f**–**h**, Data are presented as biological replicates ± s.d. *P* value obtained by one-way ANOVA with Fisher’s LSD. Source data

Given that our non-targeted metabolomic profiling suggested that inulin profoundly altered gut metabolites (Extended Data Fig. 3a), we next sought to identify metabolites derived from inulin. By analysing the ^13^C-labelled metabolome, we identified ^13^C-labelled metabolites, including fatty acids, nucleotides and vitamins (Fig. 4a and Supplementary Fig. 6), suggesting that inulin was utilized by gut microbes for biosynthesis. Next, we examined whether inulin-derived metabolites could be transported to the liver via the gut–liver axis. To this end, we overlapped the metabolic profiles of mouse stool and portal vein serum (Extended Data Fig. 3c). Pentadecanoic acid was the only ^13^C-inulin labelled metabolite concomitantly enriched in mouse stool as well as portal vein serum (Fig. 4b,c). Changes in non-labelled metabolite were also observed. Phosphatidylserine (16:0/16:0), a metabolite reported to be depleted in liver biopsies from NASH patients^17–20^, was enriched in stool and portal vein serum (Fig. 4b), whereas the detrimental metabolite sphingosine, a serum biomarker for NASH patients^21^, was depleted (Fig. 4b). These findings indicate that utilization of inulin by gut microbiota generates beneficial metabolites that are absorbed and then transported to the liver. Other ^13^C-labelled faecal metabolites, such as pantothenate and pyridoxal, correlated with *P. distasonis*, but were not enriched in portal vein serum. However, the limits of detection and absorption rate are potential confounding factors that hinder identification of differences in other metabolites.

### *P. distasonis*-produced pentadecanoic acid ameliorates NASH

To identify the gut microbiota–metabolite interplay, we performed a correlation analysis. As shown in Fig. 4d,e, *P.* *distasonis* was positively correlated with pentadecanoic acid, implying that it might be a downstream metabolite of *P. distasonis*. In line with our hypothesis, *P. distasonis* culture supernatant had elevated pentadecanoic acid upon addition of inulin (Fig. 4f). In germ-free mice, low levels of pentadecanoic acid (C15:0) were detected, which could be attributed to dietary intake and endogenous biosynthesis from odd-chained precursors or branched-chain amino acids^22^. Nevertheless, administration of *P. distasonis* to germ-free mice for 10 days significantly increased pentadecanoic acid levels in stool and portal vein serum (Fig. 4g). Compared with faecal samples, the smaller increase in pentadecanoic acid in the portal vein serum is probably due to absorptive loss in the gut. *P. distasonis* thus biosynthesizes pentadecanoic acid in vitro and in vivo.

Previous work implicates pentadecanoic acid as a beneficial metabolite^18,19,23^. We assessed the preventive effect of pentadecanoic acid in CDHFD-induced NASH in mice (Fig. 4h, Supplementary Fig. 7 and Supplementary Table 8). We found that pentadecanoic acid alleviated serum ALT (*P* = 0.0168), AST (*P* = 0.0393), TNF-α (*P* = 0.0084) and IL-6 (*P* = 0.0441) (Fig. 4h), as well as hepatic TBARs (*P* = 0.0017) (Supplementary Fig. 7a), thus confirming the hepaprotective effect of pentadecanoic acid in NASH.

### Inulin, *P. distasonis* or pentadecanoic acid protect against NASH

**Fig. 5:**
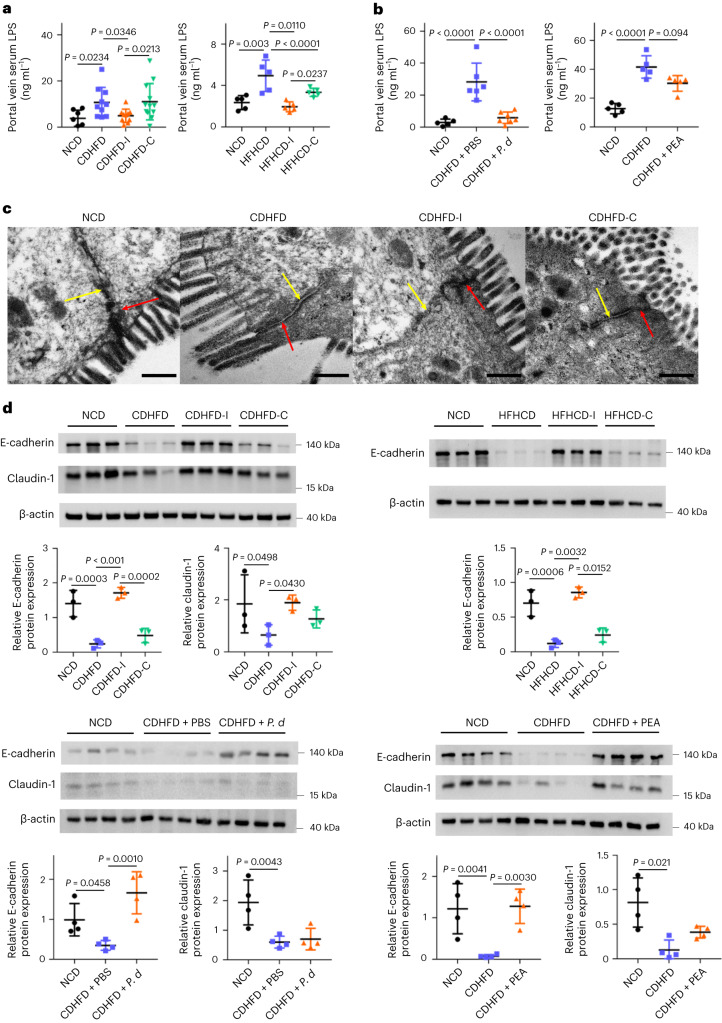
Inulin, *P. distasonis* and pentadecanoic acid restored gut barrier function. **a**, Portal vein serum LPS in CDHFD- or HFHCD-induced NASH mouse models treated with inulin or cellulose. Between 7 and 12 mice were used in each group in the first model: NCD (*n* = 7), CDHFD (*n* = 10), CDHFD-I (*n* = 10) and CDHFD-C (*n* = 12). Five mice were used in each group in the second model. **b**, Portal vein serum LPS in a CDHFD-induced NASH model treated with *P. distasonis* or pentadecanoic acid. Between five and seven mice were used in each group. **c**, Representative TEM images of the gut epithelium from mice fed NCD, CDHFD, CDHFD-I or CDHFD-C. Scale bars, 400 nm. Red and yellow arrows indicate a tight junction and adherence junction, respectively. **d**, Tight and adherence junction marker expression in NASH mouse models treated with inulin, cellulose, *P. distasonis* (*P. d*) or PEA, *n* = 3–4 per group. **a**,**b**,**d**, Data are presented as biological replicates ± s.d. *P* value obtained by one-way ANOVA with Fisher’s LSD. Source data

Dysbiosis-driven gut barrier dysfunction is an important contributor to NASH development. We therefore asked whether inulin also impacts gut barrier function in the context of NASH. We determined bacterially derived lipopolysaccharides (LPS) levels, an indicator of gut barrier function, in portal vein serum. Both CDHFD and HFHCD induced LPS levels, an effect reversed by inulin (all *P* < 0.05), *P. distasonis* (*P* < 0.0001) or pentadecanoic acid (*P* = 0.0094) (Fig. 5a,b), suggesting that inulin, *P. distasonis* or pentadecanoic acid restored gut barrier function. Consistently, scanning electron microscopy demonstrated that CDHFD compromised gut barrier function, as indicated by the loss of adherens and tight junctions, which were restored in the inulin-supplemented group (Fig. 5c). This was further confirmed with western blotting of the tight junction protein E-cadherin and the adherens protein claudin-1. Both proteins were downregulated in CDHFD-treated mice, but were rescued by inulin (Fig. 5d). In the high-fat, high-cholesterol model, E-cadherin was downregulated, an effect reversed by inulin (Fig. 5d). *P. distasonis* and pentadecanoic acid rescued E-cadherin expression in CDHFD-treated mice (Fig. 5d). By contrast, cellulose supplementation failed to restore gut barrier function (Fig. 5a–d). Our data collectively indicated that insulin, *P. distasonis* and pentadecanoic acid restored gut barrier function in diet-induced NASH, thereby limiting LPS translocation into the circulation.

**Fig. 6:**
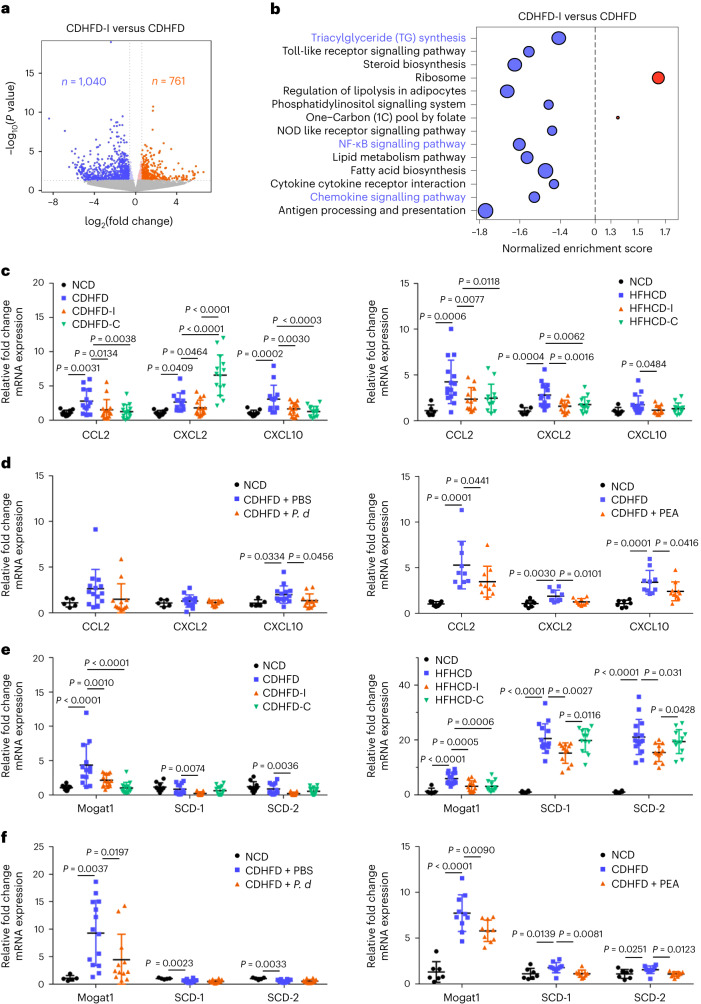
Inulin suppressed hepatic inflammation and TG synthesis pathways through enriching *P. distasonis* and pentadecanoic acid. RNA-seq analysis of liver tissues from mice fed CDHFD or CDHFD-I. **a**, Volcano plot showing differentially expressed genes. **b**, Kyoto Encyclopedia of Genes and Genomes pathway enrichment. Five mice were used in each group. *P* value determined by DESeq2. **c**,**d**, qPCR validation of pro-inflammatory cytokines in the liver tissues from mouse NASH models treated with inulin (**c**), cellulose (**c**), *P. distasonis* (*P. d*) (**d**) or PEA (**d**). **e**,**f**, qPCR validation of triacylglyceride synthesis genes in the liver tissues in mouse NASH models supplemented with inulin (**e**), cellulose (**e**), *P. d* (**f**) or PEA (**f**). **c**,**e**, Between 10 and 15 mice were used for CDHFD-induced NASH models: NCD (*n* = 10), CDHFD (*n* = 13), CDHFD-I (*n* = 15) and CDHFD-C (*n* = 14). Between 6 and 15 mice were used for HFHCD-induced NASH models: NCD (*n* = 6), HFHCD (*n* = 15), HFHCD-I (*n* = 12) and HFHCD-C (*n* = 12). **d**,**f**, Between 5 and 14 mice were used for the CDHFD treated with *P. d* experiments: NCD (*n* = 5), CDHFD + PBS (*n* = 14) and CDHFD + *P. d* (*n* = 12). Between 7 and 10 mice were used for the CDHFD treated with PEA experiments: NCD (*n* = 5), CDHFD (*n* = 10) and CDHFD + PEA (*n* = 10). **c**–**f**, Data are presented as means of biological replicates ± s.d. *P* value obtained by one-way ANOVA with Fisher’s LSD. Source data

**Extended Data Fig. 4:**
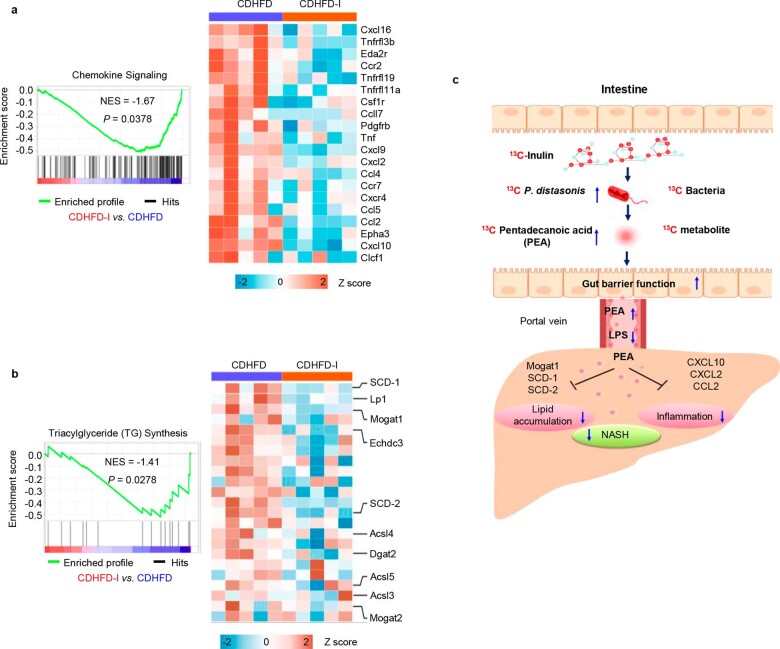
Inulin ameliorated NASH through modulating Chemokine signaling and triacylglyceride synthesis pathway. **a** Enrichment plots of chemokine signaling pathway and heatmap of differentially expressed pro-inflammatory genes. **b** Enrichment plot of triacylglyceride synthesis and heatmap of differentially expressed triacylglyceride synthesis genes. **a,b**, 5 mice were used in each group. *P* value obtained with gene set enrichment analysis. **c** The overall summary of this study. The supplementation of inulin in mouse NASH models lead to the enrichment of *P. distasonis* and pentadecanoic acid, which restores gut barrier function and downregulated NASH related regulatory pathway (Created with BioRender.com).

Having shown that inulin is protective against NASH by modulating gut microbiota and metabolites, we next investigated its molecular mechanism of action. RNA-sequencing (RNA-seq) of liver tissues from mice fed CDHFD and CDHFD-I (Fig. 6a) revealed that inulin inhibited multiple NASH-related pathways (Fig. 6b). In particular, inulin downregulated chemokine signalling, TG biosynthesis and nuclear factor kappa-light-chain-enhancer of activated B cells (NF-κB) pathways (Fig. 6b, Extended Data Fig. 4a,b and Supplementary Fig. 8). Validation by qPCR confirmed that mRNA expression of pro-inflammatory cytokines CCL2, CXCL2 and CXCL10 in liver was significantly suppressed by inulin in both the CDHFD and HFHCD model (Fig. 6c). *P. distasonis* or pentadecanoic acid treatment in the CDHFD model also suppressed expression of CCL2, CXCL2 and CXCL10 (Fig. 6d). Similarly, expression of the TG synthesis regulators *Mogat1*, *SCD-1* and *SCD-2* in the liver was downregulated by inulin in the CDHFD and HFHCD models (Fig. 6e) and suppressed by *P. distasonis* or pentadecanoic acid in the CDHFD model (Fig. 6f). By contrast, cellulose suppressed the expression of pro-inflammatory cytokines but had no effect on lipogenesis genes (Fig. 6c,f). Inulin, *P. distasonis* or pentadecanoic acid also reversed NASH-induced activation of NF-κB, a transcription factor for pro-inflammatory chemokines and lipogenesis genes^24,25^, as evidenced by RNA-seq and western blotting of phospho-p65 (Supplementary Fig. 8). Our data indicate that inulin suppressed diet-induced NASH by enriching *P. distasonis* and its protective metabolite pentadecanoic acid (Extended Data Fig. 4c).

Finally, we compared the protective effect of pentadecanoic acid with that of a control fatty acid (palmitic acid) in methionine–choline-deficient (MCD) diet-induced NASH (Supplementary Fig. 9a and Supplementary Table 9). Pentadecanoic acid, but not palmitic acid, suppressed the expression of pro-inflammatory cytokines and chemokines including TNF-α, IL-6, CCL2, CXCL2 and CXCL10, and the lipogenesis genes *SCD-1* and *SCD-2* (Supplementary Fig. 9b–d). Inulin also promotes short-chain fatty acid production by gut microbes^7^. Supplementation with acetate, a *P. distasonis*-derived short-chain fatty acid (Supplementary Fig. 9e), inhibited the mRNA expression of pro-inflammatory cytokines and chemokines, and serum ALT and AST (Supplementary Fig. 9a–f), indicating that it might act co-operatively with pentadecanoic acid to contribute to the protective effect of inulin against NASH.

## Discussion

Dietary modification is a promising strategy for ameliorating NAFLD and its progression to NASH. In this study, we systematically evaluated the protective effect of dietary fibre in the evolution of NASH in mice, and demonstrated that inulin was more effective than cellulose in alleviating NASH progression. In both dietary models, inulin administration reduced manifestations of NASH, including reduced hepatic steatosis, necro-inflammation, ballooning and fibrosis; improved liver and serum lipid profiles; and ameliorated markers of liver injury. These findings indicate that inulin is a promising dietary supplement for NASH prevention in the context of a western-style, high-calorie diet.

Given that inulin is protective against NASH as a prebiotic, we reasoned that the protective effect of inulin might be due to it modulating the gut microbiome and metabolome, which in turn protects hepatocytes against NASH. Nevertheless, studies to date have largely relied on overall microbiota composition analyses and the specific gut microbial species capable of direct utilization of inulin remains unknown. To this end, we therefore adopted a ^13^C-inulin labelling strategy to elucidate the direct contribution of inulin to gut microbiota. By treating mice with ^13^C-inulin, we tracked the assimilation of inulin into the gut microbial genome, revealing that >90% of the captured ^13^C-labelled microbial DNA was under the genera of *Bacteroides* and *Parabacteroides*. Ecological network analysis revealed that inulin-derived species under the genera of *Bacteroides* and *Parabacteroides* exerted strong co-excluding associations with potential pathogens under *Enterorhabdus* sp., which are frequently isolated from colitis patients^14,15^. Hence, the fermentation of inulin promotes the outgrowth of beneficial commensals and creates niches less favourable for bacterial pathogens. Consistent with our observations, dietary fibre has been shown to selectively enrich several species in the *Bacteroides* genus in both the human gut and gnotobiotic mice, including *B. uniformis*, *B. thetaiotaomicron* and *B. xylanisolvens*, species that were reduced in individuals with obesity^26,27^. Together, our study offers insights into how inulin might reverse gut microbial dysbiosis induced by a western-style diet and suppress NASH development.

Based on ^13^C-inulin labelling, we next identified the top three inulin-derived bacteria—*B. uniformis*, *B. acidifaciens* and *P. distasonis*. These bacteria have been reported to ameliorate obesity, metabolic syndrome, inflammation and improve insulin resistance^11,28–30^. We sought to functionally validate inulin-enriched bacteria, demonstrating that *P. distasonis* treatment effectively suppressed NASH in mice, as evidenced by the dramatic reductions in hepatic steatosis, inflammation and serum makers of NASH. This finding was supported by a previous study reporting that *P. distasonis* could ameliorate metabolomic syndrome and obesity in mice^12^. Taking advantage of ^13^C-inulin labelling in conjunction with non-targeted metabolomics, we first identified pentadecanoic acid as a key metabolite downstream of *P. distasonis*. ^13^C-labelled pentadecanoic acid was enriched in both stool and portal vein of mice after inulin treatment. Pentadecanoic acid is an odd-chain saturated fatty acid and a fermentation product of gut microbes^13^. Critically, a significant correlation was observed between ^13^C-labelled *P*. *distasonis* and pentadecanoic acid levels in mice. We further demonstrated that *P. distasonis* directly biosynthesized pentadecanoic acid in vitro and in germ-free mice. Finally, we validated pentadecanoic acid as a hepatoprotective metabolite in experimental NASH. In agreement with our results, pentadecanoic acid has been shown to suppress liver injury by restoring mitochondrial function and repressing inflammation^20,23,31^. These data suggest that inulin mediates a *P. distasonis*–pentadecanoic acid axis that alleviates NASH.

We next elucidated the events downstream of inulin-modulated microbiota and metabolites. Disruption of gut barrier function contributes to NASH pathogenesis by promoting the entry of enteric pathogens or endotoxins into the portal circulation. Connected with this, our diet-induced NASH models were associated with leaky gut, as evidenced by increased serum LPS levels and reduced expression of the tight junction proteins E-cadherin and claudin-1. By contrast, inulin, *P. distasonis* or pentadecanoic acid treatment restored gut barrier function in these models, resulting in normalized serum LPS and E-cadherin/claudin-1 expression. Restoration of gut barrier function by inulin or *P. distasonis* may, therefore, underlie their protective effects against NASH development. In the liver, hepatic lipid accumulation and pro-inflammatory signalling are the key manifestations of NASH. We revealed that inulin downregulated expression of genes involved in TG synthesis and pro-inflammatory chemokine signalling in both CDHFD- and HFHCD-induced NASH. Treatment with *P. distasonis* or pentadecanoic acid phenocopied the effects of inulin in suppressing pro-inflammatory cytokine and chemokine signalling and TG synthesis, inferring the key role of the *P. distasonis*–pentadecanoic acid axis in ameliorating NASH. Similar to our findings, dietary fibre and its fermentation metabolites have been shown to enhance gut barrier function and suppress inflammation^32–34^. Together, our work inferred that inulin-derived *P. distasonis* and its metabolite pentadecanoic acid improve gut barrier function, thereby suppressing NASH progression. In addition to *P. distasonis*, other studies have shown that inulin-enriched *Bacteroides* and *Blautia* alleviate NASH via an acetate-free fatty acid receptor 2 axis^7^. Hence, inulin-targeted intestinal bacteria exert a beneficial effect on NAFLD and NASH via generation of protective metabolites.

In summary, using diet-induced NASH models we demonstrated that dietary inulin is effective in suppressing diet-induced NASH in mice (Extended Data Fig. 4c). We provided the direct link between dietary inulin and its target bioactive intestinal bacteria *P. distasonis* and its metabolite pentadecanoic acid using ^13^C-inulin stable isotope labelling. *P. distasonis* recapitulated the beneficial effect of inulin by restoring gut barrier function and inhibiting pro-inflammatory signalling. This study advanced our understanding of the molecular basis of inulin in NASH prevention via the modulation of gut microbiota and metabolites.

## Methods

### Animal experiments

All animal experiments were approved by the Animal Experimentation Ethics Committee of the Chinese University of Hong Kong. Mice were housed in specific pathogen-free facilities under a 12 h light/12 h dark cycle. Food and water were provided ad libitum. No statistical methods were used to predetermine sample size, but our sample sizes are similar to those in previous reports^35^. Mice were randomly assigned to different groups. No animals were excluded from the analysis. Data collection and analysis were not performed in a blinded manner.

In the first model, two diet-induced NASH models were established in C57BL/6 mice. In the first model, 7–8-week-old male mice were randomly assigned to one of four groups: NCD, CDHFD, CDHFD supplemented with 10% inulin (CDHFD-I) or CDHFD with 10% cellulose (CDHFD-C) (Supplementary Tables 1 and 2).

In the second model (Supplementary Table 4), 7–8-week-old male C57BL/6 mice were randomly assigned to one of three groups: NCD, HFHCD or HFHCD supplemented with 10% inulin (HFHCD-I) or 10% cellulose (HFHCD-C). Fructose (23 g l^−1^) was added to drinking water in the HFHCD model. Mice were harvested 16 weeks after dietary intervention.

To validate the role of inulin-derived bacteria, the top species enriched in the ^13^C-fraction, *P. distasonis* (DSM 108218)*, B. acidifaciens* (DSM 15896) and *B. uniformis* (DSM 108148), were selected for validation. *E. coli* (ATCC MG1655) was used as a negative control. All strains were cultured in Wilkins–Chalgren Anaerobe Broth (Thermo Fisher Scientific, catalogue no. CM0643B) for 24 h at 37 °C. Anaerobic conditions were maintained by an anaerobic jar (Sigma-Aldrich) and anaerobic gas generator (AnaeroPack, Thermo Fisher Scientific). Bacterial cells were collected via centrifugation at 4,000*g* for 10 min. Cell pellets were resuspended in sterilized and oxygen-deprived phosphate-buffered saline (PBS) and quantified using counting chambers (Marienfeld) under a microscope. Male C57BL/6 mice (7–8 weeks old) receiving CDHFD were randomly assigned to one of five groups: PBS, *E. coli*, *P. distasonis, B. acidifaciens* *or B. uniformis*. For each strain, 5 × 10^8^ cells in 100 μl PBS was gavaged every two days. Mice were harvested after 16 weeks.

To validate the role of pentadecanoic acid, a NASH model was established by feeding 7–8-week-old male C57BL/6 mice with CDHFD or CDHFD plus 0.4% (w/w) pentadecanoic acid for 16 weeks. To compare the role of pentadecanoic acid with a control fatty acid (palmitic acid) and short-chain fatty acid (acetate), another NASH model was established by feeding mice an MCD diet (Supplementary Table 9). Eight-week-old male mice were pretreated with MCD control diet for 1 week, then randomly assigned to one of five groups: MCD control diet, MCD diet, MCD + 0.4% pentadecanoic acid, MCD + 0.4% palmitic acid or MCD + 0.4% sodium acetate. Mice were harvested after 10 days of treatment.

### In vivo ^13^C-inulin labelling

To track gut microbes capable of utilizing inulin or cellulose, uniformly (97%) labelled [U-^13^C]-inulin (Isolife, catalogue no. U-10302) or [U-^13^C]-cellulose (Isolife, catalogue no. U-10508) were provided to mice before sacrifice. In brief, a subgroup (*n* = 5) of mice from the CDHFD-I (or CDHFD-C) groups were given CDHFD incorporating 10% ^13^C-inulin or ^13^C-cellulose for 36 h. Mouse stools were collected at baseline (^12^C control) and at the end of experiment, and stored in liquid nitrogen.

### Germ-free mice

To identify whether *P. distasonis* could biosynthesize pentadecanoic acid in vivo, 8-week-old male germ-free BALB/c mice were fed CDHFD-I diet, randomly assigned to one of two groups (*n* = 5 per group) and received a single dose of *P. distasonis* (DSM 108218) or PBS via oral gavage, respectively. Mice were harvested 10 days after inoculation with *P. distasonis*. Mouse stools and portal vein serum were collected for targeted metabolomic assessments.

### Intraperitoneal glucose tolerance test

An intraperitoneal glucose tolerance test was performed using mice receiving dietary treatment for 12 weeks. Before performing the test, mice were fasted for approximately 16 h with plentiful water supplied. Each mouse was injected intraperitoneally with 10% glucose solution (1 g per kg of body weight). Tail vein blood was collected at 0, 15, 30, 60 and 120 min. Blood glucose was measured using a glucose meter (Yuwell, catalogue no. 680CR). Pressure was briefly applied to the incision using 75% ethanol wipes, to prevent further blood loss. After the experiment, mice were placed in a clean cage with water and food available and were monitored for 1 h.

### Intraperitoneal insulin tolerance test

An intraperitoneal insulin tolerance test was performed using mice receiving dietary treatment for 12 weeks. Before performing the test, mice were fasted for approximately 5 h with plentiful water supplied. Each mouse was injected intraperitoneally with 0.1 U ml^−1^ insulin (Gibco) solution (0.5 U per kg of body weight). Tail vein blood was collected at 0, 15, 30, 60 and 120 min. Blood glucose was measured using a glucose meter (Yuwell, catalogue no. 680CR). Pressure was briefly applied to the incision using 75% ethanol wipes, to prevent further blood loss. After the experiment, mice were placed in a clean cage with water and food available, and were monitored for 1 h.

### DNA extraction, gradient centrifugation and quantification

Faecal DNA was extracted using a PowerSoil DNA isolation kit (MO BIO Laboratories). To isolate ^13^C-labelled DNA, isopycnic density gradient centrifugation was performed with faecal DNA from mice that had received ^13^C-inulin or ^13^C-cellulose for 0 and 36 h, as described^36,37^. In brief, 3.0 μg of DNA was added to CsCl and adjusted to 1.725 g ml^−1^ with gradient buffer, followed by centrifugation at 45,000 r.p.m. (∼180,000 g force with the average radius of the rotor) for 44 h at 20 °C in the Vti65.2 rotor (Beckman Coulter). The density gradients were separated into 15 fractions with a NE-1000 single syringe pump (Neq Era Pump Systems). The density of each fraction was determined using an AR200 refractometer (Reichert). DNA was precipitated with 600 μl of polyethylene glycol 6000, washed with 70% ethanol and redissolved in 30 µl of ultrapure water.

### Shotgun metagenome sequencing

Shotgun metagenome sequencing was performed with faecal DNA isolated from mice fed NCD, CDHFD, CDHFD-I, CDHFD-C and ‘peak fraction’ from inulin- or ^13^C-inulin-treated samples (Supplementary Fig. 2) on an Illumina NovaSeq 6000 System (Novogene)^38^. The target sequencing depth was approximately 12 GB per sample. Peak fractions (5 ng of genomic DNA) were amplified Primer Mix 3 (Vazyme) for seven cycles, and a library was prepared using the VAHTS Universal Plus DNA Library Prep Kit for Illumina (Vazyme). Raw reads were filtered using KneadData (v.0.7.2) and assigned by the Kraken taxonomic annotation pipeline using NCBI RefSeq databases and normalized with the cumulative sum scaling method^38^. Differential analysis was achieved by a multivariable association with linear model MaAsLin2. The criteria used were: relative abundance more than 0.05%, *P* < 0.05, and fold change more than 1.5 or less than −1.5. Microbial network analysis was computed by SparCC.

### 16S rRNA sequencing

High-throughput sequencing was performed with faecal DNA from mice fed NCD, CDHFD, CDHFD-I and CDHFD-C, and all fractions from inulin- or ^13^C-inulin-treated faecal samples. An amplification library was prepared with the primer pair 341F/806R targeting 16S rRNA gene V3–V4 and sequenced on an Illumina NovaSeq 6000 System (Novogene). Data were processed by FastQC (v.0.11.9), MultiQC (v.1.9) and then analysed by QIIME2 (v.2019.4.0). Taxonomic assignment was achieved by vsearch taxonomic classifier and annotated with Greengenes reference database (release of gg-13-8-99).

### 16S rRNA gene abundance determination

16S rRNA gene abundance (V3–V4 hypervariable region) in each fraction was determined by qPCR with the following primer pair: 341F (CCTAYGGGRBGCASCAG)/806R (GGACTACHVGGGTWTCTAAT) on a CFX96 Optical Real-Time Detection System (Bio-Rad). Amplification reactions were performed in triplicate using a two-step protocol including: 3 min at 95 °C and 40 cycles consisting of 15 s at 95 °C and 1 min at 60 °C. A standard curve was performed with 10-fold dilution series of plasmids containing 16S rRNA gene (V3–V4 hypervariable region).

### Metabolomic profiling and analyses

Metabolites from mouse stool and serum were extracted using an acetonitrile/methanol/water (2:2:1, v/v/v) mixture. The extract was dried and reconstituted in 50% acetonitrile for instrument analysis. A pooled quality control sample was prepared and periodically analysed together with true samples. Non-targeted metabolomic analysis was performed by Biotree on a 1290 Infinity series UHPLC System (Agilent Technologies) coupled to a Triple time-of-flight (TOF) 6600 mass spectrometer (AB Sciex) equipped with an electrospray ionization source. Briefly, chromatographic separation was achieved using a Waters BEH Amide column (2.1 × 100 mm, 1.7 µm) at 25 °C. The mobile phase consisted of water with 25 mM ammonium acetate and 25 mM ammonium hydroxide (A) and acetonitrile (B). The flow rate was 0.5 ml min^−1^. Mass spectrometry analysis was operated in information-dependent basis mode under positive and negative modes. The 12 most abundant ions were selected, fragmented and sent to a TOF mass analyser to acquire MS2 information. Data pretreatment including peak deconvolution, alignment and integration were achieved by XCMS (v.3.2) implemented in R language. Metabolite identification was based on an in-house MS2 database. MetaboAnalyst was employed for pathway analysis. Heatmaps were generated by Complex Heatmap.

### q-SIP calculation

The calculation was conducted according to a q-SIP workflow embedded in the HTS-SIP R package^39^. The confidence interval over 90% was determined with running bootstrap replicates (*n* = 1,000), where the excess atom fraction of ^13^C was positive and non-overlapping zero were considered statistically significant.

### δ^13^C measurements

Around 10 μl of mouse serum, 10 mg of mouse colon tissue or hepatic tissue from mice that received ^13^C-inulin or ^13^C-cellulose were prepared and dried in a 60 °C oven for 48 h. Dried samples were ground to power using a sterilized pestle. Isotope analysis of the samples was performed on an isotope ratio mass spectrometer combined with an elemental analyser.

### RNA extraction, reverse transcription and sequencing

Total RNA of liver tissues from mice fed NCD, CDHFD and CDHFD-I was extracted using TRIzol reagent (Thermo Fisher Scientific). The integrity and purity of the RNA were determined using a nanodrop spectrophotometer (Thermo Fisher Scientific) and electrophoresis. Complementary DNA was obtained via a two-step reverse transcription kit (TaKaRa). RNA-seq was performed by Novogene.

### Haematoxylin and eosin staining

Tissues were embedded in paraffin wax, cut into 4-μm sections and mounted on glass slides. Sections were dewaxed in xylene and hydrated in descending ethanol solutions. Sections were stained with haematoxylin for 15 min and 1% eosin Y for 30 s, and mounted on stained and dried slides with xylene. Slides were blind scored by two investigators according to the mouse NAFLD scoring system^40,41^. The criterion for hepatic steatosis was determined on the basis of the area affected: 0 (<5%), 1 (5%–33%), 2 (33%–66%) and 3 (>66%). The criterion for necro-inflammation was based on the number of inflammation foci under a ×200 field: 0 (<0.5), 1 (0.5–1), 2 (2–4) and 3 (>4). The criterion for ballooning was based on the number of ballooning cells: 0 (none), 1 (few balloon cells), 2 (many balloon cells).

### Hepatic TG

Hepatic TG were extracted from 10% liver tissue homogenates with *tert*-butyl alcohol/methanol/Triton X-100 (3:1:1 v/v/v). The extracted lipids were quantified using a Wako E-test TG kit (Wako Pure) following the manufacturer’s protocols.

### Hepatic TBARs

Hepatic lipid peroxidation was assessed by quantifying TBAR formation. Forty-five microlitres of 10% liver homogenate was incubated with 675 μl of 20% acetic acid, 675 μl of 0.8% 2-thiobarbituric acid (Sigma-Aldrich) solution and 405 μl of distilled water for 1 h in a 95 °C water bath. The resulting solution (600 μl) was mixed with 750 μl of *n*-butanol/pyridine (15:1, v/v) and 150 μl of distilled water. The mixture was vortexed vigorously and centrifuged. The absorbance of the upper layer was measured with a spectrophotometer at 532 nm. A standard curve was generated with a series dilution of 1,1,3,3-tetramethoxypropane.

### Hydroxyproline assay

Hepatic hydroxyproline was assessed using a hydroxyproline assay kit (Jiancheng Bioengineering) according to its standard protocol.

### Oil Red O staining

Frozen tissue embedded in optimal cutting temperature compound was sectioned to 5 μm thickness under cryostat mode. Sections were mounted on glass slides and fixed in 10% formalin (Sigma-Aldrich) for 1 h. Sections were stained with 60% Oil Red O working solutions for 15 min and haematoxylin for 2 min. Cover slips were mounted on the sections using melting glycerol gelatin (Sigma-Aldrich) in a 50 °C oven. Images were taken under a microscope (Olympus IX83) and quantified by ImageJ software according to the reported protocol^42^.

### Sirius Red staining

Paraffin sections was dewaxed and hydrated. Sections were stained with haematoxylin for 10 min and Picro-Sirius Red solution for 60 min. Slides were washed twice with acidified water, dehydrated using 100% ethanol and xylene, and mounted with DPX Mountant (Sigma). Hepatic fibrosis was assessed in slides stained with Picro-Sirius Red under a ×40 field and scored according to the scoring system established for rodents^40^.

### Serum detection

Serum ALT, AST, CHOL and TG levels were determined using a Catalyst One Chemistry Analyzer (IDEXX Laboratories) following the manufacturer’s instructions. Forty microlitres of haemolysis-free serum was diluted to 120 μl with physiological saline (0.9% NaCl). Diluted serum and slides for ALT, AST, CHOL and TG detection were loaded together for automatic analysis. Serum TNF-α and IL-6 were quantified using enzyme-linked immunosorbent assay kits (Jianglai Bioengineering) according to their protocols.

### Protein extraction and quantification and western blot

Proteins from mouse colon tissues were extracted with CytoBuster reagent (Sigma-Aldrich) and quantified using a protein assay kit (Bio-Rad) according to the manufacturer’s protocols. Western blotting was performed with the following antibodies: E-cadherin (Cell Signaling Technology, catalogue no. 4A2; dilution 1:1,000), claudin-1 (Invitrogen, catalogue no. 2H10D10; dilution 1:300), β-actin (Cell Signaling Technology, catalogue no. 13E5; dilution 1:1,000), GAPDH (Cell Signaling Technology, catalogue no. D16H11; dilution 1:1,000), phospho-NF-κB p65 (Cell Signaling Technology, catalogue no. 93H1; dilution 1:1,000), NF-κB p65 (Cell Signaling Technology, catalogue no. L8F6; dilution 1:1,000) and lamin A/C (Cell Signaling Technology, catalogue no. 4C11; dilution 1:1,000). Quantification was determined using Image Lab 6.1 (Bio-Rad).

### LPS detection

LPS levels in mouse portal vein serum were determined by a Mouse LPS ELISA Kit (Cusabio) following the manufacturer’s protocols.

### TEM

Mouse colon tissues embedded in parafilm blocks were processed for TEM images. Specifically, 1-mm cubes of tissue were removed from the parafilm block, dewaxed in xylene and rehydrated in 100%, 70% and 30% ethanol. Processed tissue was further washed and soaked in cacodylate buffer supplemented with 0.1 M sucrose. Ultrathin sections were processed using a Leica Ultracut UCT and scanned using a Philips CM100 TEM.

### Culture medium for in vitro pentadecanoic acid synthesis

The culture medium used for in vitro pentadecanoic acid synthesis comprised: casitone (10 g l^−1^), yeast extract (2.5 g l^−1^), MgSO_4_*7H_2_O (45 mg l^−1^), CaCl_2_*2H_2_O (90 mg l^−1^), M9 salt (11.28 g l^−1^), l-cysteine–HCl (0.5 g l−), haemin (5 mg l^−1^) and vitamin K (0.5 mg l^−1^). Inulin (5 g l^−1^) was added when needed.

### Bacterial viability assay

The viability of bacteria before administration to mice was measured using a LIVE/DEAD BacLight Bacterial Viability Kit (Thermo Fisher Scientific, catalogue no. L7012). Briefly, bacterial pellets were collected using the same protocol as for oral gavage, and then resuspended in sterilized and oxygen-deprived NaCl (0.85%). Components A (SYTO 9 dye) and B (propidium iodide) were mixed in a 1:1 ratio, and 3 μl of the dye mixture was added to 1 ml of bacteria suspension, followed by incubation for 15 min in the dark. Stained bacteria were then loaded onto a slide and immediately observed under a confocal microscope (Leica SP8). *E. coli* served as the positive control (live bacteria) and *E. coli* treated with 75% ethanol for 1 h served as the negative control (dead bacteria).

### Statistical analysis and reproducibility

Fisher’s statistic and nonmetric multidimensional scaling were used to determine microbial beta diversity. Principal component analysis was performed to characterize metabolites similarity among groups. Comparison between two groups was determined by Student’s *t*-test or Mann–Whitney *U*-test. For comparisons involving more than two groups, one-way analysis of variance (ANOVA) with Fisher’s least significant difference (LSD) test was used. Two-way ANOVA Fisher’s LSD test was used for comparisons among growth curves. Correlation between bacteria and metabolites was performed by partial Spearman’s correlation. All statistical analyses were completed using GraphPad Prism 7.0 (GraphPad Software) and R software (v.3.5.2). Data are presented as mean ± s.d. or mean ± s.e.m. where appropriate. *P* < 0.05 was considered statistically significant. For histological evaluation, two independent experiments were repeated showing similar results.

### Reporting summary

Further information on research design is available in the Nature Portfolio Reporting Summary linked to this article.

## Supplementary information

Supplementary Information Supplementary Figs. 1–9, Tables 1–9 and images: unprocessed scans of blots and gels.

Reporting Summary

Supplementary Table 1 Non-targeted metabolomic results from mice stool or portal vein serum. Five mice were included in each group. NCD, normal chow diet; CDHFD, choline-deficient high-fat diet; CDHFD-I, CDHFD supplemented with inulin; CDHFD-C, CDHFD supplemented with cellulose.

## Data Availability

The metagenome sequencing data, 16S high-throughput sequencing data and RNA-seq sequencing data produced in this study were deposited in the public database Genome Sequence Archive (Genomics, Proteomics & Bioinformatics 2021) in the National Genomics Data Center (Nucleic Acids Res 2022), China National Center for Bioinformation/Beijing Institute of Genomics, Chinese Academy of Sciences and are publicly accessible at https://ngdc.cncb.ac.cn/gsa with accession code CRA009819, CRA009823 and CRA009820, respectively. Non-targeted metabolomic data is available at the NIH Common Fund’s National Metabolomics Data Repository (NMDR) website, the Metabolomics Workbench via its project 10.21228/M81T5C and assigned Study ID ST002520, ST002514. All data are available in the main text, extended data or supplementary materials. Source data are provided with this paper.

## Source data

Source Data Fig. 1 Statistical source data.
Source Data Fig. 2 Statistical source data.
Source Data Fig. 3 Statistical source data.
Source Data Fig. 4 Statistical source data.
Source Data Fig. 5 Statistical source data.
Source Data Fig. 6 Statistical source data.
Source Data Fig. 1 Replicate histological images.
Source Data Fig. 3 Replicate histological images.
Source Data Fig. 5 Unprocessed western blots and/or gels.
Source Data Extended Data Fig. 1 Statistical source data.
Source Data Extended Data Fig. 1 Replicate histological images.

## Code availability

All customized scripts used in this study were incorporated and uploaded to https://github.com/tree2010/1.Manuscript_nature-microbiology_2023.
