# Supplementary material for: Parabacteroides distasonis uses dietary inulin to suppress NASH via its metabolite pentadecanoic acid
Source: Nat Microbiol. 2023 Jun 29;8(8):1534–48. doi: 10.1038/s41564-023-01418-7 (PMC10390331; doi:10.1038/s41564-023-01418-7)

nature microbiology

Article

https://doi.org/10.1038/s41564-023-01418-7

Parabacteroides distasonis uses dietary inulin to suppress NASH via its metabolite pentadecanoic acid

In the format provided by the authors and unedited

List of changes

1. Remove the extra letter “C” in figure **3e** Serum AST.
2. Supplemental figure **3d**: corrected the units for hepatic triglycerides and hepatic TBARS
3. Supplemental figure **7a**: corrected the units for hepatic triglycerides and hepatic TBARS
4. Supplemental tables **3 & 5**, reformatted the size of “*”

This file contains:
Supplementary Figures 1-9
Supplementary Tables 1-9
Supplementary Images, unprocessed scans of blots and gels

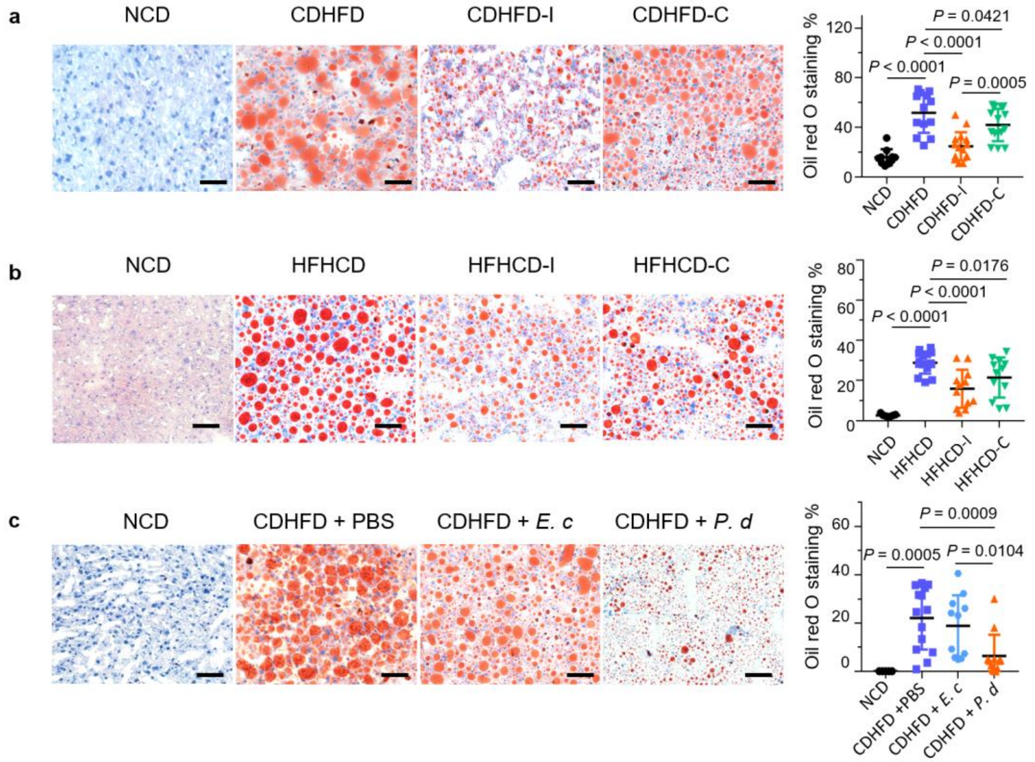

Supplemental Figure 1. Inulin, cellulose, or *P. distasonis* reduced Oil Red O staining score. a Image and score of Oil Red O staining of mice liver that received NCD (n=10), CDHFD (n=13), CDHFD-I (n=15), or CDHFD-C (n=15), respectively. **b** Image and score of mice liver that received NCD (n=7), HFHCD (n=15), HFHCD-I (n=12), or HFHCD-C (n=12), respectively. **c** Image and score of mice liver that received NCD (n=5), CDHFD + PBS (n=14), CDHFD + *E. c* (n=11), or CDHFD + *P. d* (n=12), respectively. CDHFD: choline deficient high fat diet; CDHFD-I: CDHFD + inulin; CDHFD-C: CDHFD + cellulose; HFHCD: high fat high cholesterol diet; HFHCD-I: HFHCD + inulin; HFHCD-C: HFHCD + cellulose; *E.c*: *E. coli*; *P. d*: *P. distasonis*. Data are presented as mean of biological replicates ± SD. *P* value was obtained by one-way ANOVA with Fisher's LSD method. Scale bars, 50 μm.

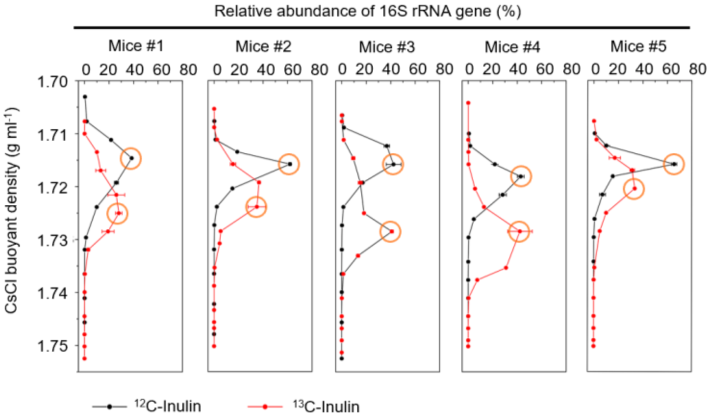

Supplemental Figure 2. Quantitative distributions of 16S rRNA gene. Paired data was collected from 5 mice, where stool collected at 0 h and 36 h of the ^{13}C-incubation experiments represent ^{12}C-Inulin and ^{13}C-Inulin treatment, respectively. Black and red points represent ^{12}C-Inulin or ^{13}C-Inulin treated sample, respectively. Normalized data were shown as the ratio of the gene copy number in each fraction to the total quantities in each treatment. Data was showed in means ± SD. The orange circle means data was selected for short-gun metagenome sequencing.

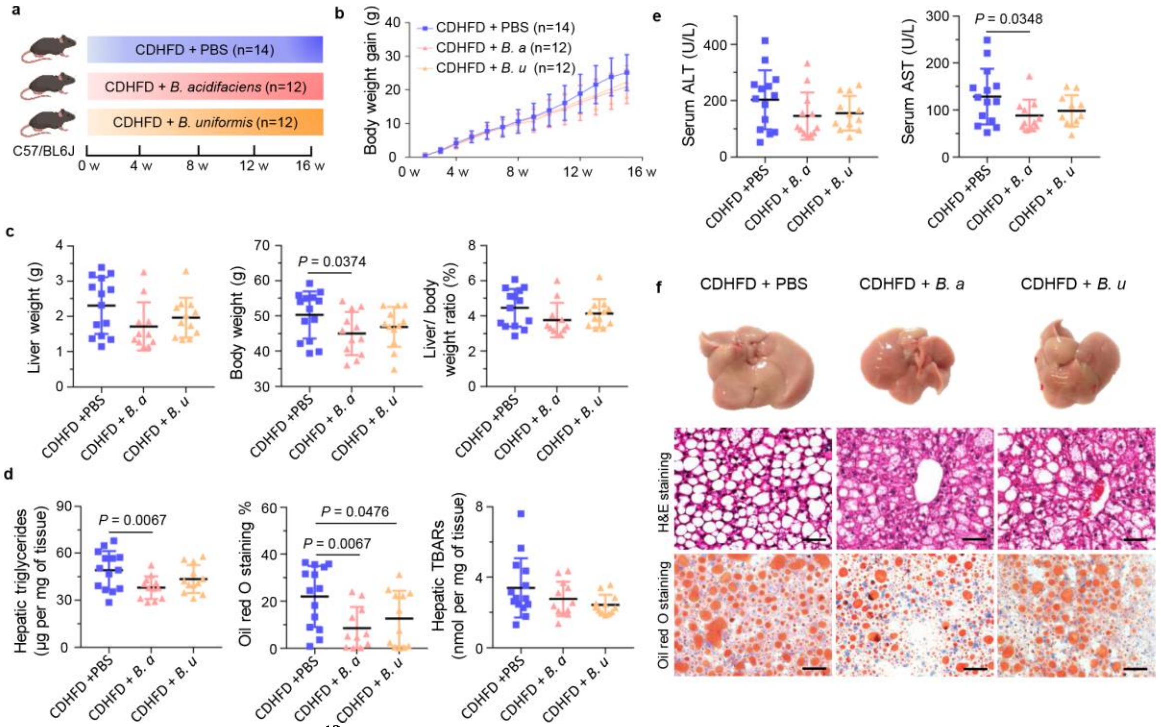

Supplemental Figure 3. ^{13}C-Inulin labelled *B. acidifaciens* and *B. uniformis* ameliorated CDHFD induced NASH in mice. a Experiment design of CDHFD induced NASH model (Created with BioRender.com). **b** Growth curve of body weight gain under different treatments. Data are presented as mean of biological replicates ± SD. *P* value obtained with two-way ANOVA Fisher's LSD test. **c** Liver weight, body weight, and liver/body weight ratio. **d** Hepatic triglyceride level, Oil red O staining score, and hepatic TBARs level. **e** Serum ALT, AST from mice fed CDHFD + PBS, CDHFD + *B. a*, and CDHFD + *B. u*, respectively. **b-e** 12-14 mice were used in each group, including CDHFD (n=14), CDHFD + *B. acidifaciens* (*B. a*) (n=12), CDHFD + *B. uniformis* (*B. u*) (n=12). Data are presented as mean of biological replicates ± SD. *P* value obtained with one-way ANOVA using Fisher's LSD. **f** Representative gross morphology, H&E staining, and Oil red O staining of liver from mice fed CDHFD + PBS, CDHFD + *B. a*, and CDHFD + *B. u*, respectively. Scale bars, 50 μm. One slide per mouse was stained.

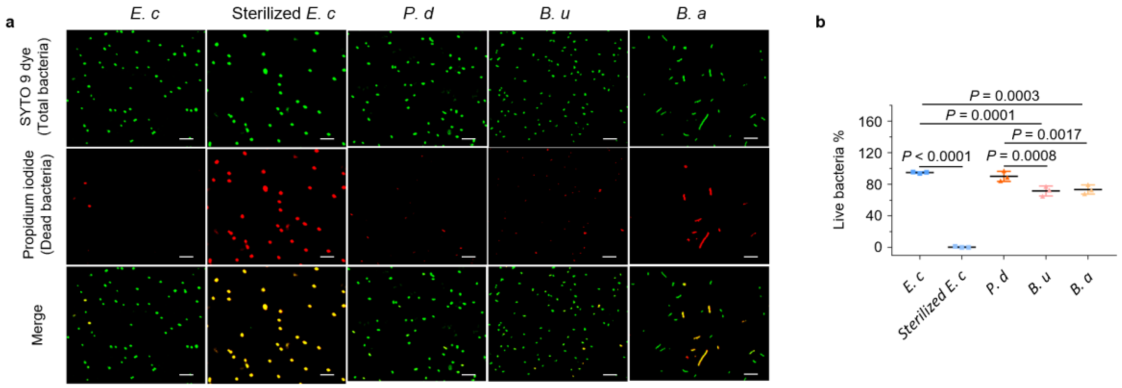

Supplemental Figure 4 Bacterial viability measurement. a Representative confocal images of stained bacteria including *E. c*, sterilized *E. c*, *P. d*, *B. u*, and *B. a*, respectively. Total bacteria was stained SYTO 9 dye with green fluorescence; dead bacteria was stained with Propidium iodide dye with red fluorescence. *E. c* and ethanol sterilized *E. c* served as positive and negative control, respectively. Scale bars, 30 μm. **b** Quantification of bacterial viability. Data are presented as mean of biological replicates (n=3) ± SD. *P* value obtained with one-way ANOVA using Fisher's LSD.

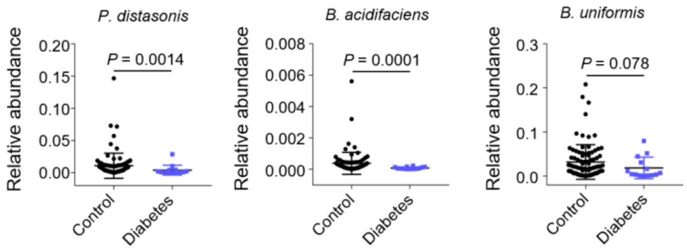

Supplemental Figure 5. The relative abundance of the top three inulin derived bacteria in a clinical cohort. The clinical data was obtained from a public database. A total of 105 subjects were included in this clinical cohort, including 91 healthy controls and 14 diabetes patients. Gut microbial composition was determined by short-gun metagenome sequencing data. Data was represented as mean of biological replicates ± SD. Statistical significance was determined by two tailed Mann-Whitney U test.

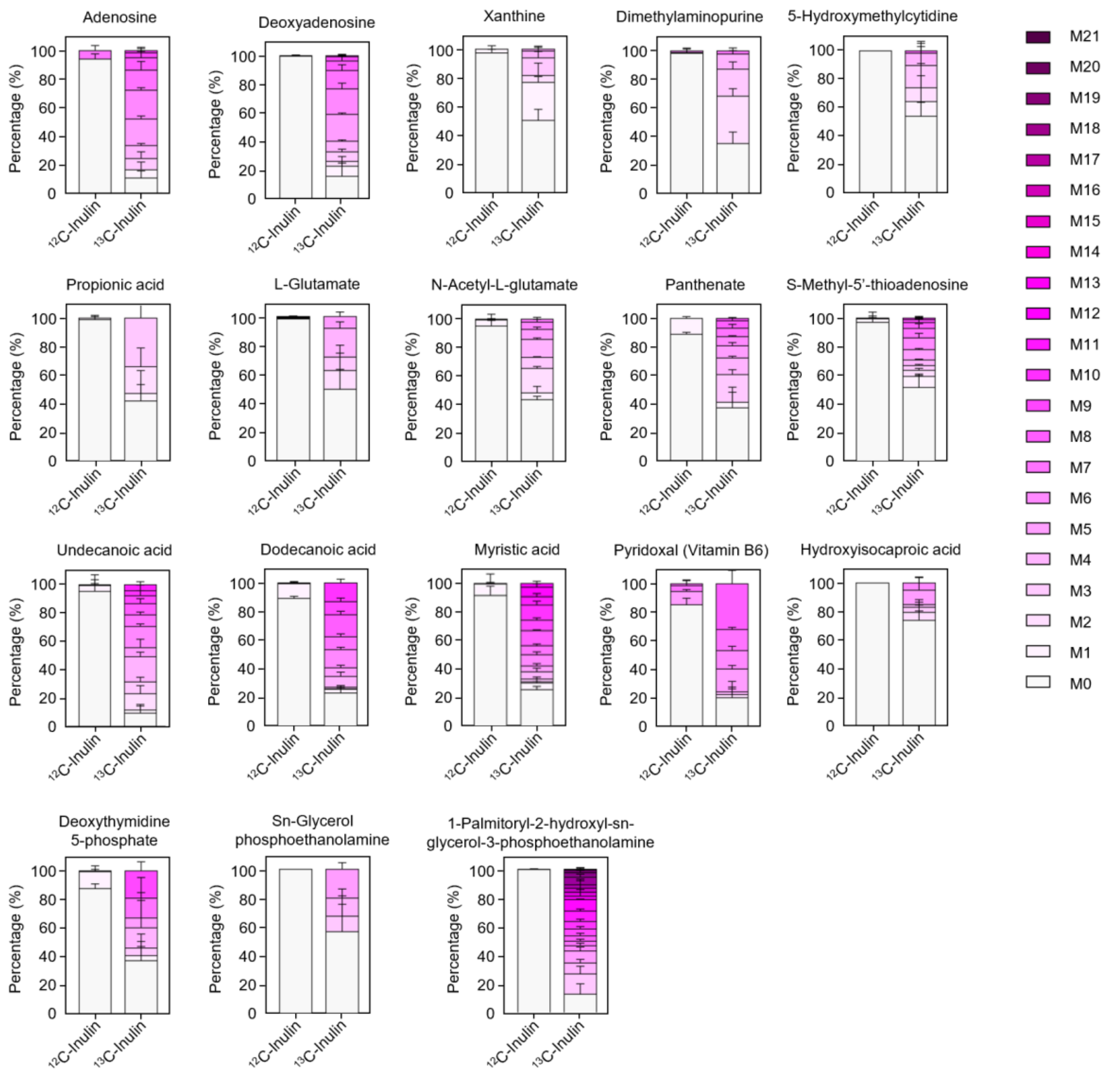

Supplemental Figure 6. Isotopologue distribution of labelled metabolites in stool of mice treated with CDHFD-I. Stool samples collected at 0 h or 36 h replicated ^{12}C-Inulin or ^{13}C-Inulin, respectively. The percentage of ^{13}C labeling was presented as mean of 3 biological replicates with ± SEM.

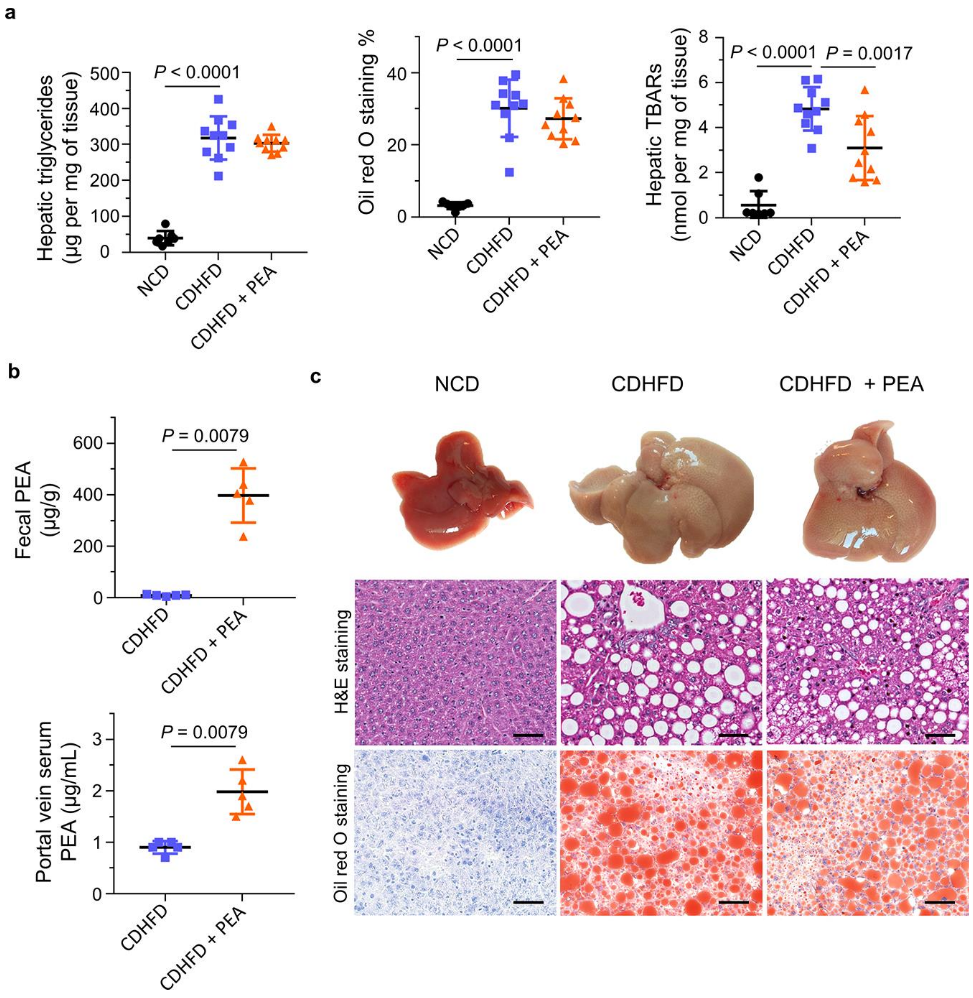

Supplemental Figure 7. Administration of PEA in mice fed CDHFD. a hepatic triglyceride level, Oil red O staining score, and hepatic TBARs level of mice treated with NCD (n=7), CDHFD (n=10), or CDHFD + PEA (n=10). Data are presented as mean of biological replicates ± SD. *P* value was obtained by one-way ANOVA with Fisher's LSD method. **b** Quantification of fecal and portal vein serum PEA levels. n=5 mice were used in each group. Data are presented with mean of biological replicates ± SD. *P* value was obtained by two-tailed Mann Whitney test. **c** Representative gross morphology, H&E staining, and Oil red O staining of liver from mice fed NCD, CDHFD, and CDHFD + PEA, respectively. Scale bars, 50 µm. One slide per mouse was stained.

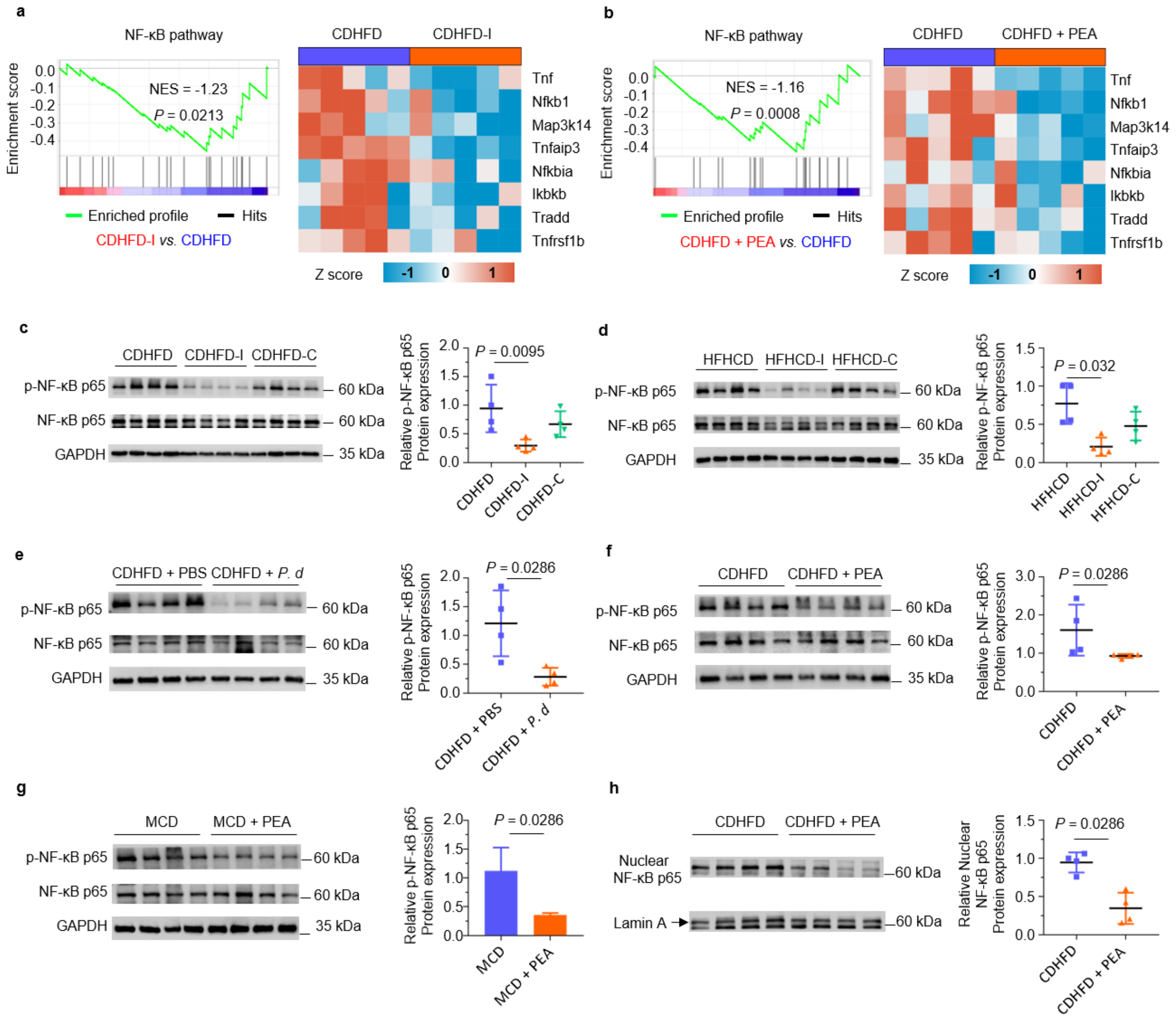

Supplemental Figure 8. Inulin, *P. distasonis*, or pentadecanoic acid suppressed NF-κB activation. Enrichment plots of NF-κB pathway and heatmap of differentially expressed genes in mice fed CDHFD *vs.* CDHFD-I **a** or in mice fed CDHFD *vs.* CDHFD + PEA **b**. **a-b**, 5 mice were used in each group. *P* value was obtained by gene set enrichment analysis. Phosphorylated NF-κB expression in mice fed CDHFD **c** or HFHCD **d** that treated with inulin or cellulose. Phosphorylated NF-κB expression in mice fed CDHFD and treated with *P. d* **e** or PEA **f**. Phosphorylated NF-κB expression in mice fed MCD diet and treated with PEA **g**. Nuclear Phosphorylated NF-κB expression **h** in mice fed CDHFD or CDHFD treated with PEA. **c-h**, n=4 biological replicate samples were used in each group, statistic data are presented as means ± SD.

P value obtained with one-way ANOVA using Fisher's LSD. *P. d*: *P. distasonis*; PEA: pentadecanoic acid.

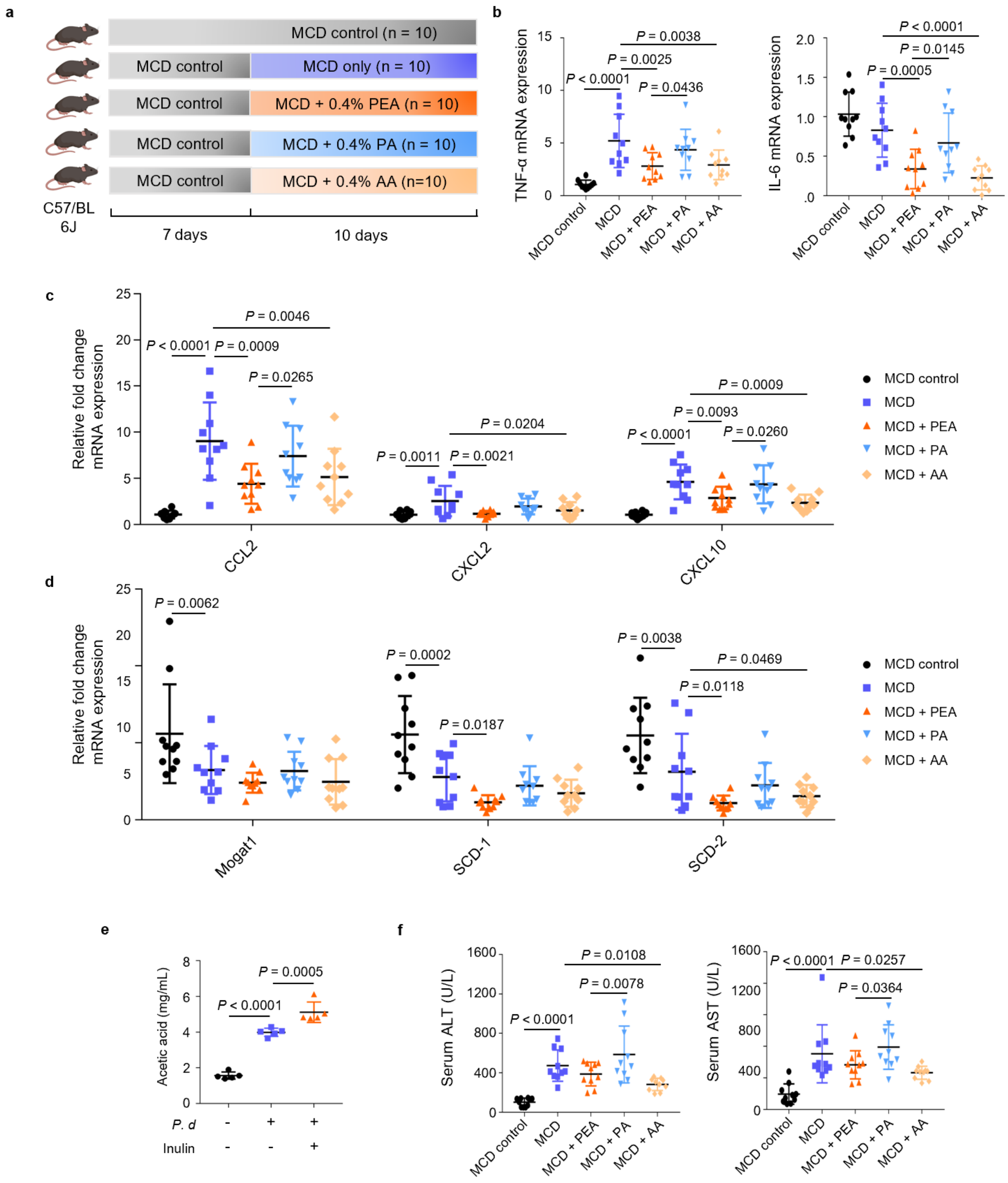

Supplemental Figure 9. Comparison of the effects of pentadecanoic acid with palmitic acid or acetic acid in MCD induced NAFLD/NASH. a Study design of MCD induced

NAFLD/NASH model with pentadecanoic acid (PEA), palmitic acid (PA) or acetate (AA) treatment (Created with BioRender.com). **b** mRNA expression levels of TNF-a and IL6. **c** qPCR validation pro-inflammatory chemokine and cytokine expression in the liver tissues. **d** qPCR validation of triacylglyceride synthesis genes in the liver tissues. **e** Quantification of acetic acid level in conditioned medium with or without inoculation of *P. distasonis*. 5 biological replicate samples were used in each group. **f** Serum ALT and AST. **a-d, f** 10 mice were used in each group. **a-f**, Data are presented as mean of biological replicates ± SD. *P* value obtained with one-way ANOVA using Fisher's LSD.

Supplemental Table 1. Diet composition of Normal chow diet (NCD)

Diet Formulas	NCD
Product #	Laboratory Rodent Diet 5001
	gm%
Protein	28.67
Total fat	13.38
Carbohydrate	57.94
Kcal/gm	4.09
Ingredient	Gm g/Kg
Protein	24.1
Fat (ether extract)	5.00
Fat (acid hydrolysis)	6.40
Fiber (Crude)	5.2
Starch	21.90
Sucrose	3.15
Minerals	6.90
Vitamins	4.6

This diet is purchased from LabDiet, US.

Supplemental Table 2. Diet composition of Choline deficient high fat diet (CDHFD)

Diet Formulas	CDHFD	CDHFD + Inulin (CDHFD-I)	CDHFD + Cellulose (CDHFD-C)
Product #	D19042402	D19042403	D19042404
	gm%	gm%	gm%
Protein	27.2	25.6	24.7
Carbohydrate	27.3	22.1	24.8
Fat	36.2	34.0	32.9
Kcal/gm	5.43	5.11	4.94
Ingredient	gm	Gm	Gm
Casein, 80 Mesh	200	200	200
L-Cystine	3	3	3
Maltodextrin 10	125	96.8	125
Sucrose	68.8	68.8	68.8
Cellulose, BW200	25	25	100
Inulin	0	75	0
Soybean Oil	25	25	25
Lard	245	245	245
Mineral Mix, V10001	10	10	10
DiCalcium Phosphate	13	13	13
Calcium Carbonate	5.5	5.5	5.5
Potassium Citrate, 1H20	16.5	16.5	16.5
Vitamin Mix, V10001	10	10	10
Choline Bitartrate	0	0	0
Total	746.85	793.65	821.86

This diet is purchased from Research Diet, US.

Supplemental Table 3. Histological score of hepatic steatosis, necro-inflammation, ballooning, and fibrosis in CDHFD induced NASH model

Histological score	NCD	CDHFD	CDHFD-I	CDHFD-C
Steatosis	0.00±0.00****	2.33±0.97	0.98±0.83$^{****\#}$	1.63±0.94*
Necro-Inflammation	0.04±0.12****	1.36±1.11	0.18±0.36****	0.43±0.50***
Ballooning	0.00±0.00****	1.38±0.87	0.43±0.76***	0.73±0.70*
Fibrosis	0.00±0.00****	1.08±0.76	0.13±0.35$^{****\#}$	0.60±0.51*

$^{****}p<0.0001$, $^{***}p<0.001$, $^{**}p<0.01$, $^{*}p<0.05$ *vs.* mice fed CDHFD.

$^{\#}p<0.05$ *vs.* mice fed CDHFD-C.

10-15 mice were used in each group, including NCD (n=10), CDHFD (n=13), CDHFD-I (n=15), CDHFD-C (n=15). Data are presented as mean of biological replicates ± SD. *P* value was obtained by one-way ANOVA with Fisher's LSD test. Two independent experiments were repeated showing the similar results.

Supplemental Table 4. Diet composition of High fat high cholesterol diet (HFHCD)

Diet Formulas	HFHCD
Product #	SF11-078
	gm%
Protein	19.40
Total fat	23.00
Carbohydrate	52.40
% Total calculated digestible energy from lipids	43.70%
% Total calculated digestible energy from protein	19.70%
Ingredient	Gm g/Kg
Casein (Acid)	200
Sucrose	424
Pre-Gelled Wheat Starch	50
Canola Oil	50
Lard	181
Cellulose	50
L Methionine	3.0
Inulin	0
Calcium Carbonate	13.1
Sodium Chloride	2.6
AIN93 Trace Minerals	1.4
Potassium Citrate	2.5
Potassium Dihydrogen Phosphate	6.9
Potassium Sulphate	1.6
Choline Chloride (75%)	2.5
Cholesterol	1.9
AIN93 Vitamins	10

This diet is purchased from Specialty feeds, Australia.

Supplemental Table 5. Histological score of hepatic steatosis, necro-inflammation, ballooning, and fibrosis in HFHCD induced NASH model

Histological Score	NCD	HFHCD	HFHCD-I	HFHCD-C
Steatosis	$0.00 \pm 0.00^{****}$	2.43 ± 0.59	$1.38 \pm 1.11^{**}$	$1.67 \pm 0.98^{*}$
Necro-Inflammation	$0.00 \pm 0.00^{***}$	1.53 ± 1.06	$0.67 \pm 0.98^{*}$	$0.75 \pm 0.97^{*}$
Ballooning	$0.00 \pm 0.00^{****}$	1.47 ± 0.64	$0.58 \pm 0.51^{***}$	$0.92 \pm 0.79^{*}$
Fibrosis	$0.00 \pm 0.00^{****}$	1.27 ± 0.70	$0.33 \pm 0.49^{***}$	$0.75 \pm 0.62^{*}$

$^{****}p<0.0001$, $^{**}p<0.01$, $^{*}p<0.05$ *vs.* mice fed HFHCD.

7-15 mice were used in each group, including NCD (n=10), HFHCD (n=15), HFHCD-I (n=12), HFHCD-C (n=12). Data are presented as mean of biological replicates ± SD. *P* value was obtained by one-way ANOVA with Fisher's LSD test. Two independent experiments were repeated showing the similar results.

Supplemental Table 6. Primers used in this study

Gene name	species	Primer (forward)	Primer (Reverse)
16S rRNA	bacteria	CCTAYGGGRBGCASCAG	GGACTACHVGGGTWTCTAAT
GAPDH	mouse	CATCACTGCCACCCAGAAGACTG	ATGCCAGTGAGCTTCCCGTTCAG
CCL2	mouse	GCTACAAGAGGATCACCAGCAG	GTCTGGACCCATTCCTTCTTGG
CXCL2	Mouse	CATCCAGAGCTTGAGTGTGACG	GGCTTCAGGGTCAAGGCAAACT
CXCL10	Mouse	ATCATCCCTGCGAGCCTATCCT	GACCTTTTTTGGCTAAACGCTTTC
Mogat1	Mouse	CCAGCACTACTTTGGCATAATGC	CCTCTAGGTATGTCTGATGCAGC
SCD-1	Mouse	GCAAGCTCTACACCTGCCTCTT	CGTGCCTTGTAAGTTCTGTGGC
SCD-2	Mouse	GTCTGACCTGAAAGCCGAGAAG	GCAAGAAGGTGCTAACGCACAG
TNF-α	Mouse	CCAGACCCTCACACTCAGATC	CACTTGGTGGTTTGCTACGAC
IL-6	Mouse	TACCACTTCACAAGTCGGAGGC	CTGCAAGTGCATCATCGTTGTTC
ACTA2	Mouse	TGCTGACAGAGGCACCACTGAA	CAGTTGTACGTCCAGAGGCATAG

Supplemental Table 7. Histological score of hepatic steatosis and necro-inflammation in CDHFCD induced NASH model and treated with selected bacteria.

Histological Score	NCD	CDHFD + PBS	CDHFD + *E. c*	CDHFD + *P. d*	CDHFD + *B. a*	CDHFD + *B. u*
Steatosis	0.00±0.00****	2.36±0.84	2.36±0.81	0.92±1.00$^{****\#\#\#}$	1.67±0.78	2.08±1.08
Necro-Inflammation	0.00±0.00**	1.21±0.97	1.18±0.87	0.42±0.67$^{*\#}$	0.67±0.78	0.67±0.65

$^{*}p<0.05$, $^{**}p<0.01$, $^{****}p<0.0001$ *vs.* mice fed CDHFD + PBS.

$^{\#}p<0.05$, $^{\#\#\#}p<0.001$ *vs.* mice fed CDHFD + *E. c*.

5-14 mice were used in each group, including NCD (n=5), CDHFD + PBS (n=14), CDHFD + *E. c* (n=11), CDHFD + *P. d* (n=12), CDHFD + *B. a* (n=12), CDHFD + *B. u* (n=12). Data are presented as mean of biological replicates ± SD. *P* value was obtained by one-way ANOVA with Fisher's LSD test. Two independent experiments were repeated showing the similar results.

Supplemental Table 8. Histological score of hepatic steatosis and necro-inflammation in CDHFCD induced NASH model and treated with PEA.

Histological Score	NCD	CDHFD	CDHFD + PEA
Steatosis	0.00±0.00***	2.50±0.53	2.15±0.67
Necro-Inflammation	0.00±0.00****	1.50±0.71	1.10±0.74

$^{***}p<0.001$, $^{****}p<0.0001$ *vs.* mice fed CDHFD.

7-10 mice were used in each group, including NCD (n=5), CDHFD (n=10), CDHFD + PEA (n=10). Data are presented as mean of biological replicates ± SD. *P* value was obtained by one-way ANOVA with Fisher's LSD test. Two independent experiments were repeated showing the similar results.

Supplemental Table 9. Diet composition of methionine choline deficient (MCD) diet.

Diet Formulas	MCD Control diet	MCD diet
Product #	960441	960439
	g/kg	g/kg
Sucrose	455.3 gm	455.3 gm
Corn Starch	200.0 gm	200.0 gm
Corn Oil	100.0 gm	100.0 gm
Alphacel Non-Nutritive Bulk	30.0 gm	30.0 gm
AIN 76 Mineral Mix	35.0 gm	35.0 gm
DL Methionine	30 gm	0 gm
Choline	20 gm	0 gm
Pentadecanoic acid	0 gm	0 gm

This diet is purchased from MP Biomedicals, LLC

Unprocessed scans of blots and gels

Supplemental Figure 8c

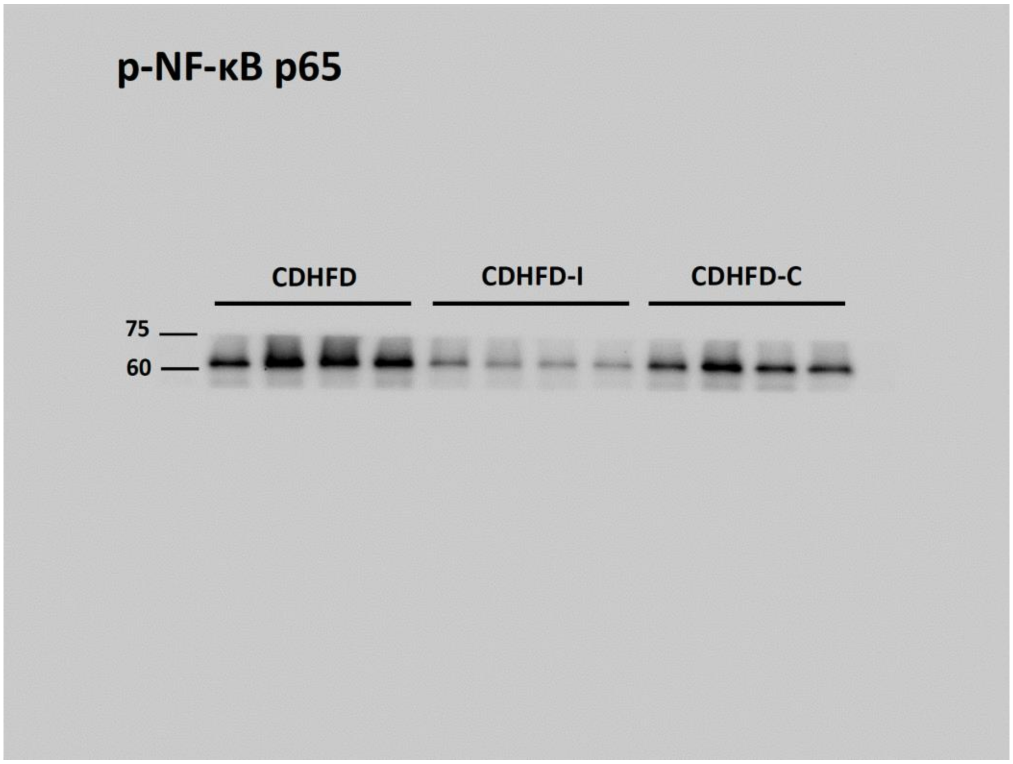

Supplemental Figure 8c

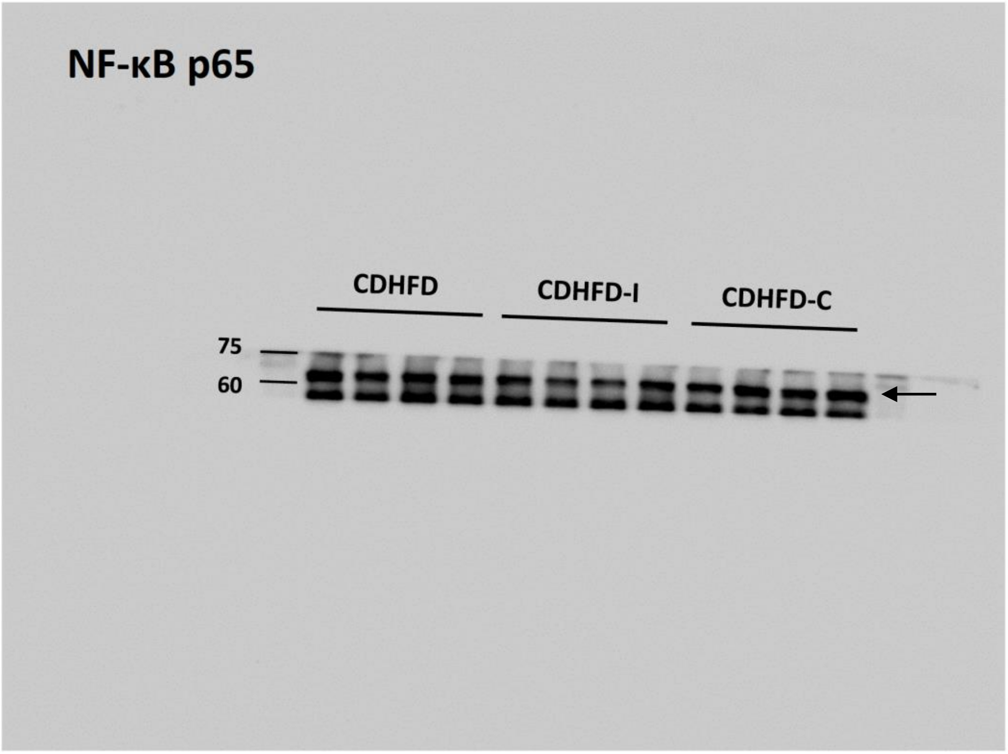

Supplemental Figure 8c

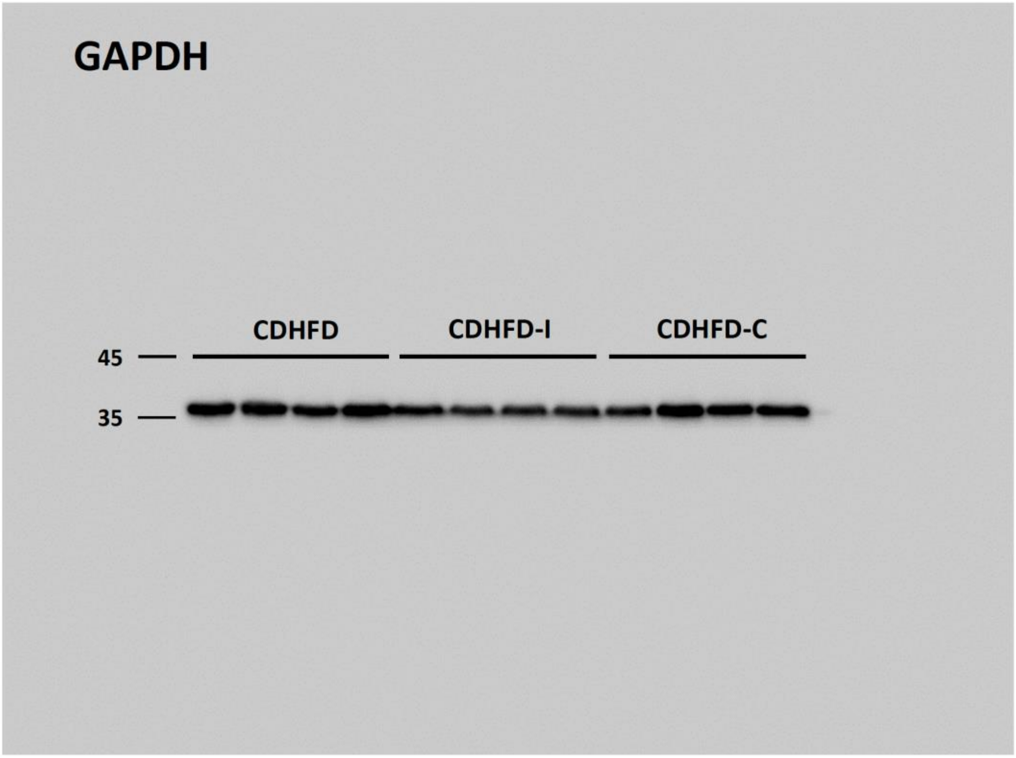

Supplemental Figure 8d

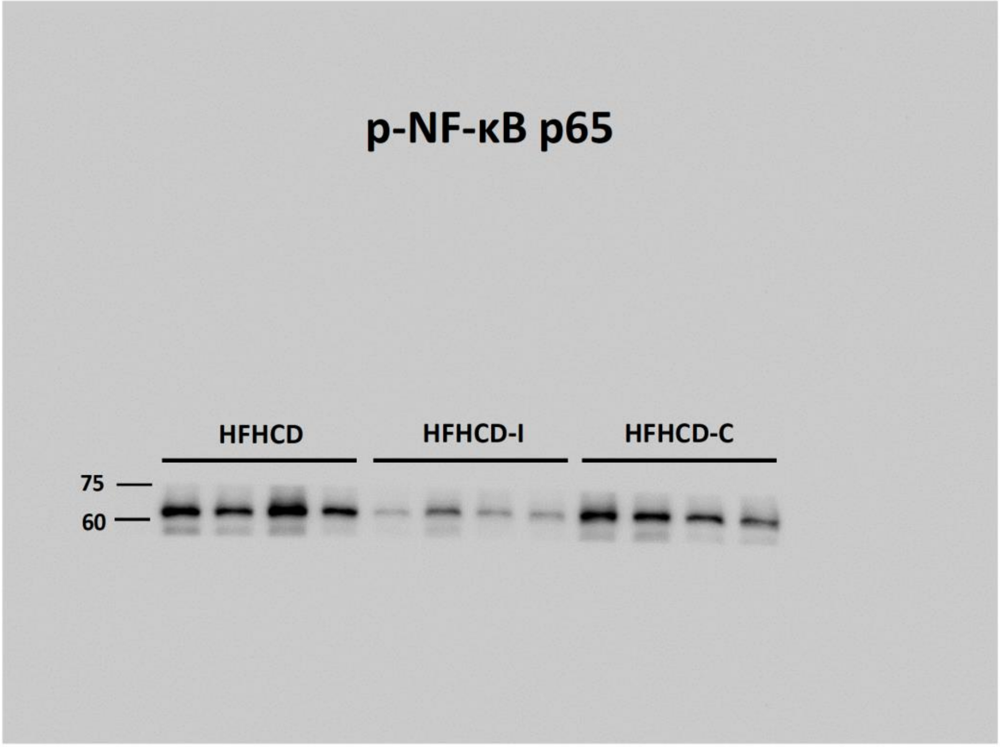

Supplemental Figure 8d

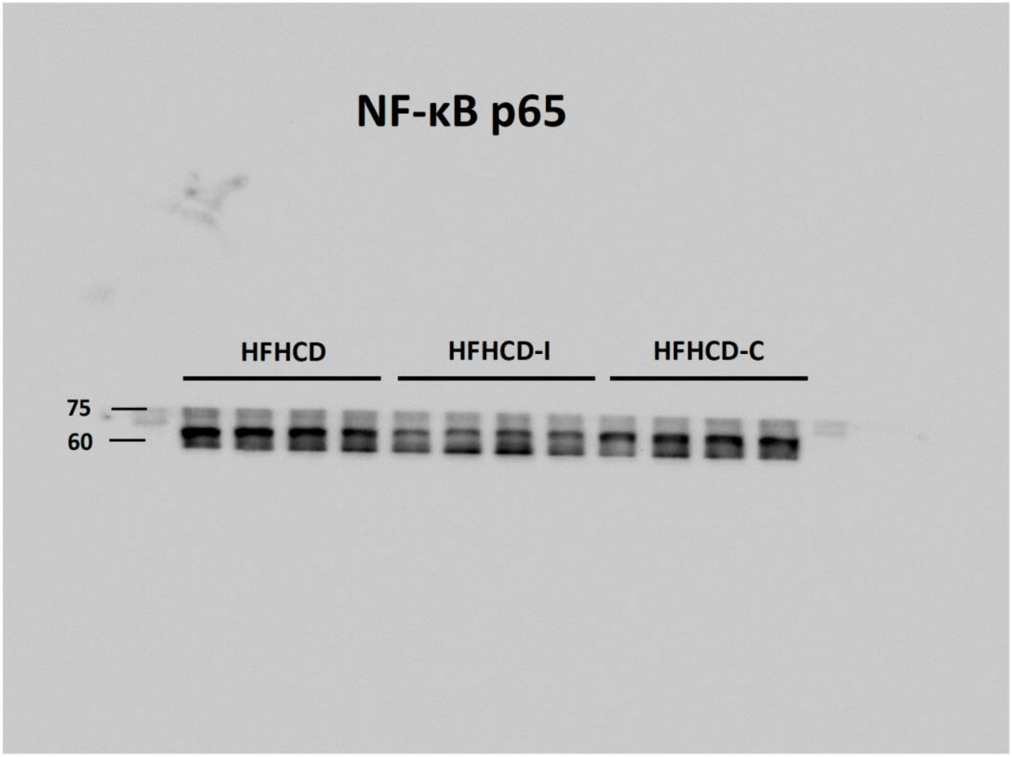

Supplemental Figure 8d

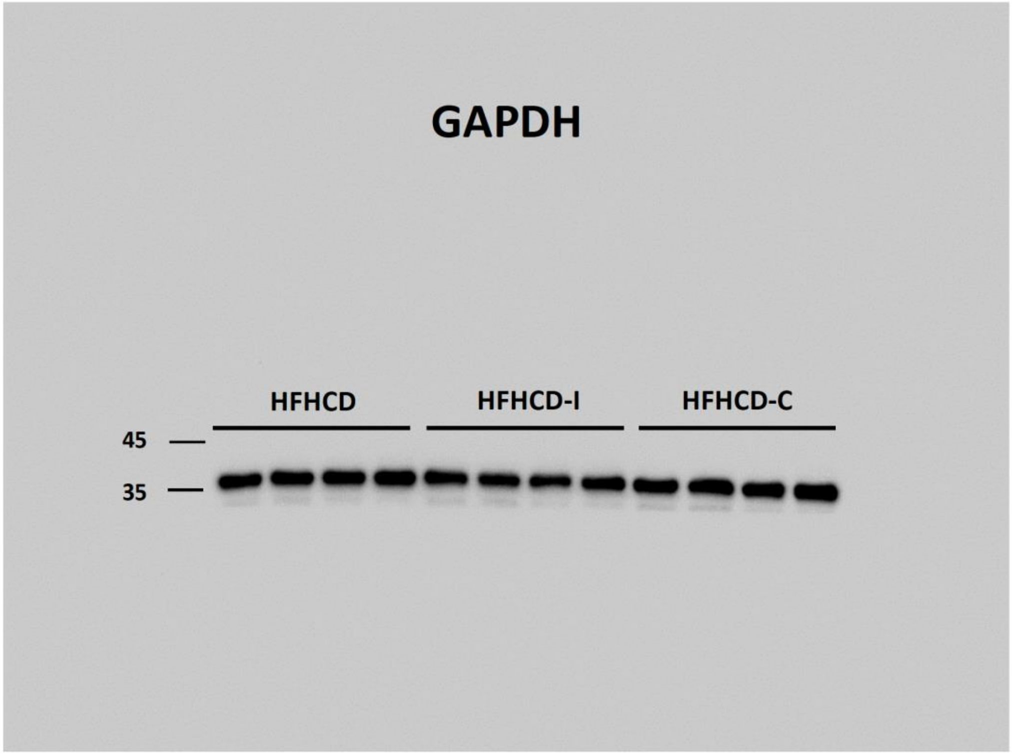

Supplemental Figure 8e

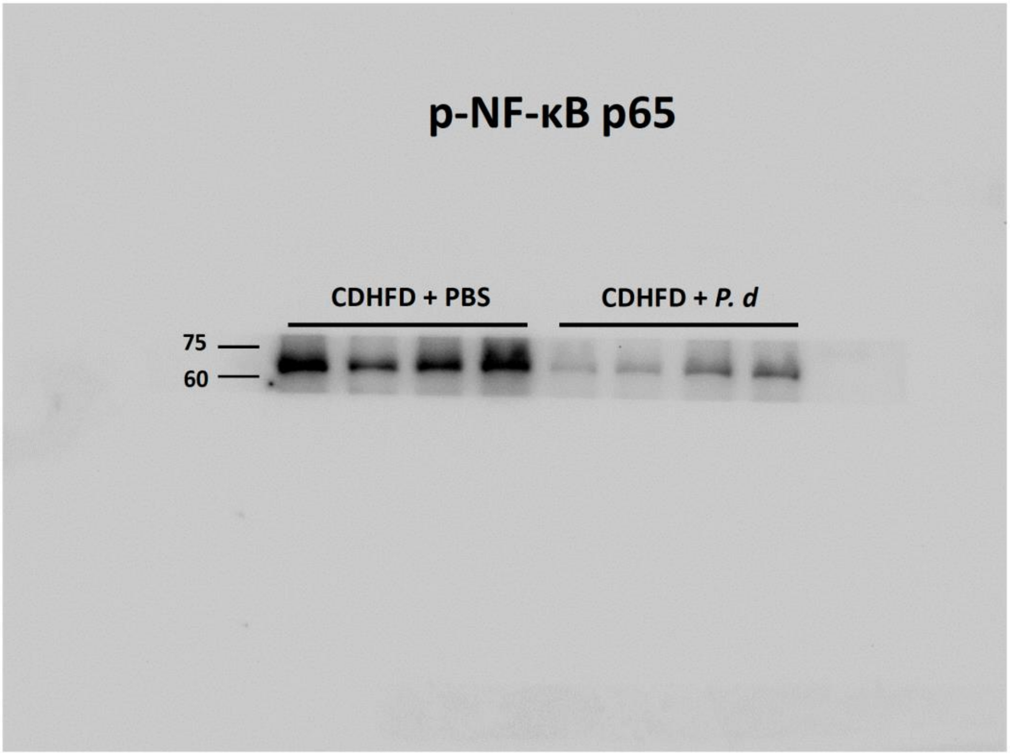

Supplemental Figure 8e

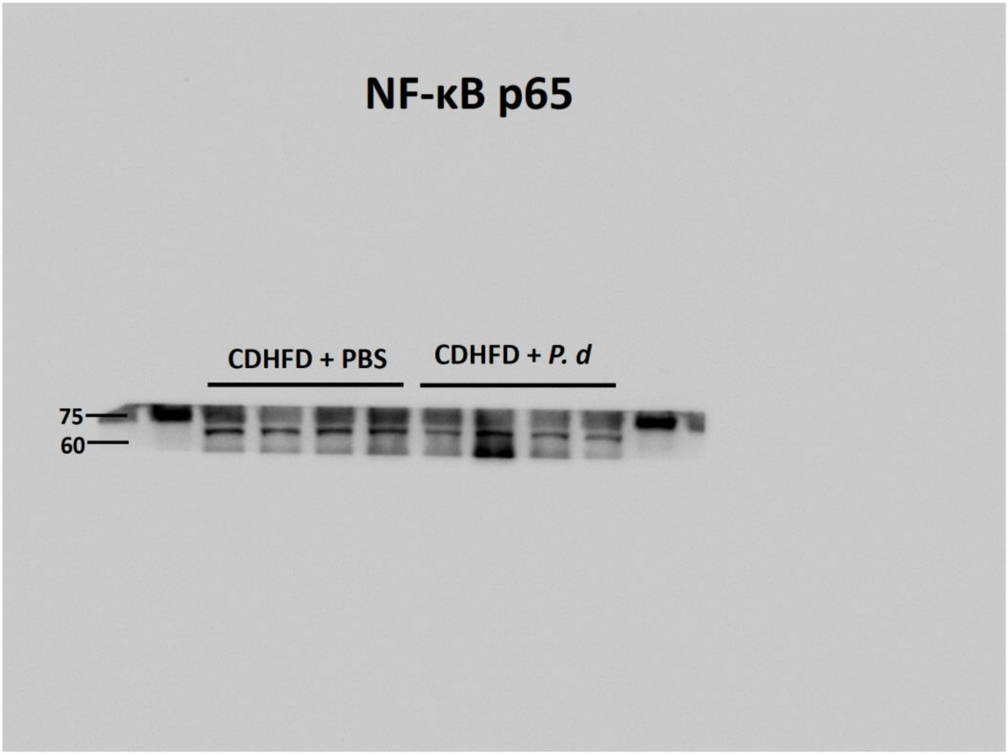

Supplemental Figure 8e

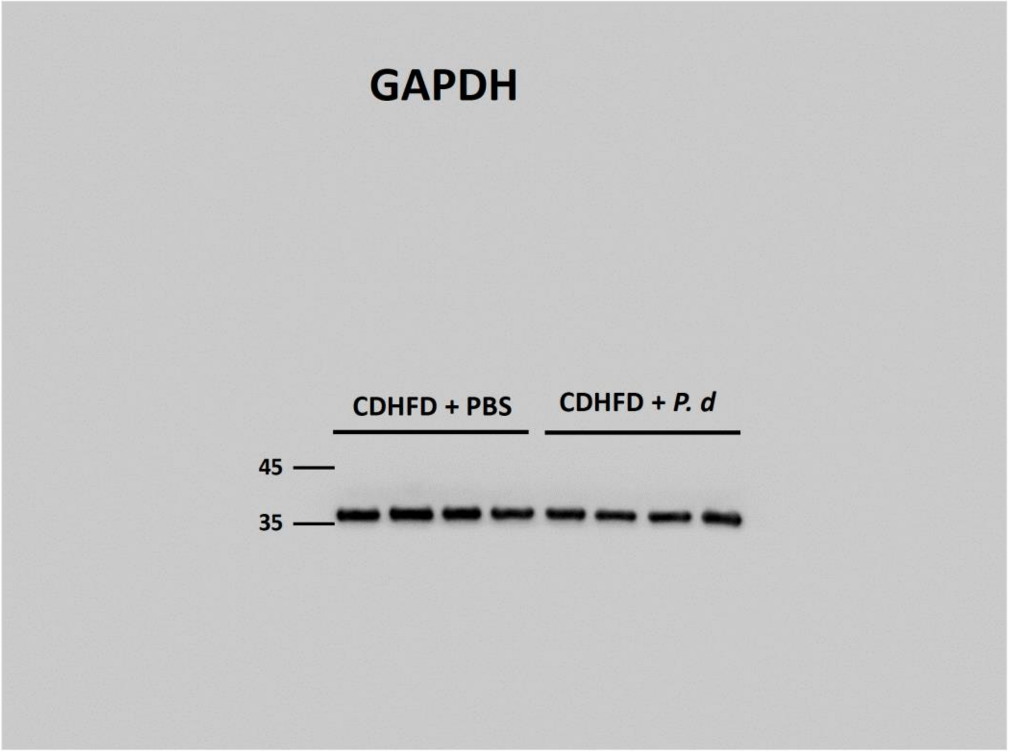

Supplemental Figure 8f

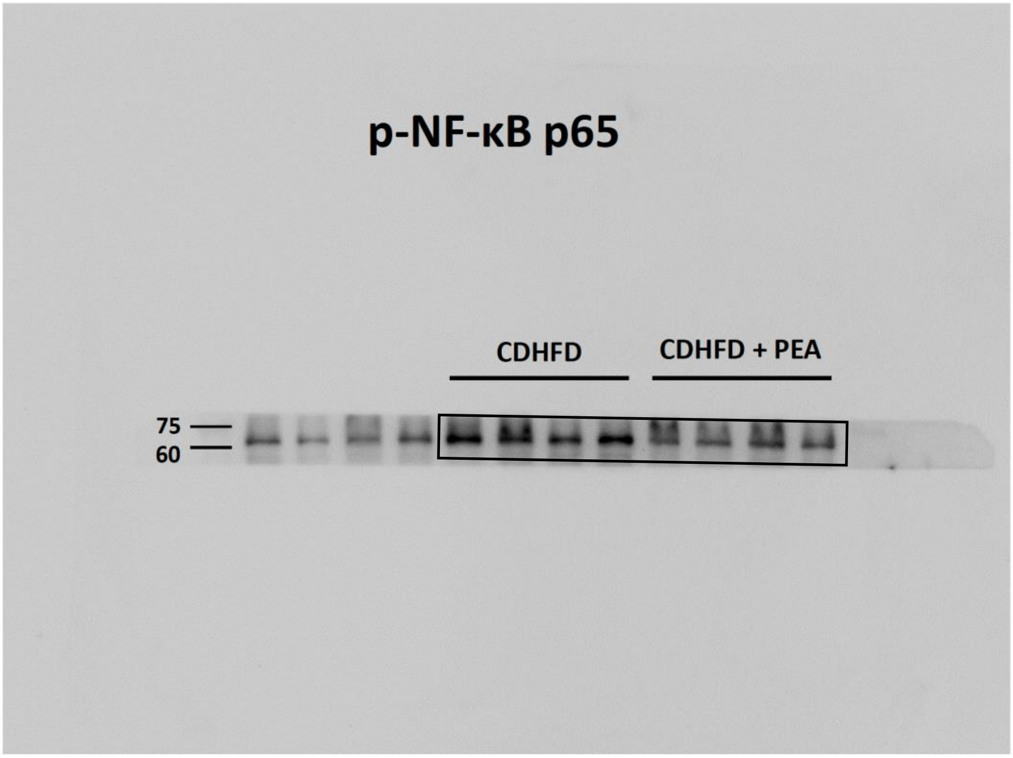

Supplemental Figure 8f

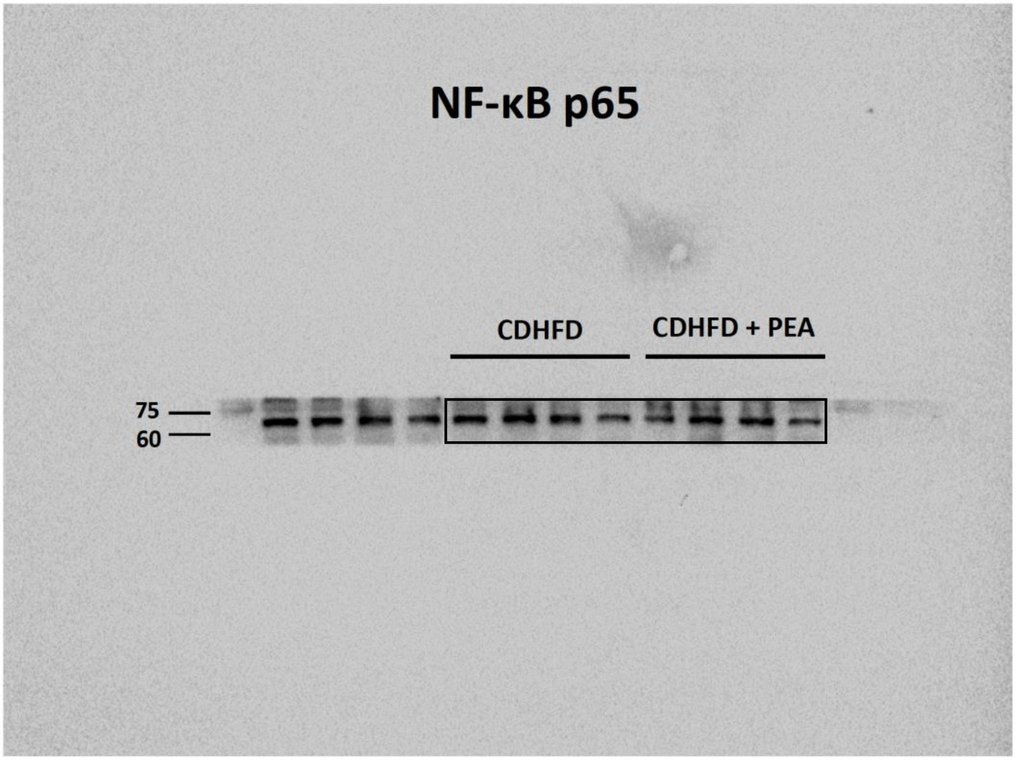

Supplemental Figure 8f

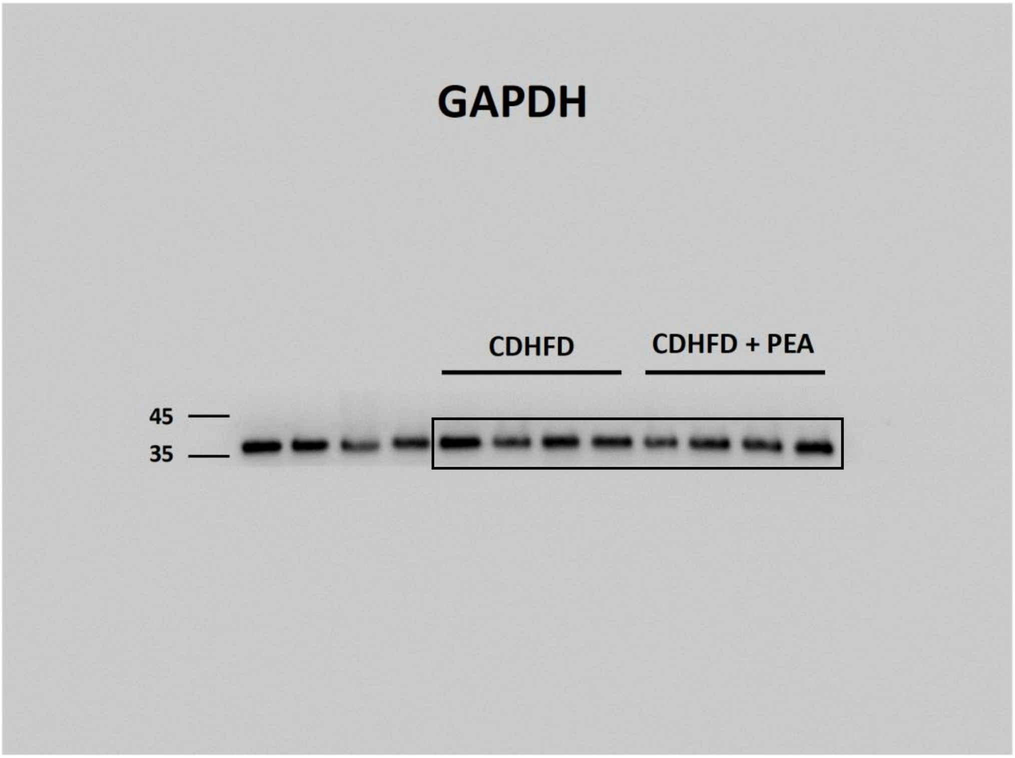

Supplemental Figure 8g

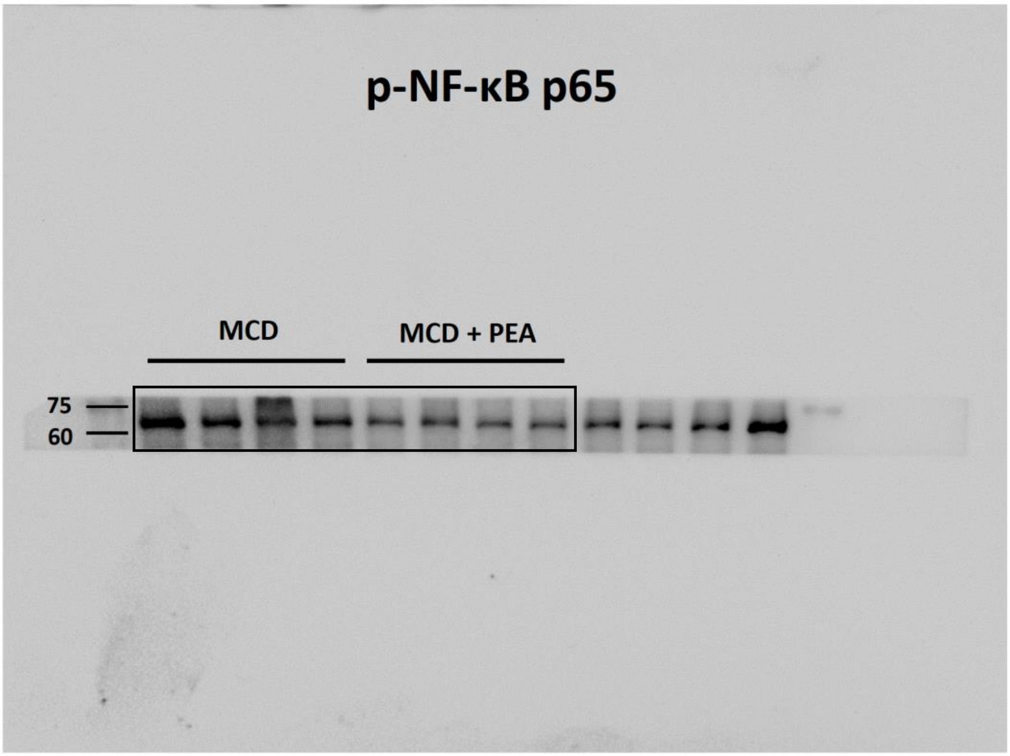

Supplemental Figure 8g

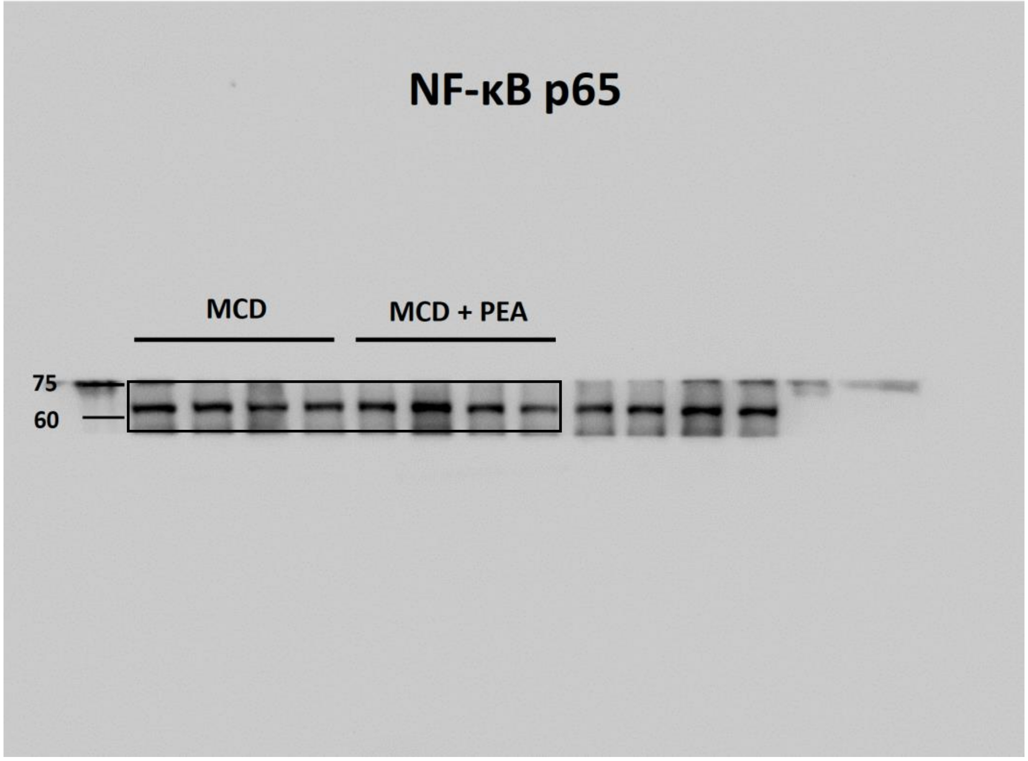

Supplemental Figure 8g

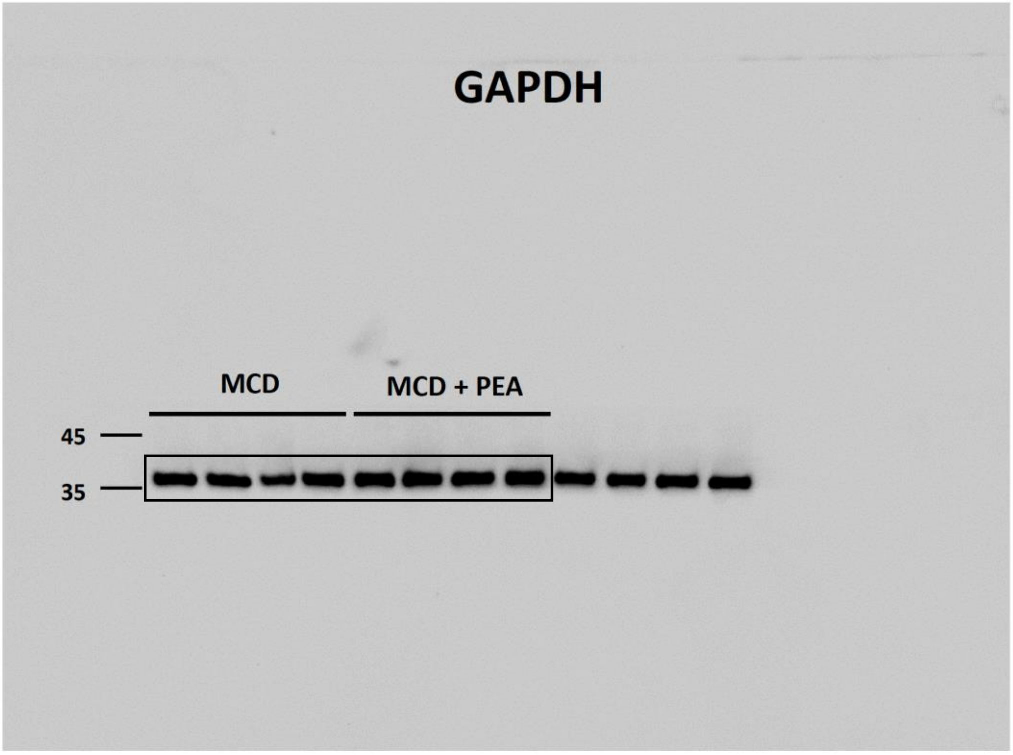

Supplemental Figure 8h

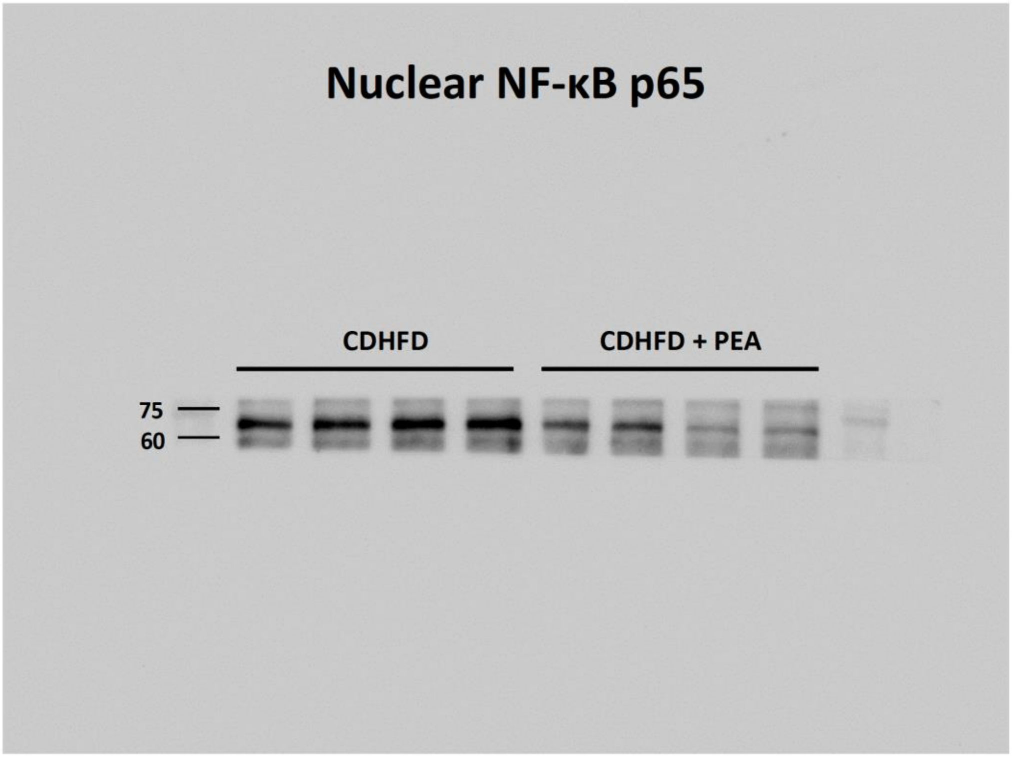

Supplemental Figure 8h

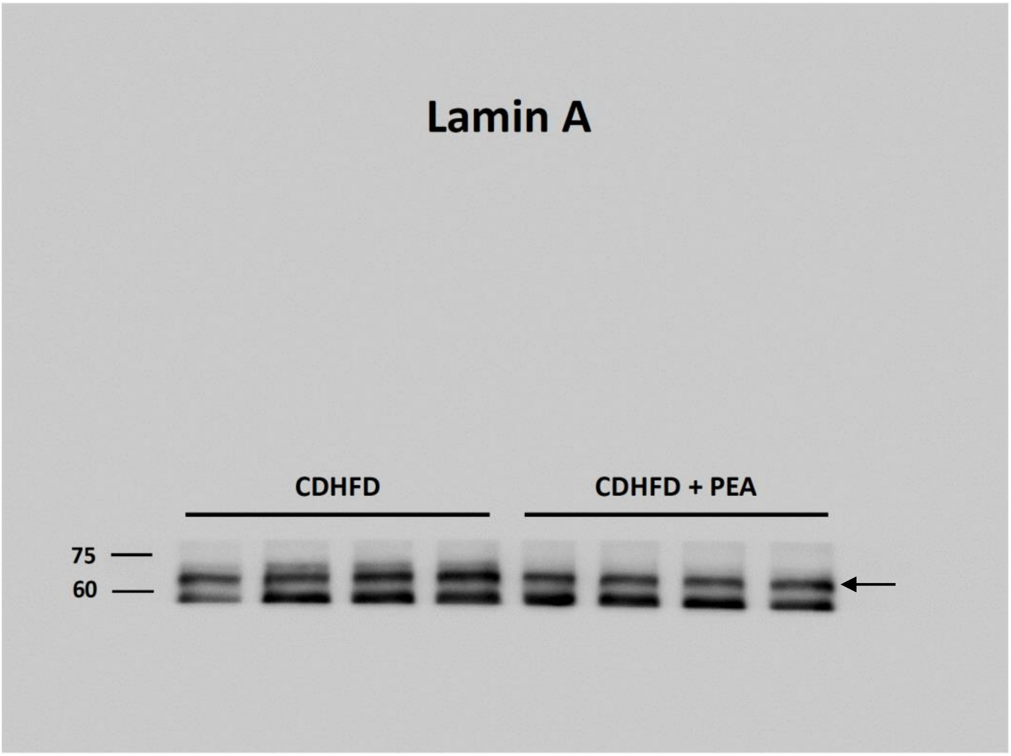

Supplement: Supplementary file 1 — Supplementary Figs. 1–9, Tables 1–9 and images: unprocessed scans of blots and gels. [file 41564_2023_1418_MOESM1_ESM.pdf]
