# Supplementary material for: Parabacteroides distasonis uses dietary inulin to suppress NASH via its metabolite pentadecanoic acid
Source: Nat Microbiol. 2023 Jun 29;8(8):1534–48. doi: 10.1038/s41564-023-01418-7 (PMC10390331; doi:10.1038/s41564-023-01418-7)
Supplement: Supplementary file 2 — Reporting Summary [file 41564_2023_1418_MOESM2_ESM.pdf]

nature portfolio

Corresponding author(s): JUN YU
Last updated by author(s): Apr 26, 2023

Reporting Summary

Nature Portfolio wishes to improve the reproducibility of the work that we publish. This form provides structure for consistency and transparency in reporting. For further information on Nature Portfolio policies, see our Editorial Policies and the Editorial Policy Checklist.

Statistics

For all statistical analyses, confirm that the following items are present in the figure legend, table legend, main text, or Methods section.

n/a	Confirmed	
☐	☒	The exact sample size (*n*) for each experimental group/condition, given as a discrete number and unit of measurement
☐	☒	A statement on whether measurements were taken from distinct samples or whether the same sample was measured repeatedly
☐	☒	The statistical test(s) used AND whether they are one- or two-sided<br>*Only common tests should be described solely by name; describe more complex techniques in the Methods section.*
☐	☒	A description of all covariates tested
☐	☒	A description of any assumptions or corrections, such as tests of normality and adjustment for multiple comparisons
☐	☒	A full description of the statistical parameters including central tendency (e.g. means) or other basic estimates (e.g. regression coefficient) AND variation (e.g. standard deviation) or associated estimates of uncertainty (e.g. confidence intervals)
☐	☒	For null hypothesis testing, the test statistic (e.g. *F*, *t*, *r*) with confidence intervals, effect sizes, degrees of freedom and *P* value noted<br>*Give P values as exact values whenever suitable.*
☒	☐	For Bayesian analysis, information on the choice of priors and Markov chain Monte Carlo settings
☒	☐	For hierarchical and complex designs, identification of the appropriate level for tests and full reporting of outcomes
☐	☒	Estimates of effect sizes (e.g. Cohen's *d*, Pearson's *r*), indicating how they were calculated

Our web collection on statistics for biologists contains articles on many of the points above.

Software and code

Policy information about availability of computer code

Data collection	Philips CM100 transmission electron microscope (FEI, Hillsboro, OR), microscope (Olympus IX83, Tokyo, Japan), confocal microscope (Leica SP8, Wetzlar, Germany)
Data analysis	Image J 1.53, Prism v7, Image Lab 6.1, R4.2.0, FastQC (v0.11.9), MultiQC (v1.9), QIIME2

For manuscripts utilizing custom algorithms or software that are central to the research but not yet described in published literature, software must be made available to editors and reviewers. We strongly encourage code deposition in a community repository (e.g. GitHub). See the Nature Portfolio guidelines for submitting code & software for further information.

Data

Policy information about availability of data

All manuscripts must include a data availability statement. This statement should provide the following information, where applicable:

- Accession codes, unique identifiers, or web links for publicly available datasets
- A description of any restrictions on data availability
- For clinical datasets or third party data, please ensure that the statement adheres to our policy

All data is available from the corresponding author upon request. All related raw data has been deposited to the public database with accession code.

Human research participants

Policy information about studies involving human research participants and Sex and Gender in Research.

Reporting on sex and gender	N/ A
Population characteristics	N/ A
Recruitment	N/ A
Ethics oversight	N /A

Note that full information on the approval of the study protocol must also be provided in the manuscript.

Field-specific reporting

Please select the one below that is the best fit for your research. If you are not sure, read the appropriate sections before making your selection.

☒ Life sciences ☐ Behavioural & social sciences ☐ Ecological, evolutionary & environmental sciences

For a reference copy of the document with all sections, see nature.com/documents/nr-reporting-summary-flat.pdf

Life sciences study design

All studies must disclose on these points even when the disclosure is negative.

Sample size	No statistical methods were used to pre-determine sample size, but our sample sizes are similar to previous reports
Data exclusions	No data points were excluded from the analysis.
Replication	Mice experiments were replicated depends on sample size; for evaluation of micrographs, at least three random area was selected and evaluated and got similar results.
Randomization	Mice used in the experiments were litter mates and age-matched, and randomized into control and experimental groups
Blinding	Data collection and analysis were not performed in a blinded manner.

Reporting for specific materials, systems and methods

We require information from authors about some types of materials, experimental systems and methods used in many studies. Here, indicate whether each material, system or method listed is relevant to your study. If you are not sure if a list item applies to your research, read the appropriate section before selecting a response.

Materials & experimental systems

n/a	Involved in the study	
☐	☒	Antibodies
☒	☐	Eukaryotic cell lines
☒	☐	Palaeontology and archaeology
☐	☒	Animals and other organisms
☒	☐	Clinical data
☒	☐	Dual use research of concern

Methods

n/a	Involved in the study	
☒	☐	ChIP-seq
☒	☐	Flow cytometry
☒	☐	MRI-based neuroimaging

Antibodies

Antibodies used	E-Cadherin (#4A2, Cell Signaling Technology? Dilution 1:1000), Claudin-1 (2H10D10, Invitrogen, Dilution 1:300), β-Actin (#13E5, Cell Signaling Technology,Dilution 1:1000), GAPDH (#D 16H11, Cell signaling Technology,Dilution 1:1000), Phospho-NF-κB p65 (93H1, Cell signaling technology,Dilution 1:1000), NF-κB p65 (L8F6, Cell signaling technology,Dilution 1:1000), and Lamin A/C (4C11, Cell signaling technology,Dilution 1:1000).
Validation	The antibodies were all commercially available and validated by respective companies.

Animals and other research organisms

Policy information about studies involving animals; ARRIVE guidelines recommended for reporting animal research, and Sex and Gender in Research

Laboratory animals	Description of research mice used for experiments can be found in the relevant figure legends and Methods
Wild animals	NA
Reporting on sex	All animal used in this study were male mice.
Field-collected samples	NA
Ethics oversight	All these mouse experiments in this study have been approved by the Animal Experimentation Ethics Committee of the Chinese University of Hong Kong.

Note that full information on the approval of the study protocol must also be provided in the manuscript.
