# Supplementary material for: Parabacteroides distasonis uses dietary inulin to suppress NASH via its metabolite pentadecanoic acid
Source: Nat Microbiol. 2023 Jun 29;8(8):1534–48. doi: 10.1038/s41564-023-01418-7 (PMC10390331; doi:10.1038/s41564-023-01418-7)

Source images for Figure 1

Including H&E staining and sirius red staining
Replicate images from a same mouse were displayed within a same page

H&E Staining

NCD group

(10 mice were included)

NCD-1

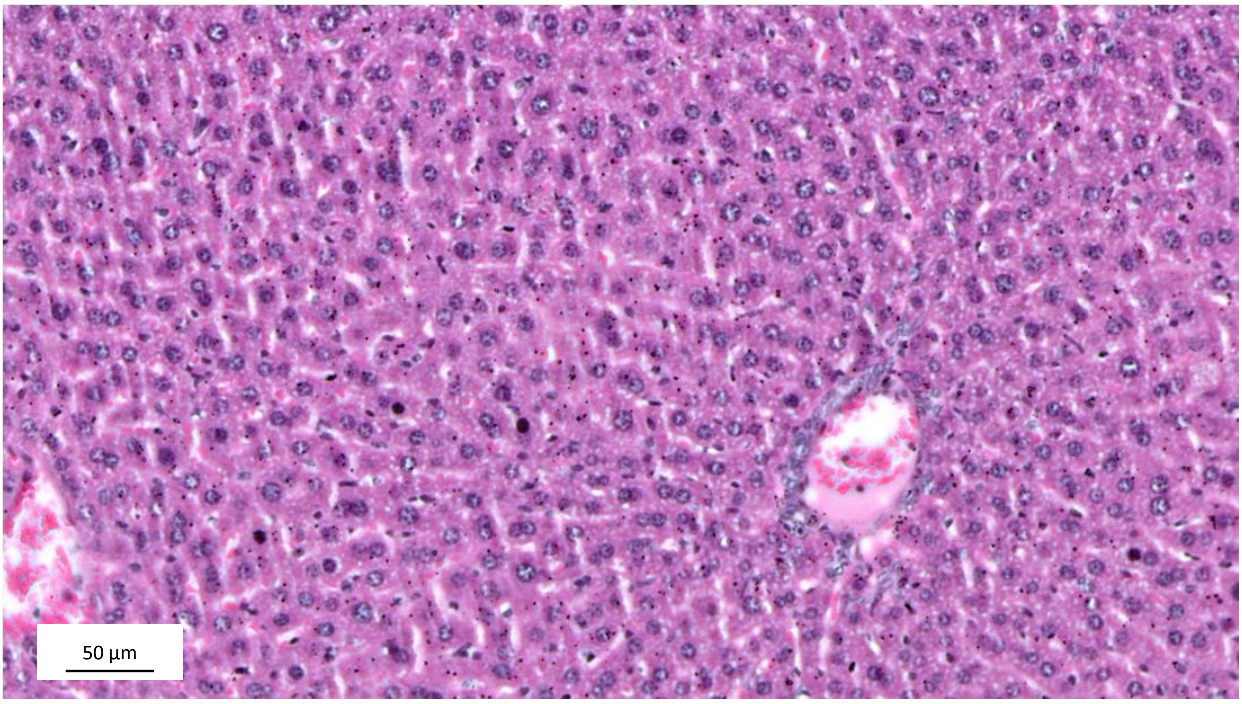

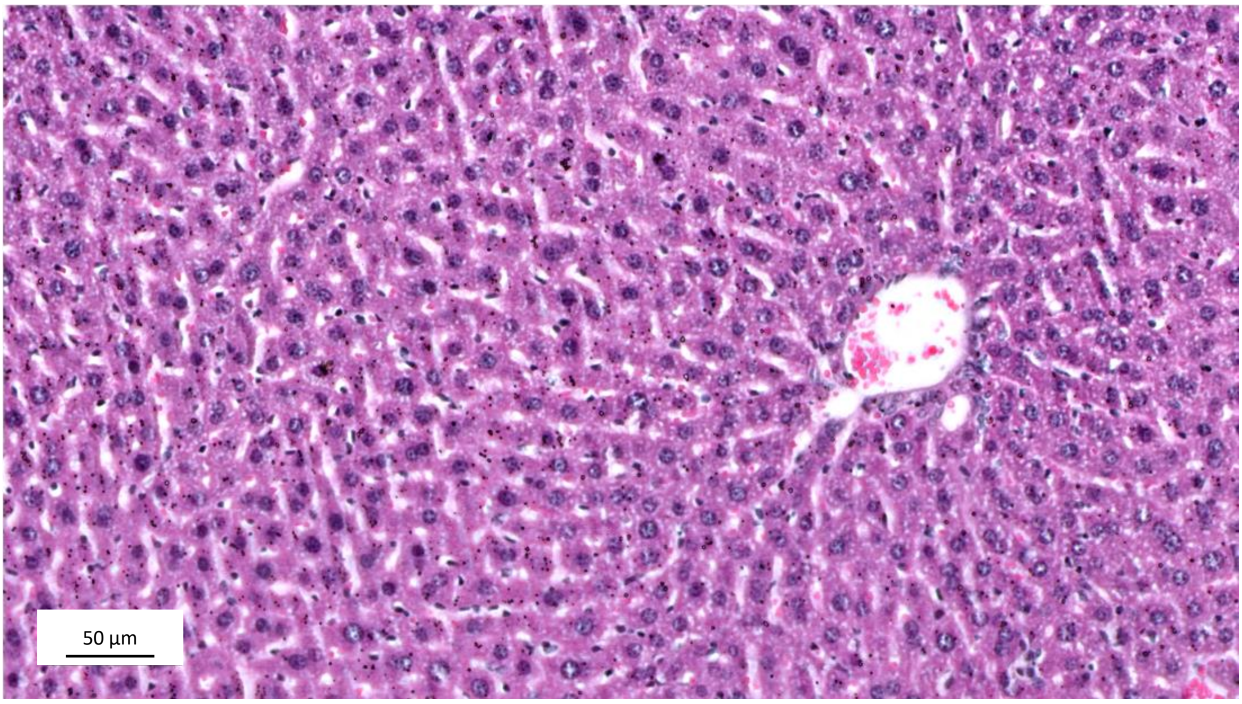

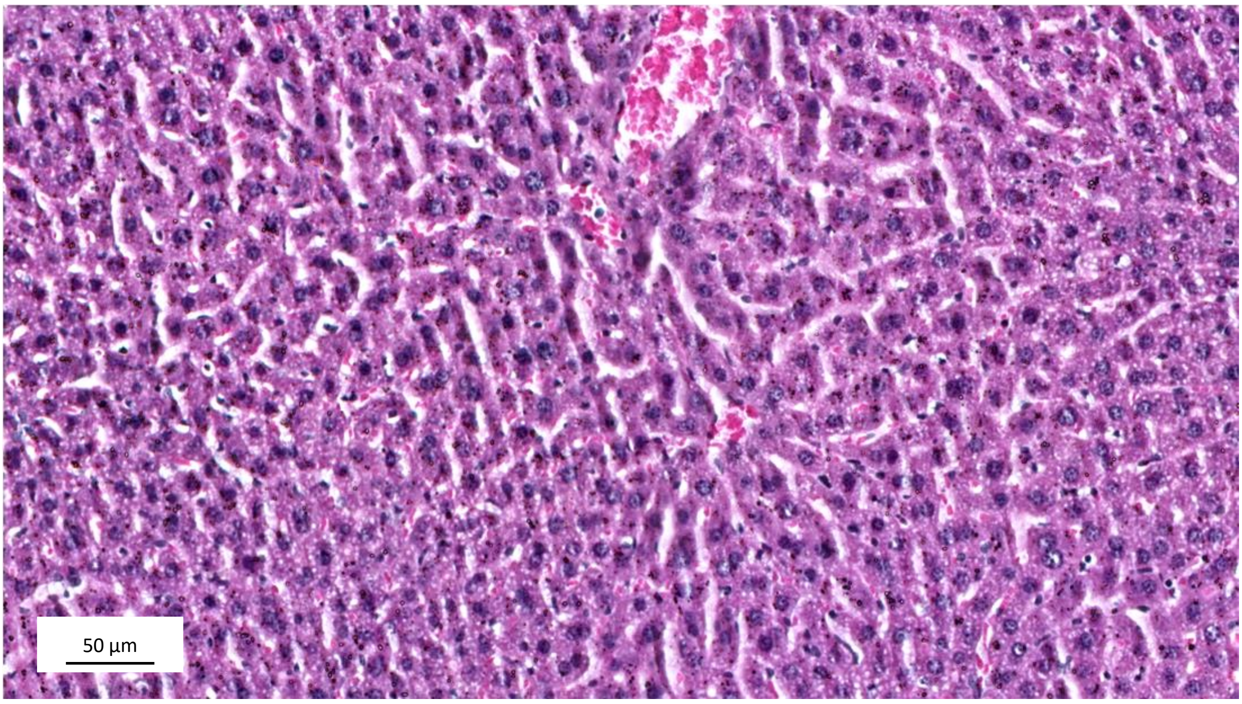

NCD-2

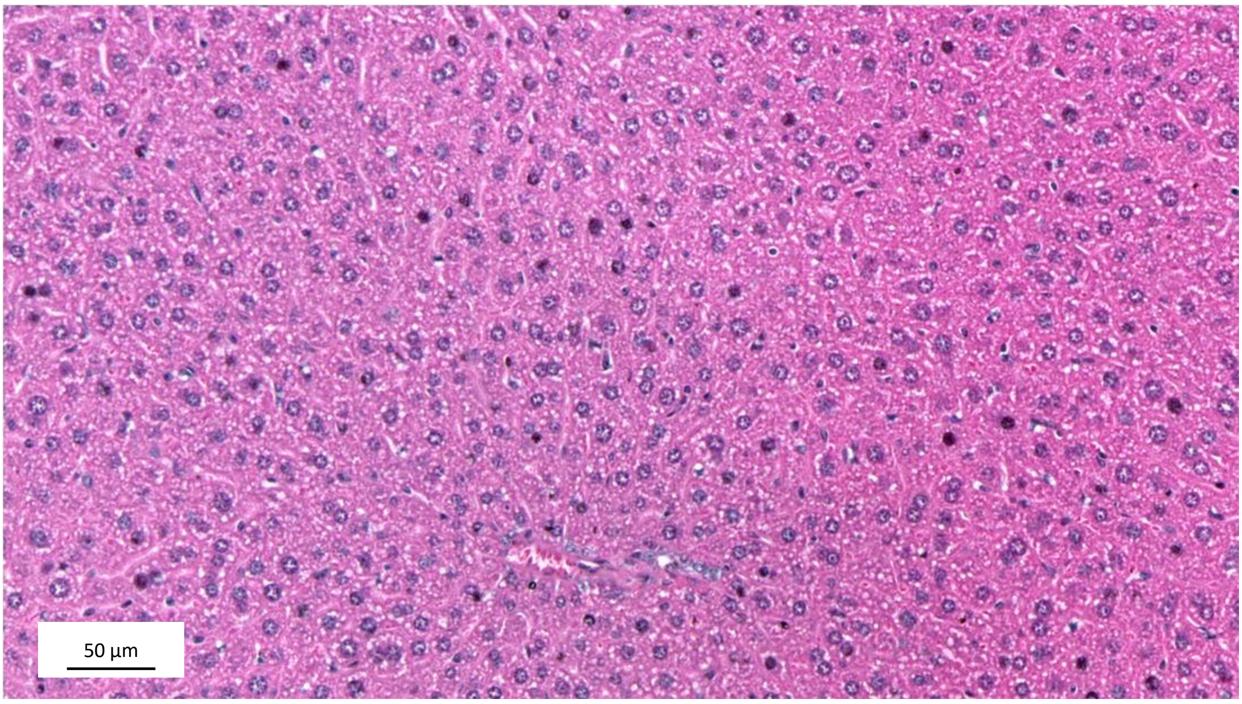

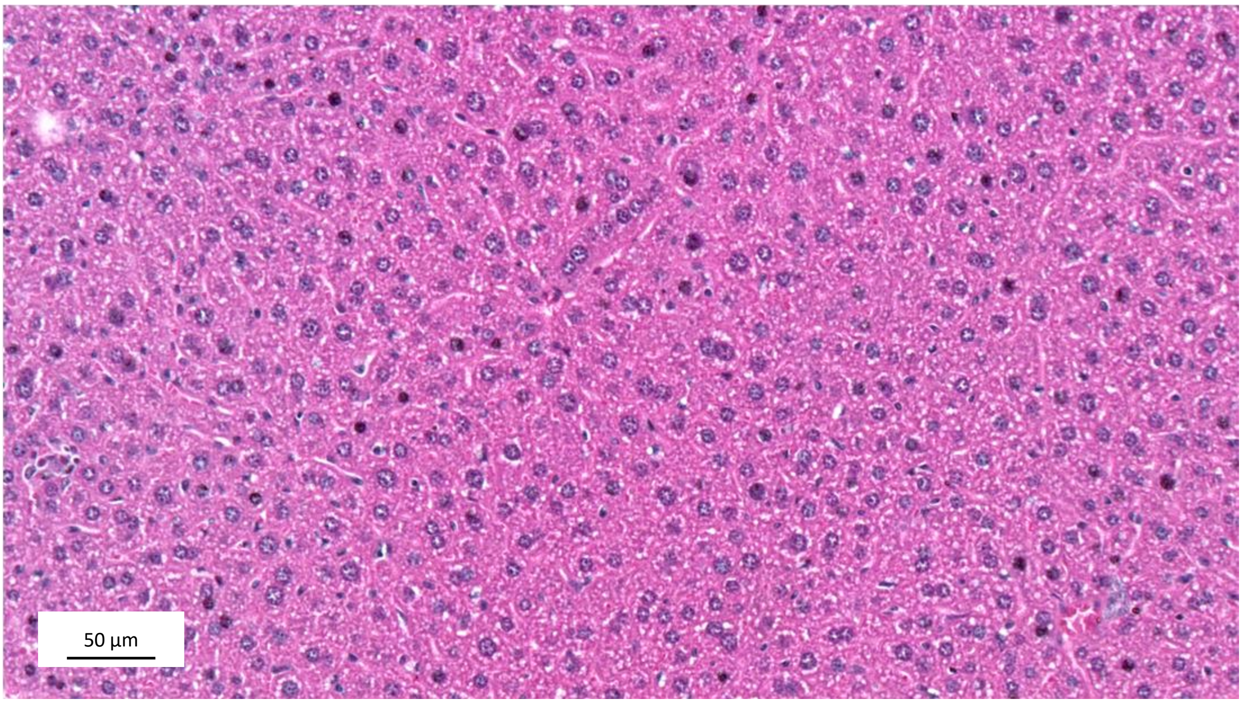

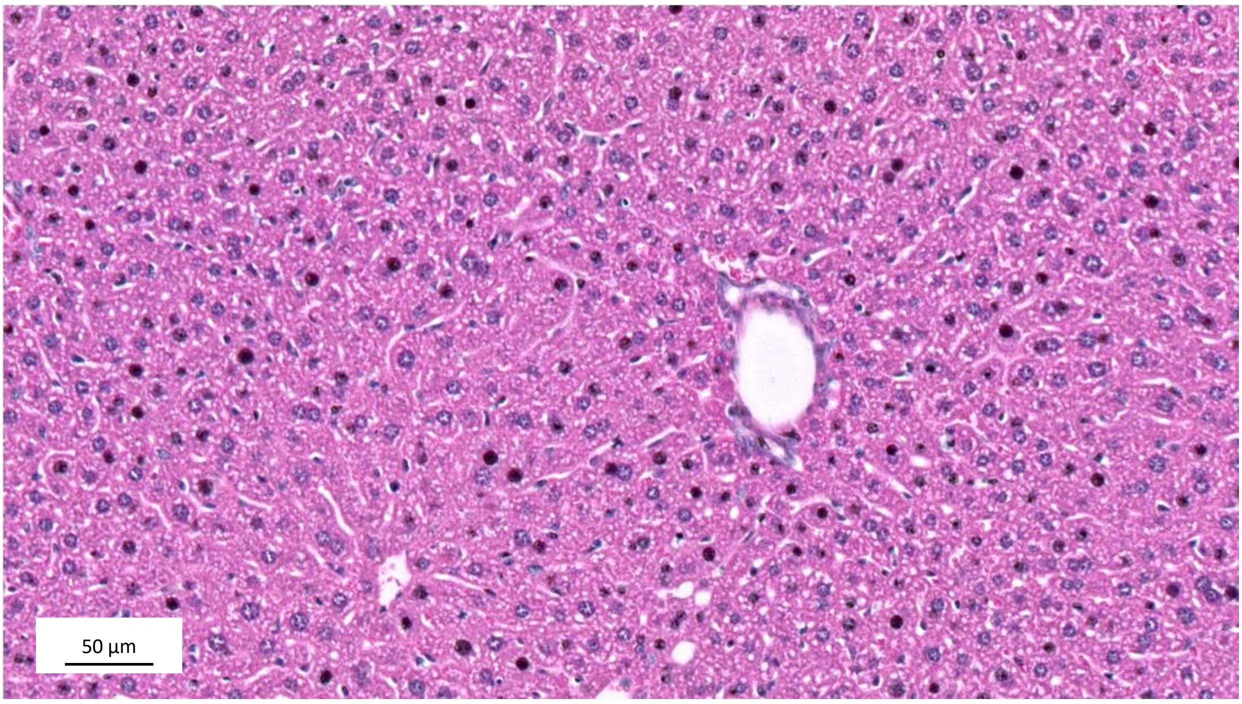

NCD-3

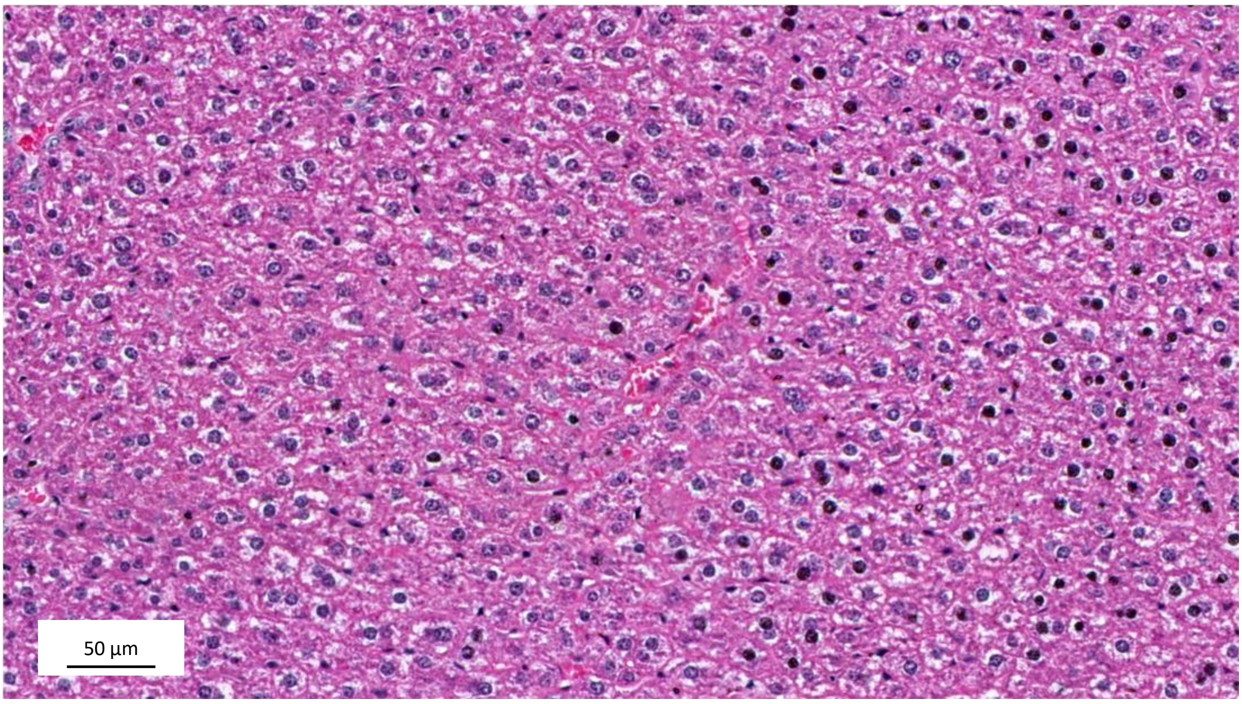

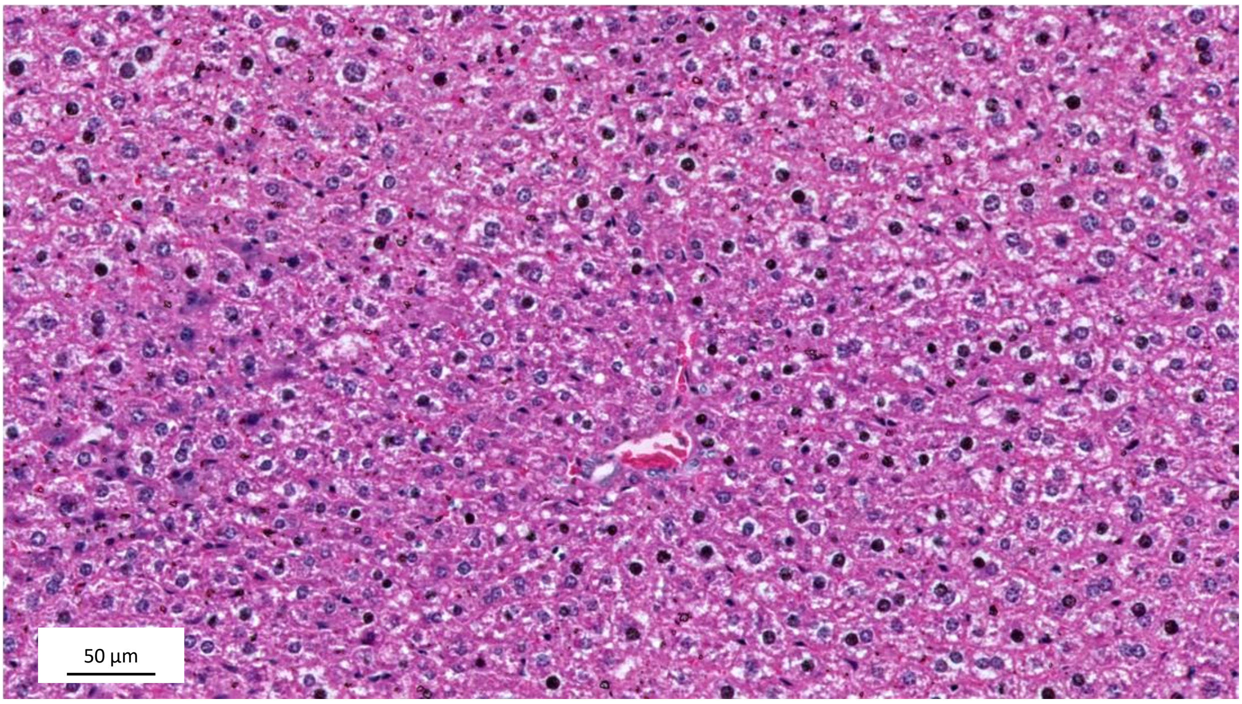

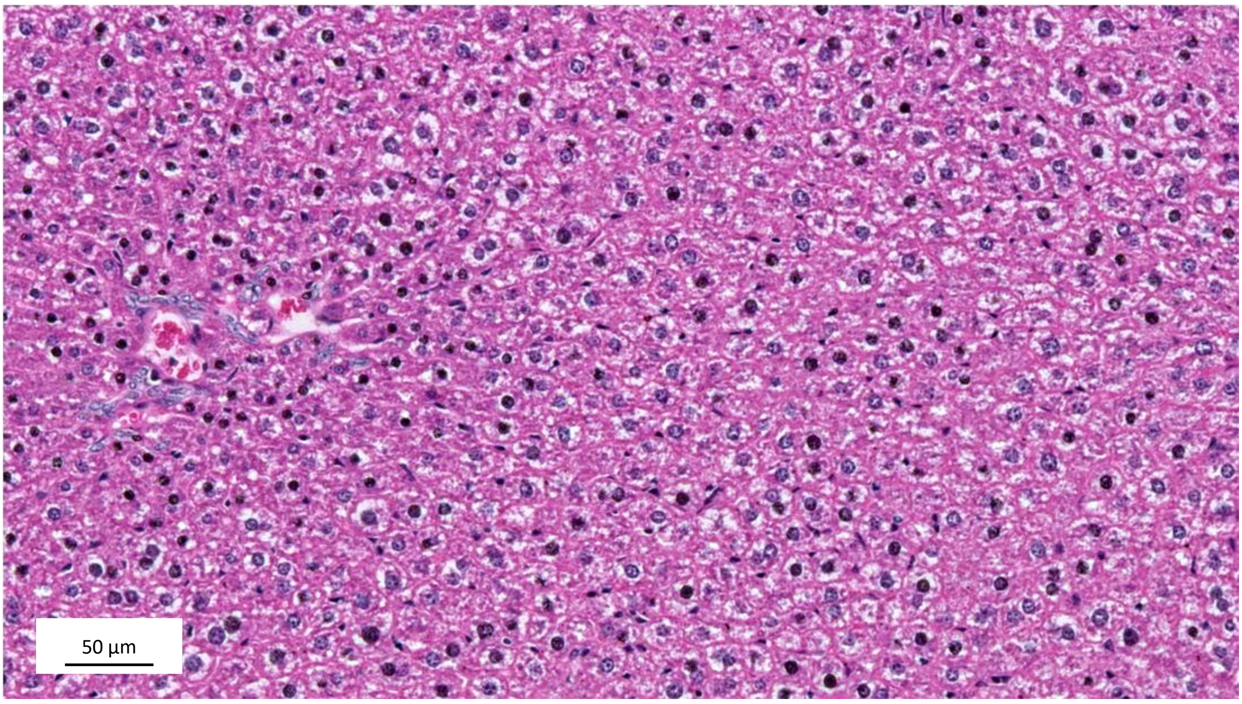

NCD-4

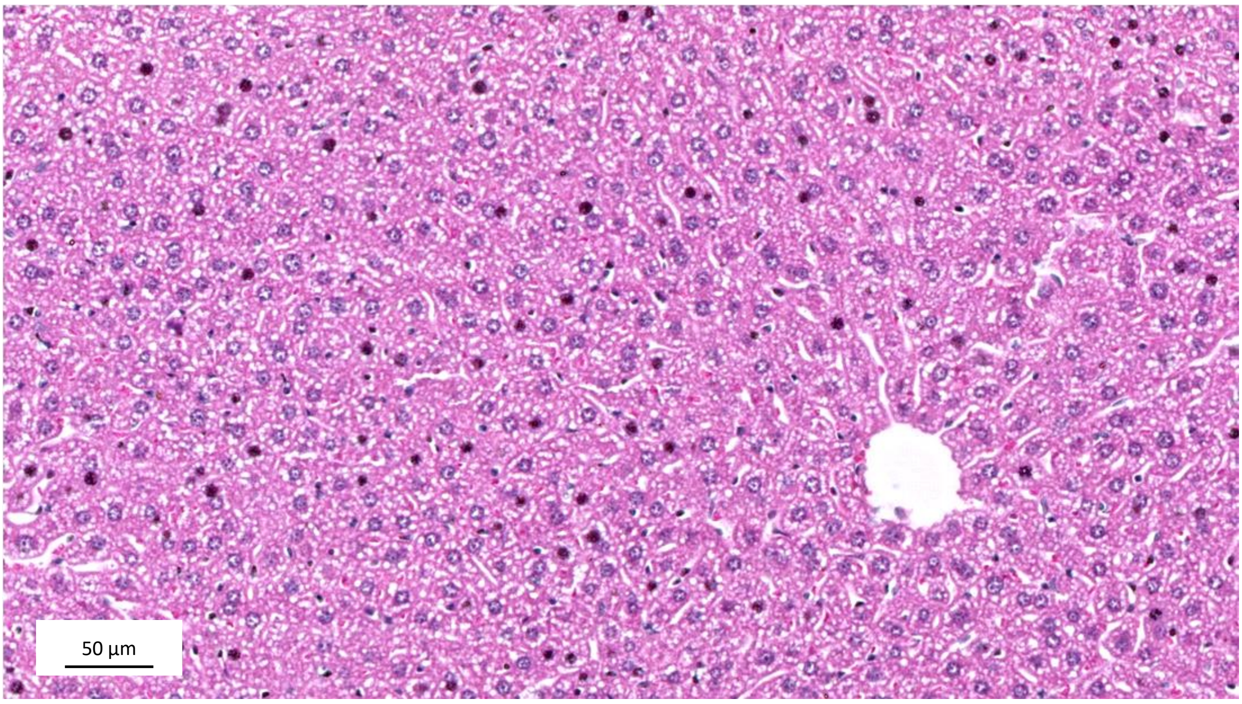

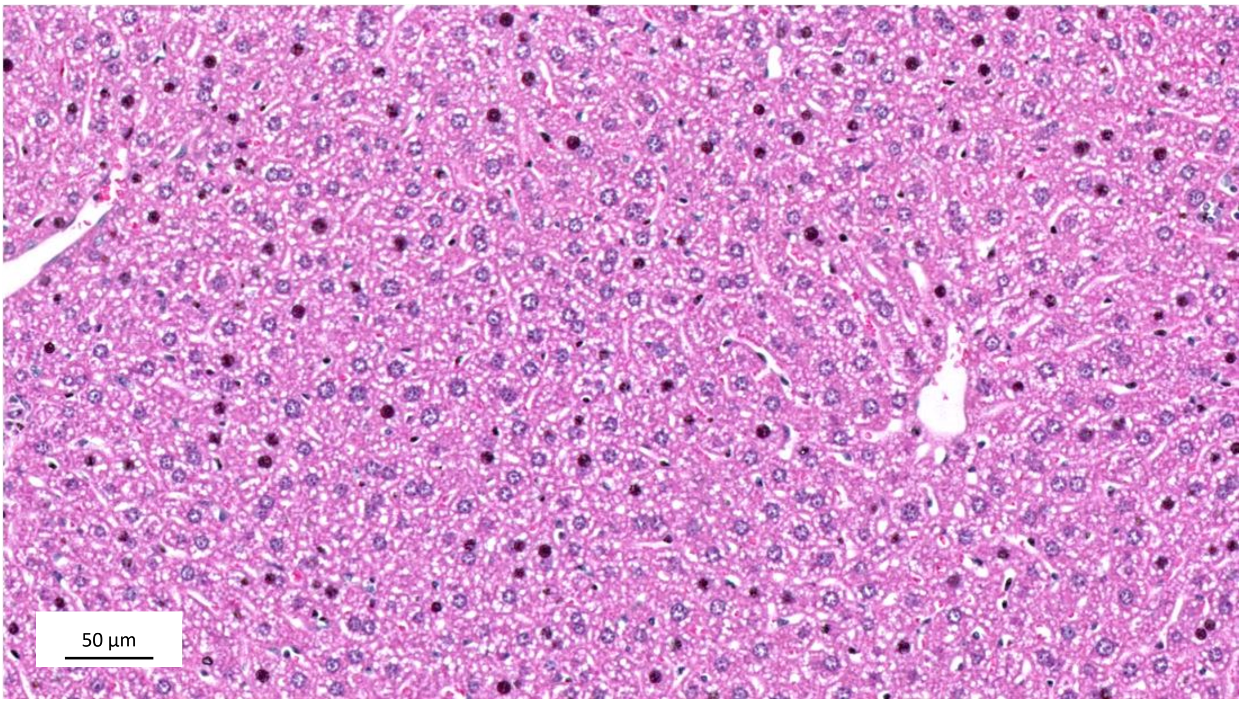

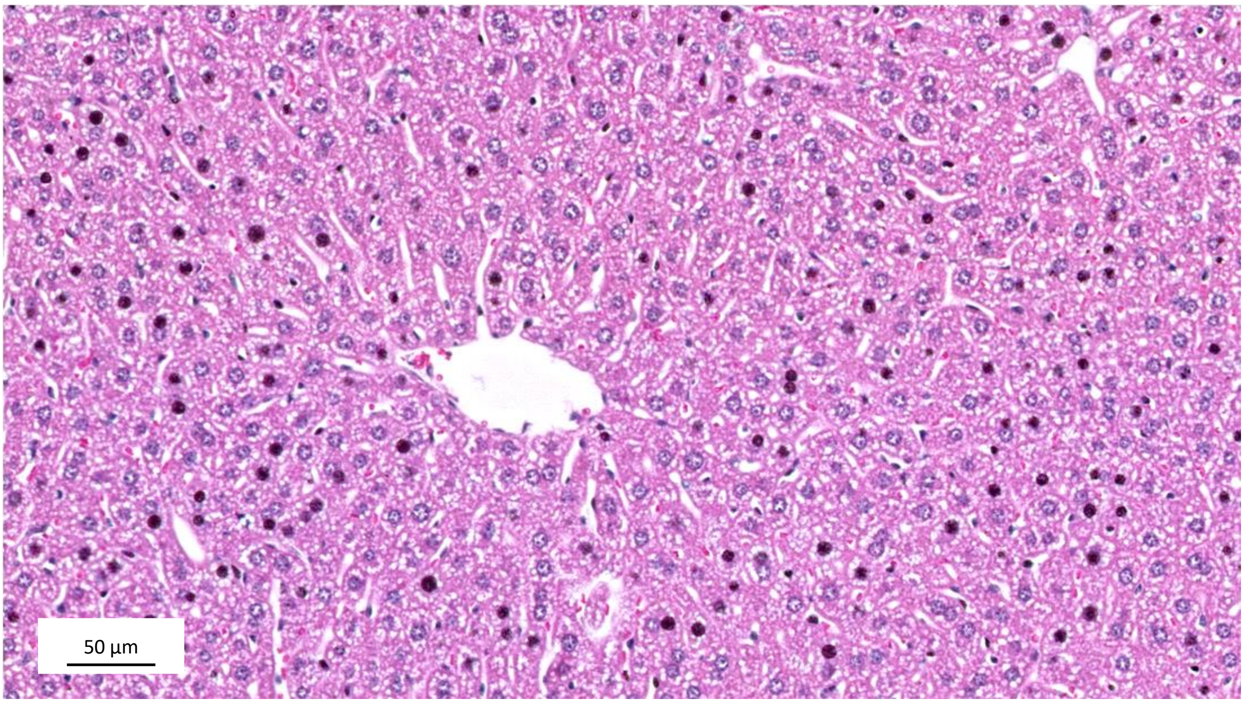

NCD-5

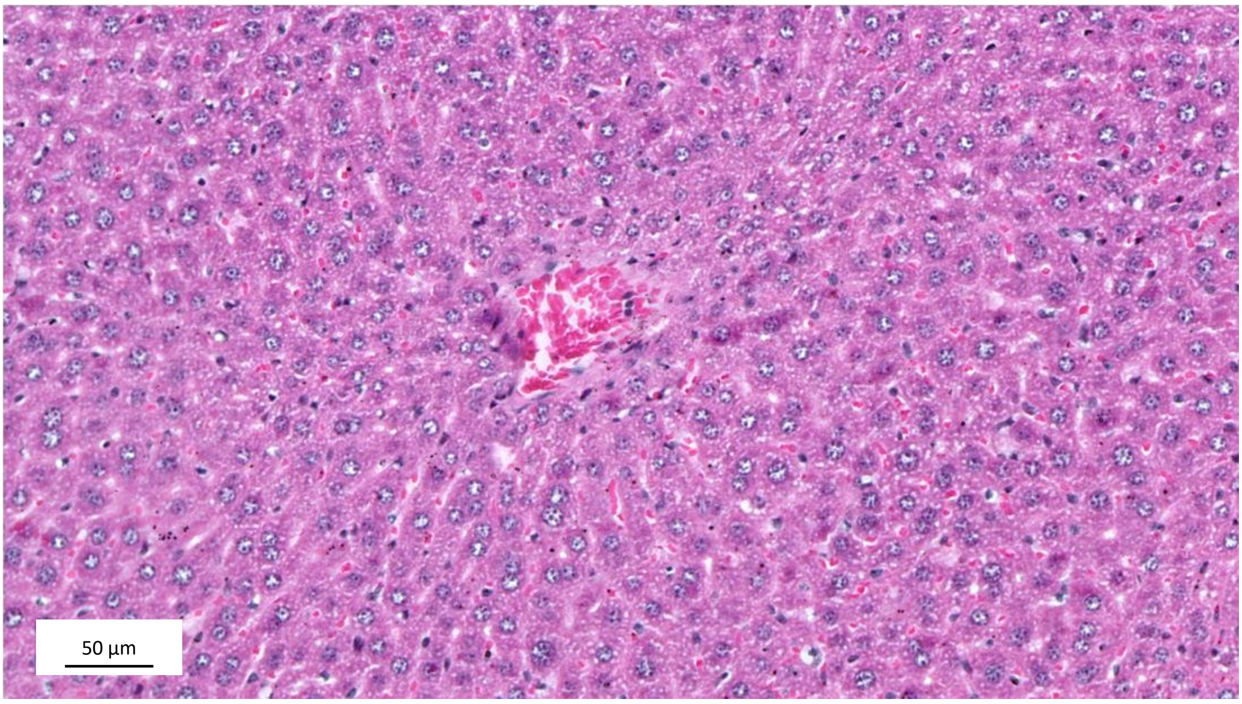

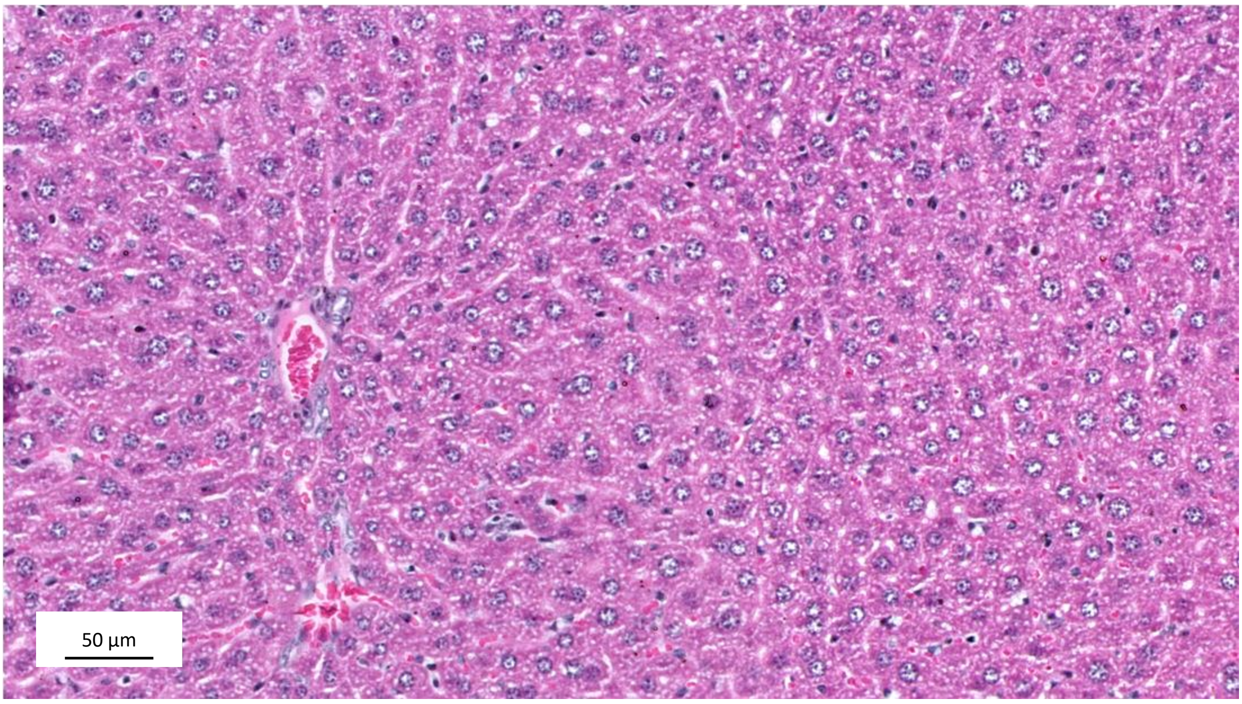

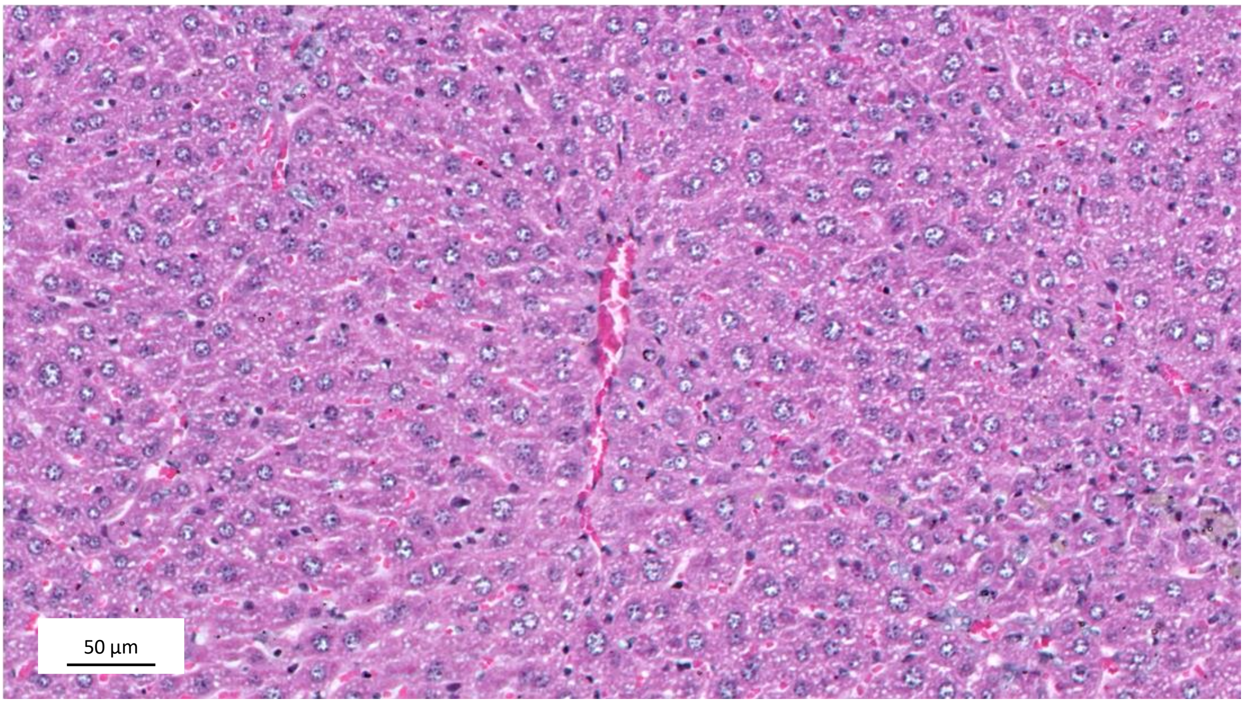

NCD-6

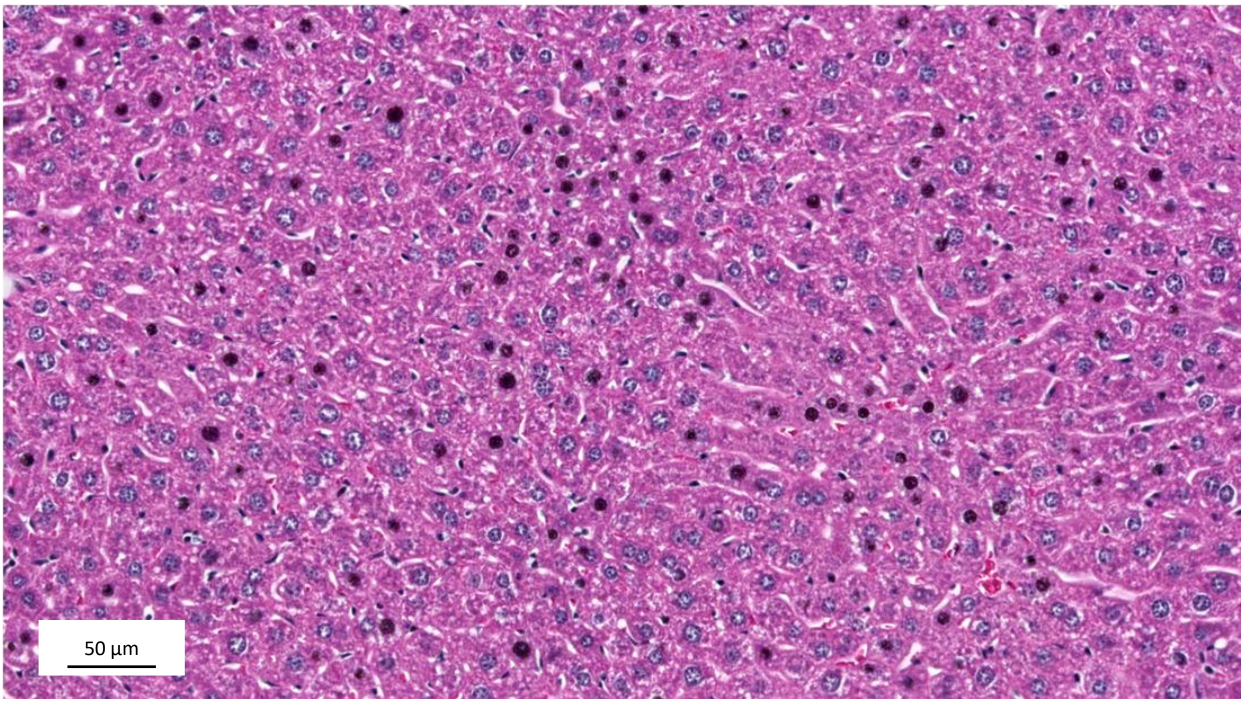

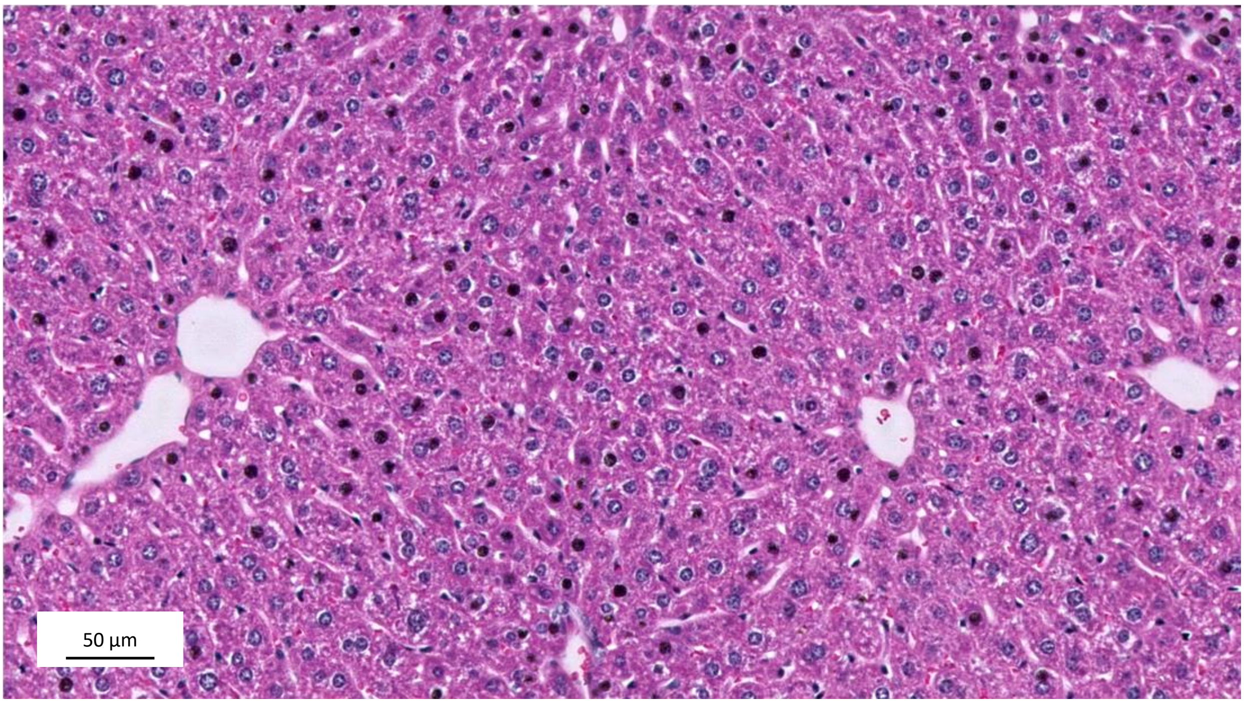

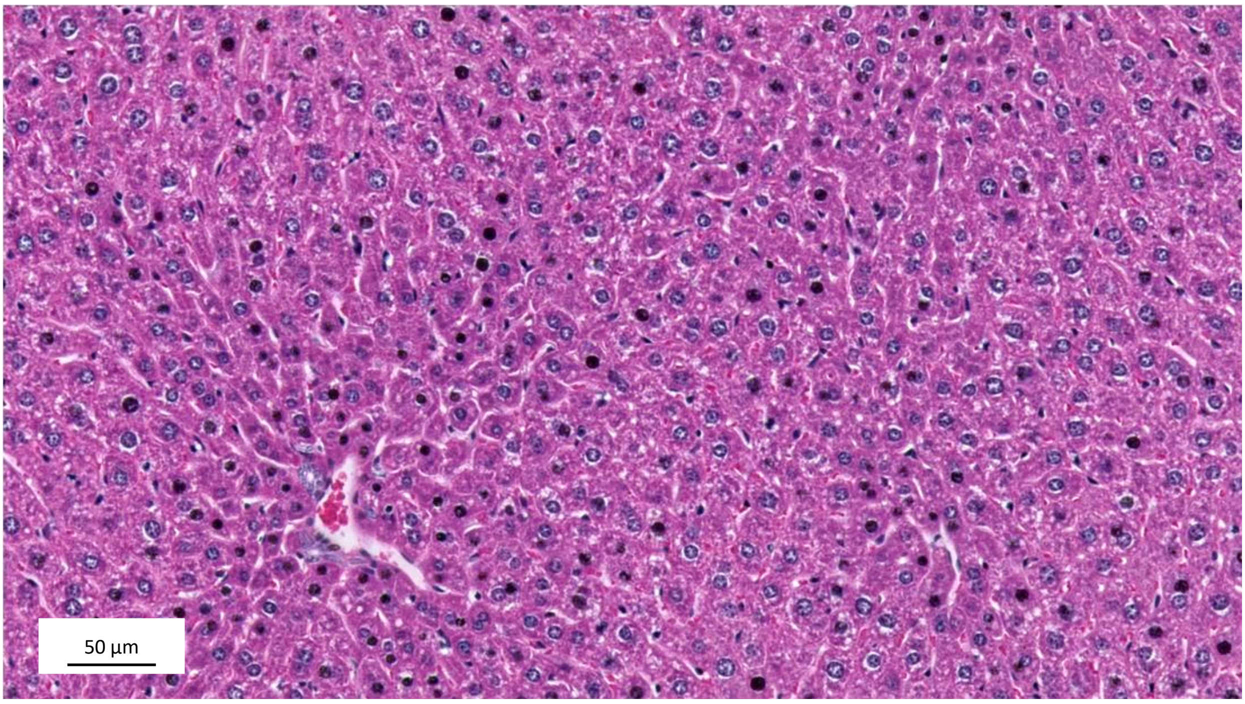

NCD-7

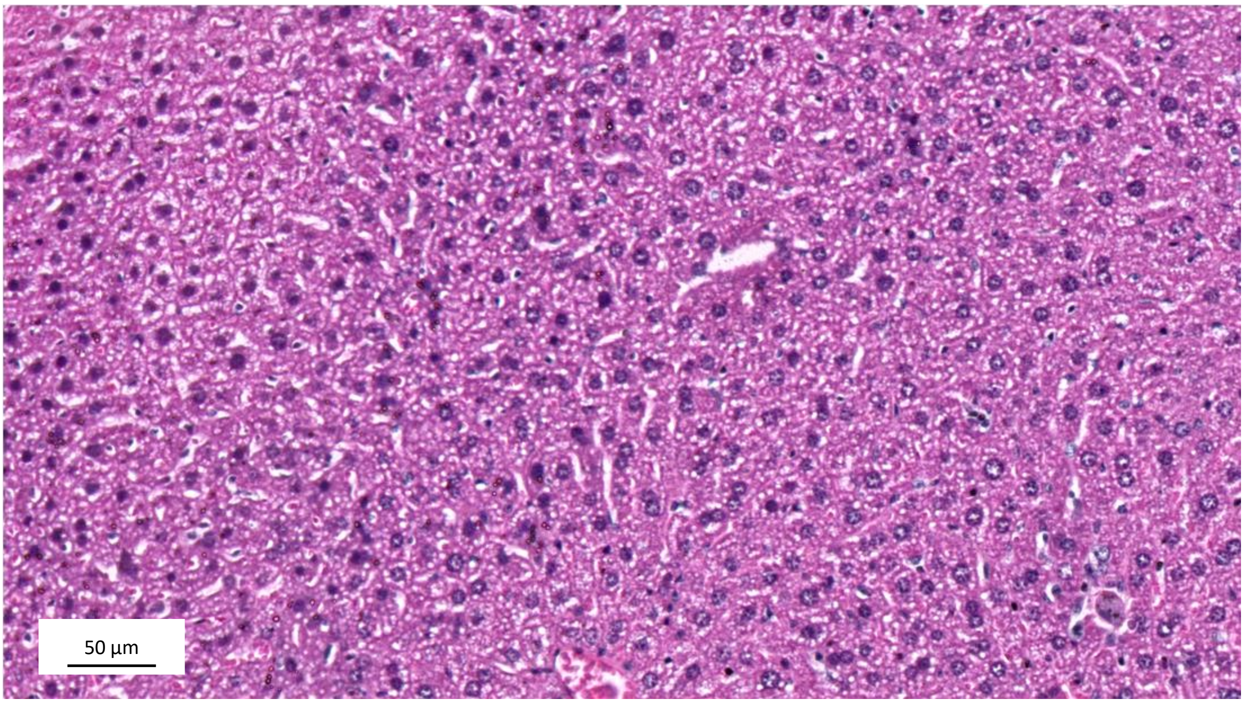

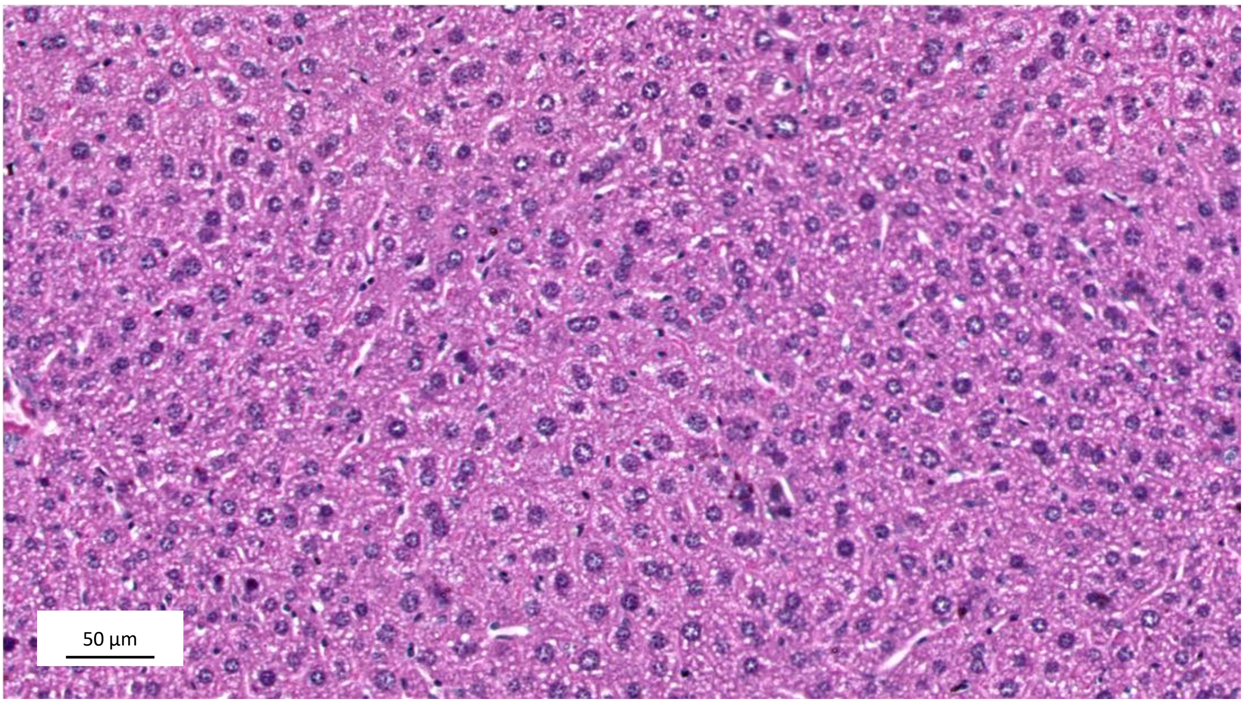

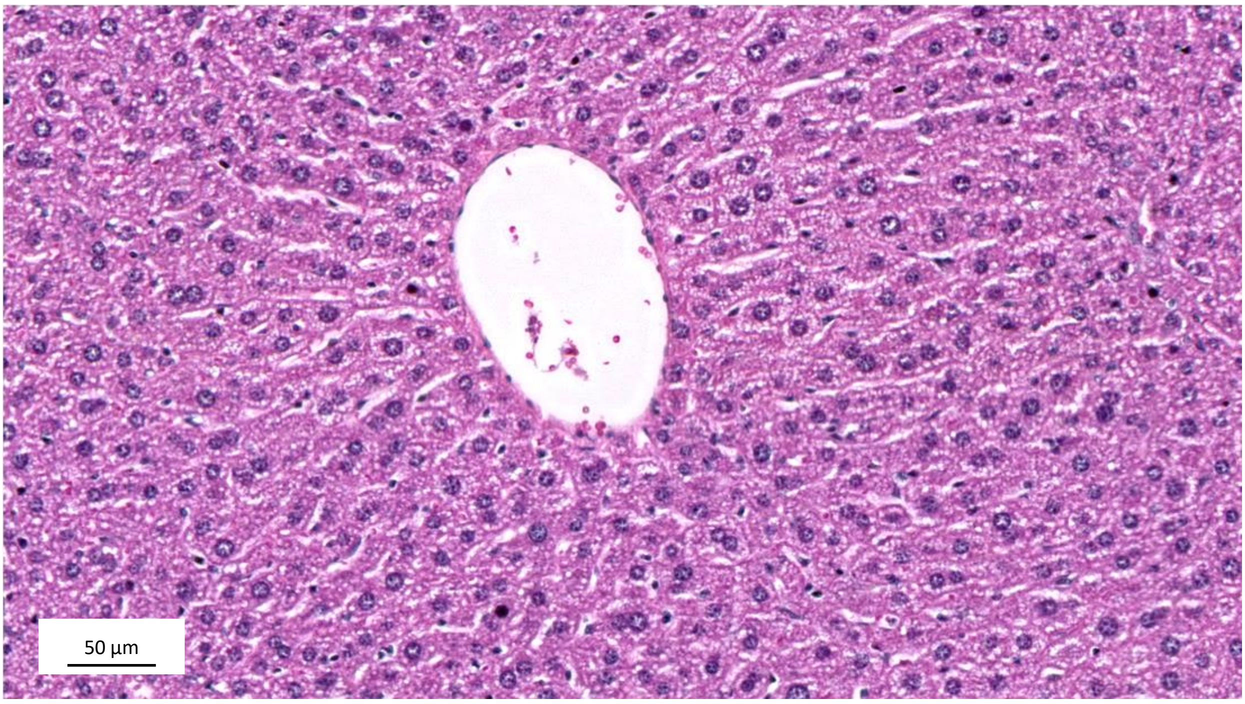

NCD-8

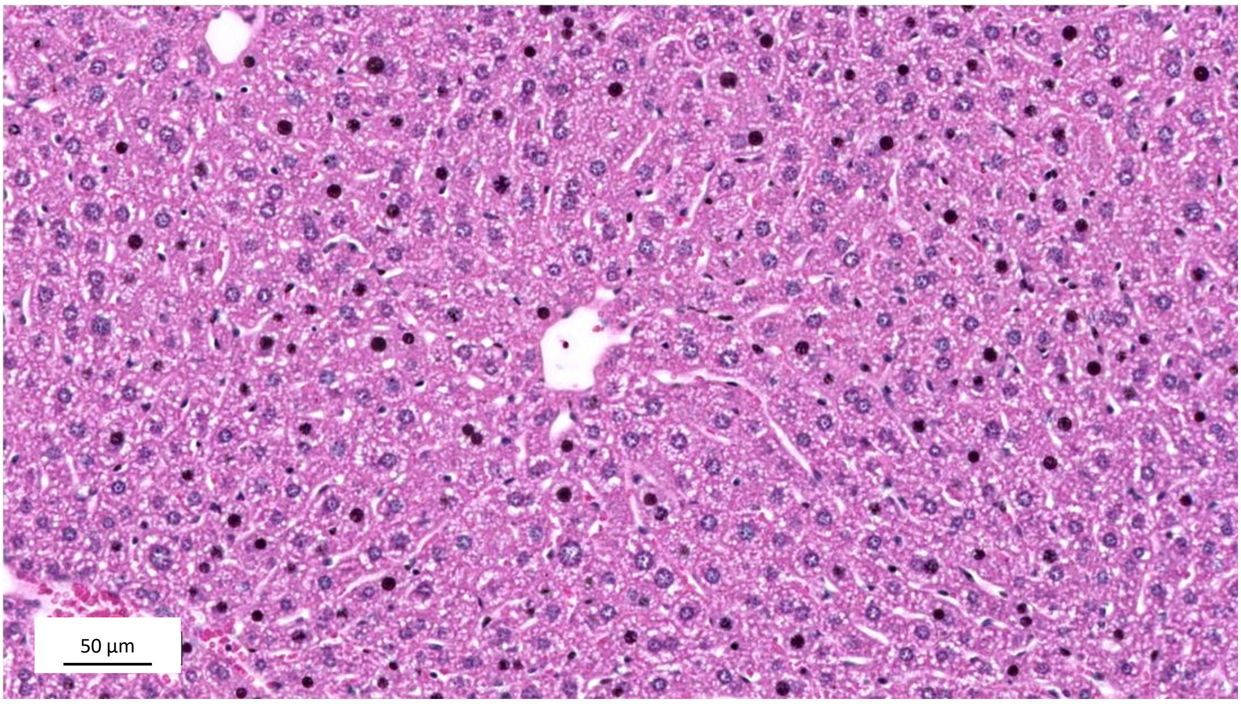

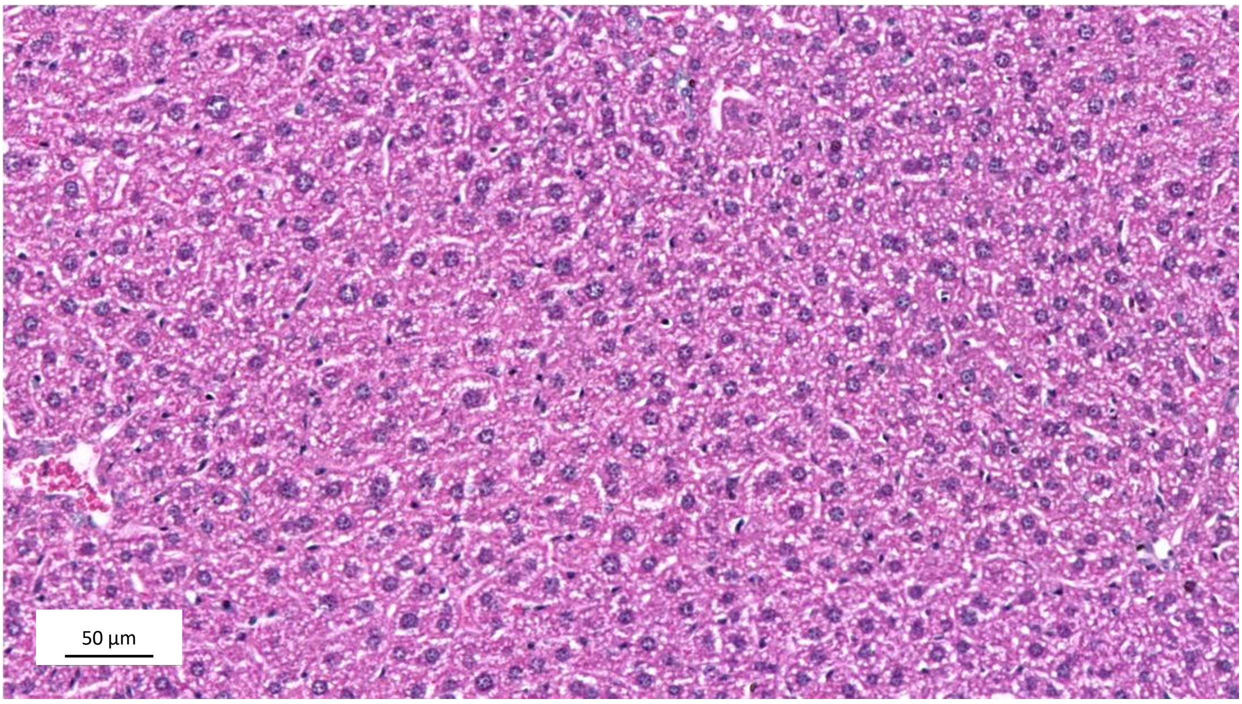

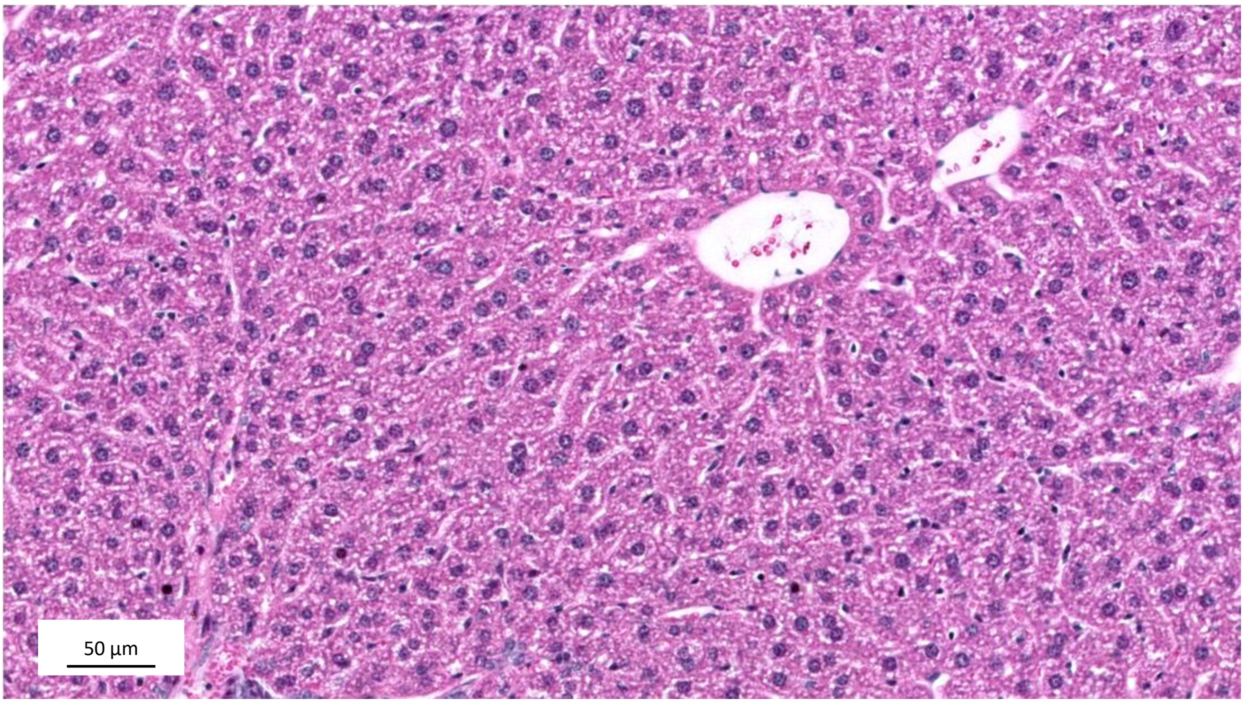

NCD-9

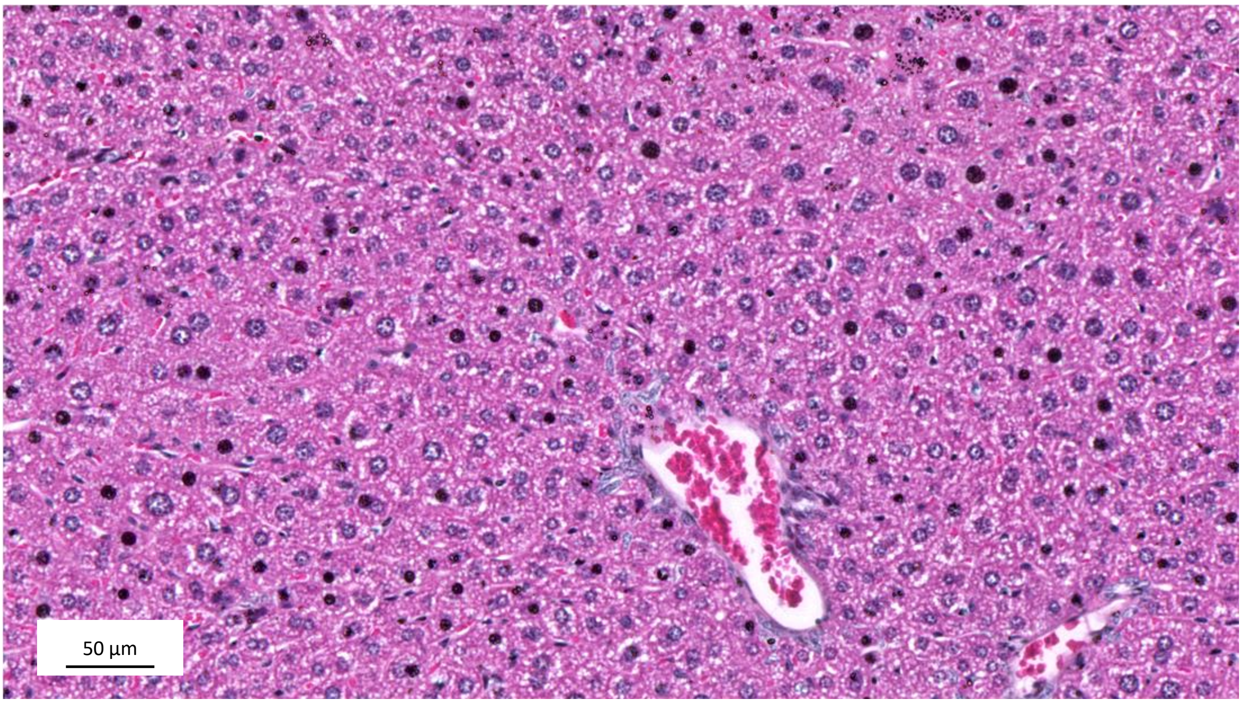

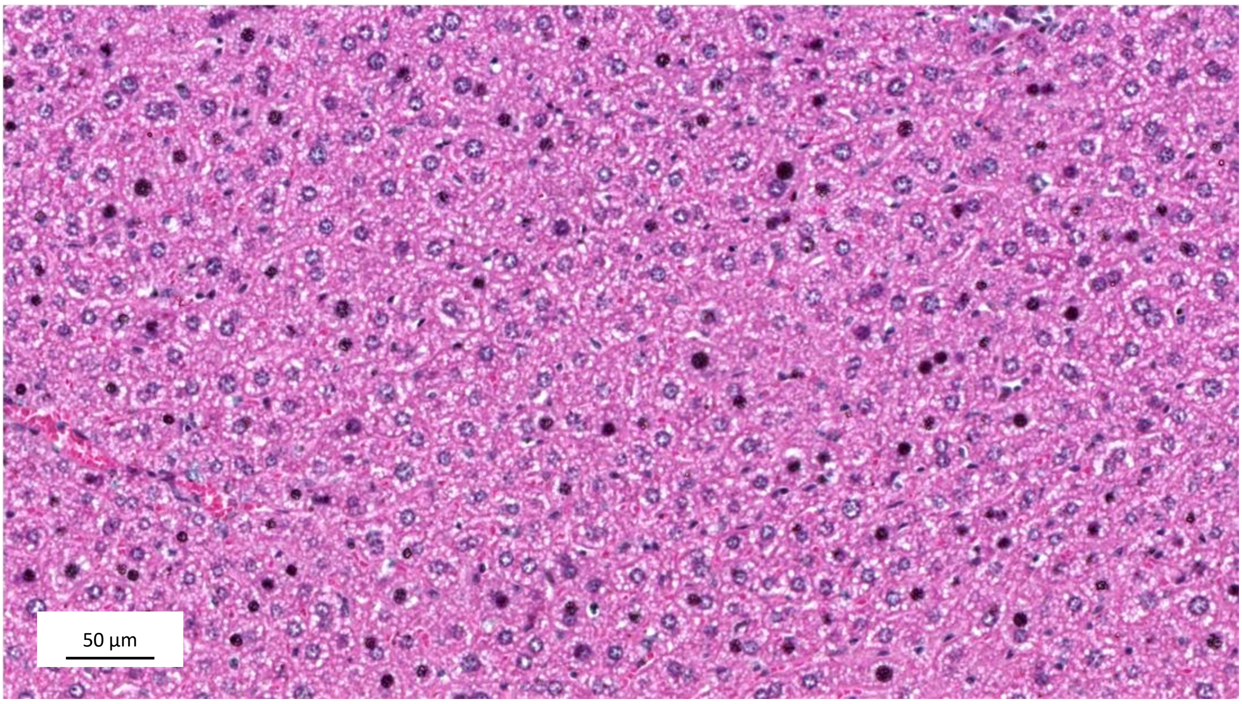

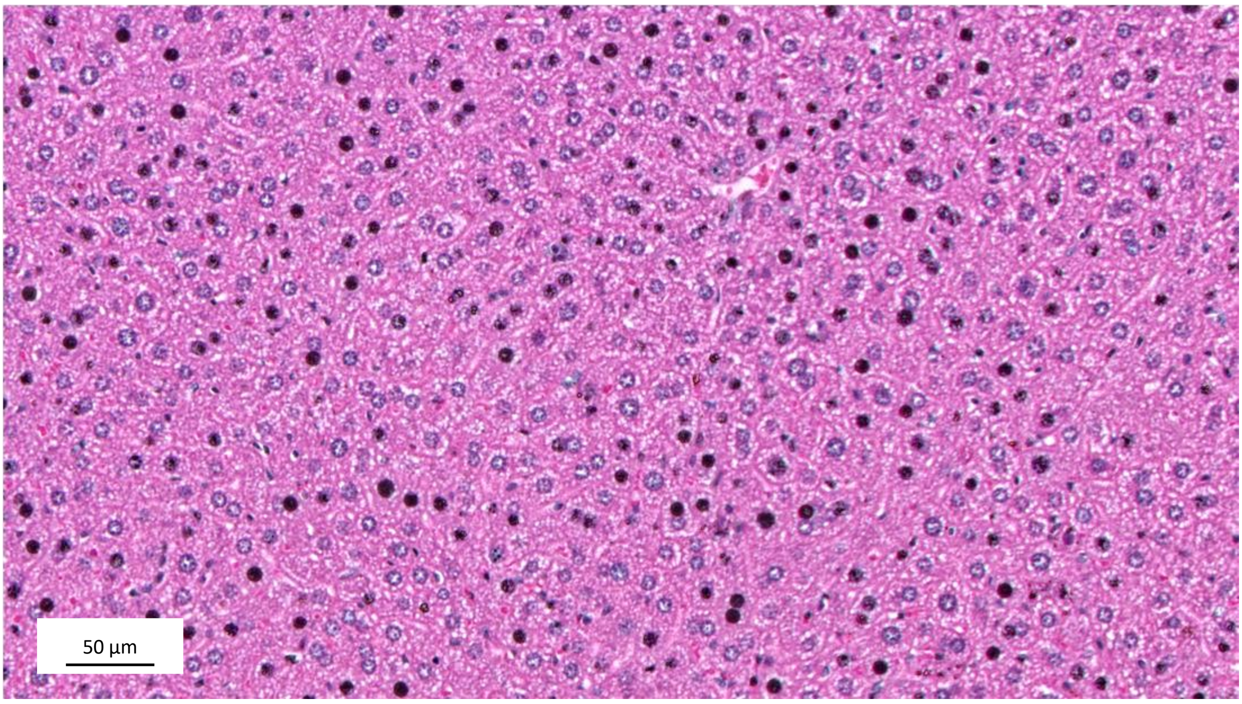

NCD-10

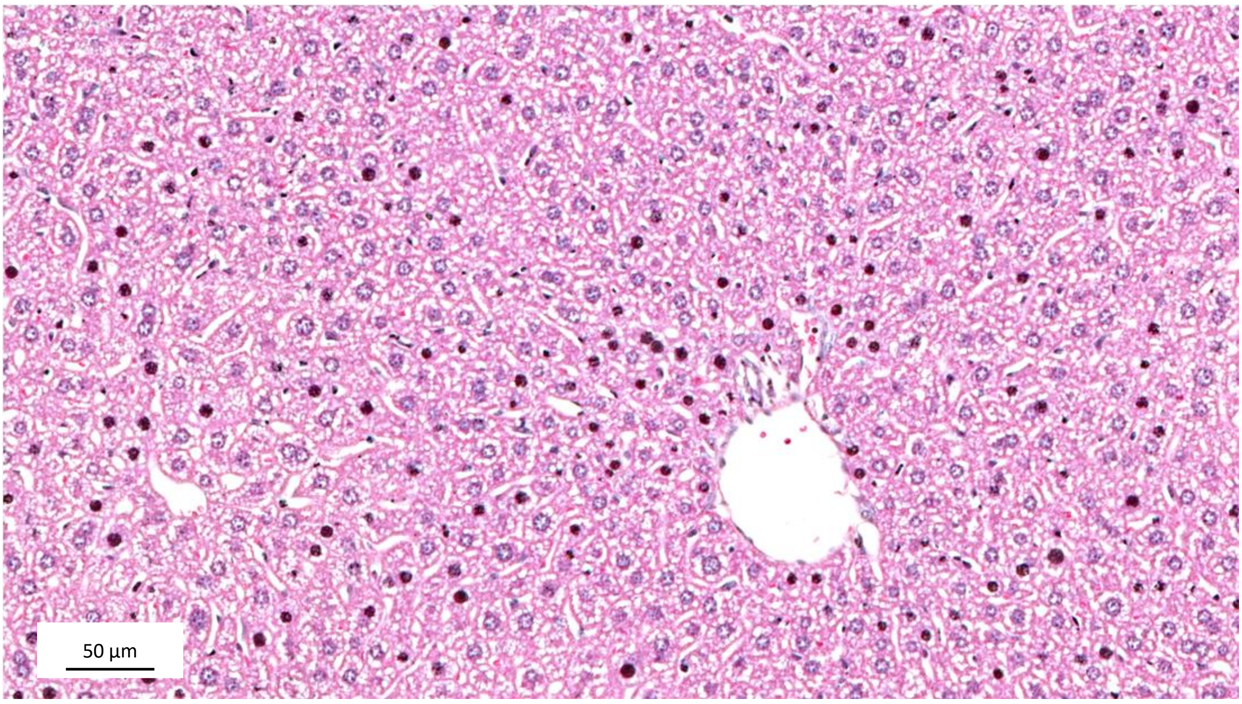

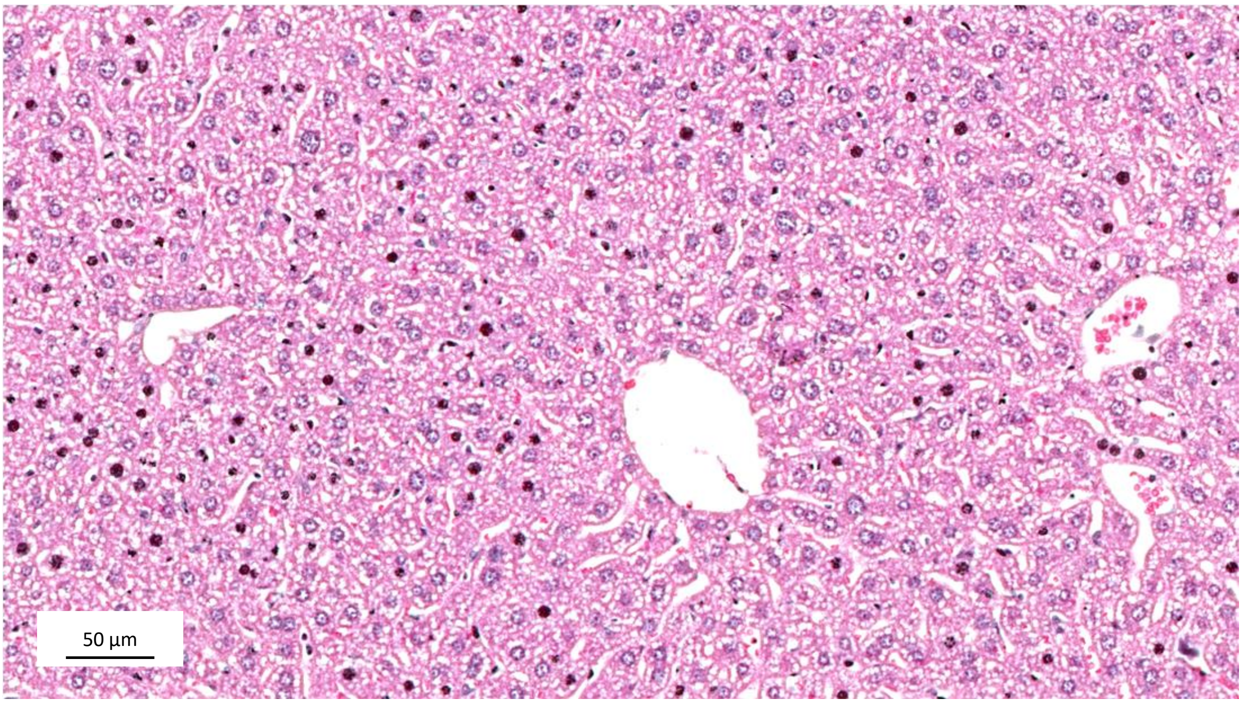

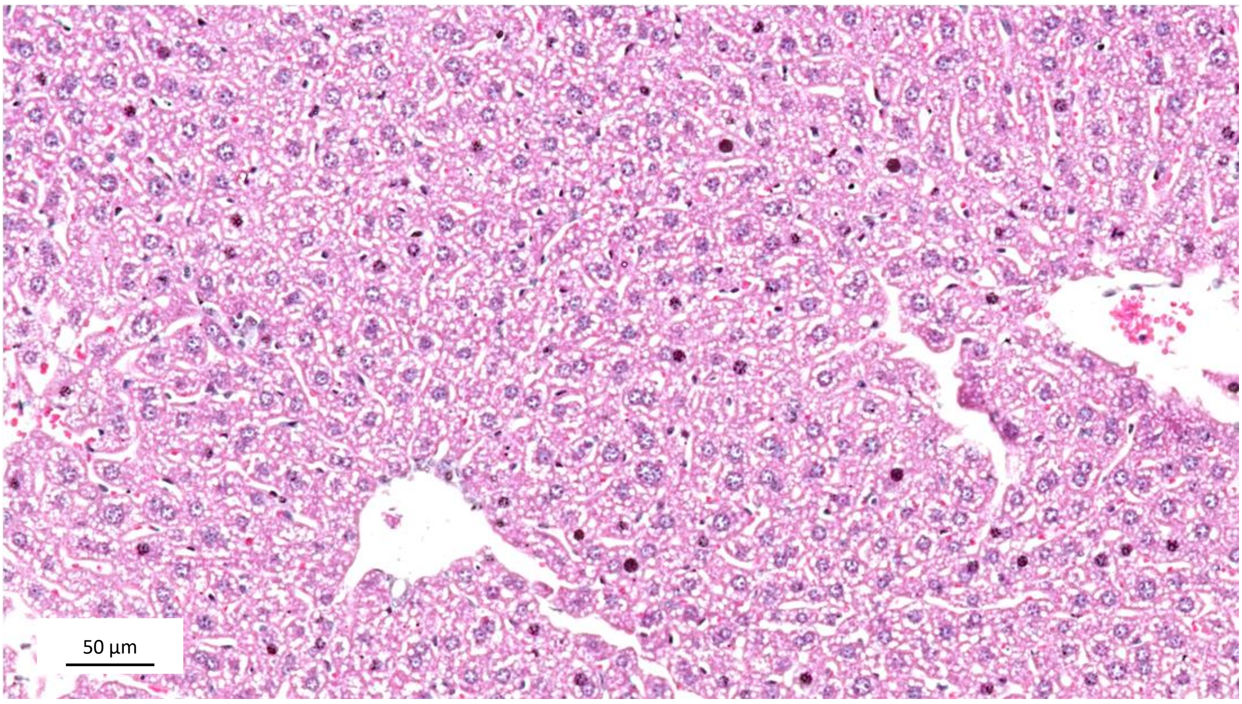

H&E Staining

CDHFD group

(13 mice were included)

CDHFD-1

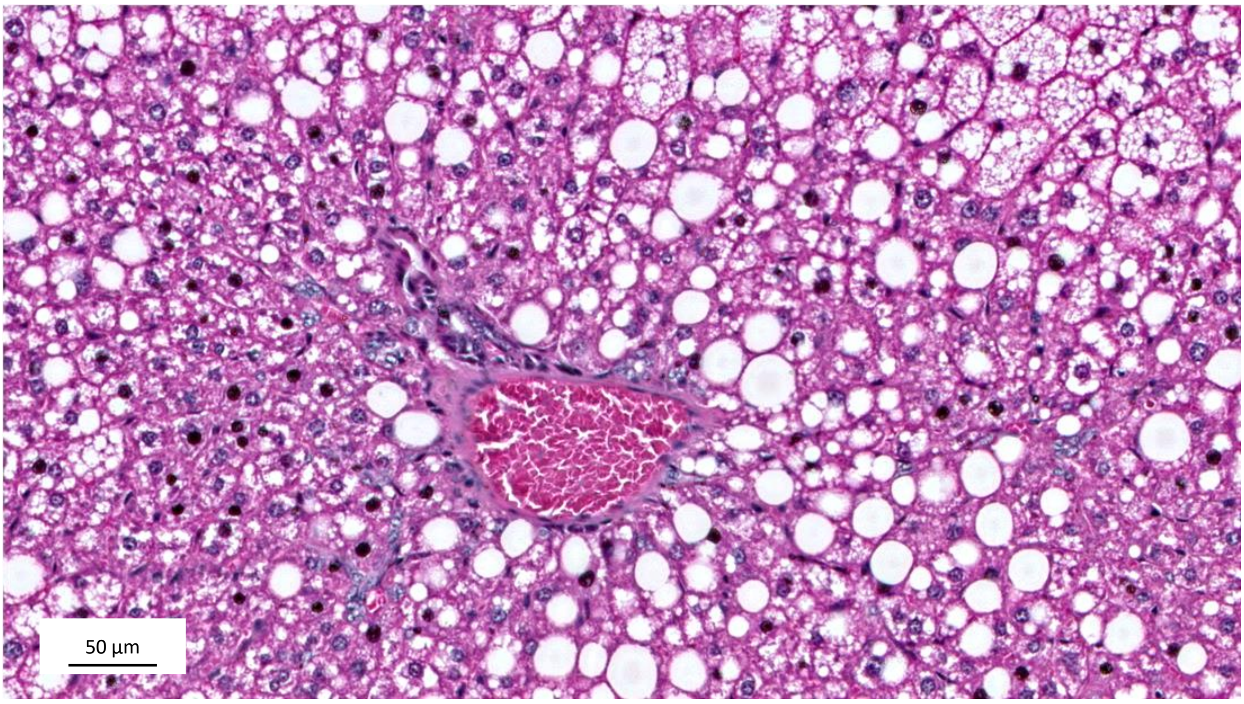

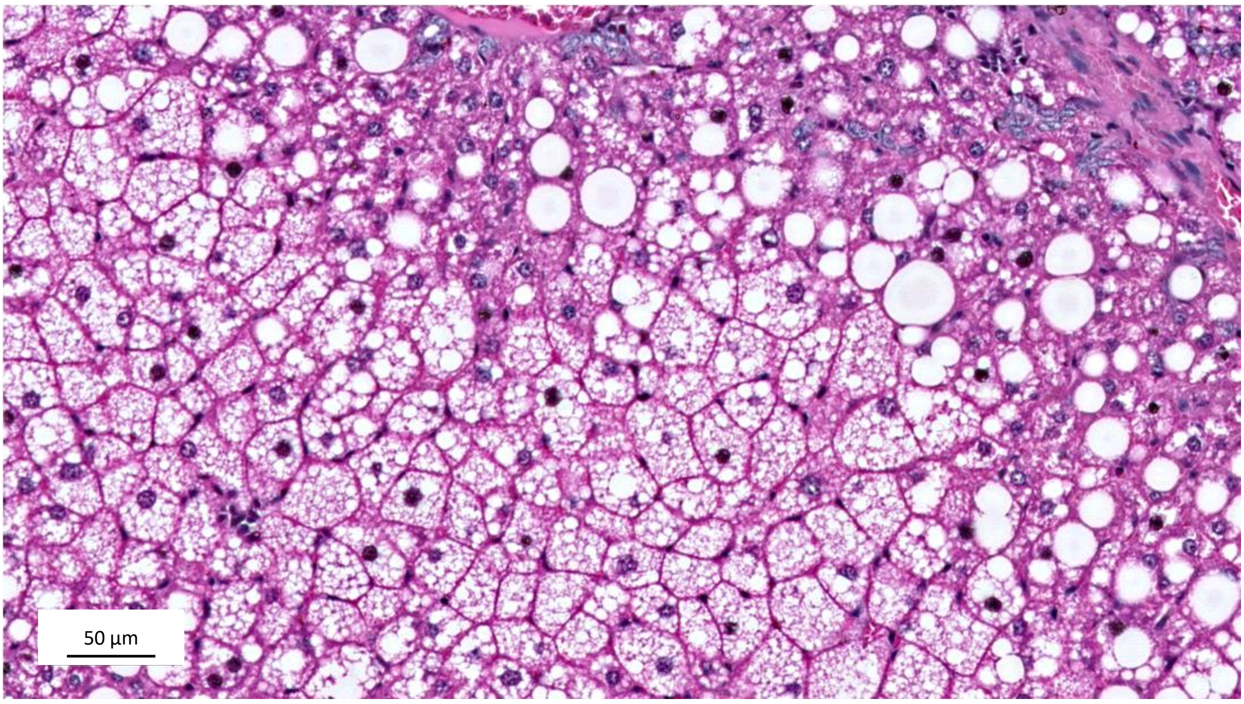

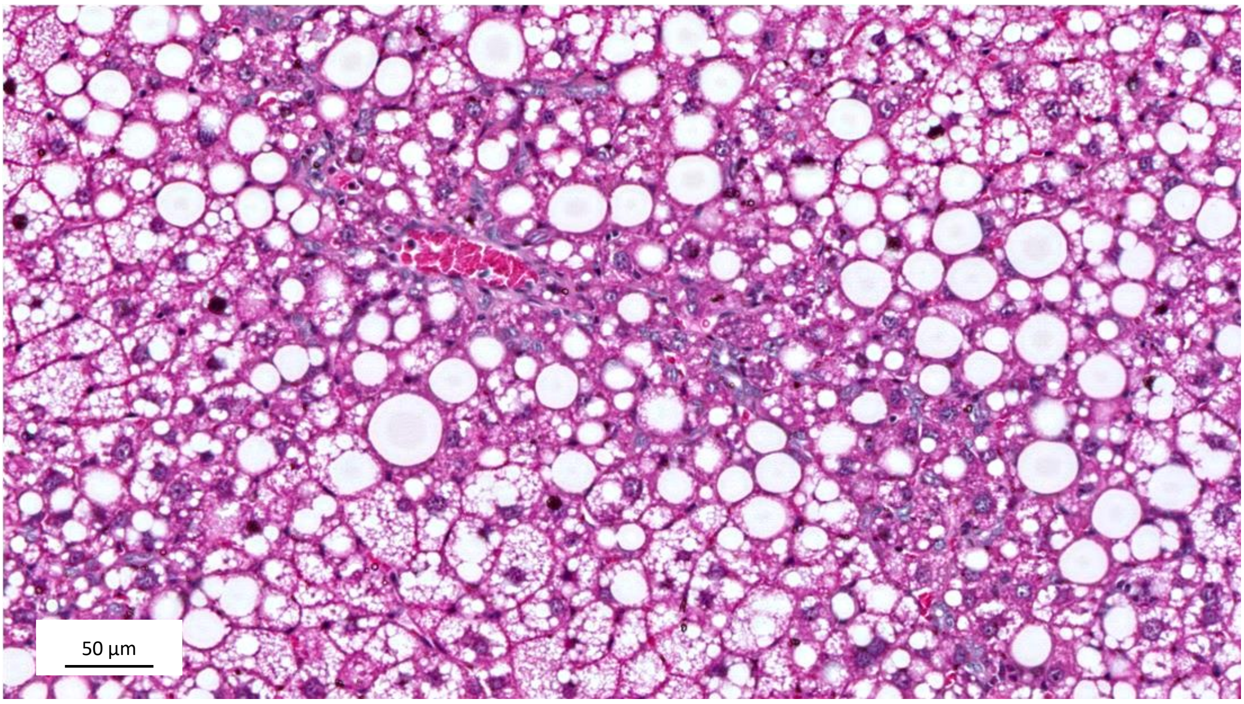

CDHFD-2

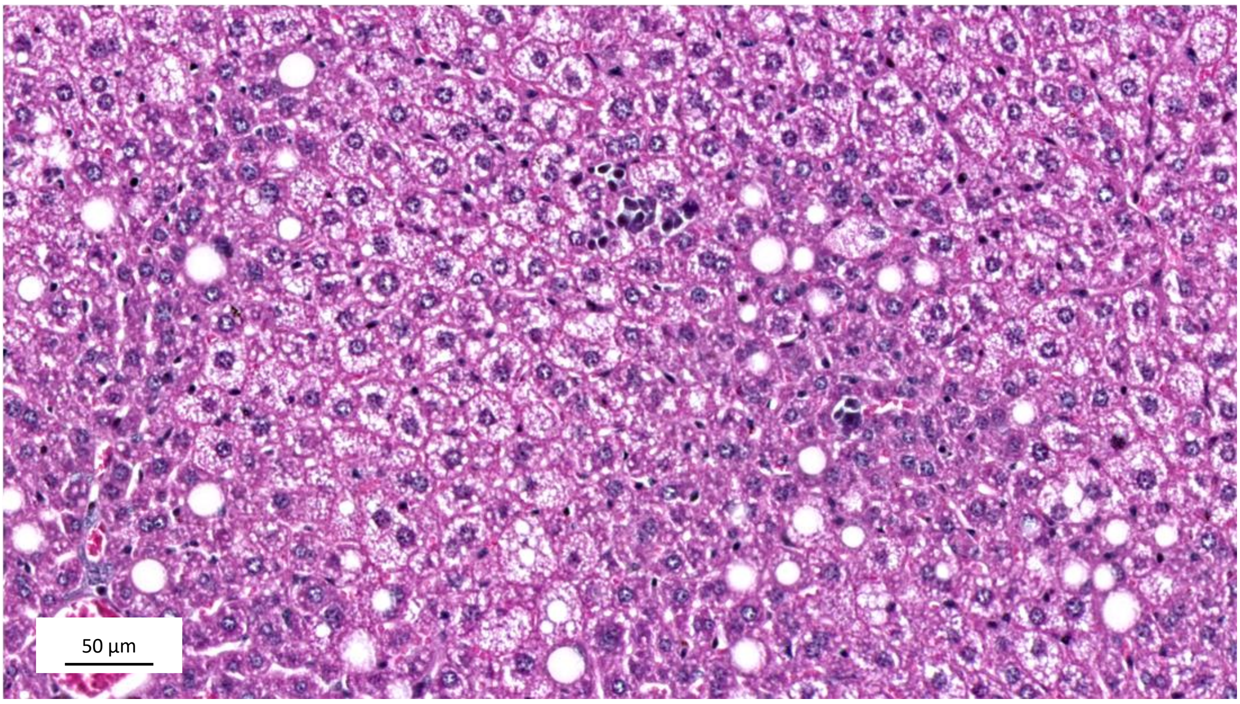

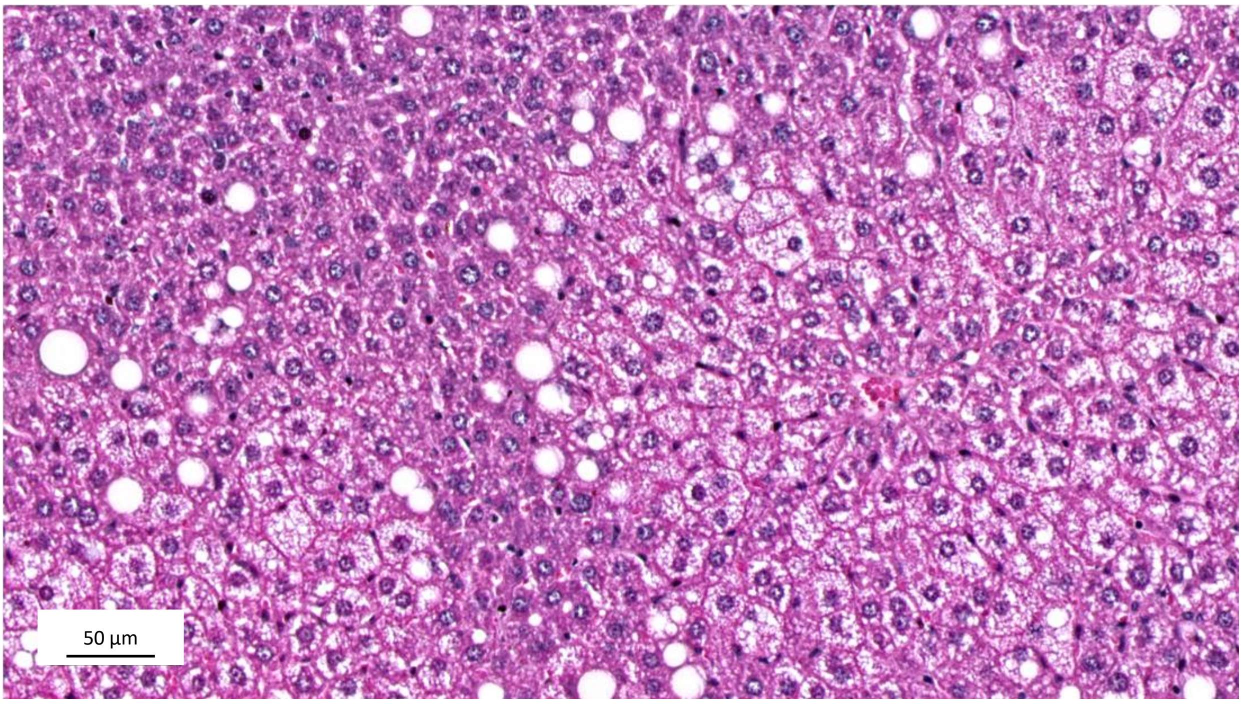

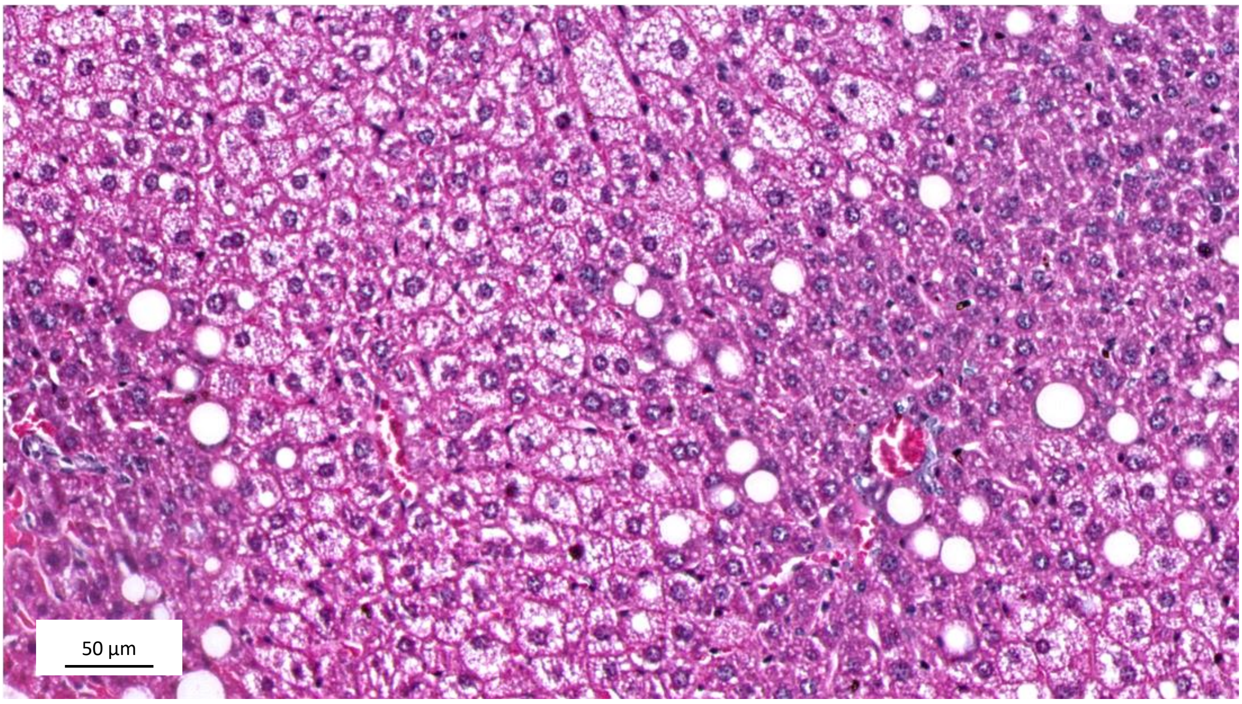

CDHFD-3

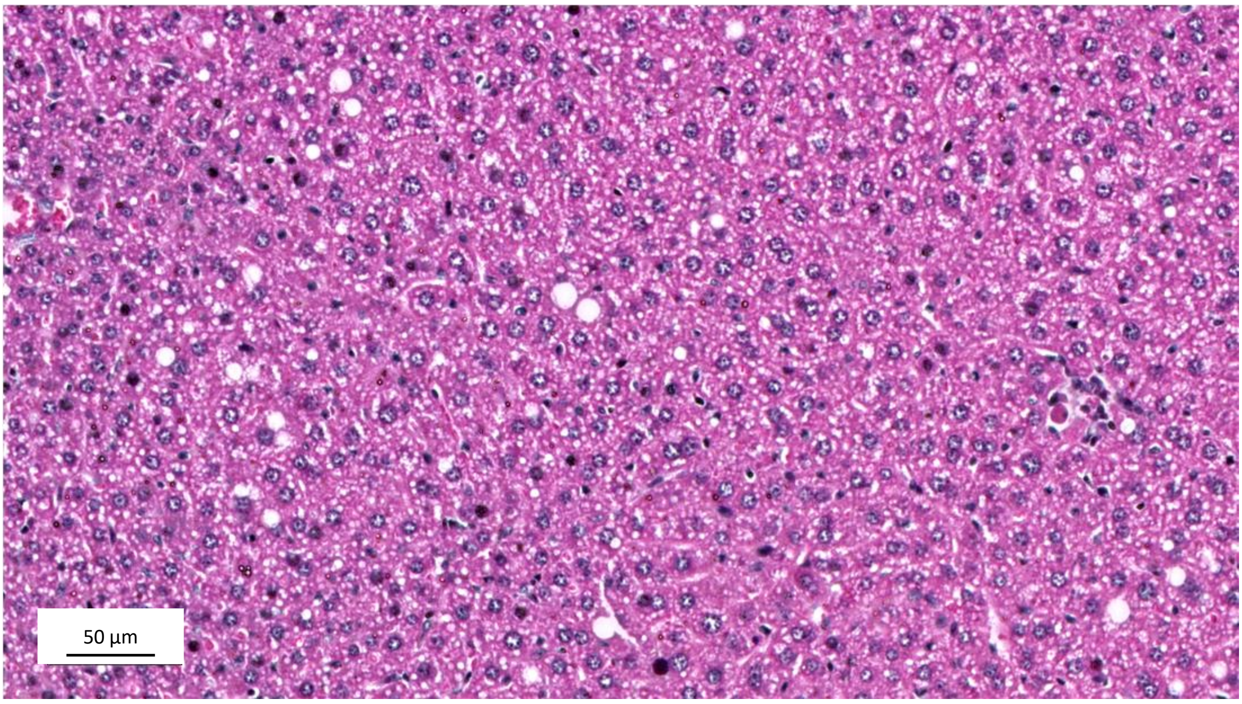

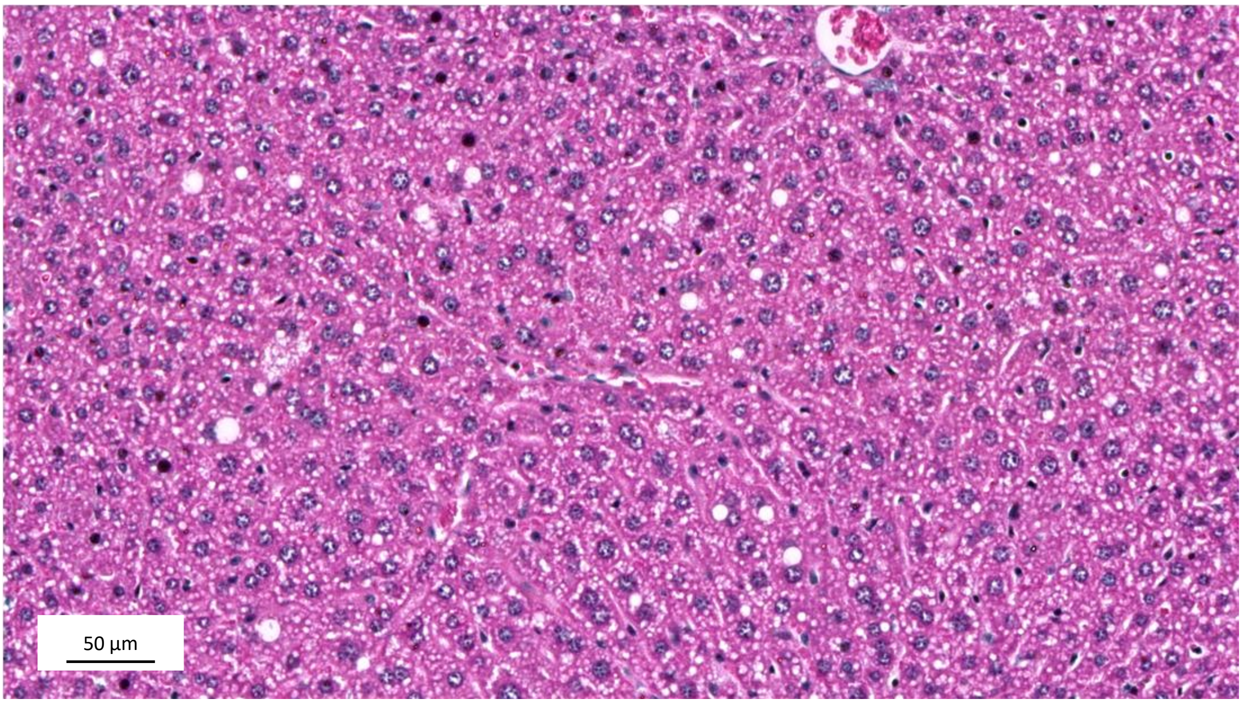

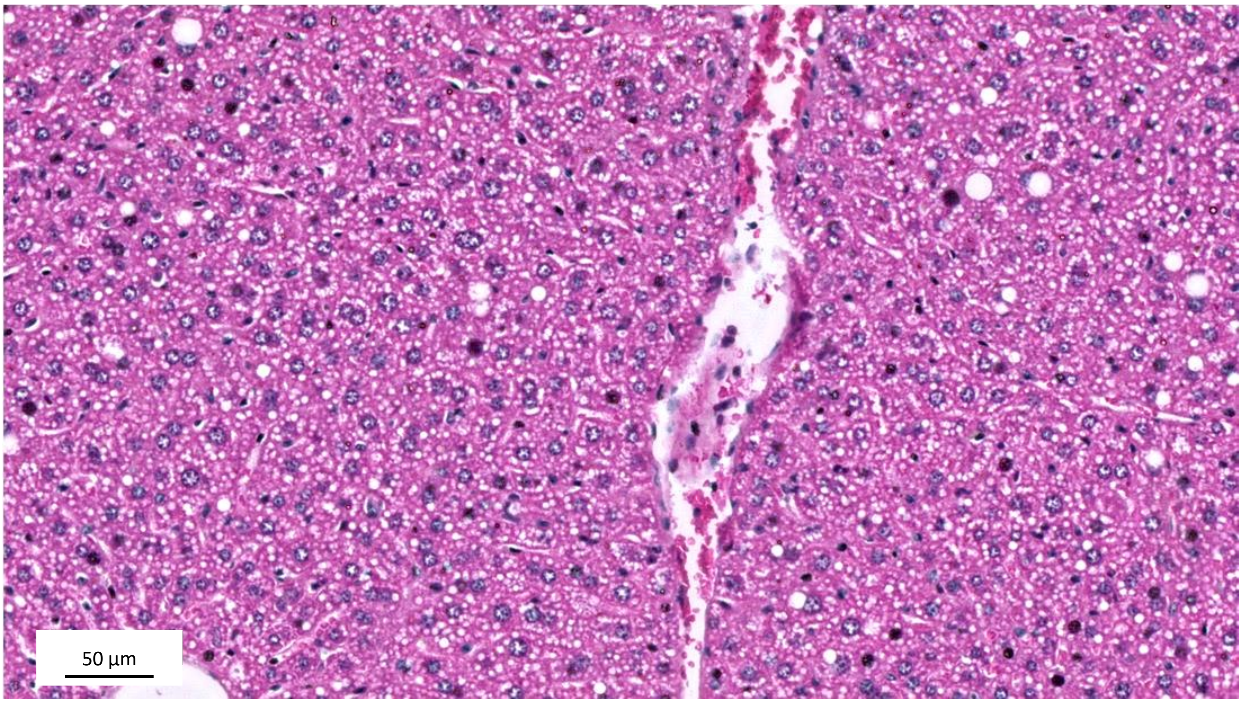

CDHFD-4

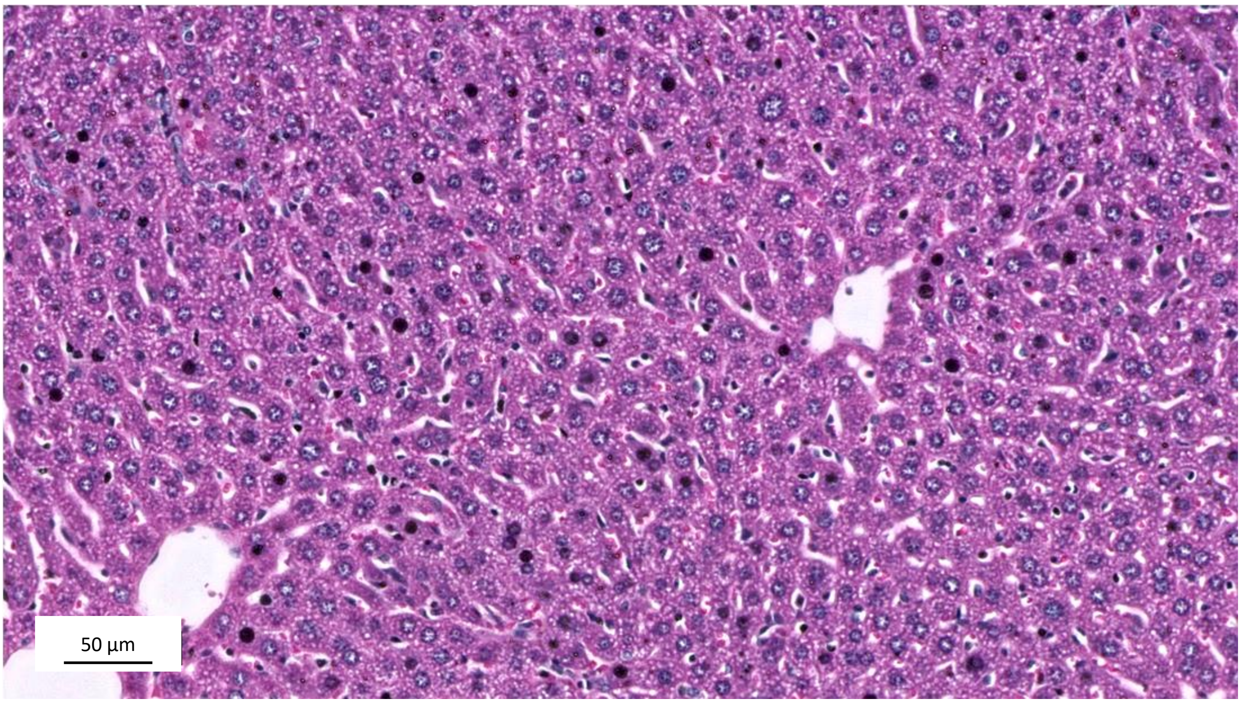

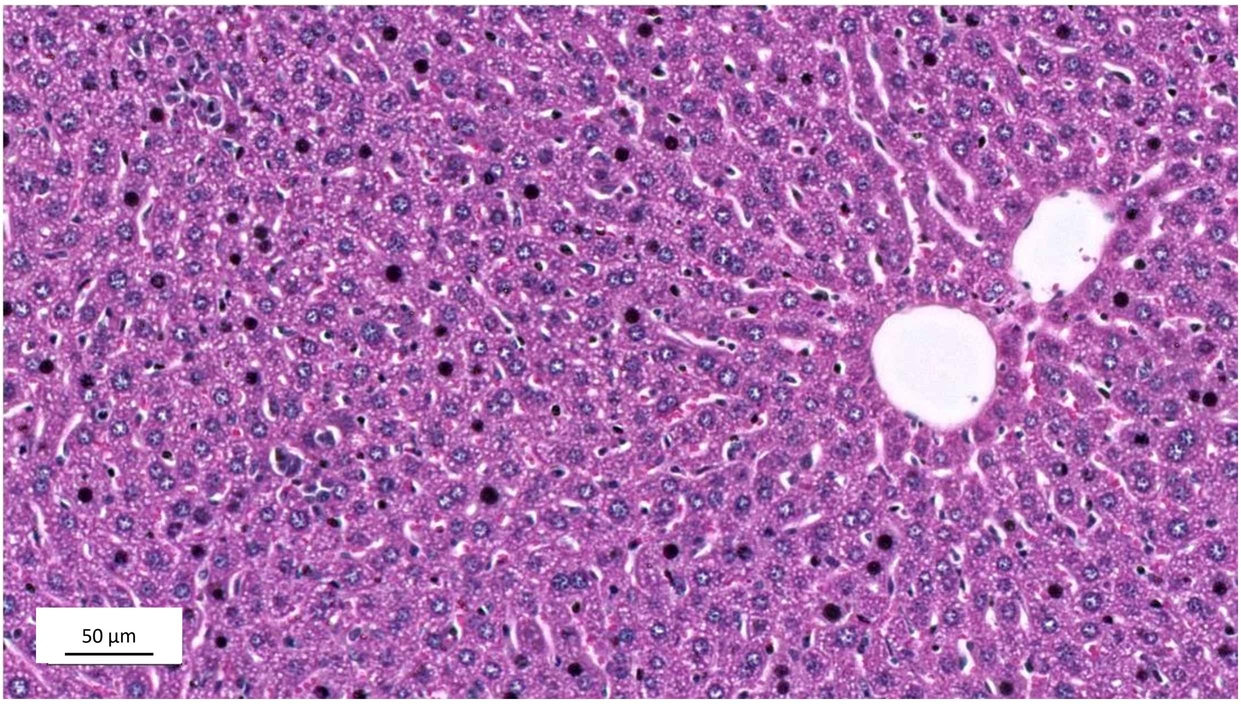

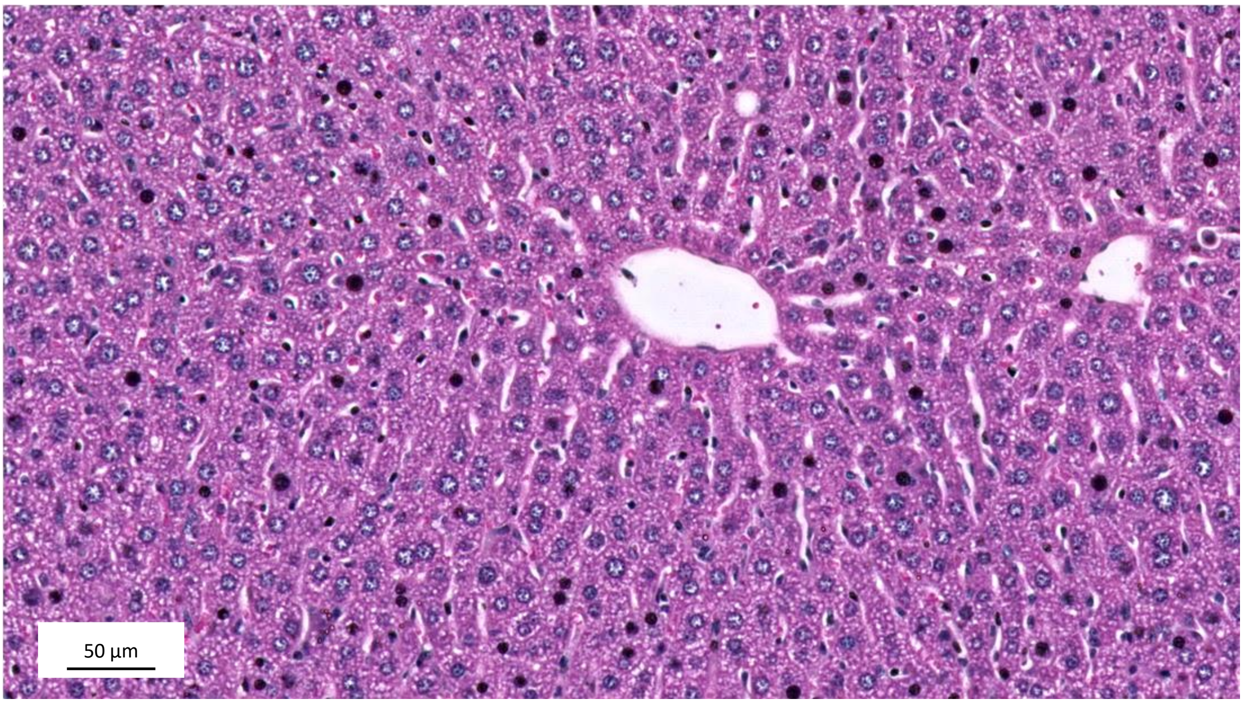

CDHFD-5

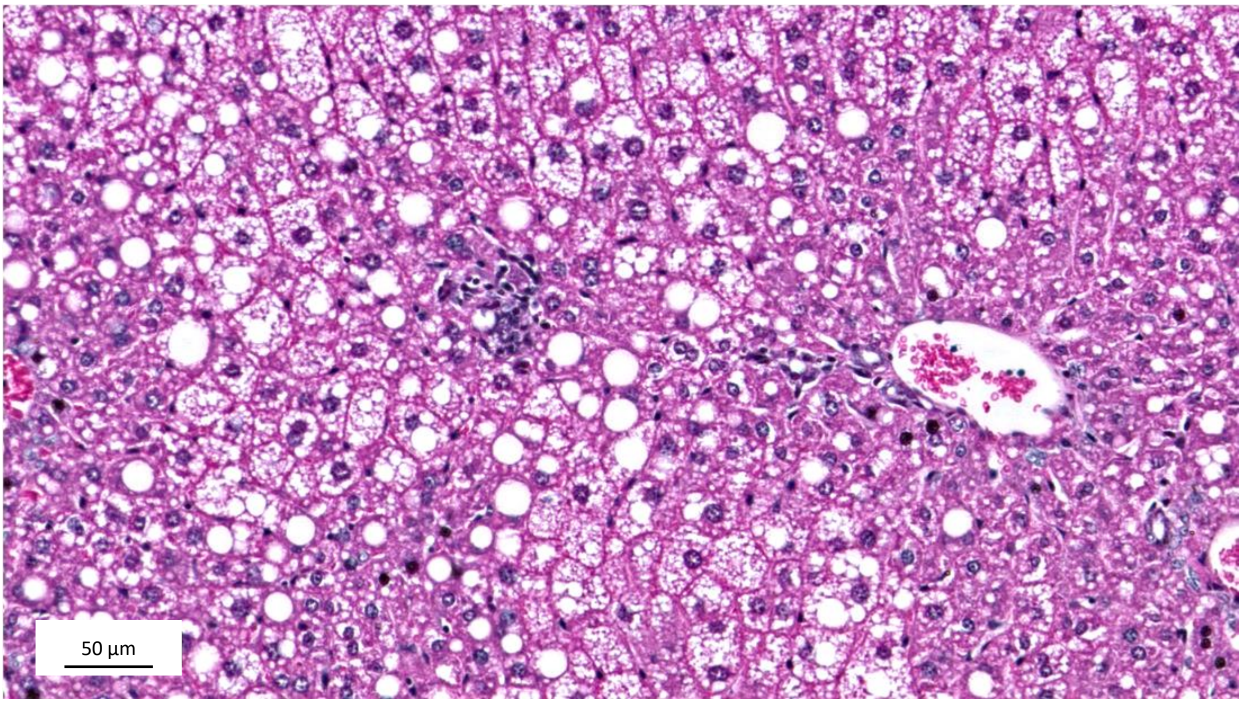

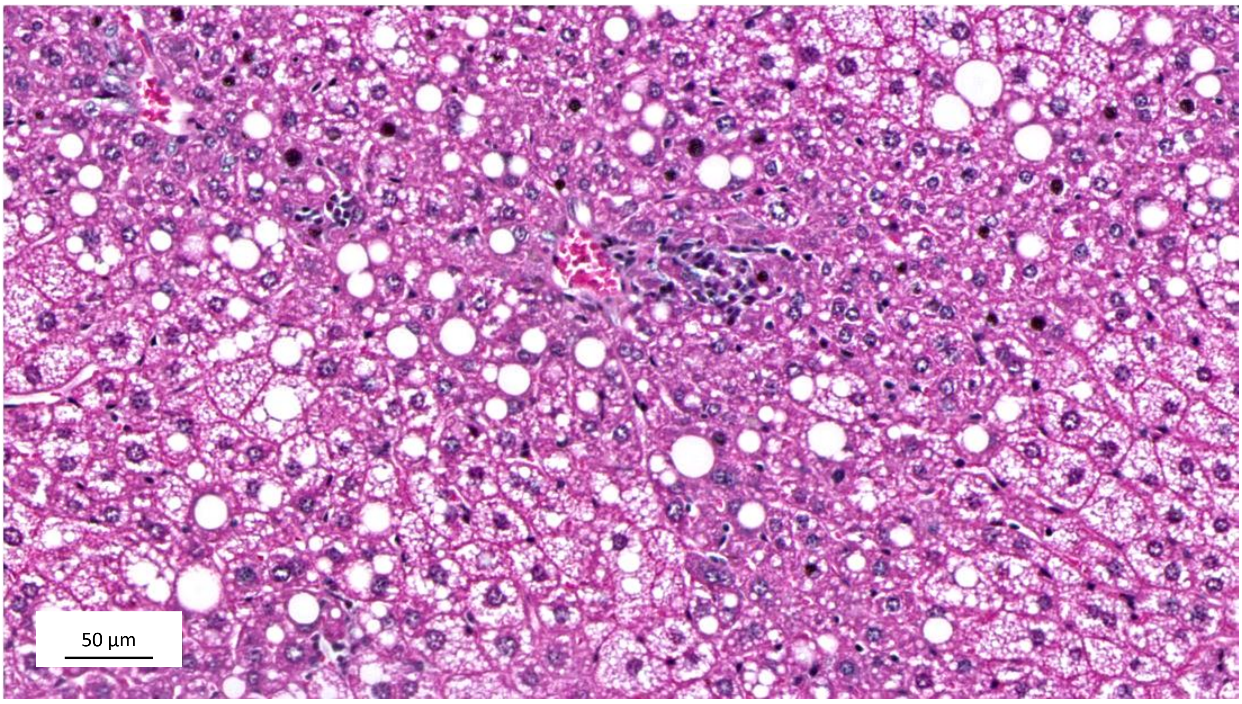

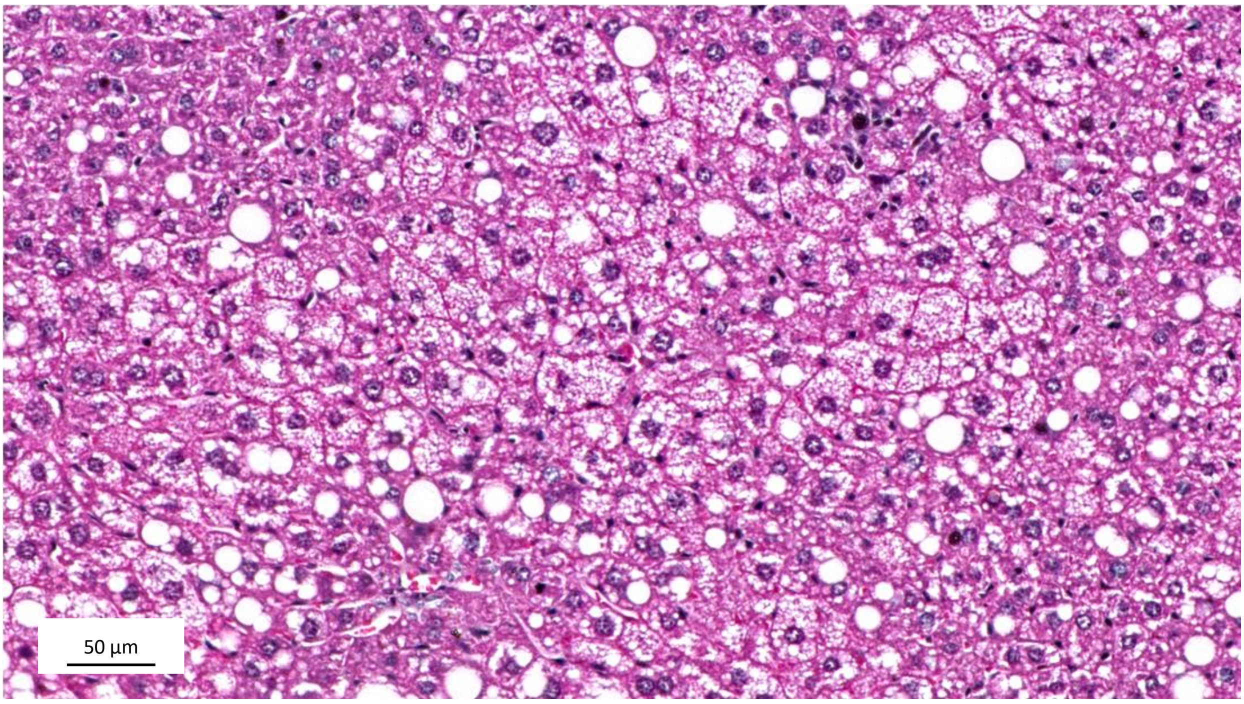

CDHFD-6

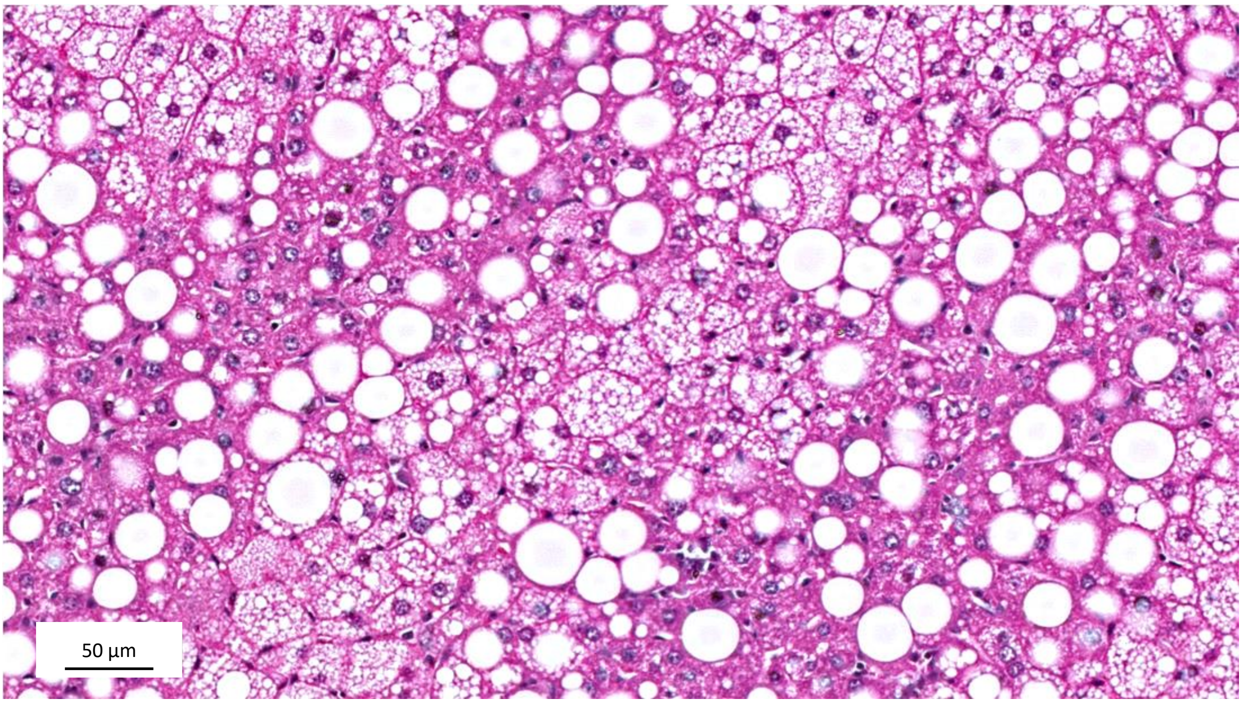

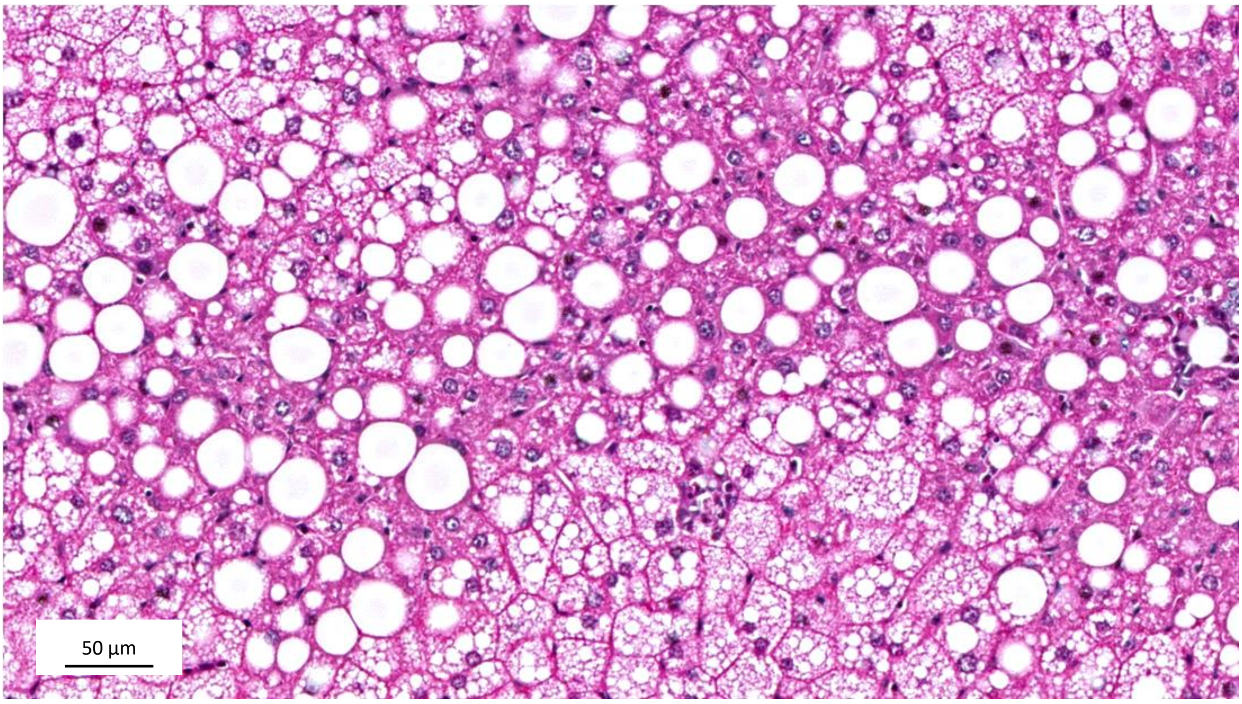

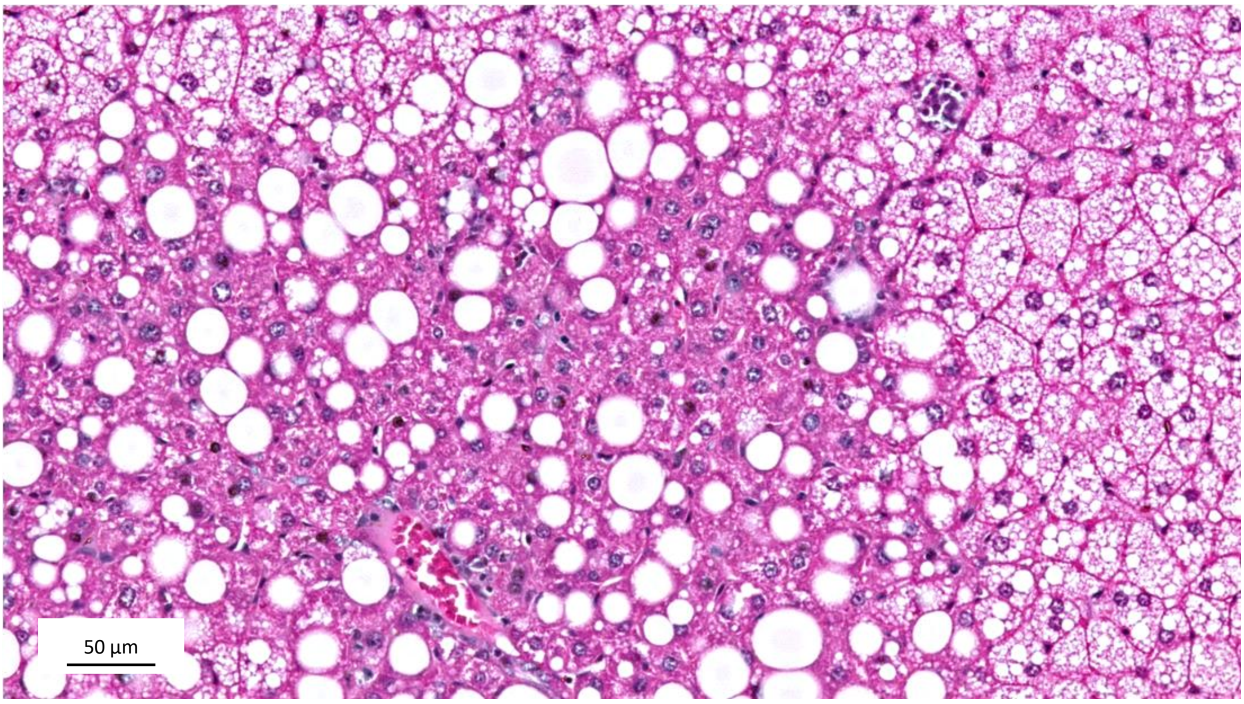

CDHFD-7

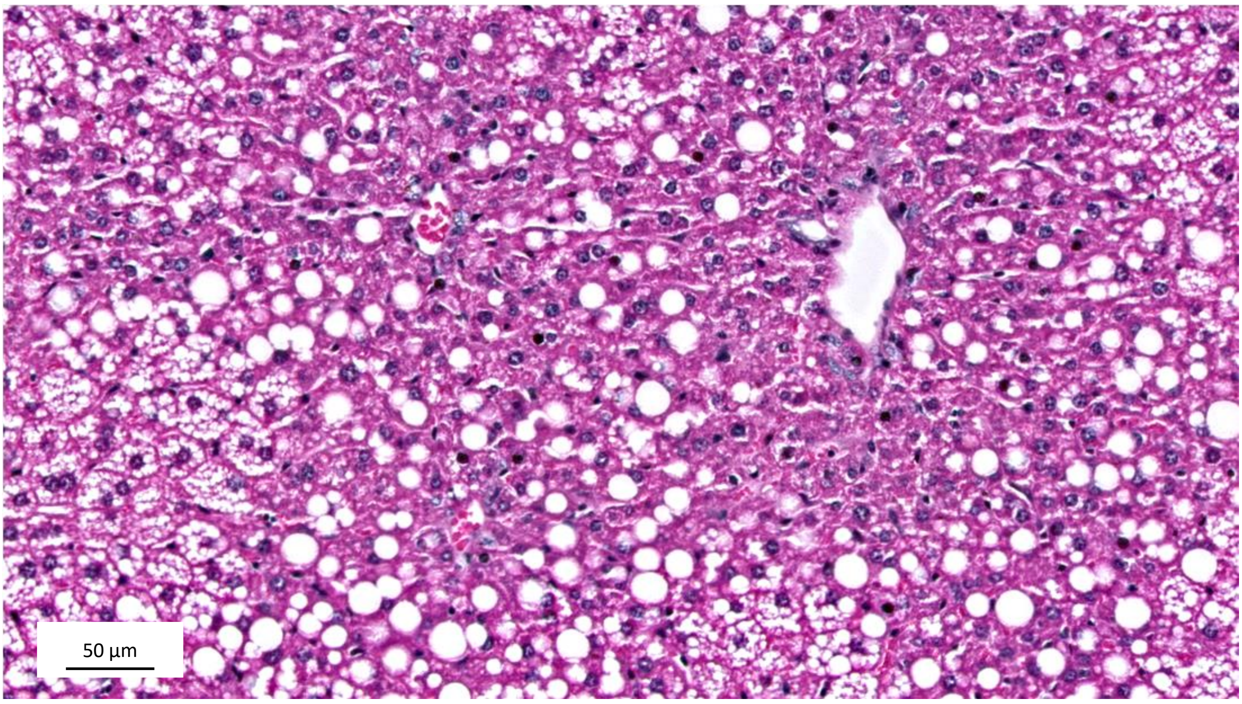

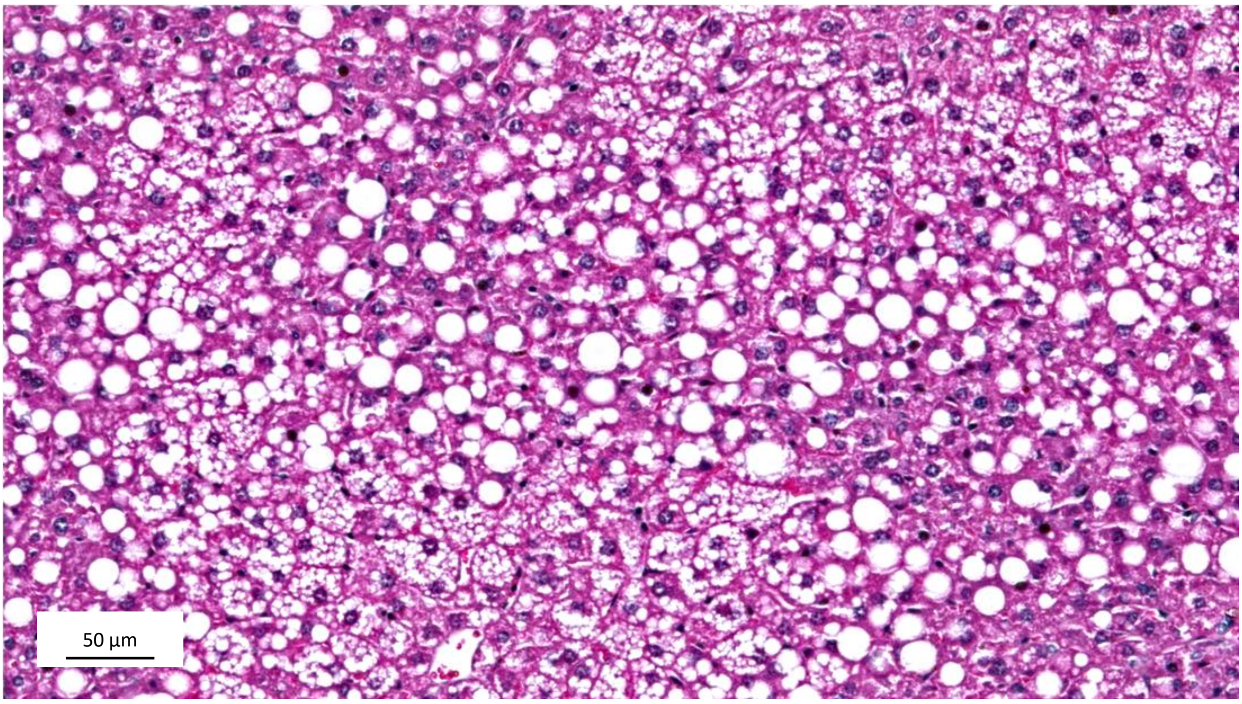

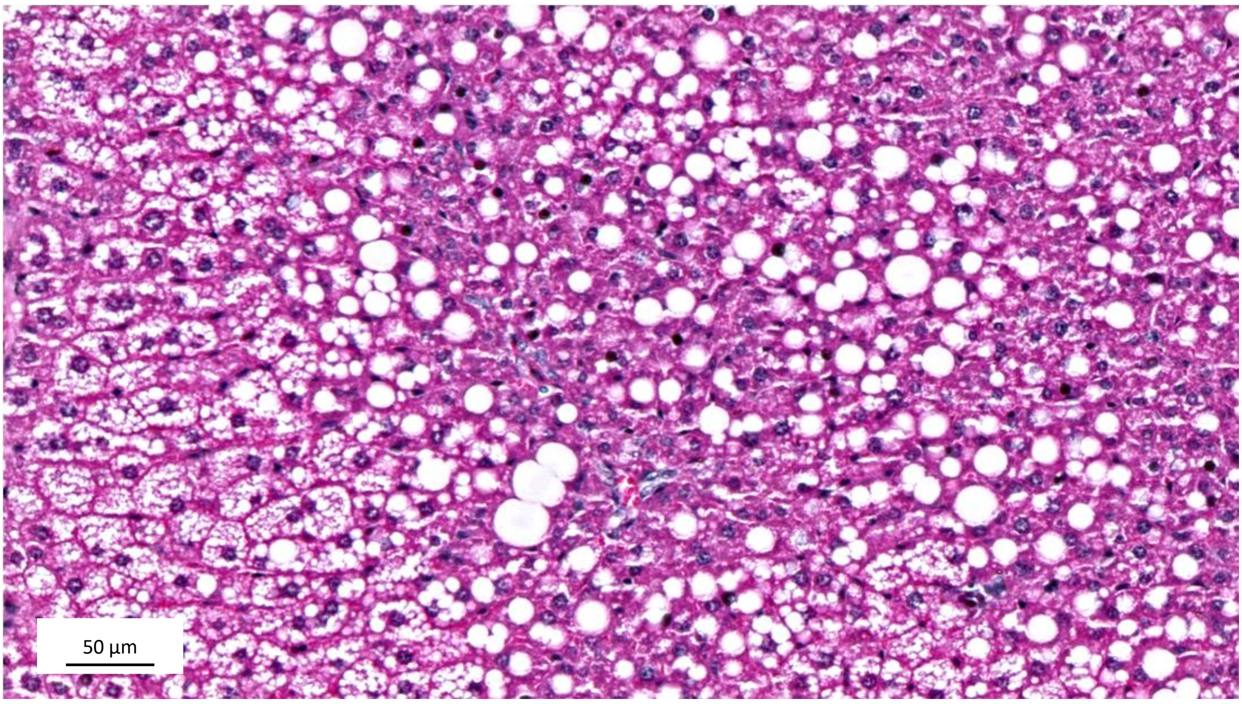

CDHFD-8

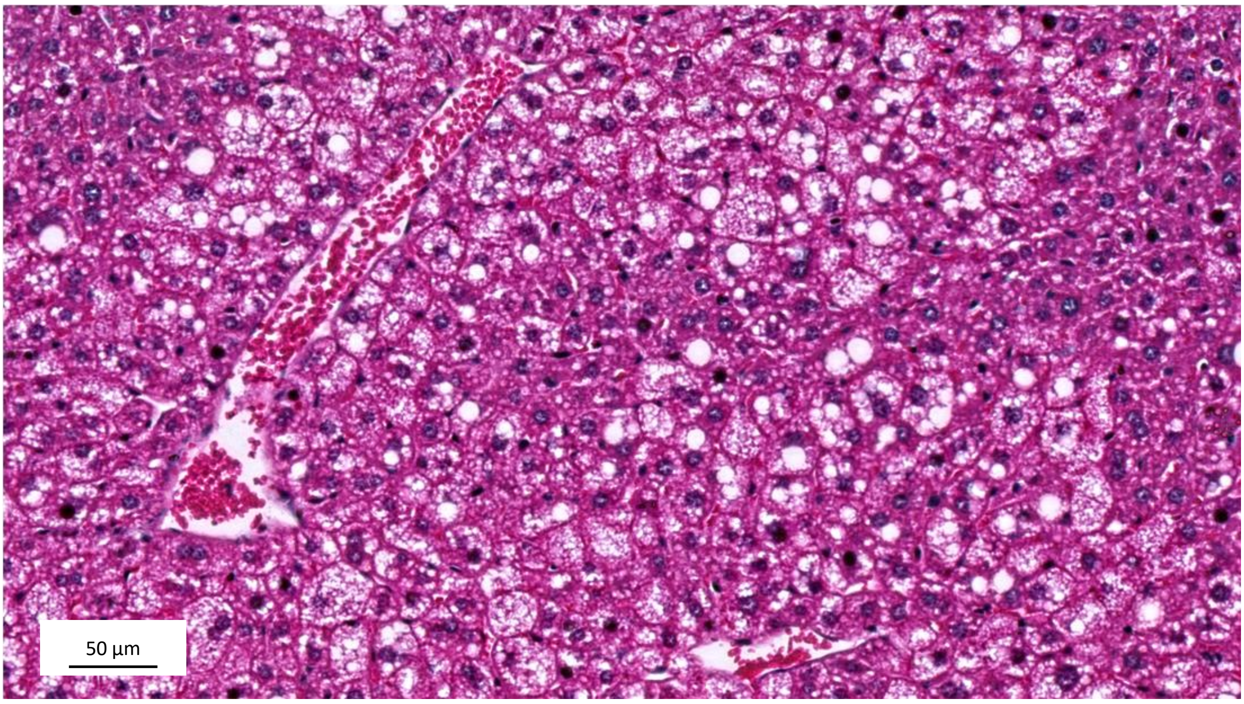

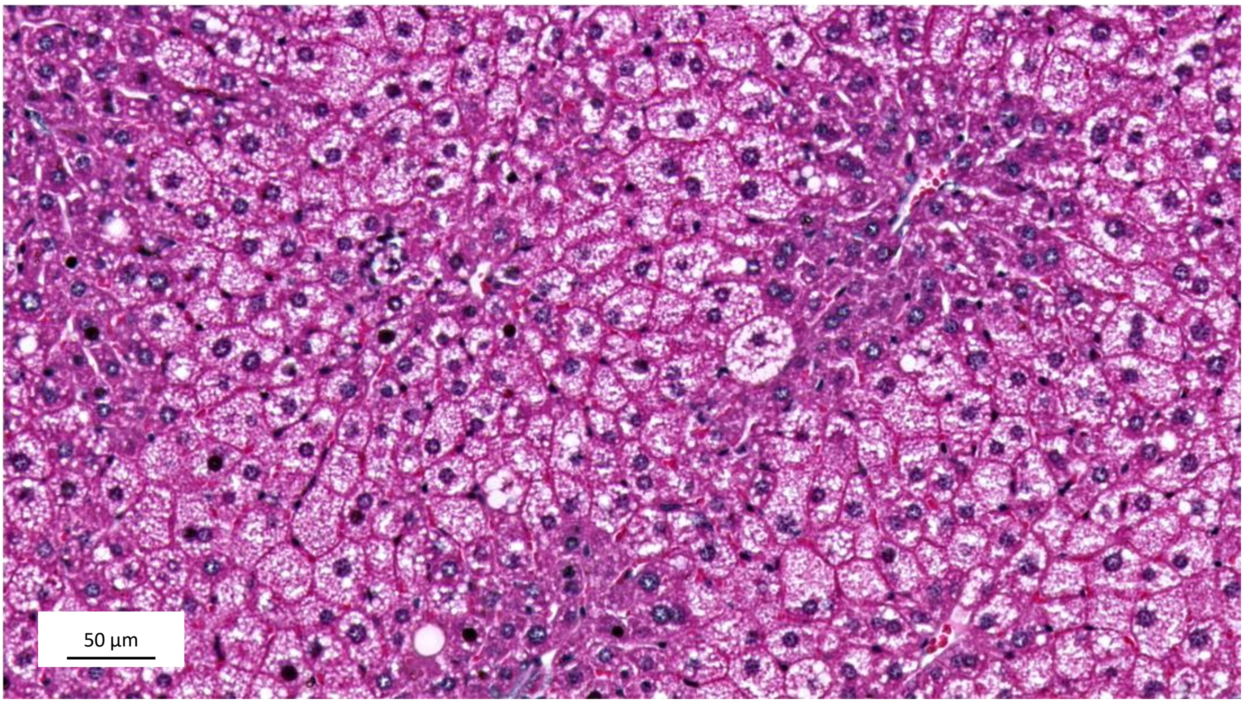

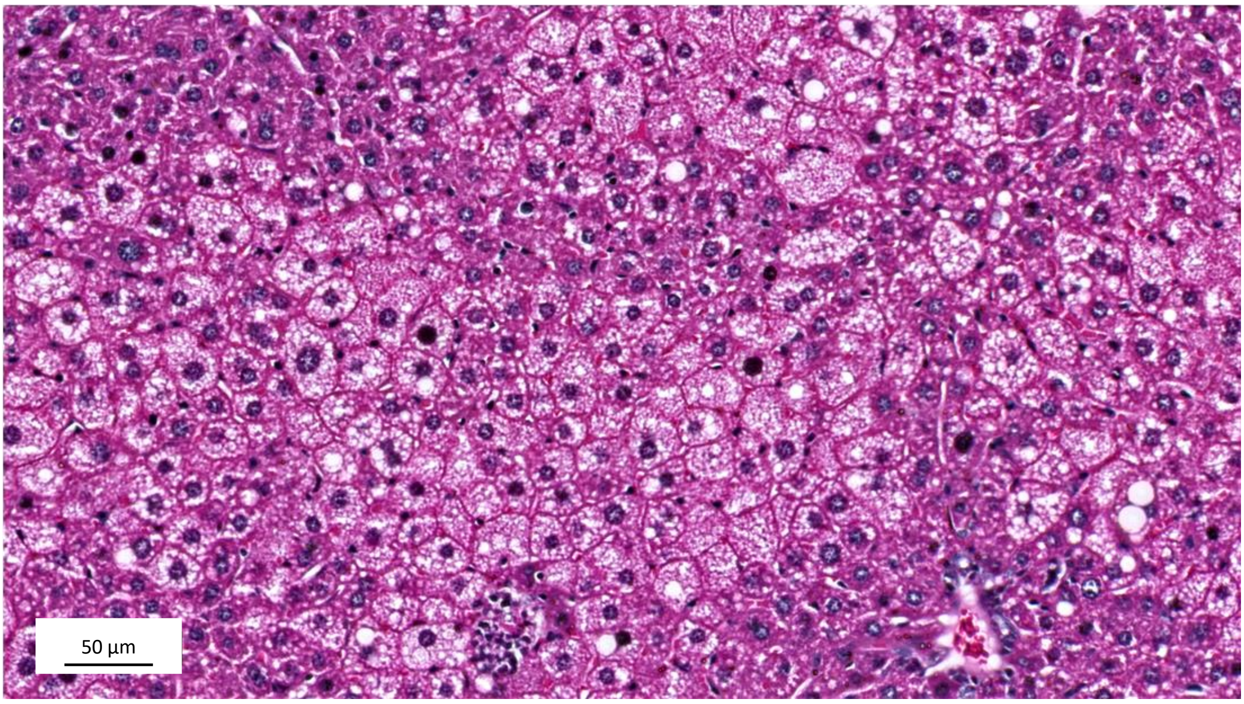

CDHFD-9

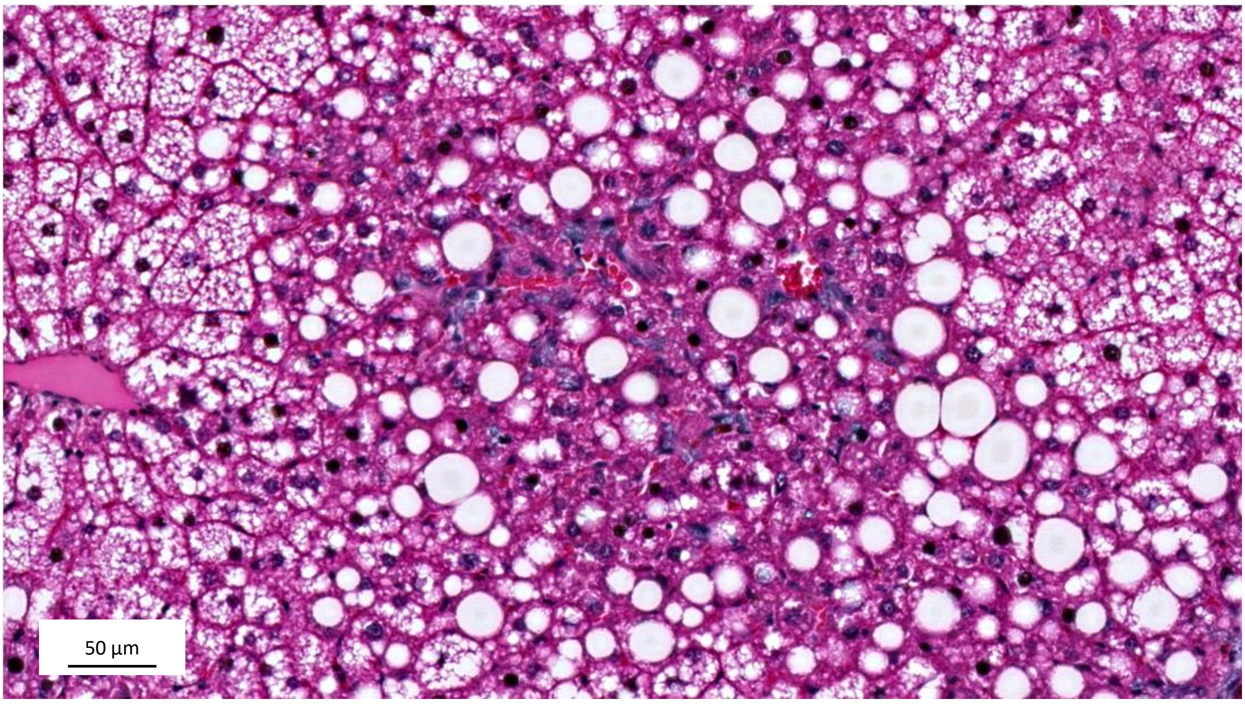

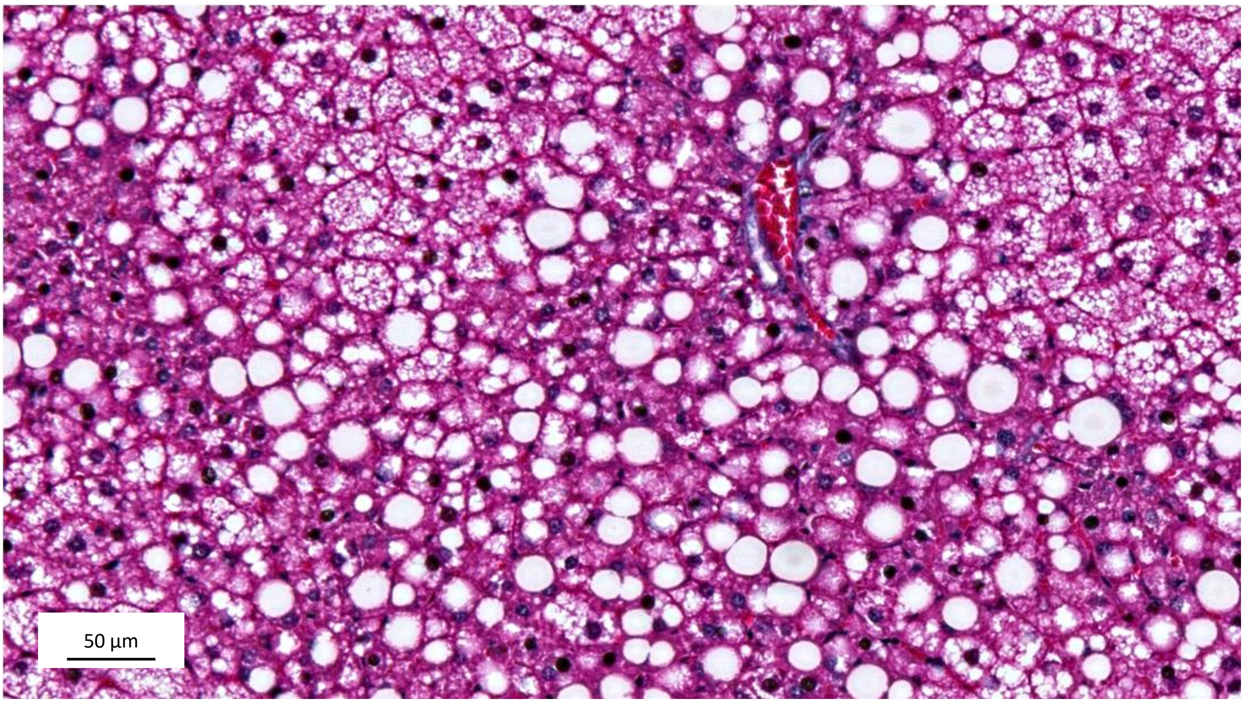

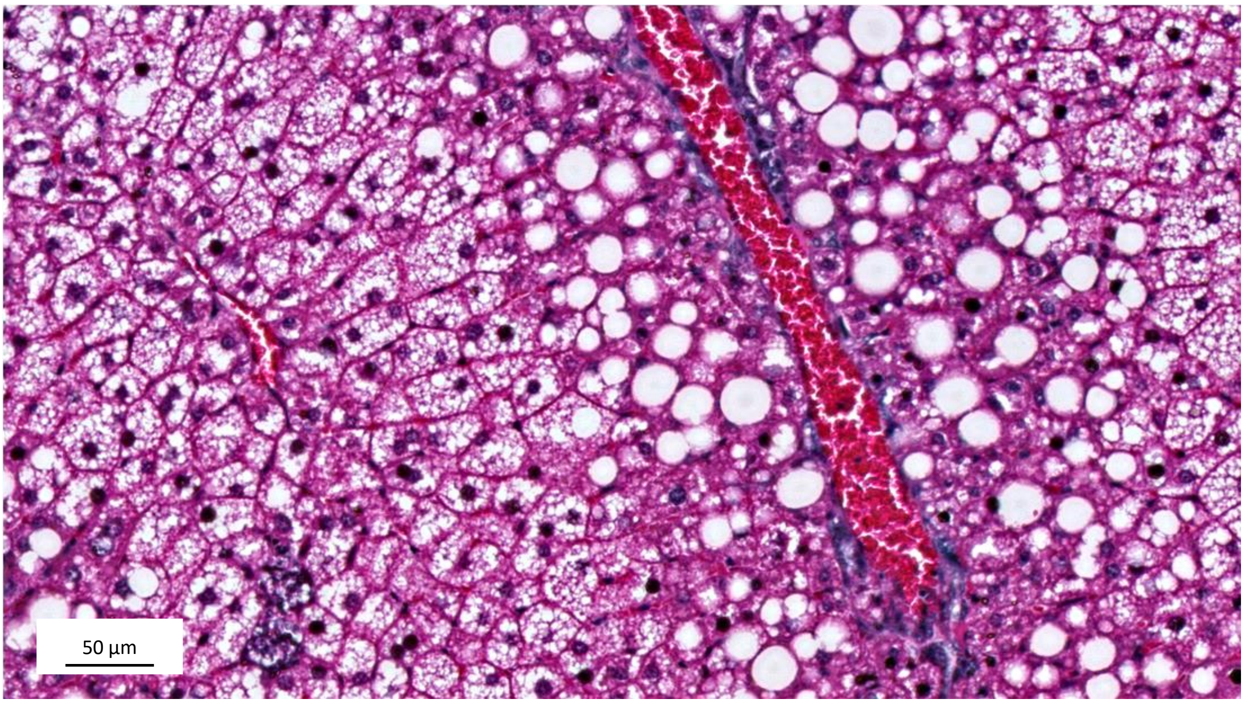

CDHFD-10

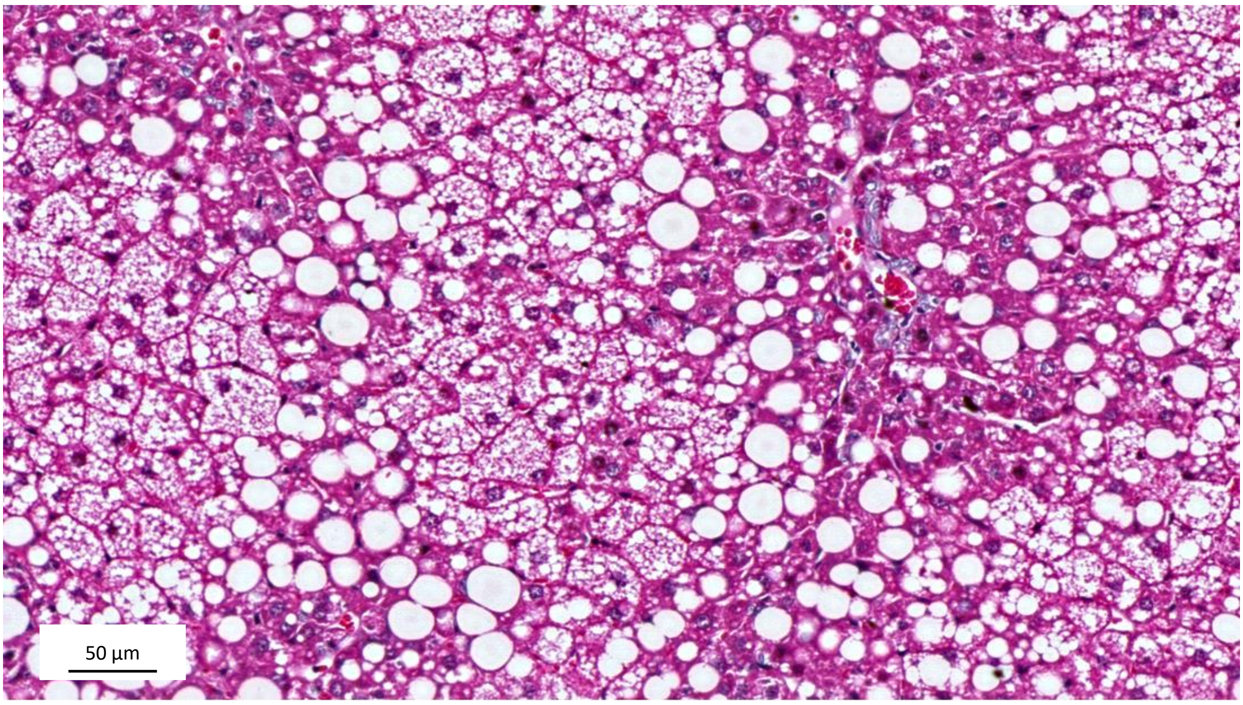

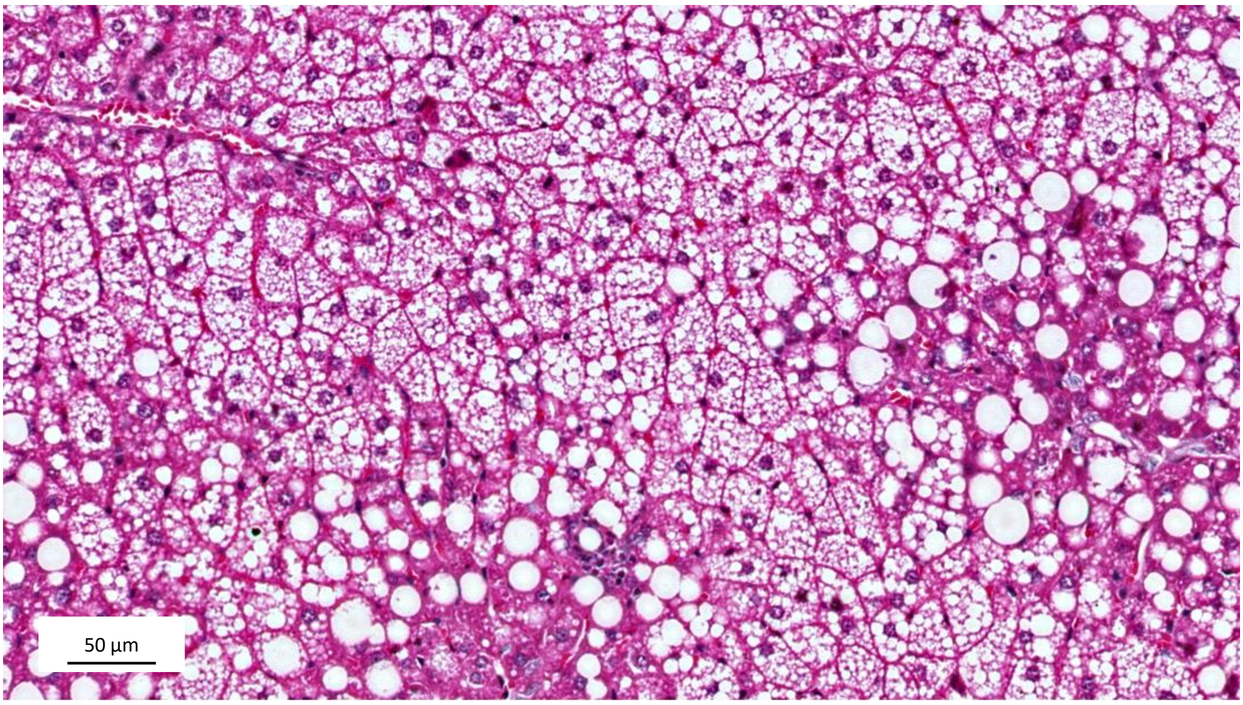

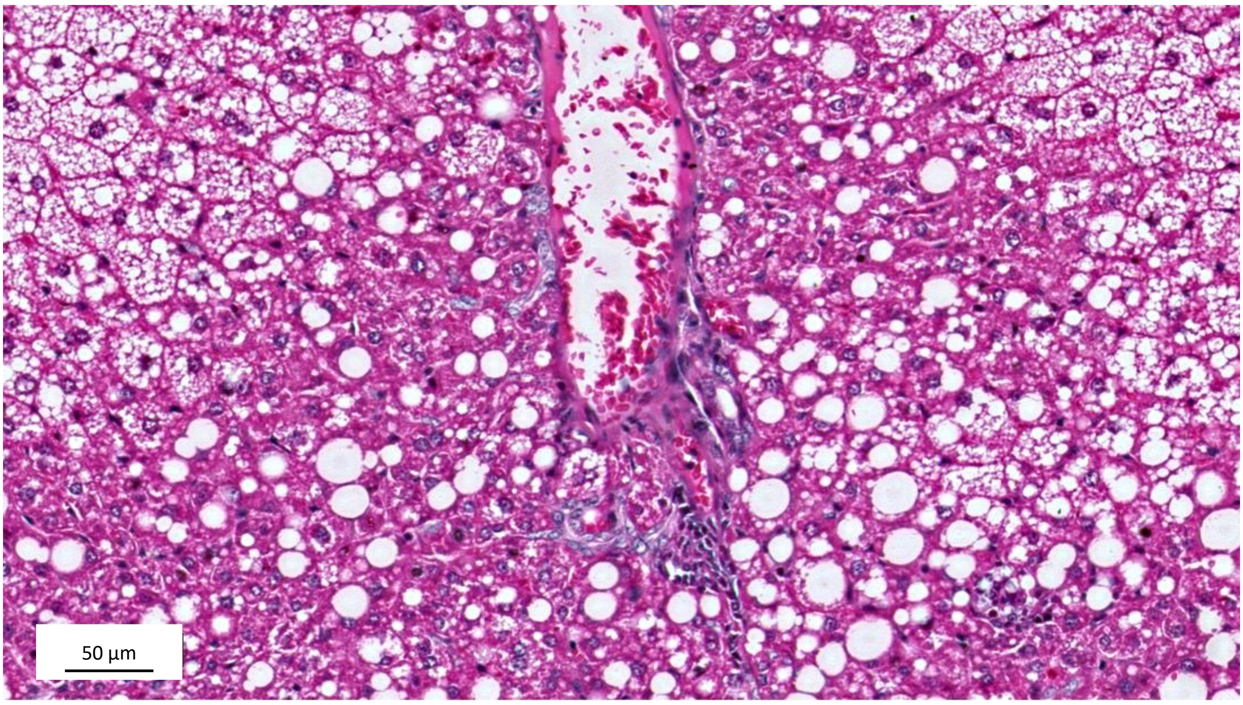

CDHFD-11

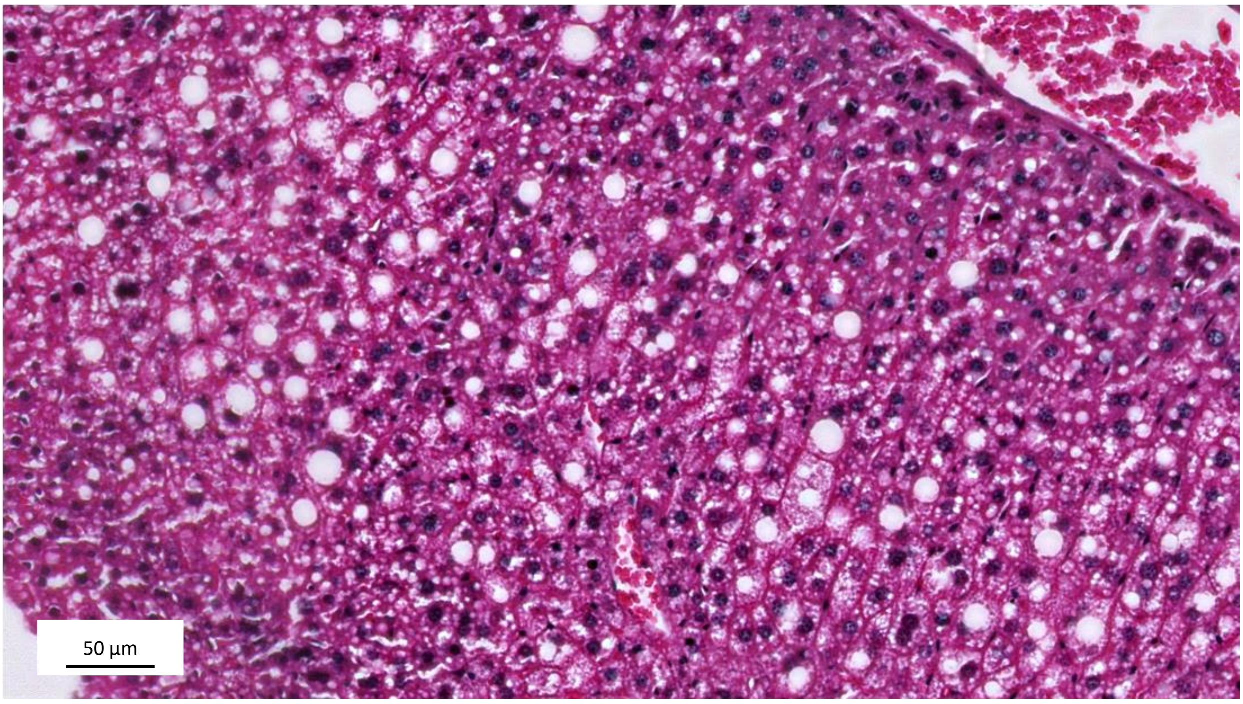

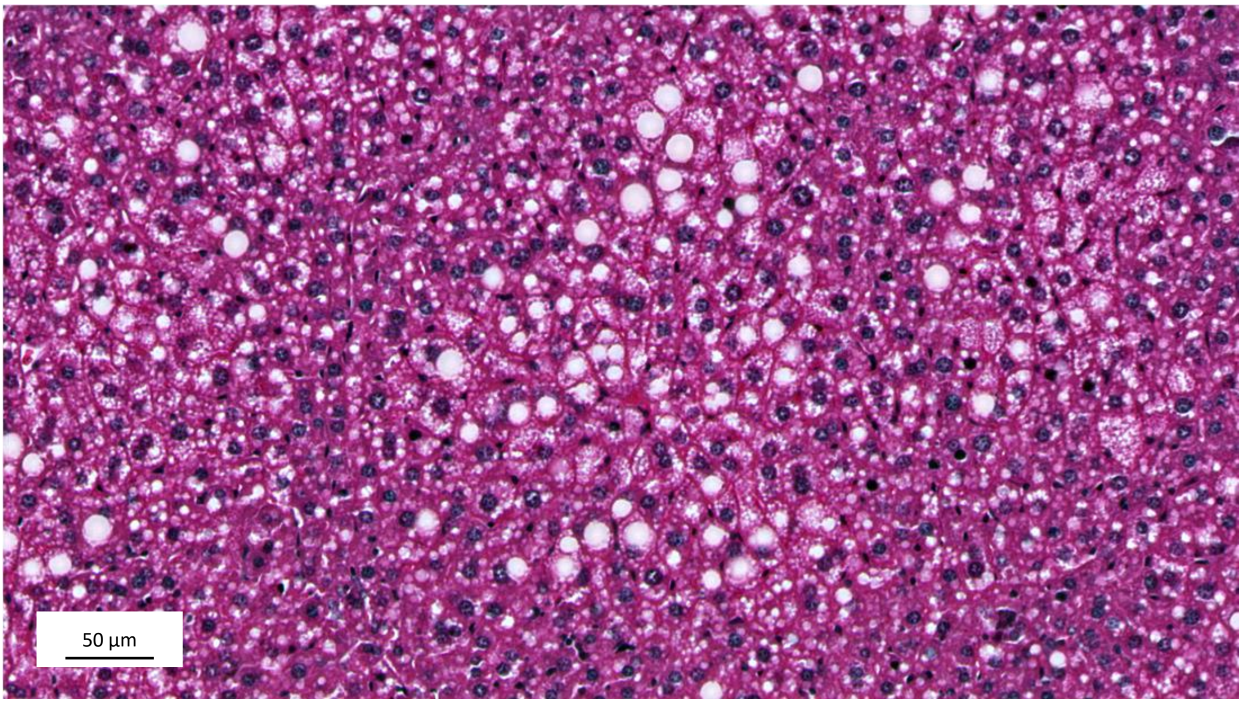

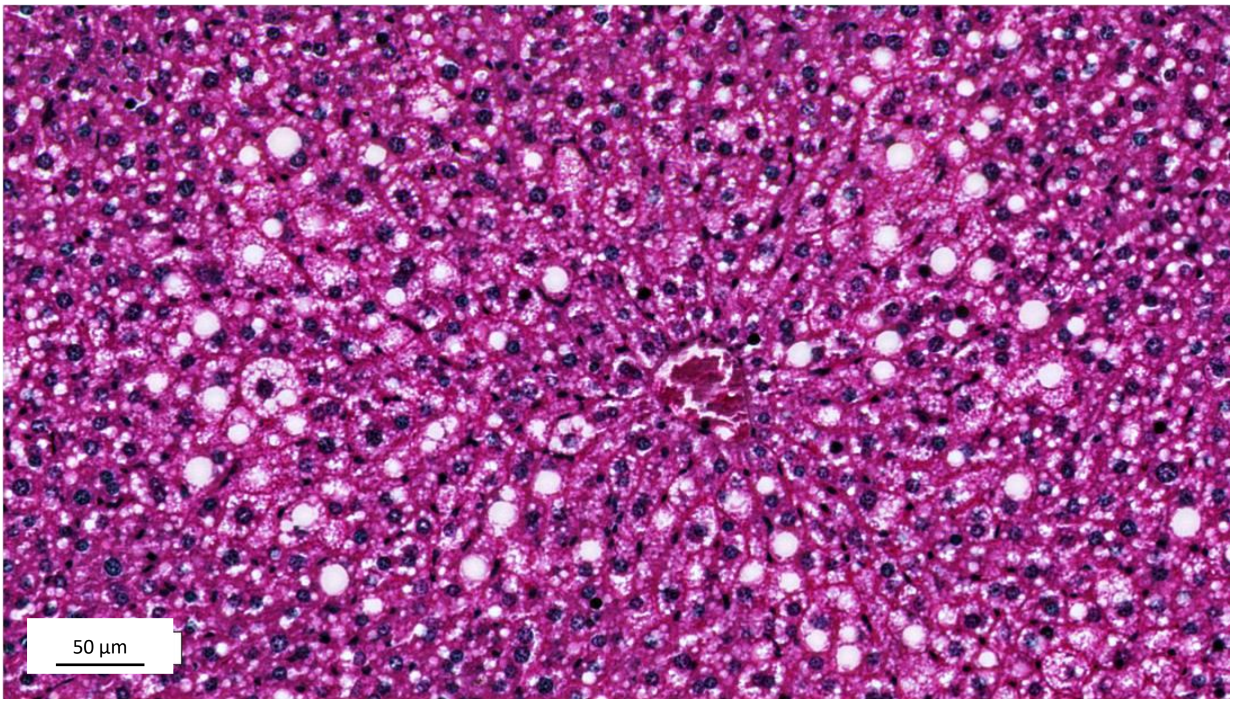

CDHFD-12

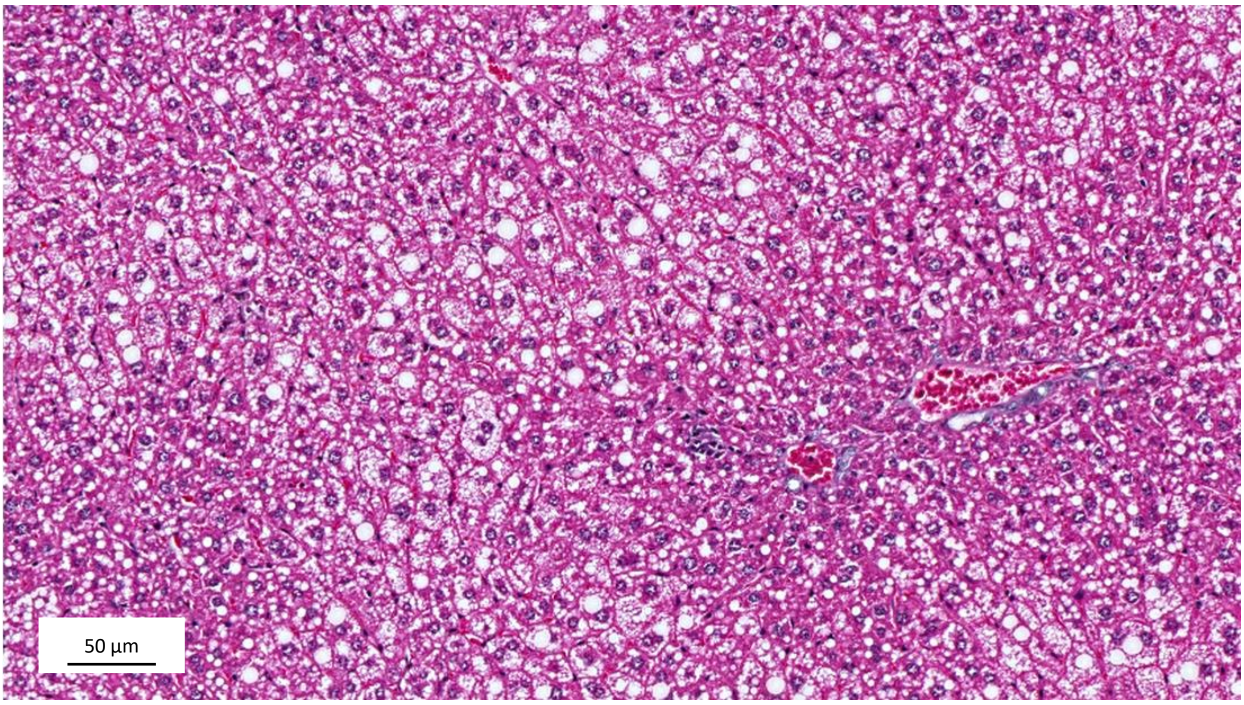

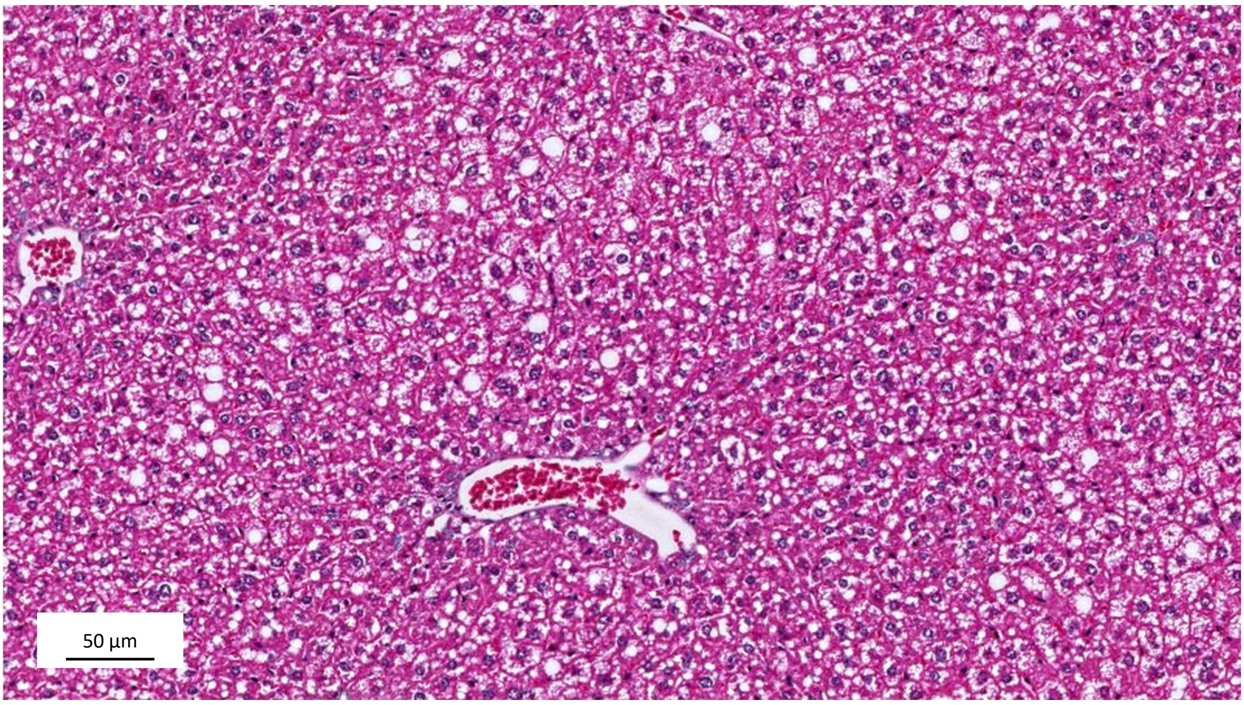

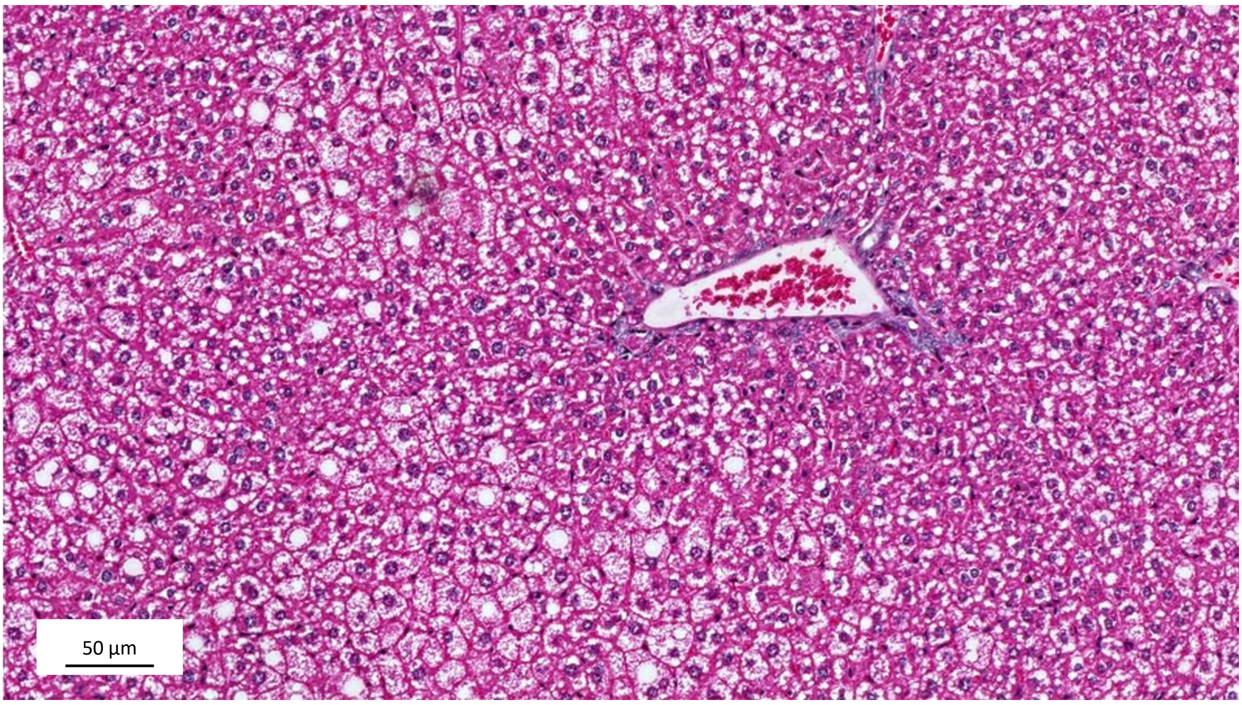

CDHFD-13

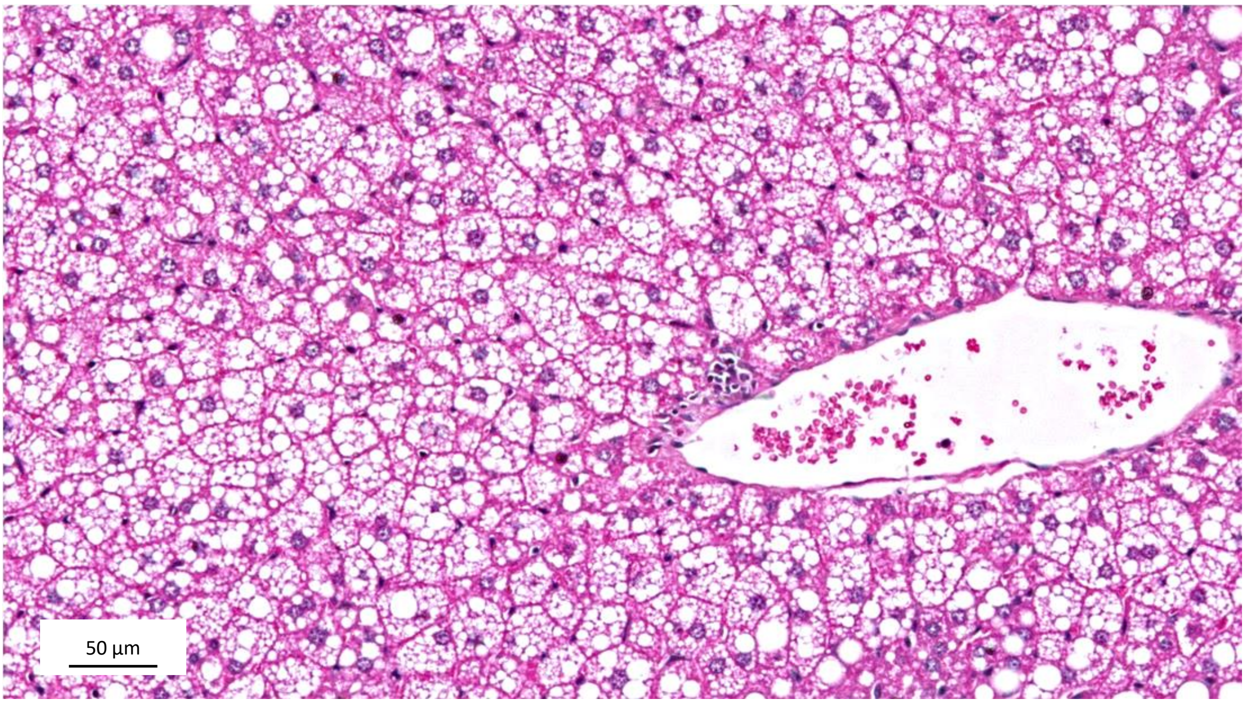

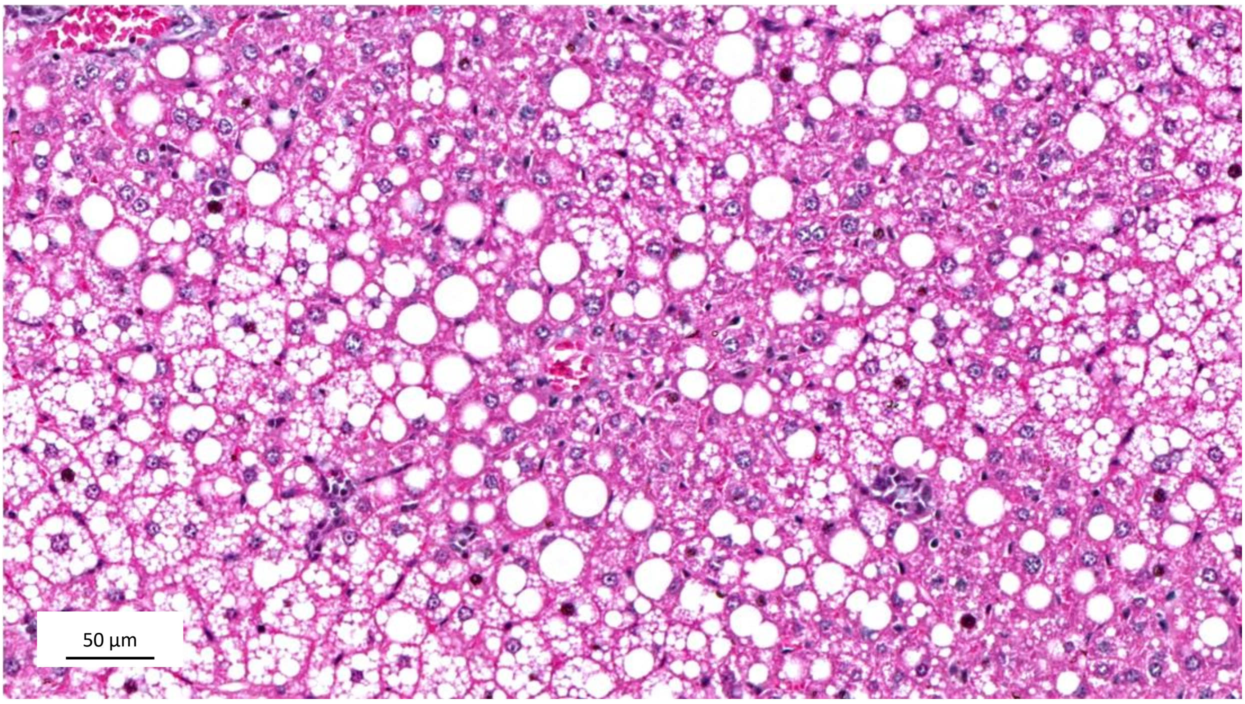

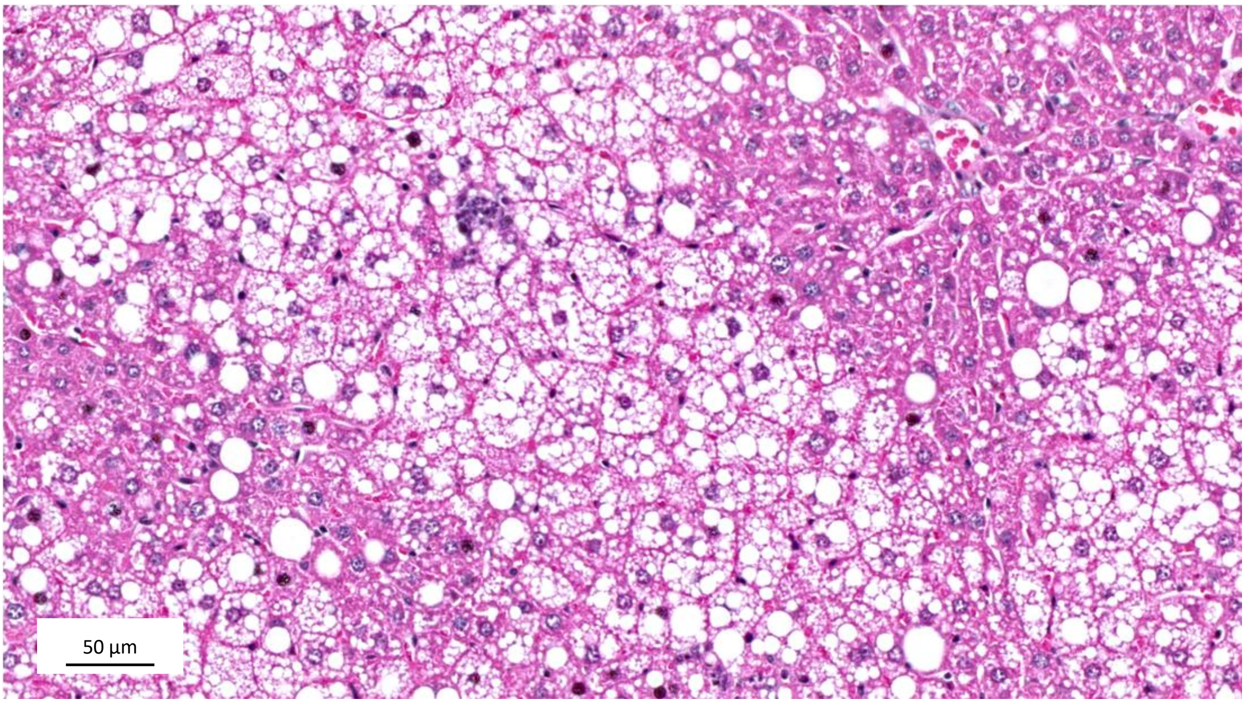

H&E Staining

CDHFD-I group

(15 mice were included)

CDHFD-I-1

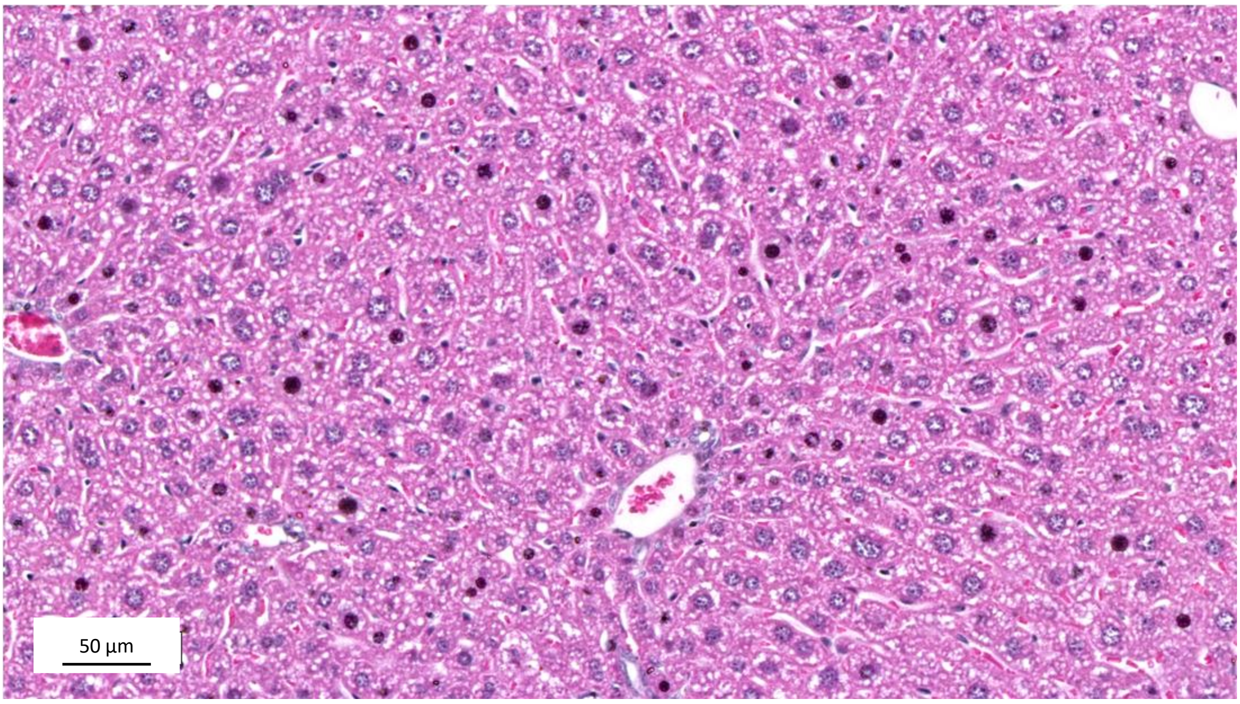

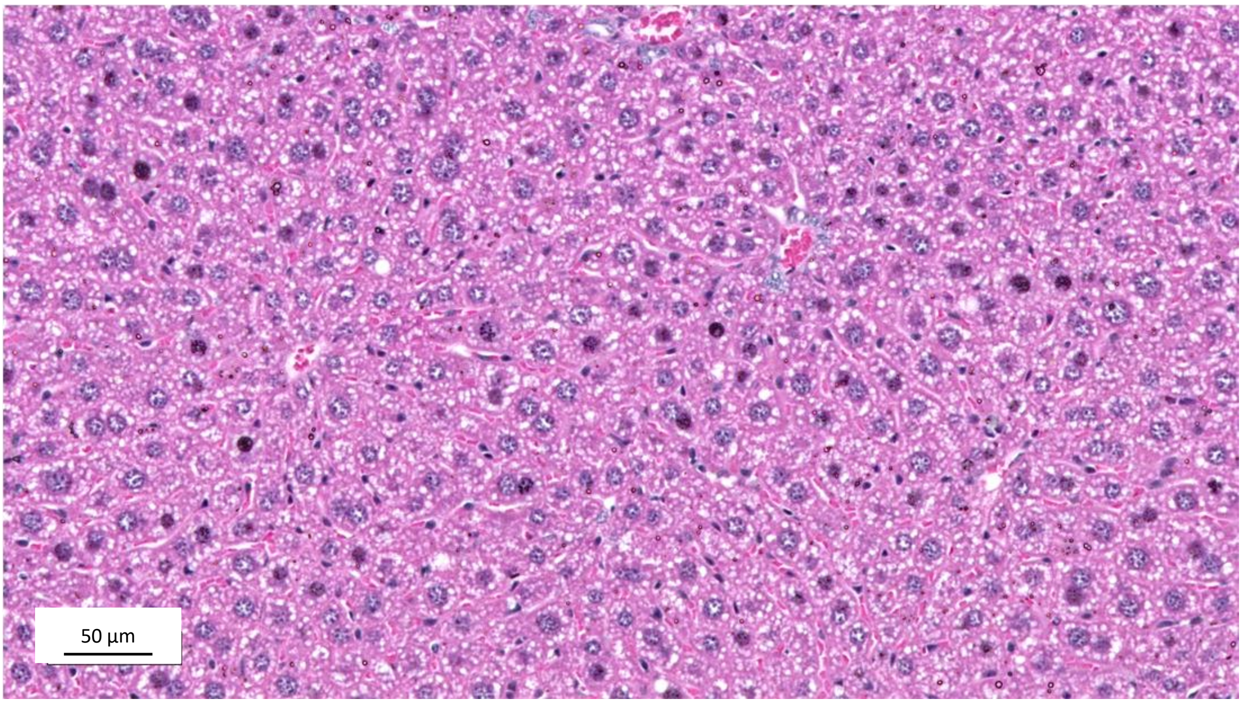

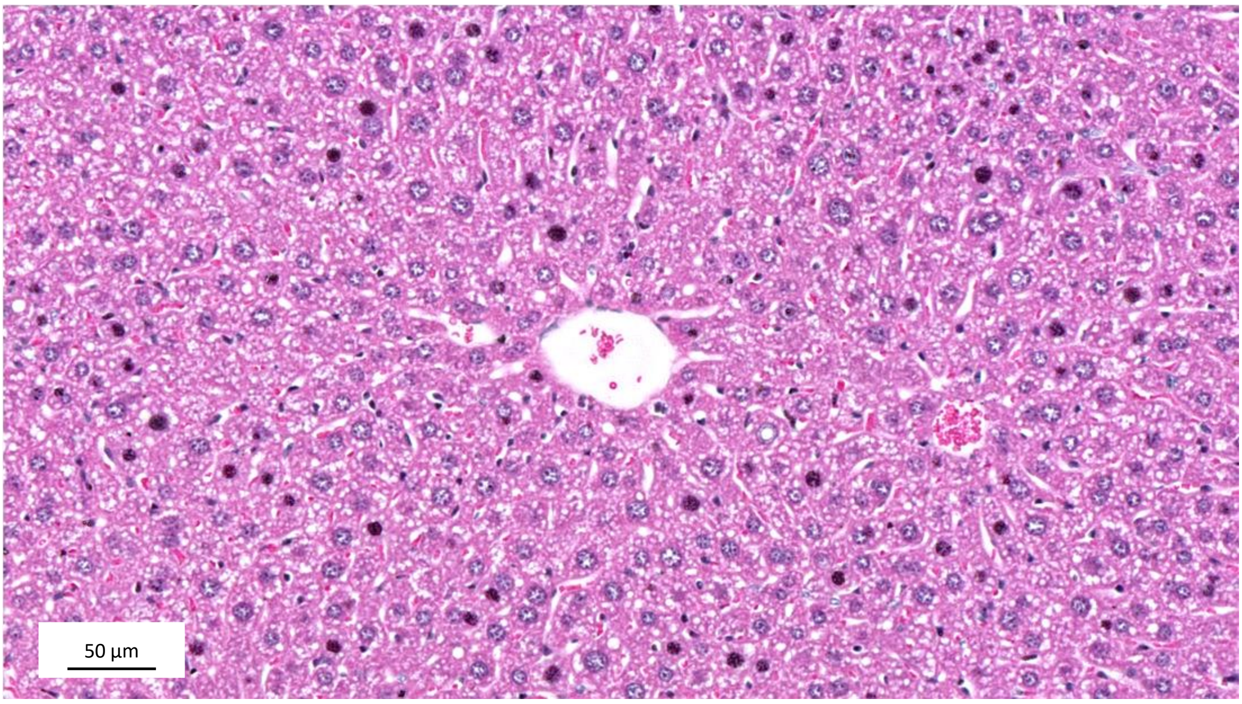

CDHFD-I-2

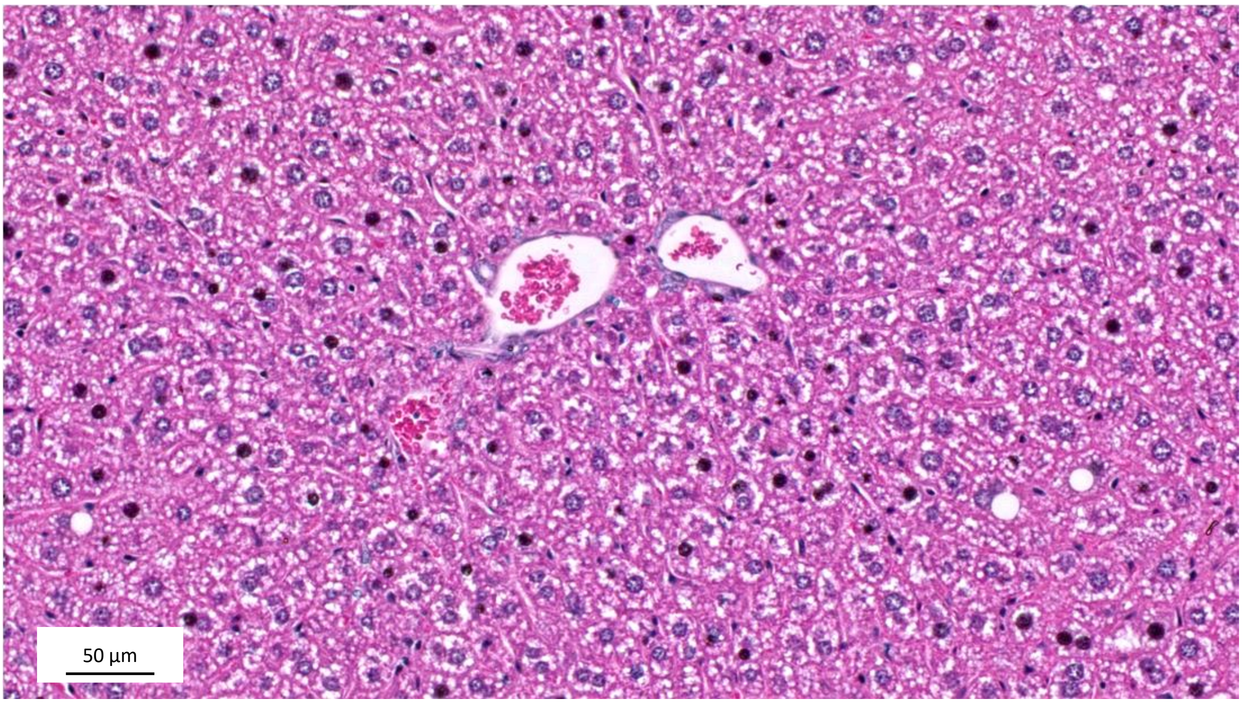

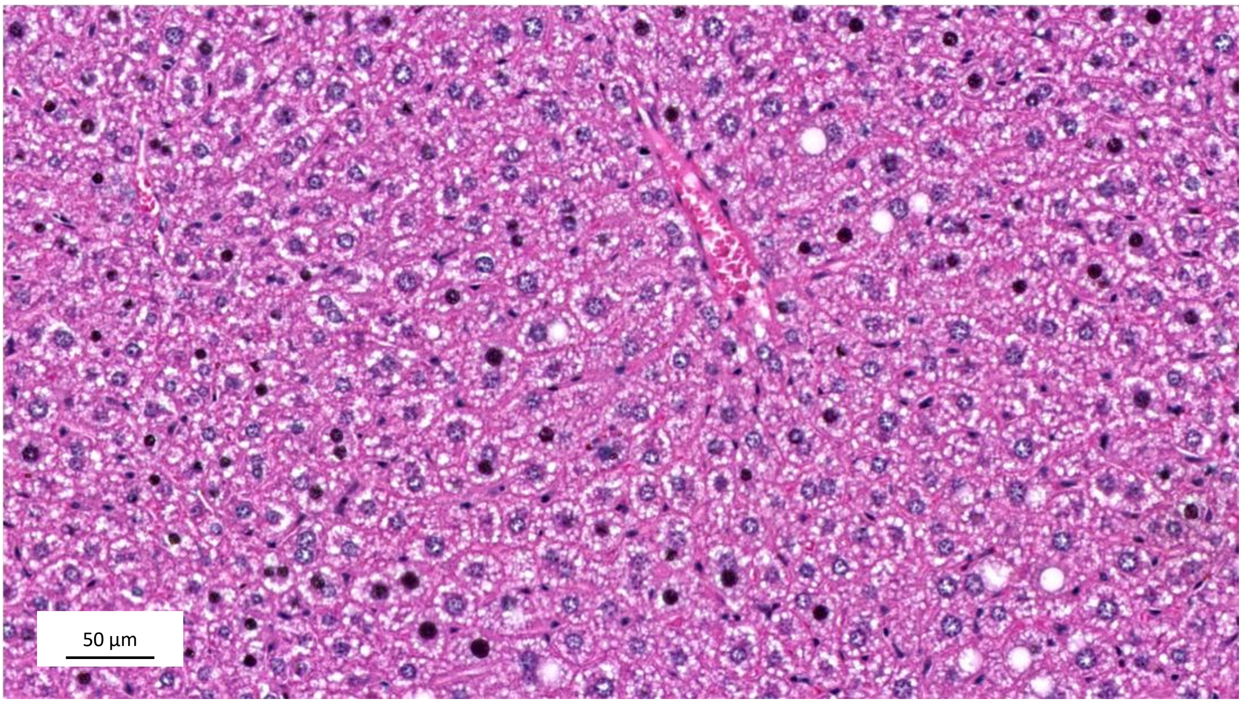

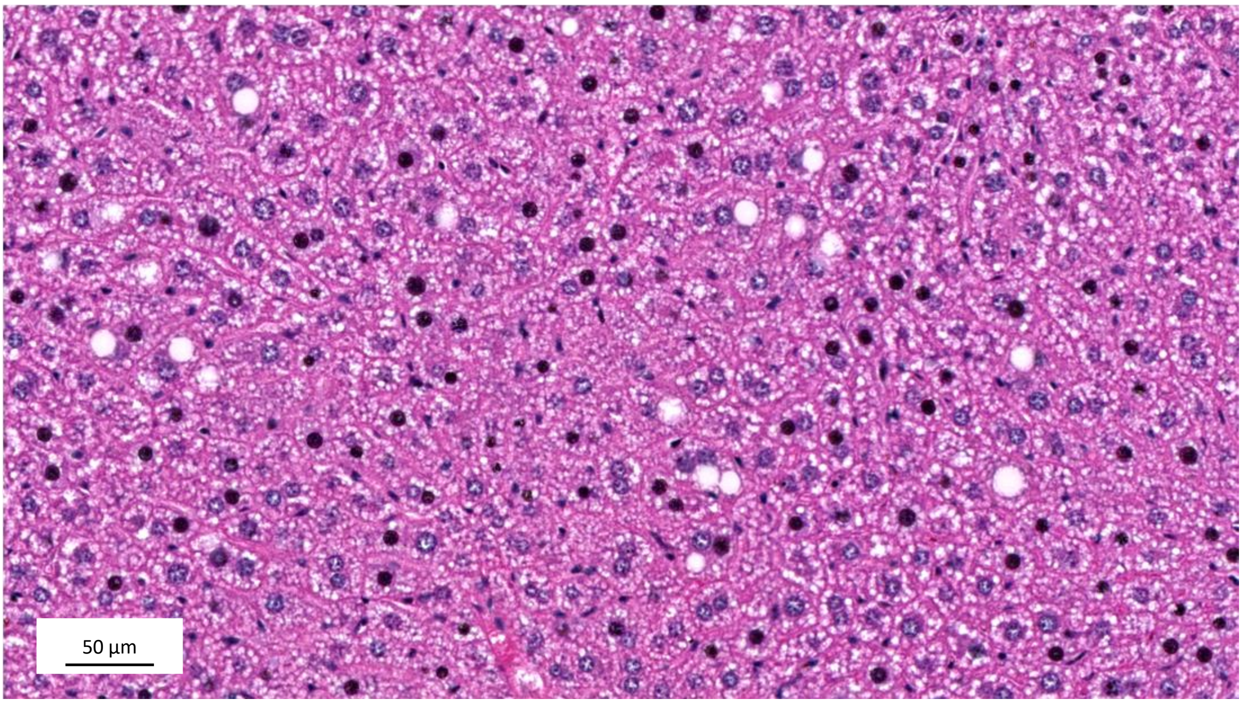

CDHFD-I-3

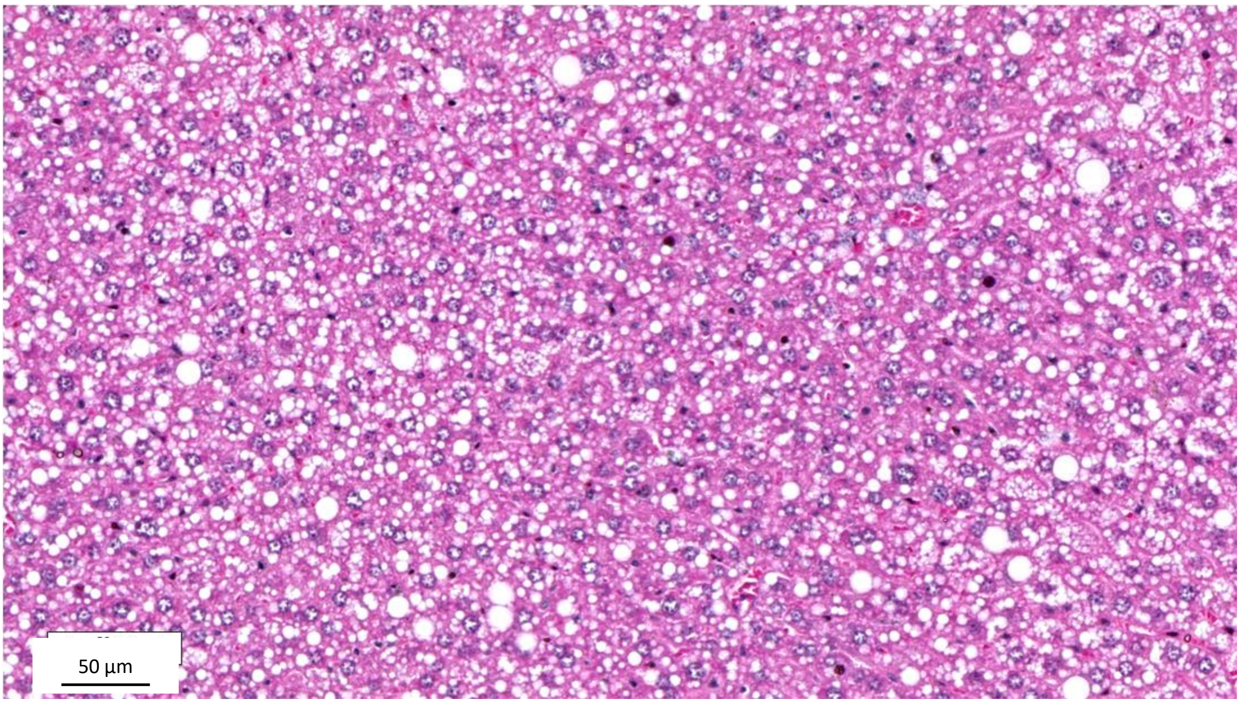

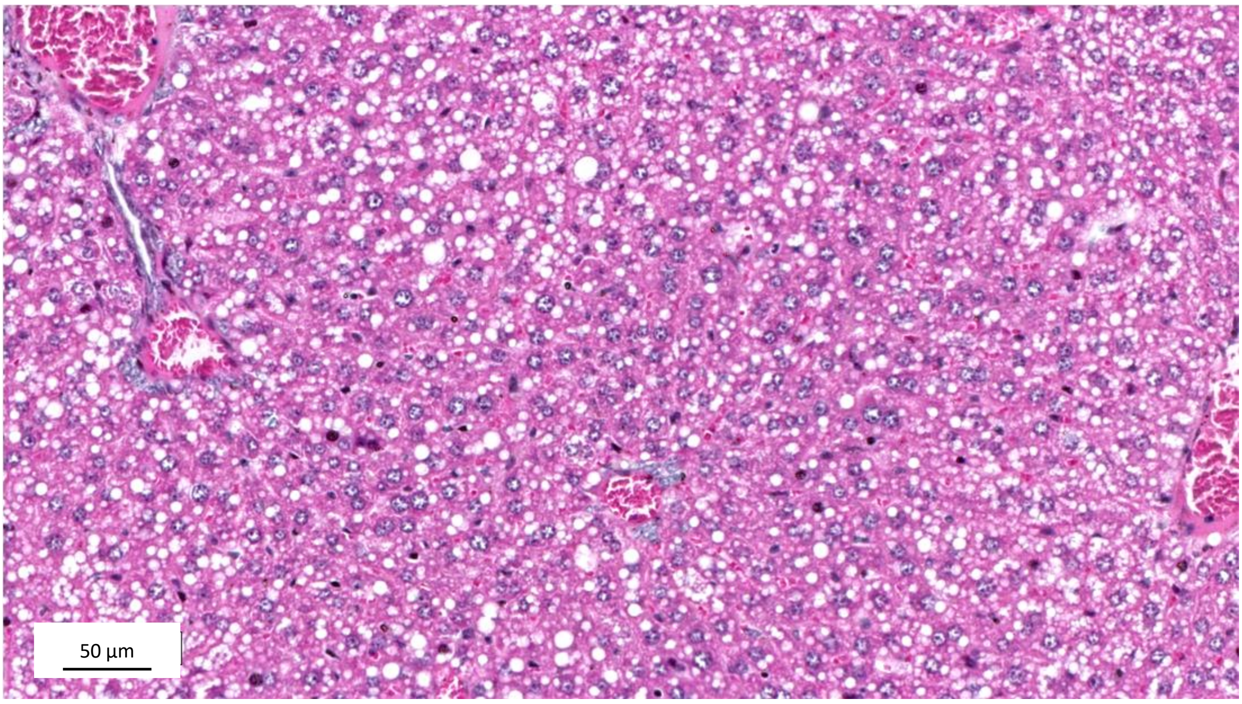

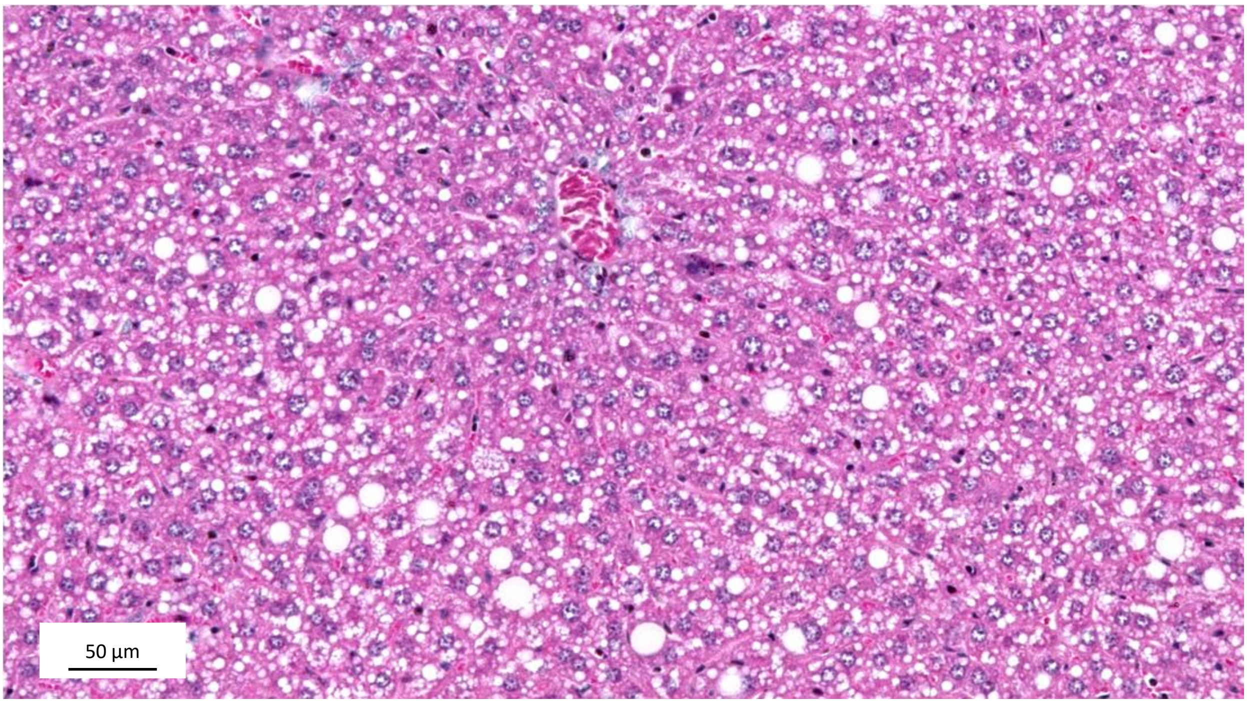

CDHFD-I-4

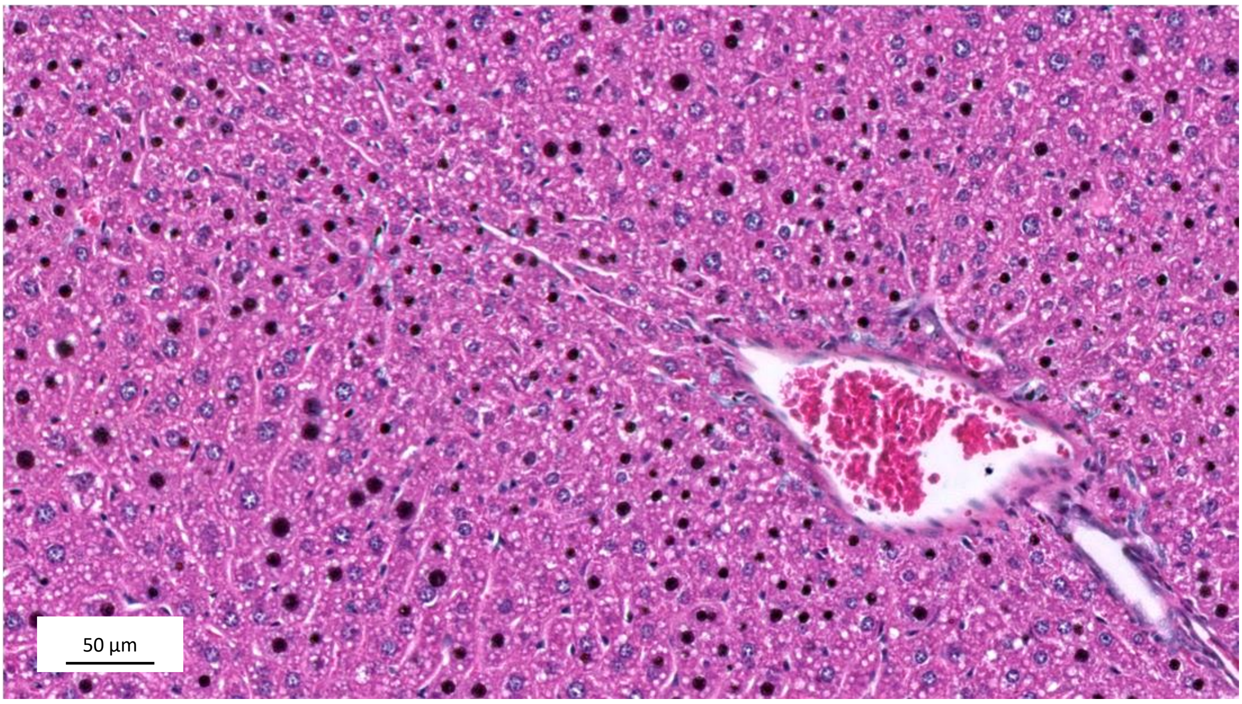

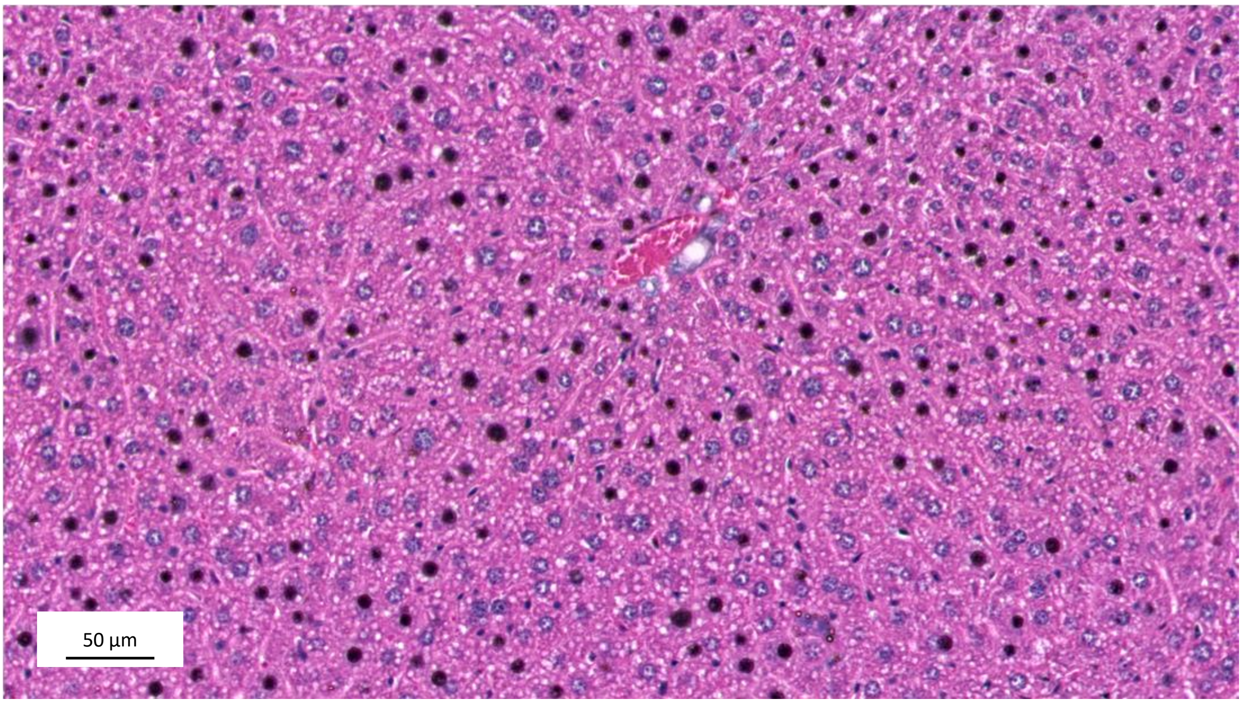

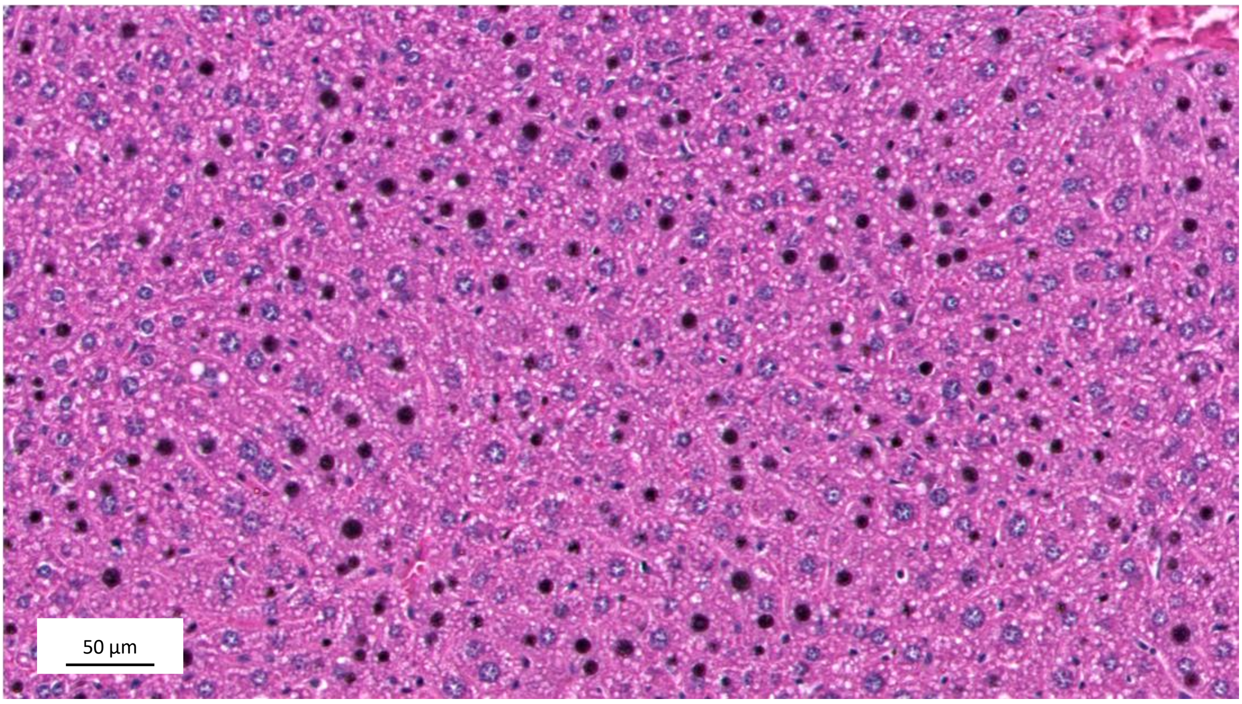

CDHFD-I-5

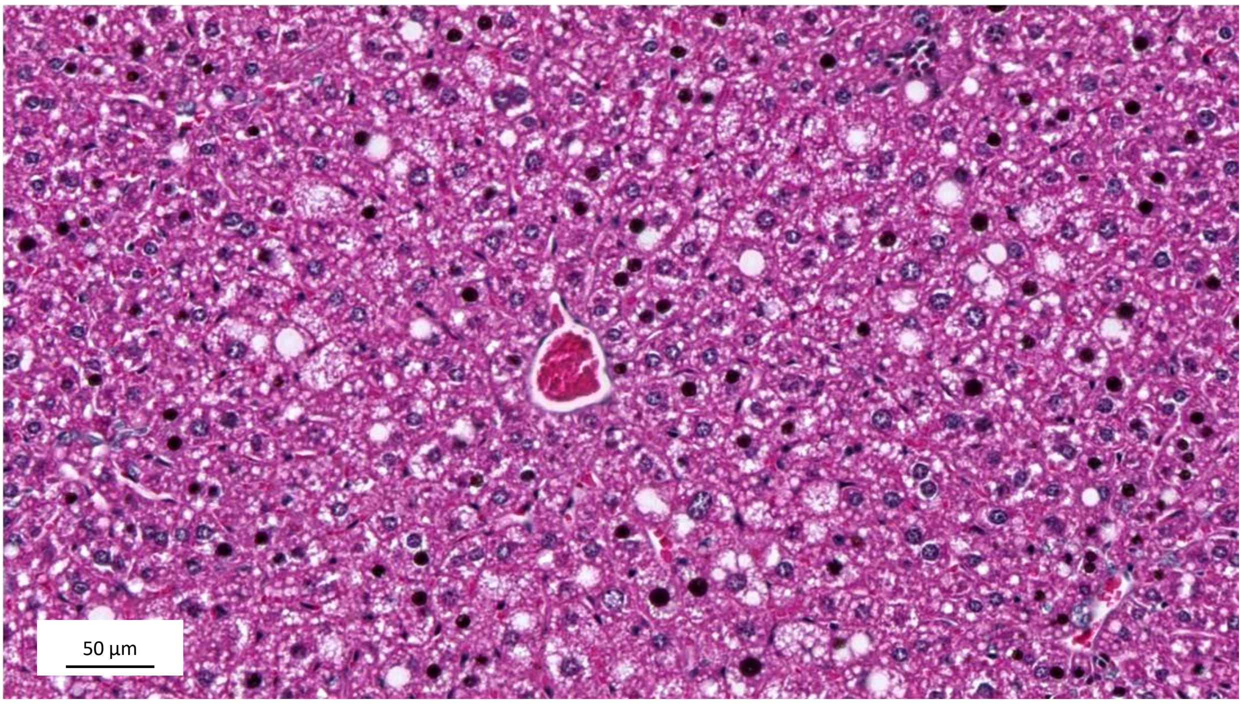

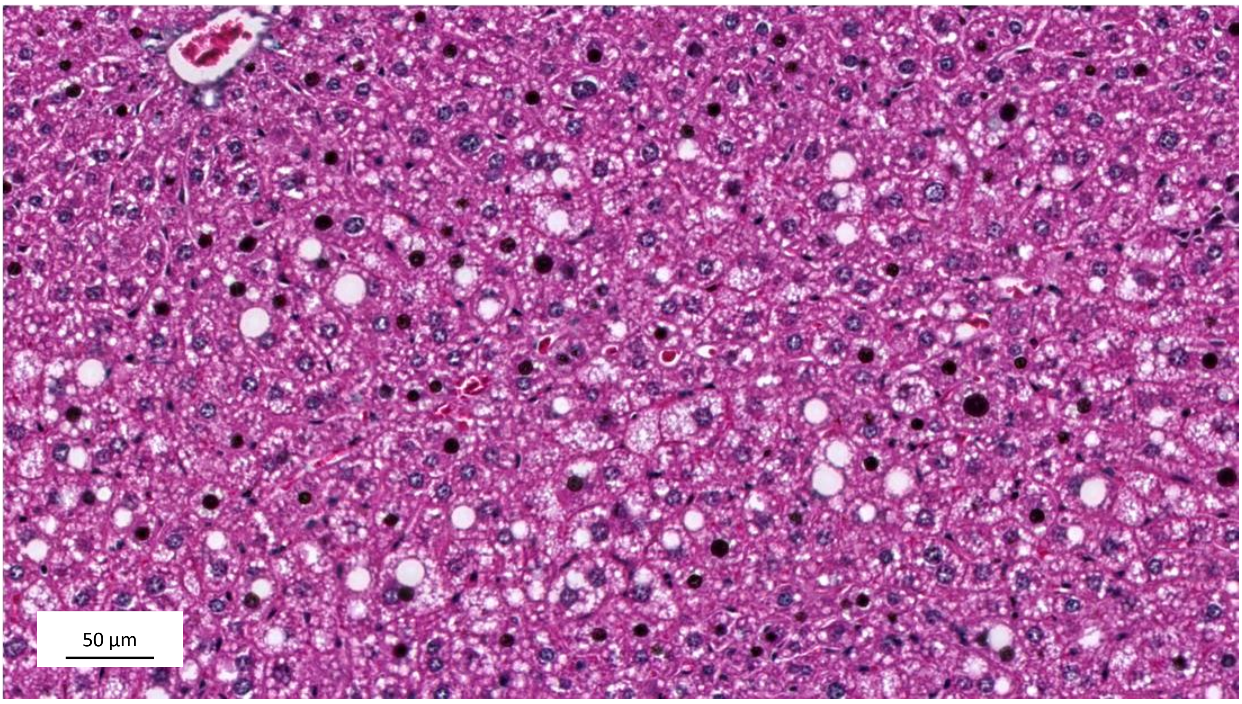

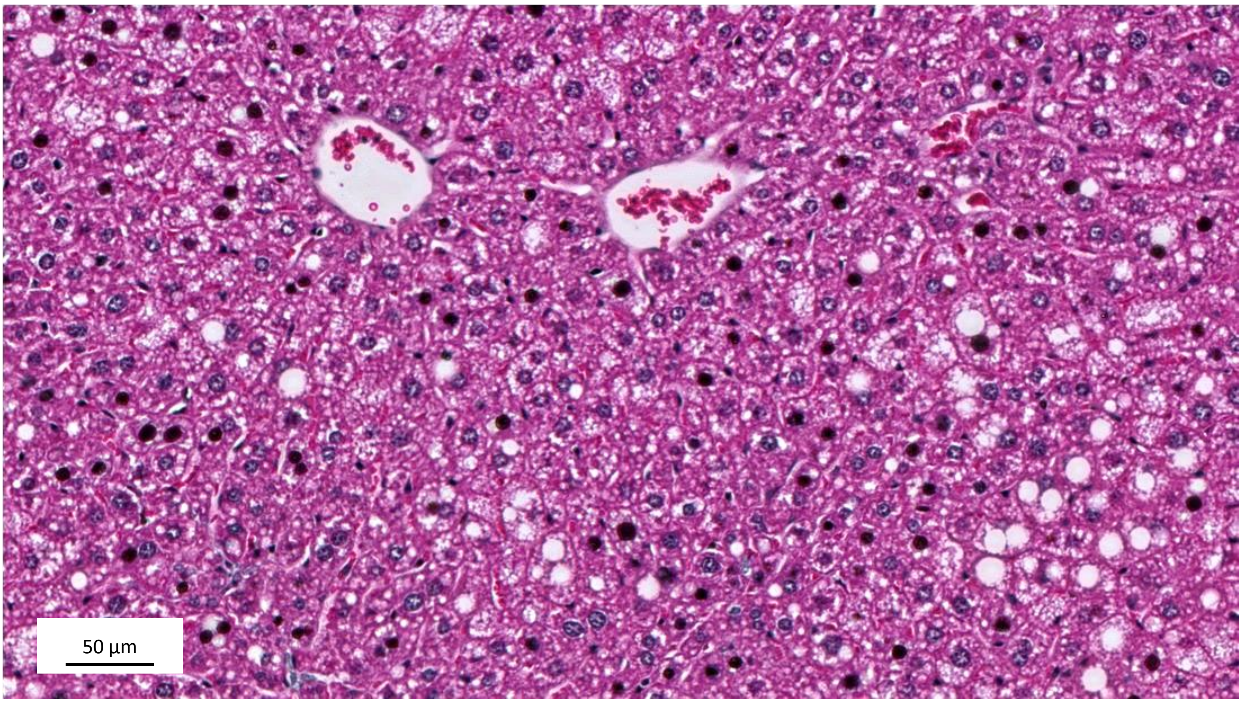

CDHFD-I-6

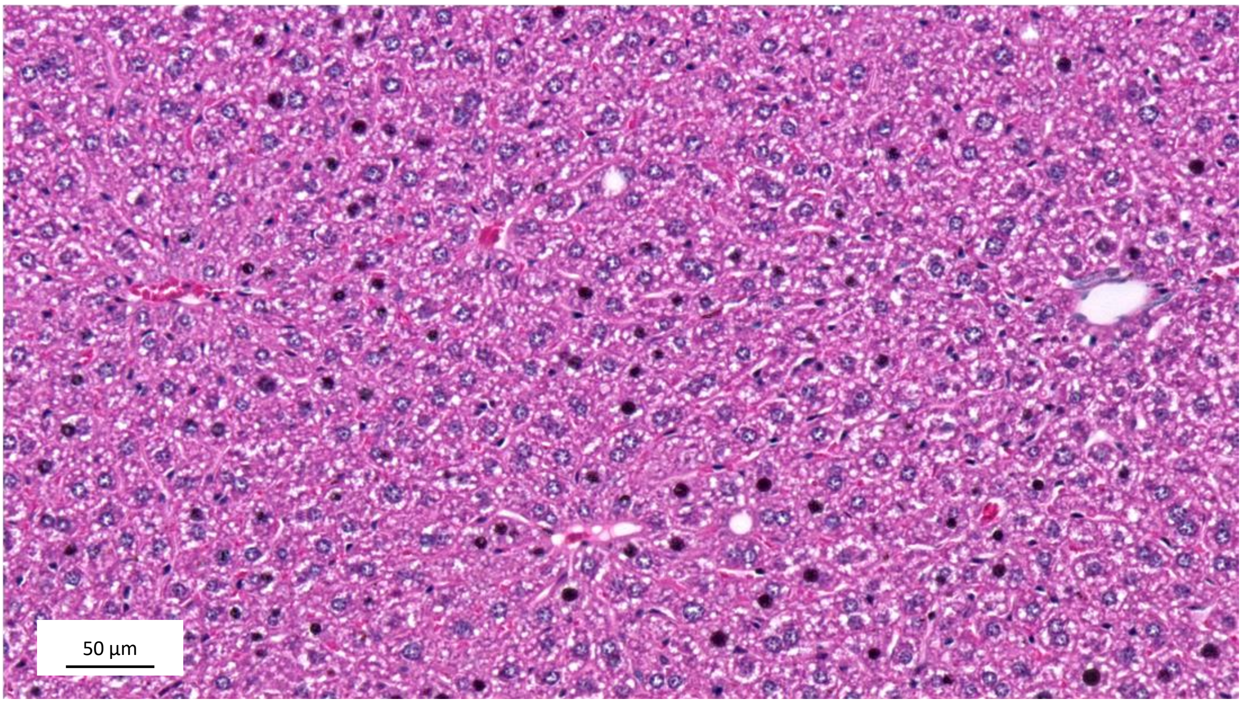

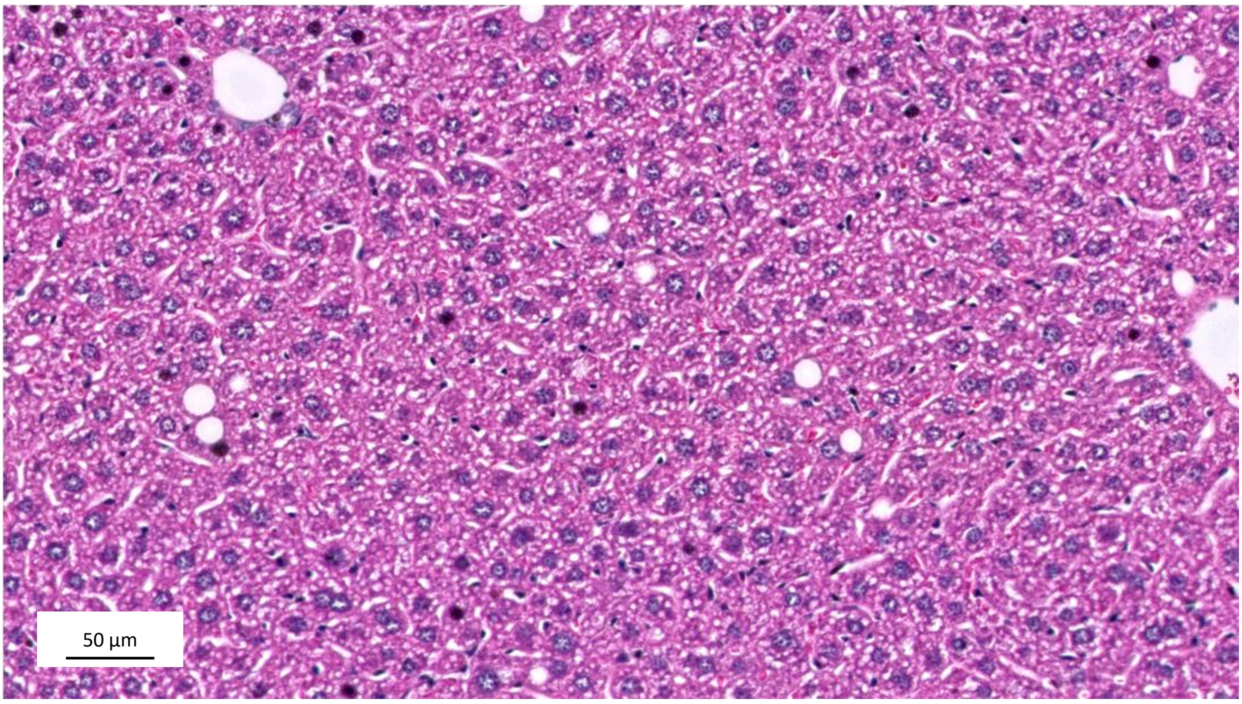

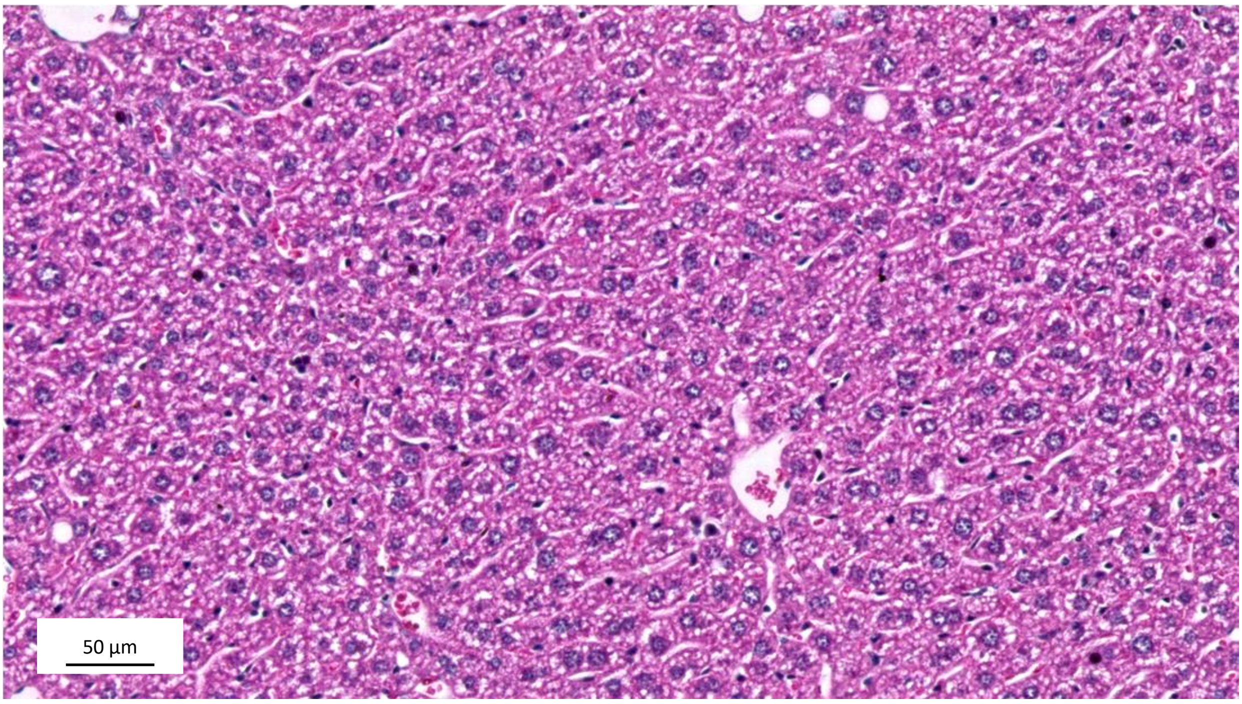

CDHFD-I-7

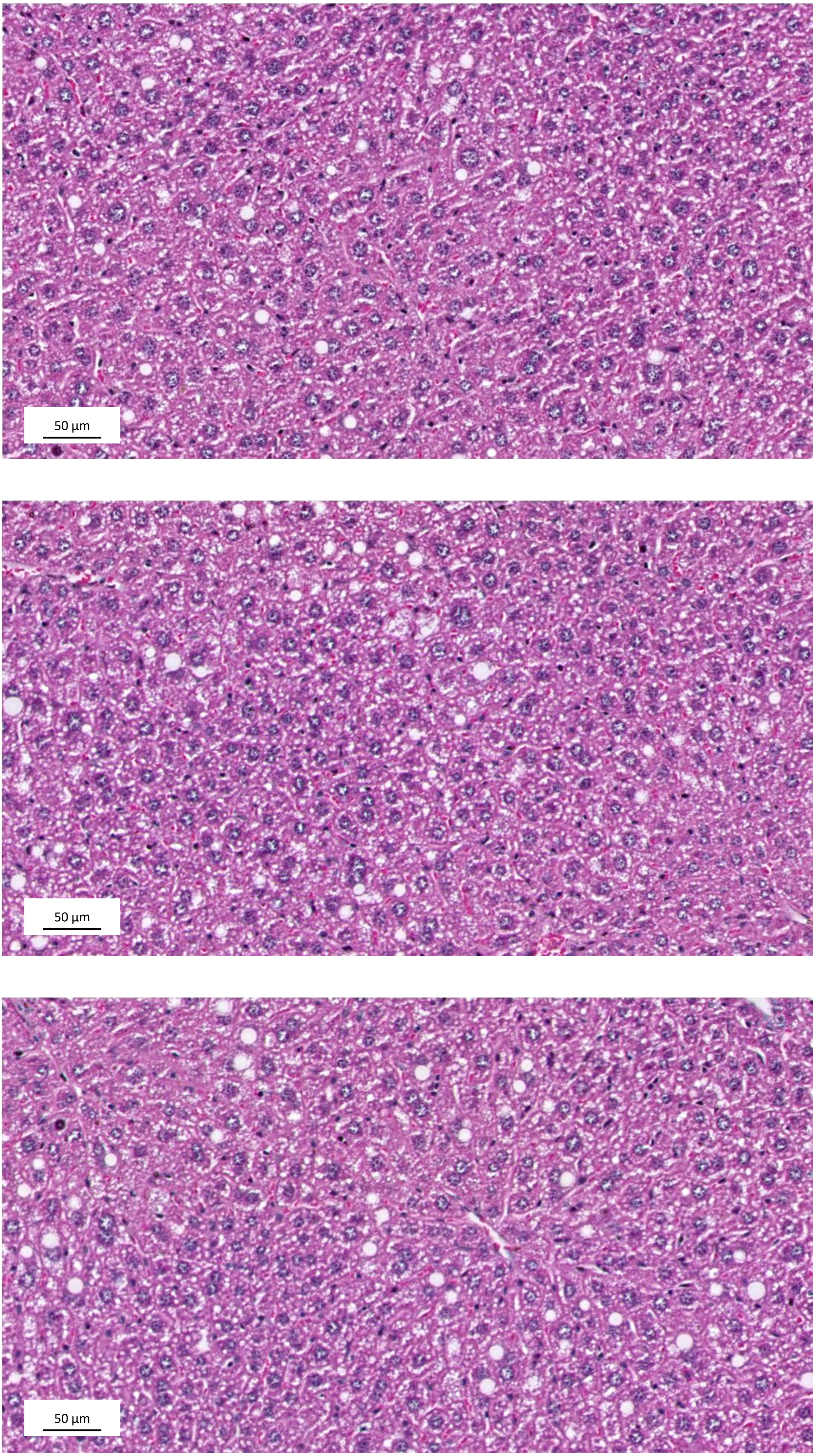

CDHFD-I-8

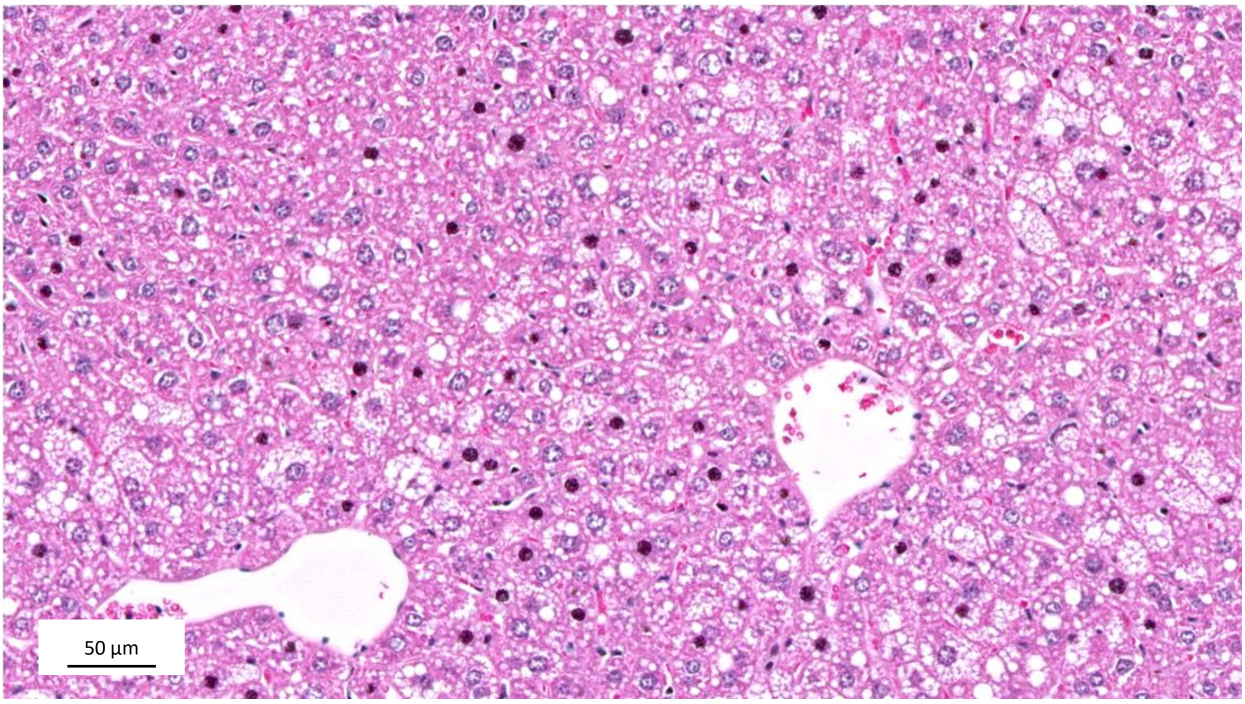

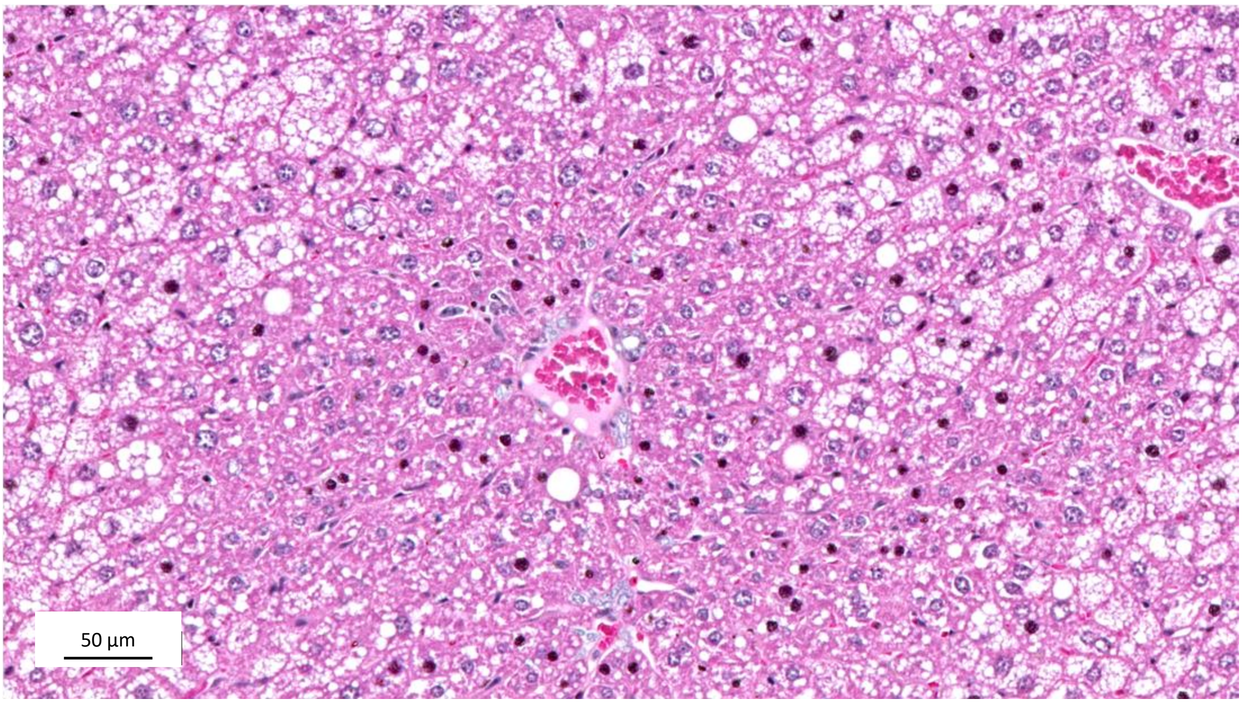

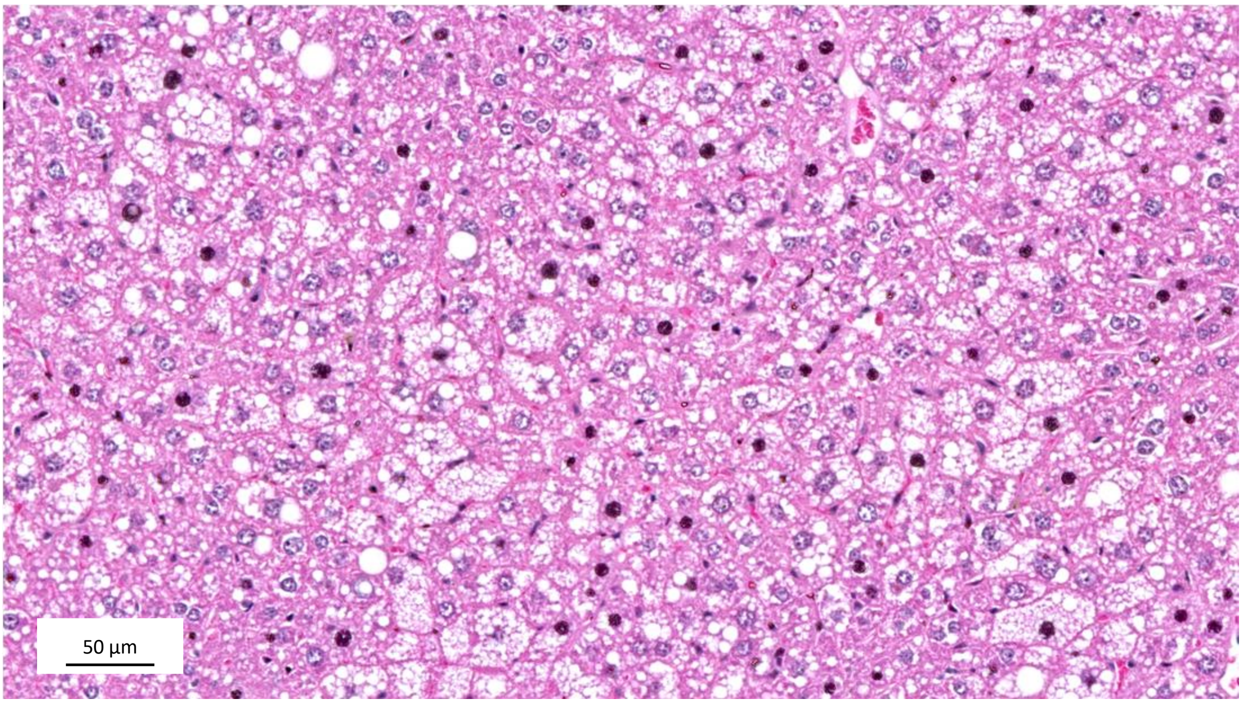

CDHFD-I-9

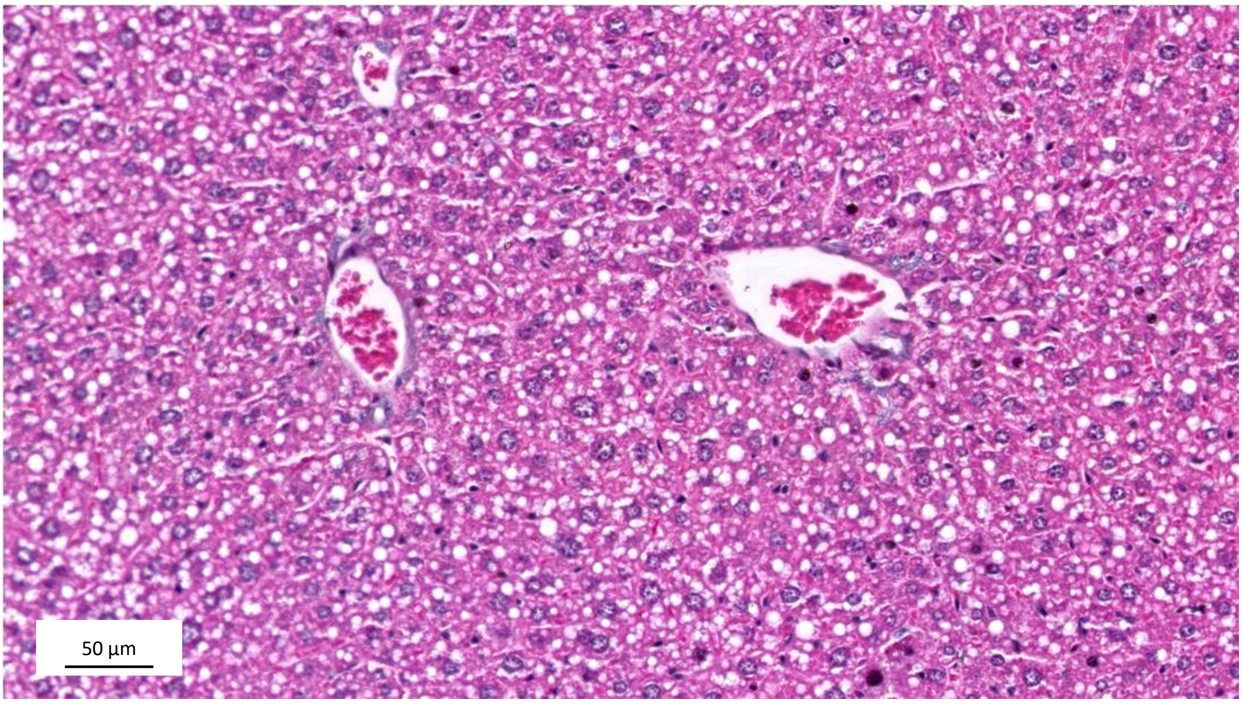

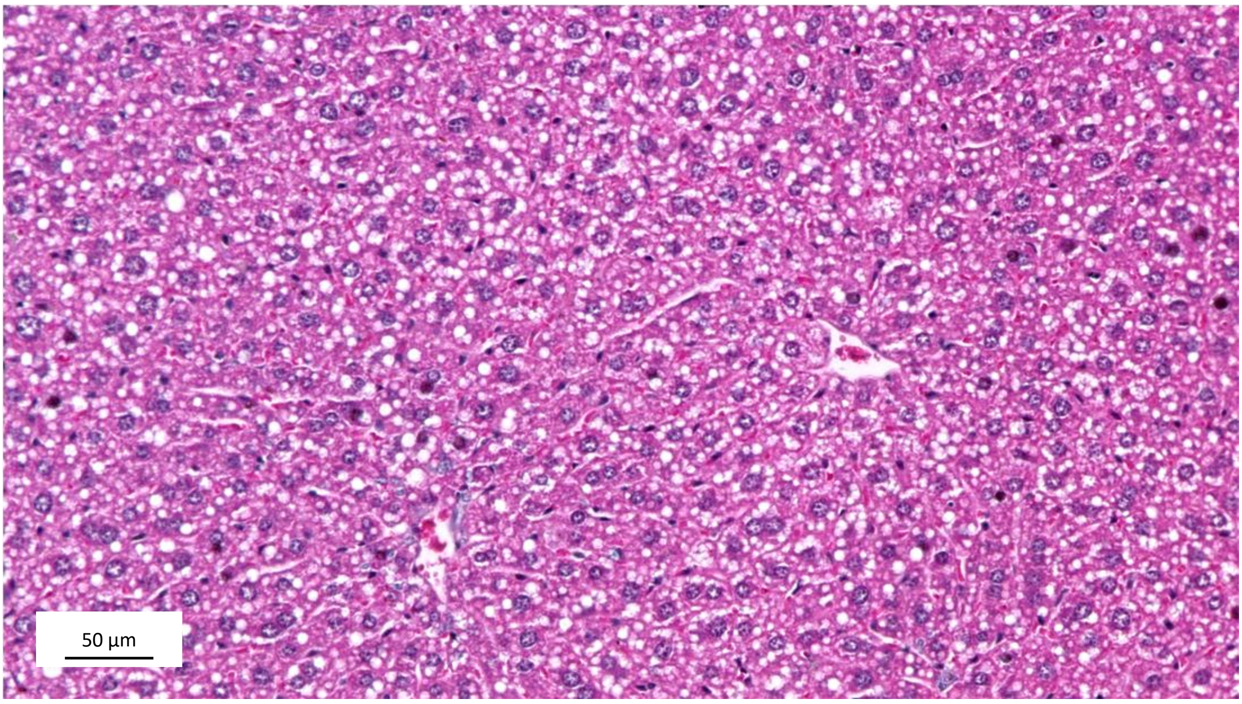

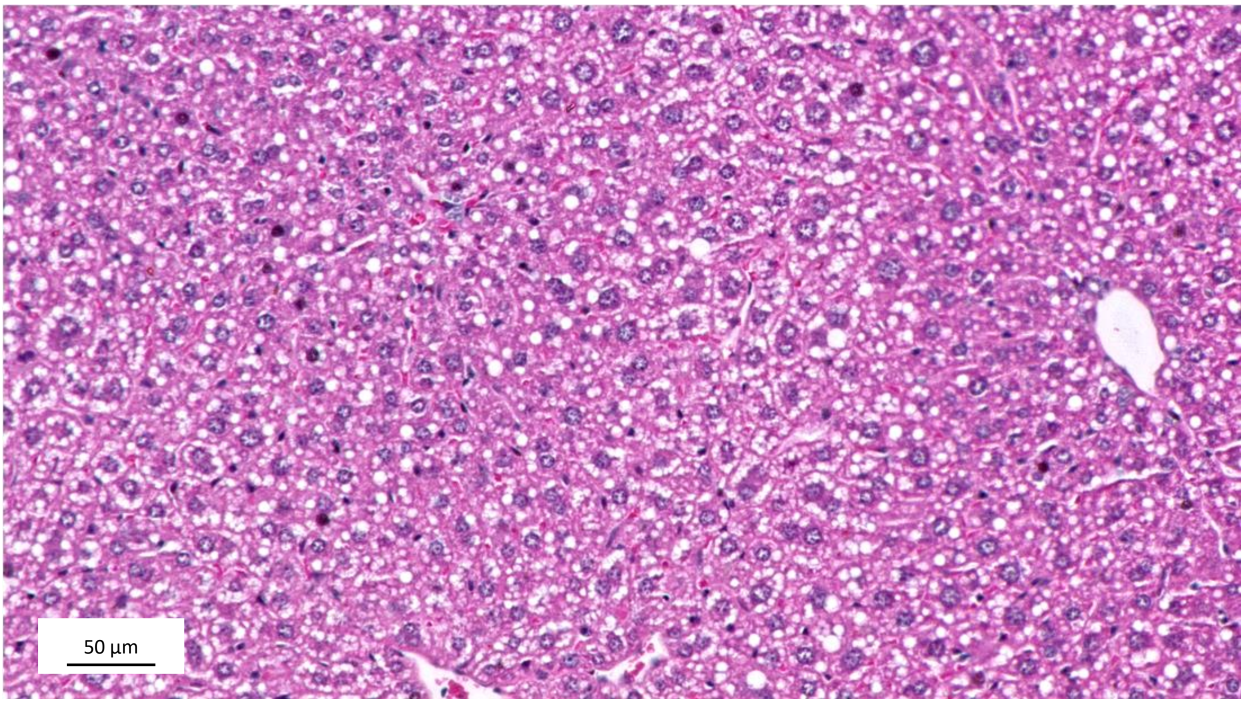

CDHFD-I-10

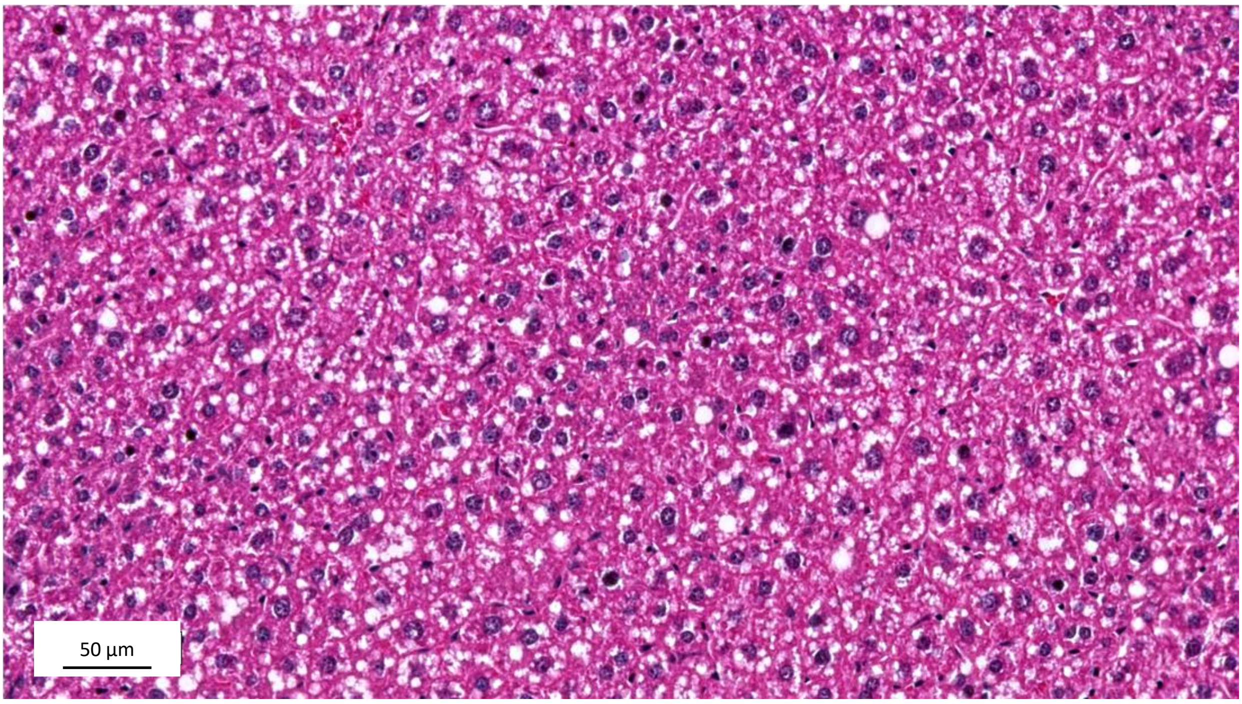

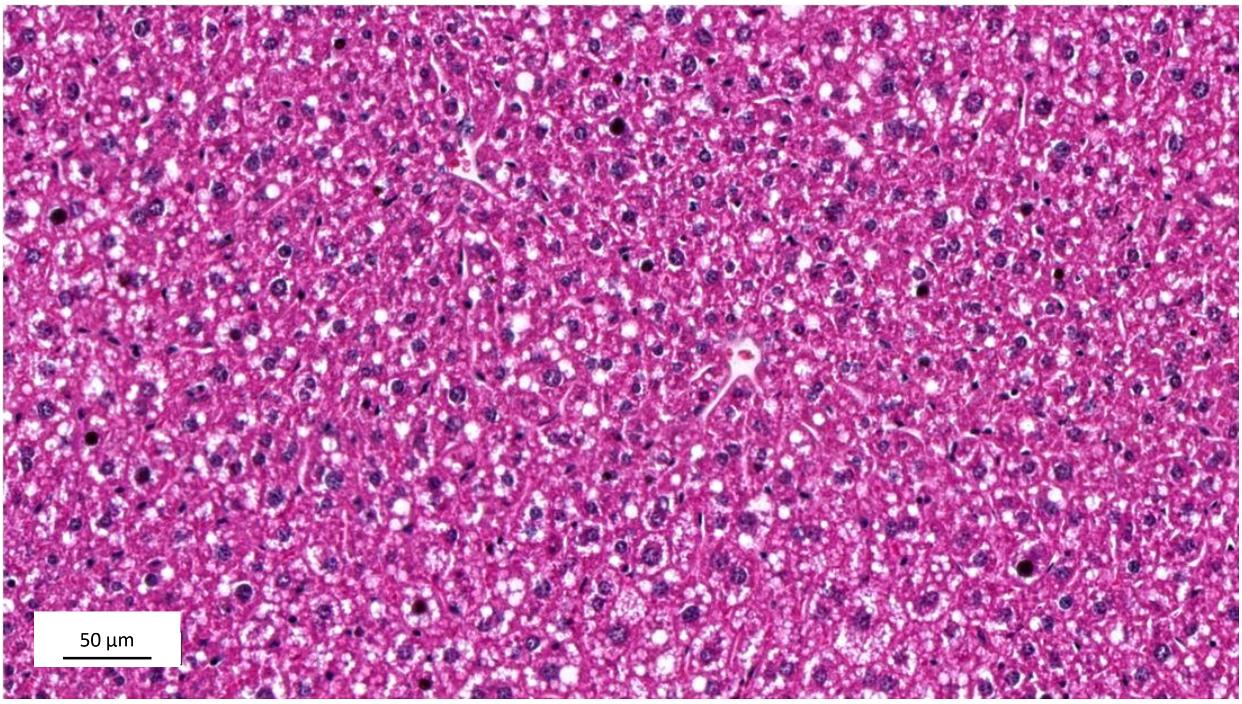

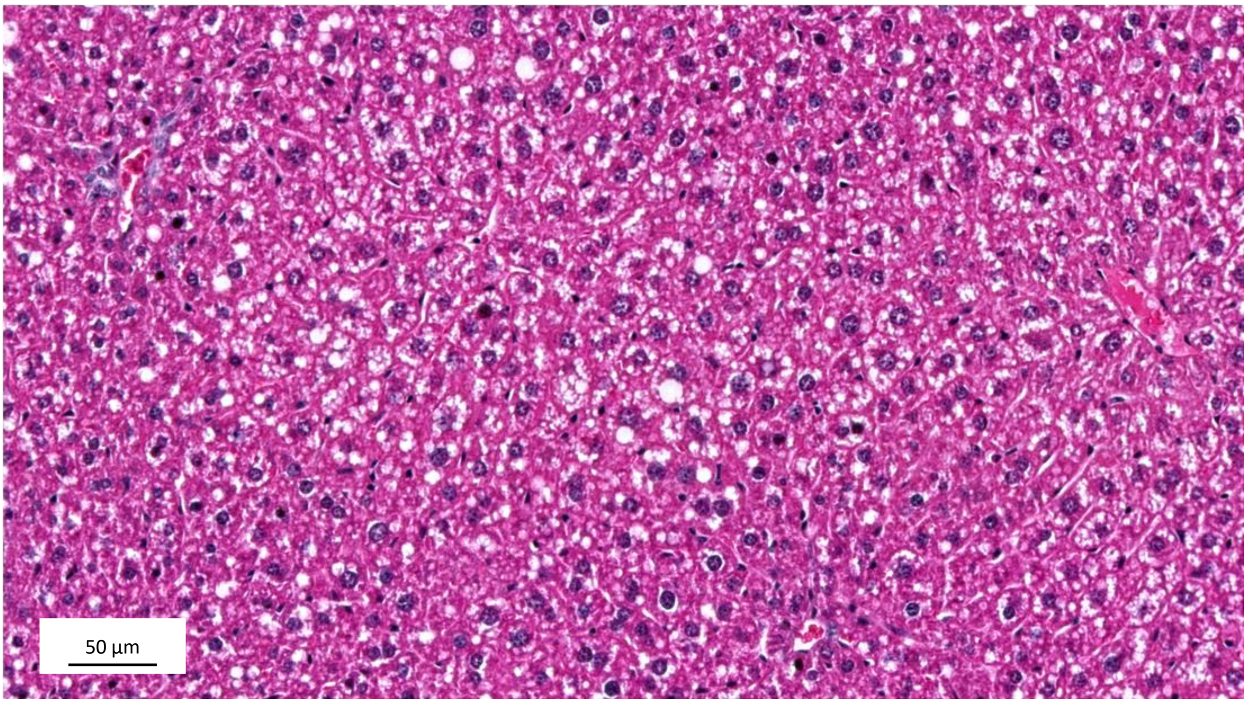

CDHFD-I-11

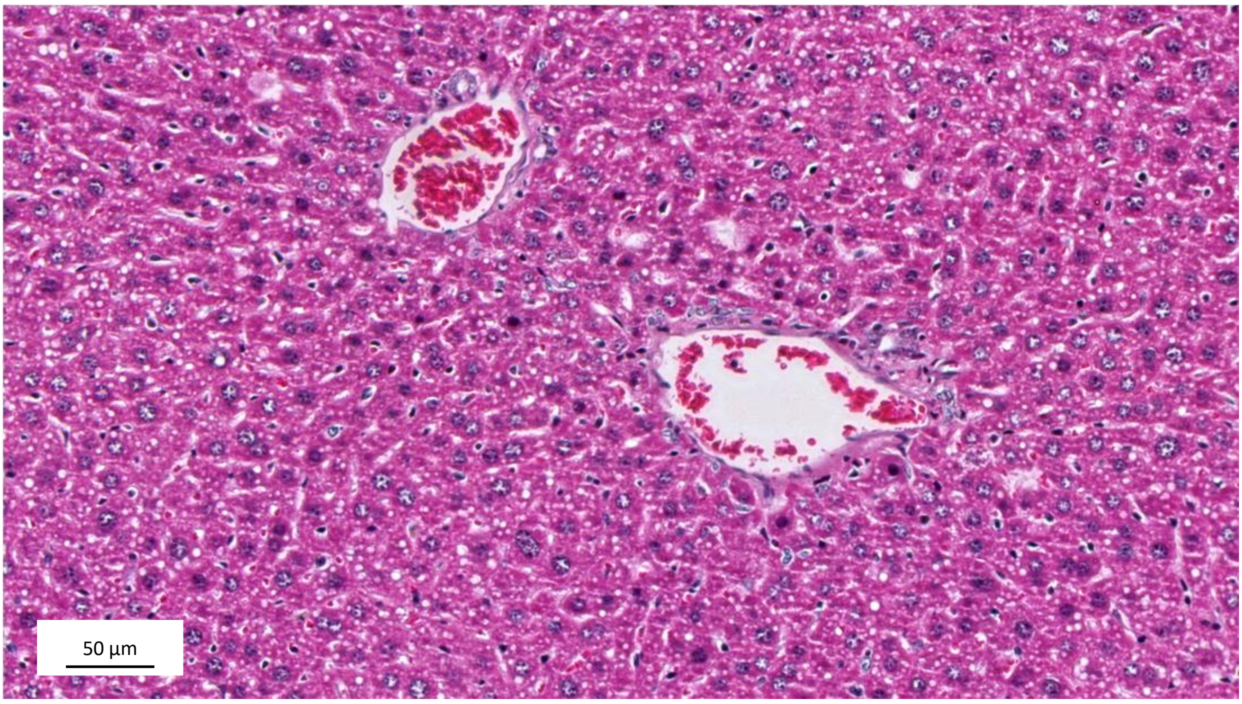

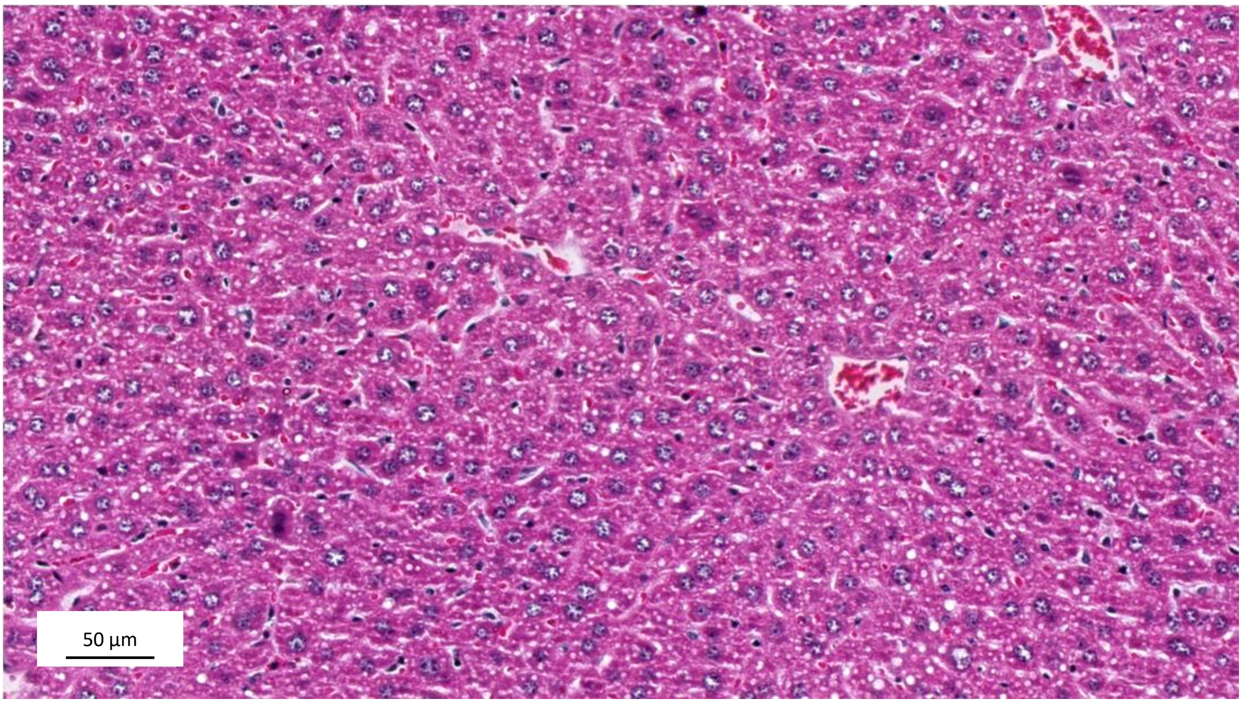

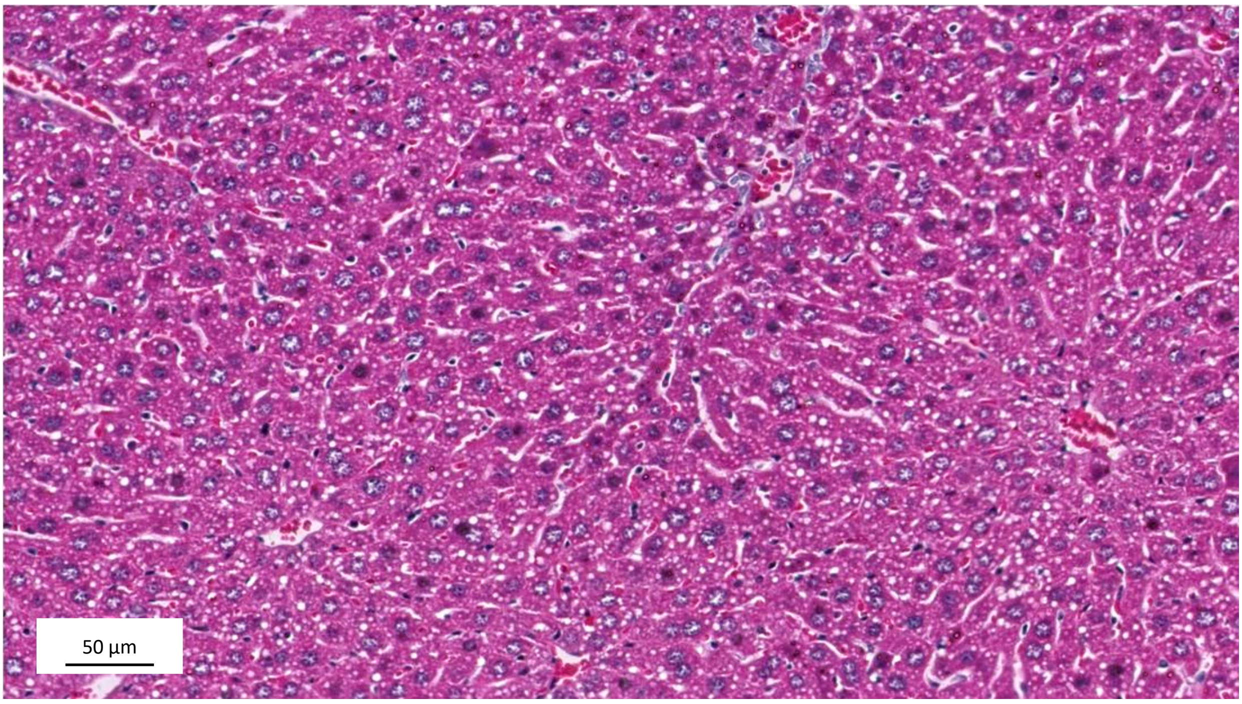

CDHFD-I-12

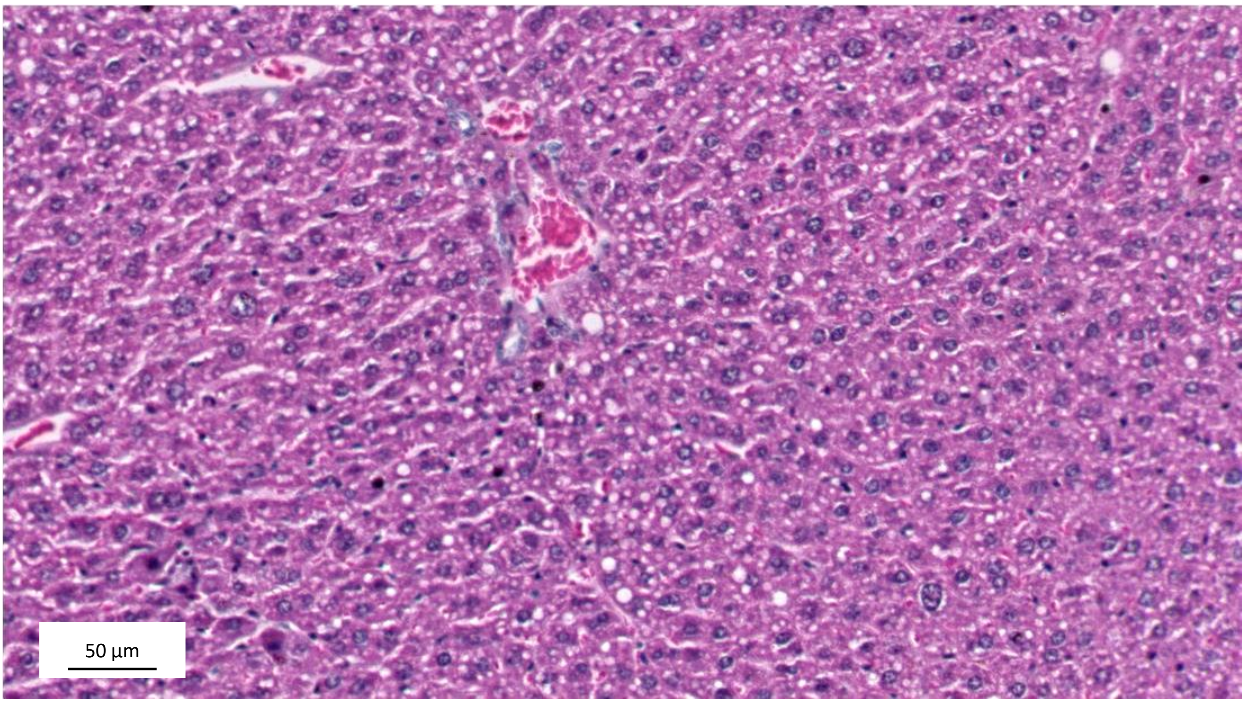

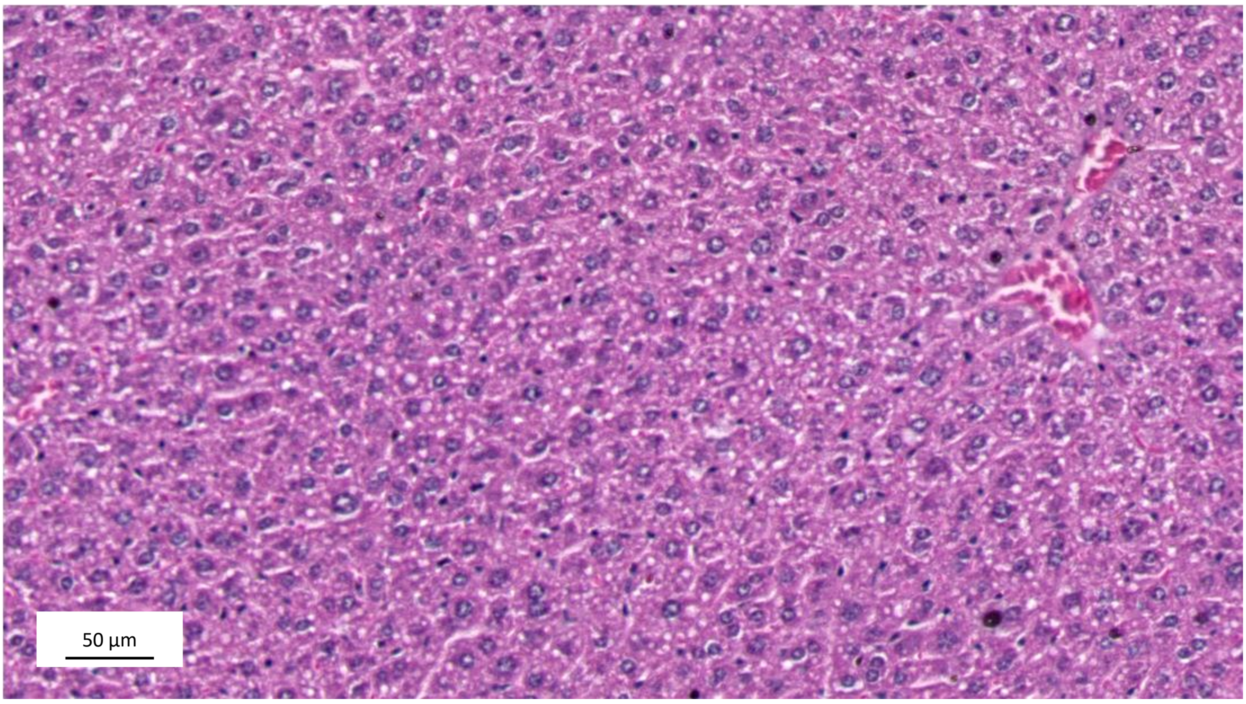

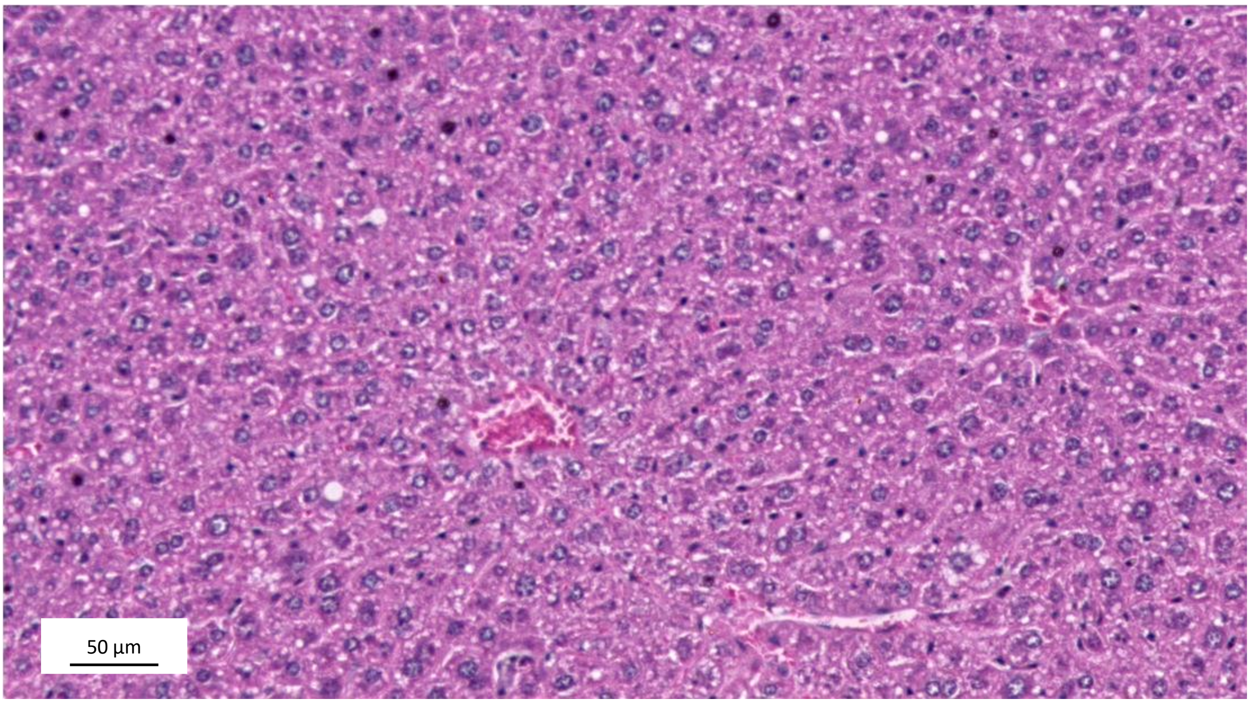

CDHFD-I-13

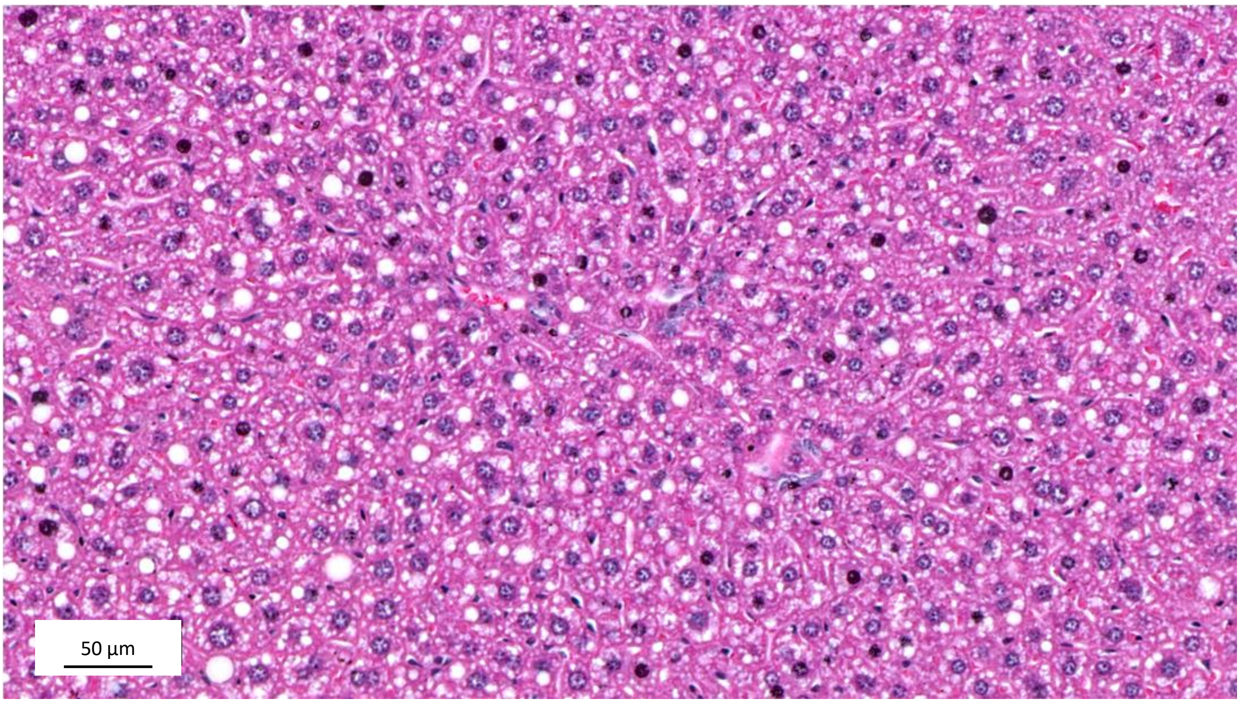

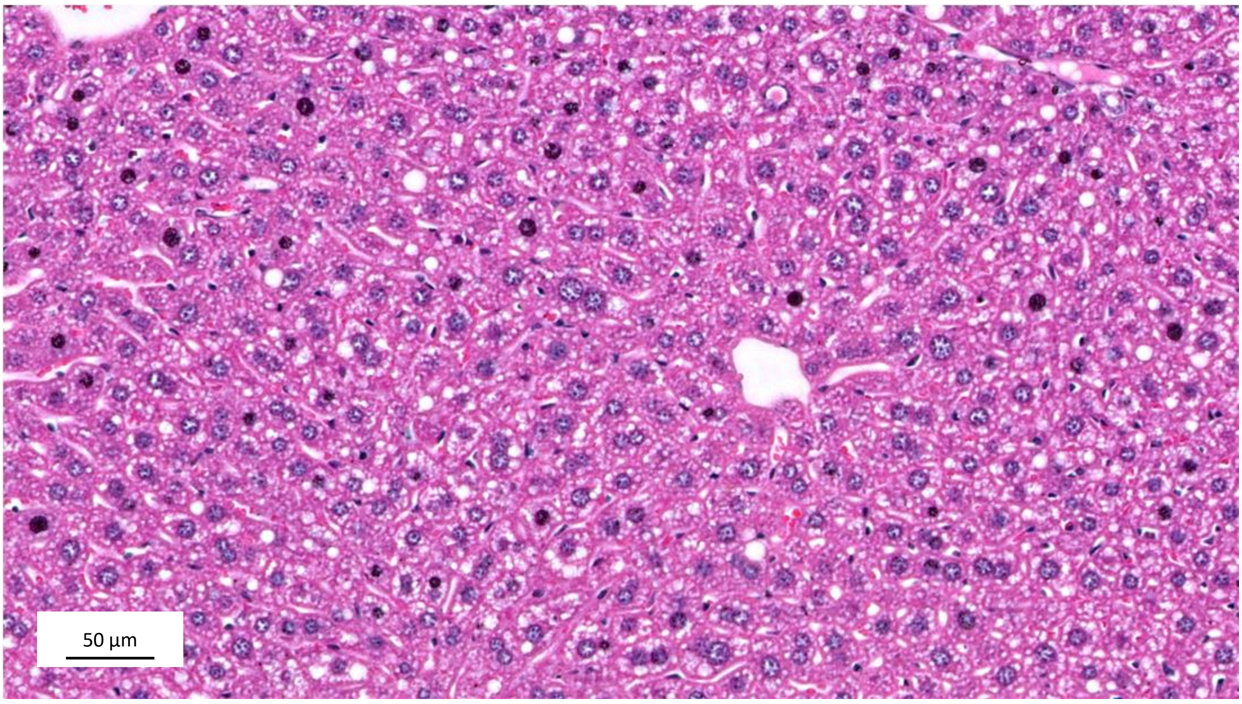

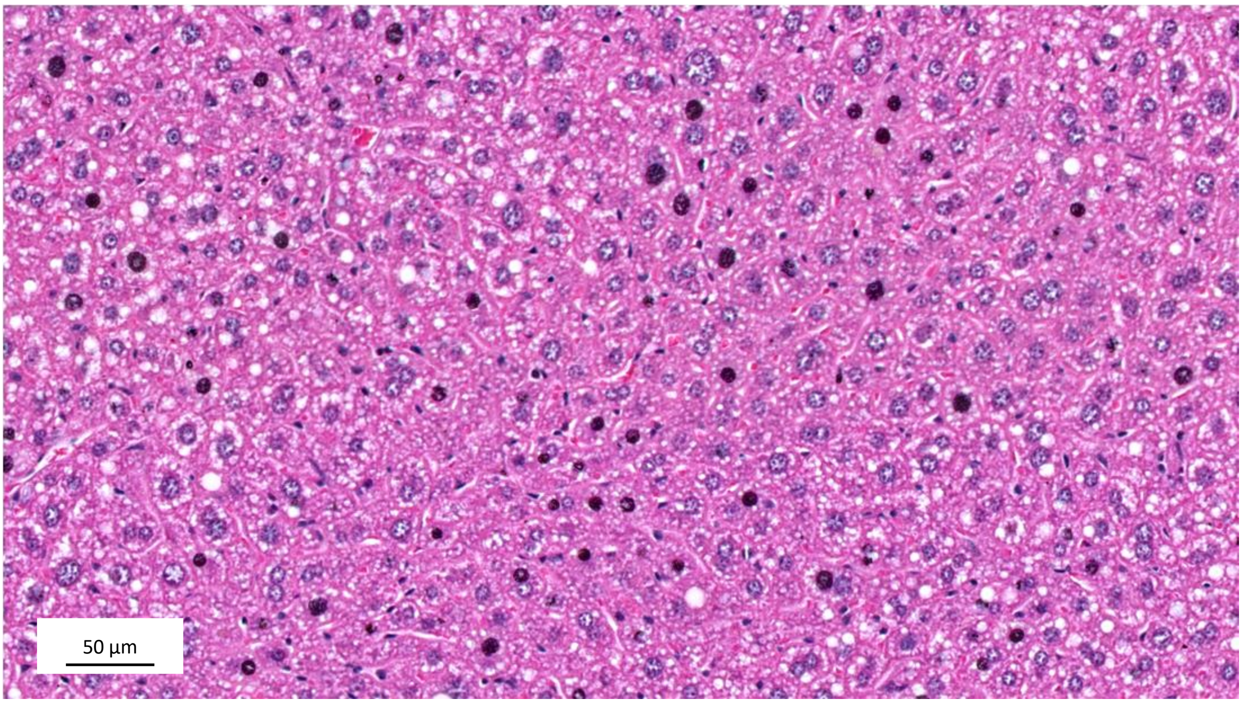

CDHFD-I-14

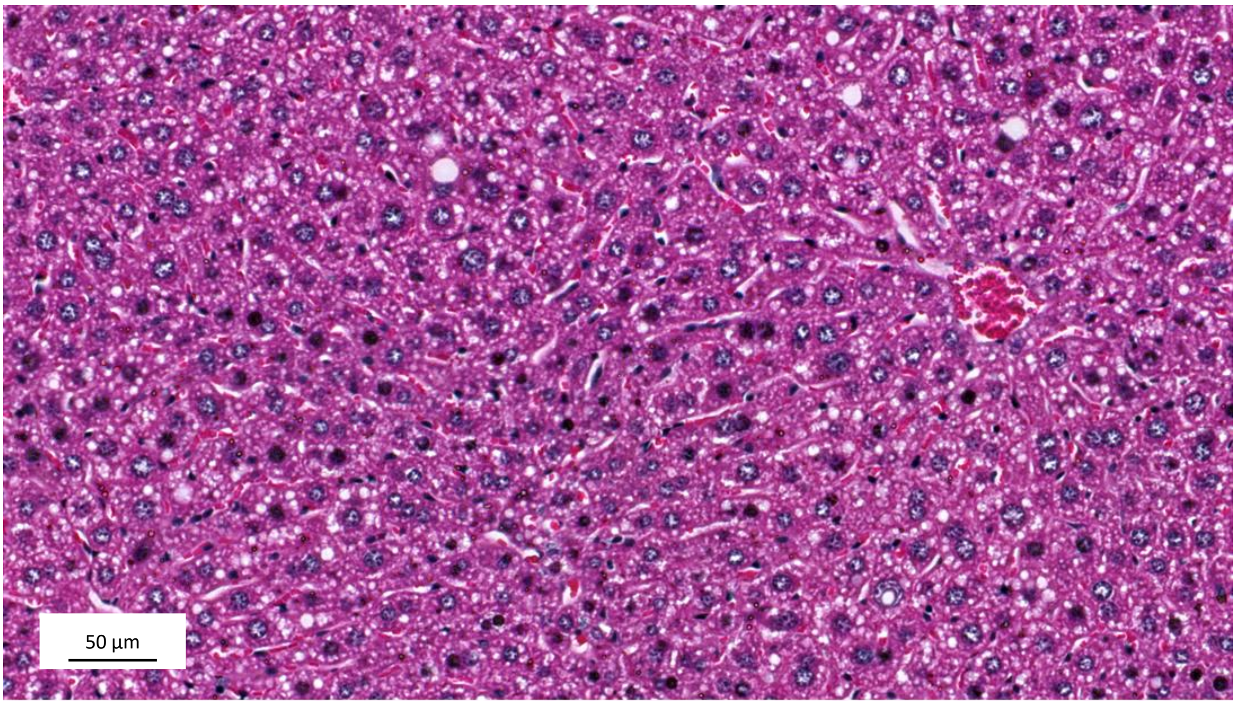

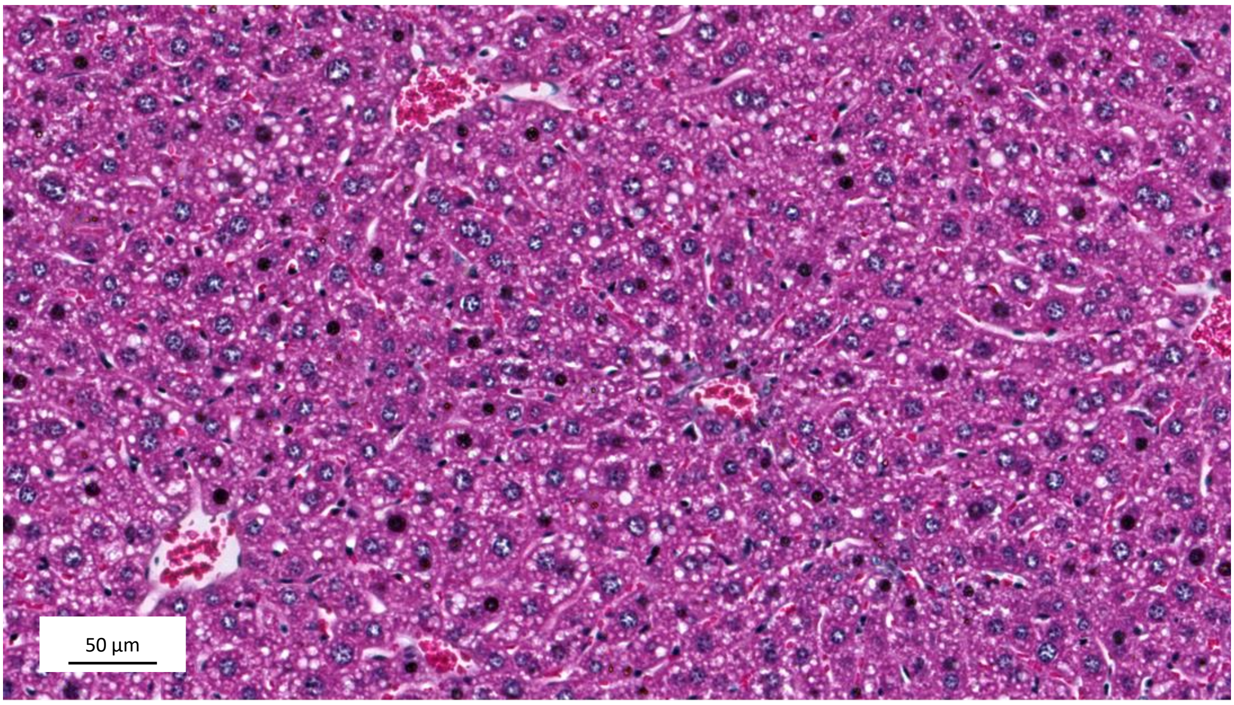

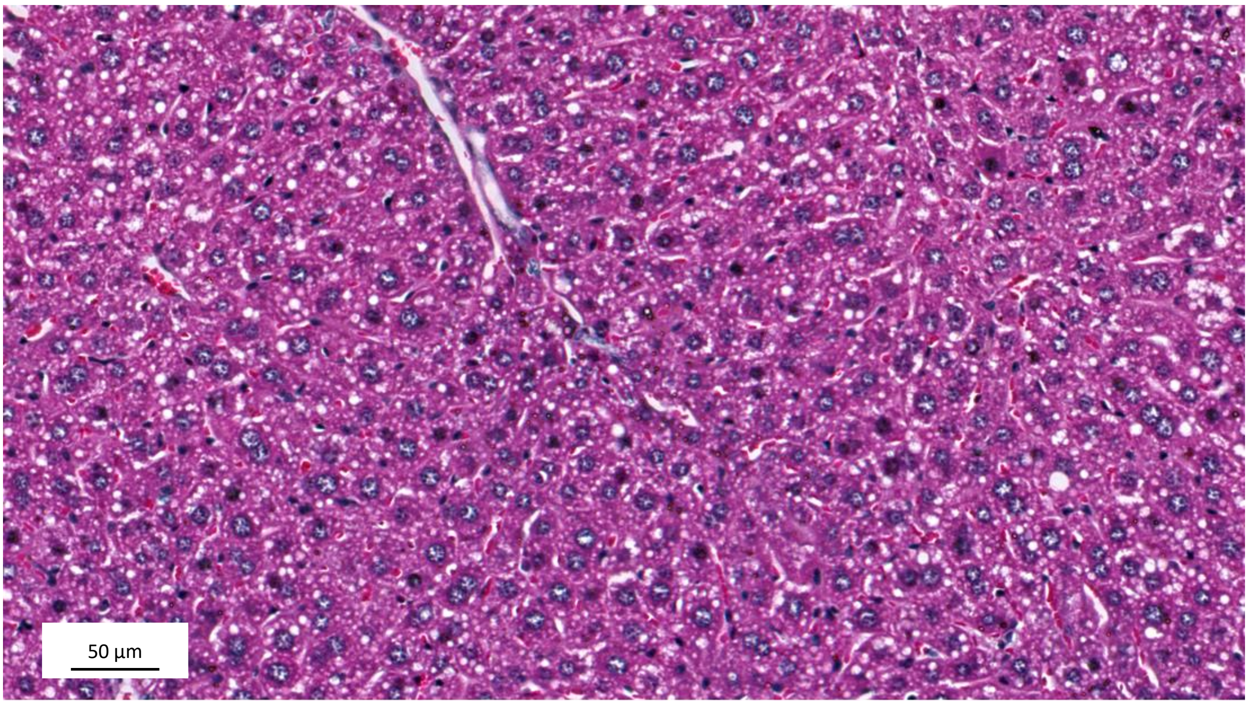

CDHFD-I-15

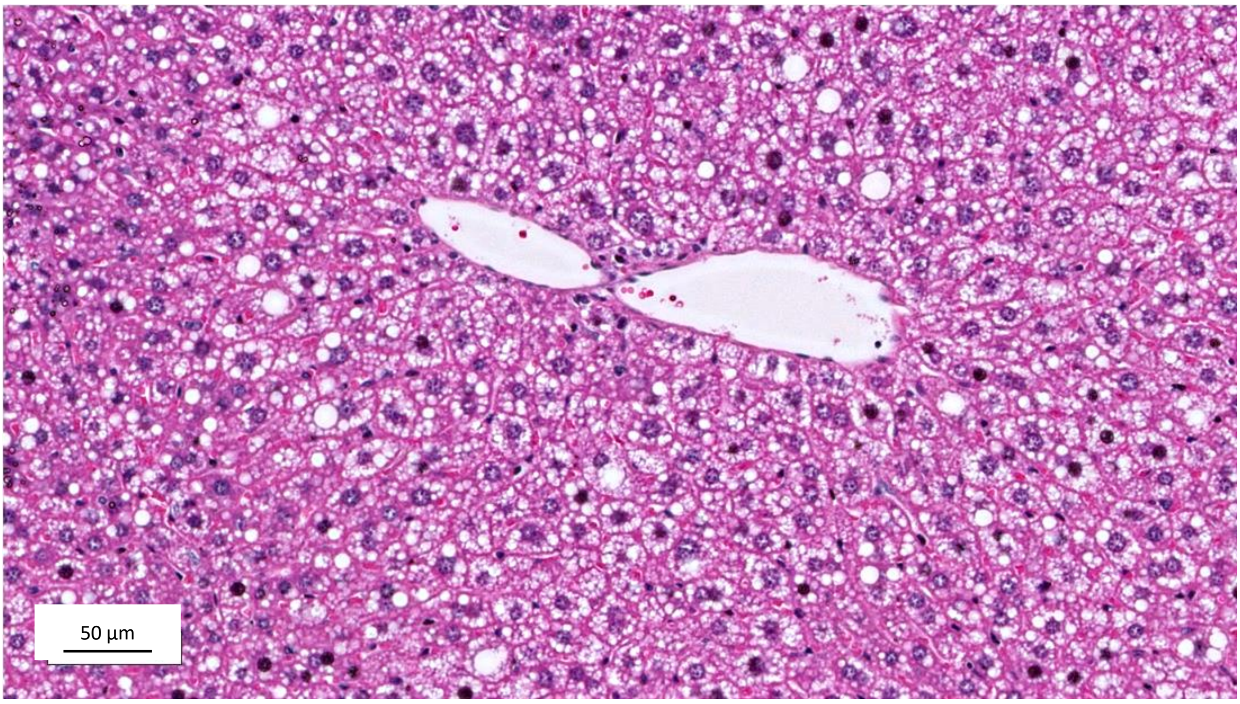

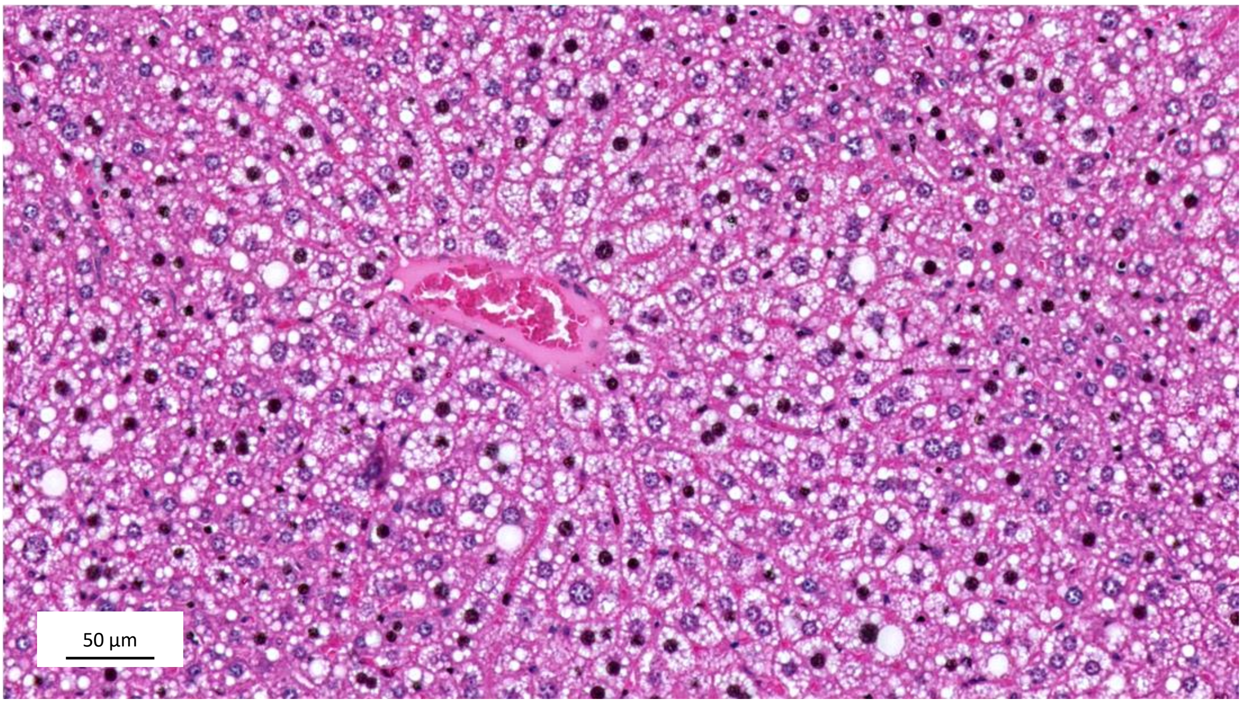

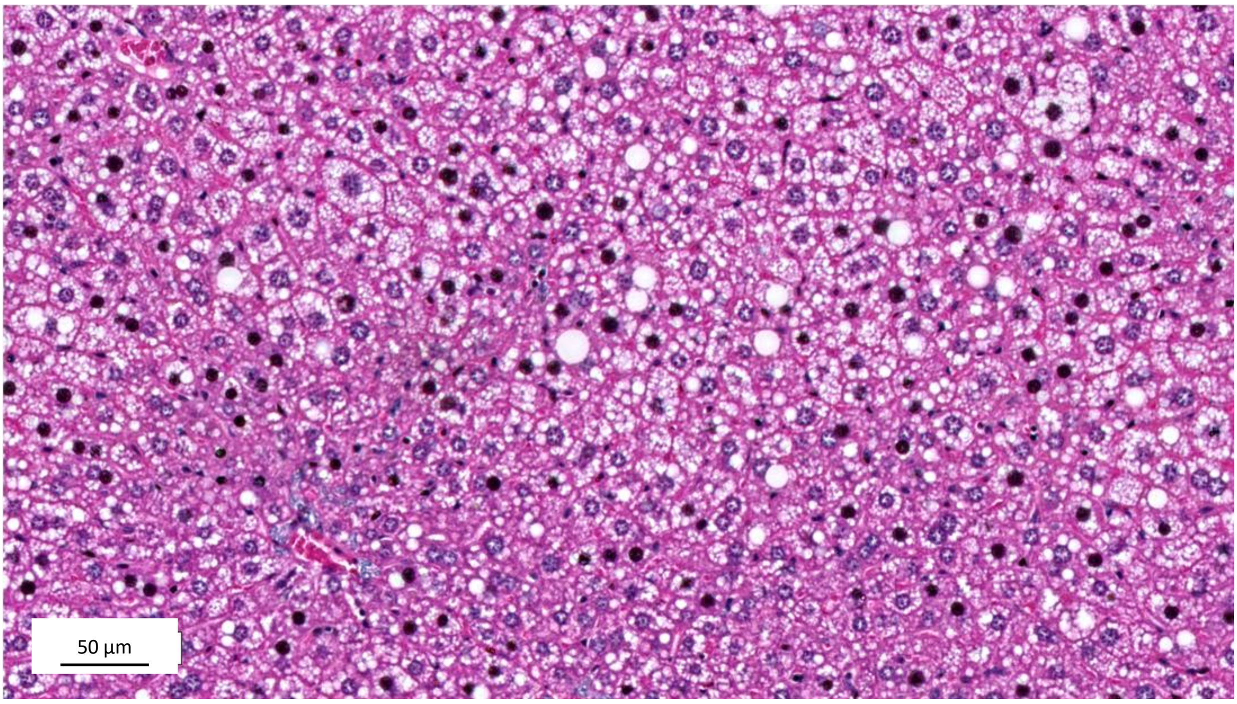

H&E Staining

CDHFD-C group

(15 mice were included)

CDHFD-C-1

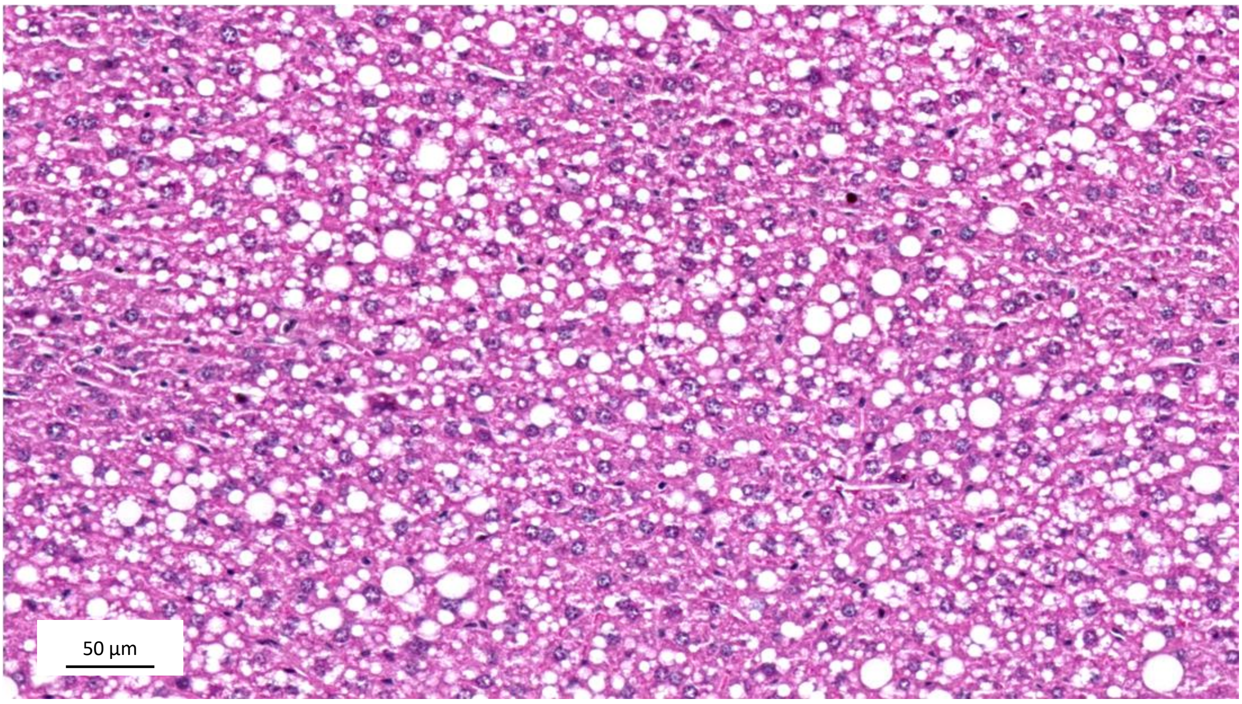

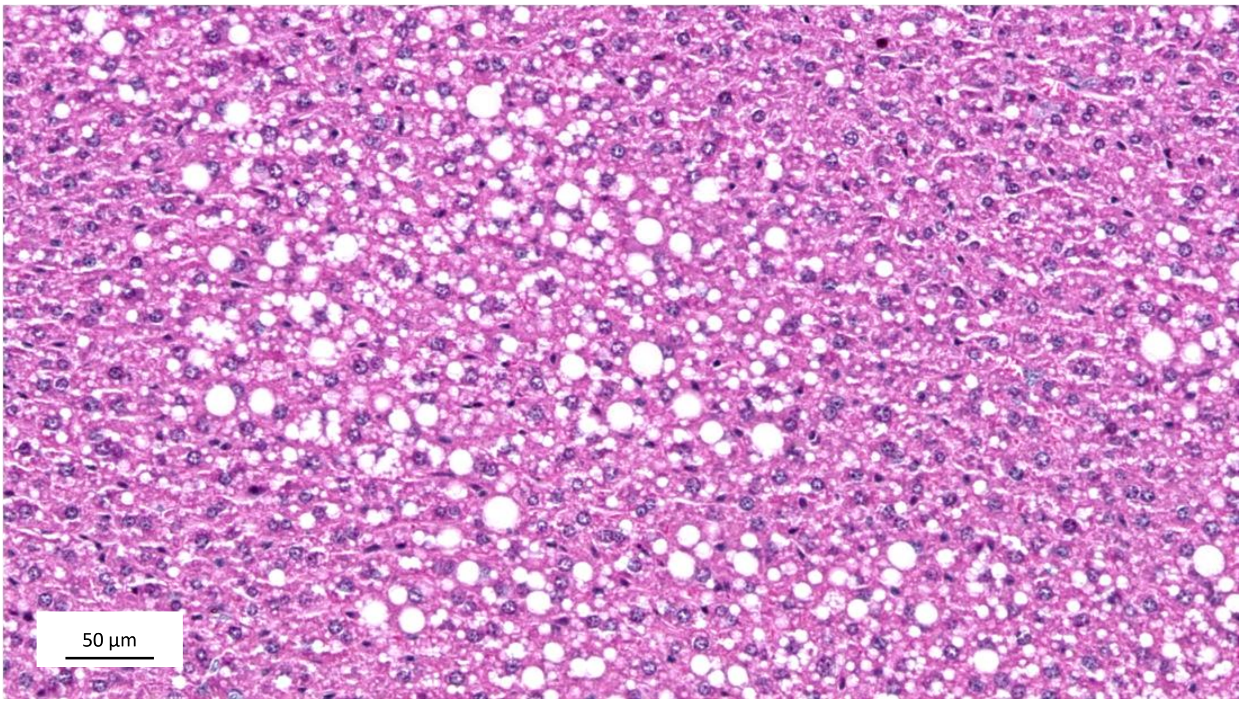

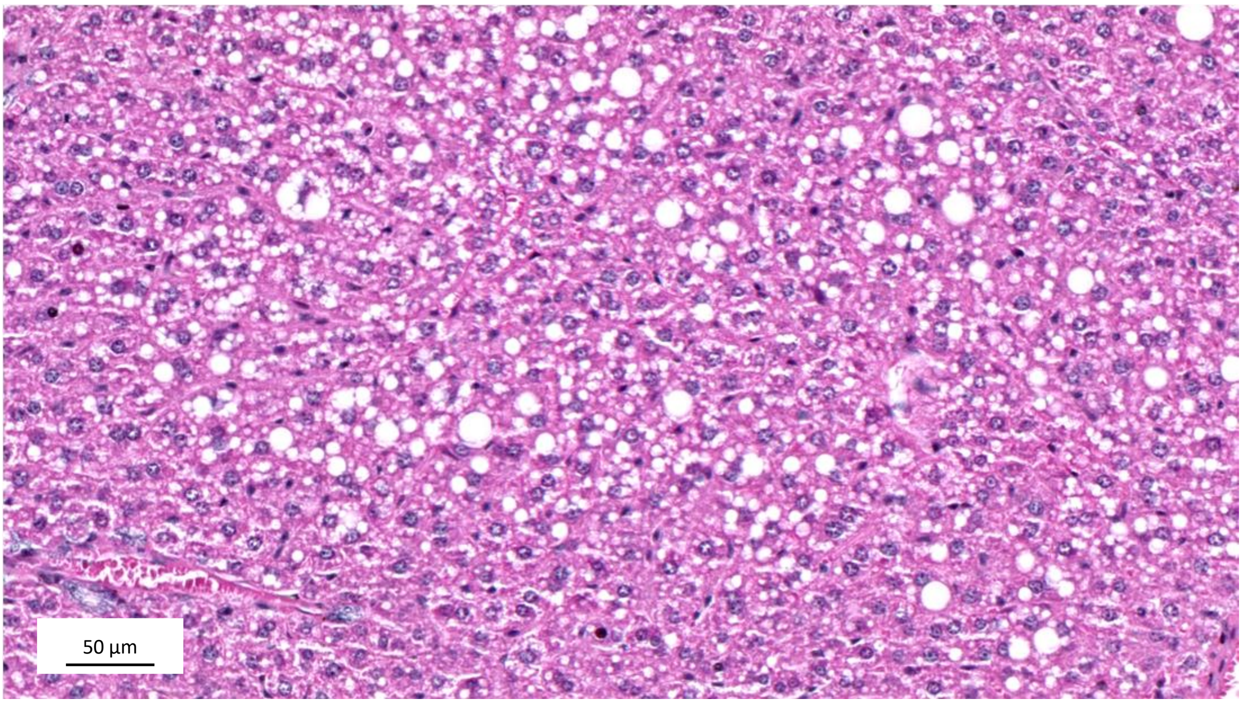

CDHFD-C-2

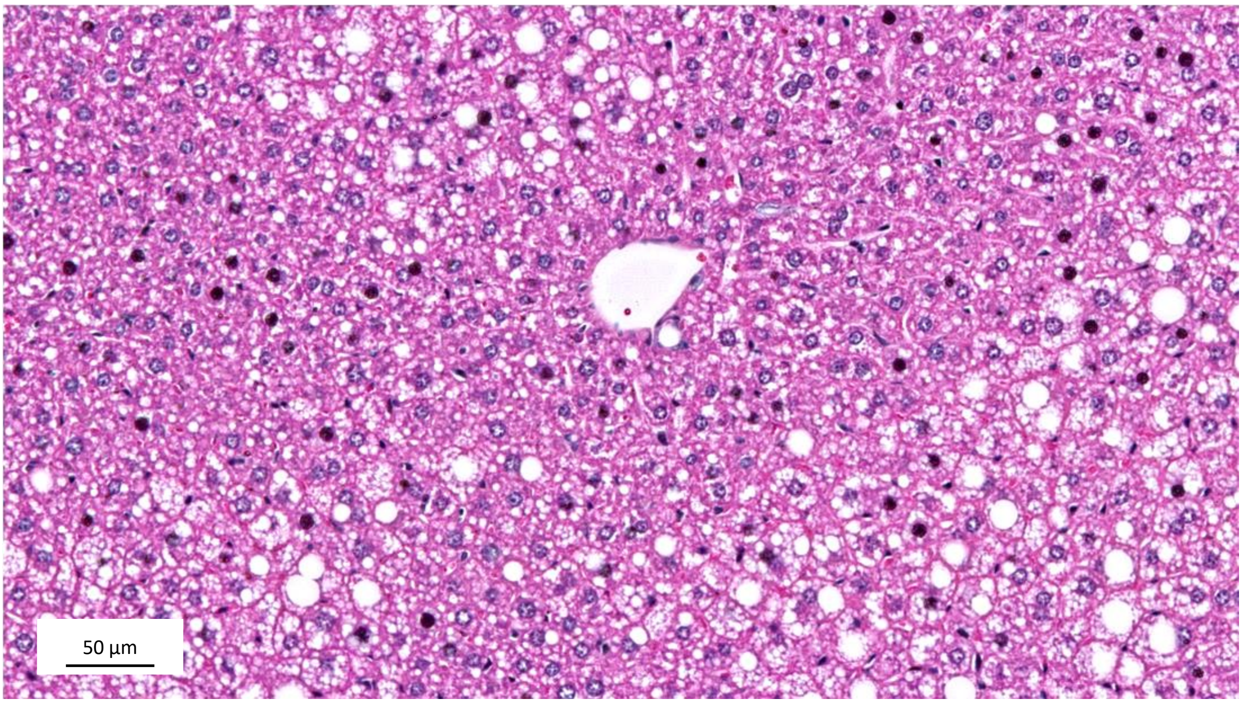

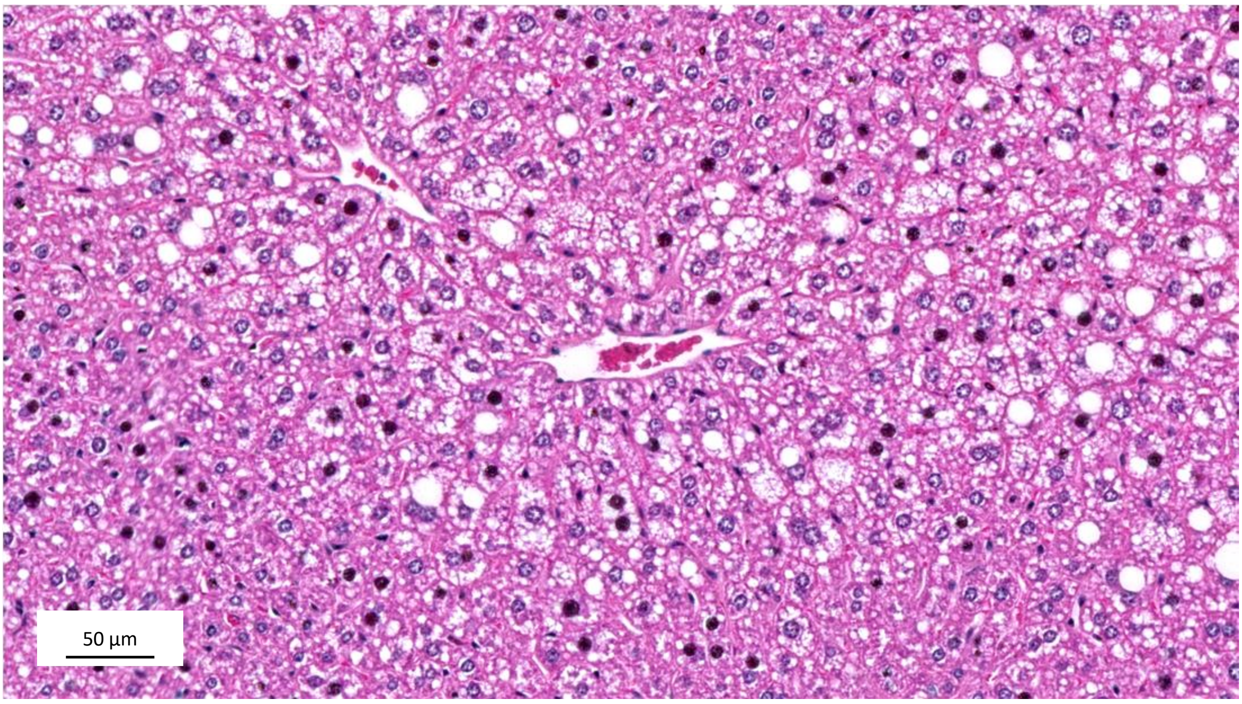

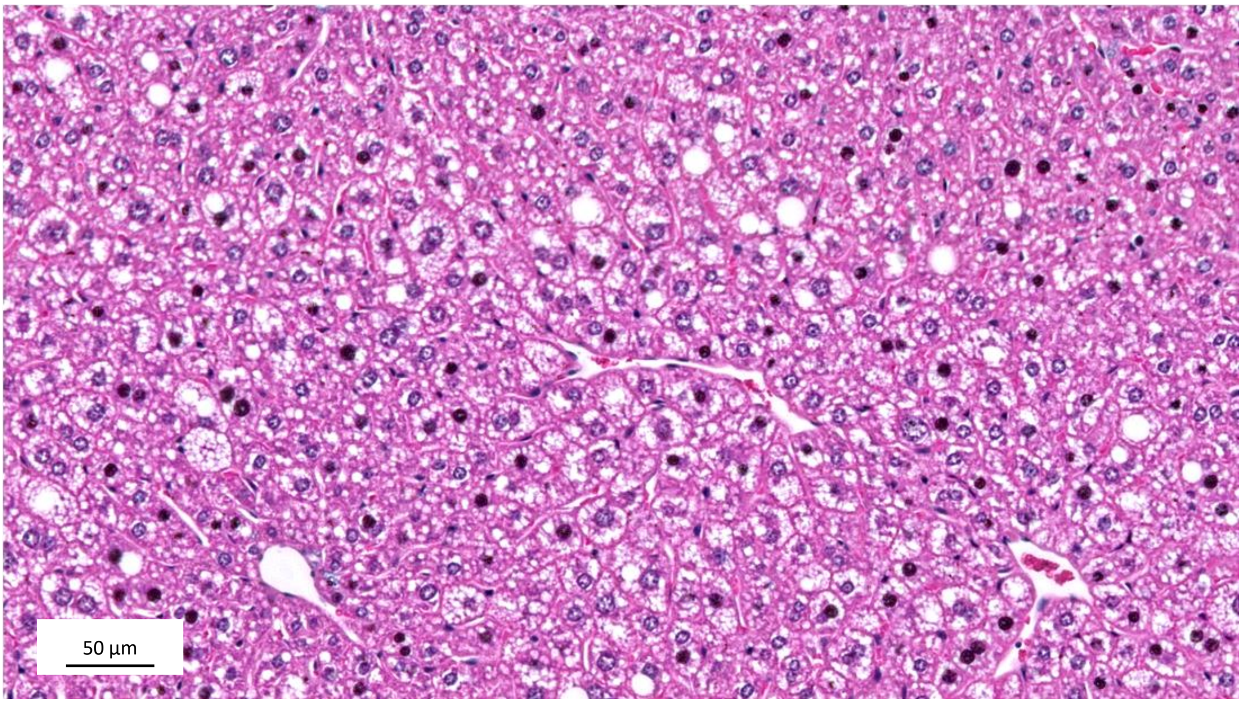

CDHFD-C-3

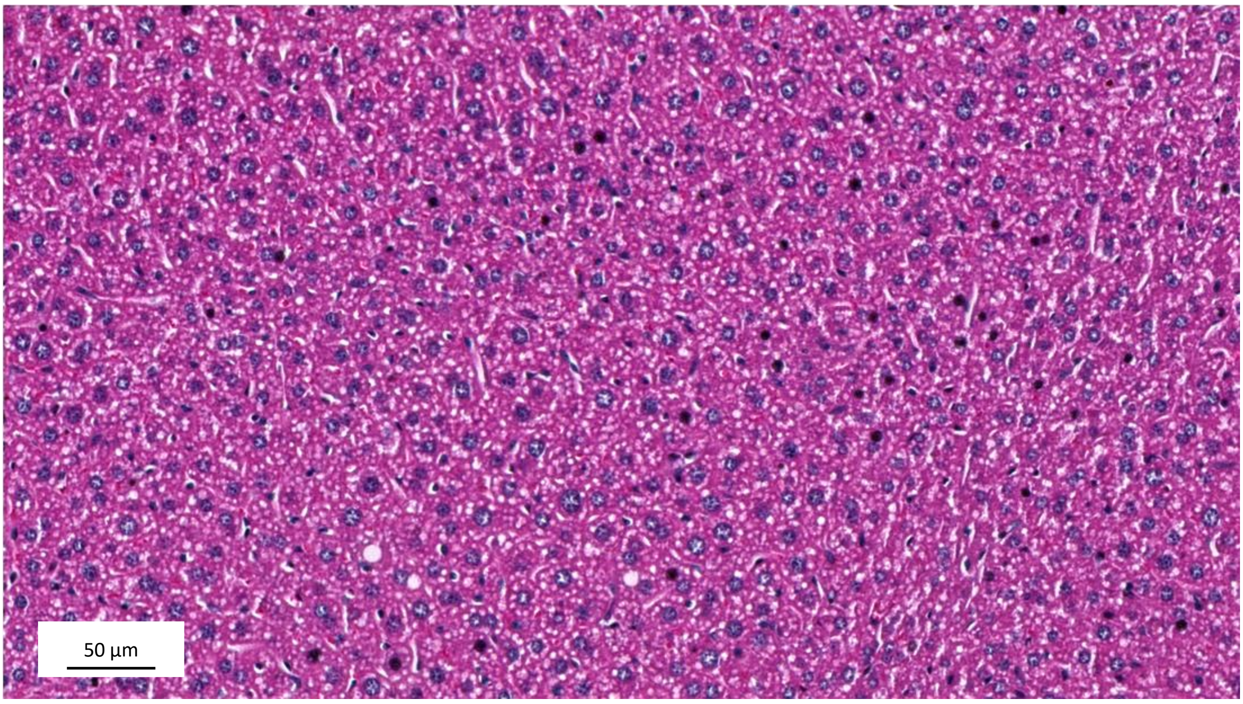

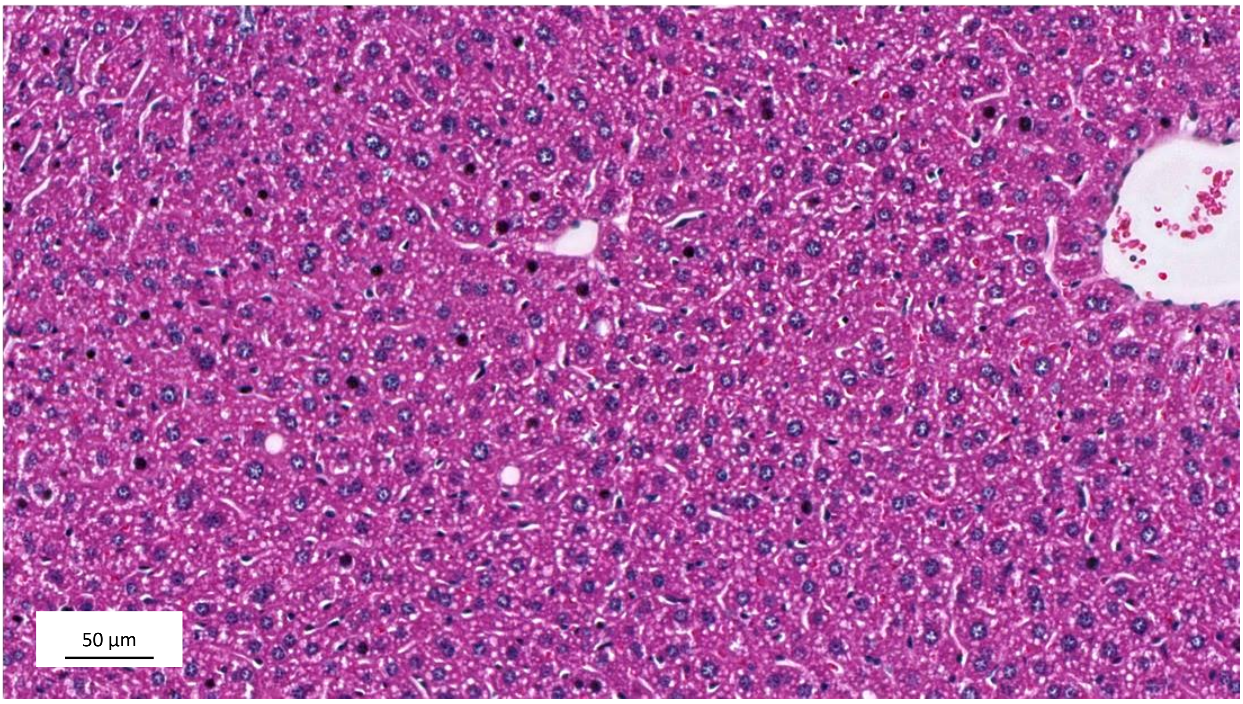

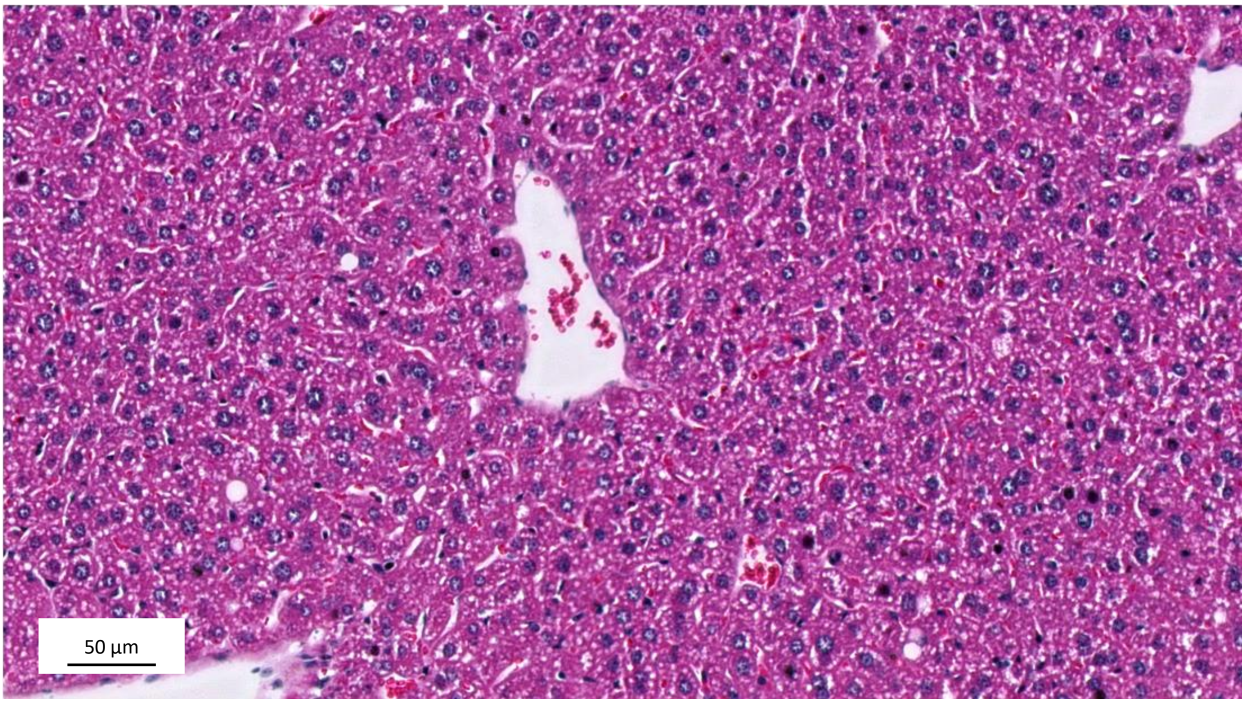

CDHFD-C-4

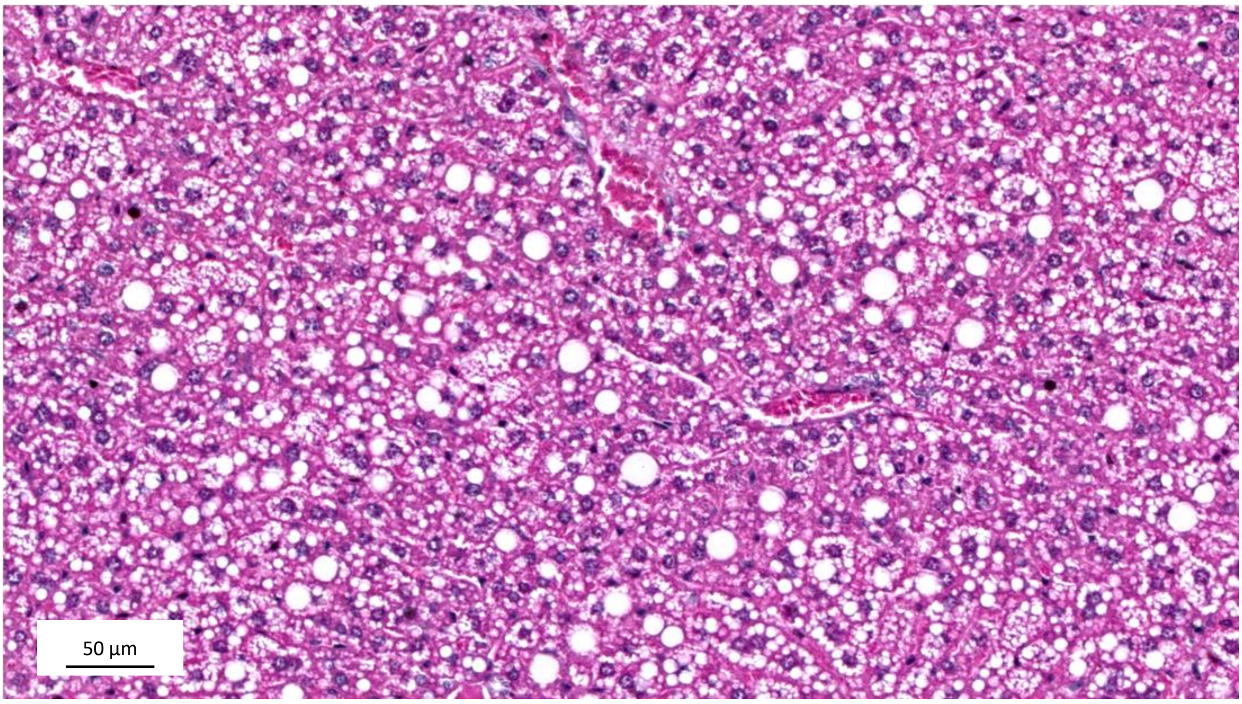

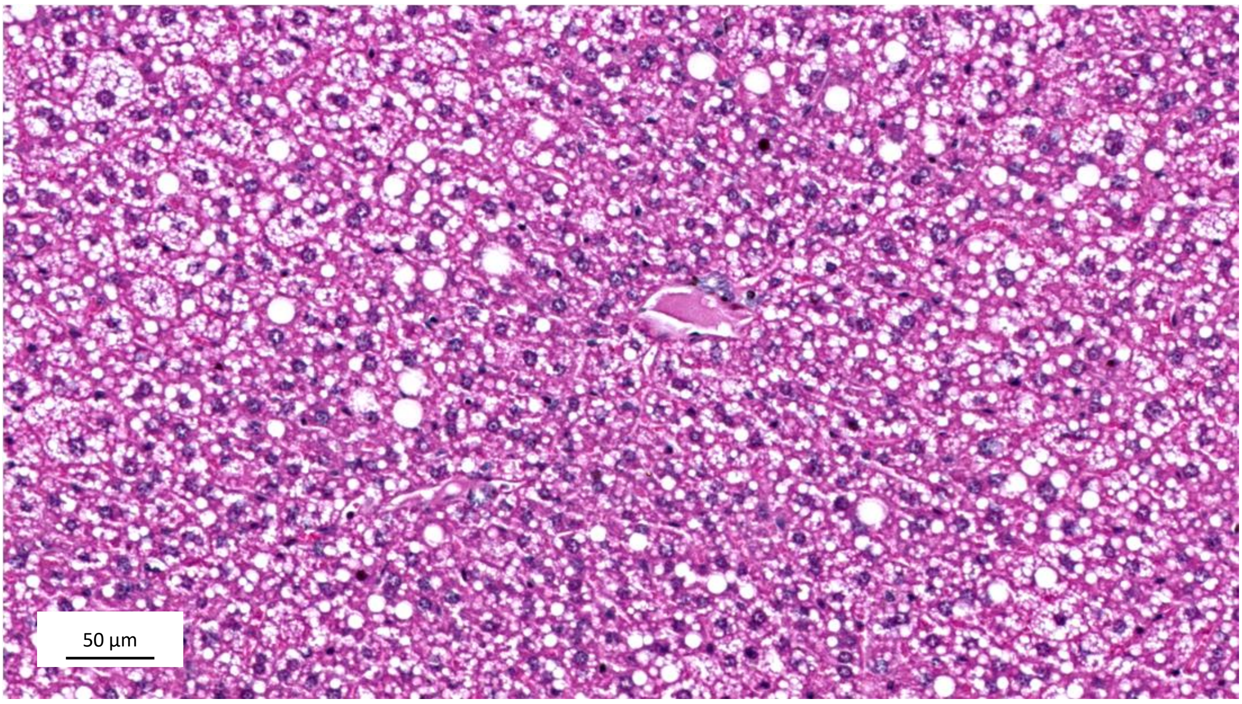

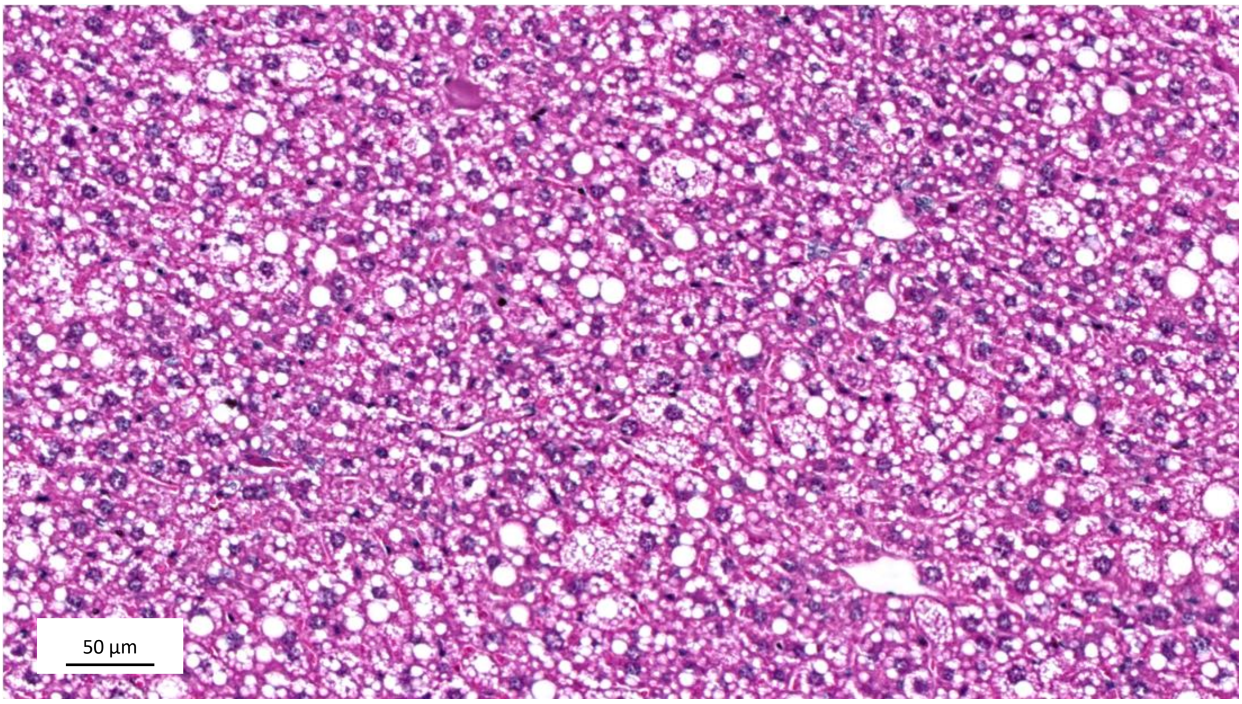

CDHFD-C-5

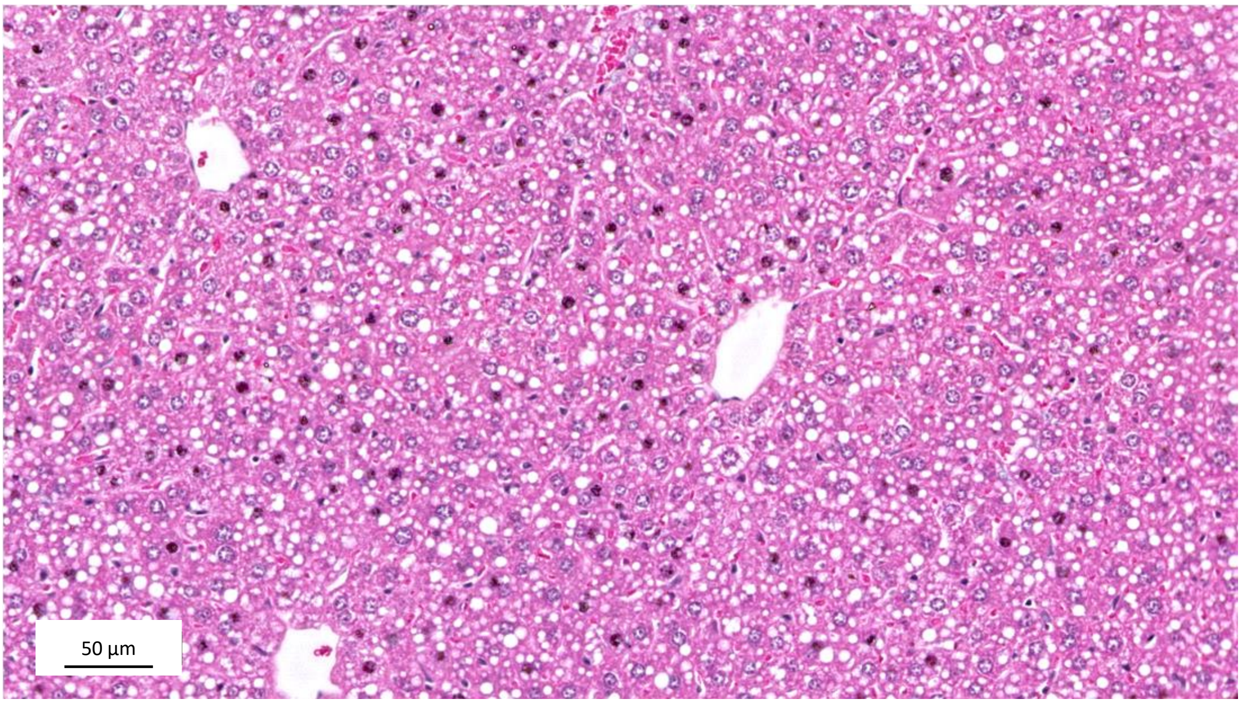

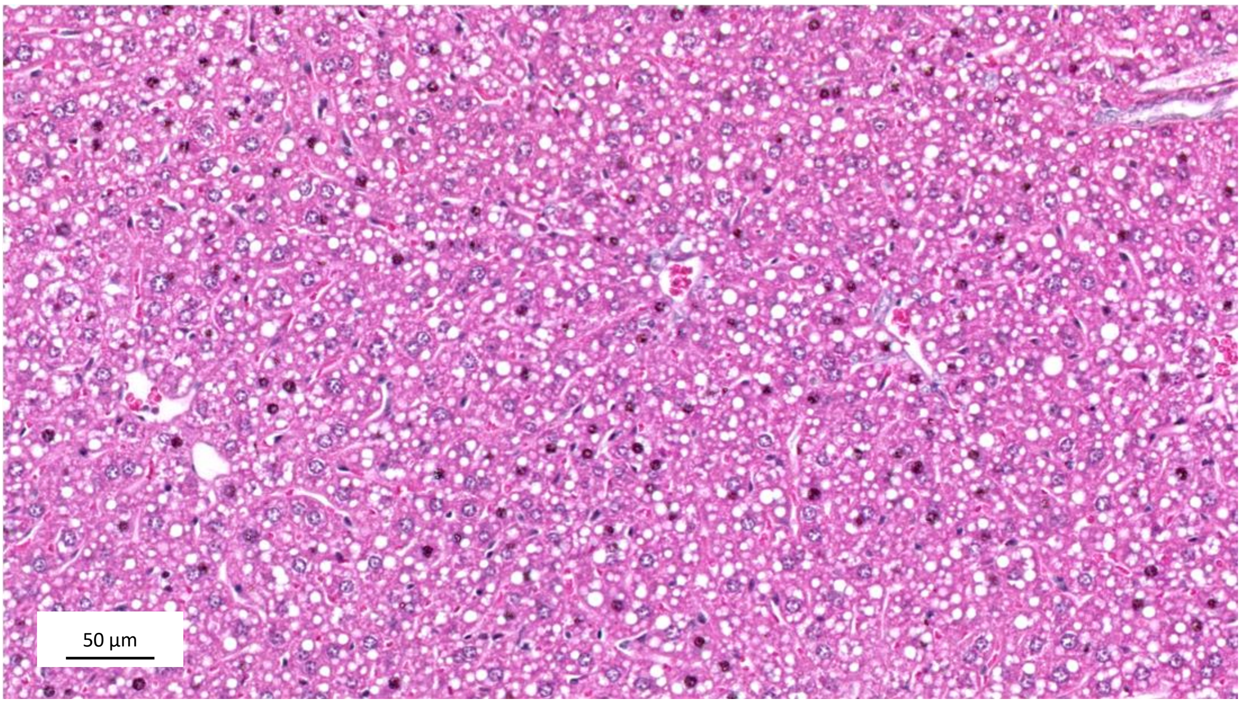

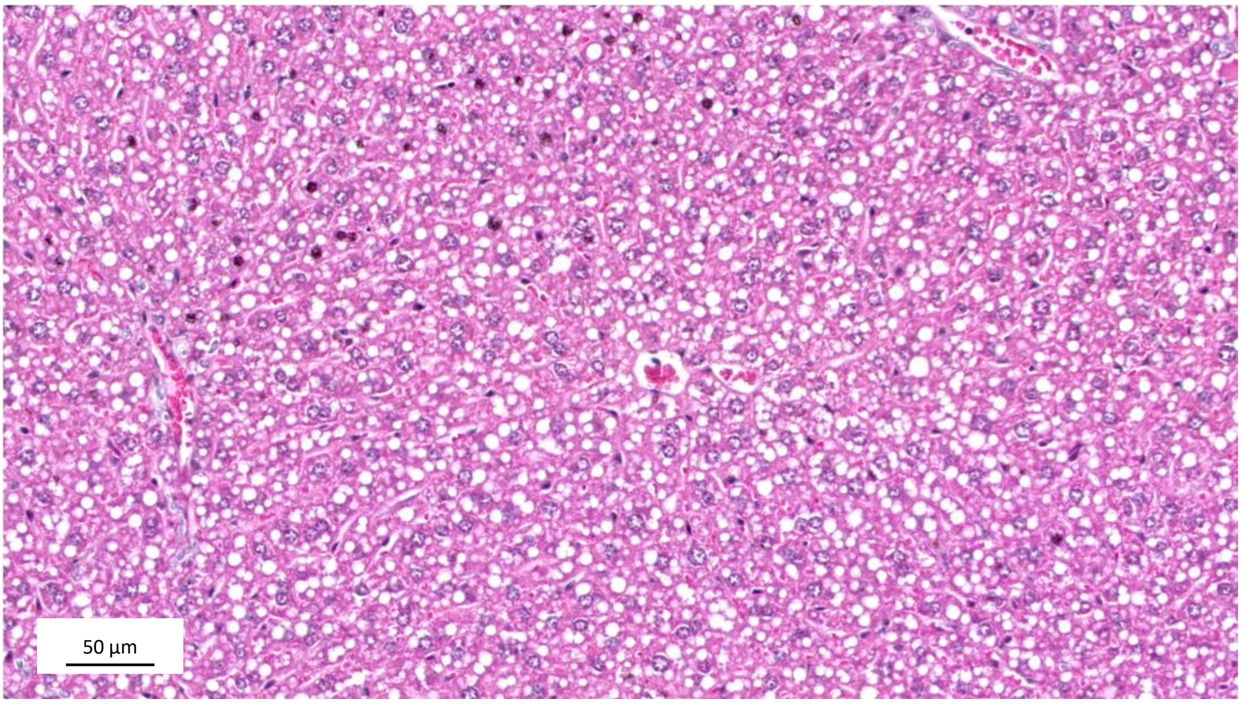

CDHFD-C-6

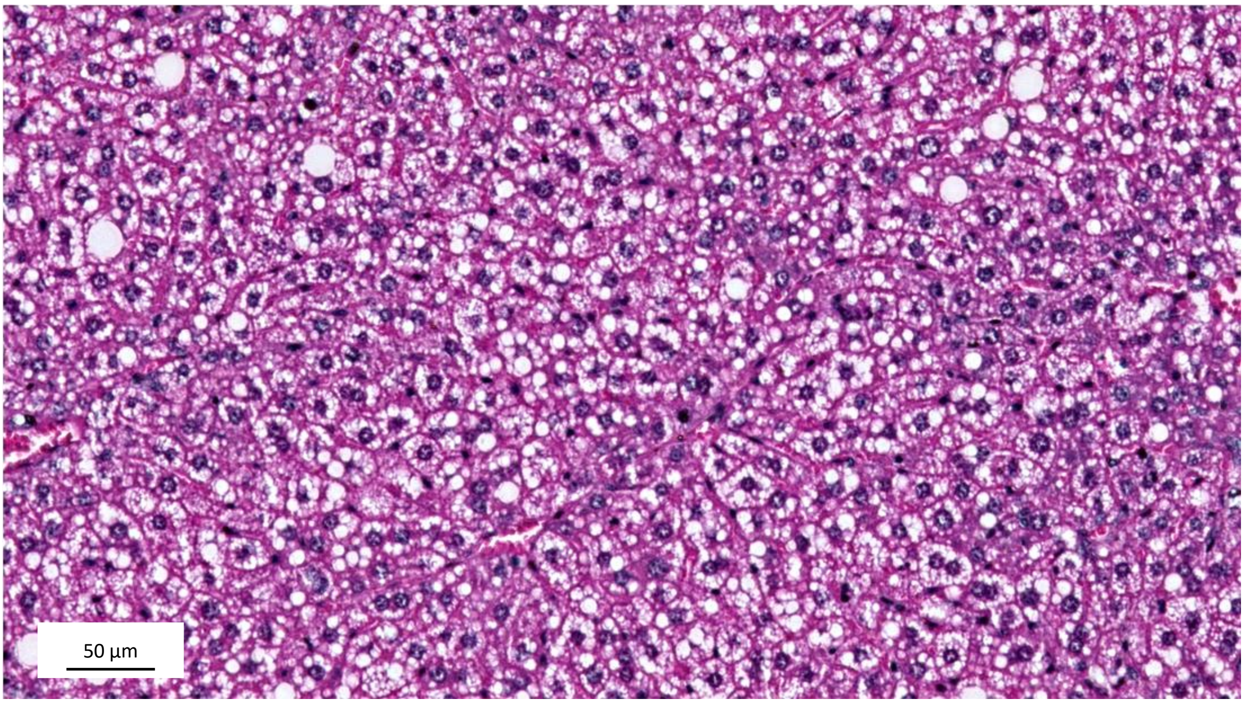

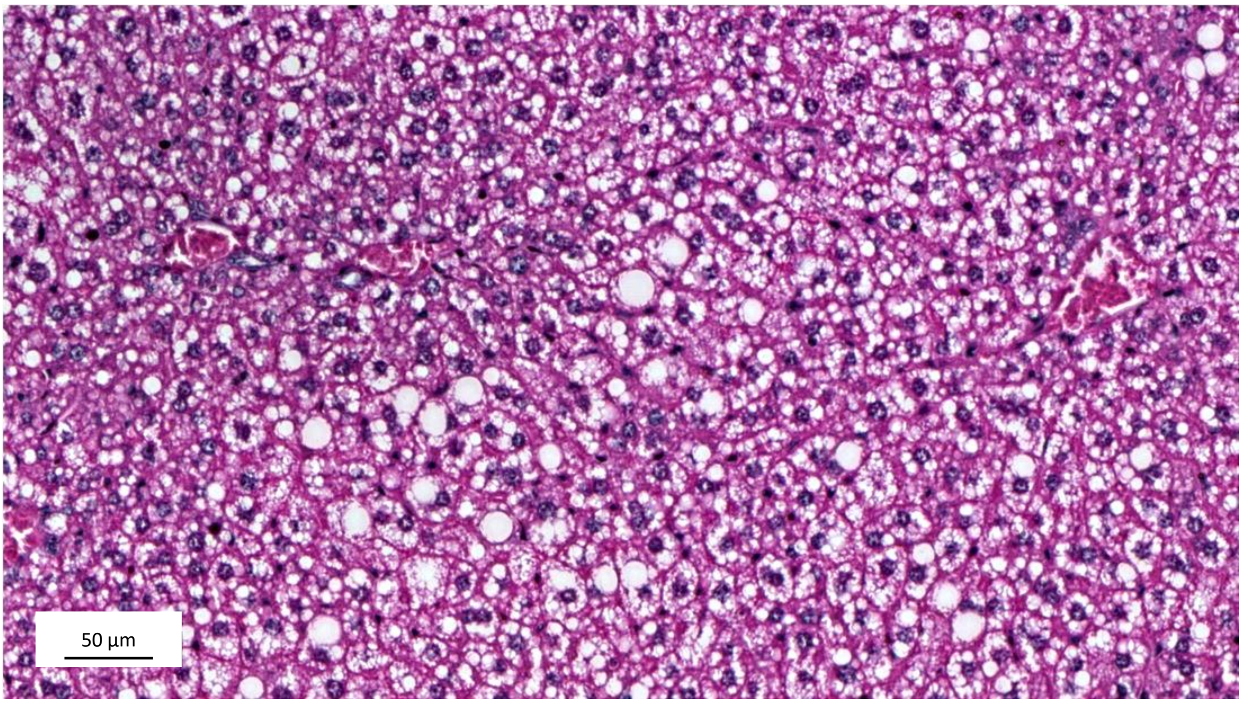

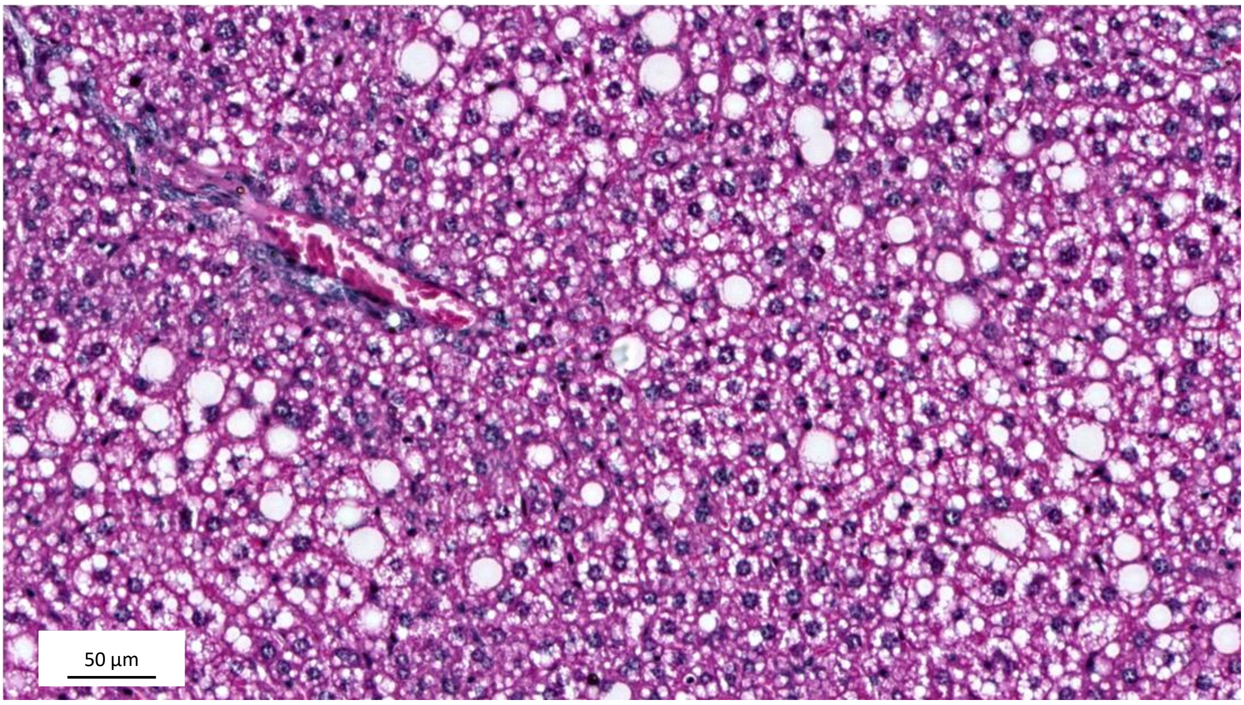

CDHFD-C-7

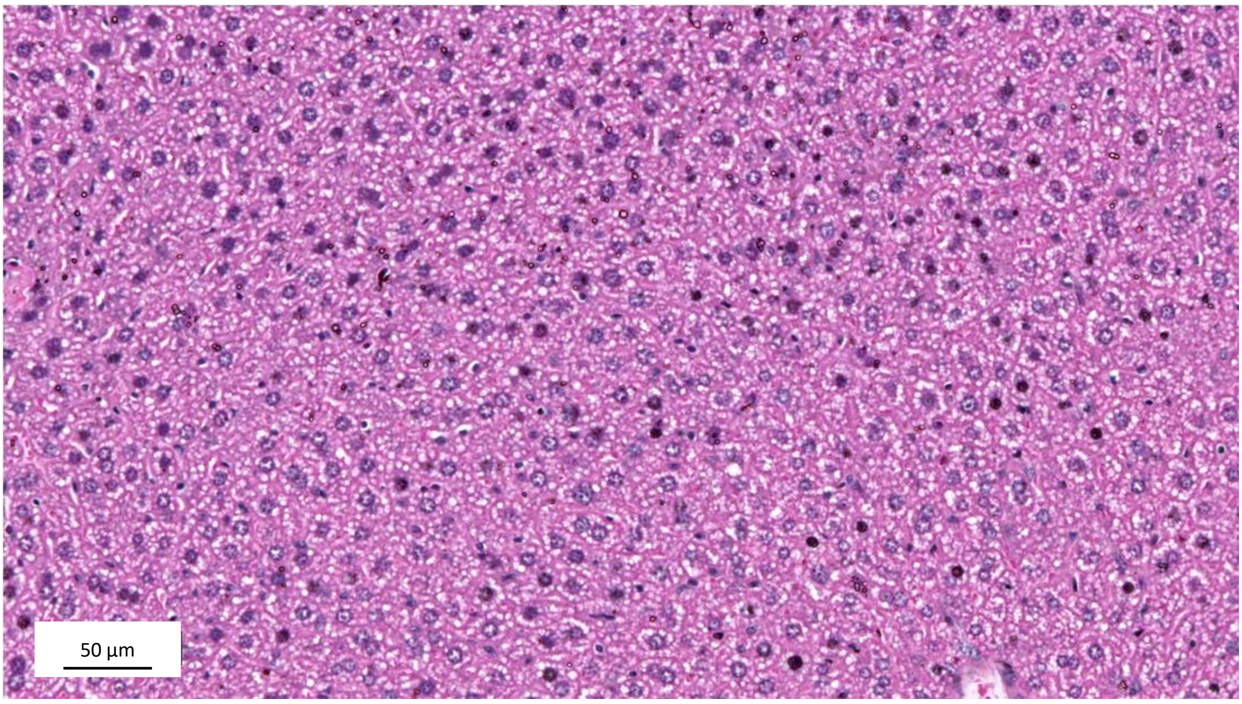

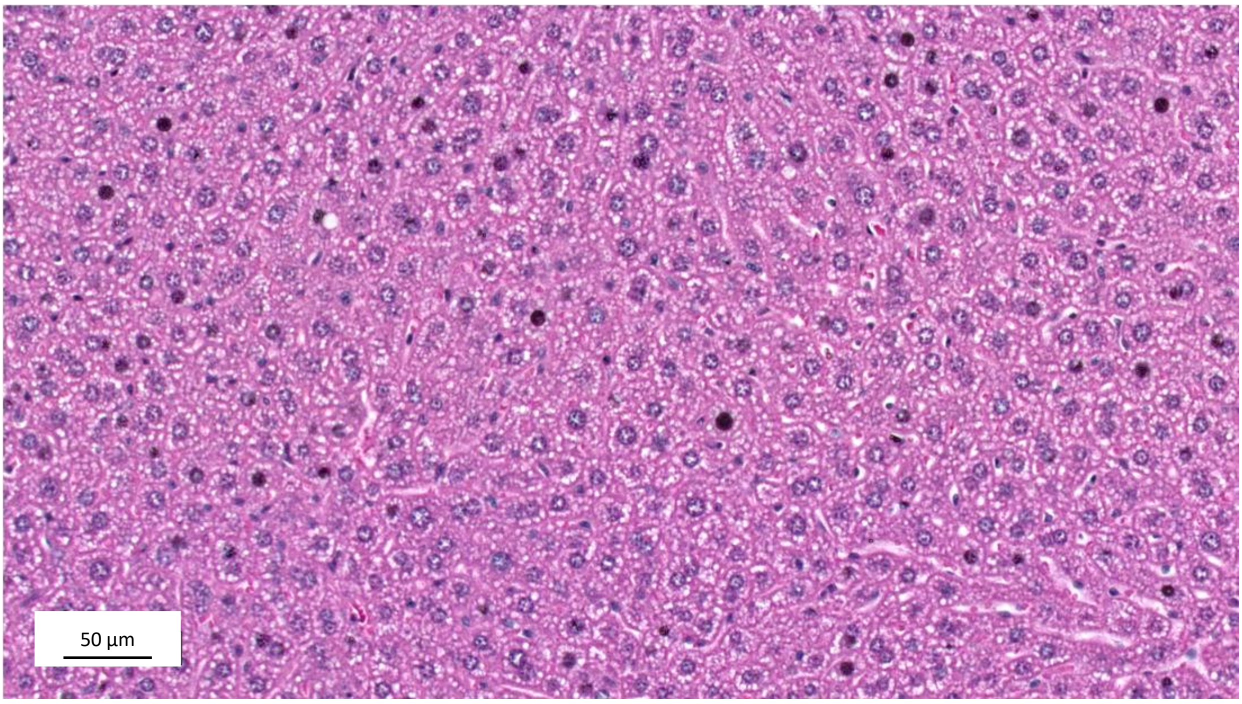

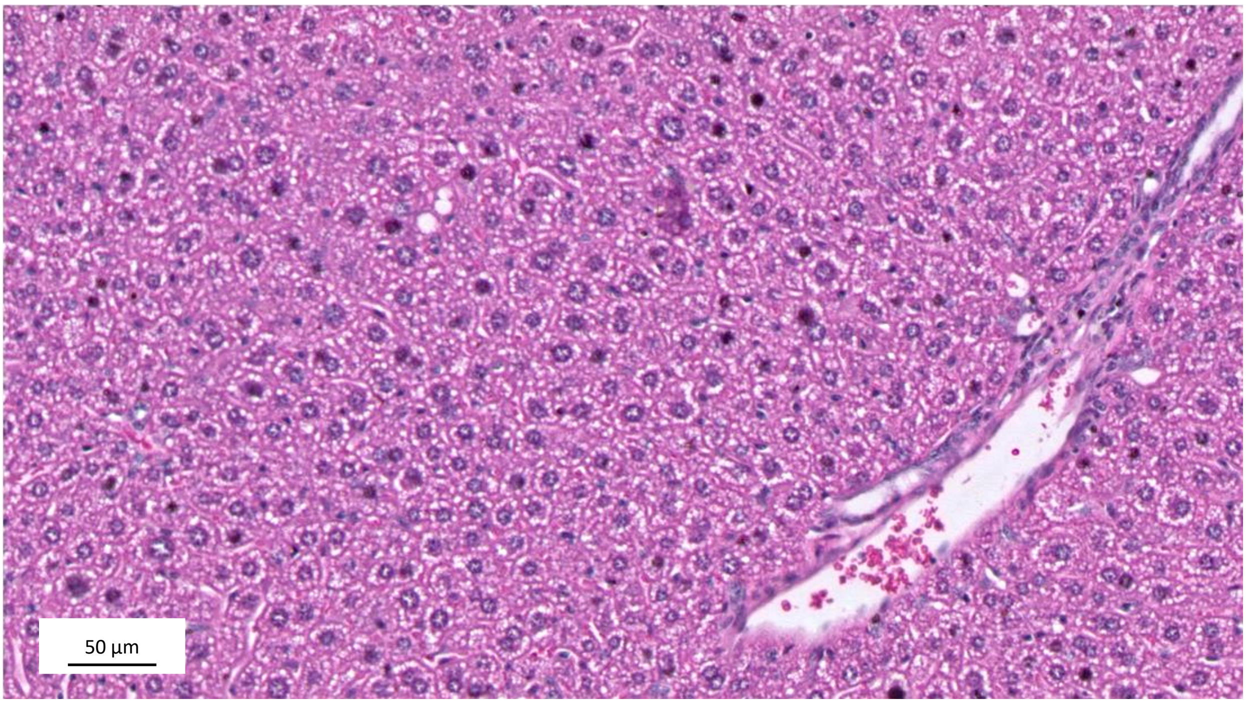

CDHFD-C-8

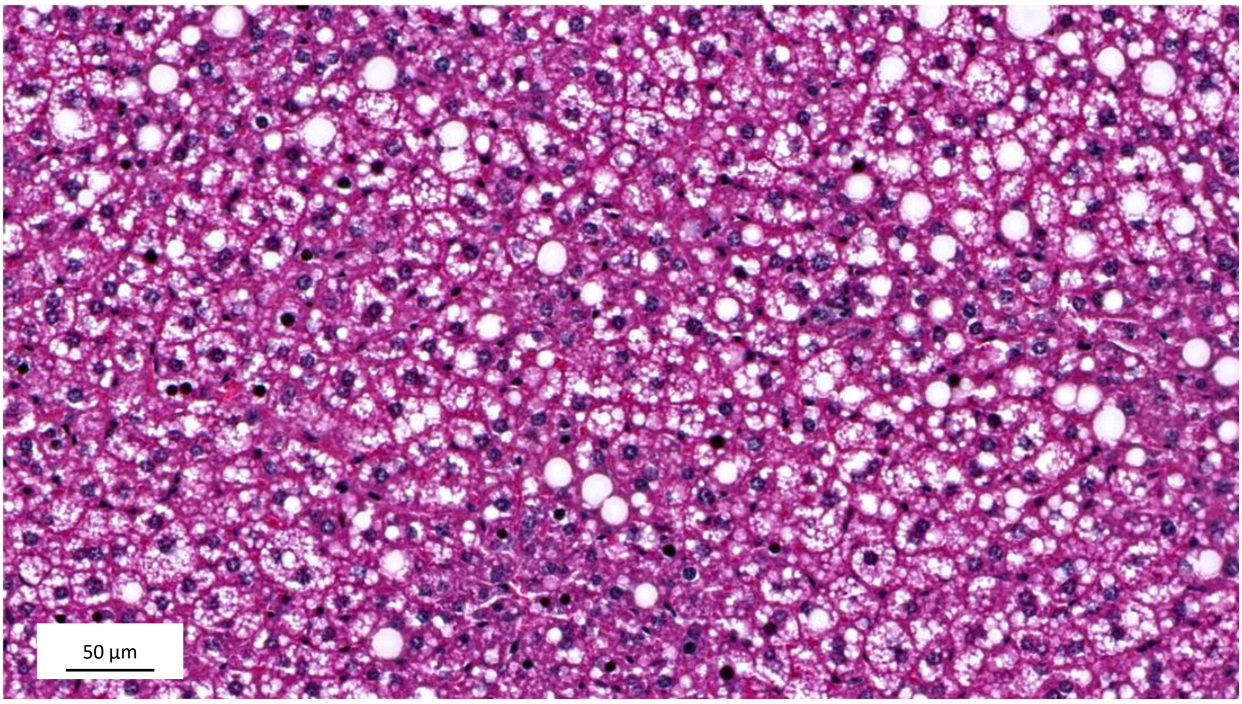

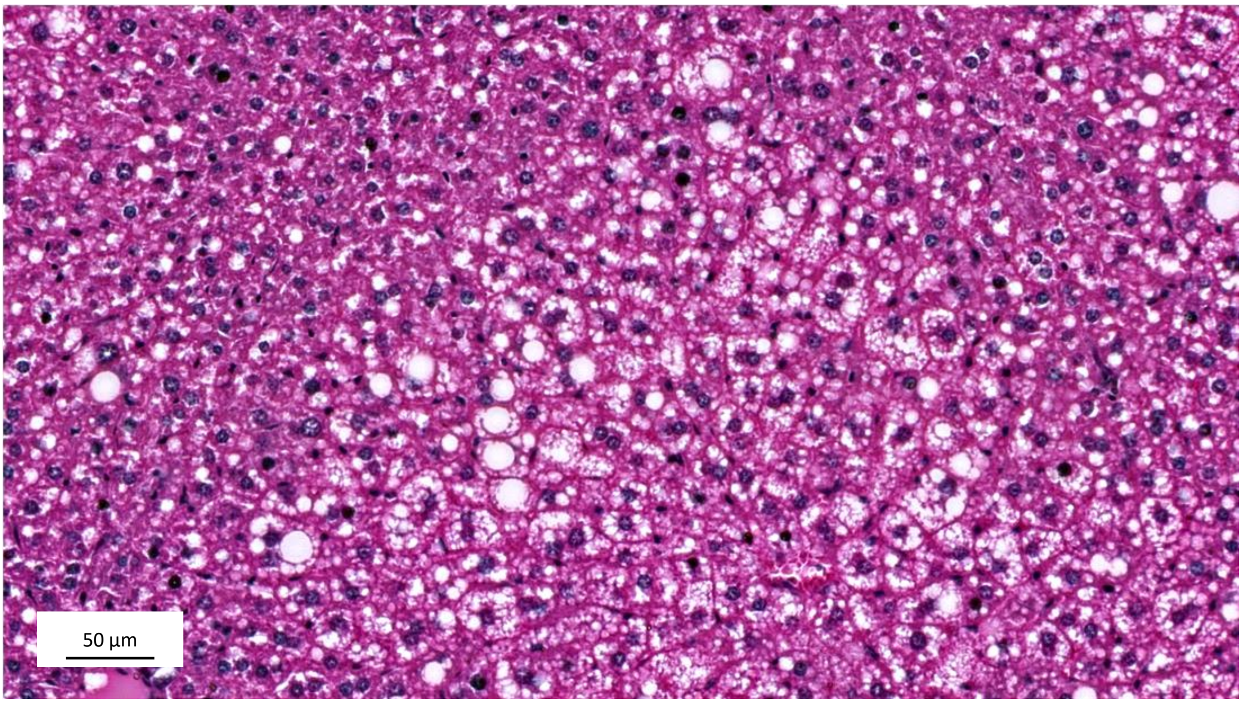

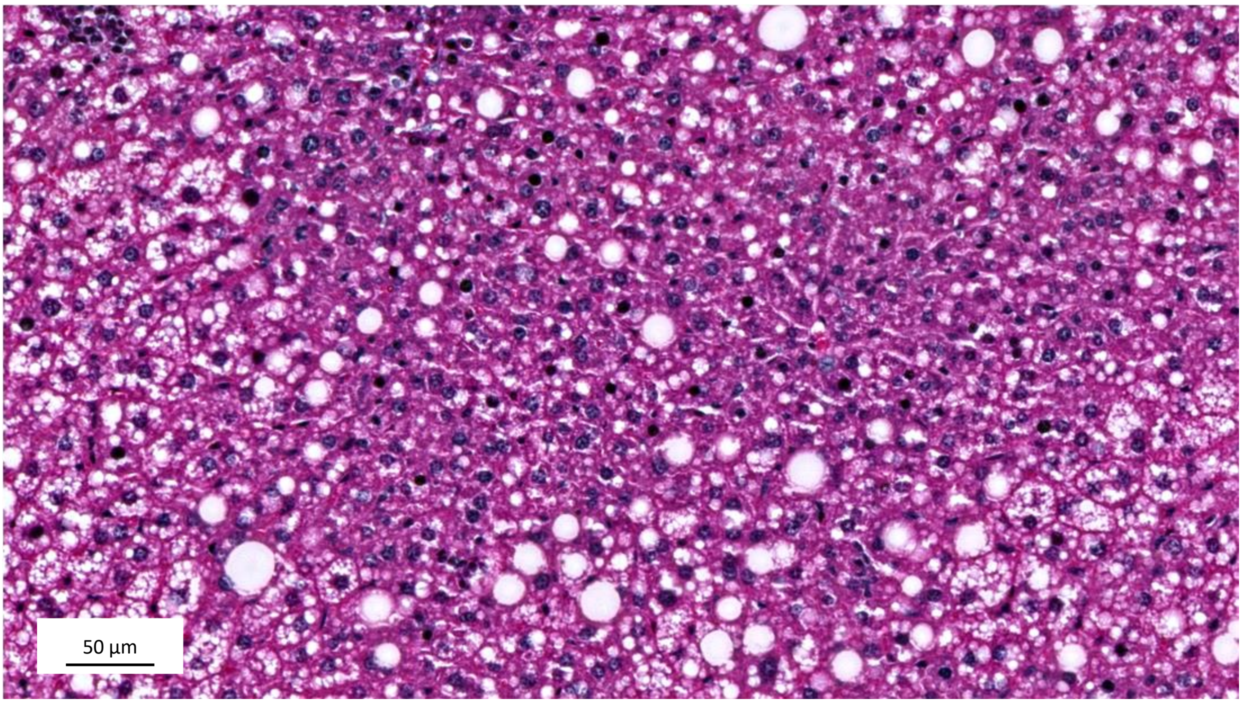

CDHFD-C-9

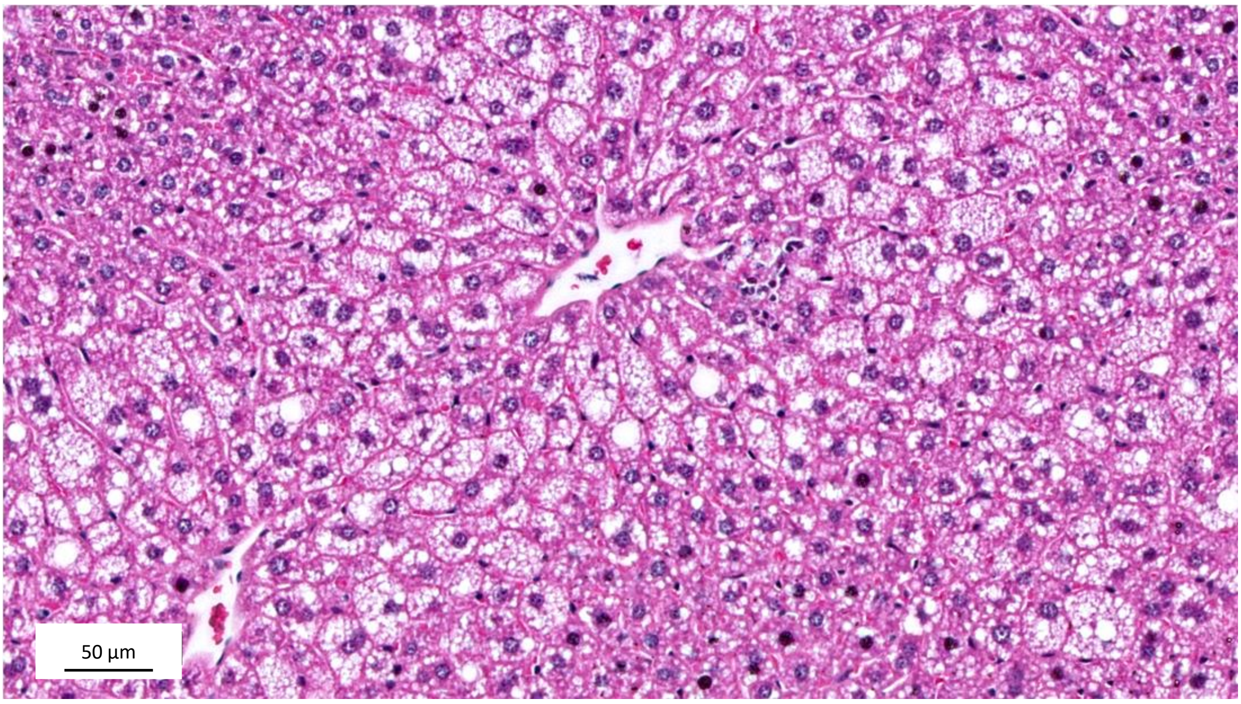

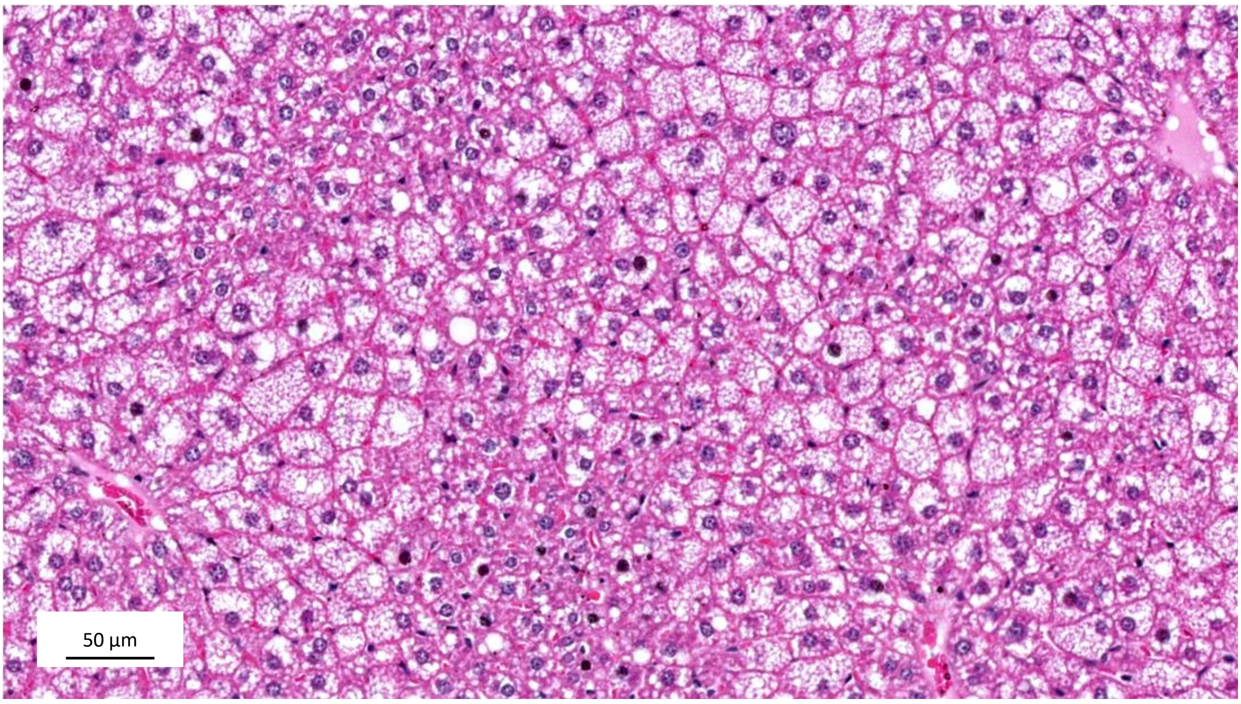

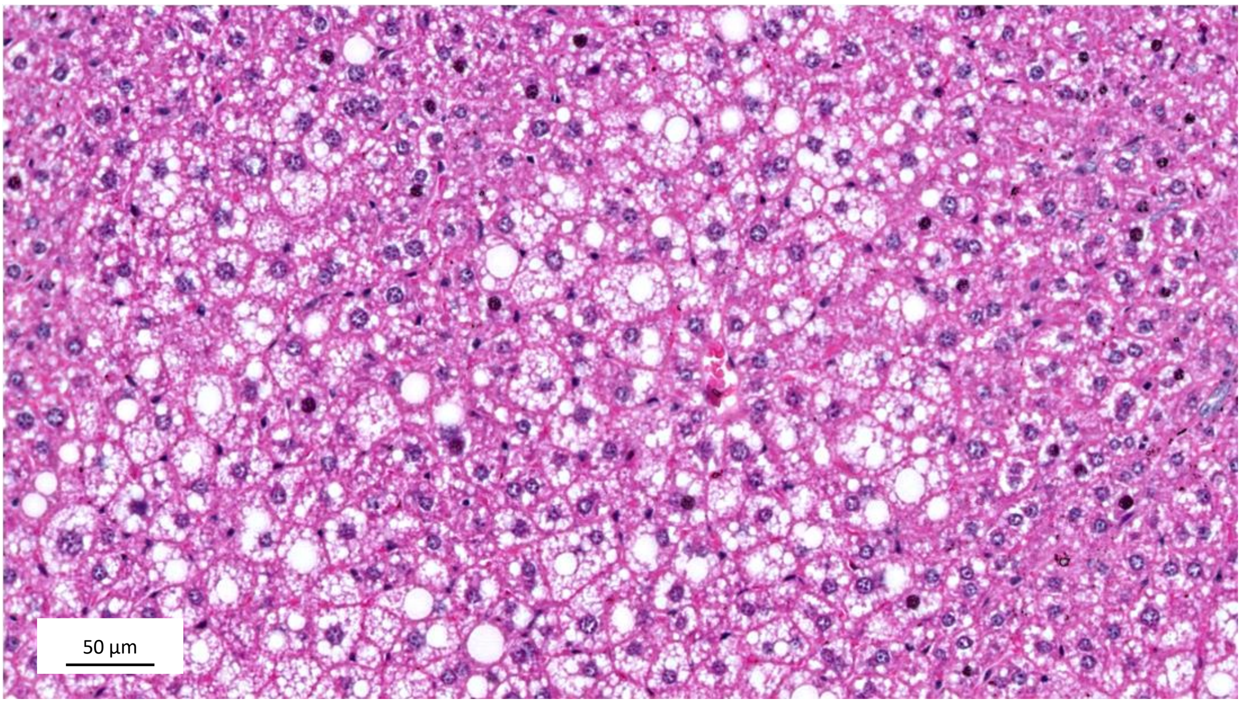

CDHFD-C-10

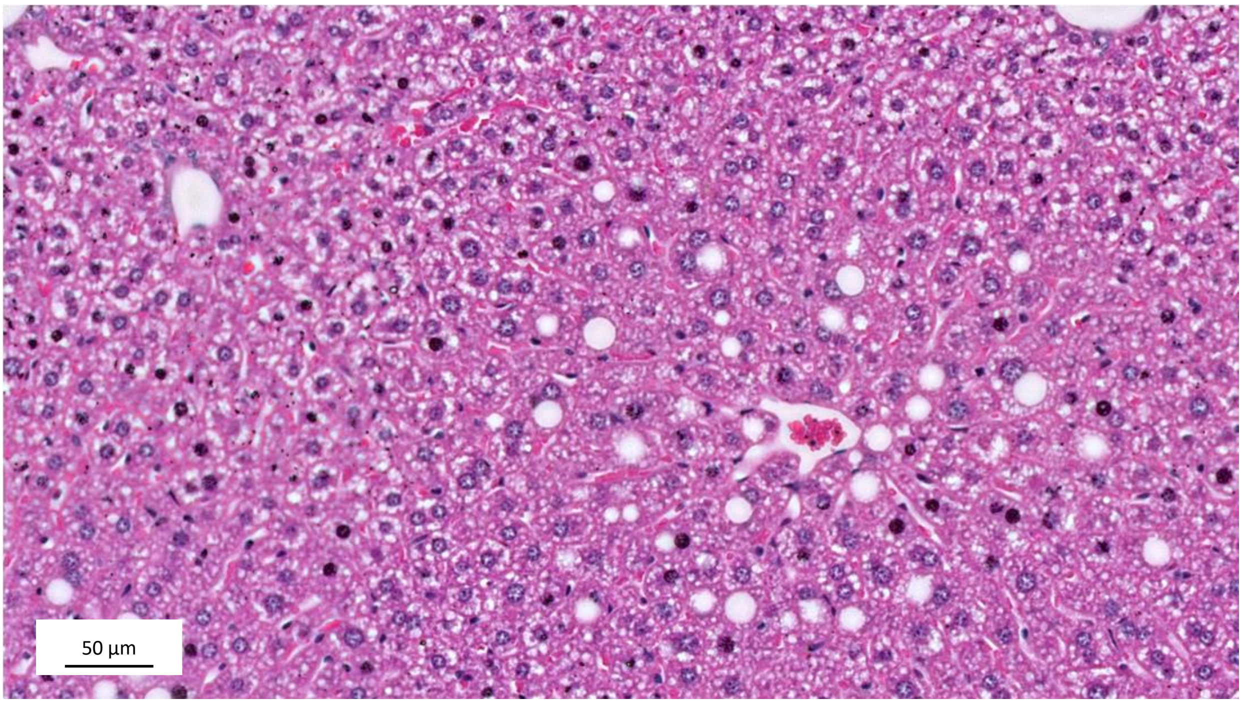

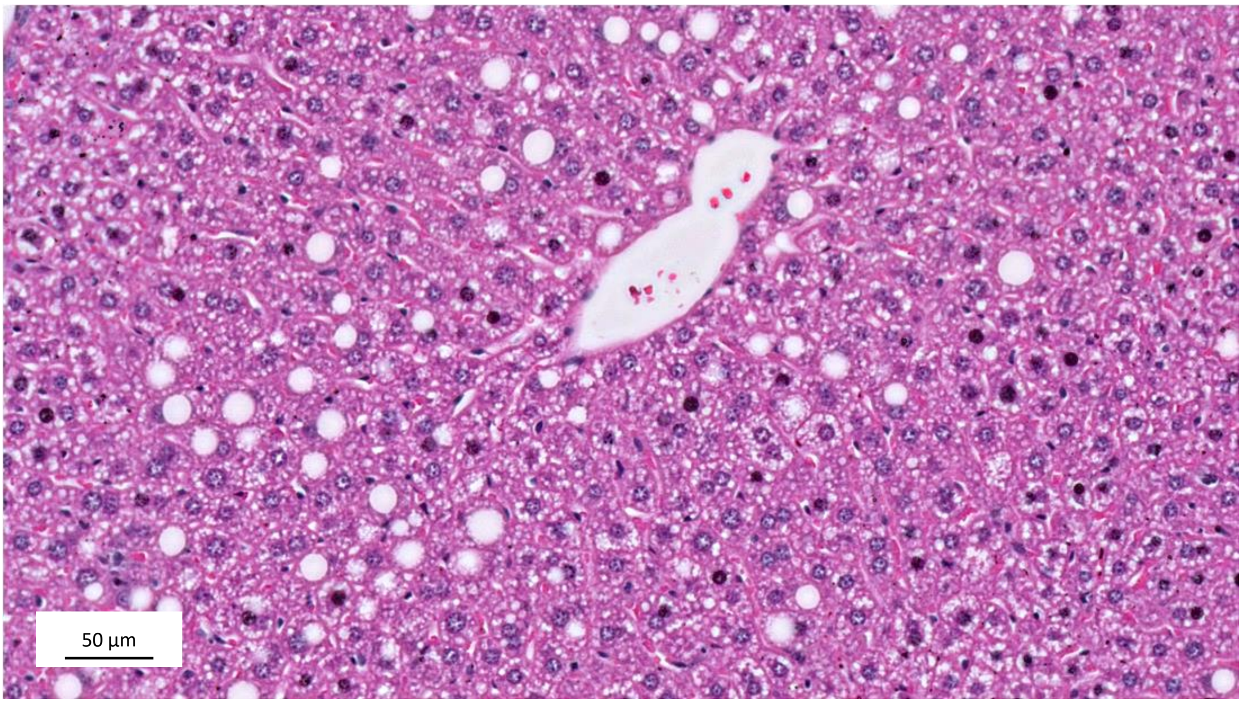

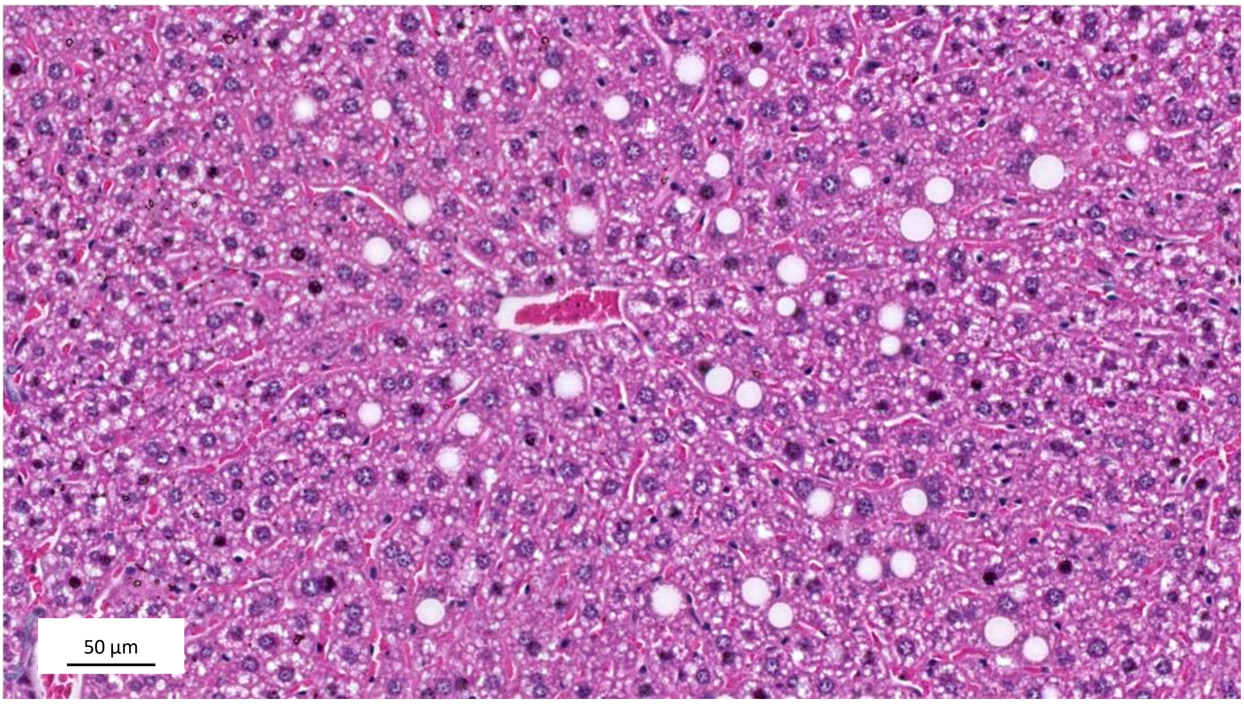

CDHFD-C-11

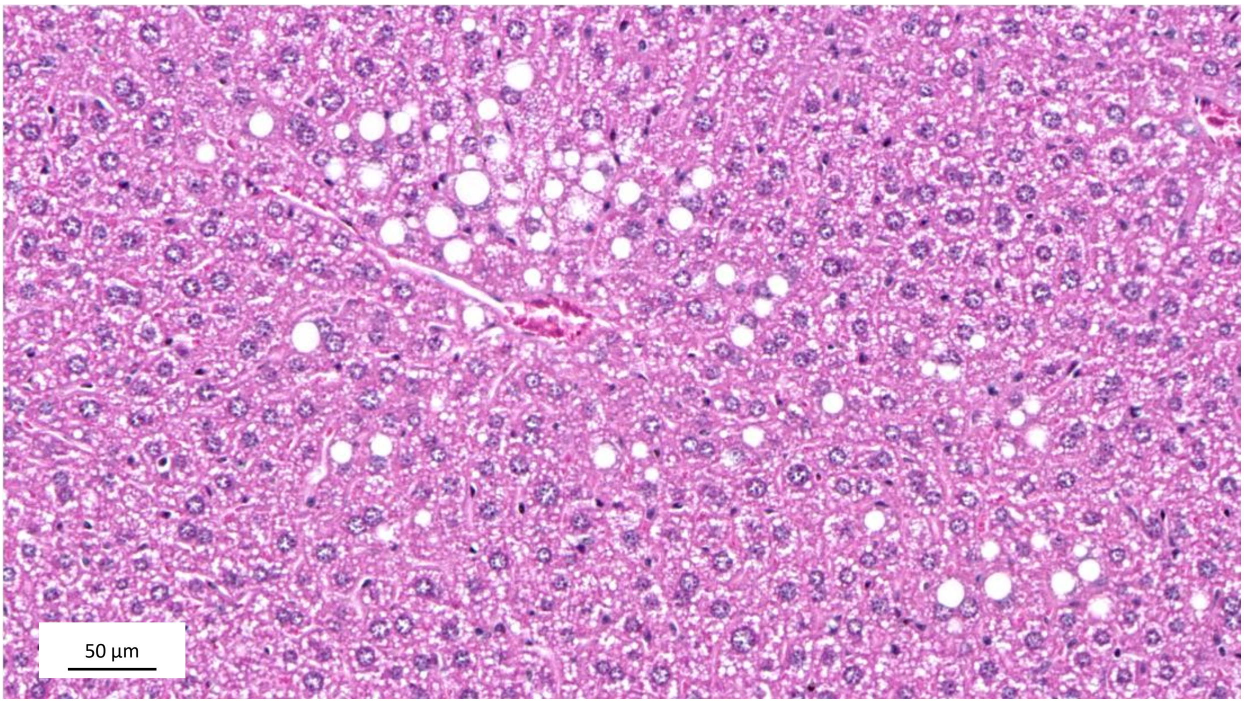

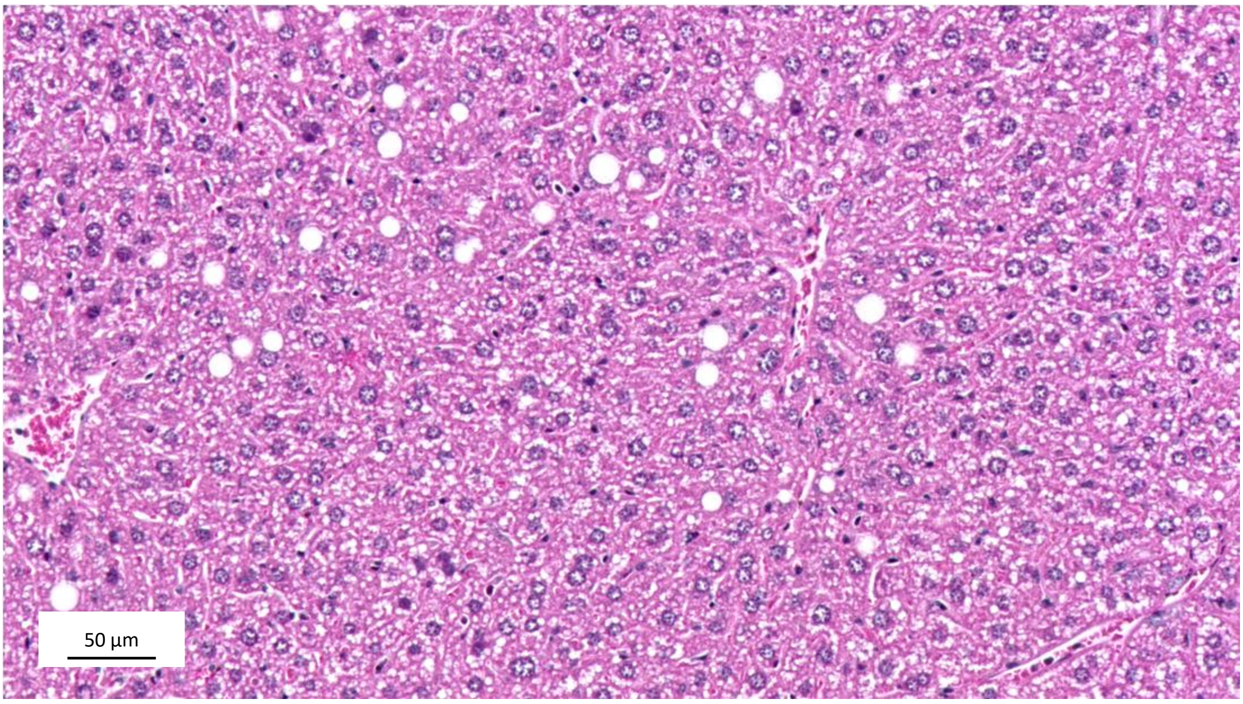

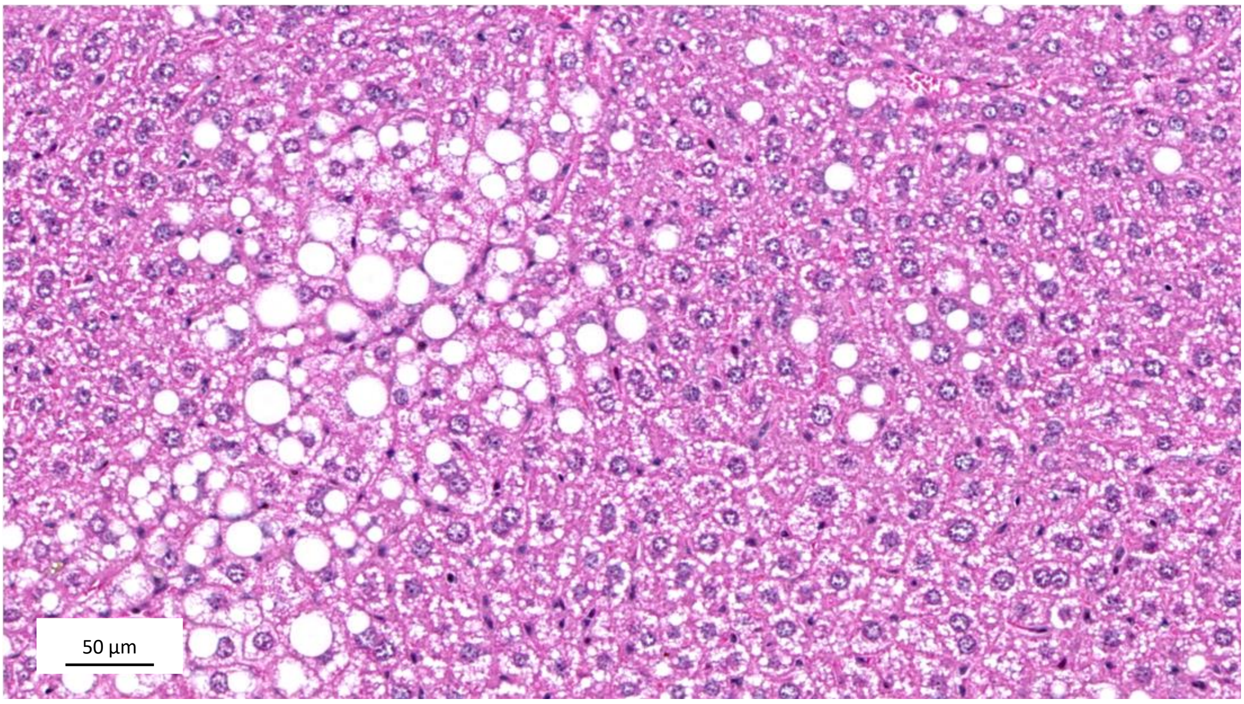

CDHFD-C-12

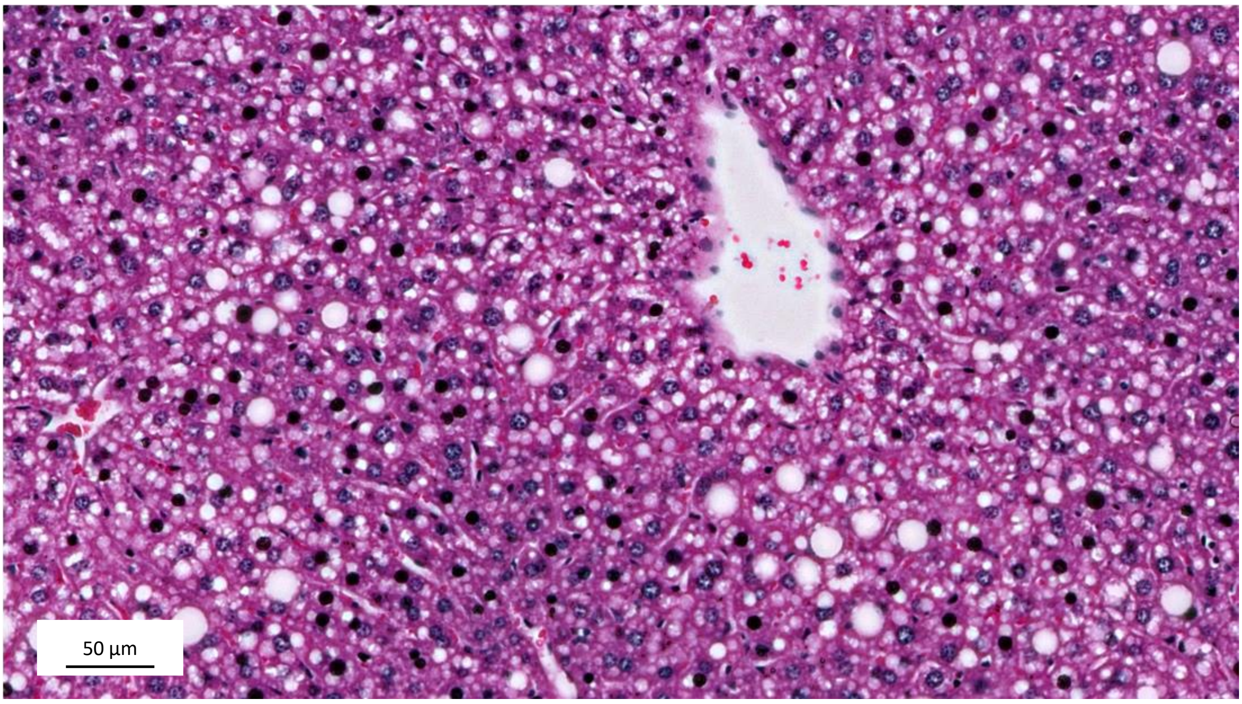

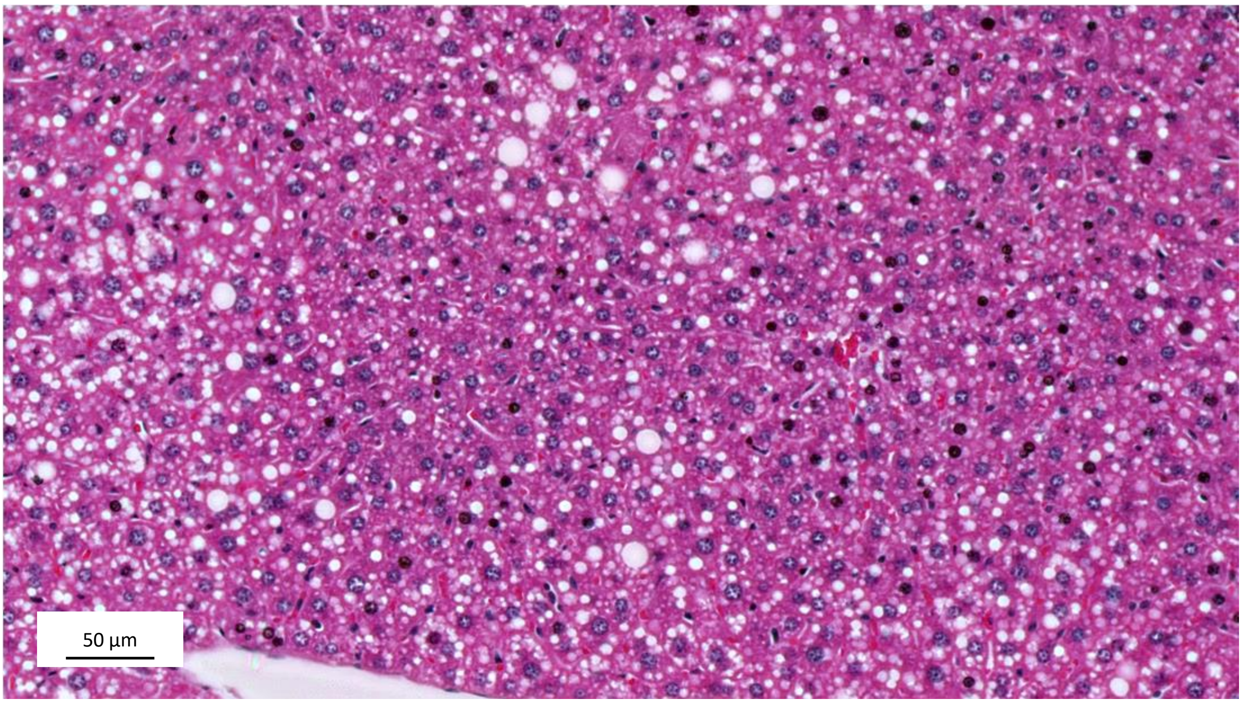

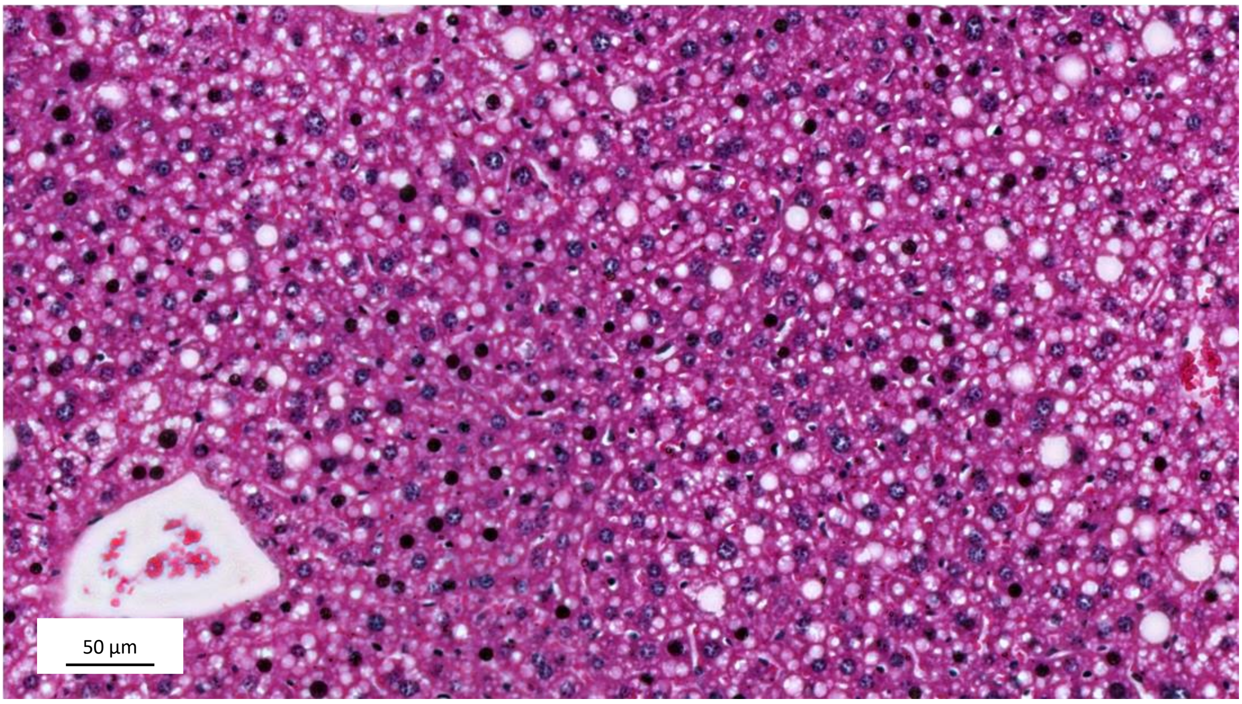

CDHFD-C-13

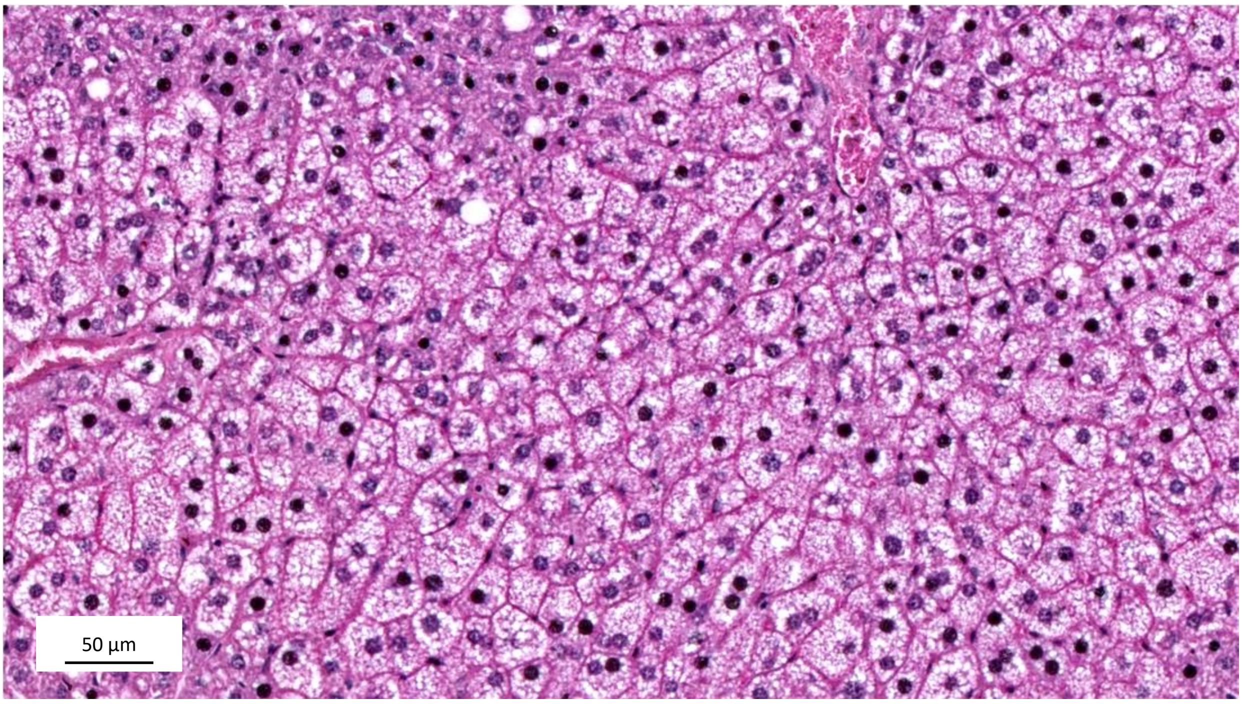

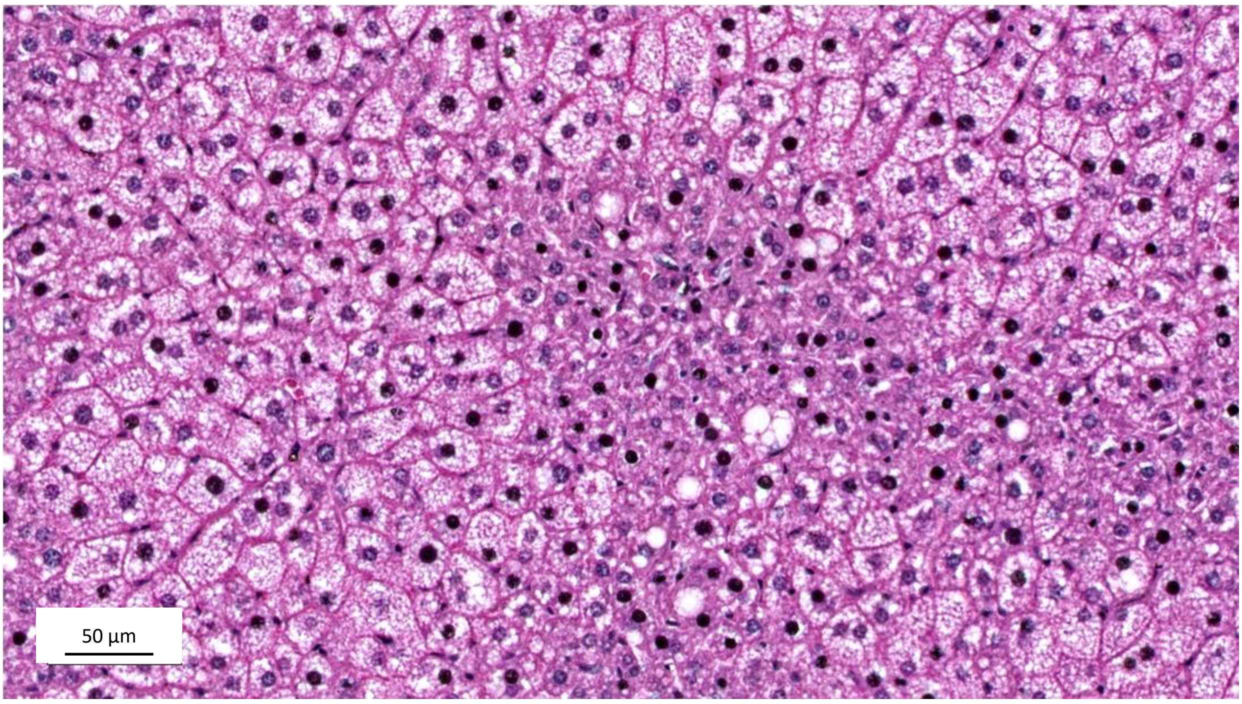

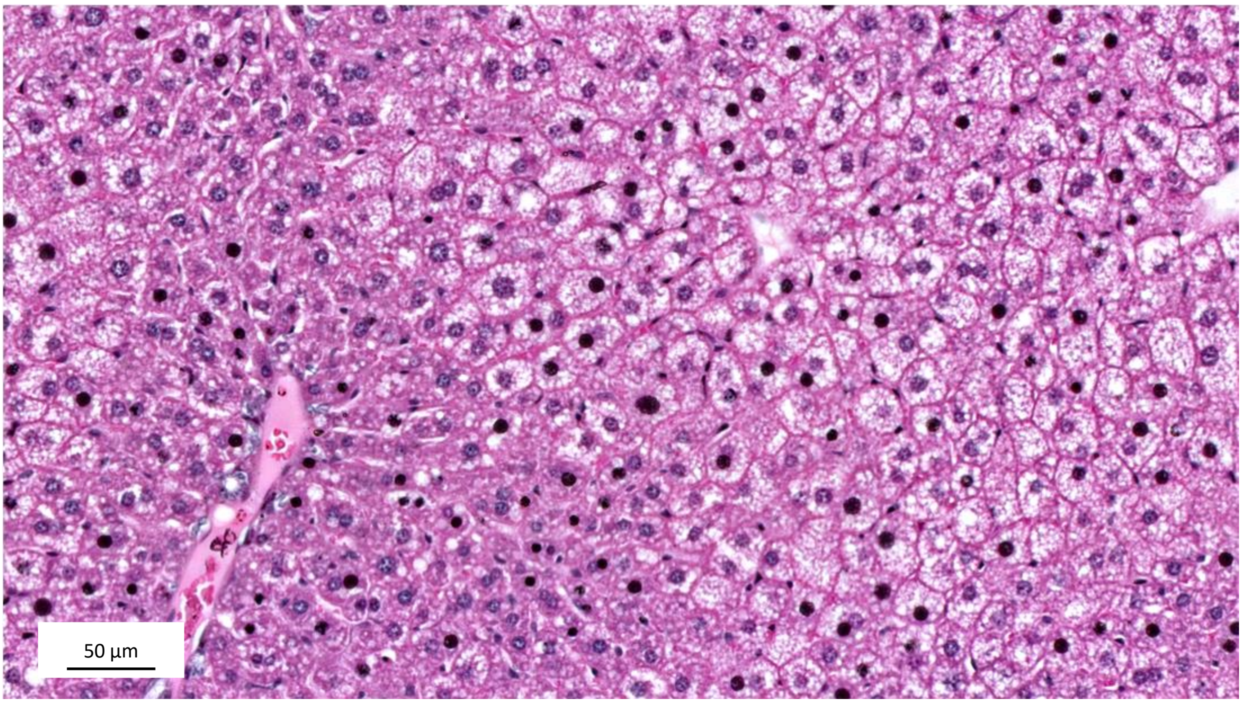

CDHFD-C-14

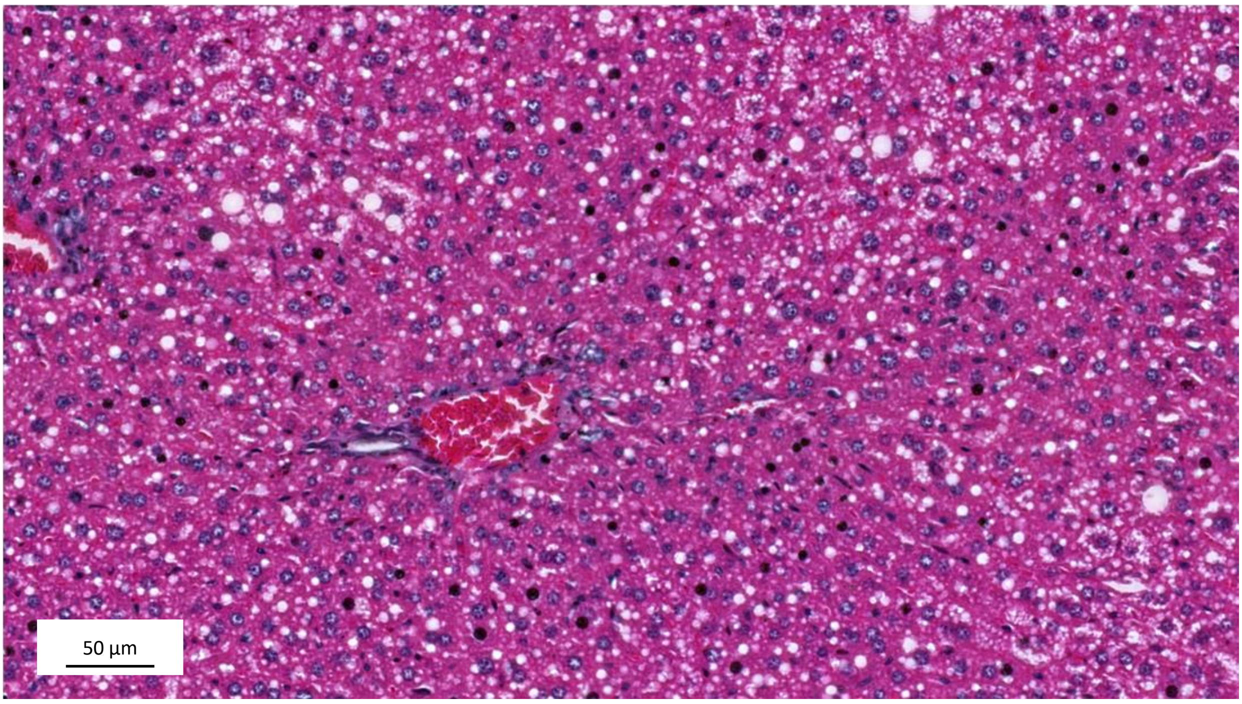

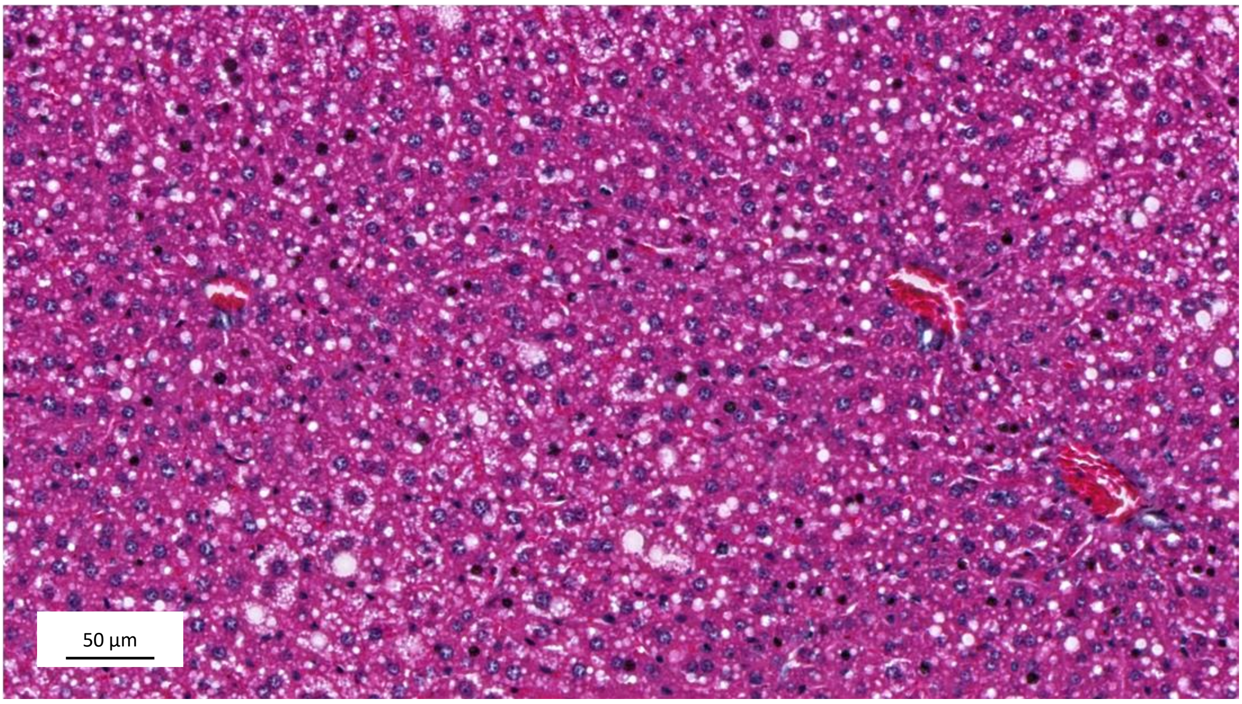

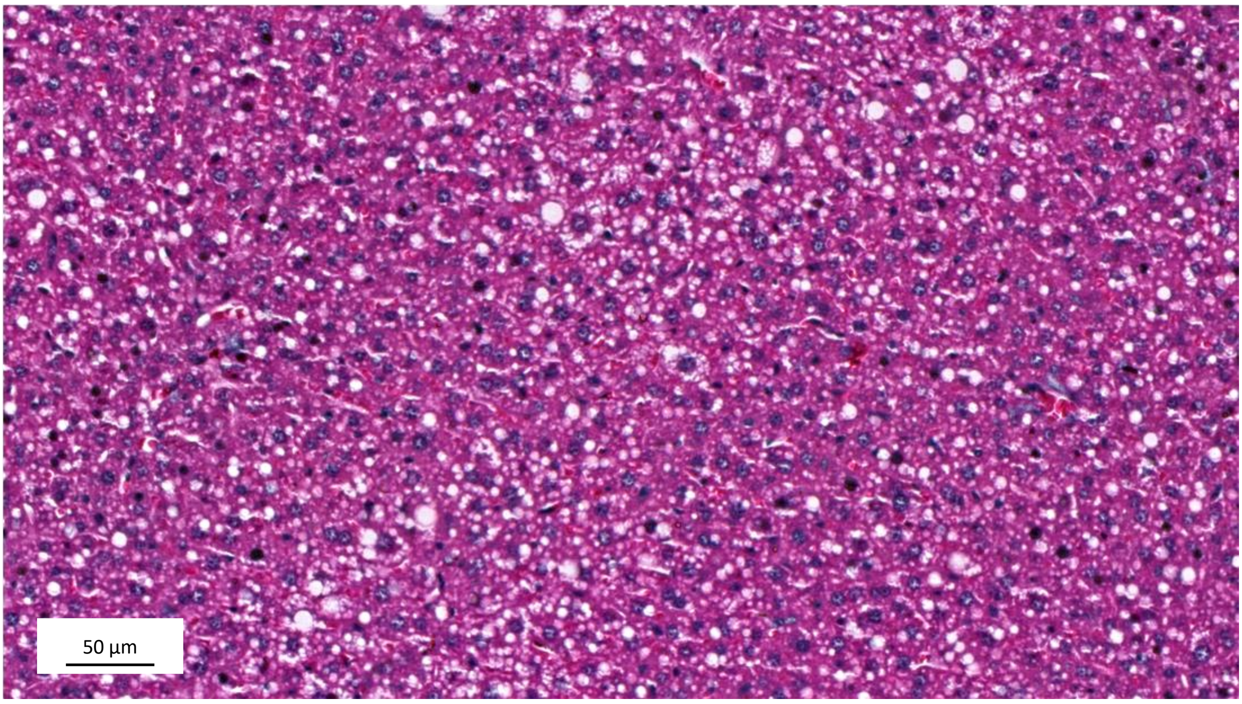

CDHFD-C-15

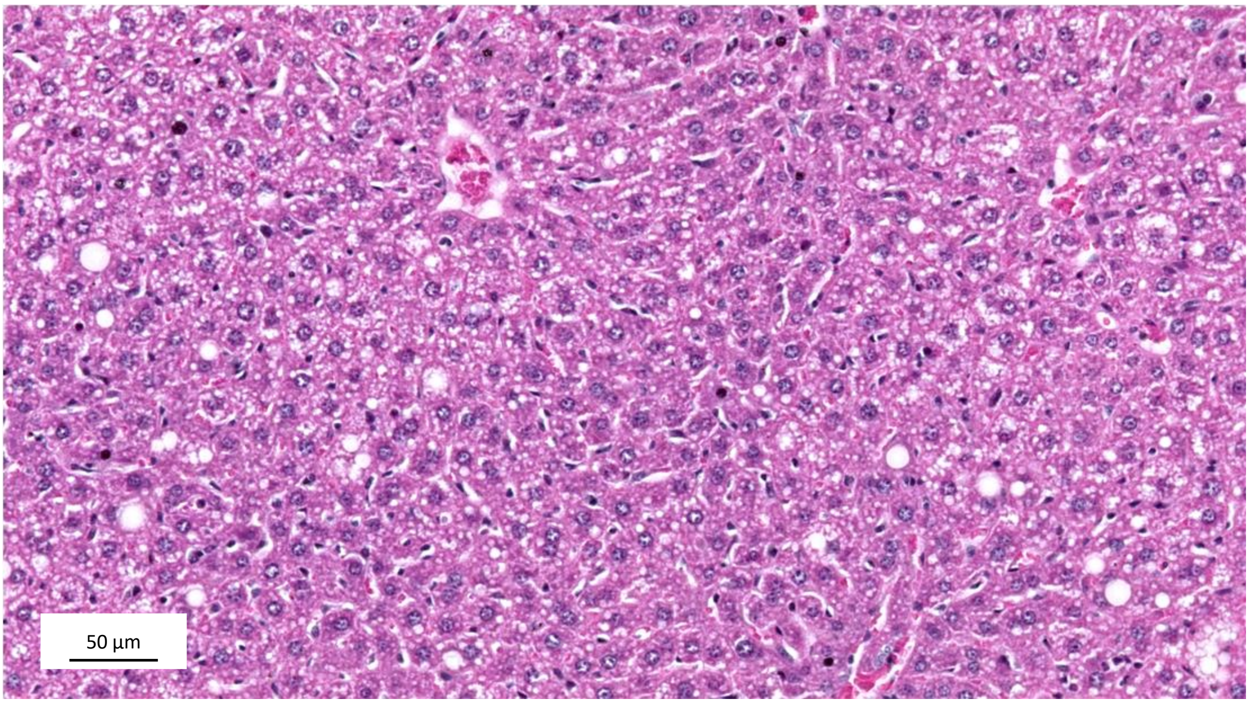

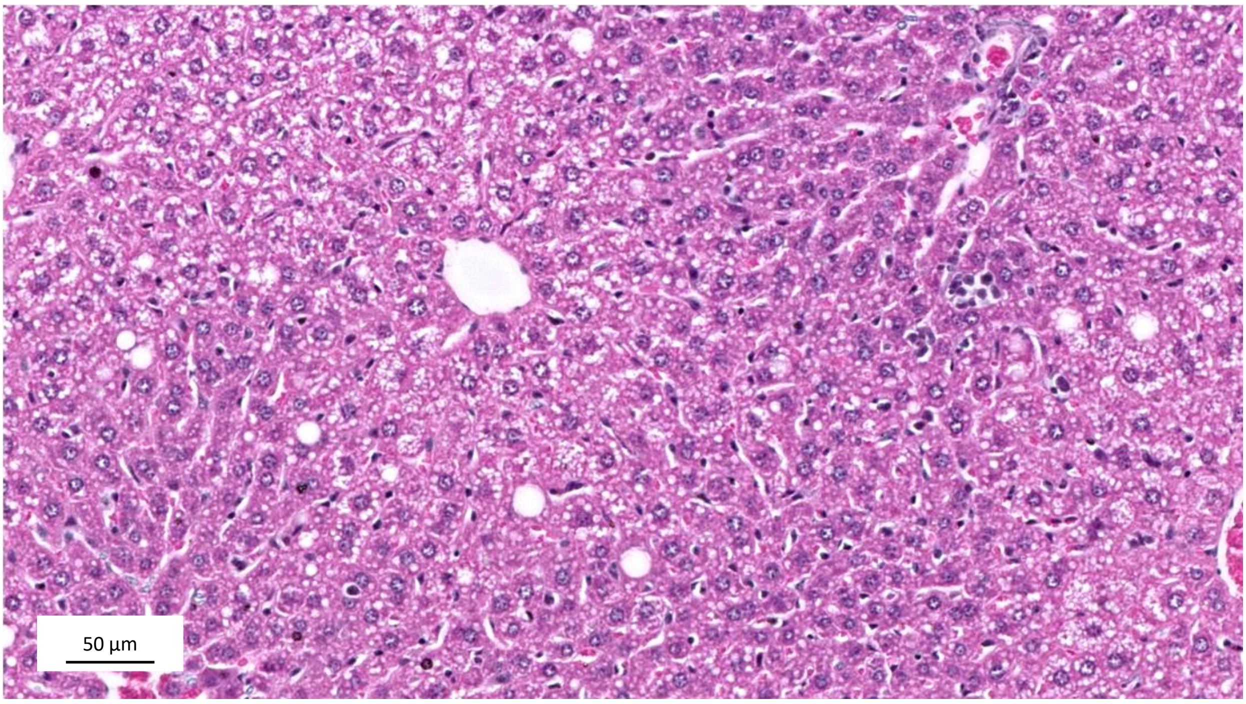

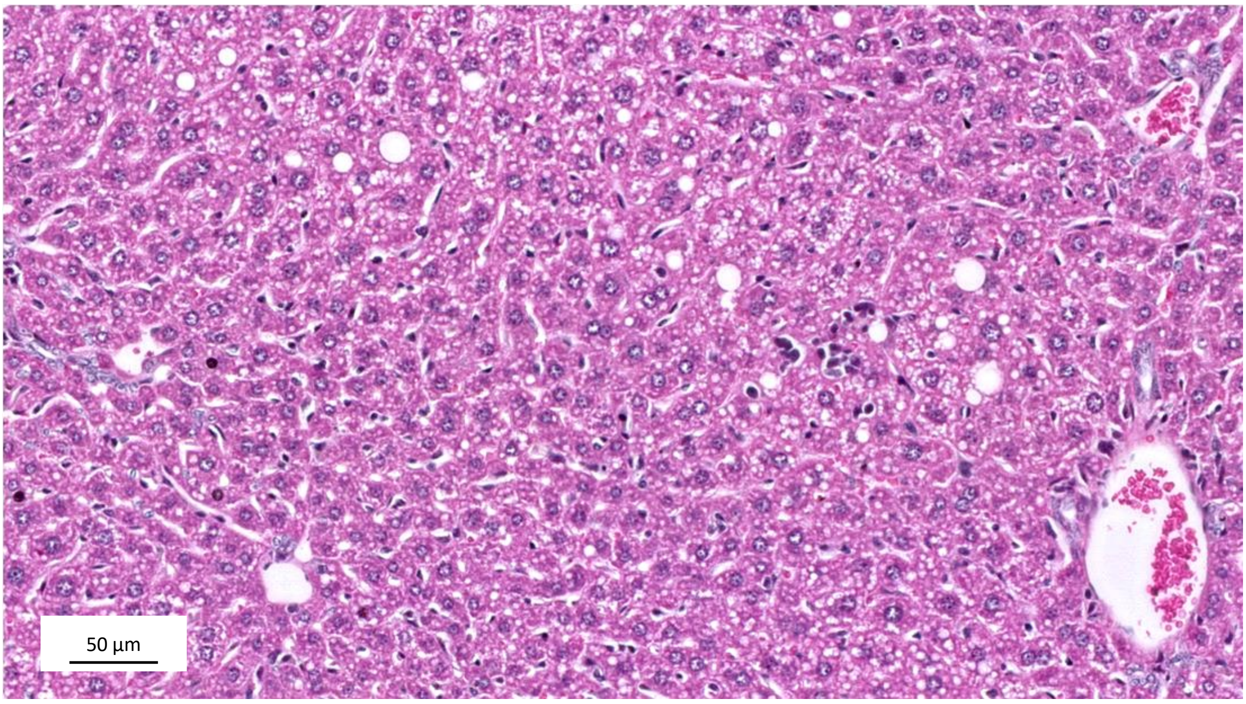

Sirius red Staining

NCD group

(10 mice were included)

NCD-1

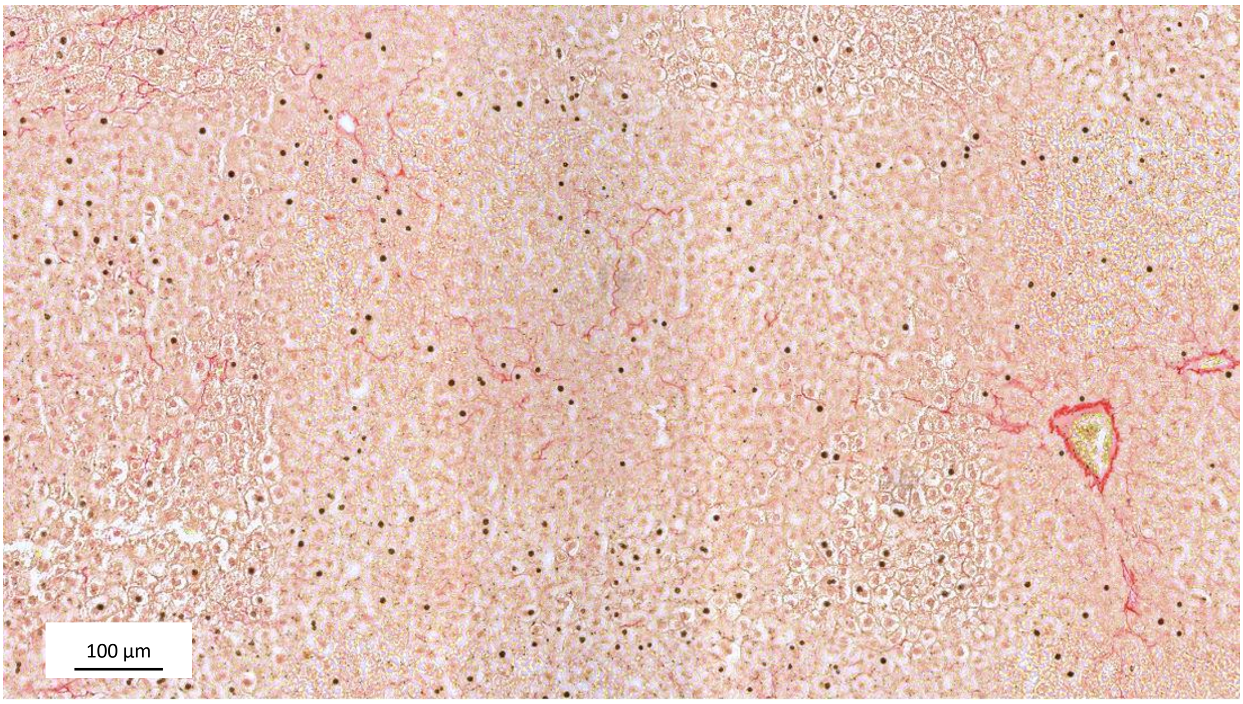

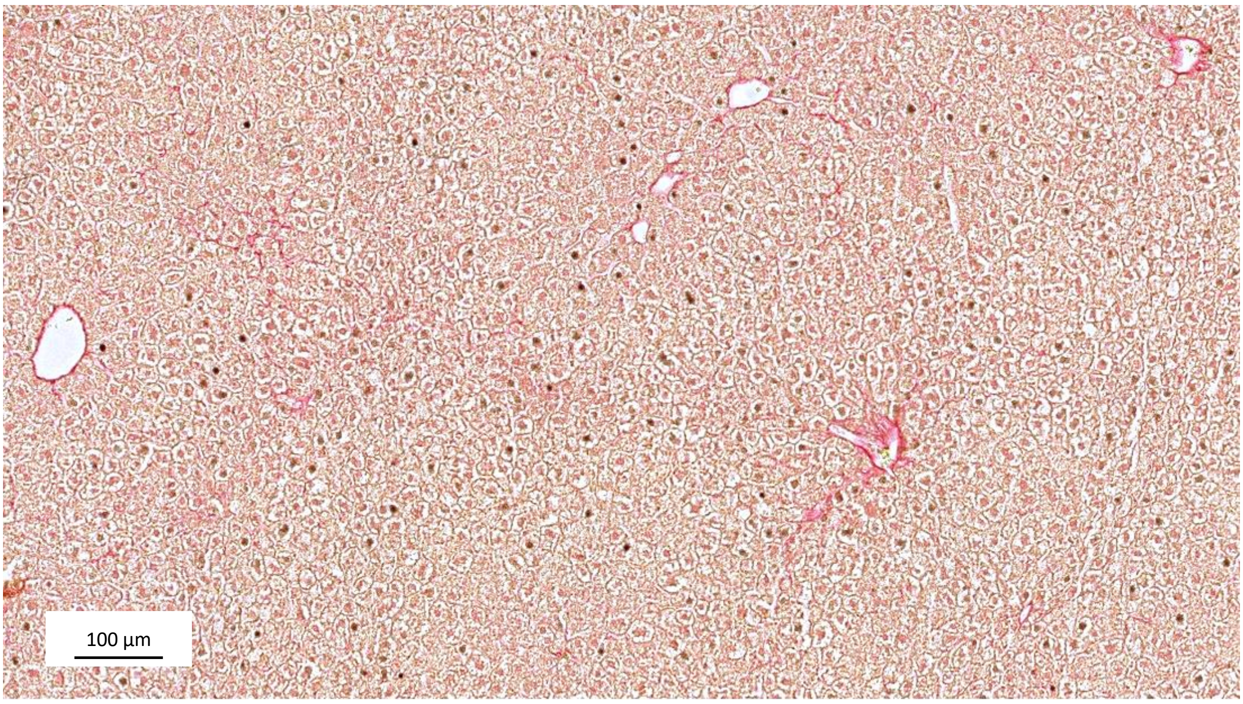

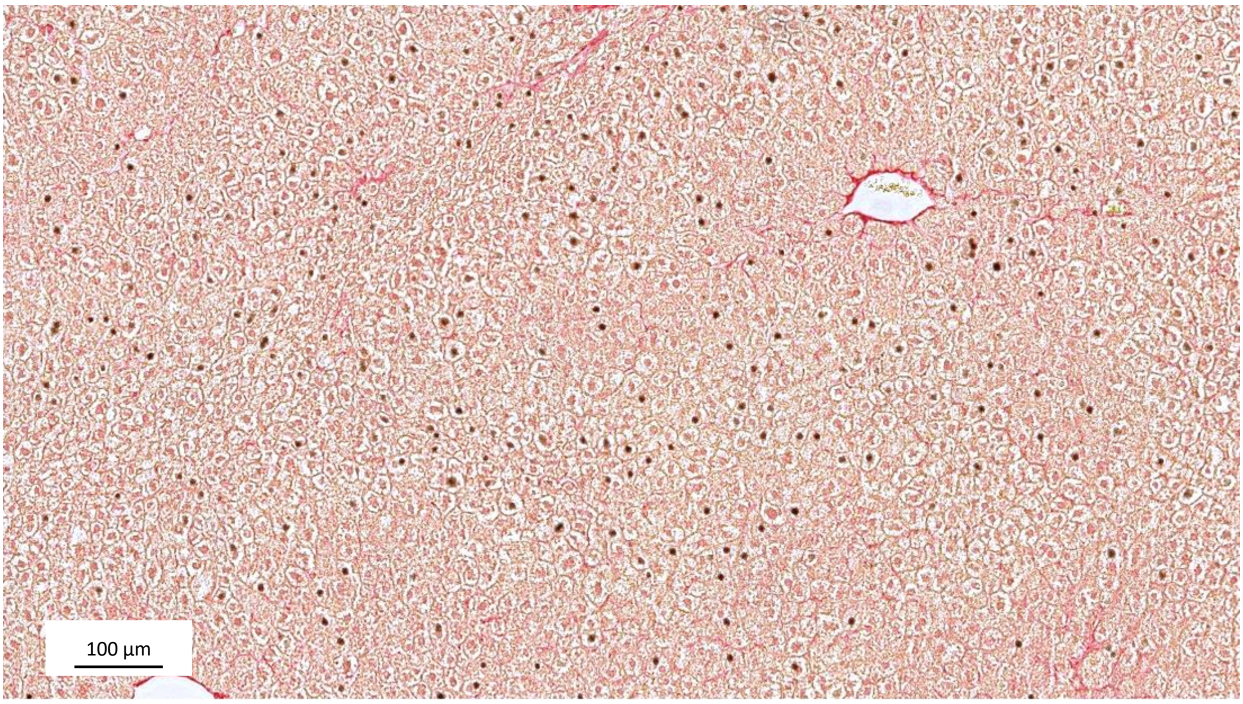

NCD-2

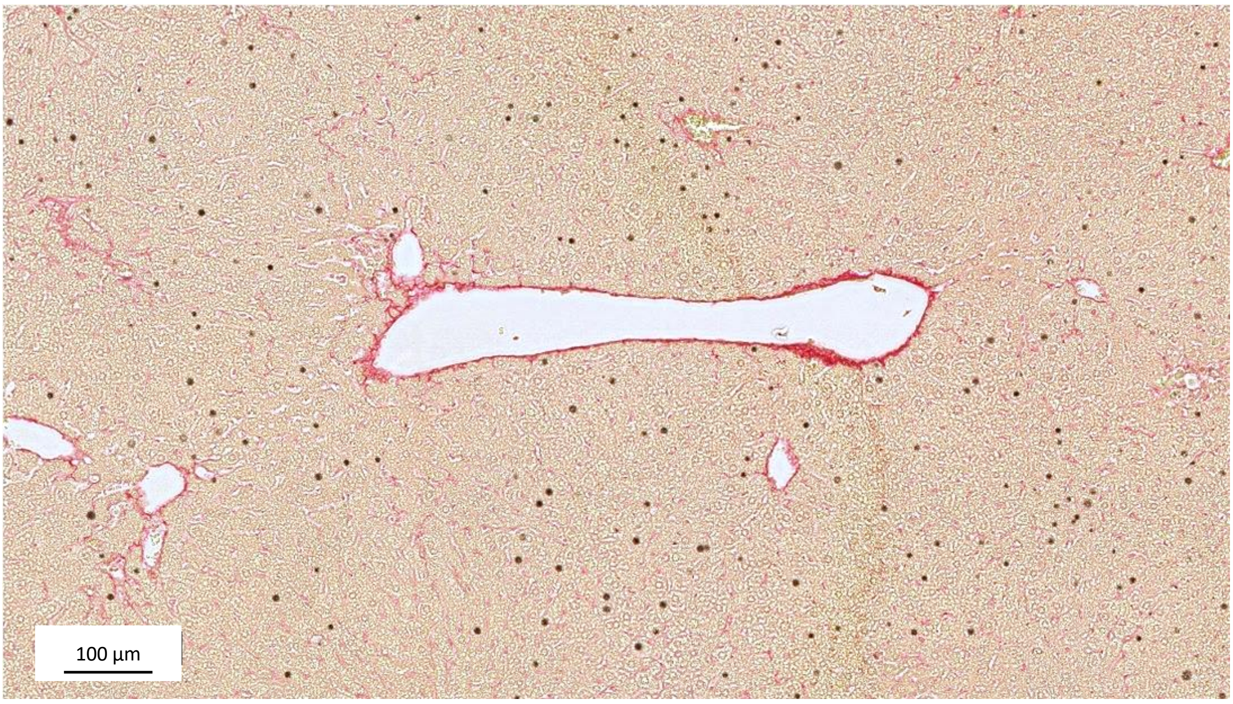

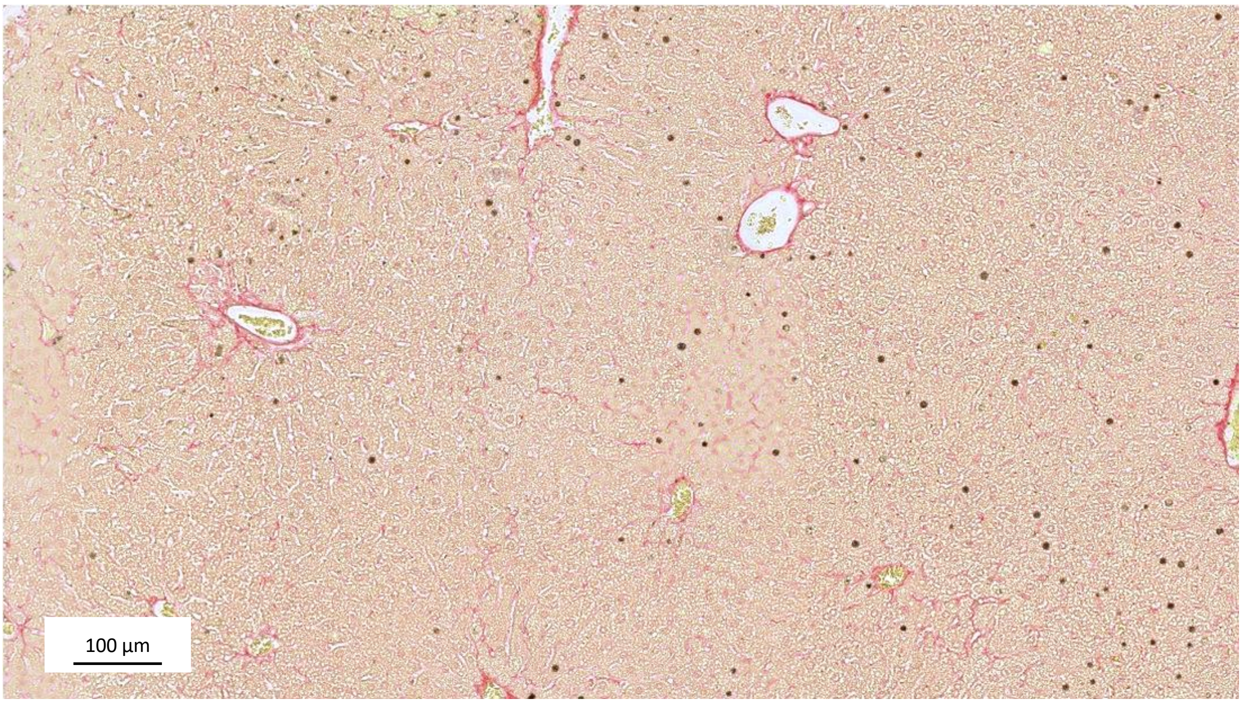

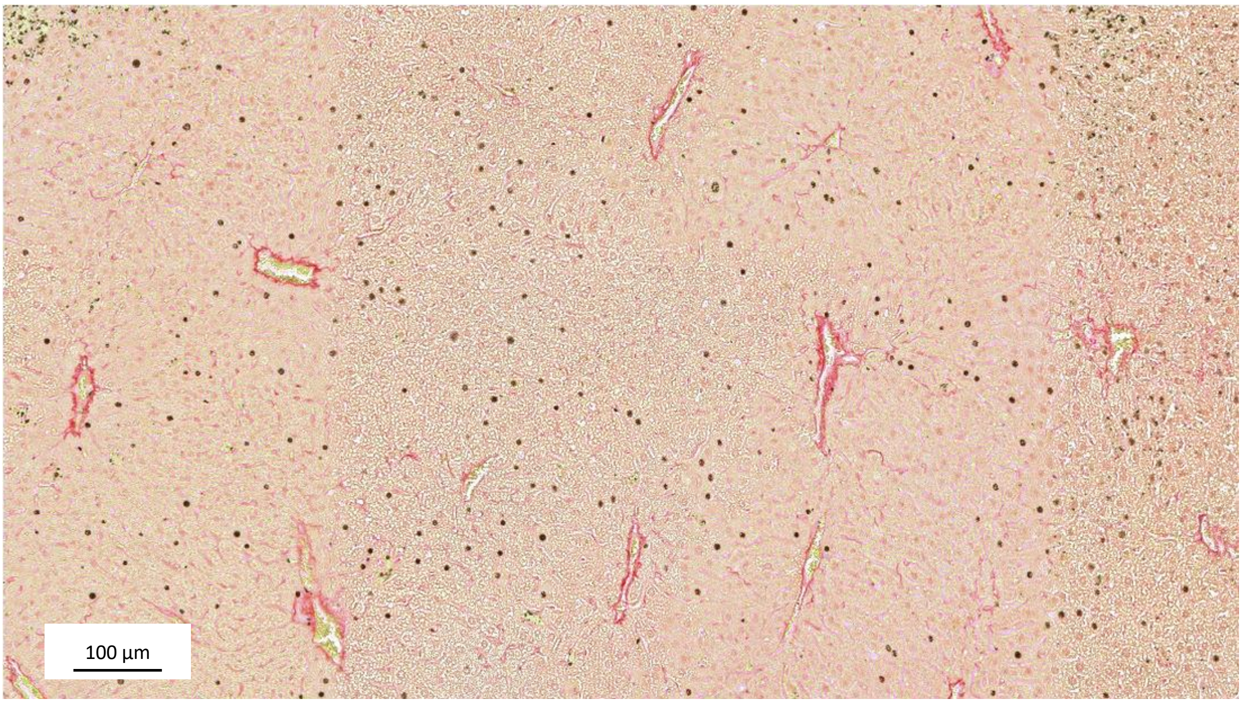

NCD-3

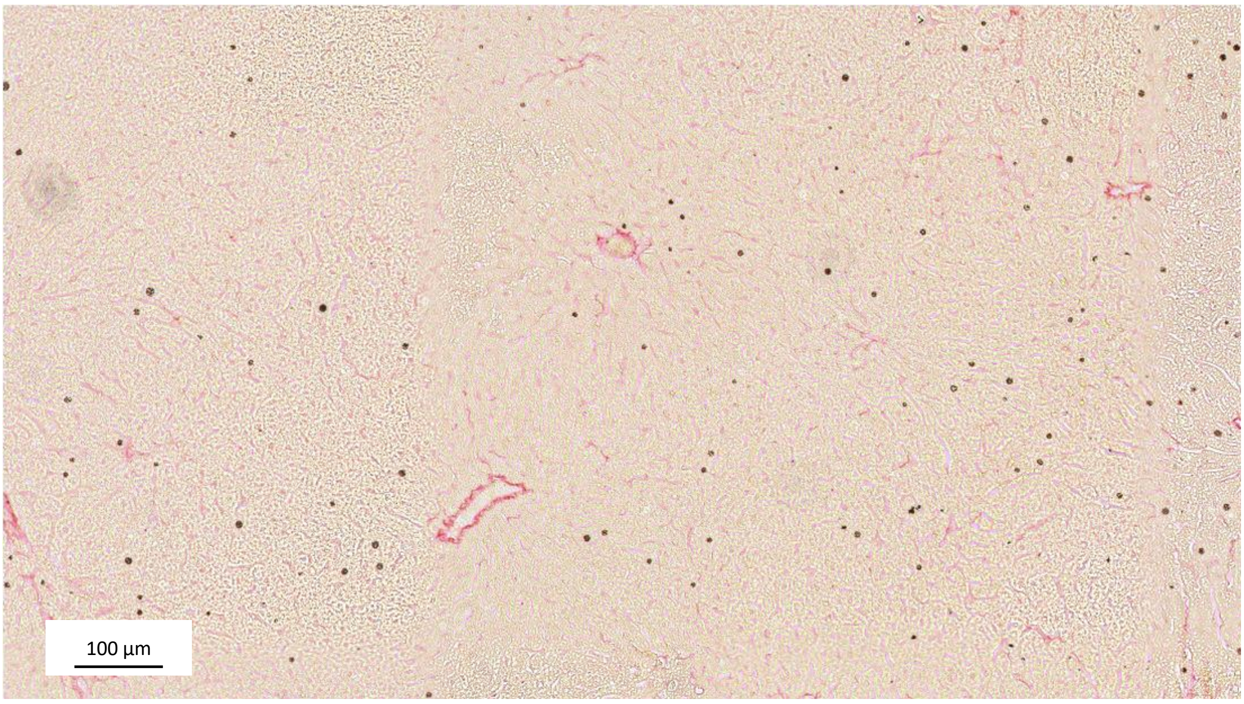

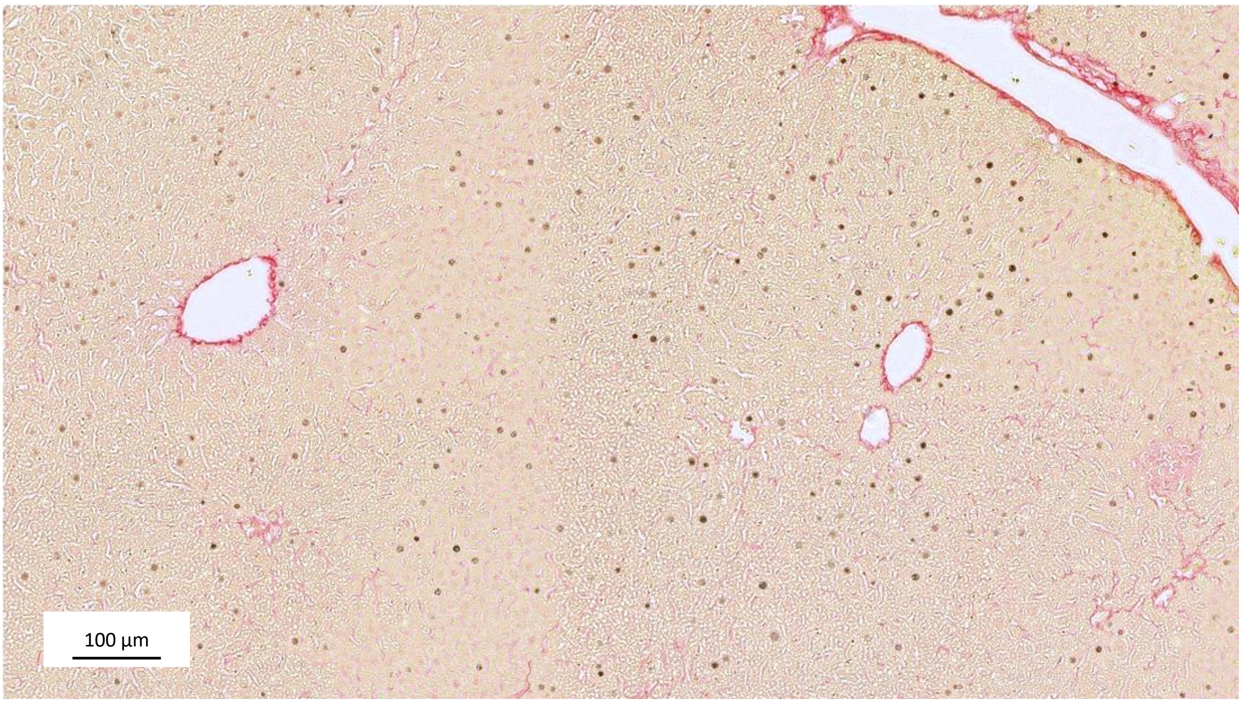

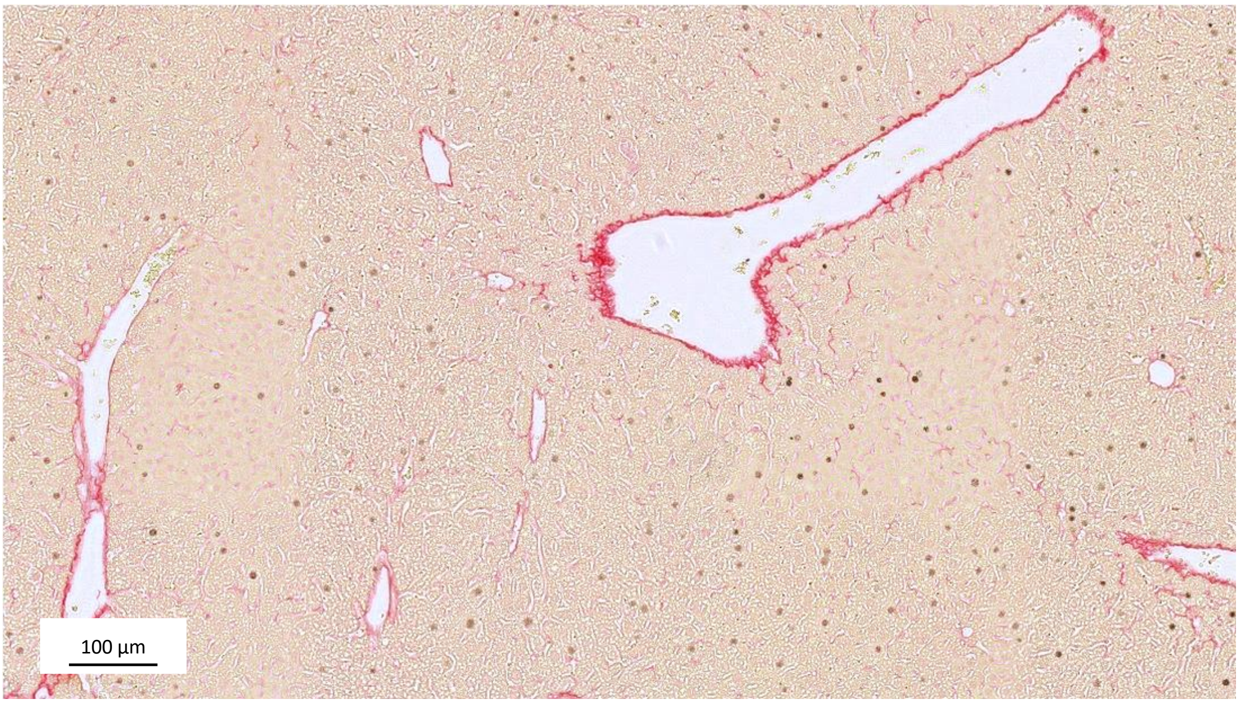

NCD-4

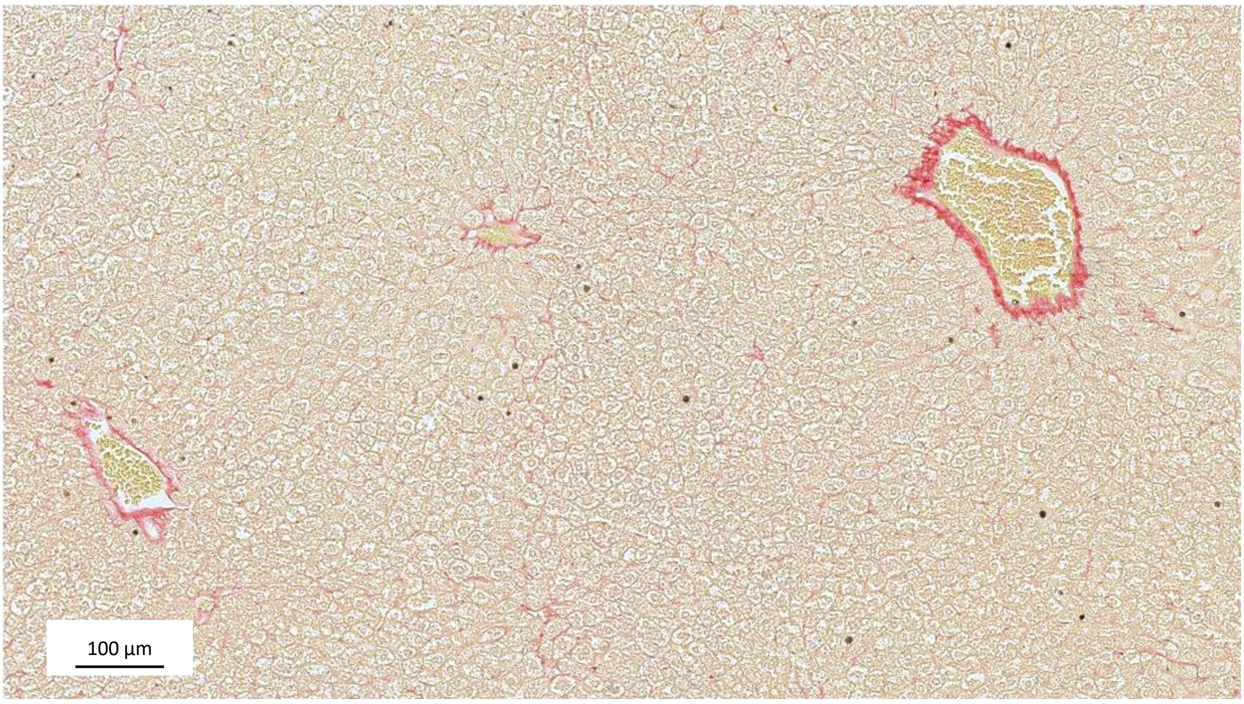

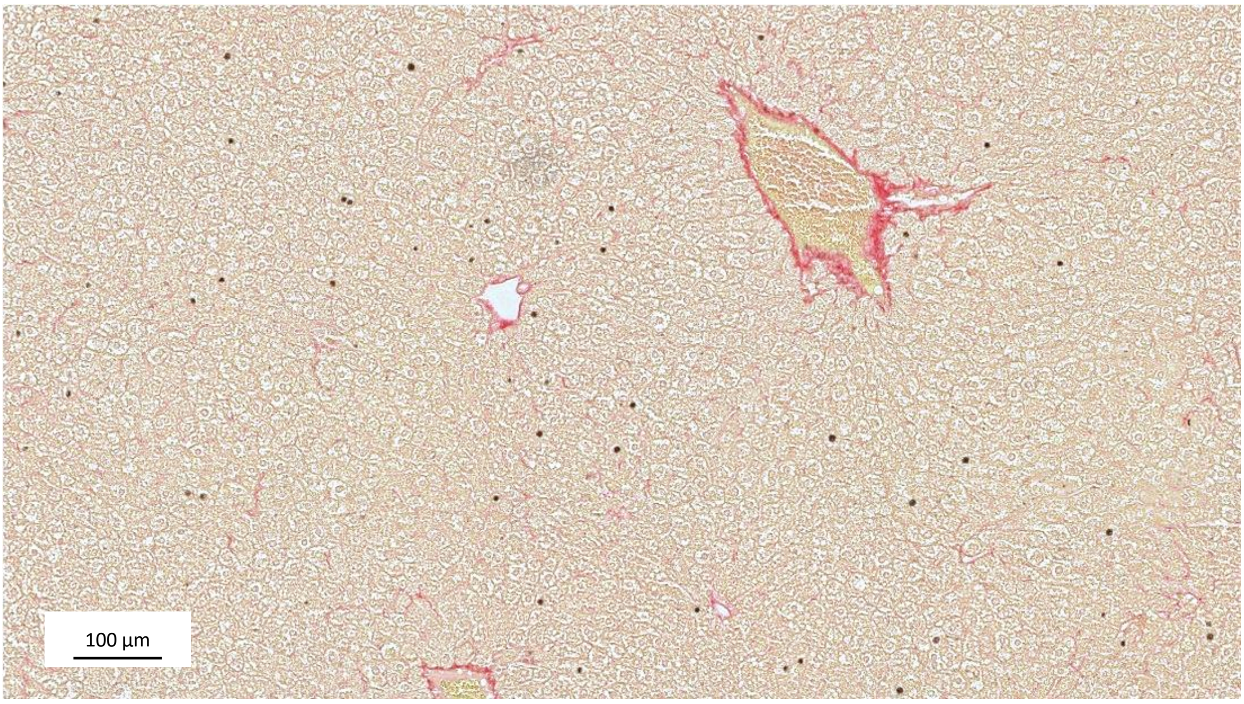

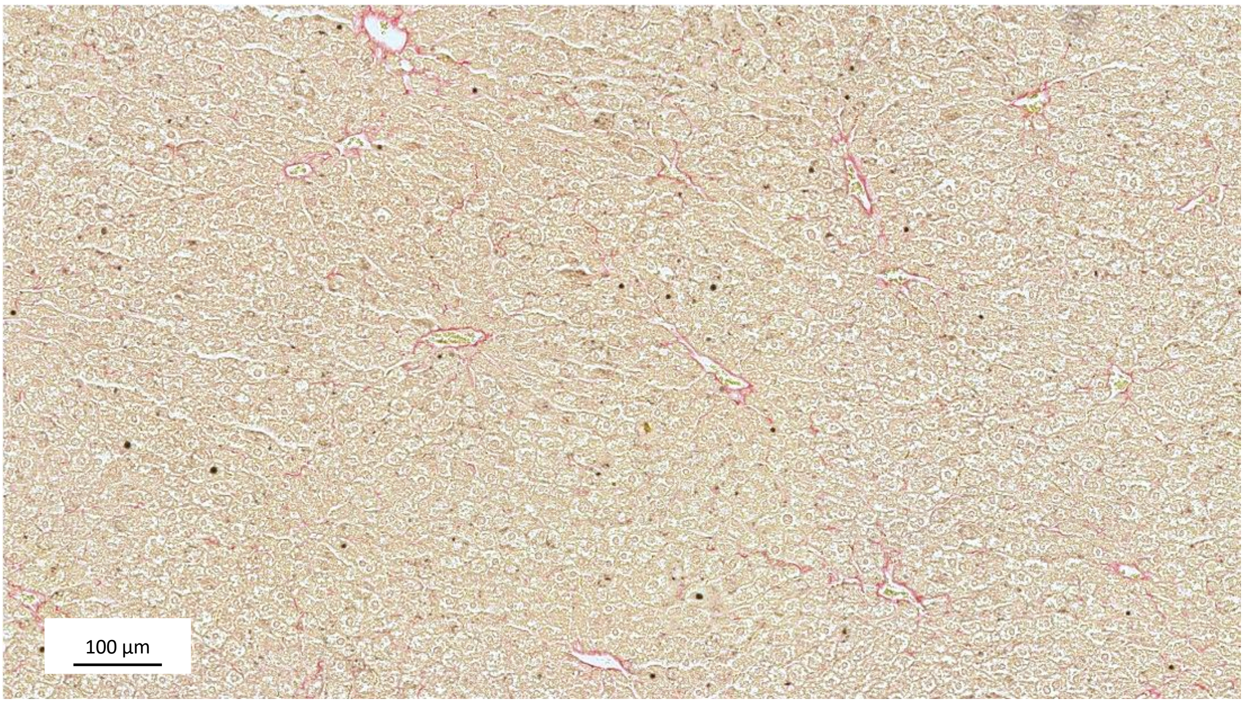

NCD-5

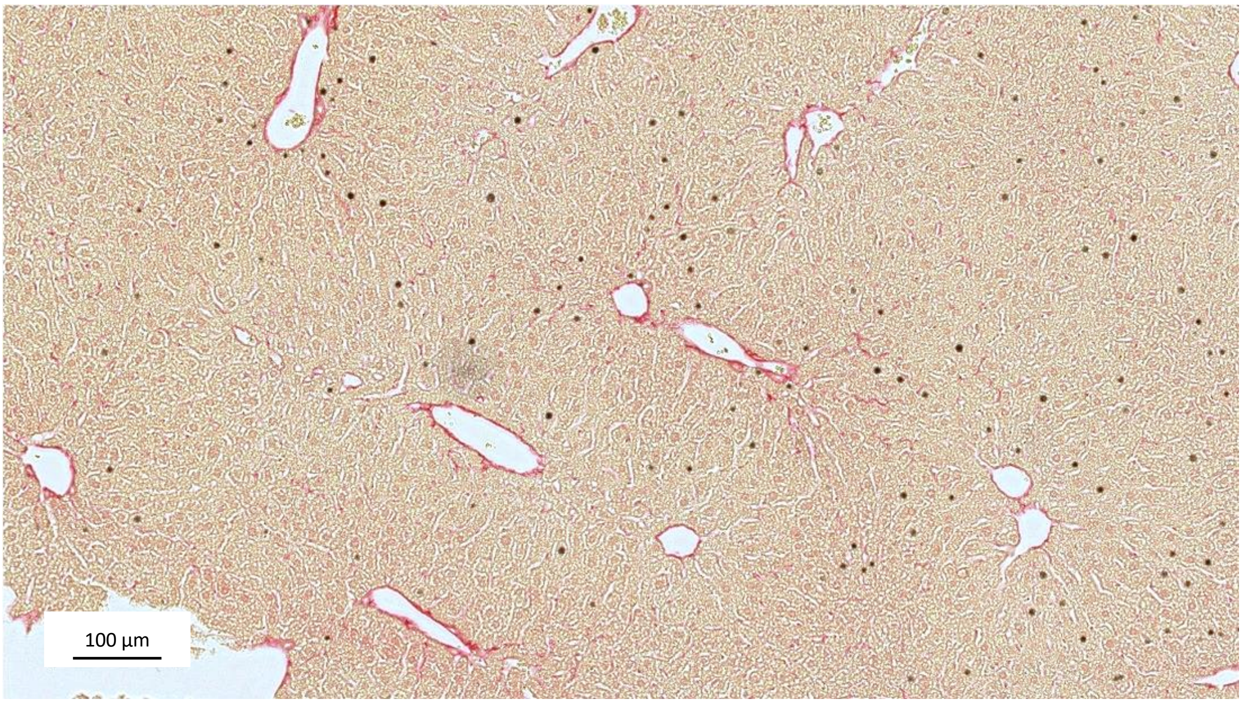

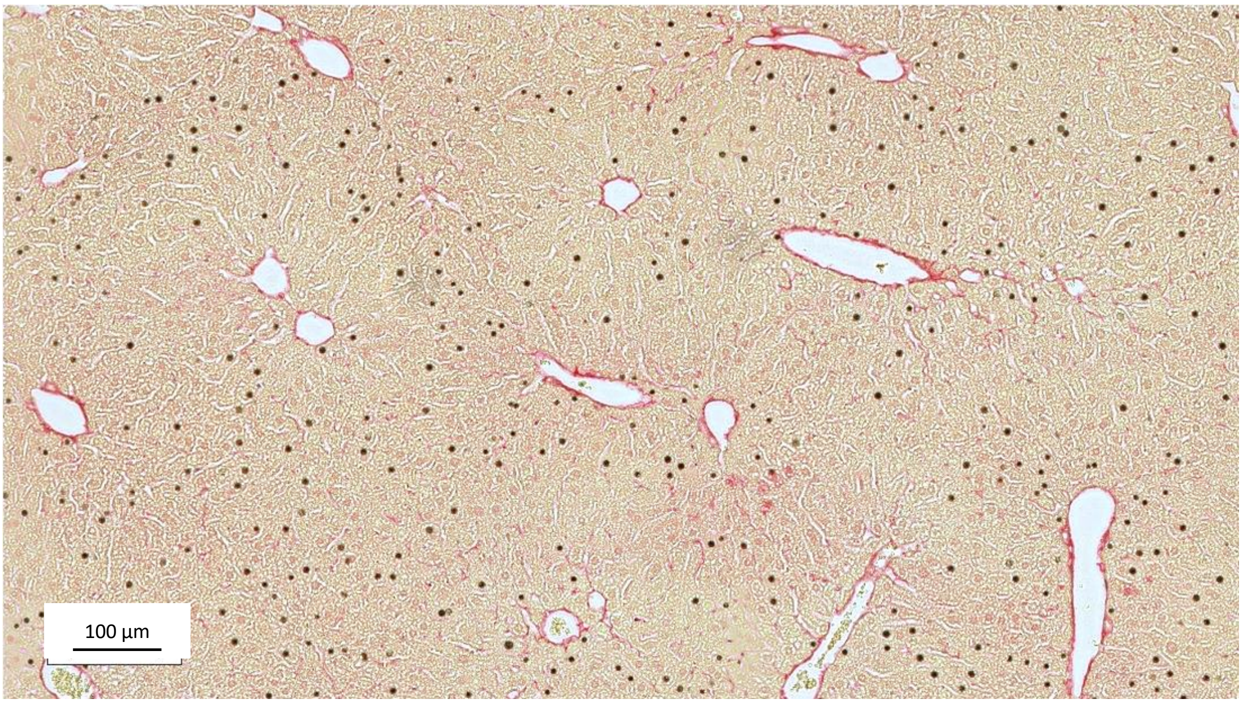

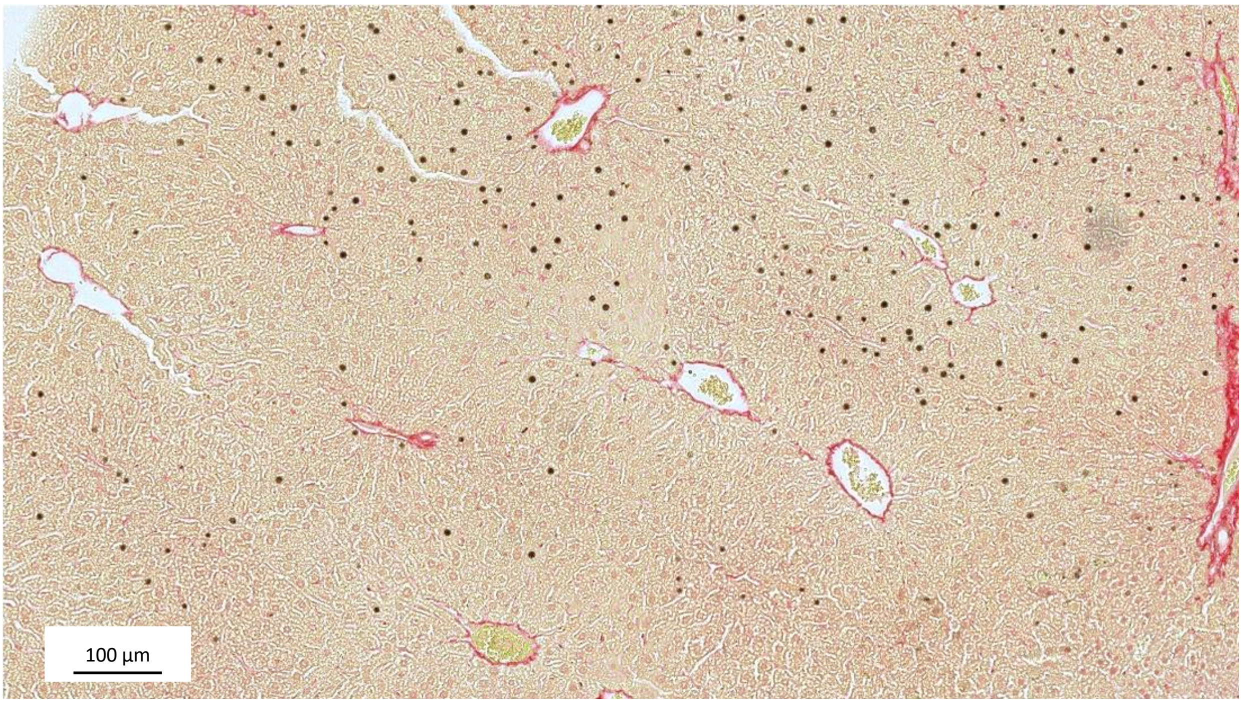

NCD-6

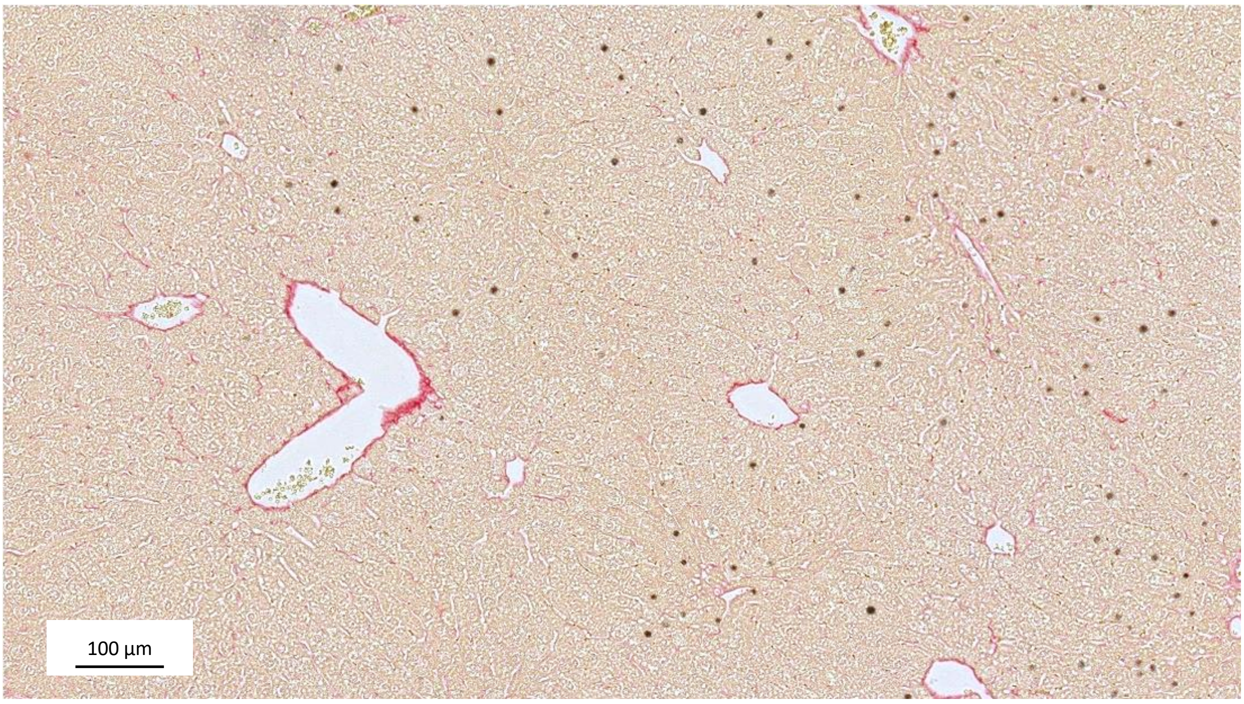

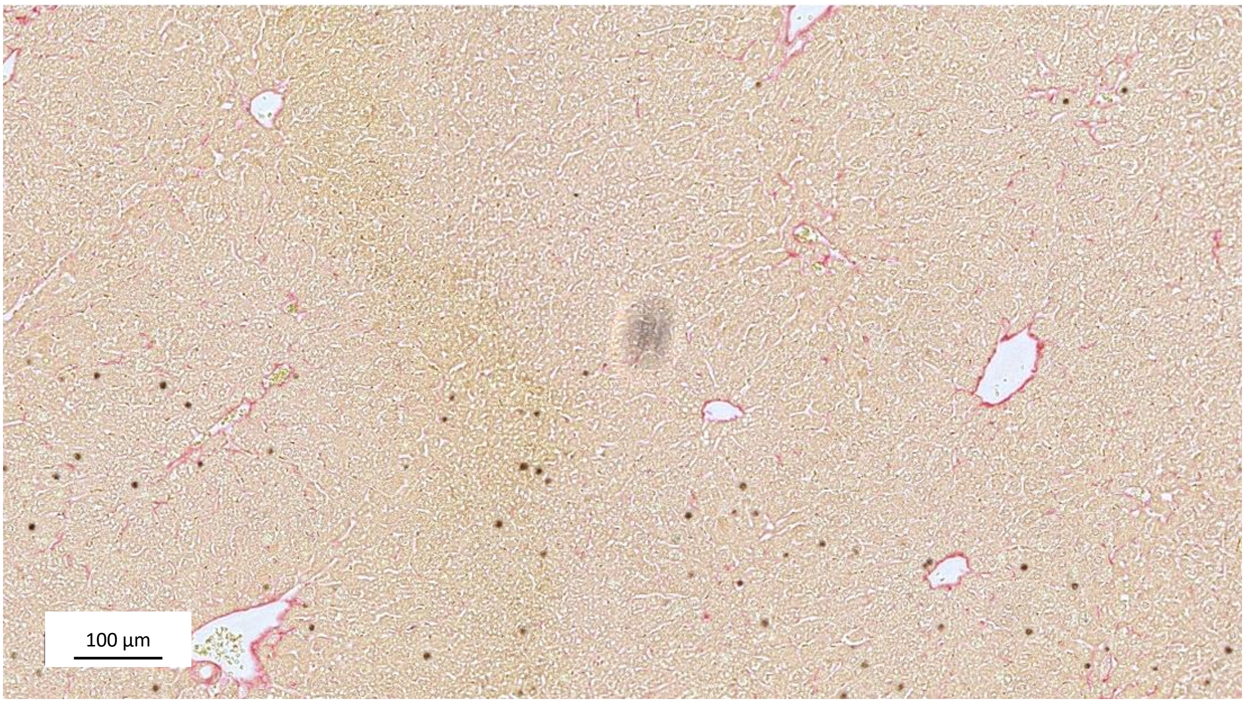

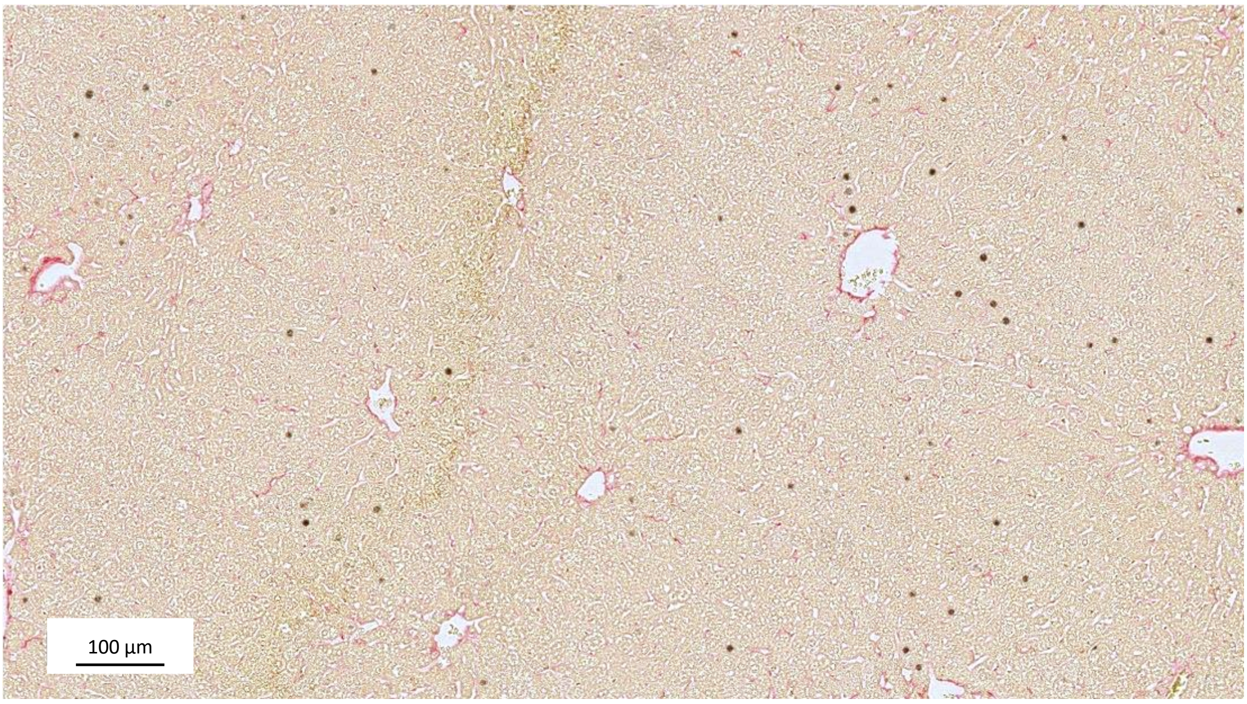

NCD-7

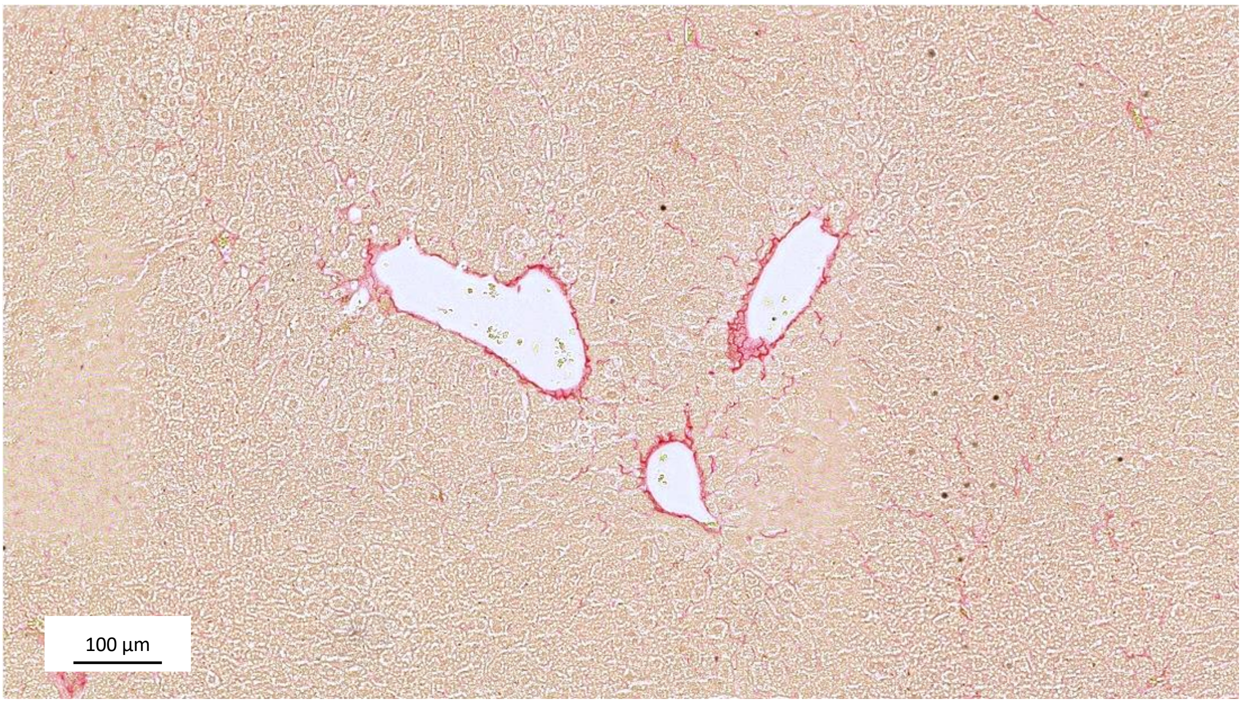

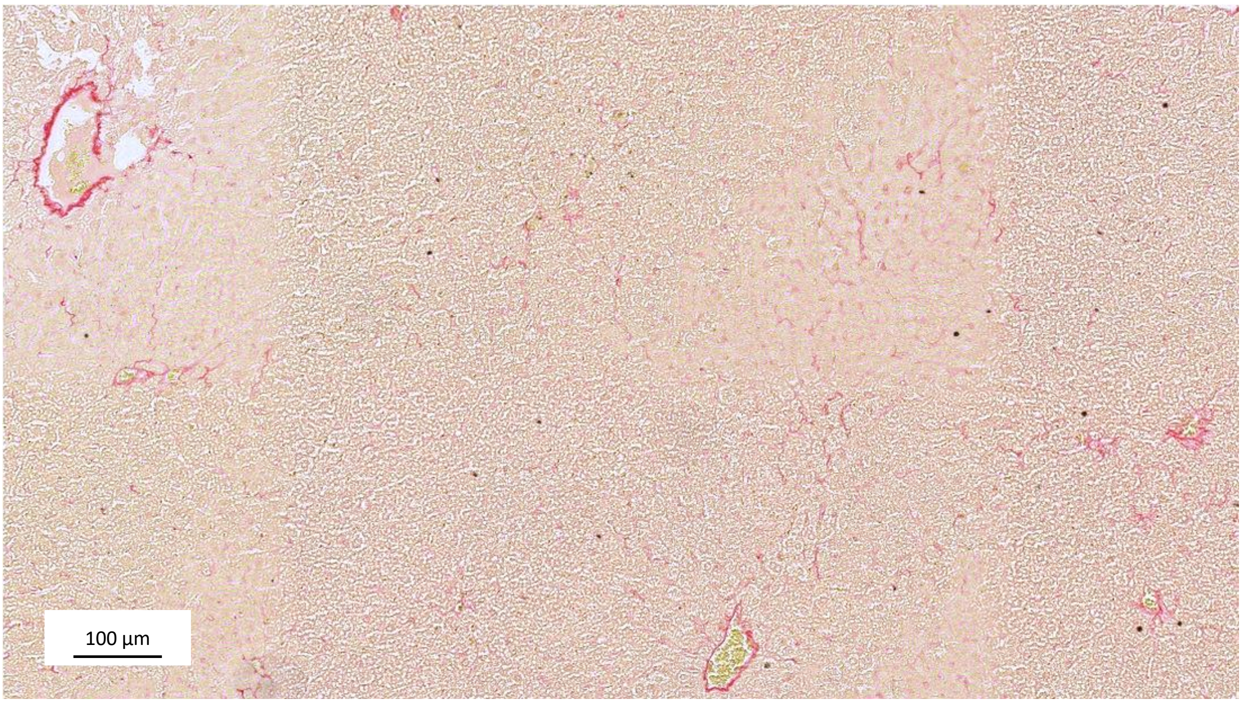

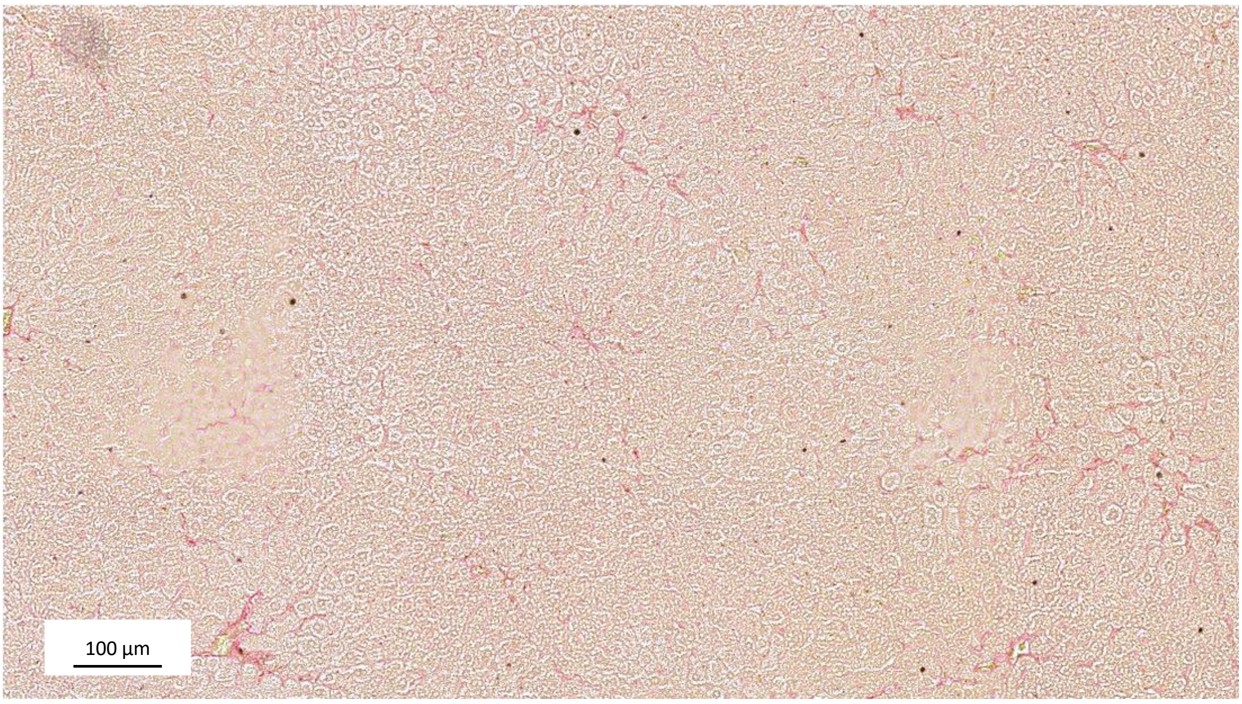

NCD-8

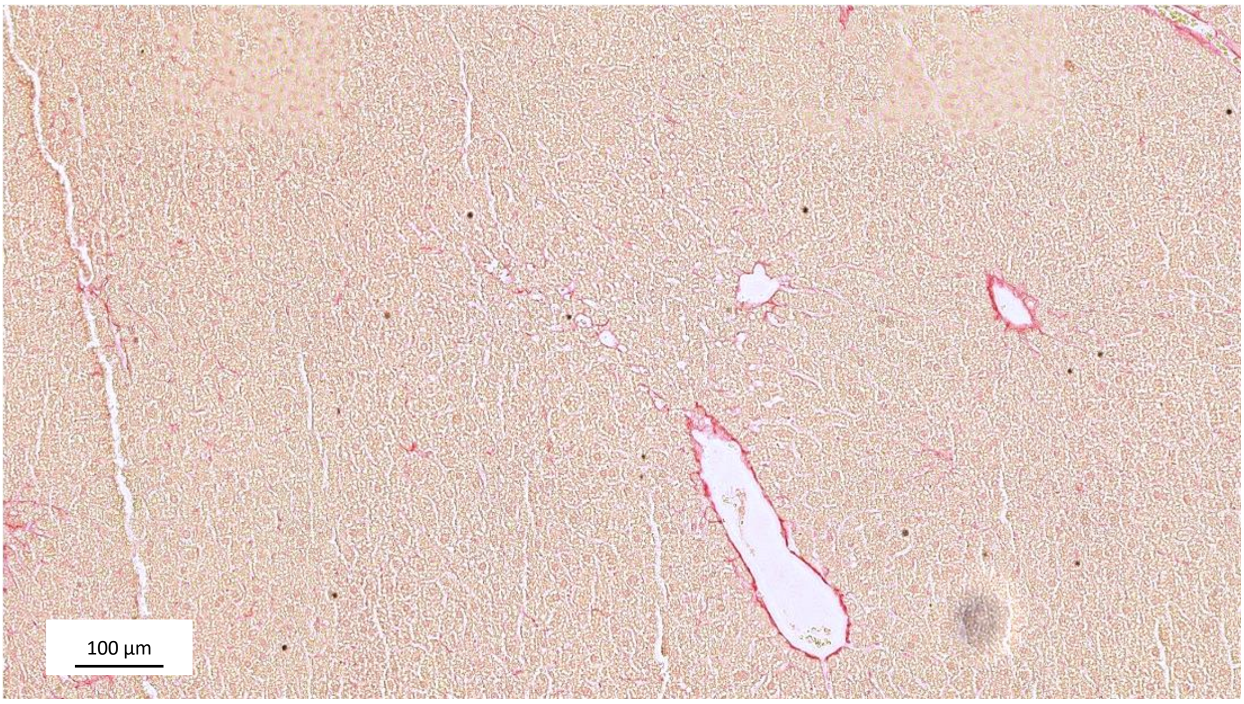

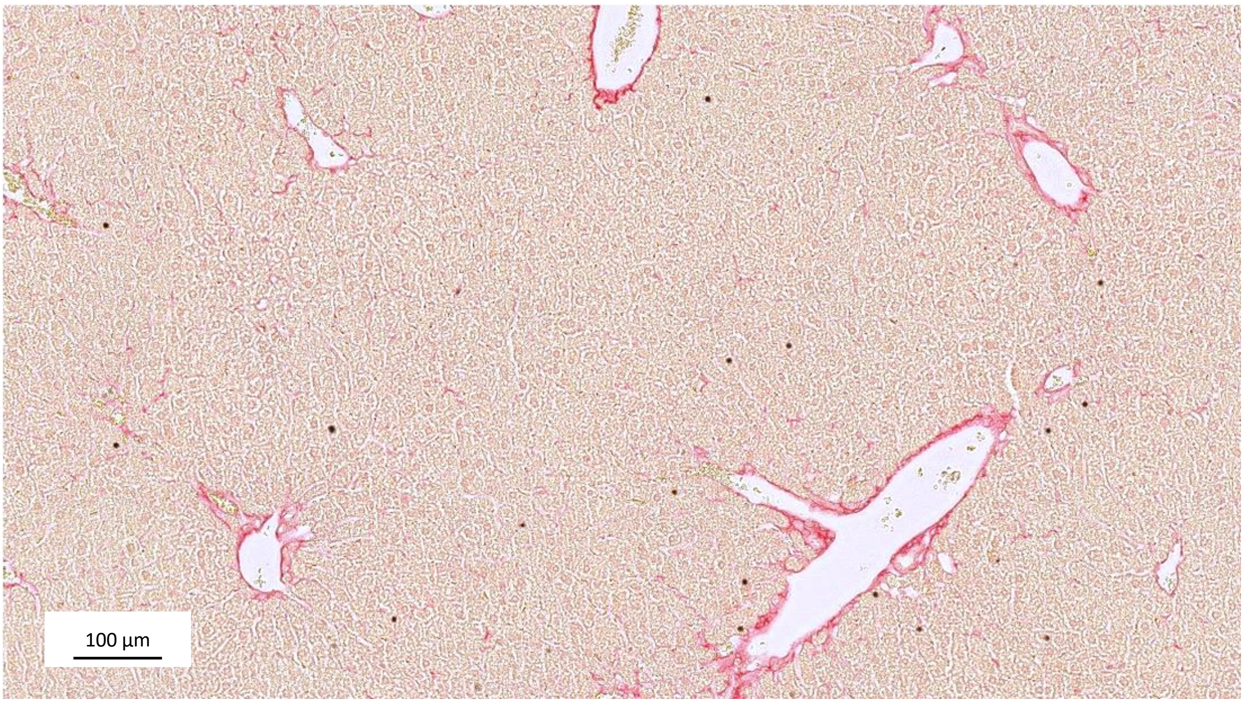

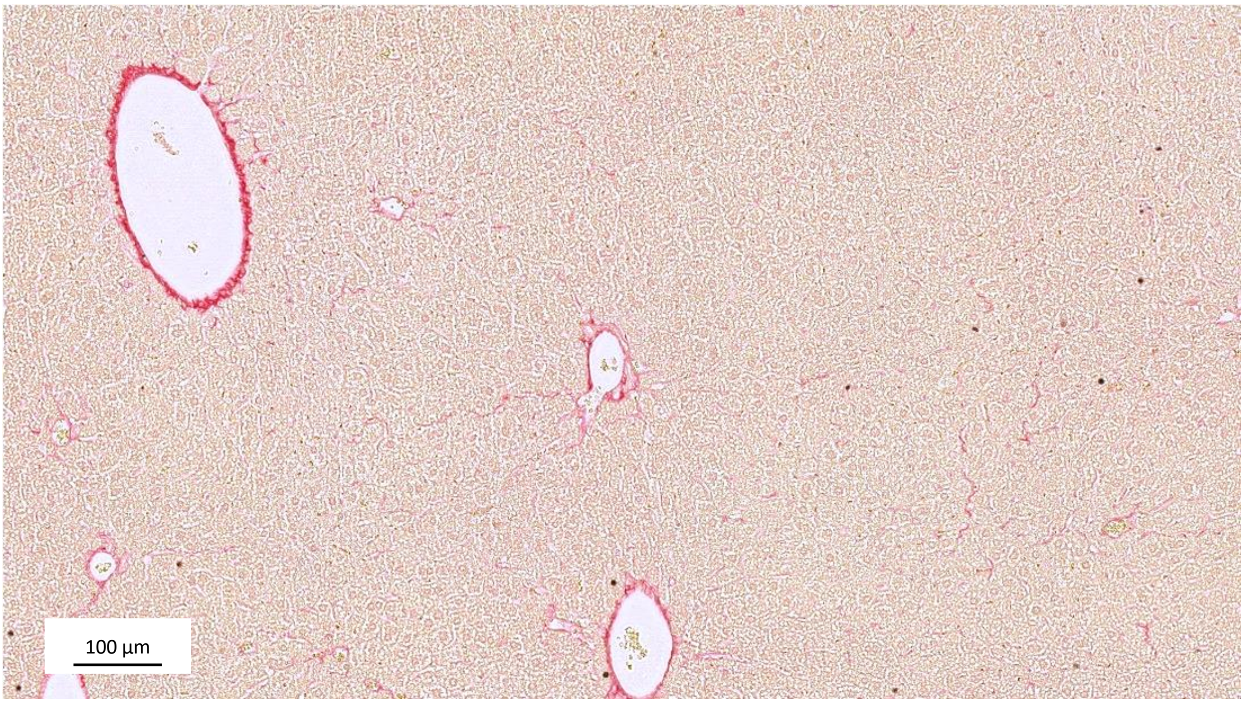

NCD-9

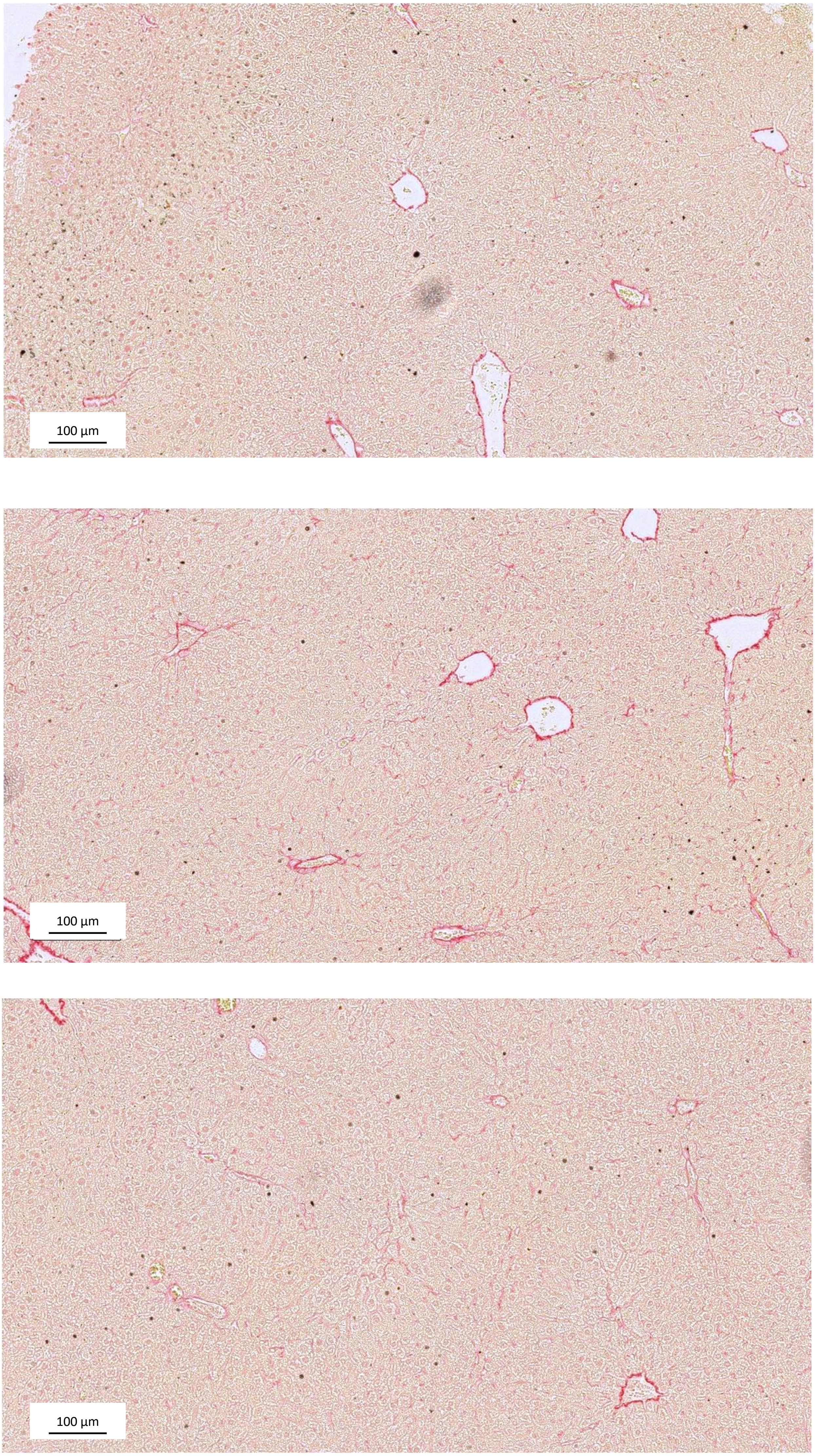

NCD-10

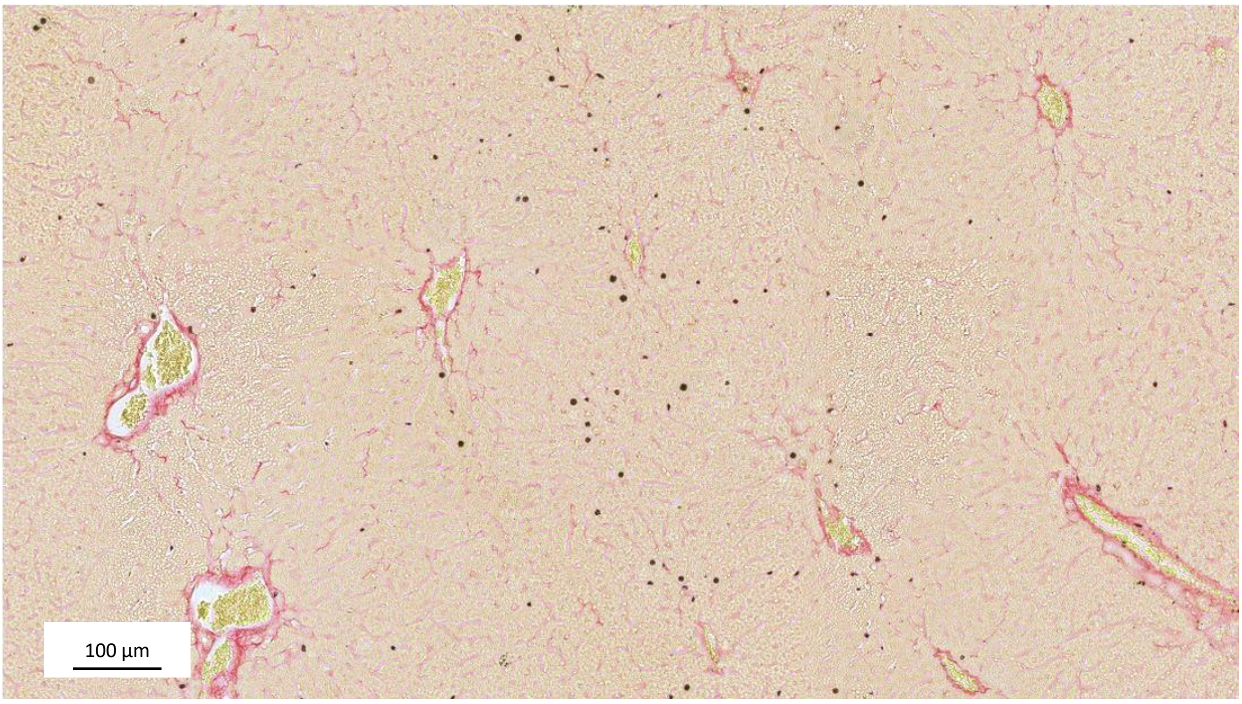

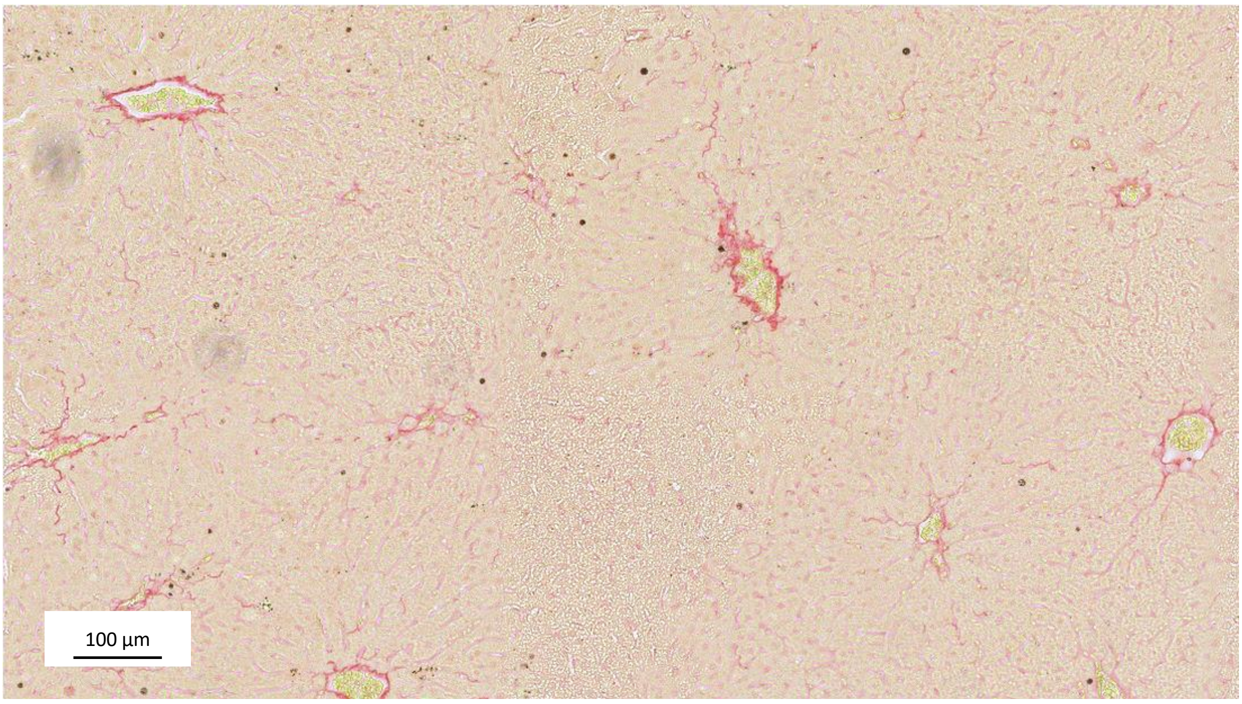

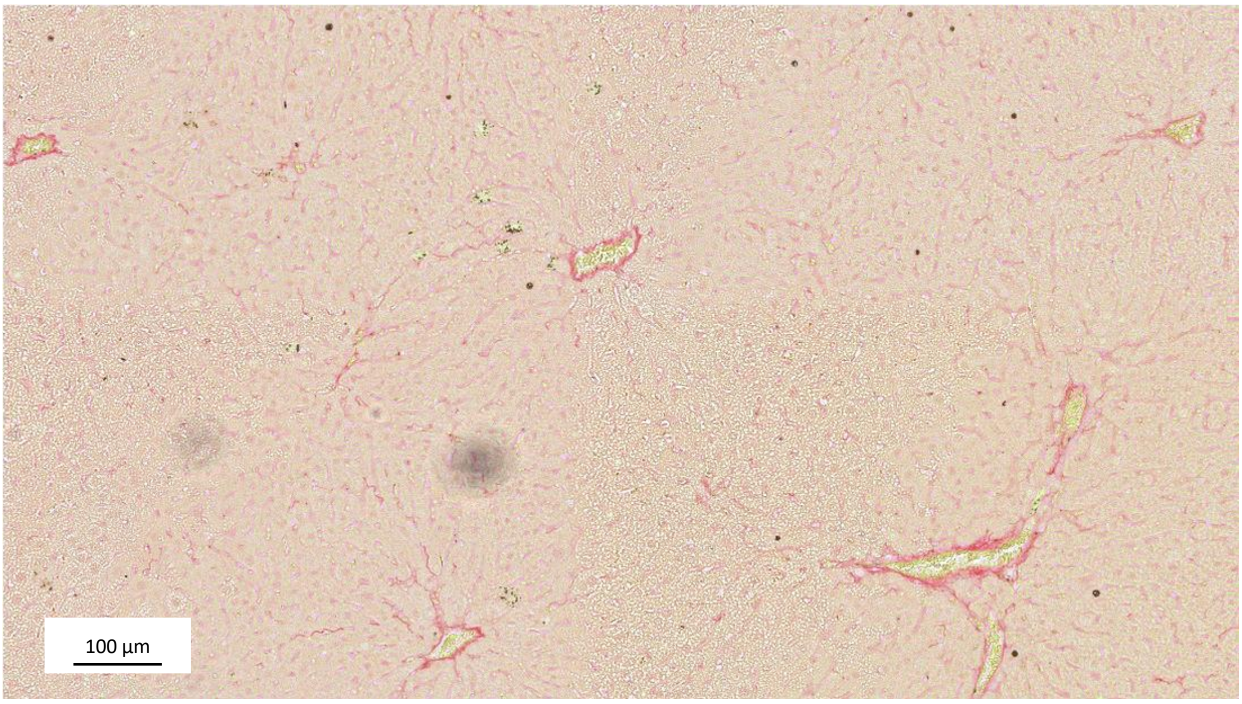

Sirius red Staining

CDHFD group

(13 mice were included)

CDHFD-1

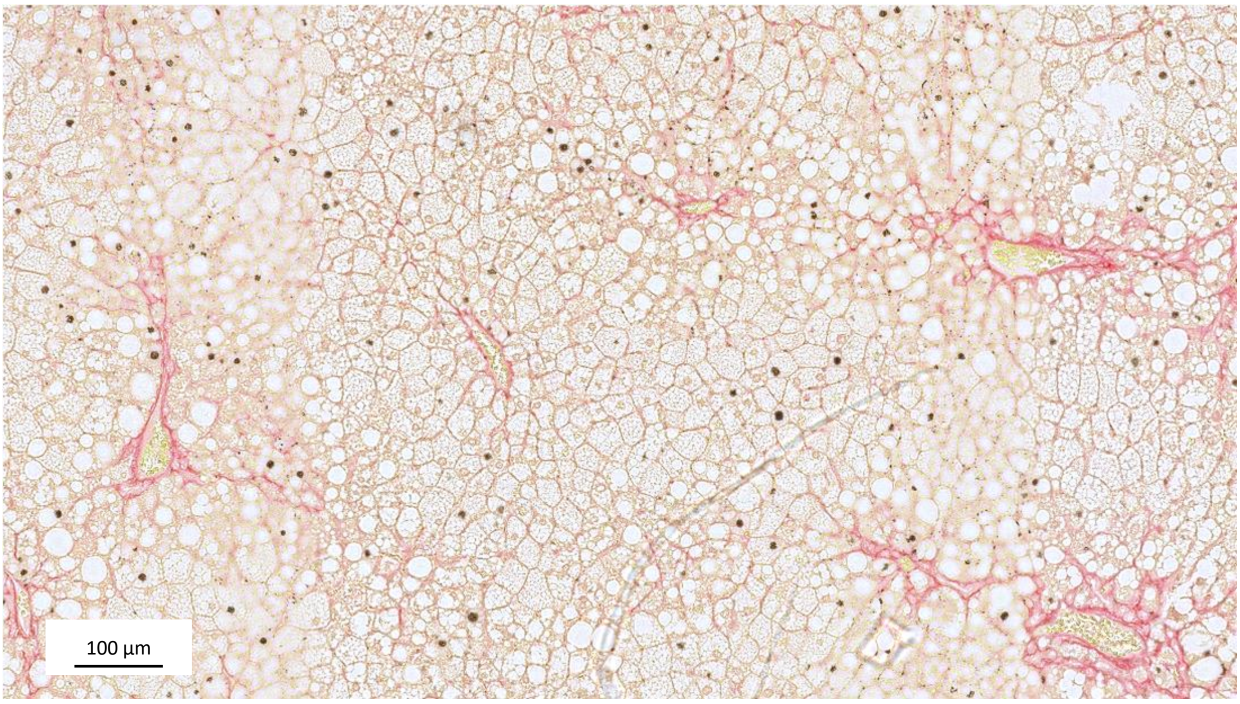

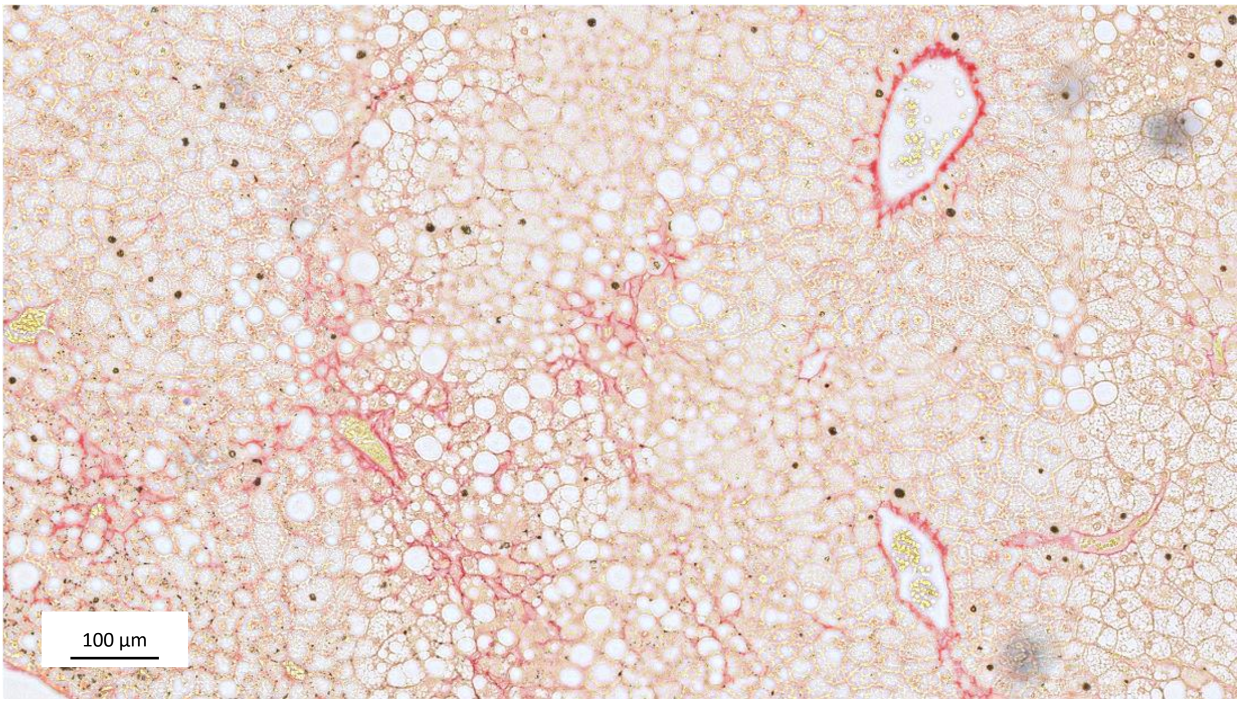

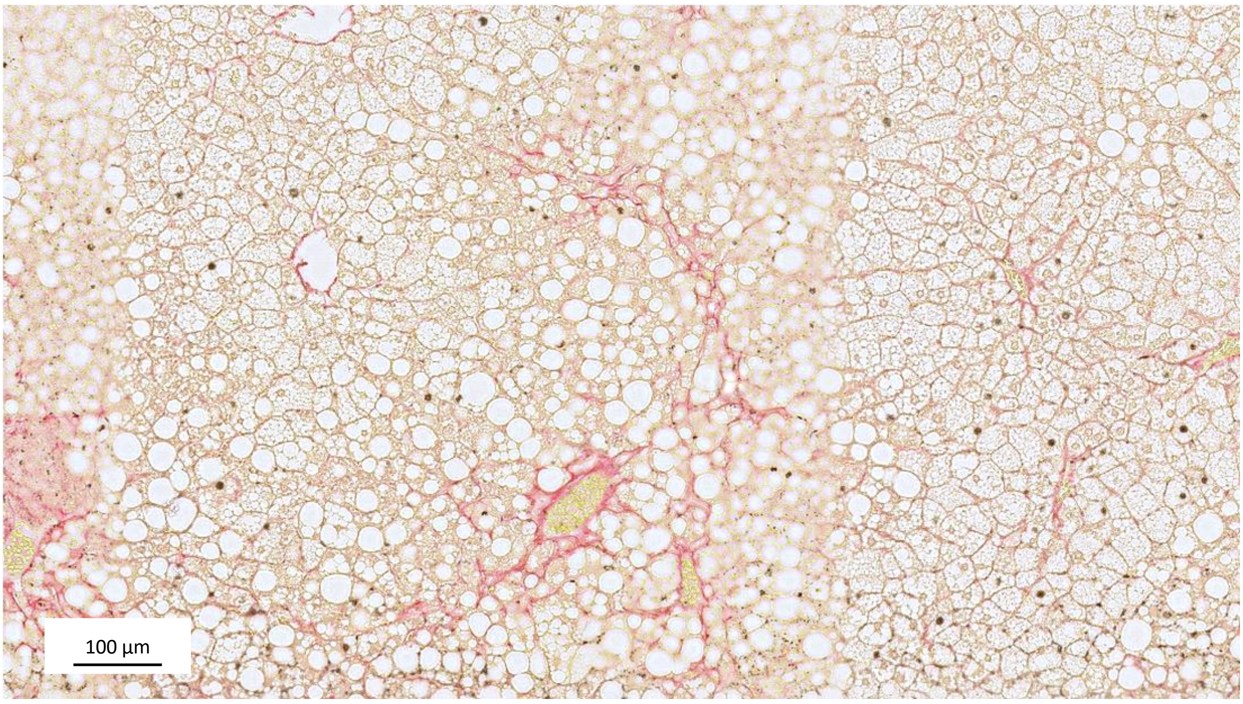

CDHFD-2

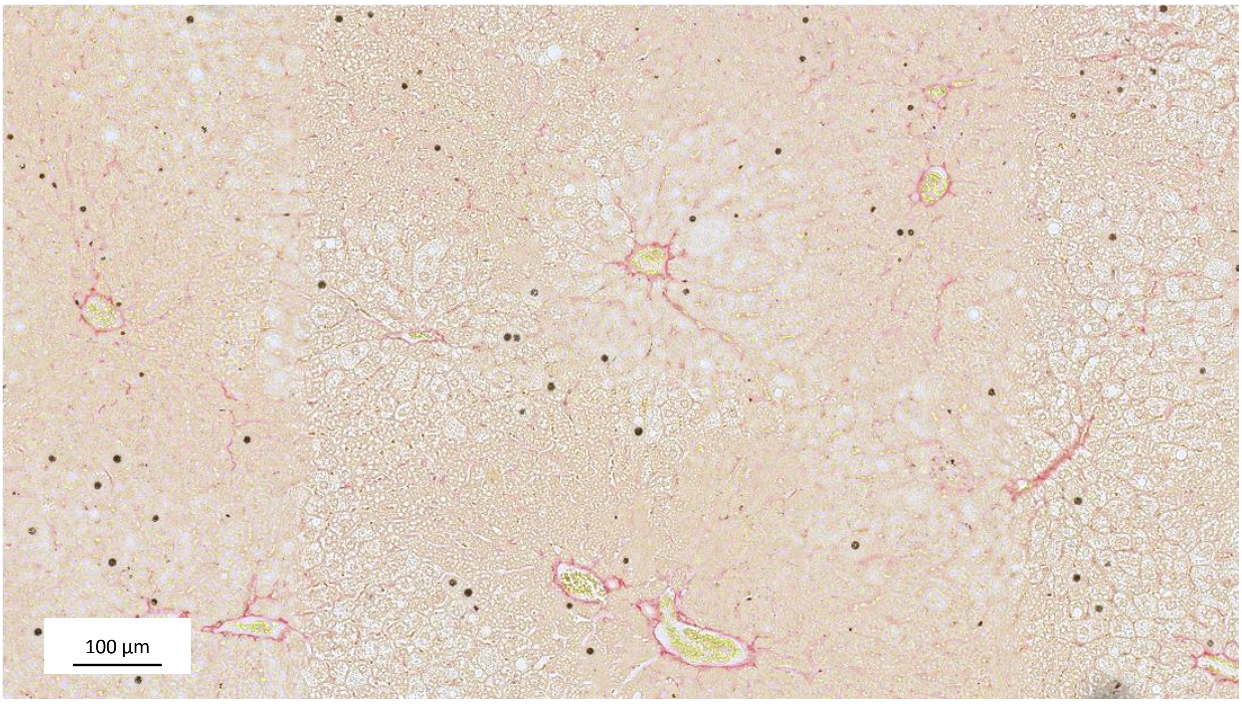

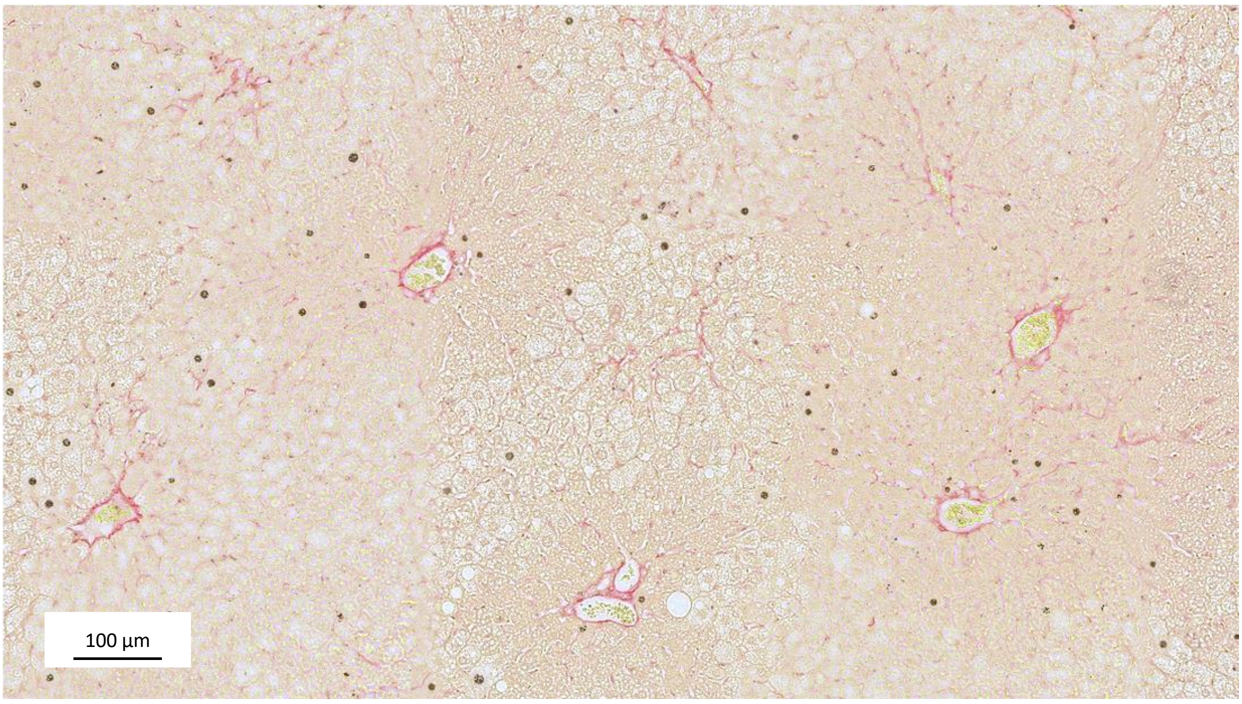

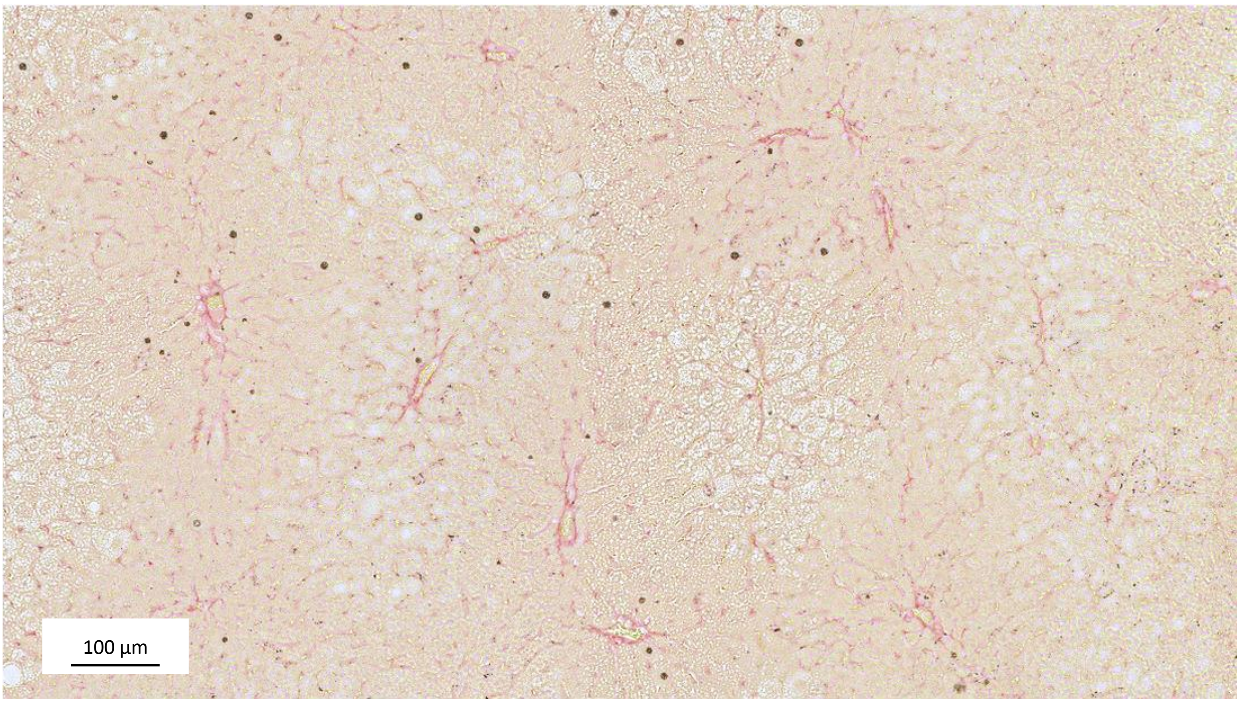

CDHFD-3

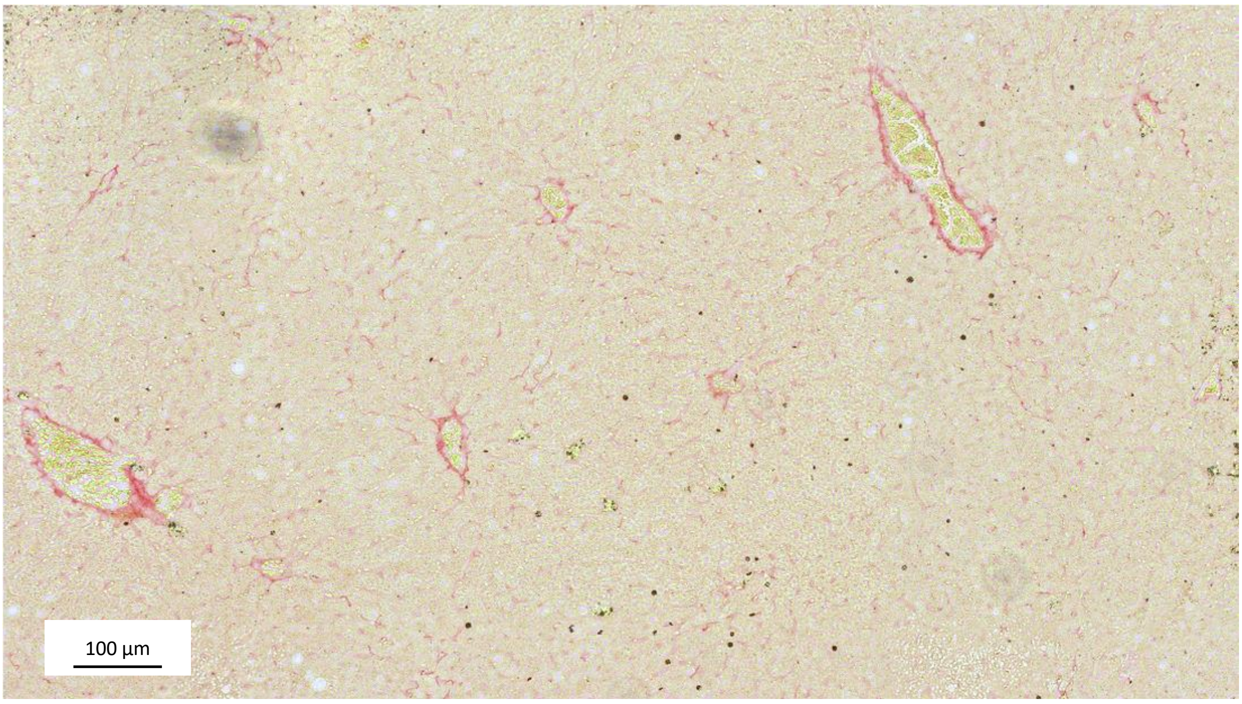

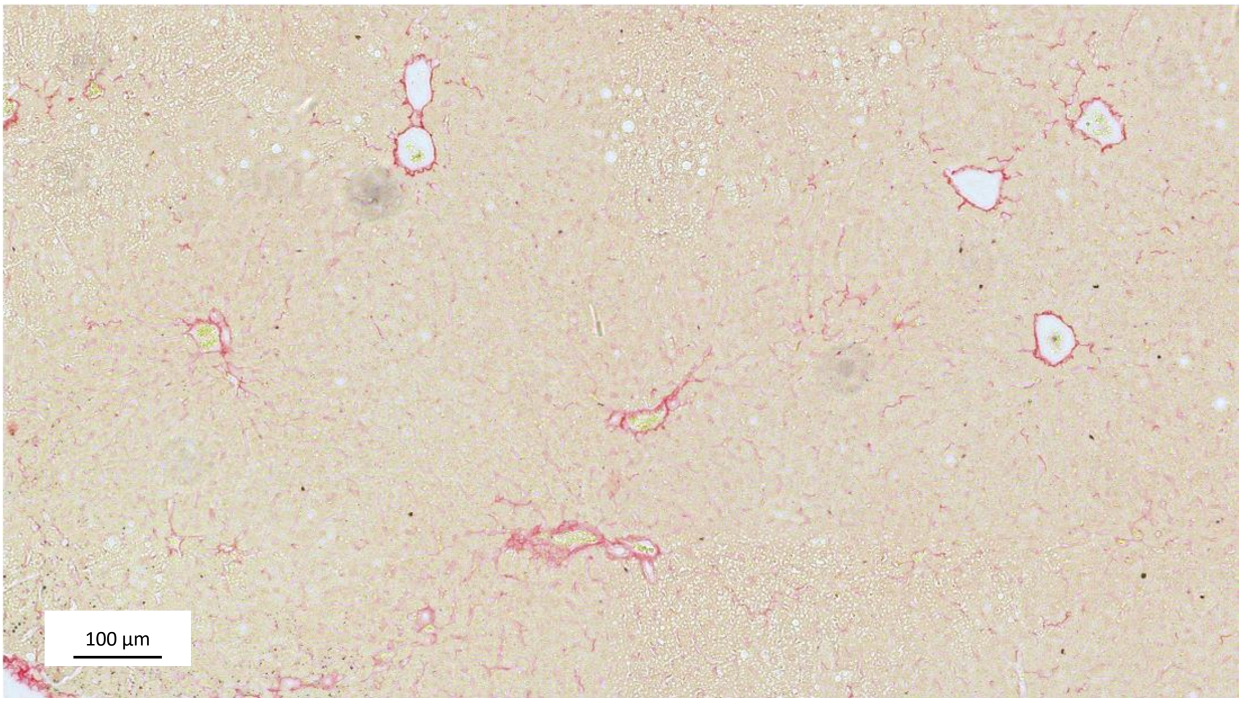

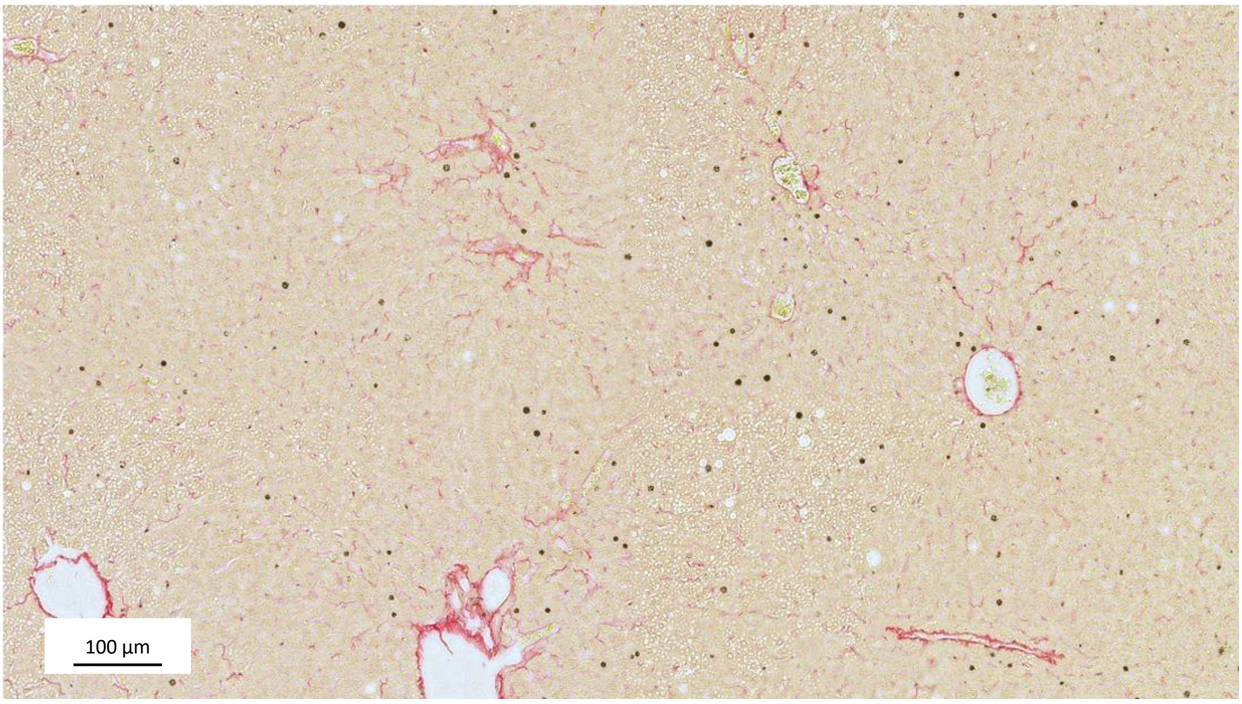

CDHFD-4

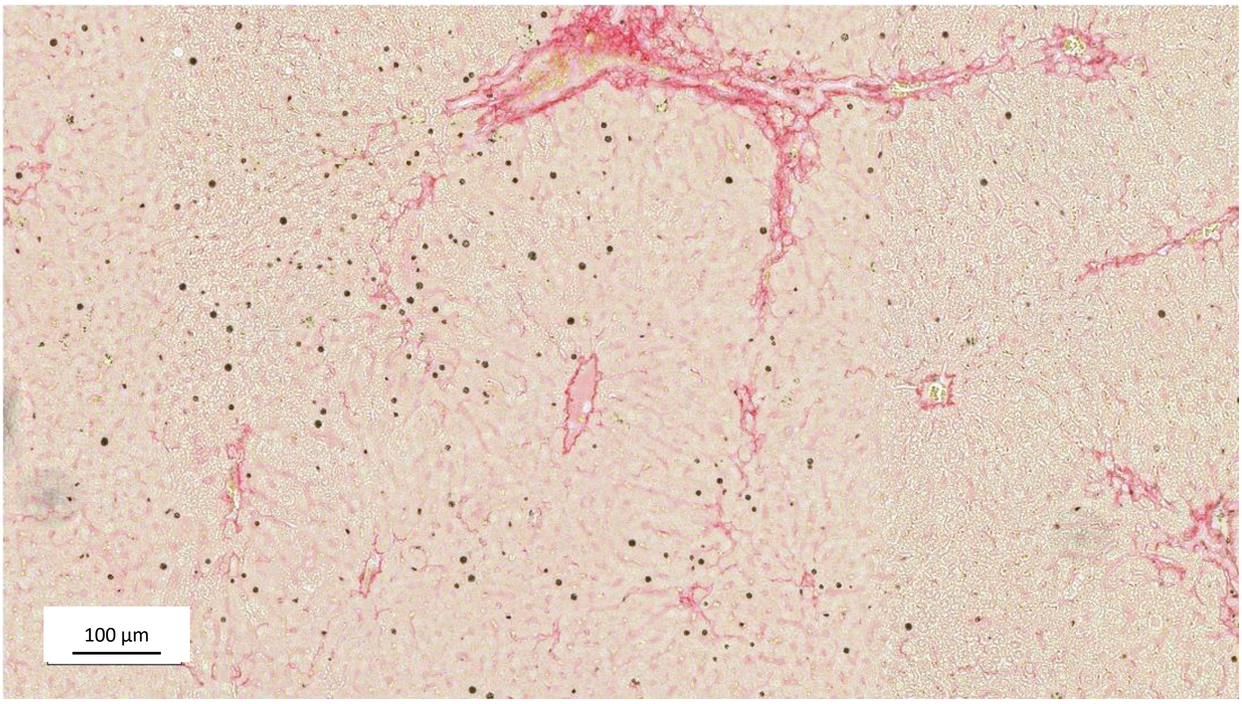

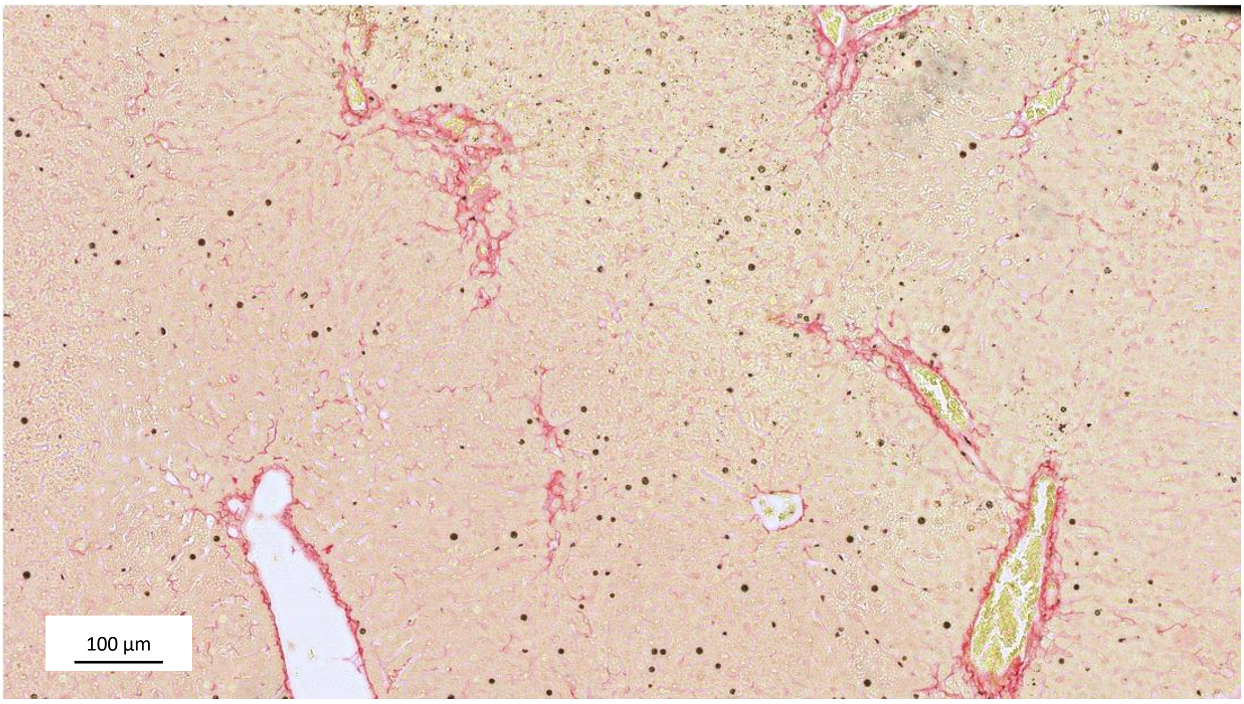

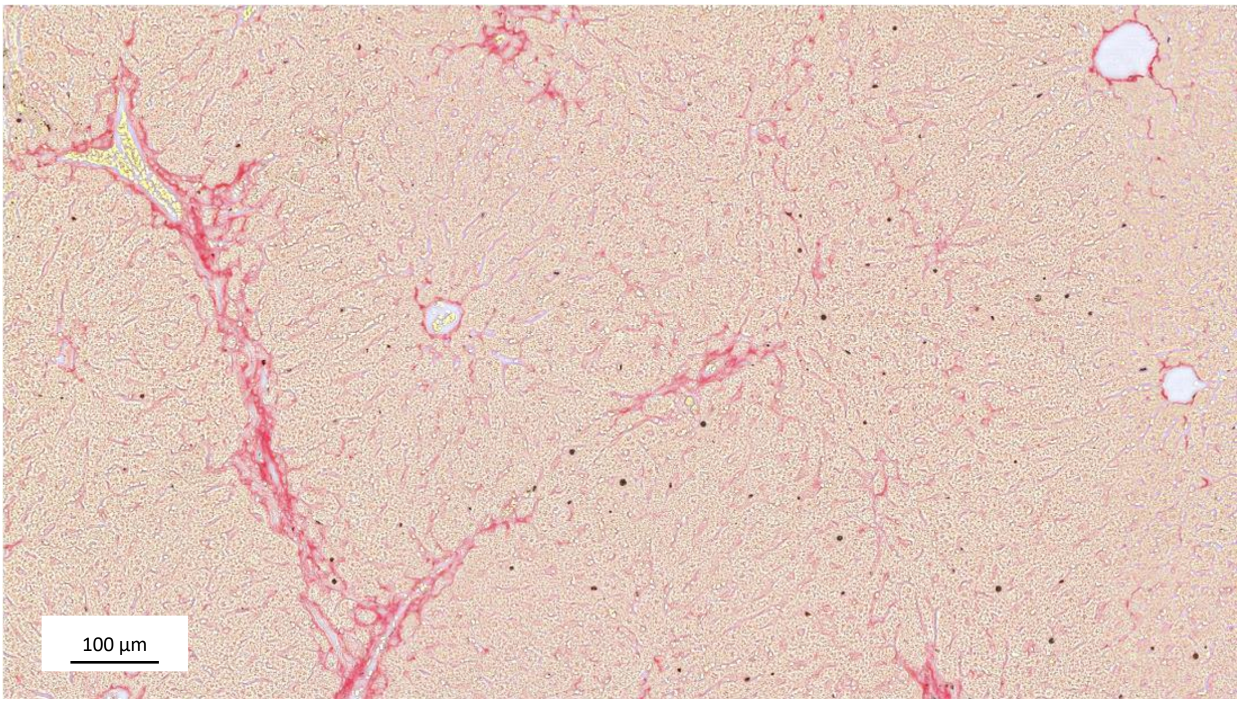

CDHFD-5

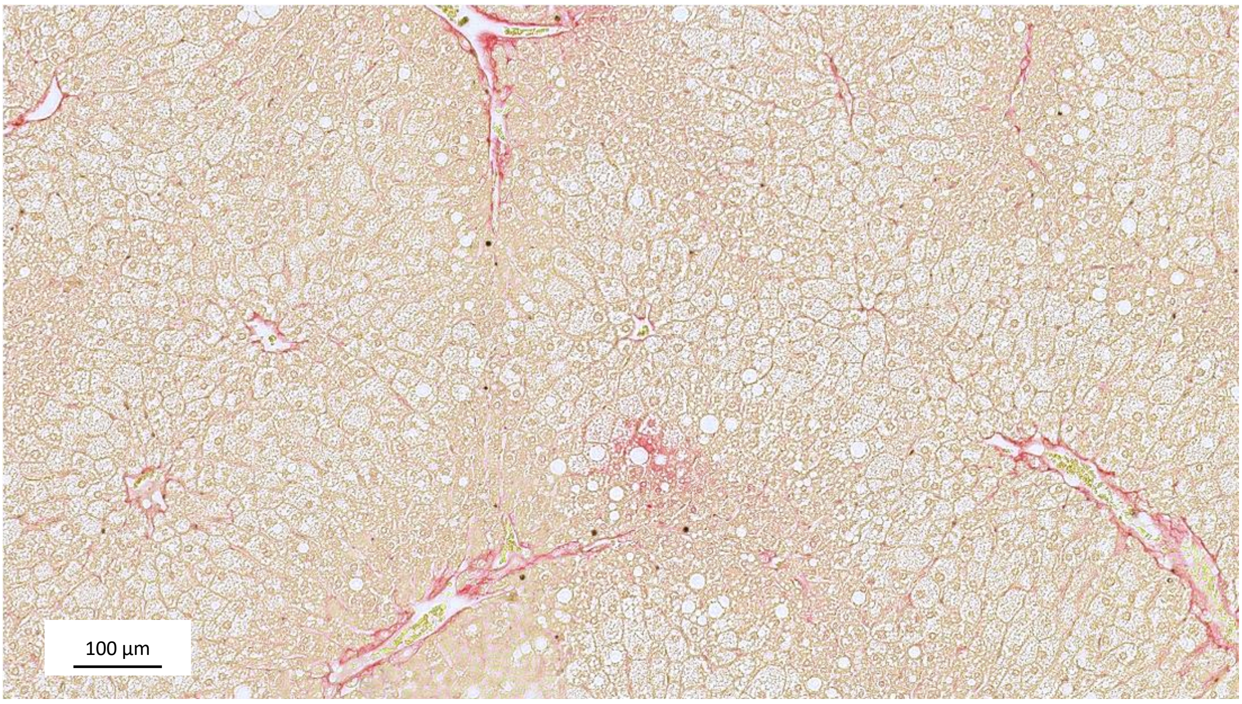

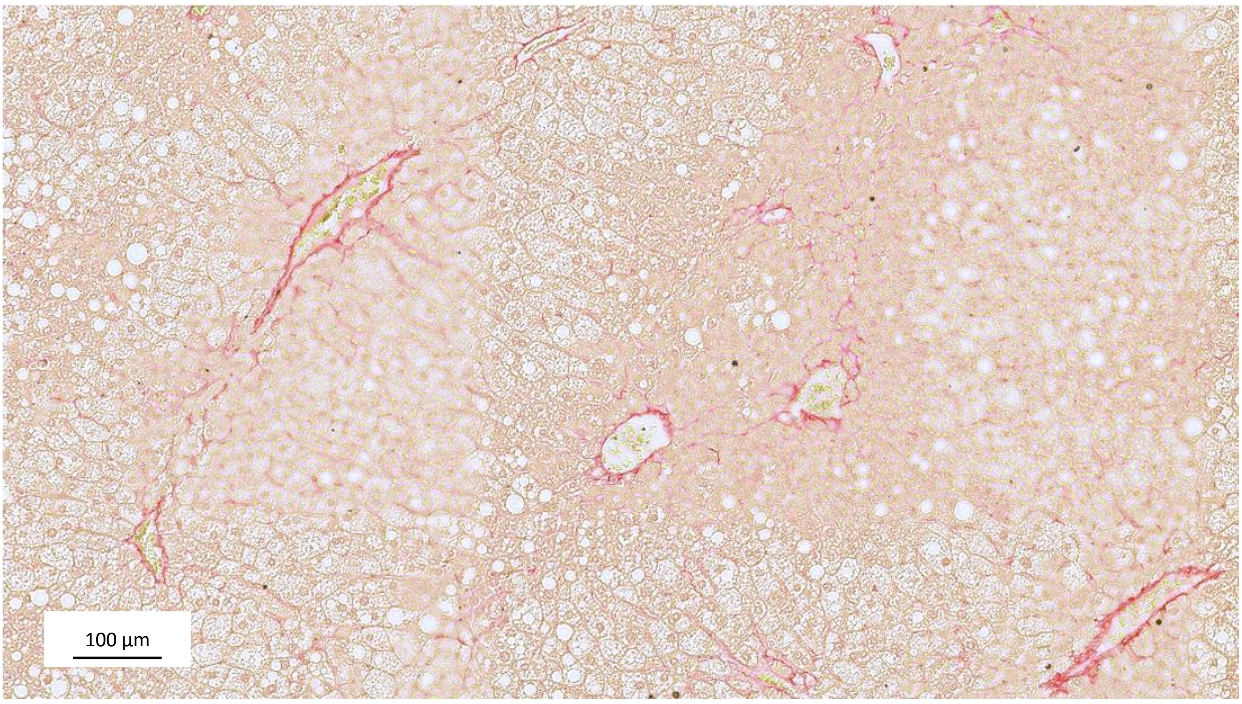

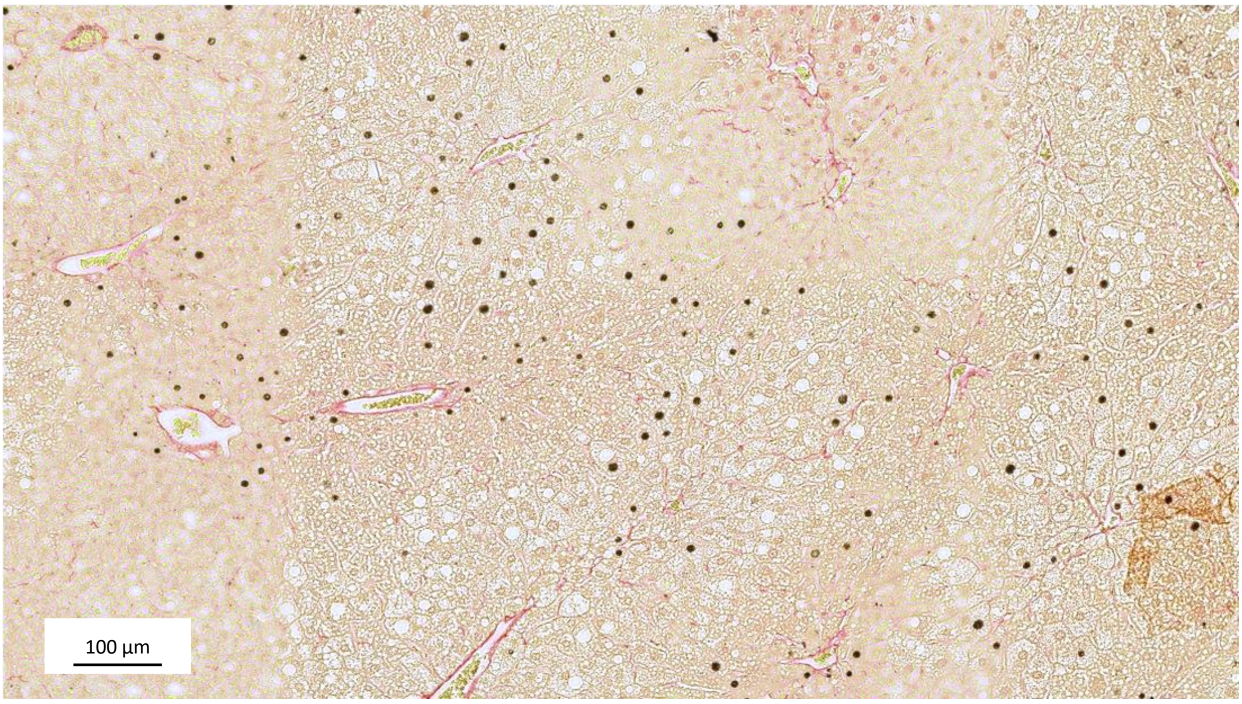

CDHFD-6

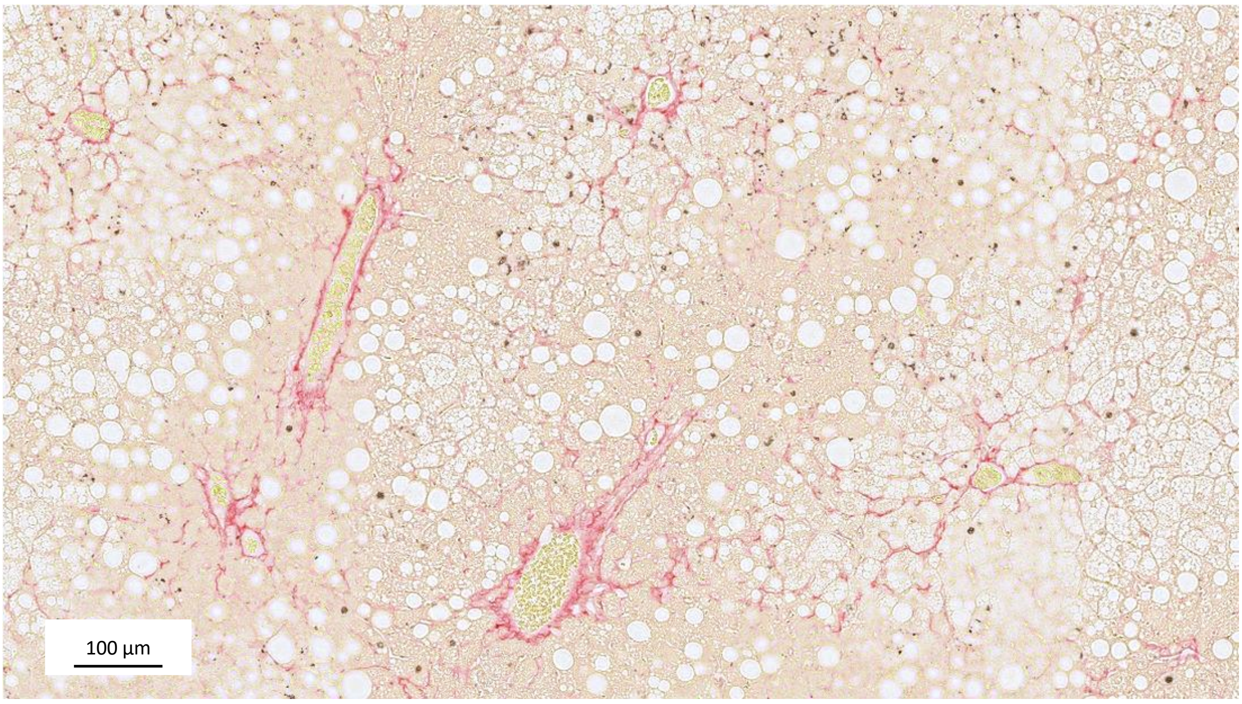

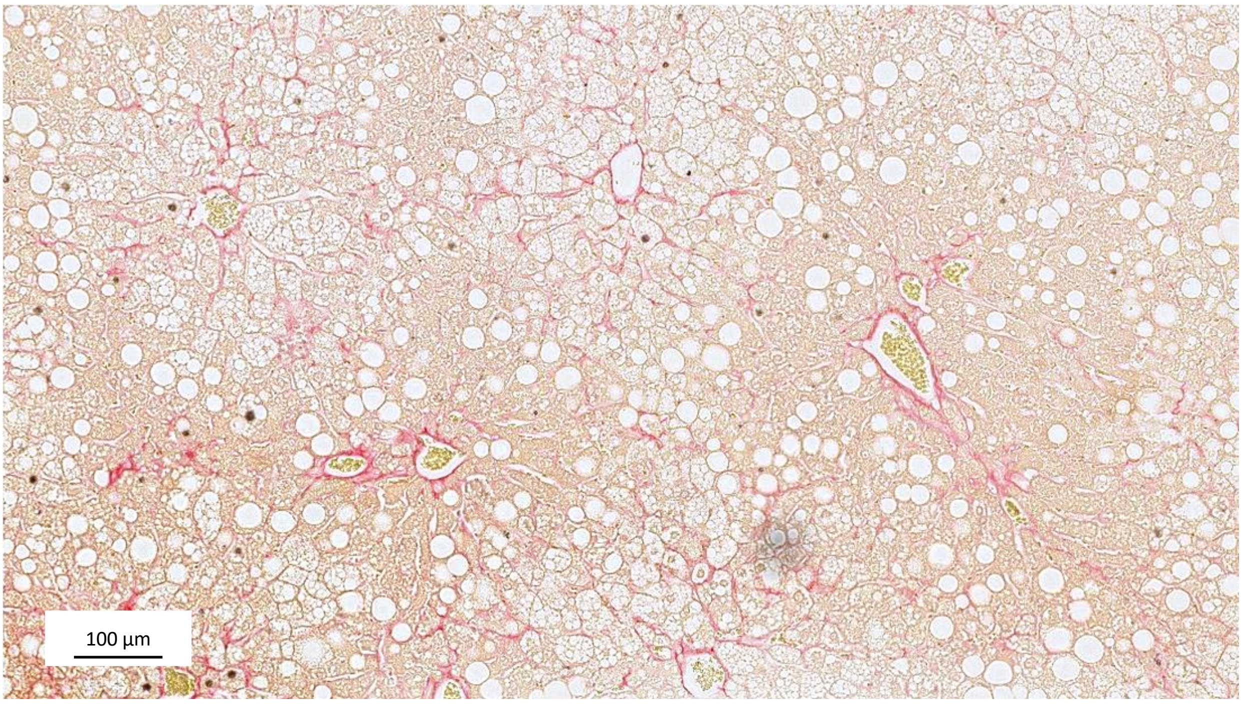

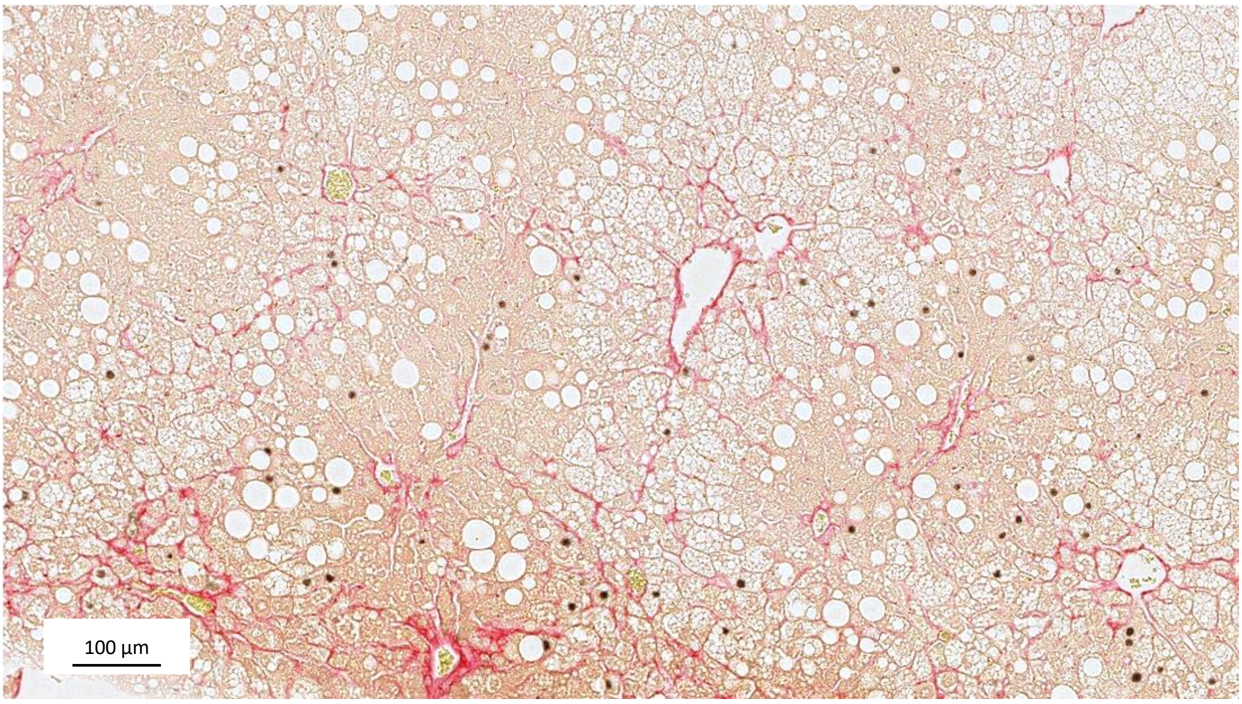

CDHFD-7

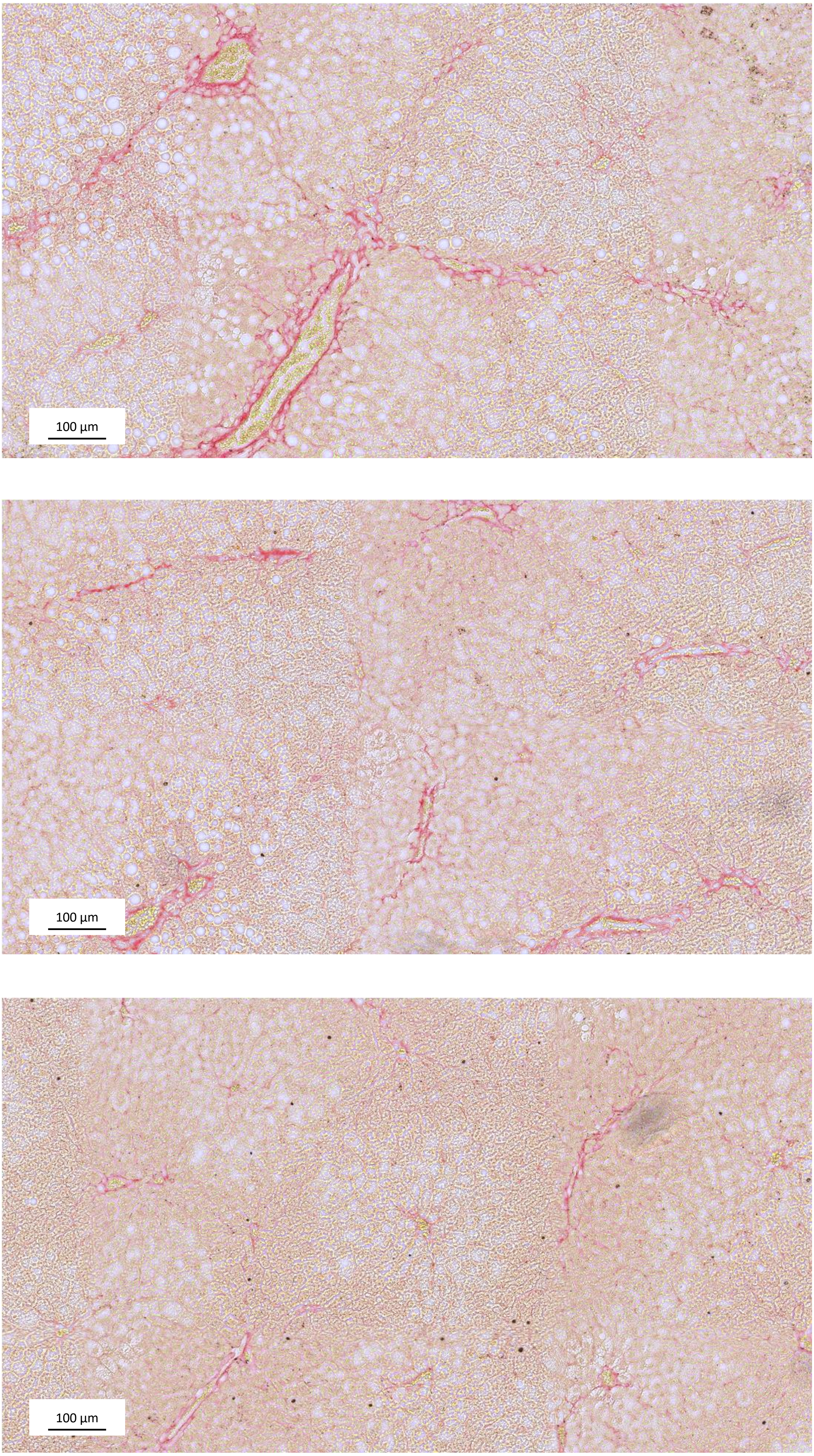

CDHFD-8

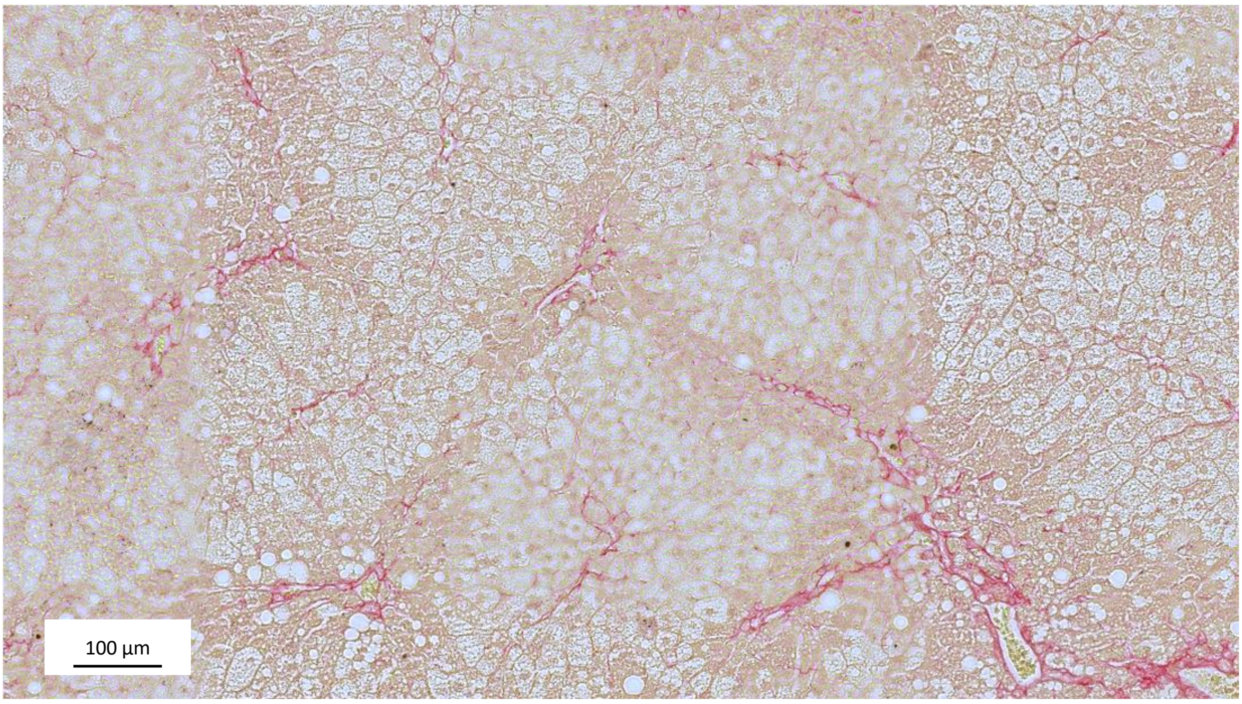

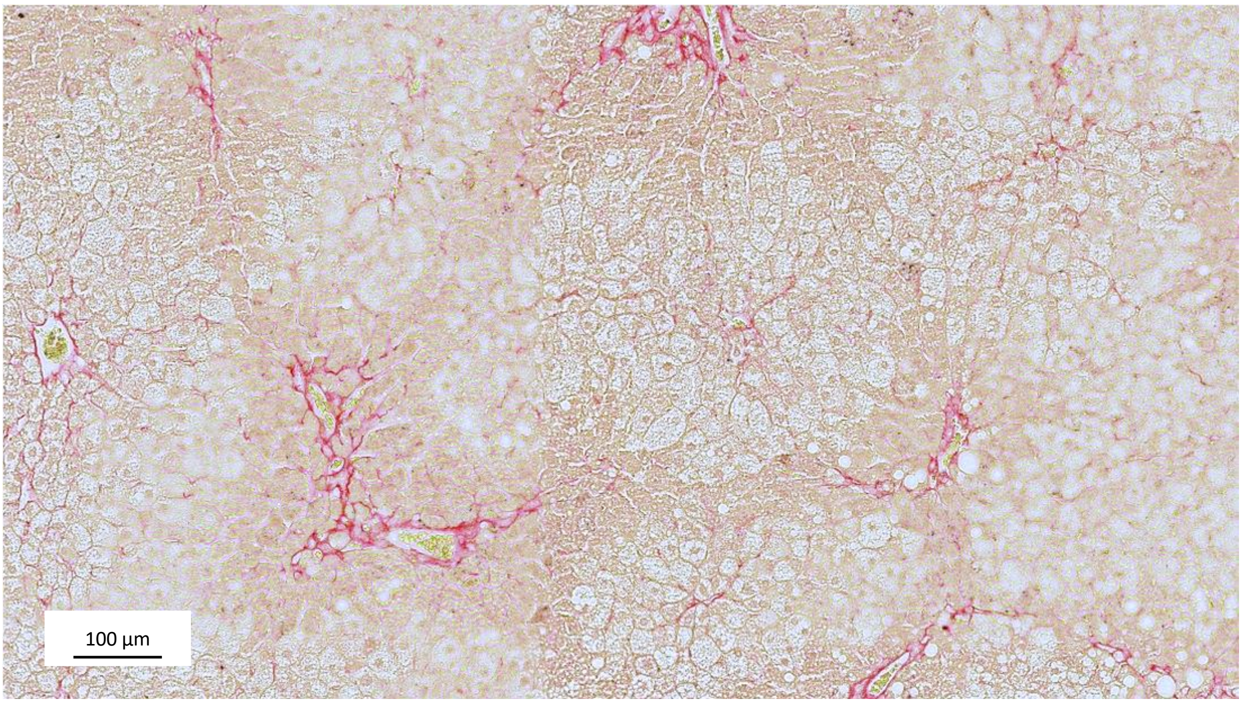

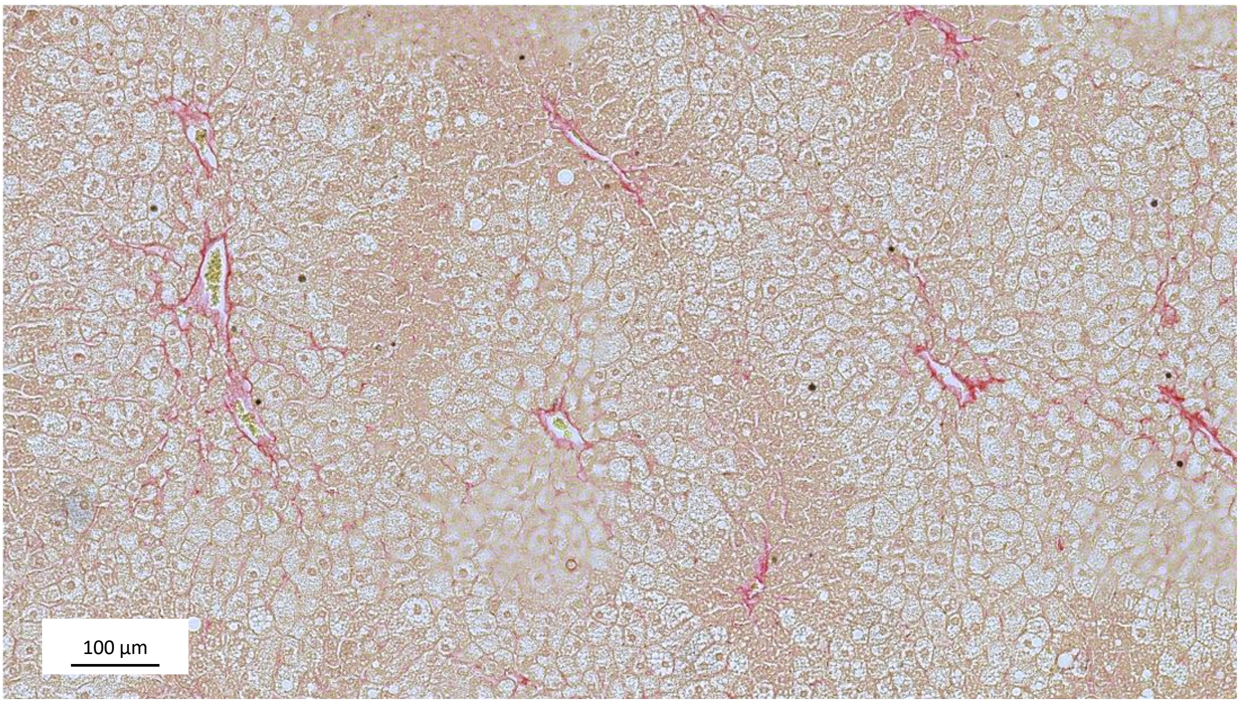

CDHFD-9

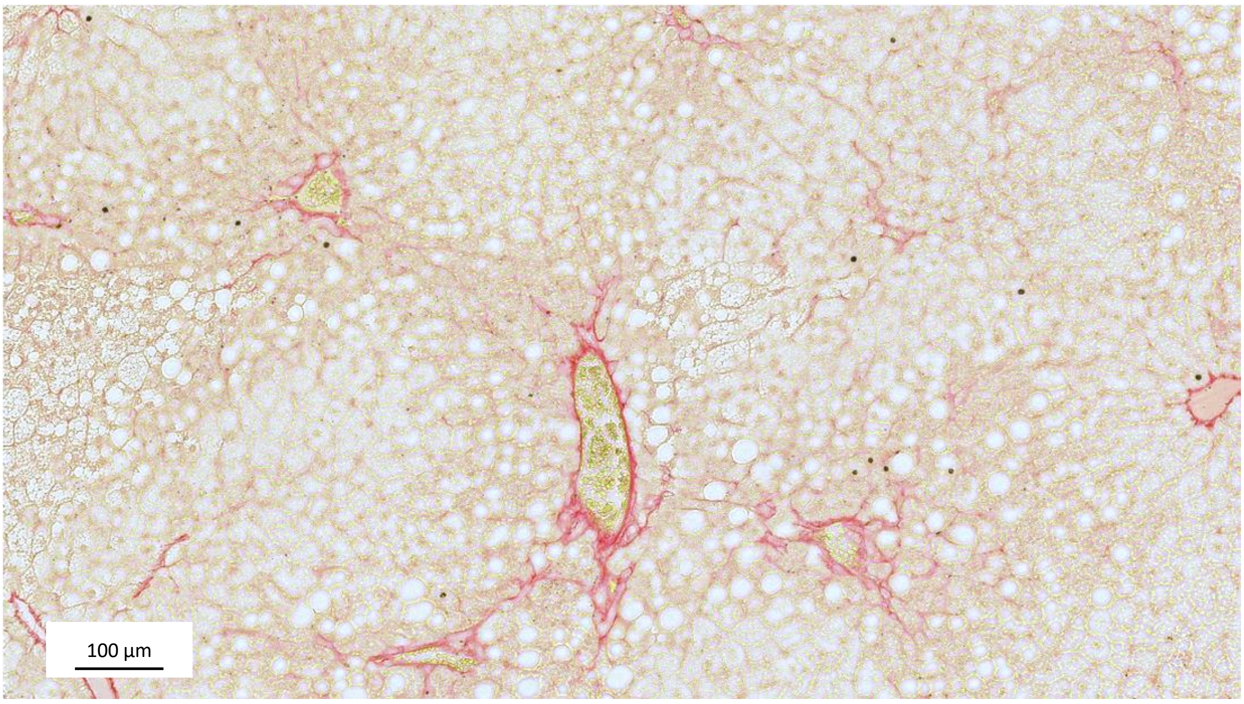

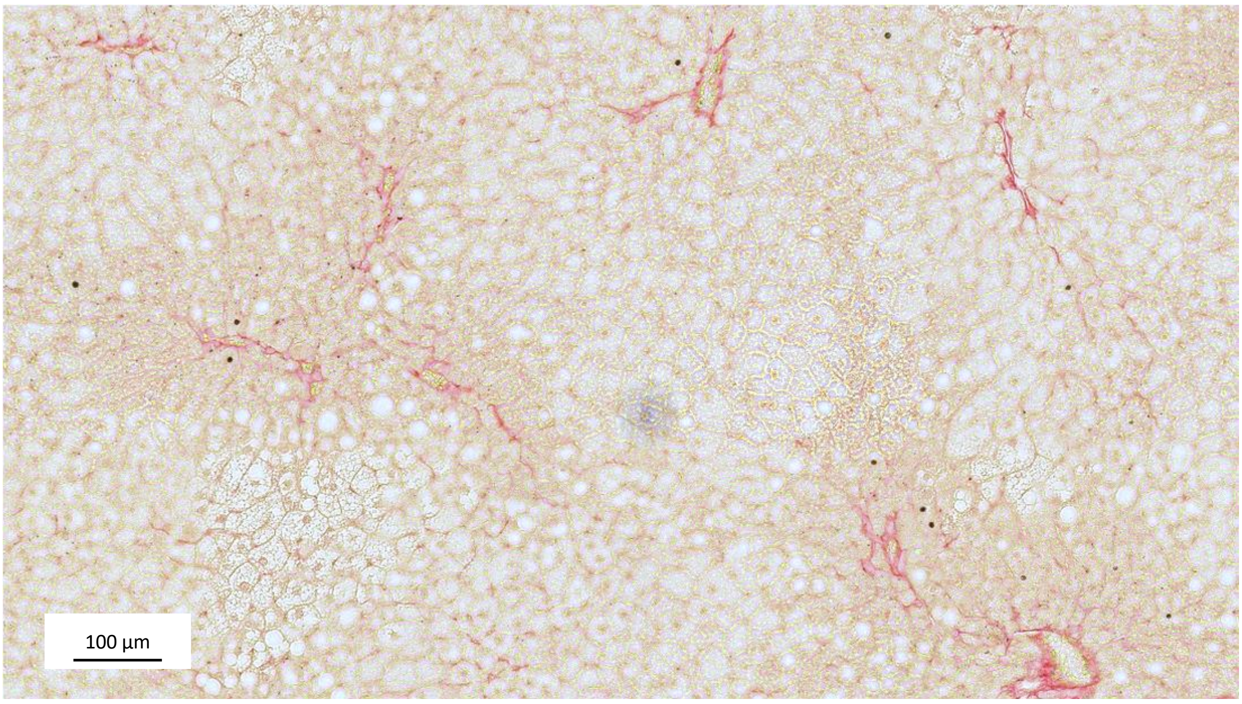

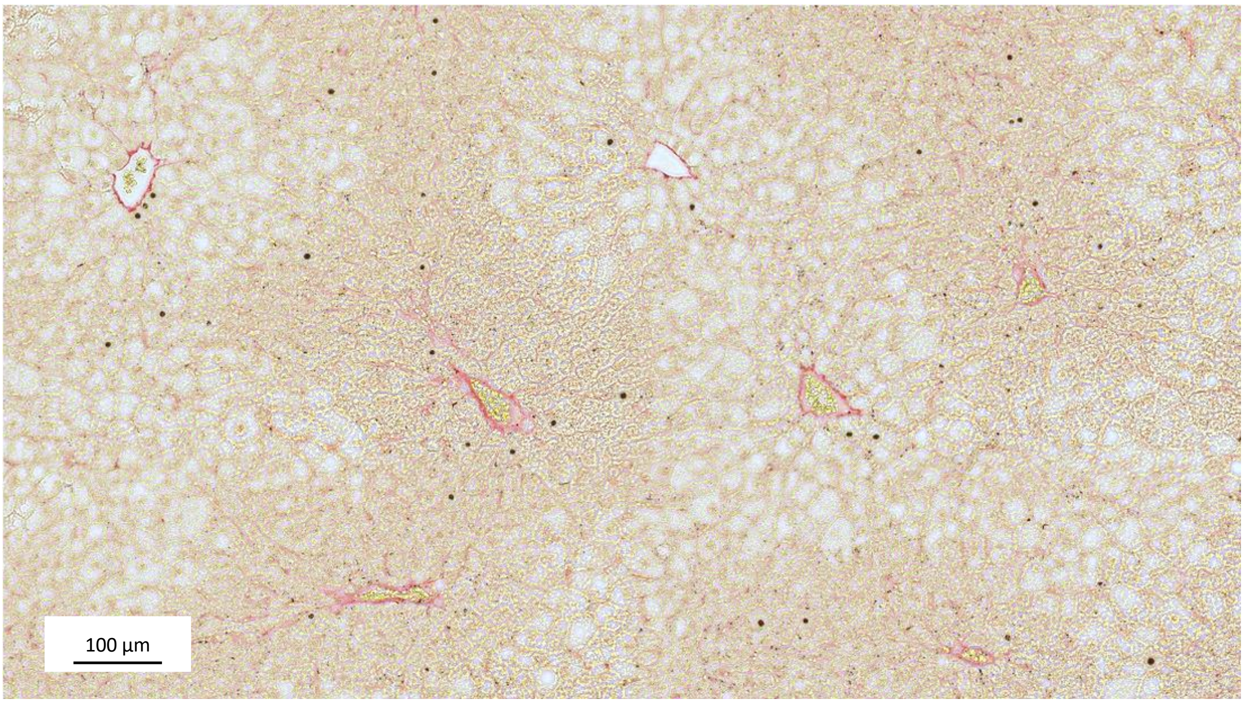

CDHFD-10

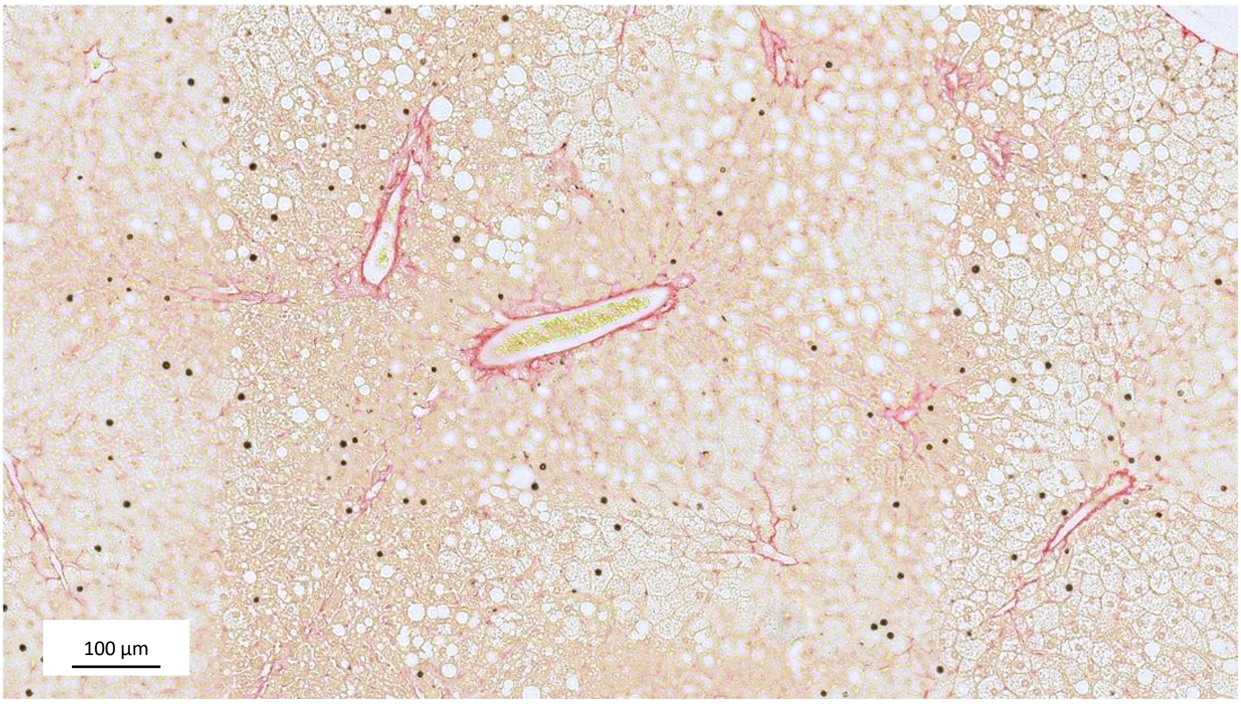

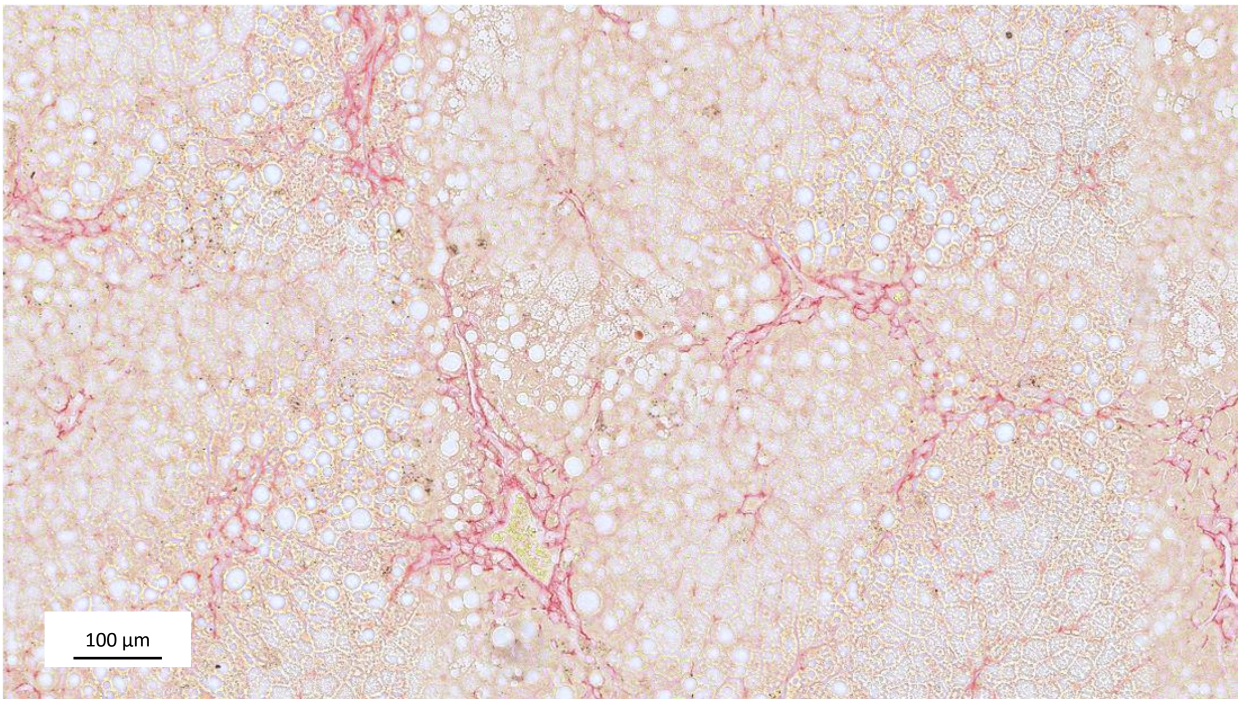

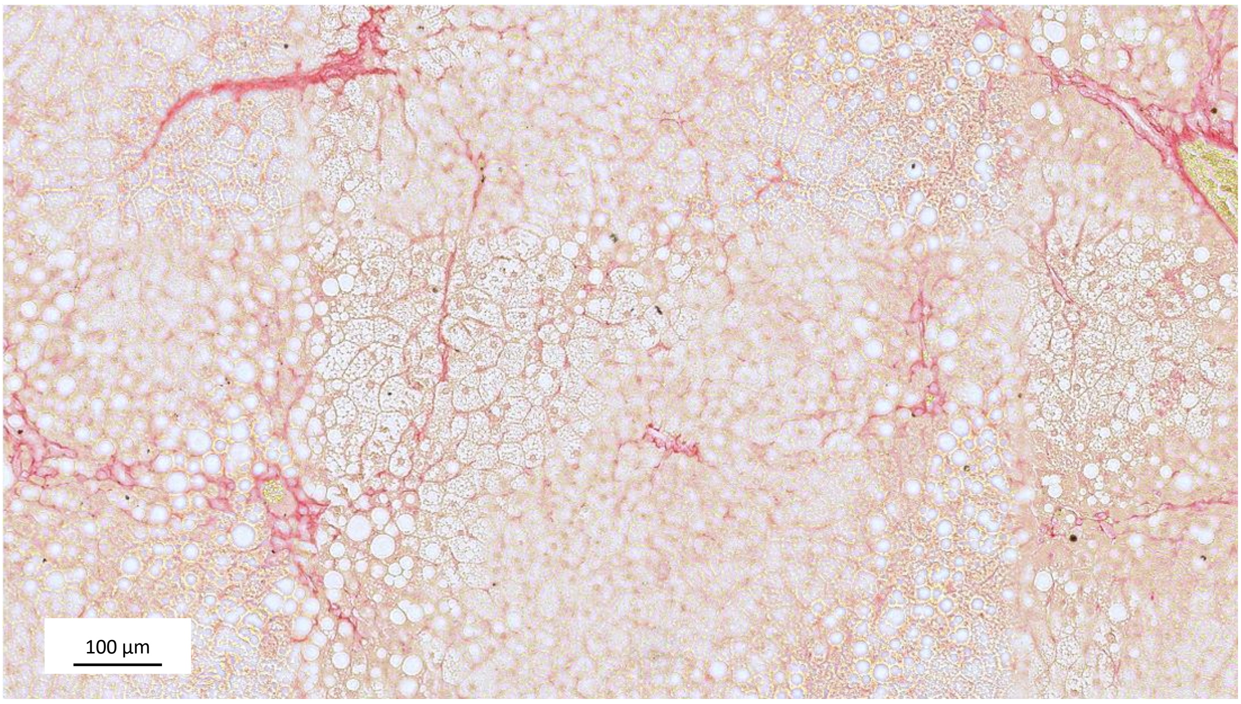

CDHFD-11

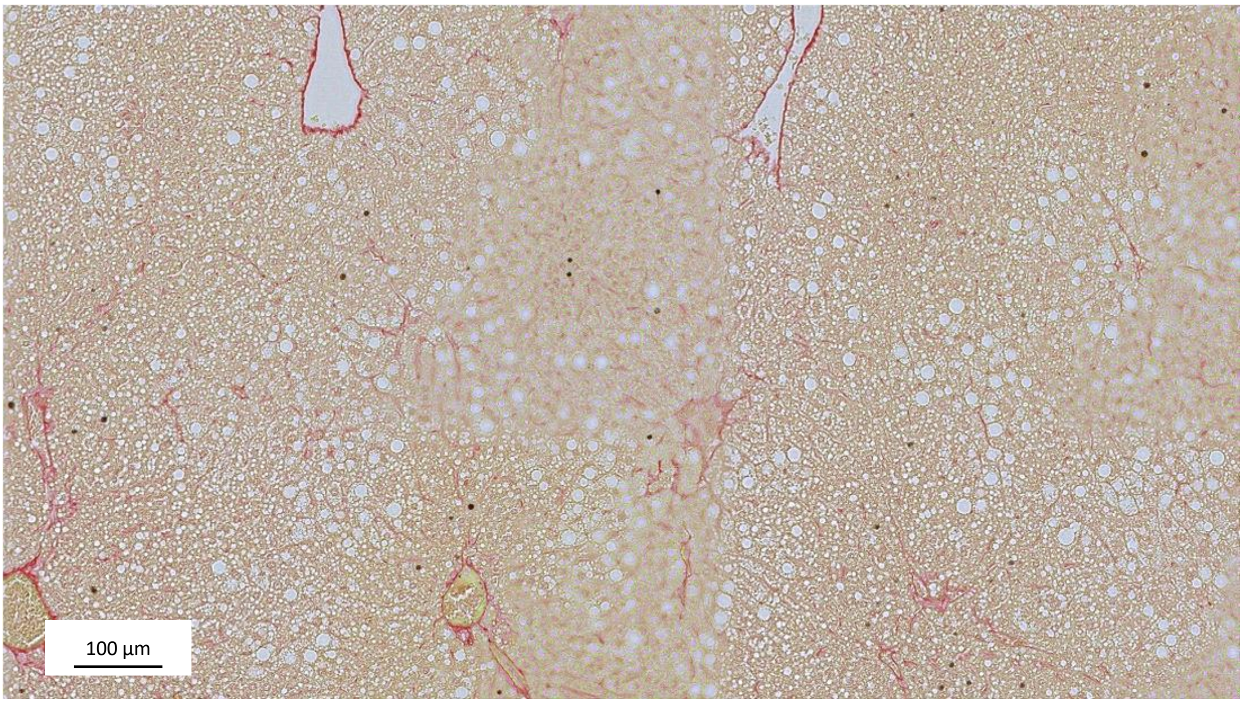

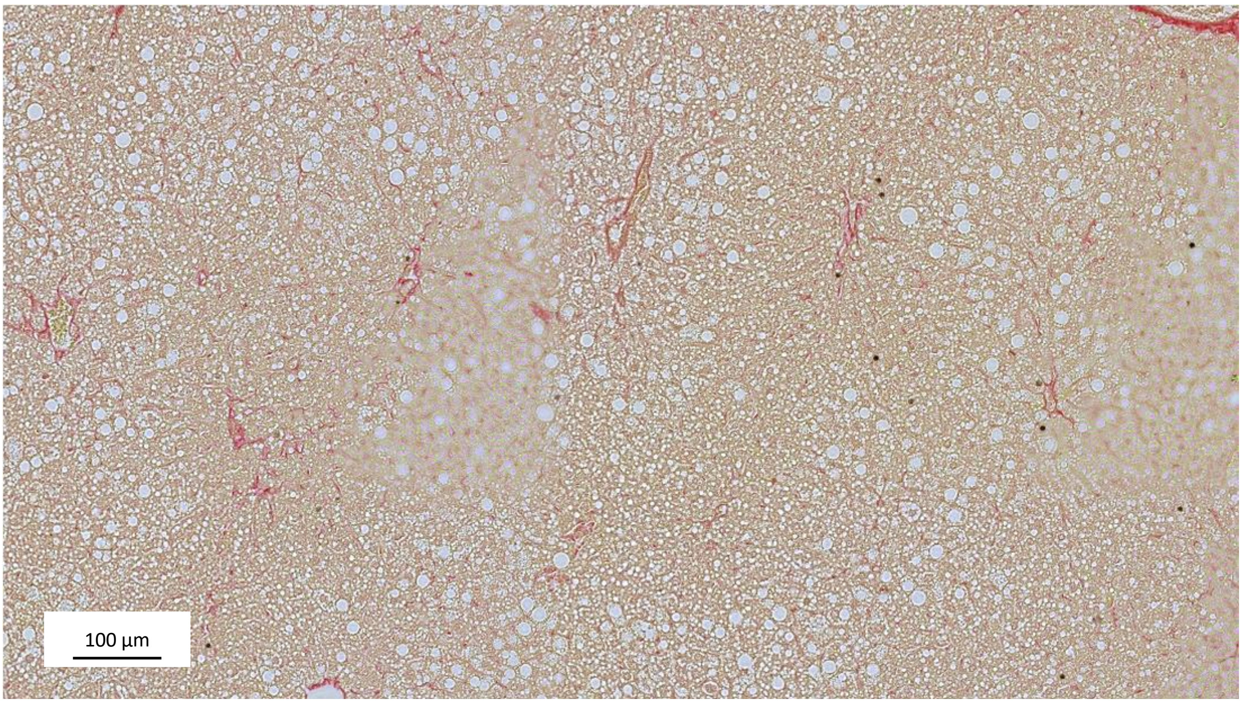

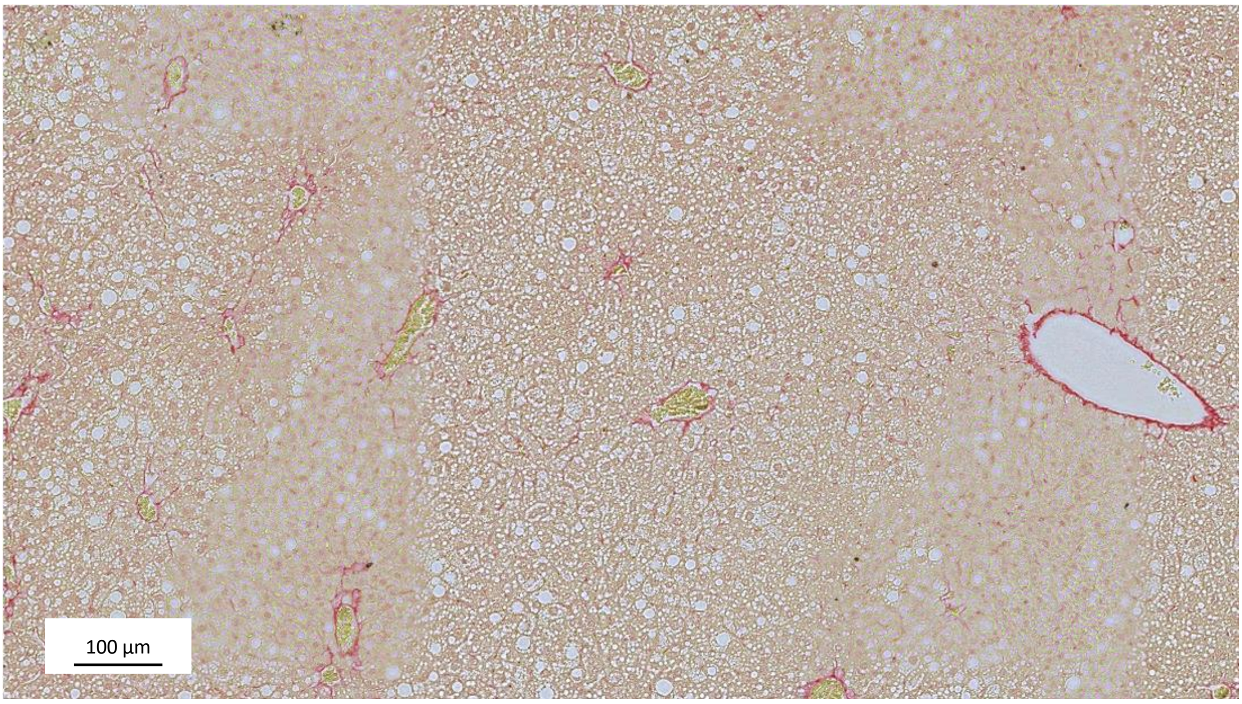

CDHFD-12

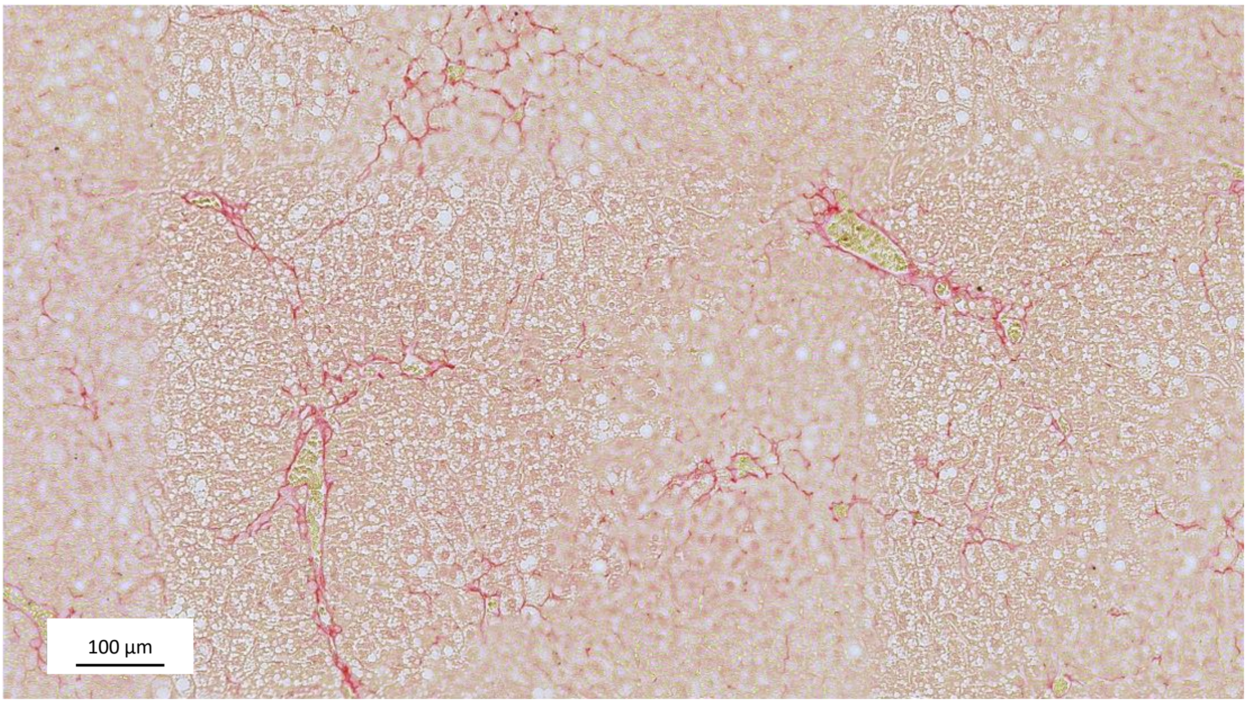

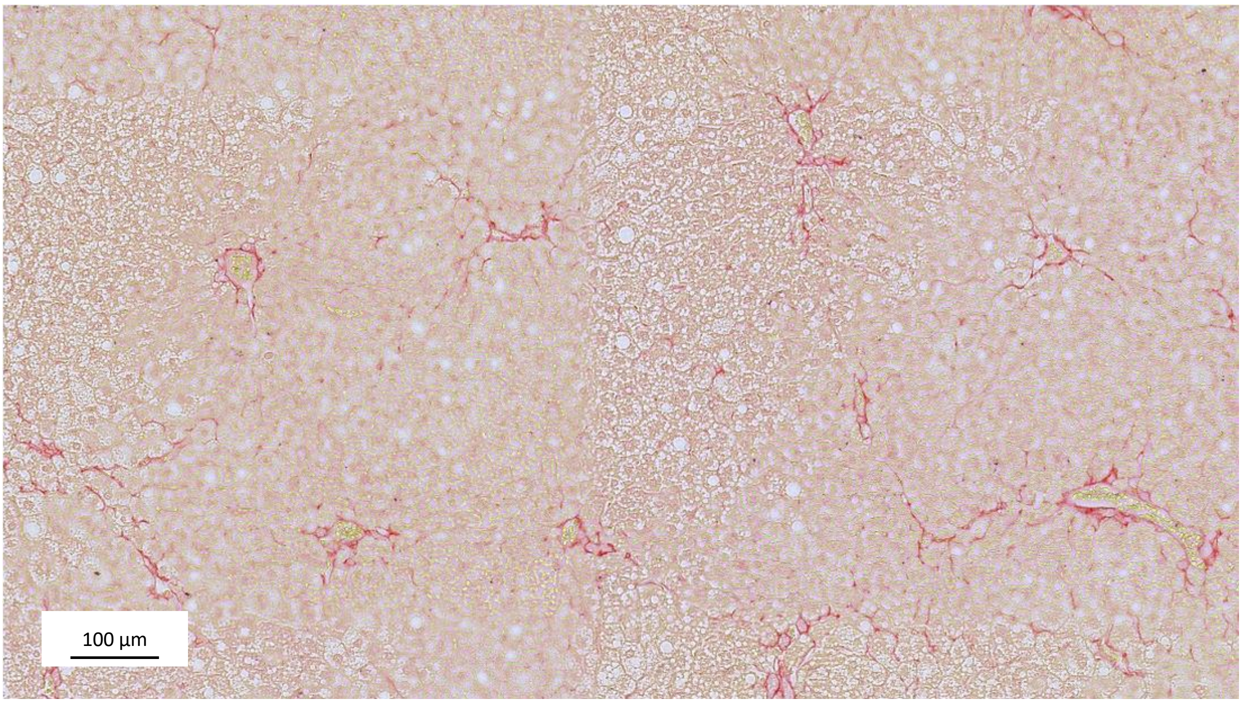

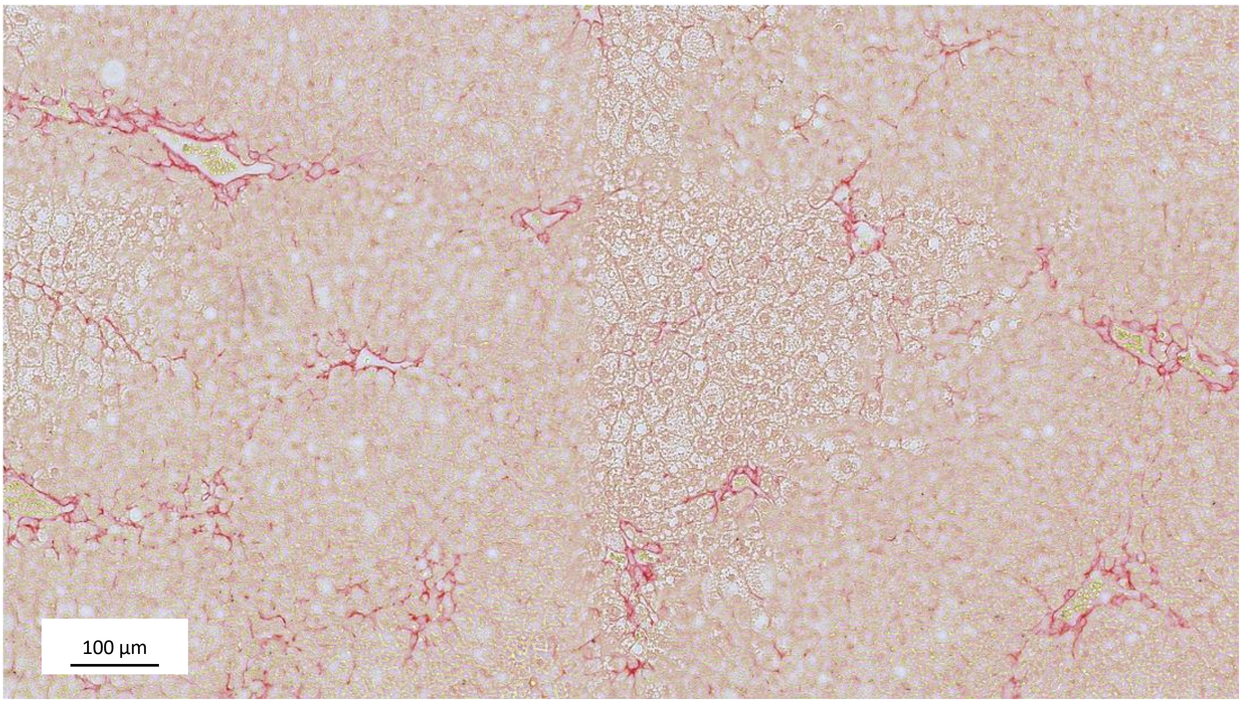

CDHFD-13

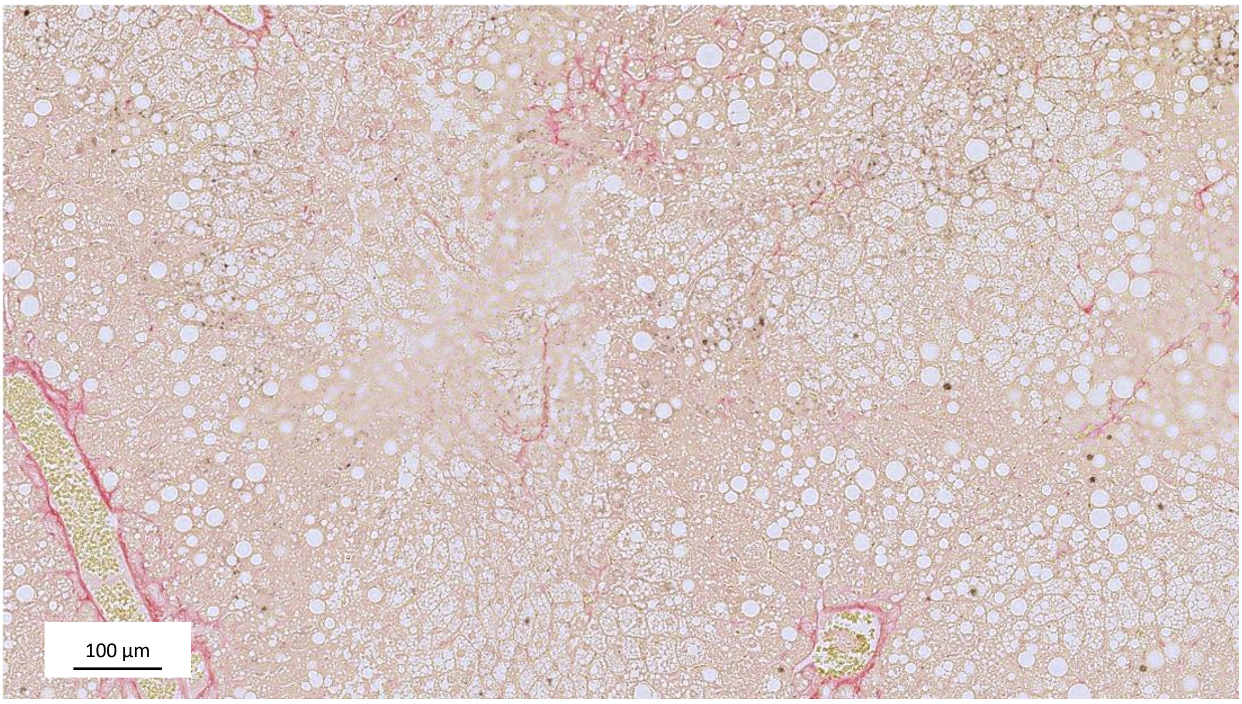

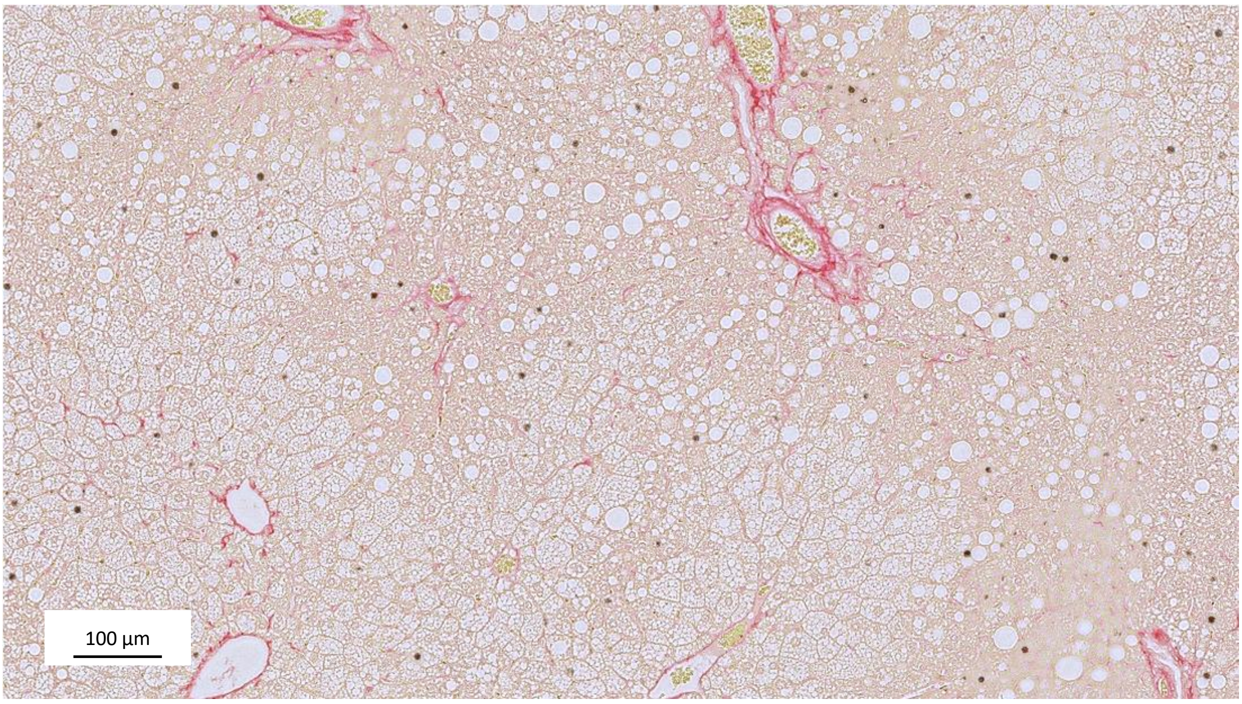

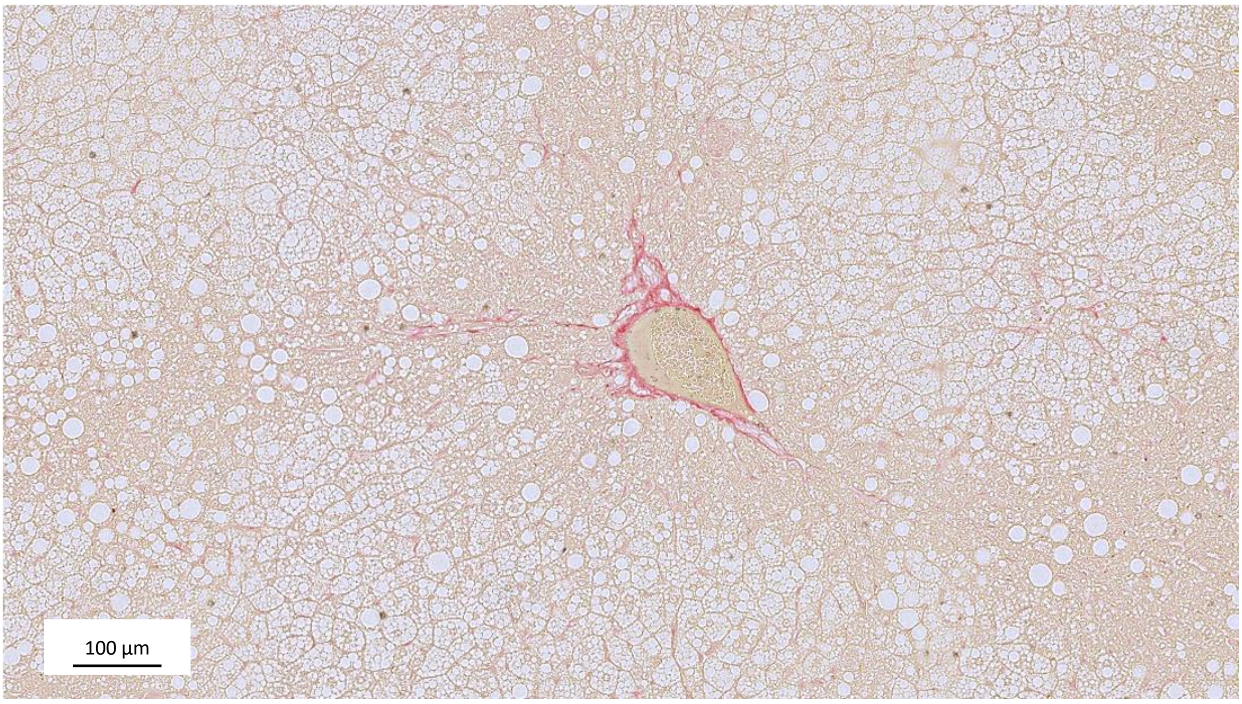

Sirius red Staining

CDHFD-I group

(15 mice were included)

CDHFD-I-1

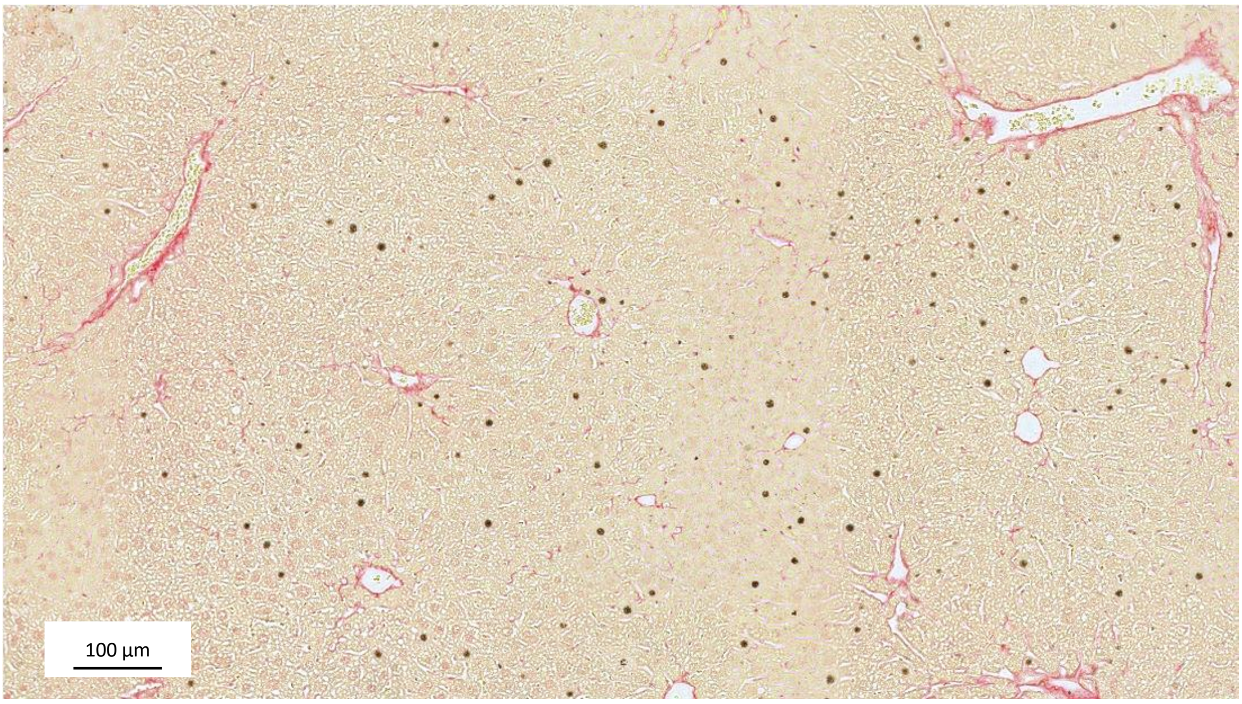

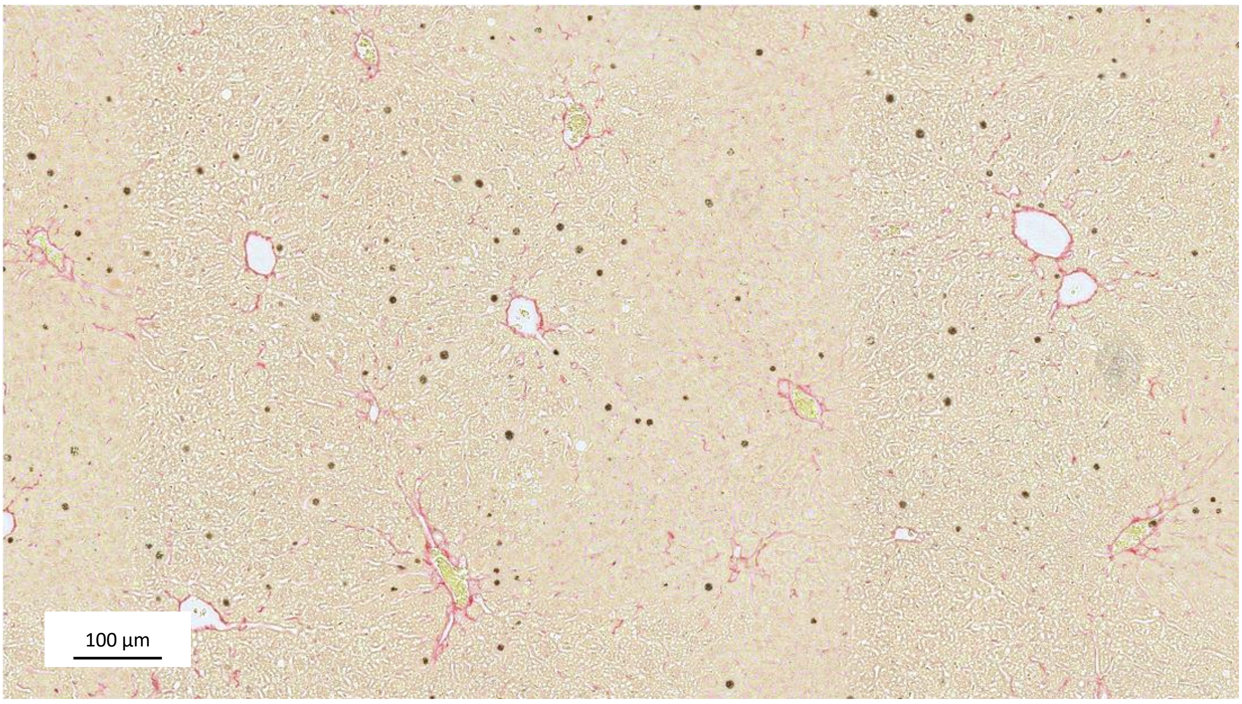

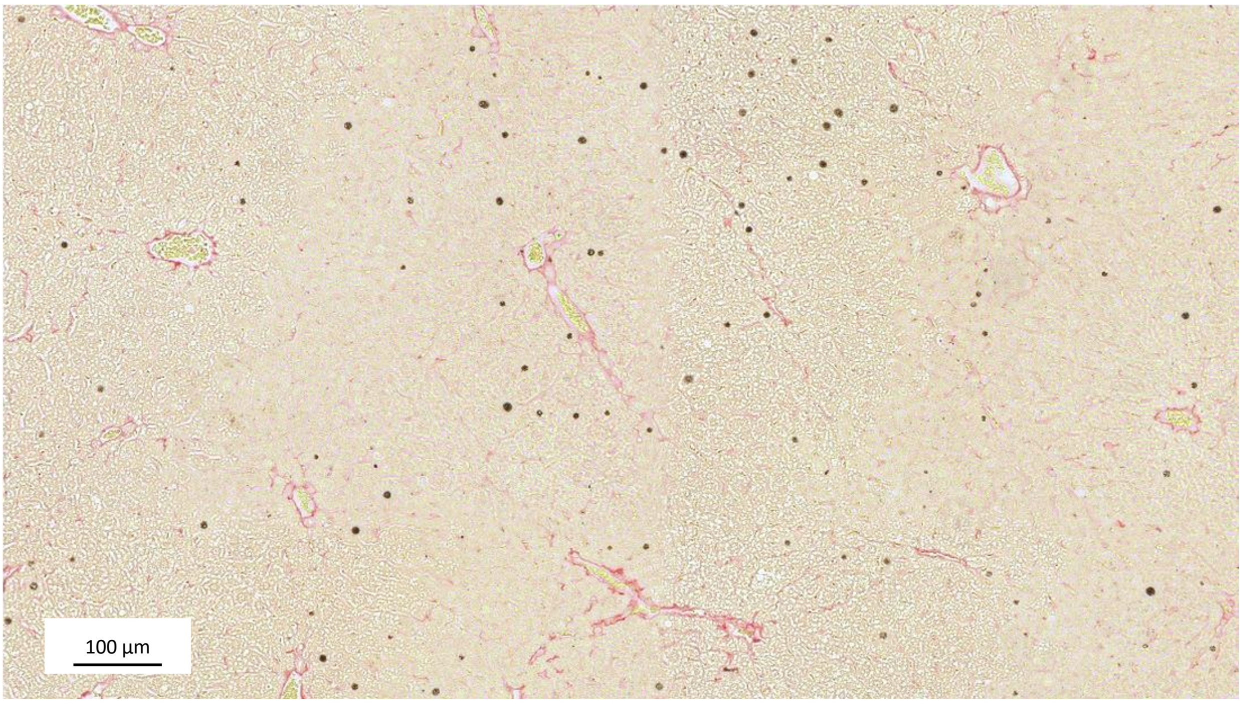

CDHFD-I-2

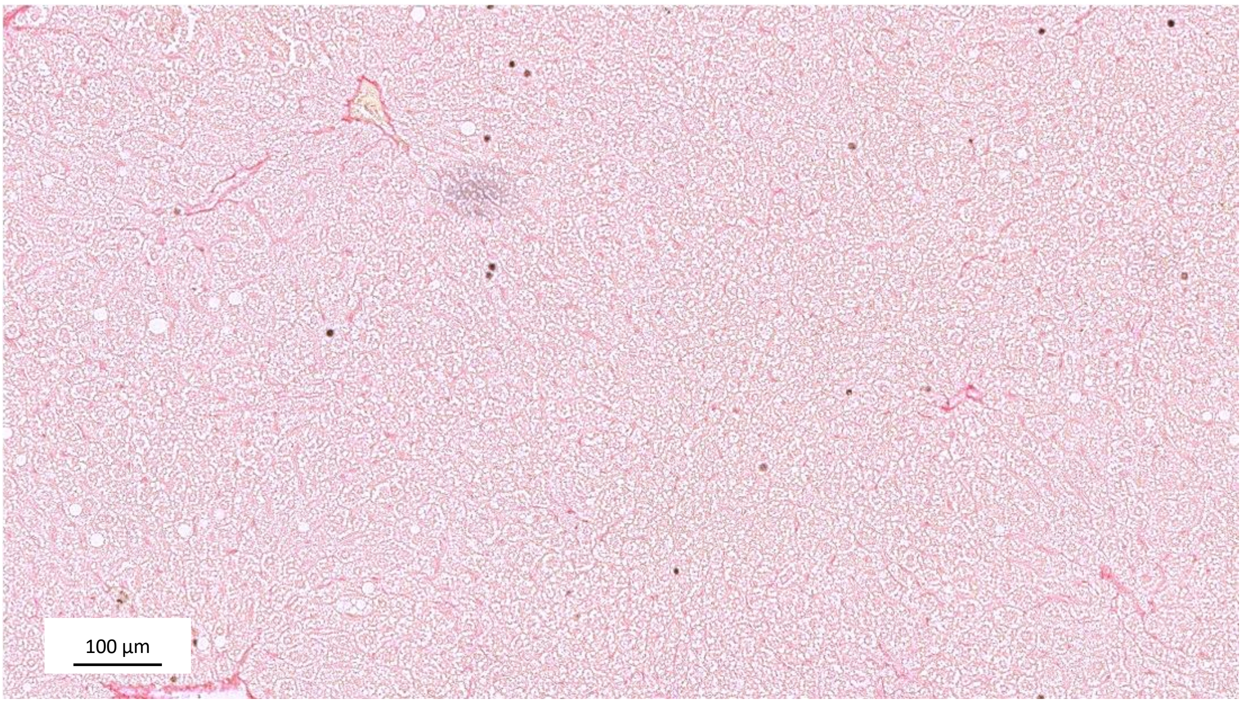

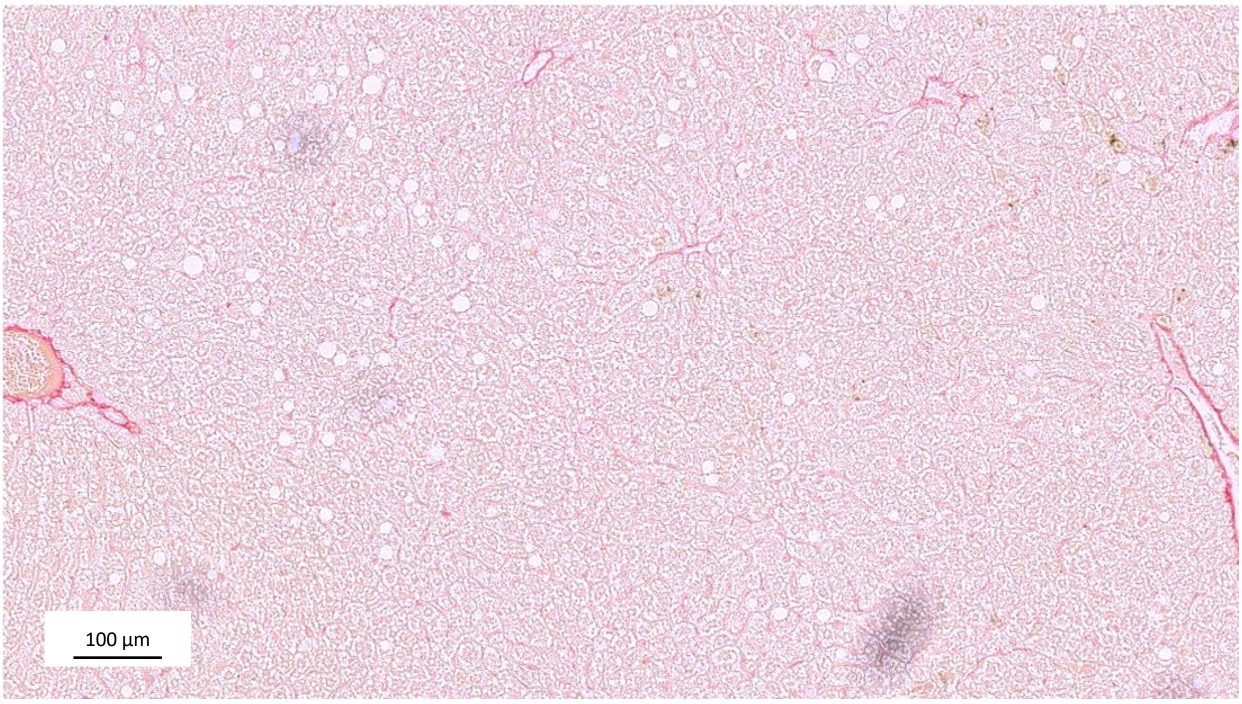

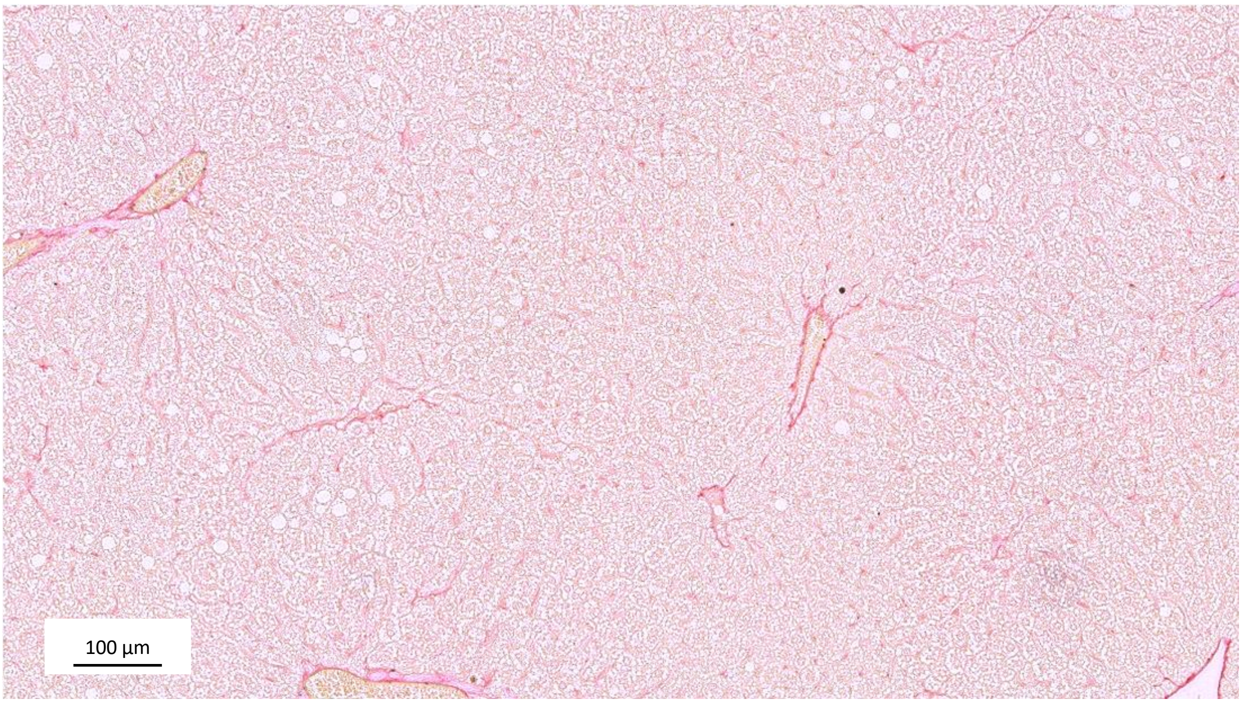

CDHFD-I-3

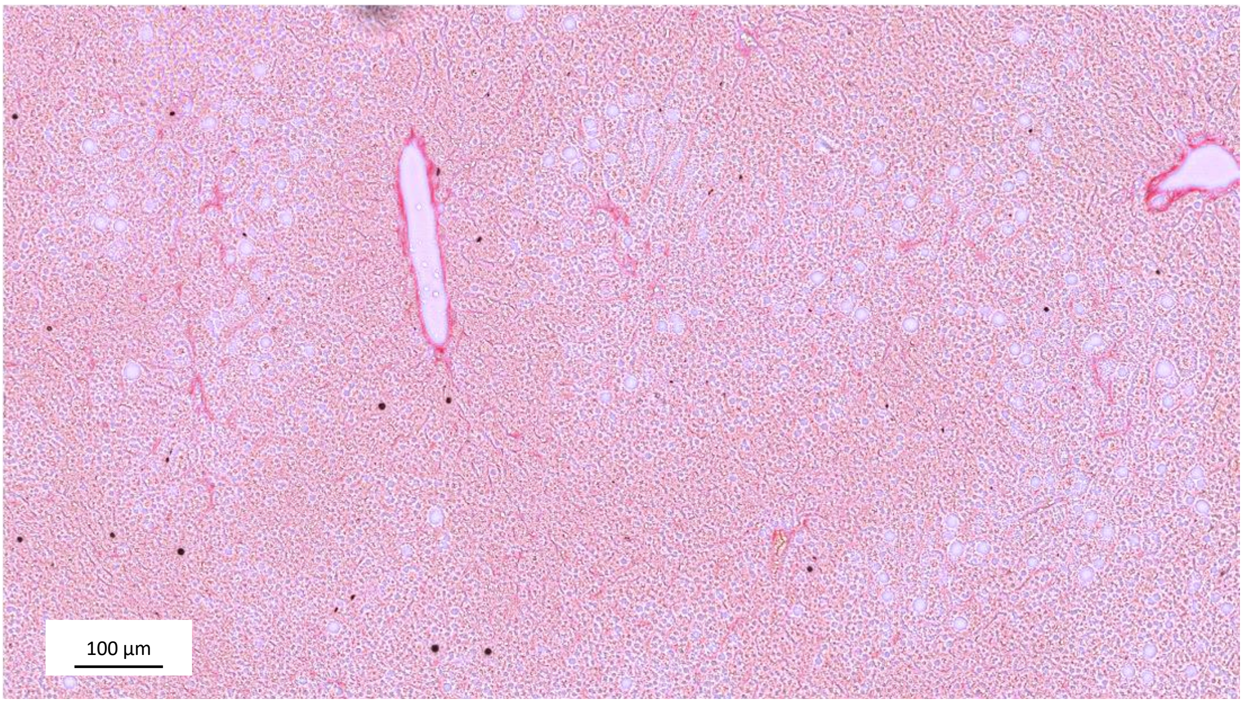

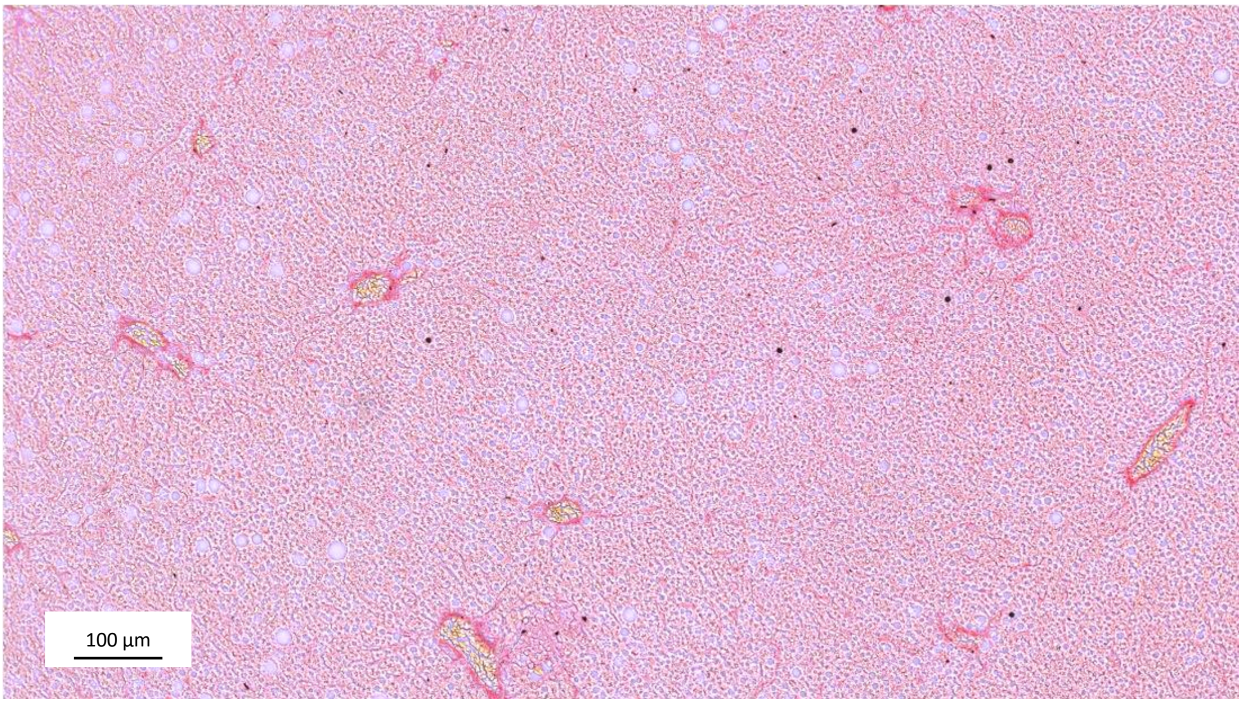

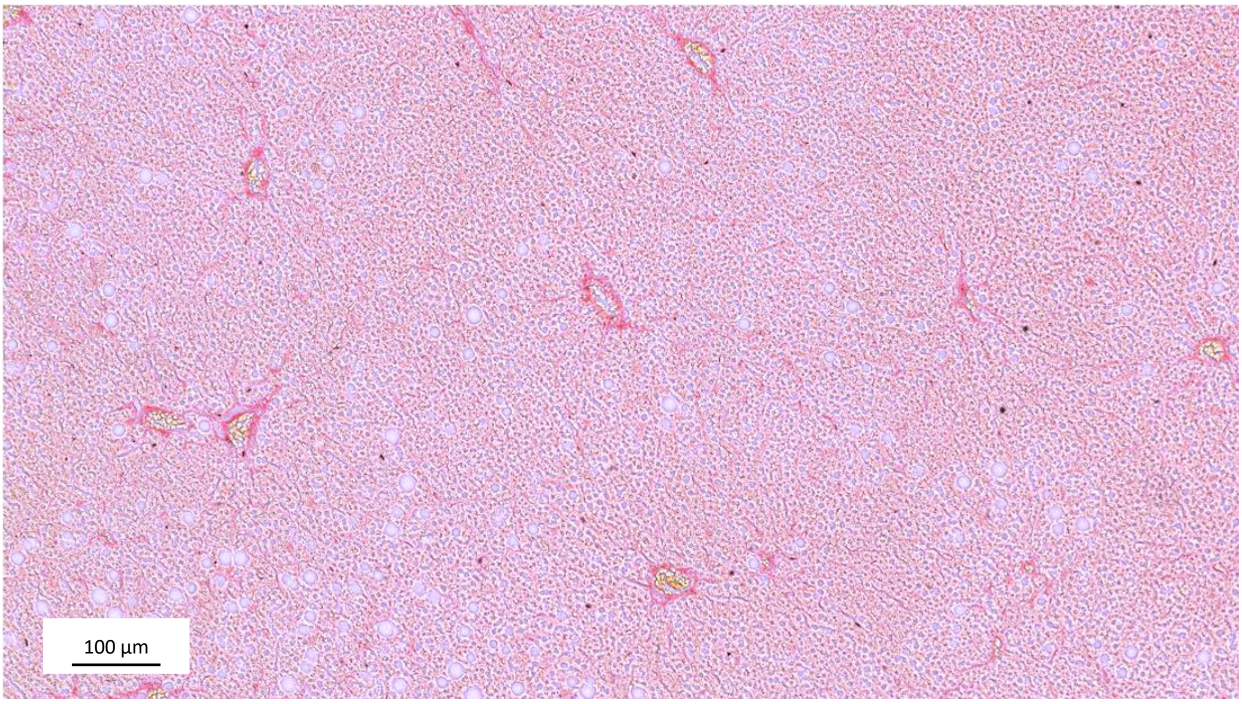

CDHFD-I-4

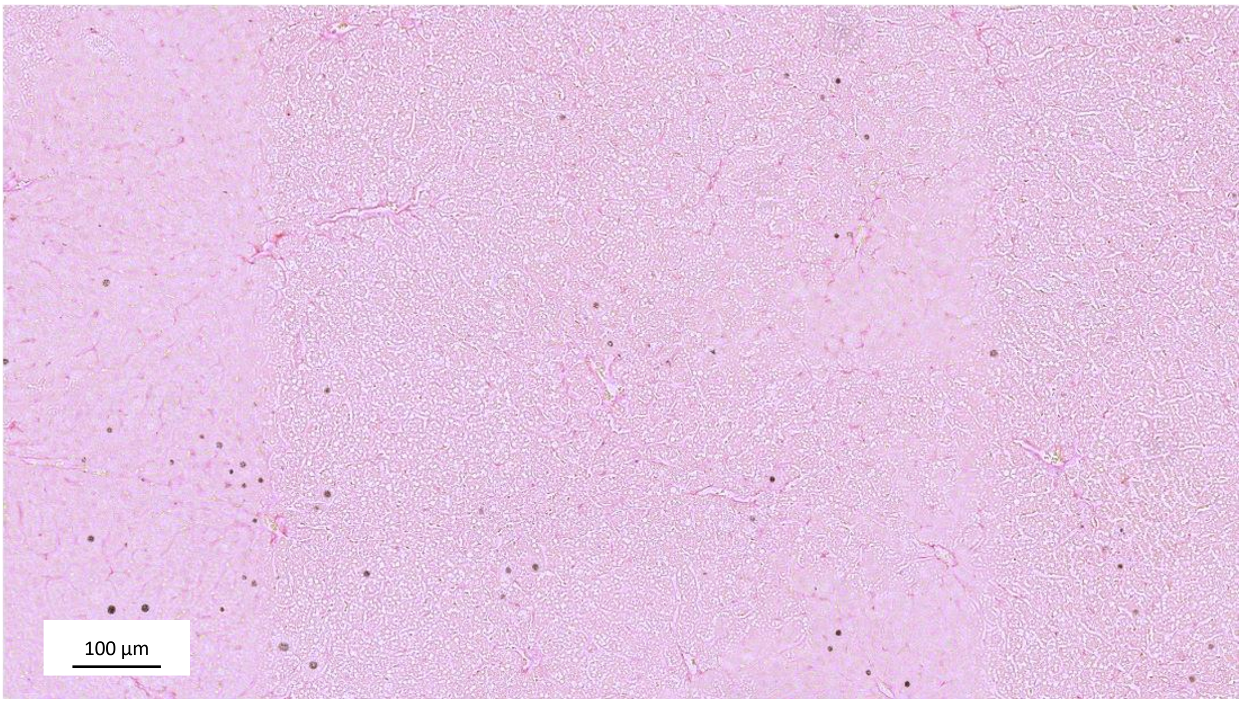

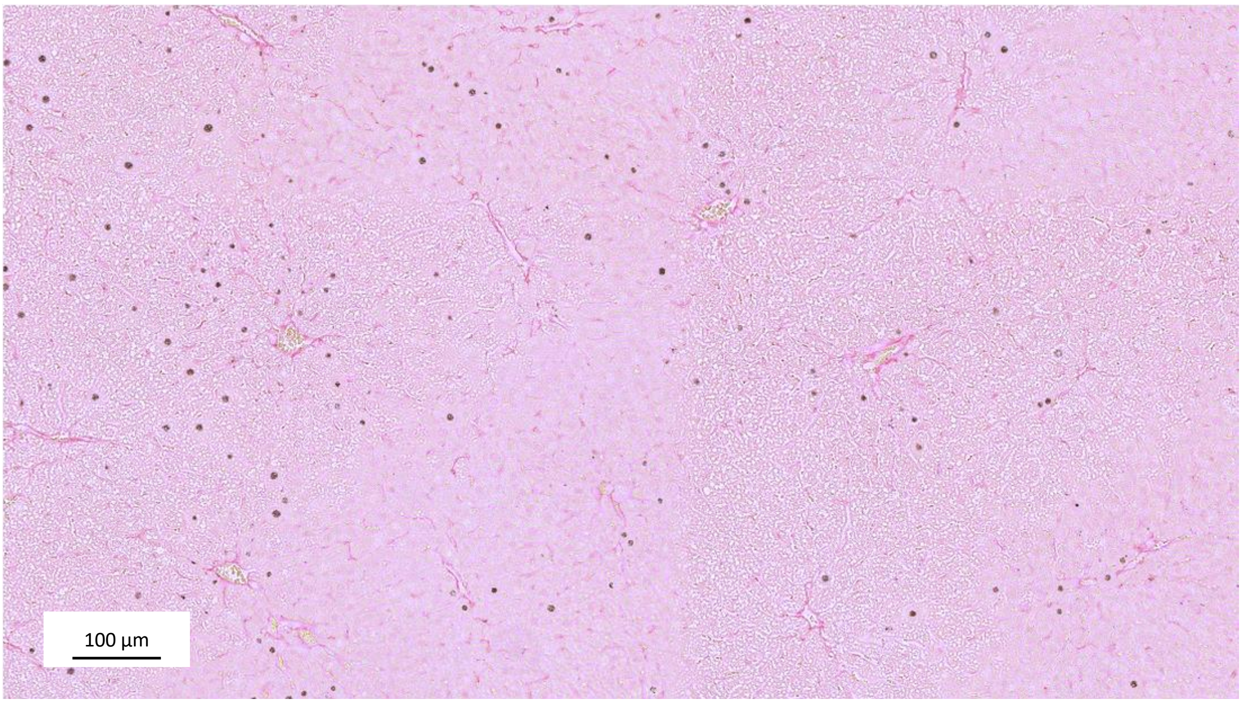

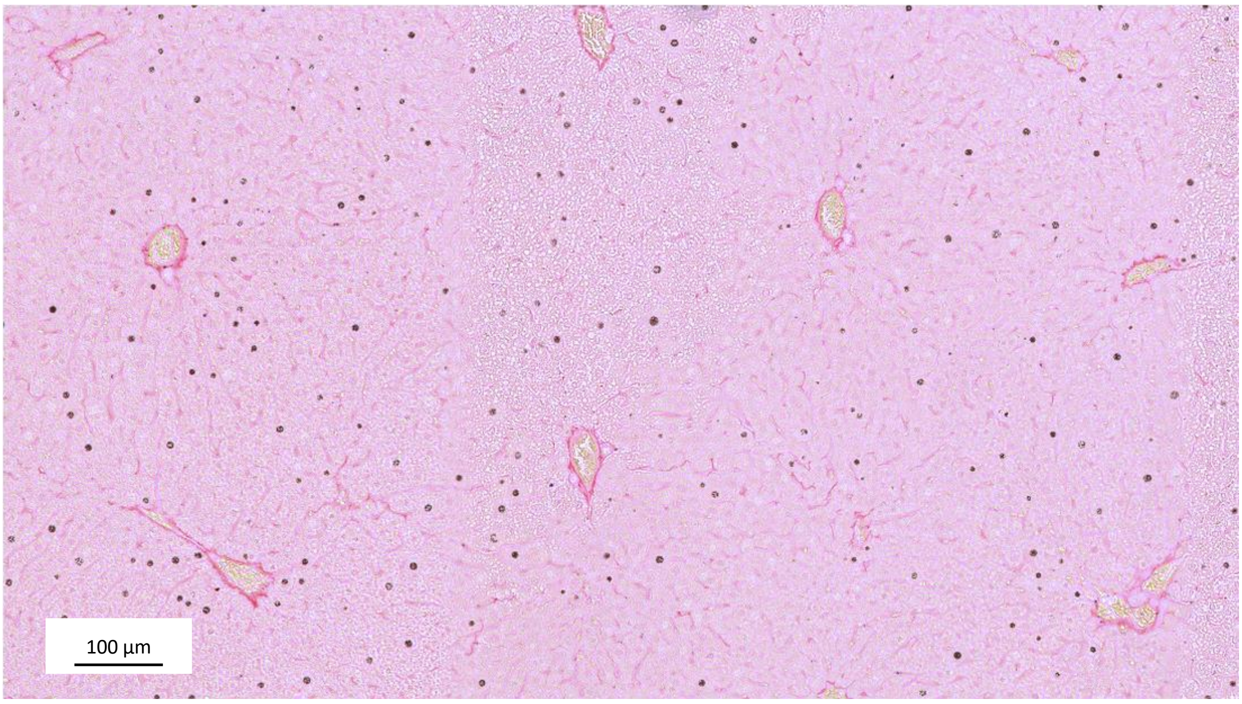

CDHFD-I-5

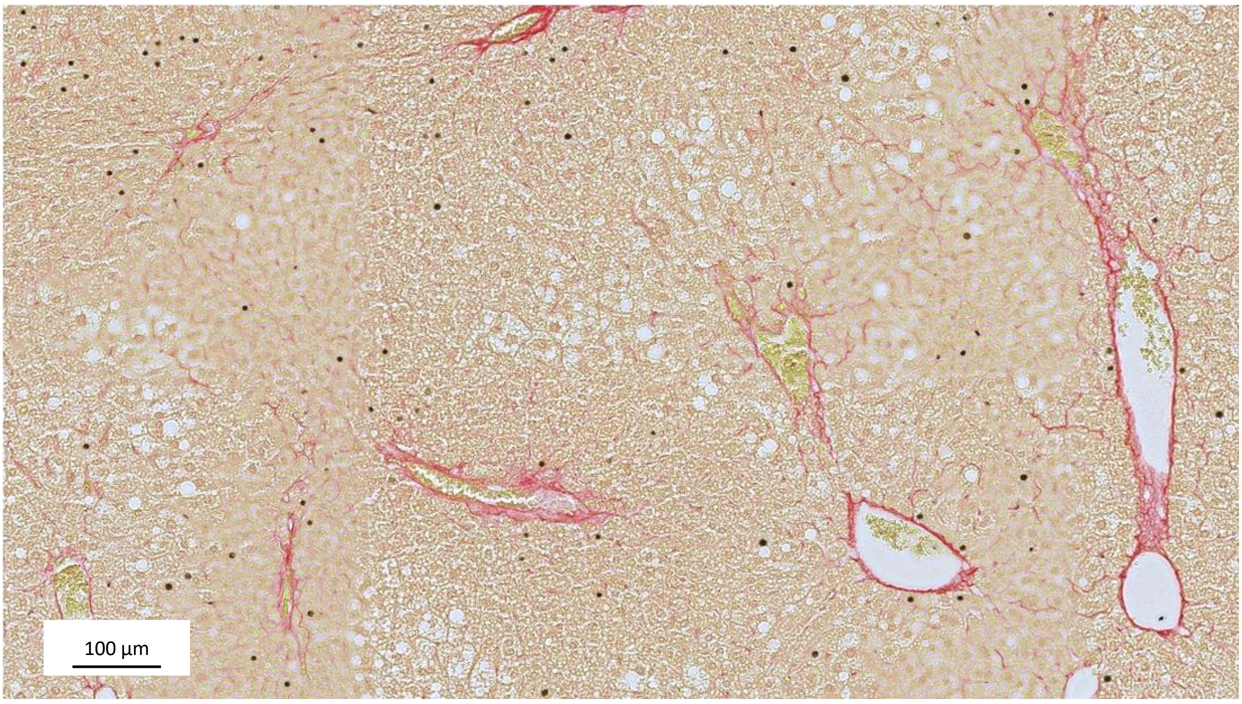

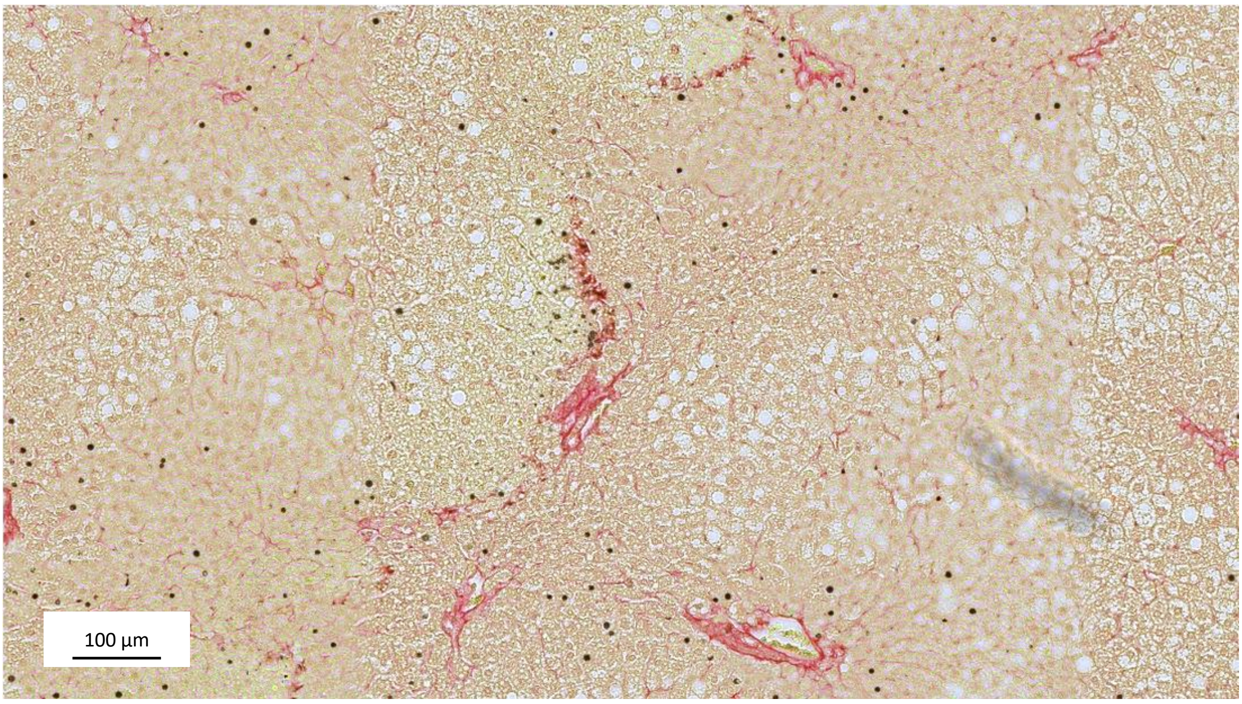

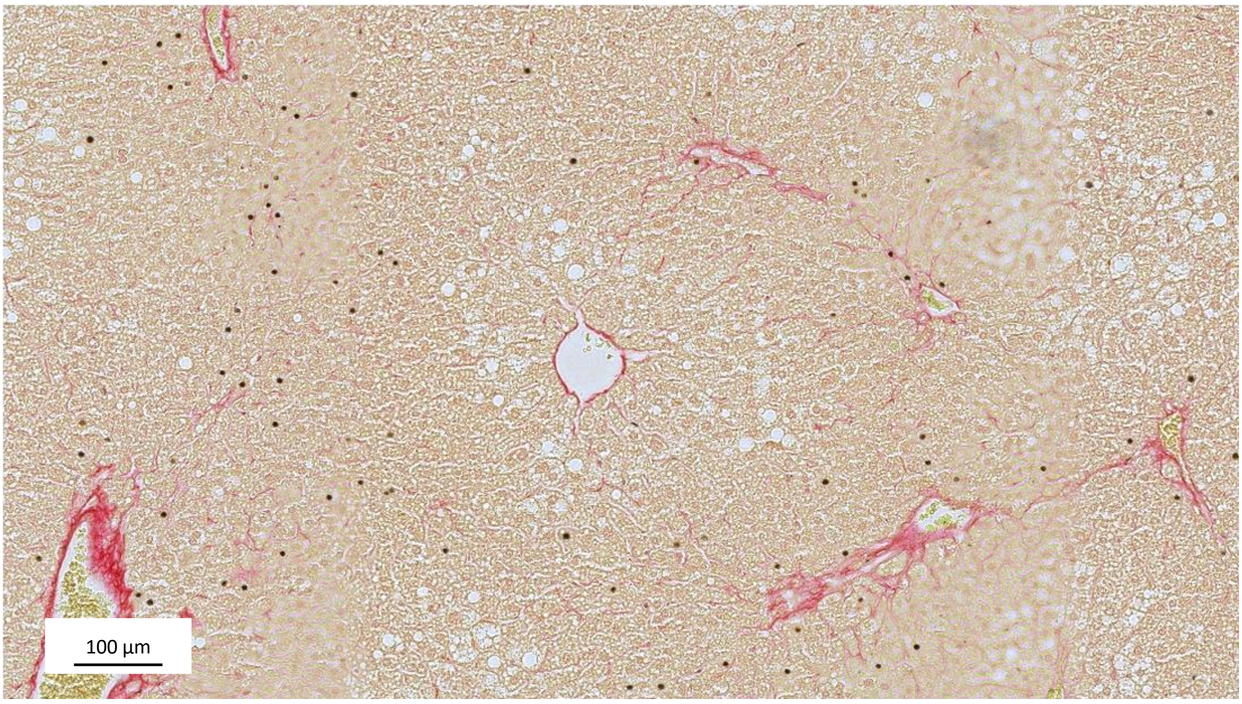

CDHFD-I-6

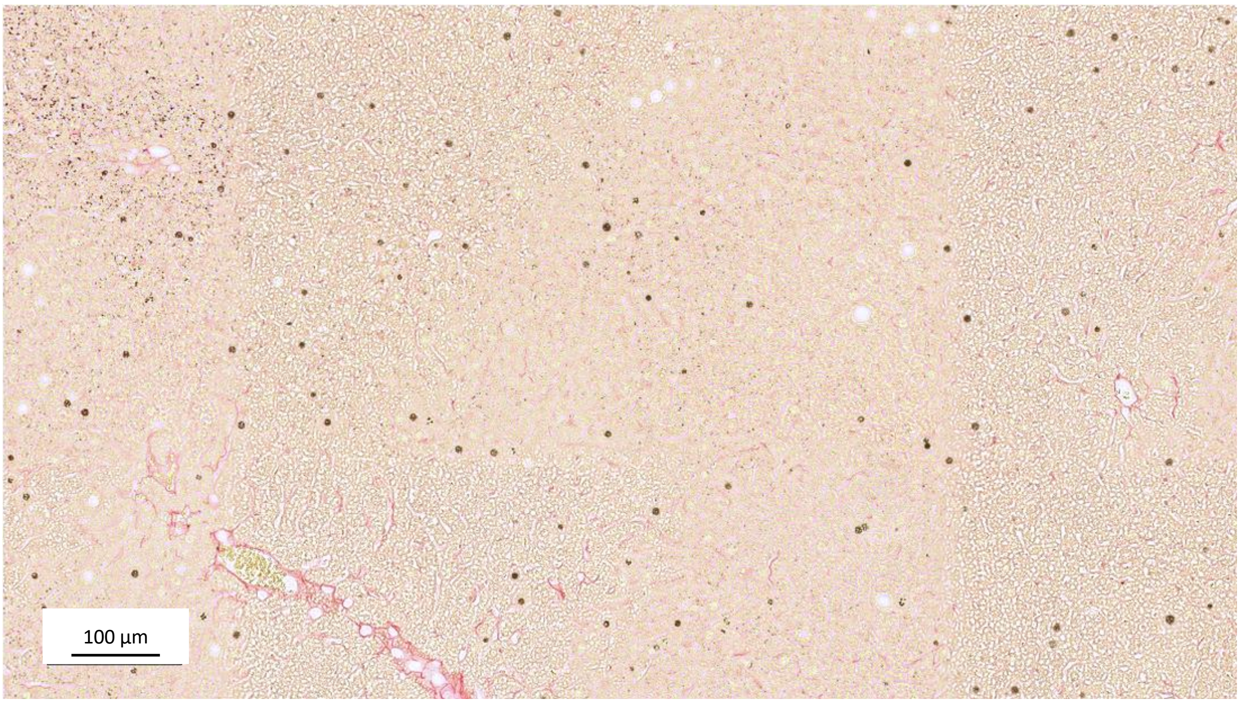

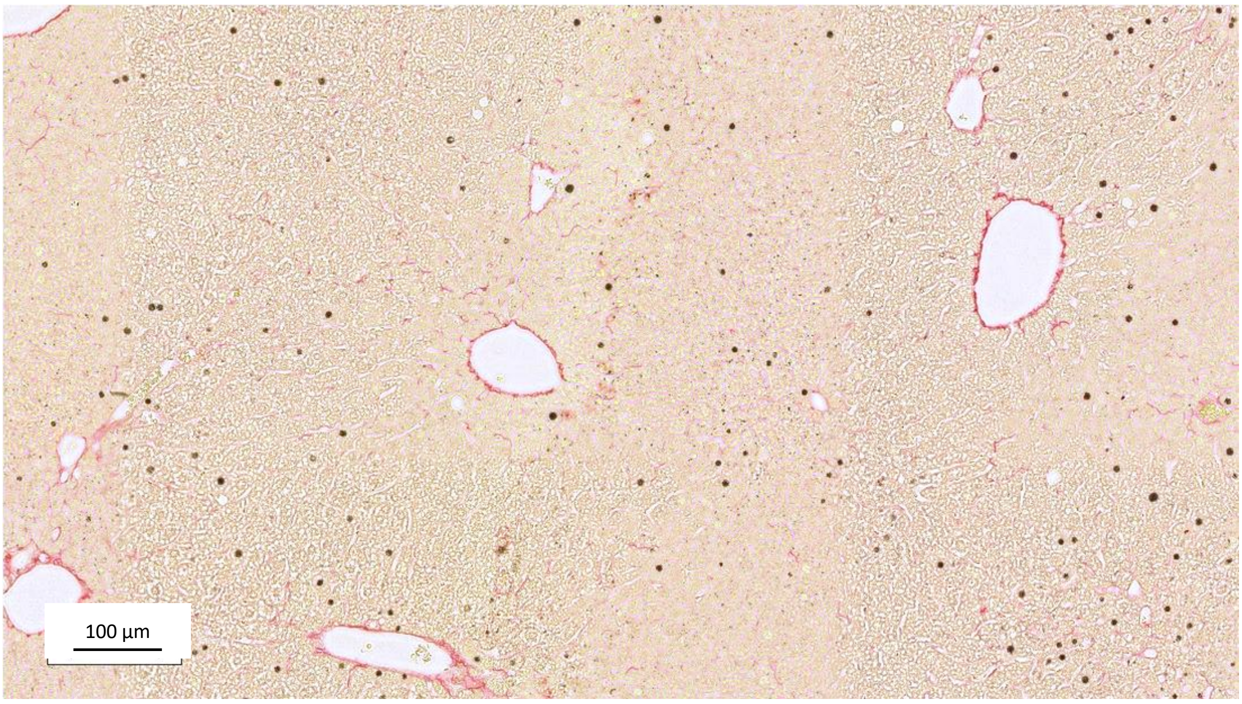

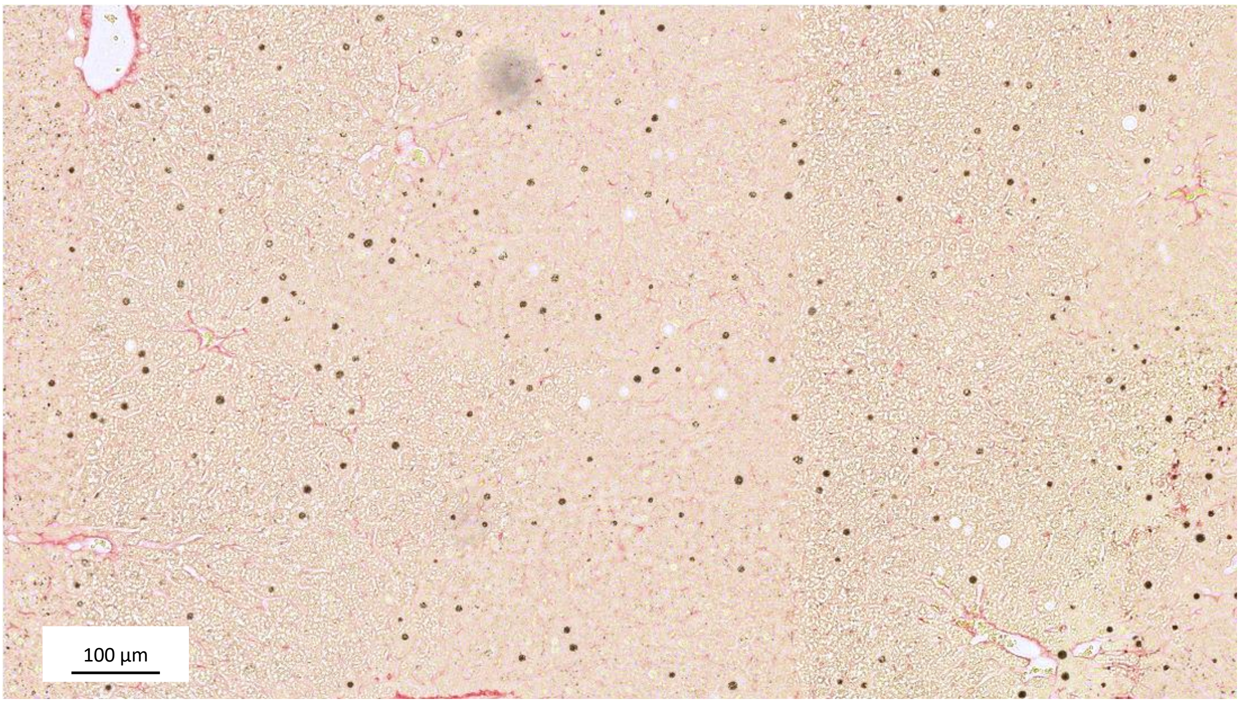

CDHFD-I-7

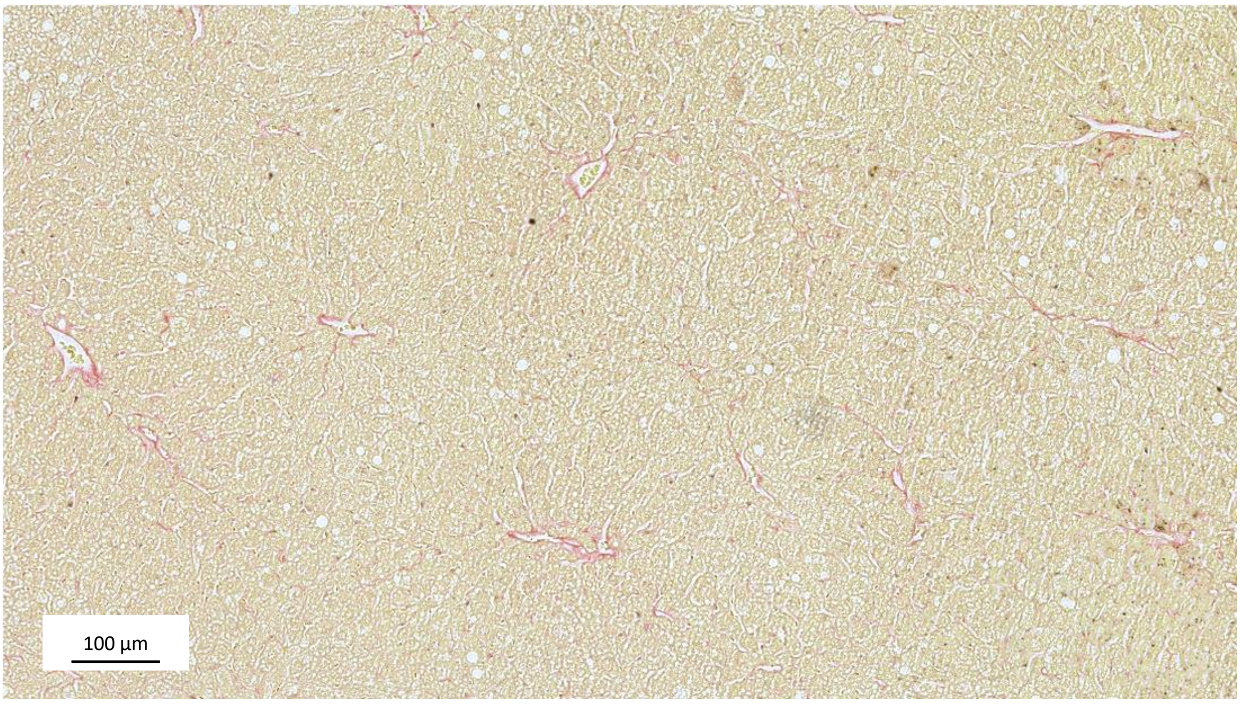

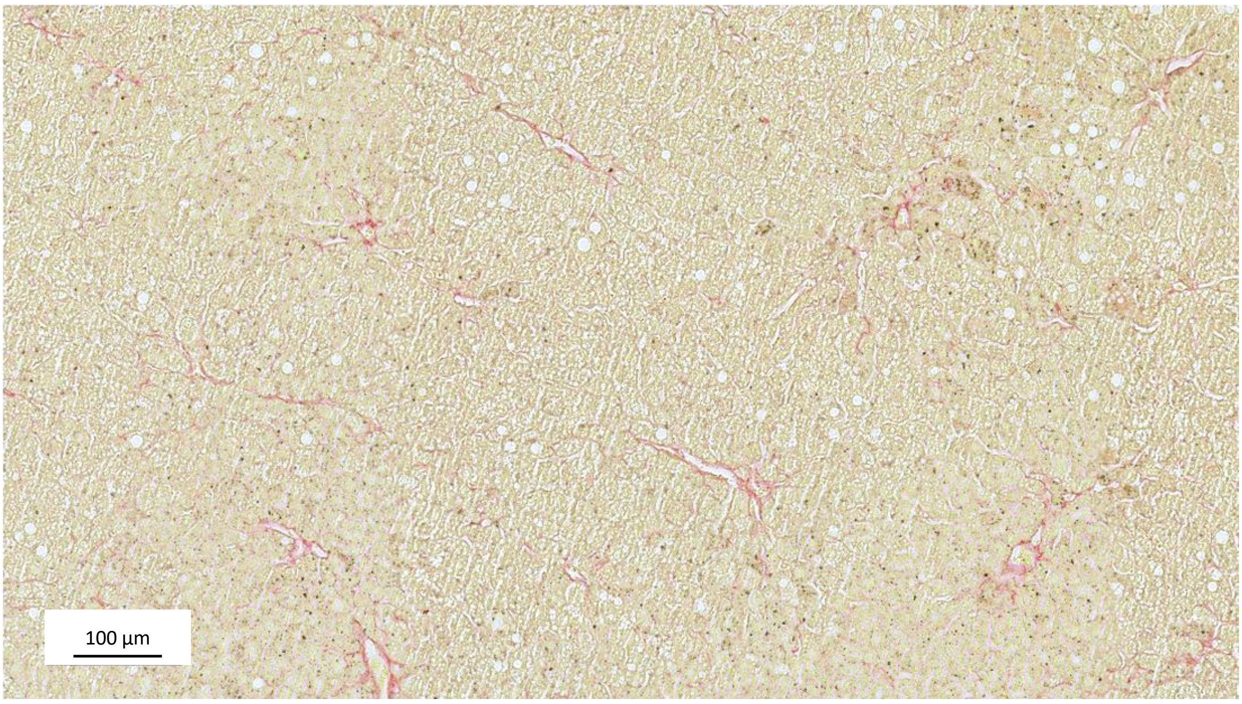

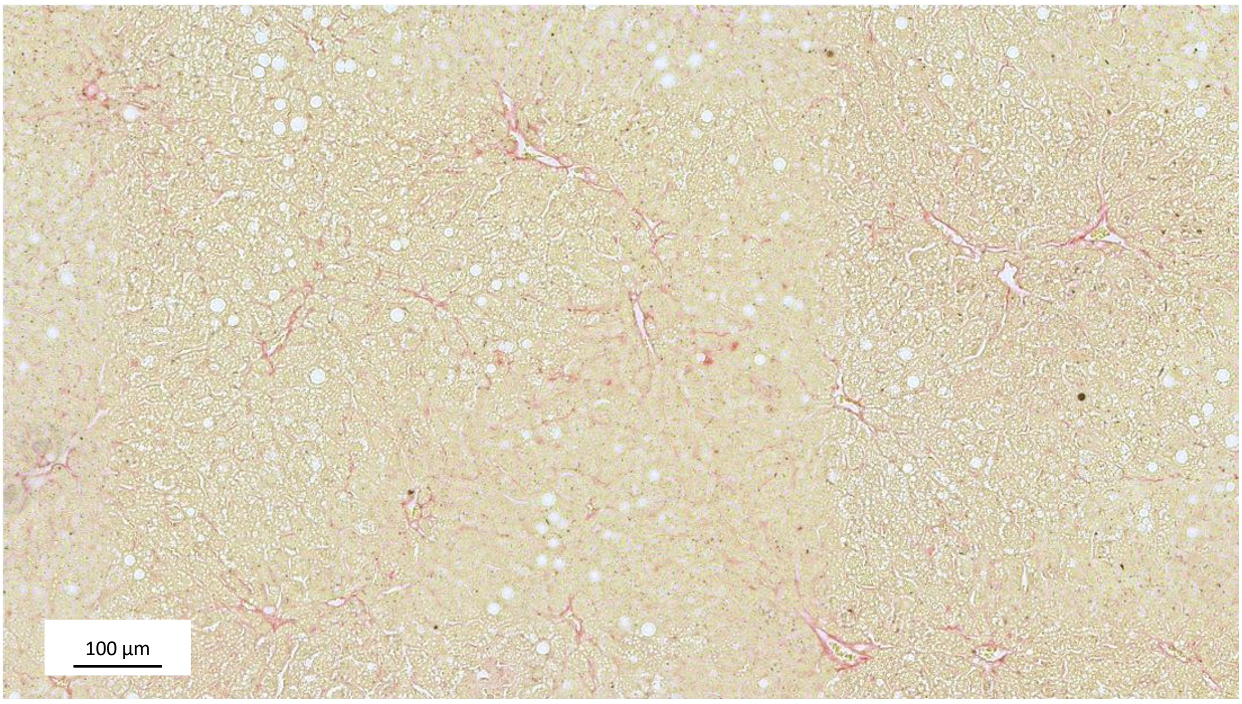

CDHFD-I-8

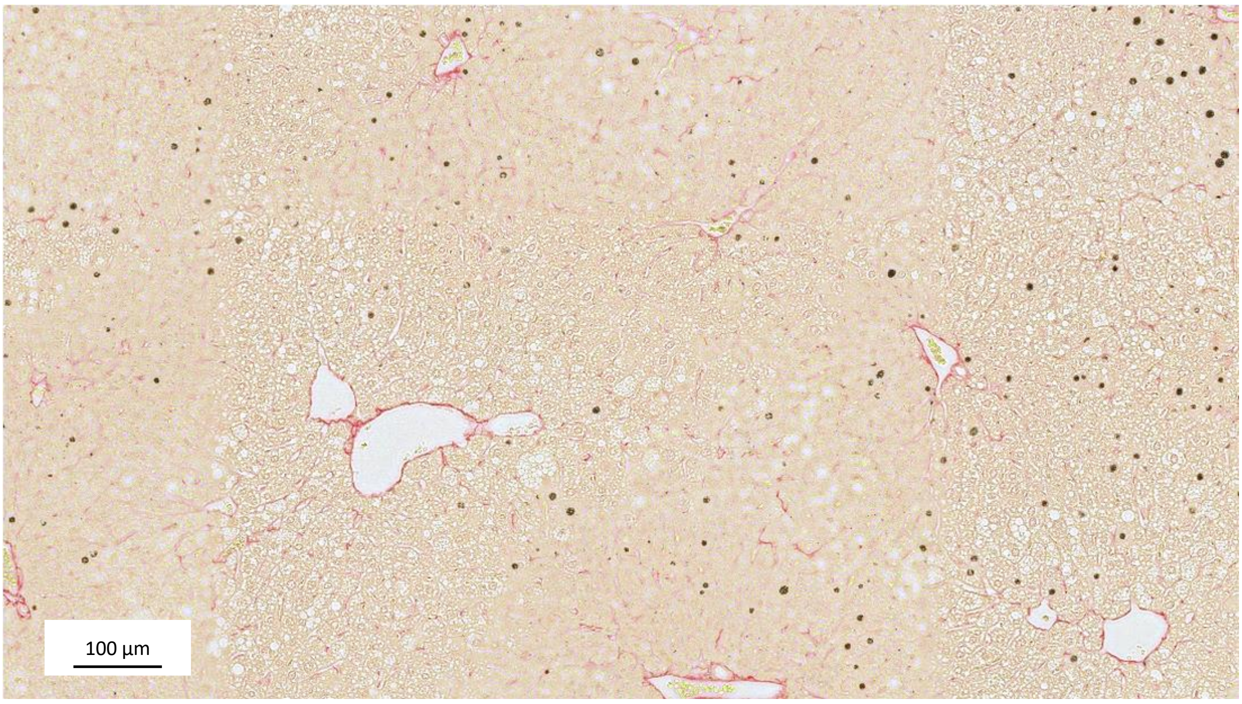

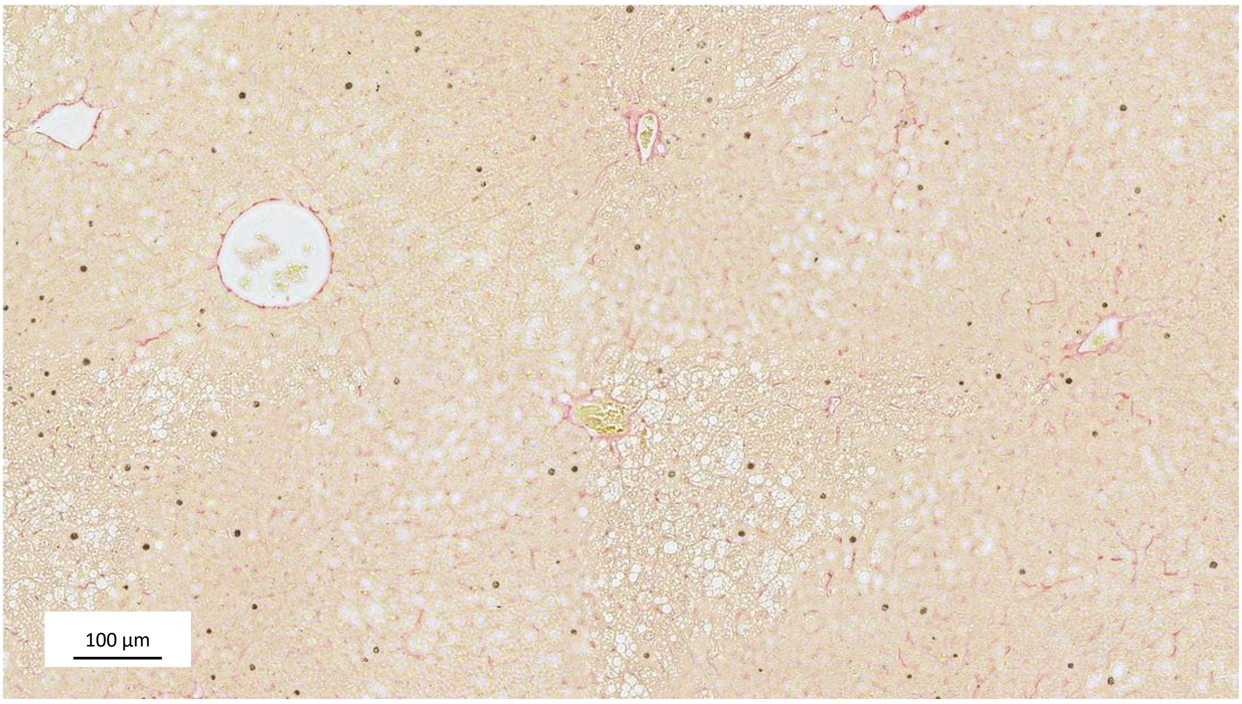

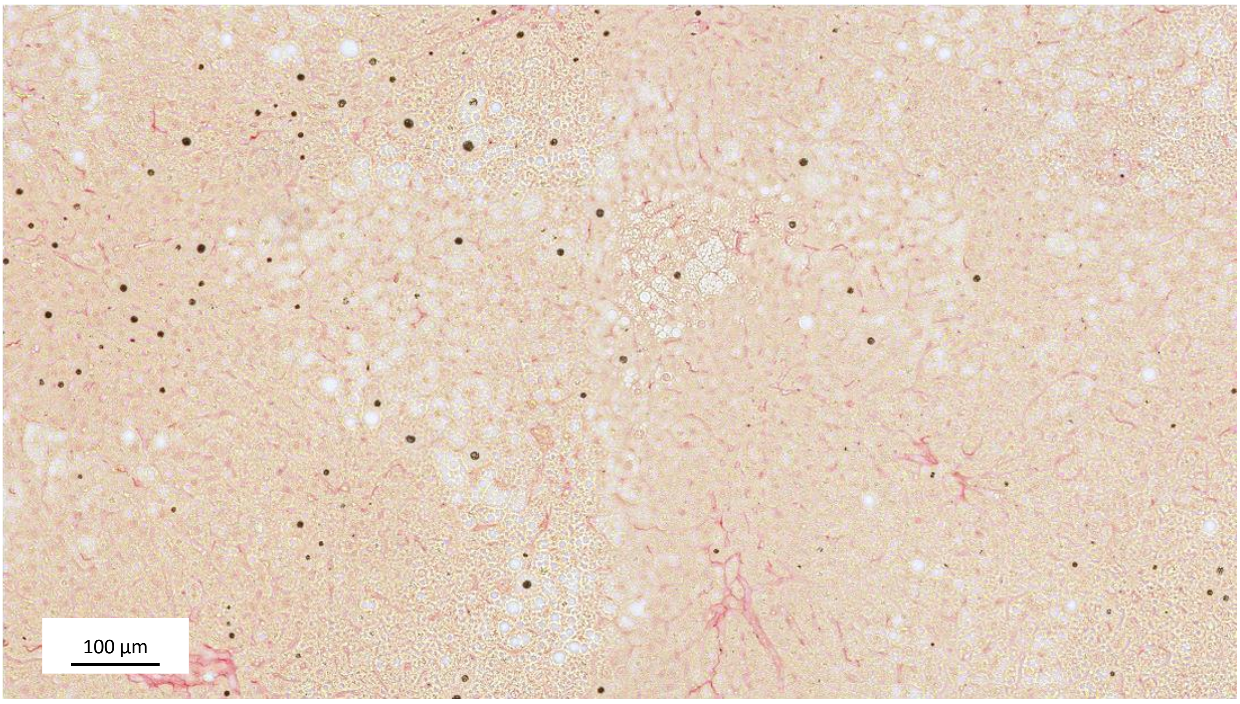

CDHFD-I-9

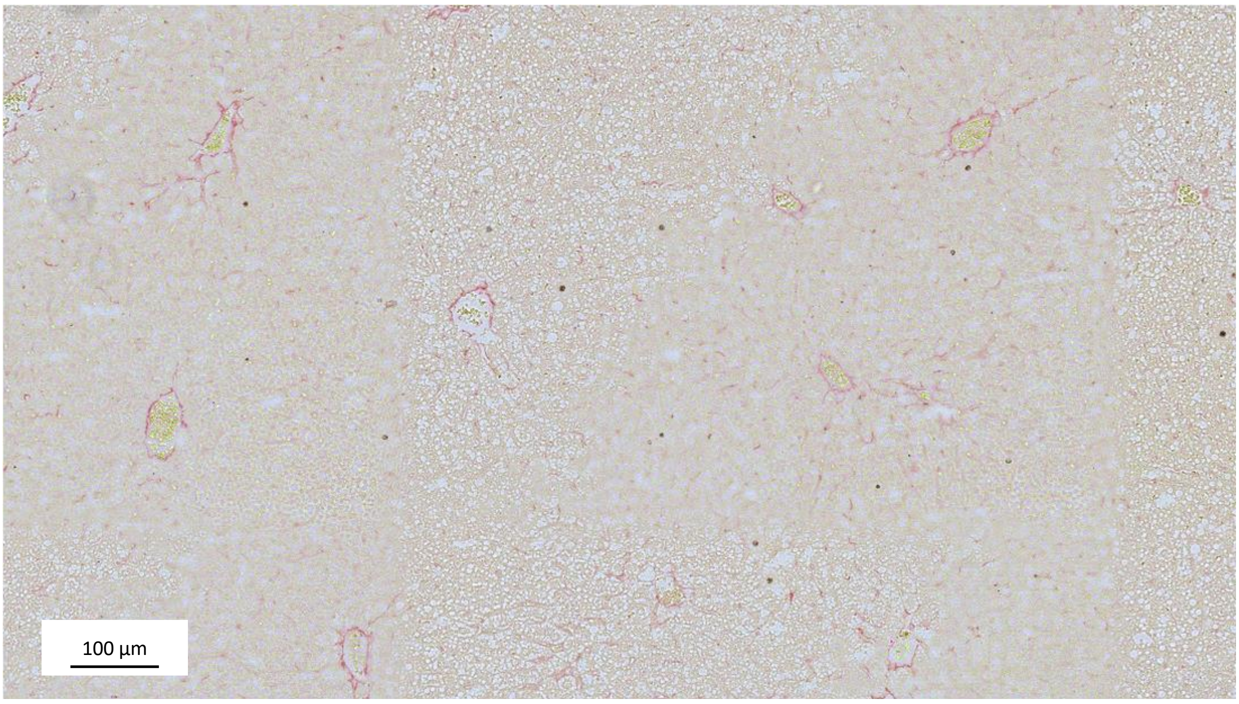

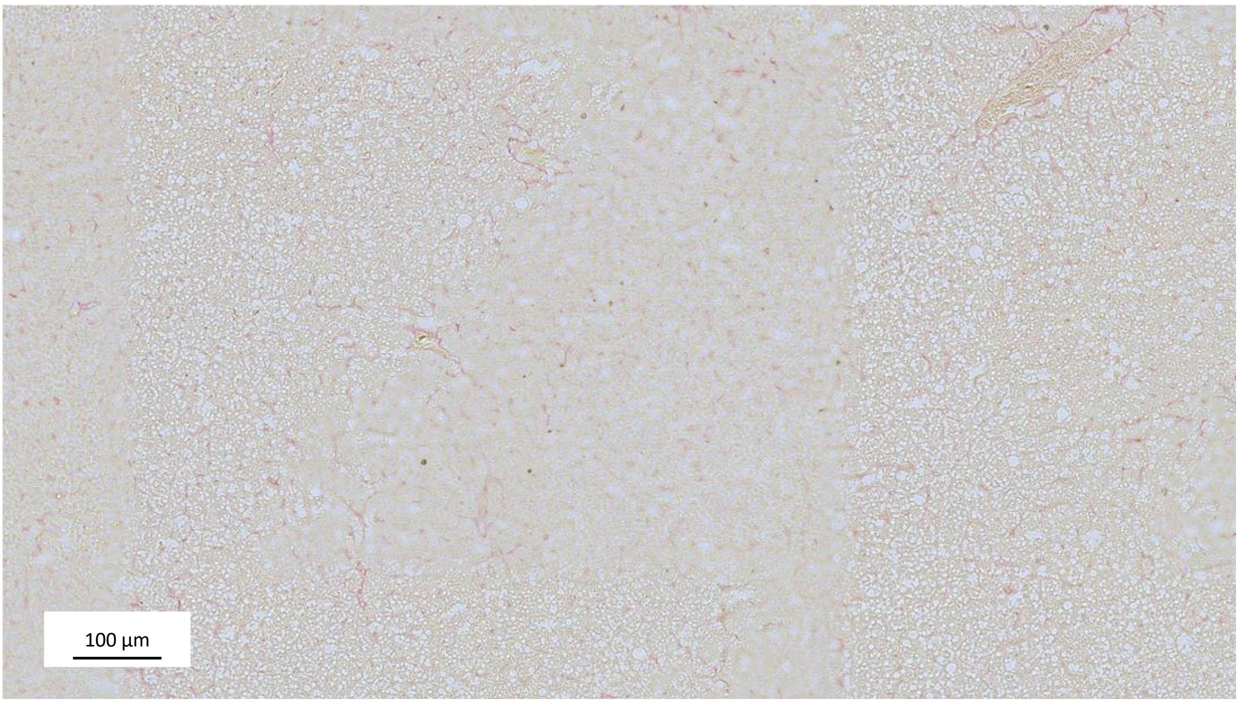

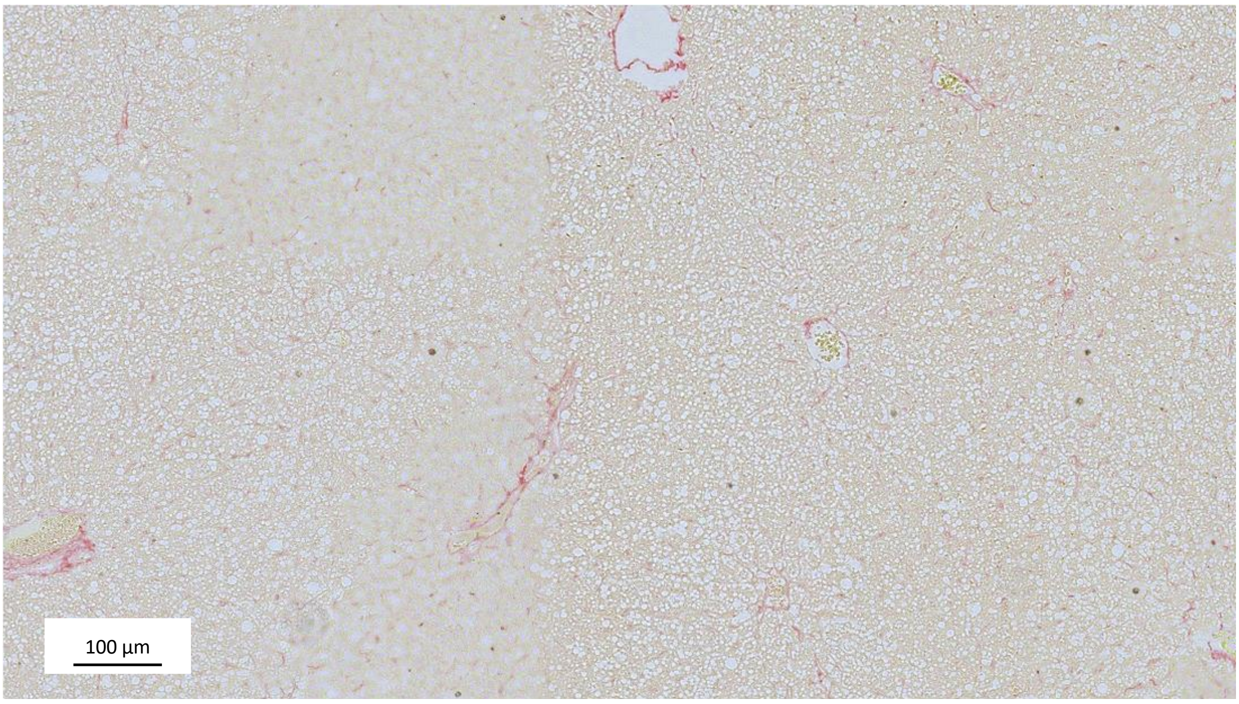

CDHFD-I-10

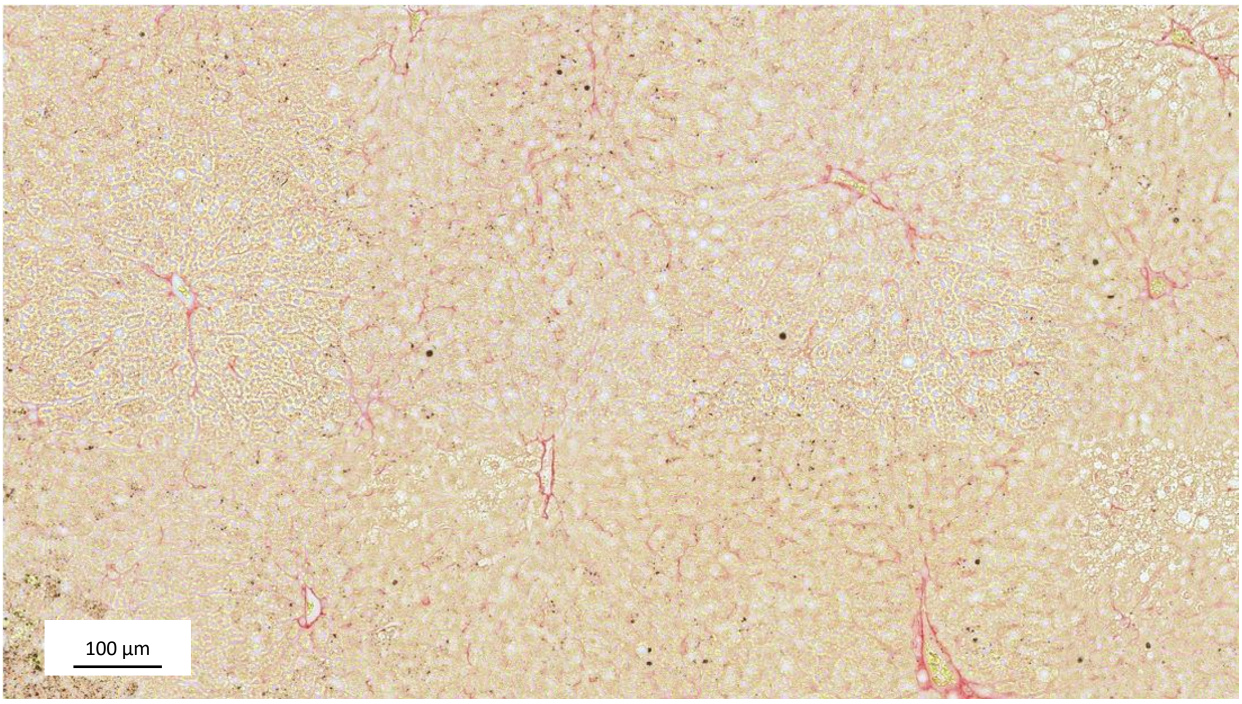

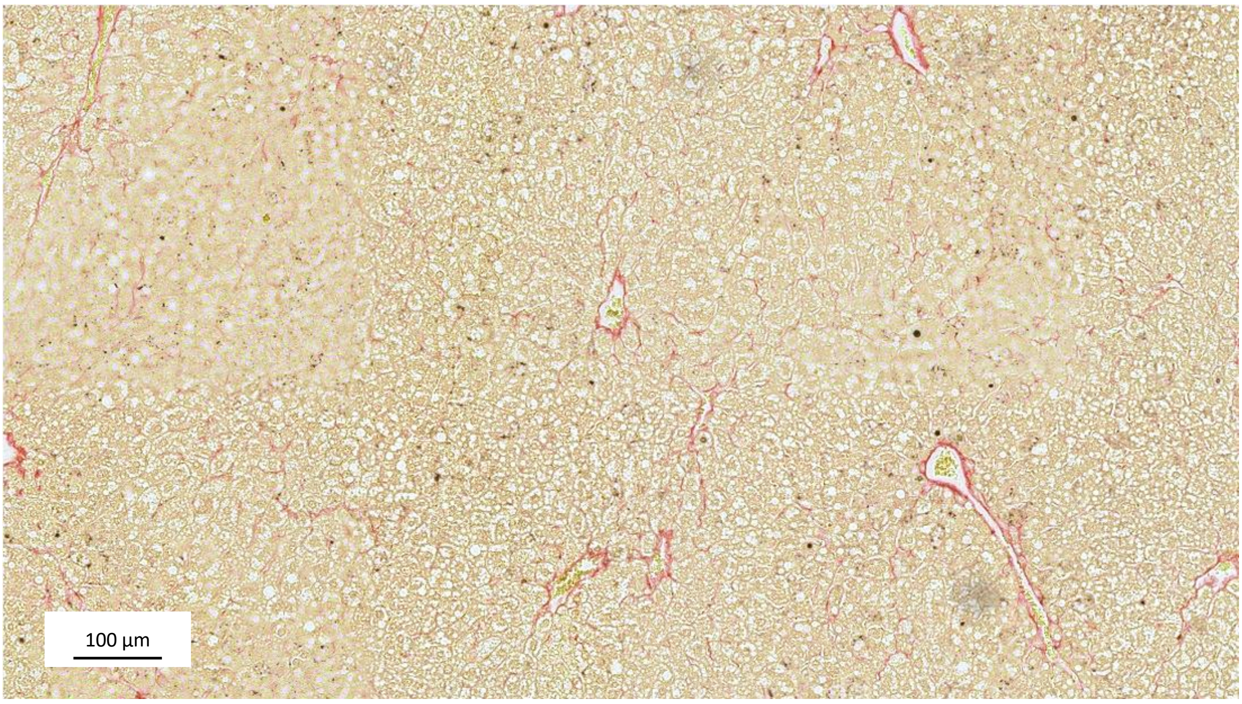

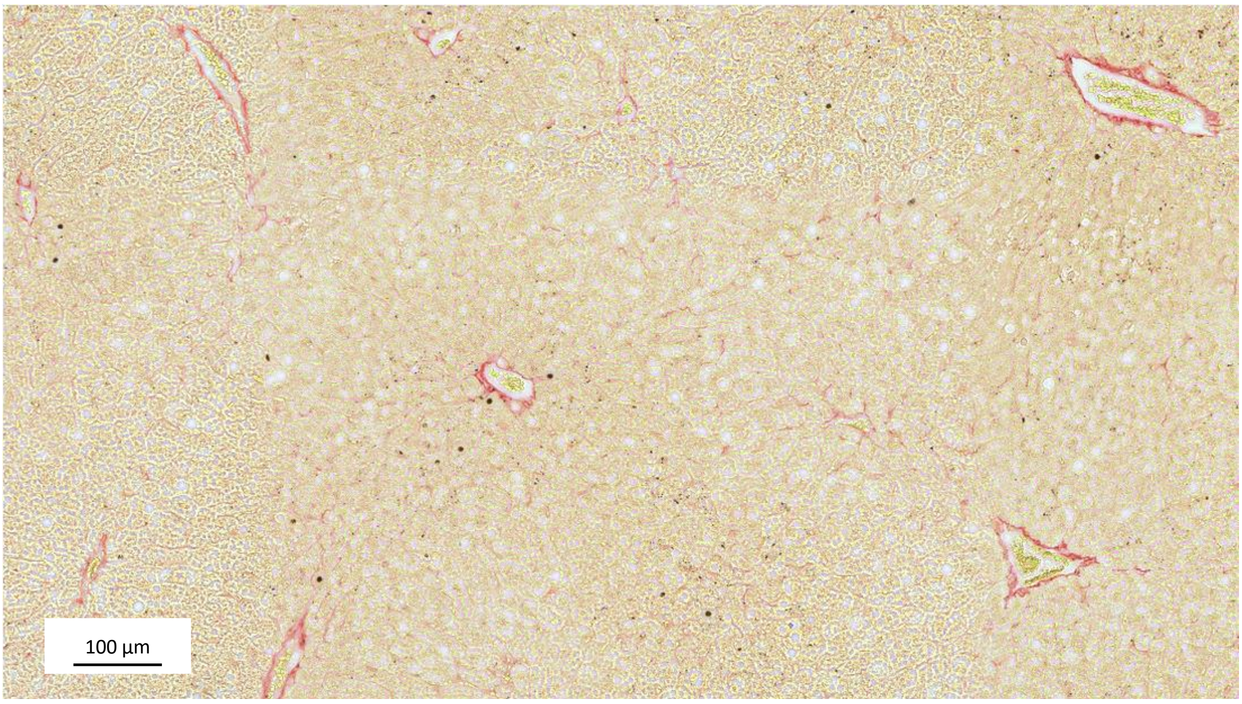

CDHFD-I-11

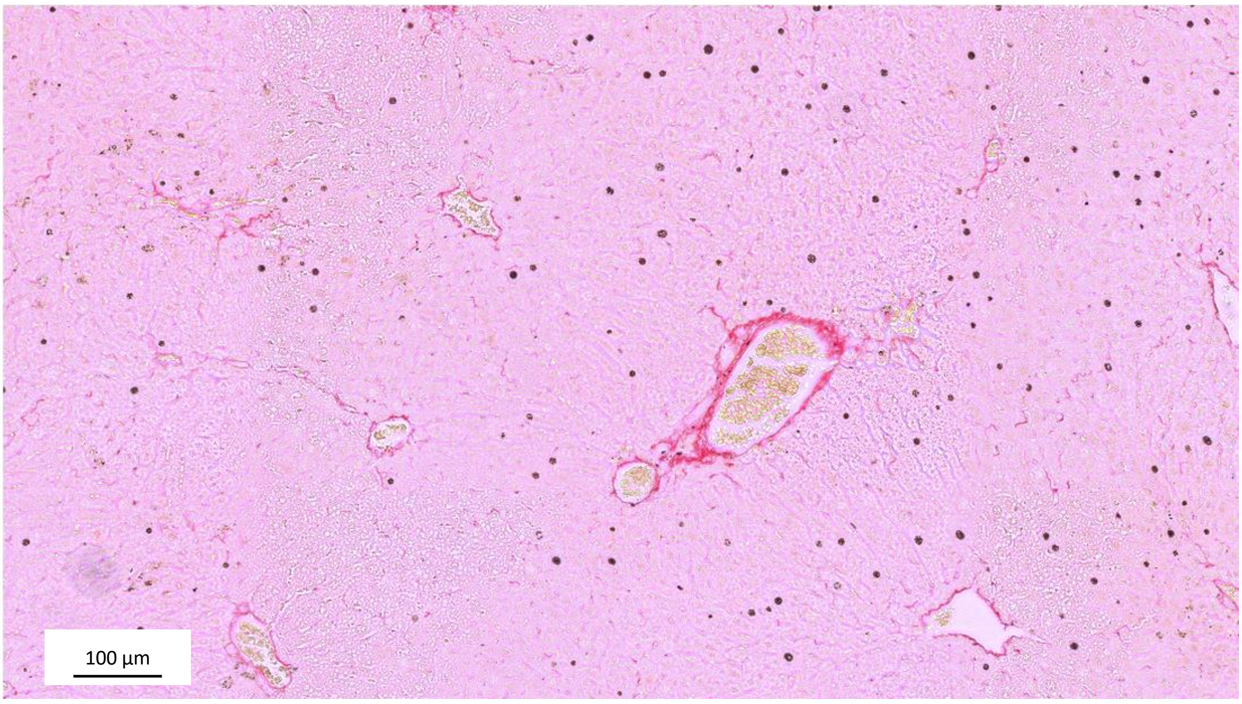

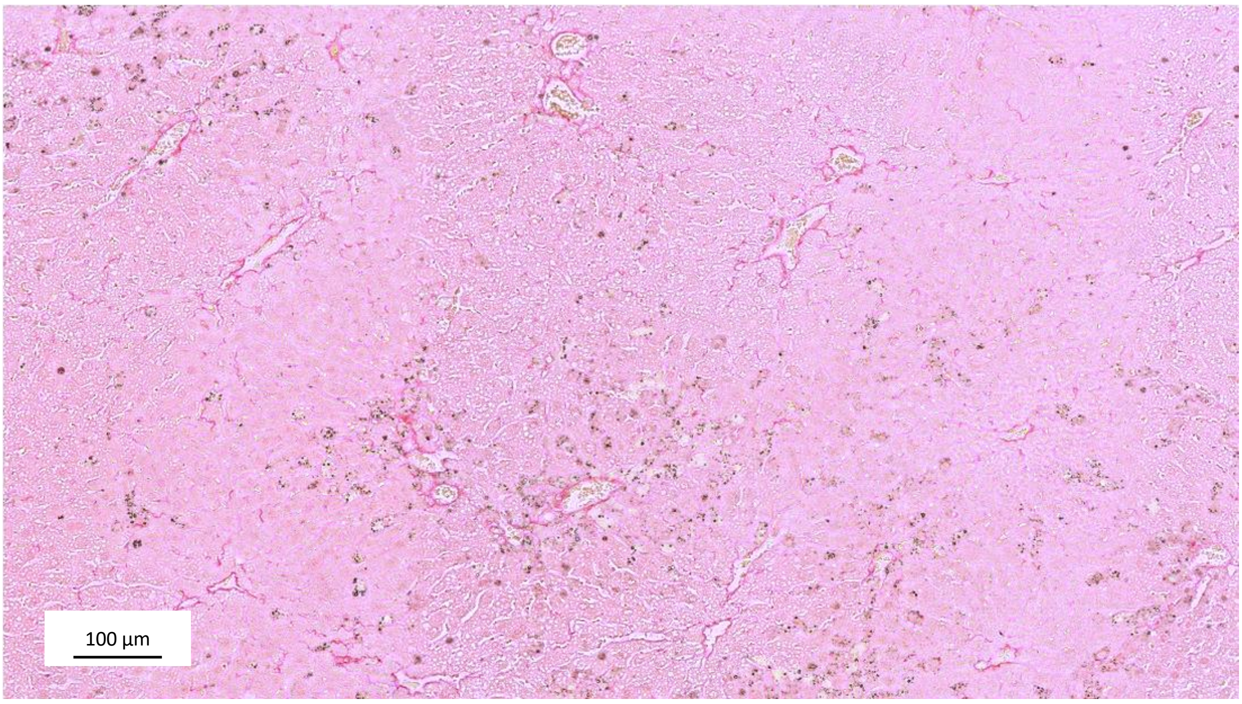

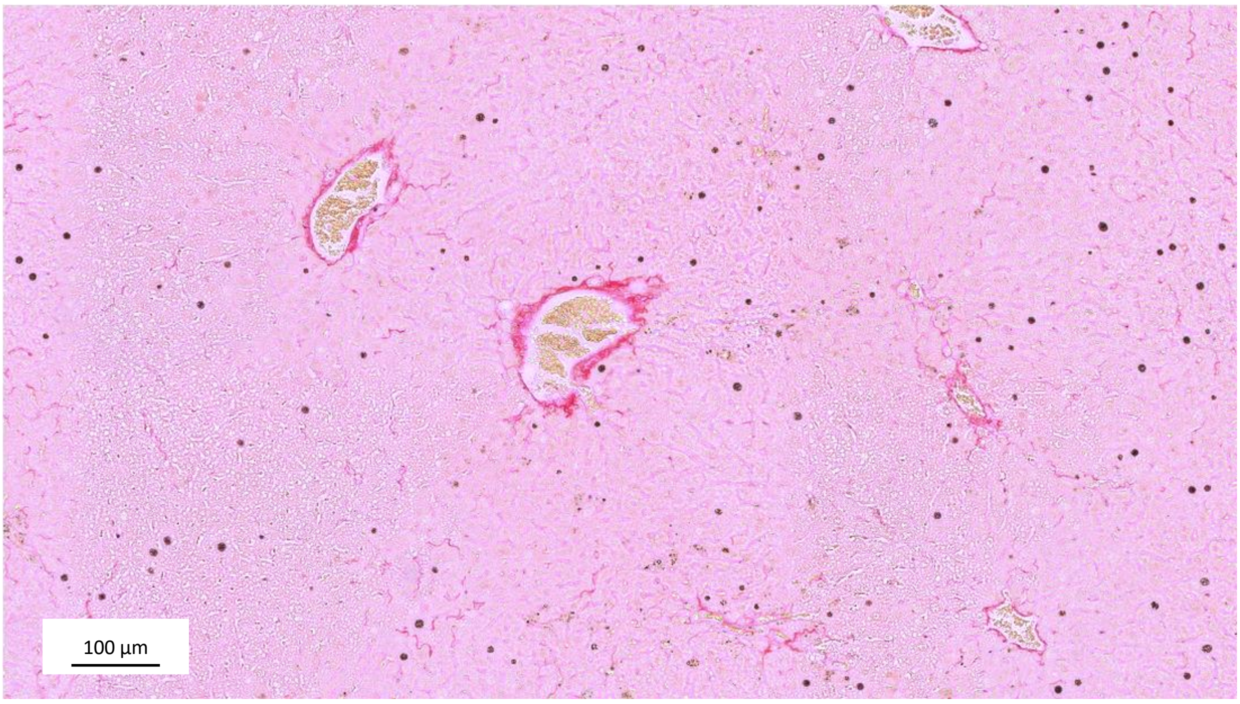

CDHFD-I-12

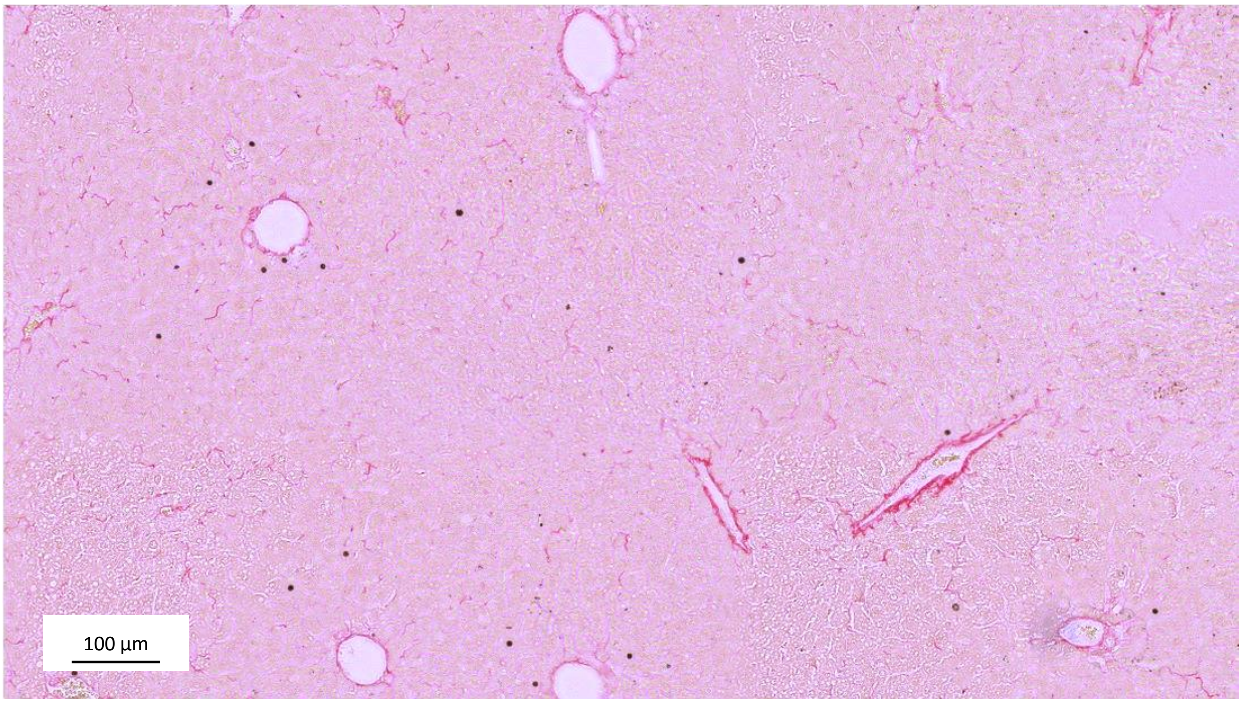

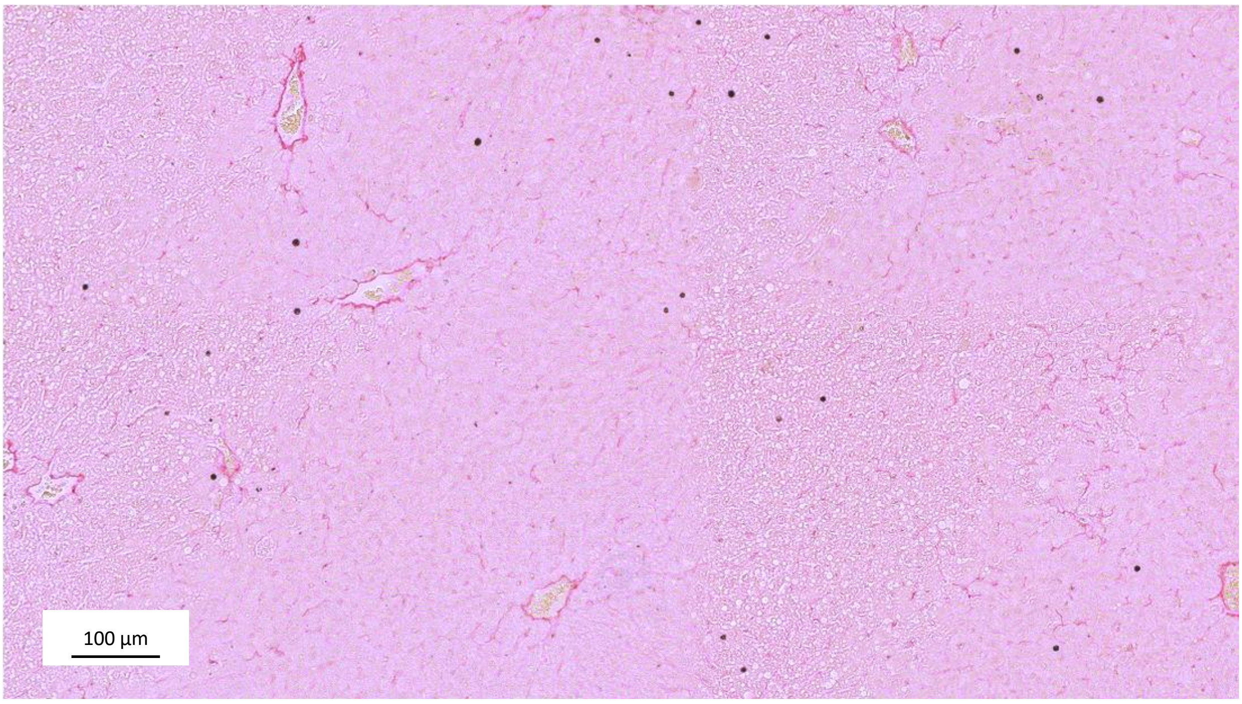

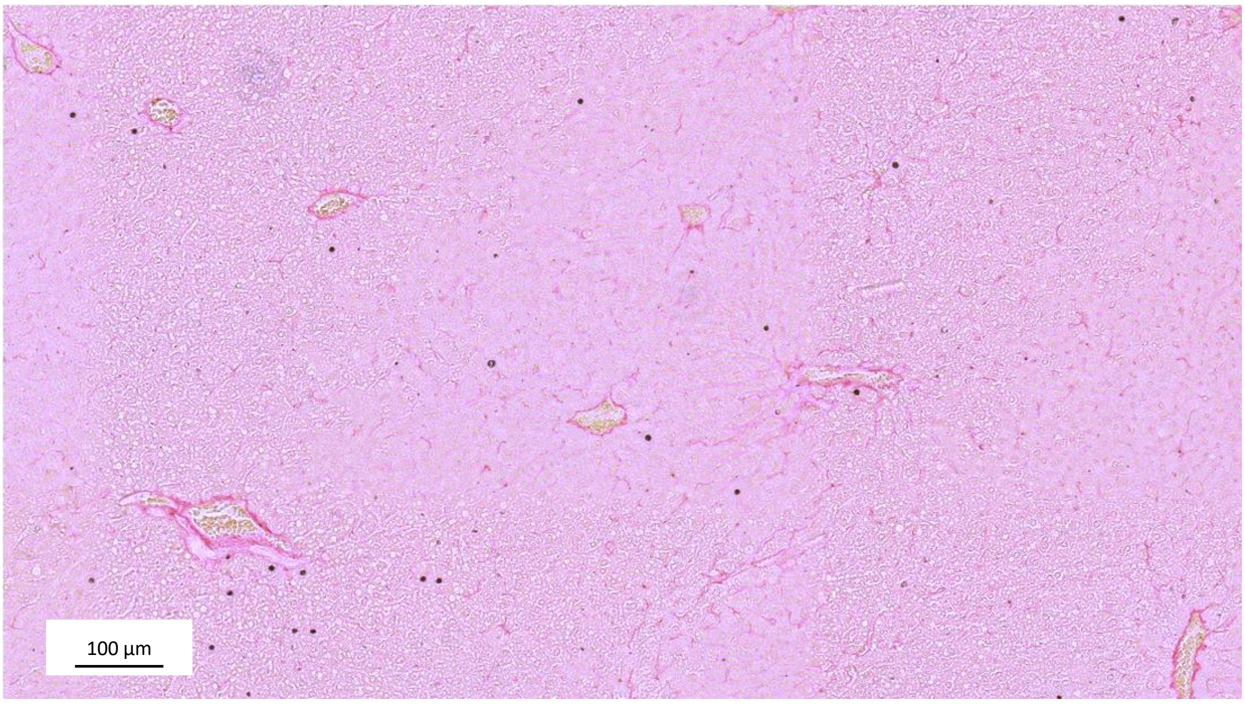

CDHFD-I-13

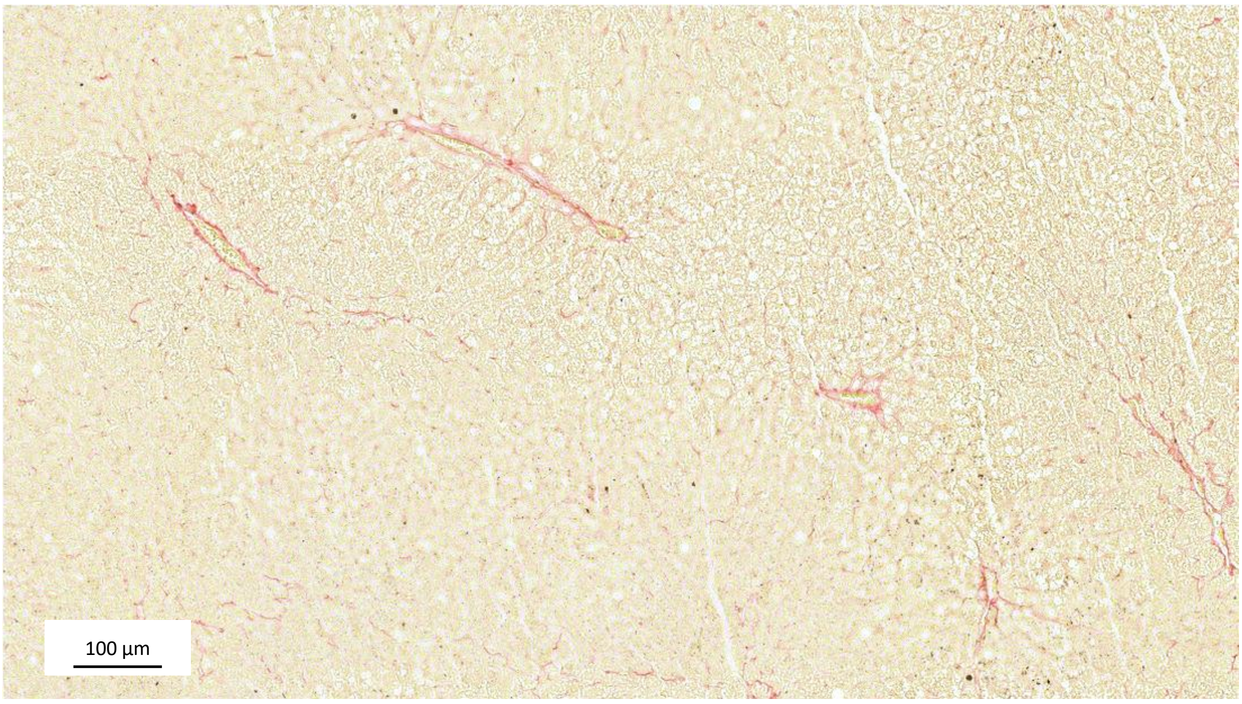

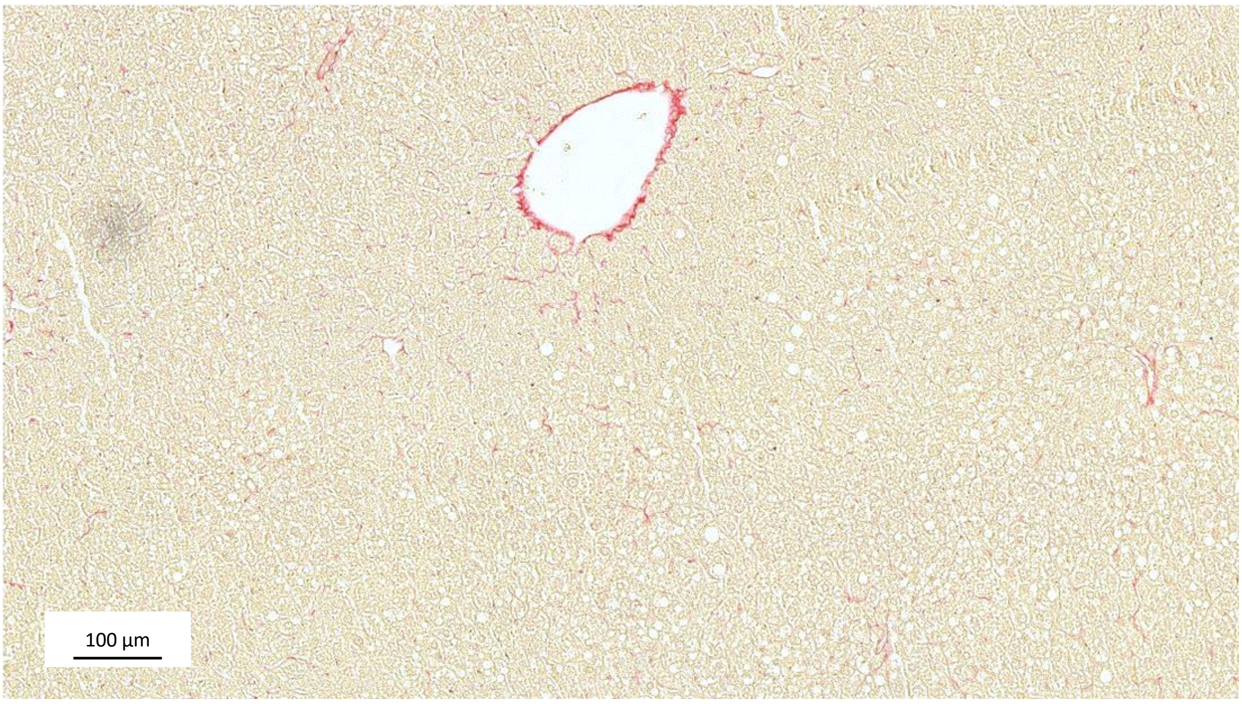

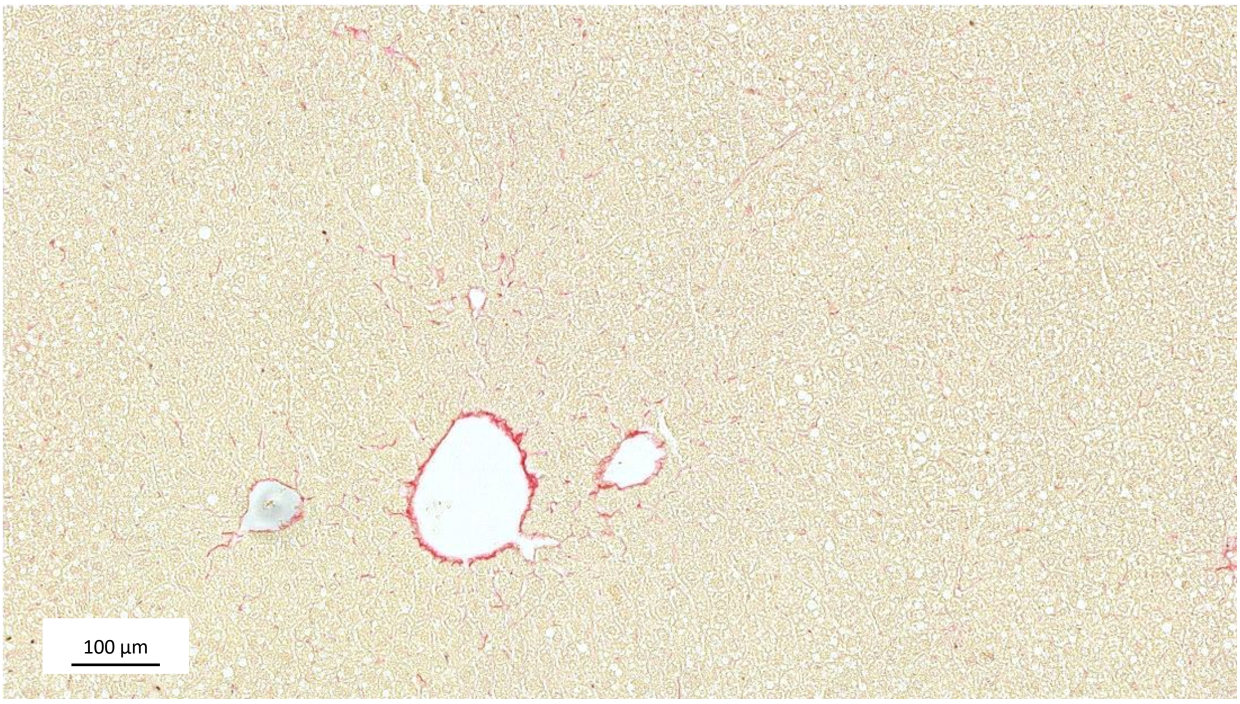

CDHFD-I-14

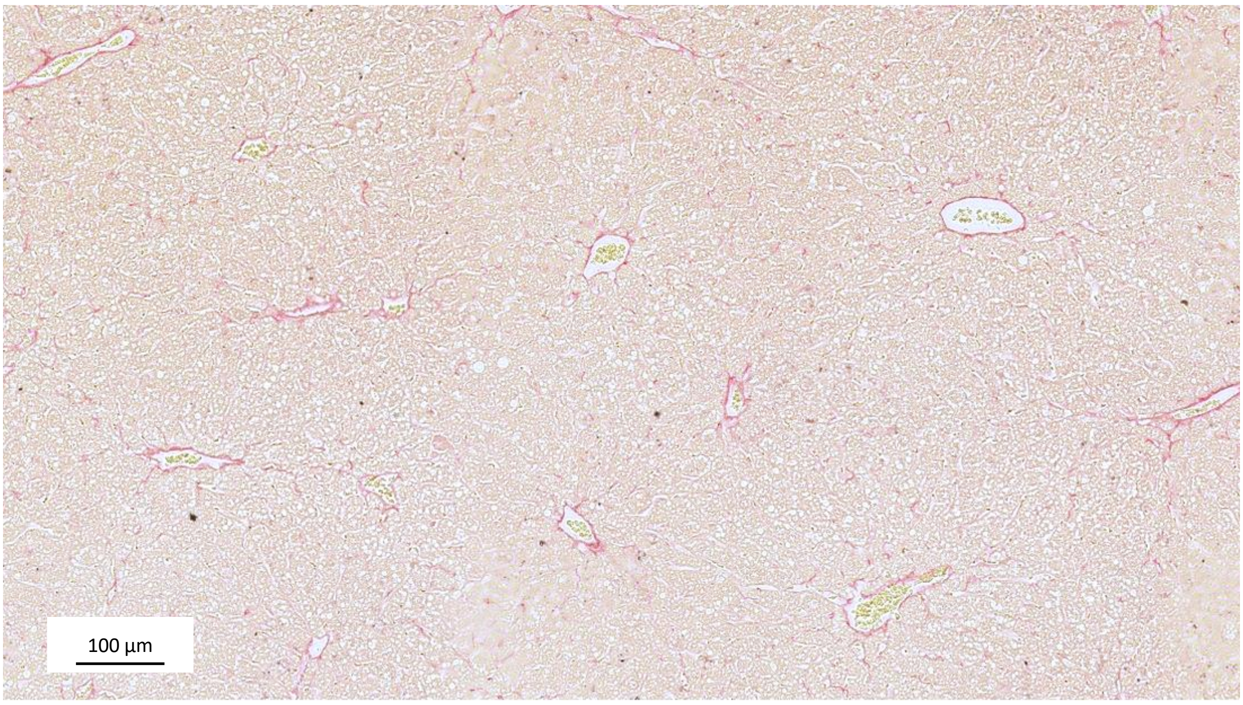

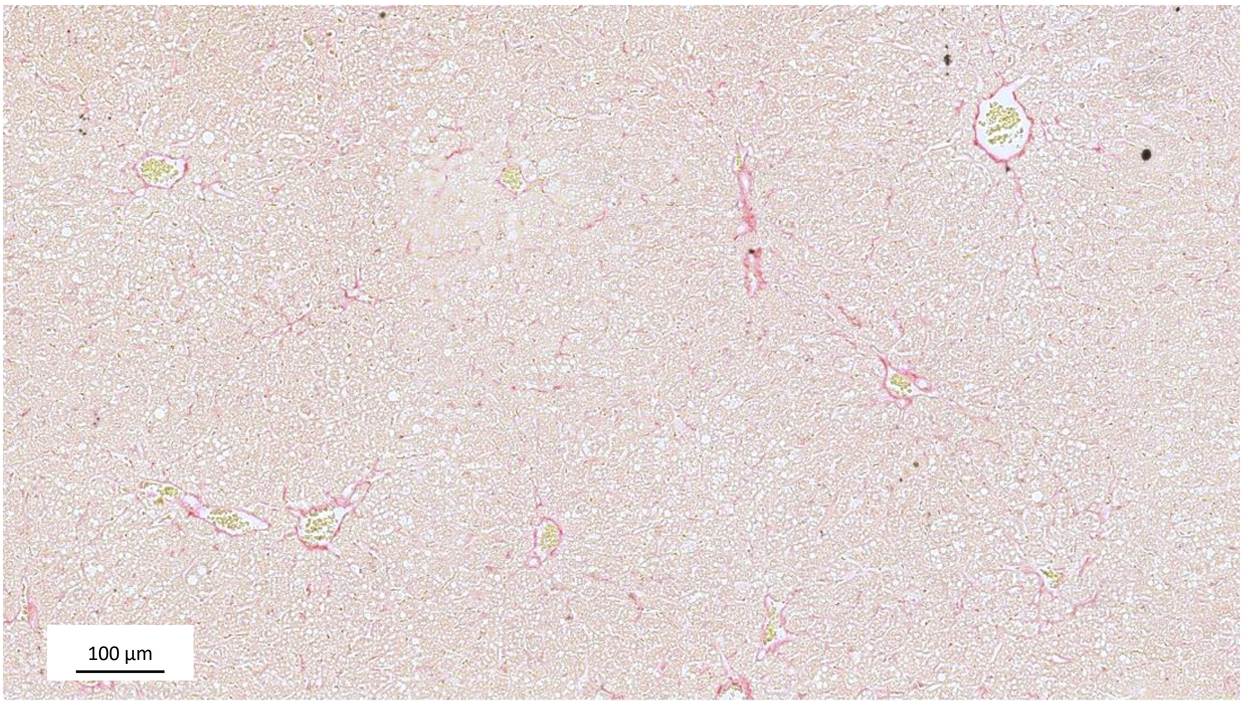

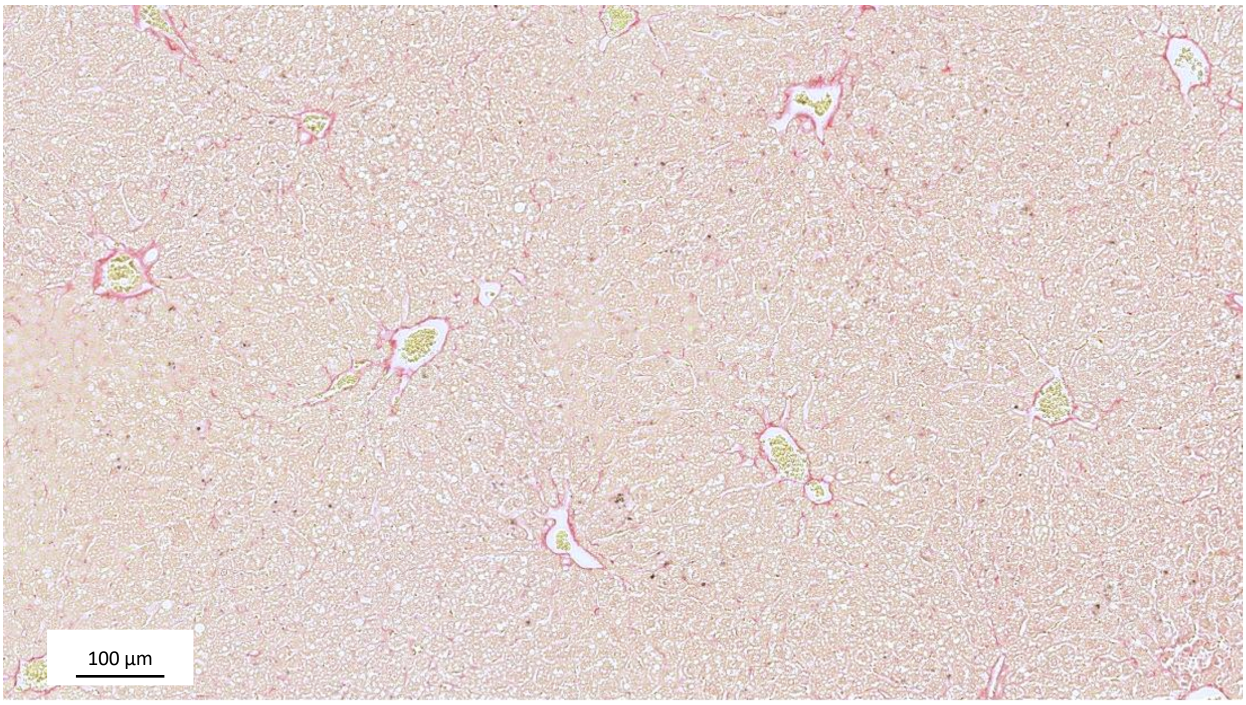

CDHFD-I-15

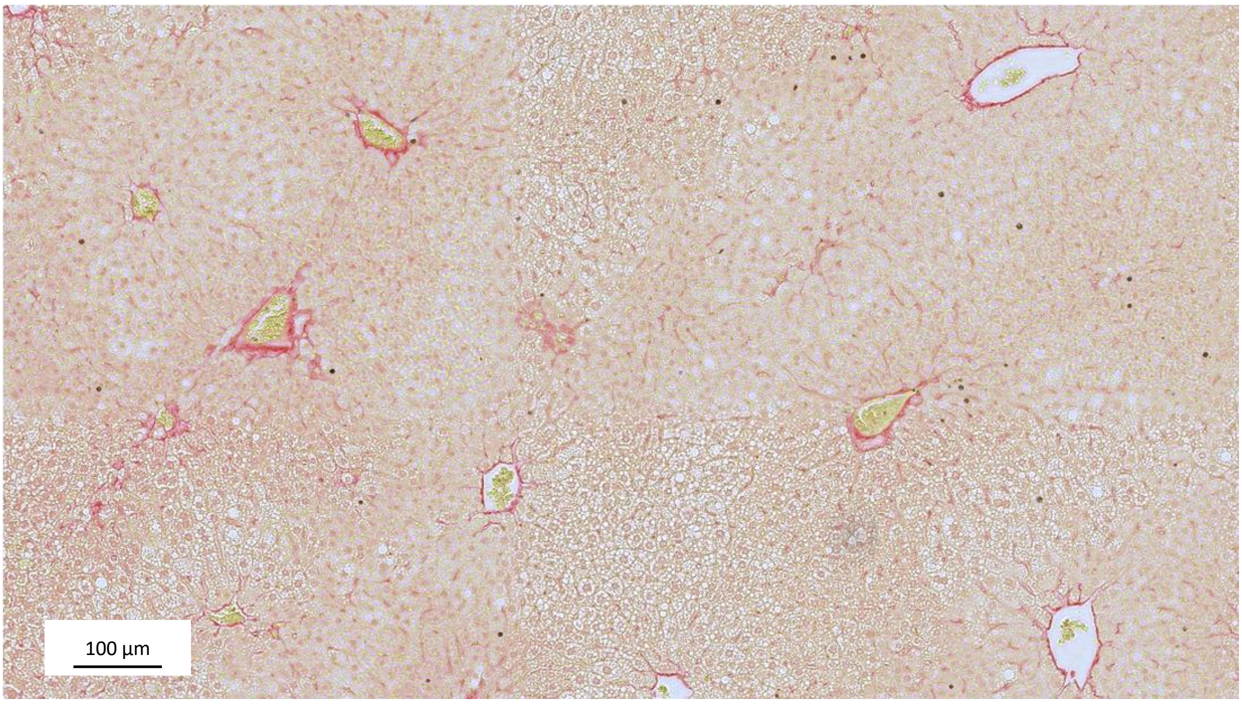

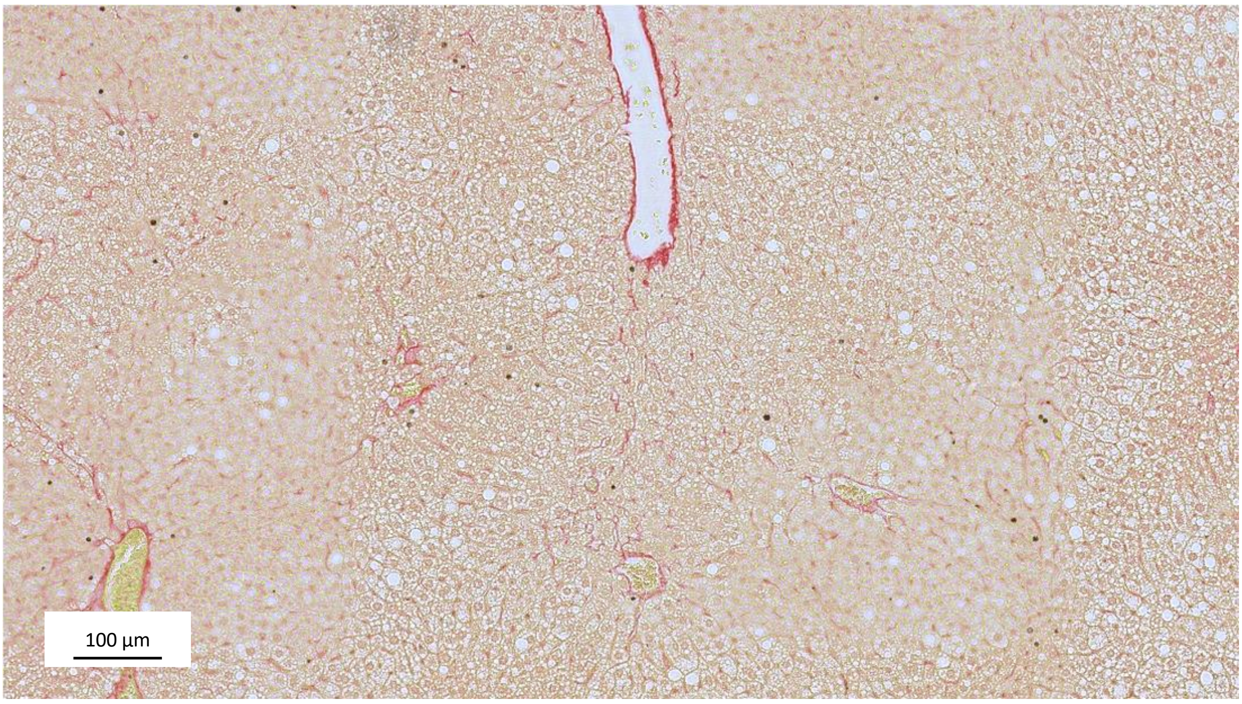

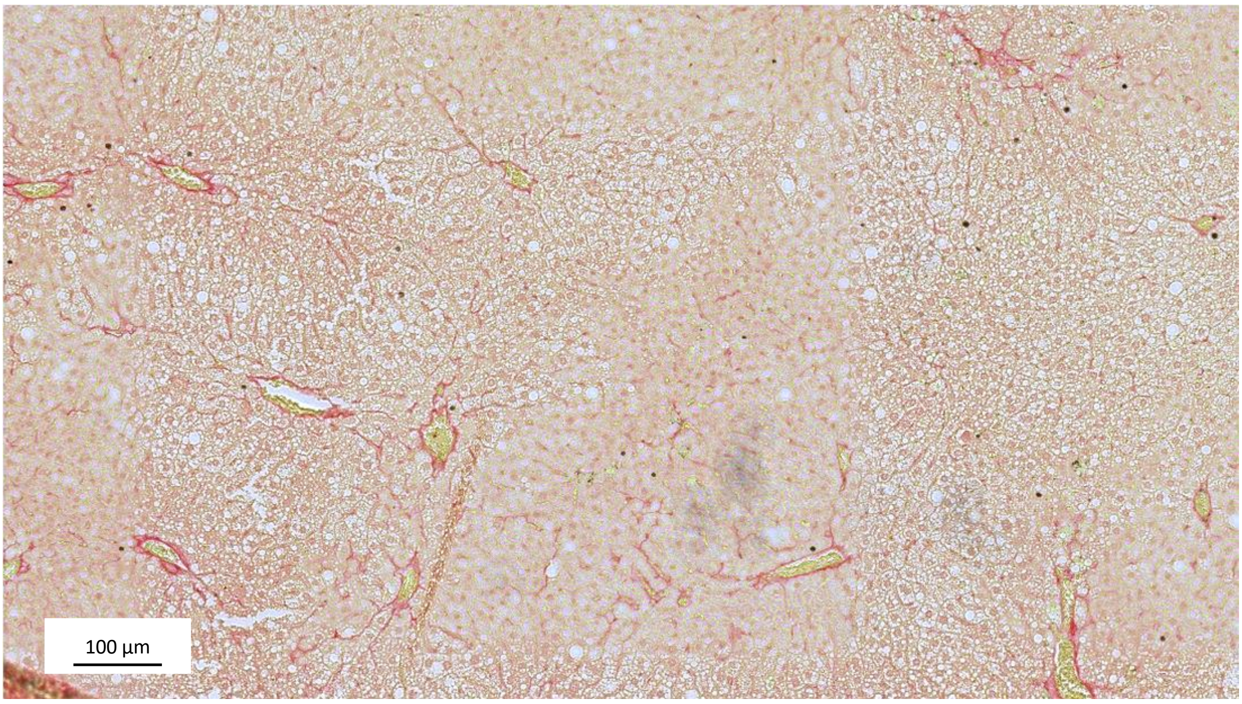

Sirius red Staining

CDHFD-C group

(15 mice were included)

CDHFD-C-1

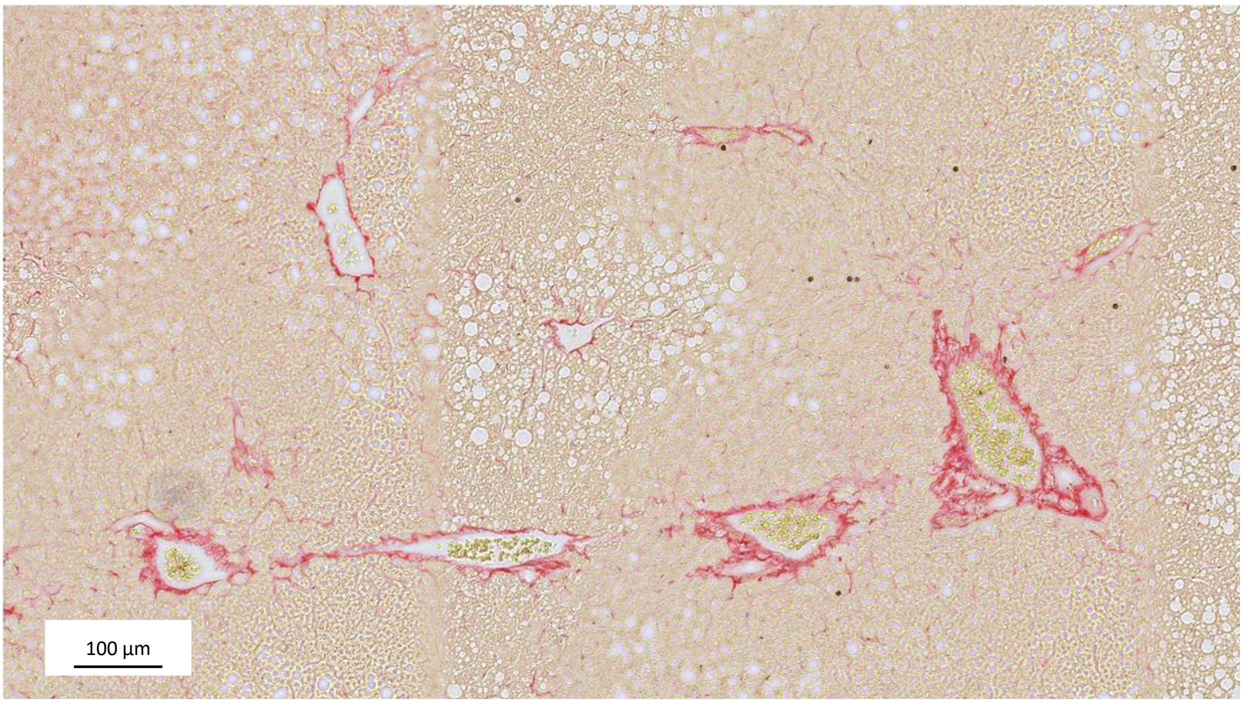

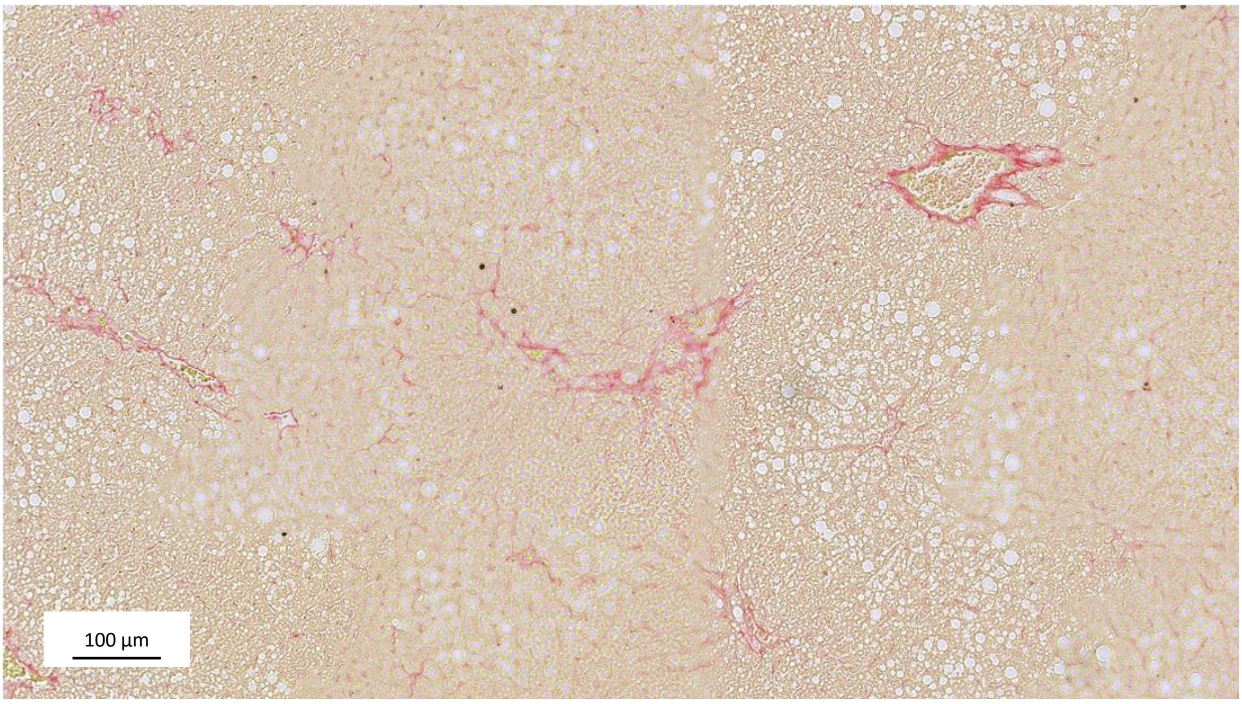

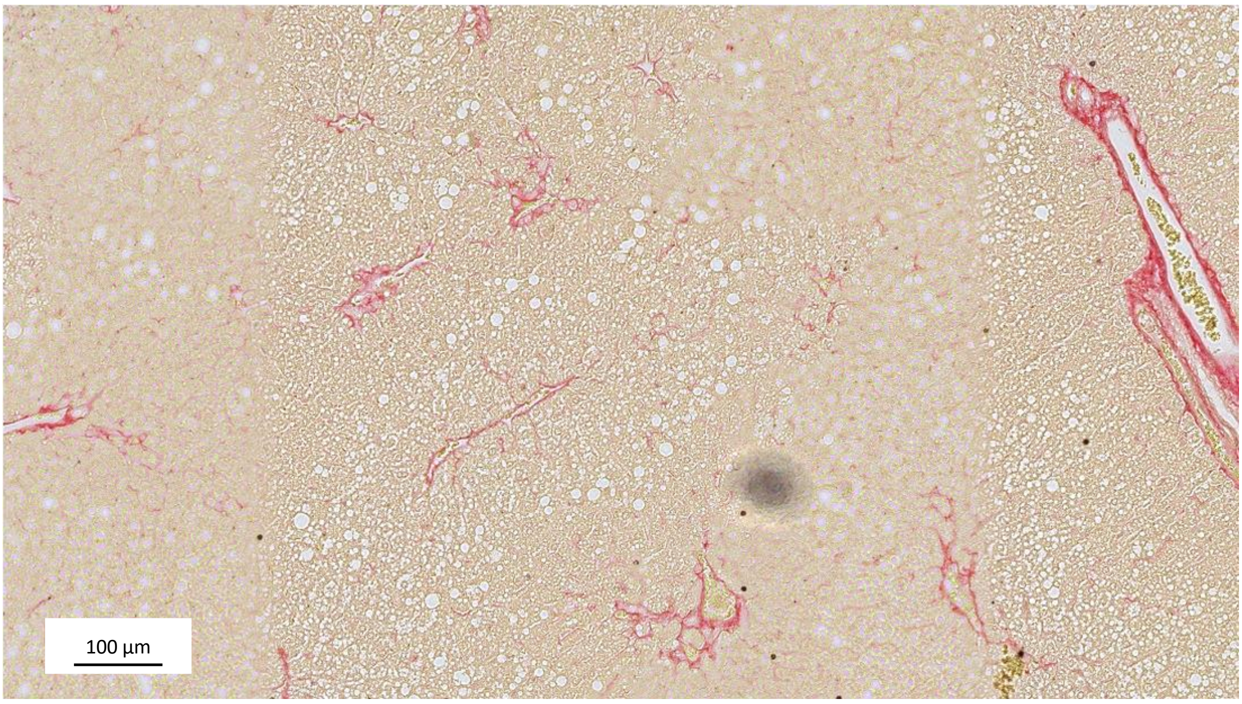

CDHFD-C-2

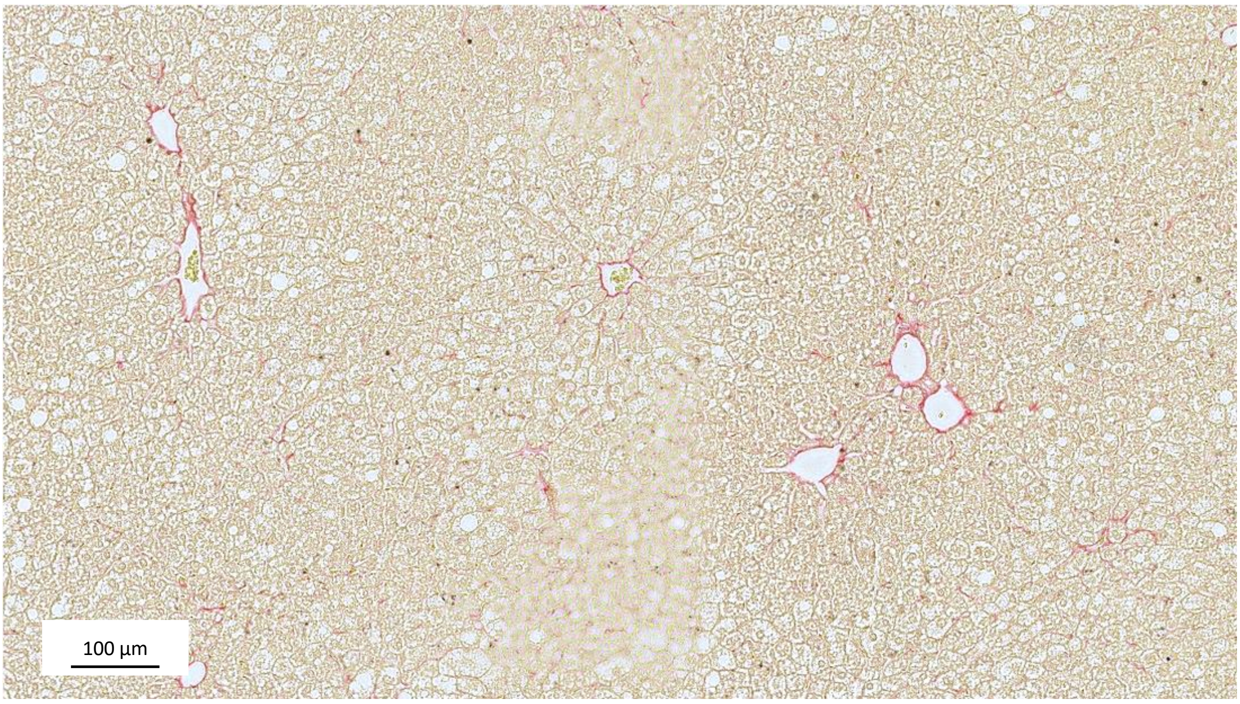

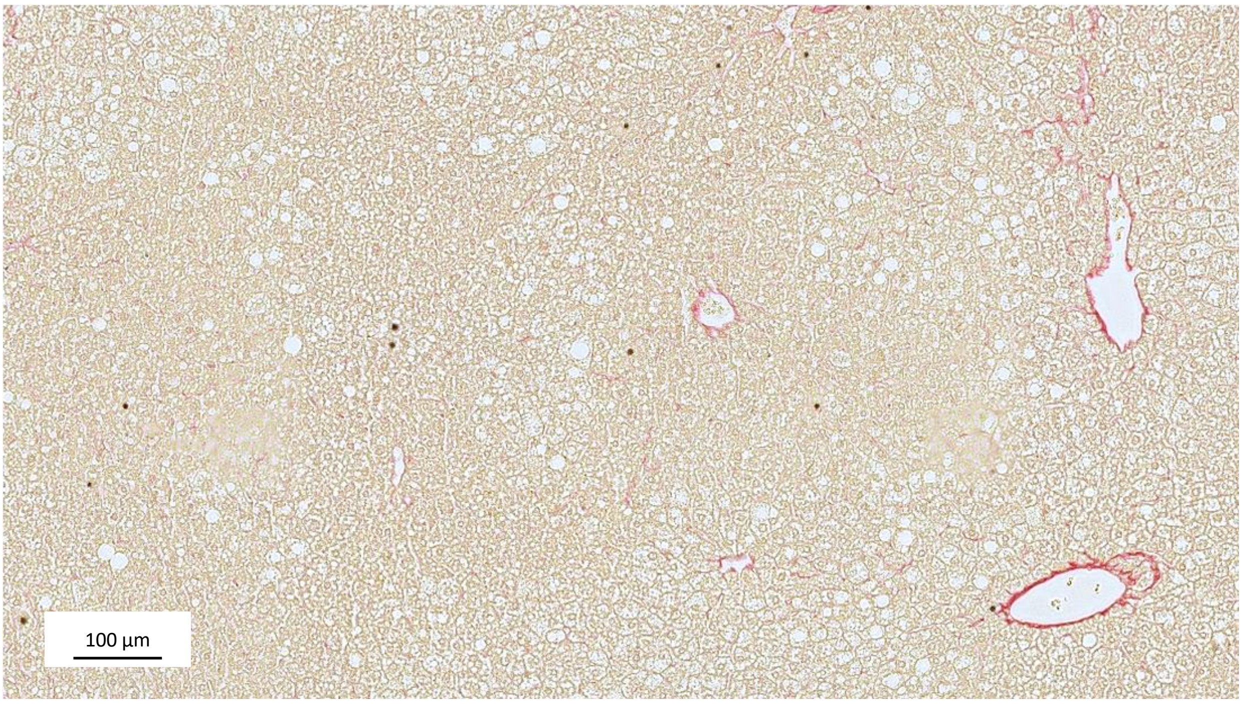

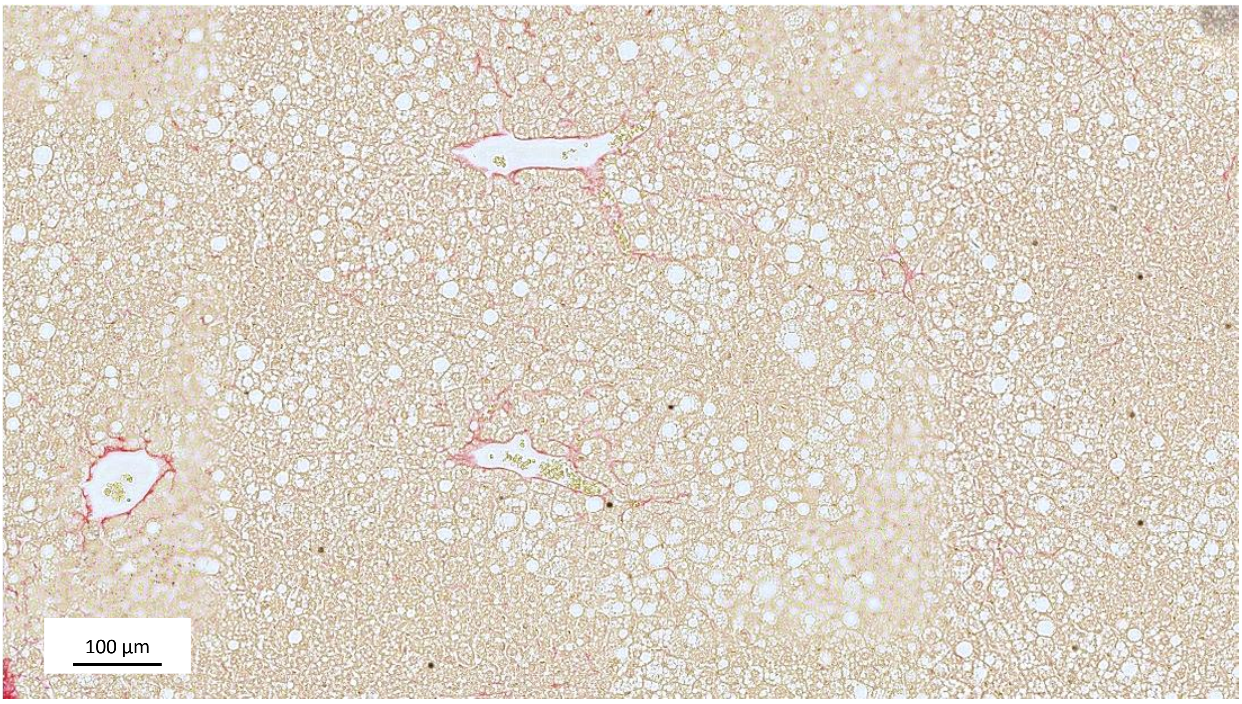

CDHFD-C-3

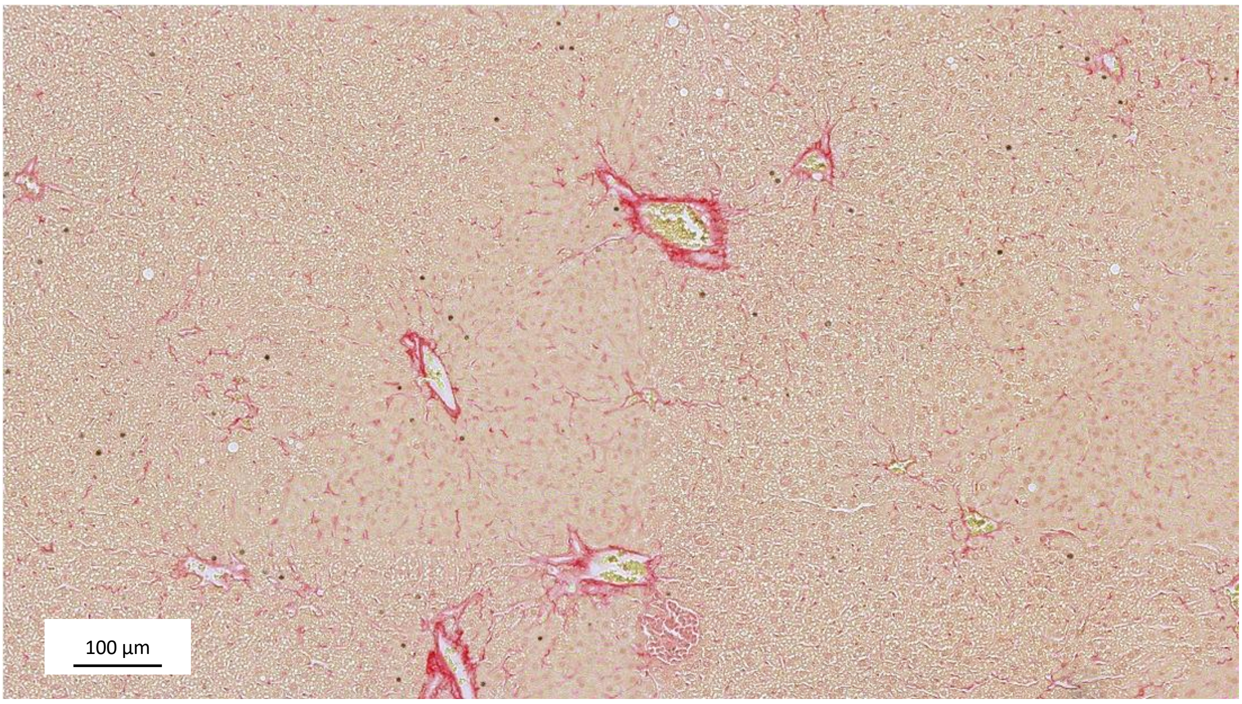

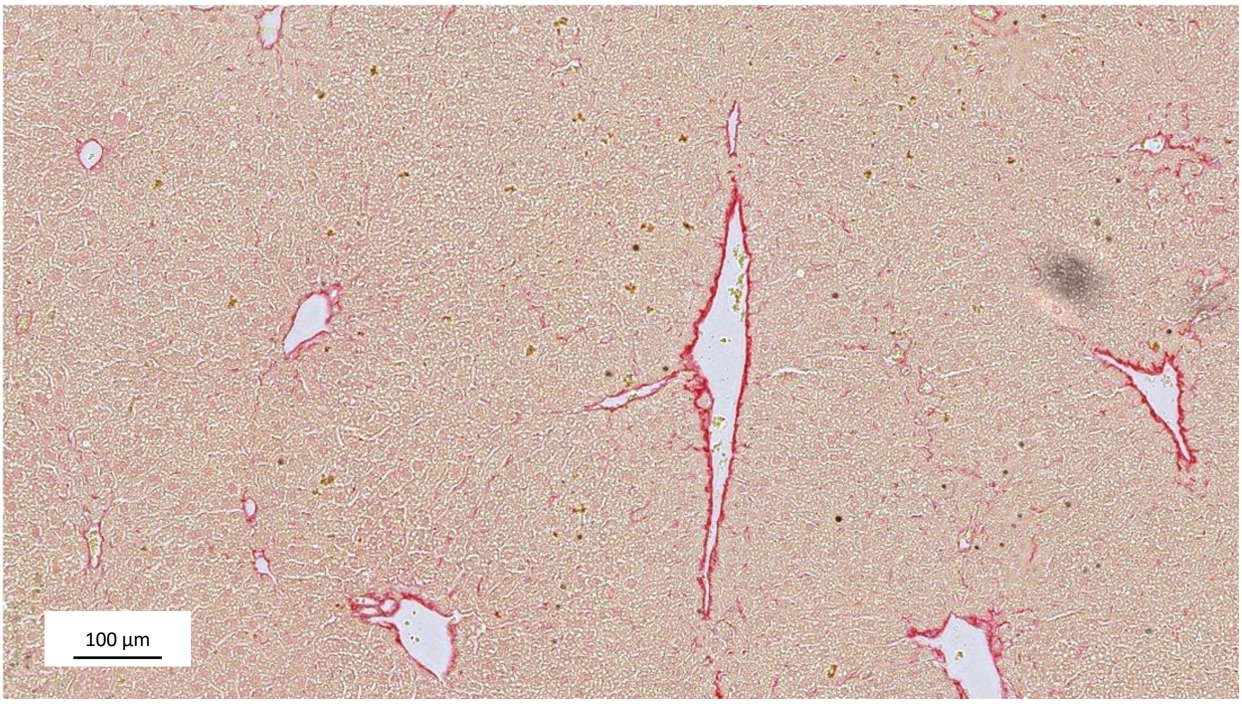

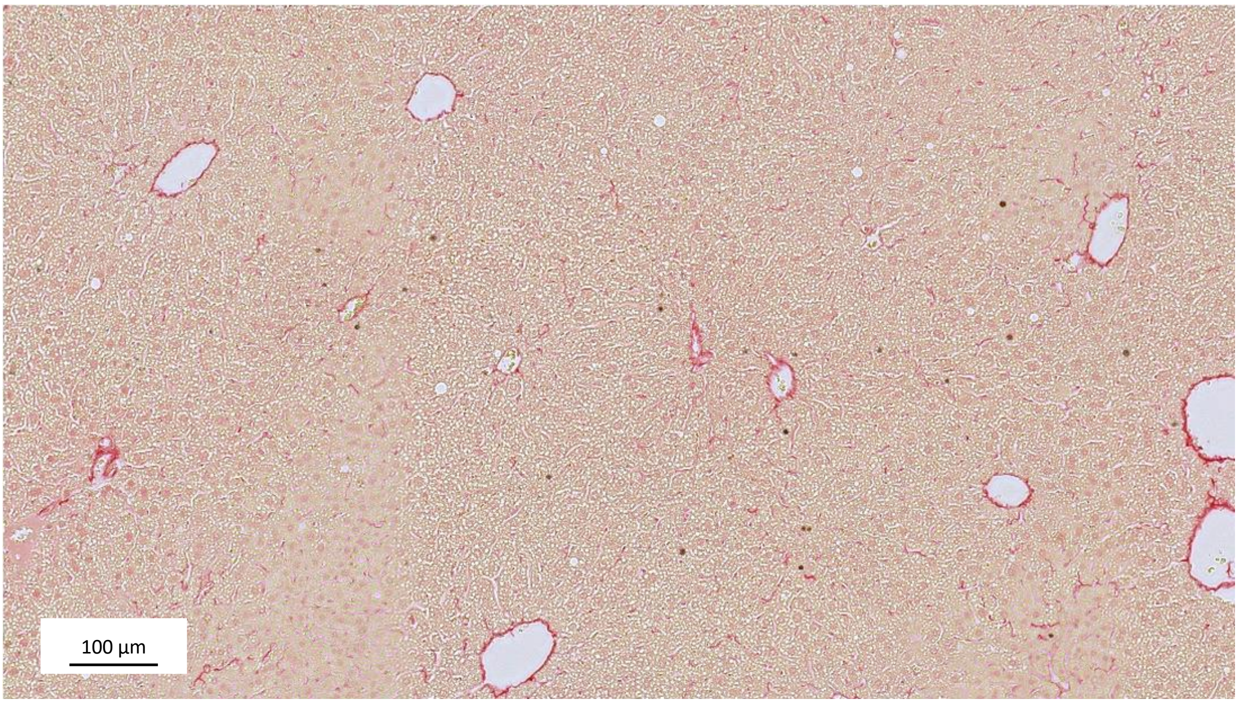

CDHFD-C-4

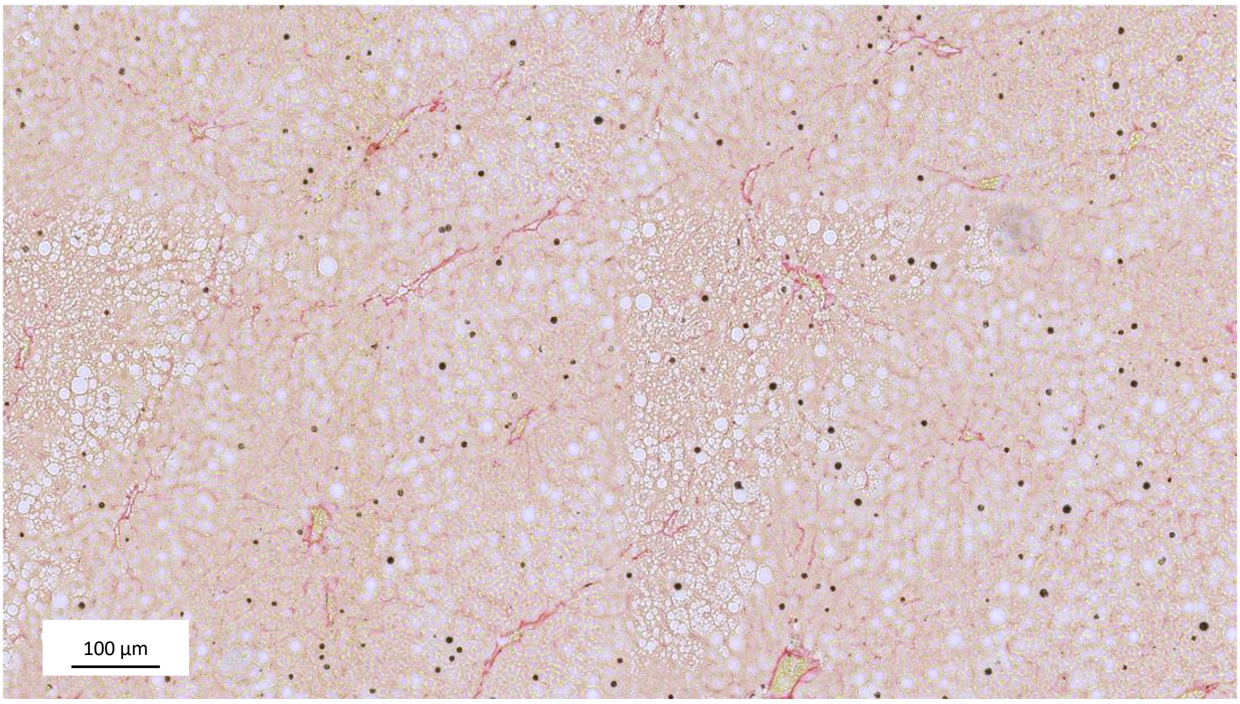

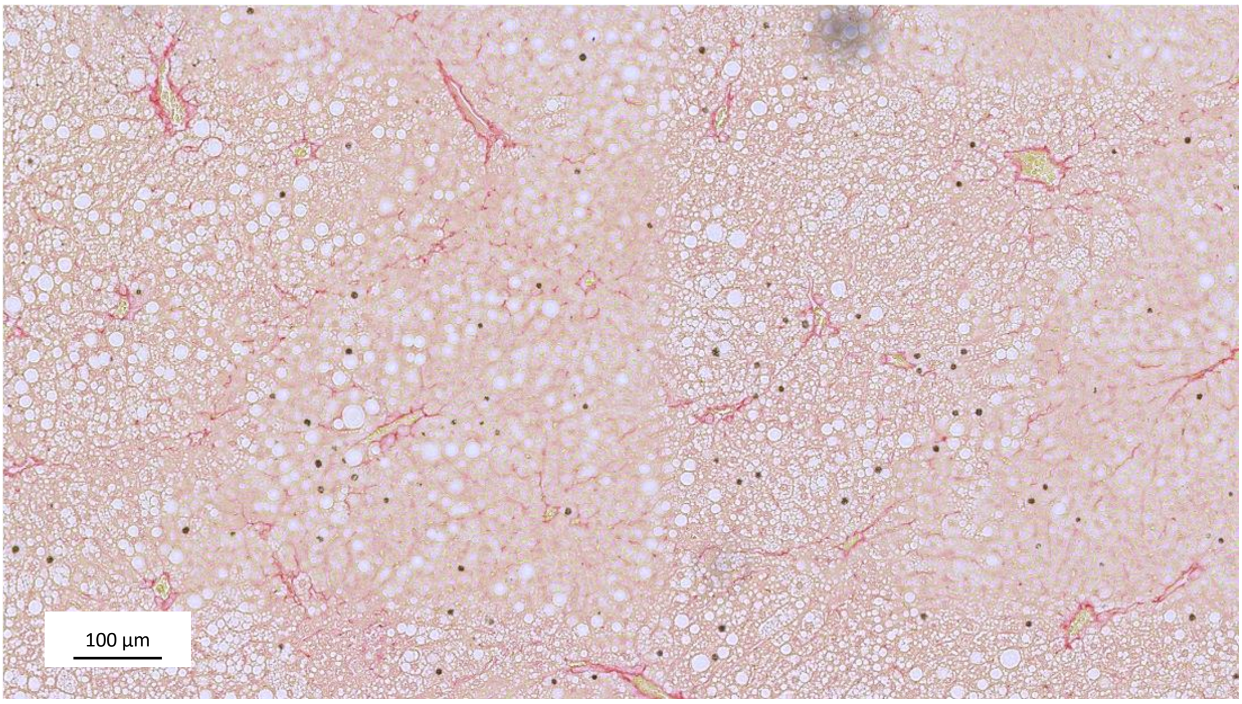

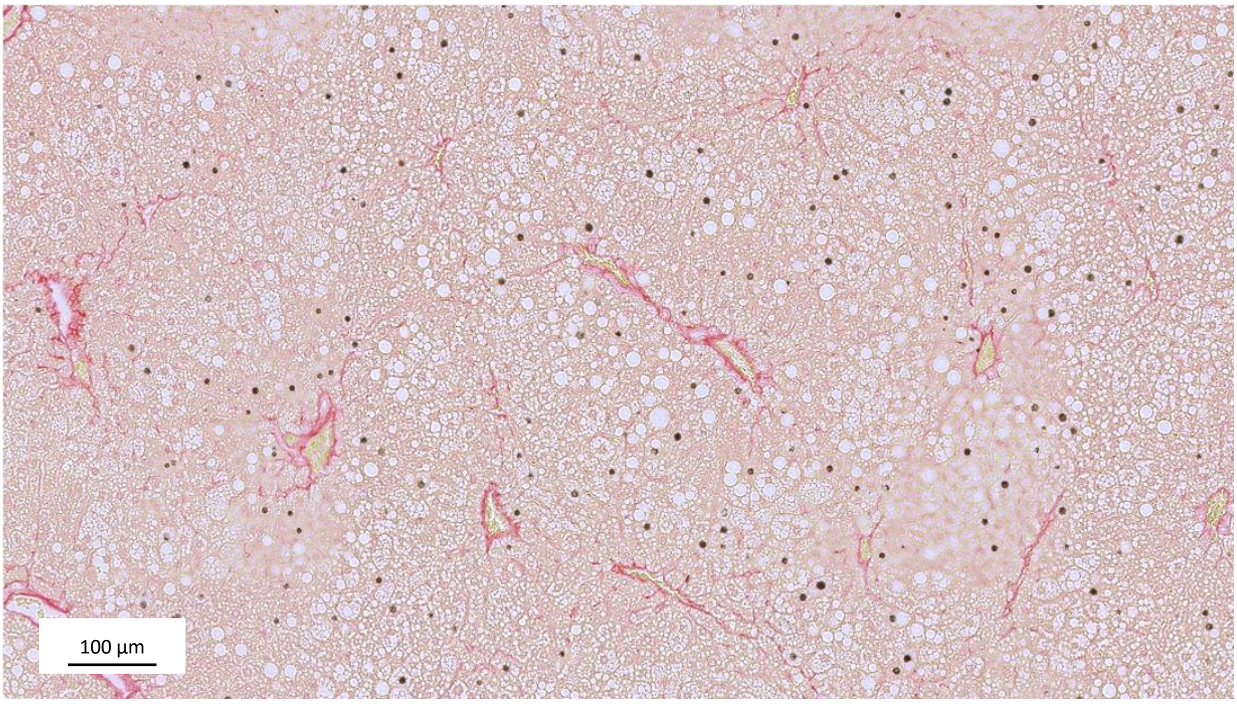

CDHFD-C-5

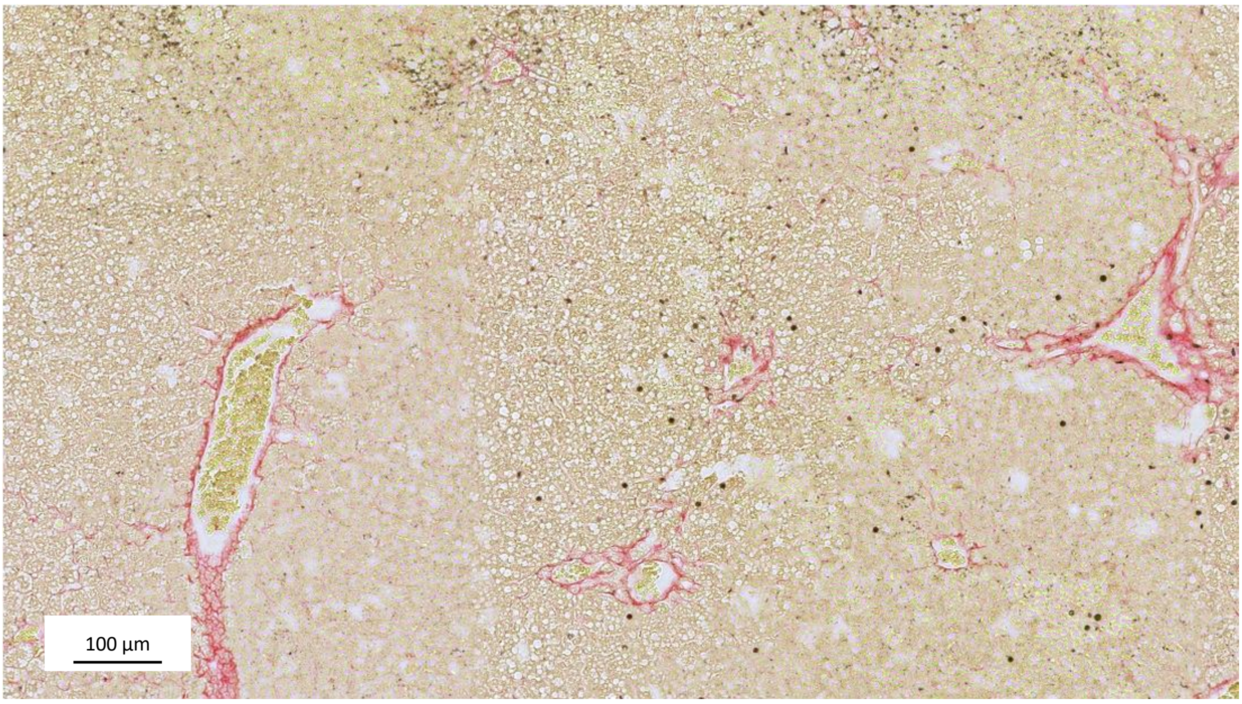

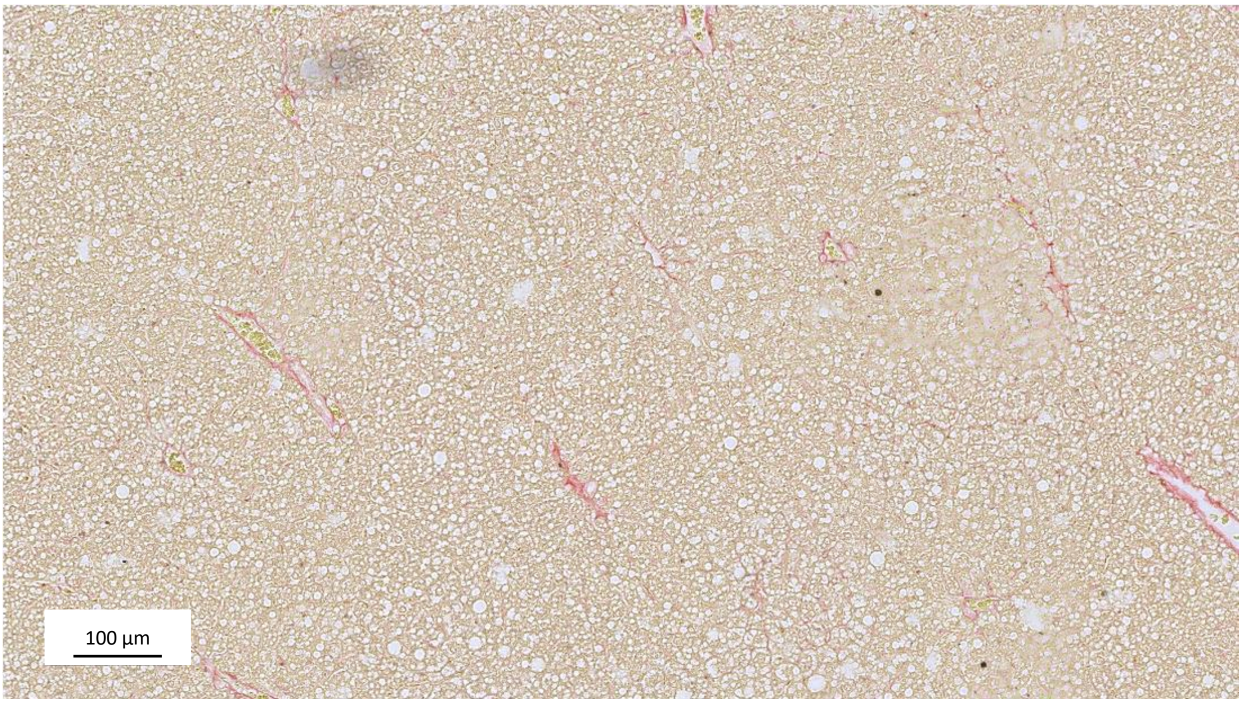

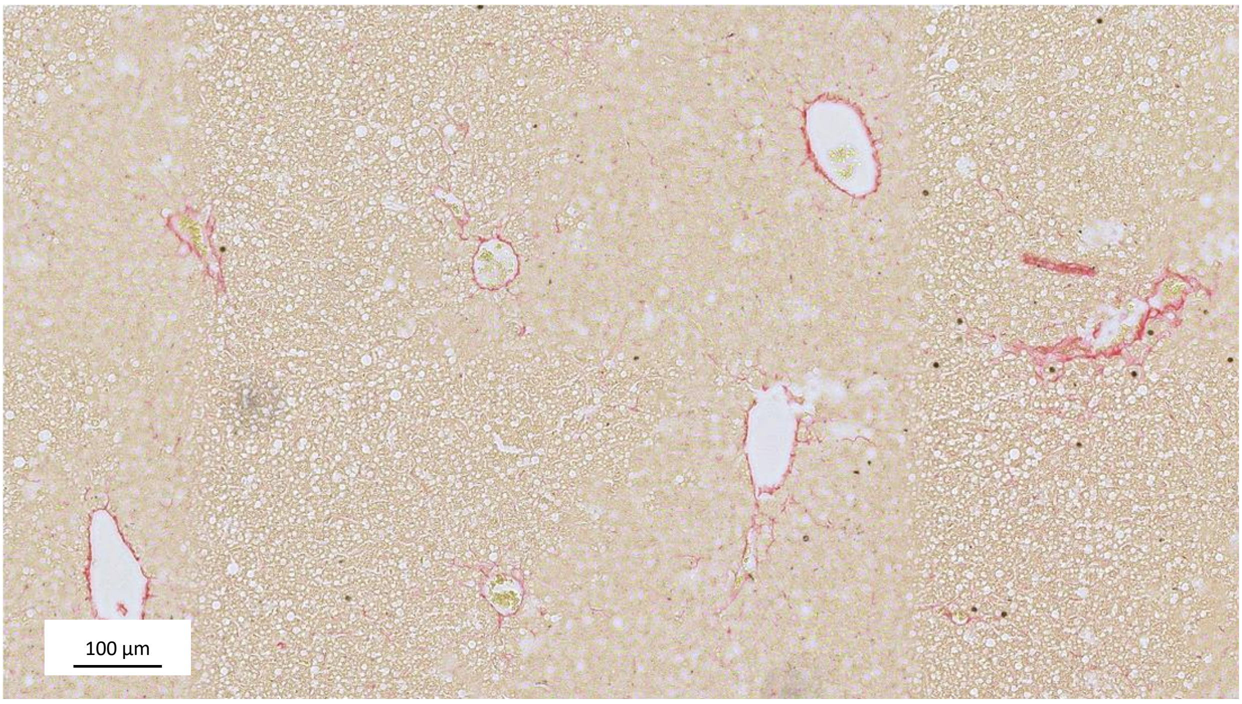

CDHFD-C-6

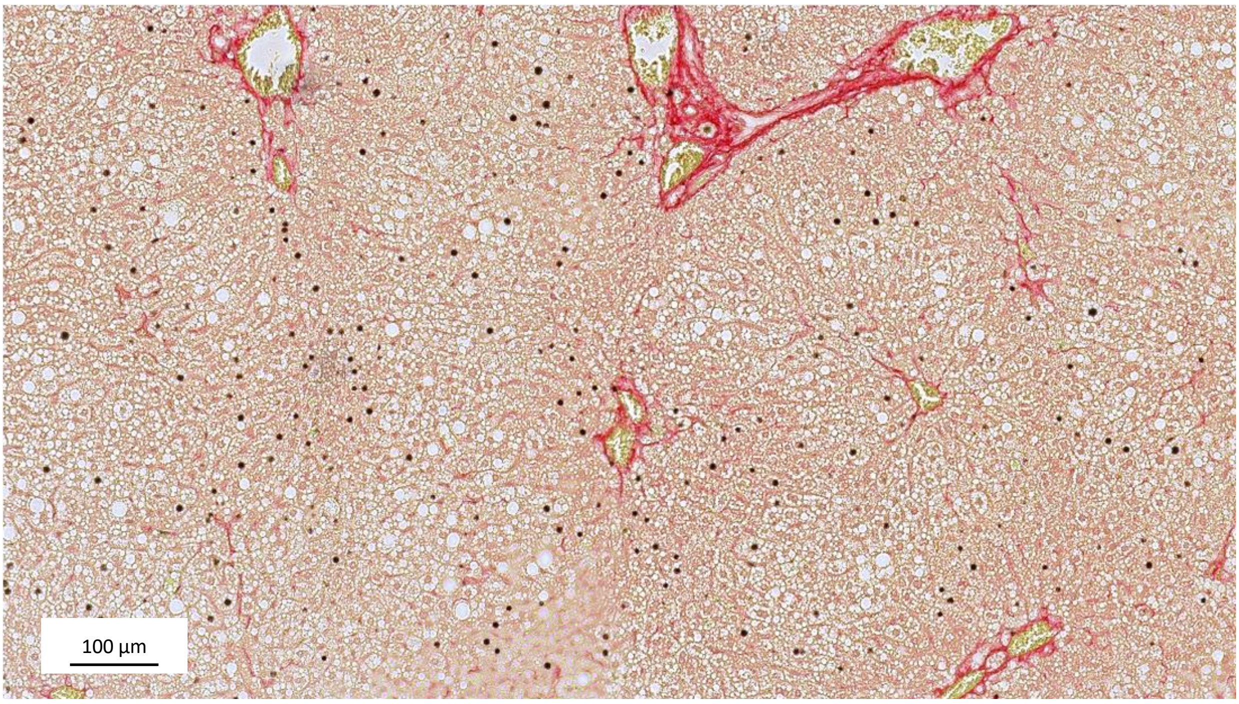

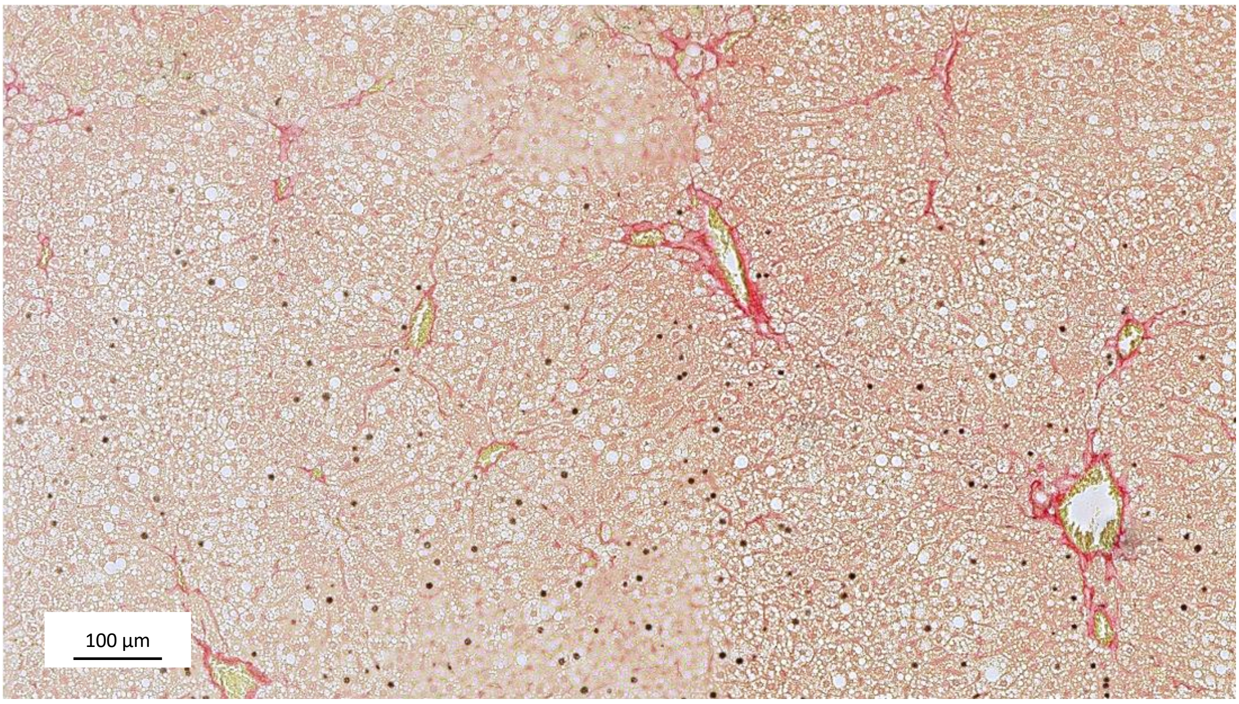

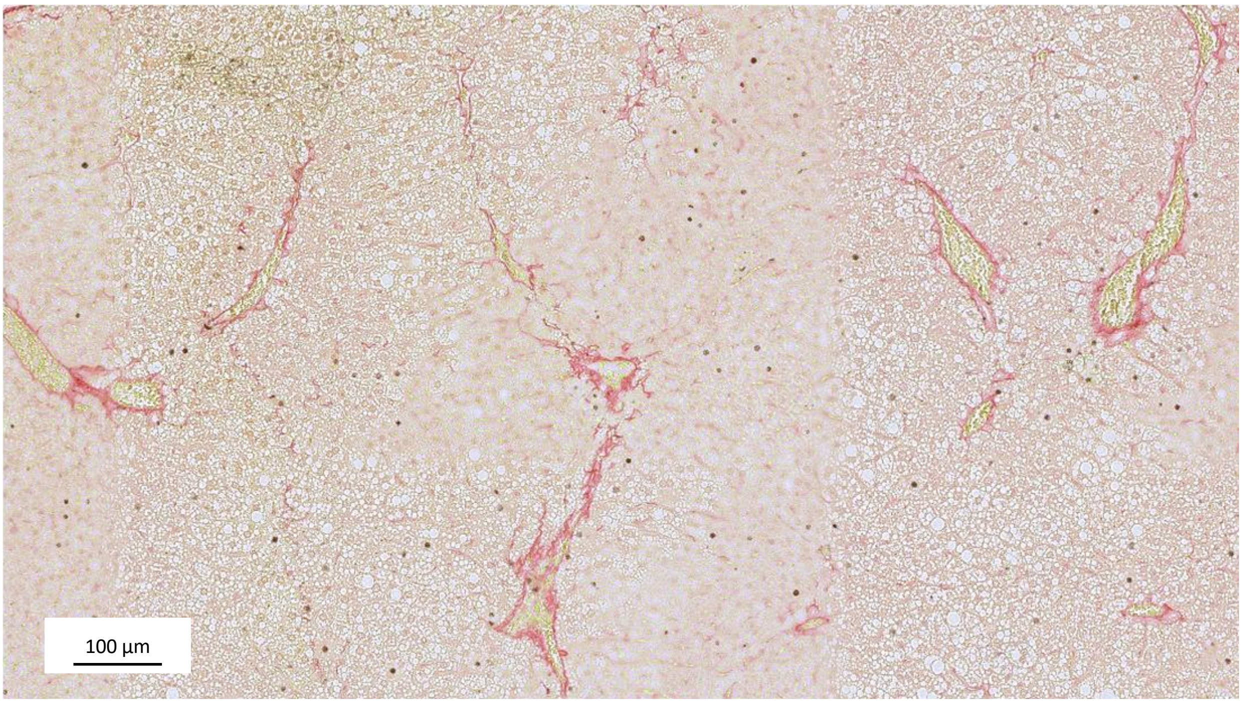

CDHFD-C-7

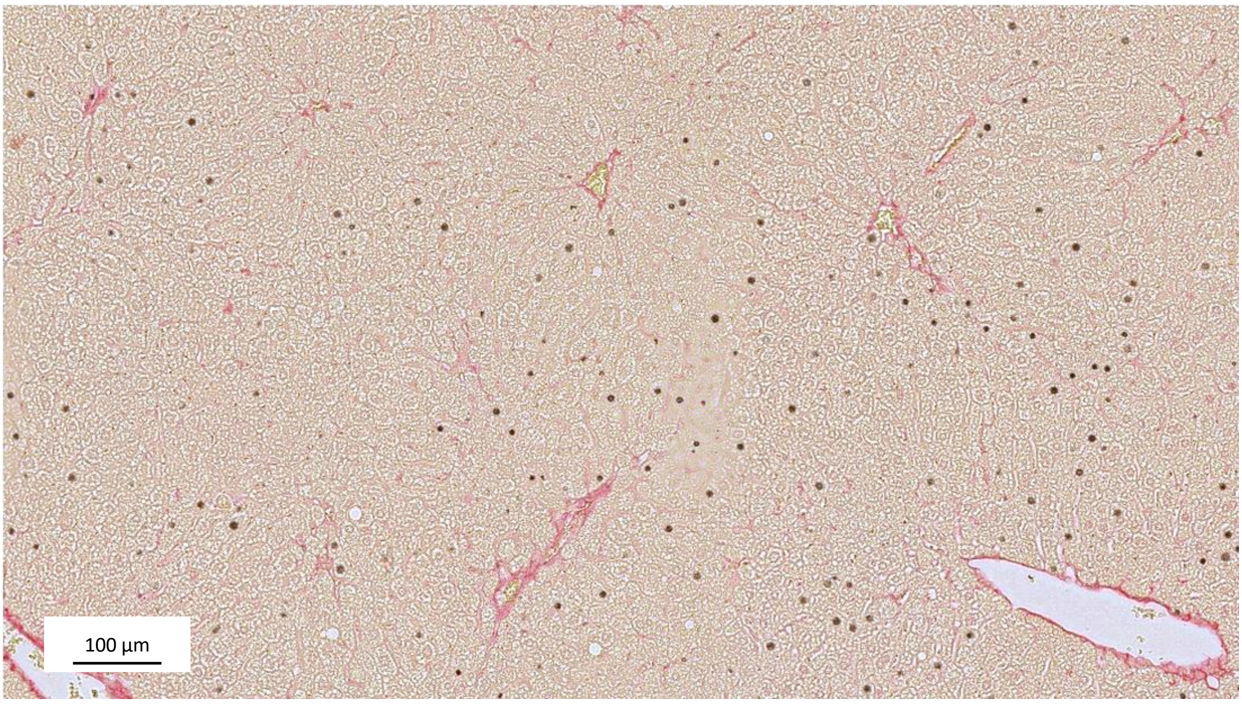

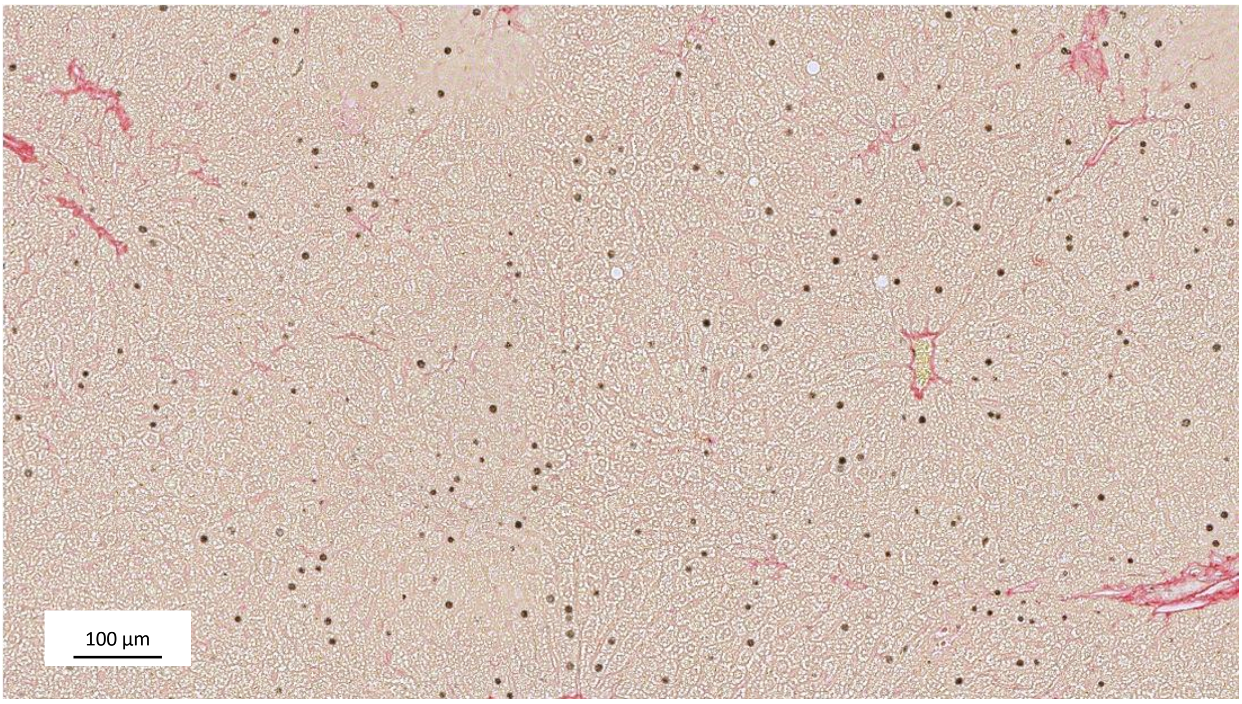

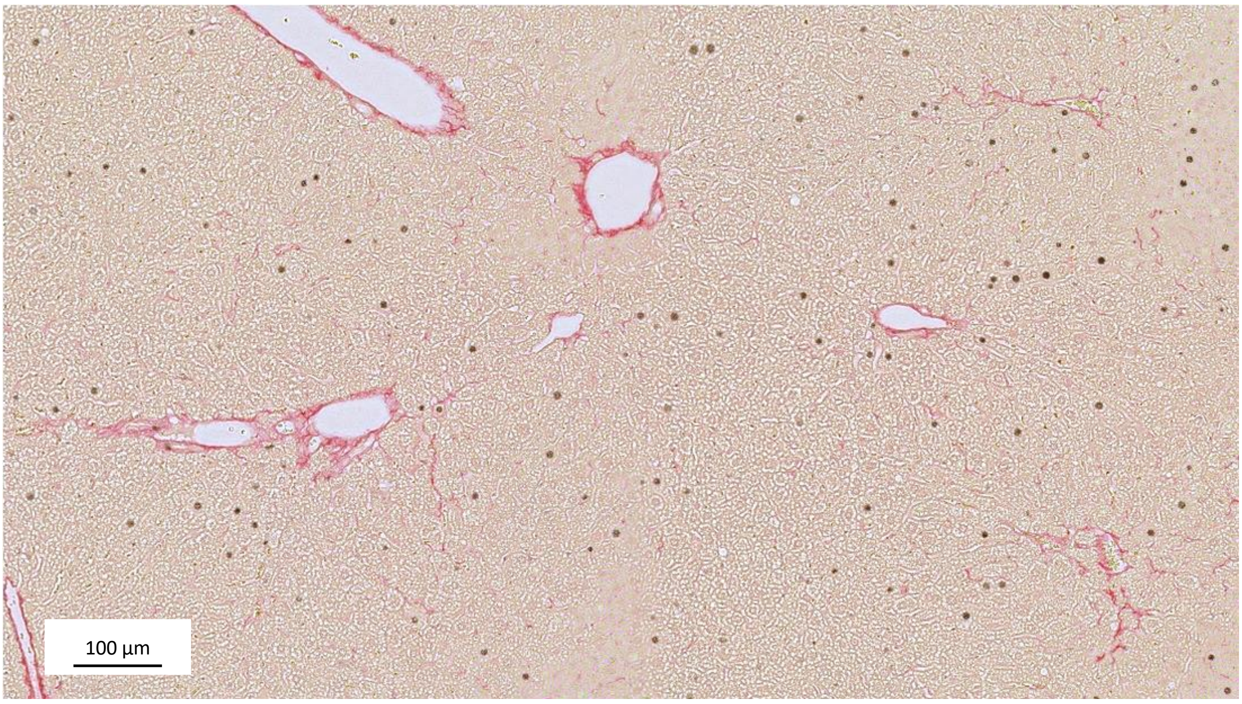

CDHFD-C-8

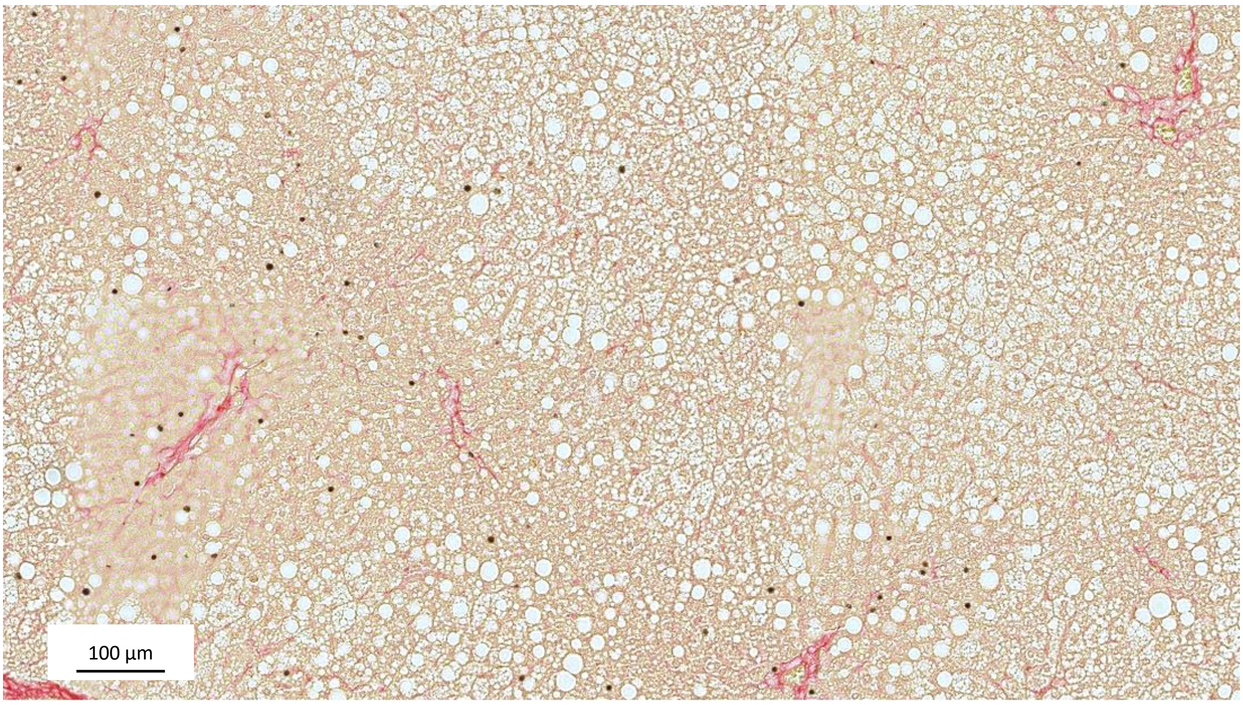

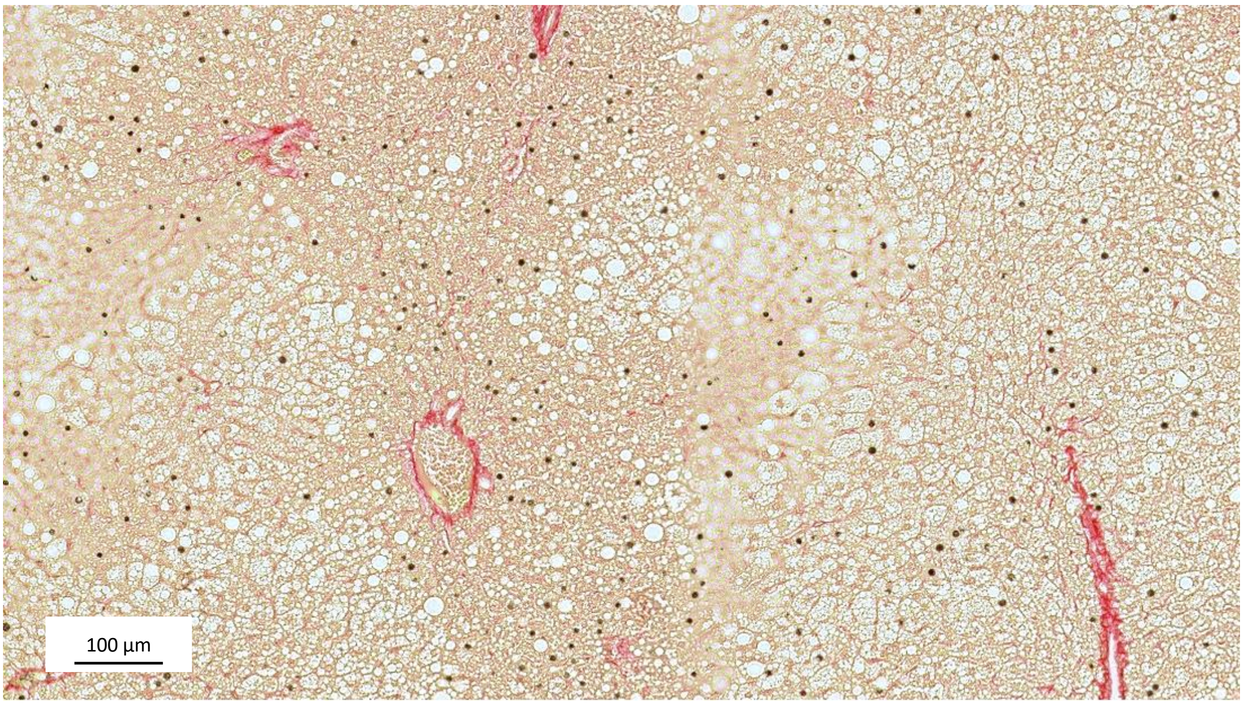

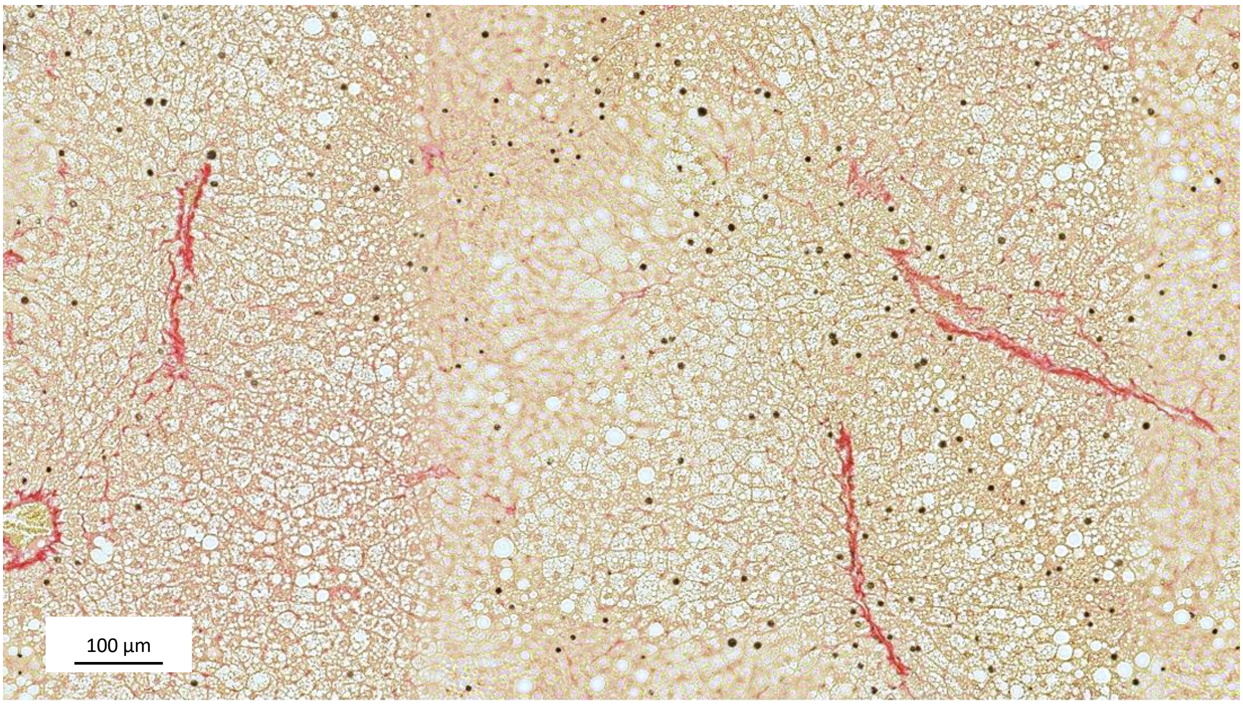

CDHFD-C-9

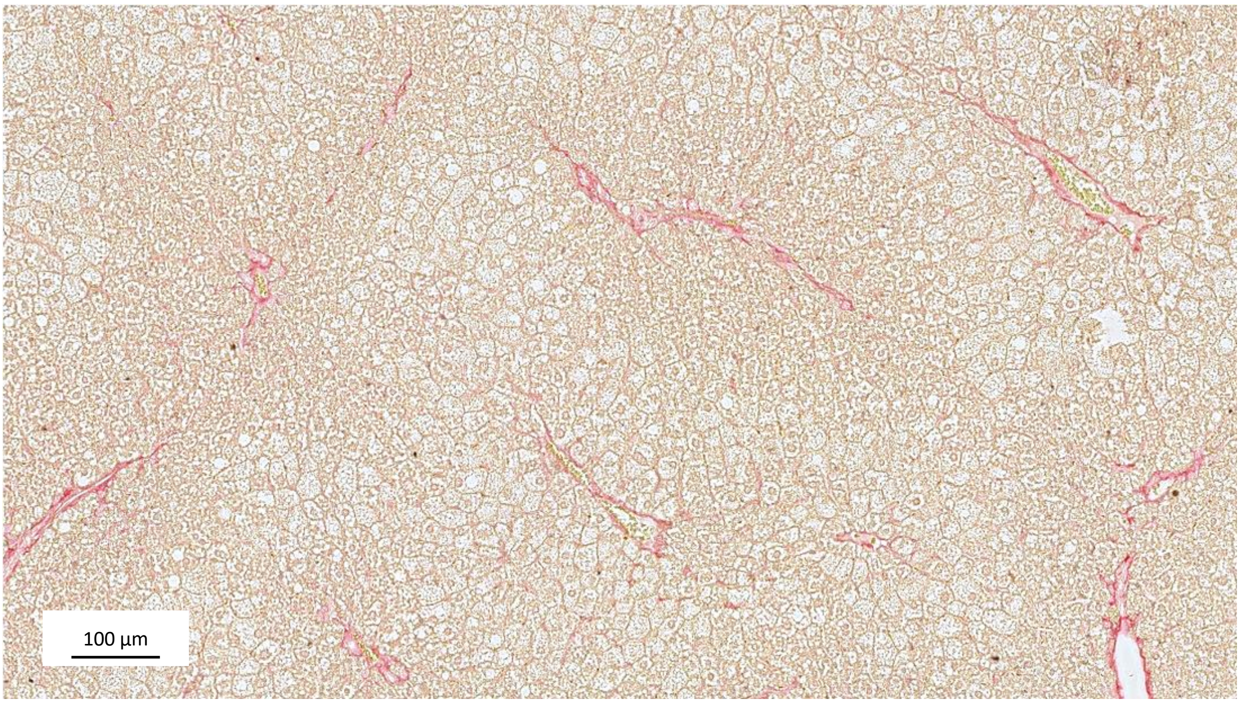

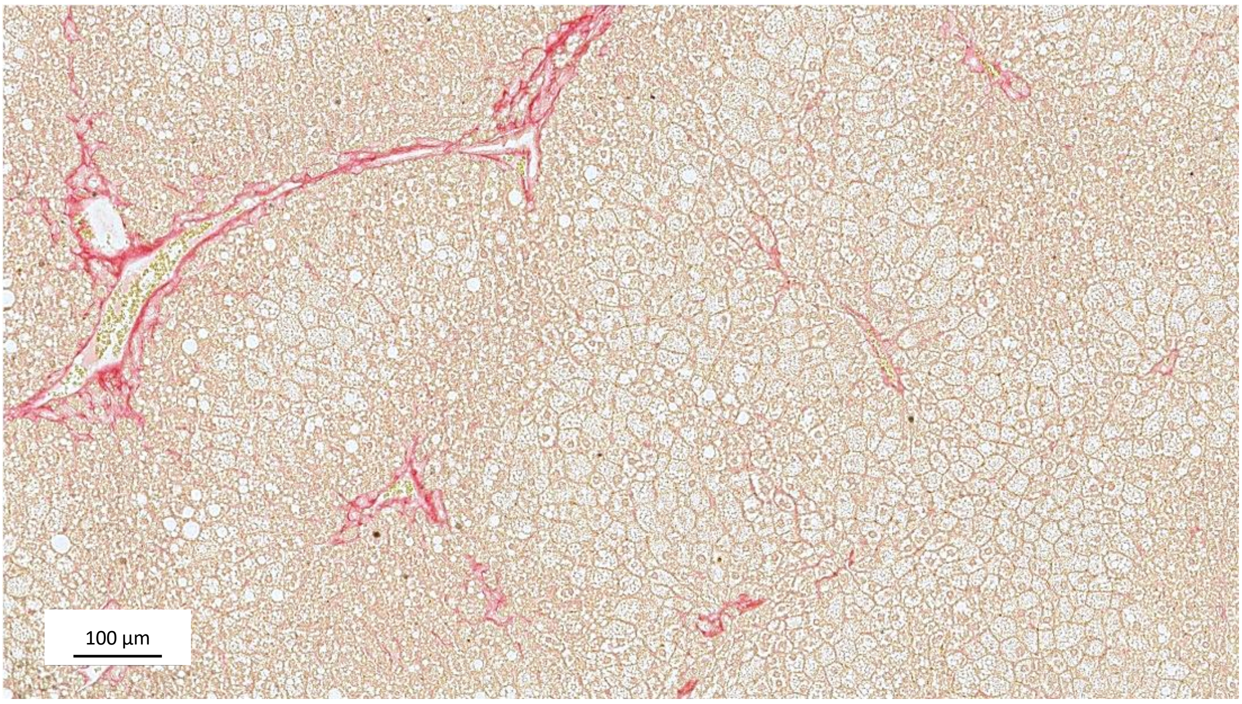

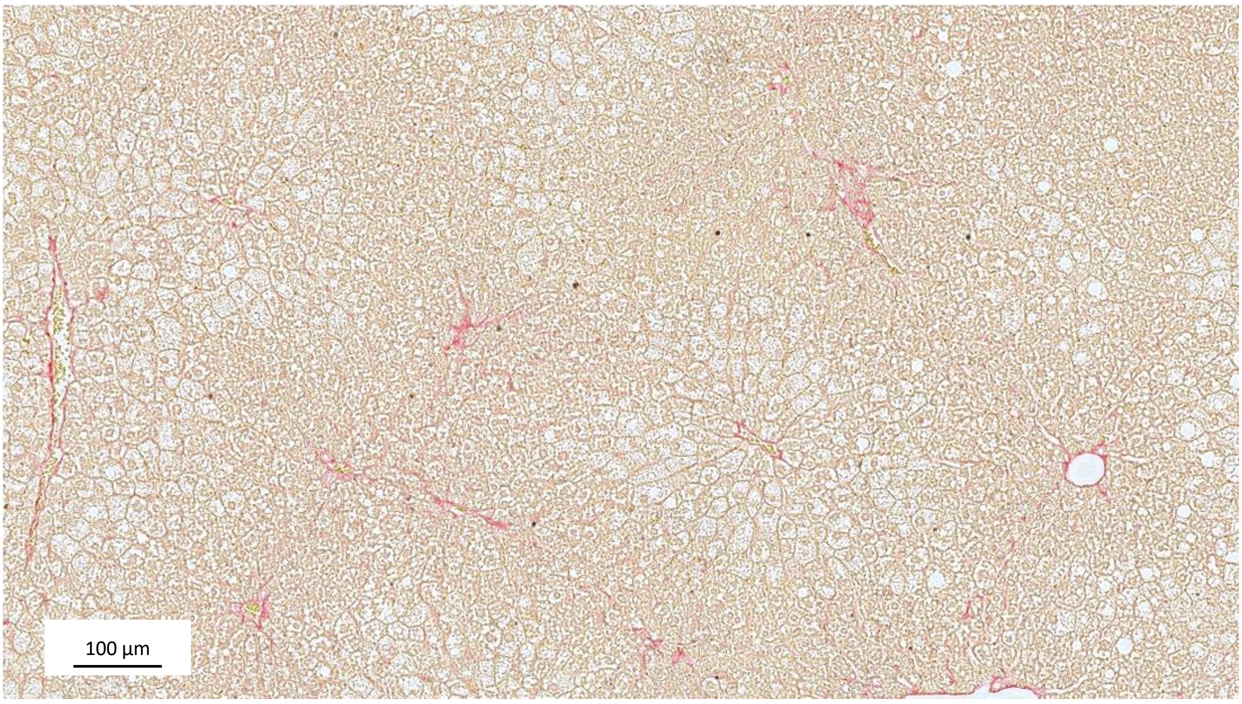

CDHFD-C-10

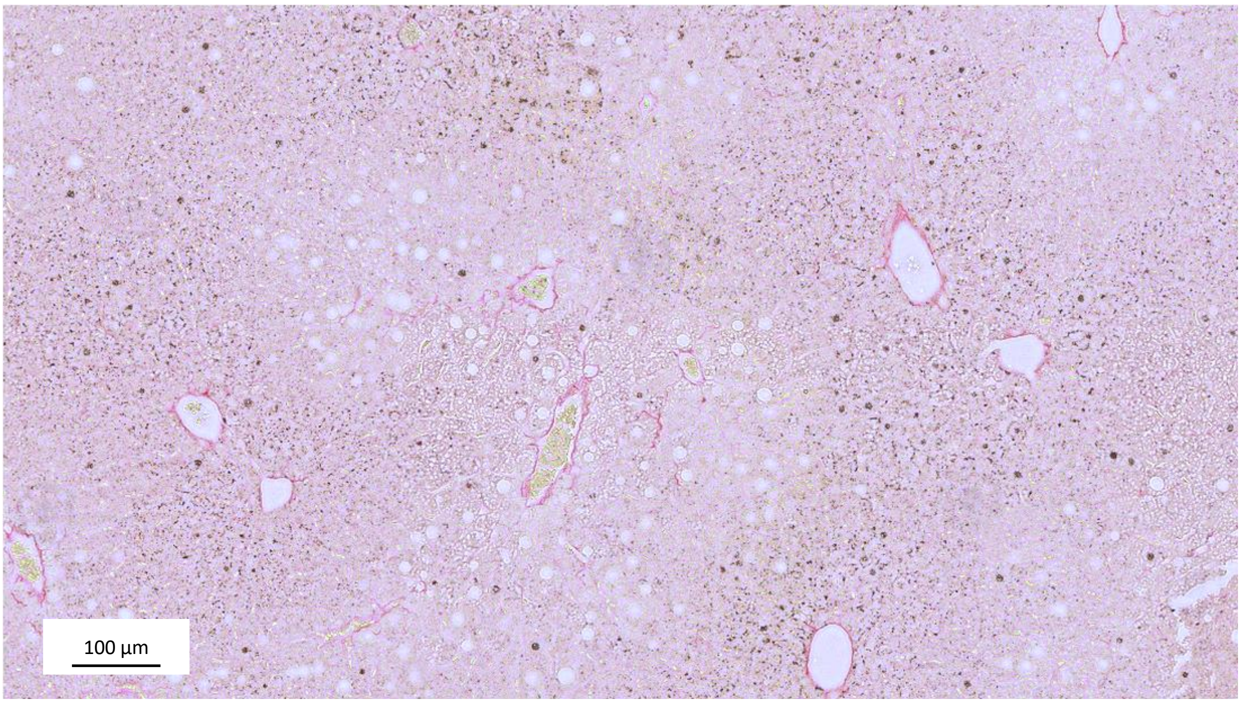

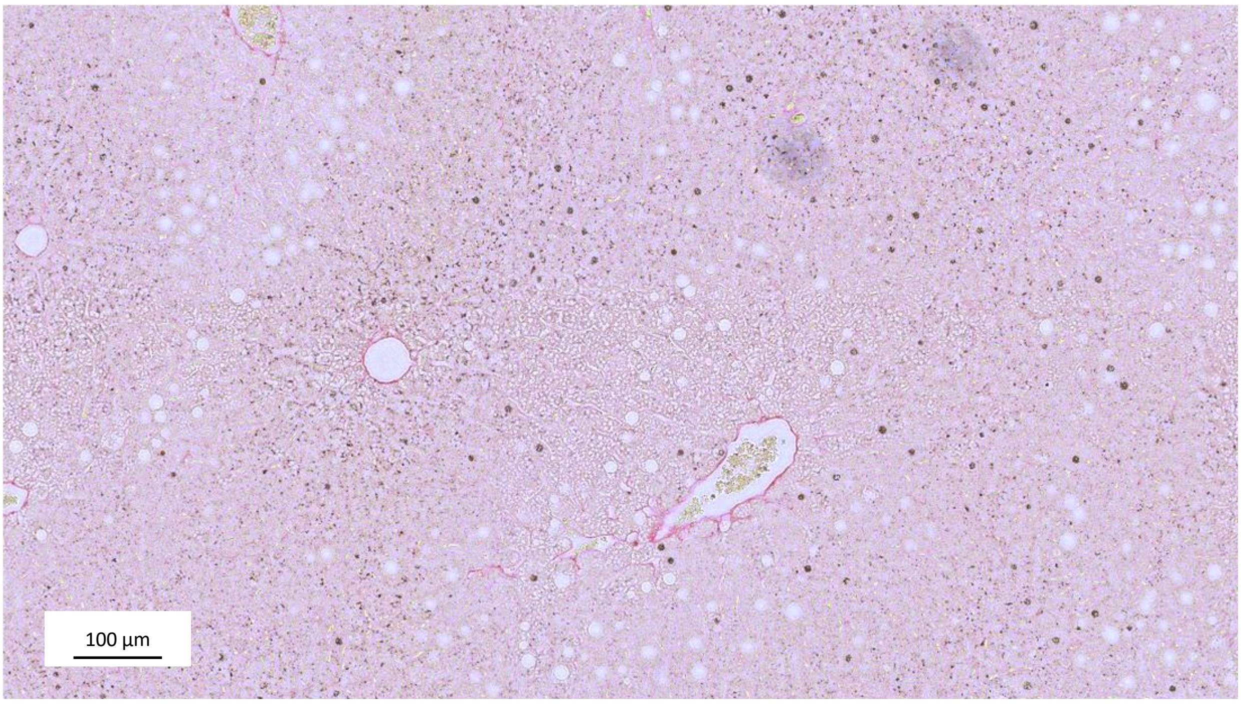

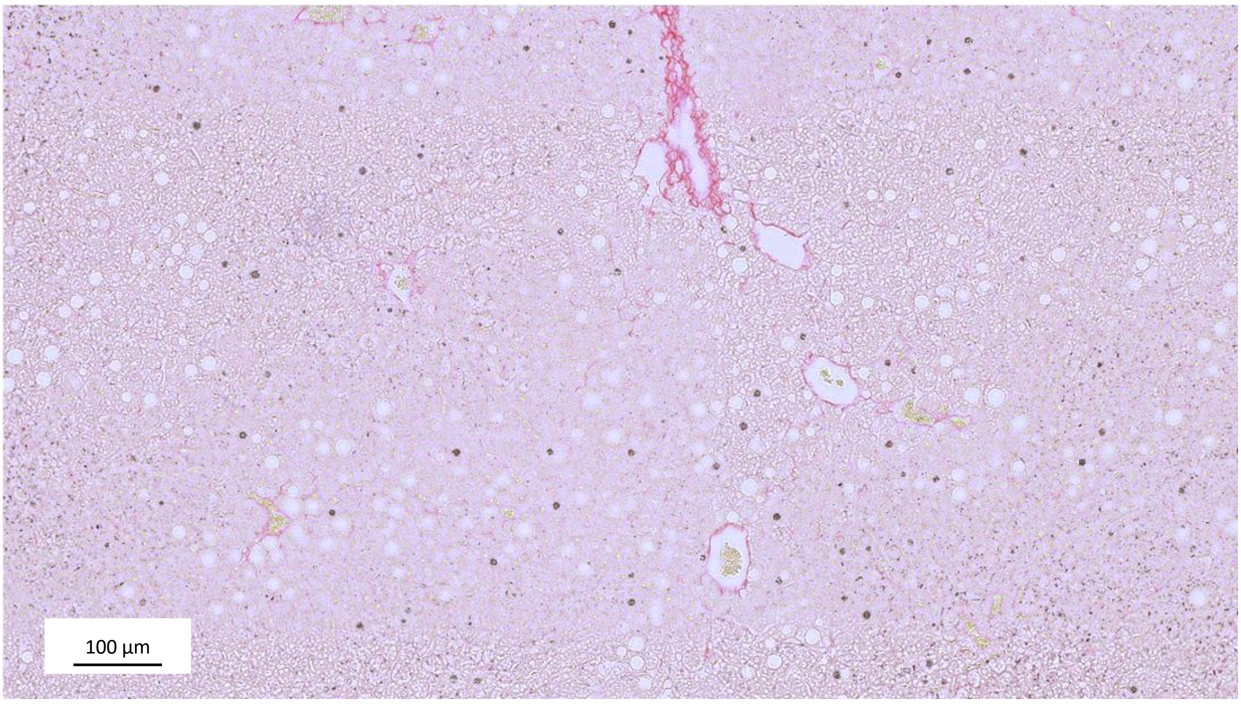

CDHFD-C-11

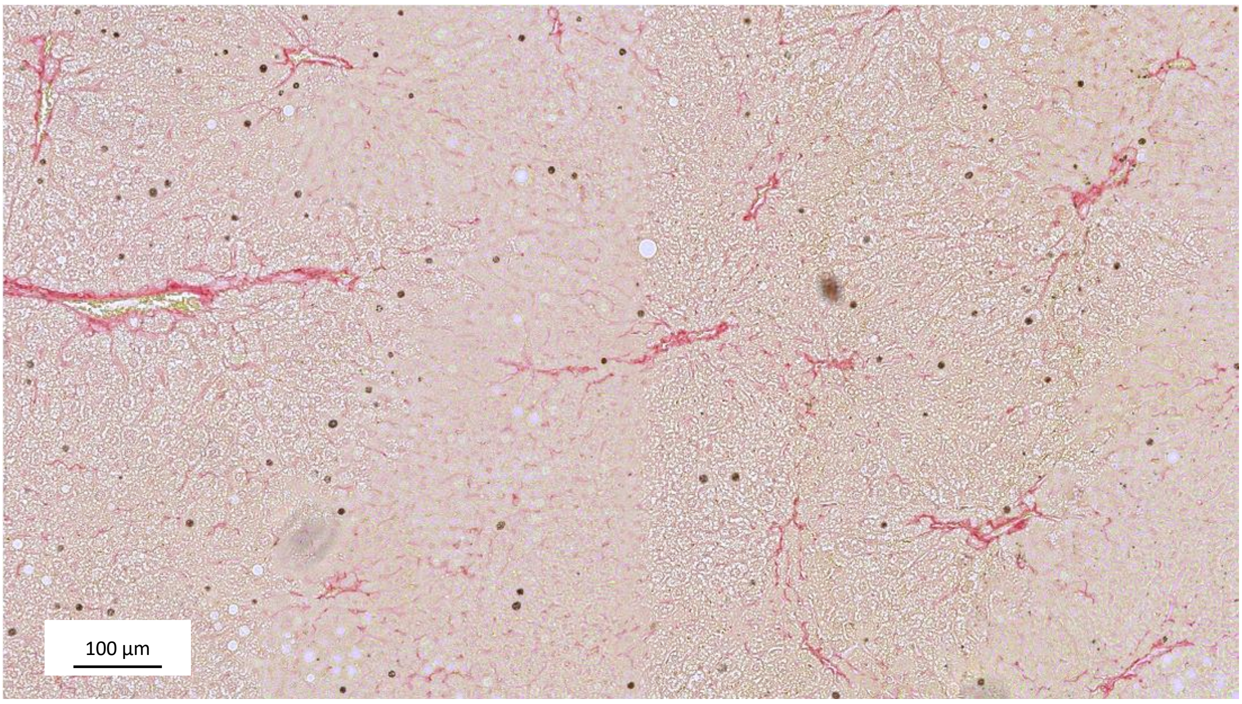

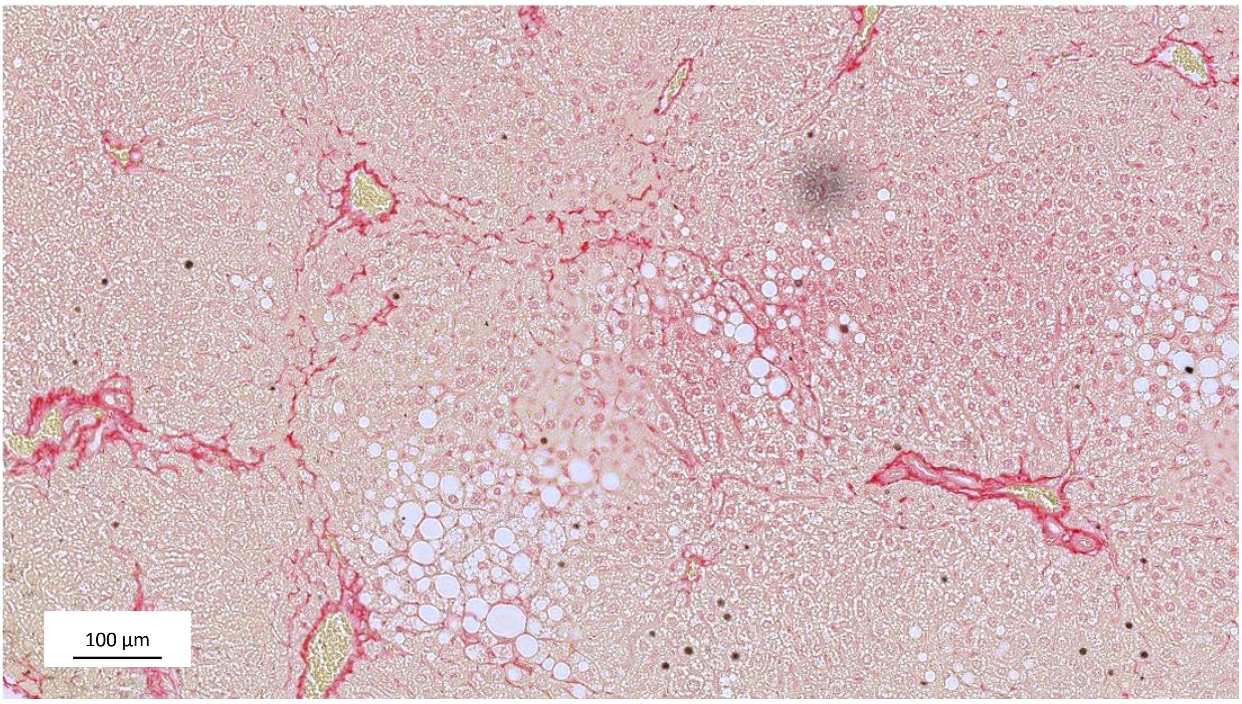

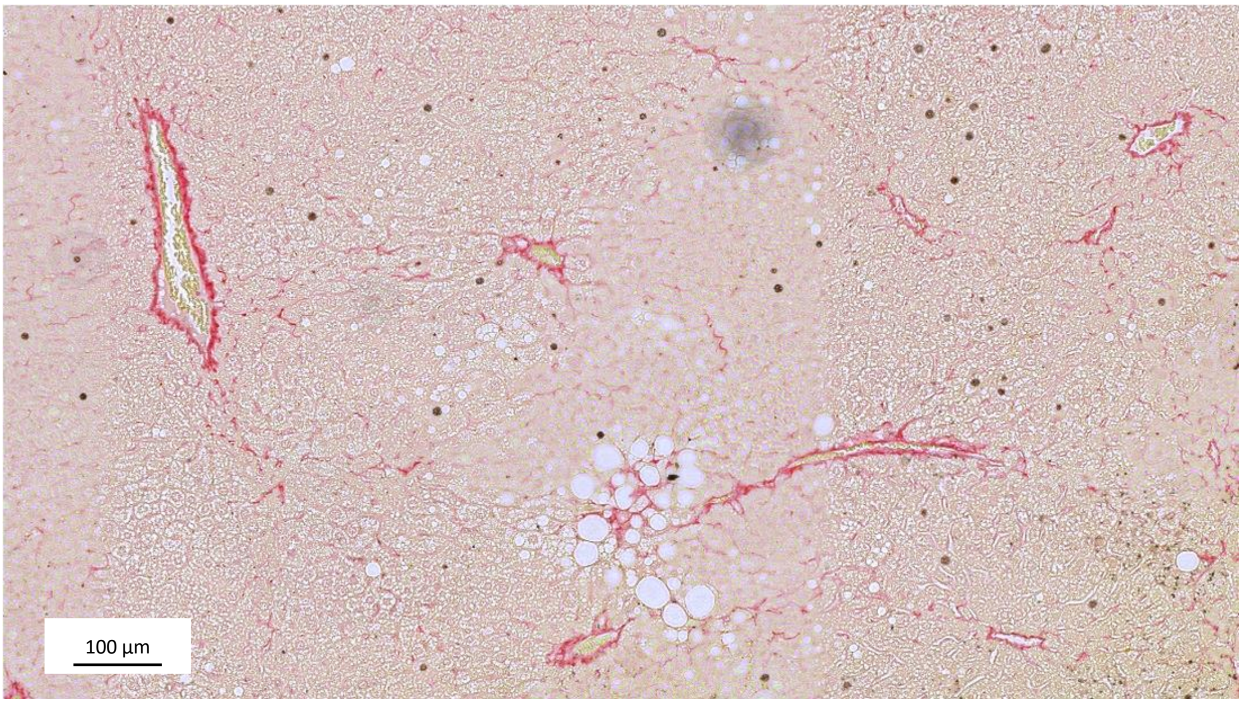

CDHFD-C-12

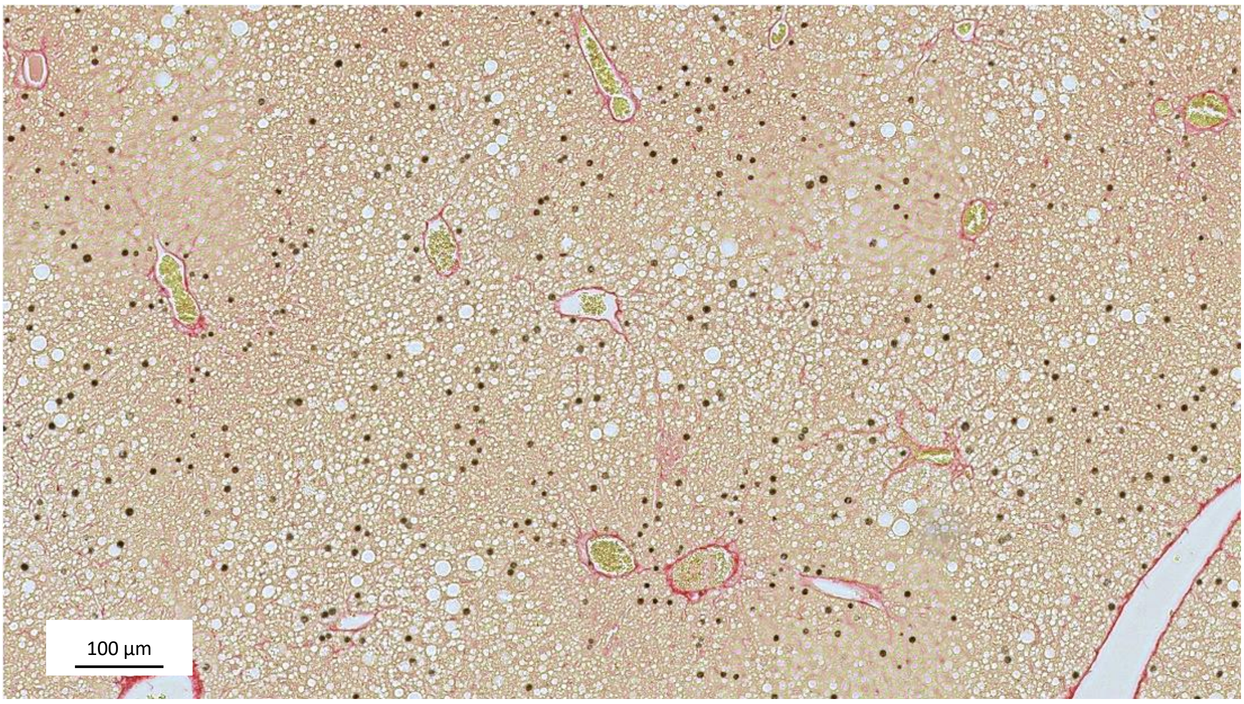

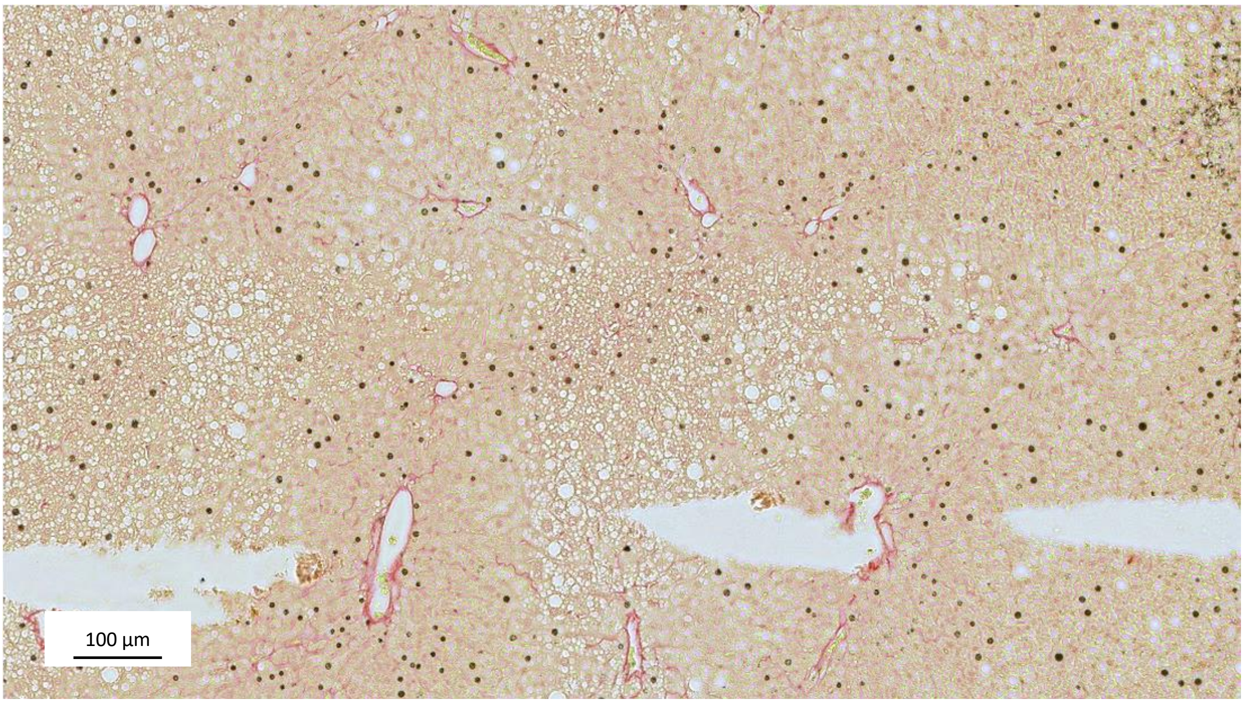

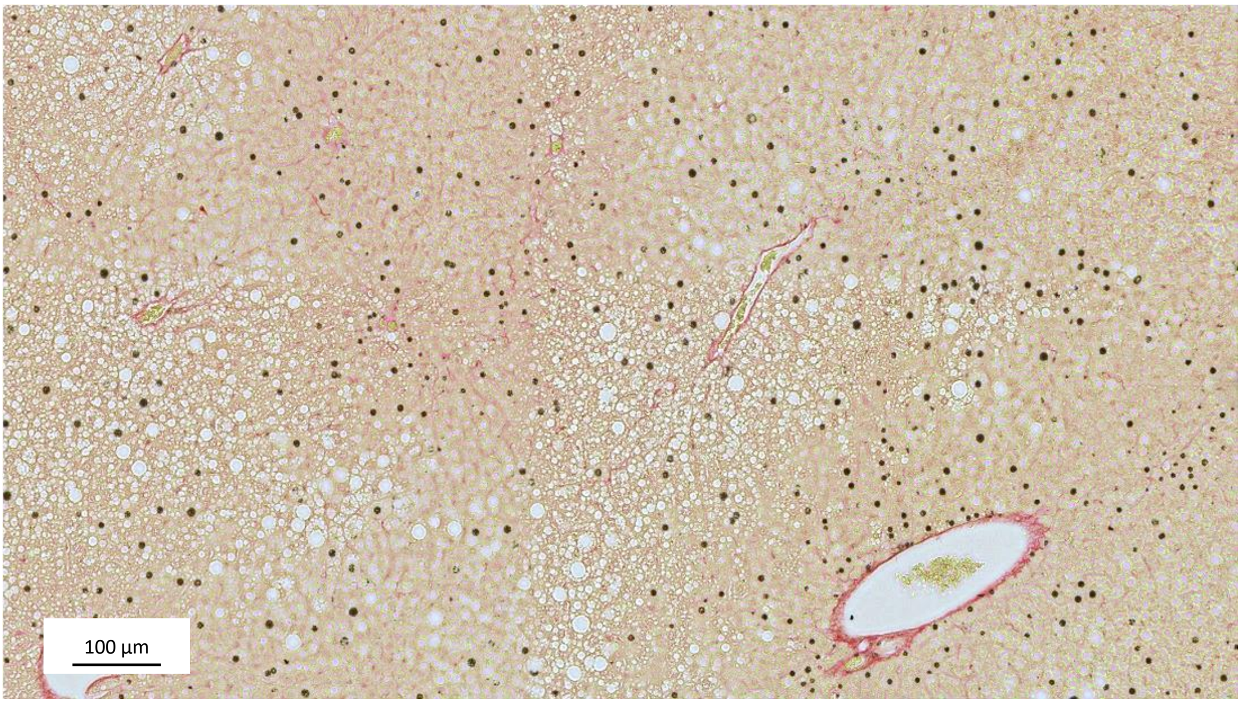

CDHFD-C-13

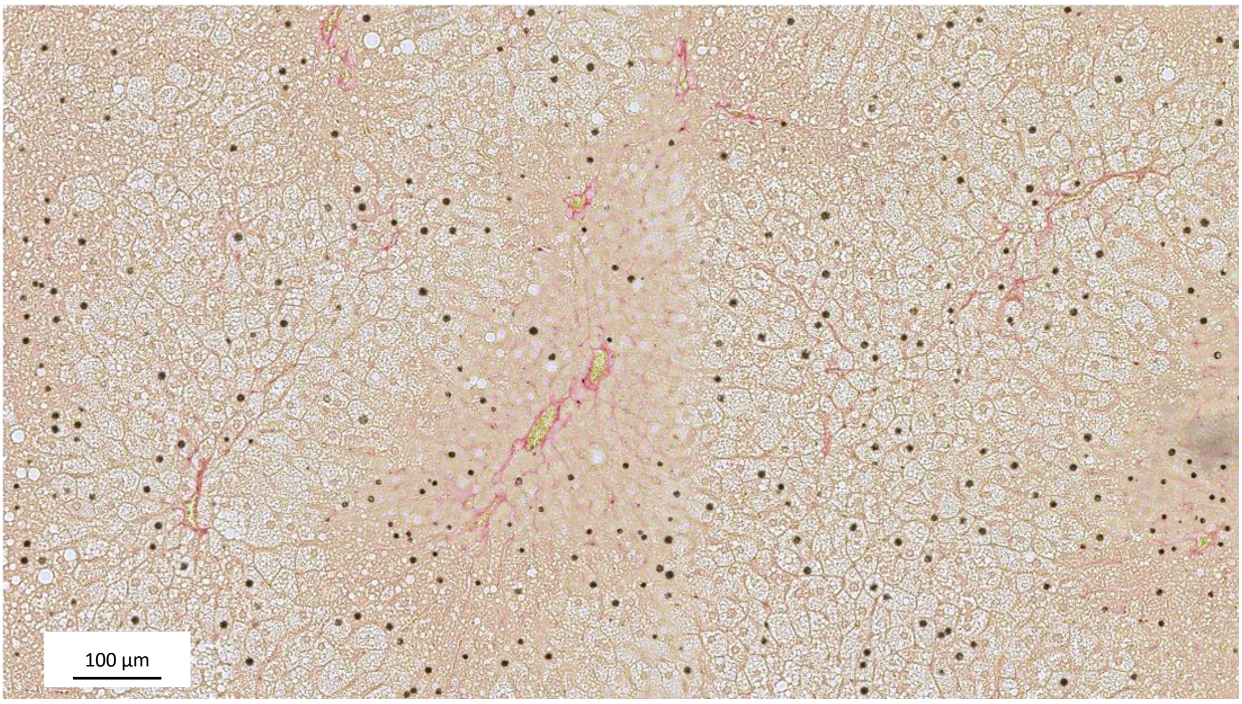

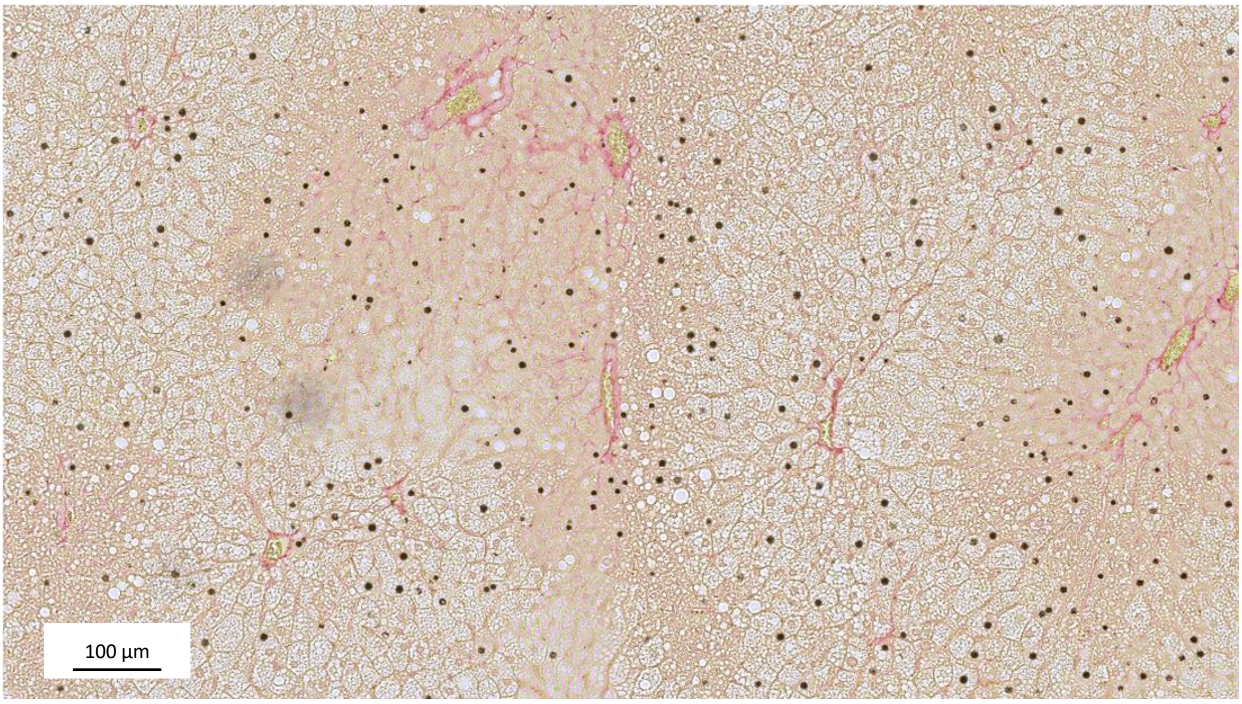

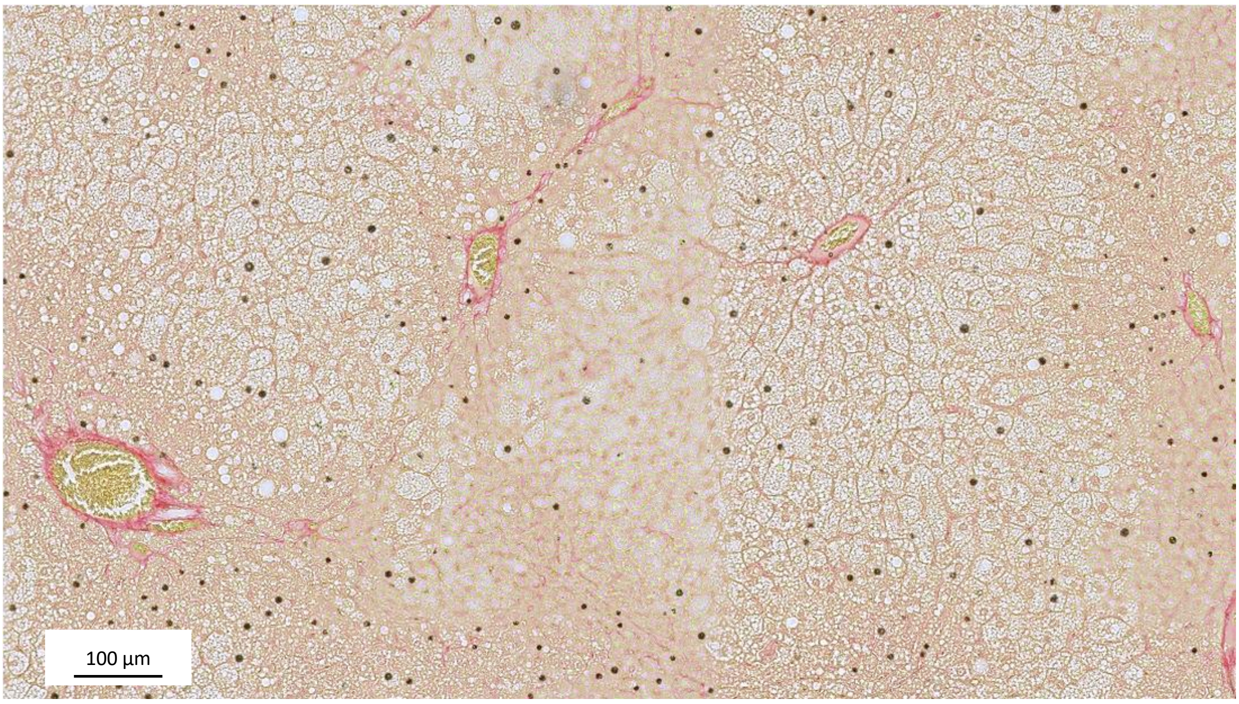

CDHFD-C-14

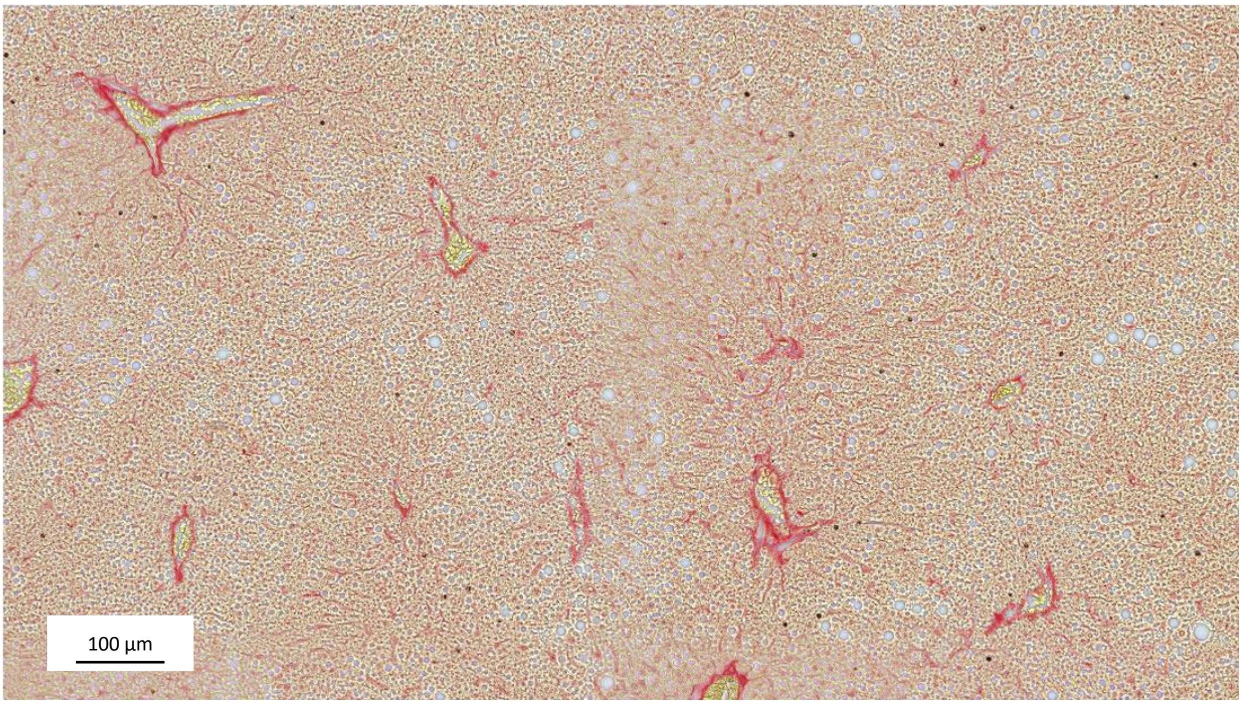

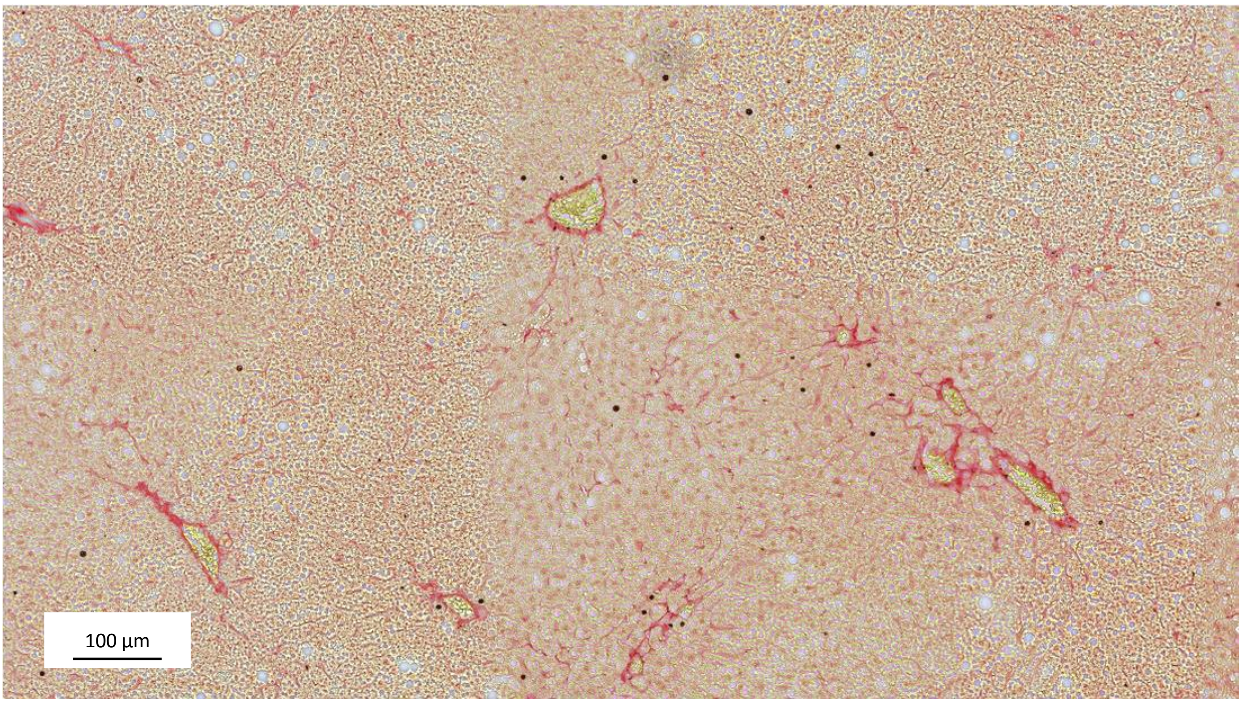

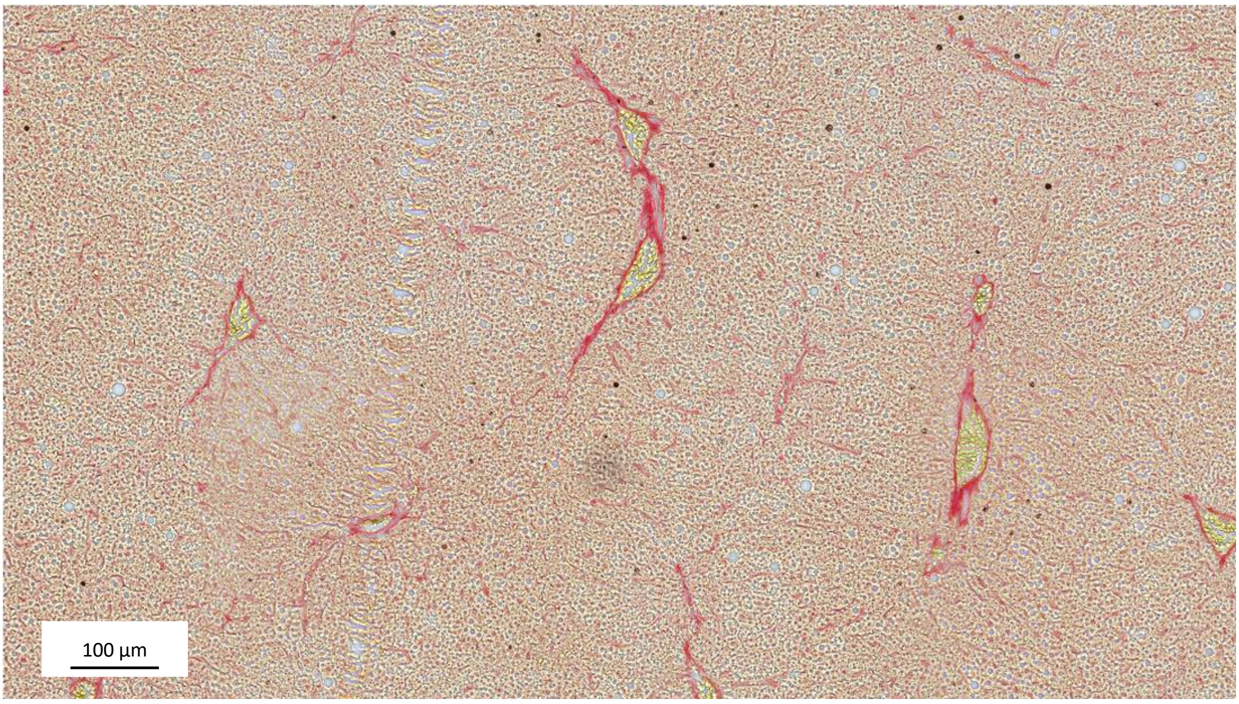

CDHFD-C-15

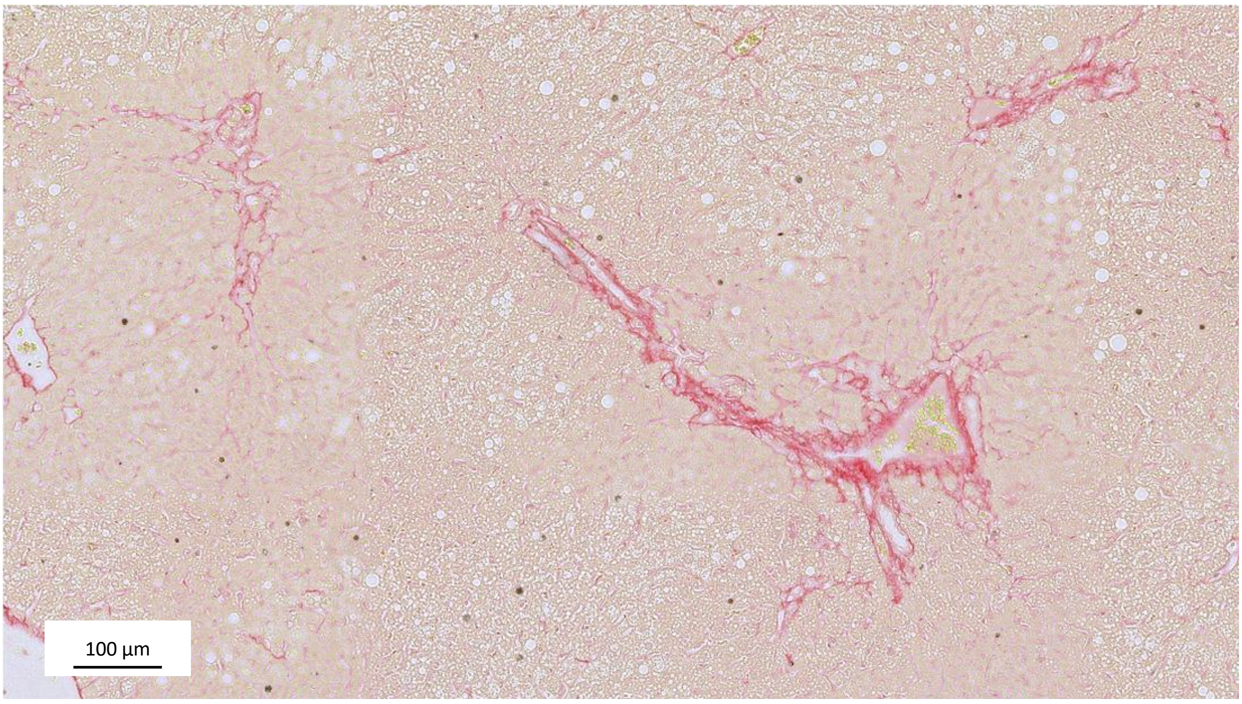

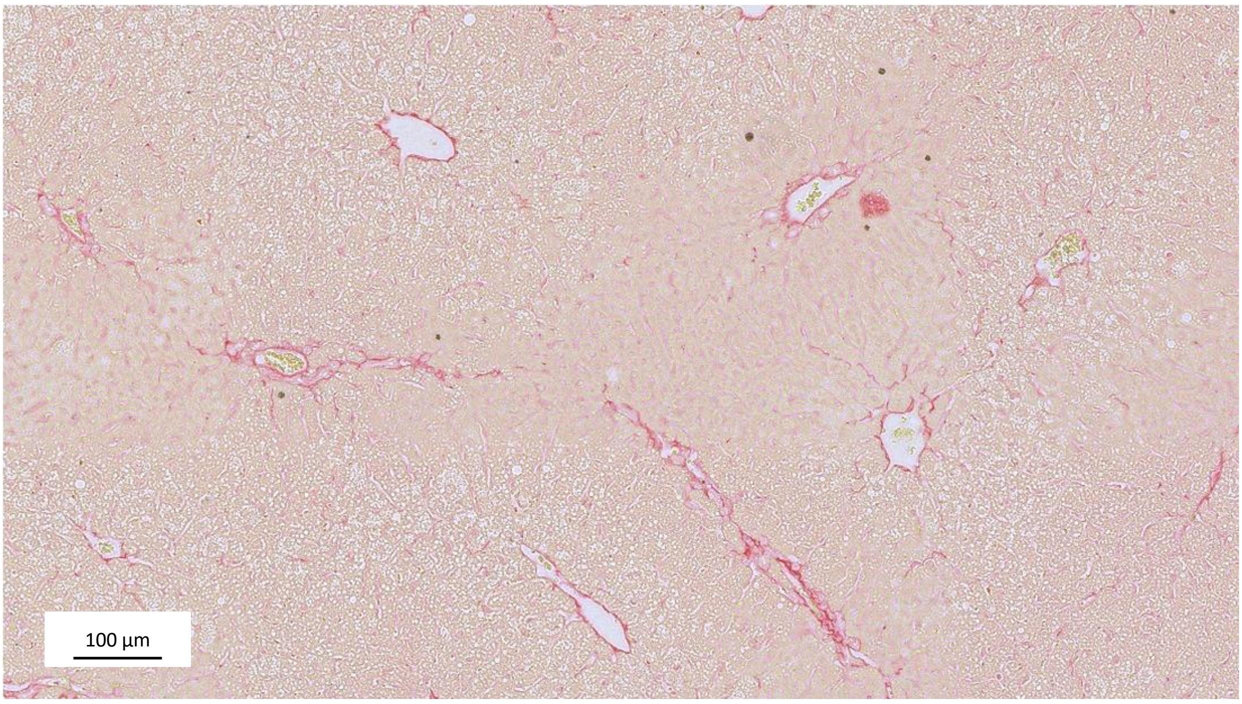

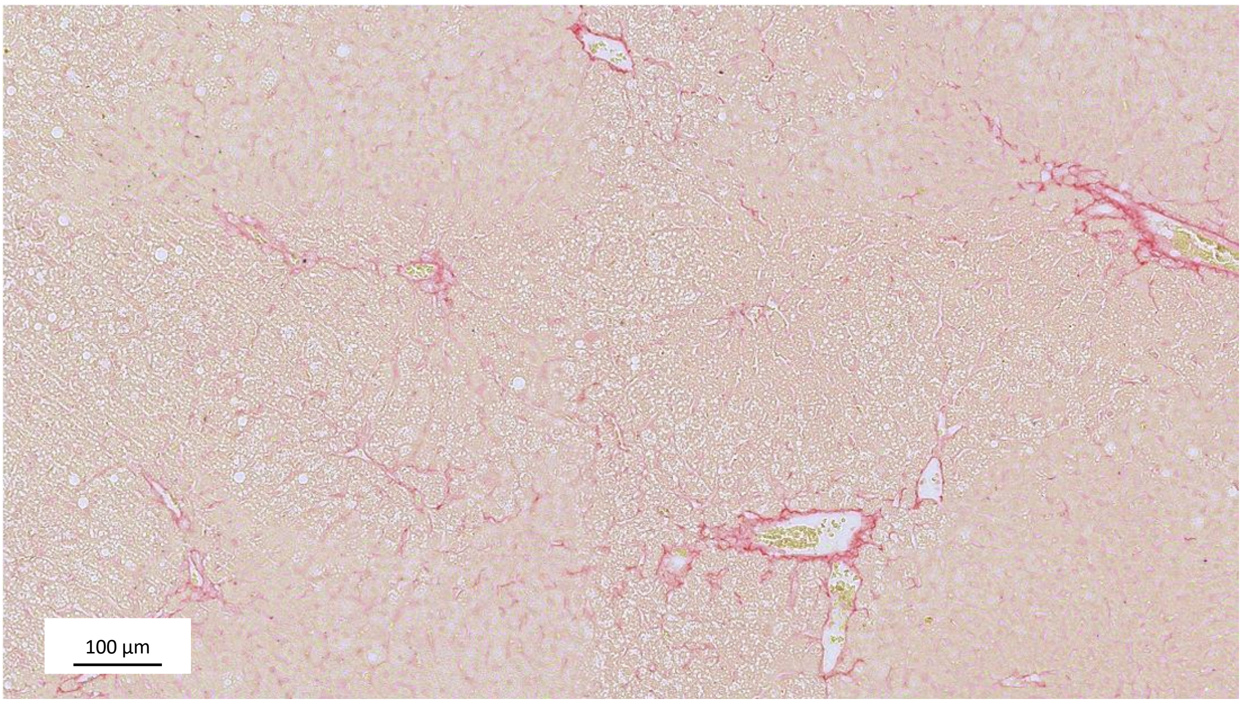

Supplement: Source Data Fig. 1 — Replicate histological images. [file 41564_2023_1418_MOESM10_ESM.pdf]
