# Supplementary material for: Parabacteroides distasonis uses dietary inulin to suppress NASH via its metabolite pentadecanoic acid
Source: Nat Microbiol. 2023 Jun 29;8(8):1534–48. doi: 10.1038/s41564-023-01418-7 (PMC10390331; doi:10.1038/s41564-023-01418-7)

Source images for Figure 3

H&E staining
Replicate images from a same mouse were displayed within a same page

H&E Staining

NCD group

(5 mice were included)

NCD-1

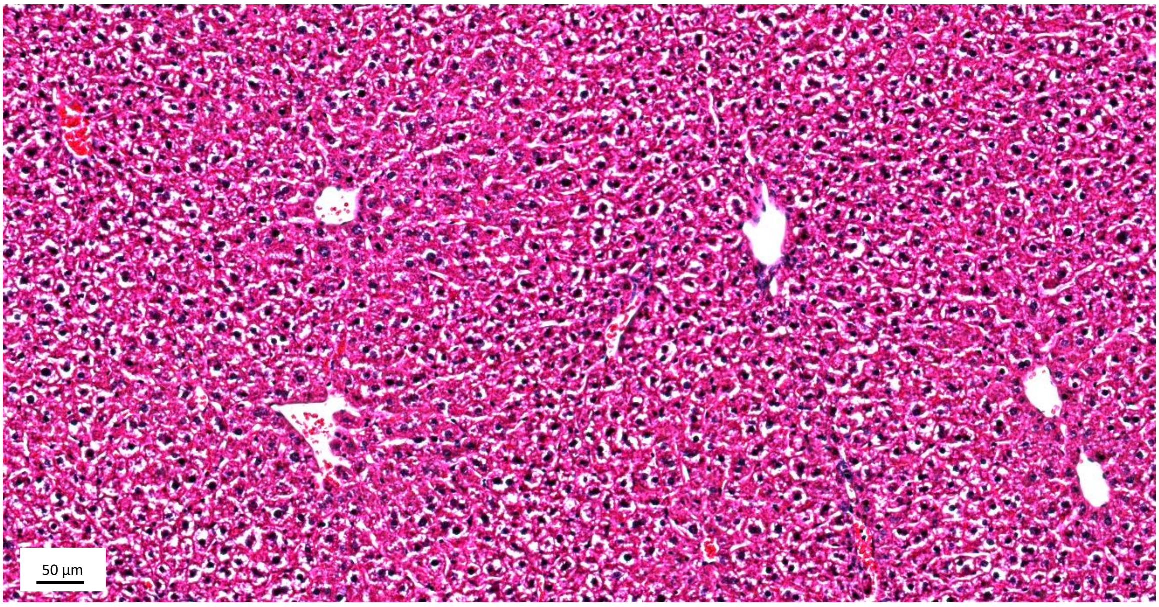

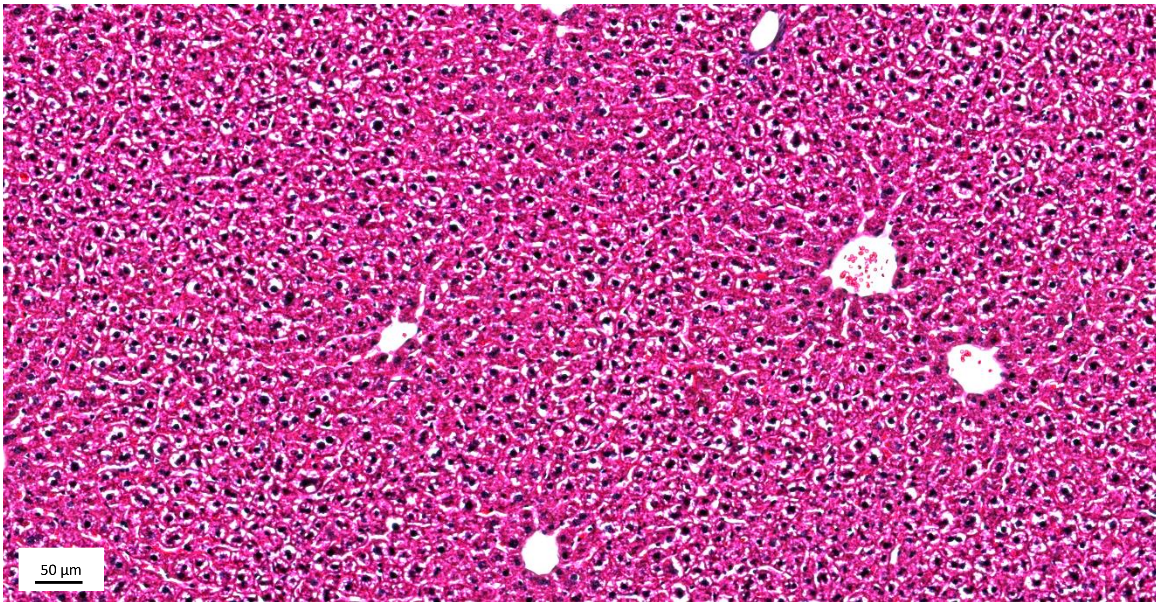

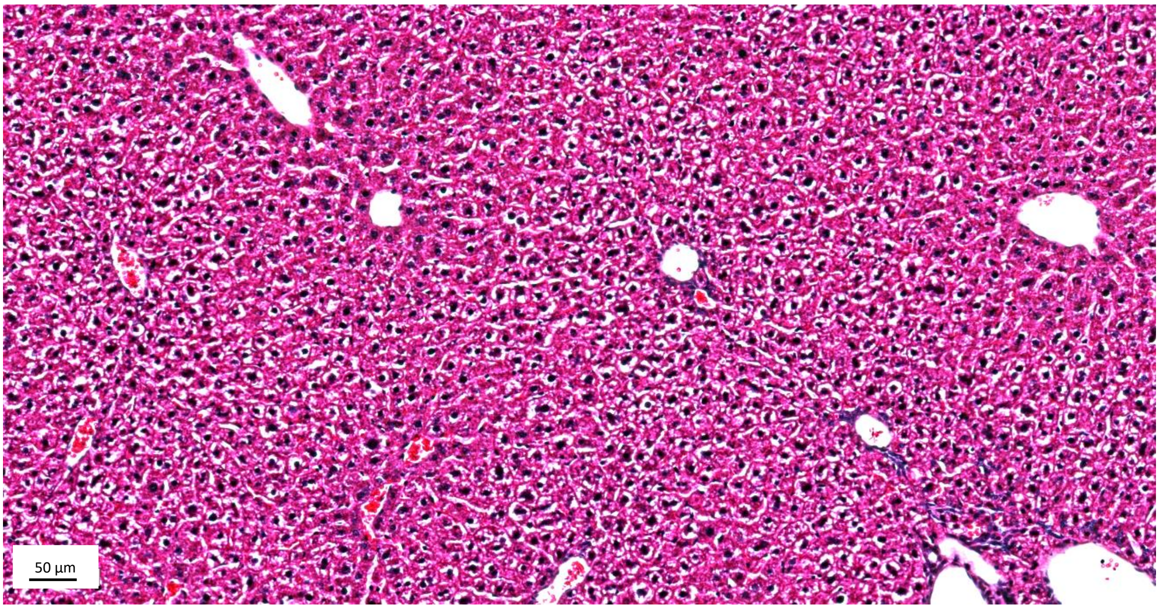

NCD-2

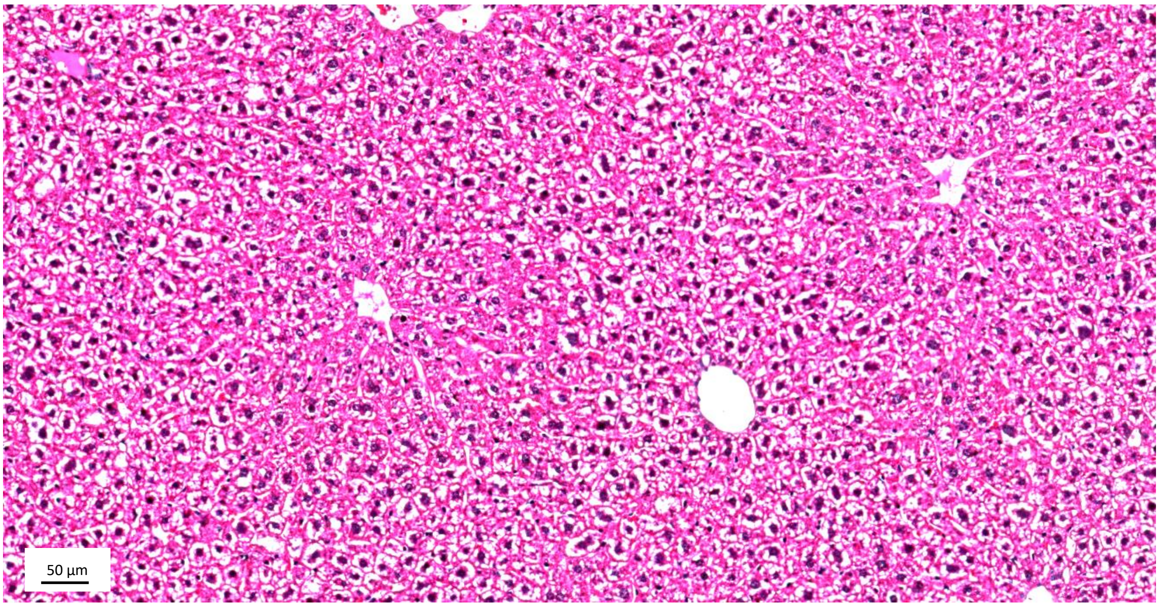

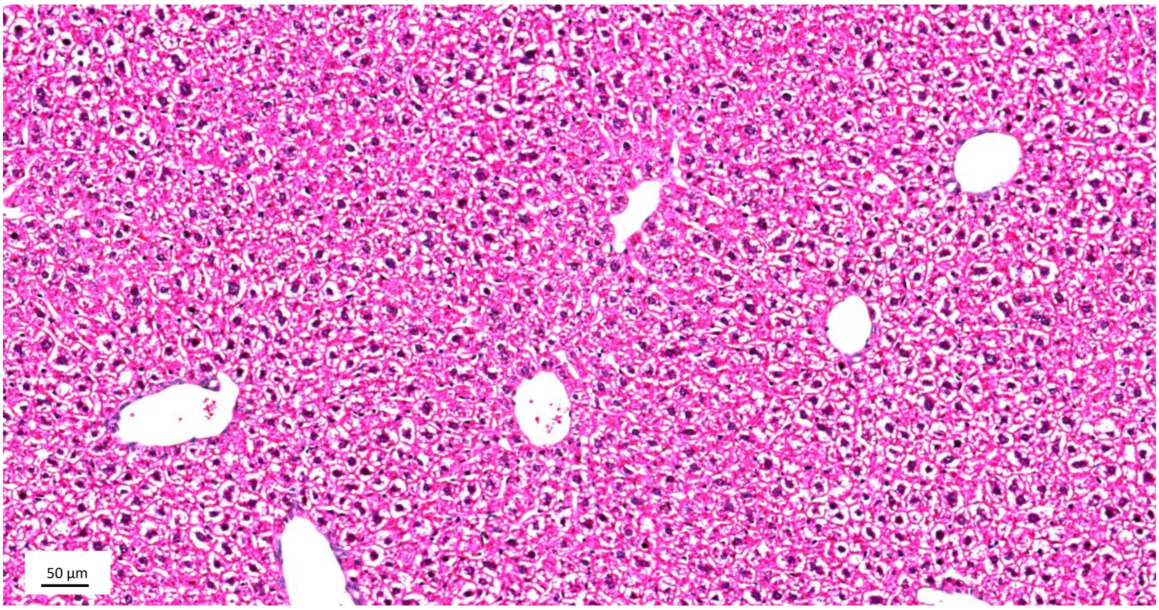

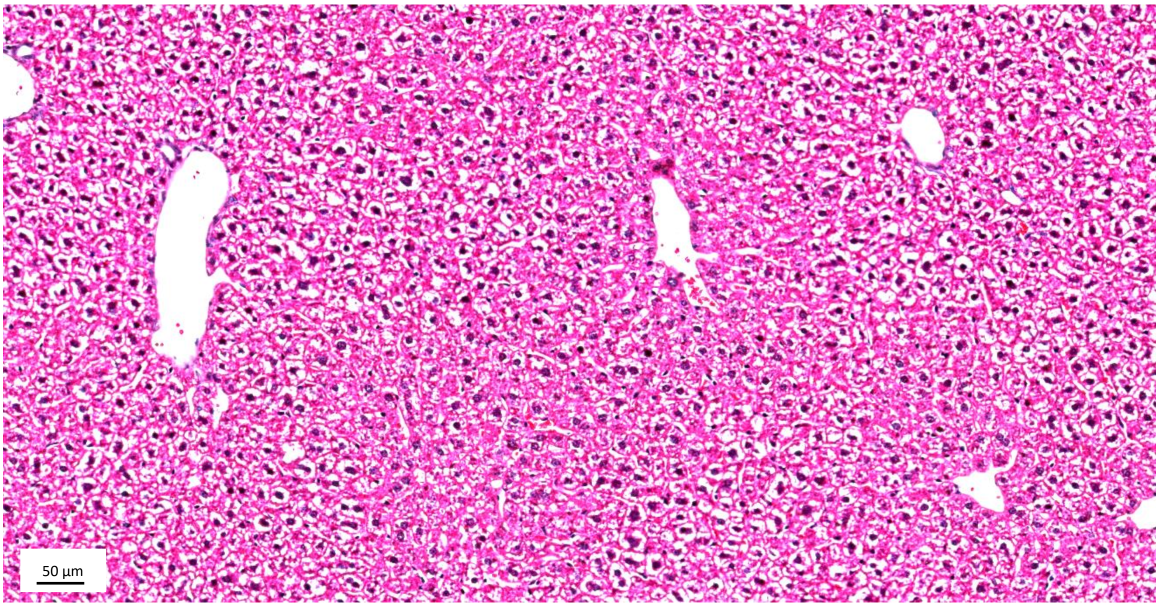

NCD-3

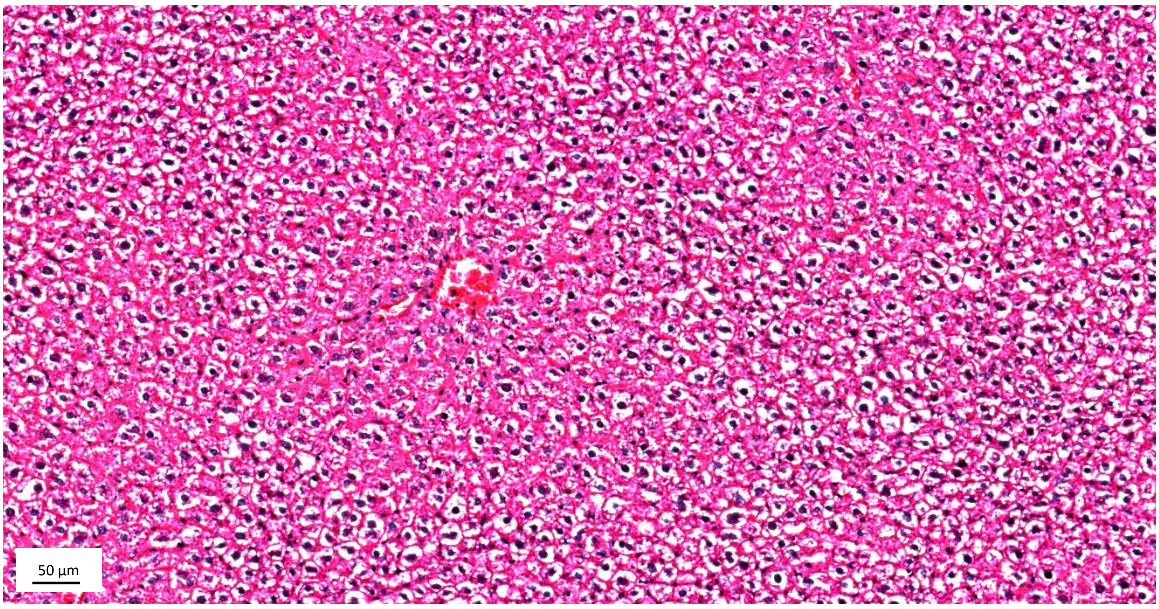

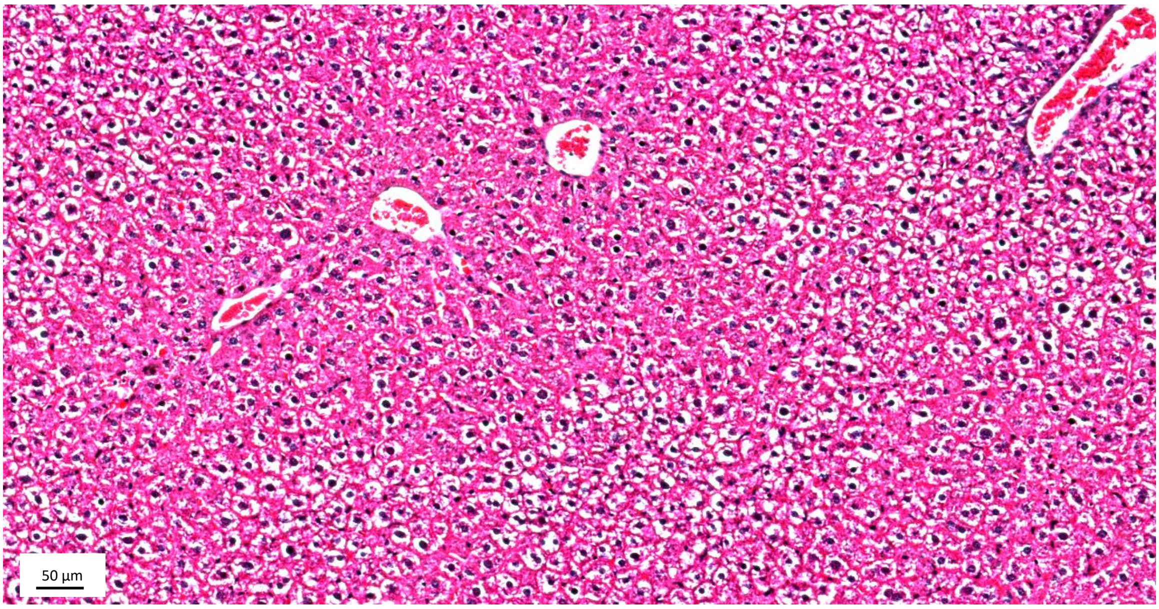

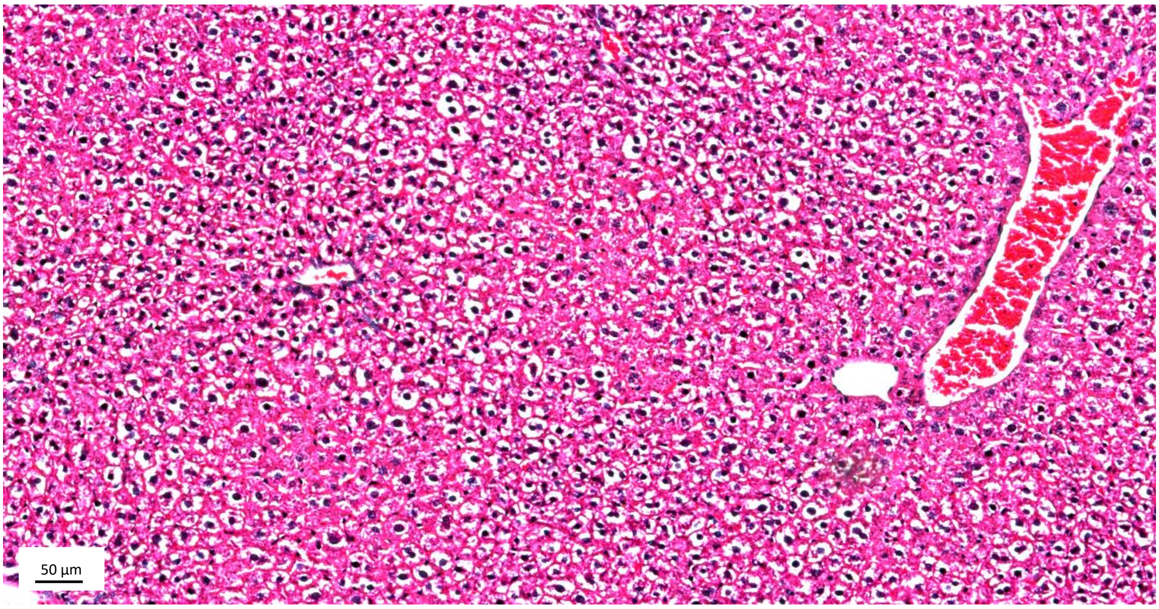

NCD-4

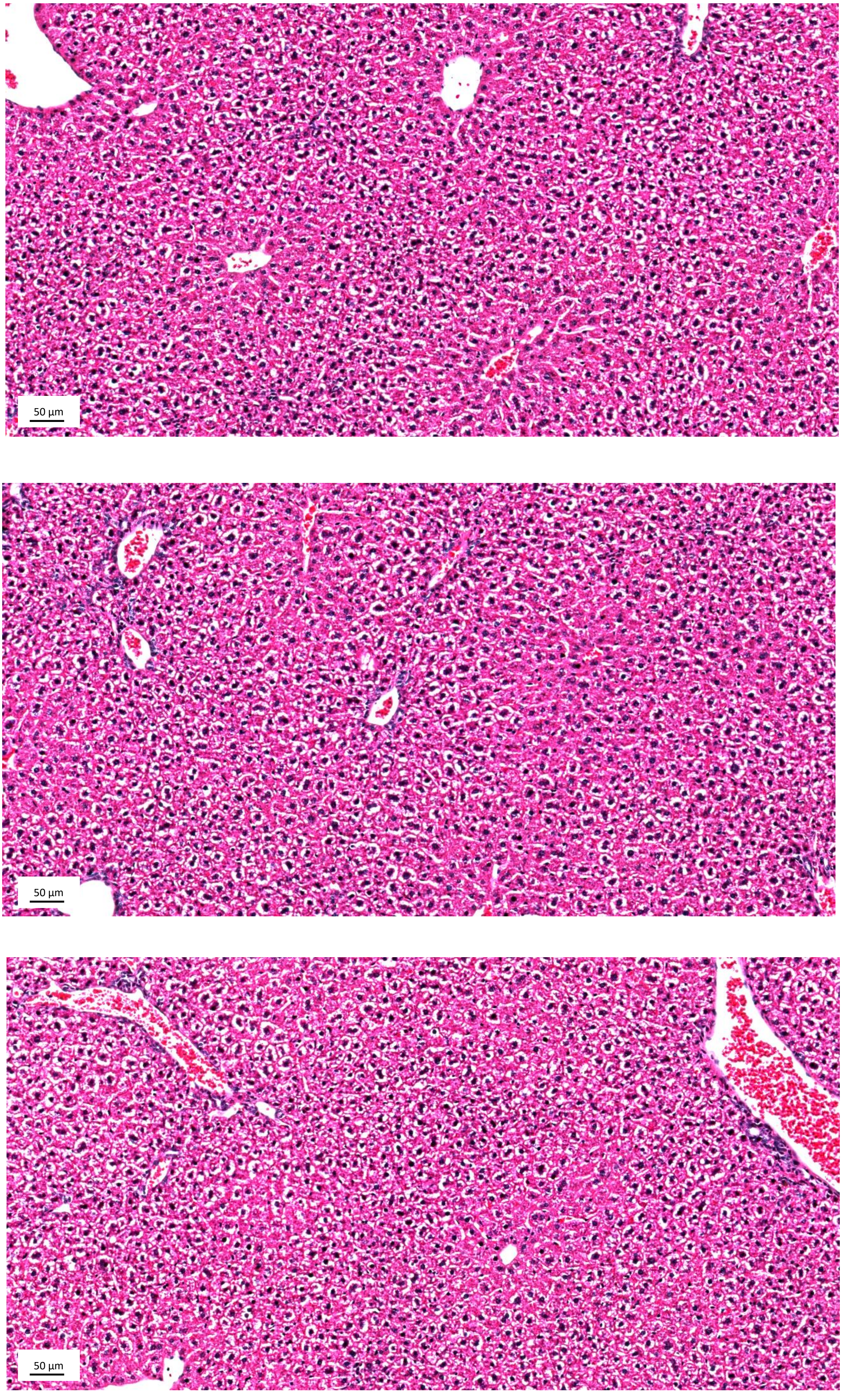

NCD-5

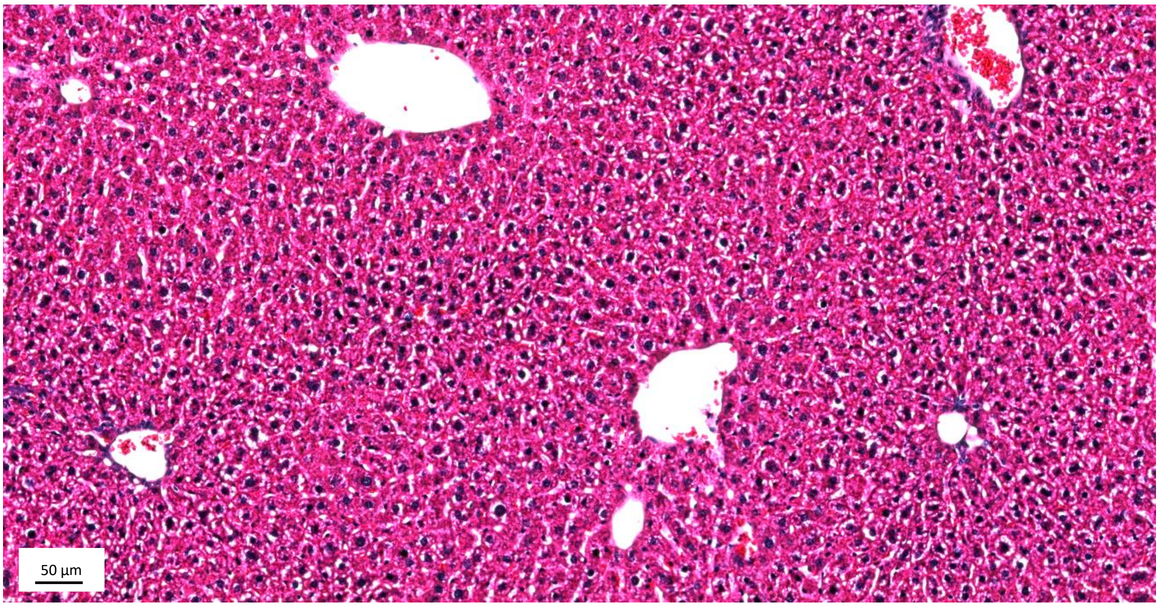

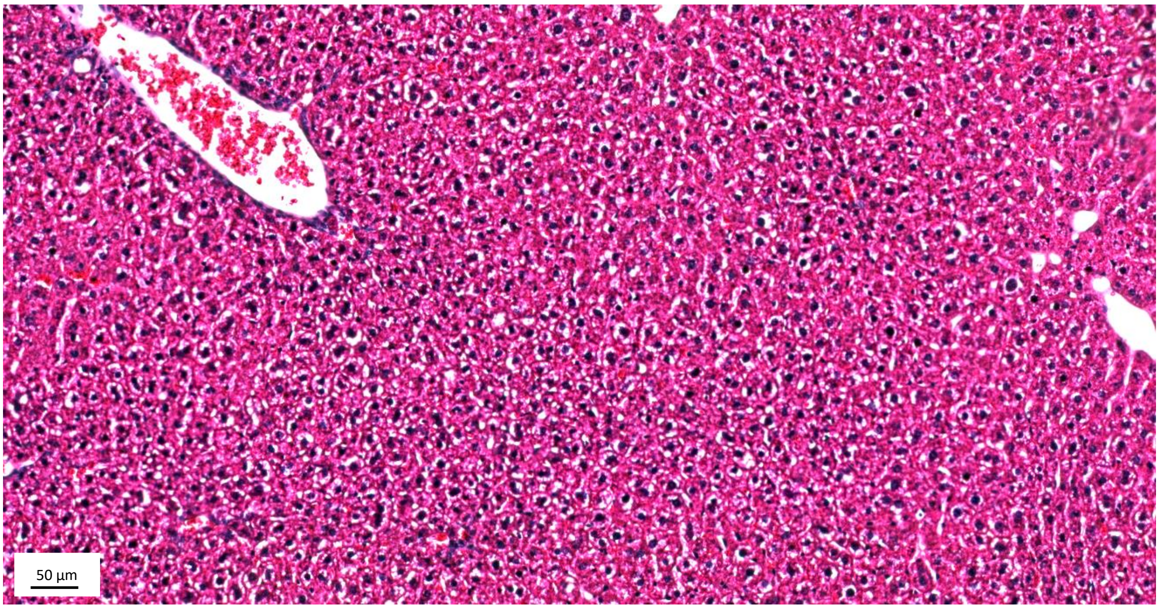

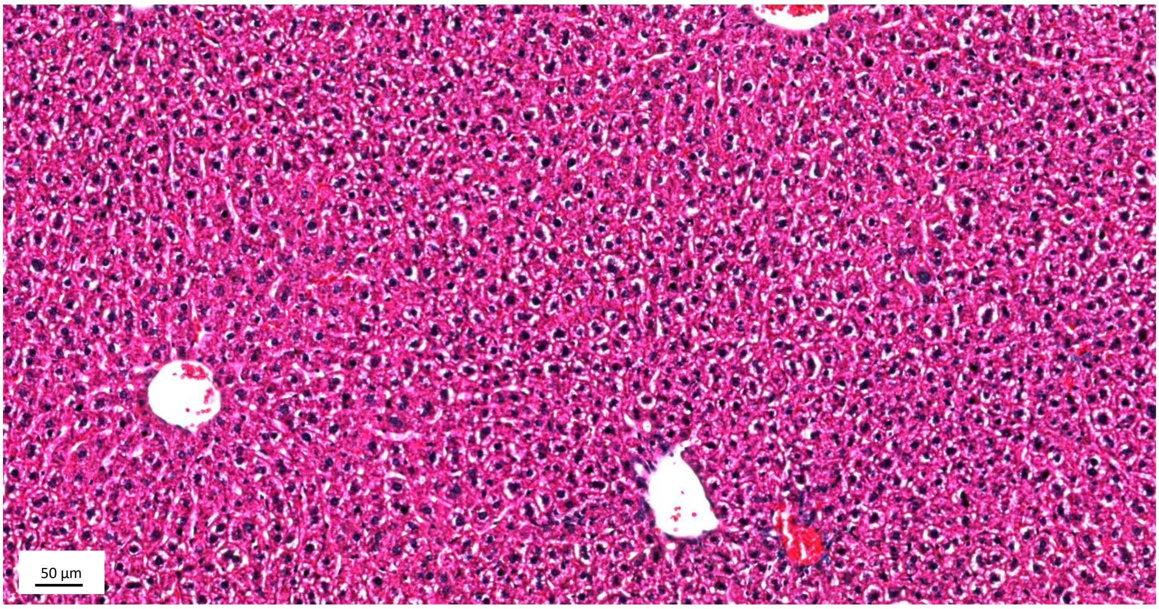

H&E Staining

CDHFD + PBS group

(14 mice were included)

CDHFD + PBS-1

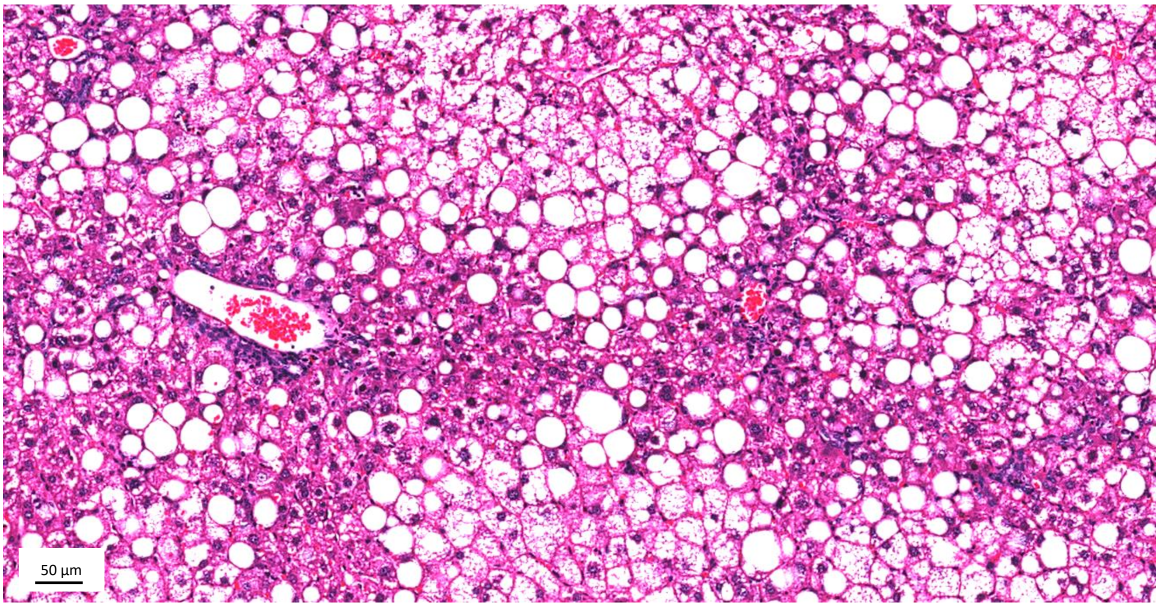

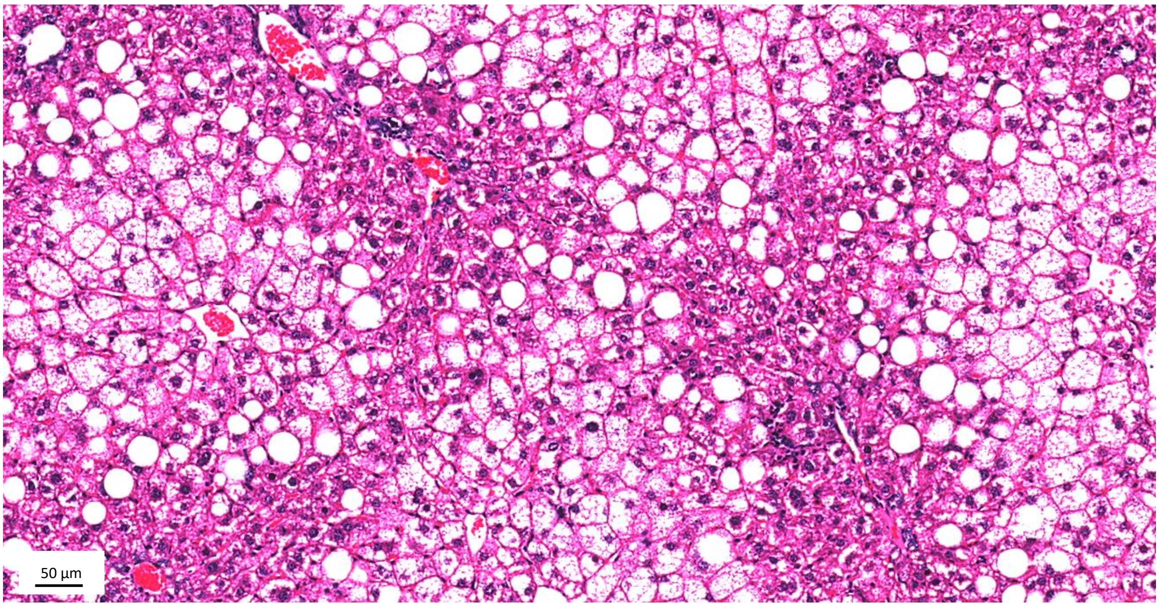

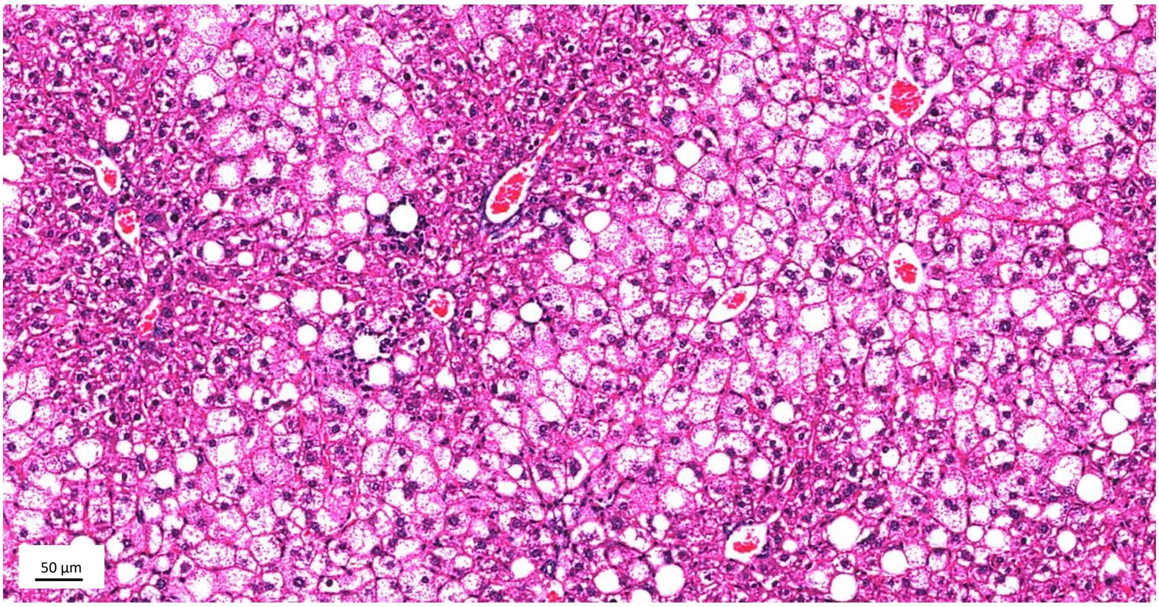

CDHFD + PBS-2

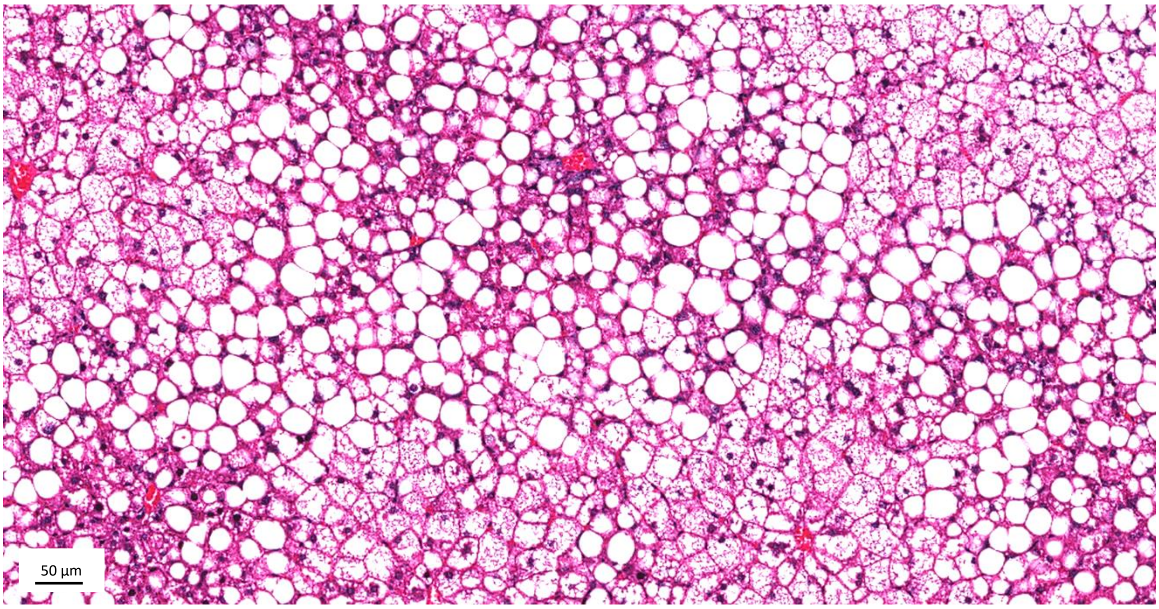

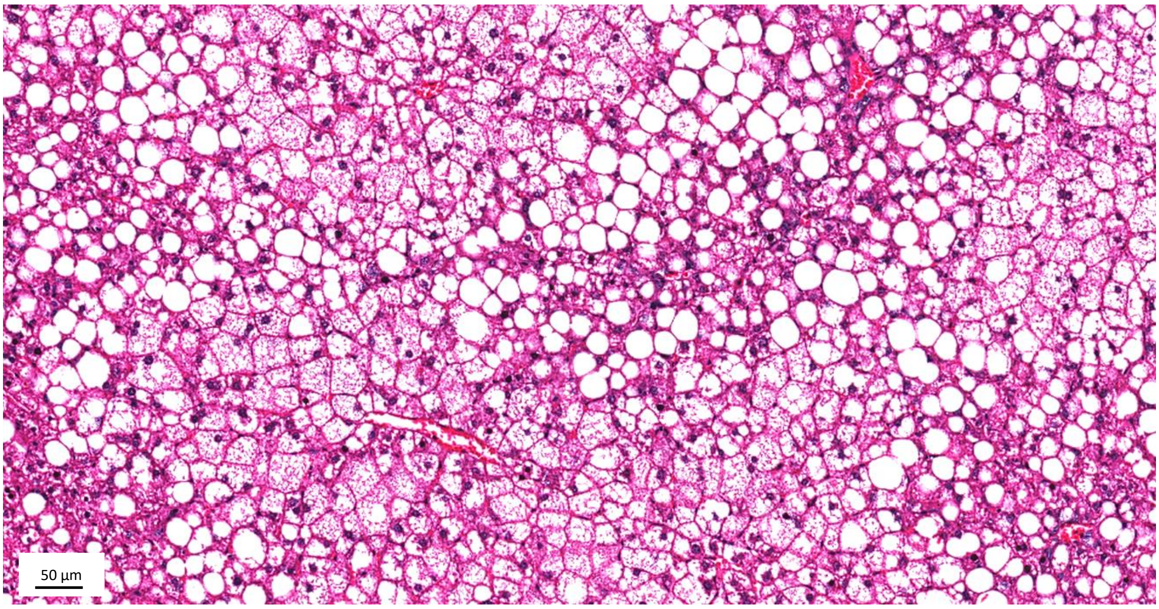

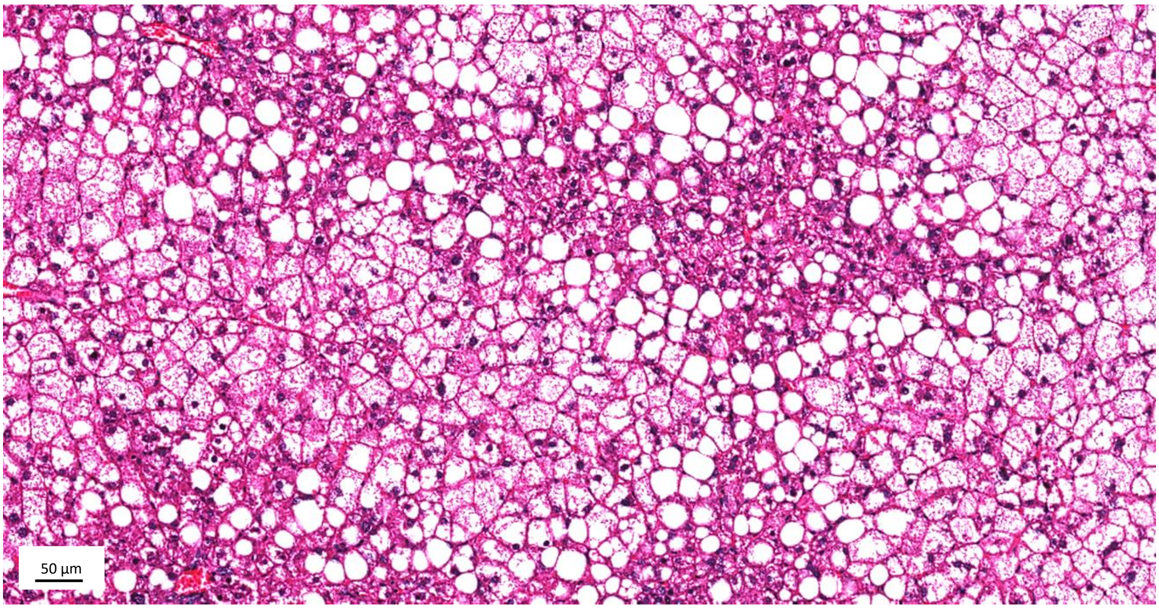

CDHFD + PBS-3

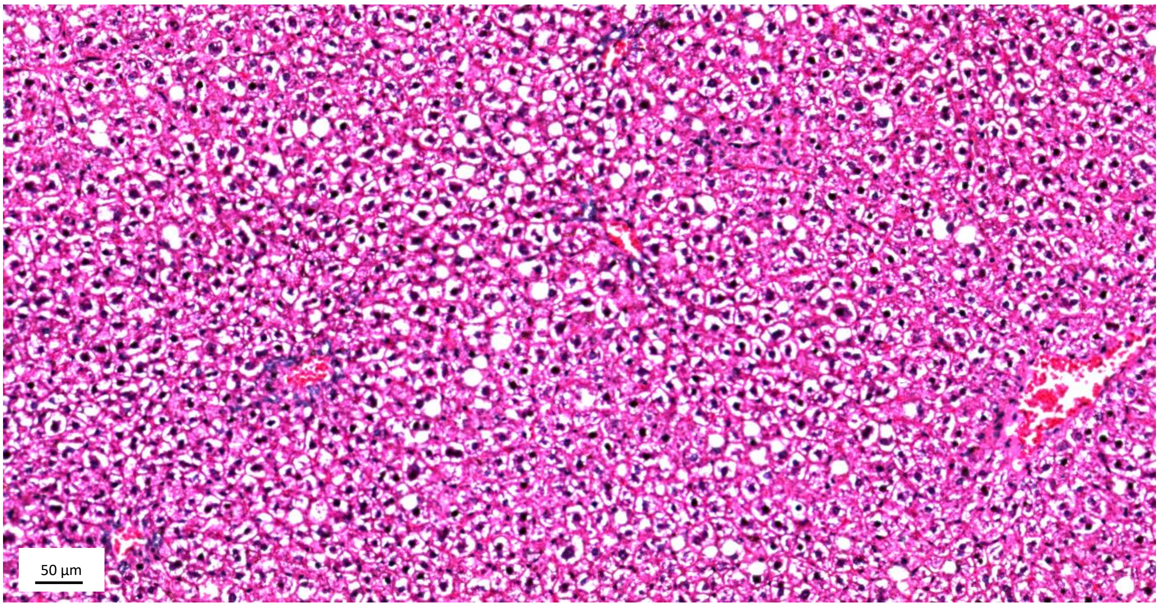

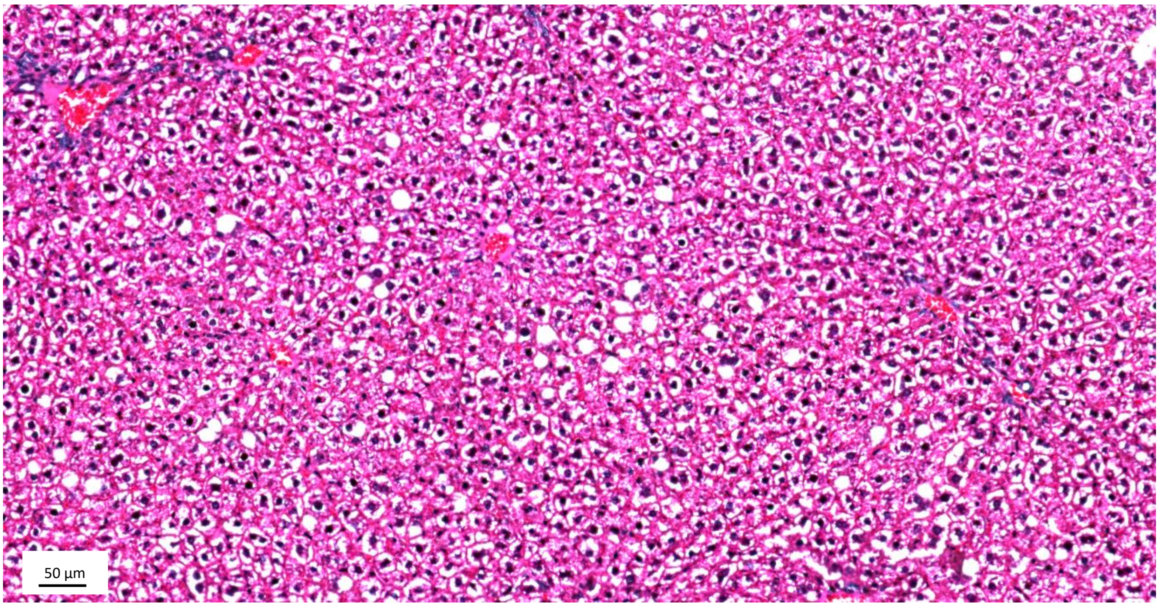

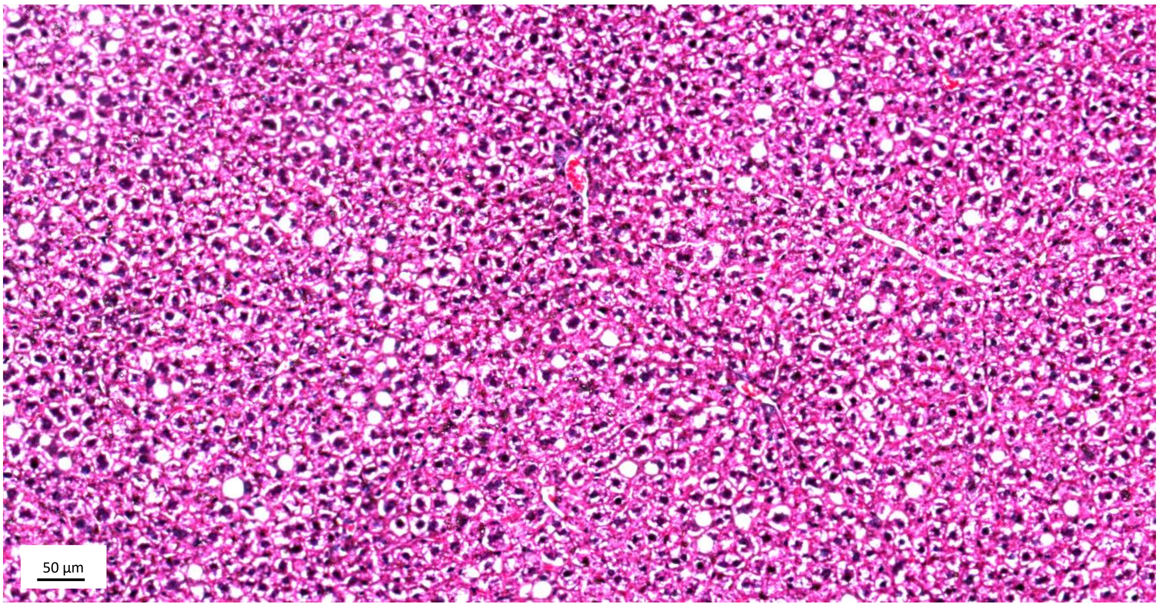

CDHFD + PBS-4

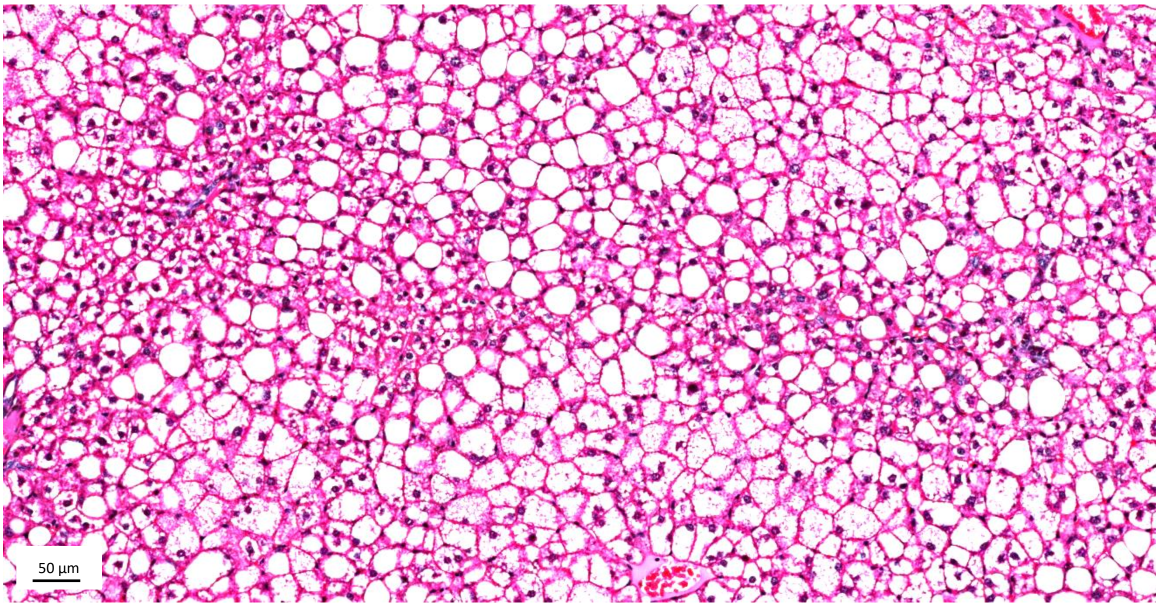

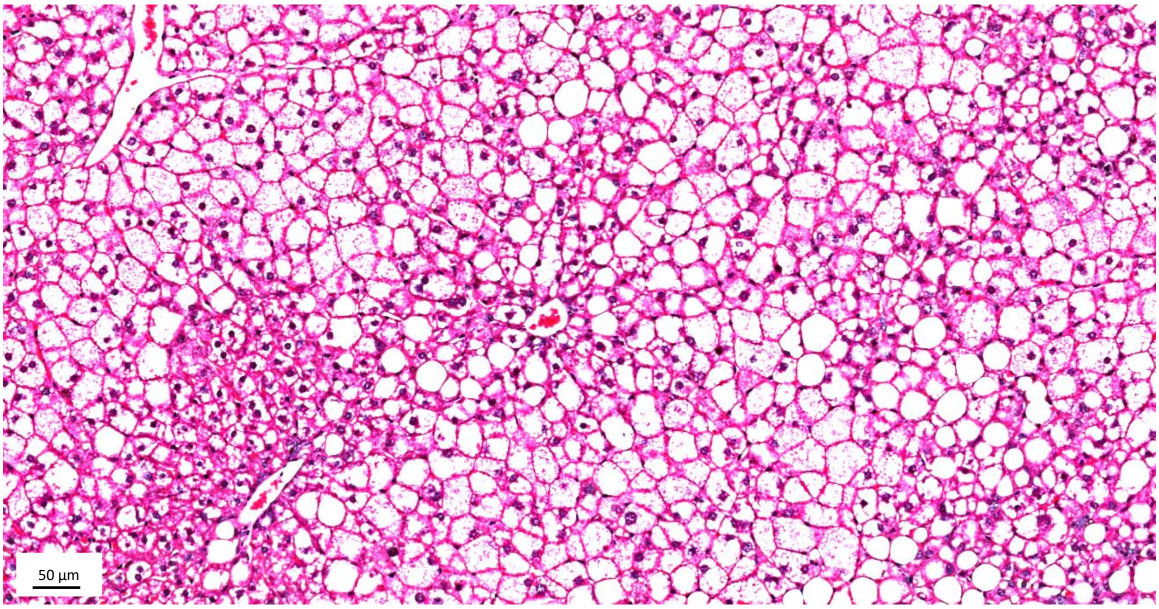

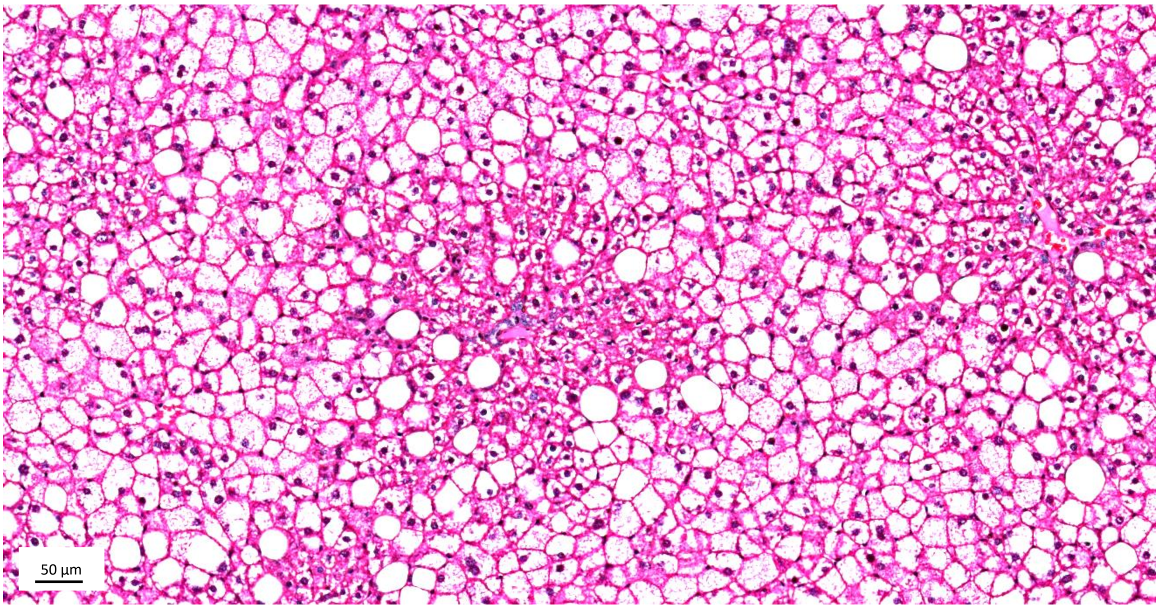

CDHFD + PBS-5

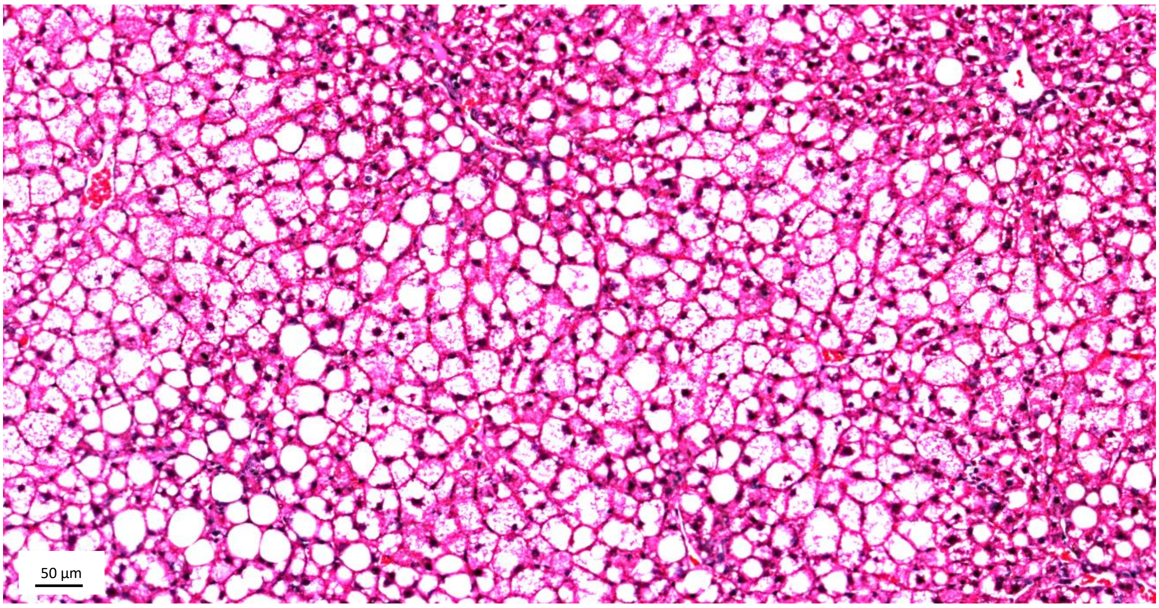

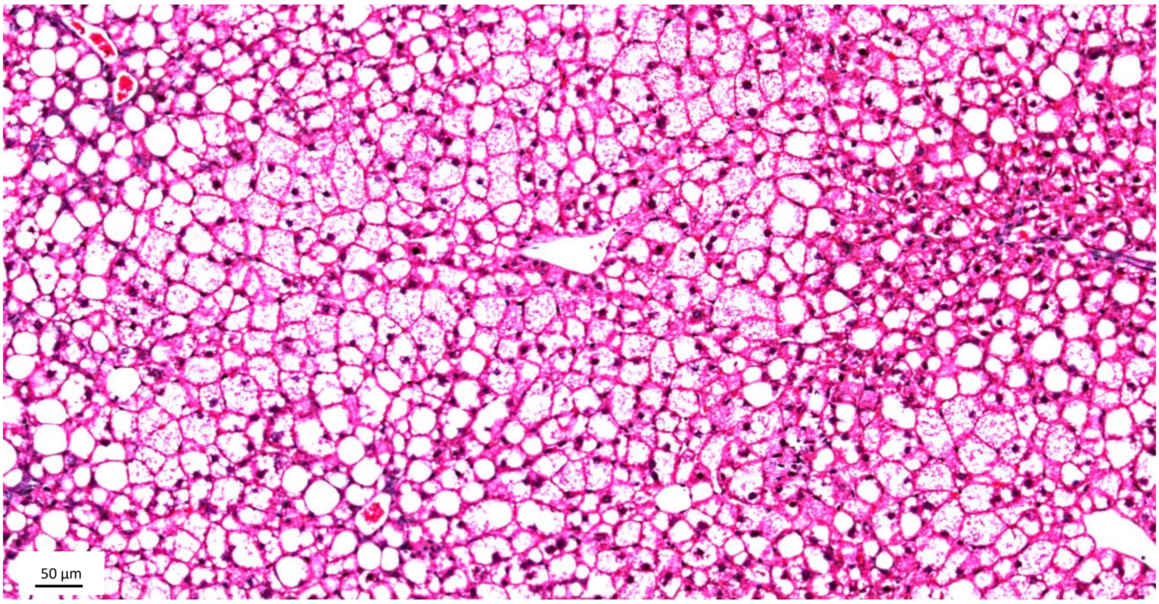

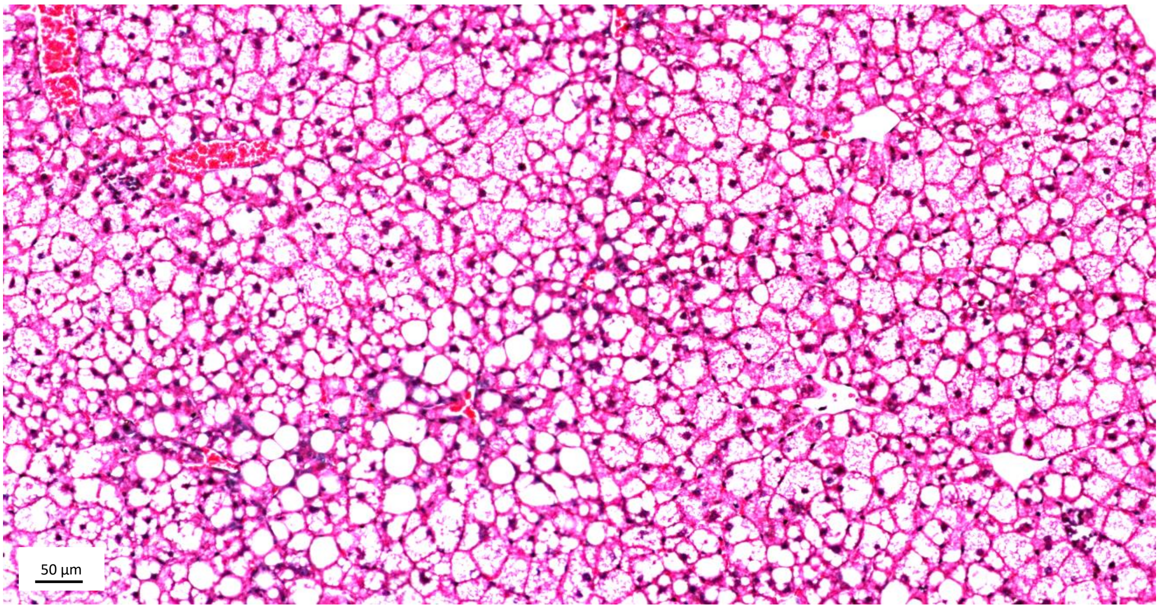

CDHFD + PBS-6

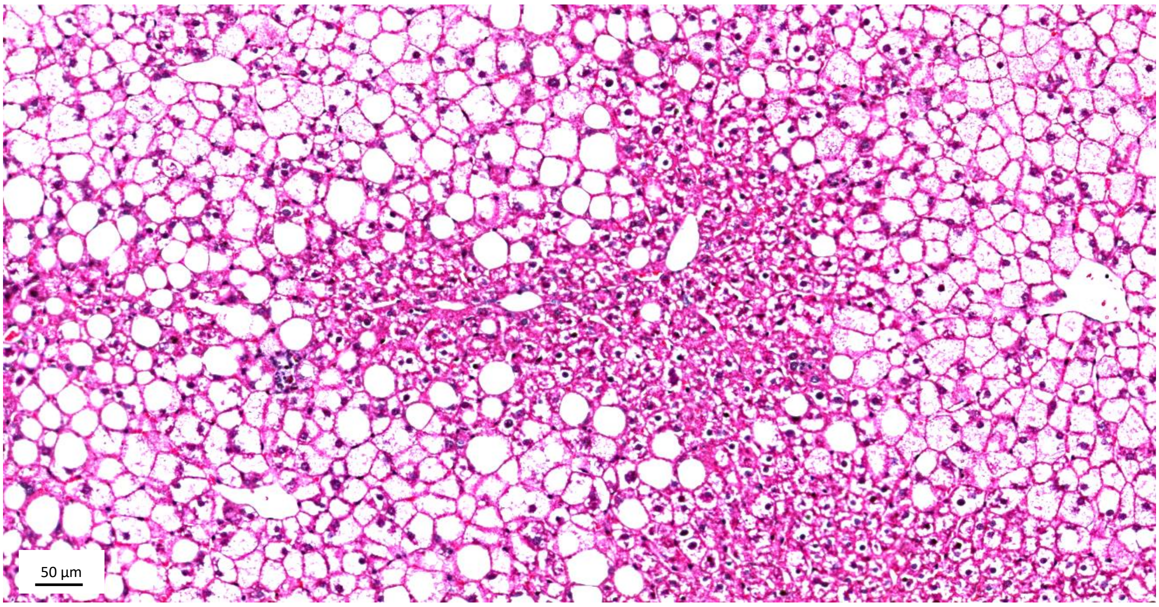

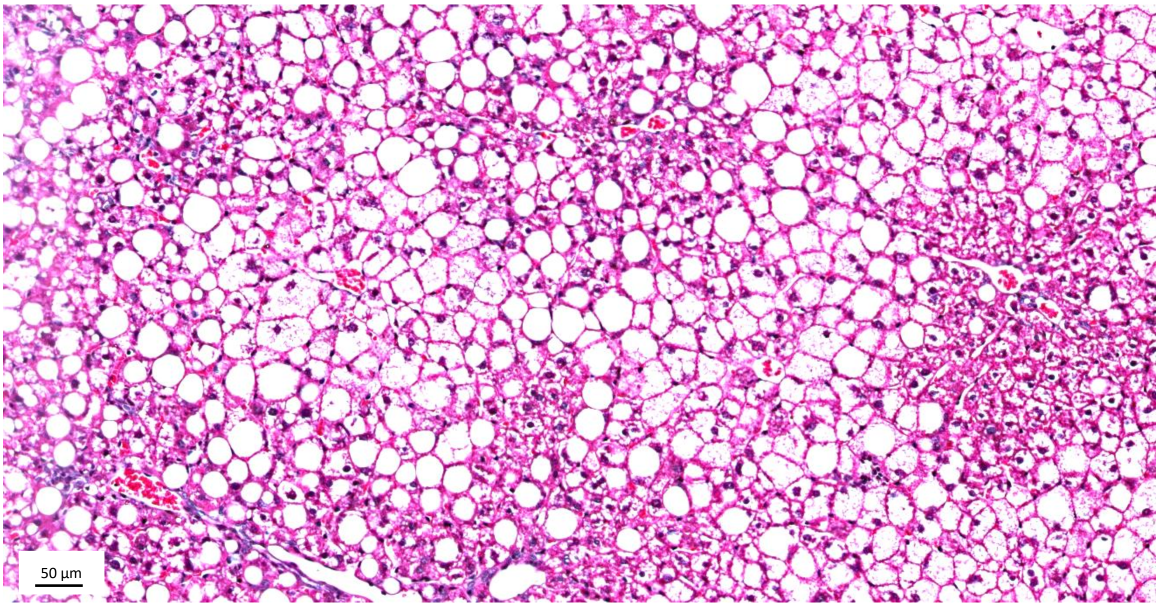

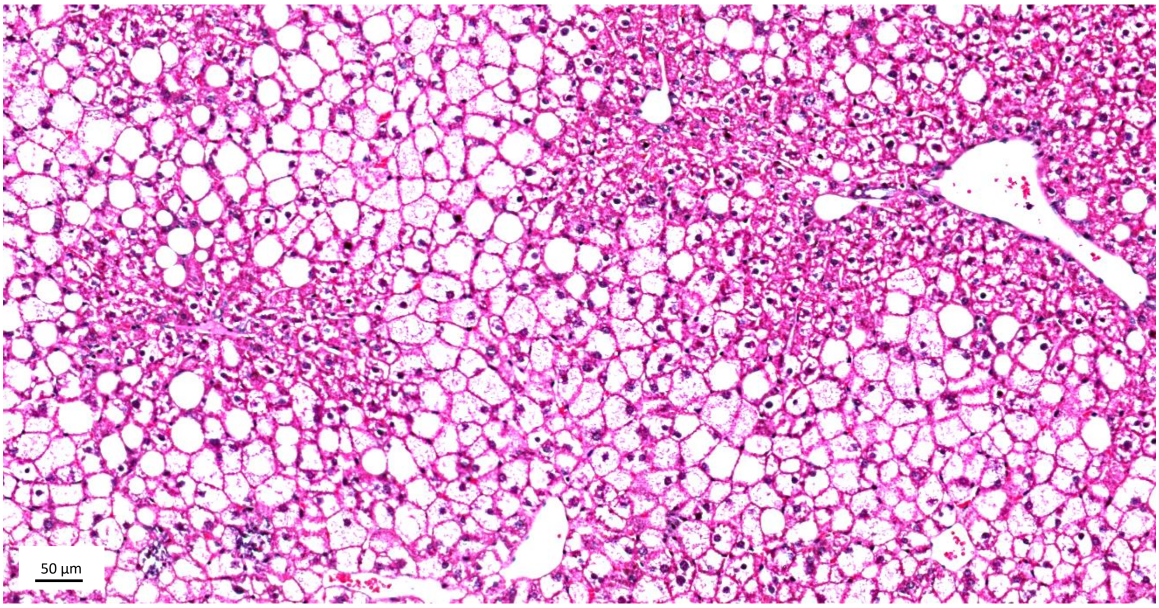

CDHFD + PBS-7

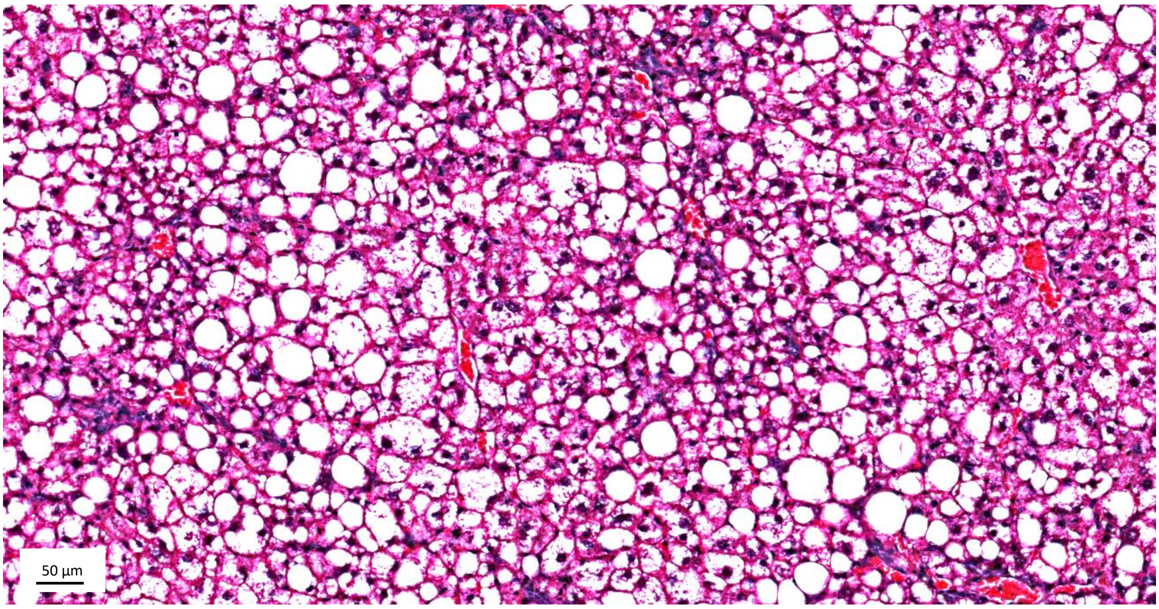

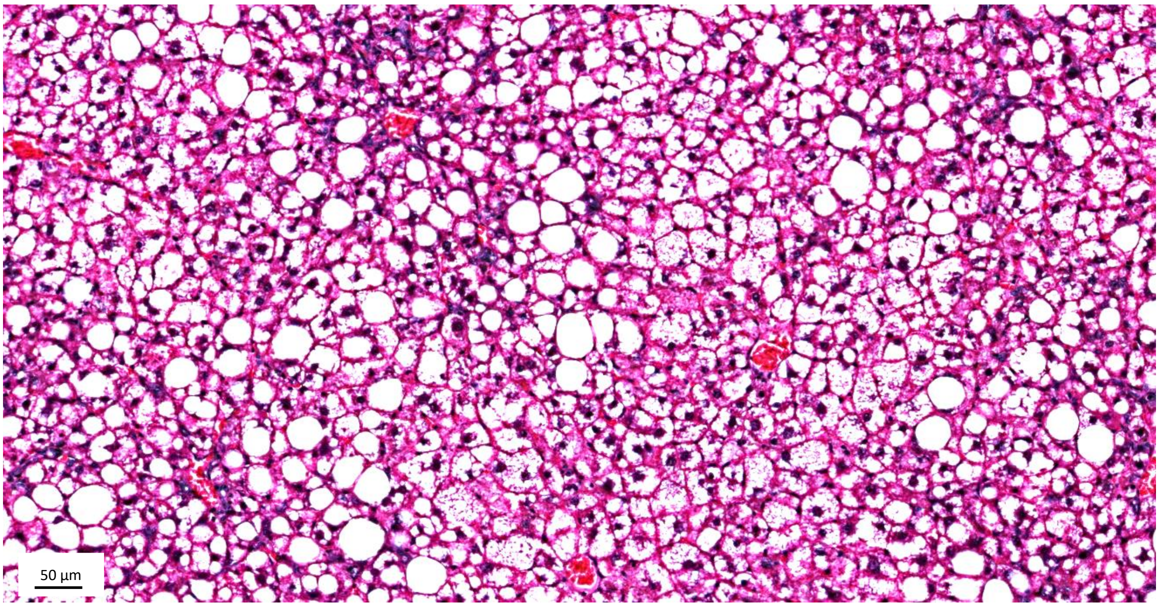

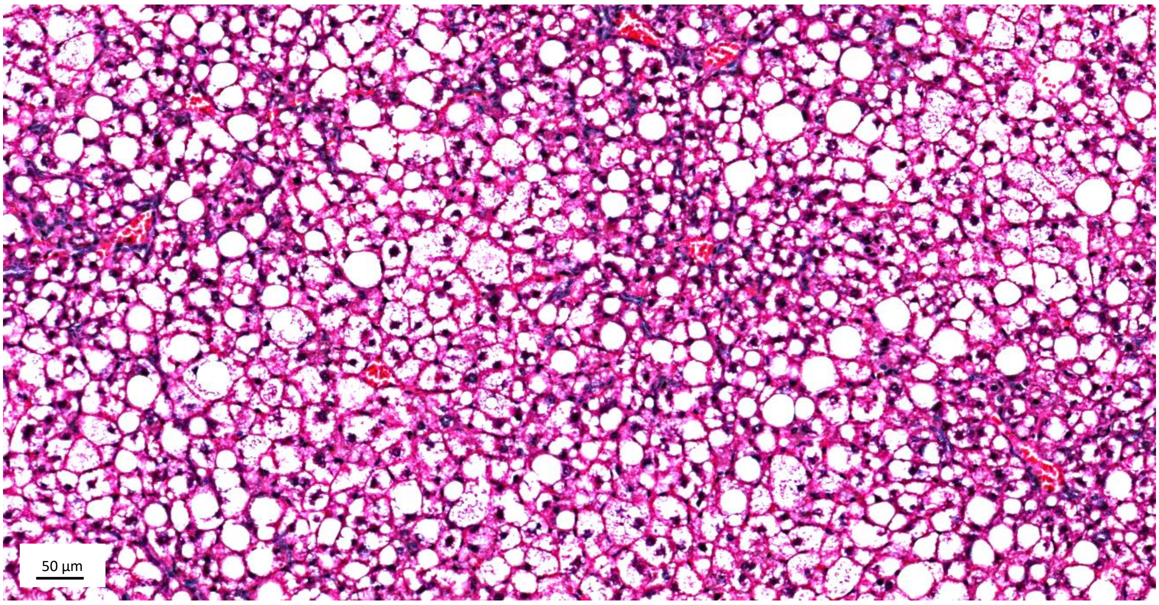

CDHFD + PBS-8

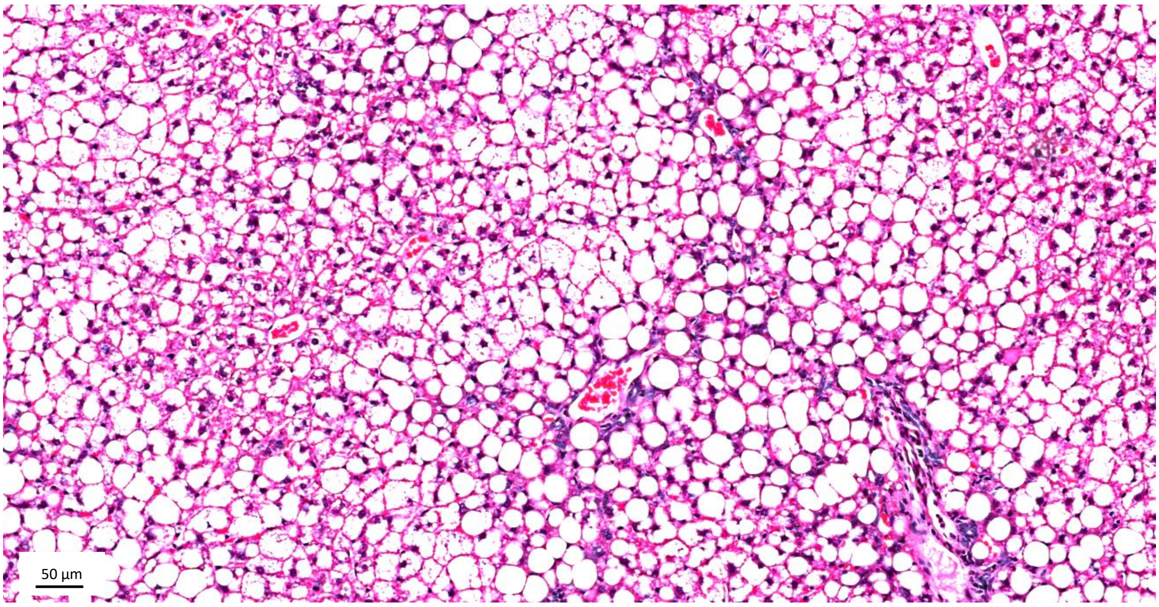

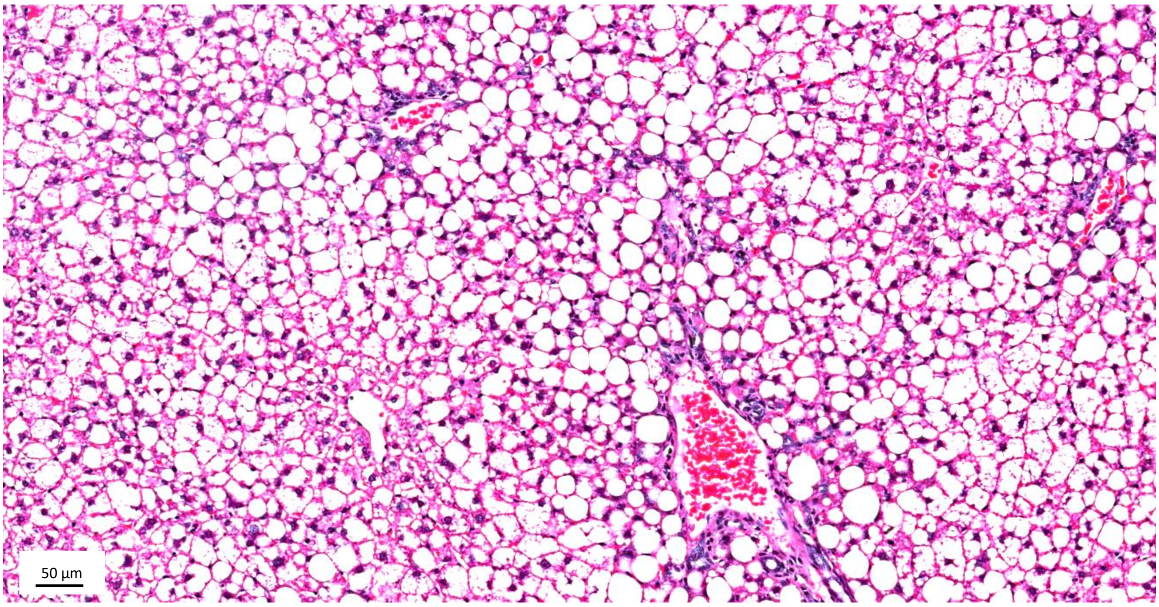

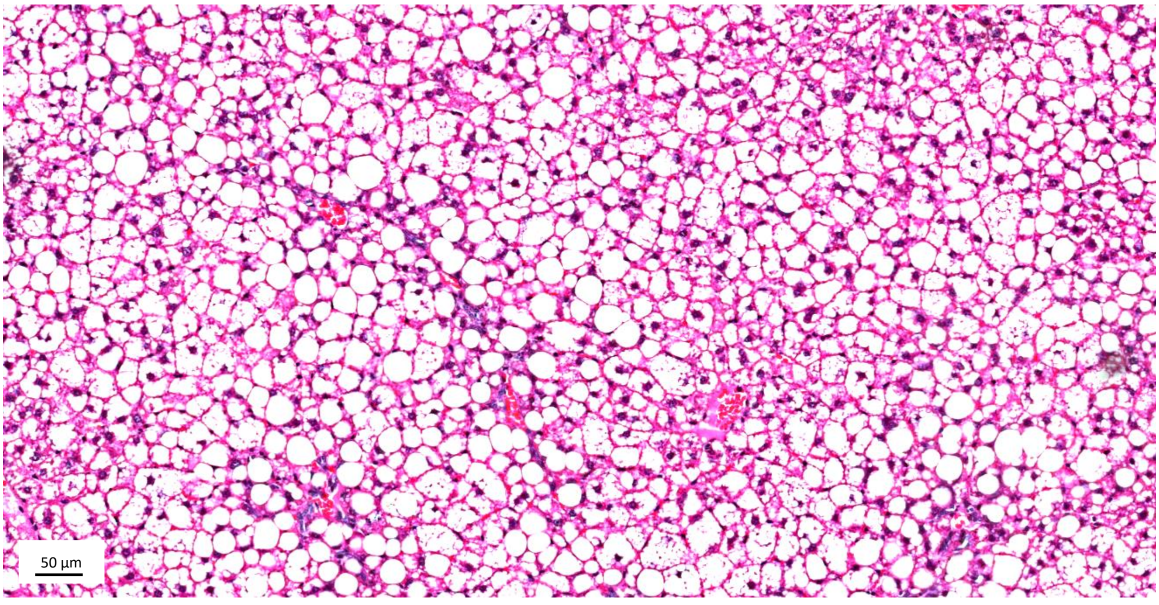

CDHFD + PBS-9

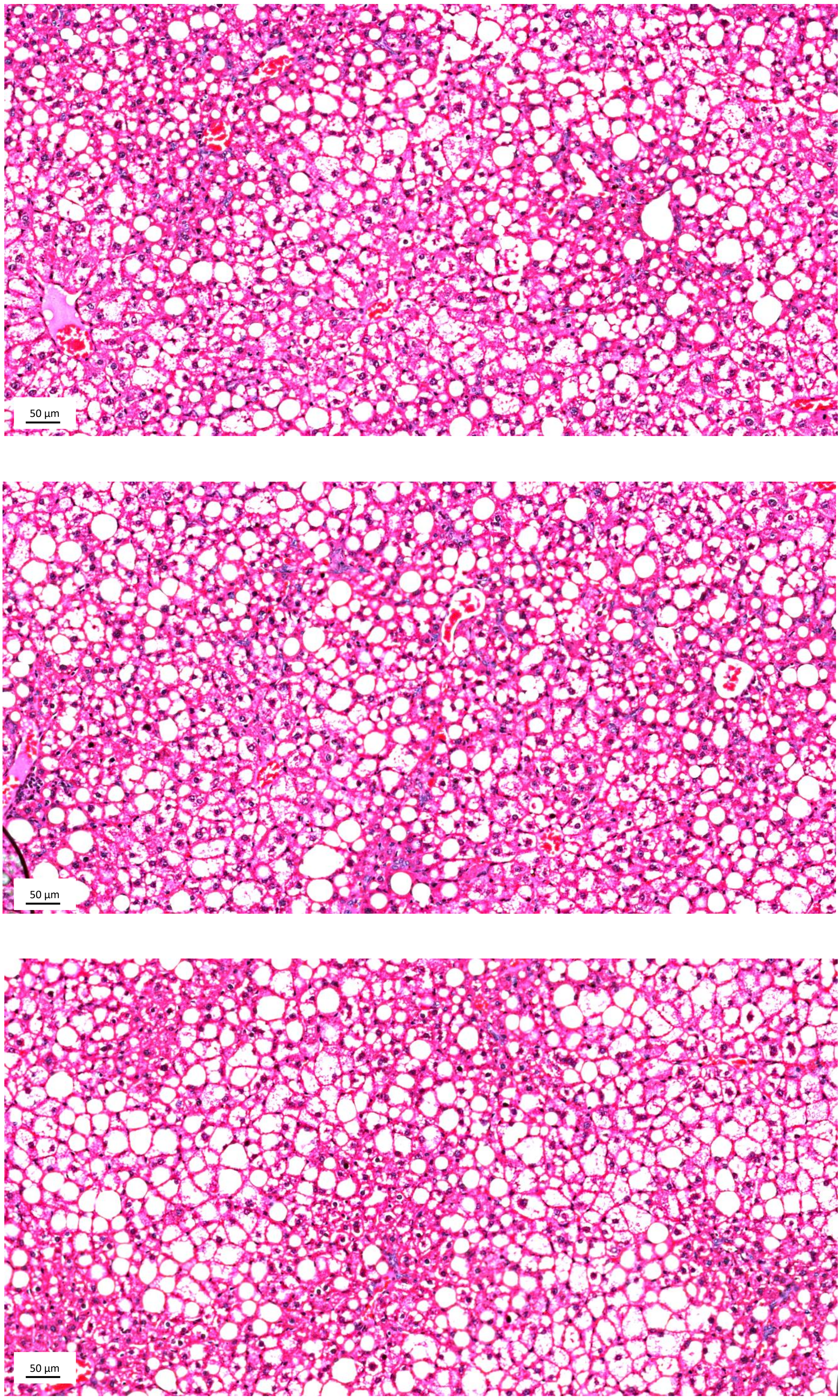

CDHFD + PBS-10

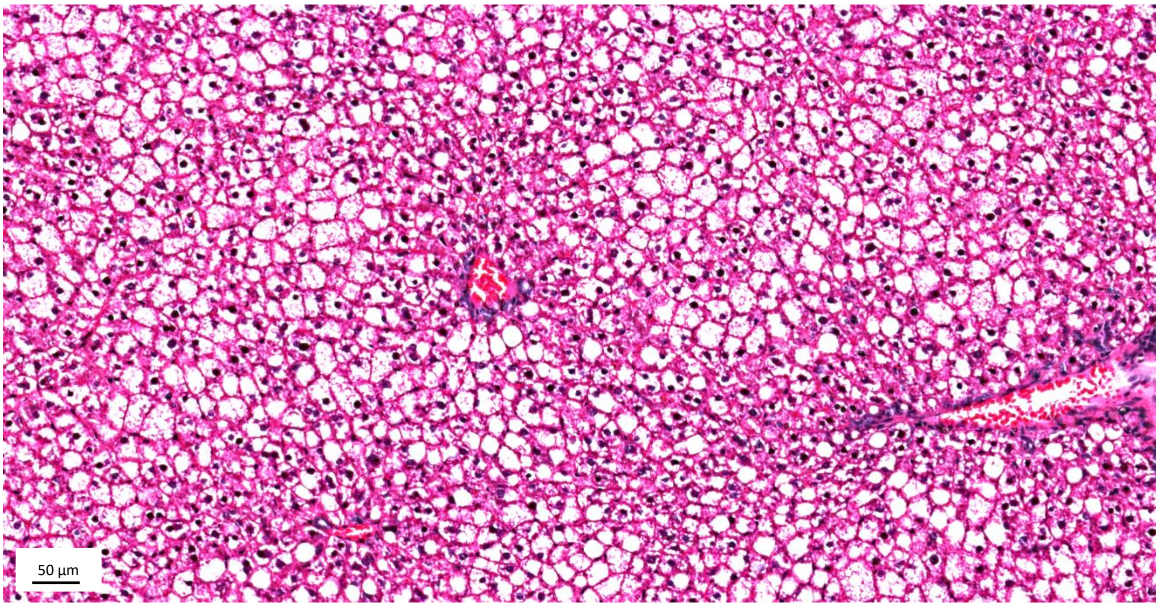

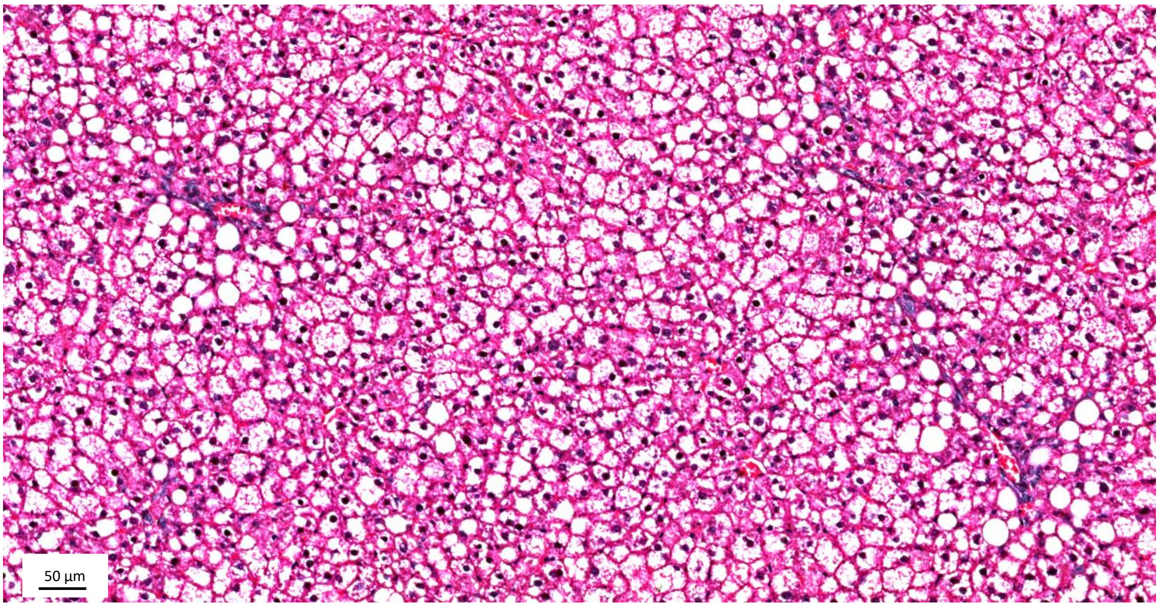

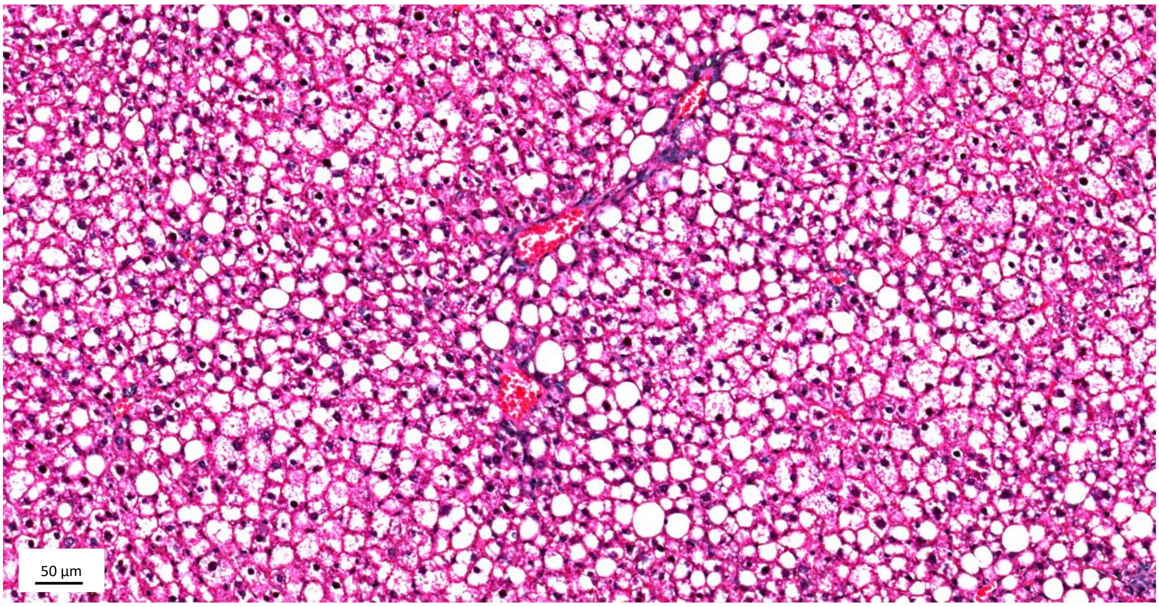

CDHFD + PBS-11

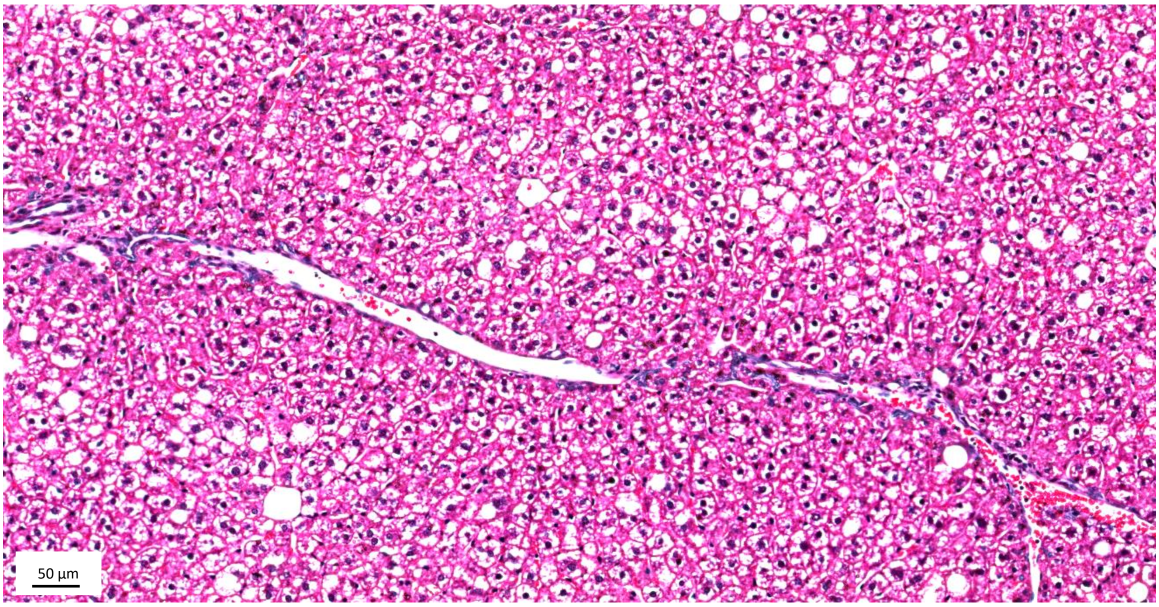

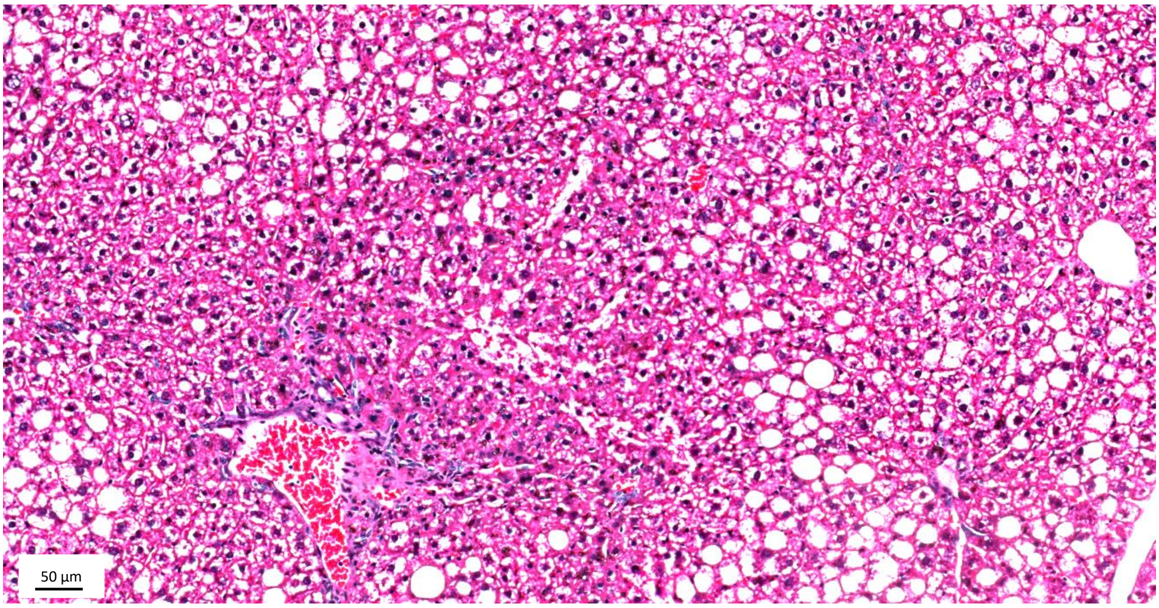

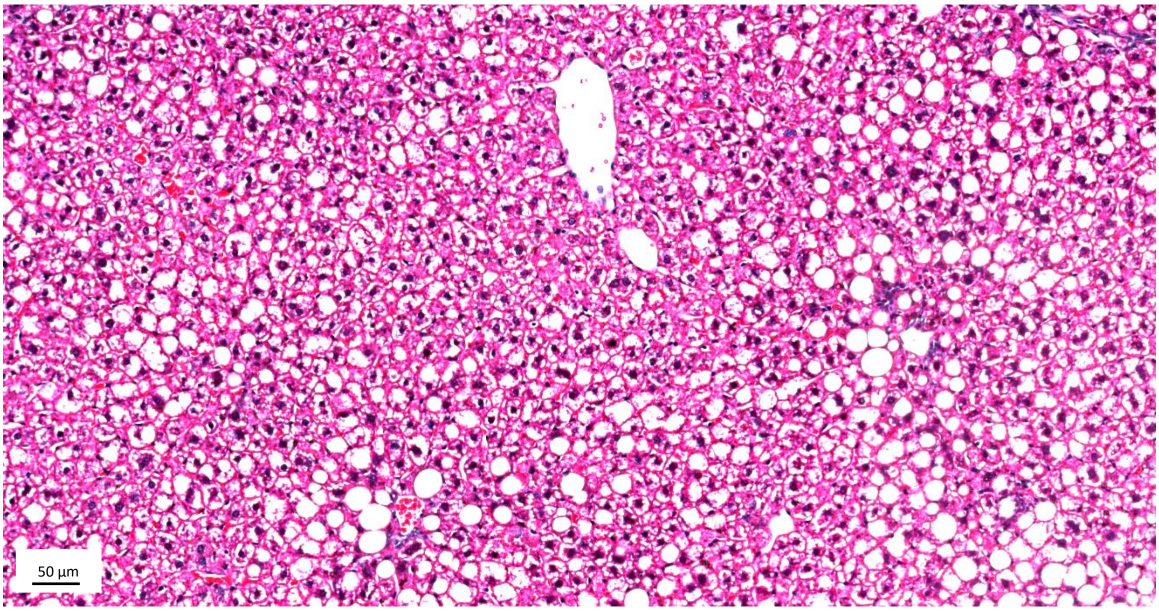

CDHFD + PBS-12

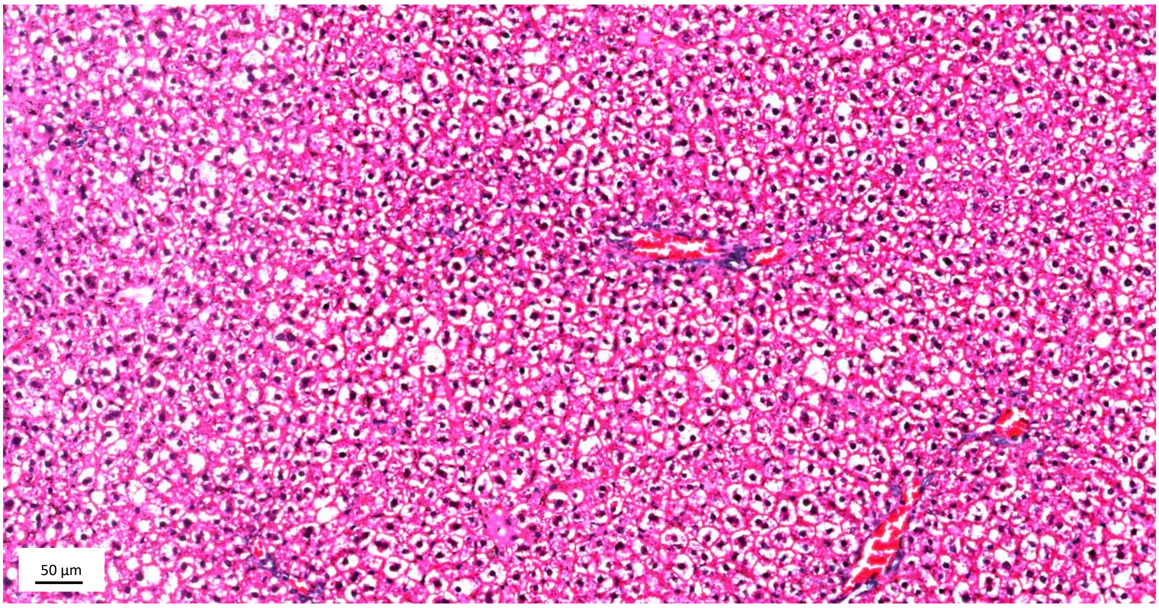

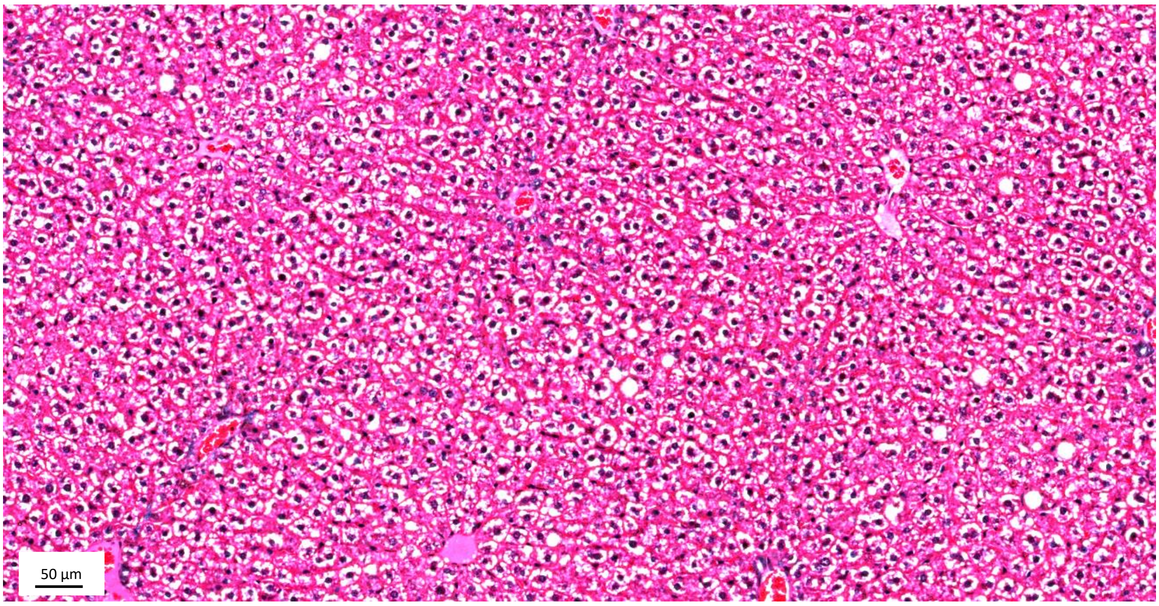

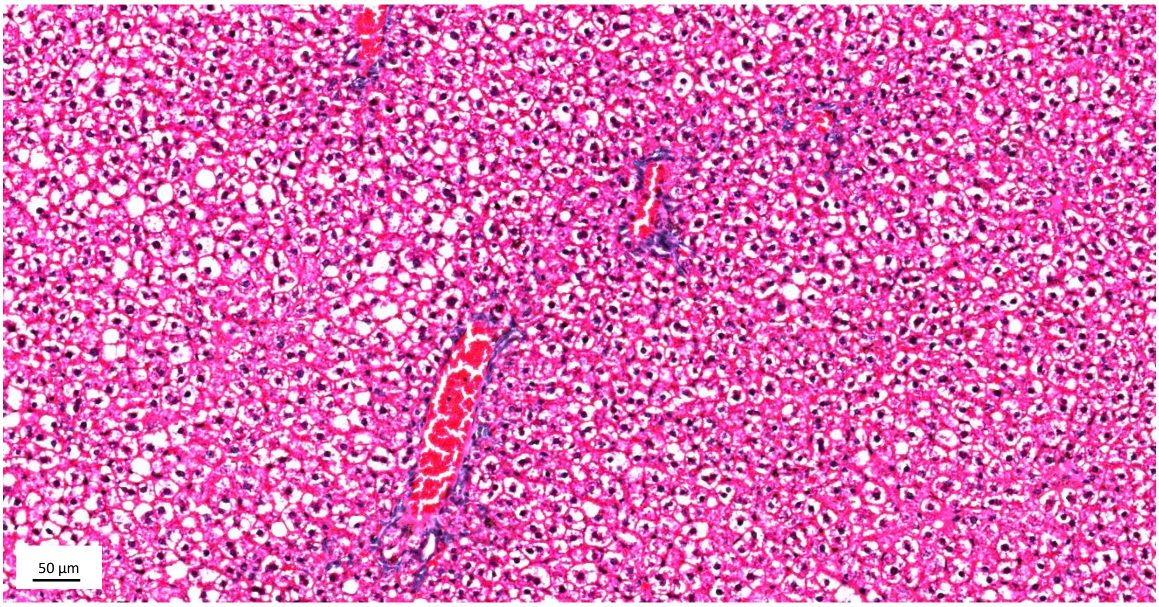

CDHFD + PBS-13

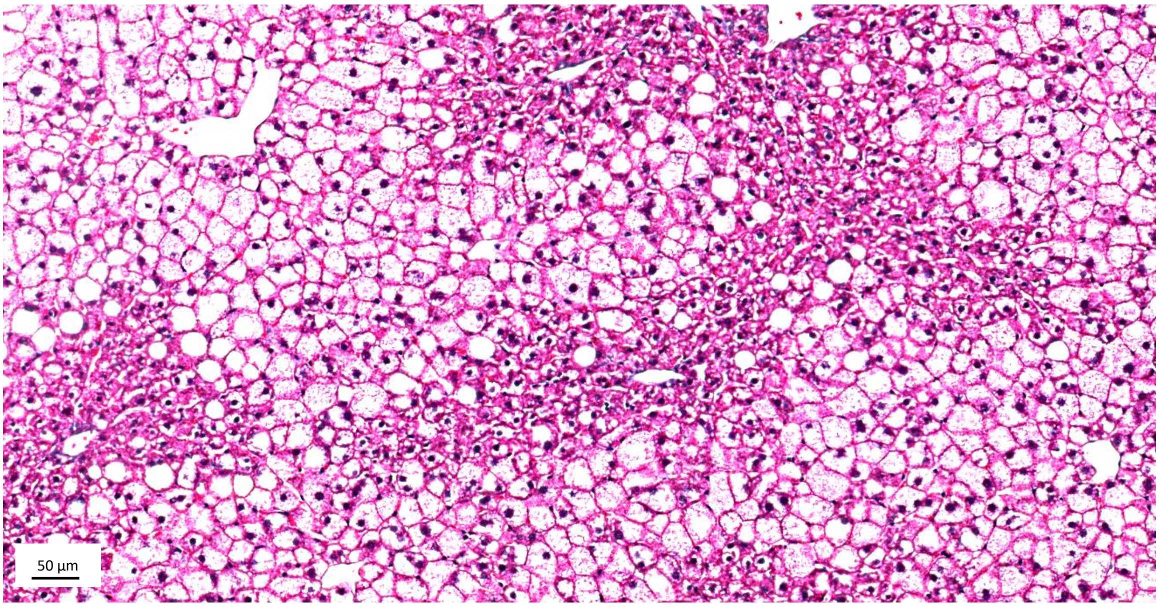

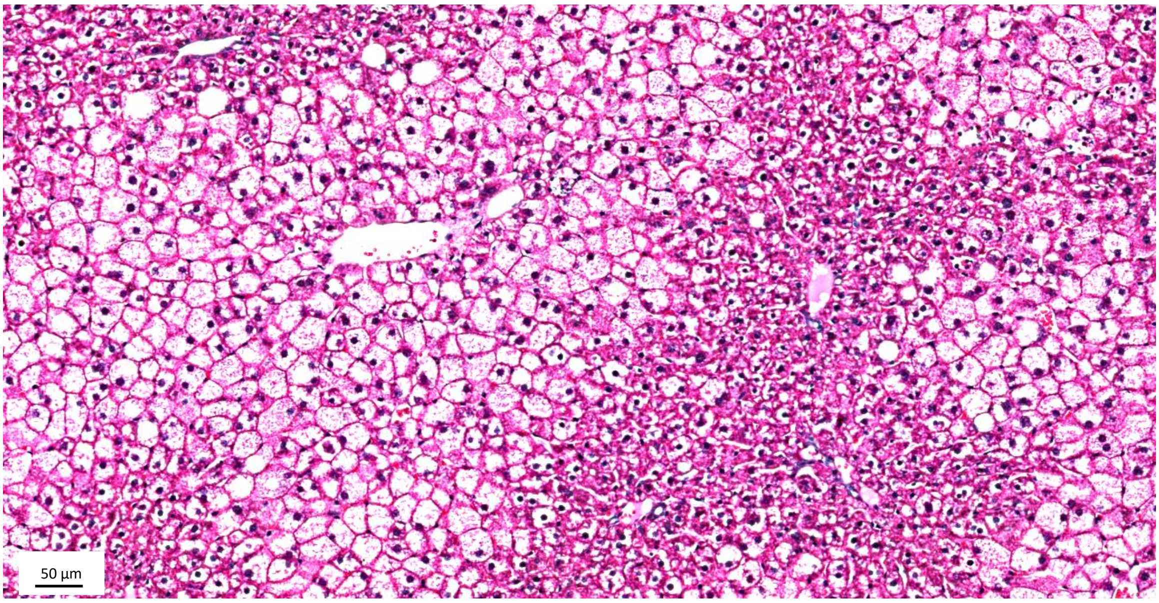

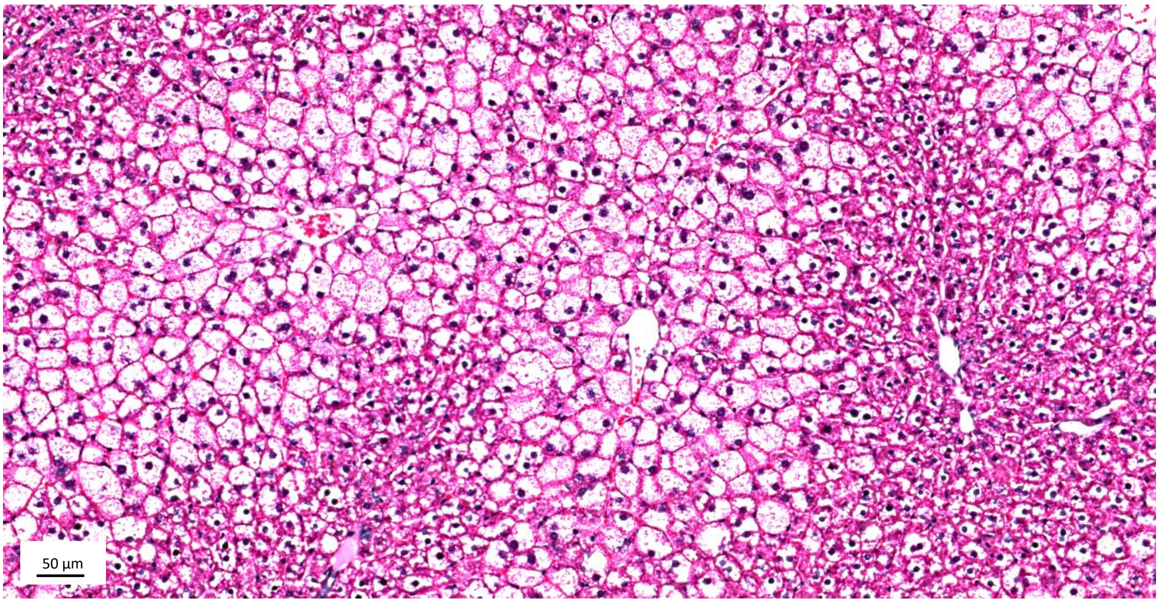

CDHFD + PBS-14

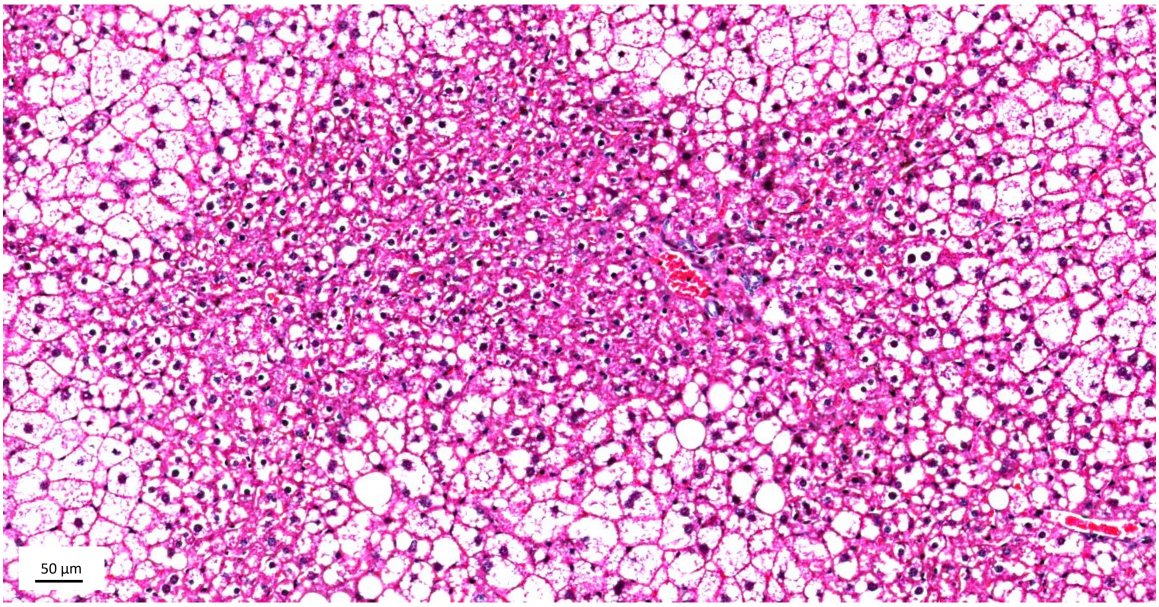

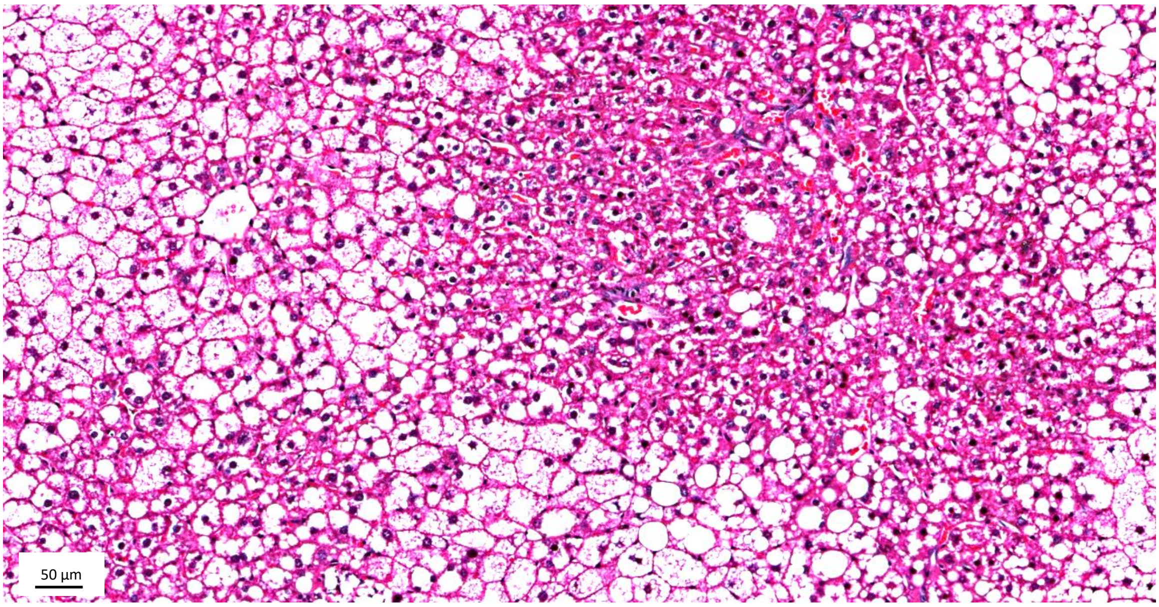

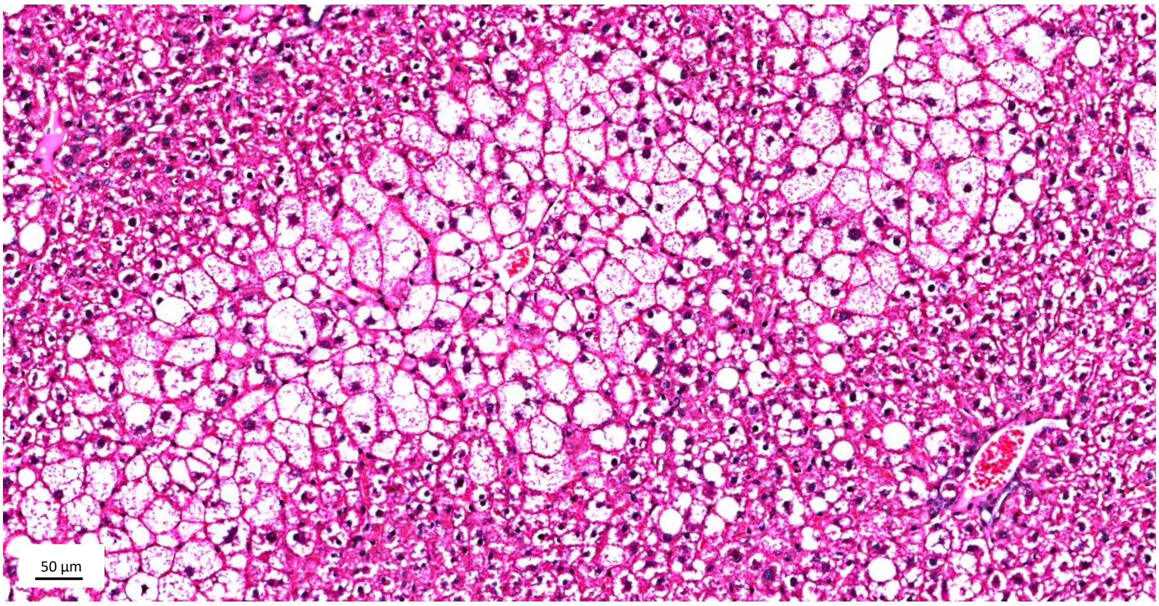

H&E Staining

CDHFD + *E. coli* group

(11 mice were included)

CDHFD + *E. coli* - 1

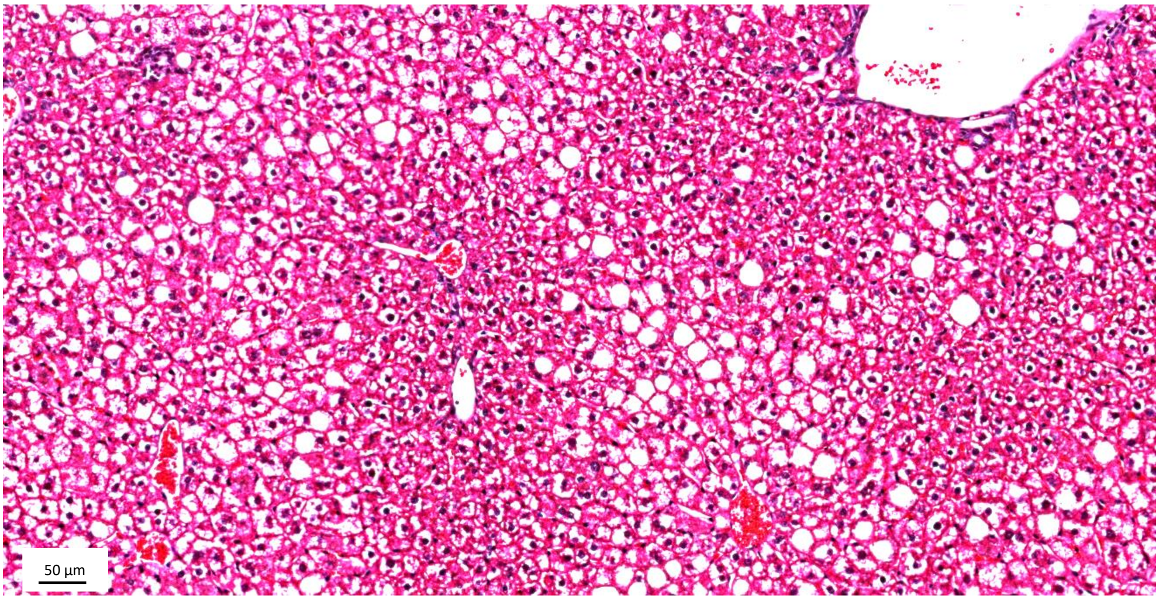

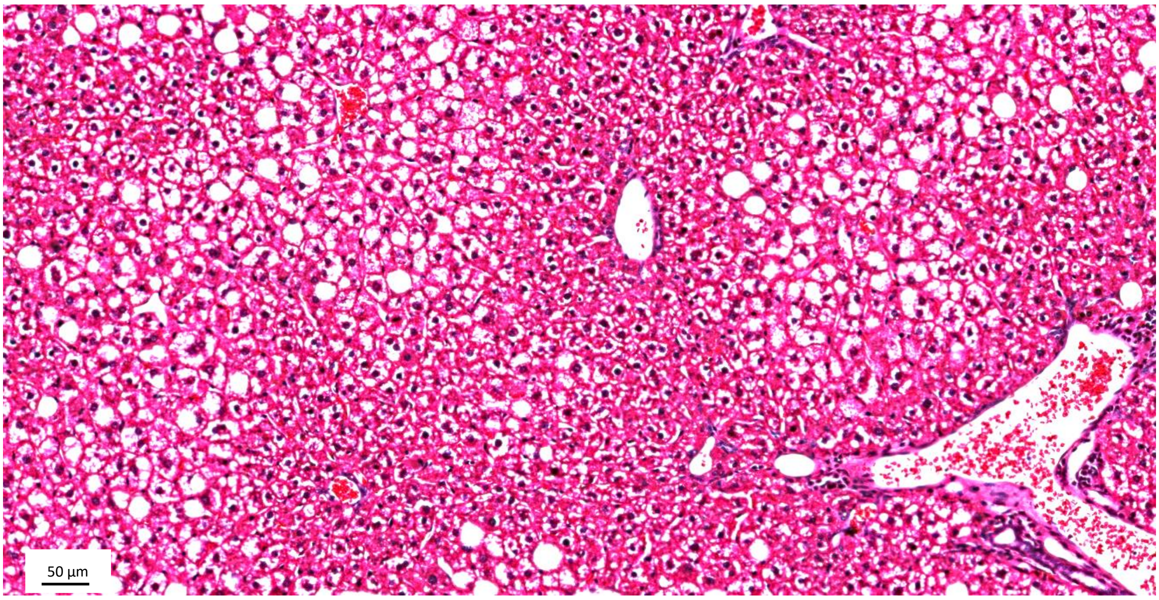

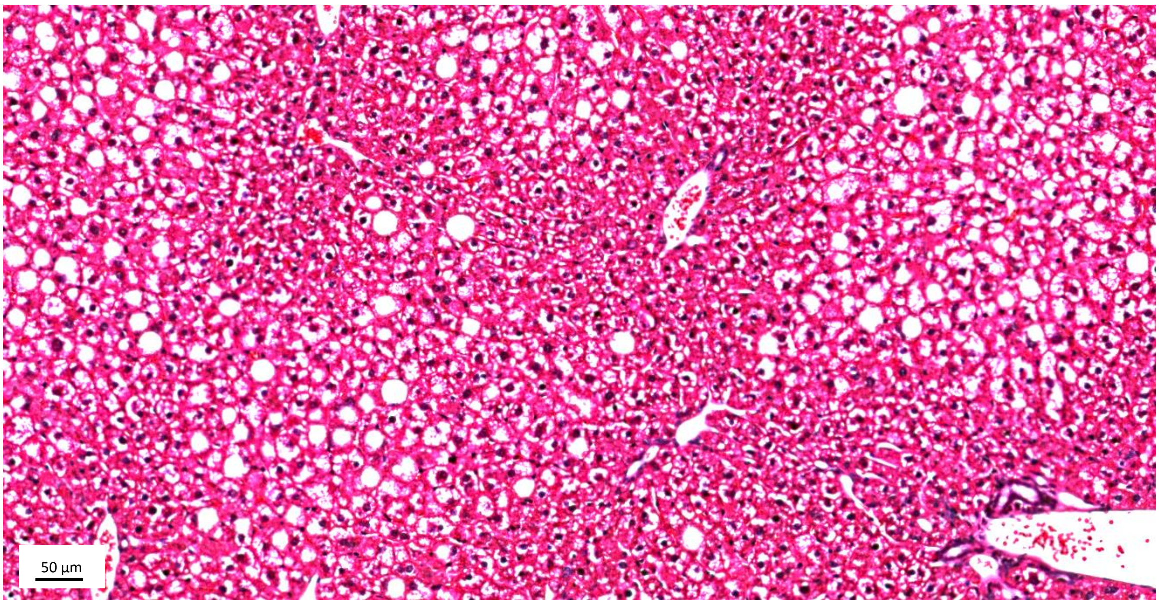

CDHFD + *E. coli* - 2

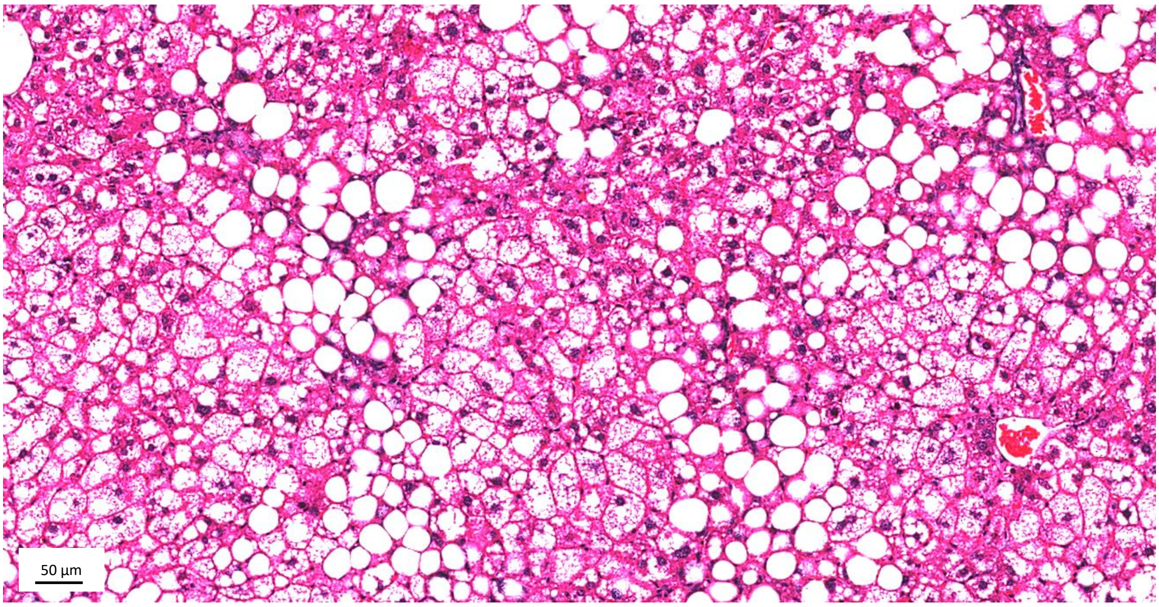

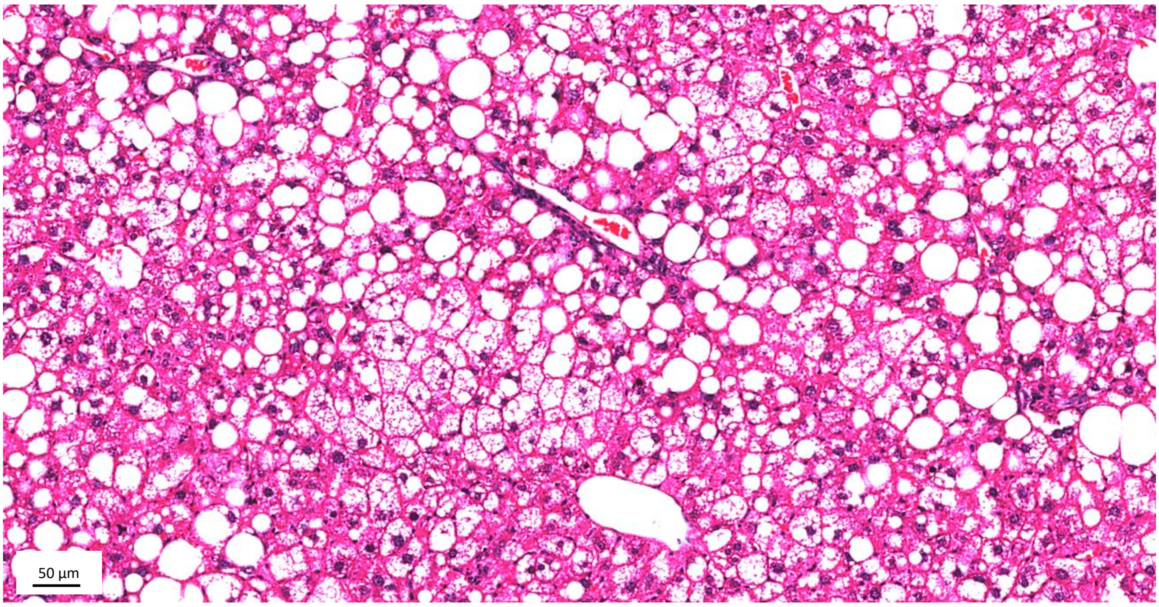

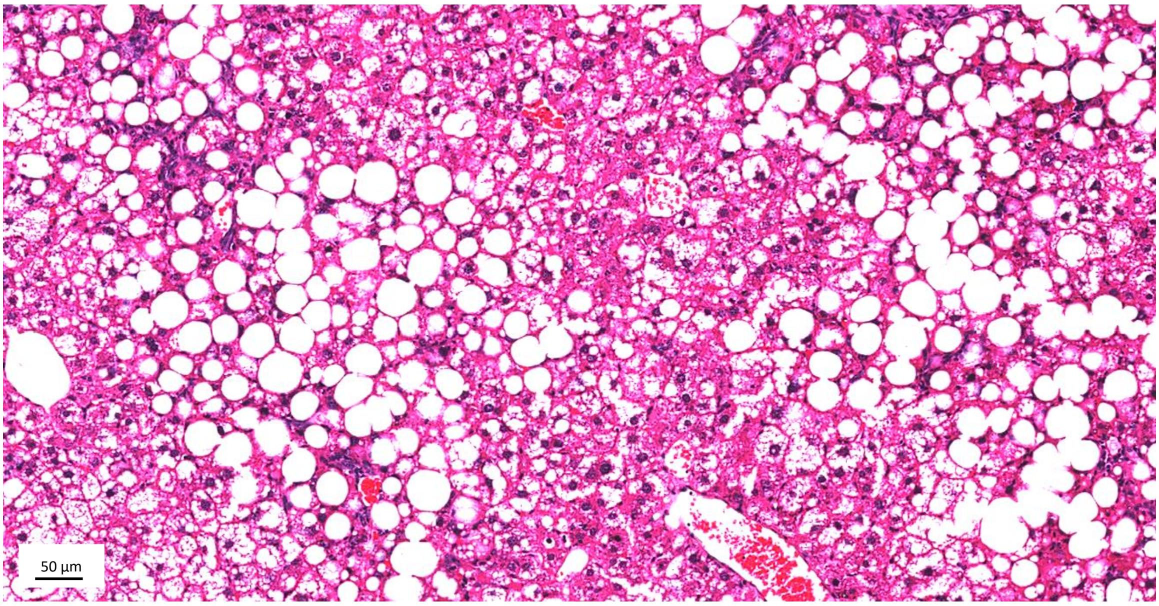

CDHFD + *E. coli* - 3

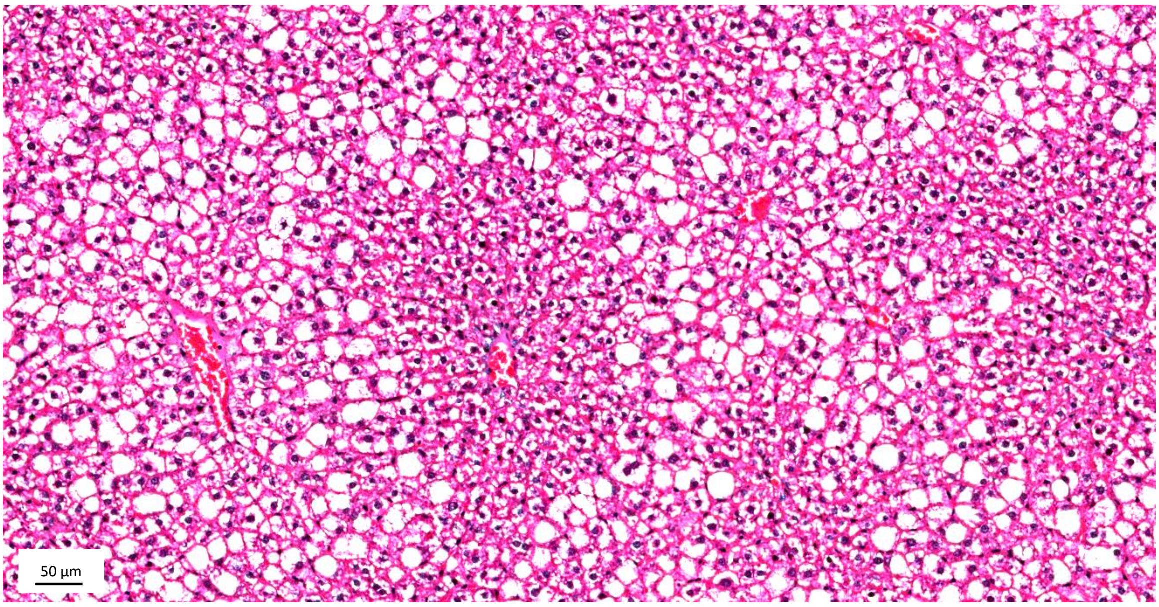

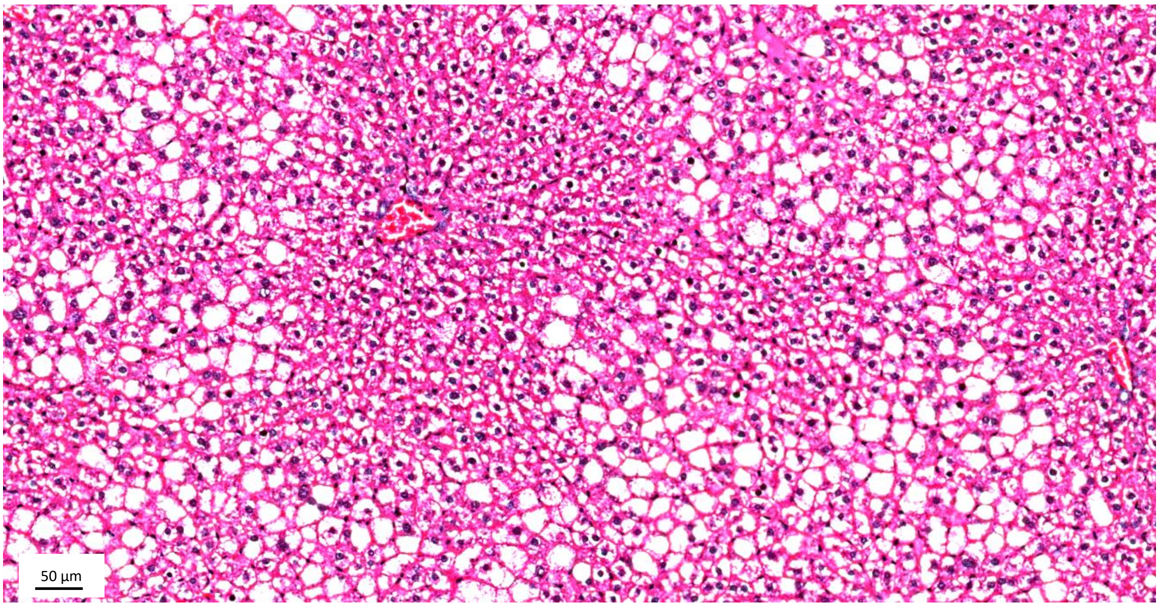

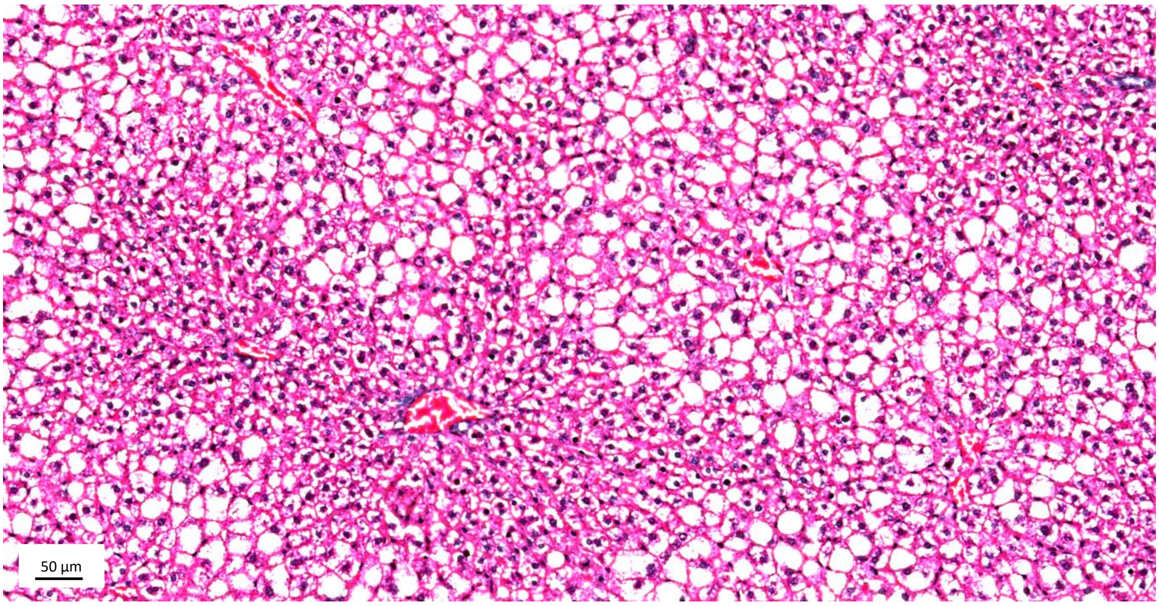

CDHFD + *E. coli* - 4

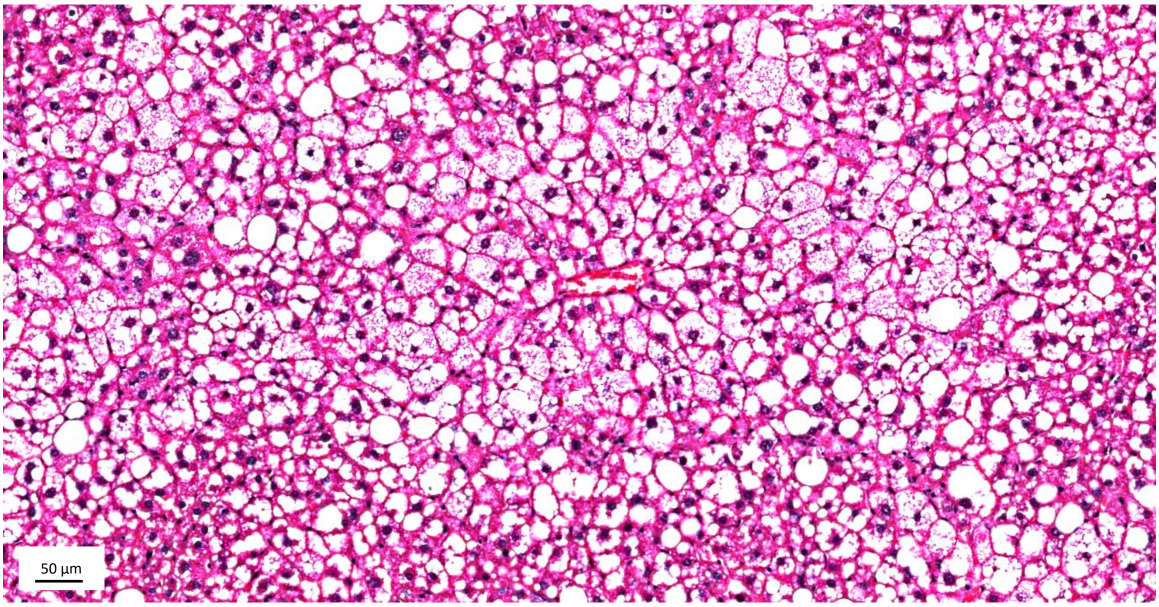

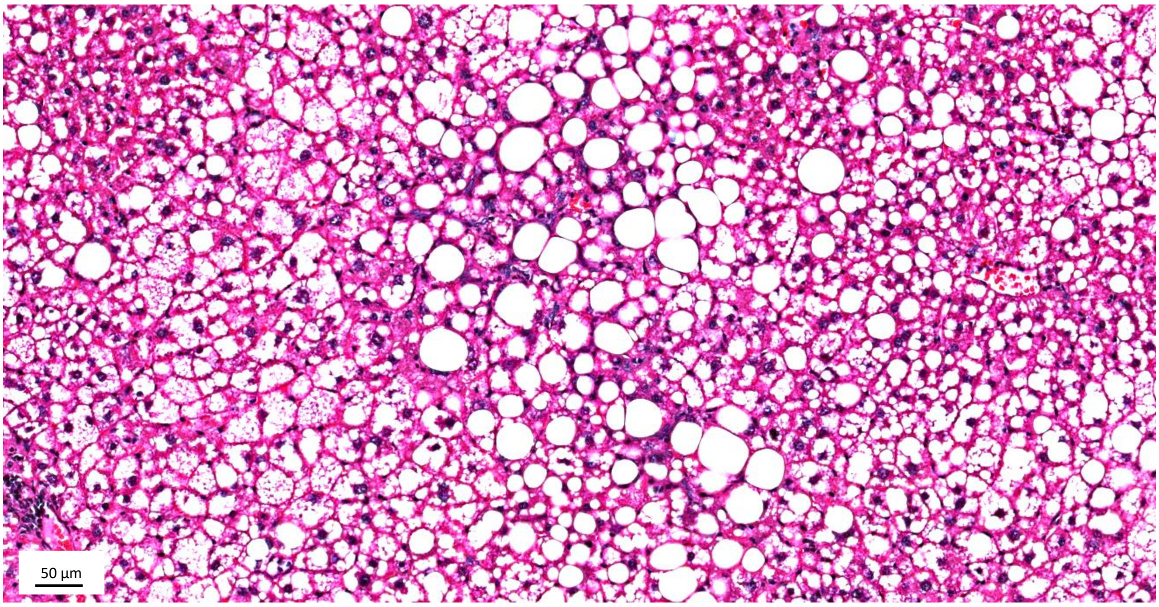

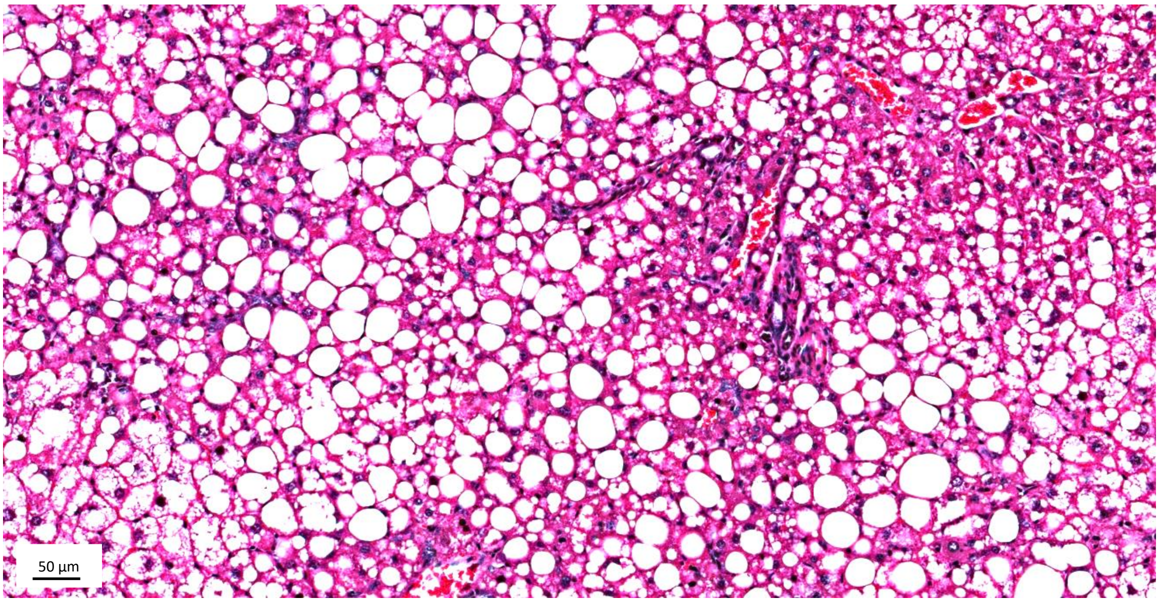

CDHFD + *E. coli* - 5

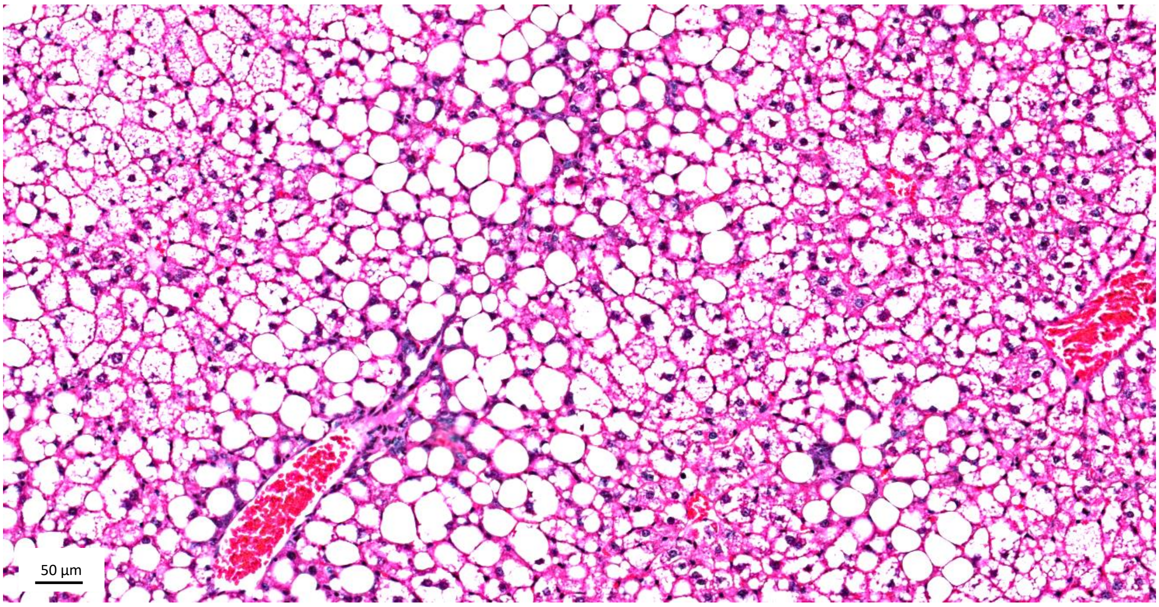

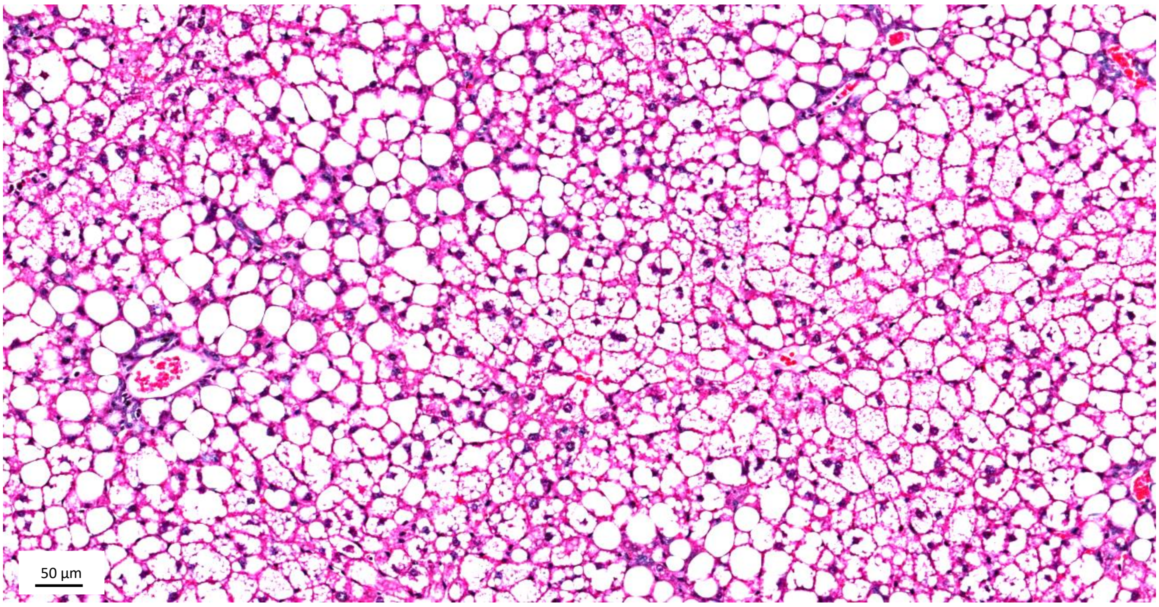

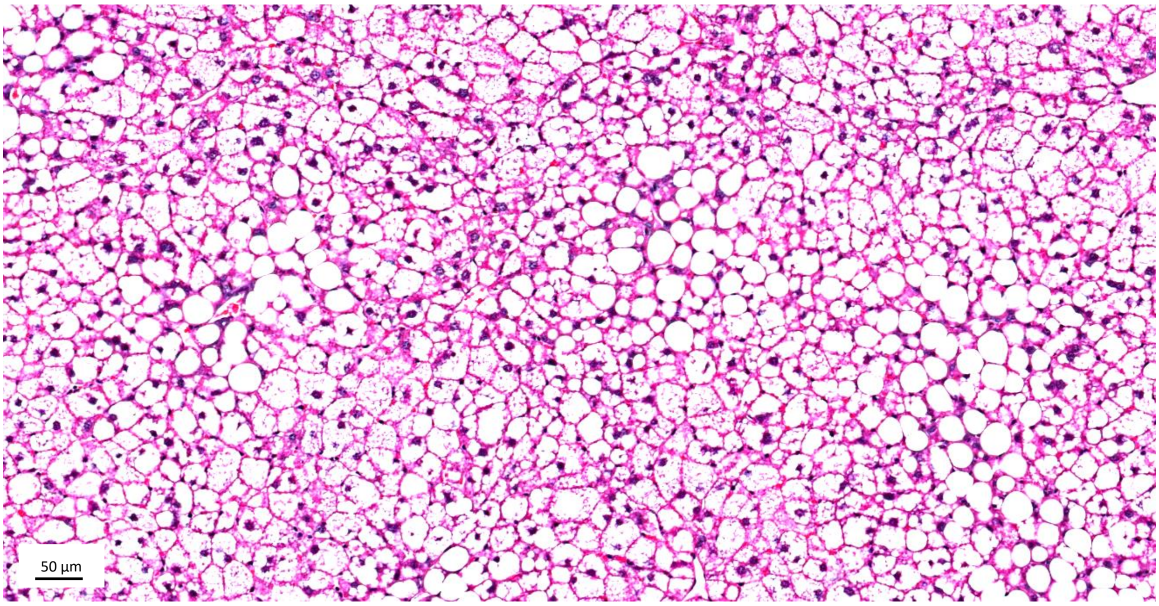

CDHFD + *E. coli* - 6

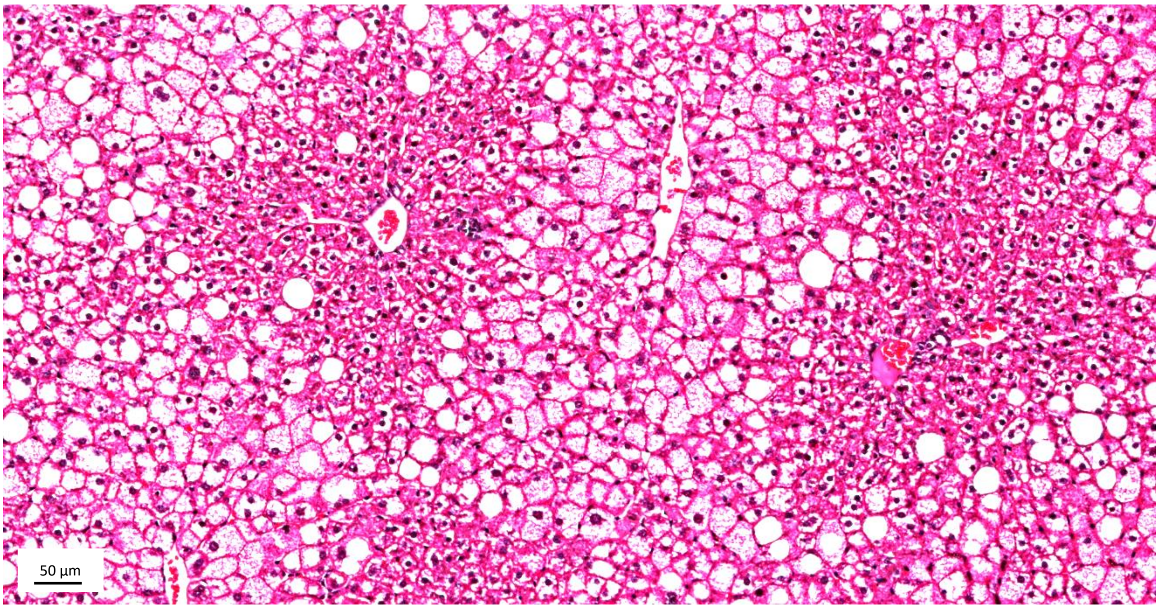

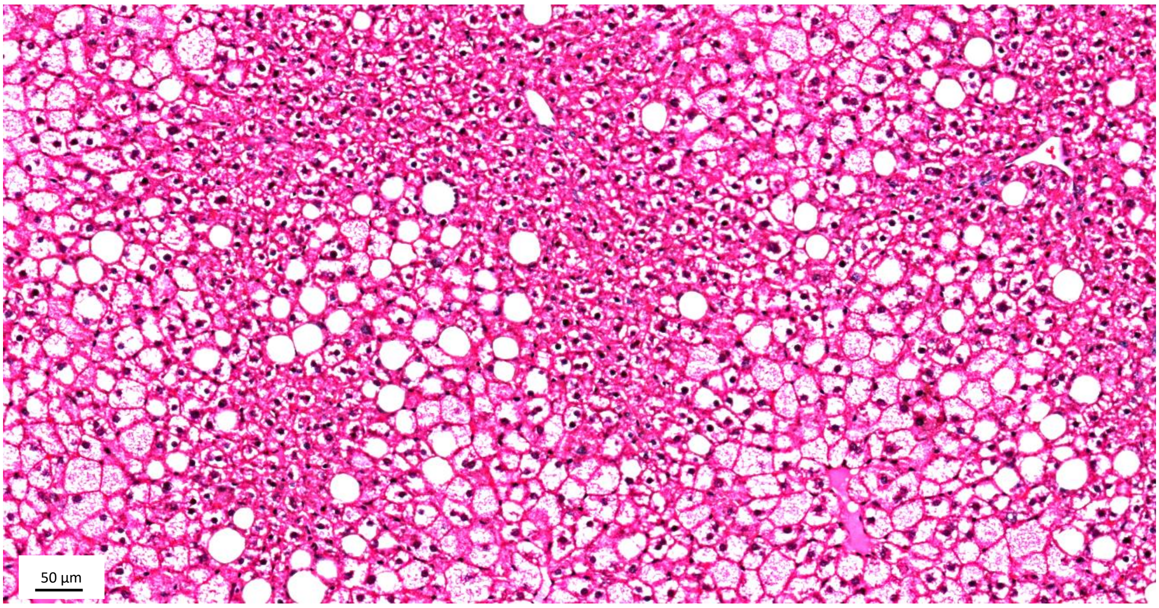

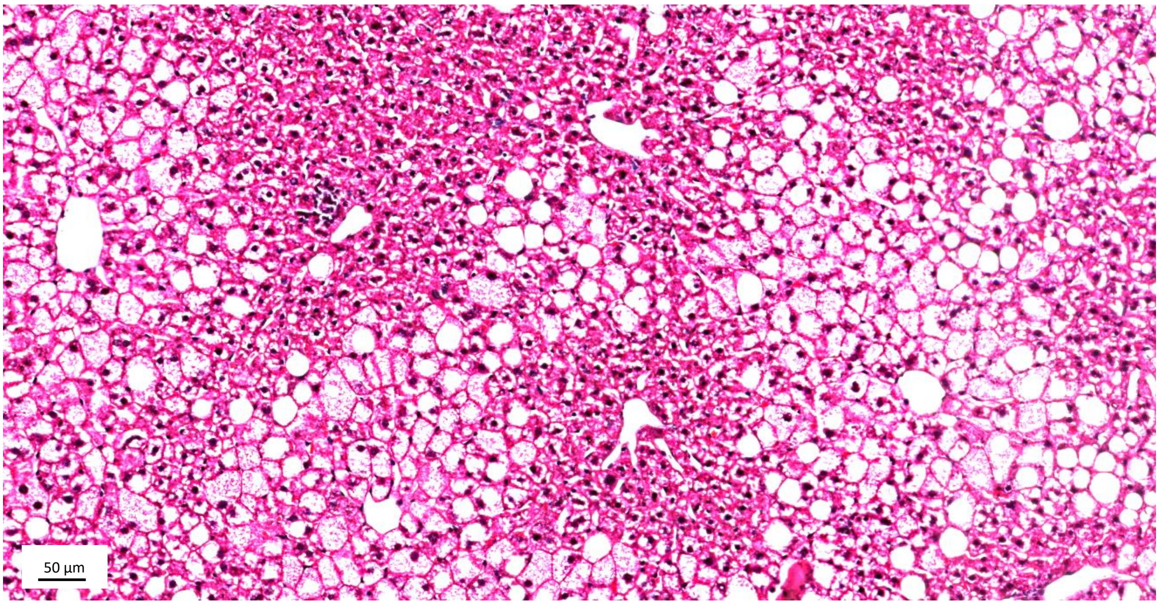

CDHFD + *E. coli* - 7

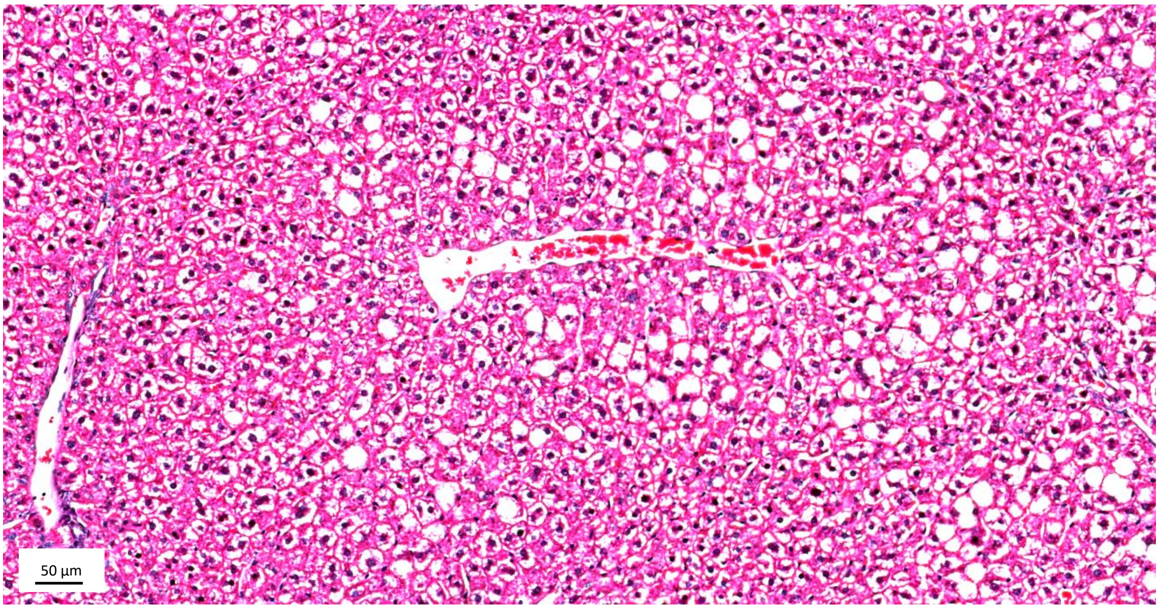

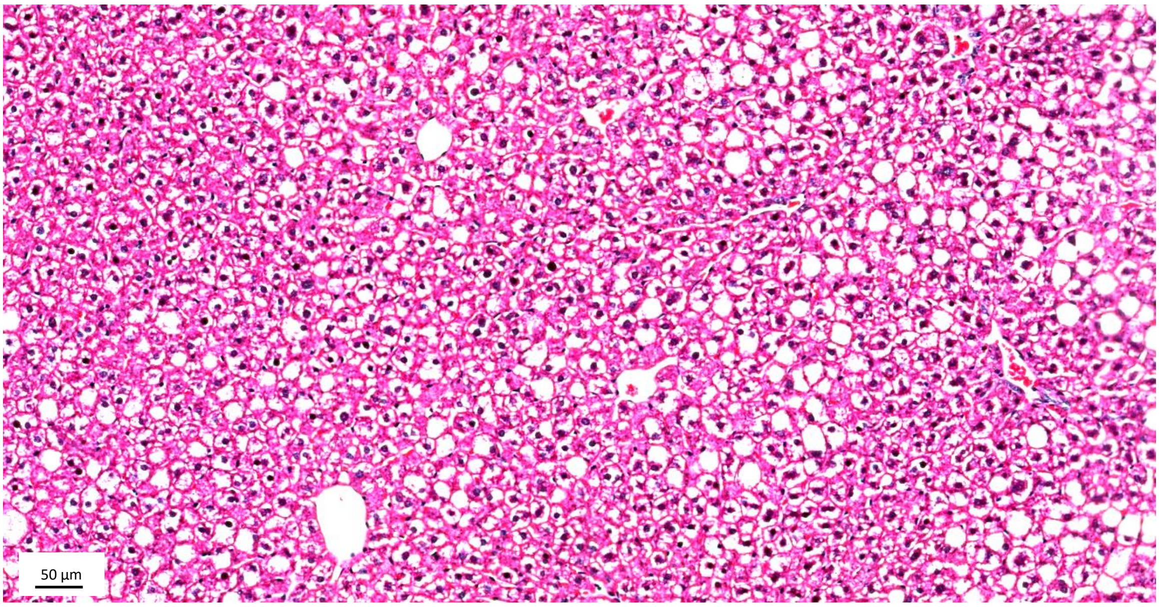

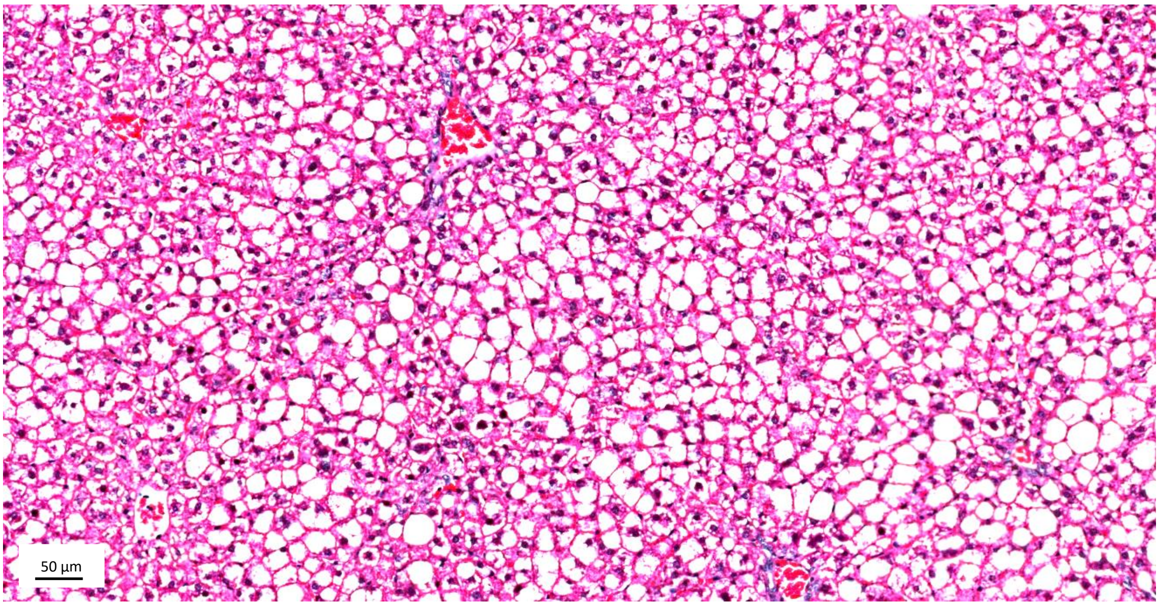

CDHFD + *E. coli* - 8

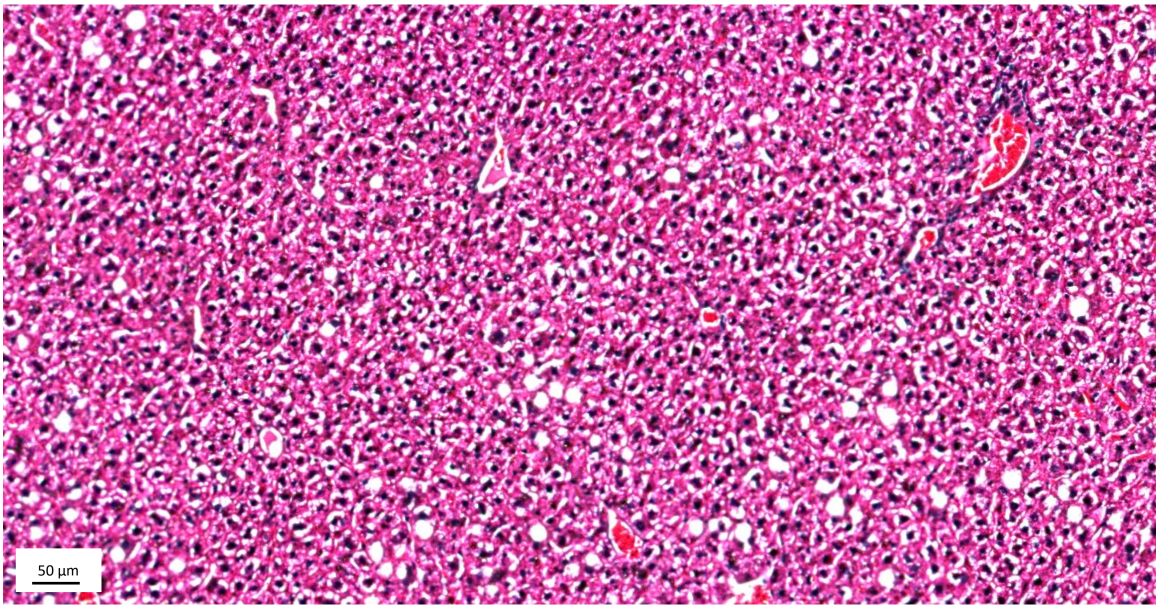

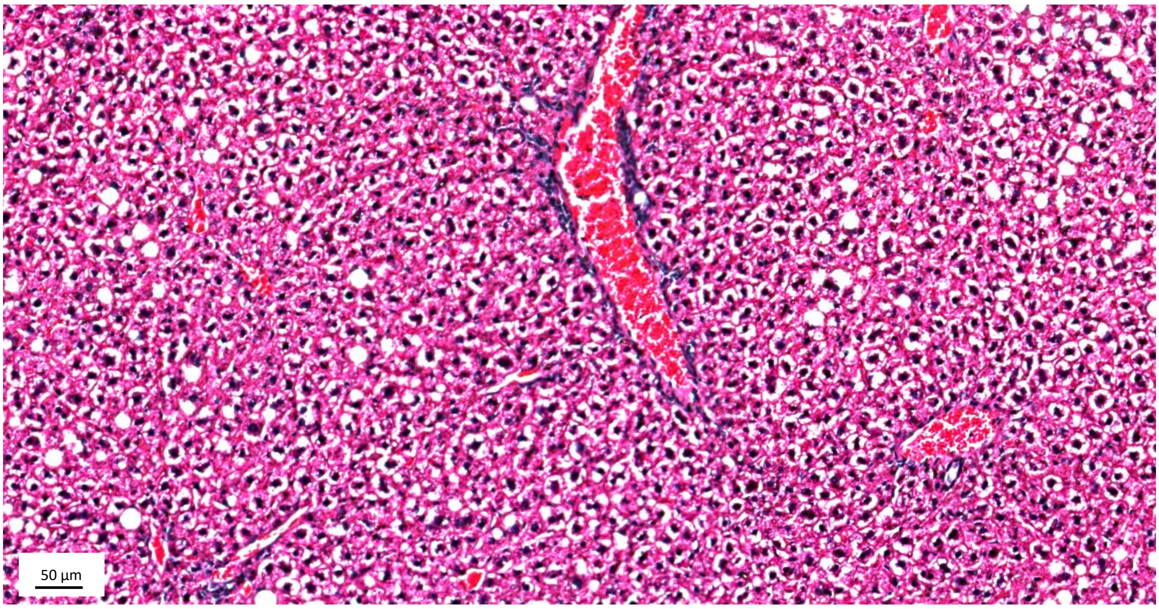

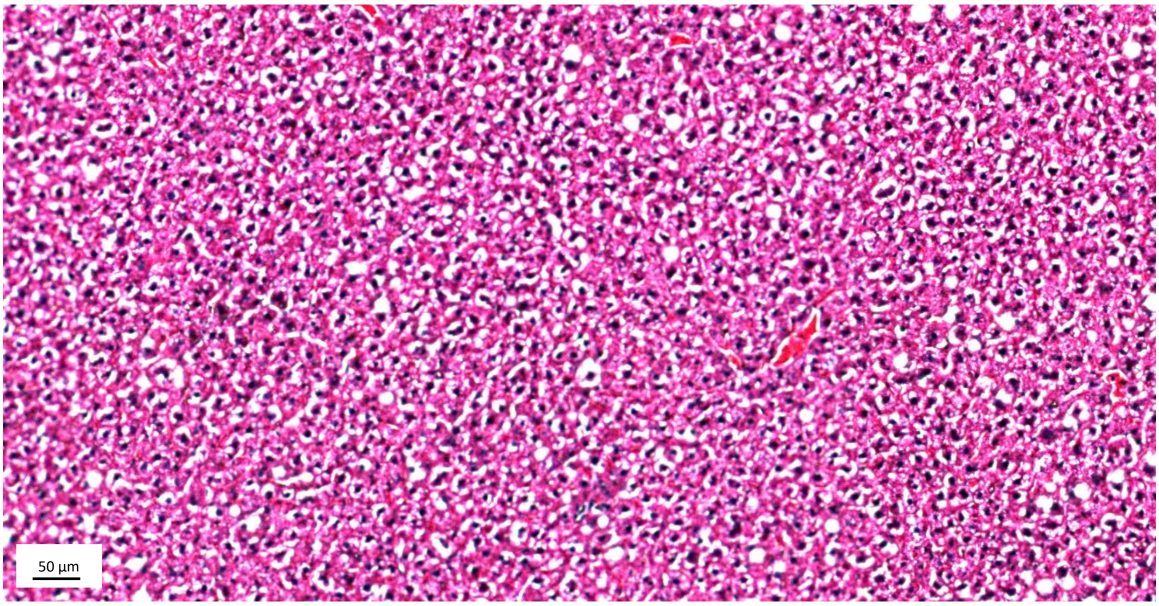

CDHFD + *E. coli* - 9

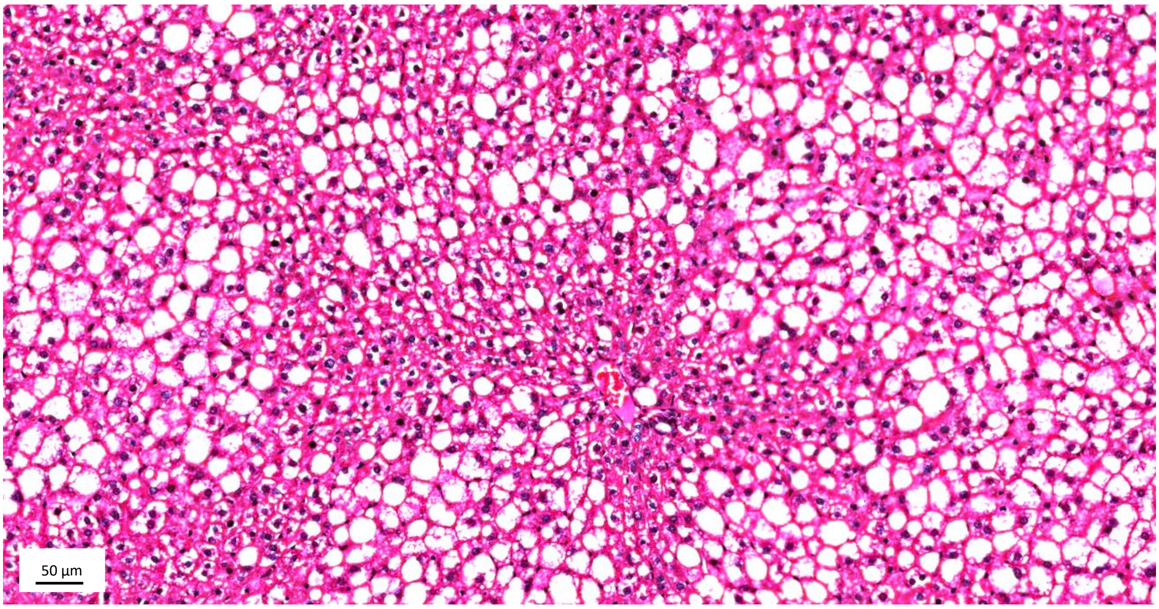

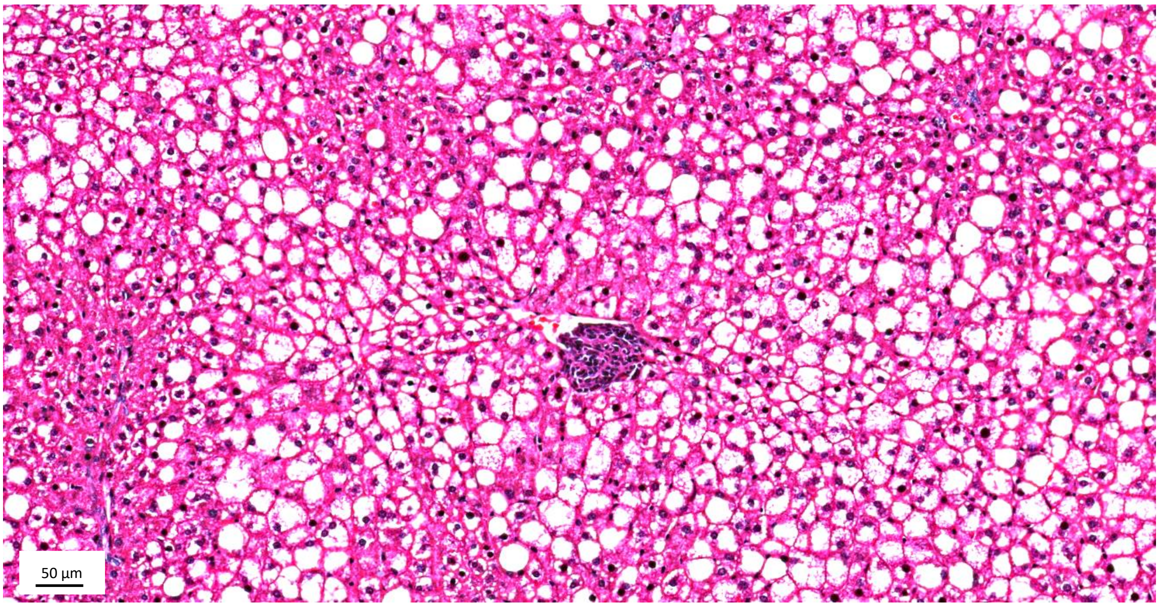

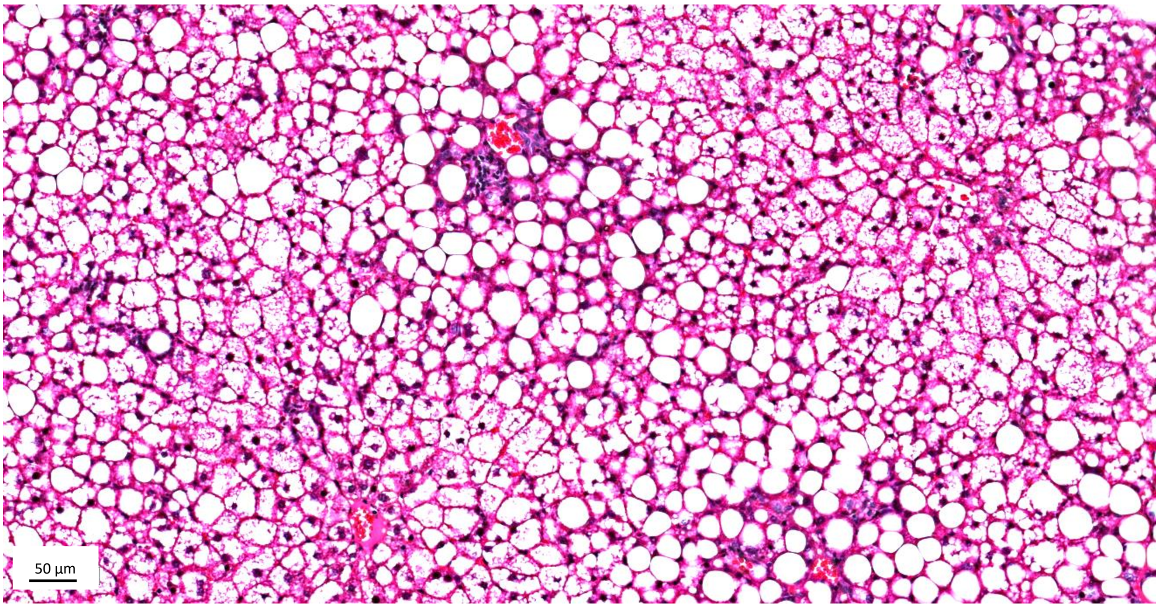

CDHFD + *E. coli* - 10

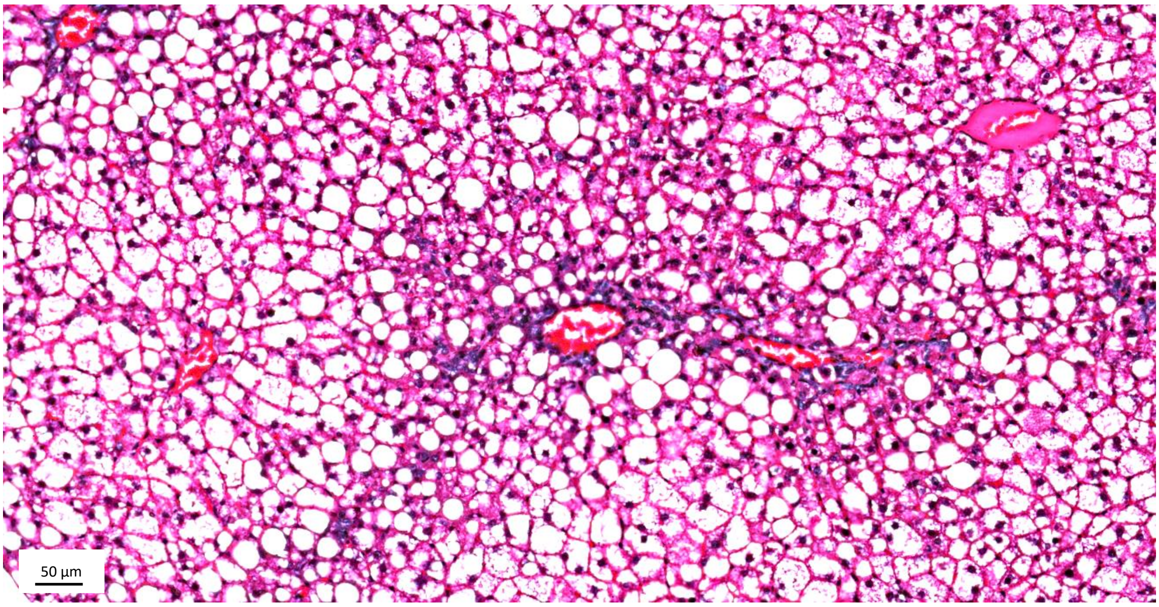

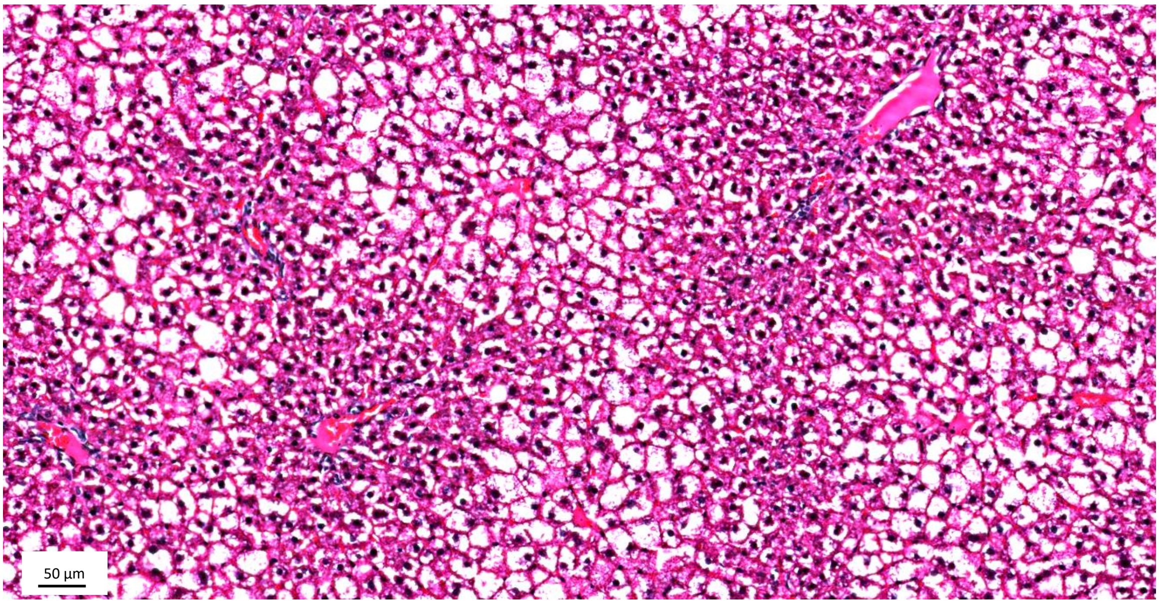

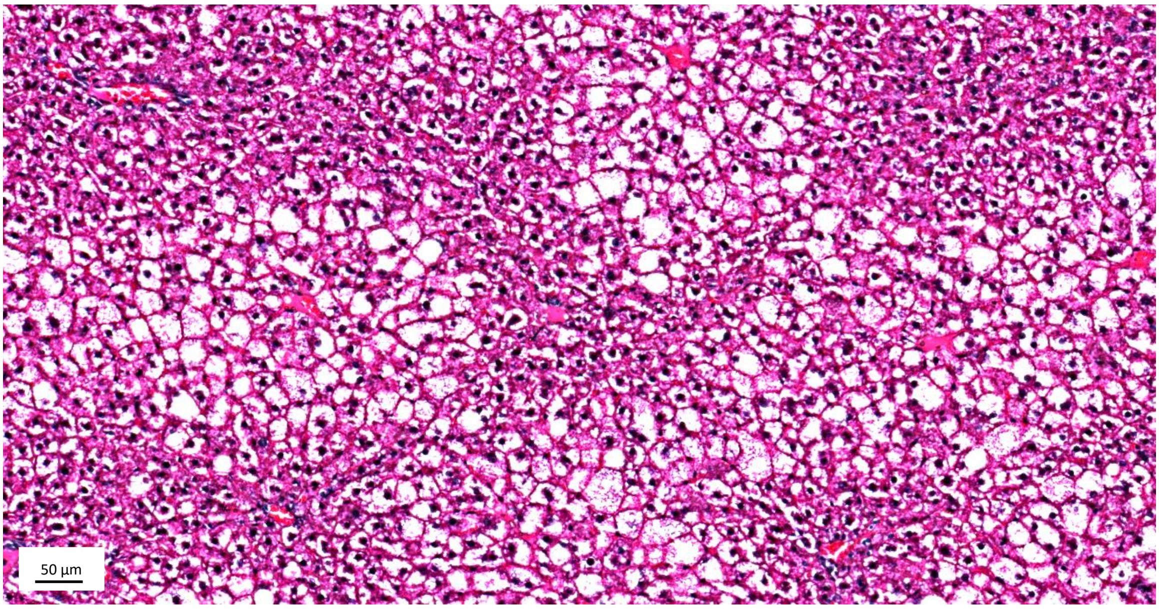

CDHFD + *E. coli* - 11

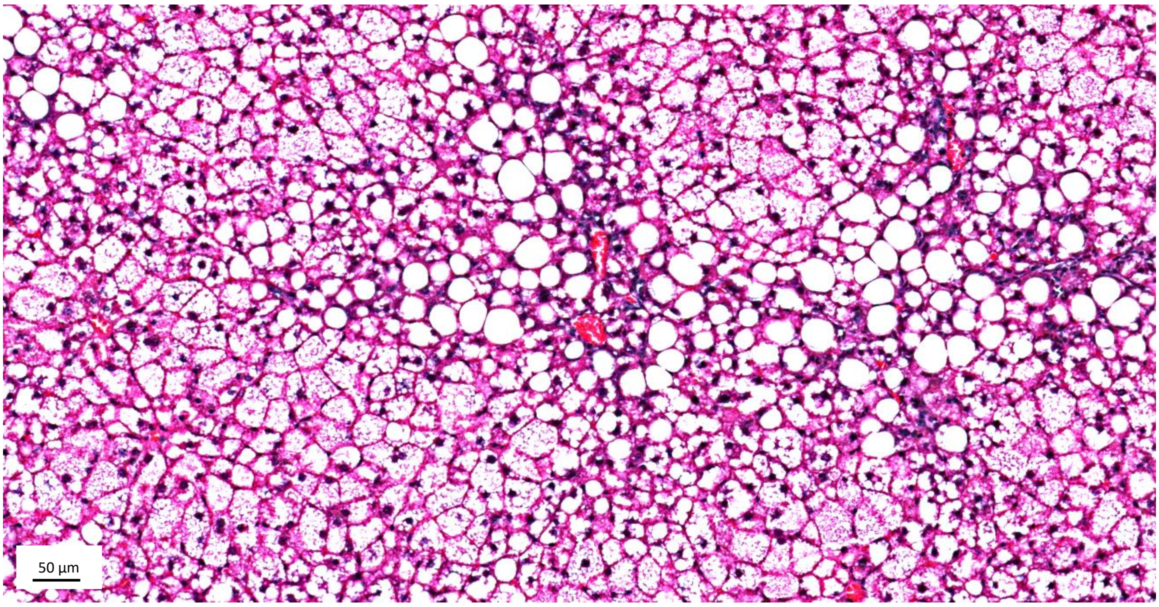

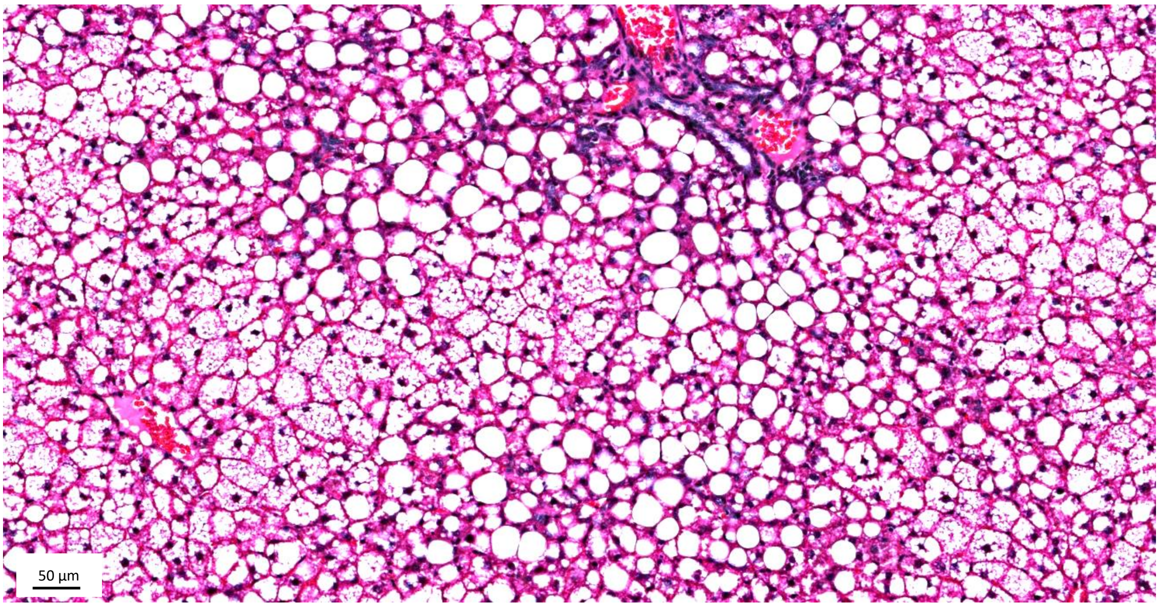

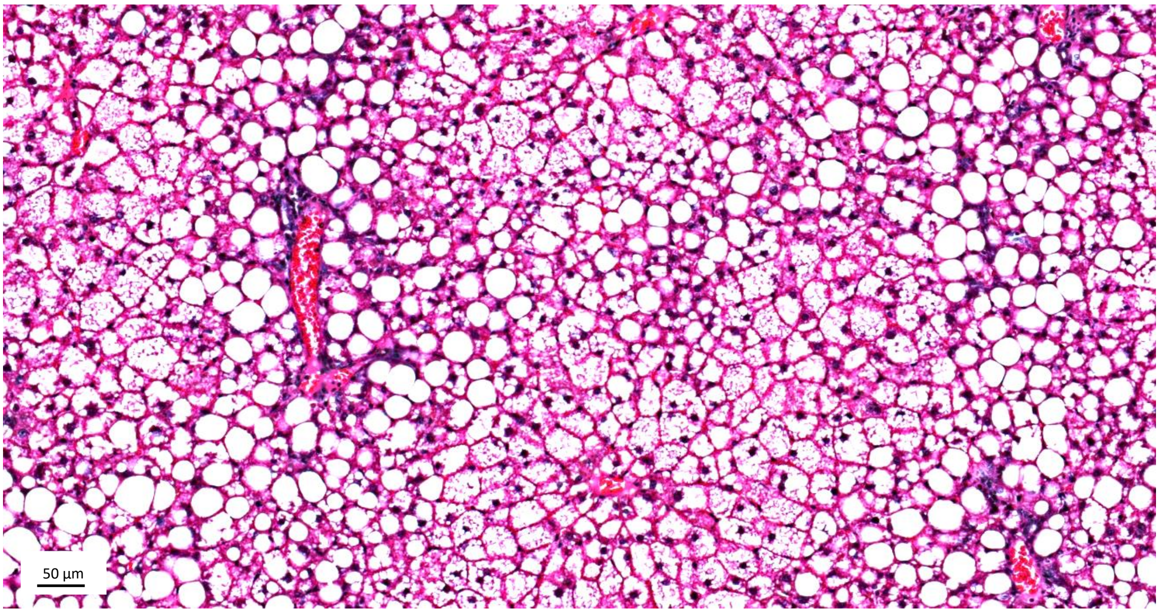

H&E Staining

CDHFD + *P. distasonis* group

(12 mice were included)

CDHFD + *P . distasonis* - 1

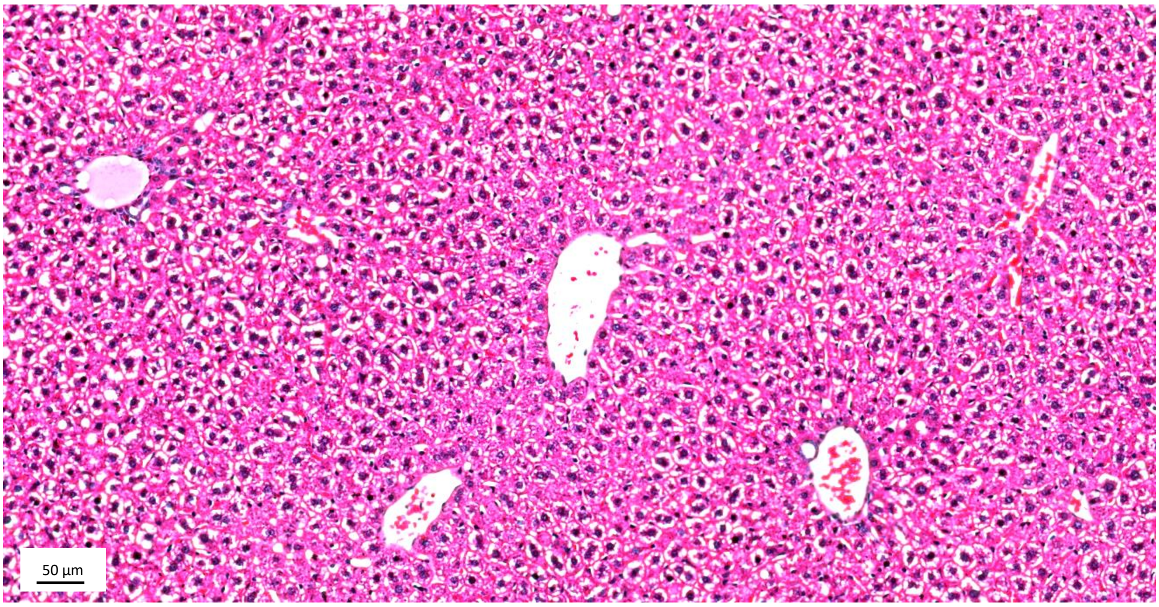

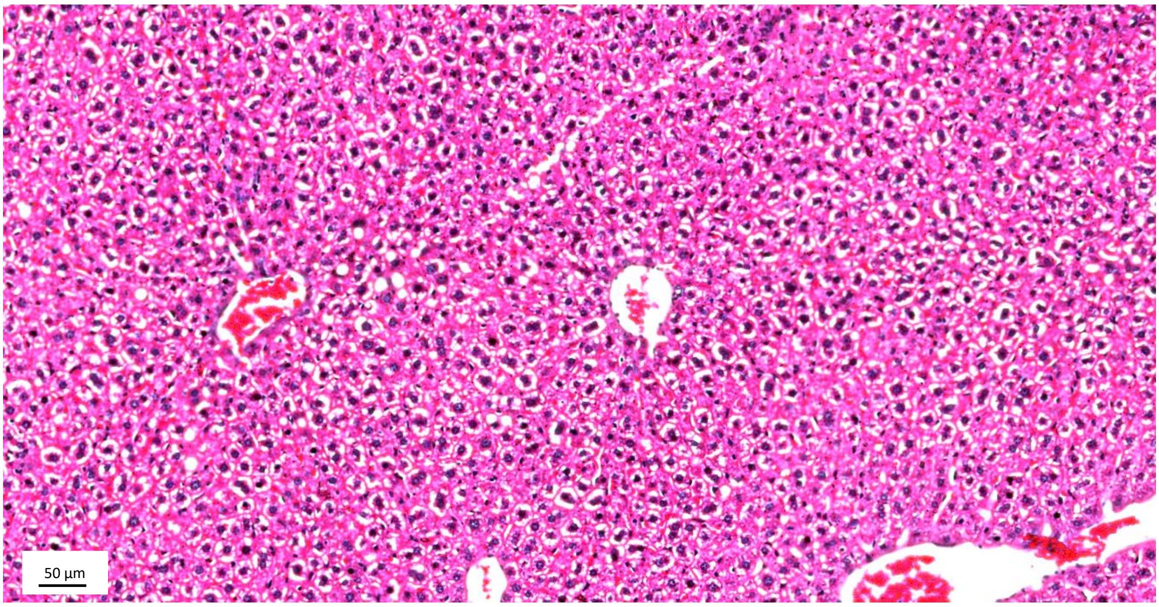

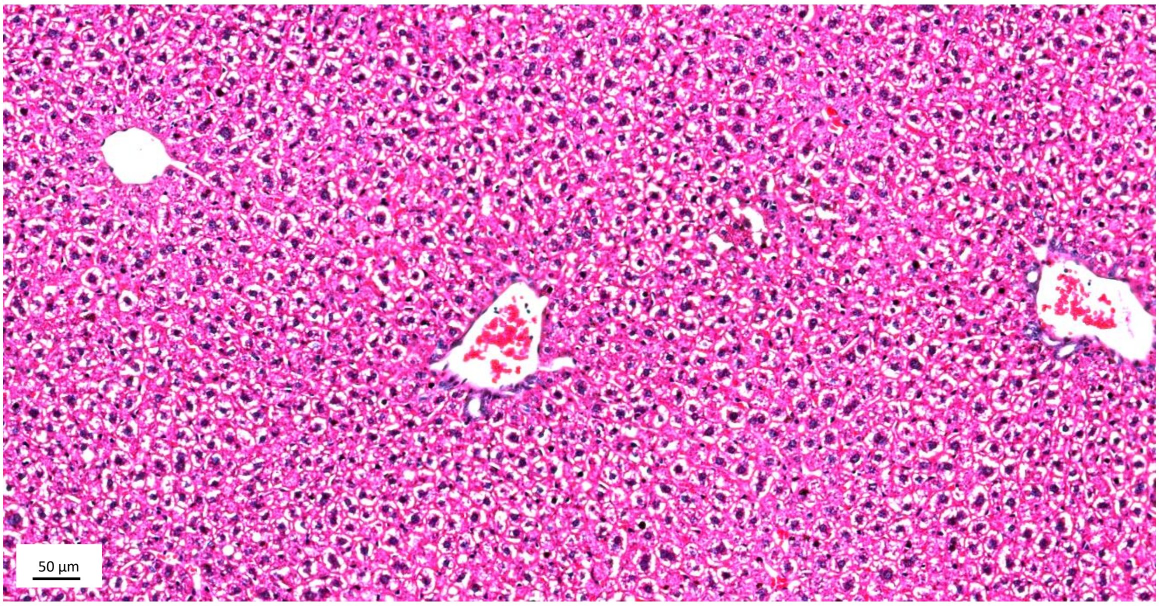

CDHFD + *P . distasonis* - 2

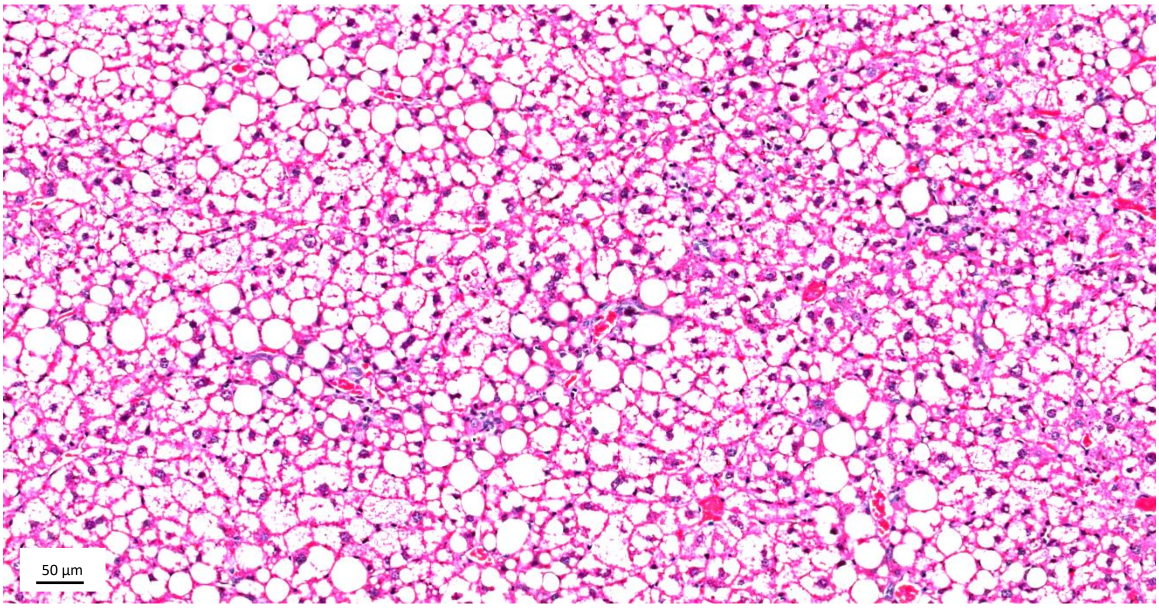

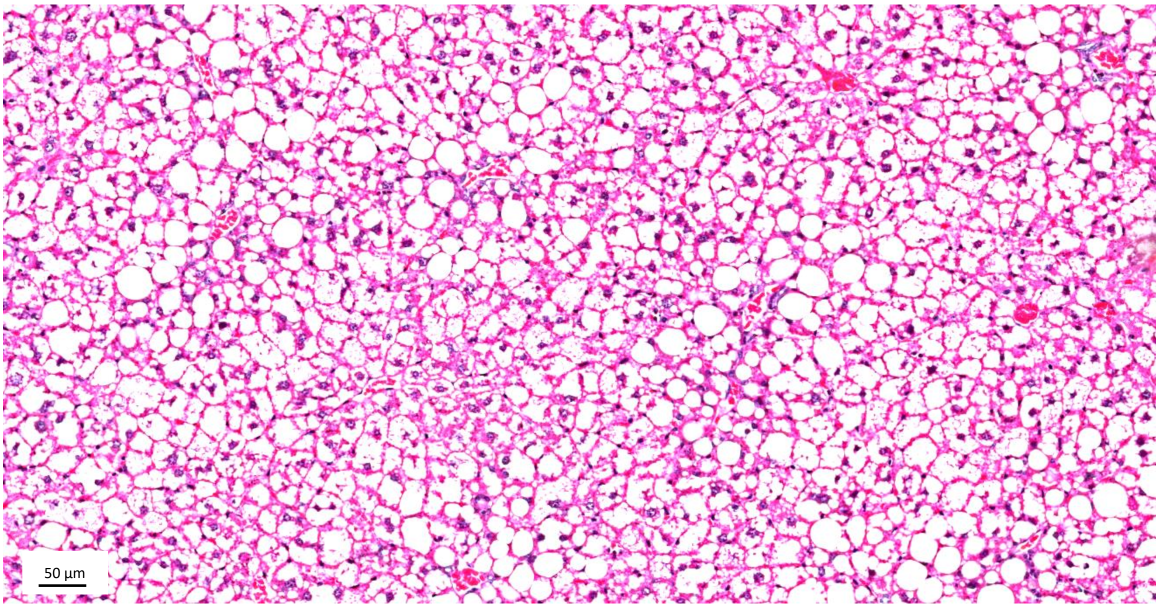

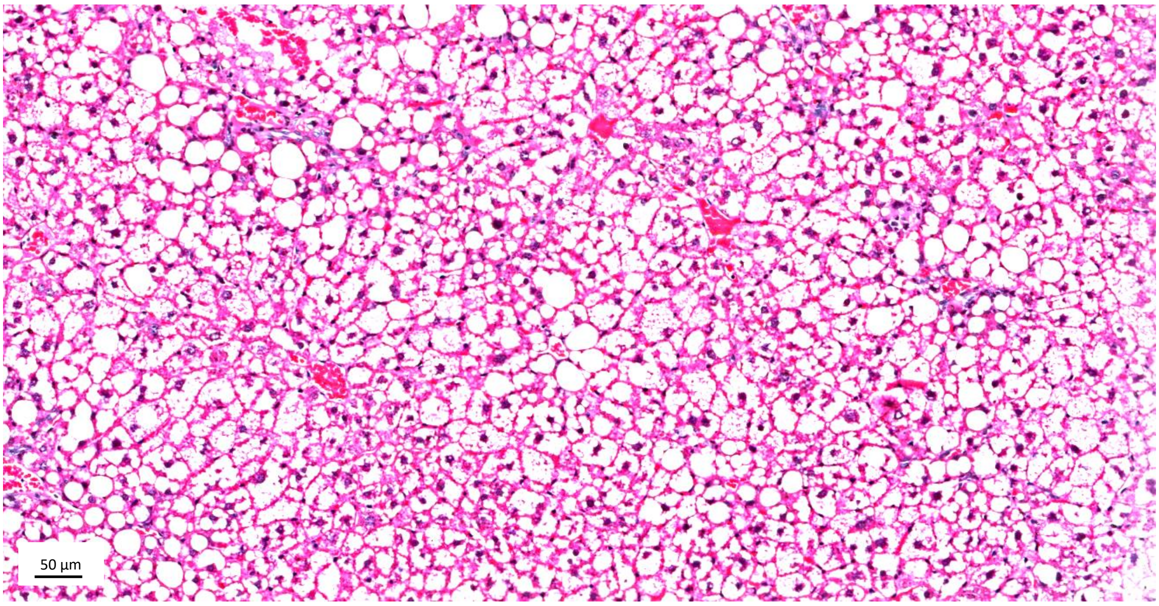

CDHFD + *P . distasonis* - 3

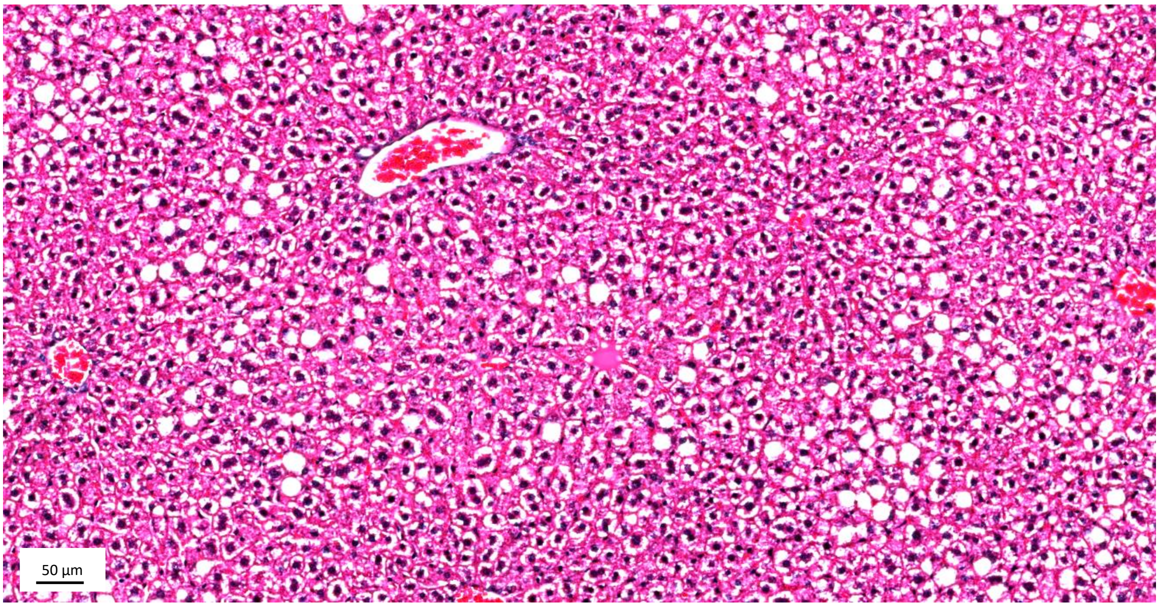

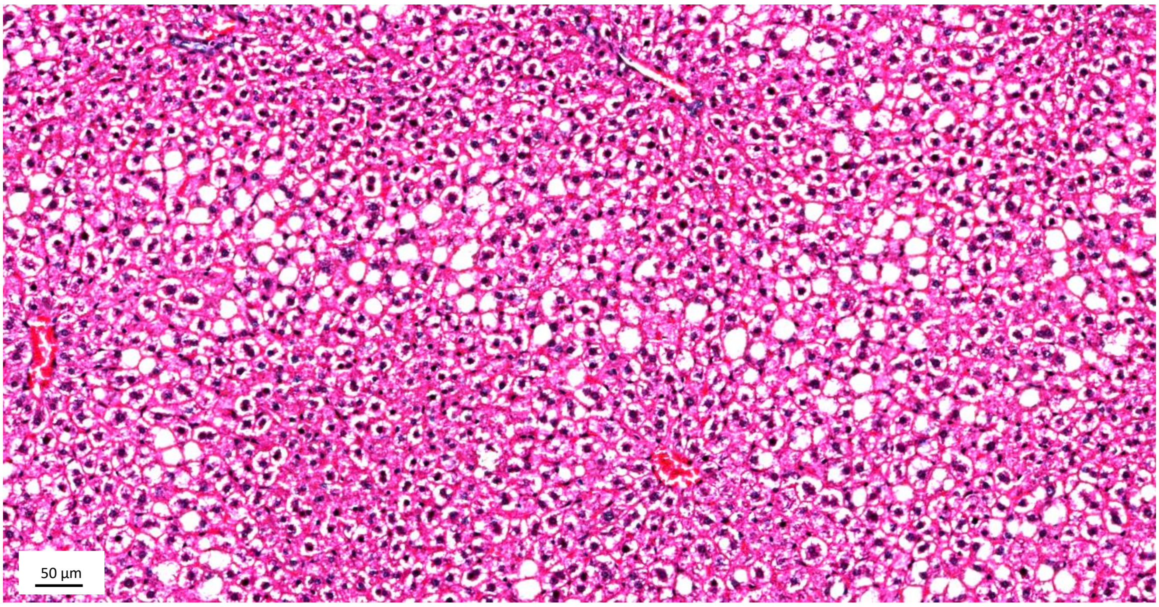

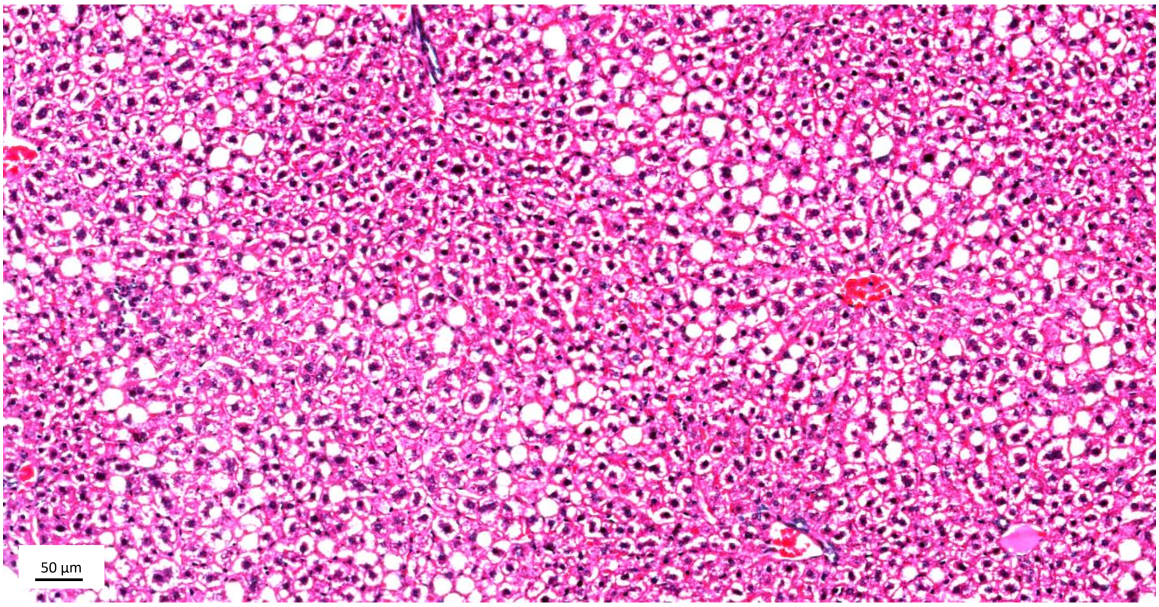

CDHFD + *P . distasonis* - 4

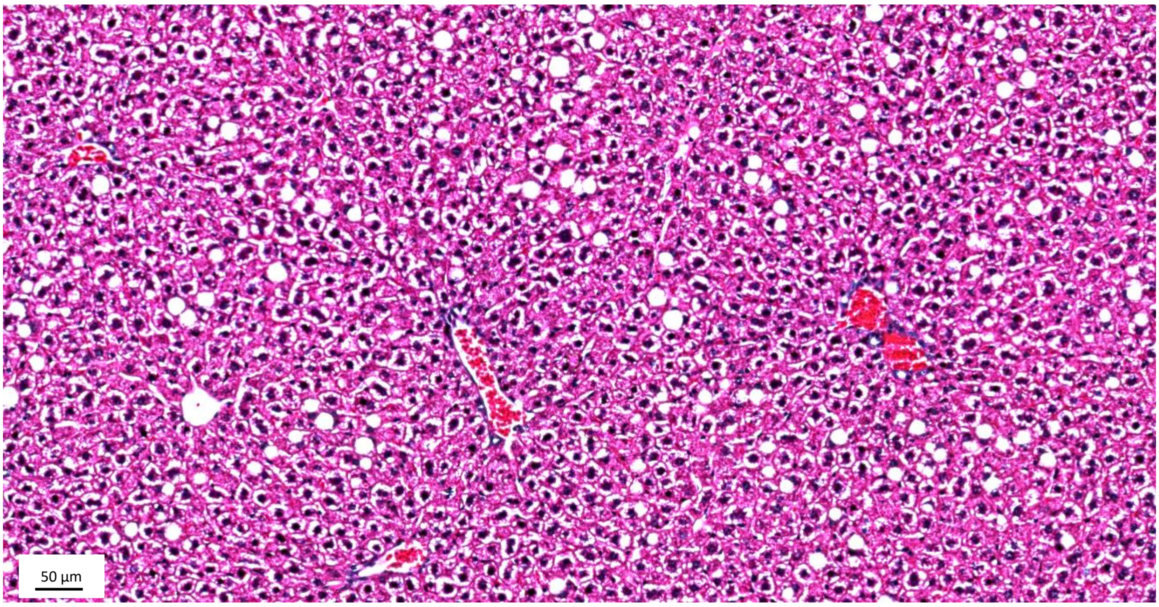

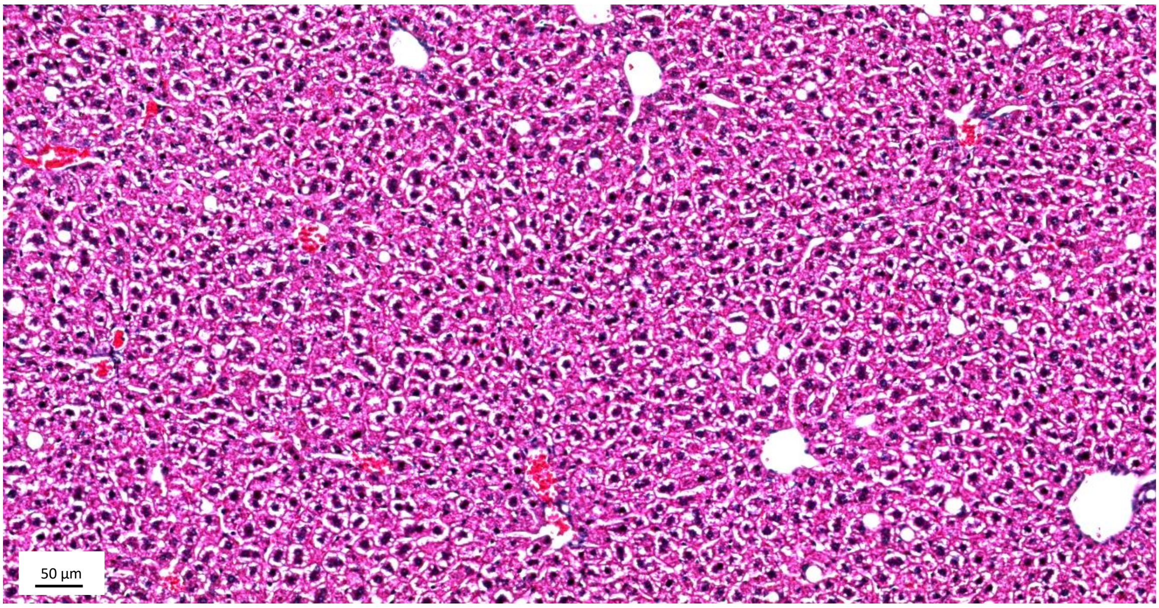

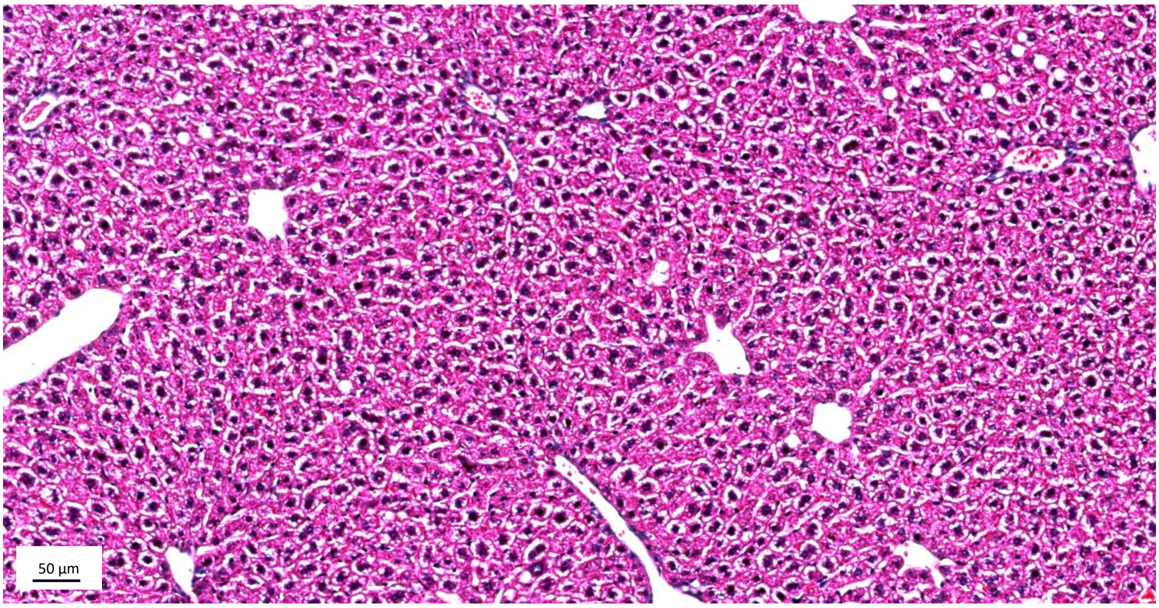

CDHFD + *P . distasonis* - 5

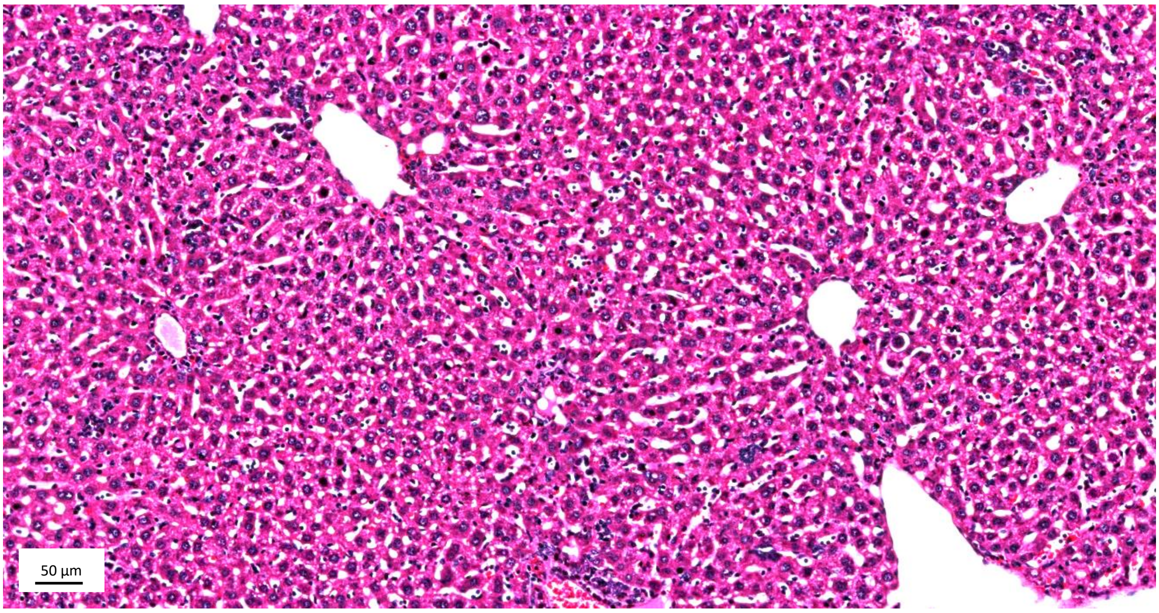

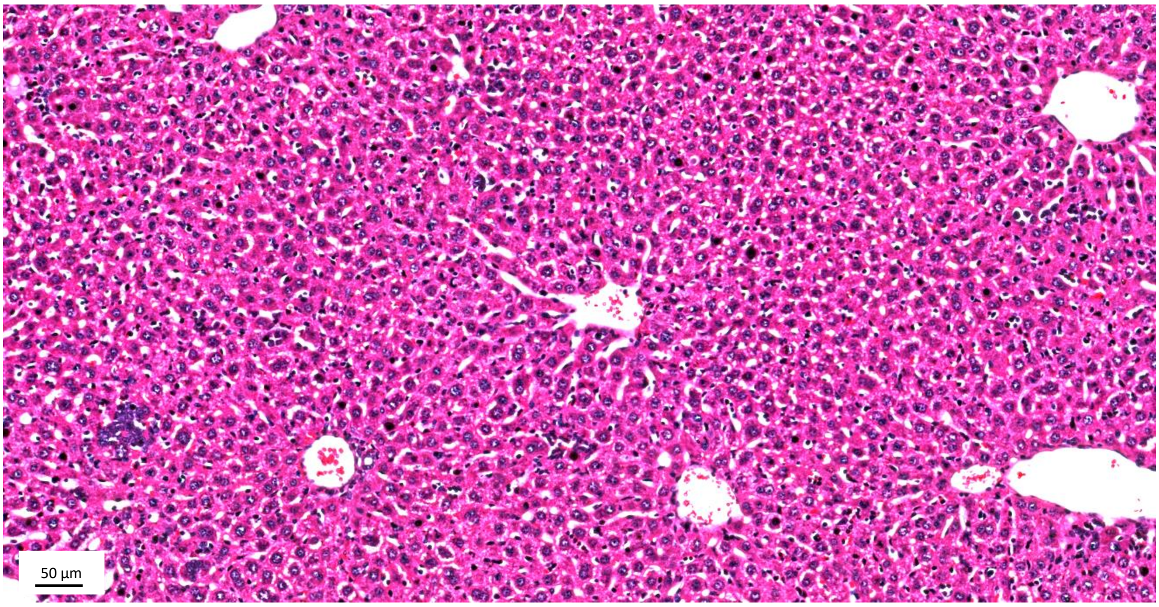

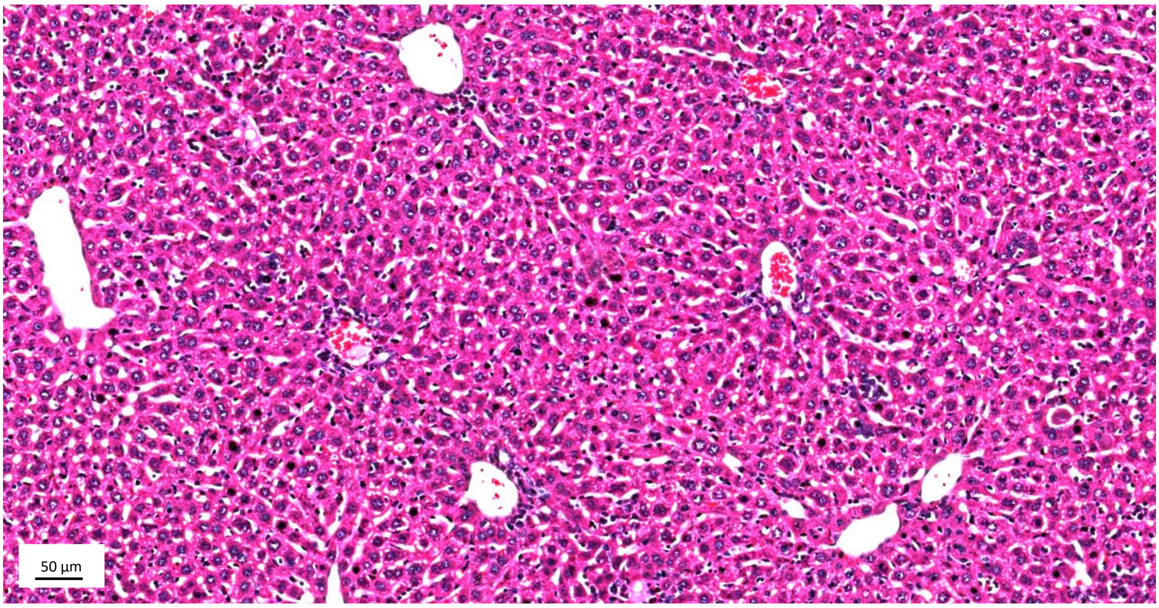

CDHFD + *P . distasonis* - 6

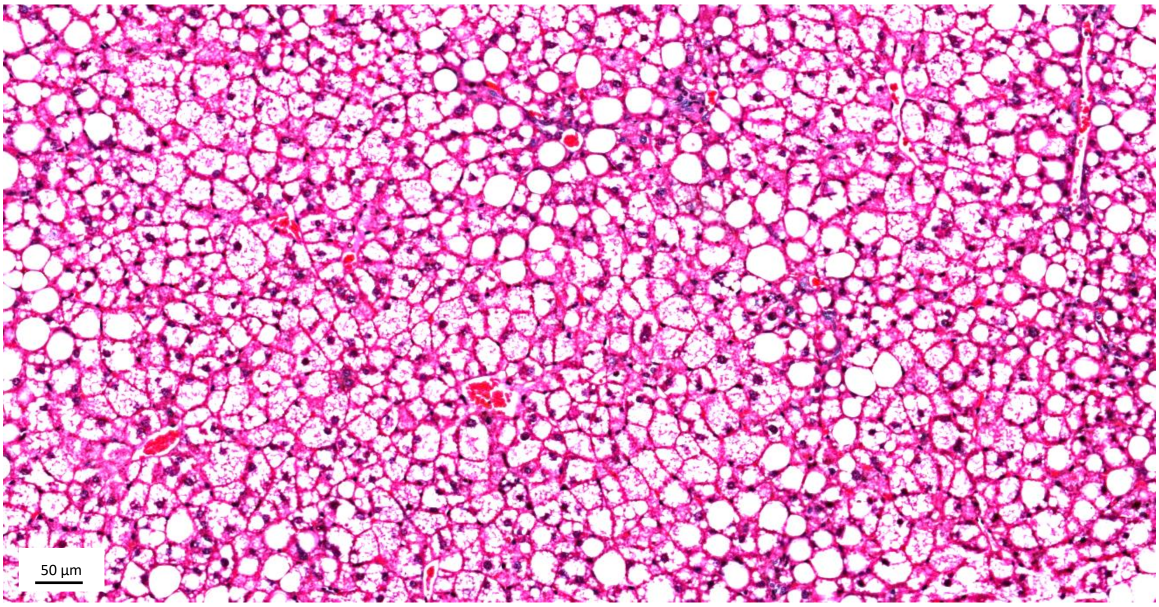

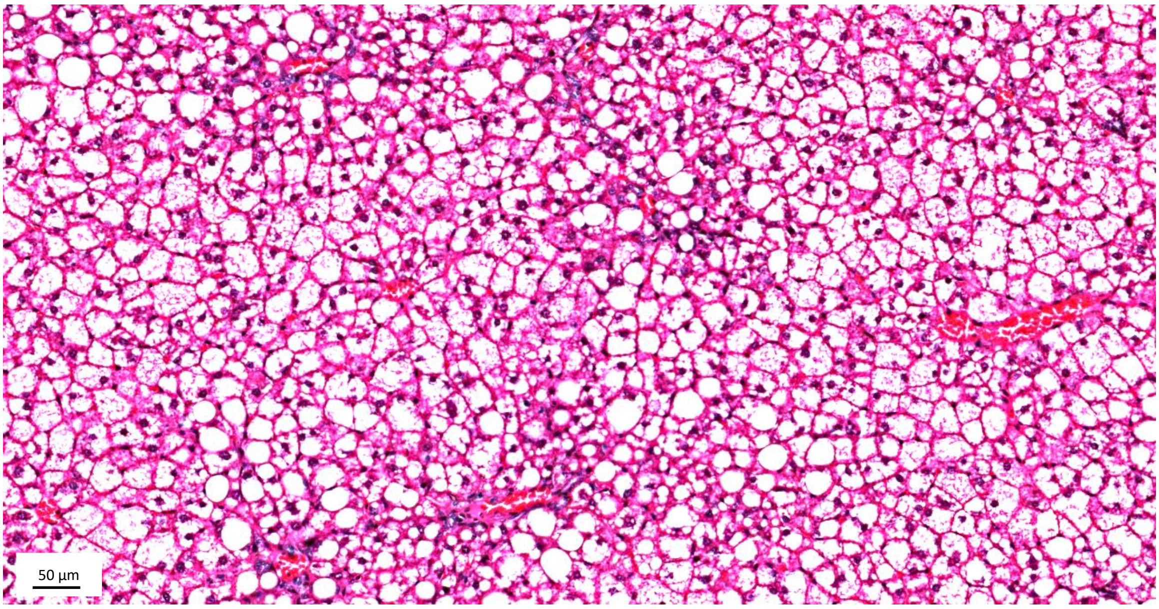

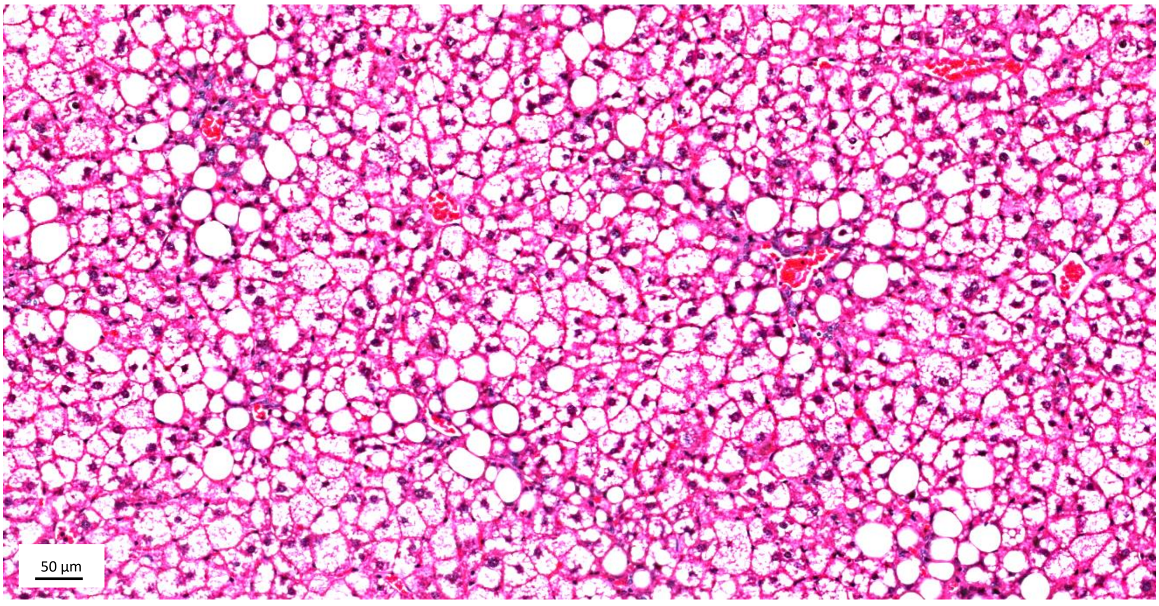

CDHFD + *P . distasonis* - 7

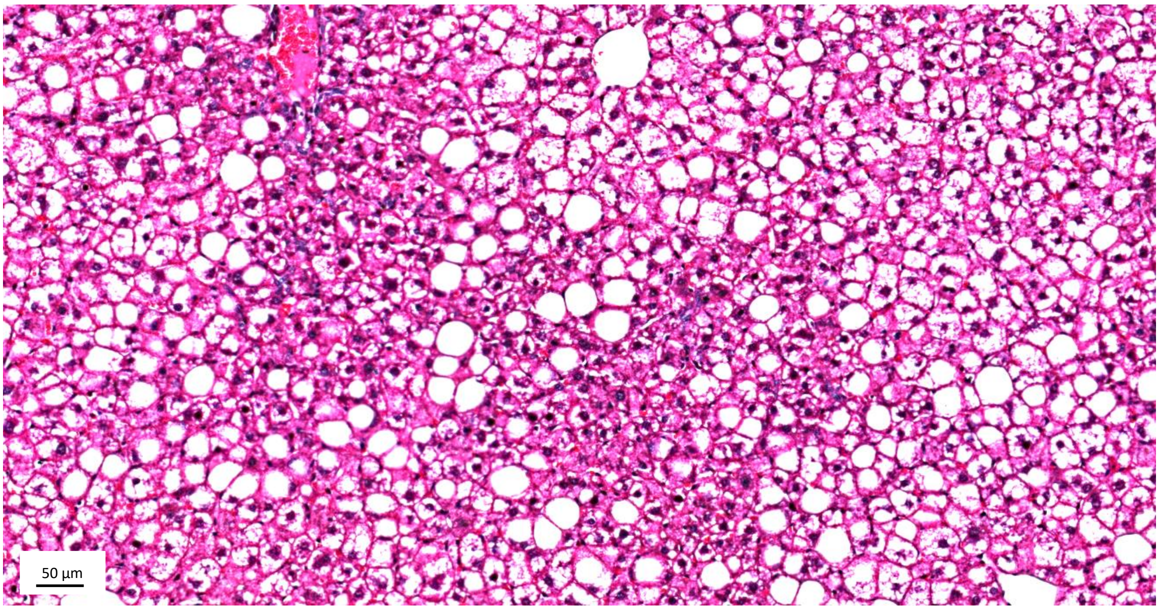

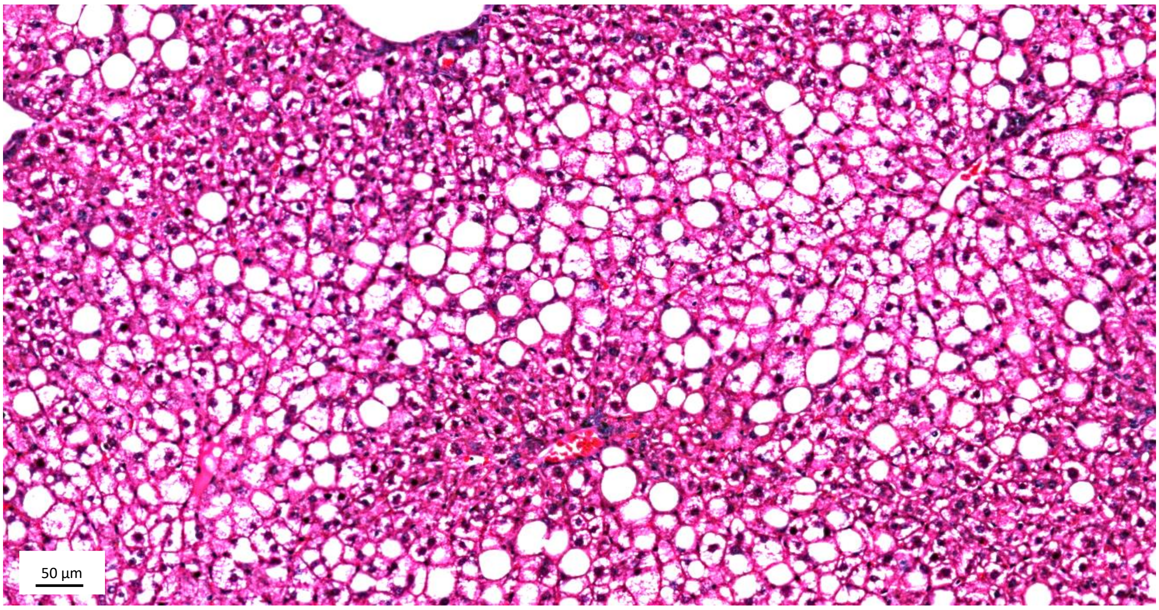

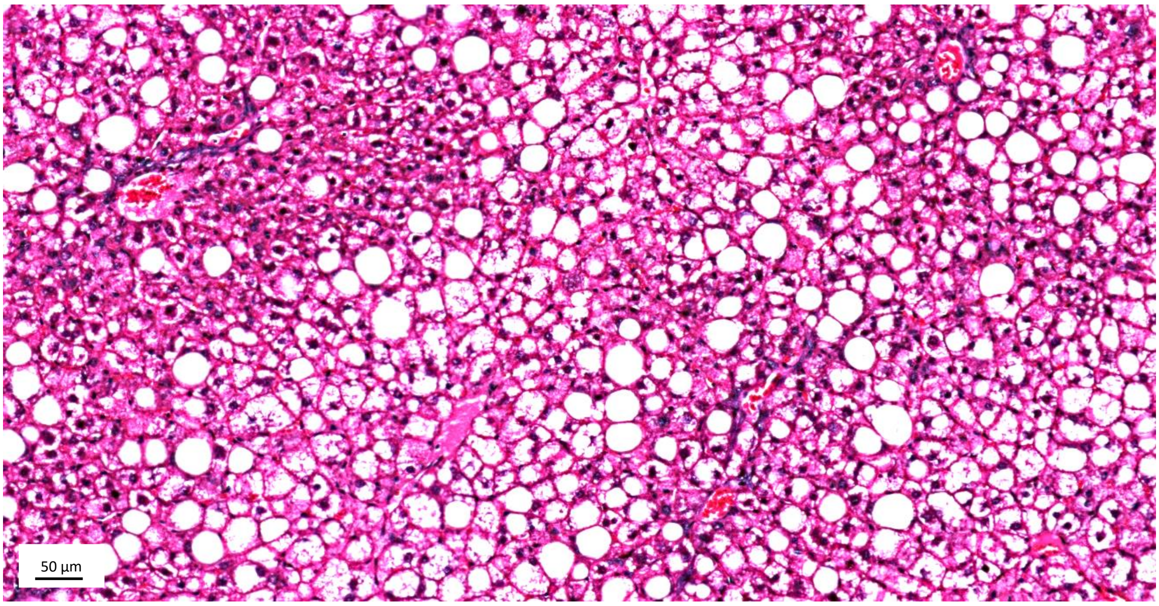

CDHFD + *P . distasonis* - 8

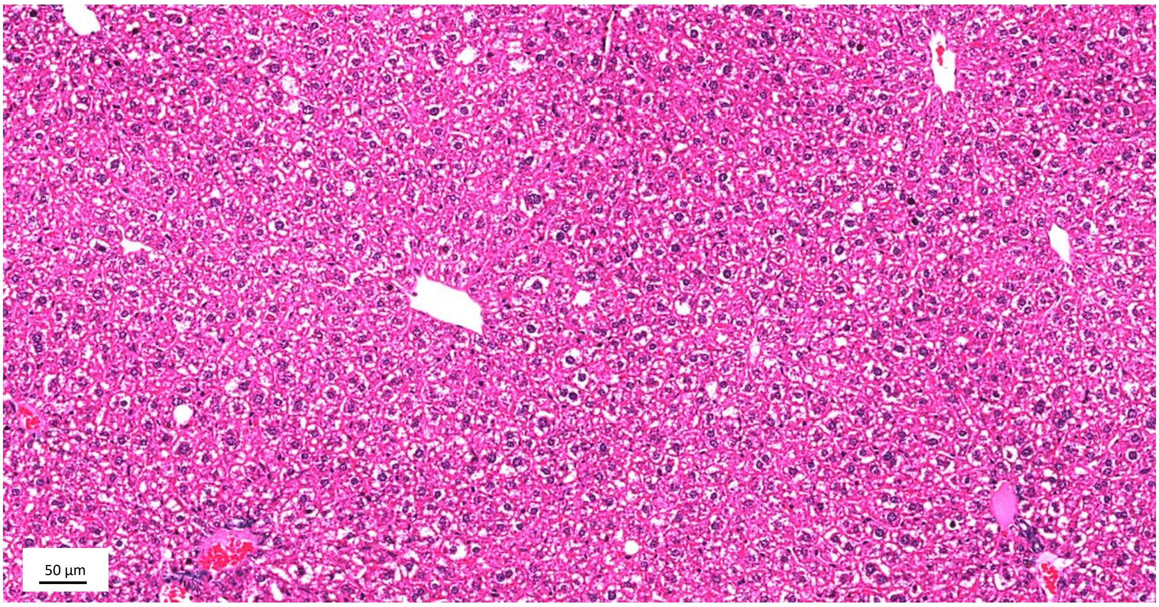

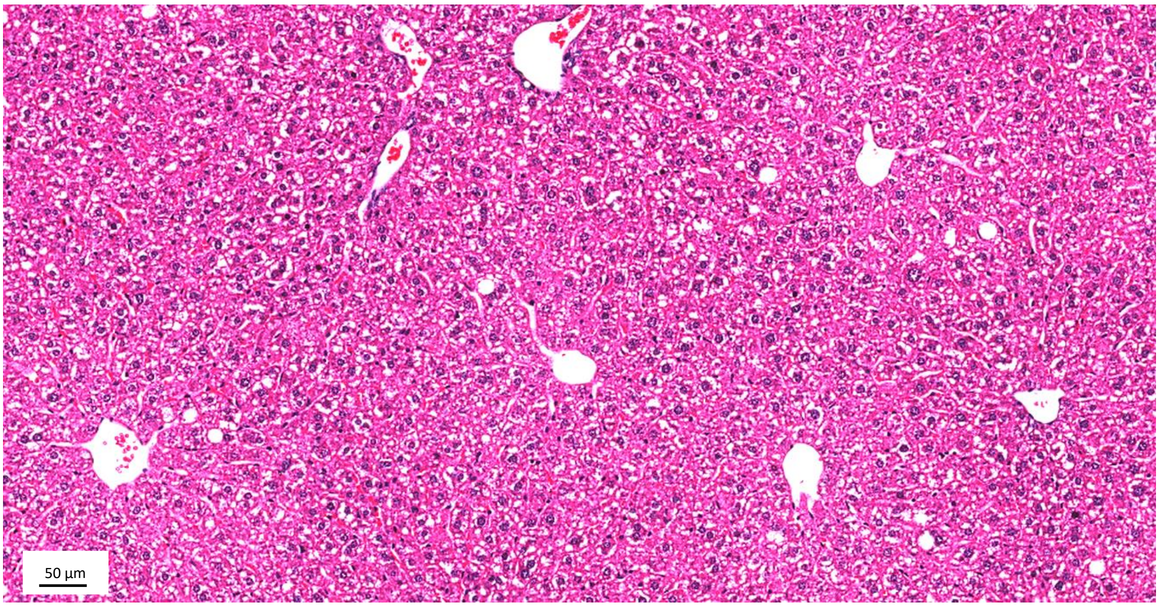

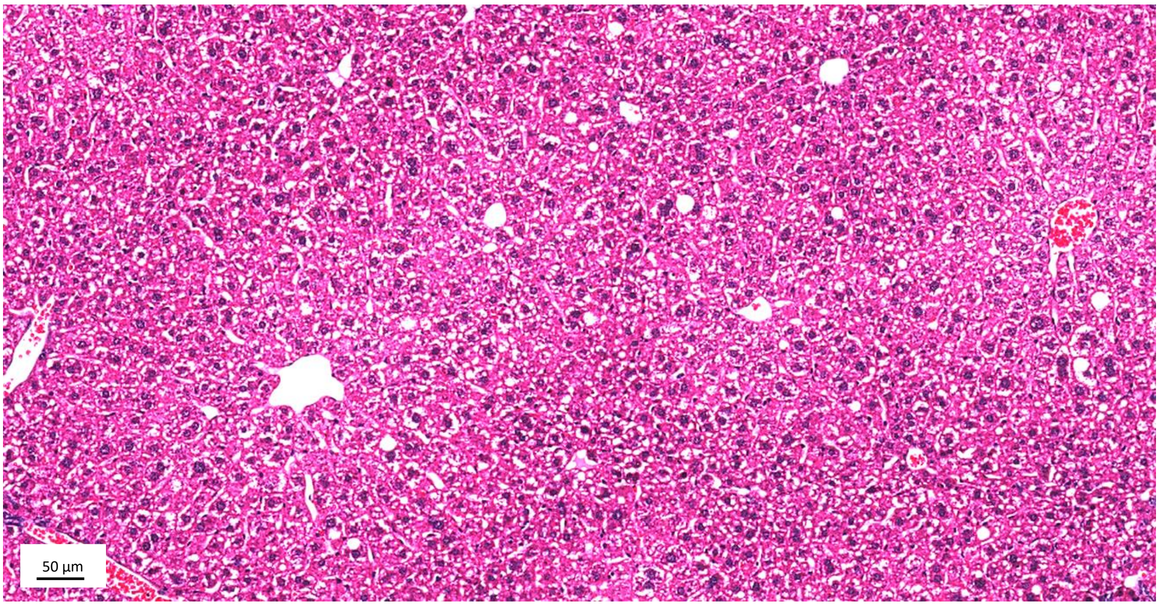

CDHFD + *P . distasonis* - 9

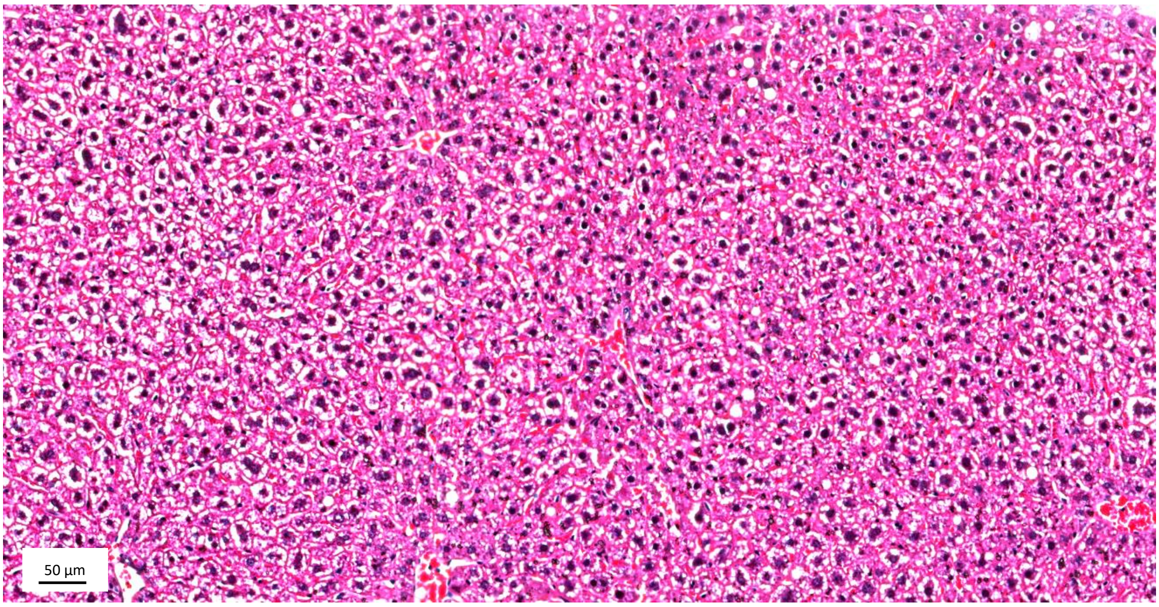

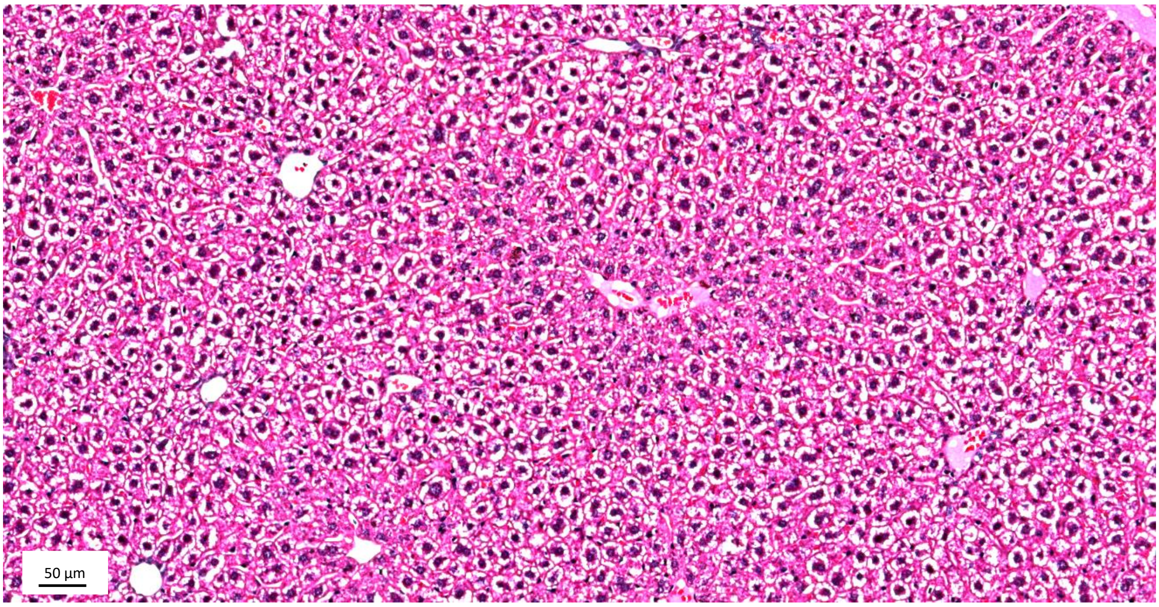

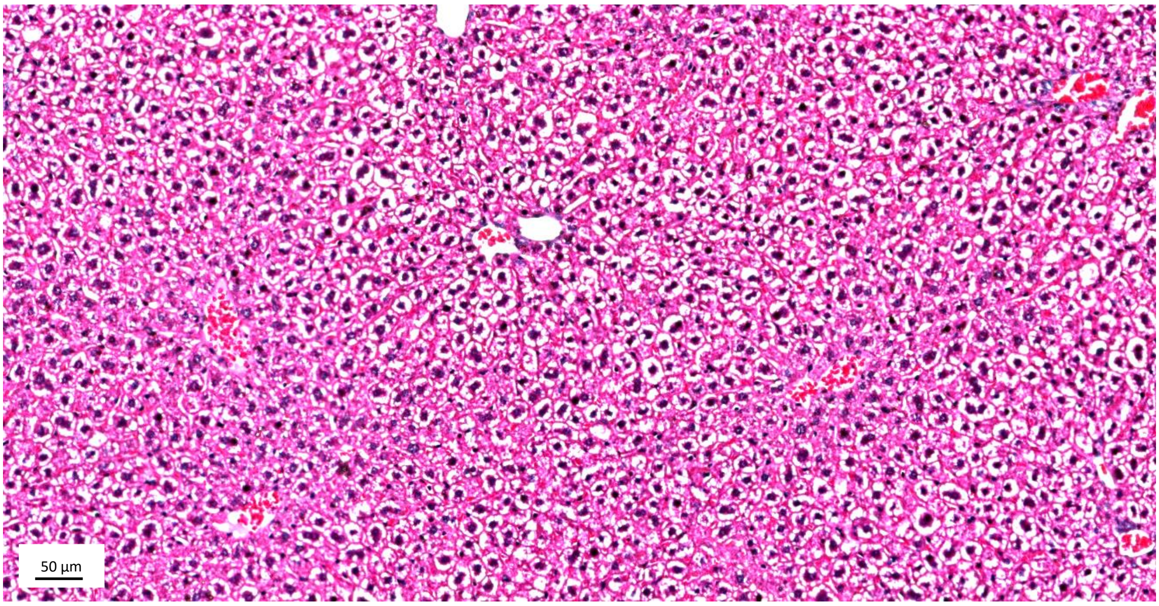

CDHFD + *P . distasonis* - 10

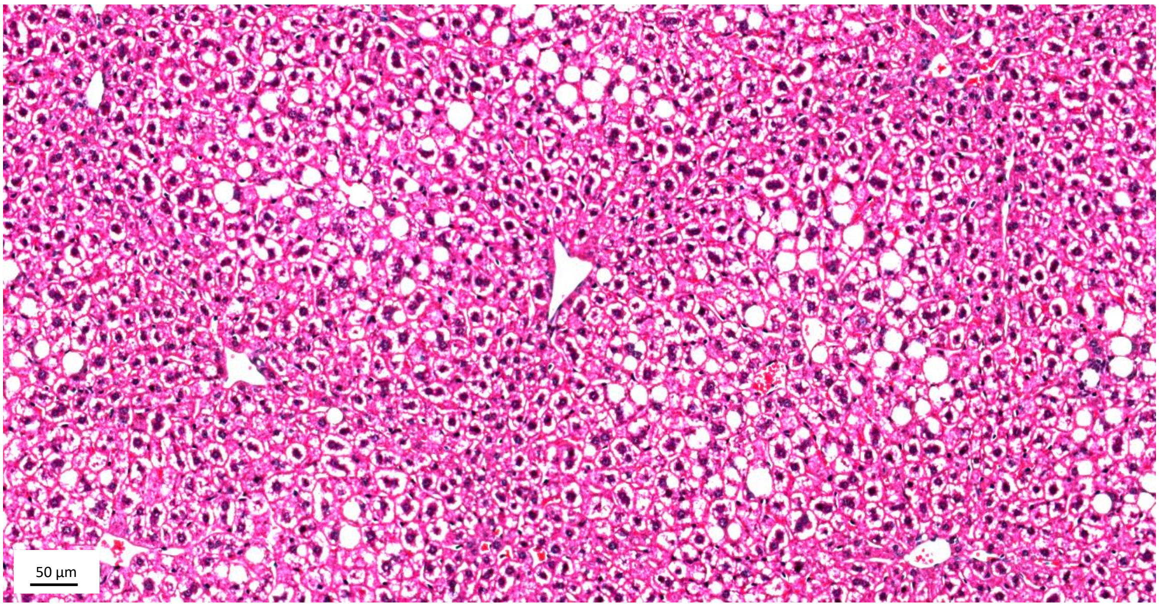

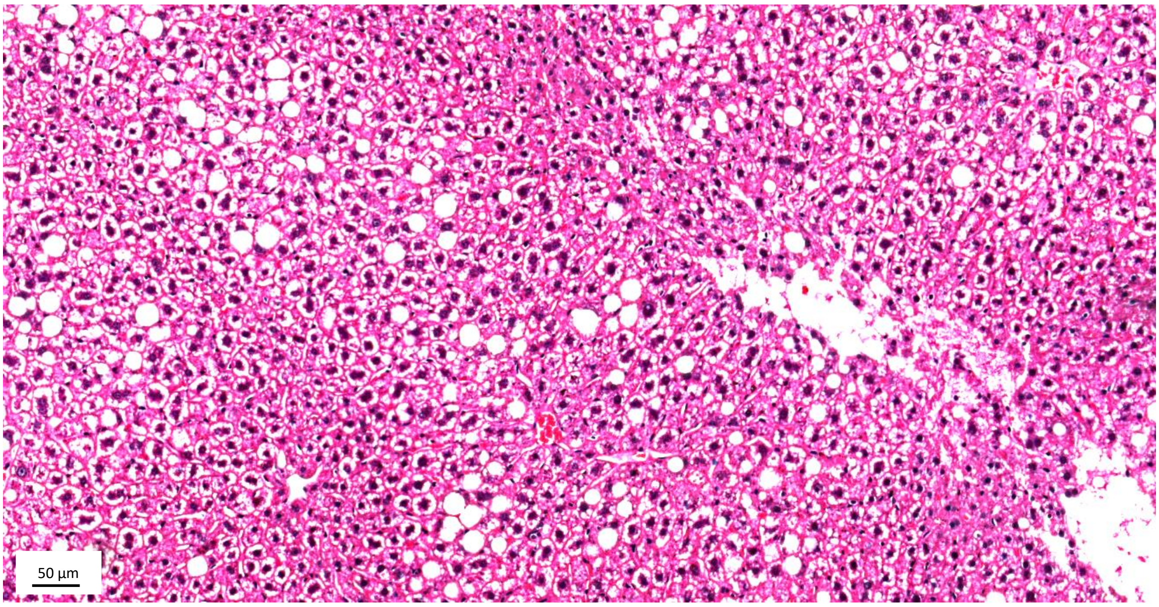

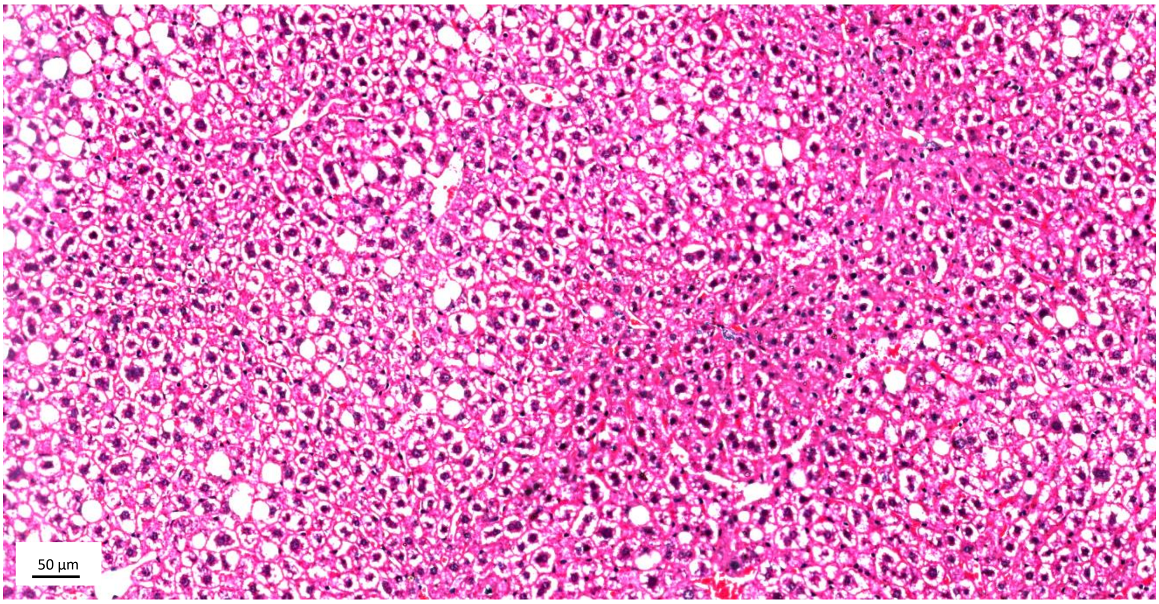

CDHFD + *P . distasonis* - 11

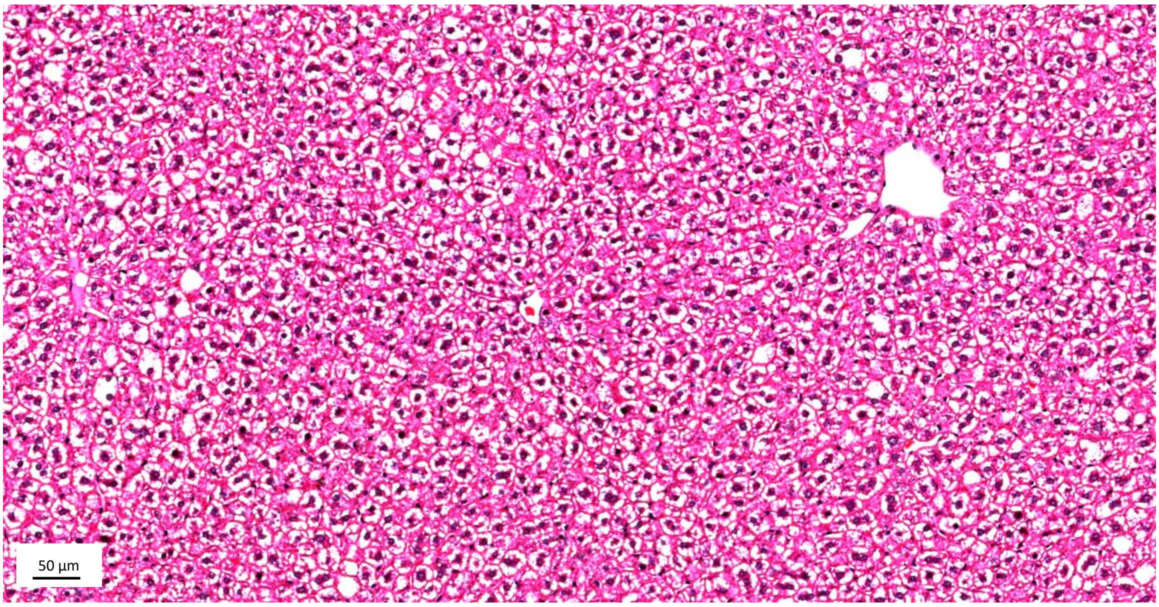

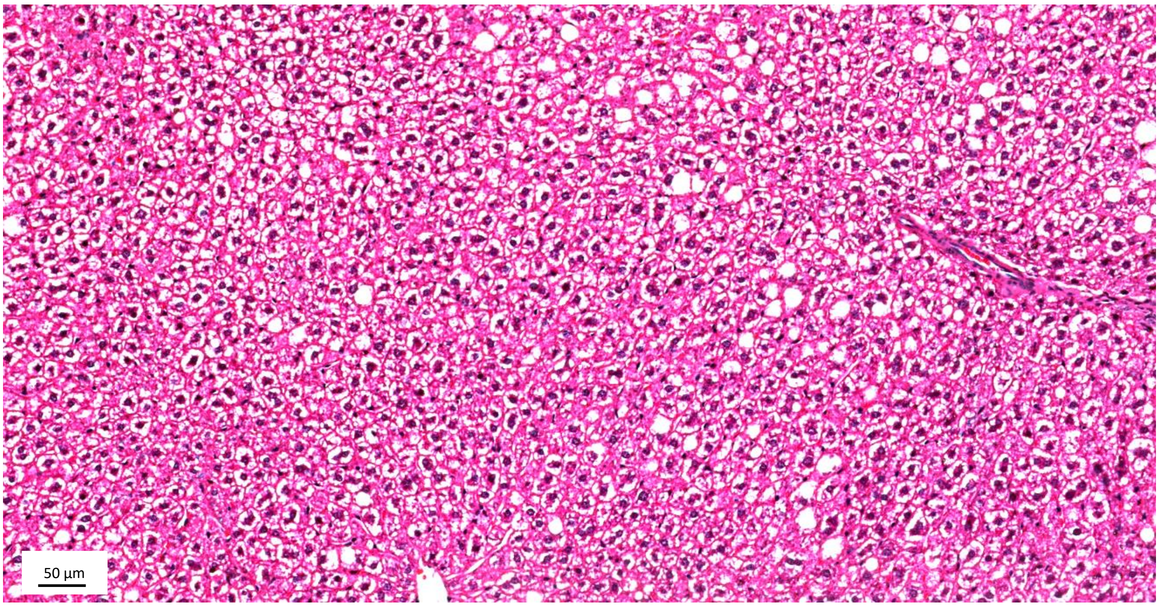

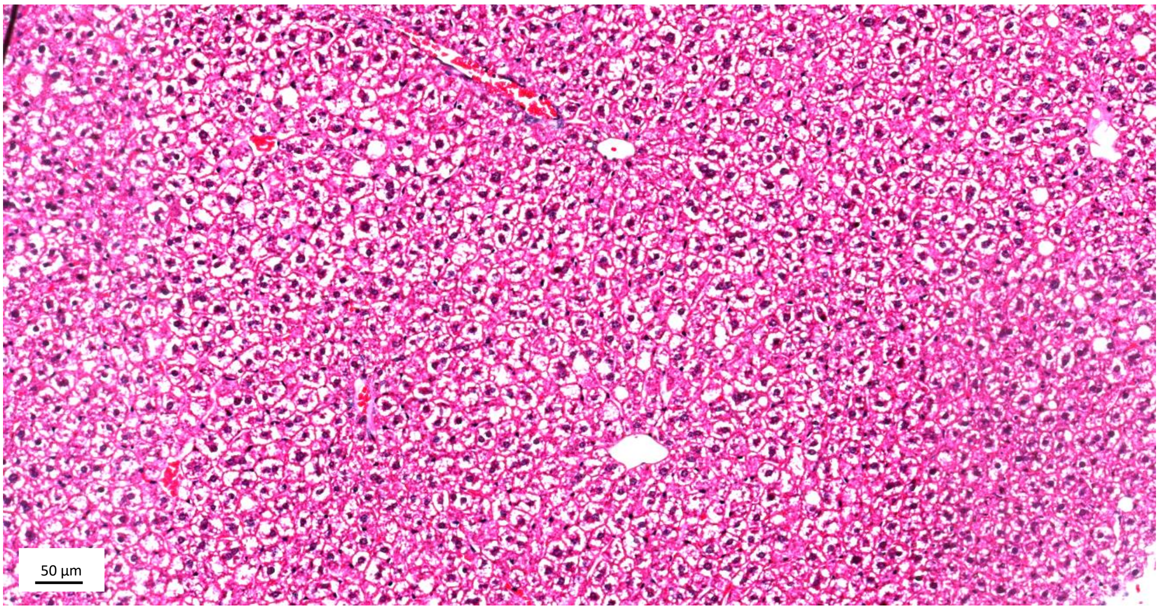

CDHFD + *P . distasonis* - 12

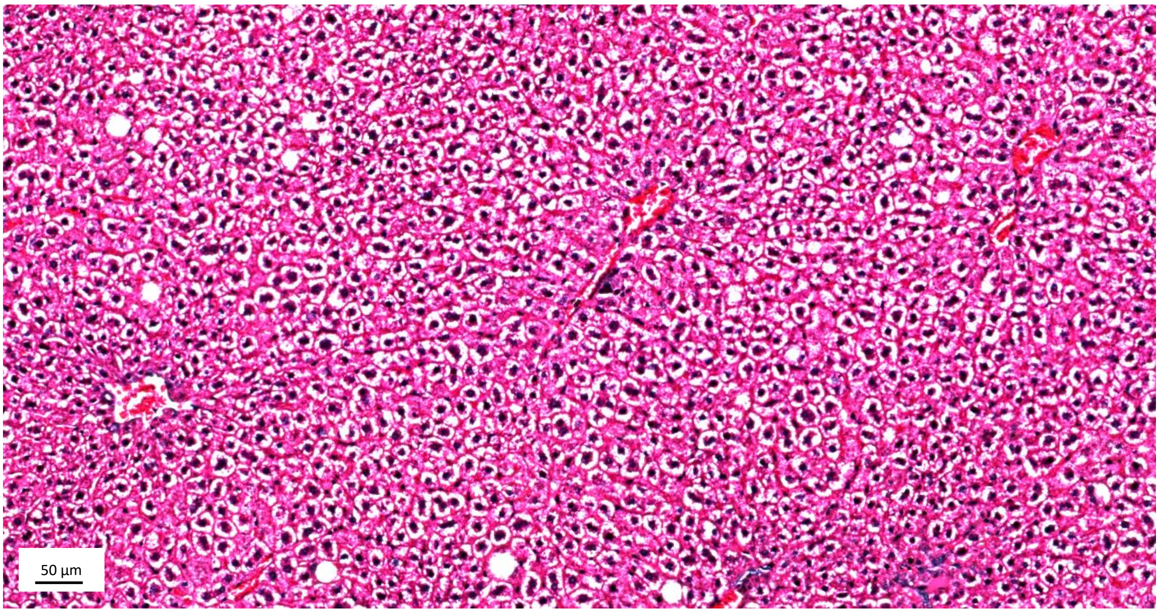

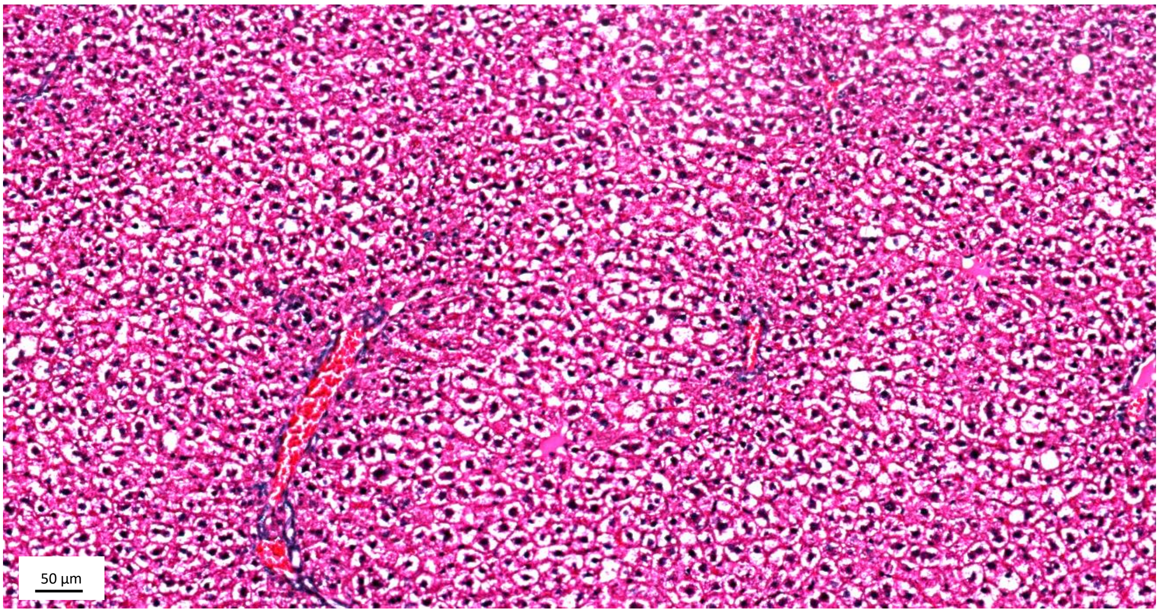

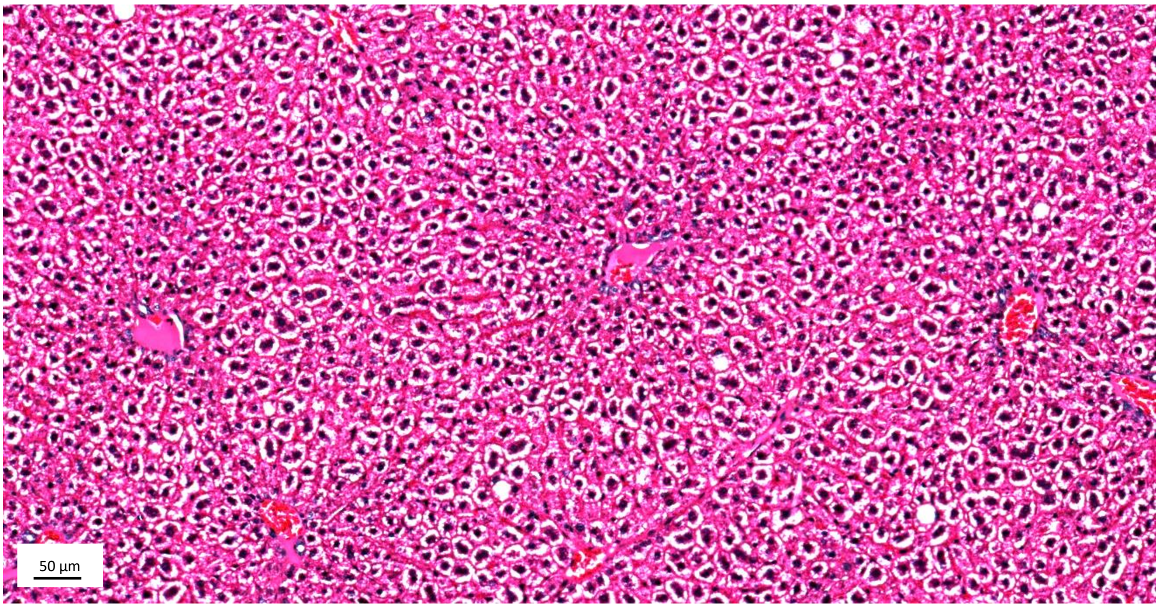

Supplement: Source Data Fig. 3 — Replicate histological images. [file 41564_2023_1418_MOESM11_ESM.pdf]
