# Supplementary figures and images for: Parabacteroides distasonis uses dietary inulin to suppress NASH via its metabolite pentadecanoic acid
Source: Nat Microbiol. 2023 Jun 29;8(8):1534–48. doi: 10.1038/s41564-023-01418-7 (PMC10390331; doi:10.1038/s41564-023-01418-7)

Figure 5d

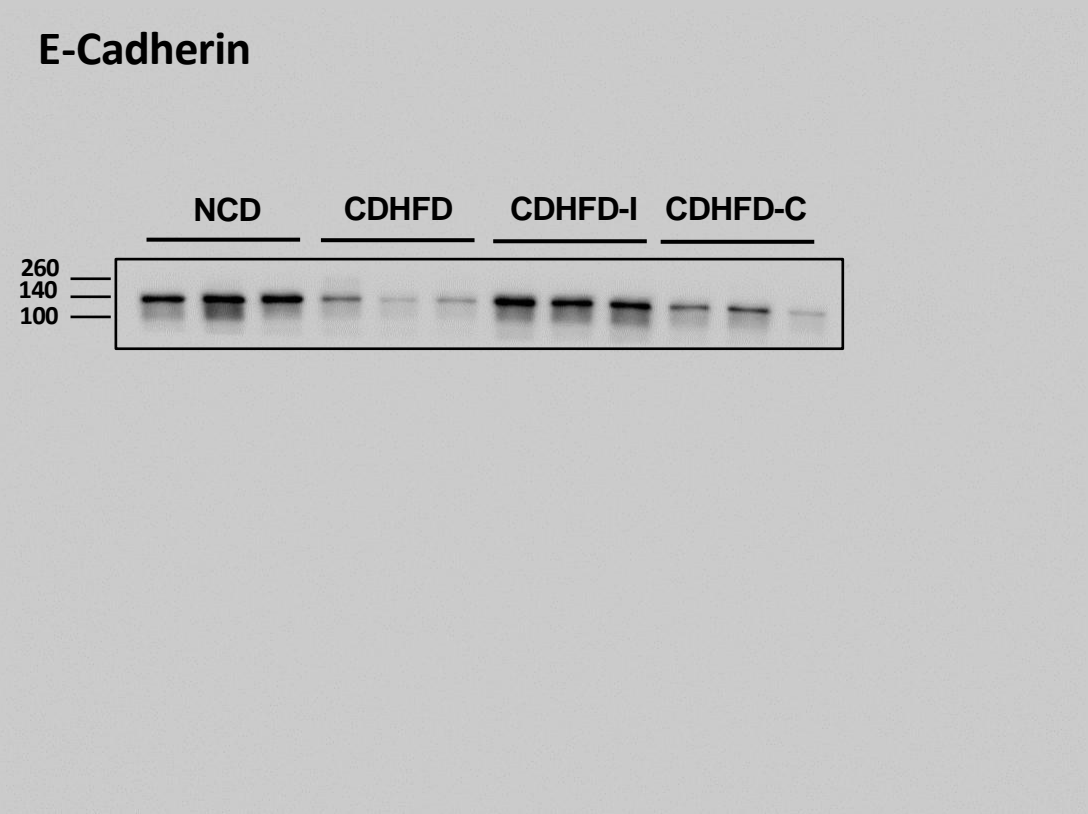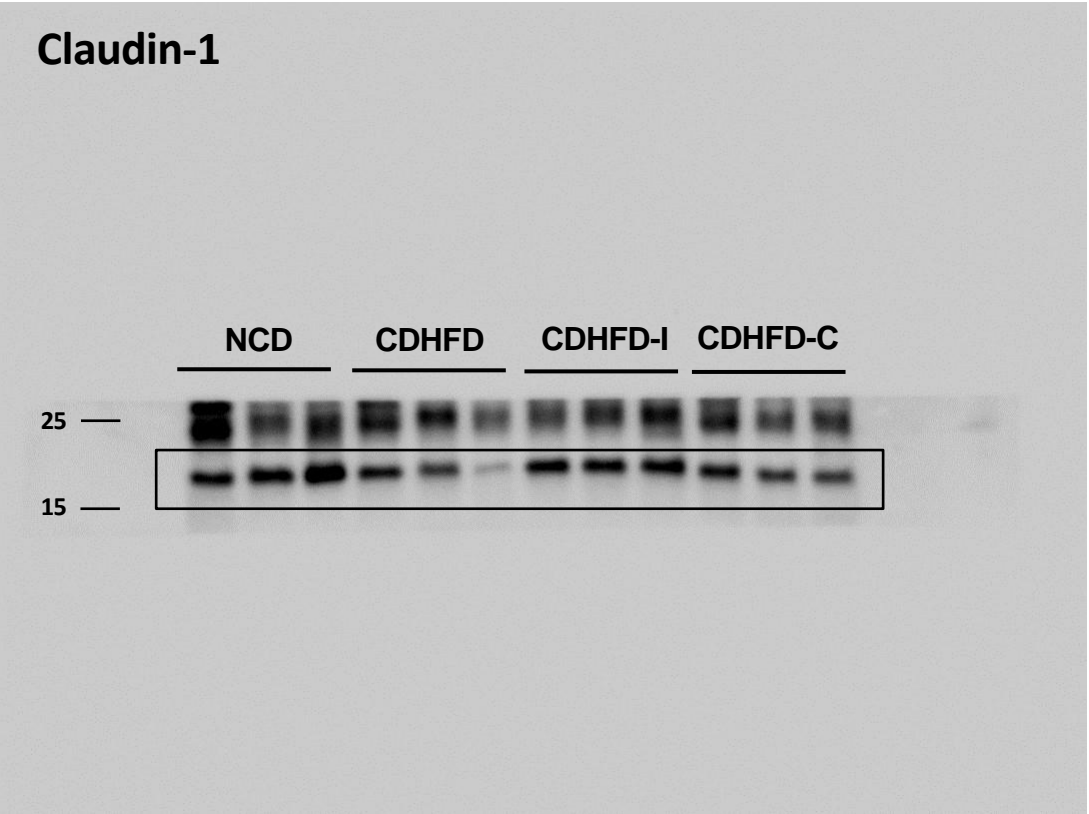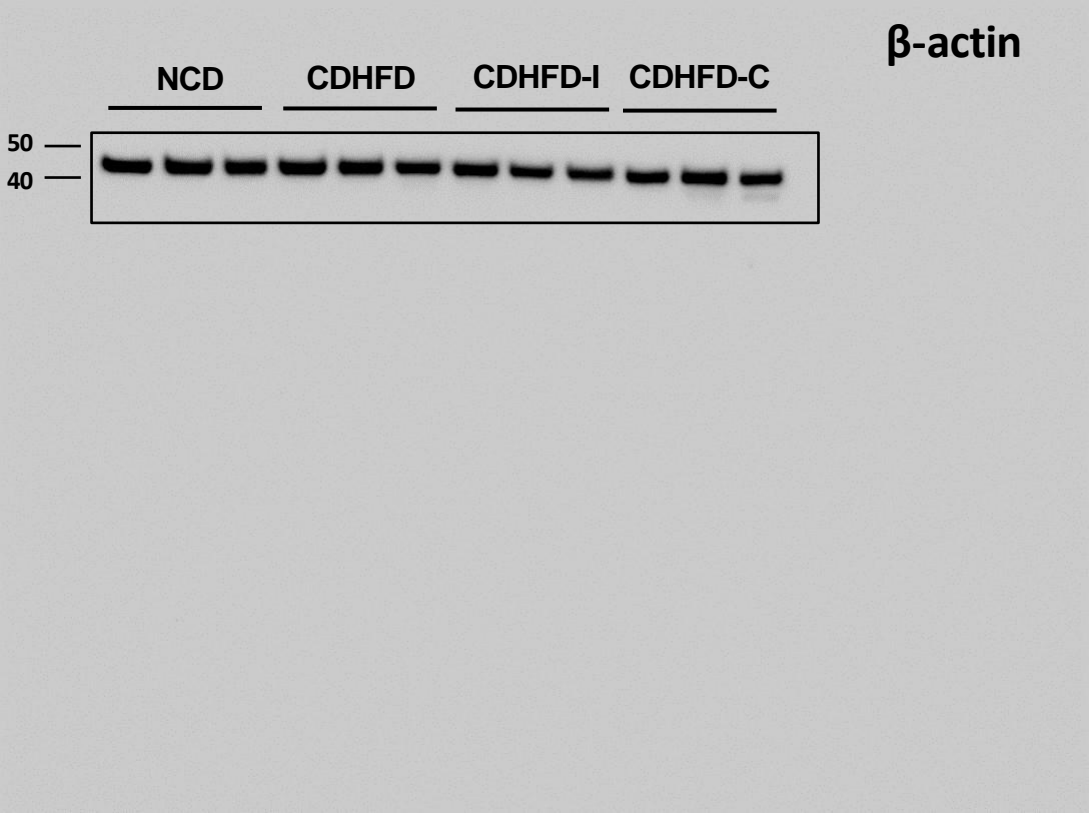

Figure 5d

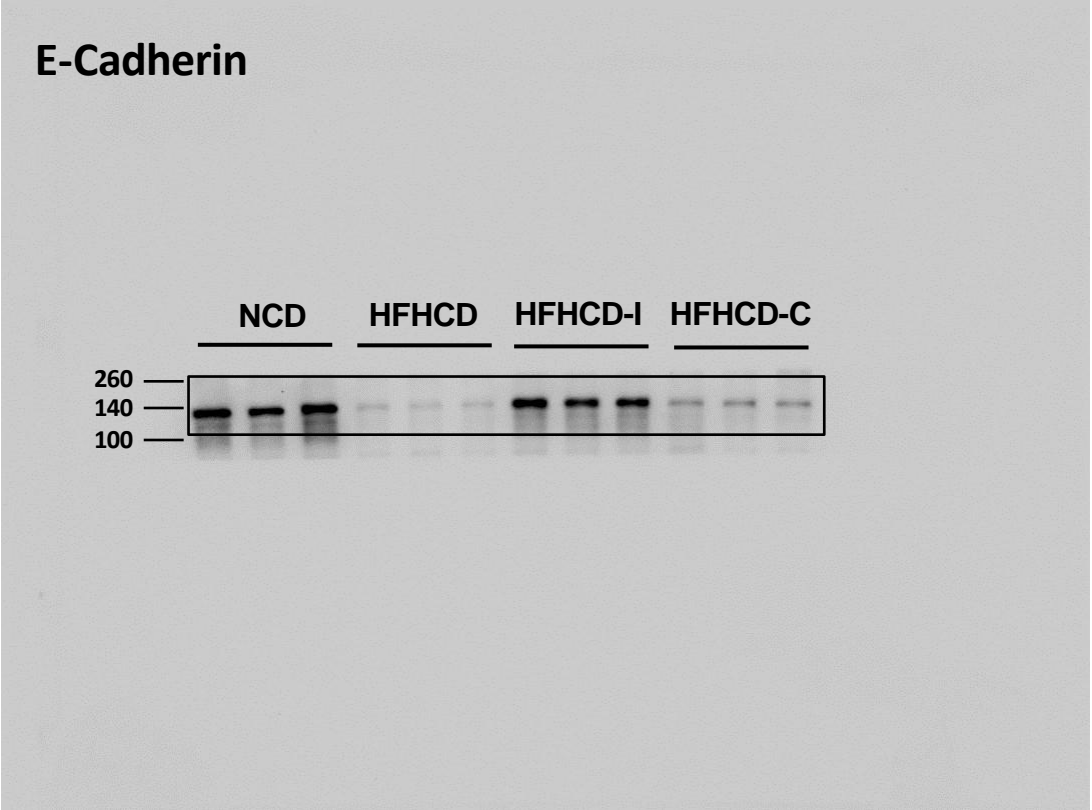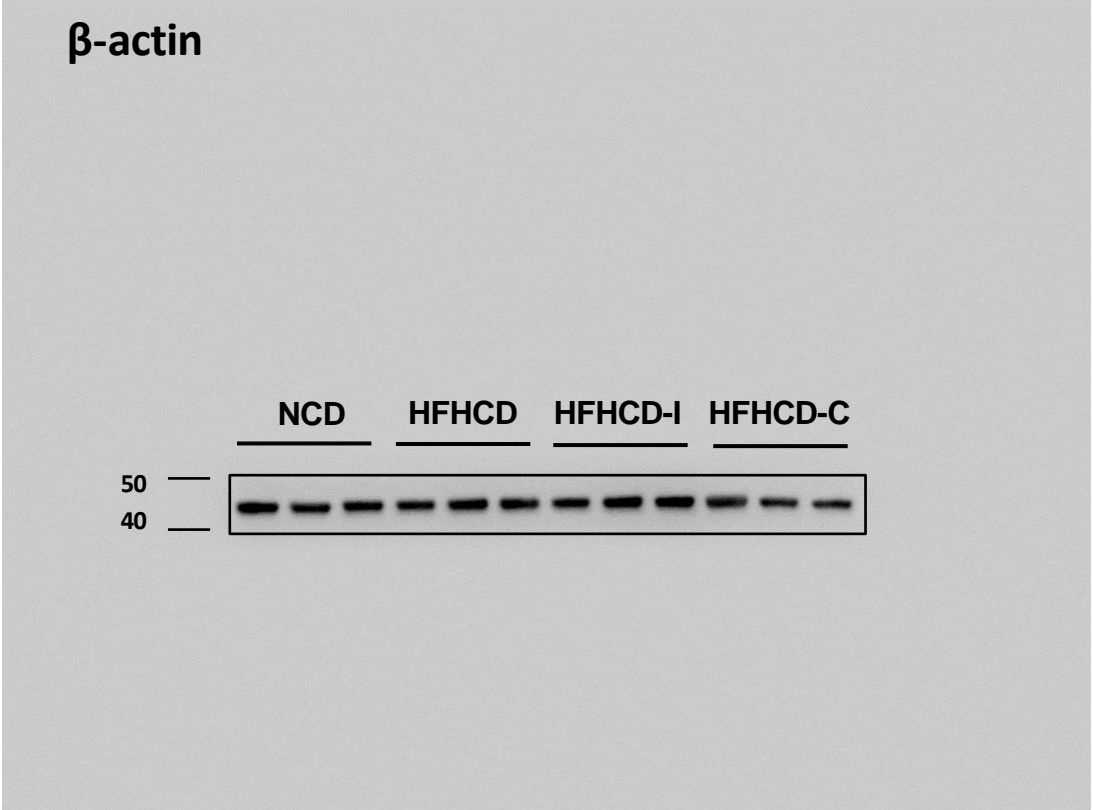

Figure 5d

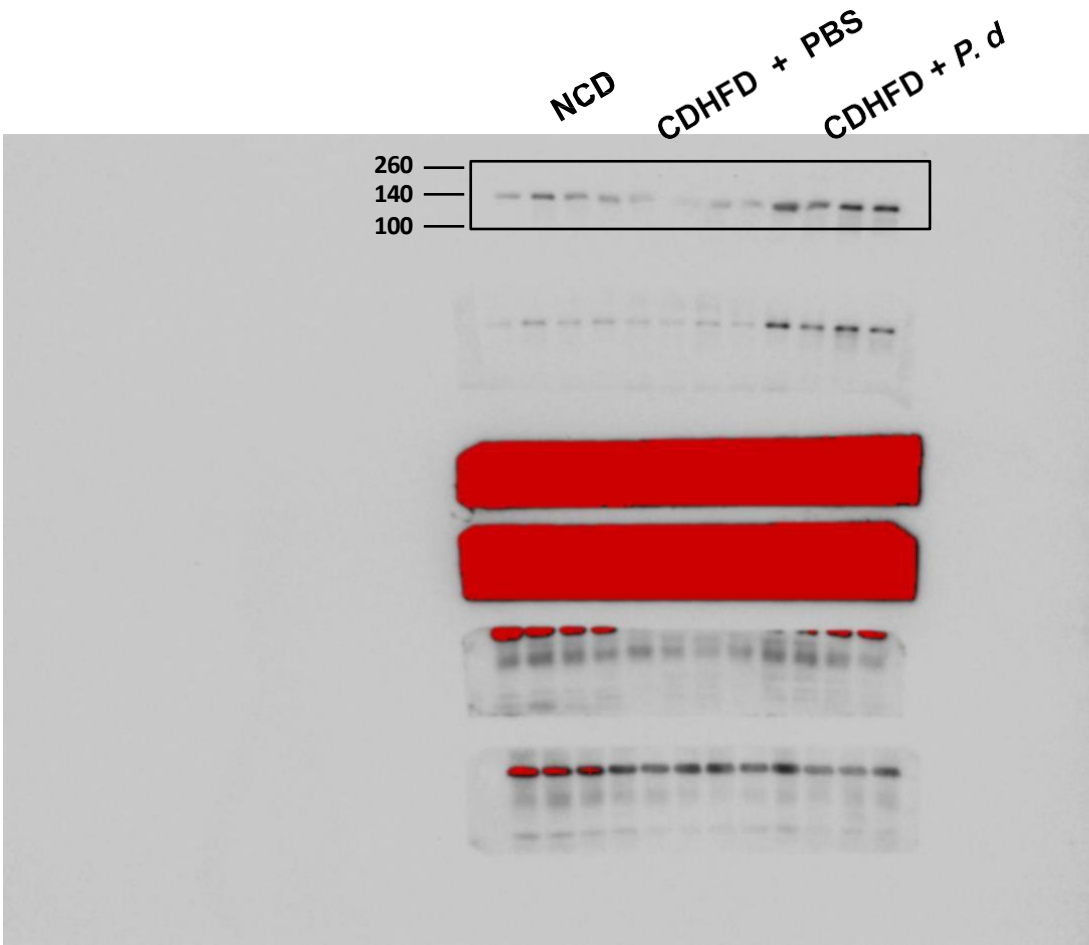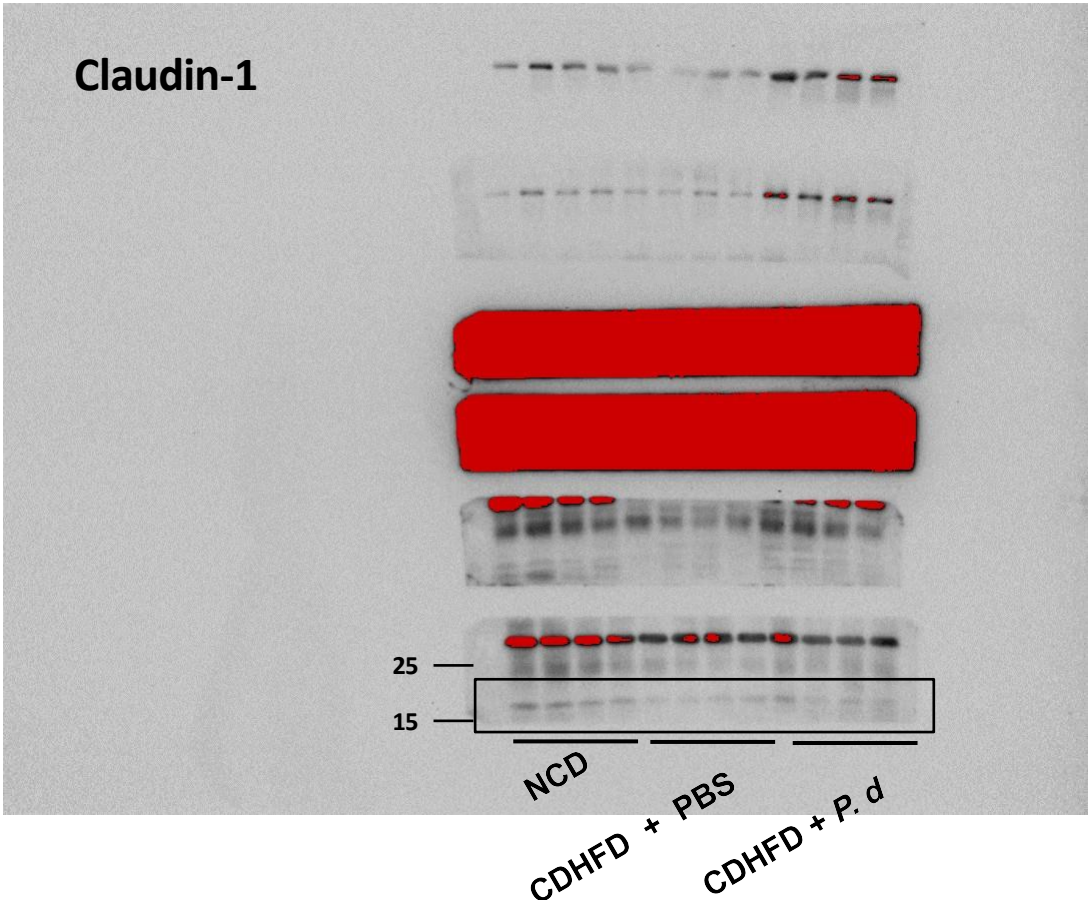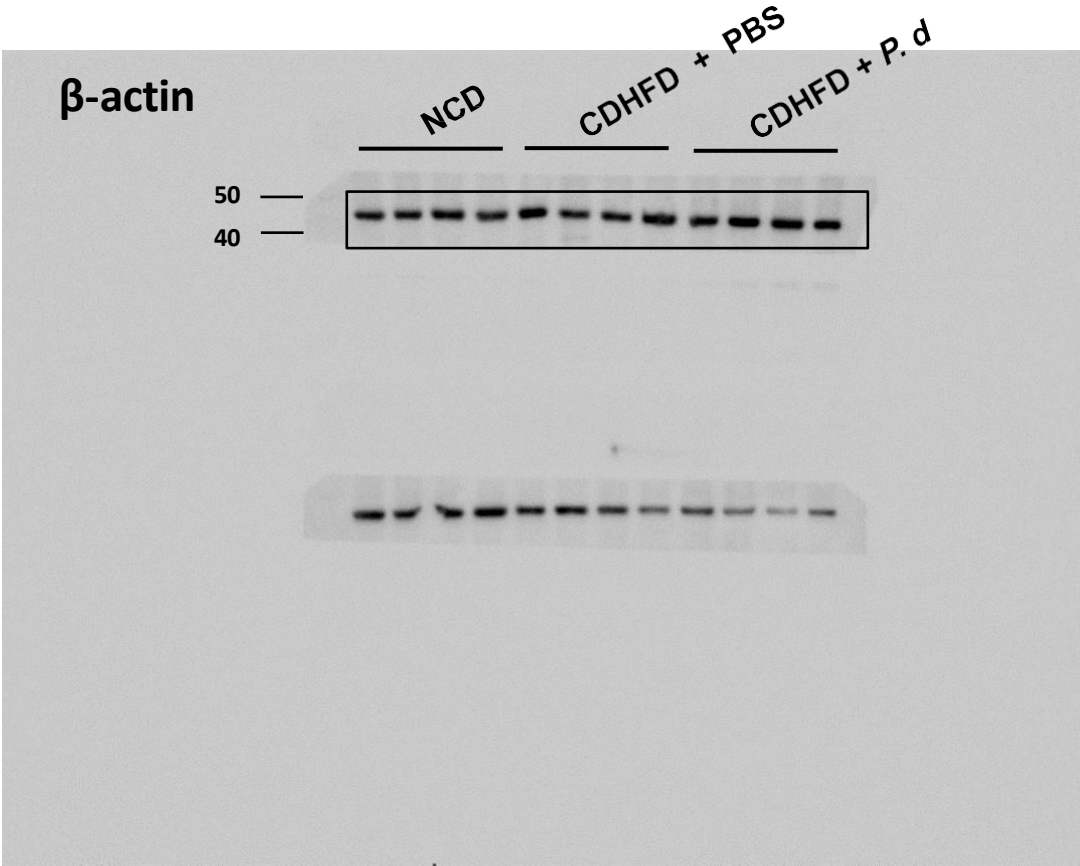

Figure 5d

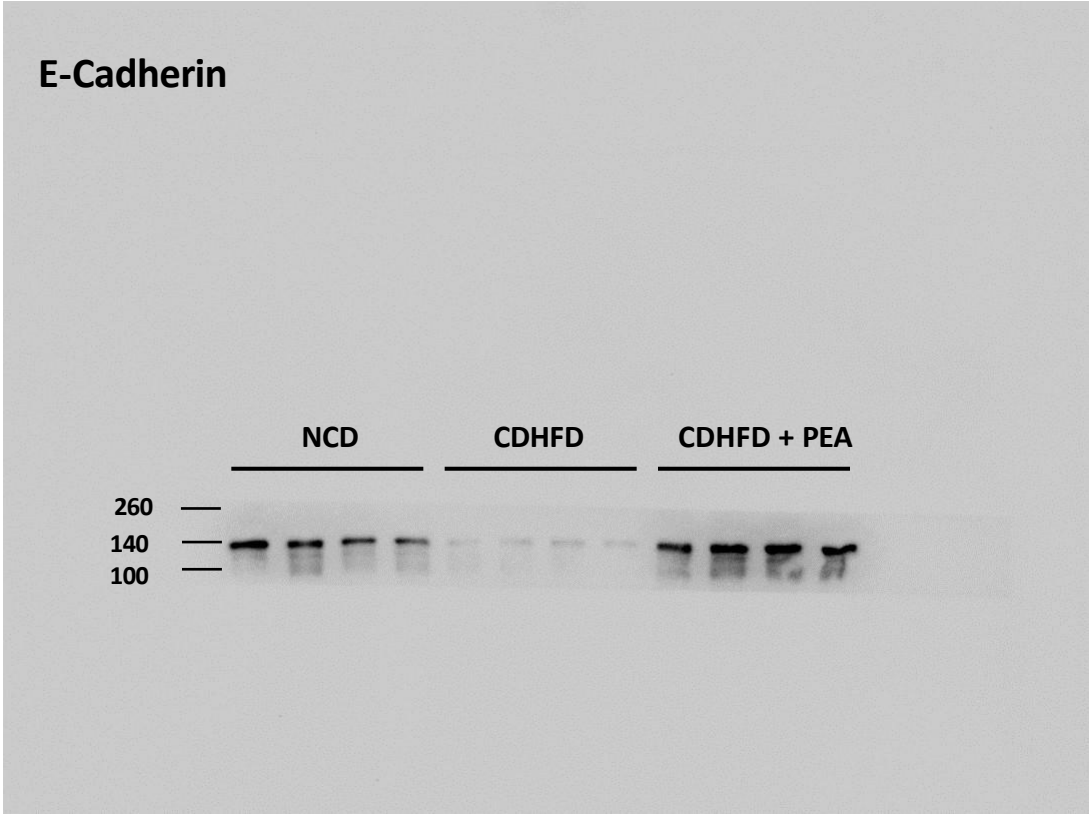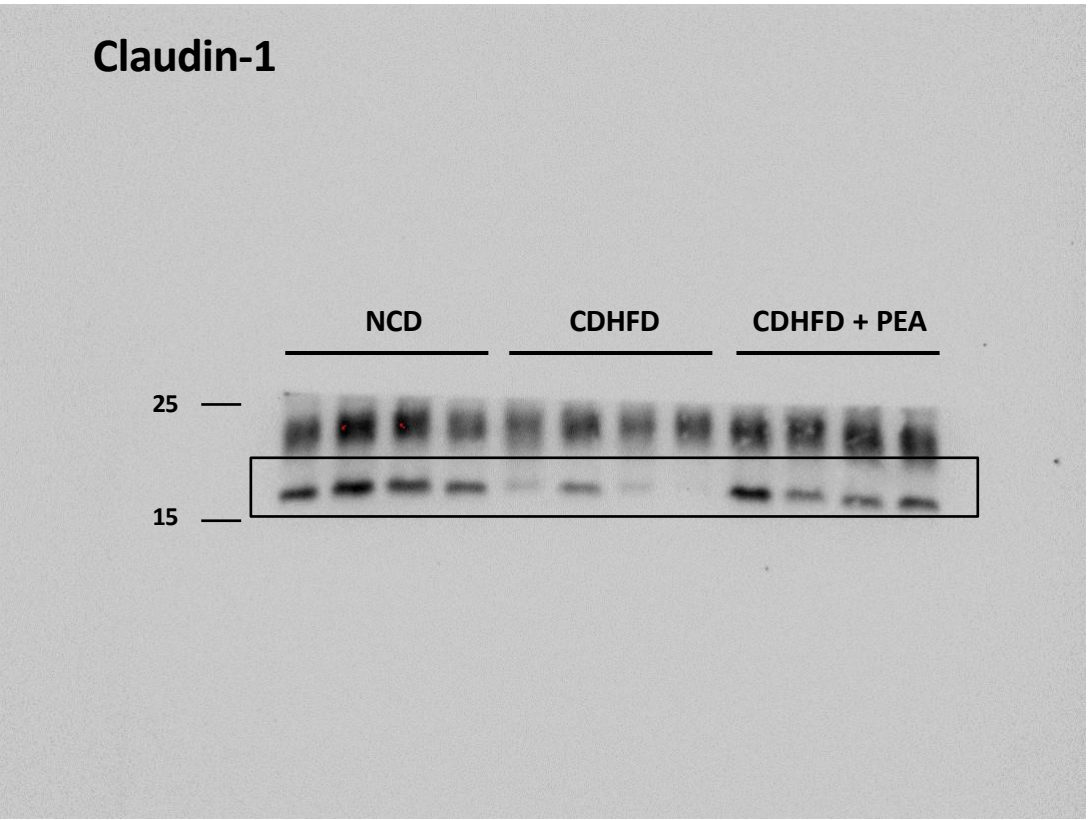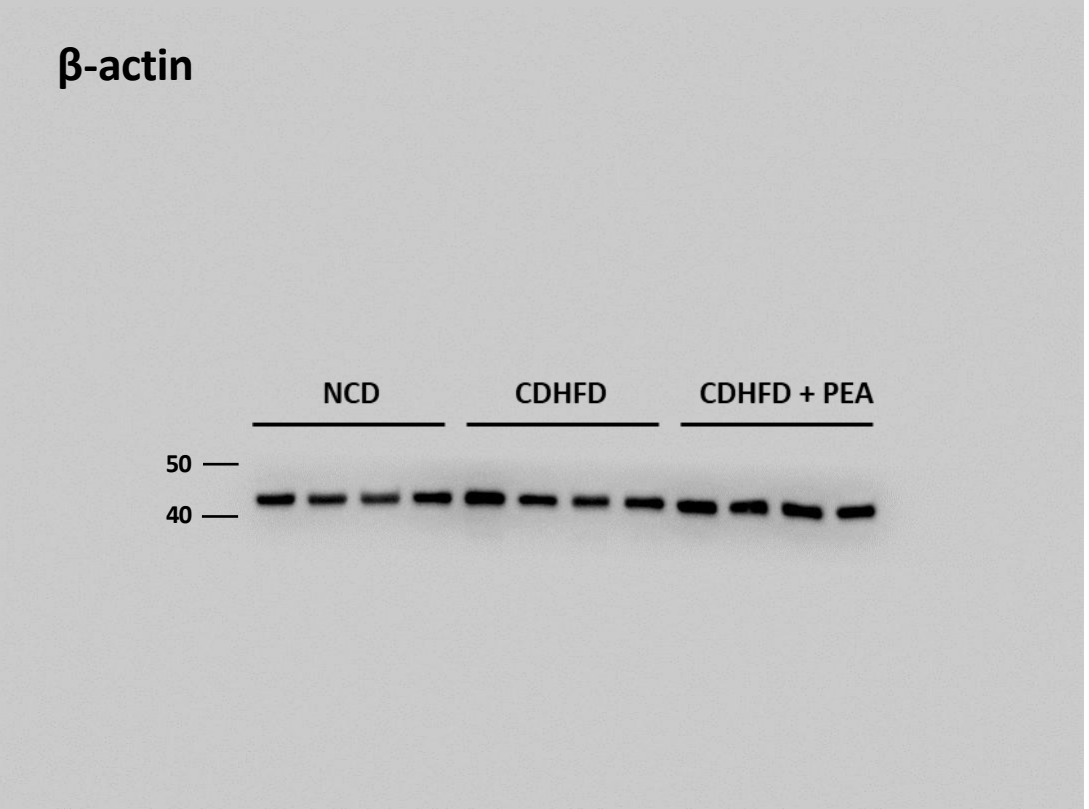

Supplement: Source Data Fig. 5 — Unprocessed western blots and/or gels. [file 41564_2023_1418_MOESM12_ESM.pdf]
