# Supplementary material for: Parabacteroides distasonis uses dietary inulin to suppress NASH via its metabolite pentadecanoic acid
Source: Nat Microbiol. 2023 Jun 29;8(8):1534–48. doi: 10.1038/s41564-023-01418-7 (PMC10390331; doi:10.1038/s41564-023-01418-7)

Source images for Extended Figure 1

Including H&E staining and sirius red staining
Replicate images from a same mouse were
displayed within a same page

H&E Staining

NCD group

(7 mice were included)

NCD-1

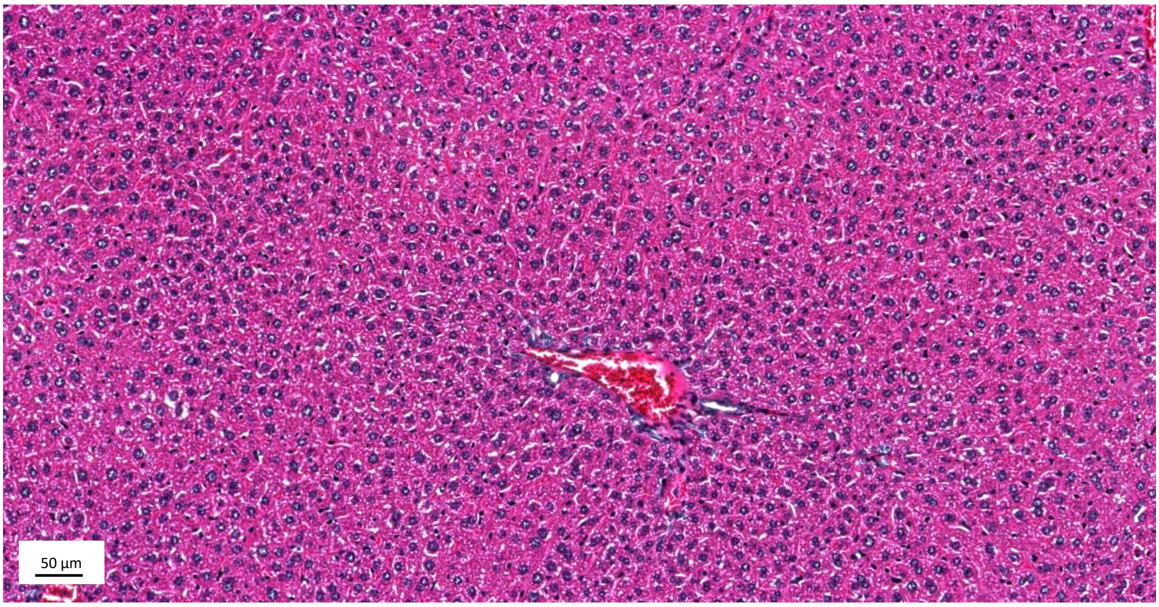

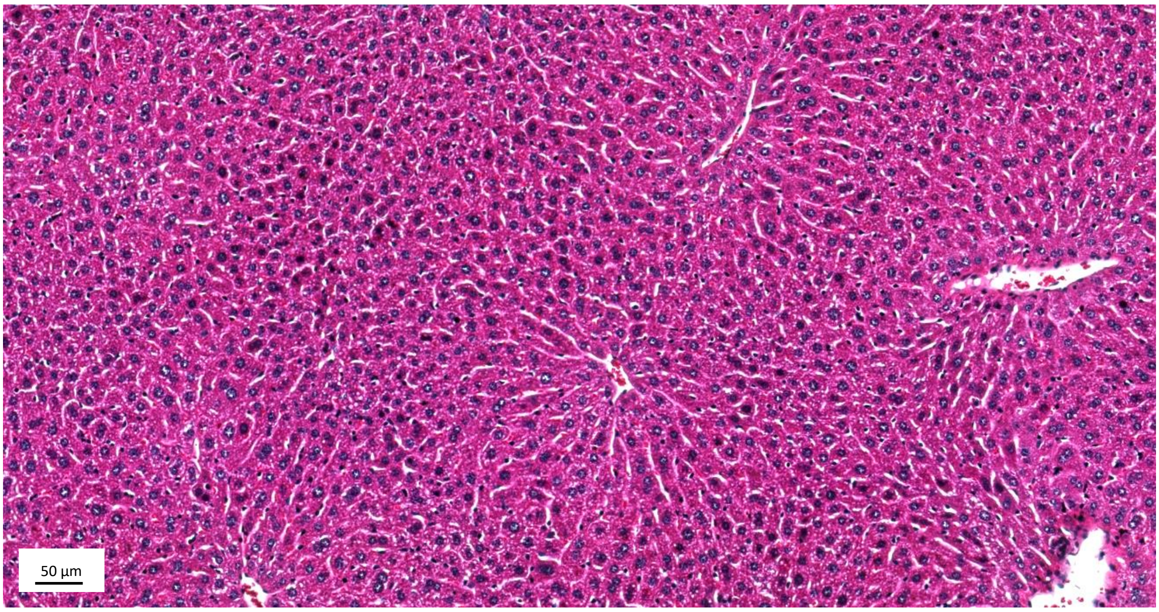

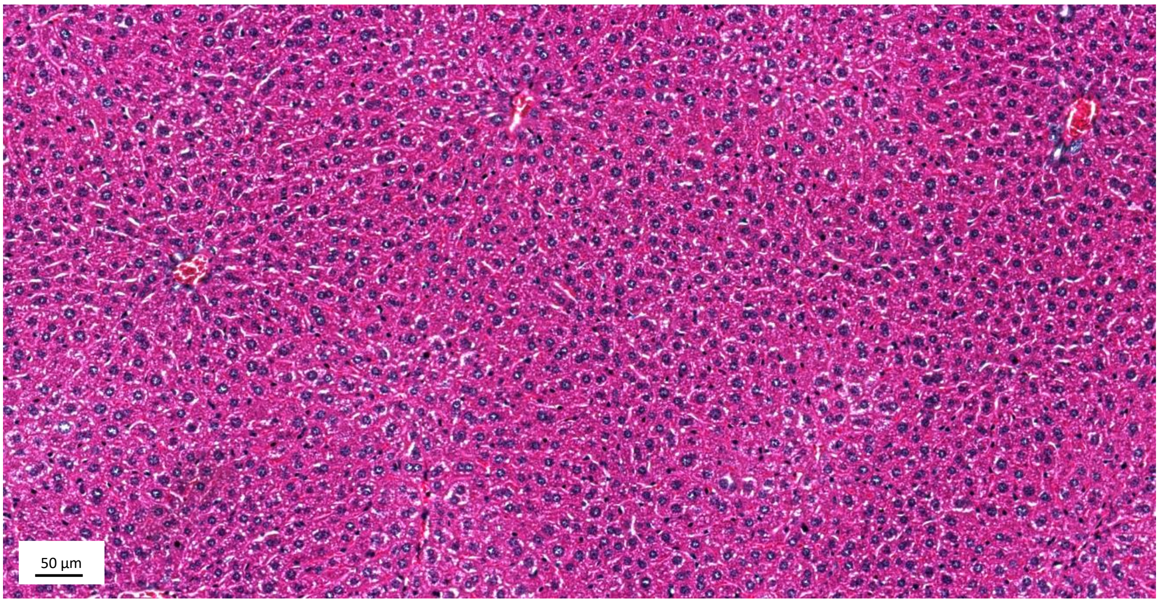

NCD-2

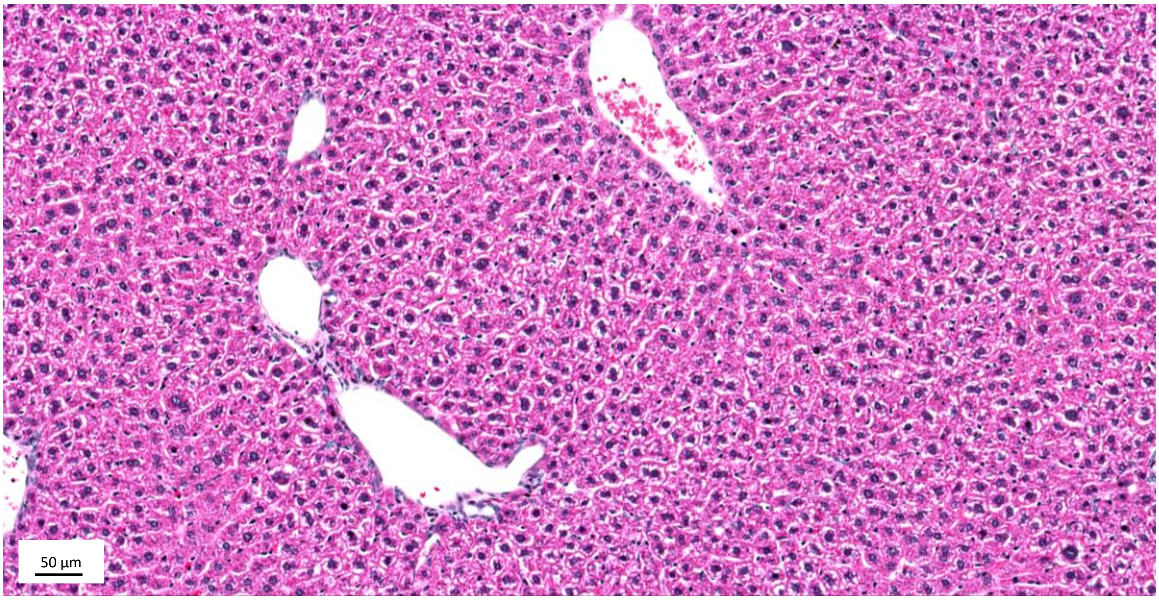

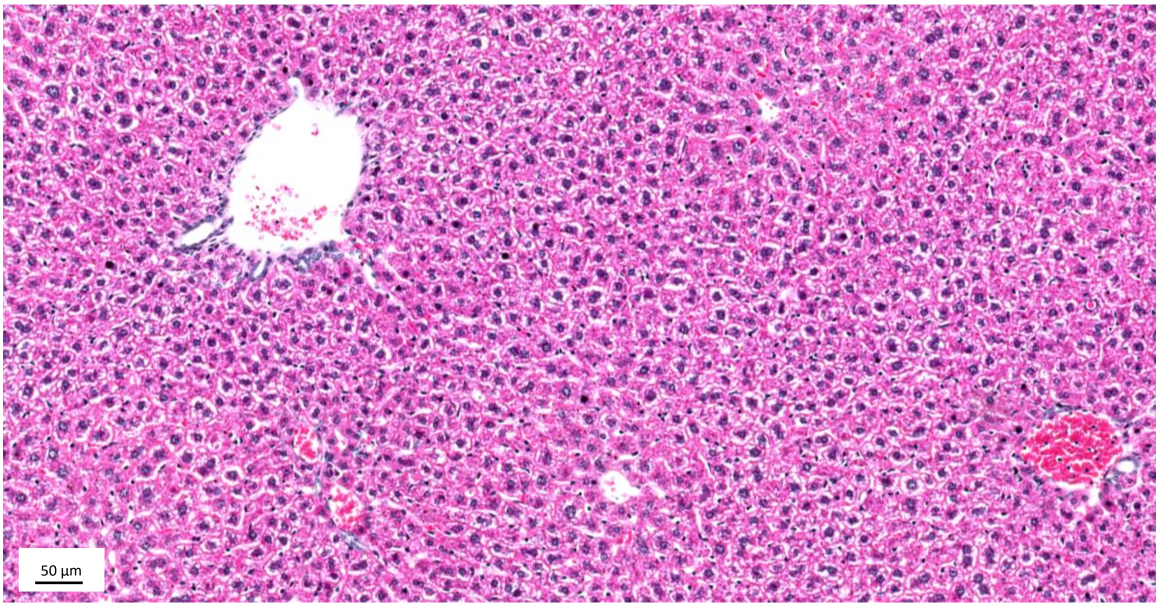

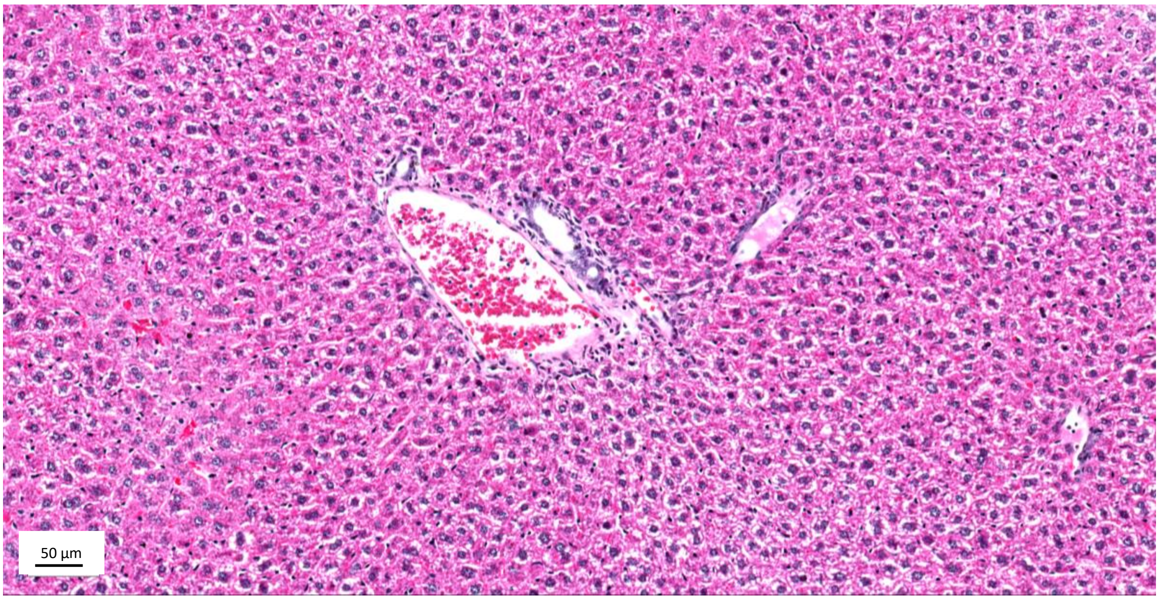

NCD-3

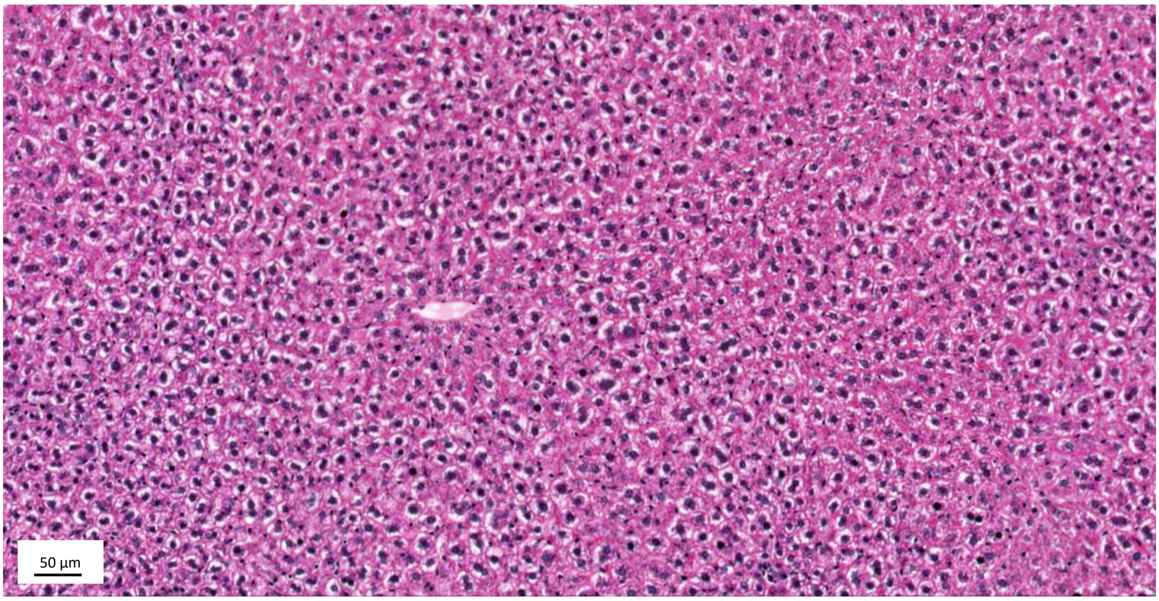

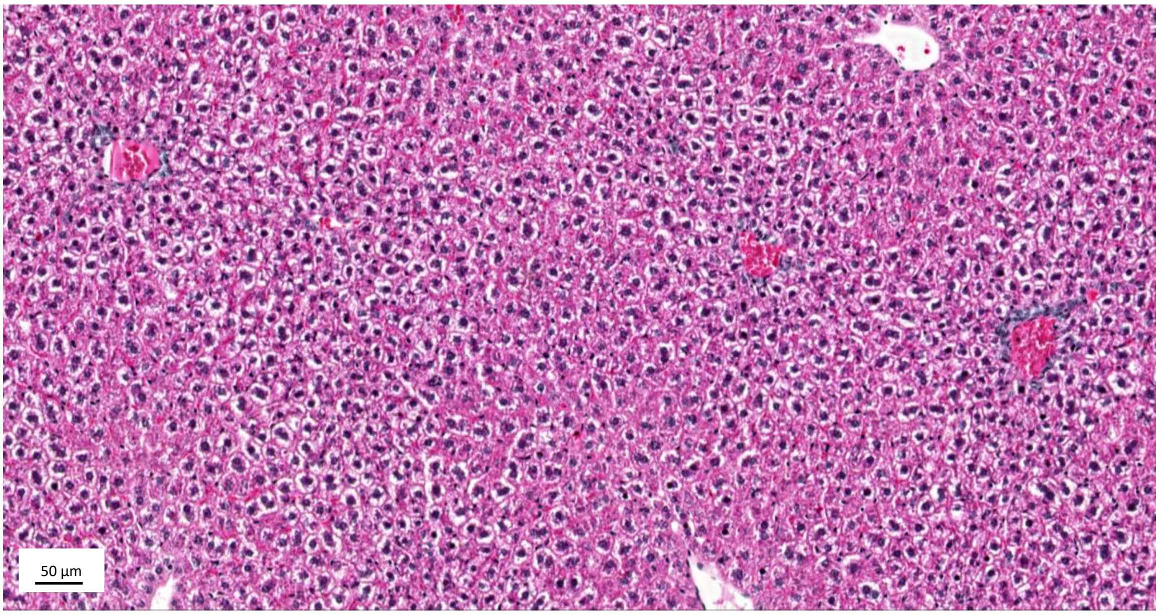

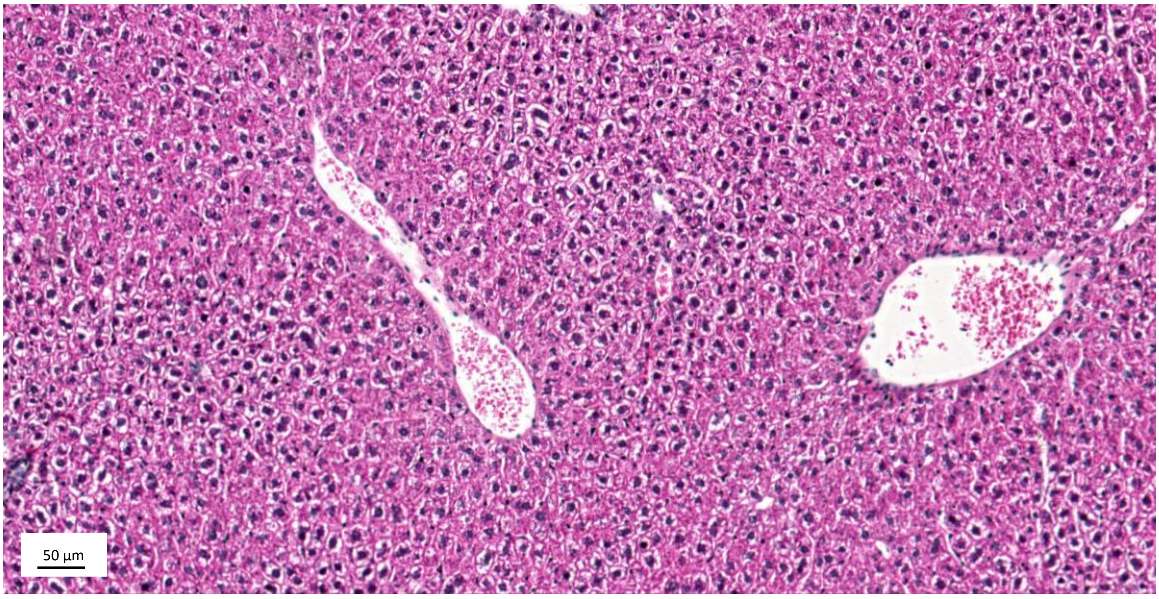

NCD-4

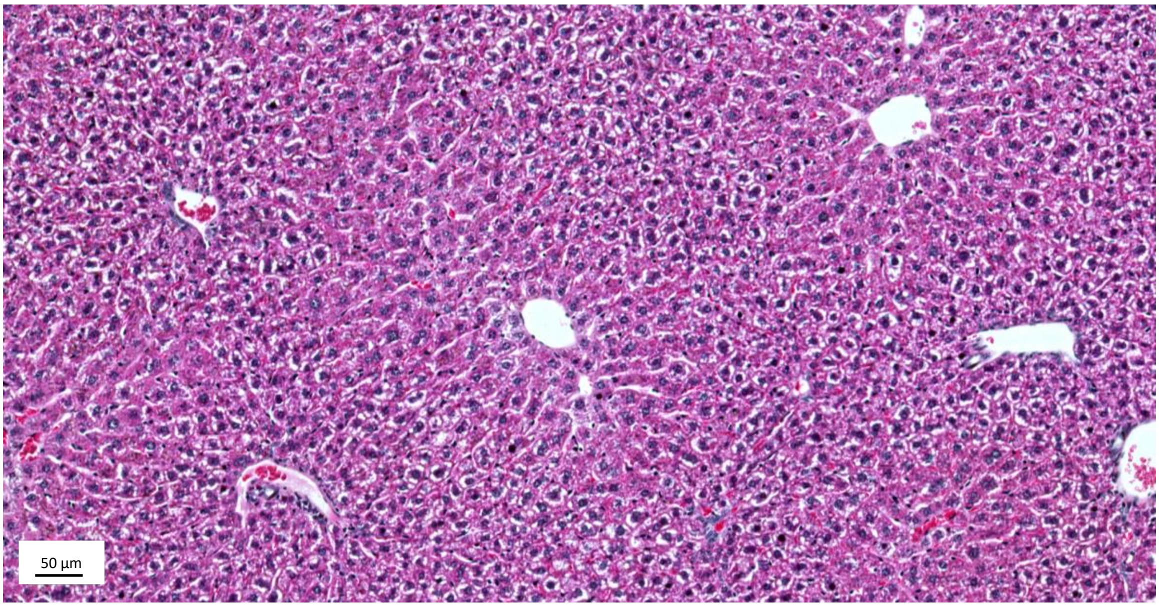

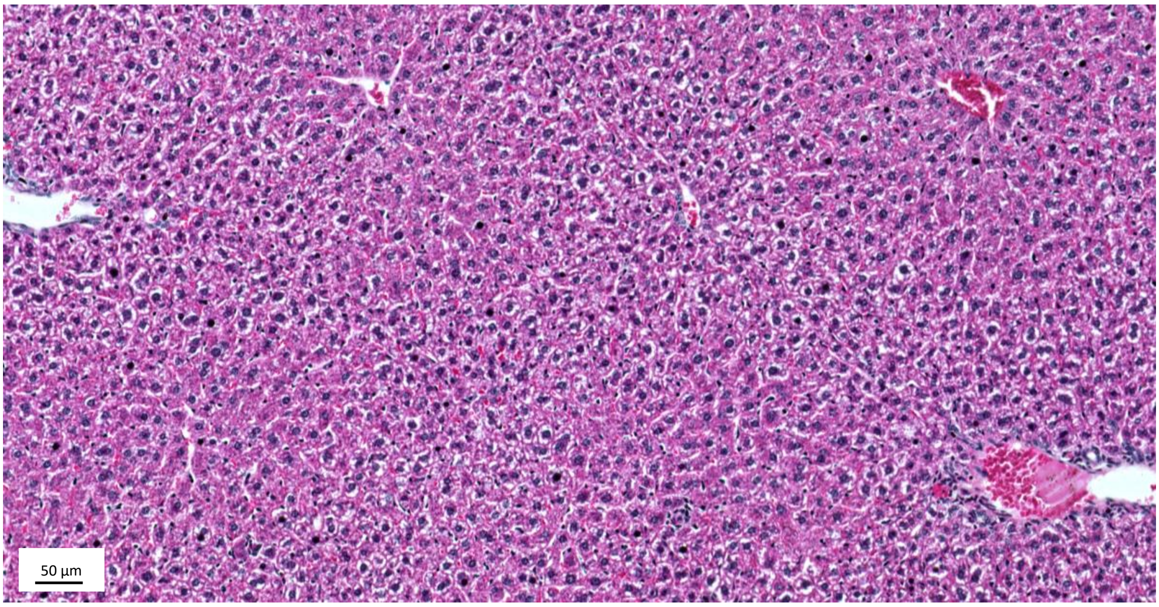

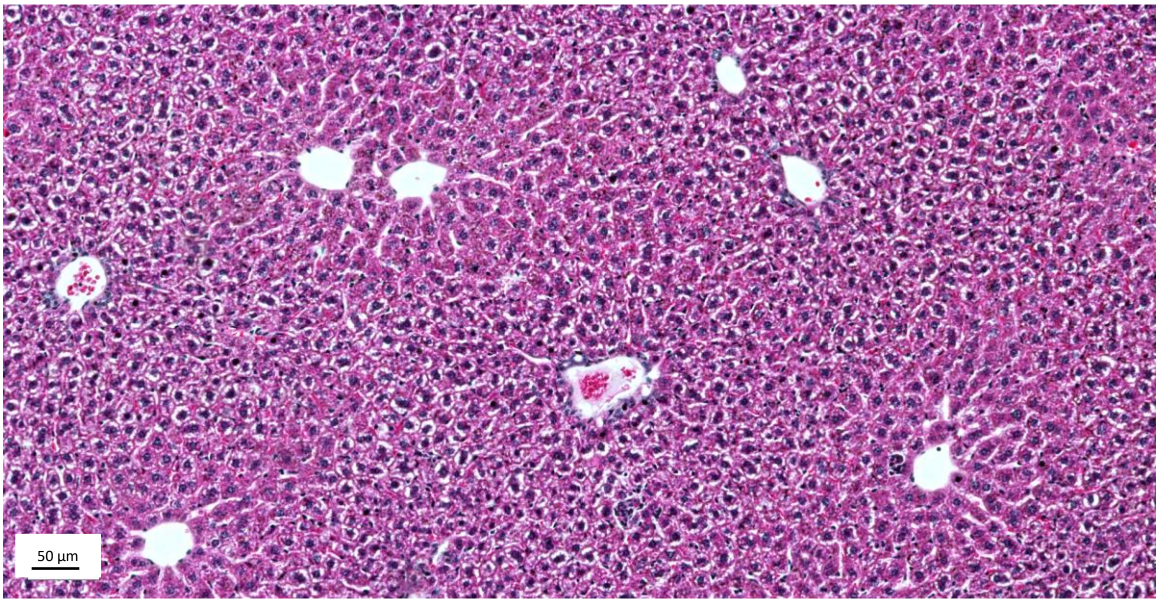

NCD-5

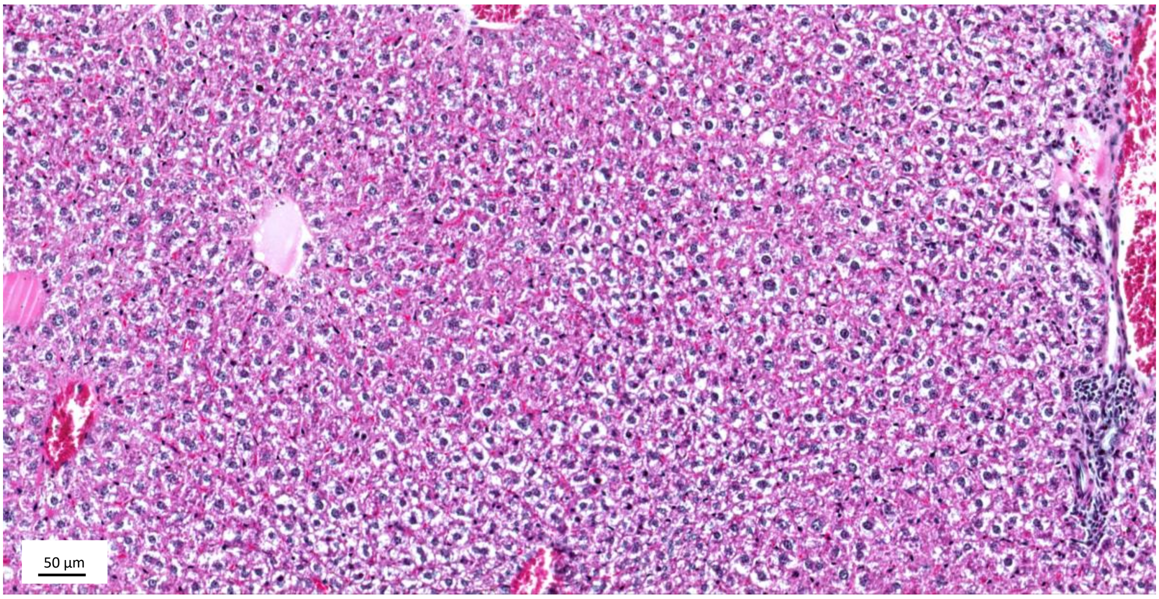

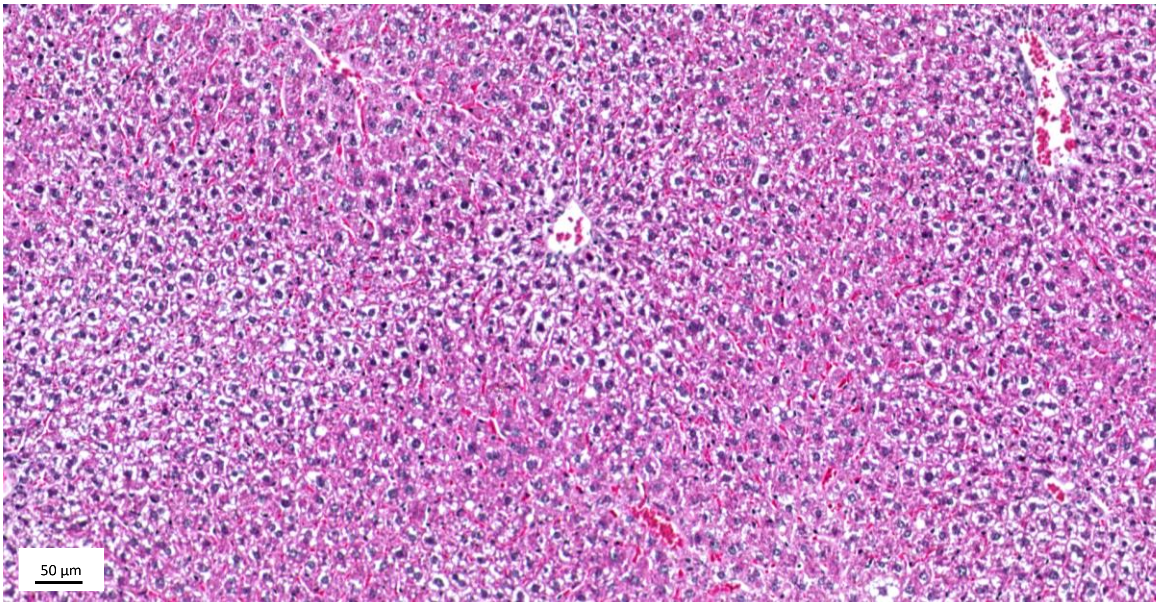

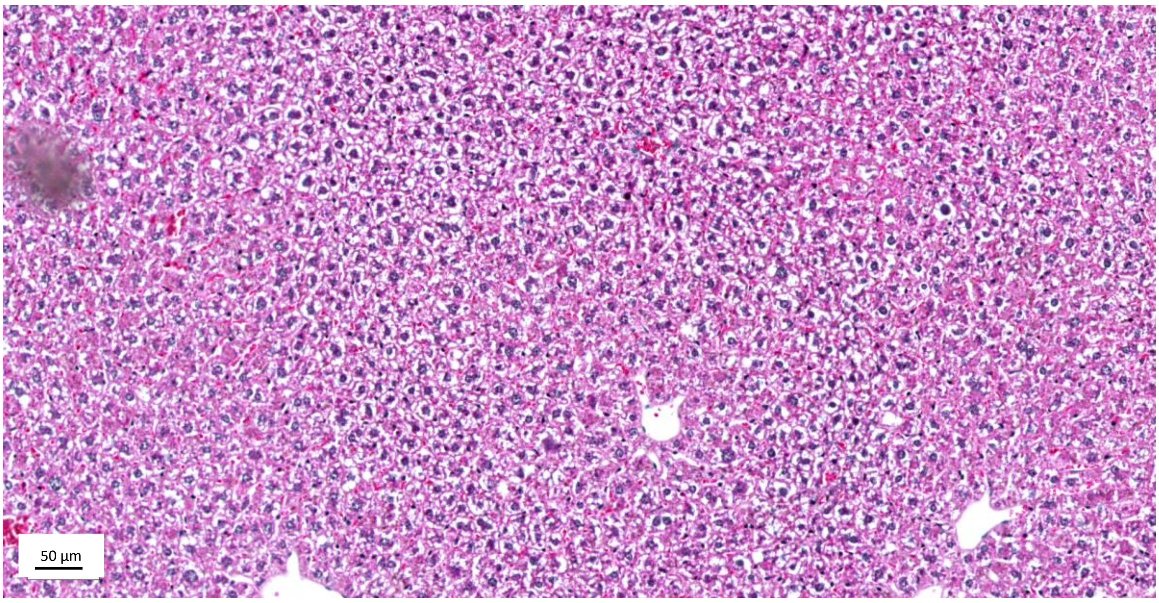

NCD-6

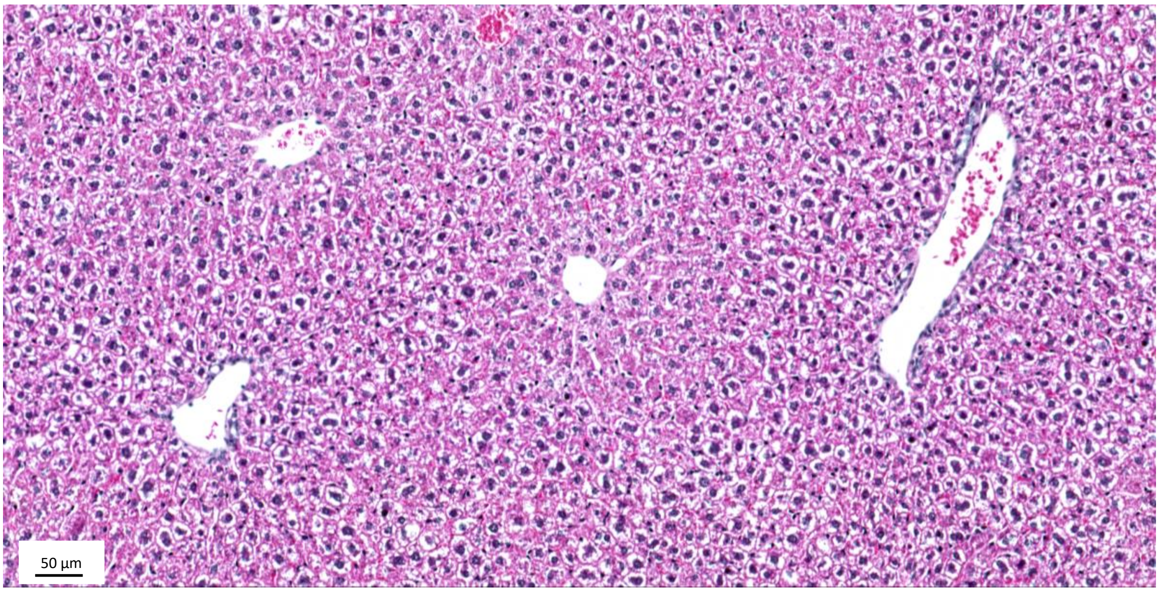

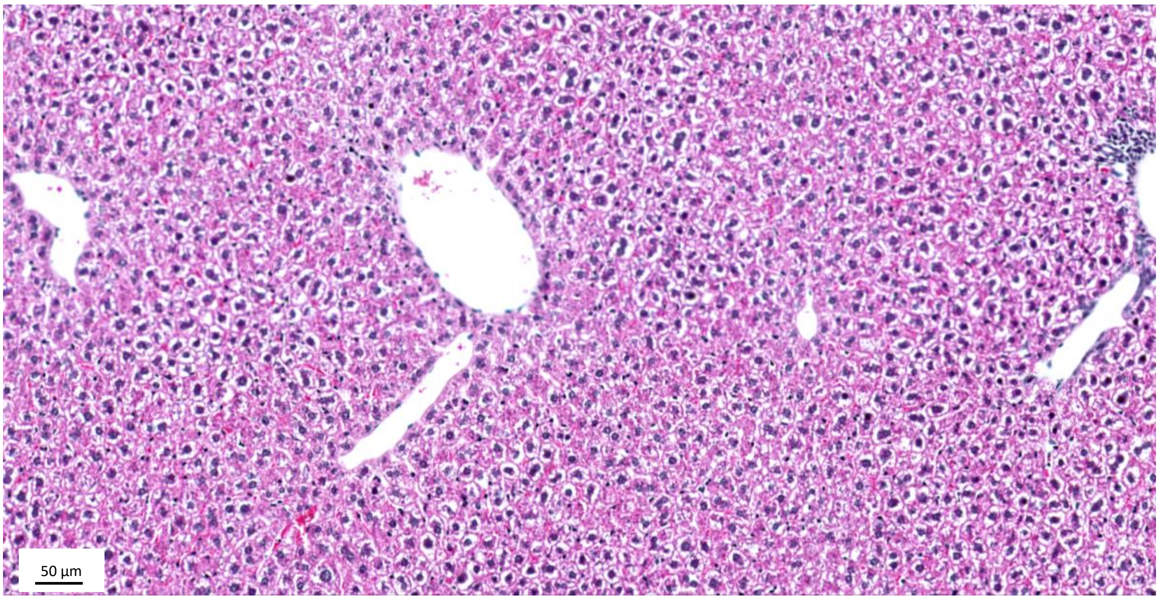

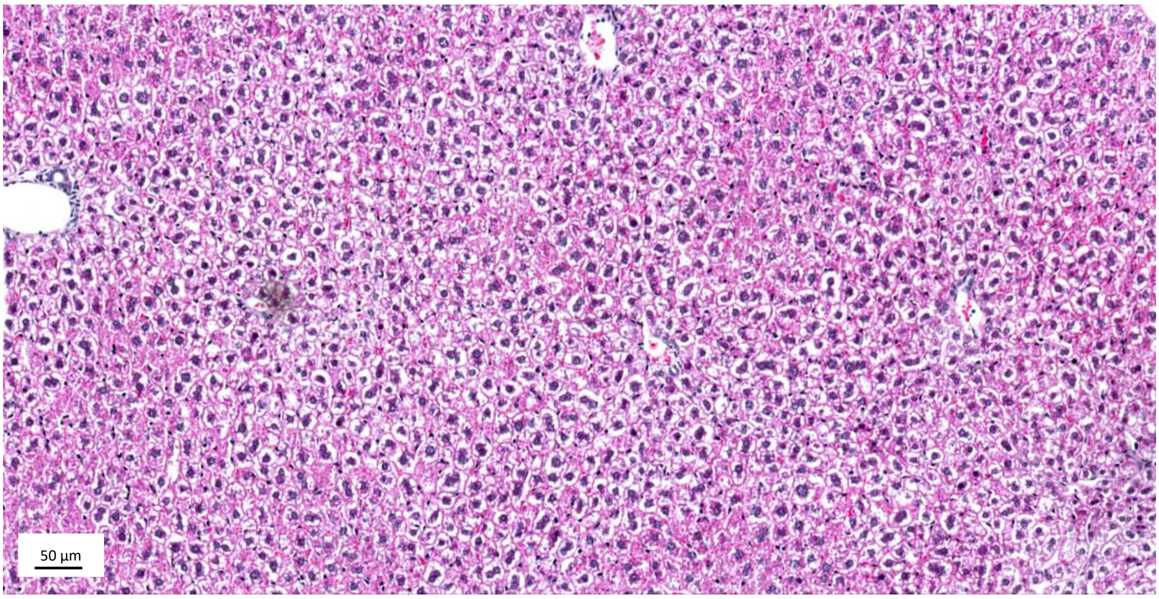

NCD-7

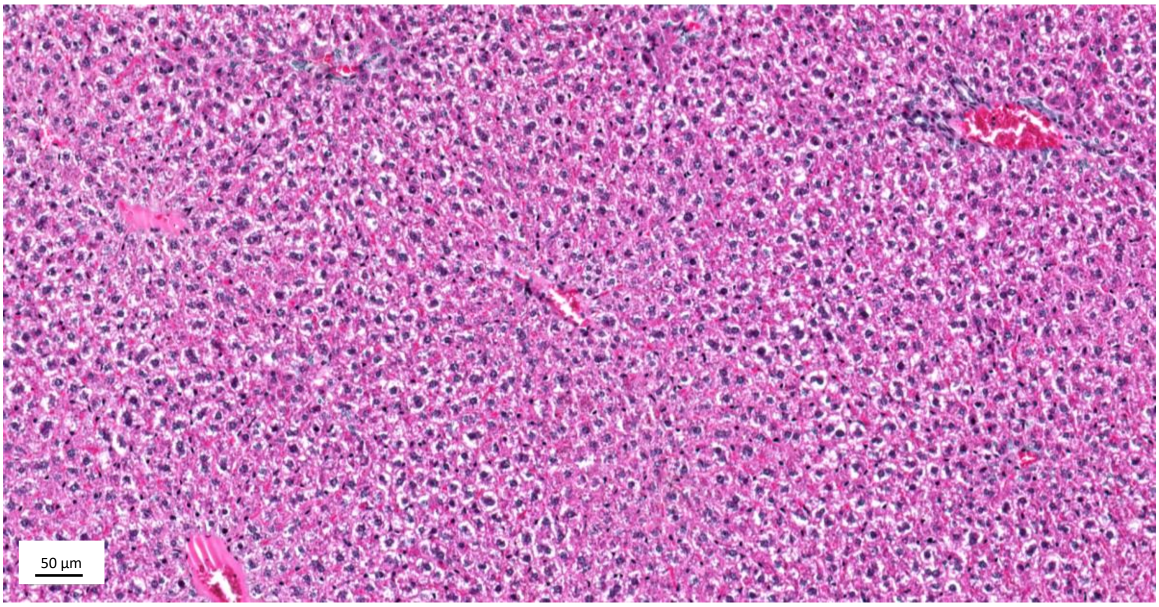

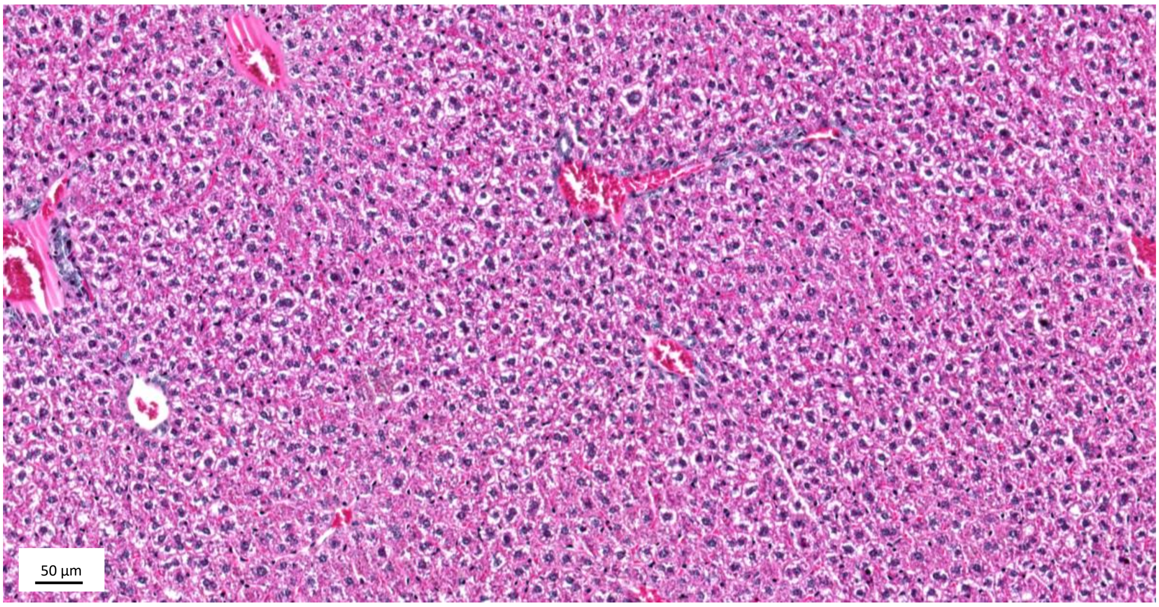

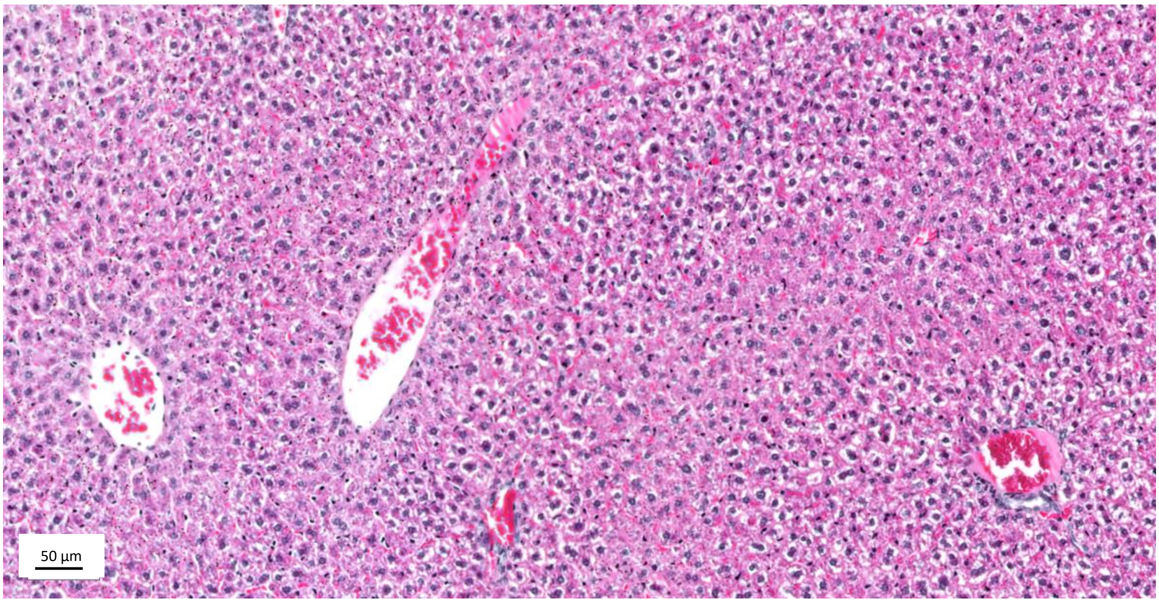

H&E Staining

HFHCD group

(15 mice were included)

HFHCD-1

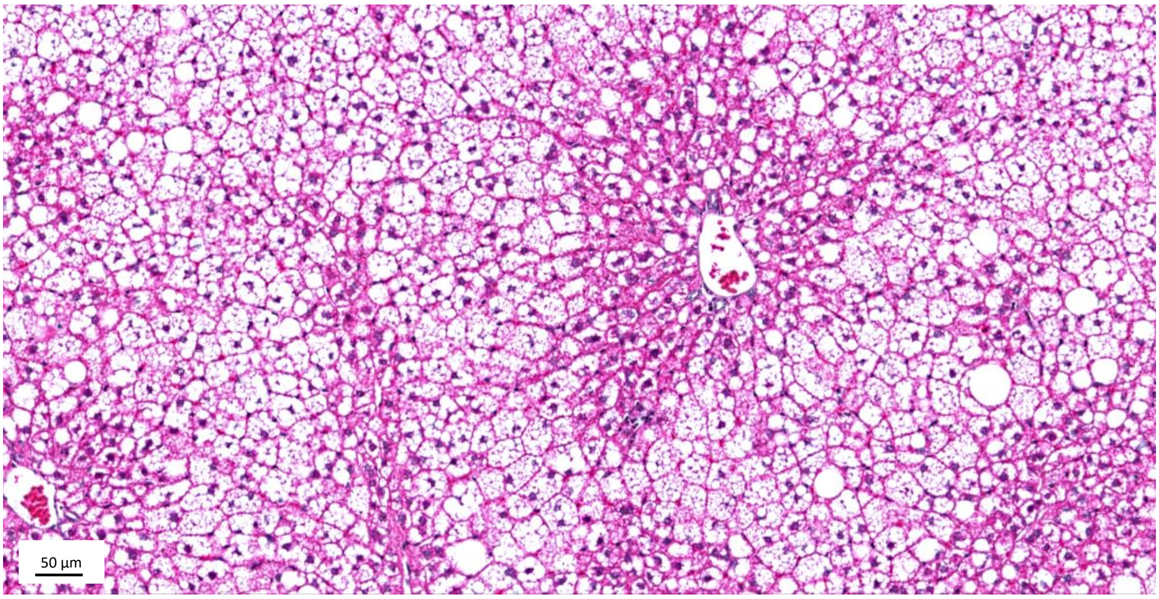

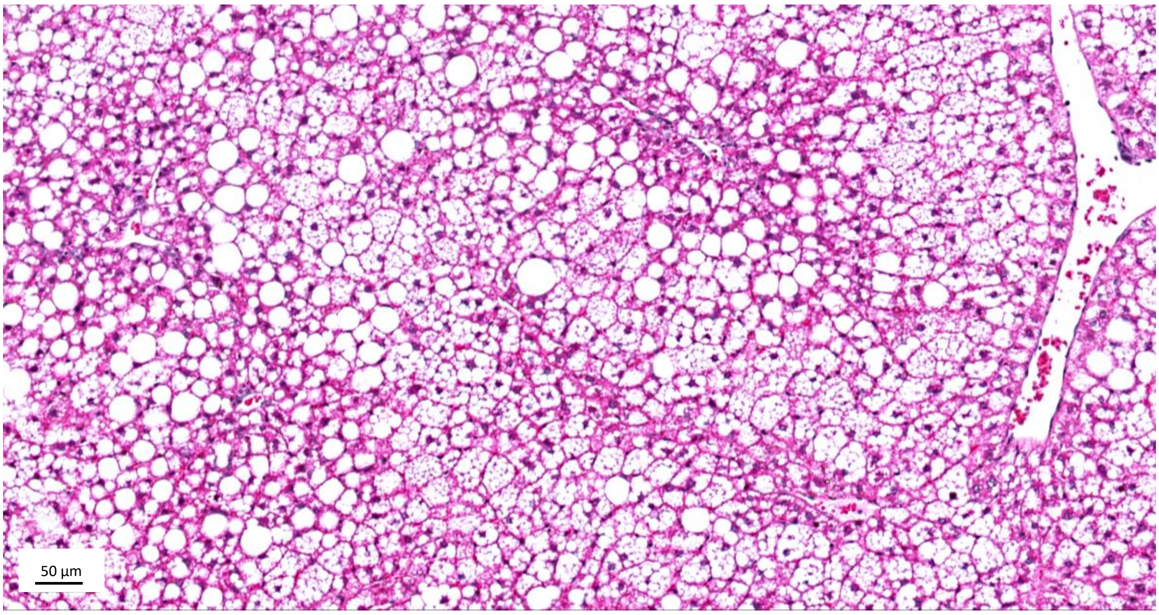

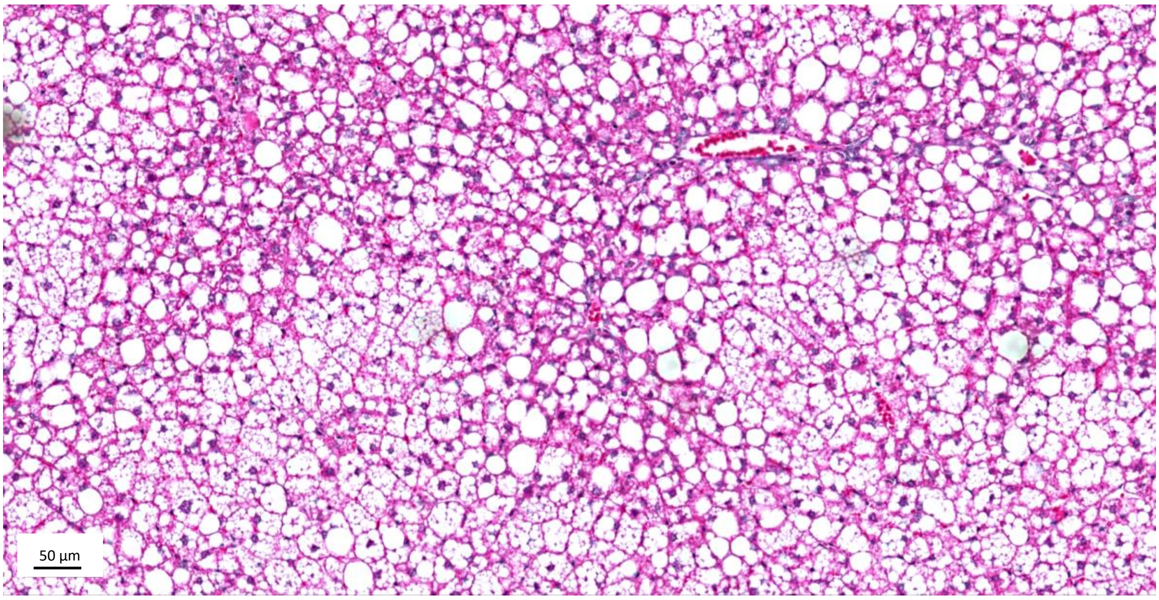

HFHCD-2

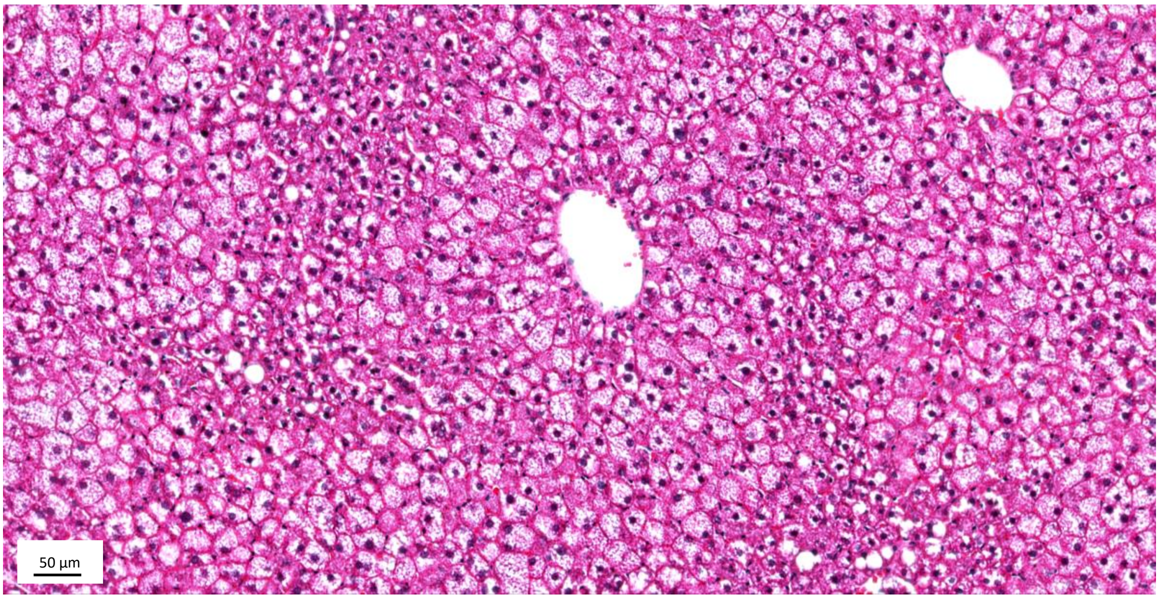

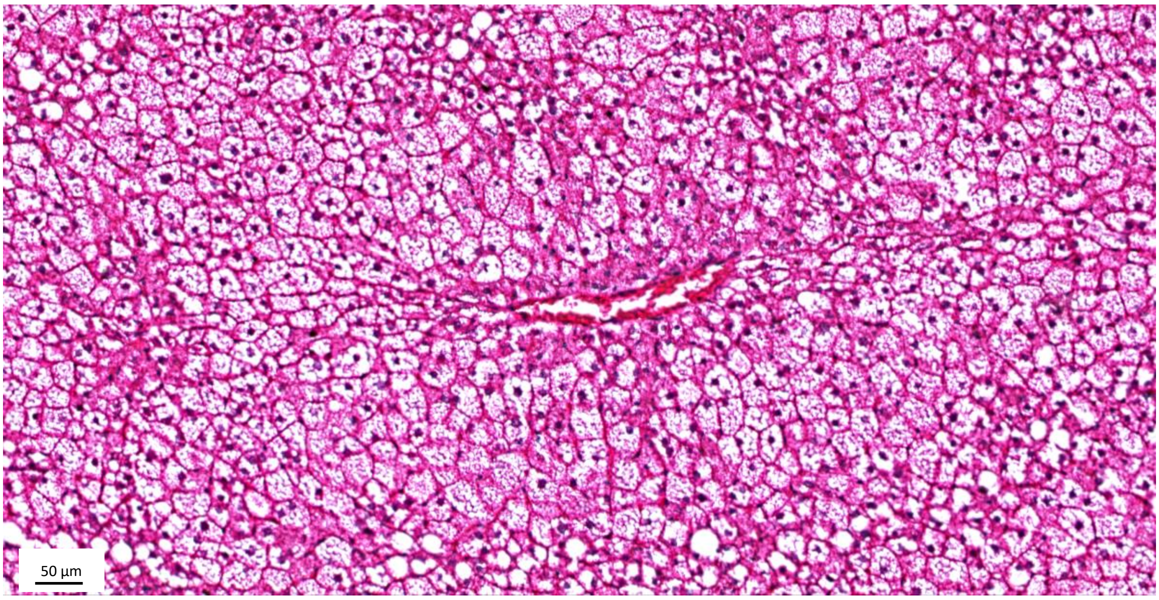

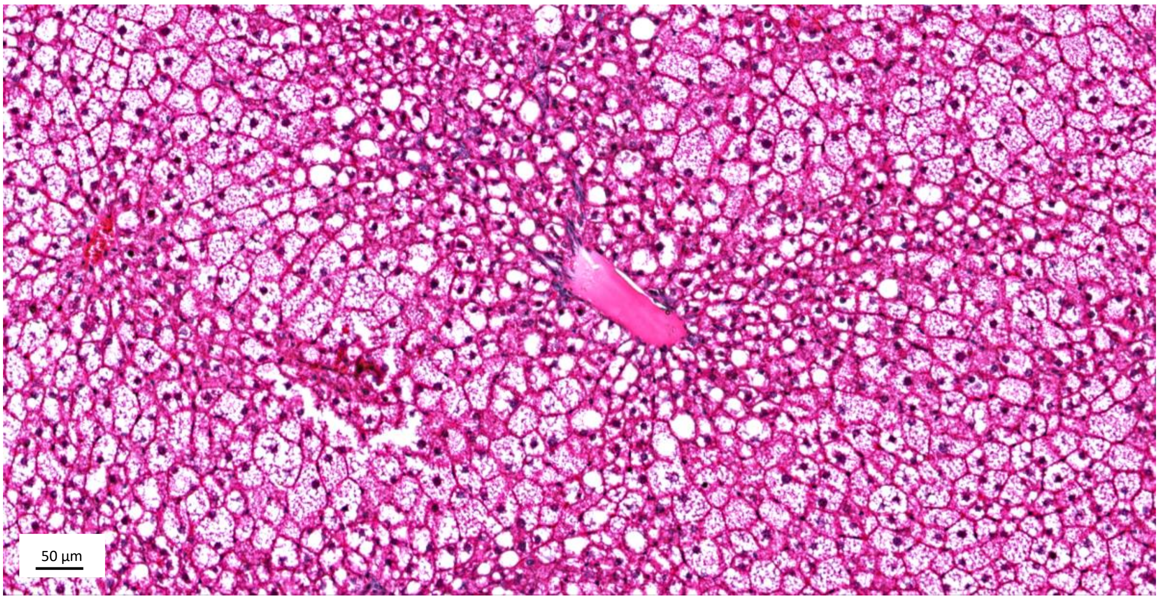

HFHCD-3

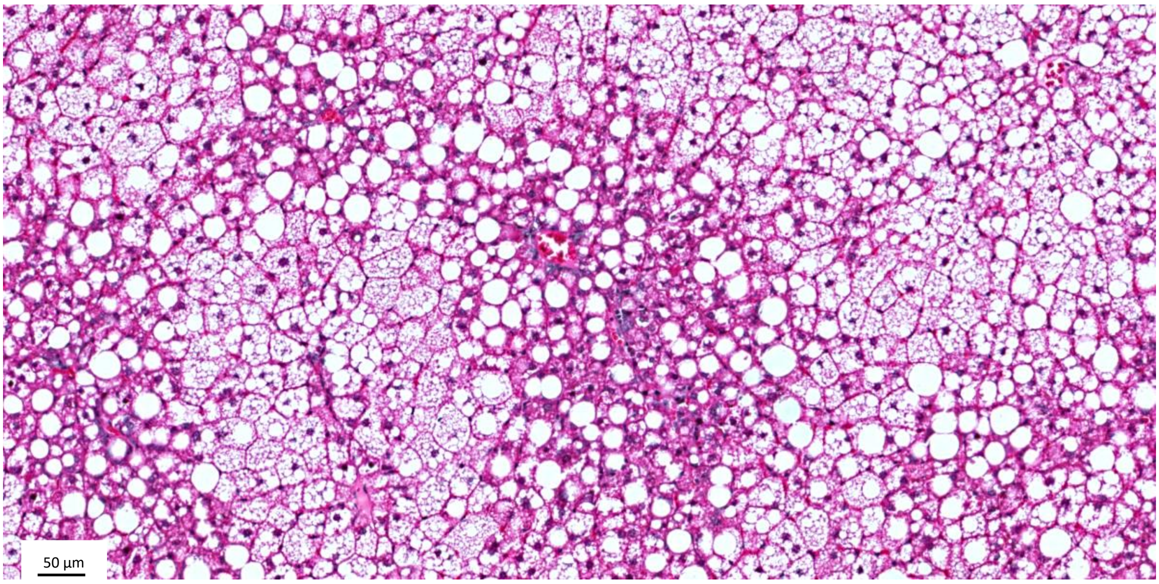

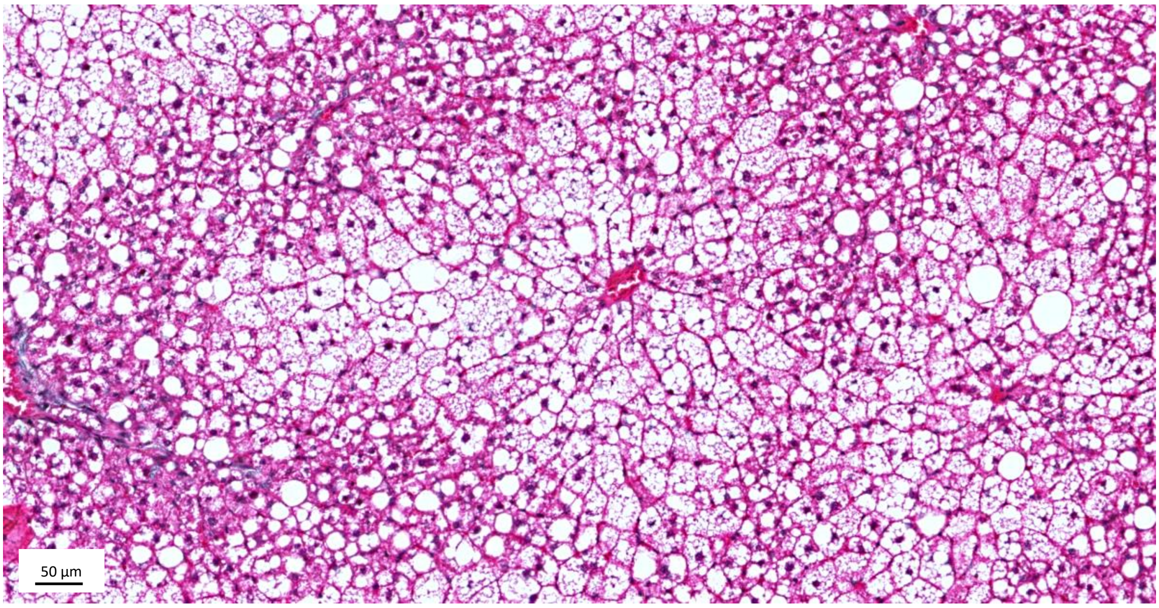

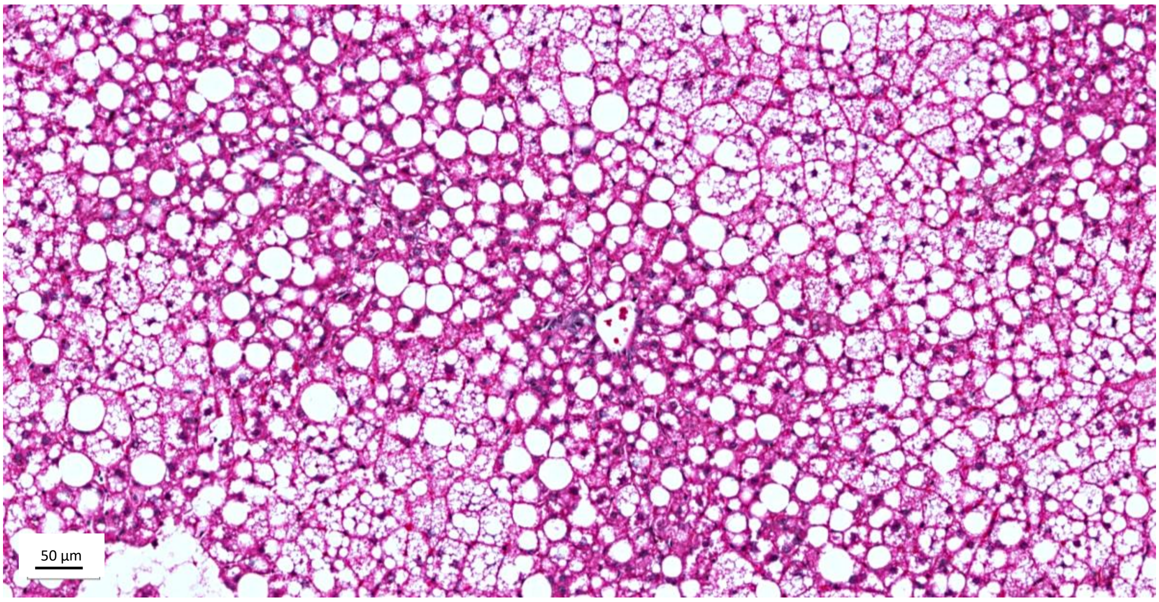

HFHCD-4

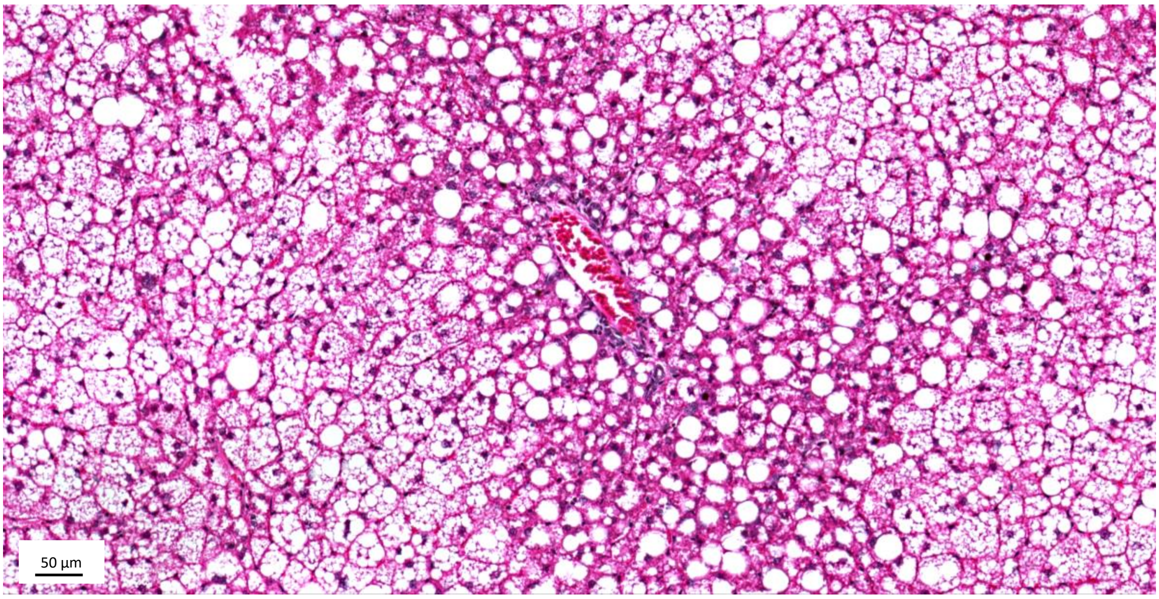

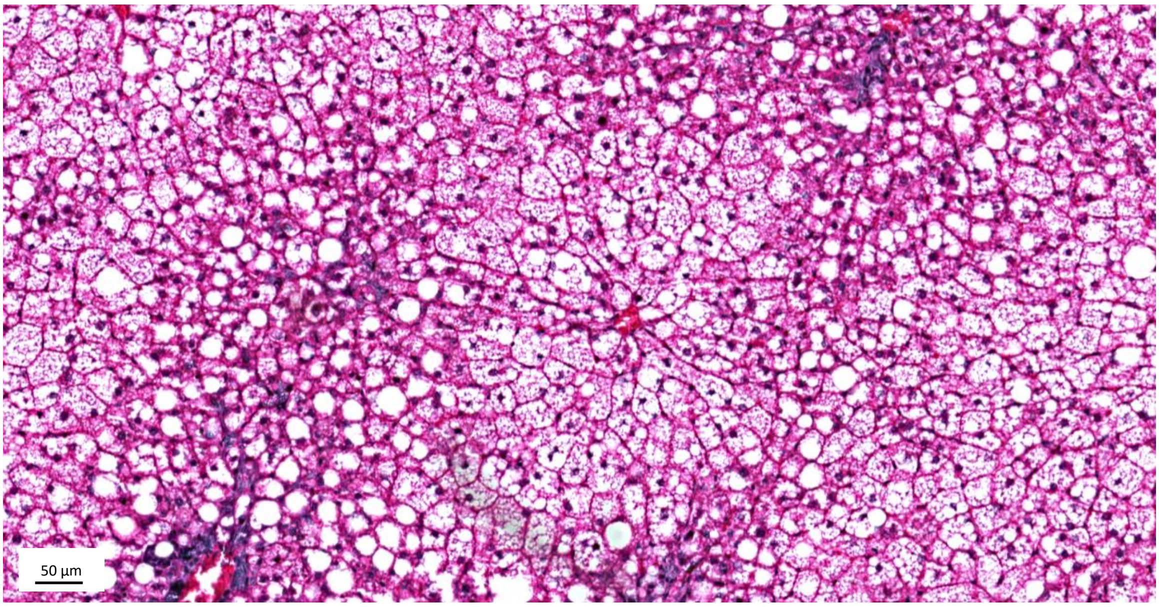

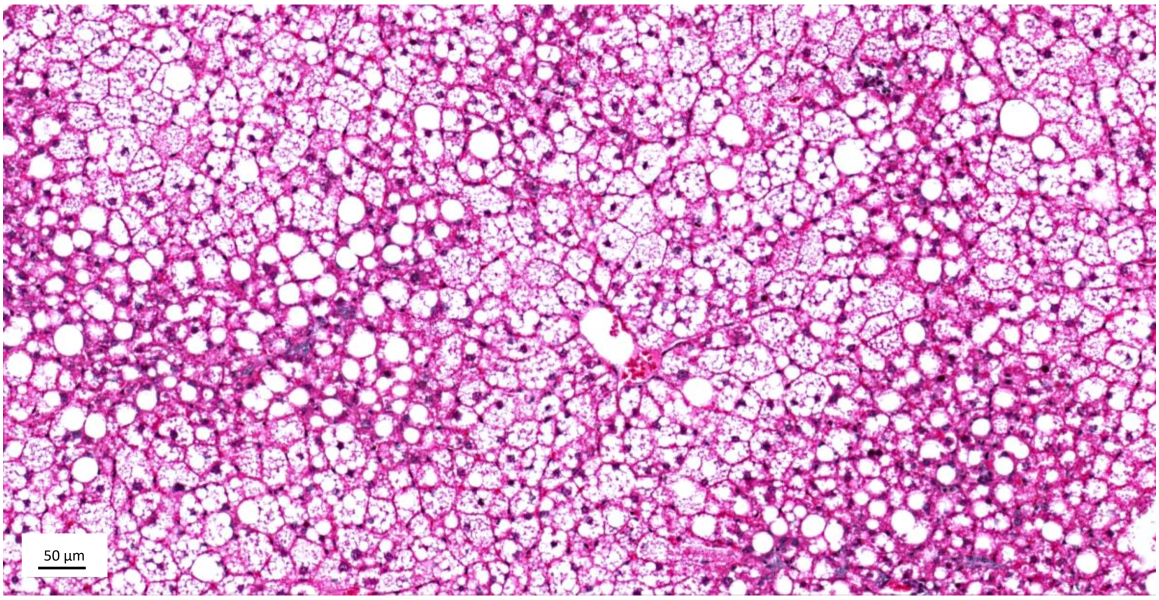

HFHCD-5

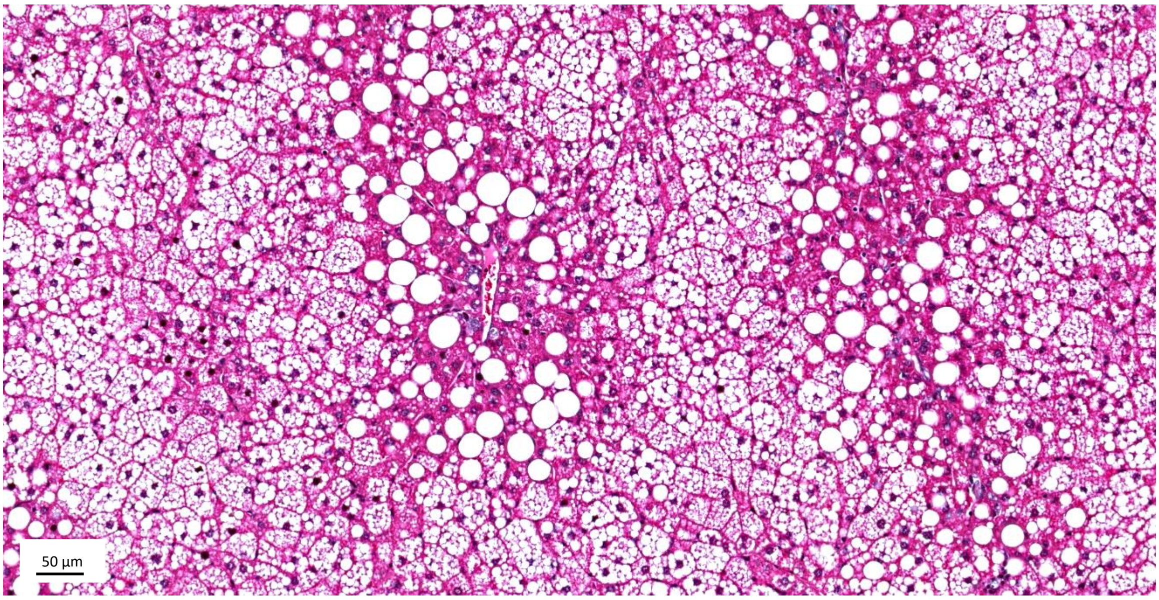

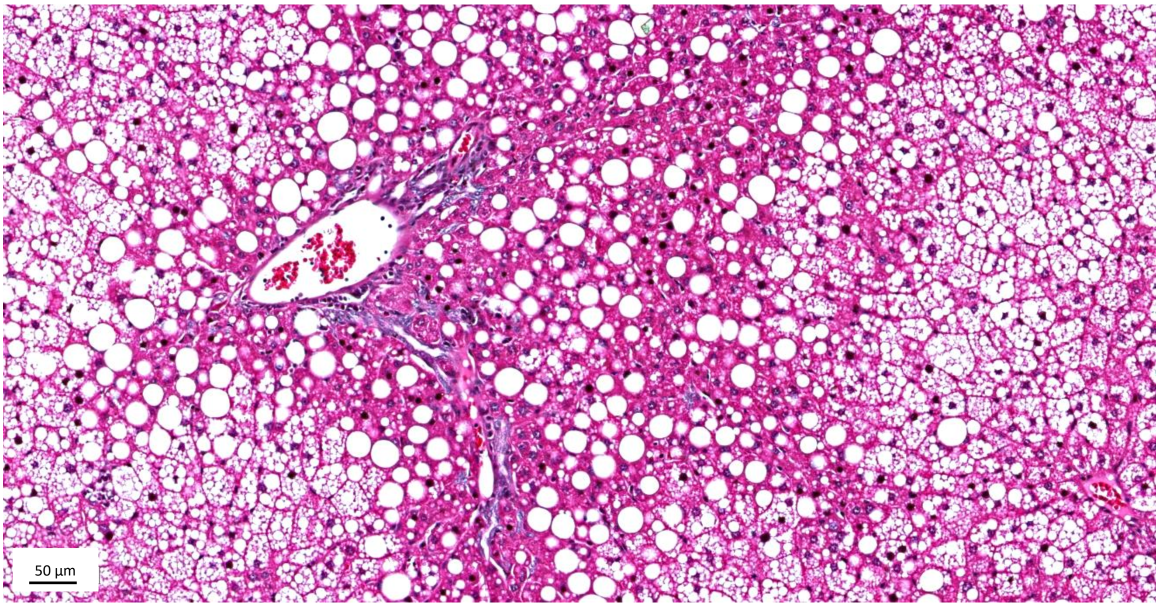

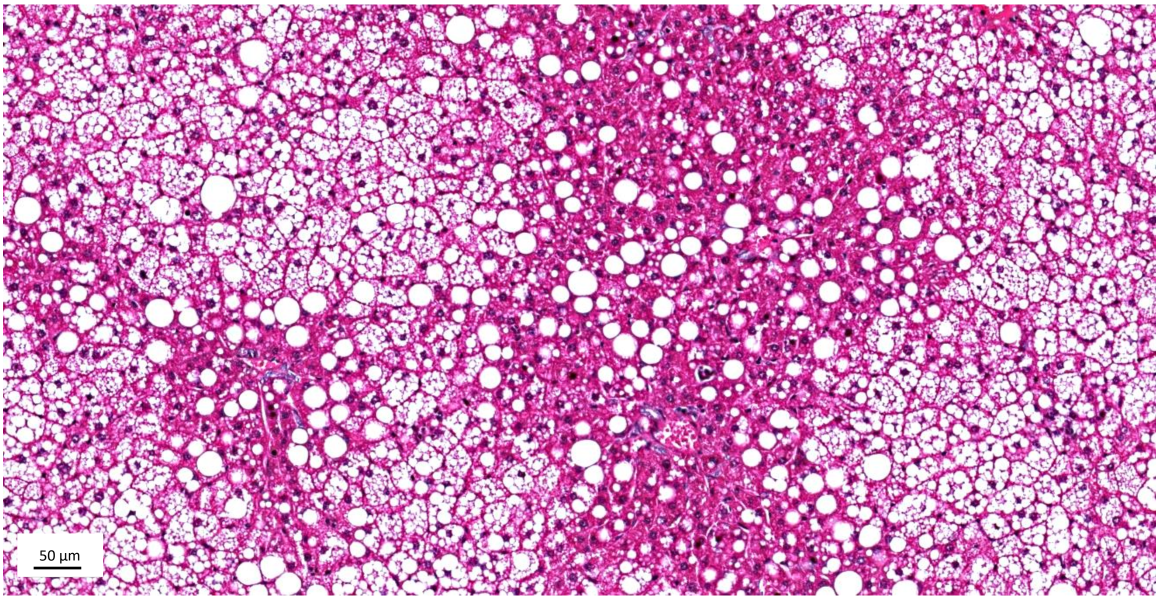

HFHCD-6

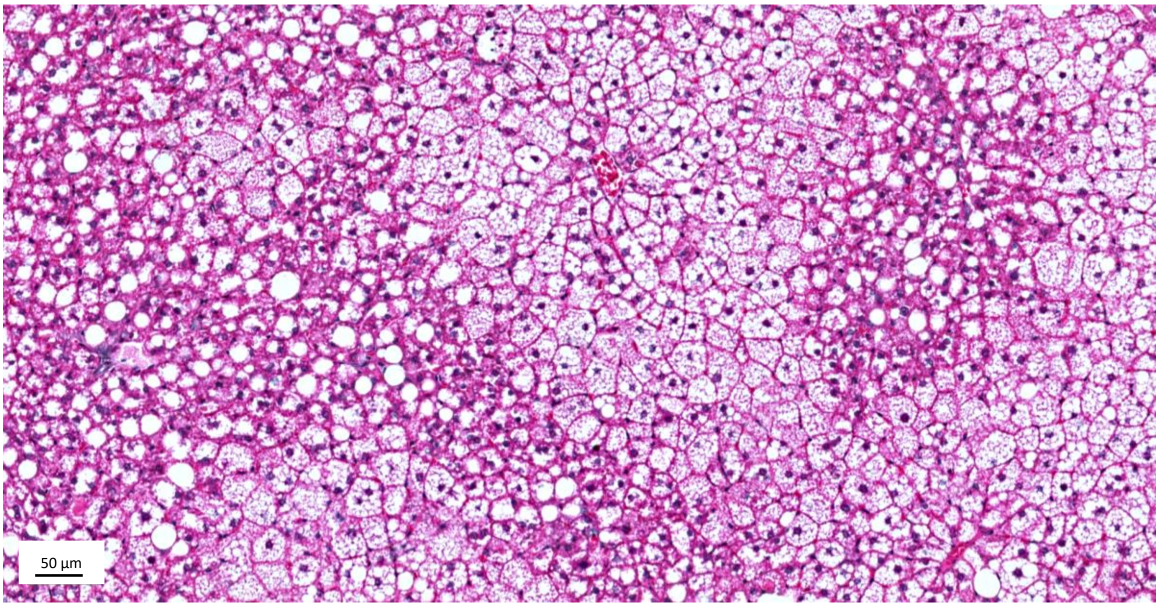

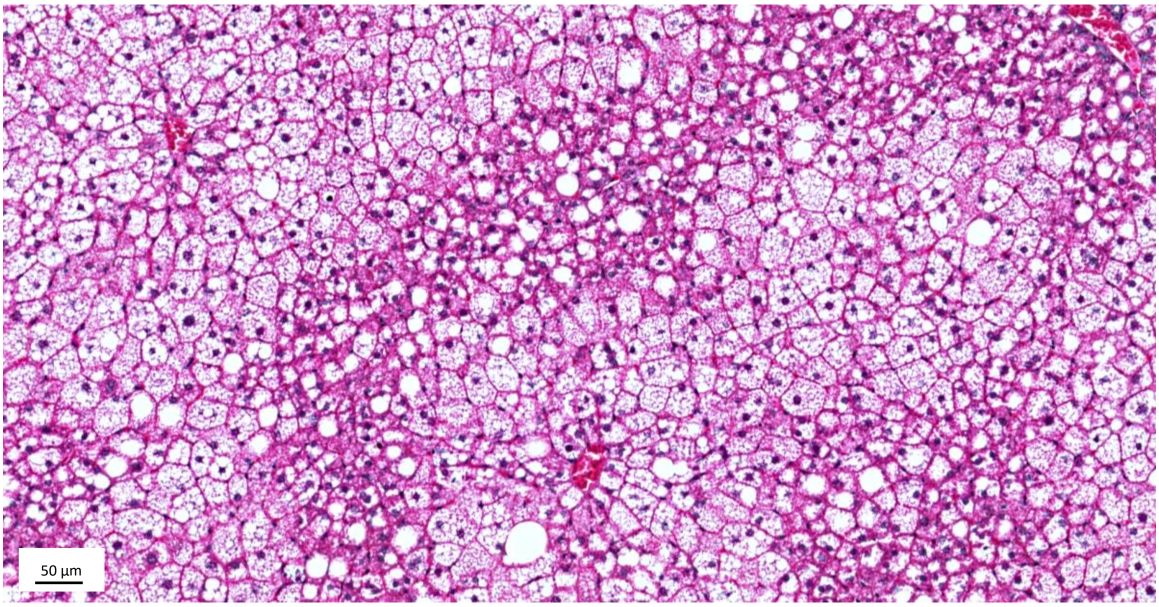

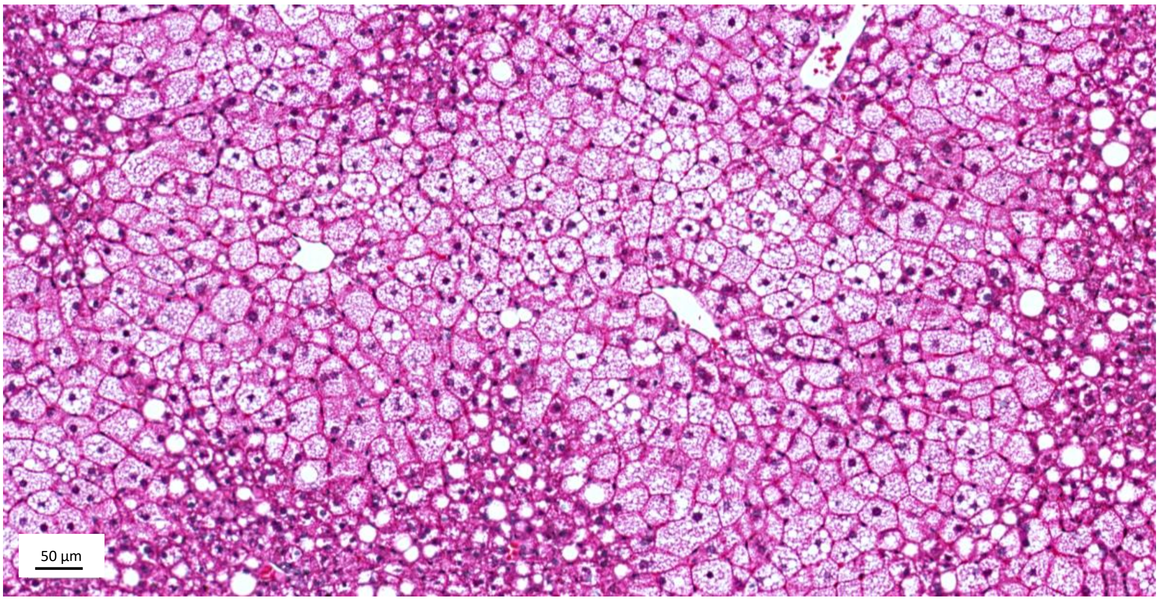

HFHCD-7

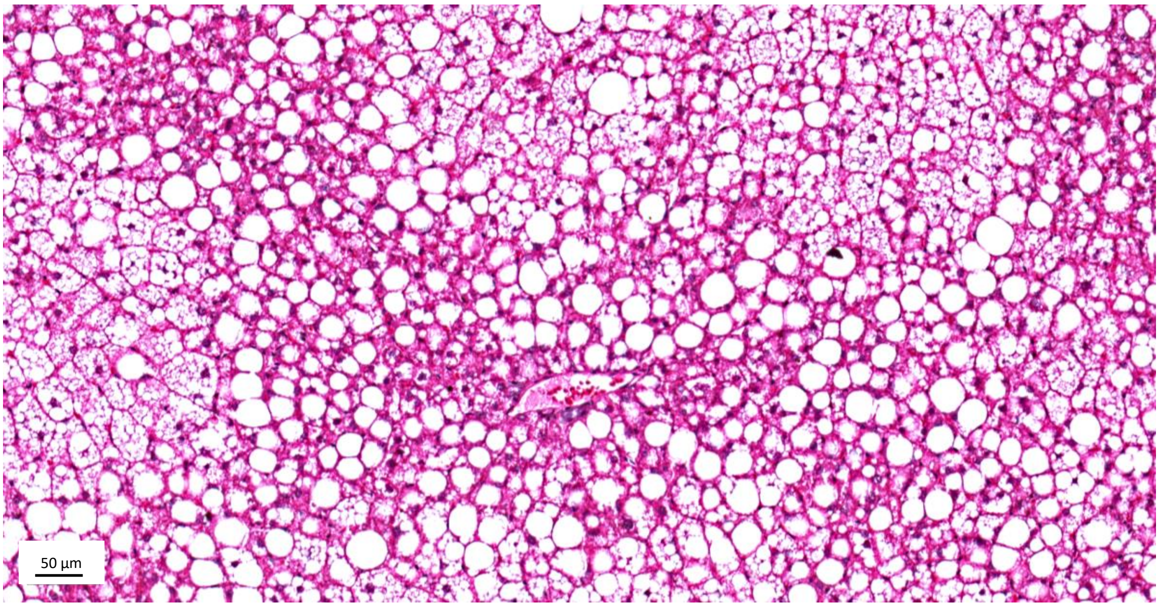

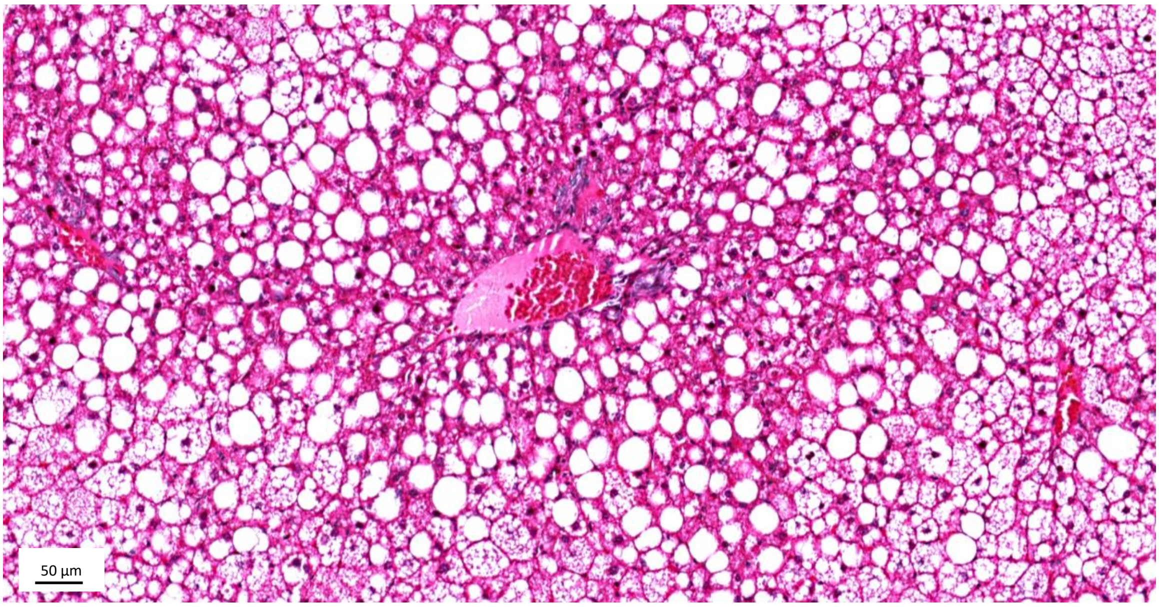

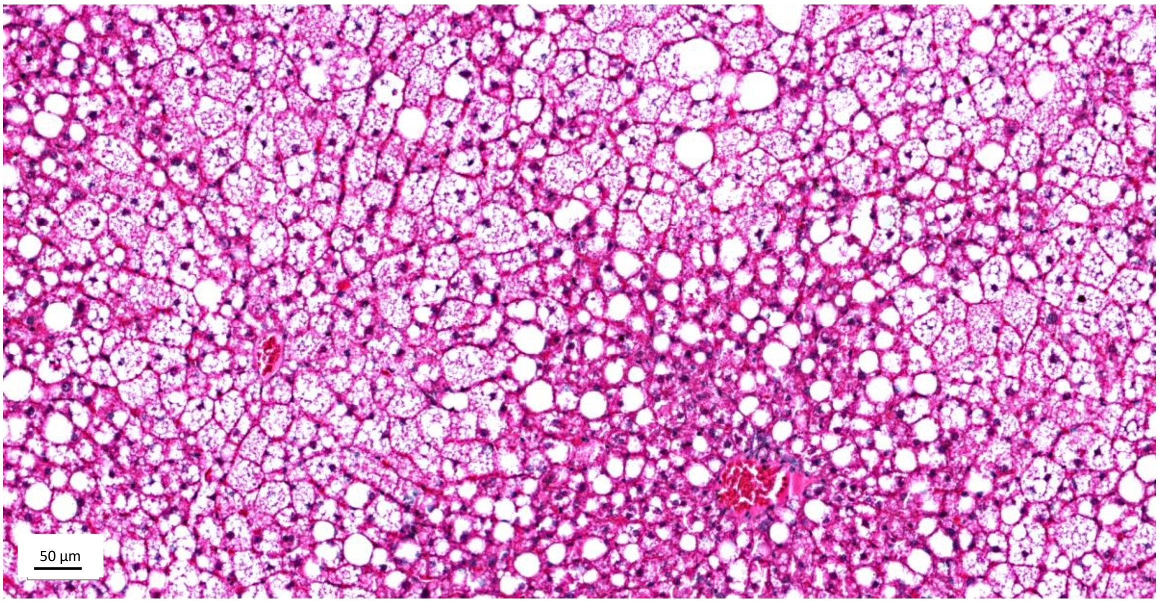

HFHCD-8

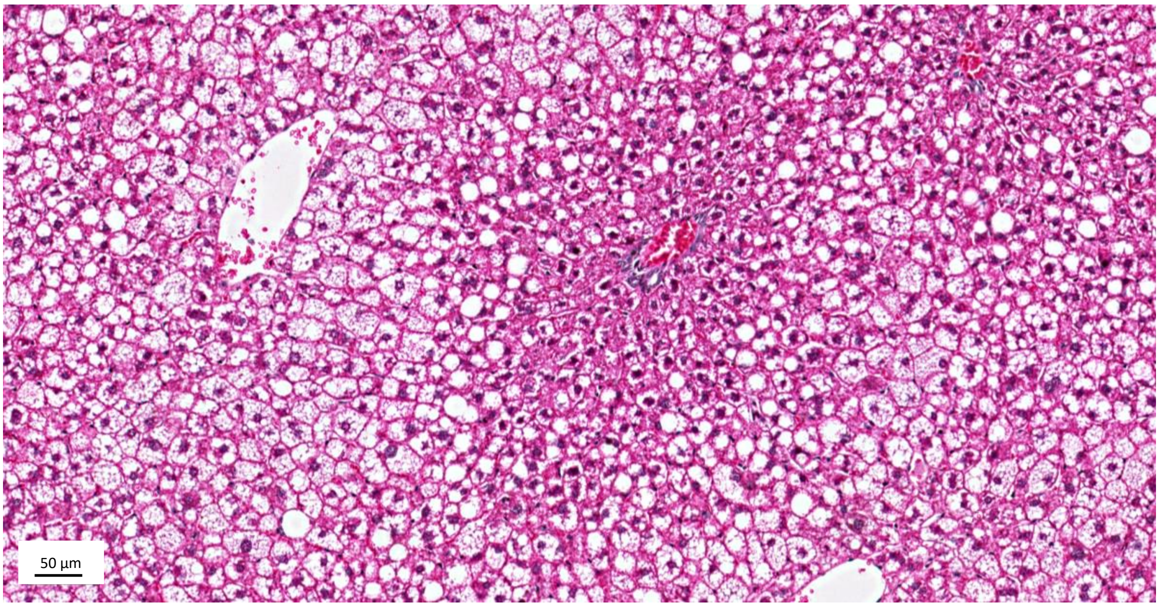

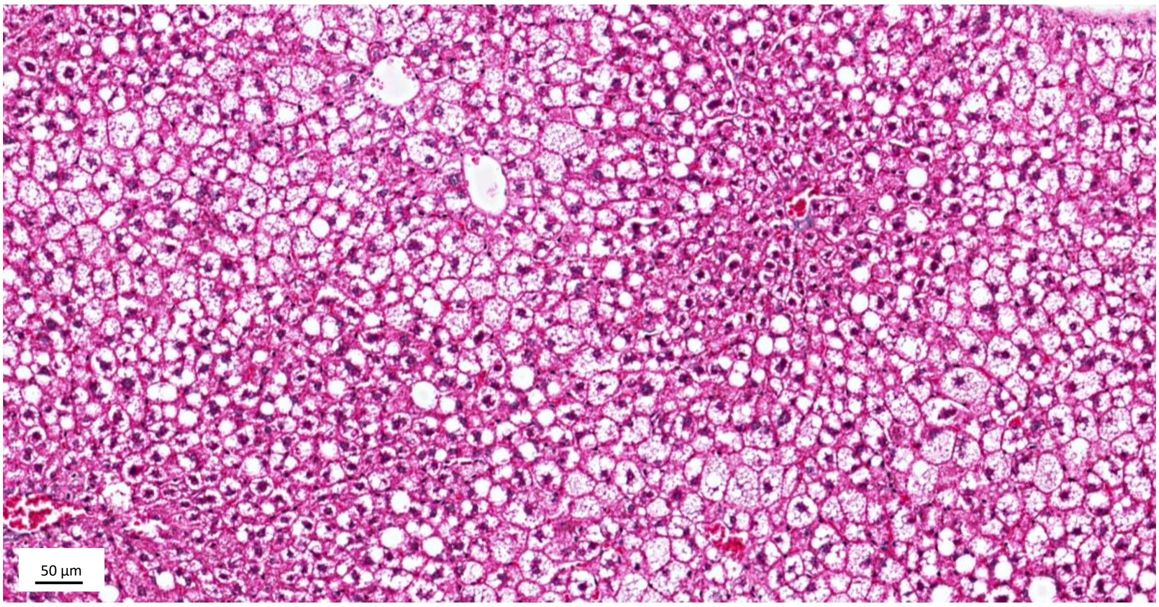

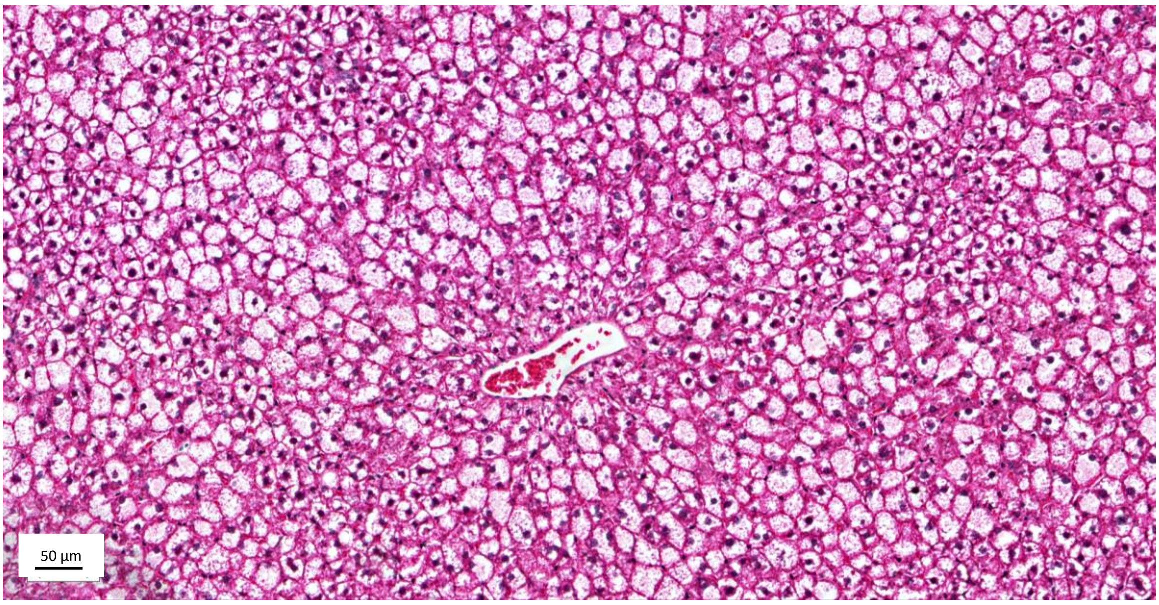

HFHCD-9

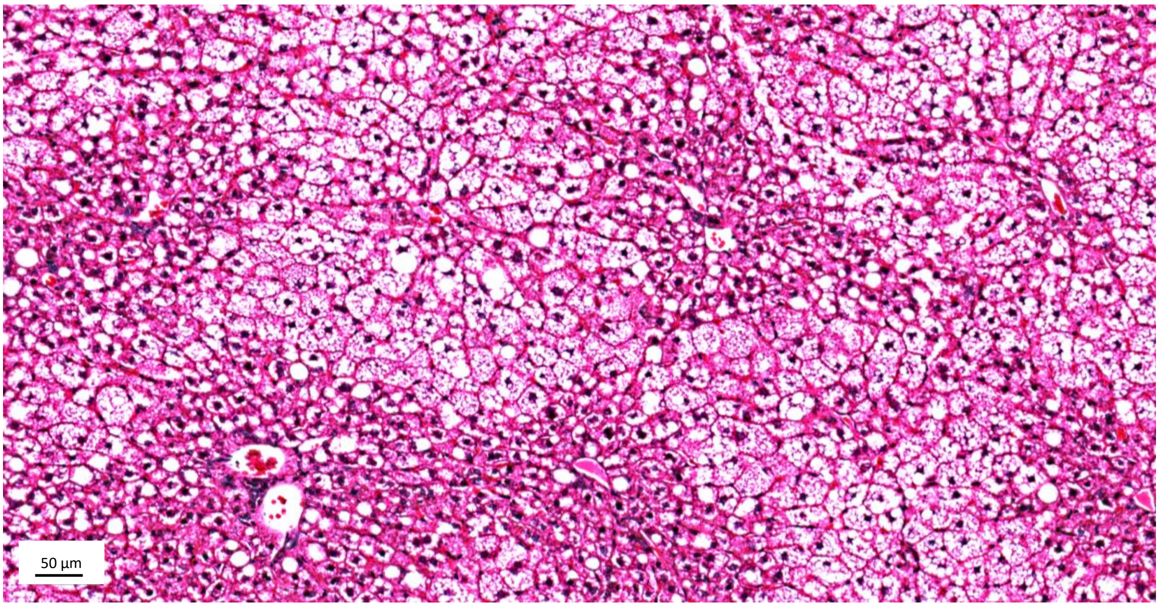

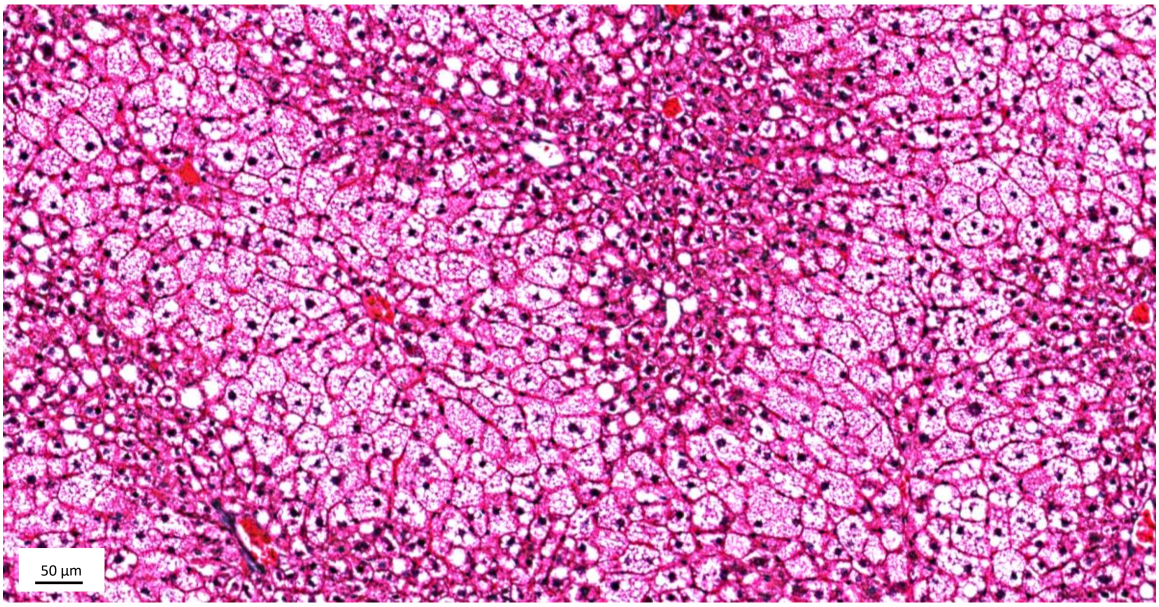

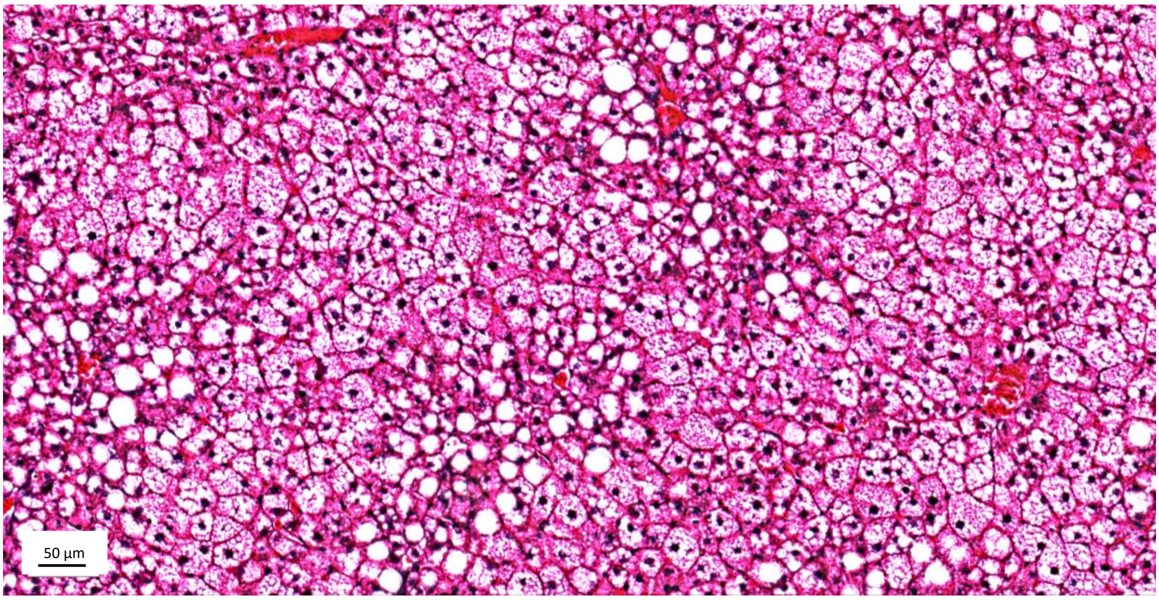

HFHCD-10

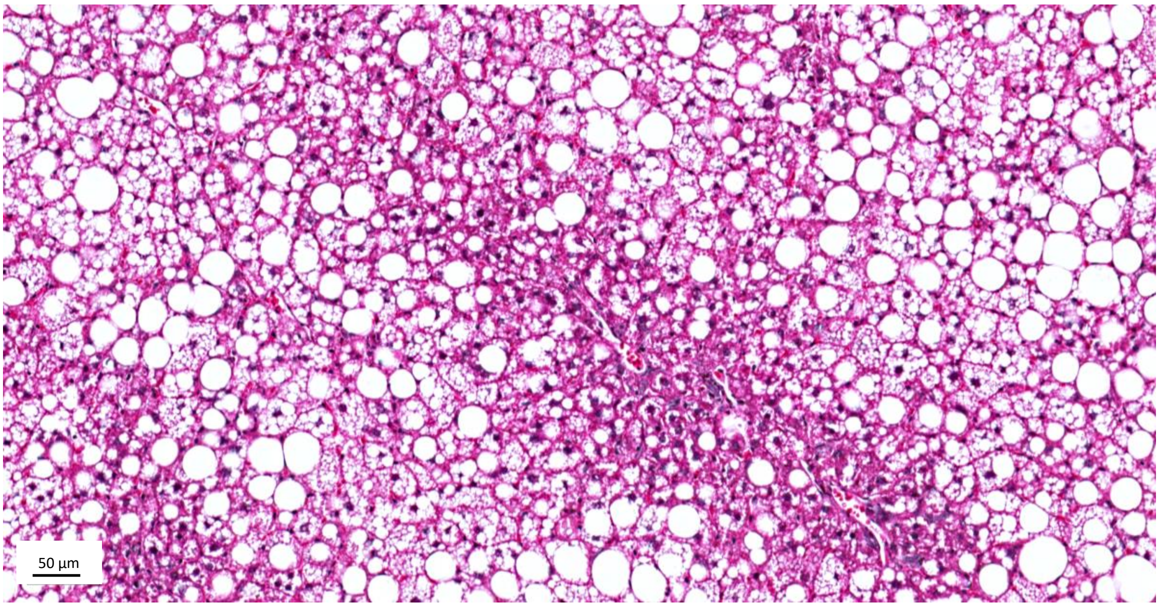

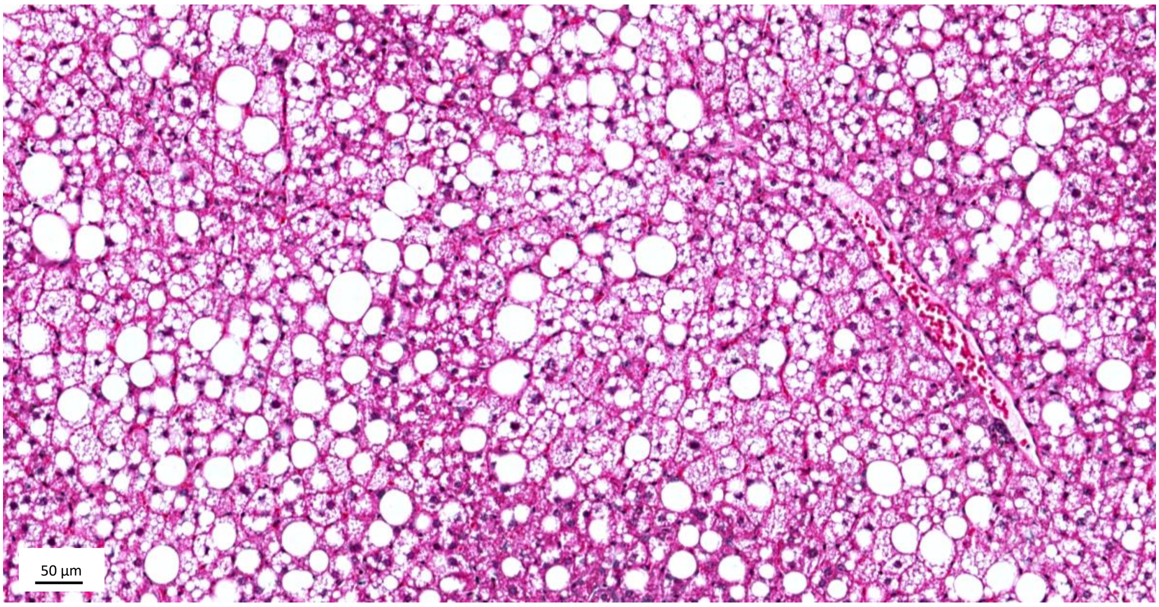

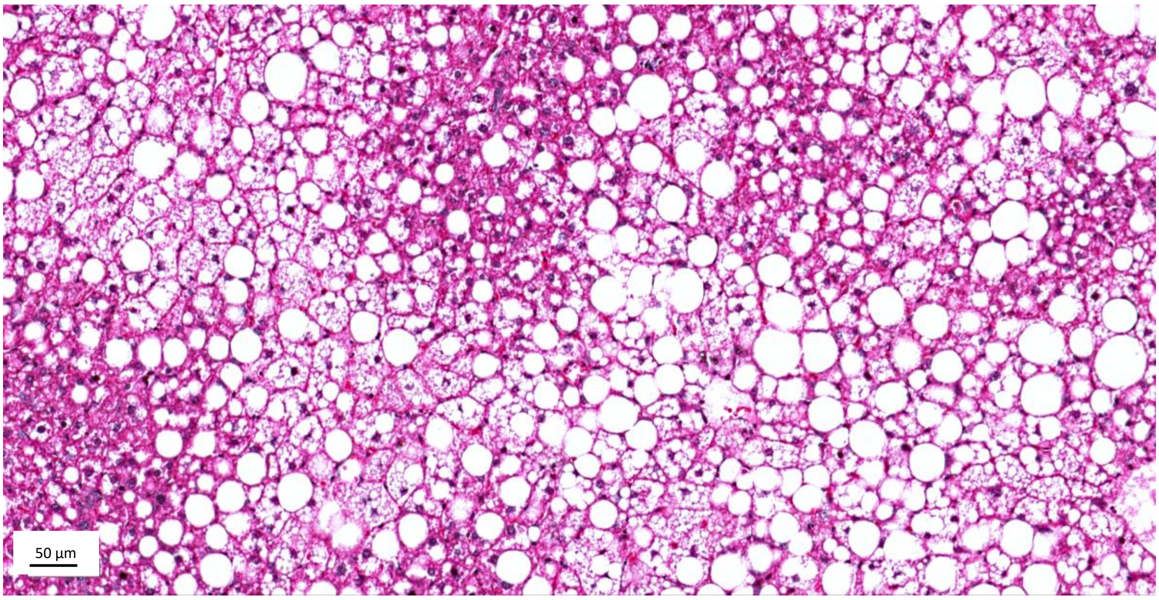

HFHCD-11

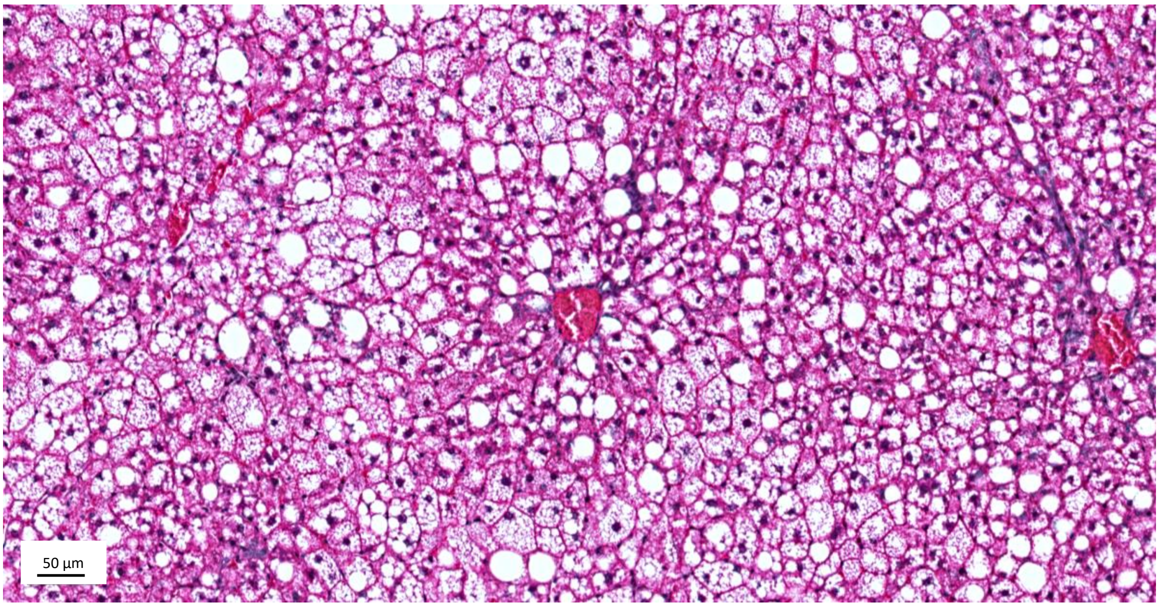

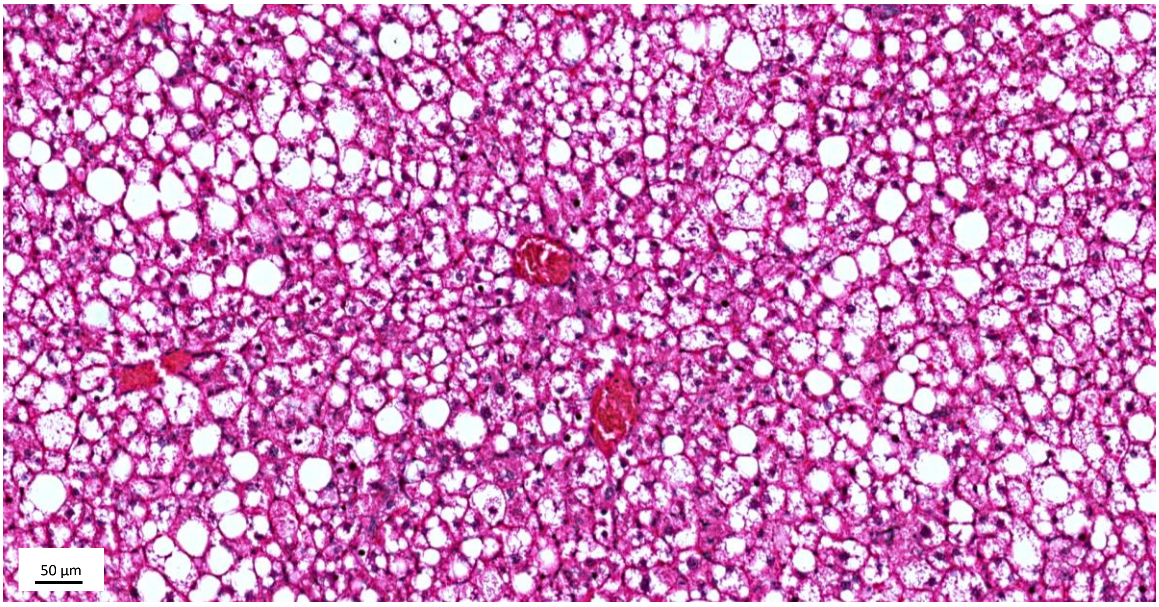

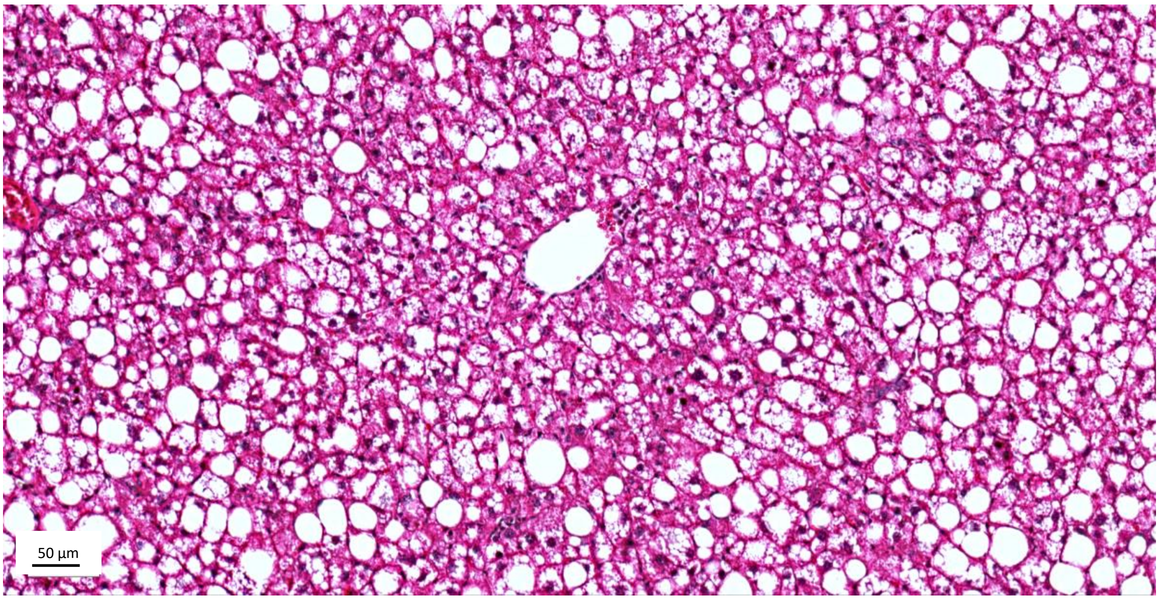

HFHCD-12

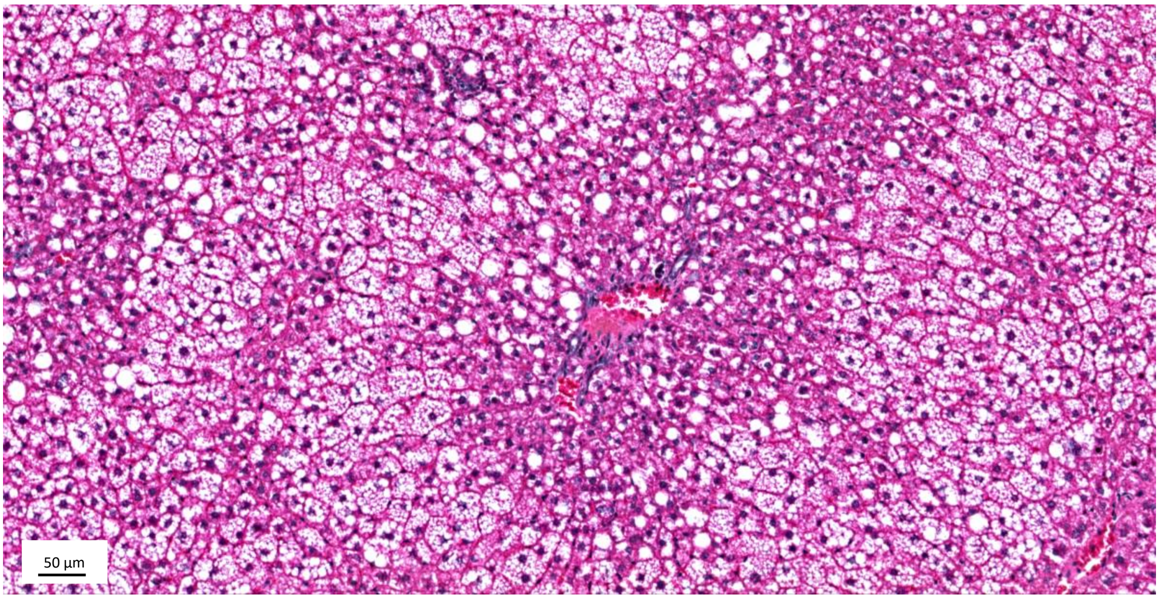

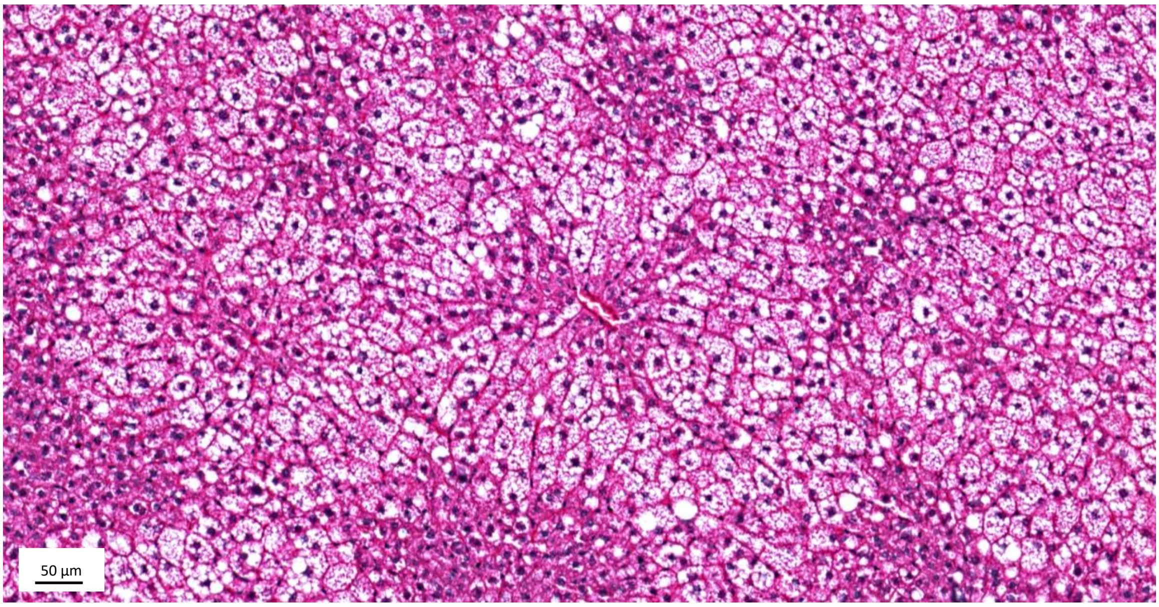

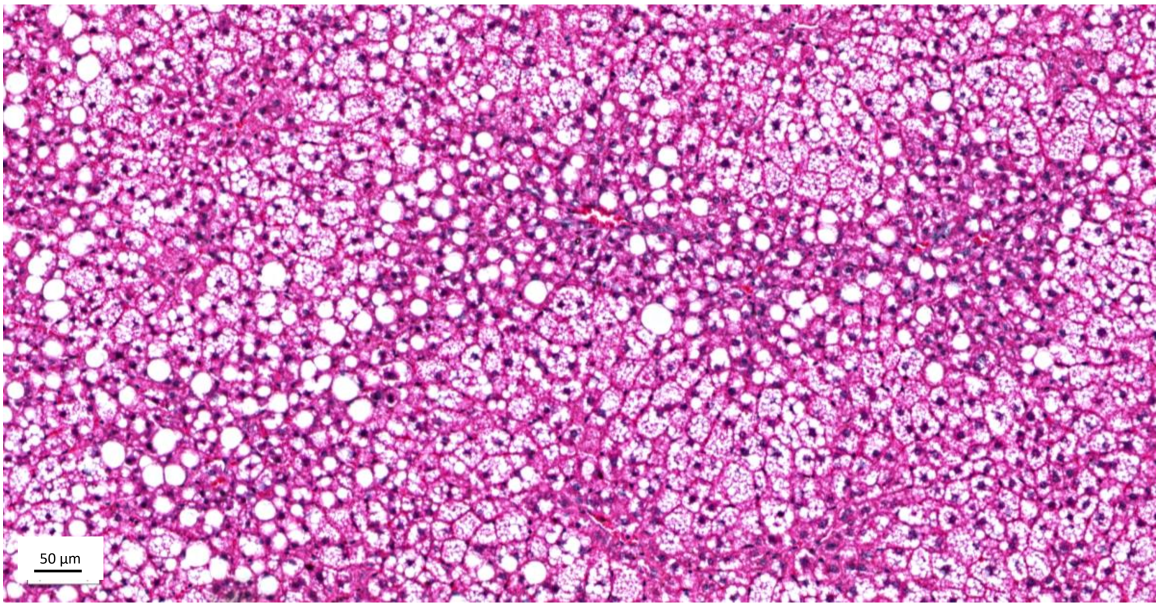

HFHCD-13

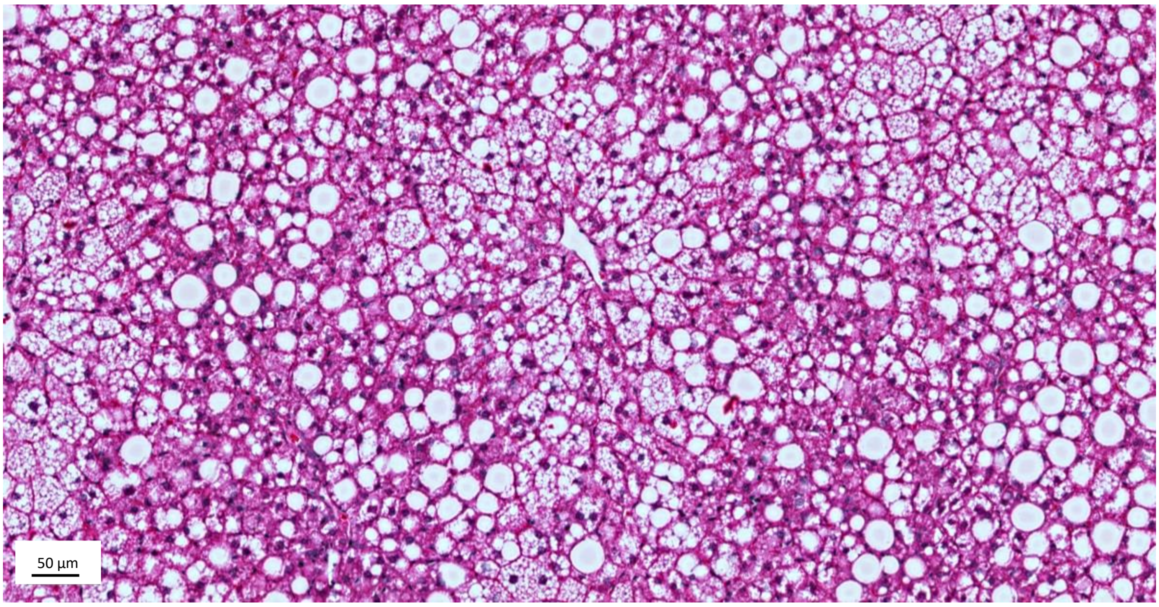

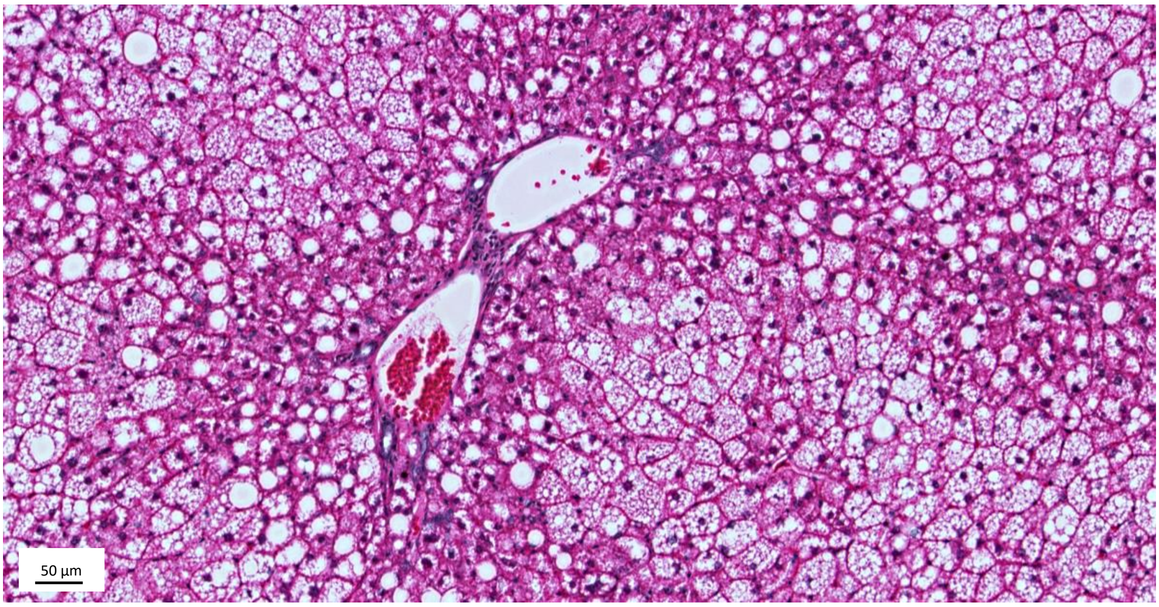

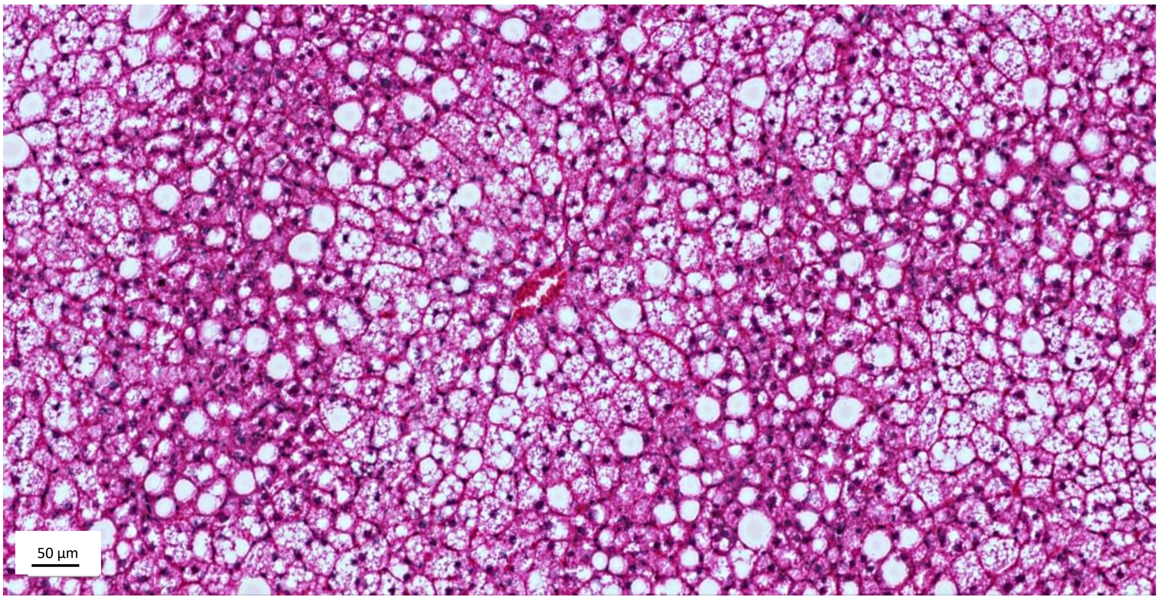

HFHCD-14

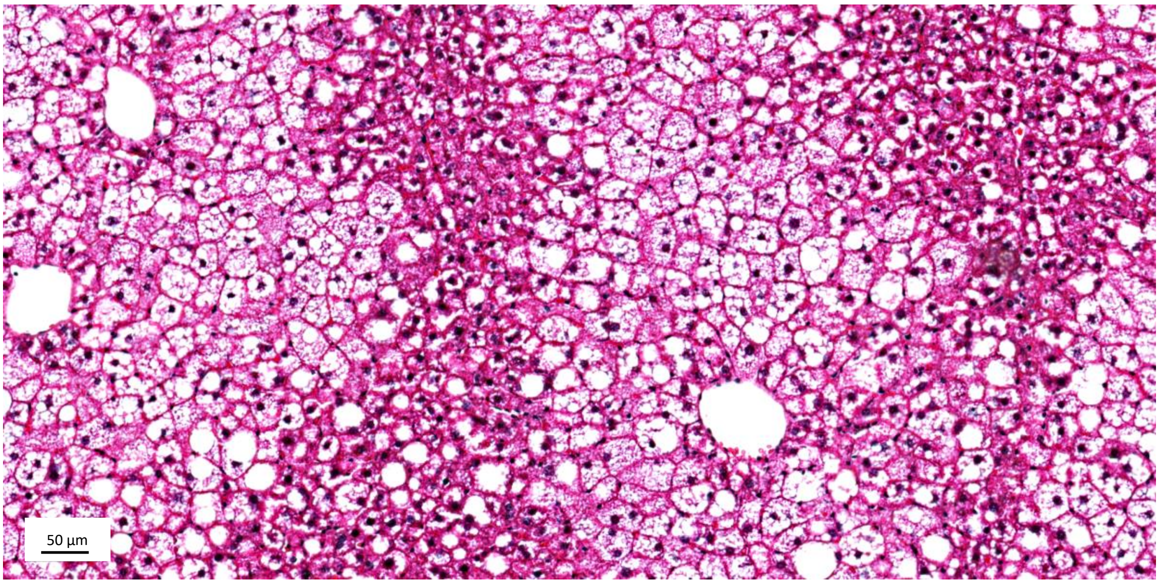

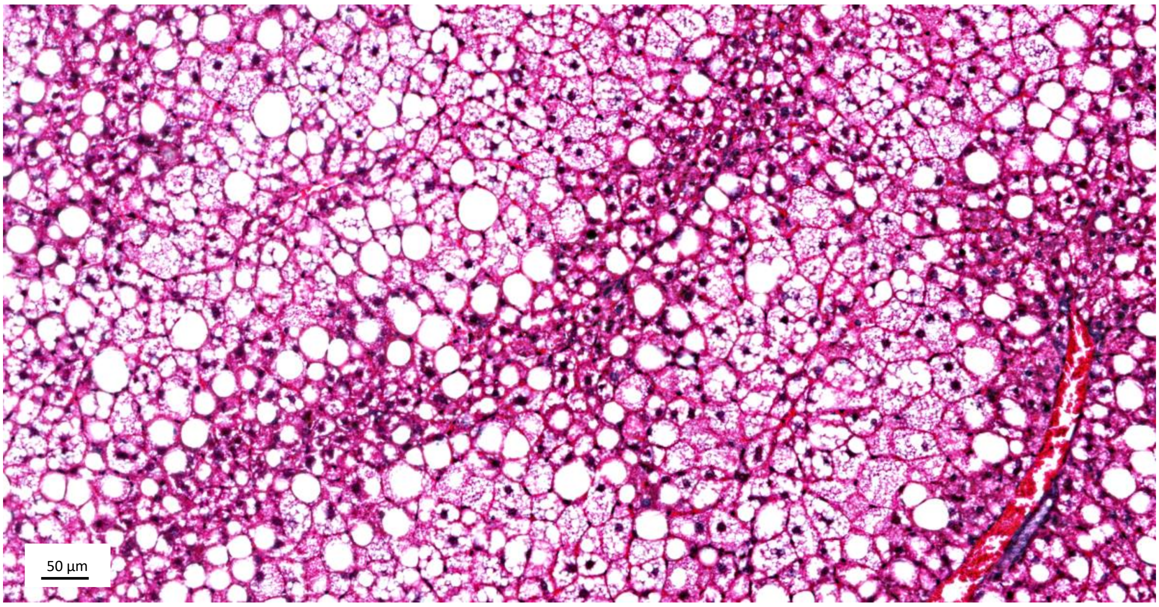

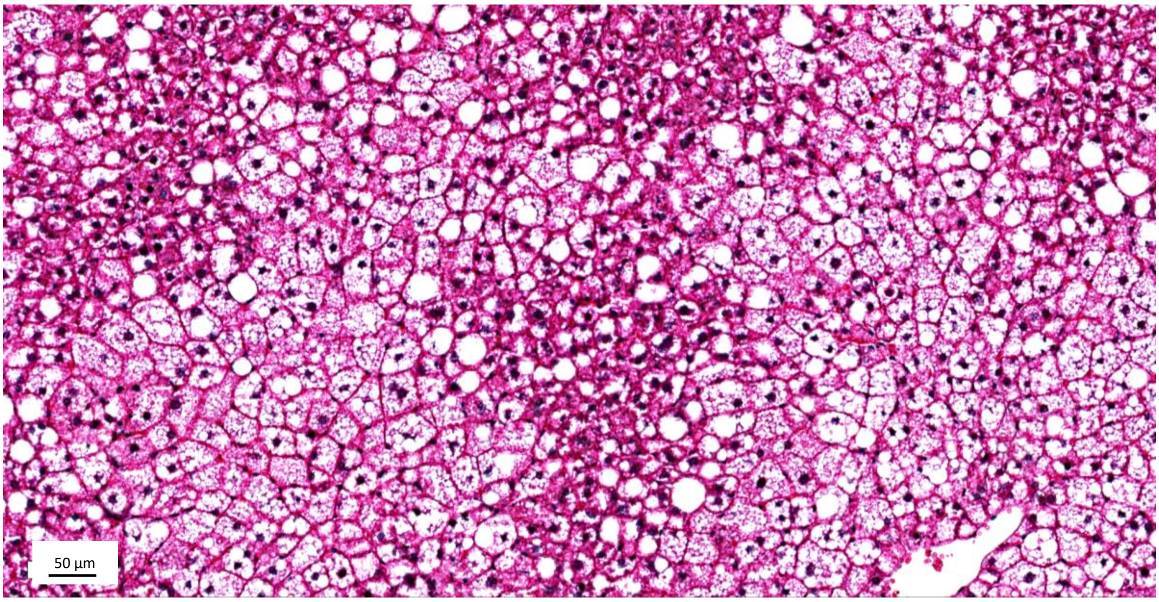

HFHCD-15

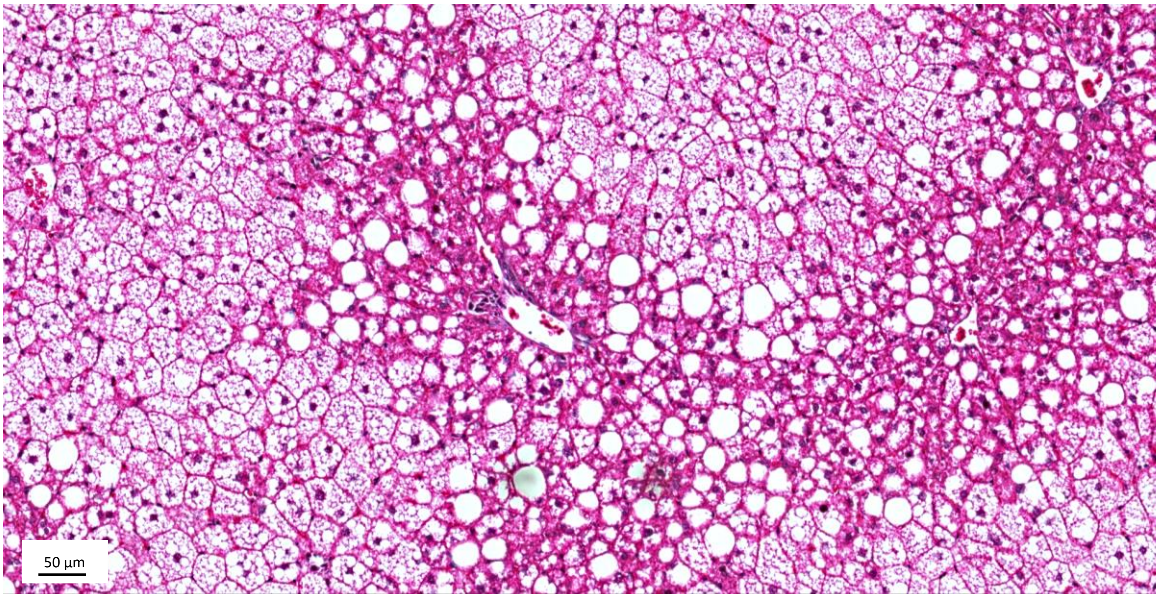

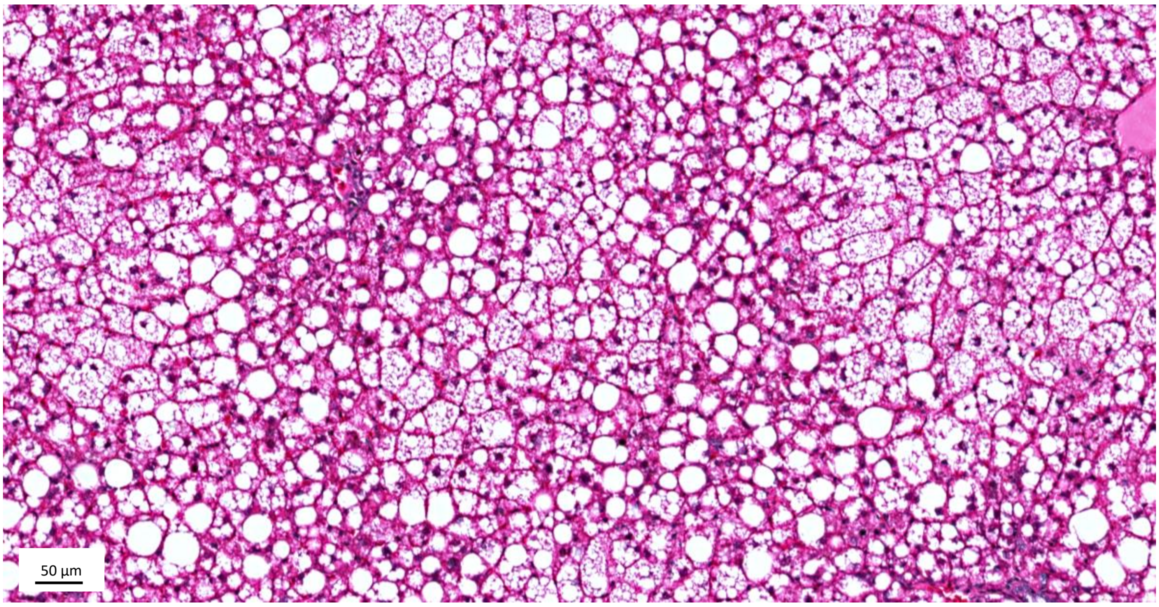

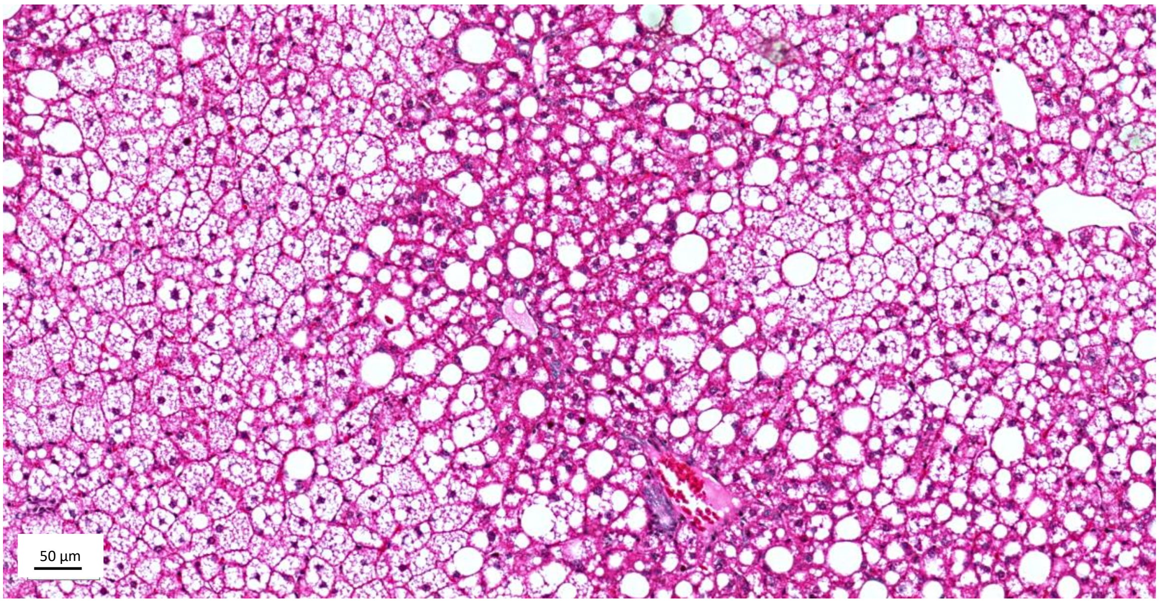

H&E Staining

HFHCD-I group

(12 mice were included)

HFHCD-I-1

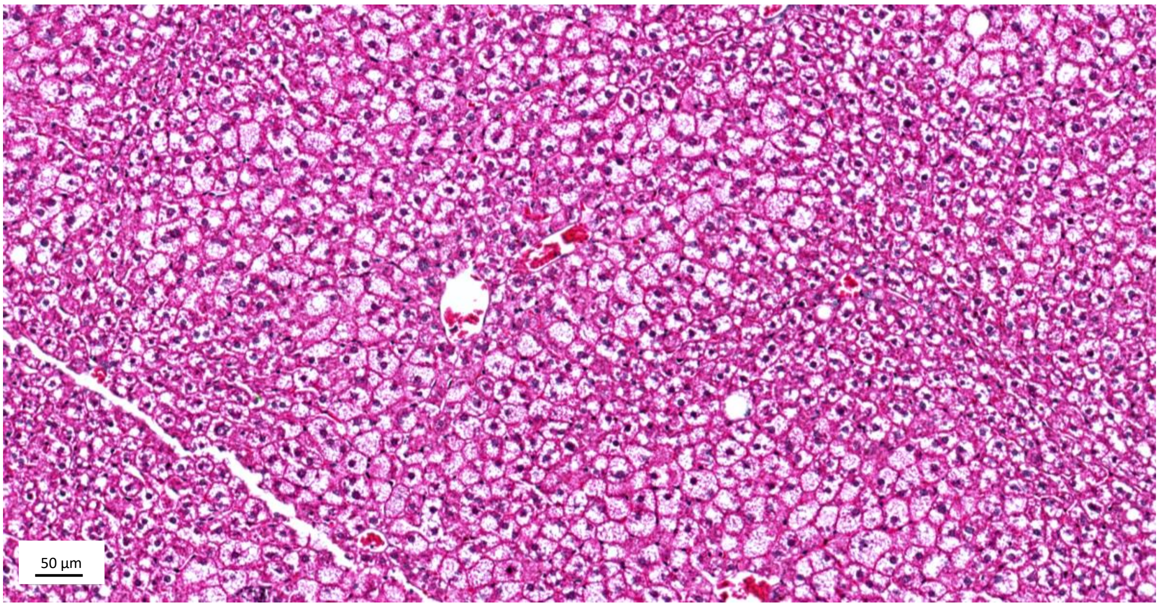

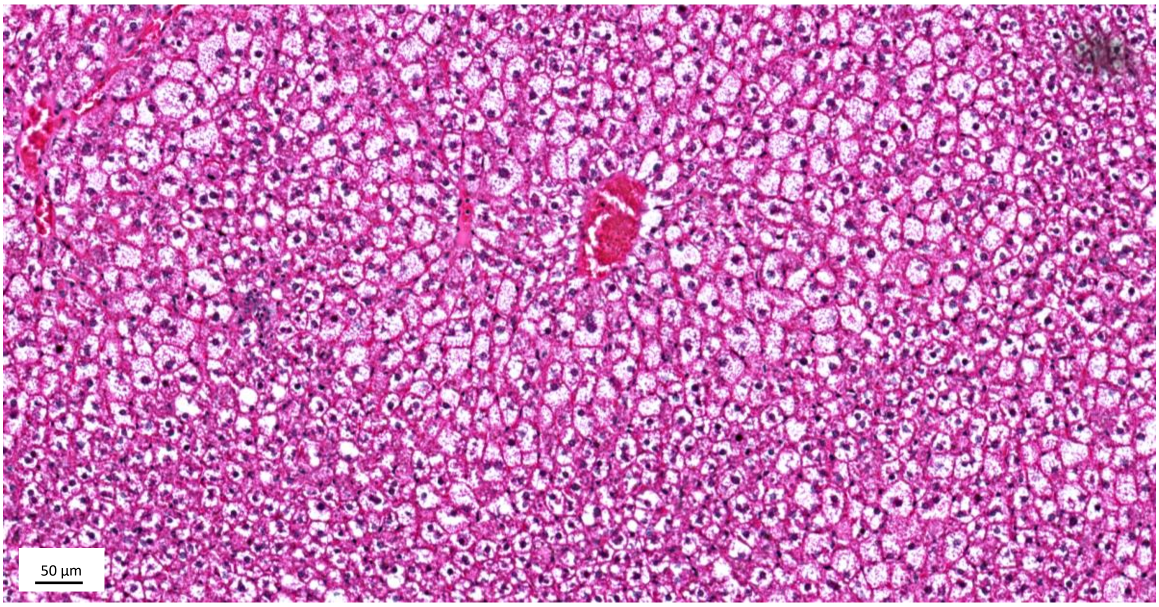

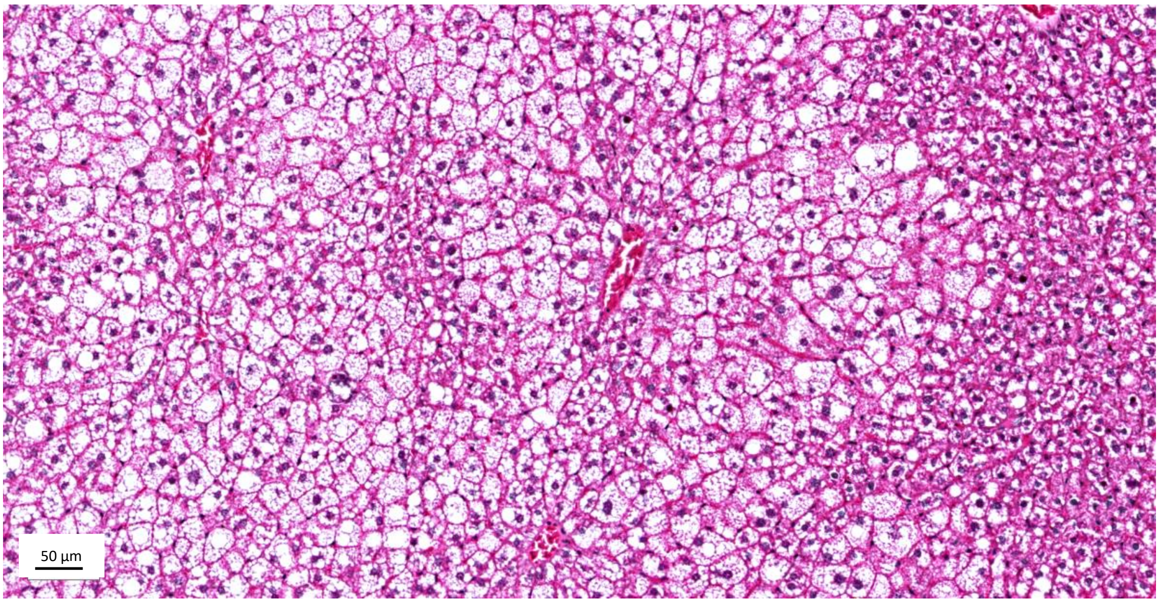

HFHCD-I-2

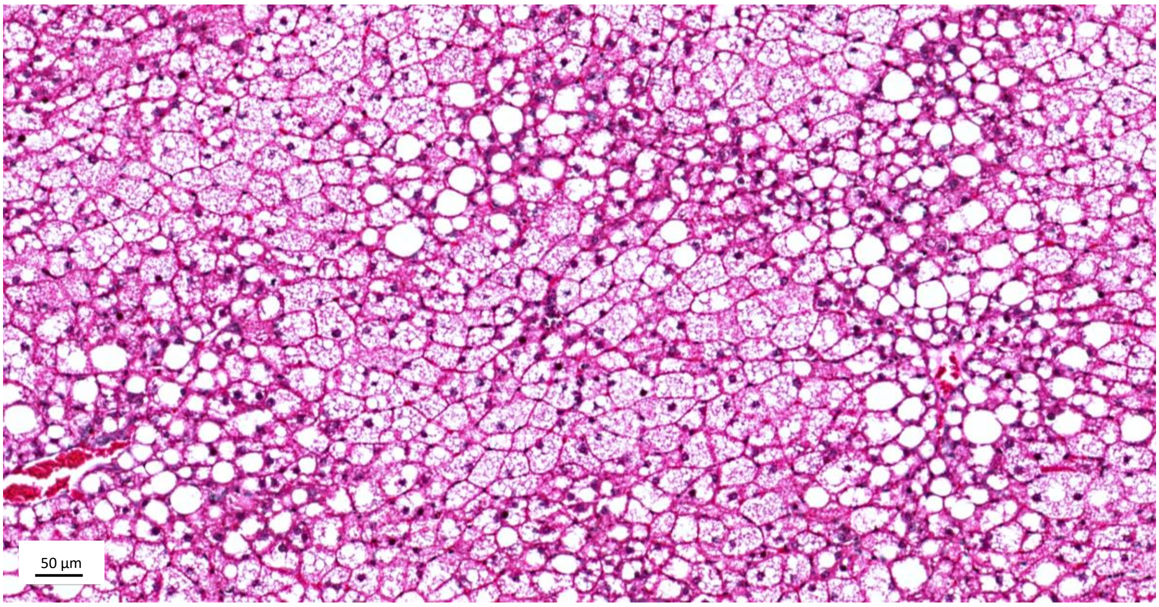

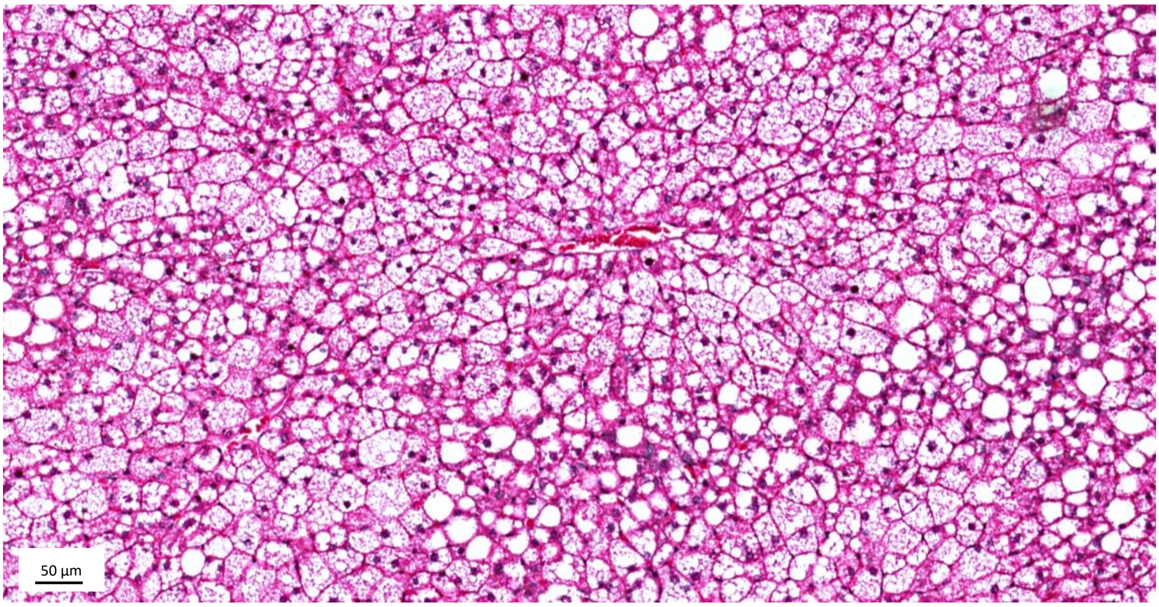

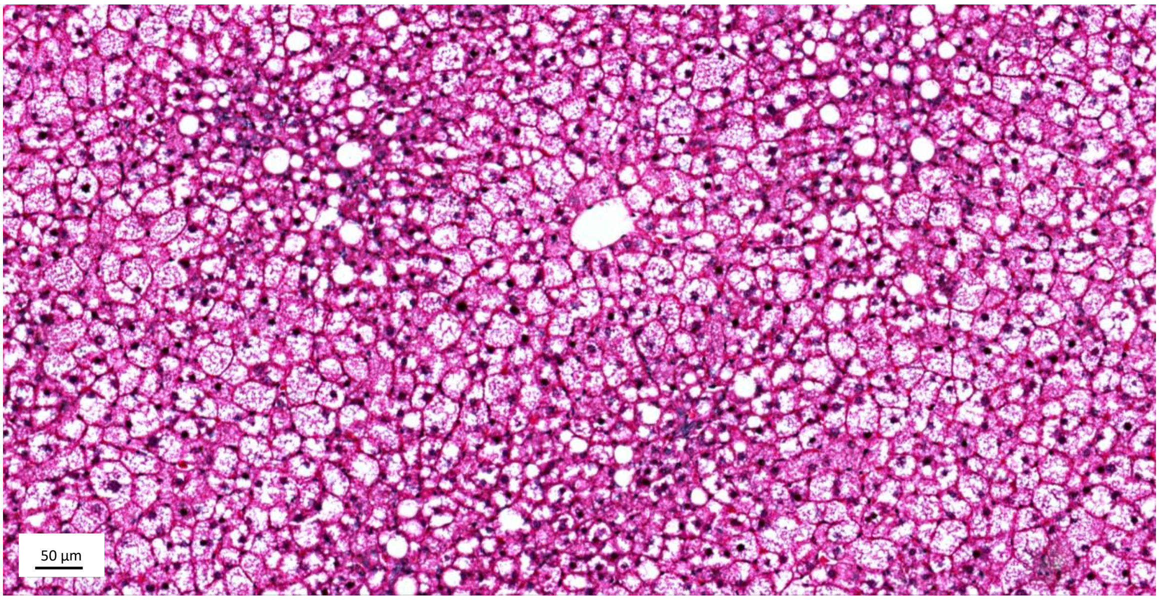

HFHCD-I-3

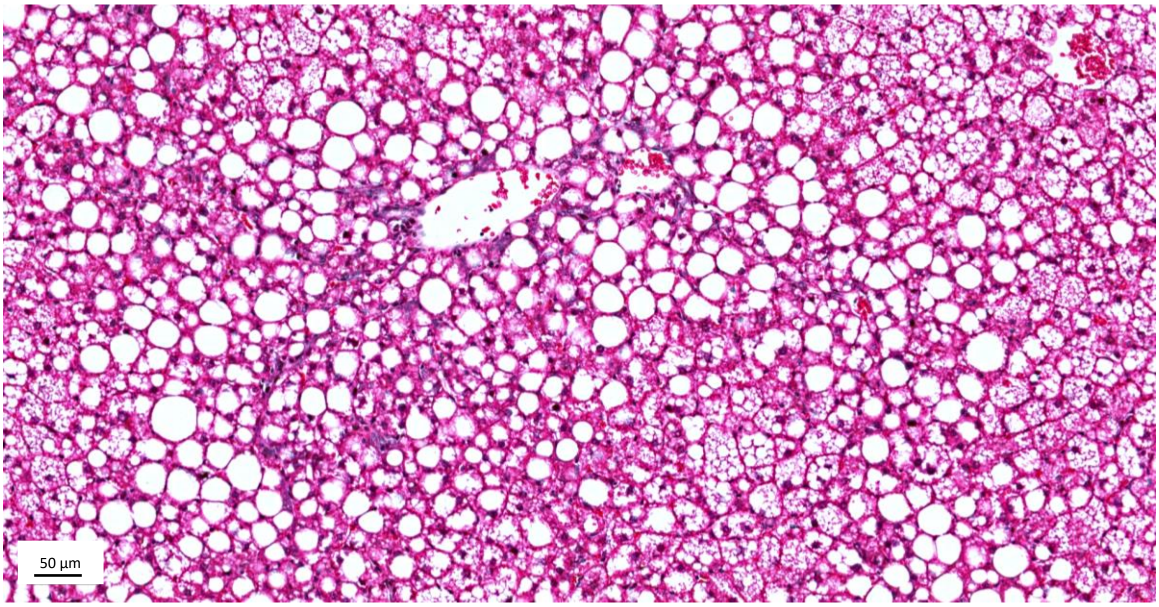

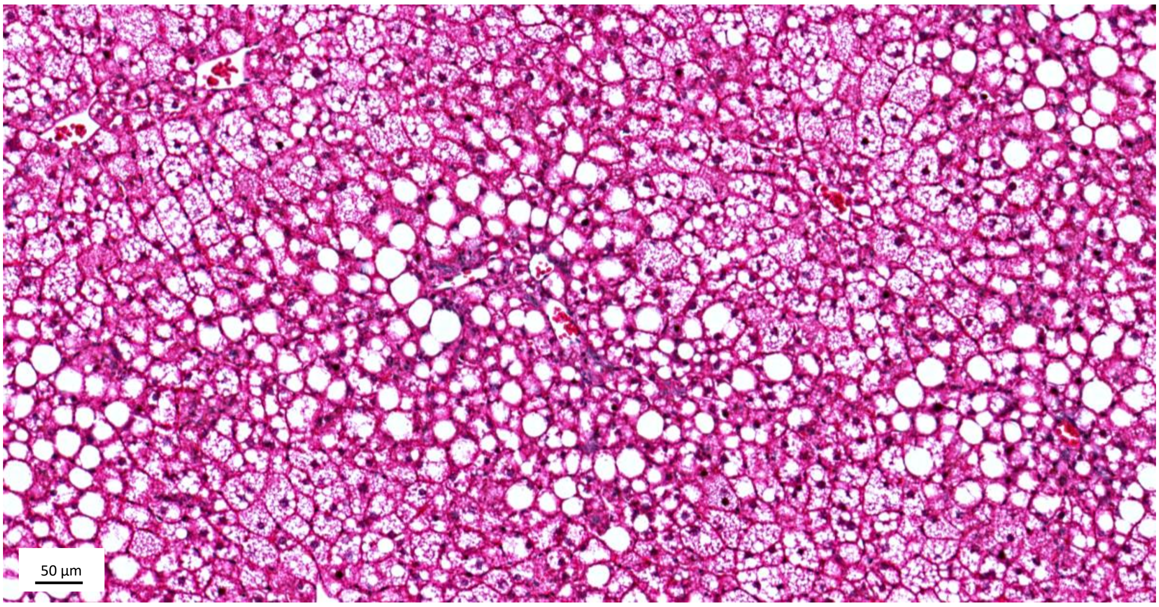

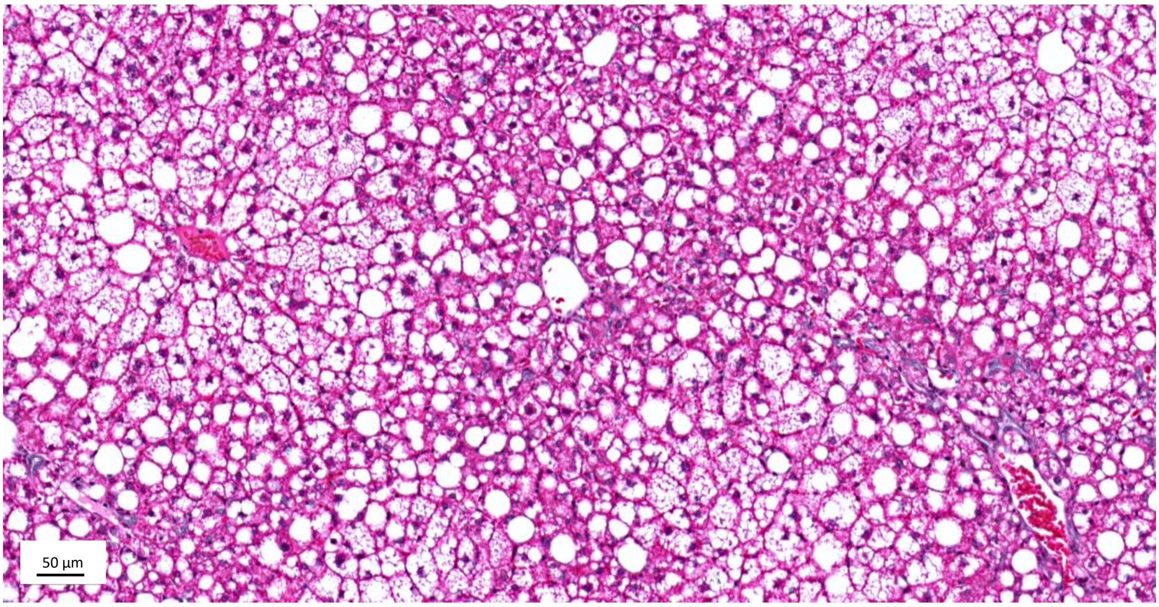

HFHCD-I-4

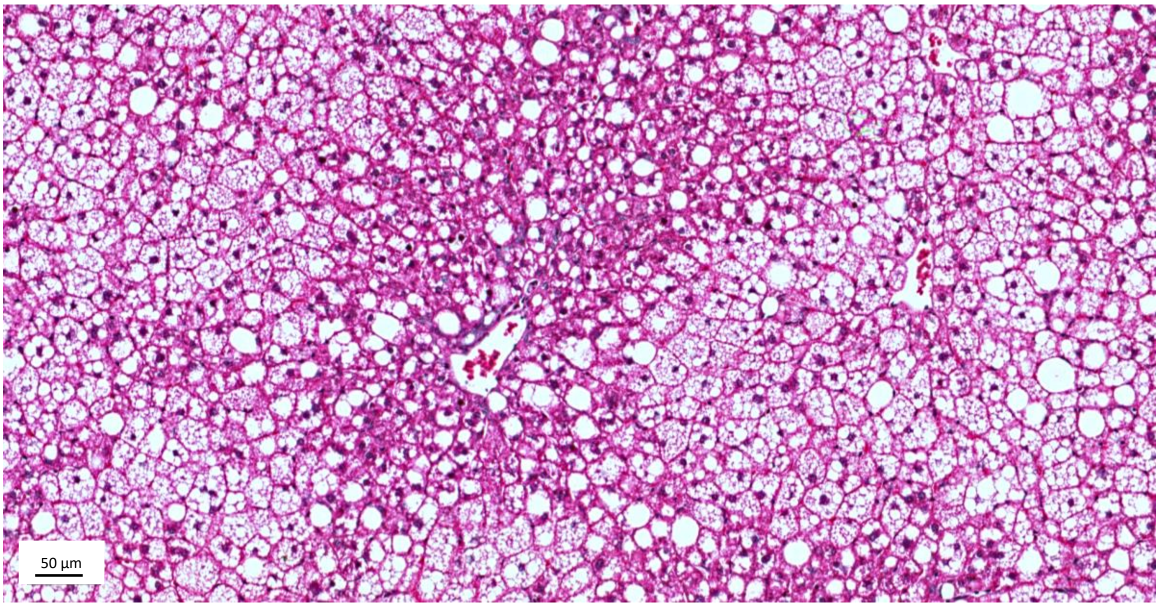

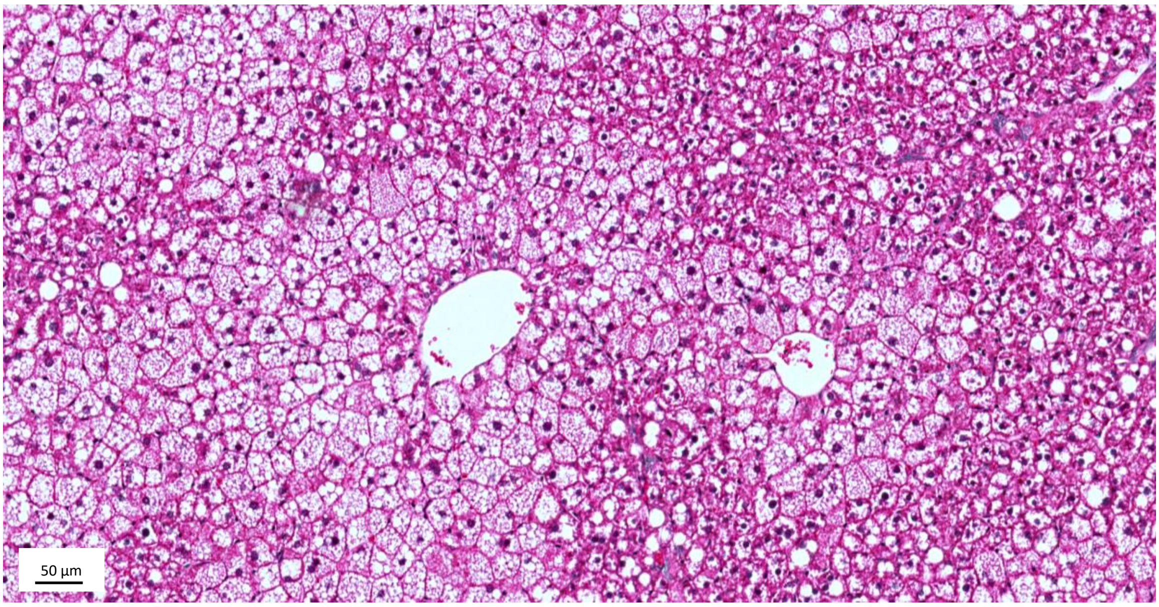

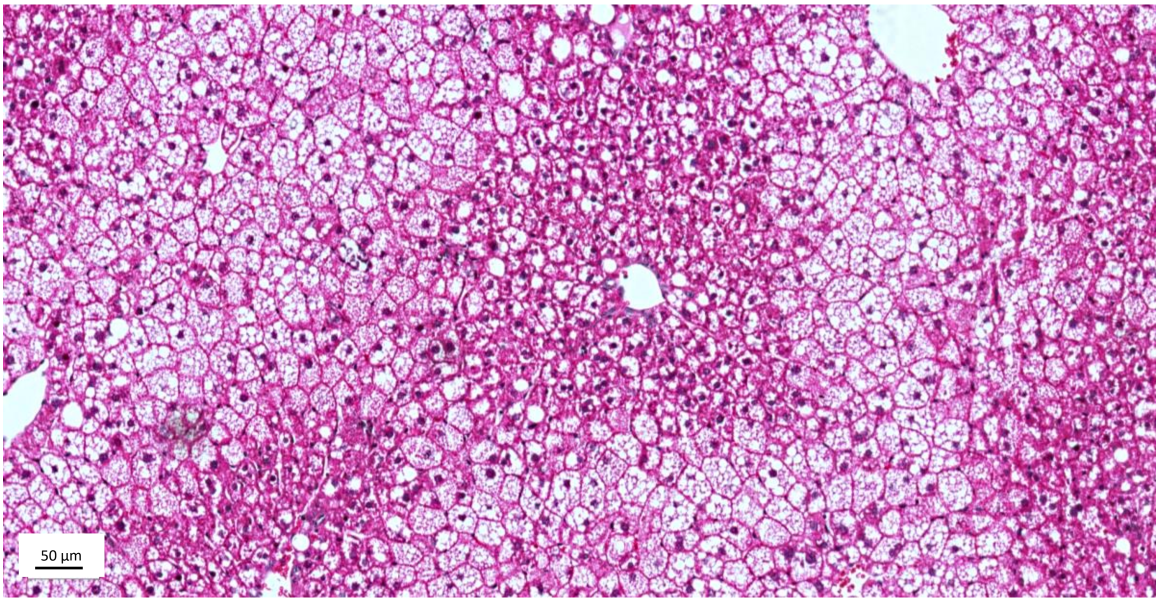

HFHCD-I-5

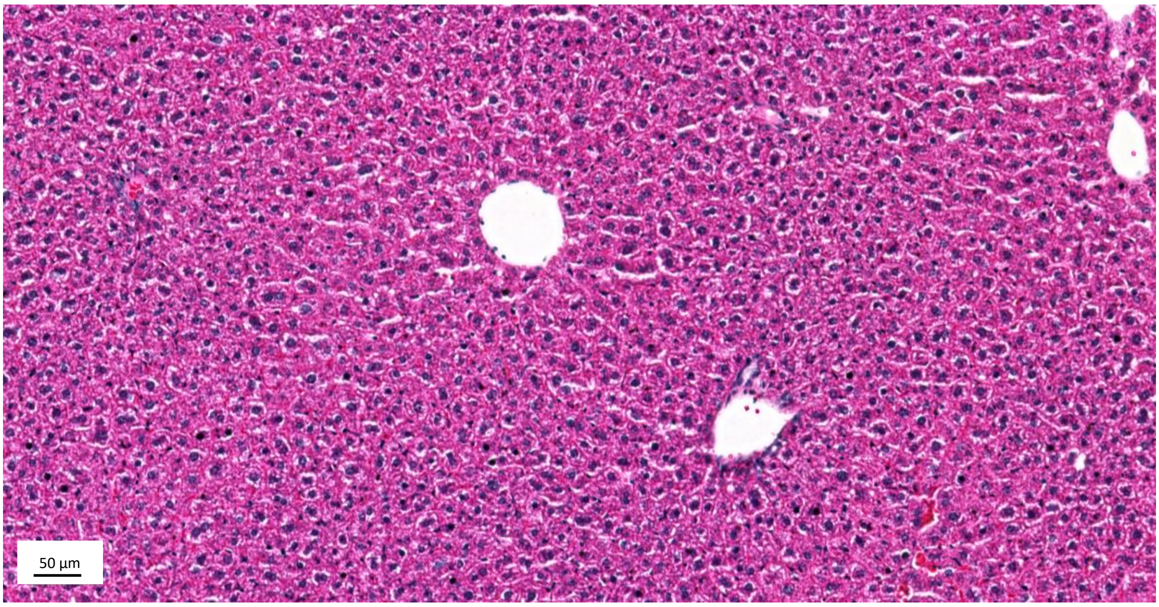

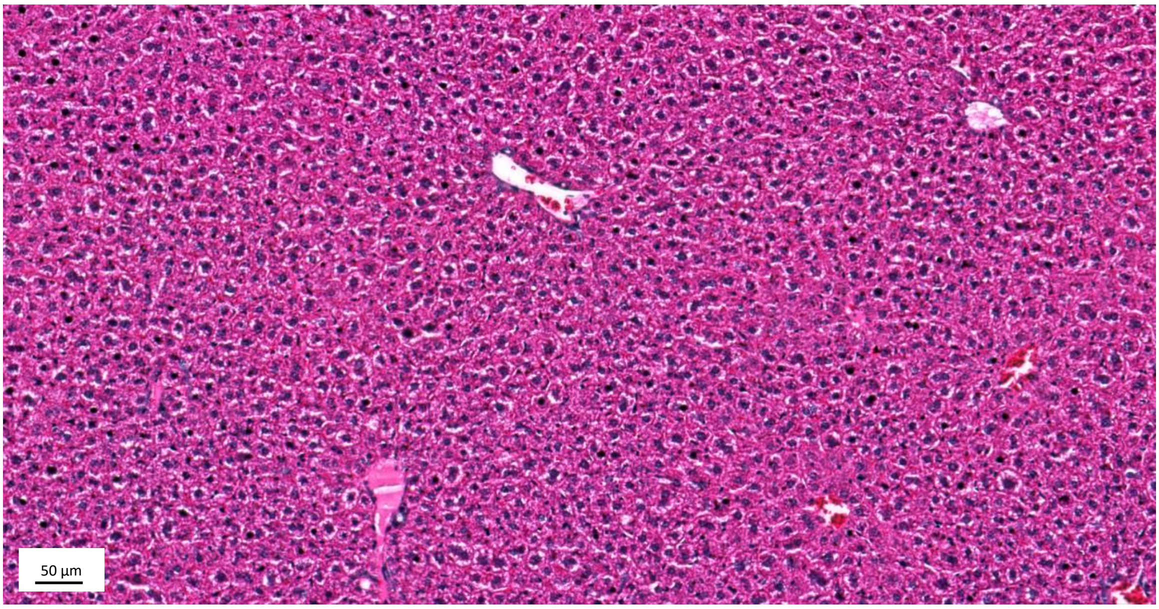

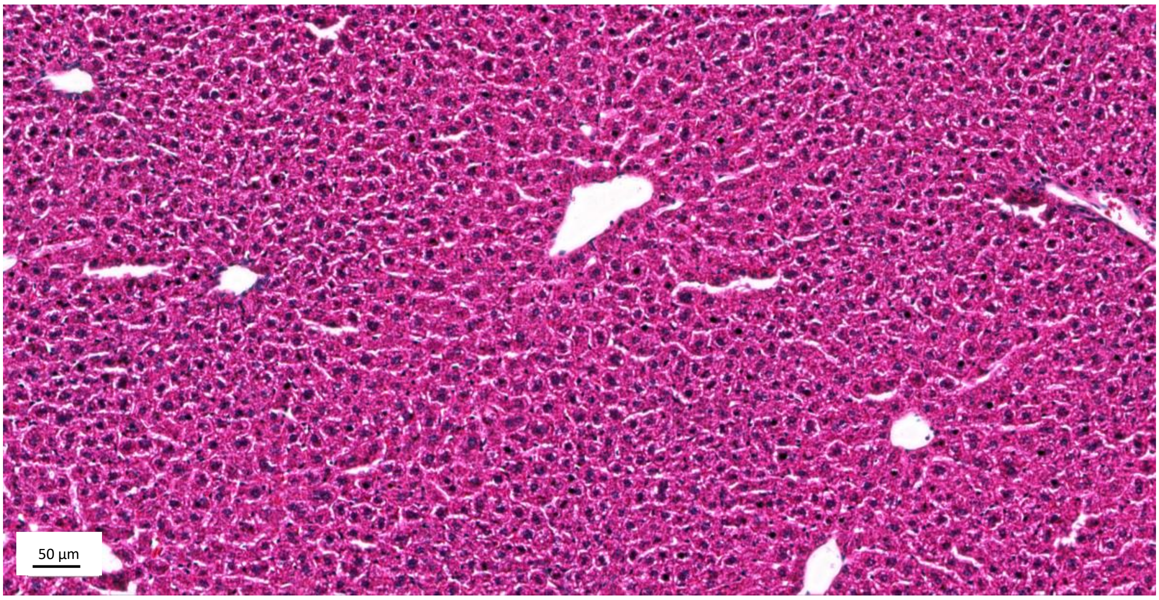

HFHCD-I-6

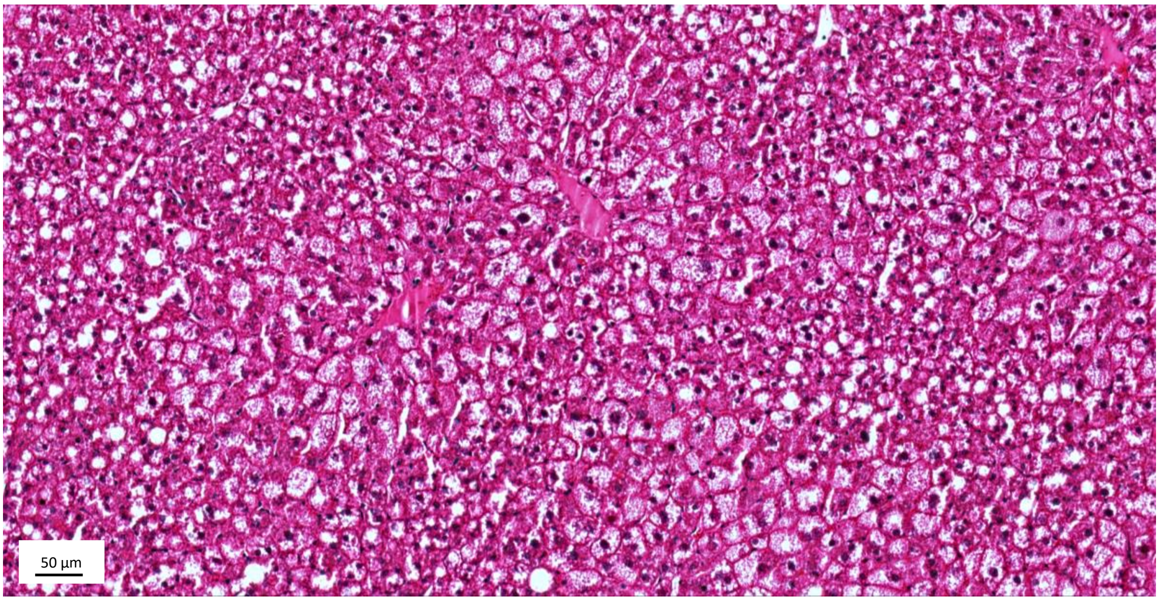

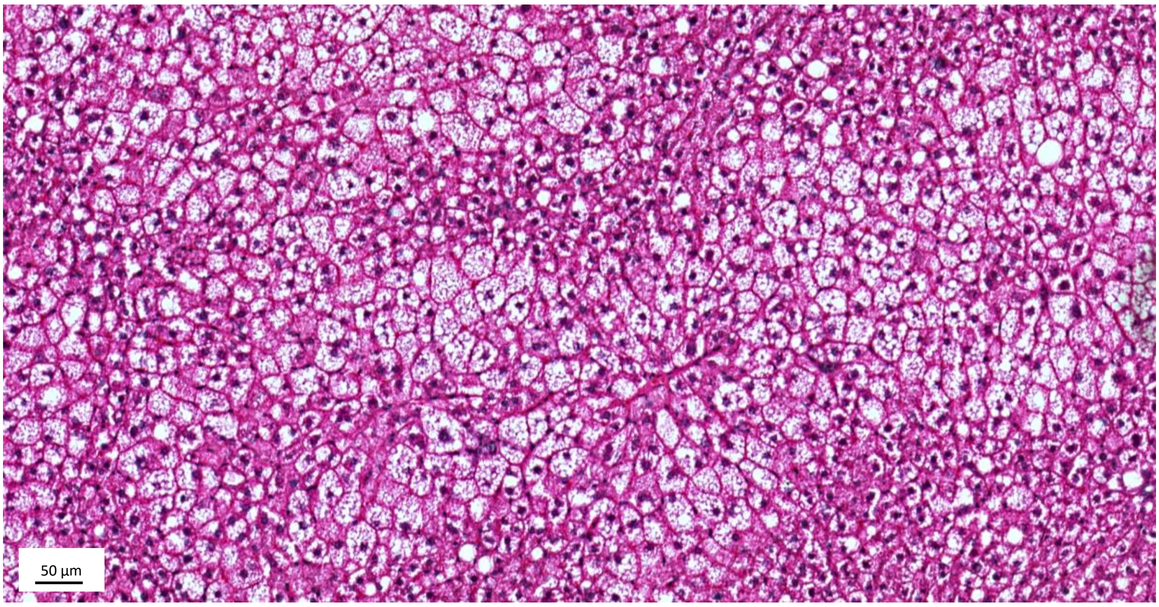

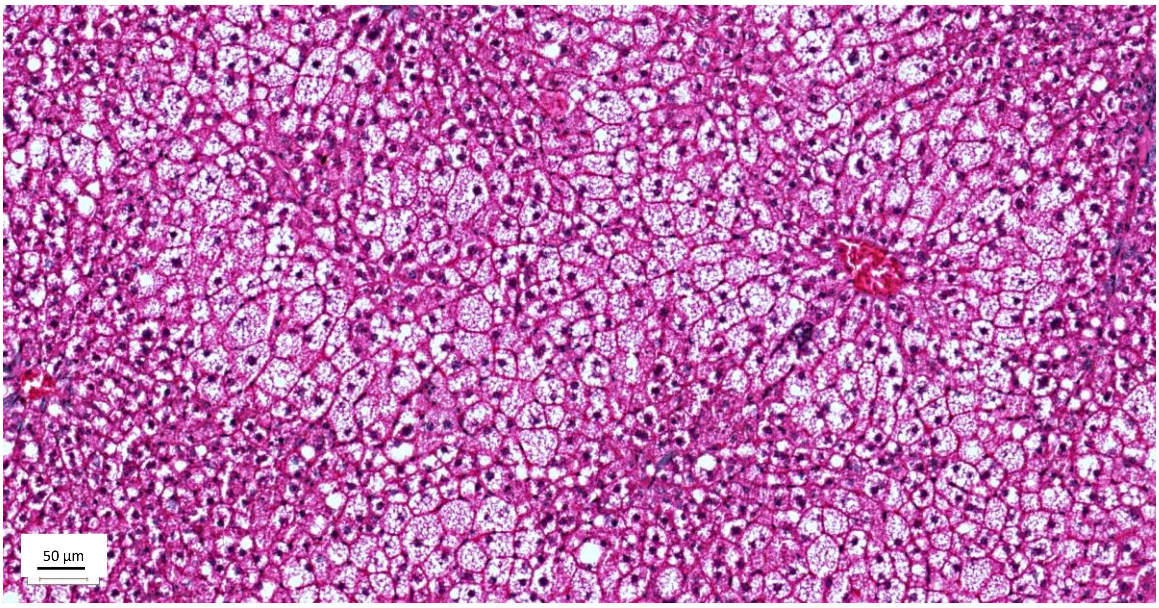

HFHCD-I-7

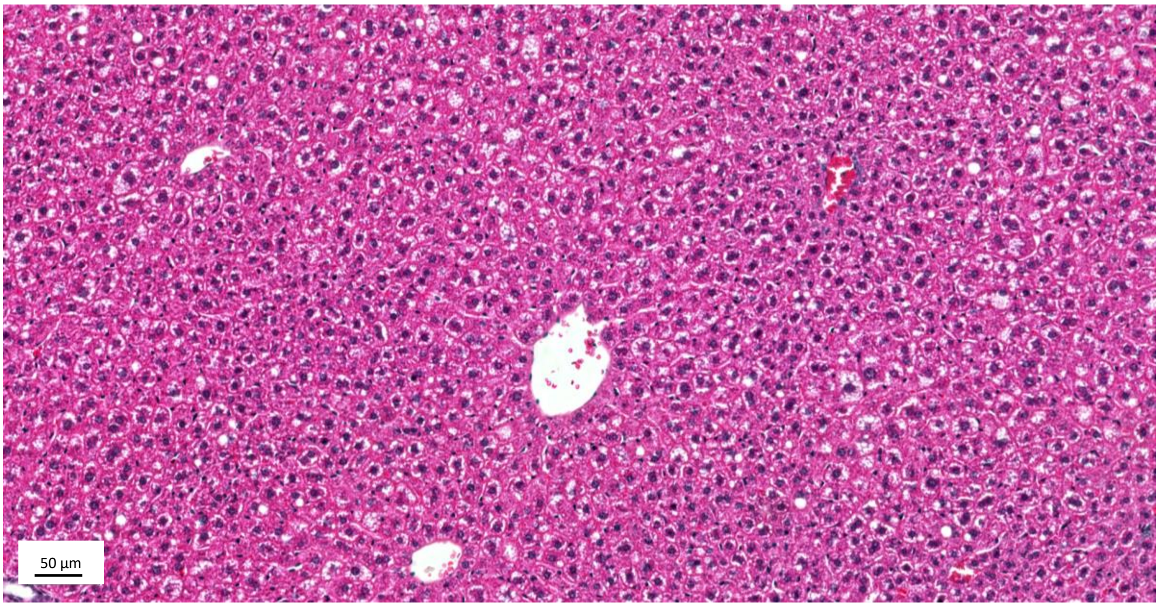

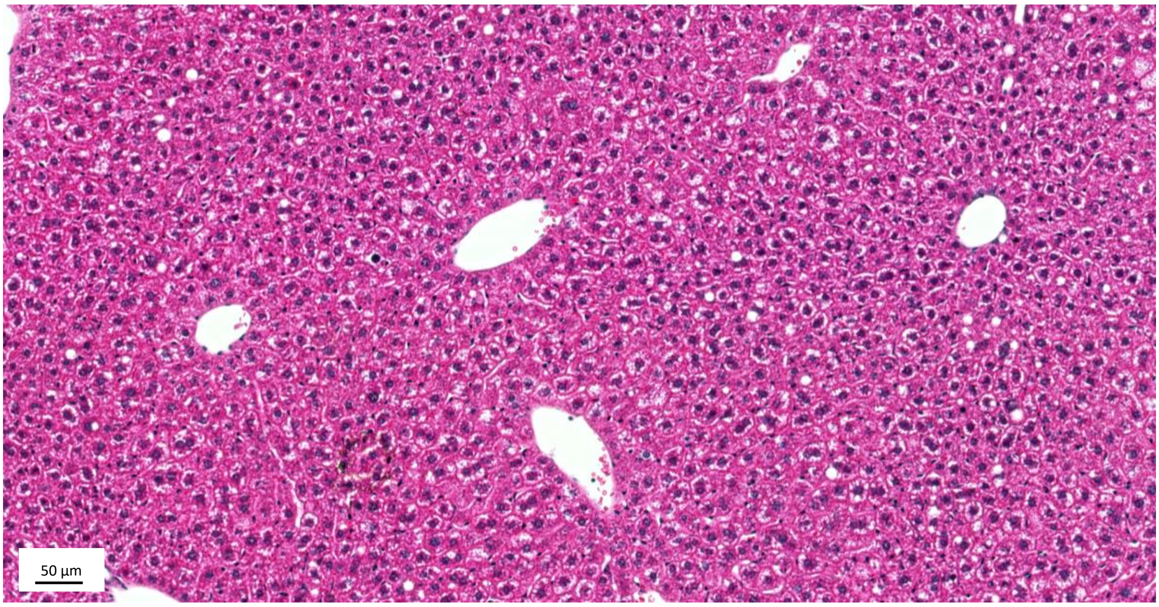

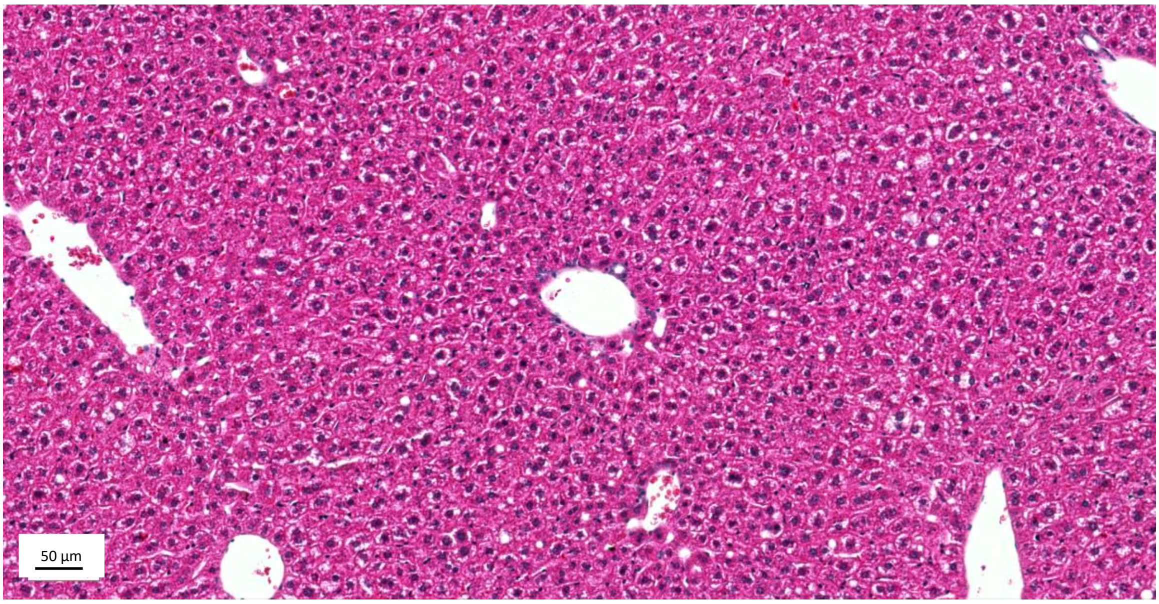

HFHCD-I-8

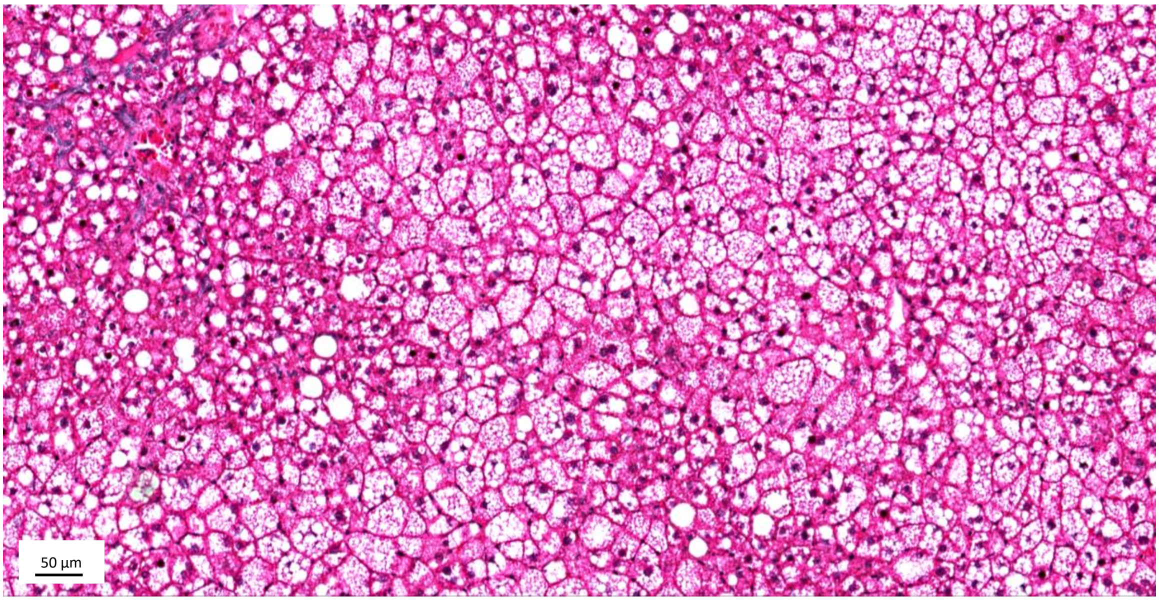

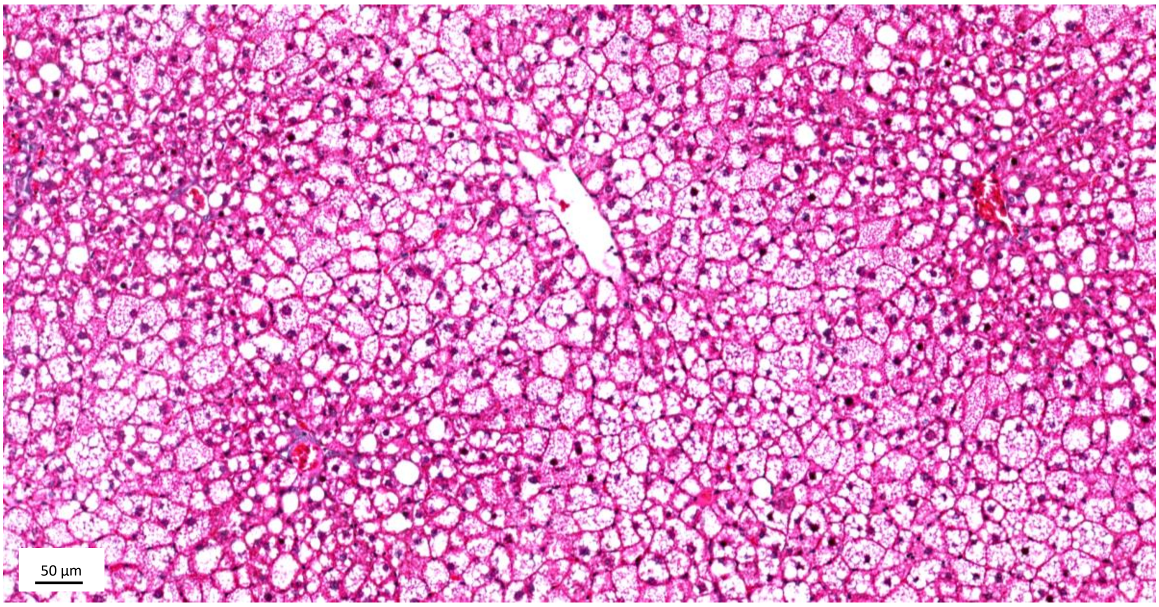

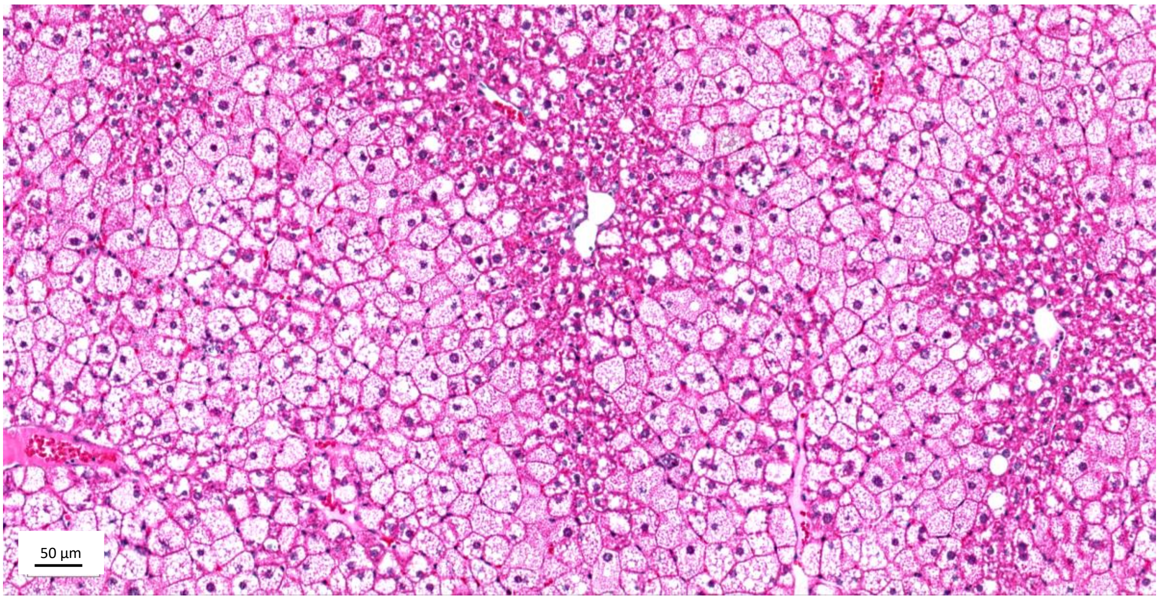

HFHCD-I-9

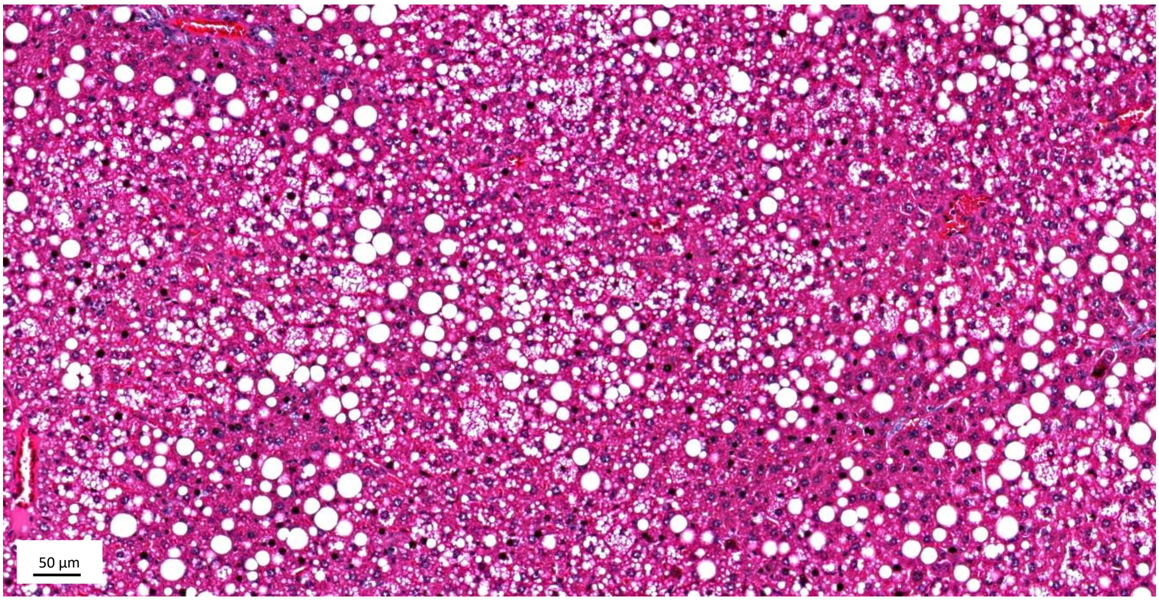

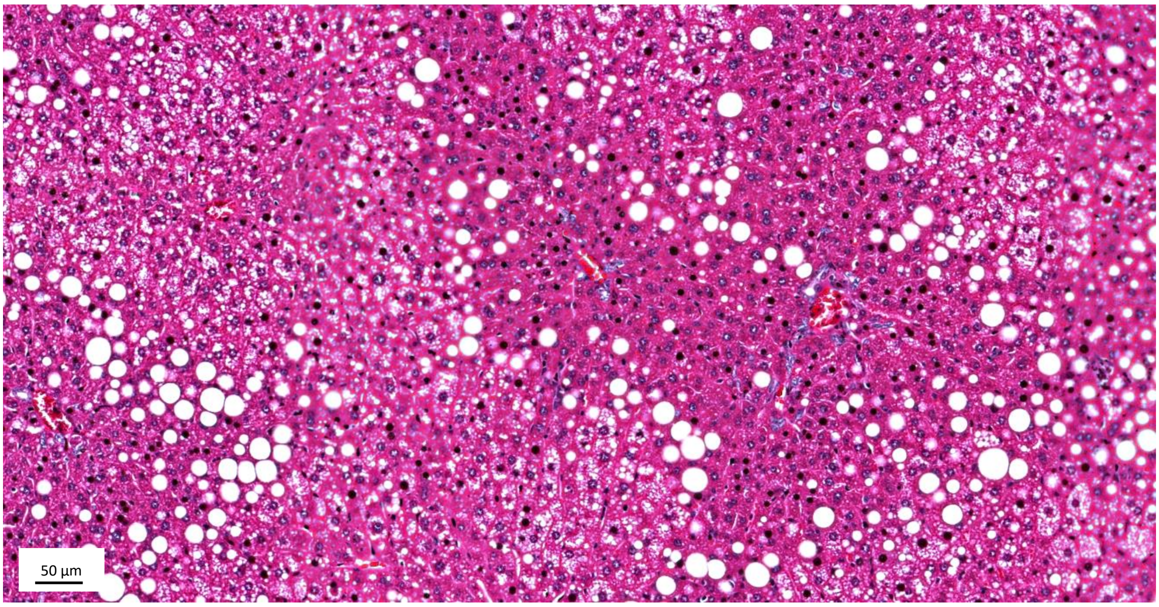

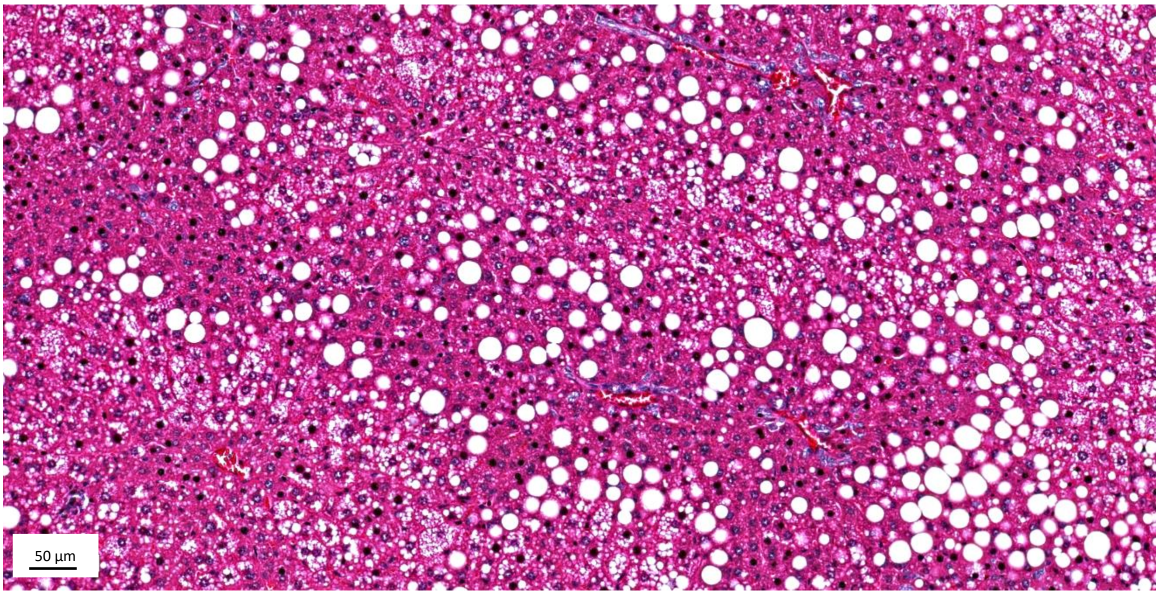

HFHCD-I-10

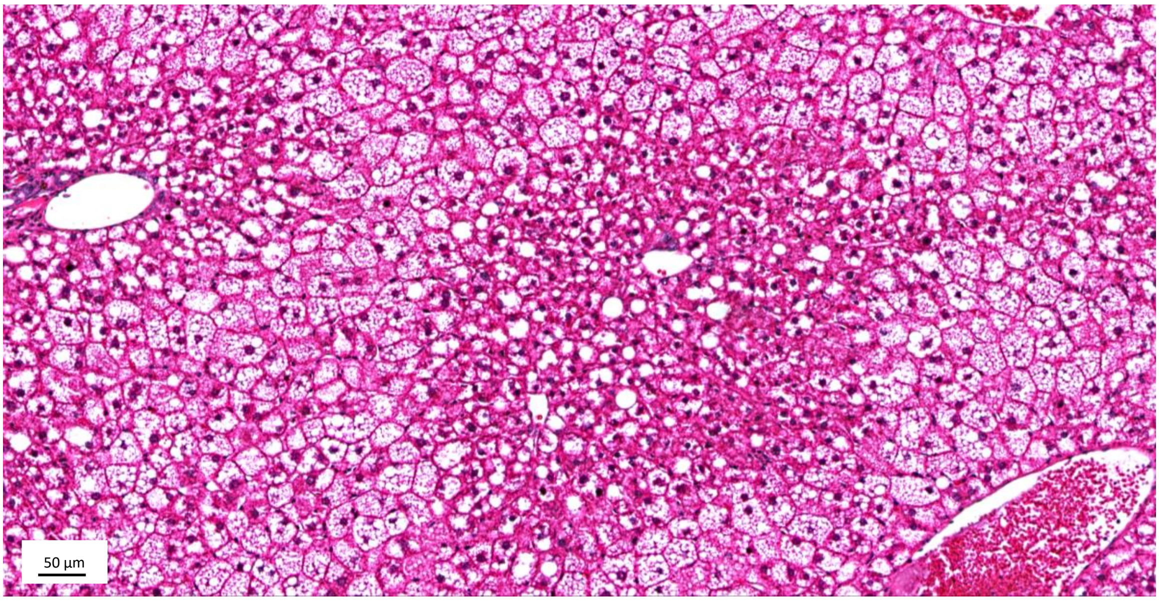

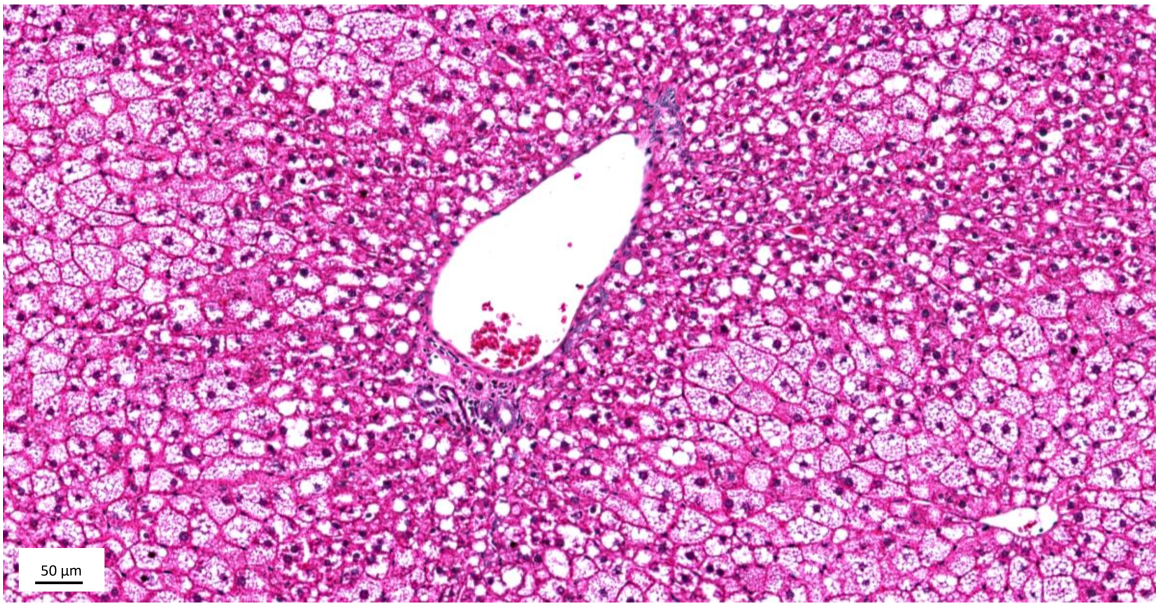

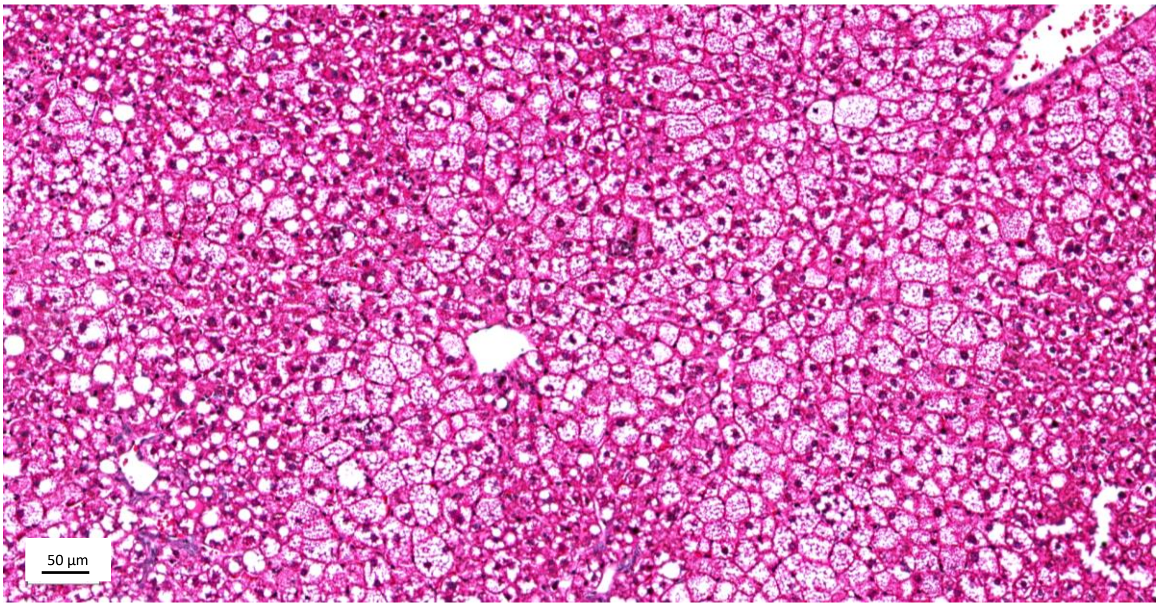

HFHCD-I-11

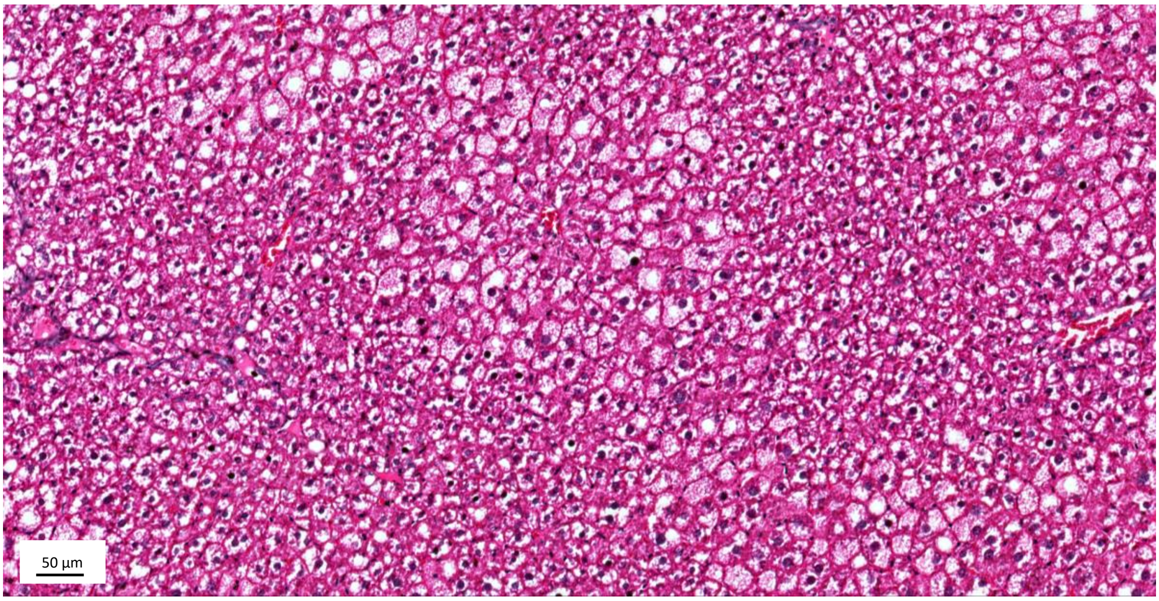

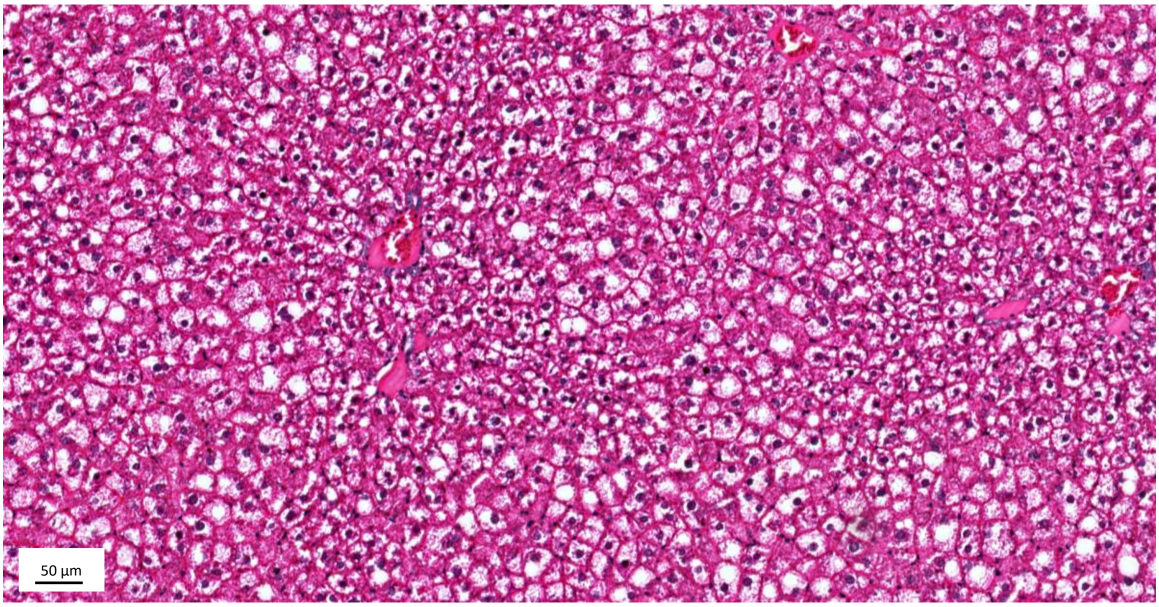

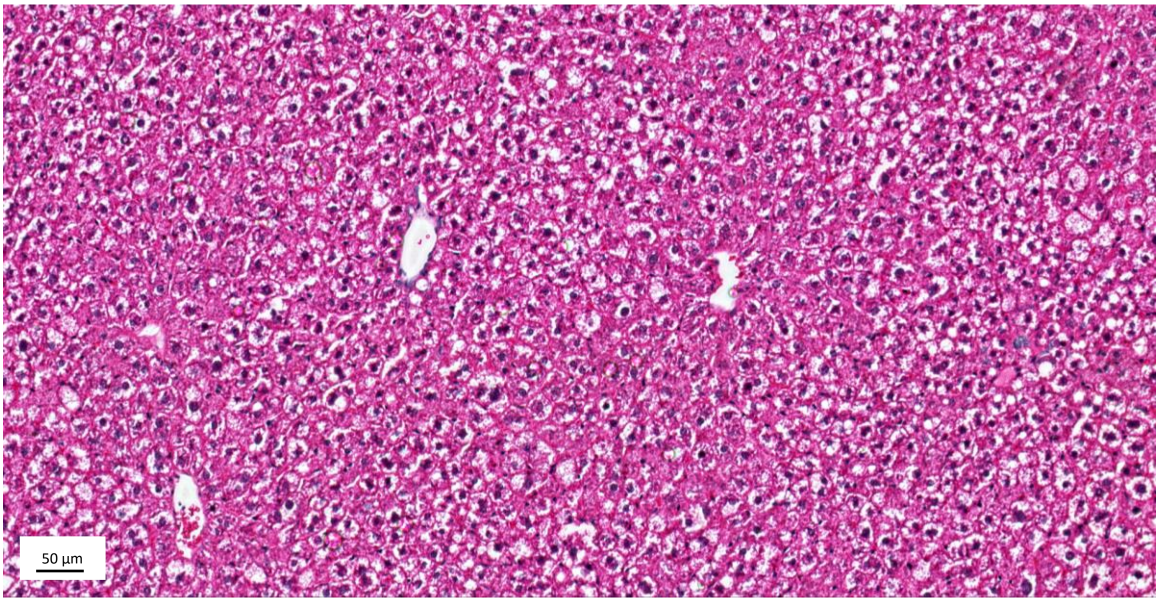

HFHCD-I-12

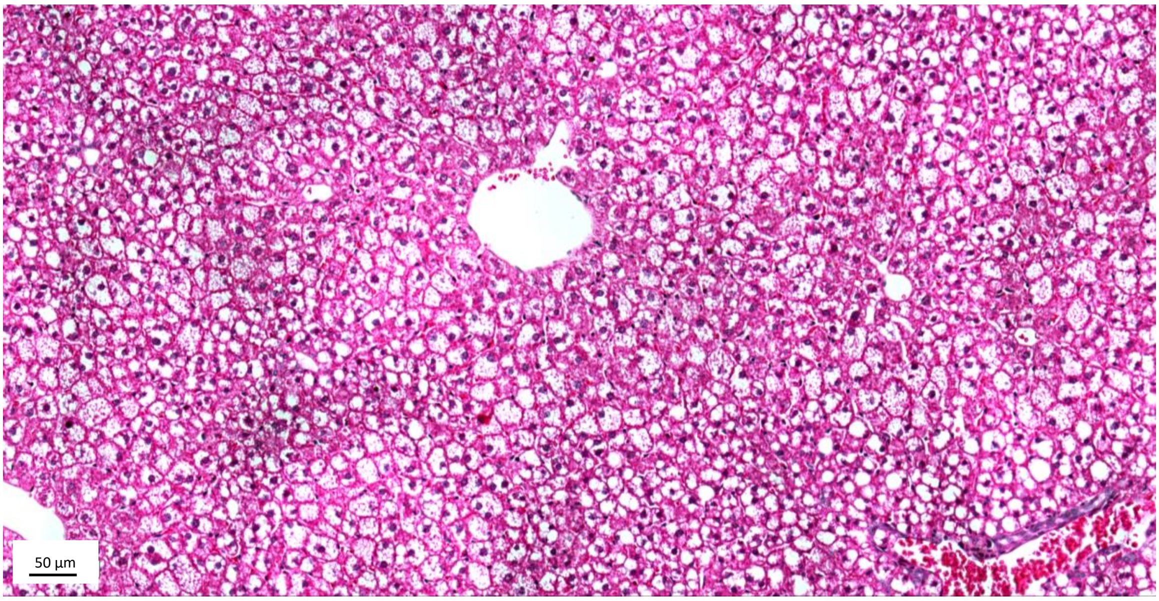

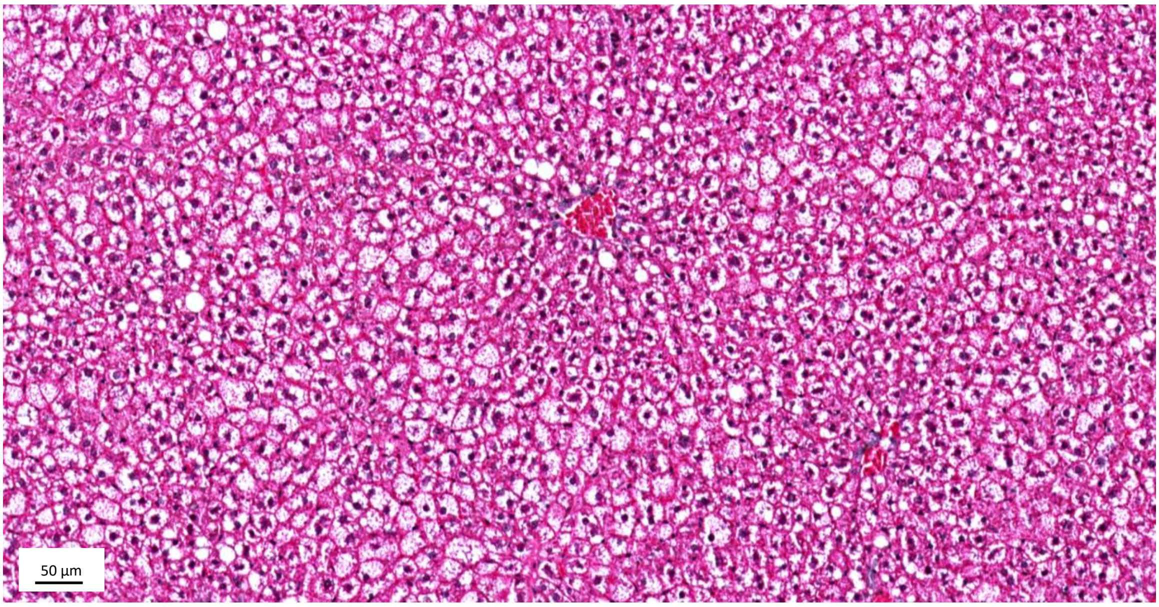

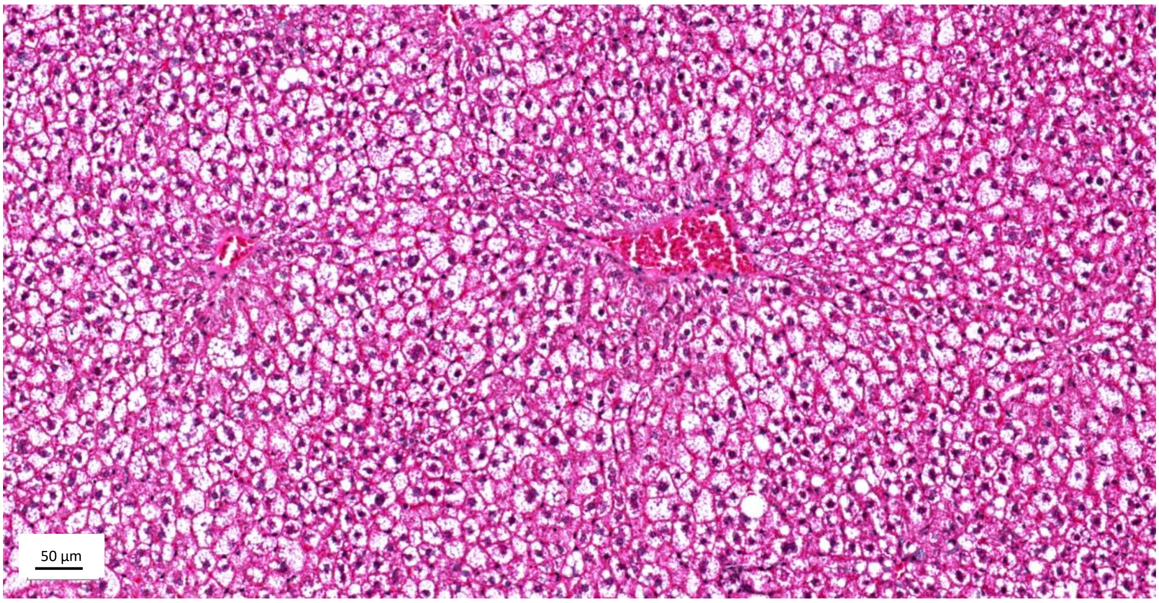

H&E Staining

HFHCD-C group

(12 mice were included)

HFHCD-C-1

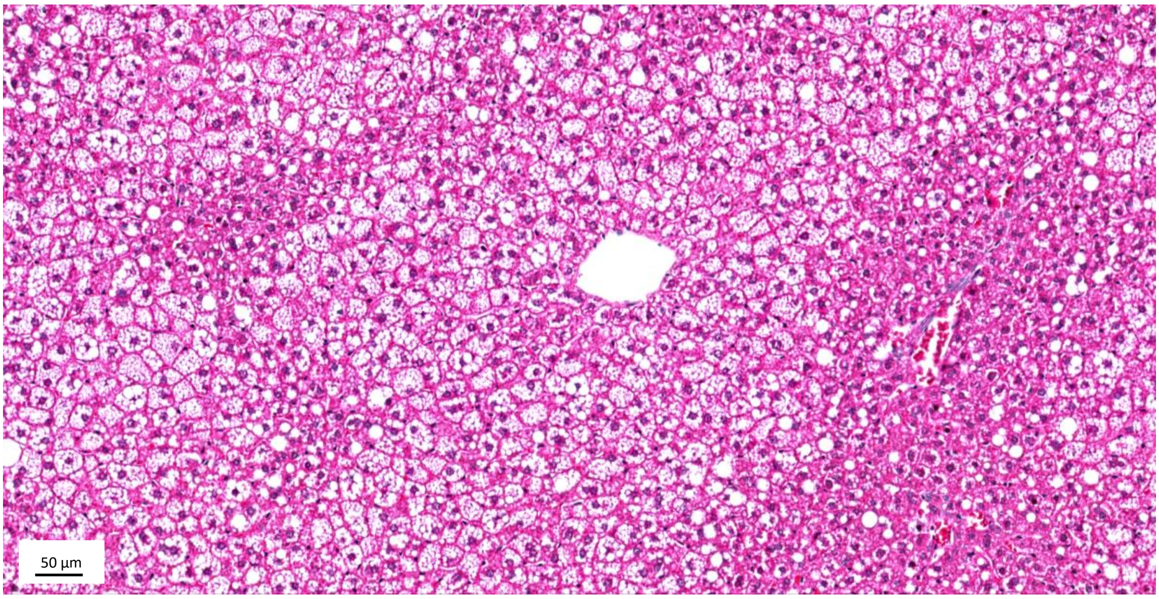

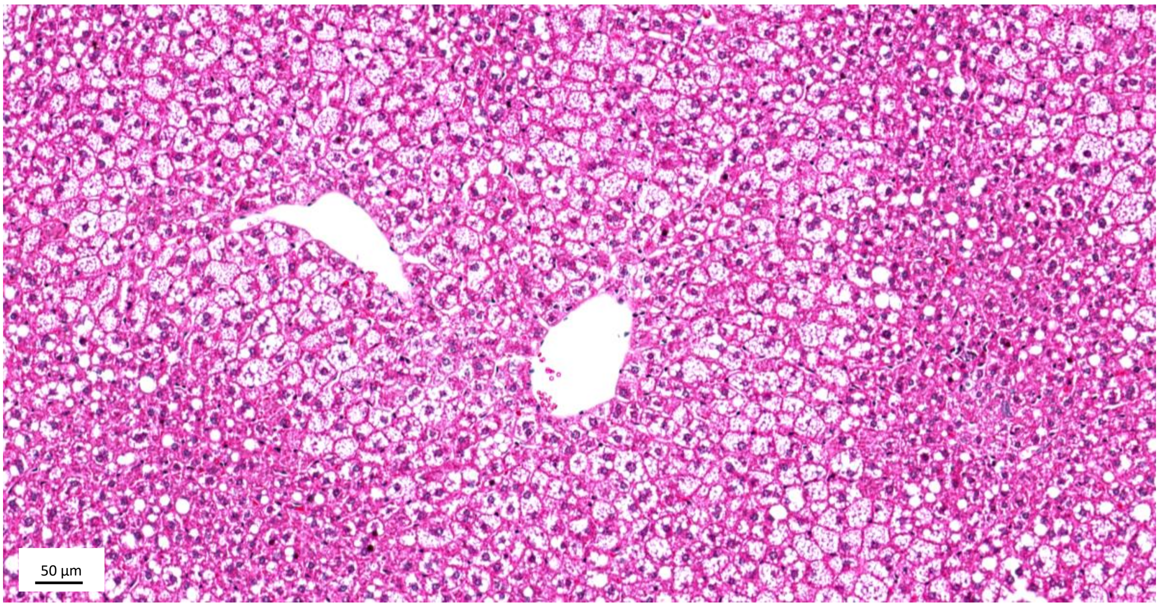

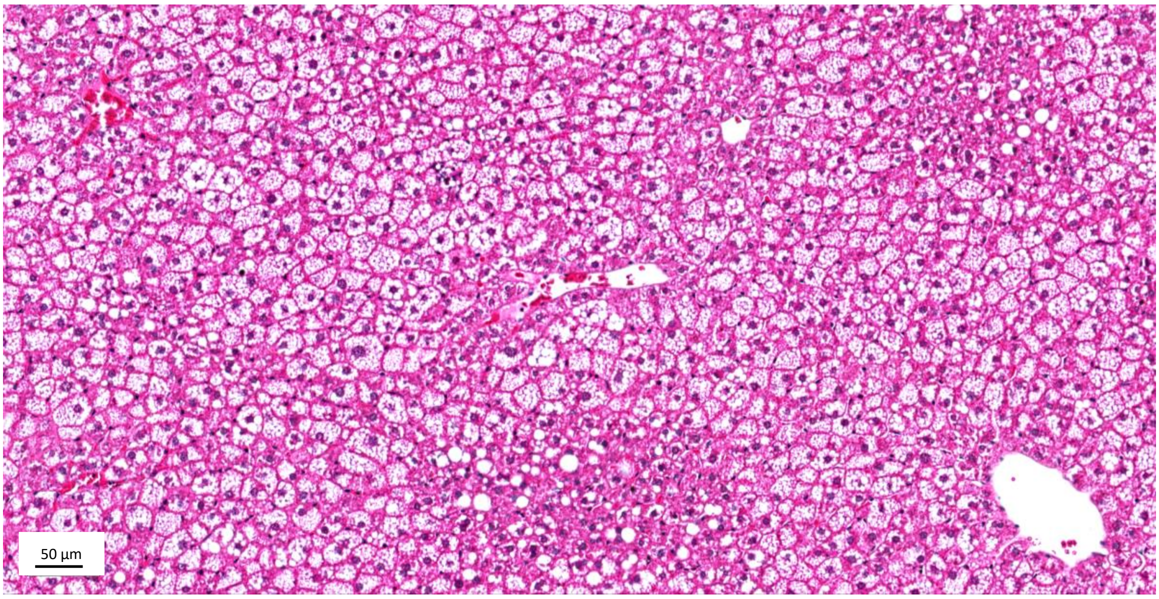

HFHCD-C-2

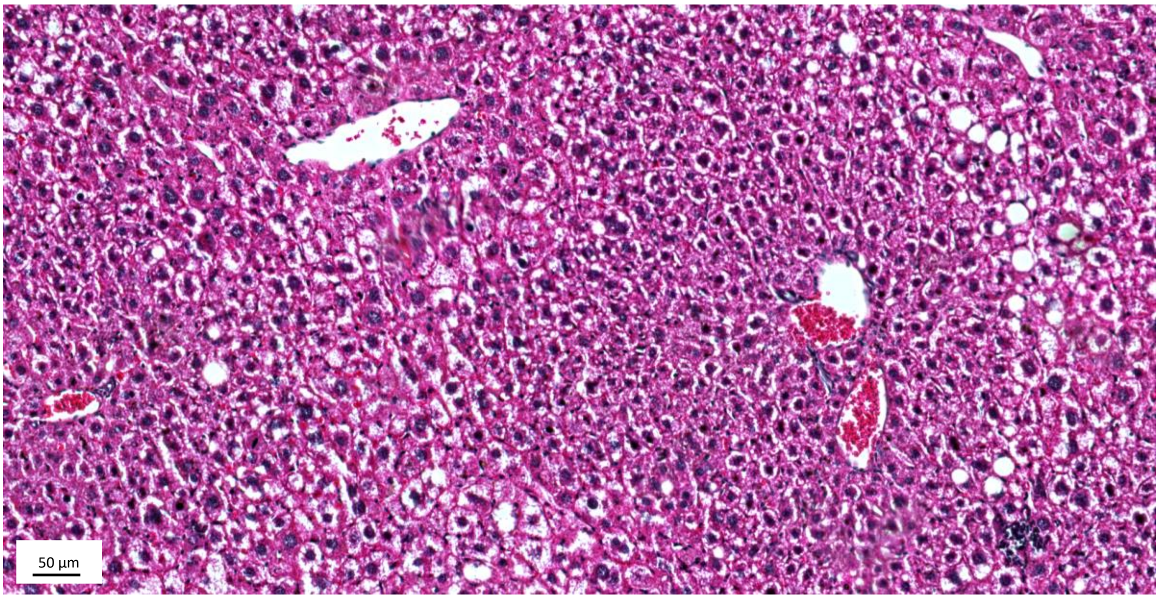

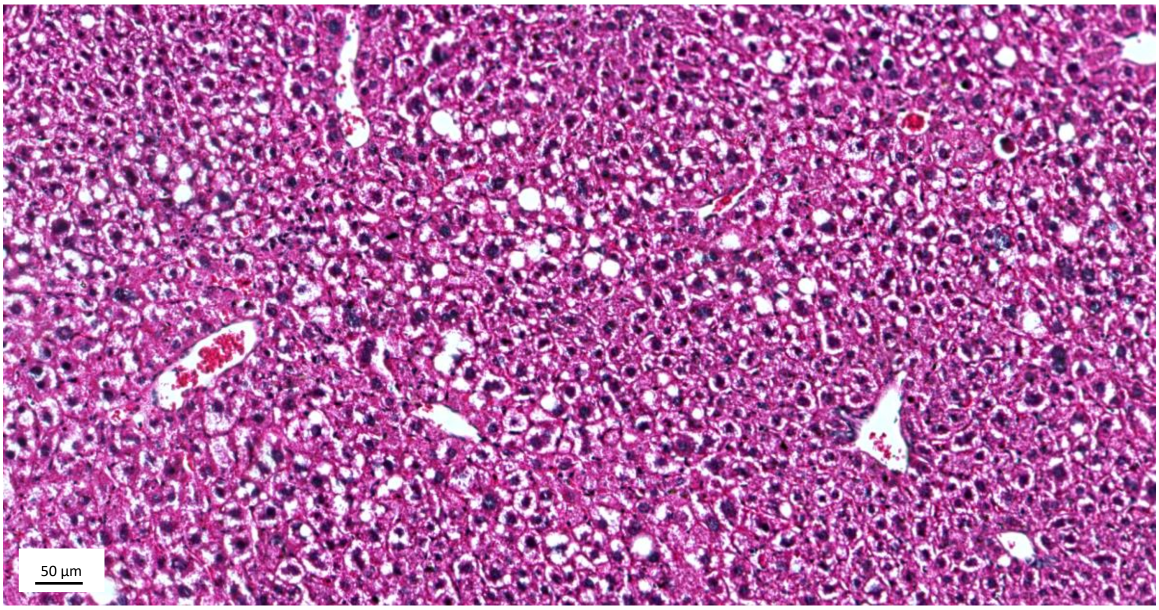

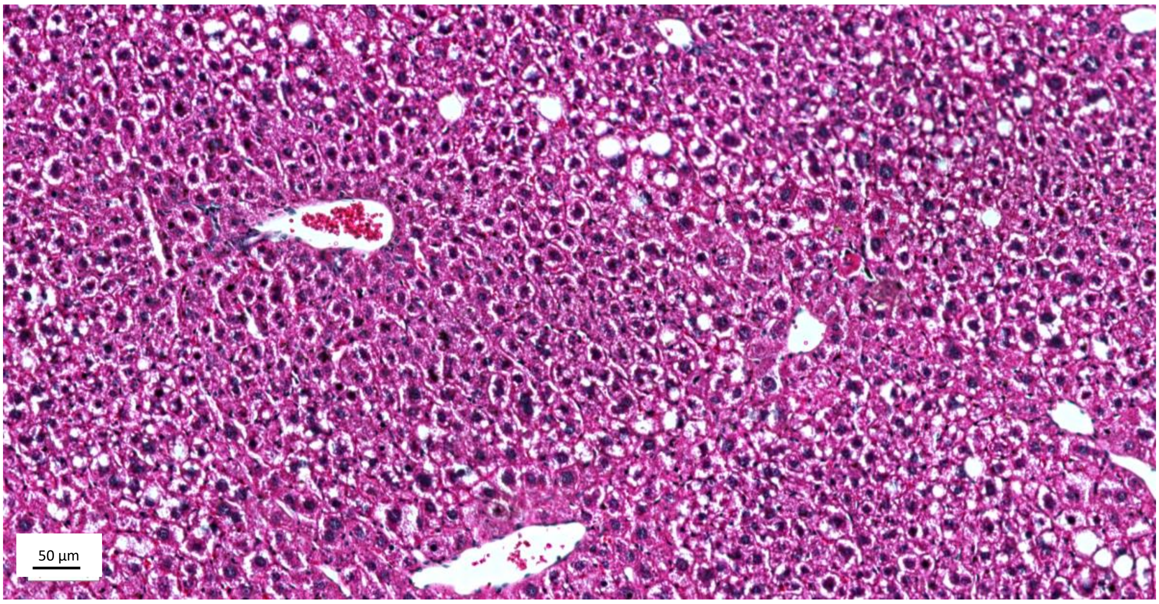

HFHCD-C-3

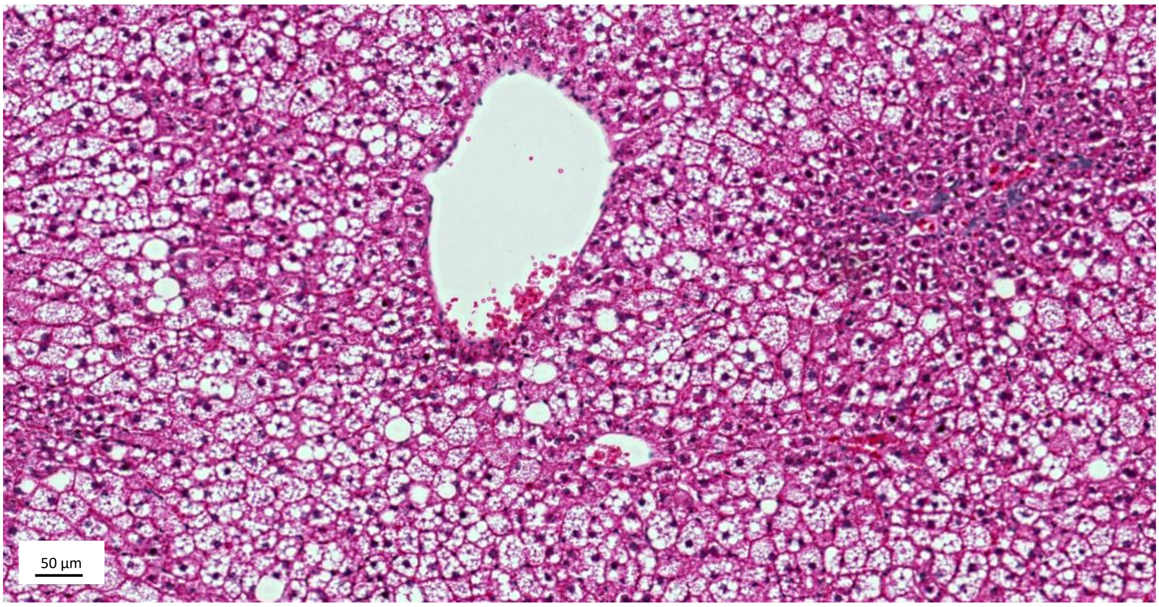

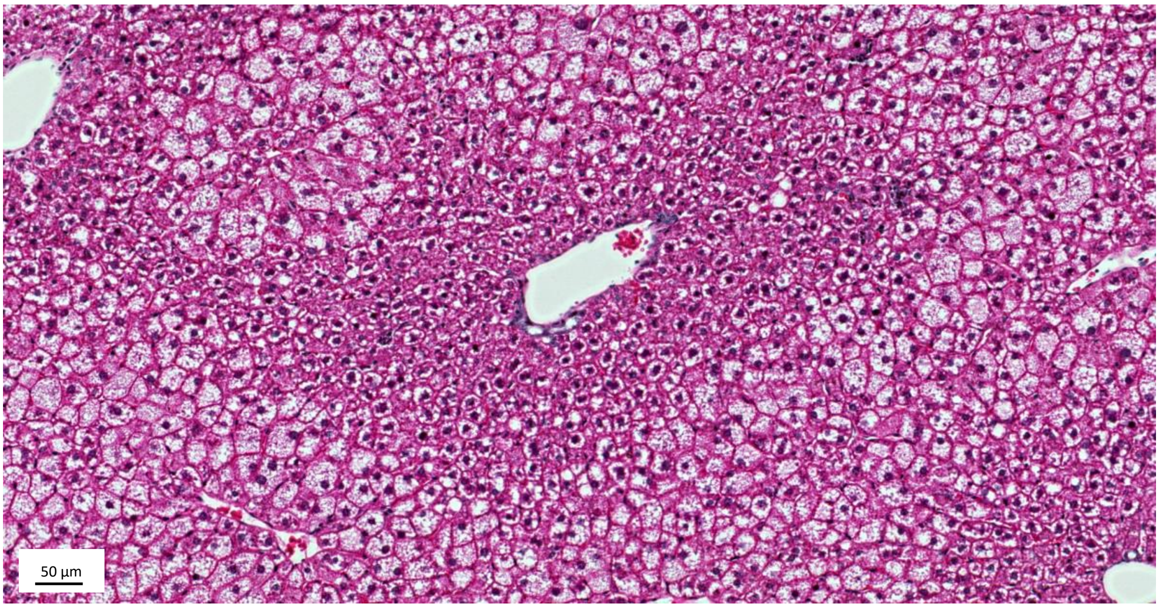

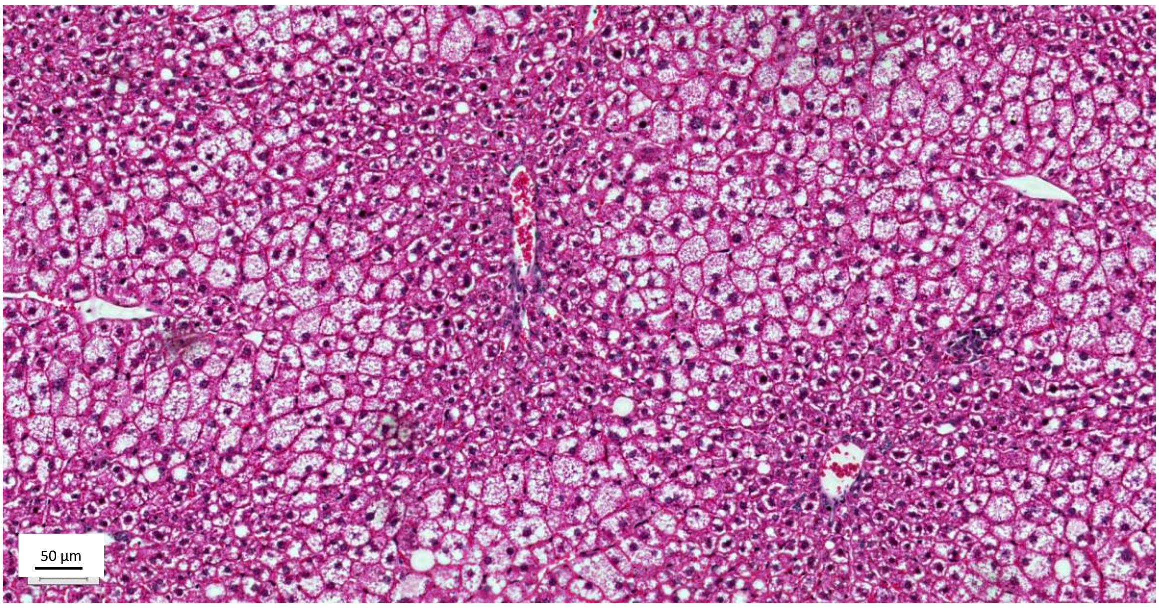

HFHCD-C-4

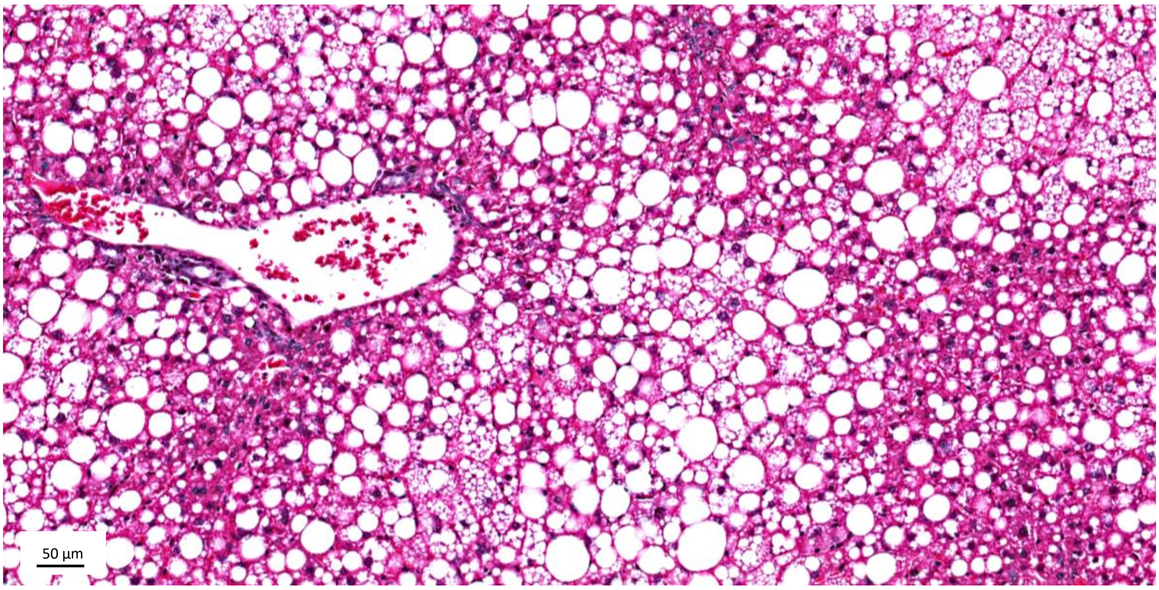

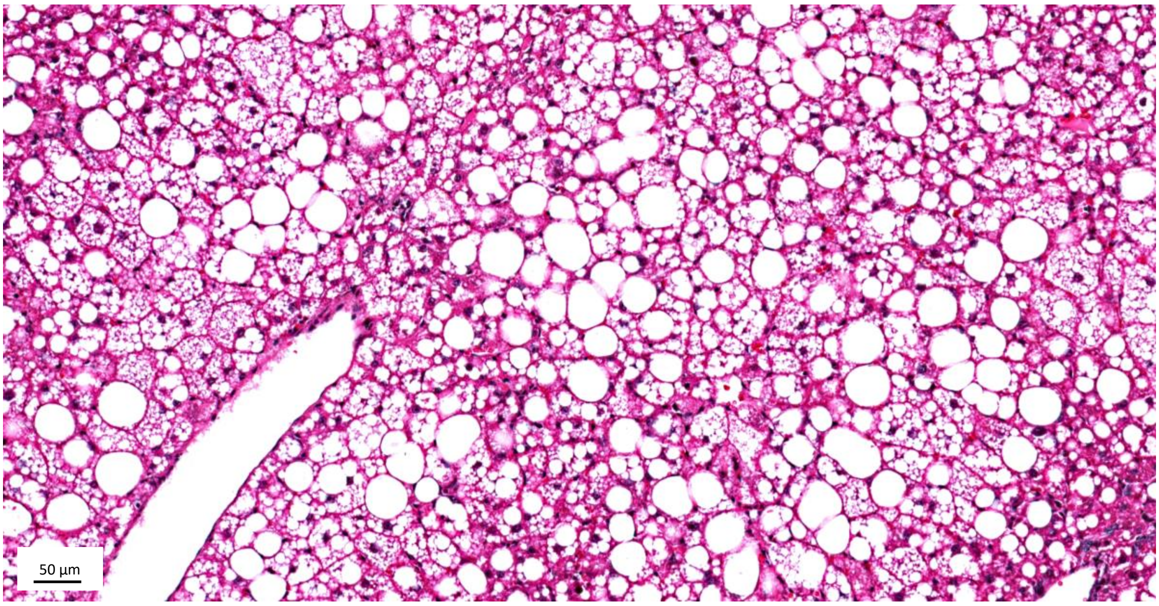

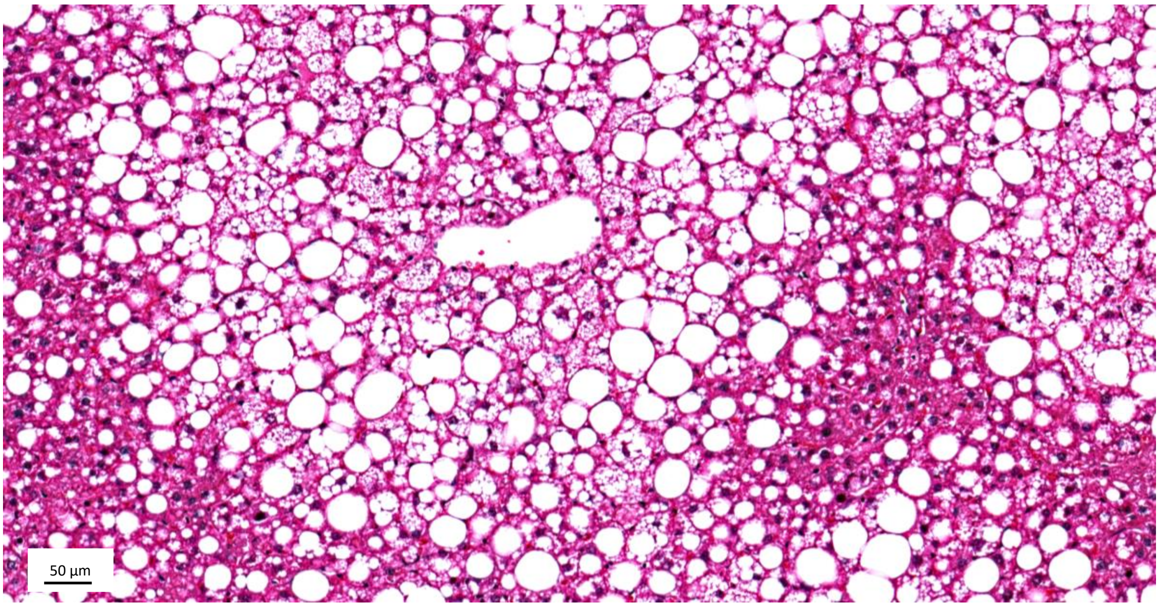

HFHCD-C-5

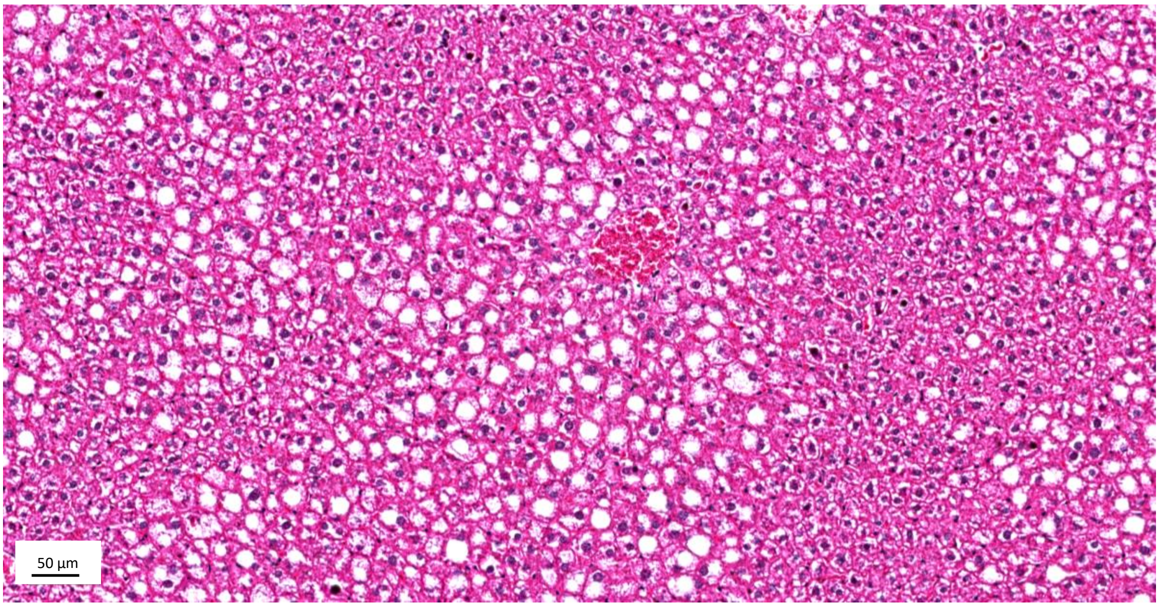

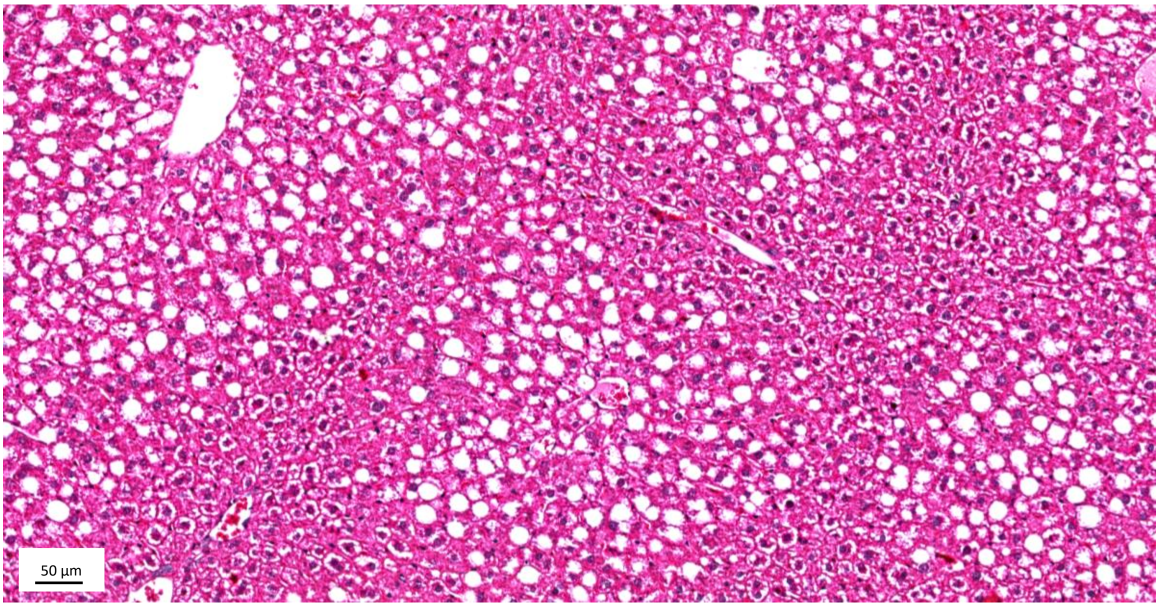

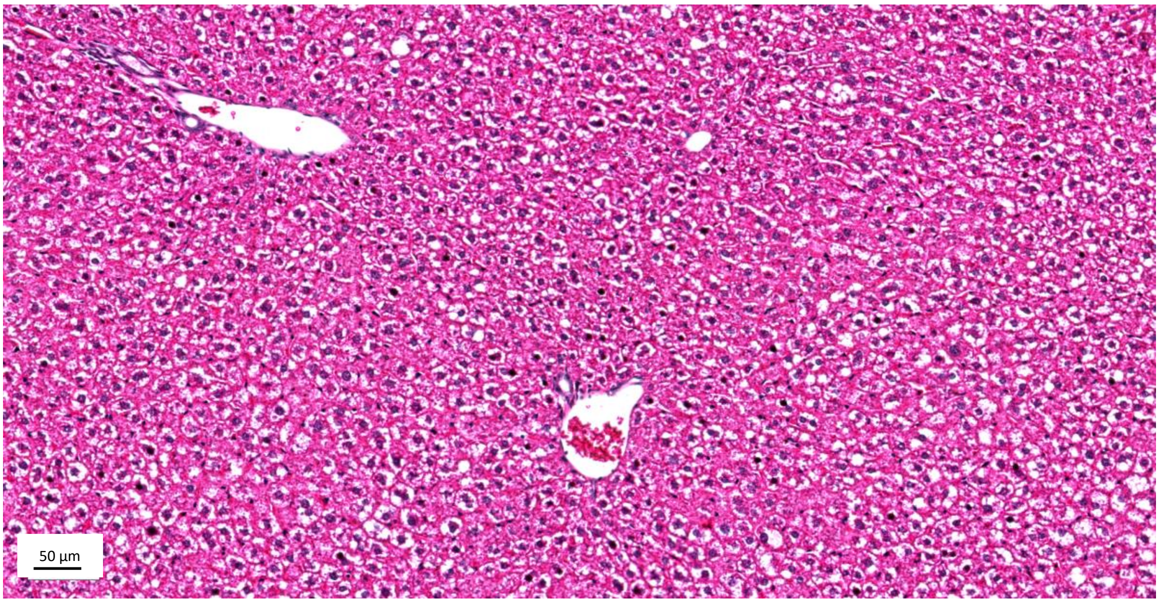

HFHCD-C-6

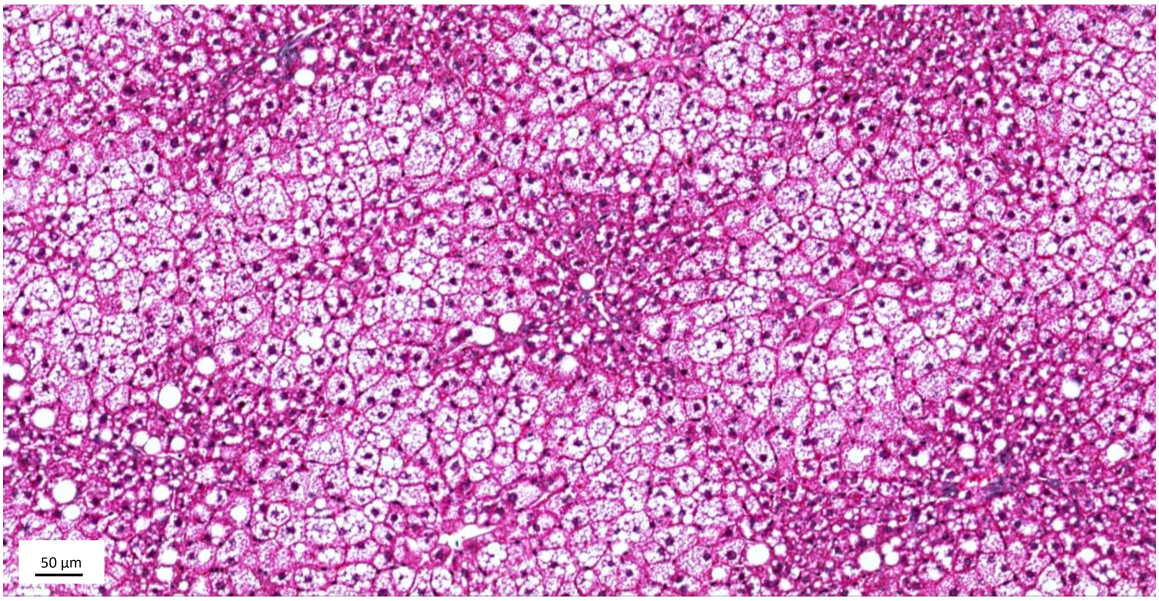

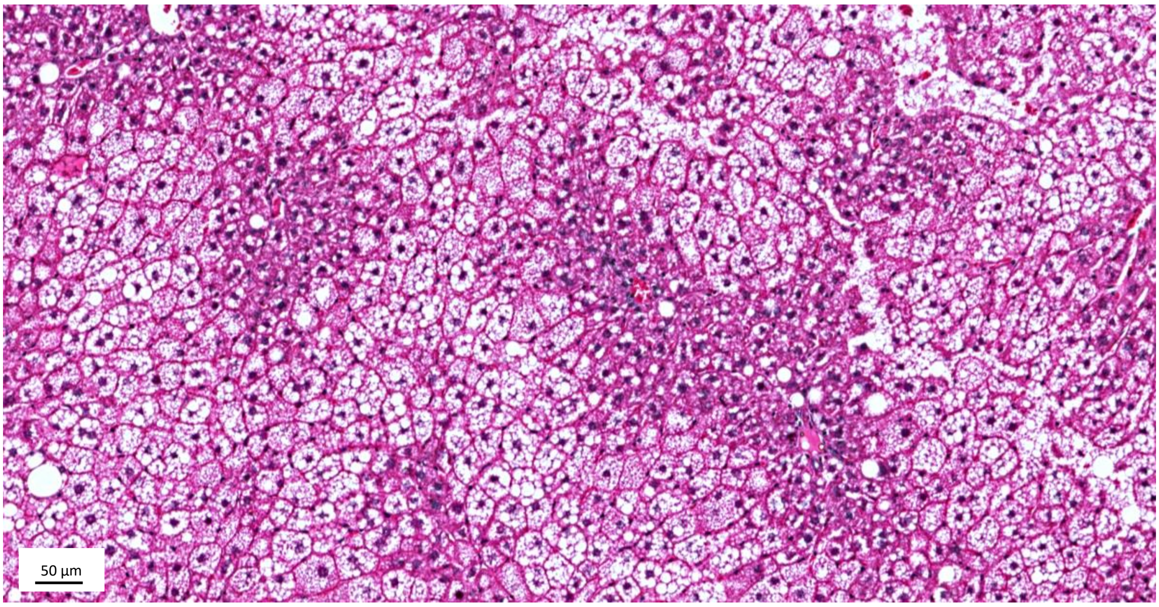

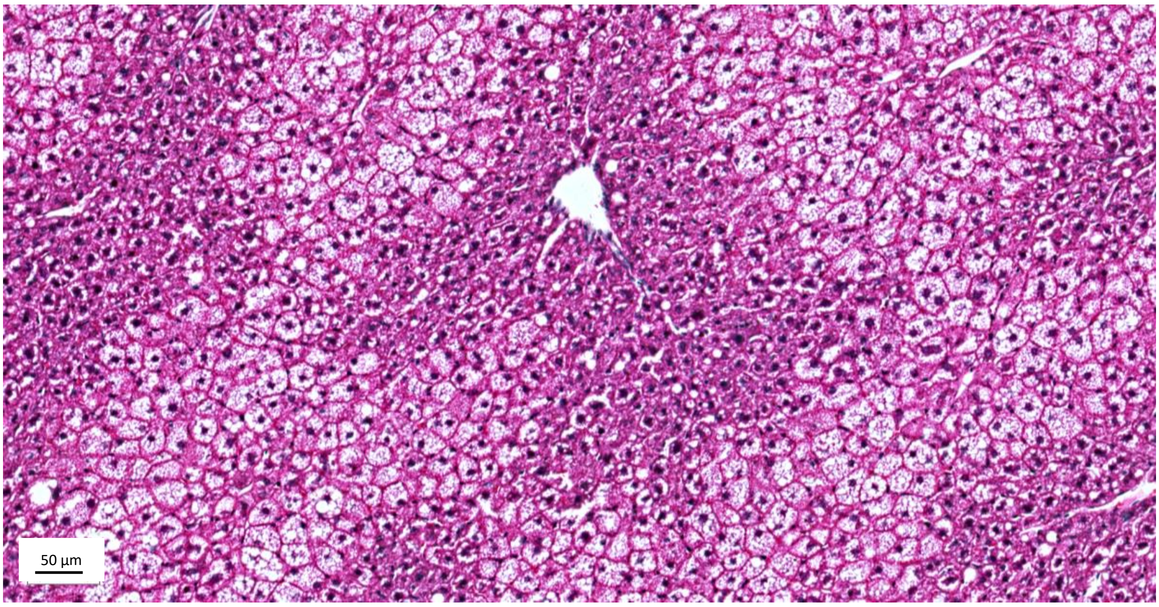

HFHCD-C-7

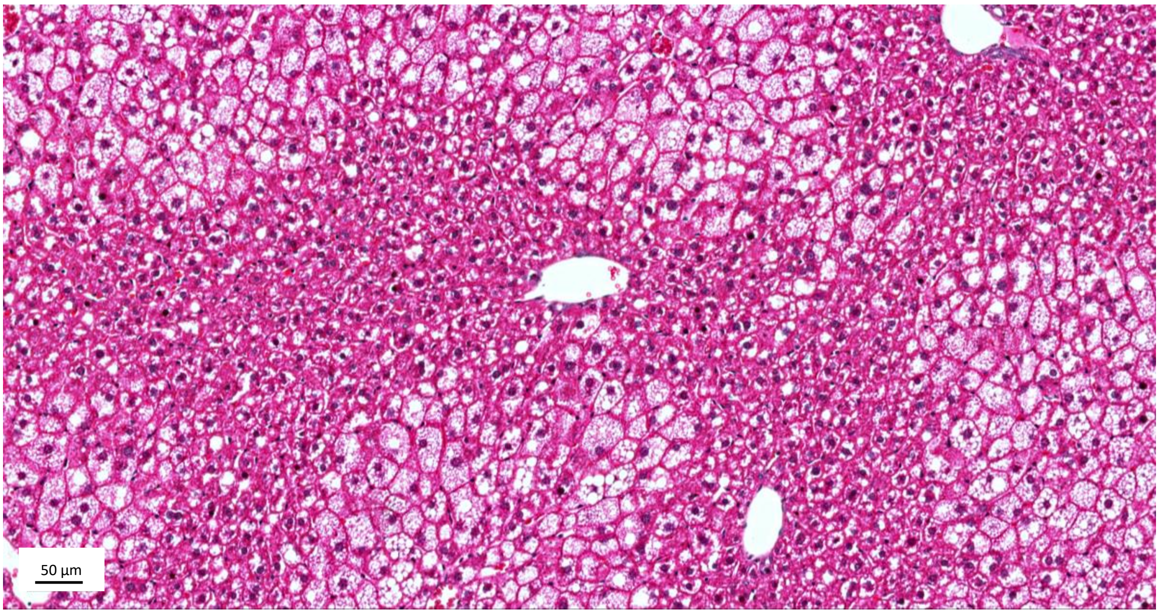

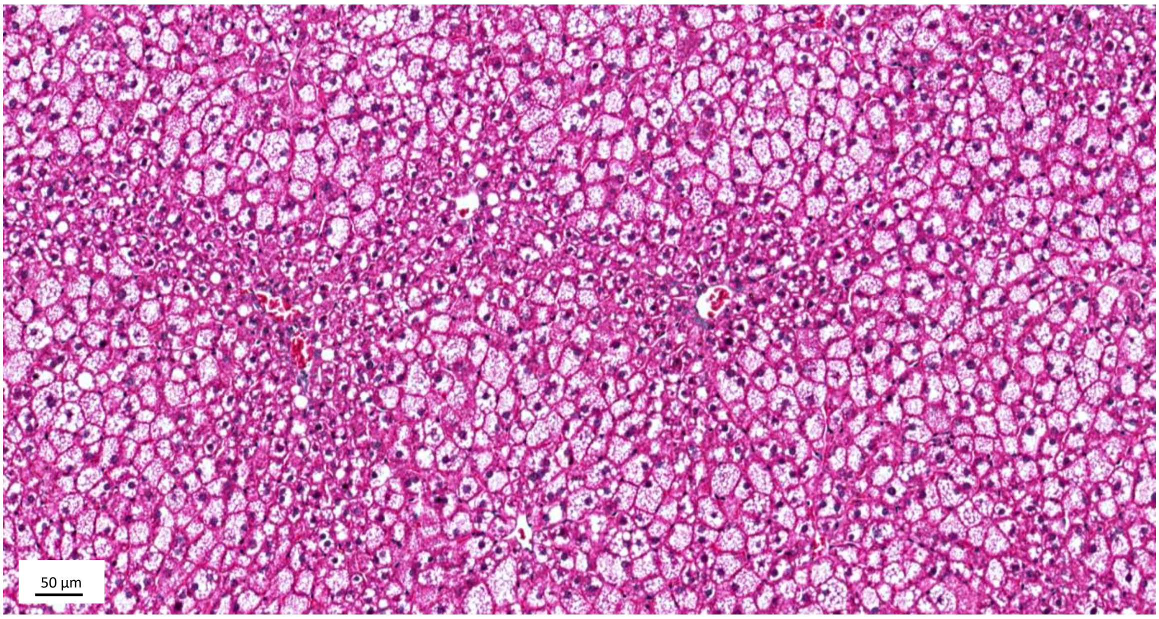

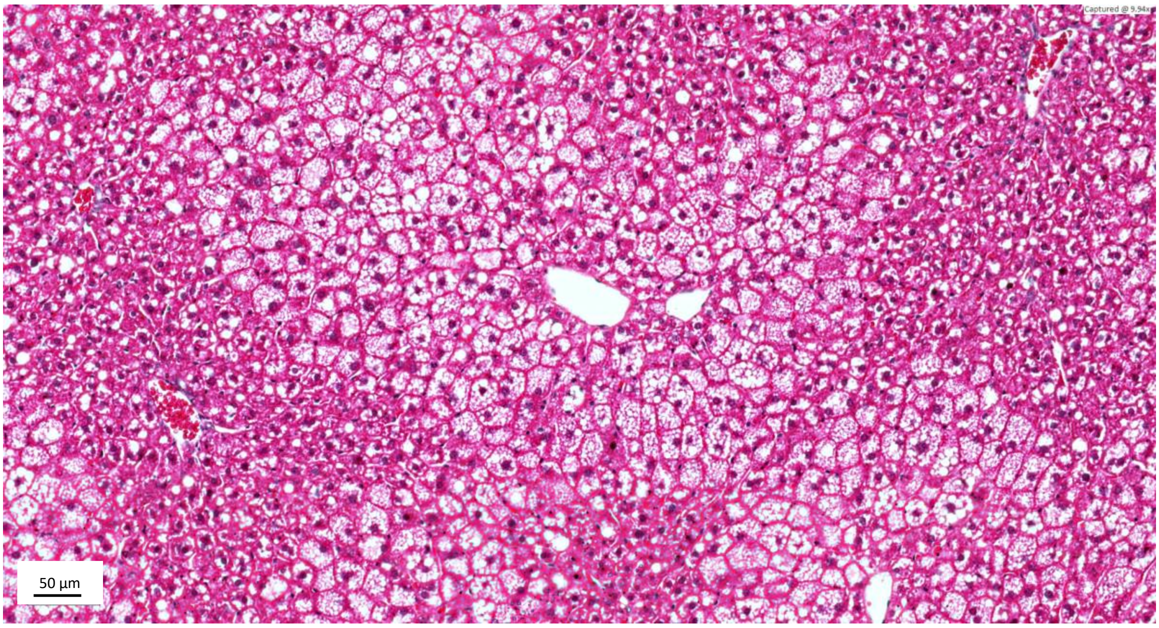

HFHCD-C-8

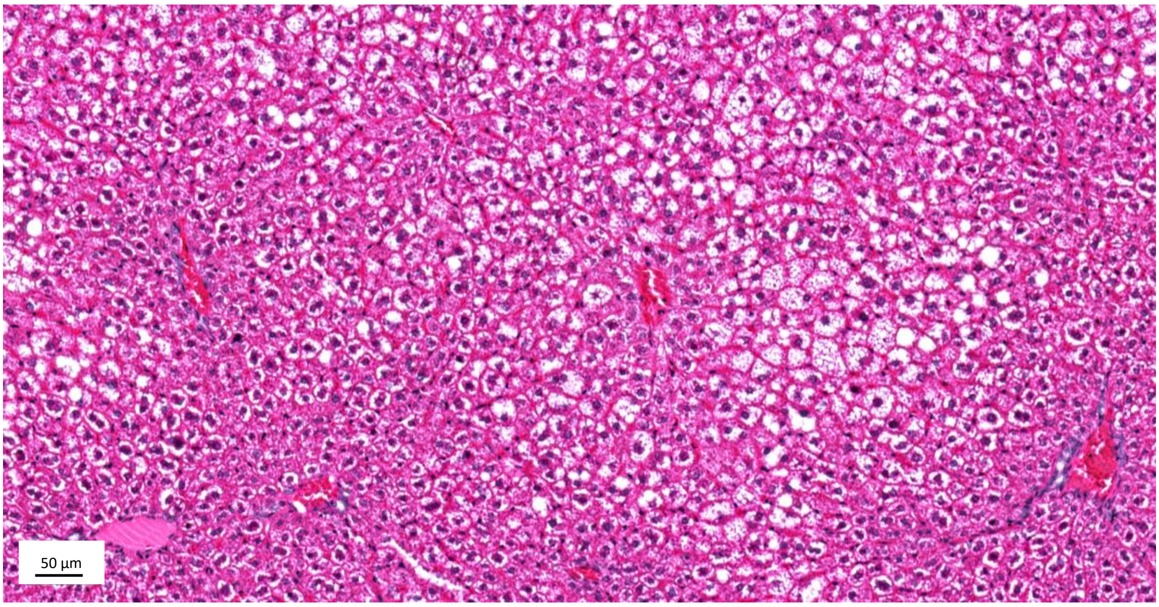

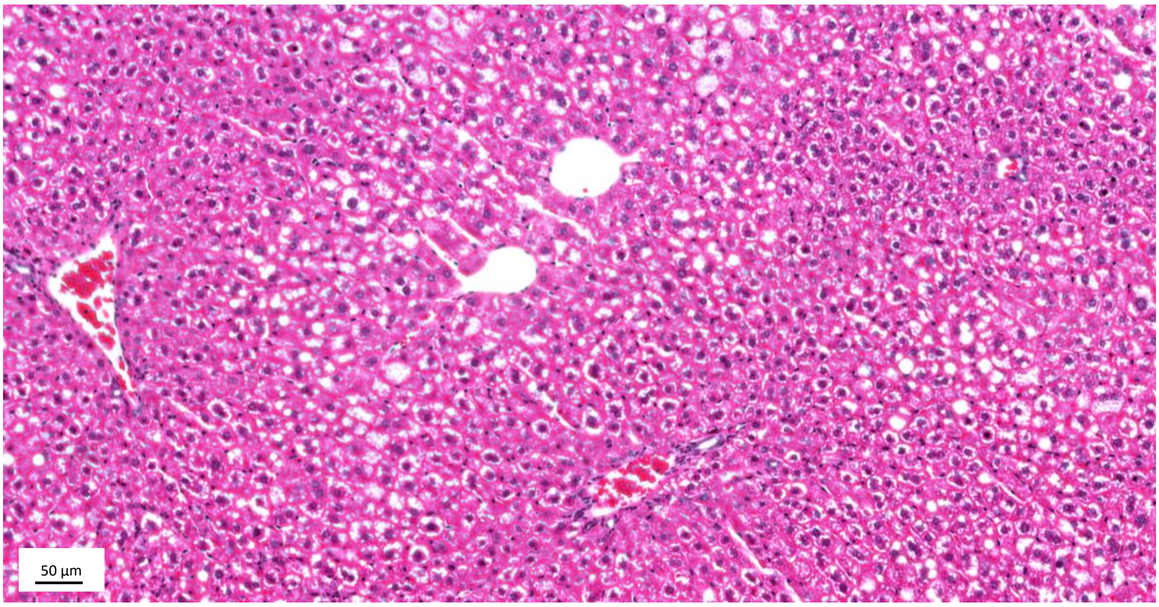

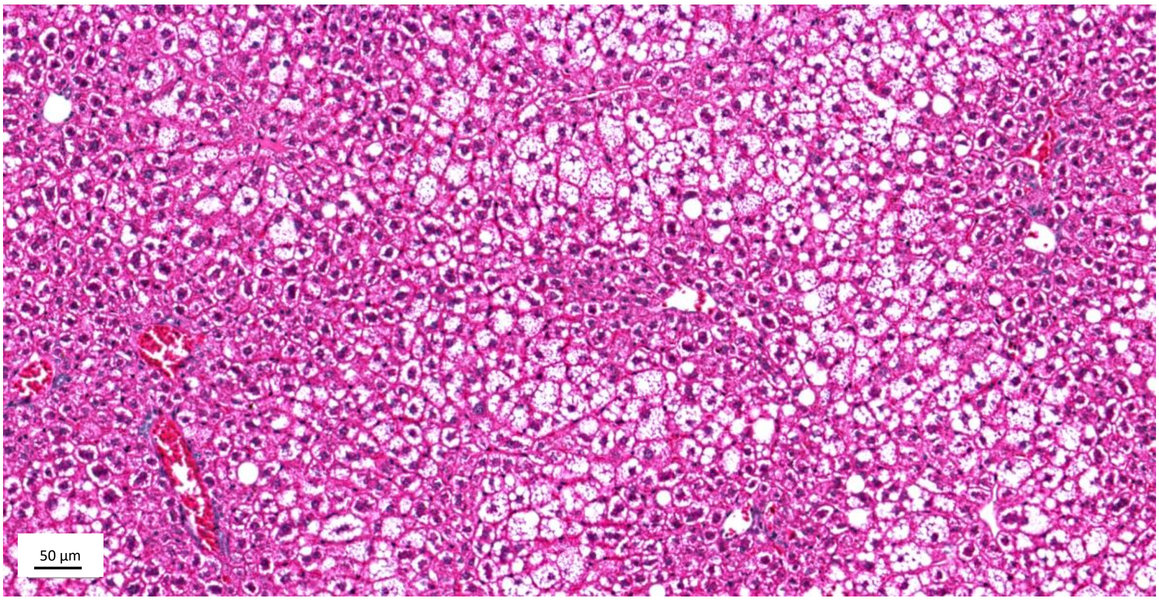

HFHCD-C-9

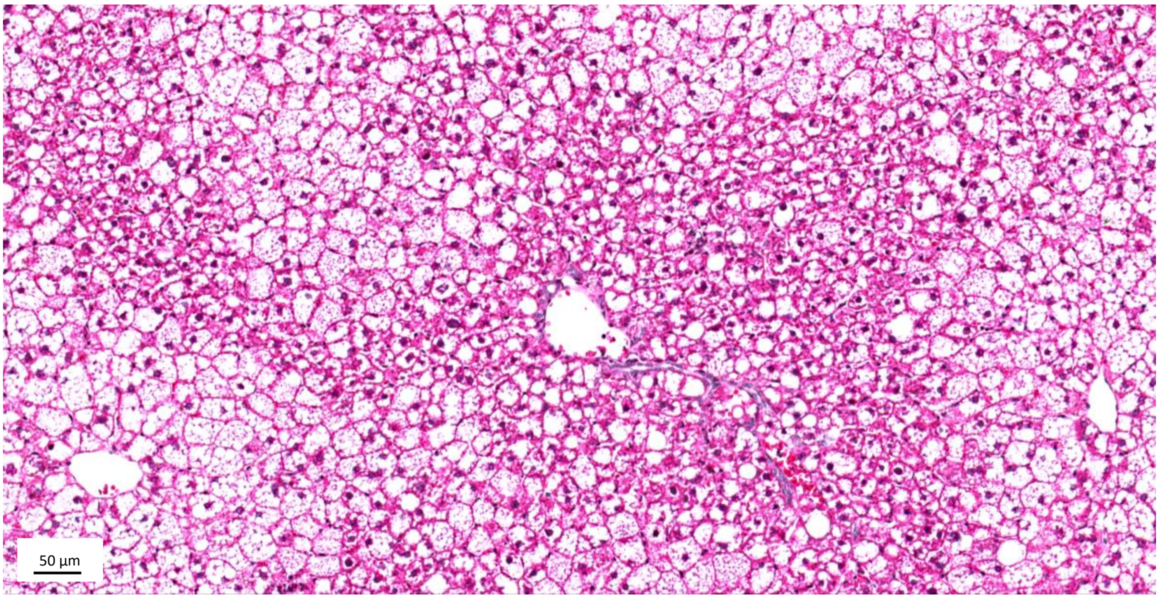

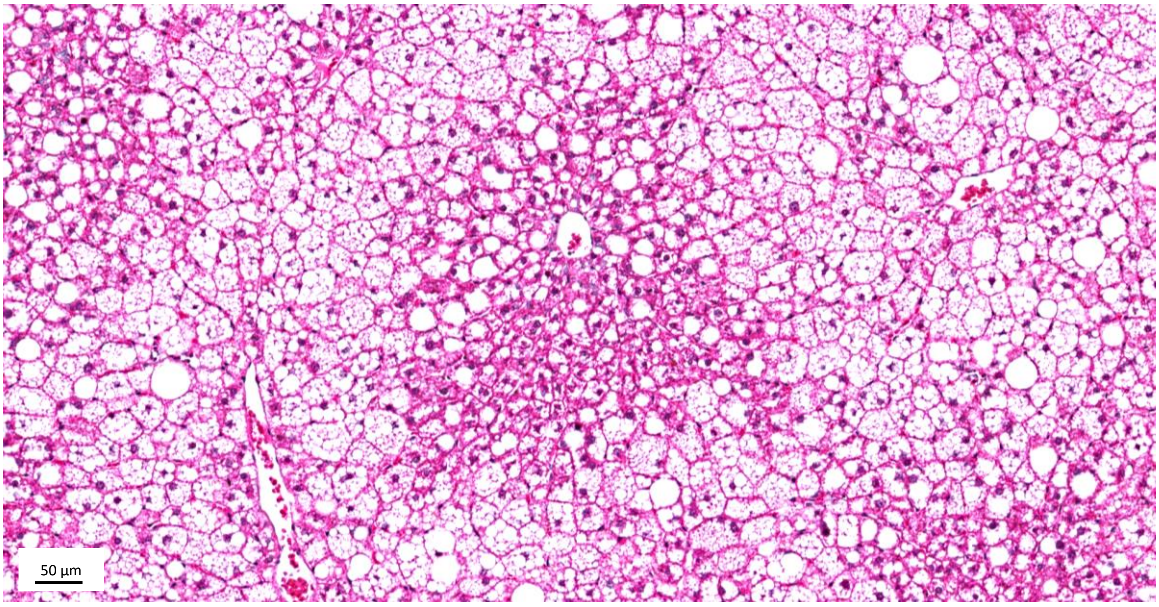

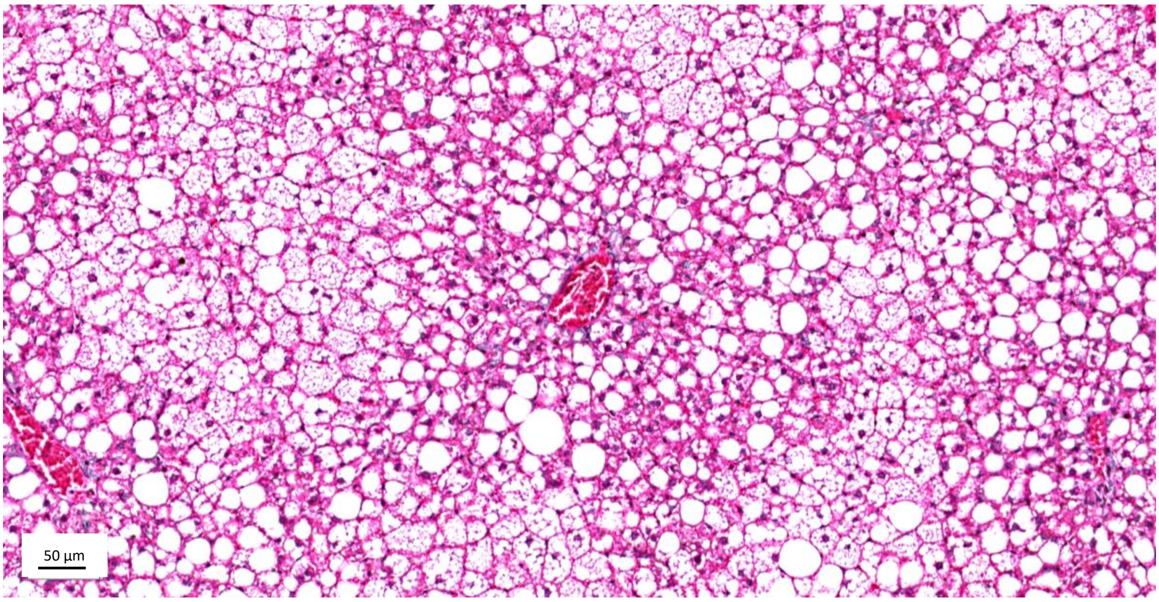

HFHCD-C-10

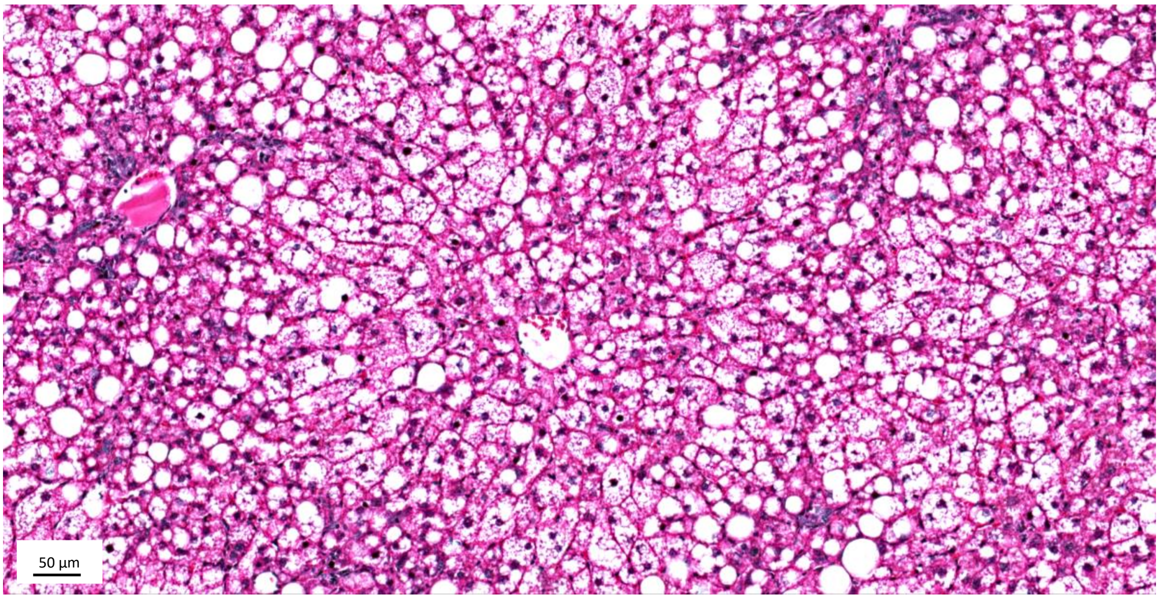

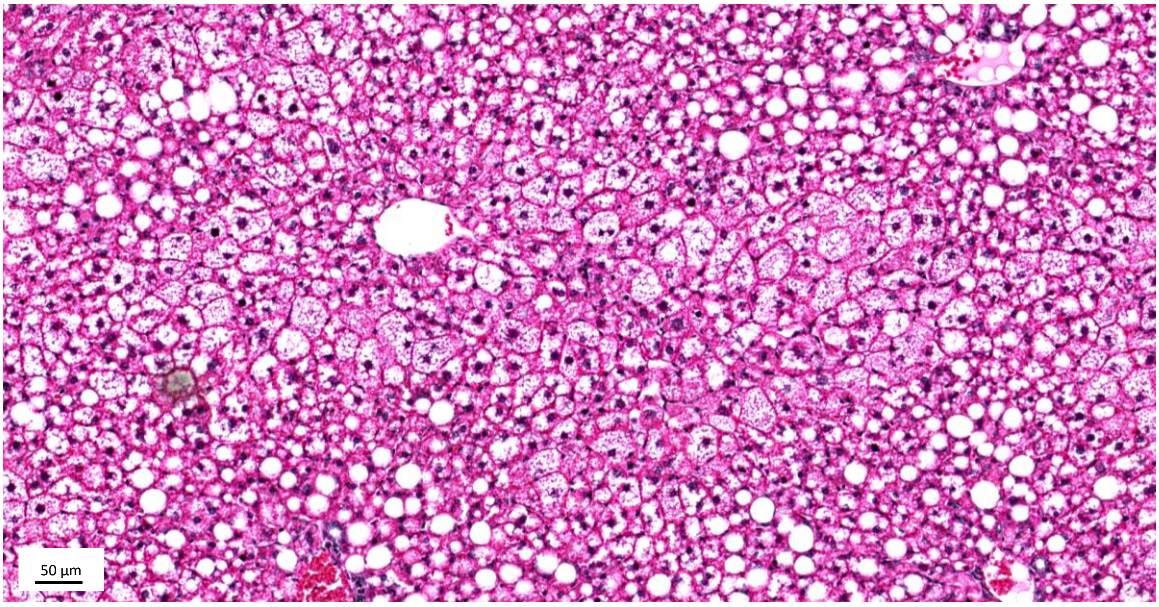

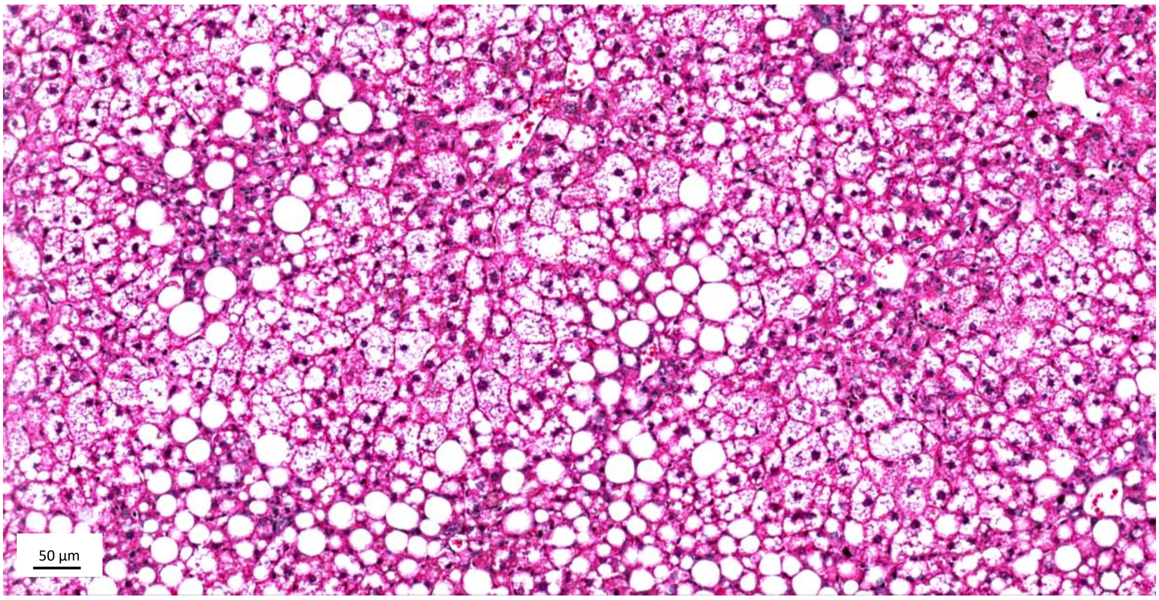

HFHCD-C-11

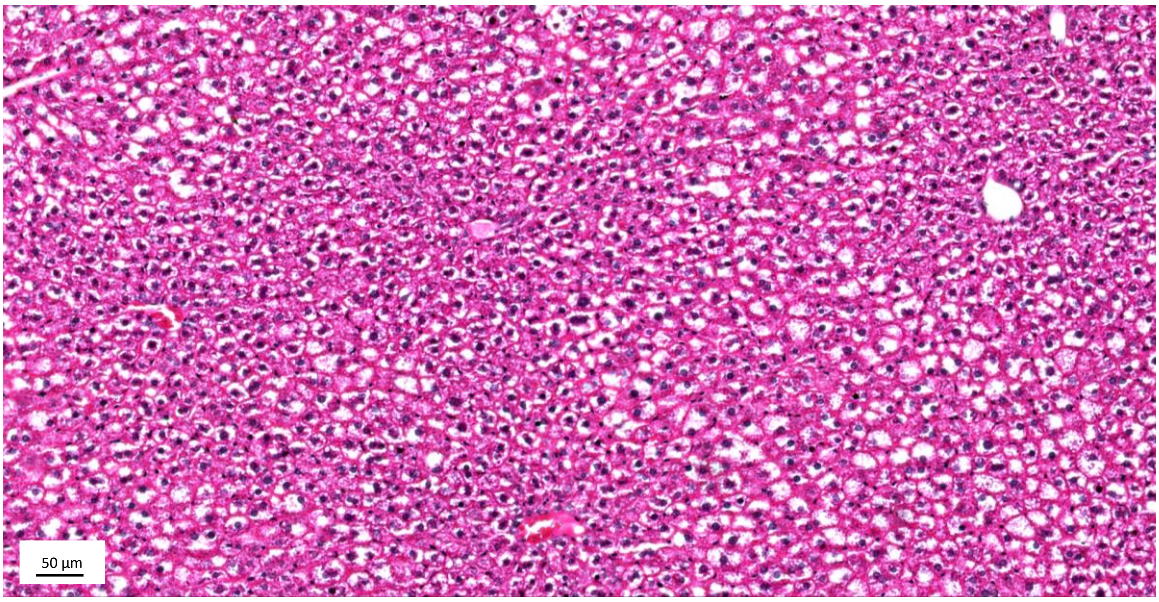

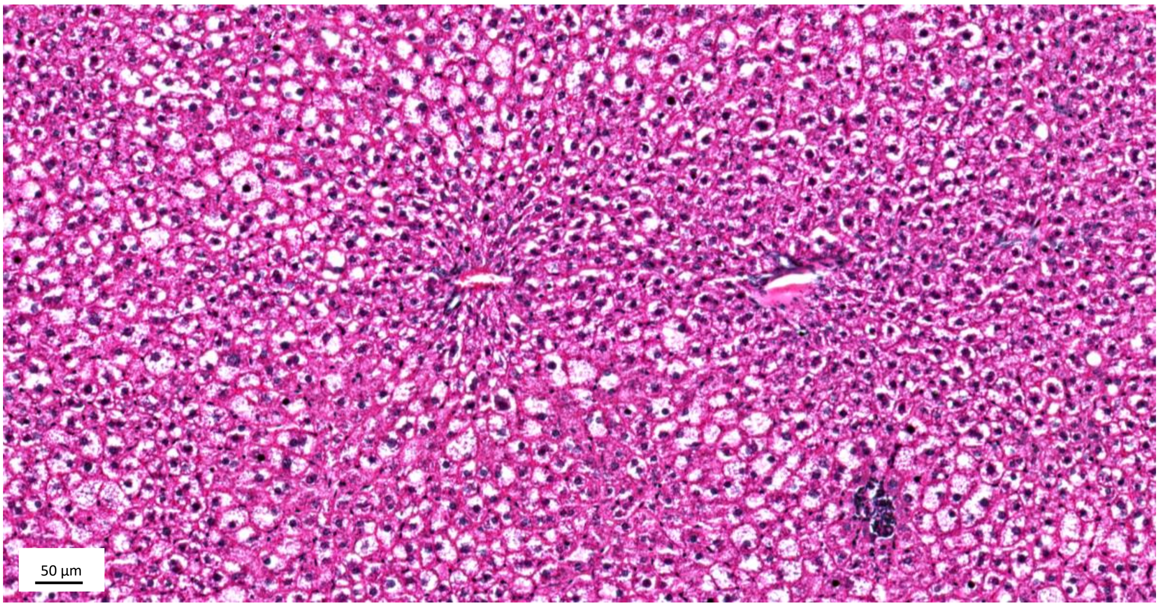

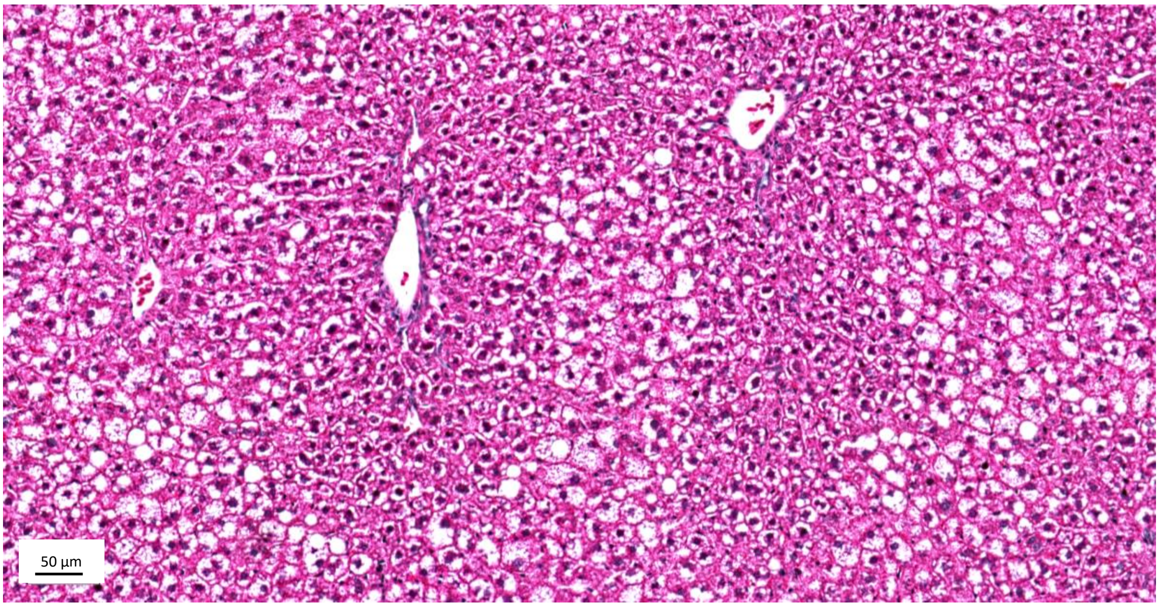

HFHCD-C-12

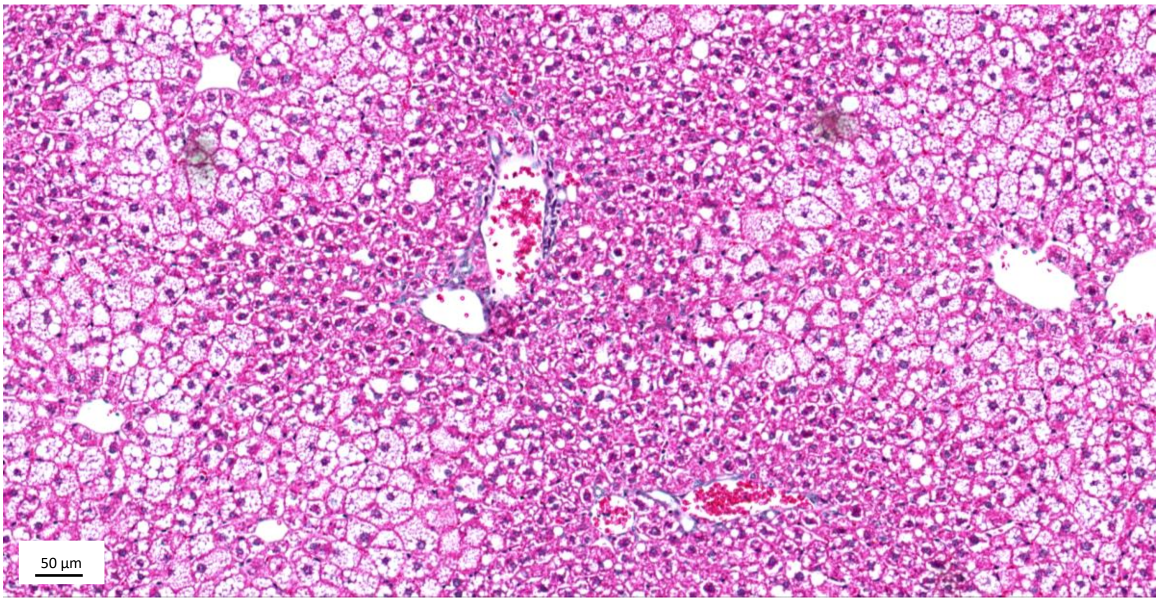

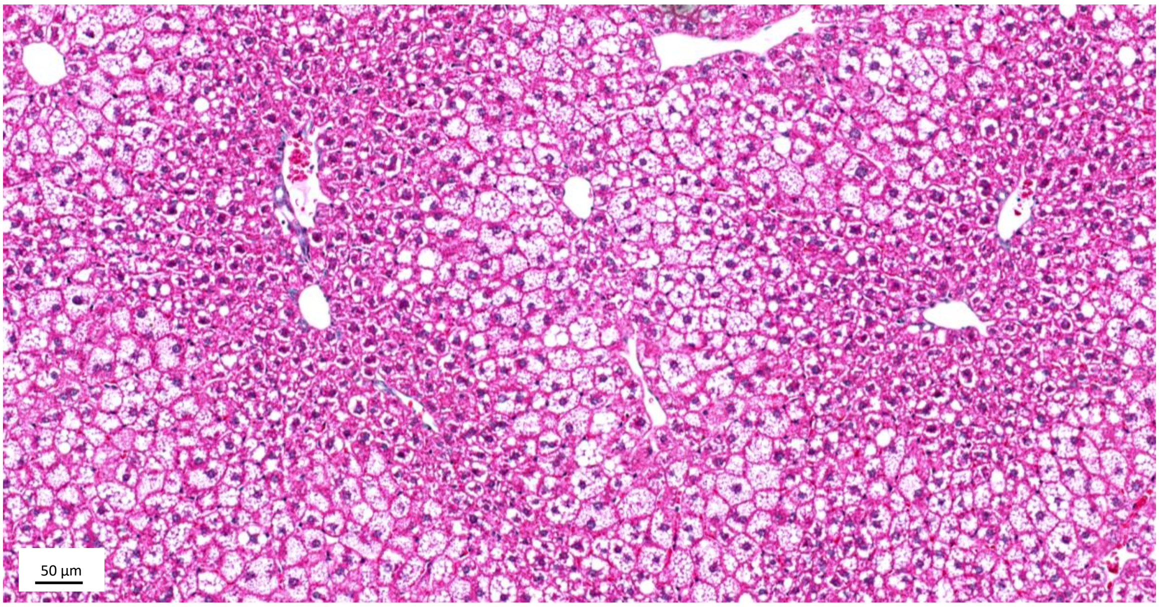

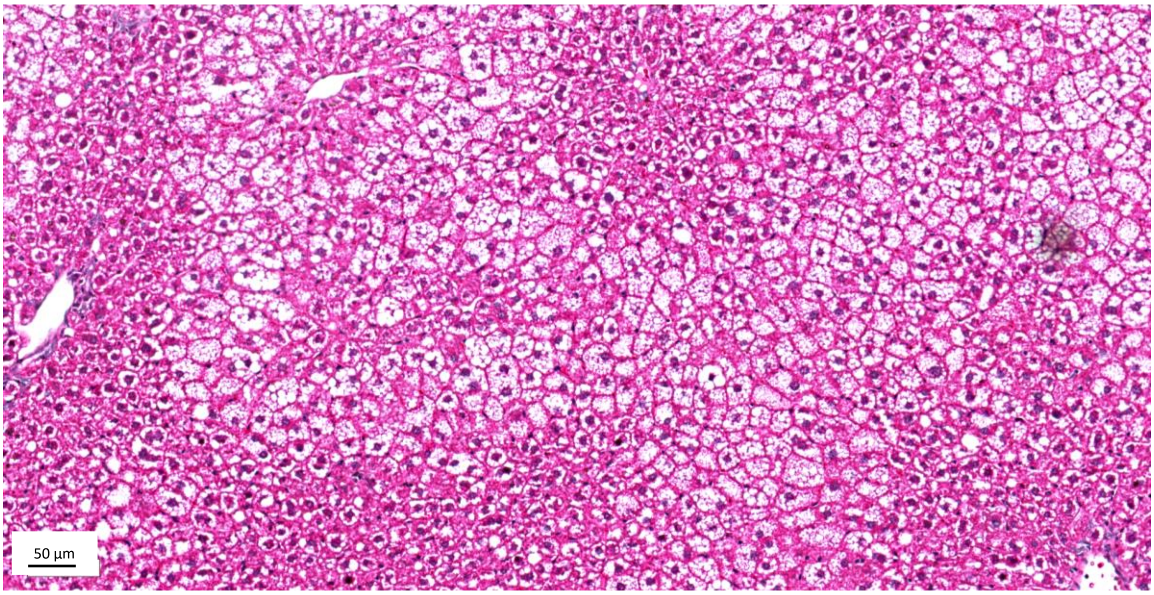

Sirius red Staining

NCD group

(7 mice were included)

NCD-1

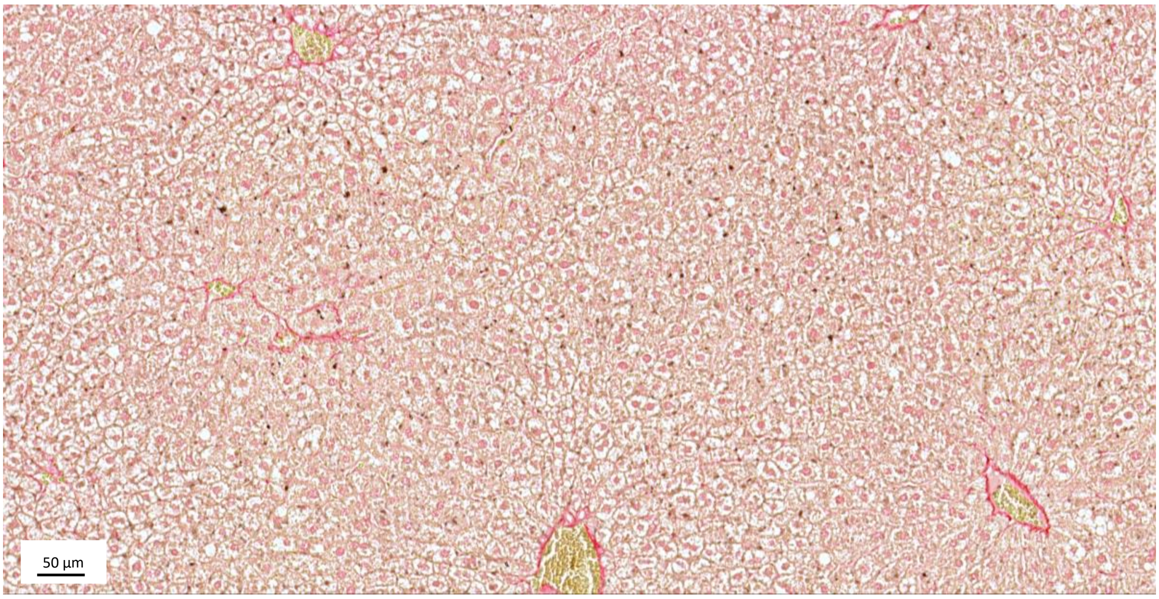

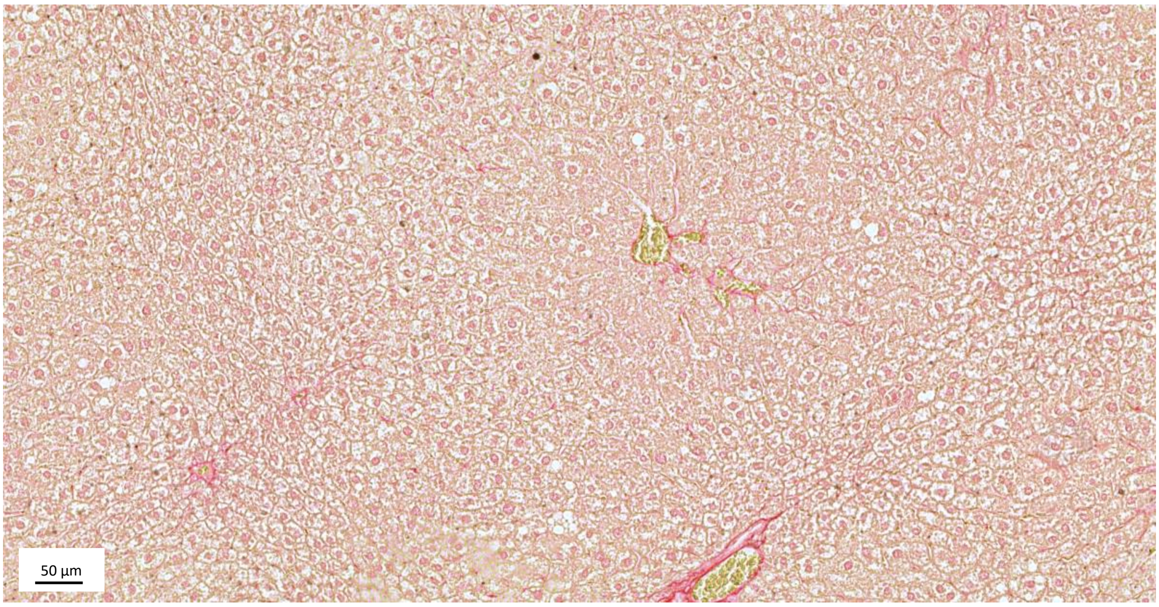

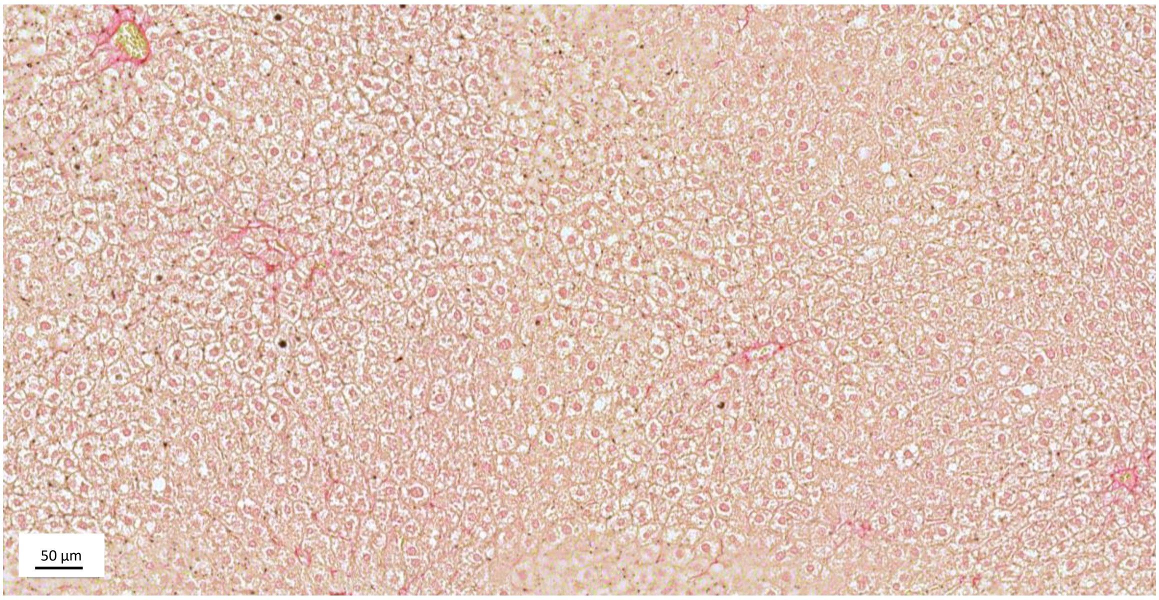

NCD-2

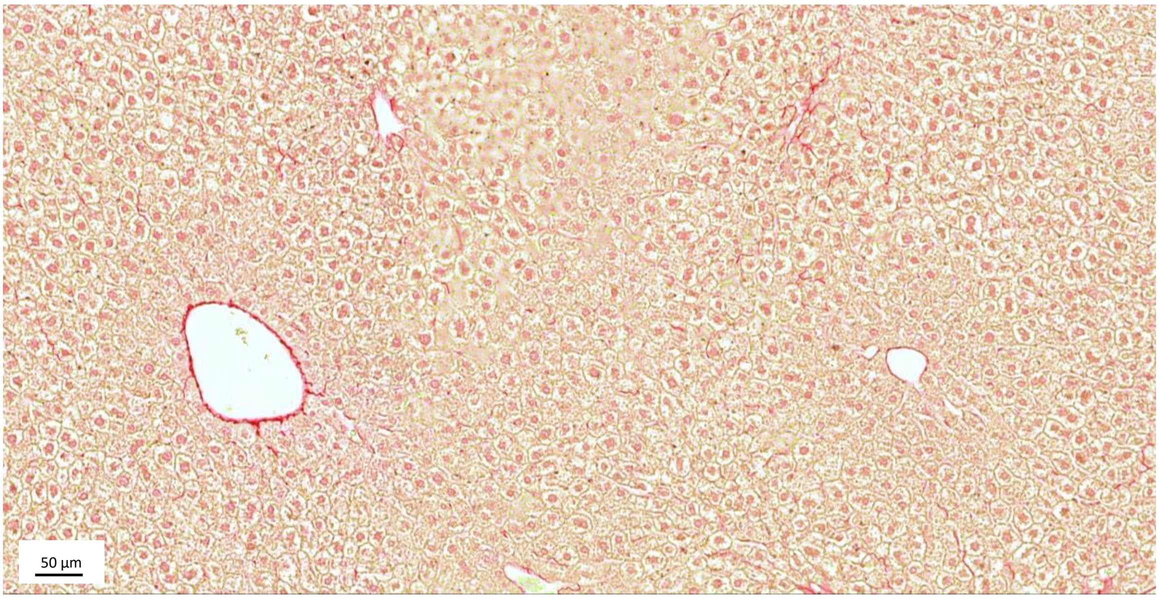

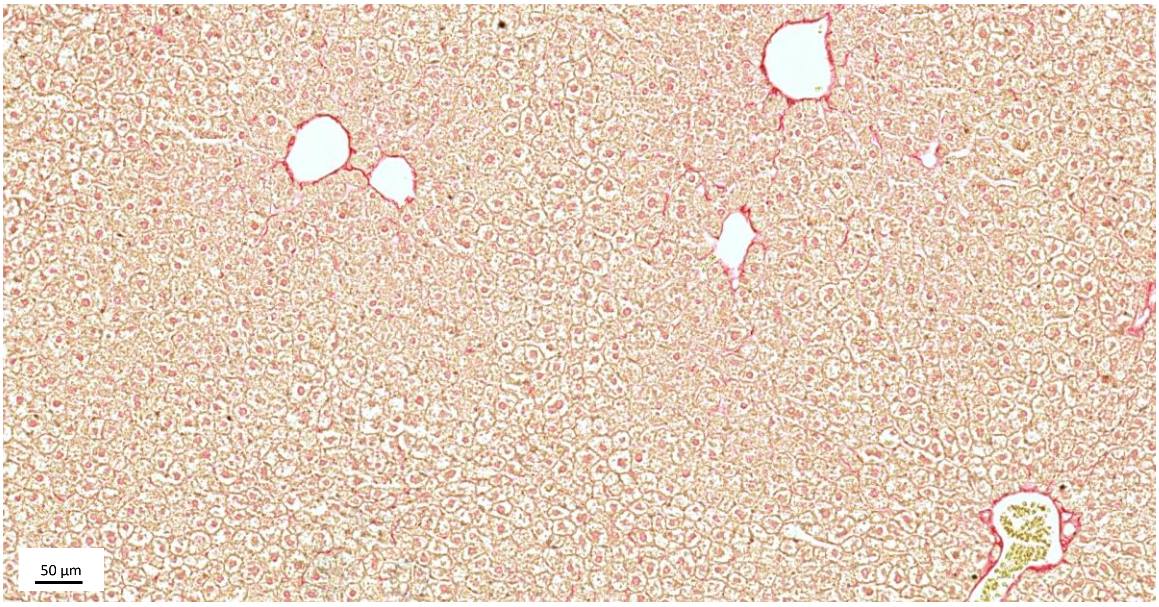

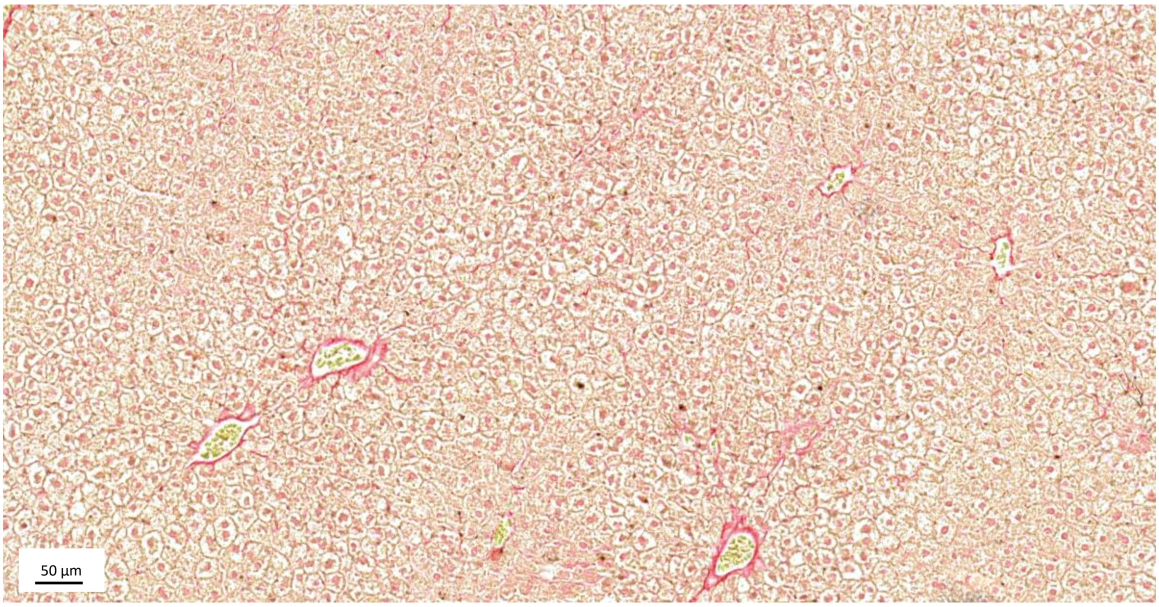

NCD-3

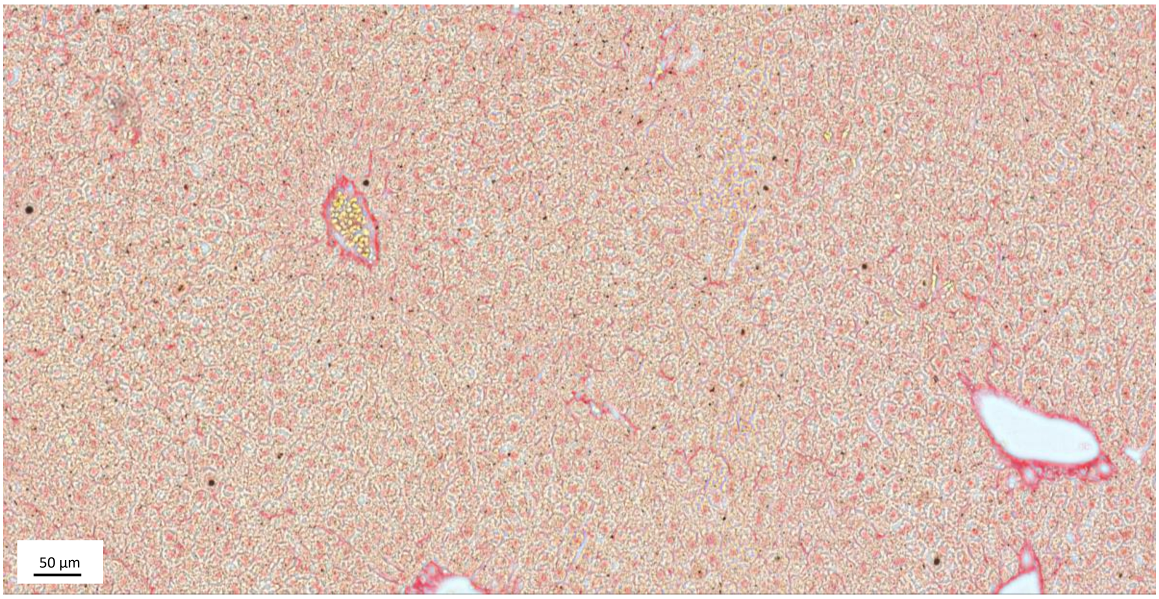

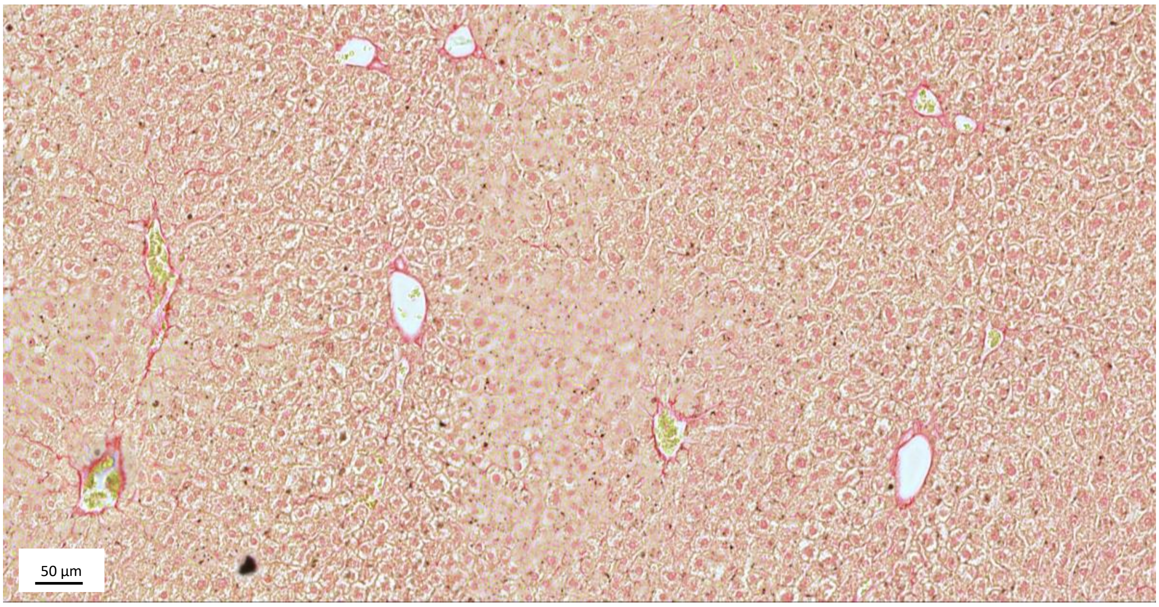

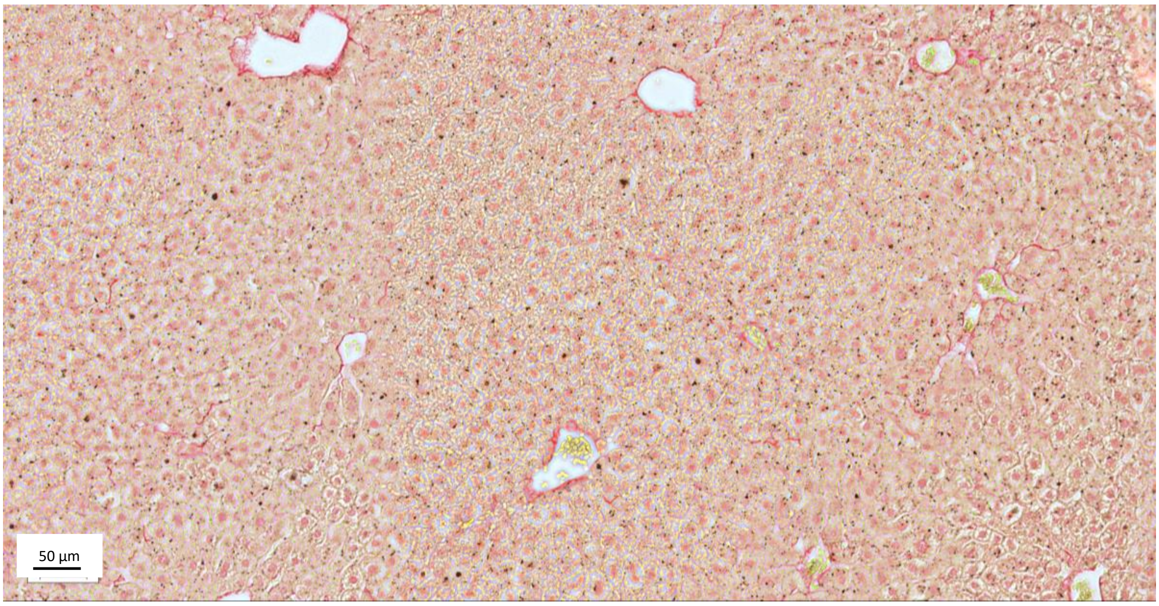

NCD-4

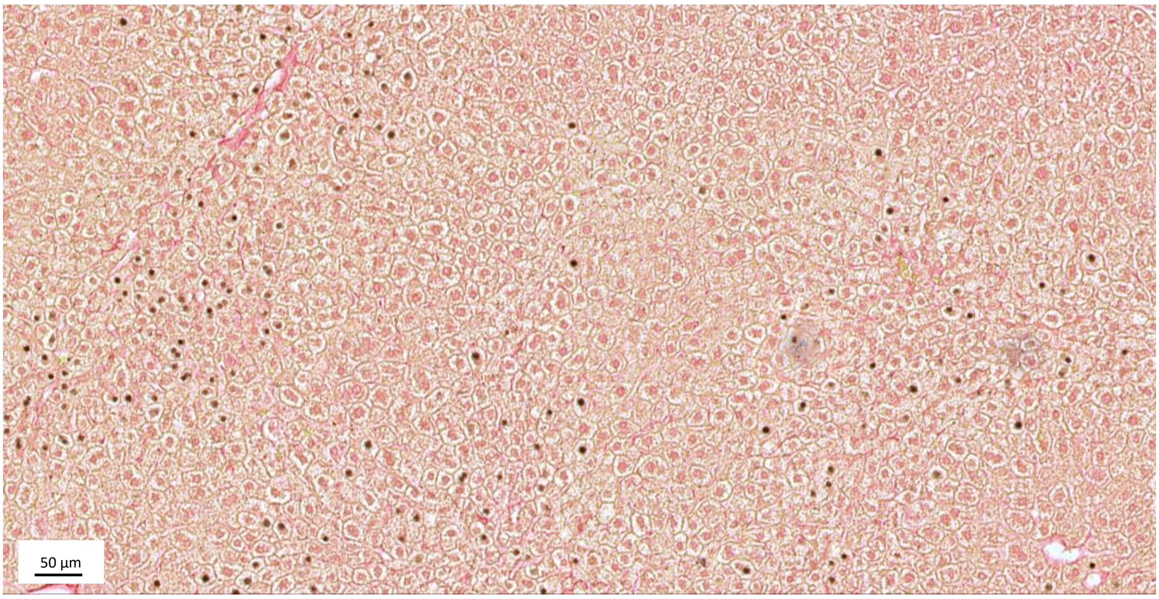

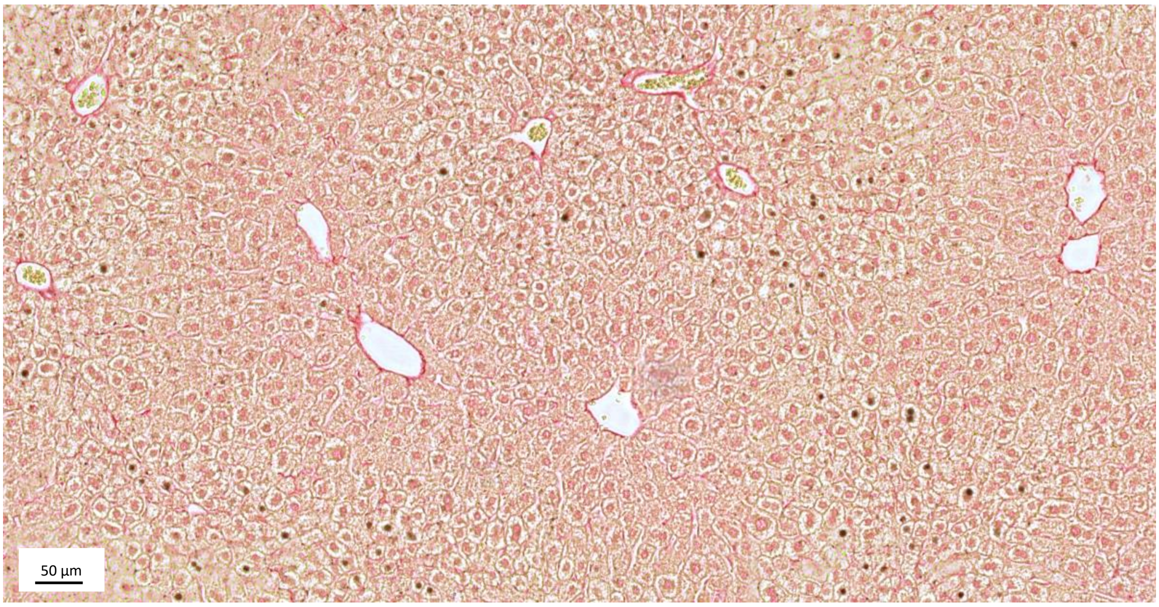

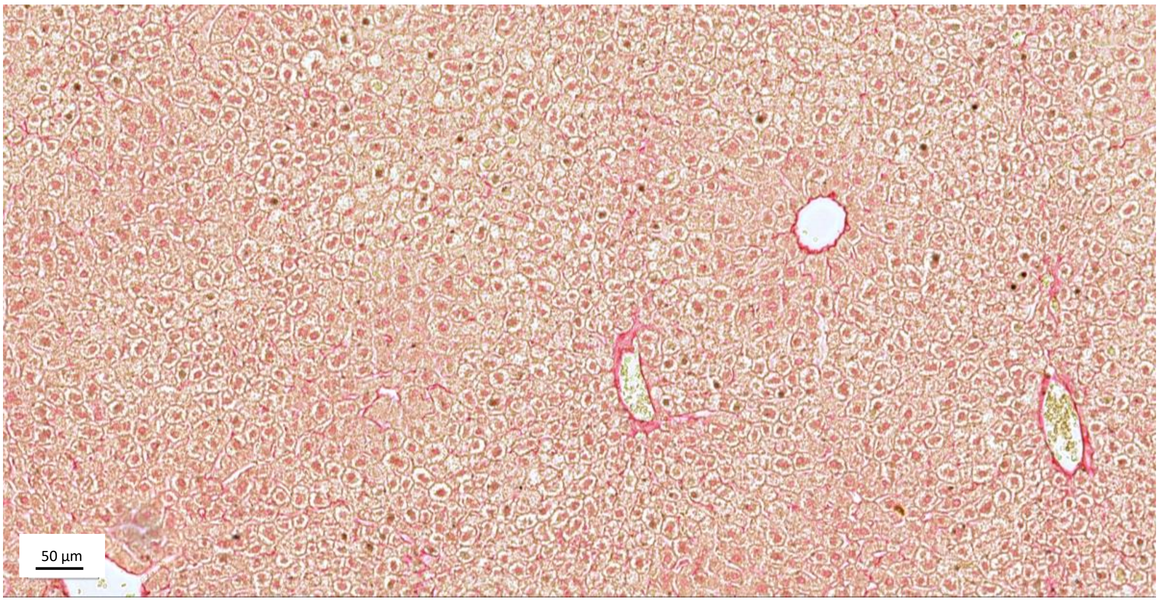

NCD-5

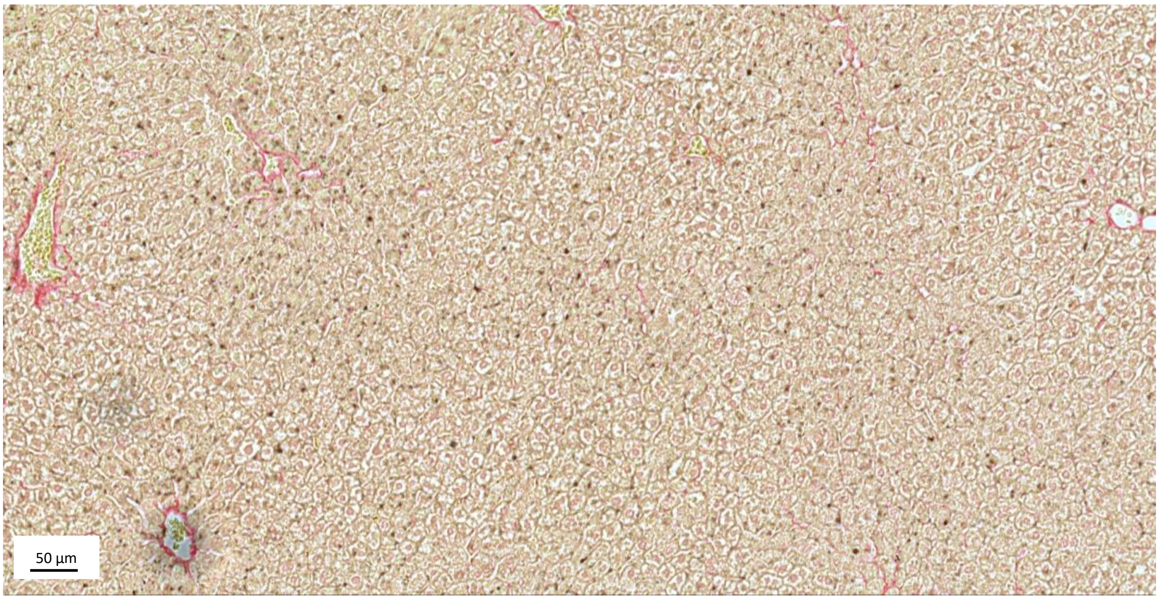

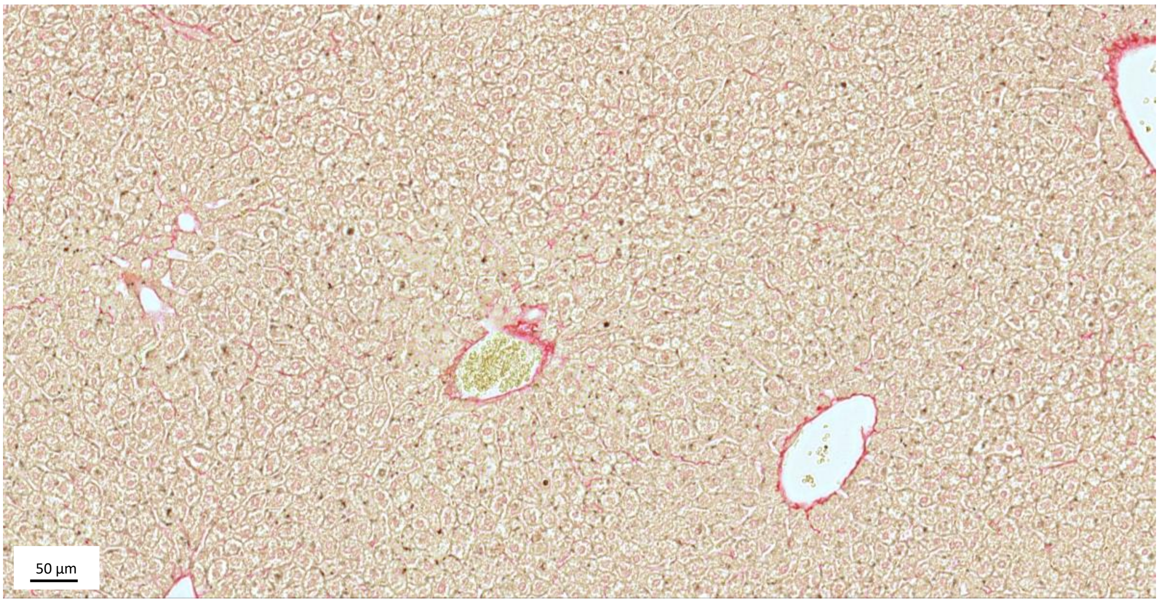

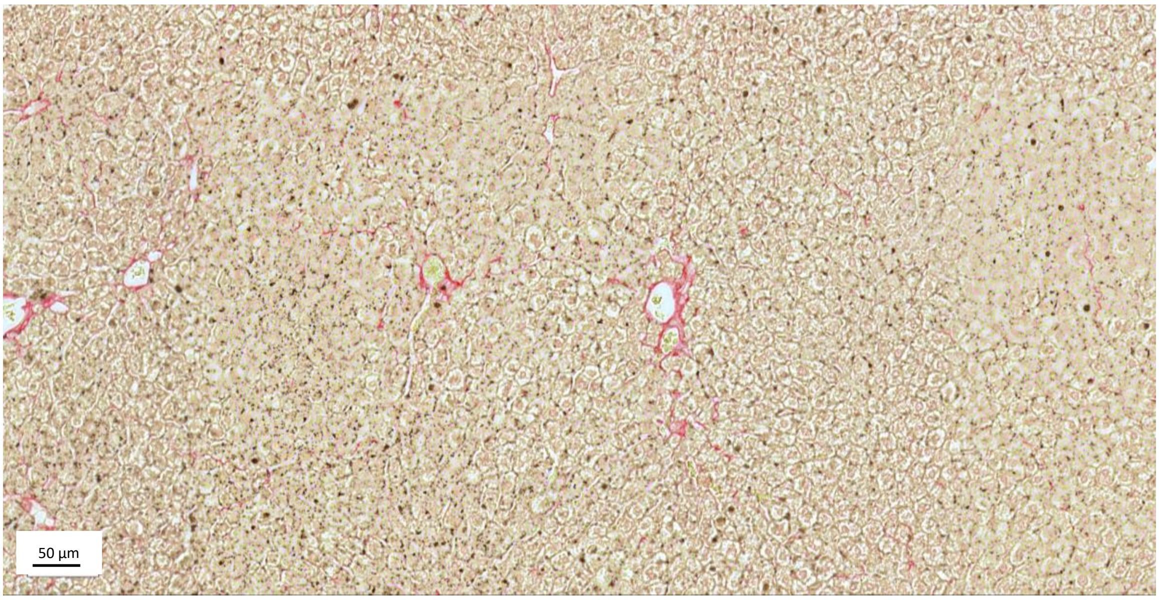

NCD-6

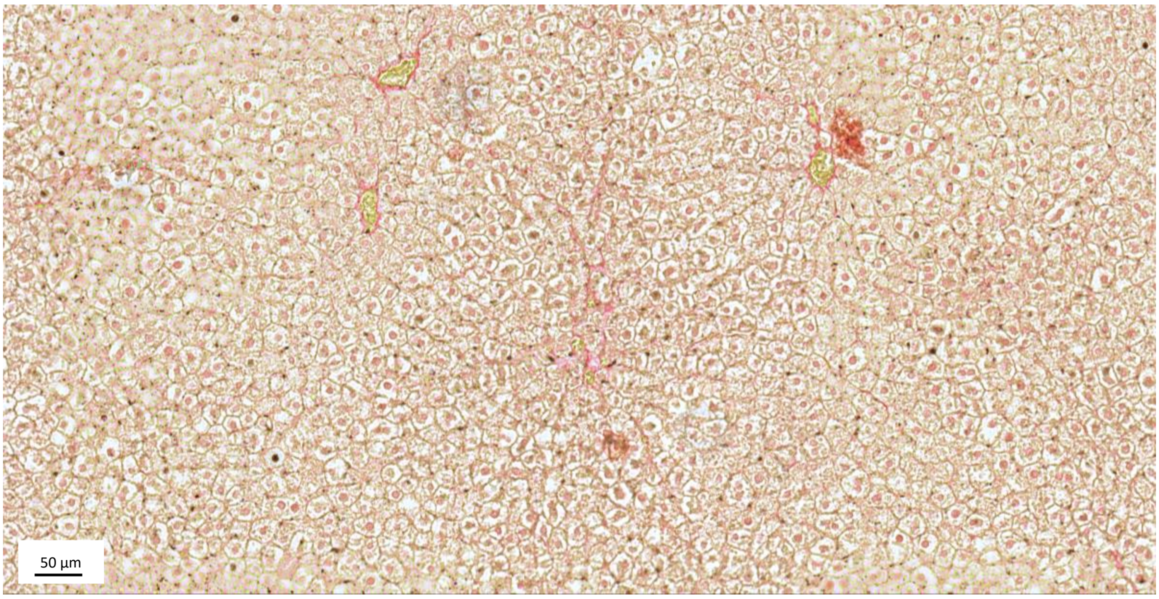

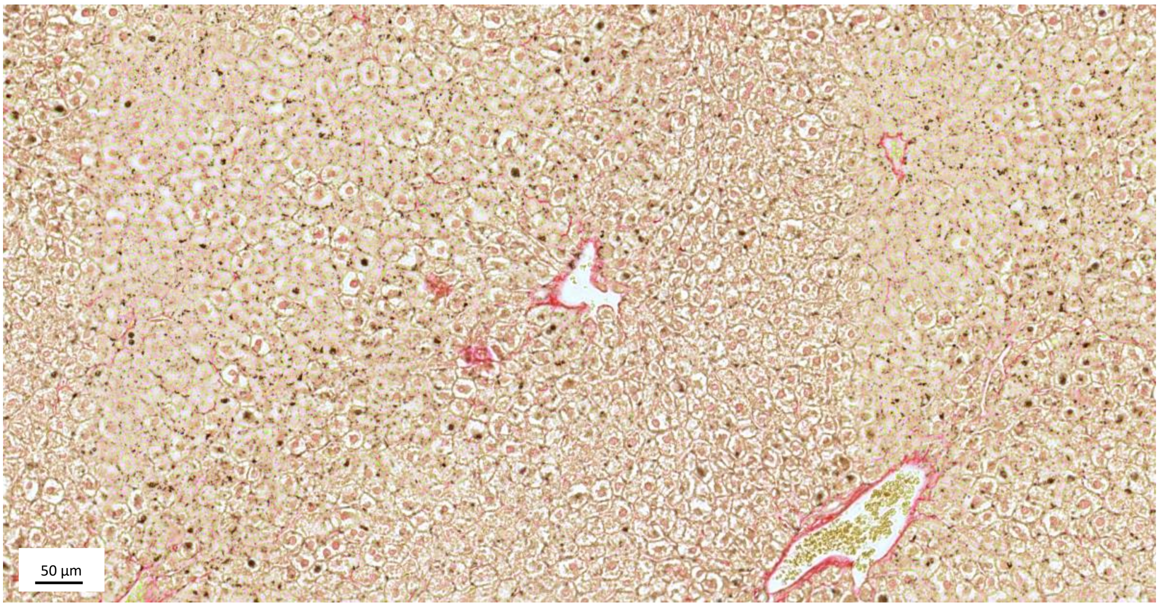

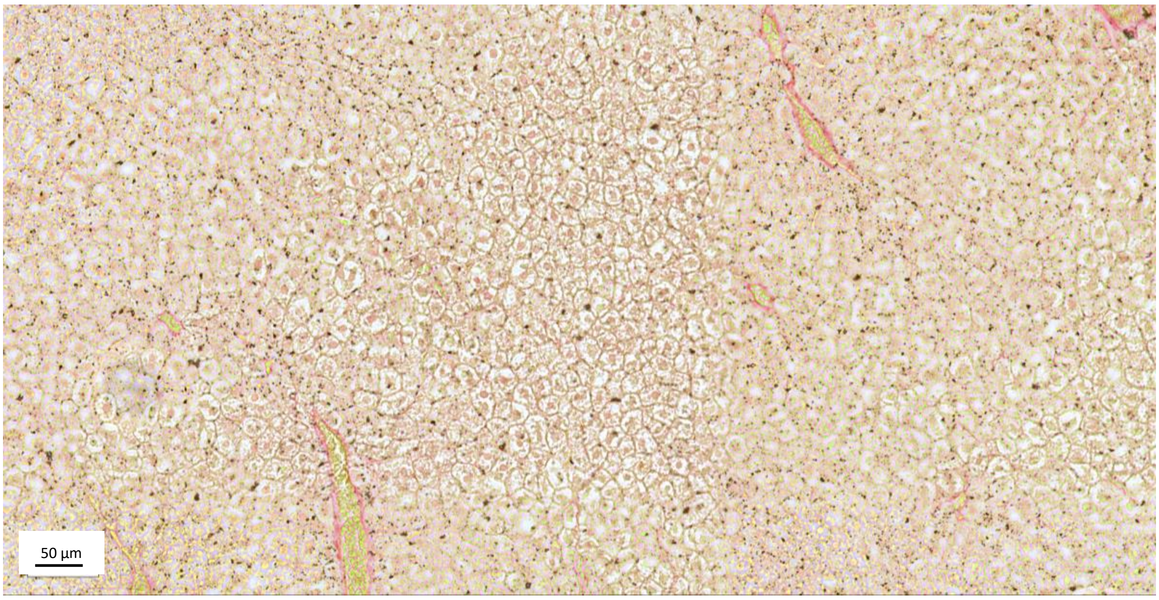

NCD-7

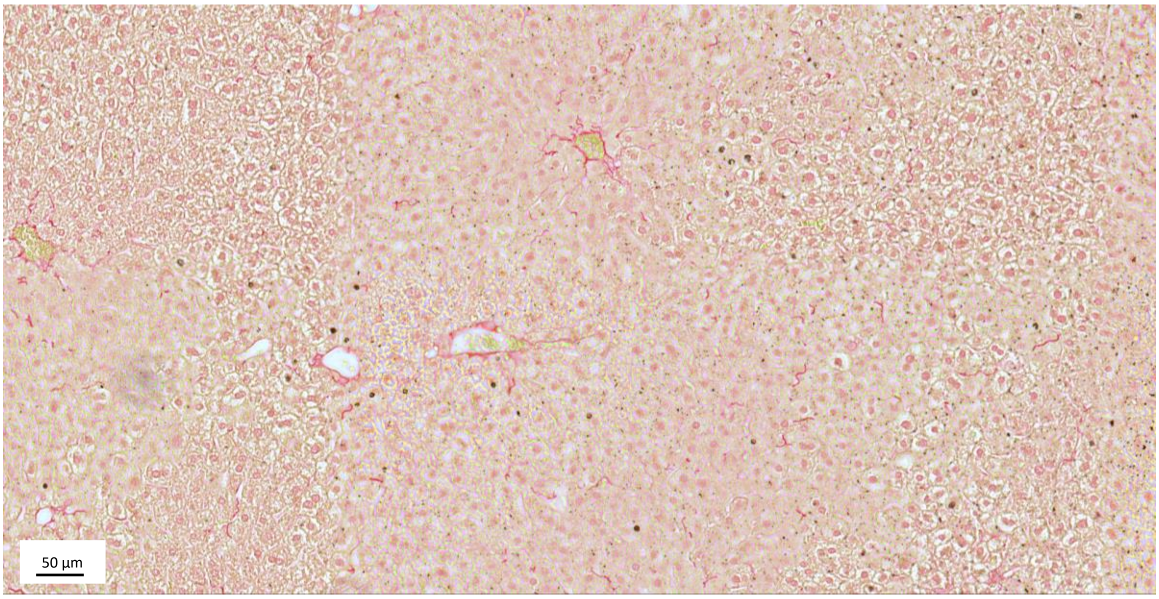

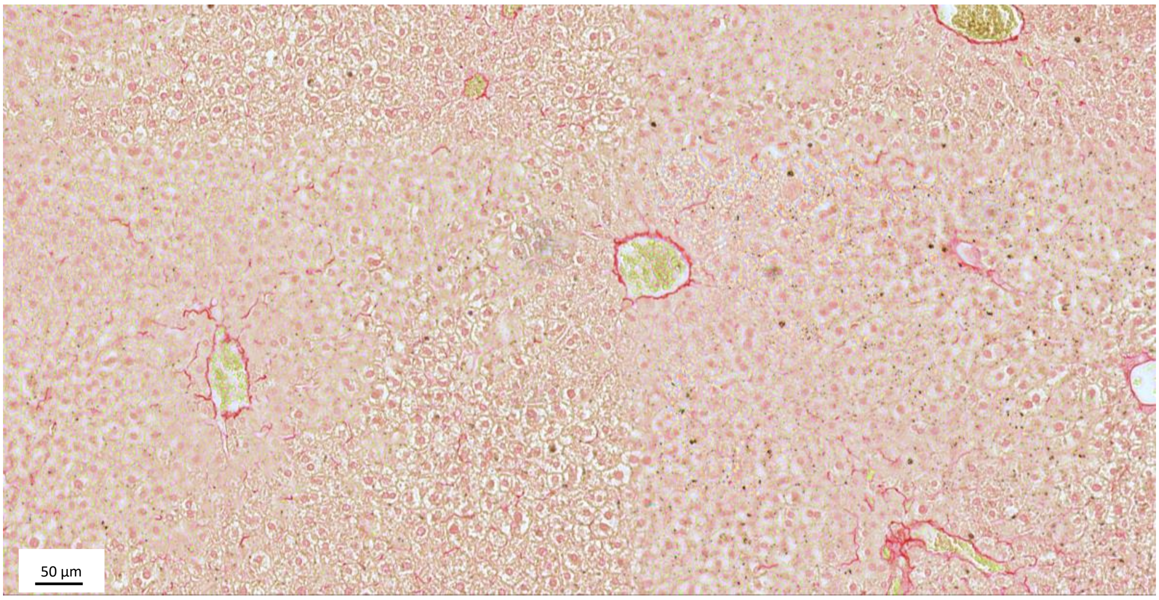

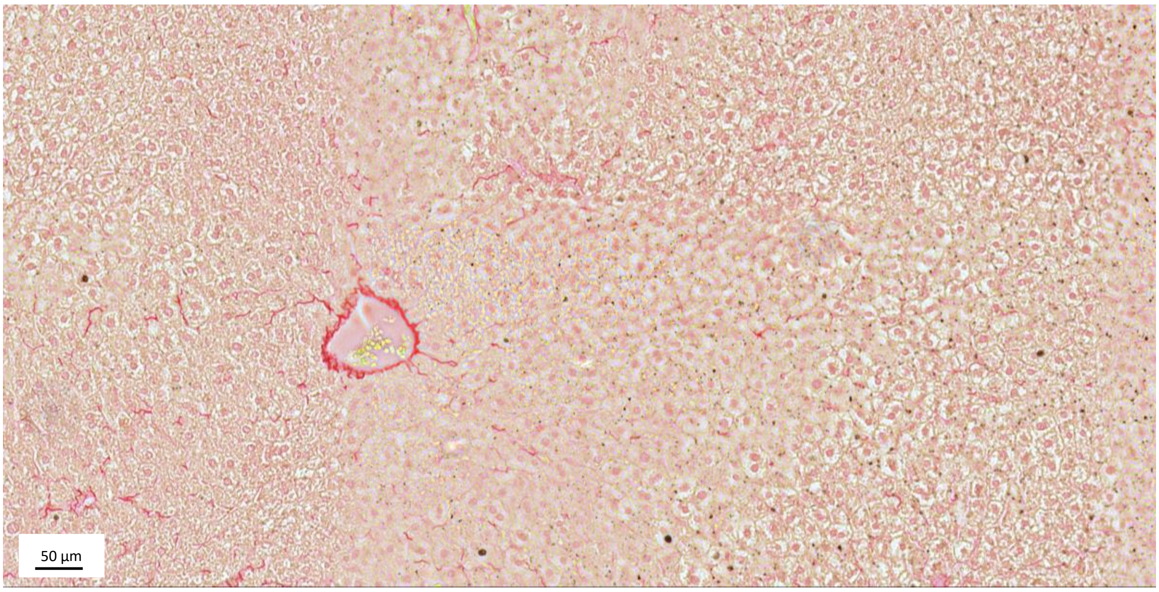

Sirius red Staining

HFHCD group

(15 mice were included)

HFHCD-1

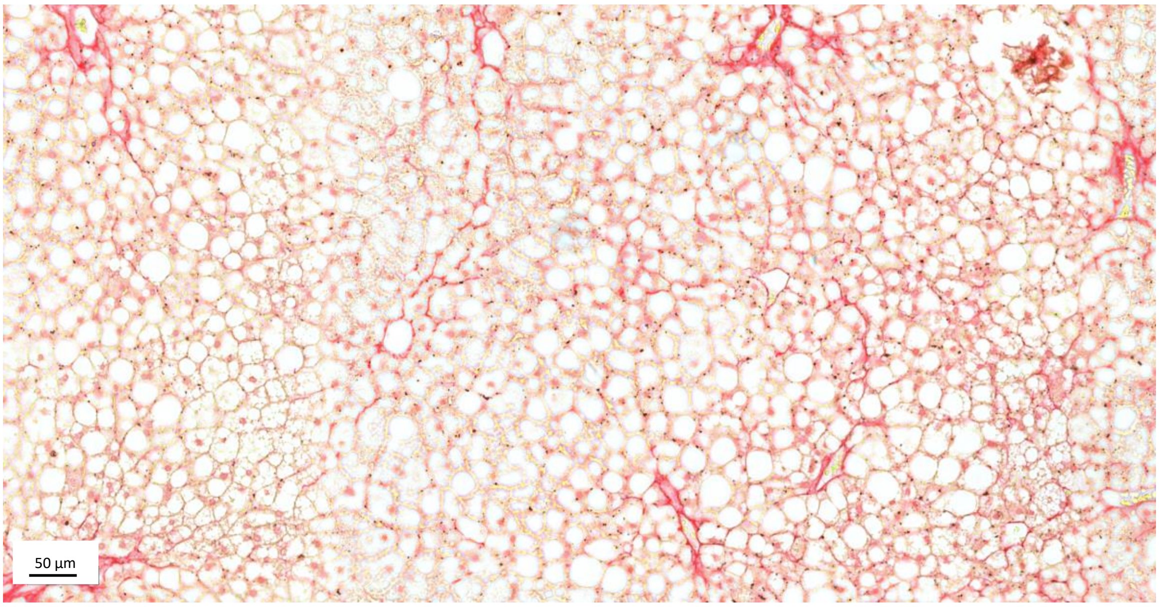

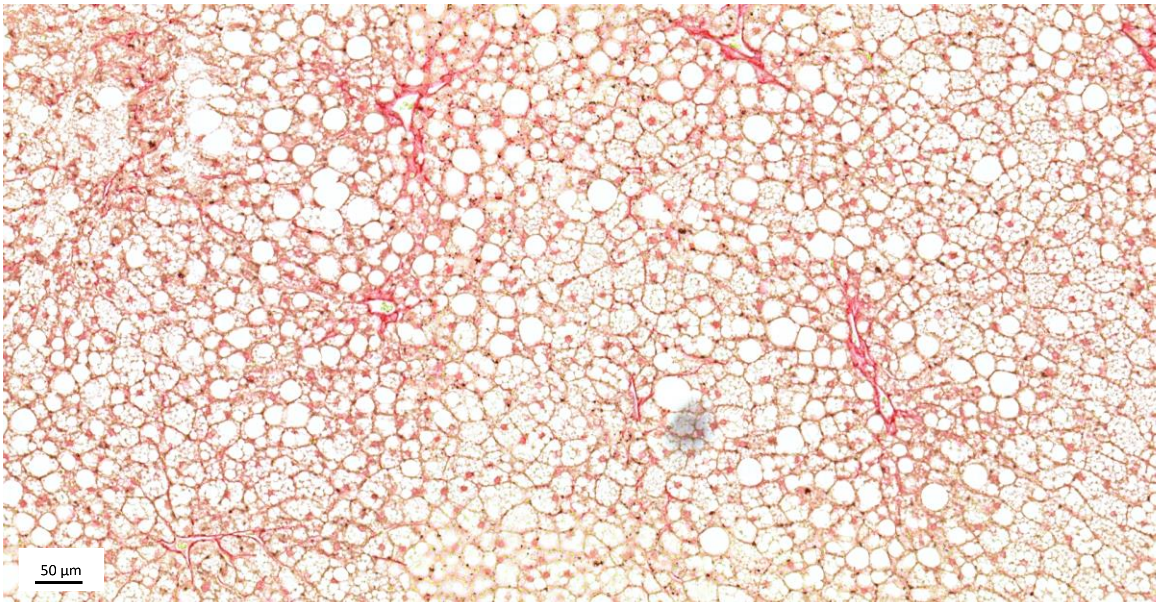

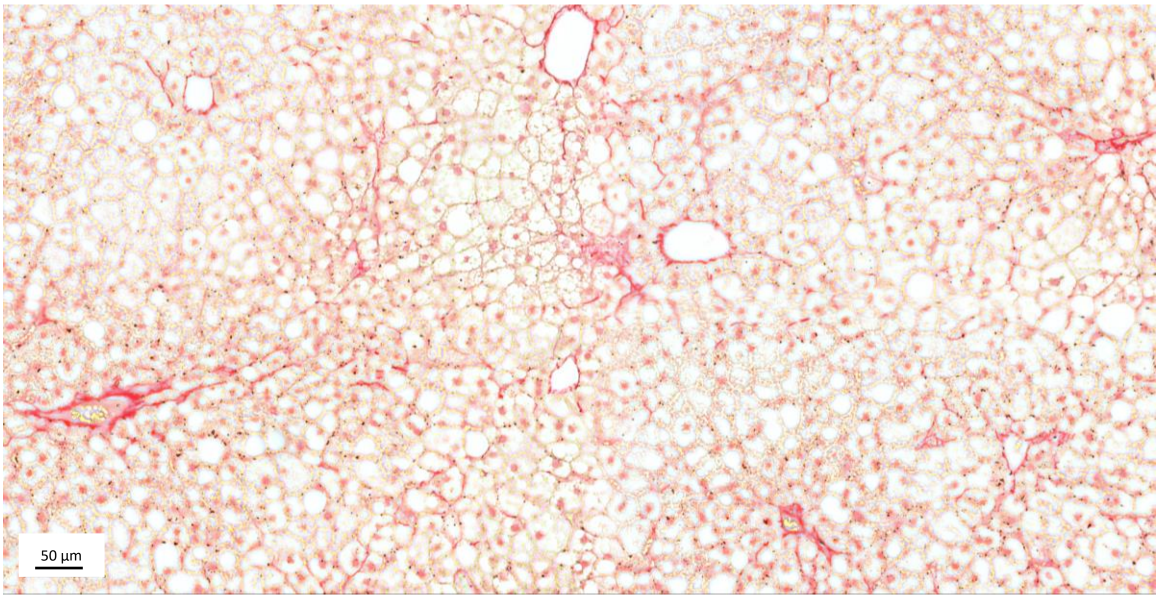

HFHCD-2

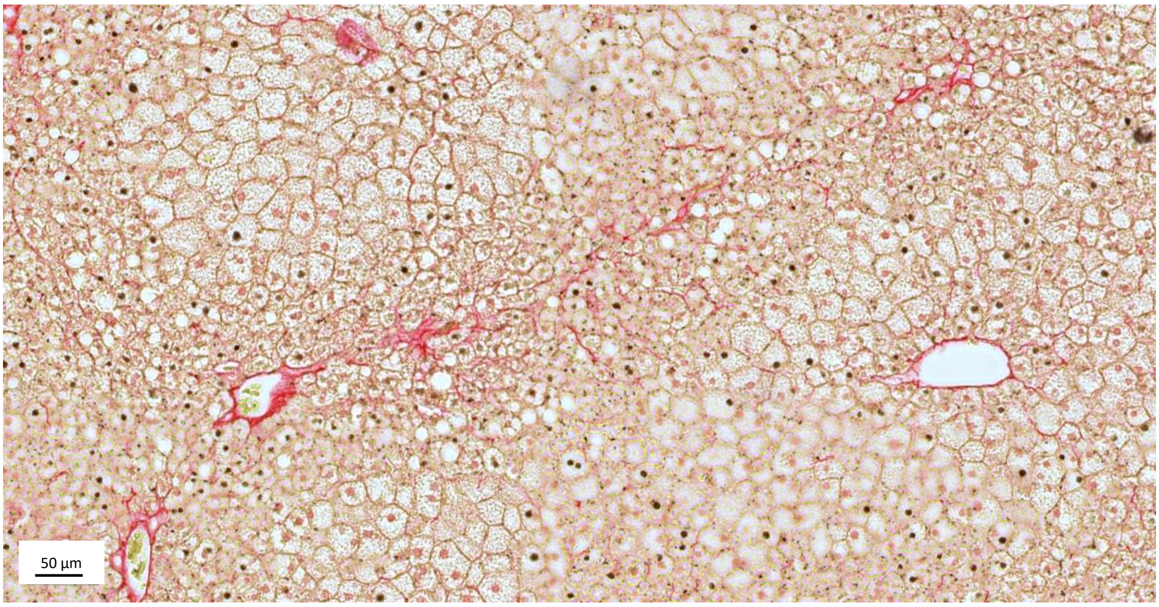

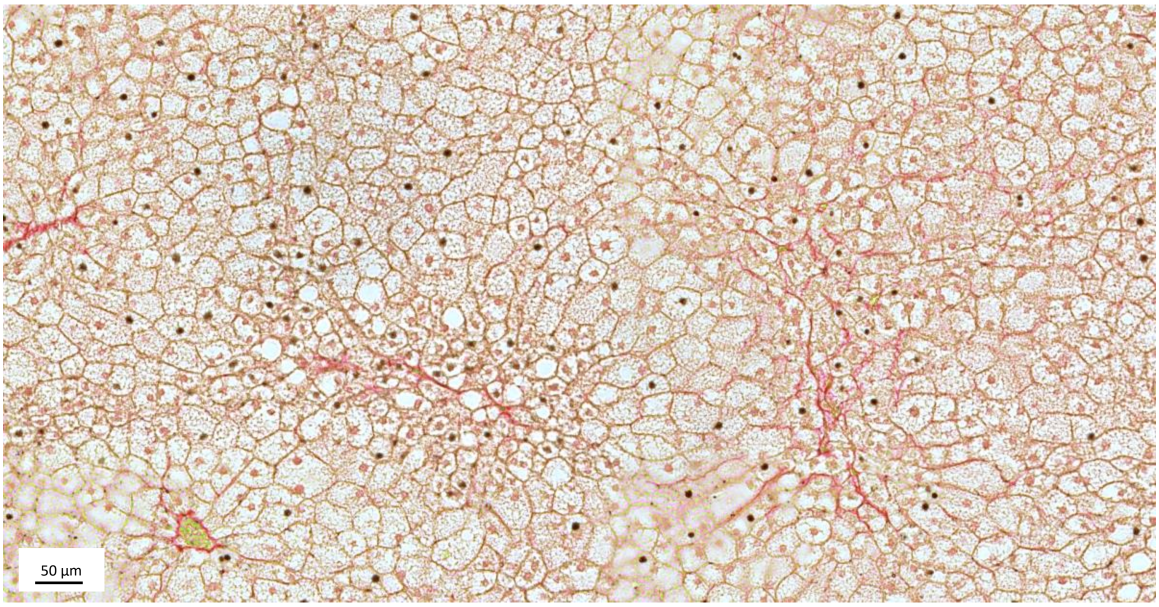

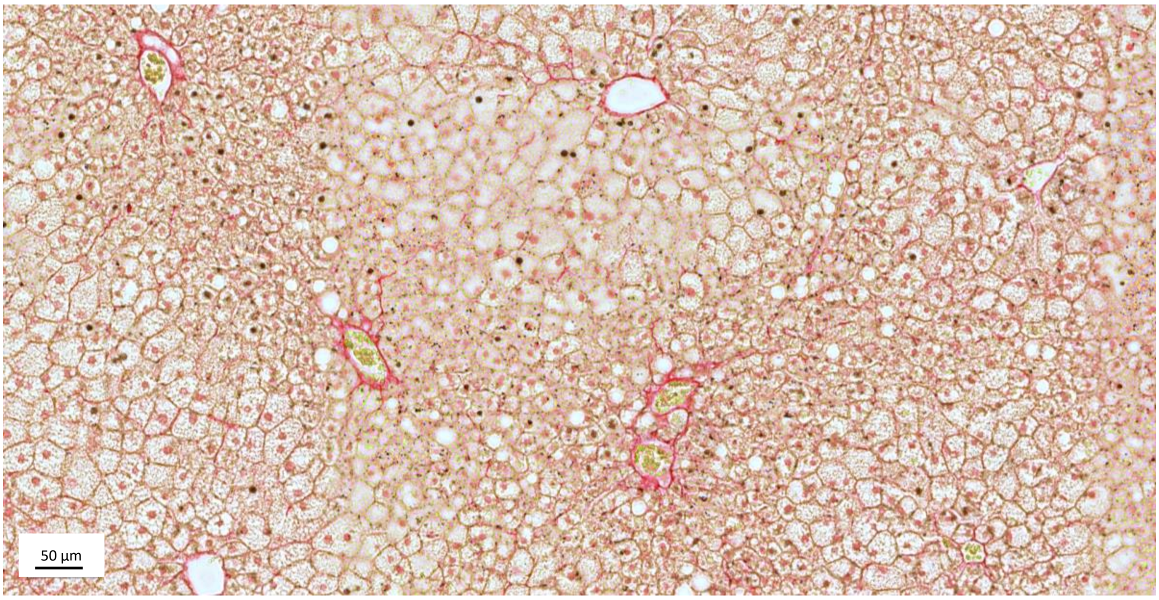

HFHCD-3

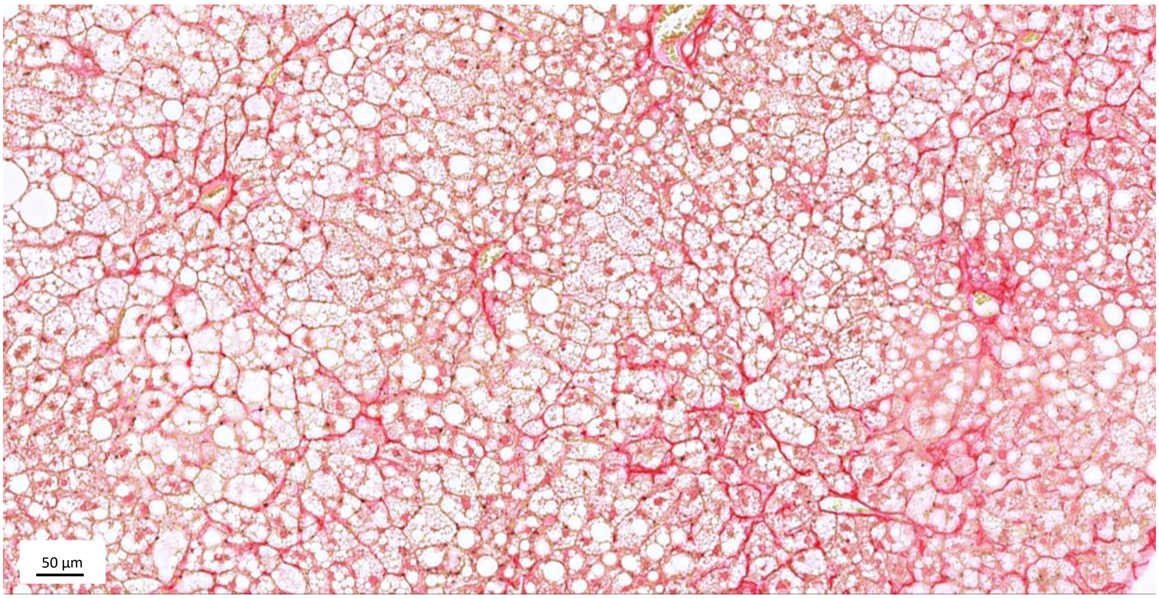

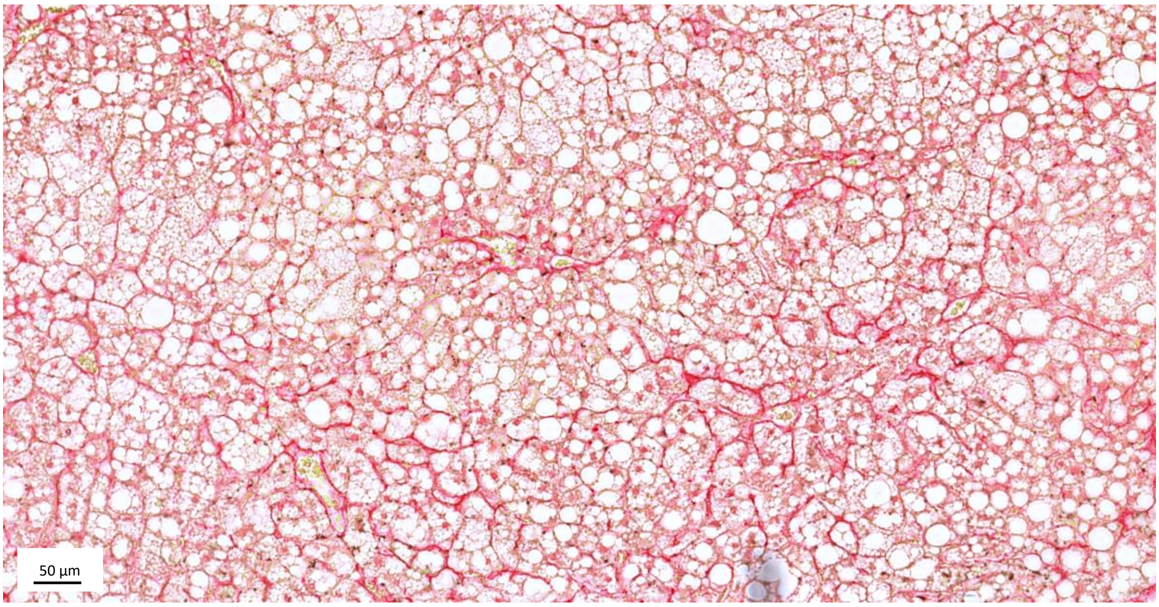

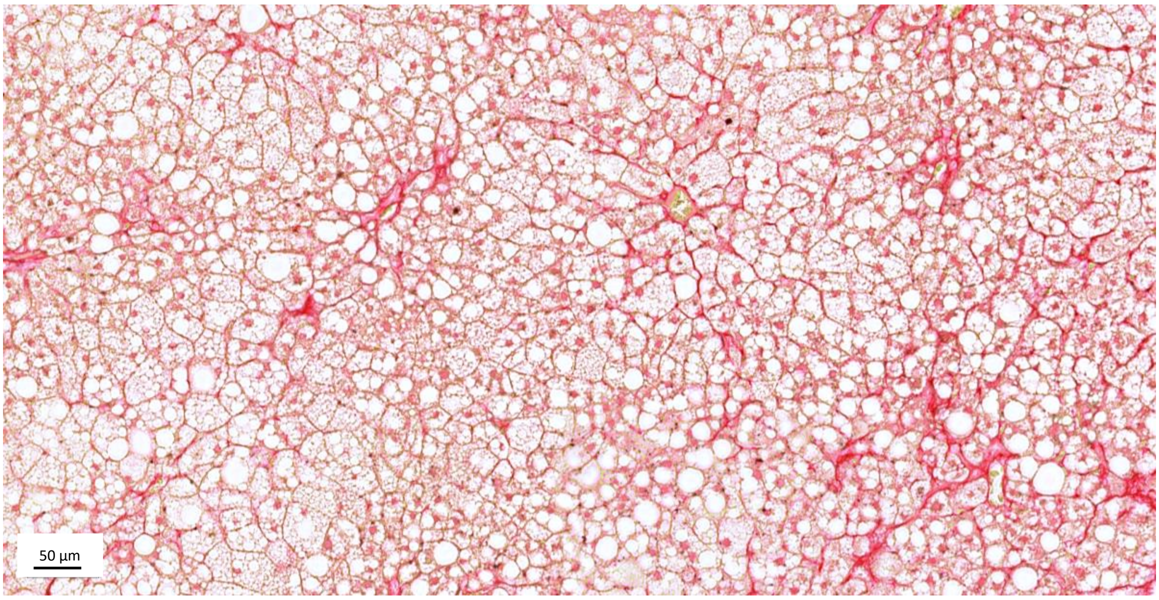

HFHCD-4

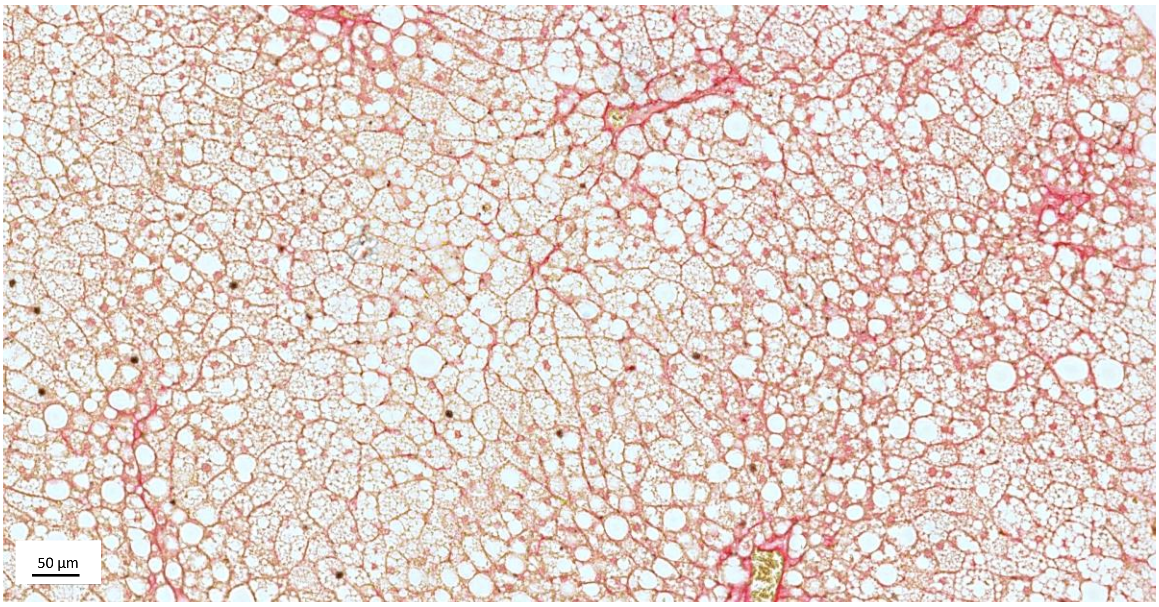

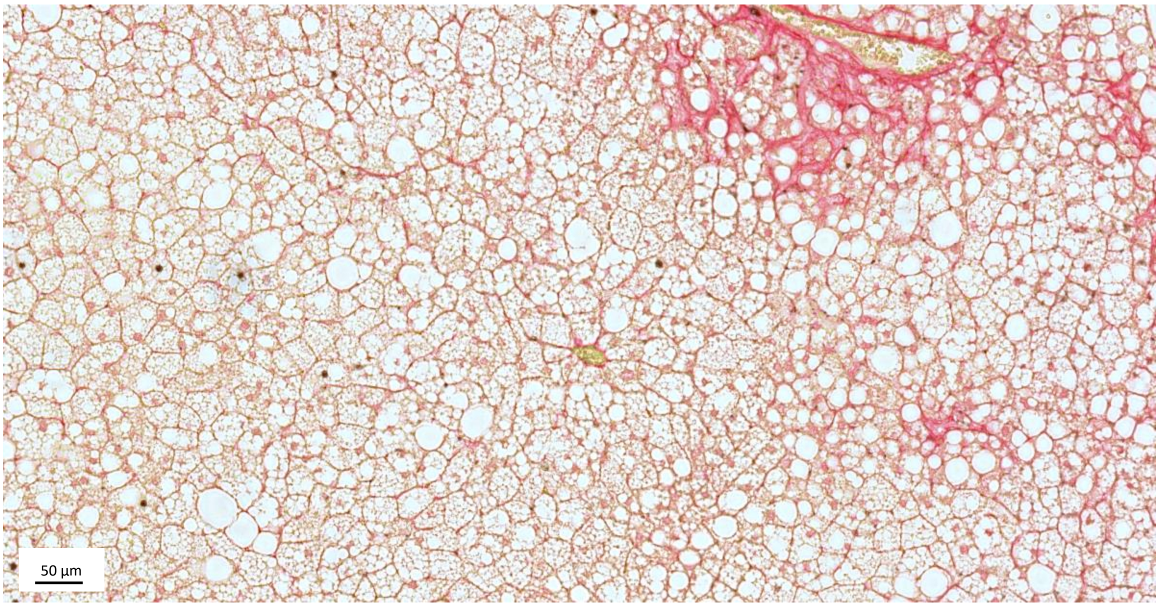

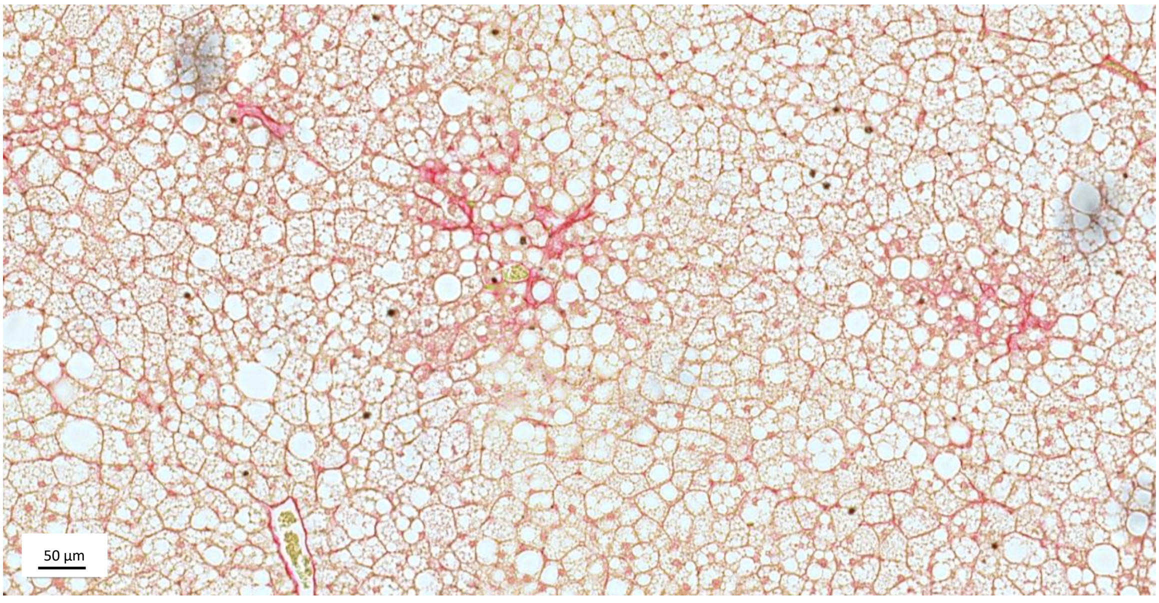

HFHCD-5

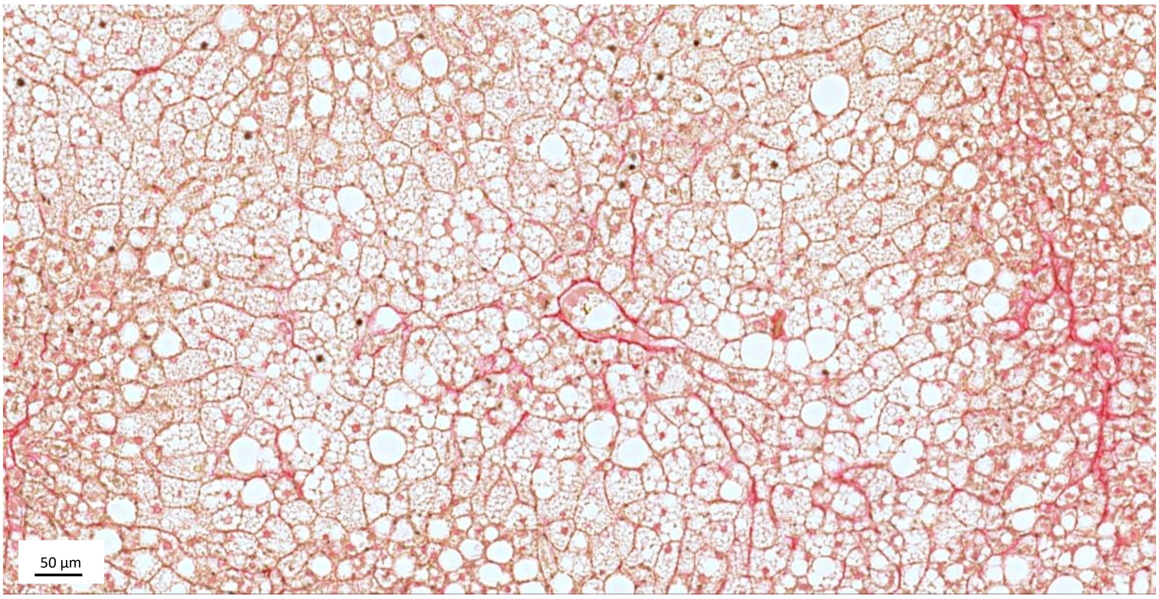

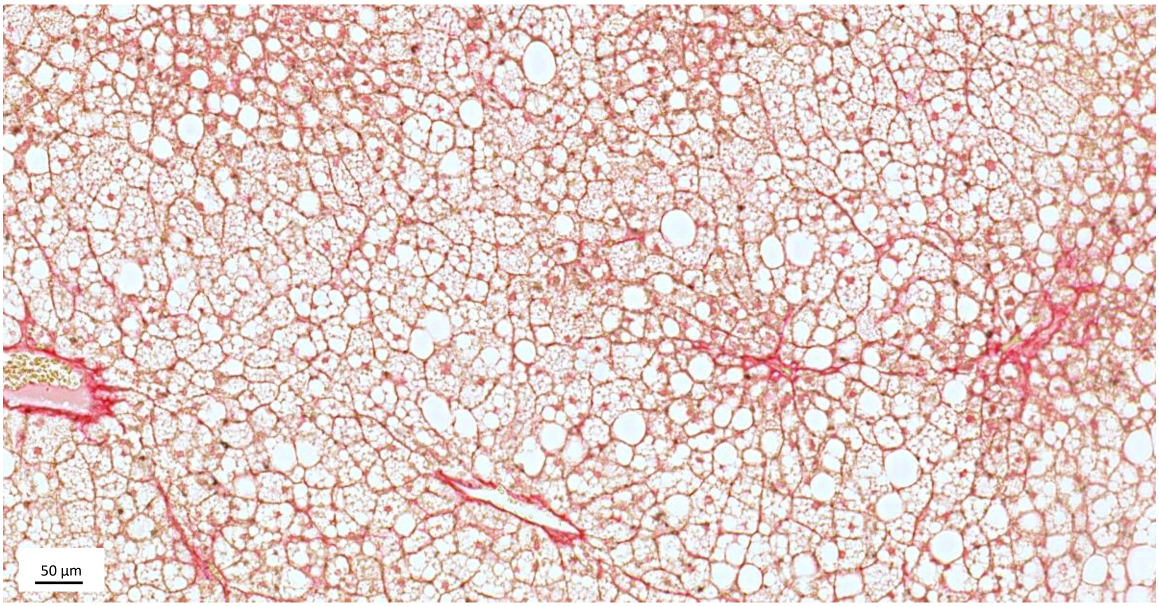

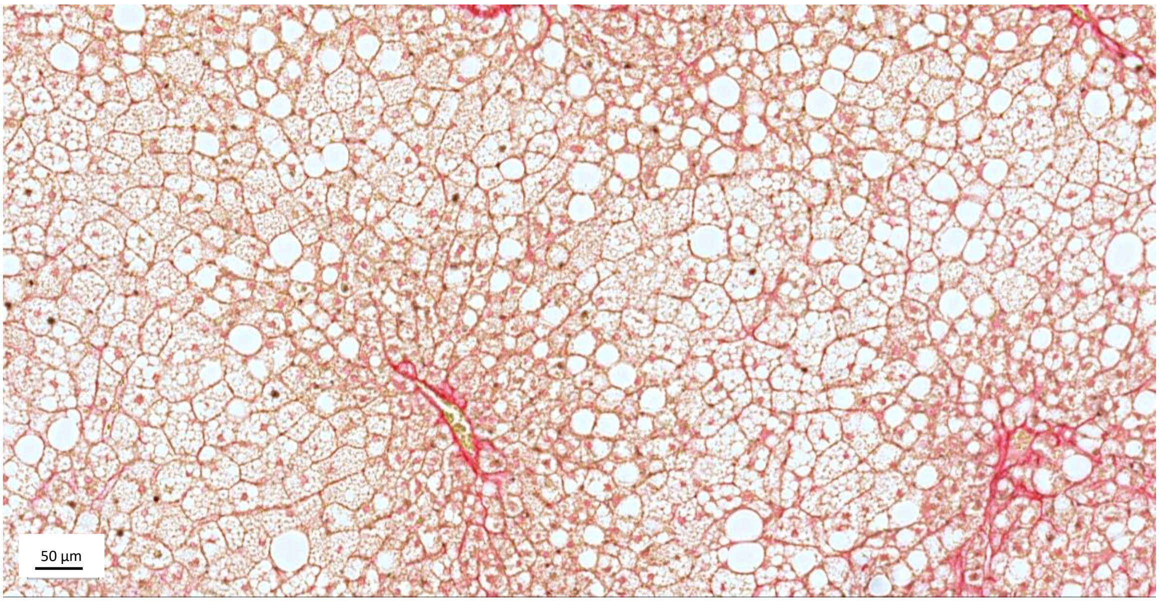

HFHCD-6

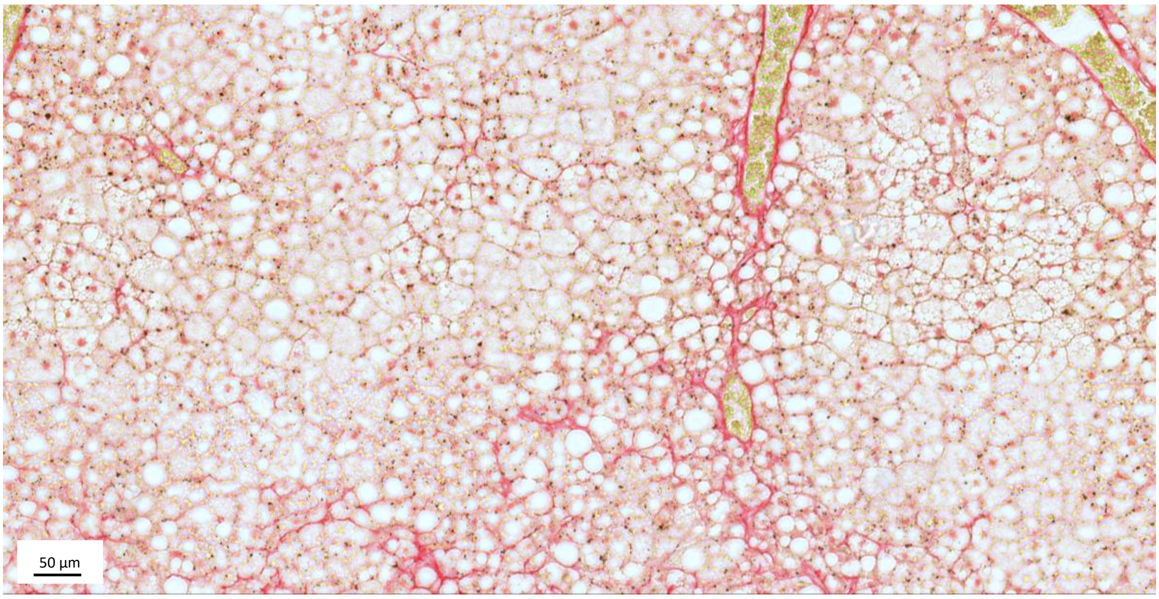

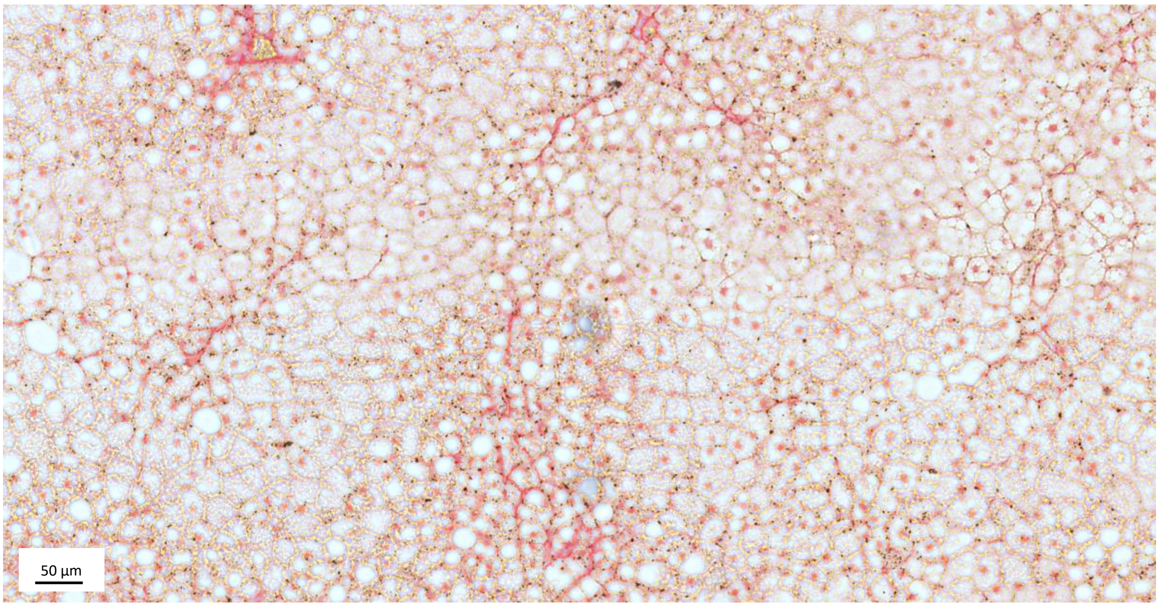

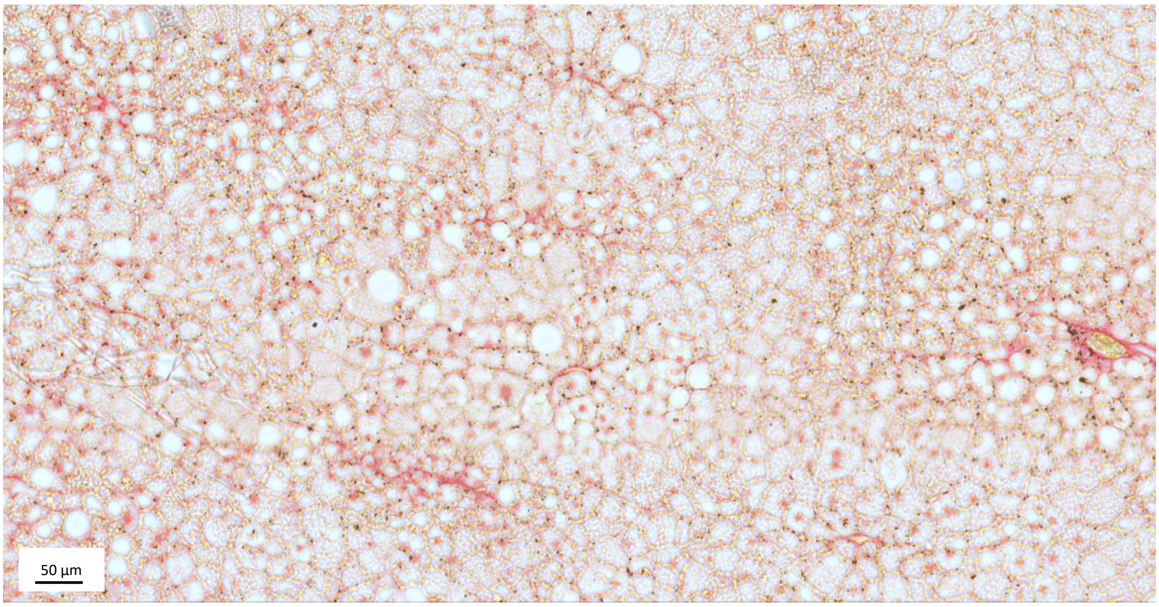

HFHCD-7

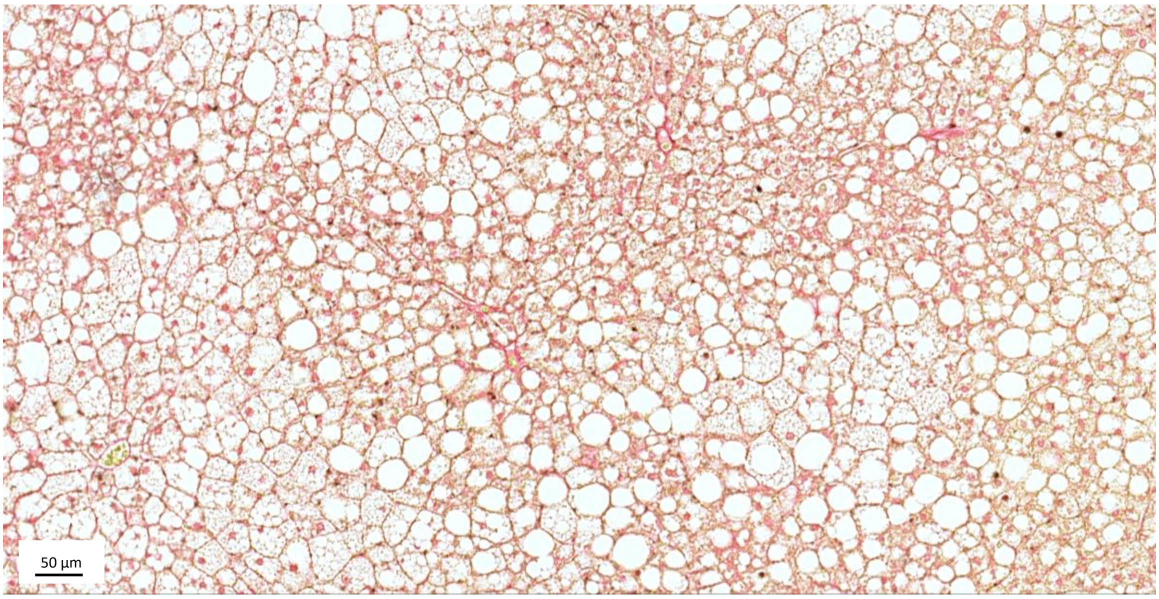

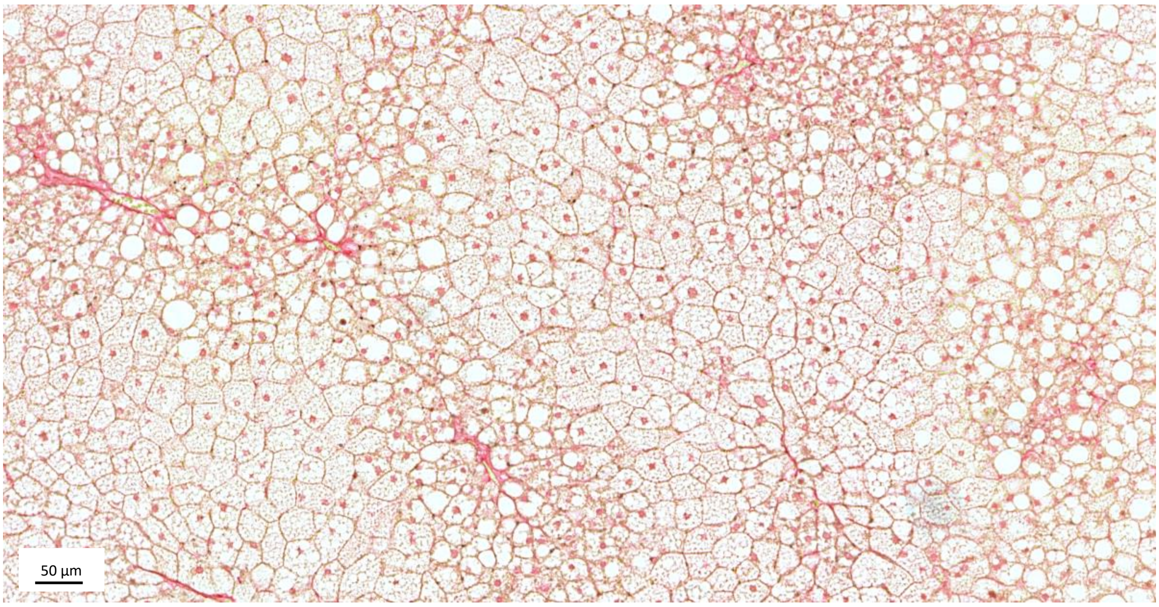

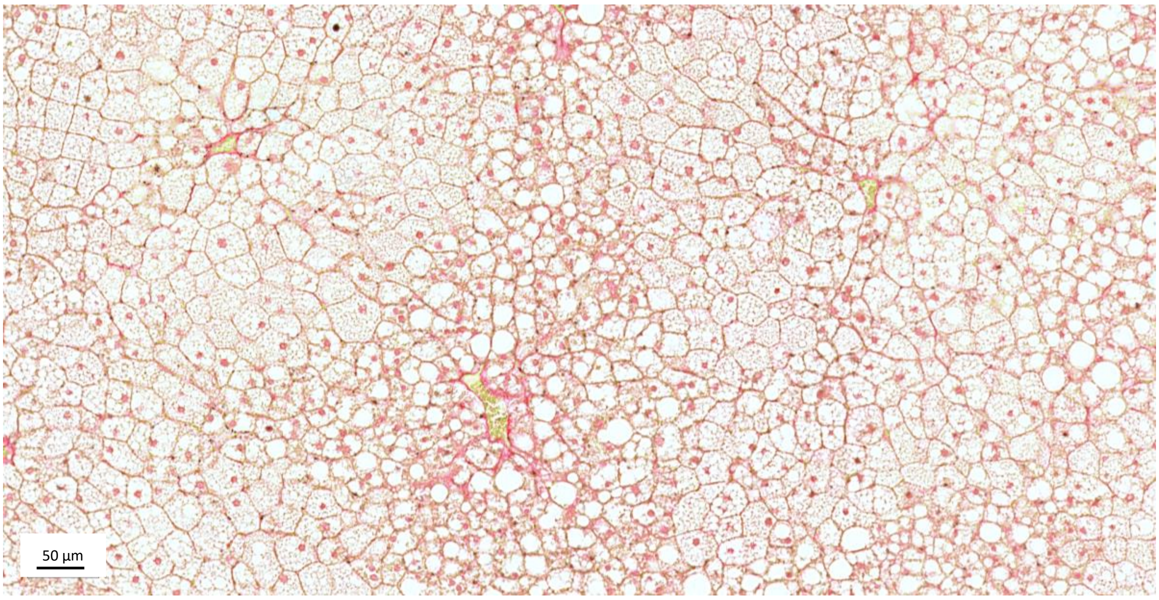

HFHCD-8

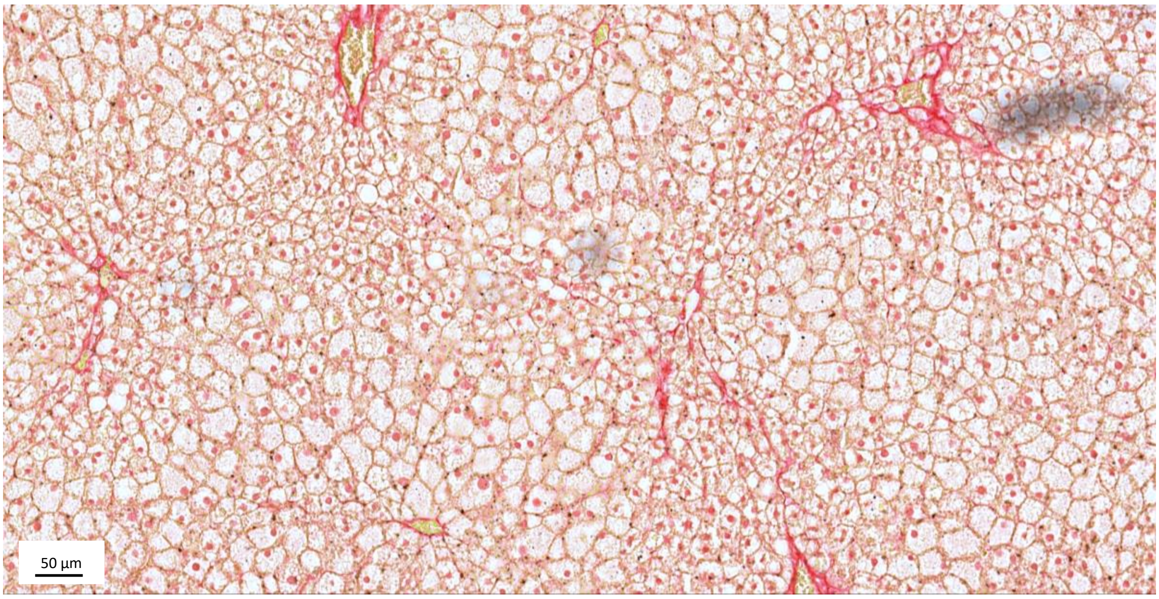

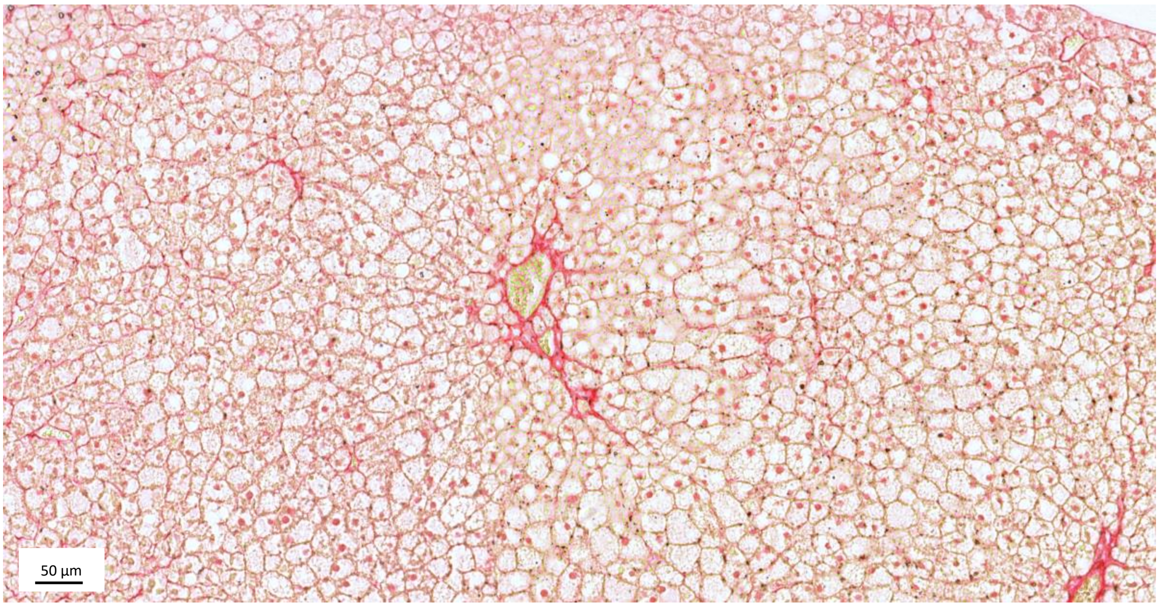

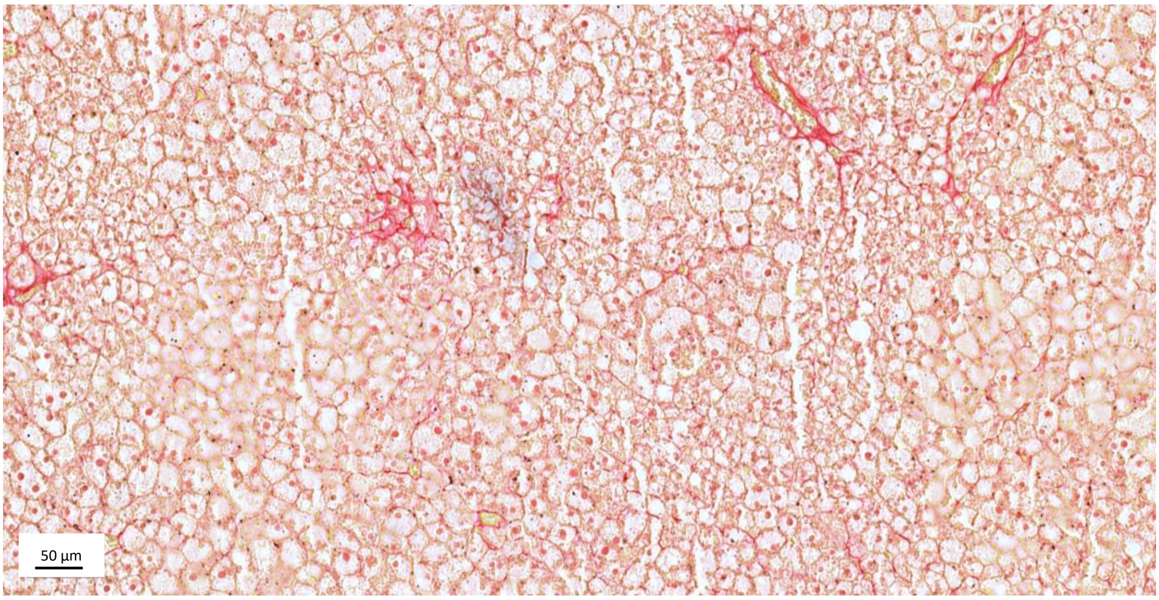

HFHCD-9

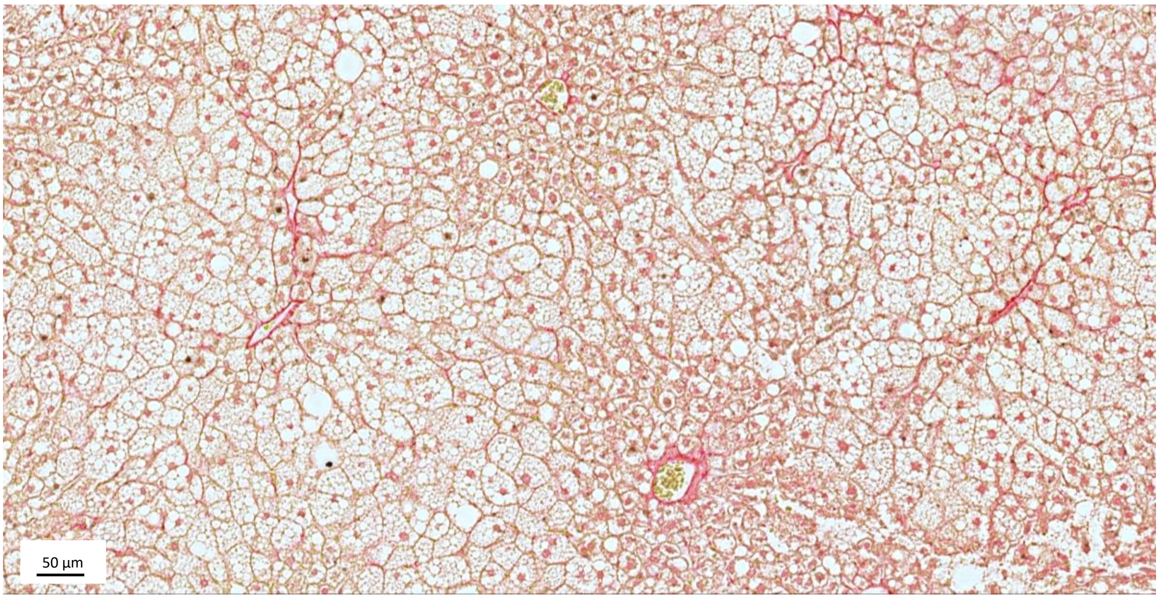

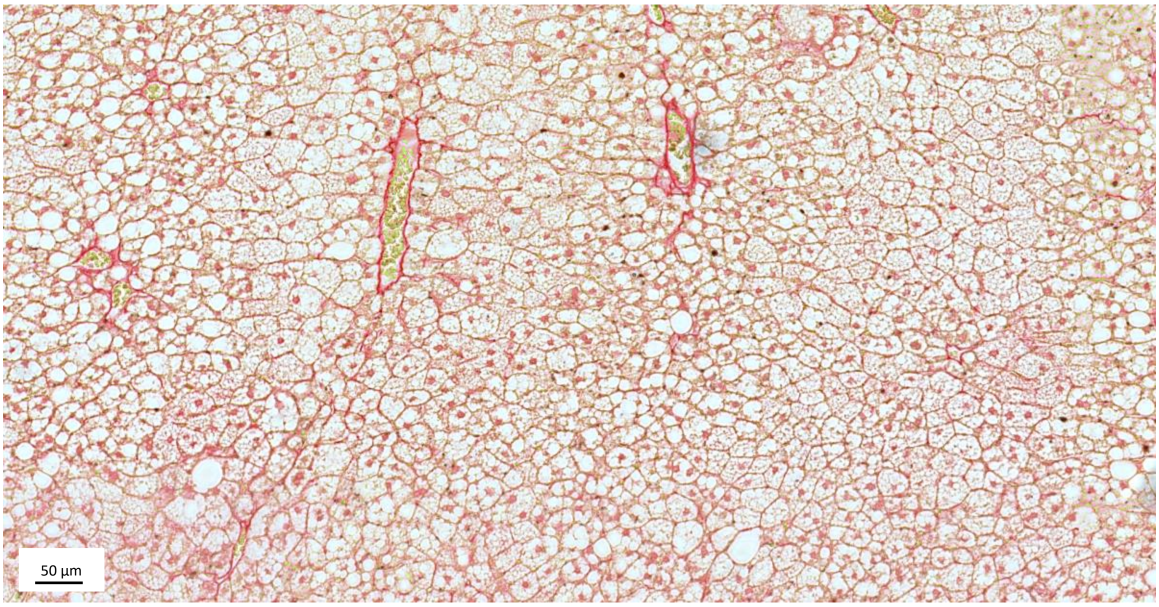

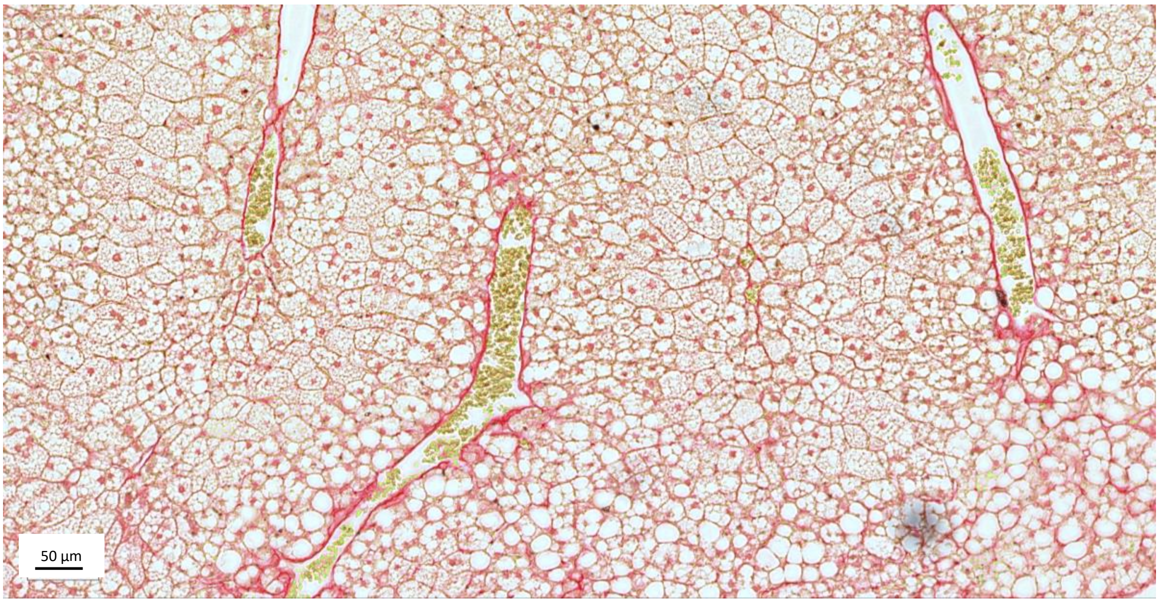

HFHCD-10

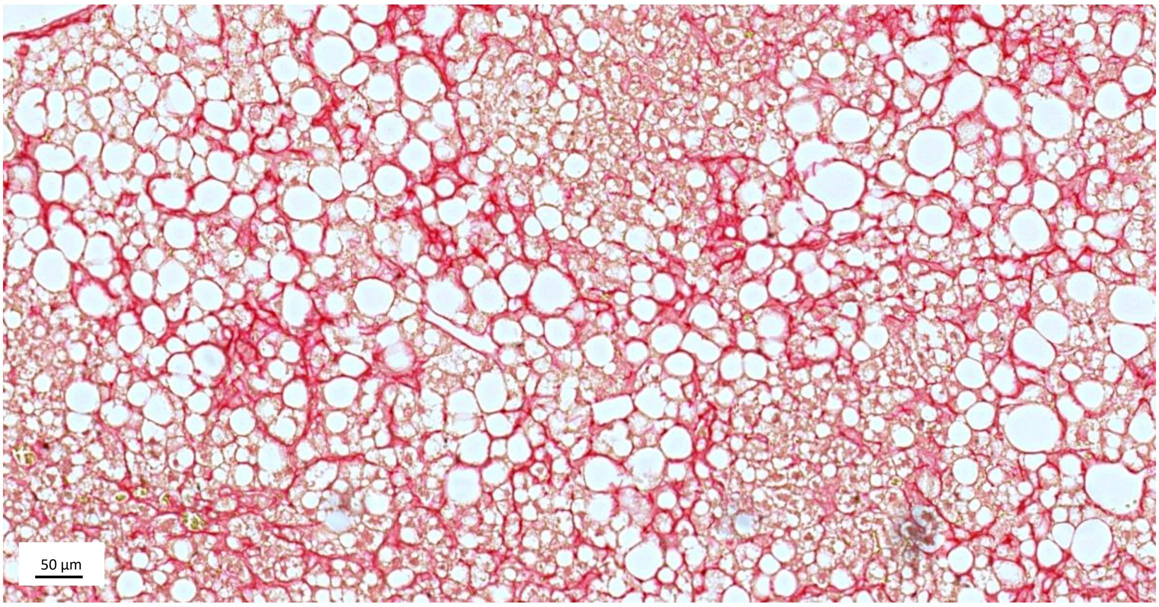

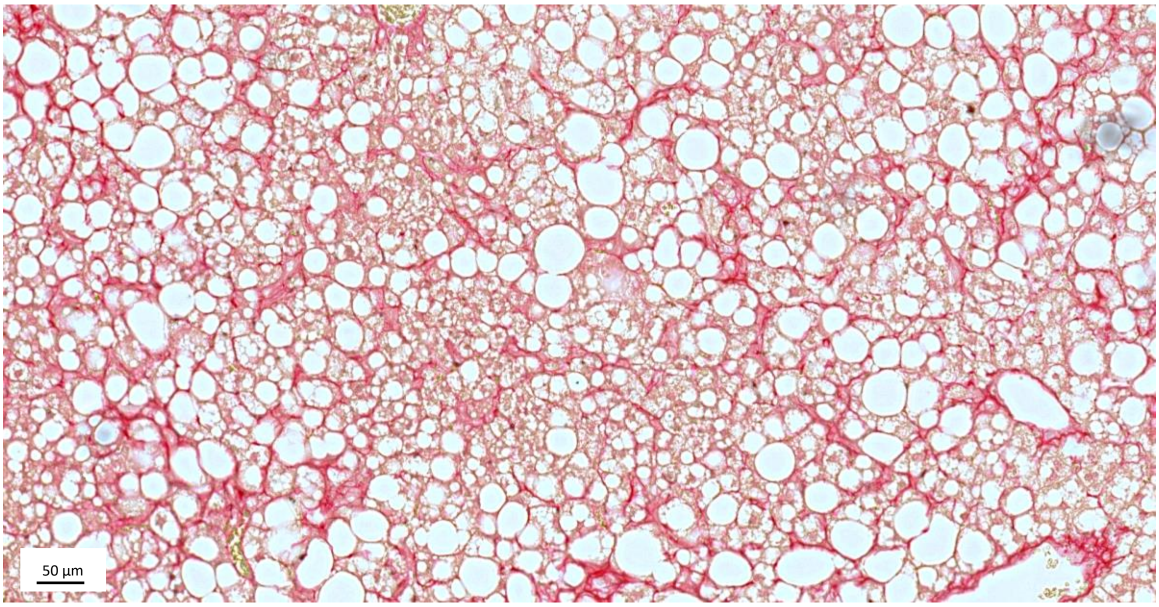

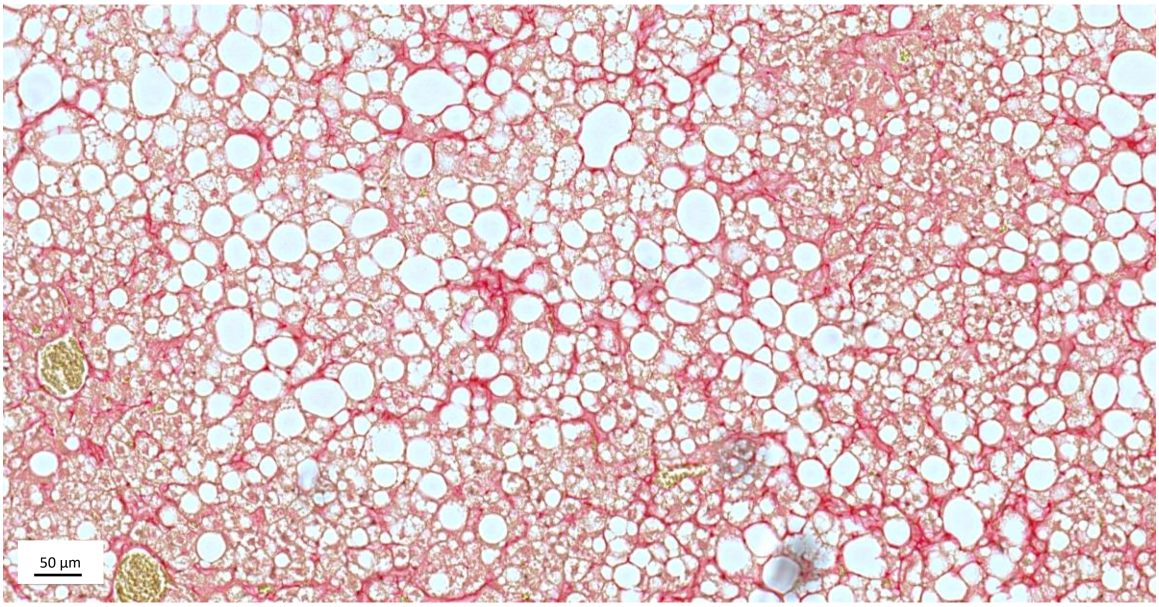

HFHCD-11

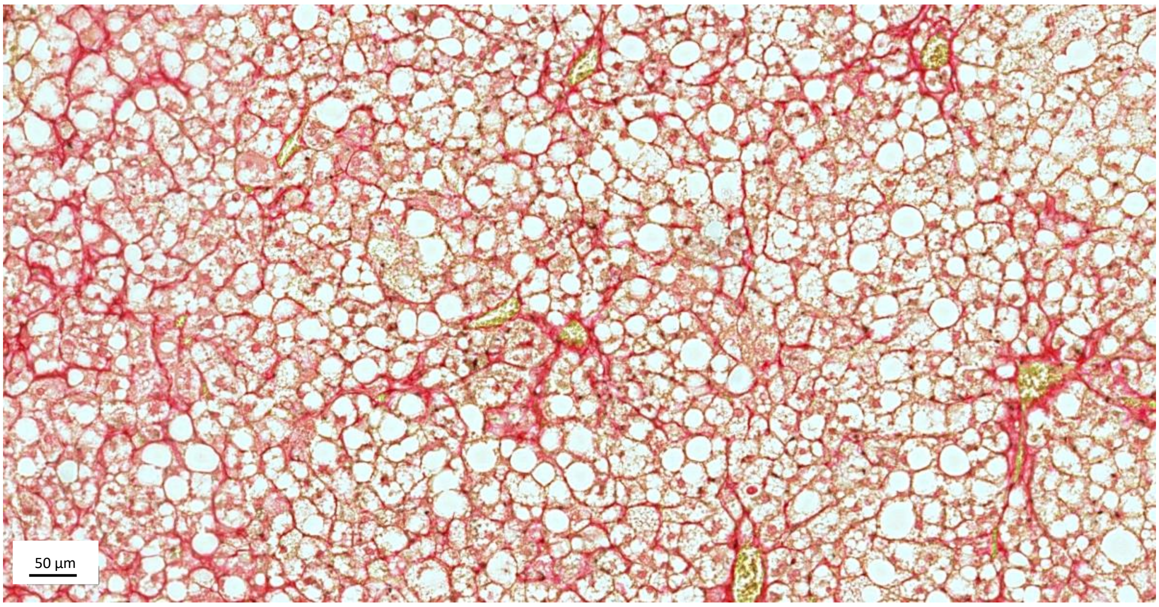

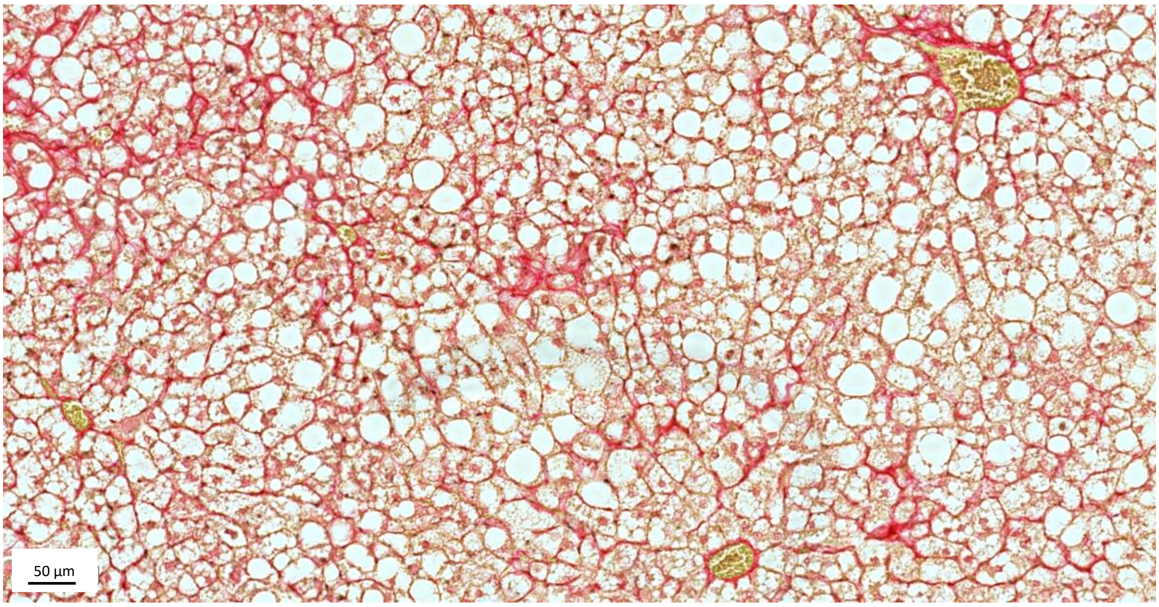

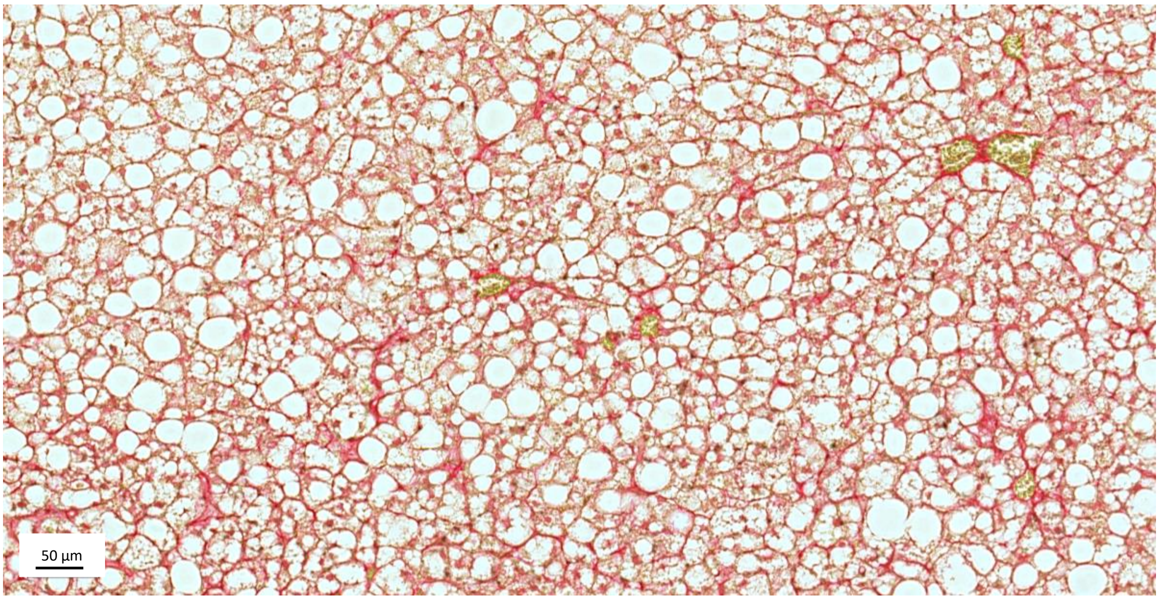

HFHCD-12

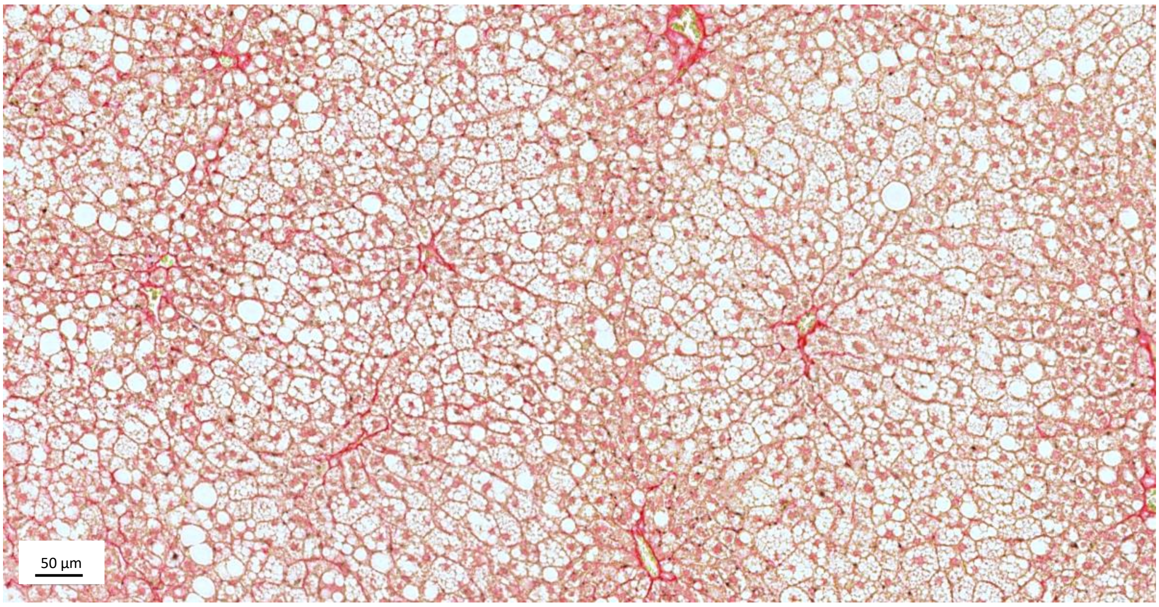

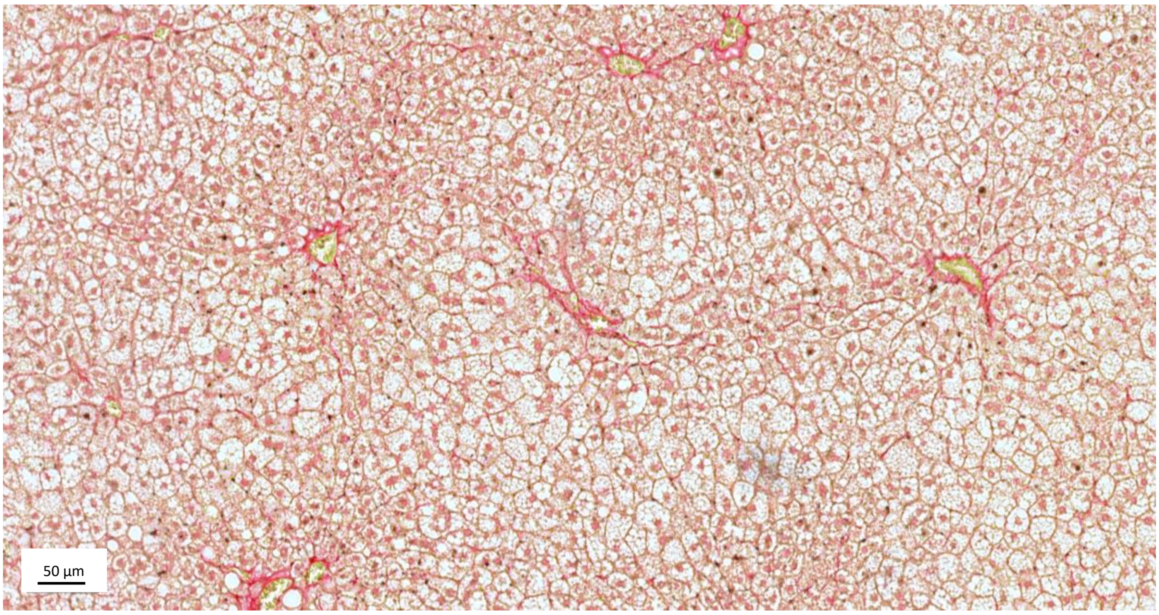

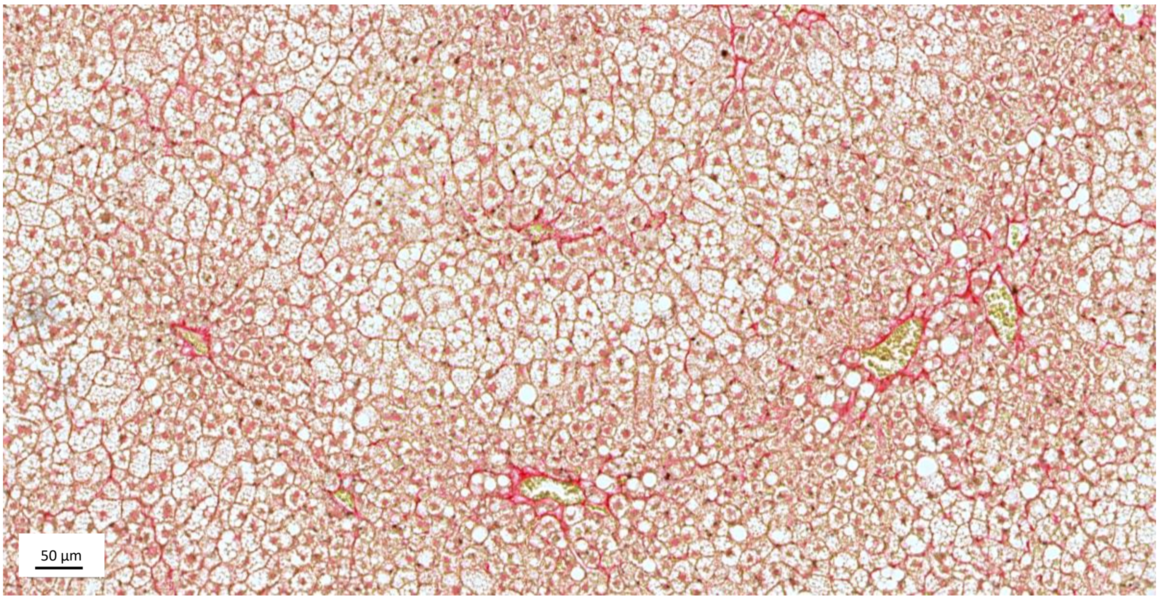

HFHCD-13

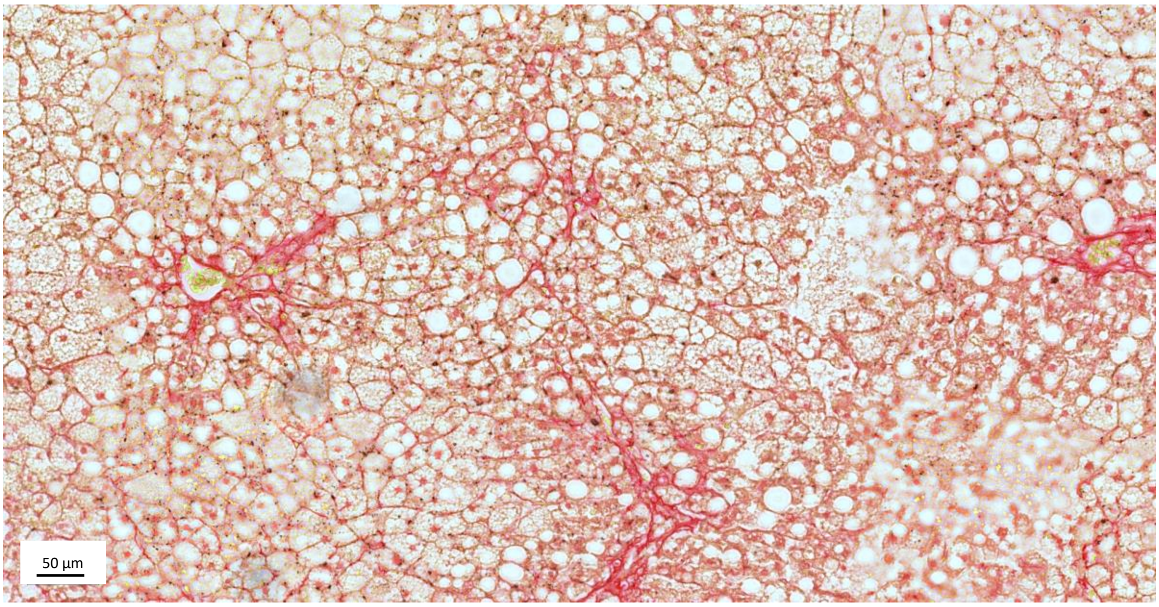

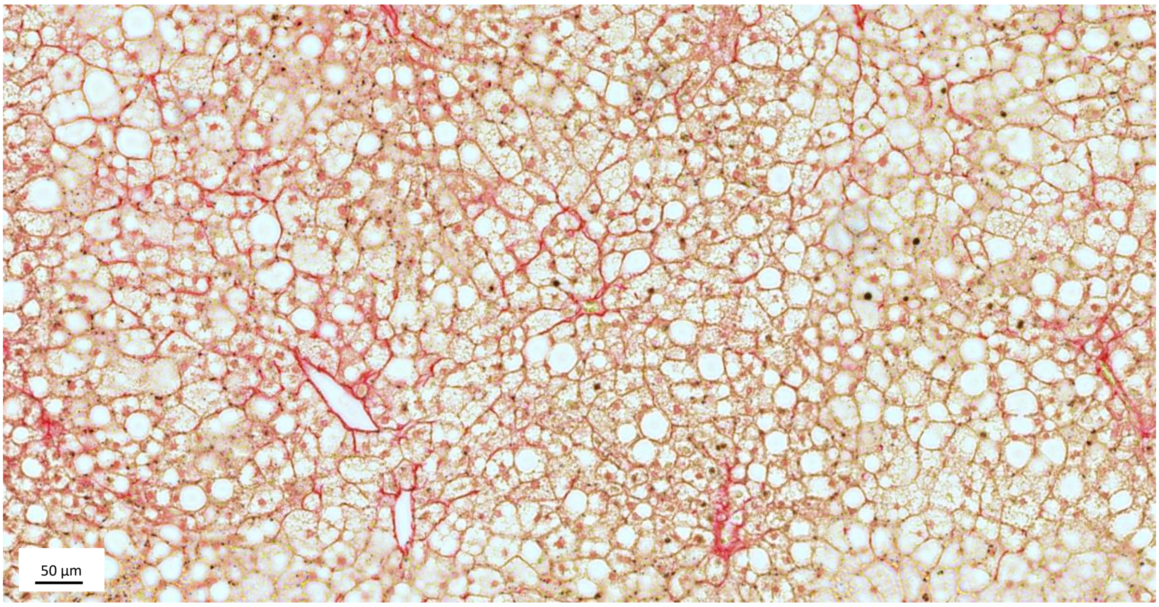

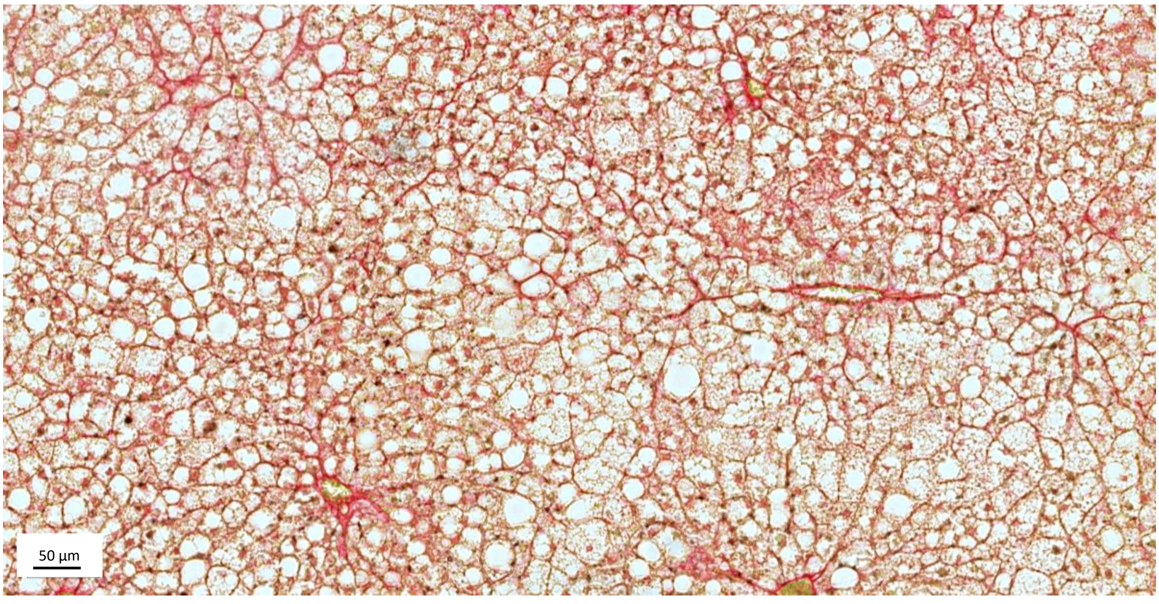

HFHCD-14

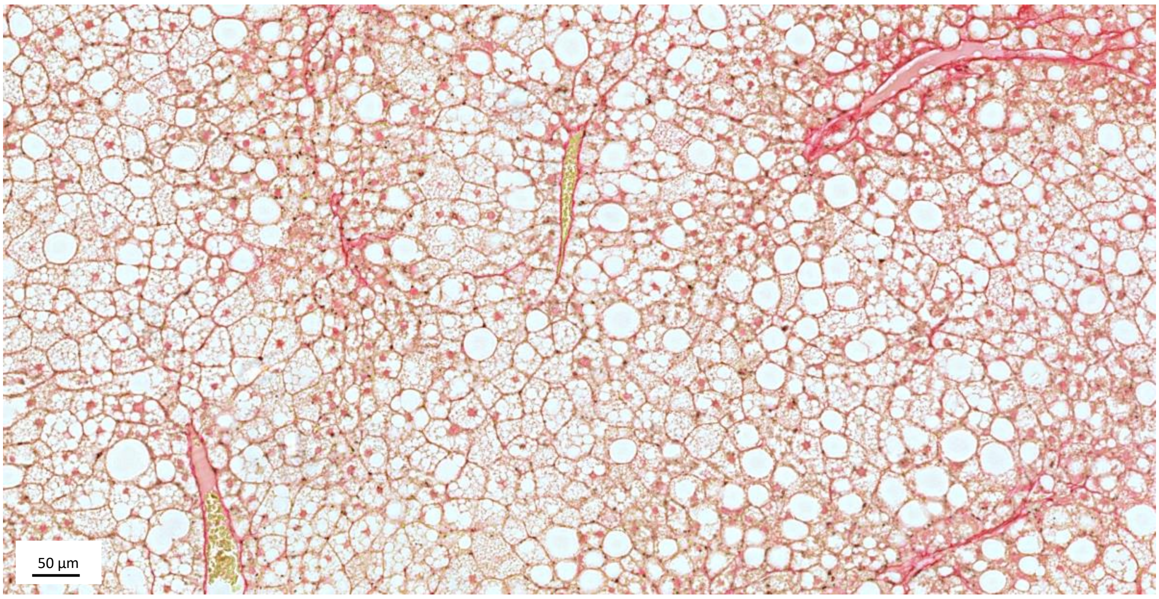

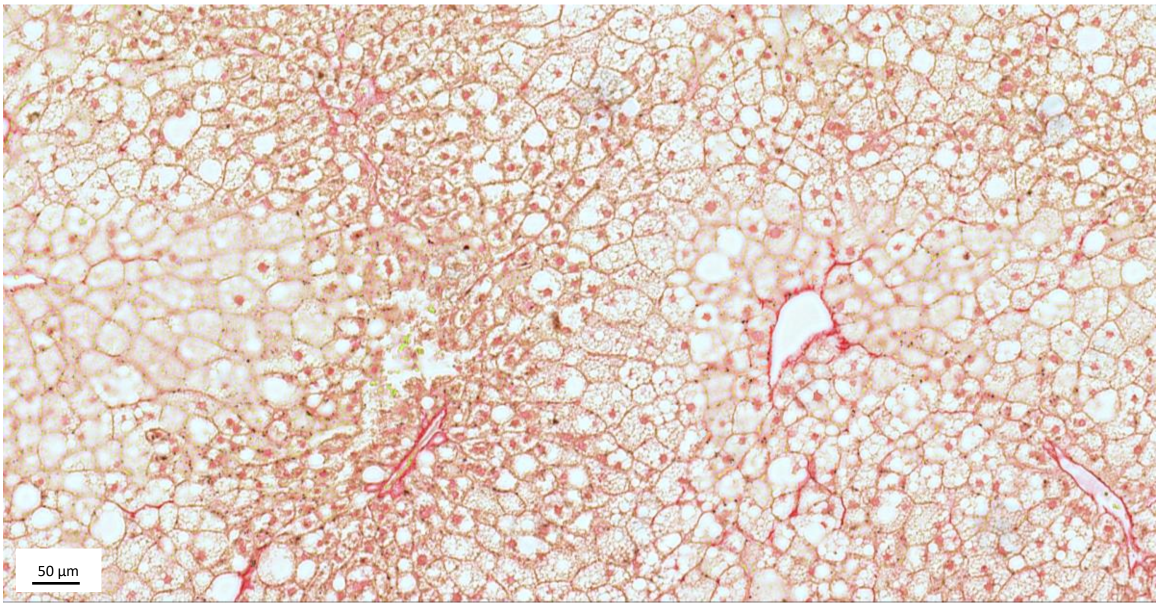

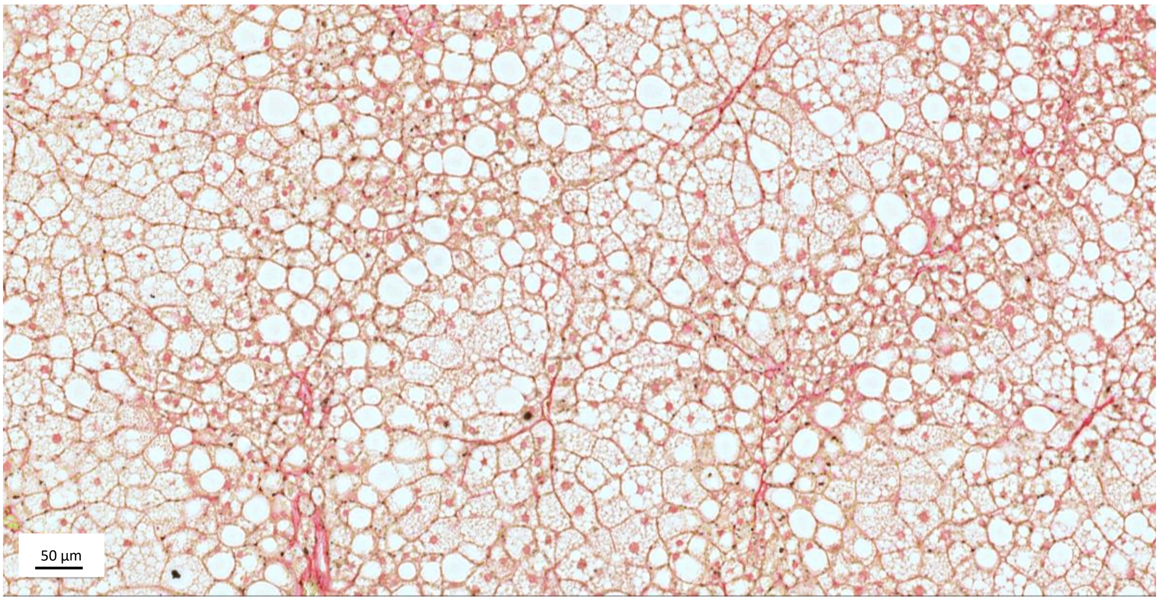

HFHCD-15

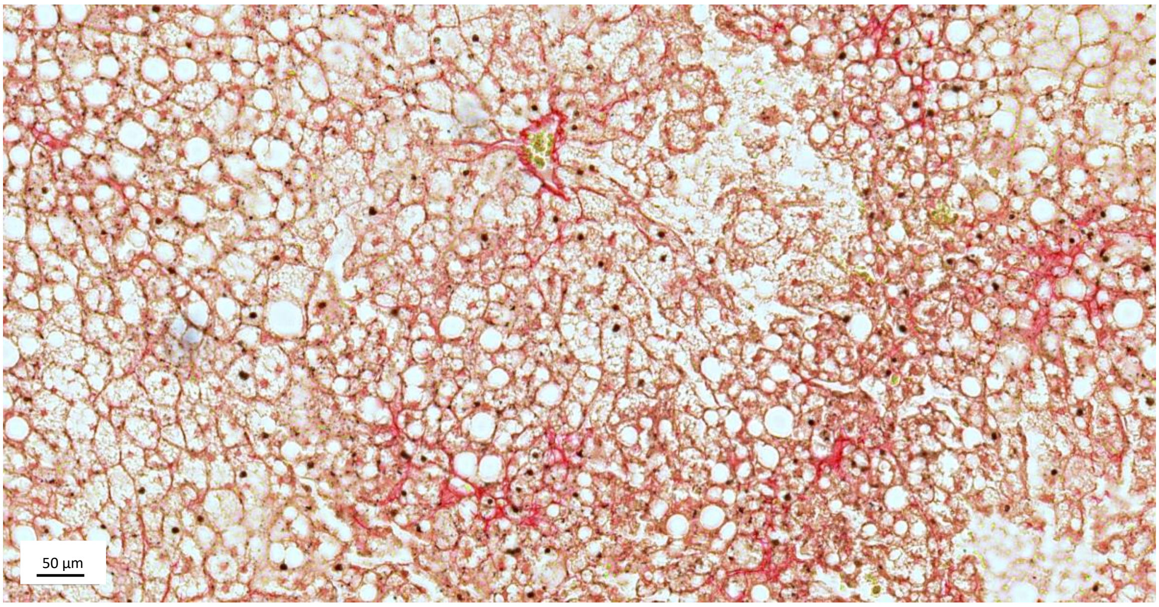

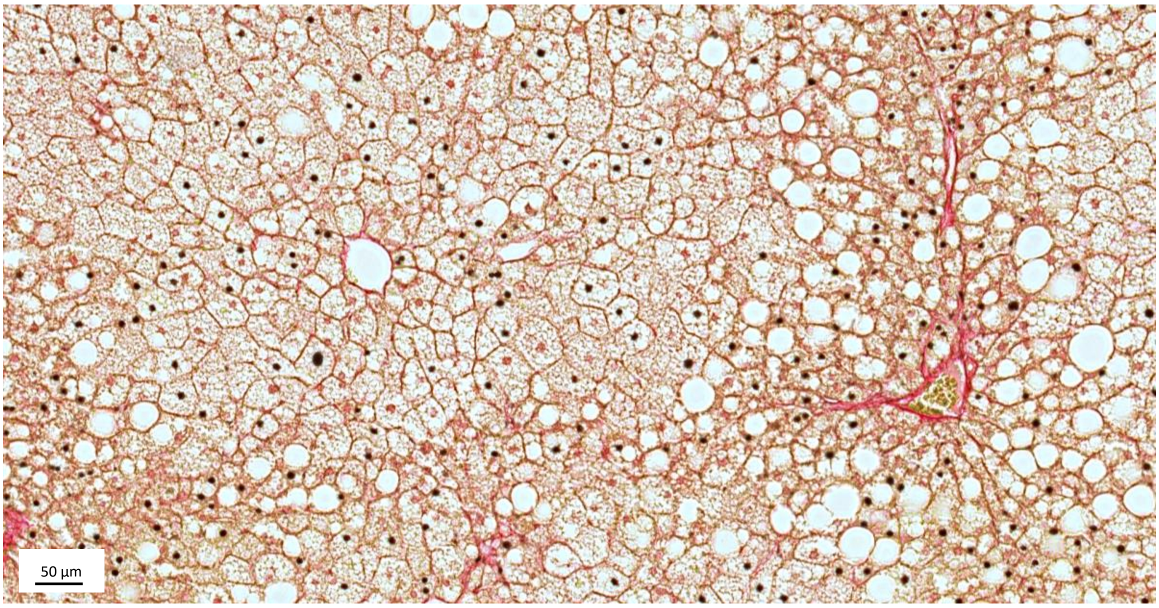

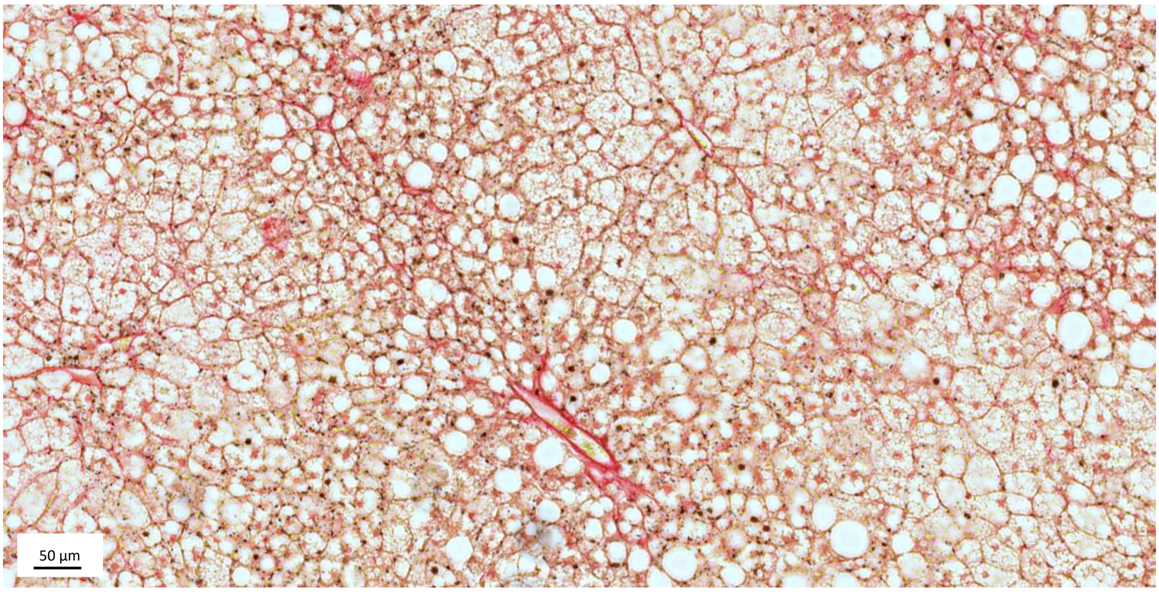

Sirius red Staining

HFHCD-I group

(12 mice were included)

HFHCD-I-1

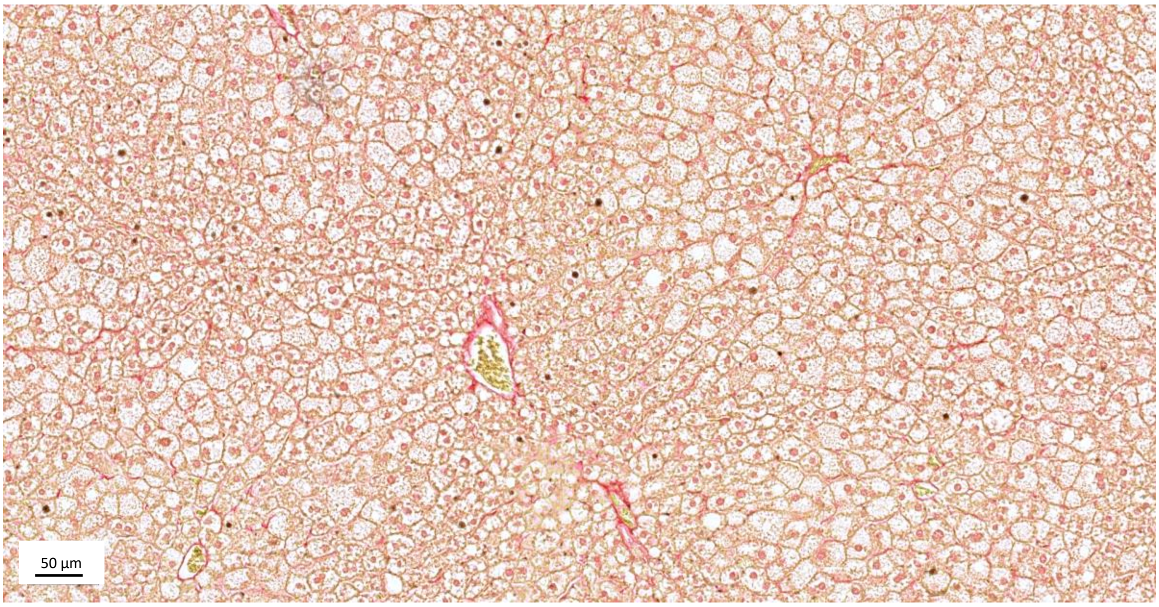

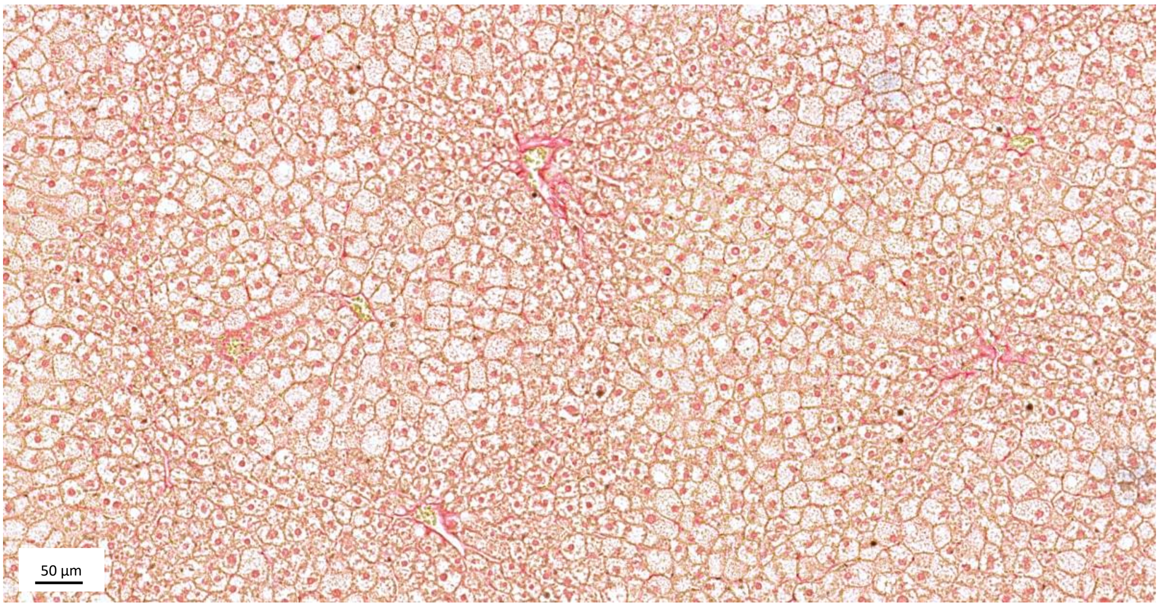

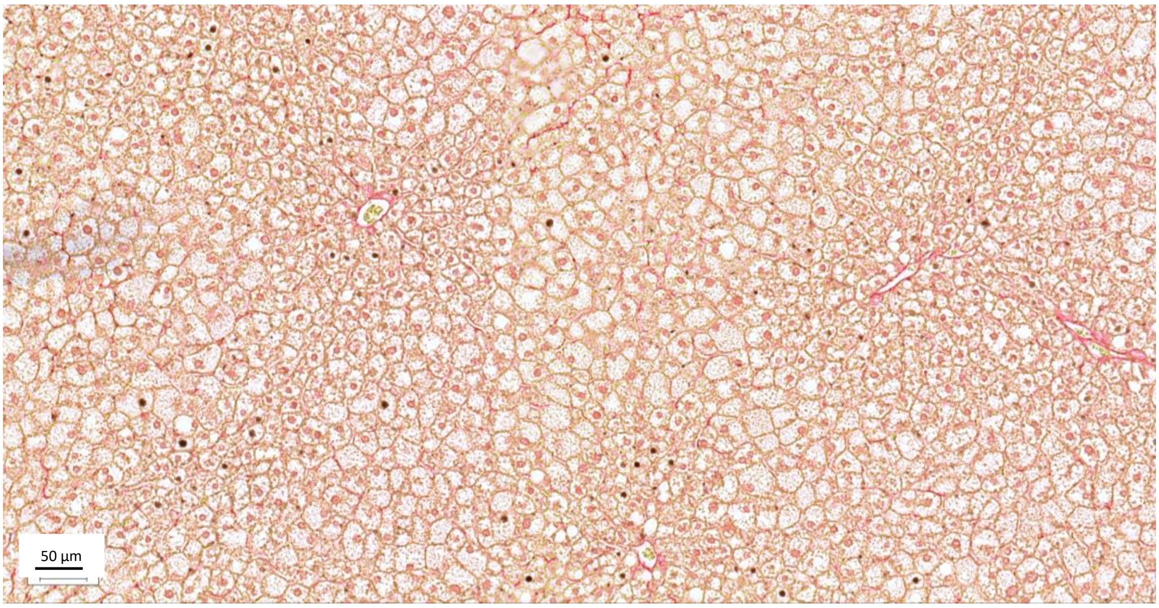

HFHCD-I-2

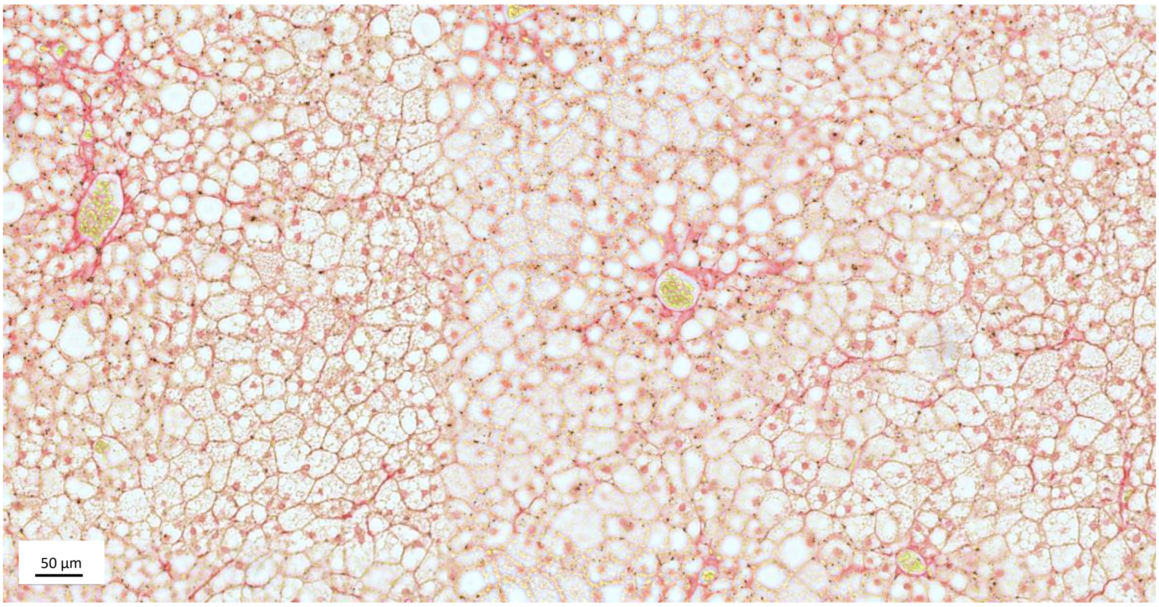

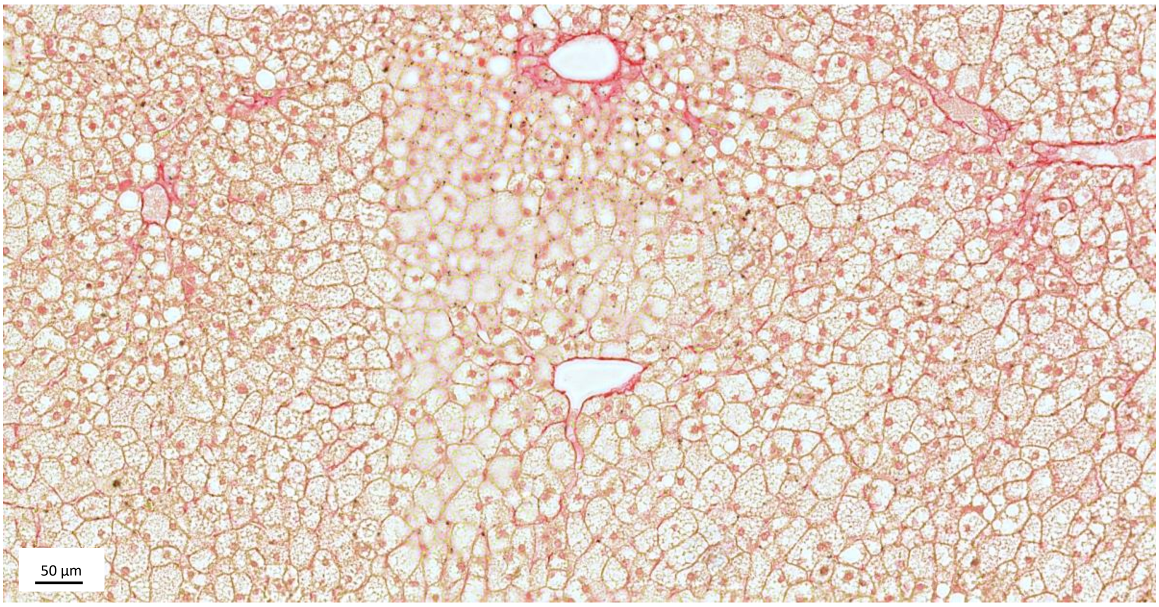

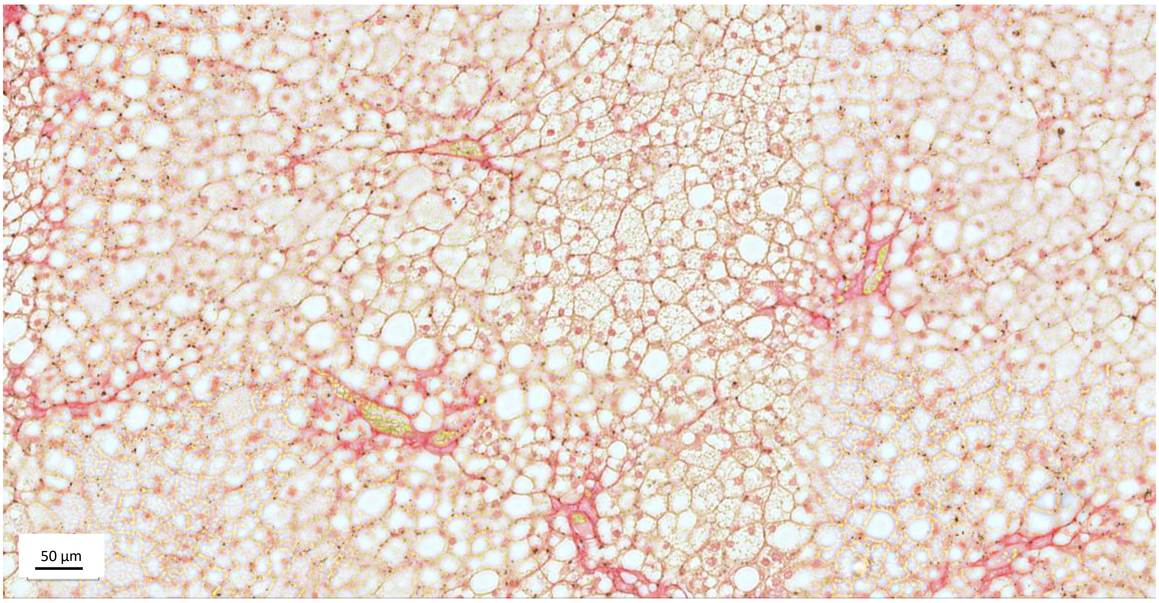

HFHCD-I-3

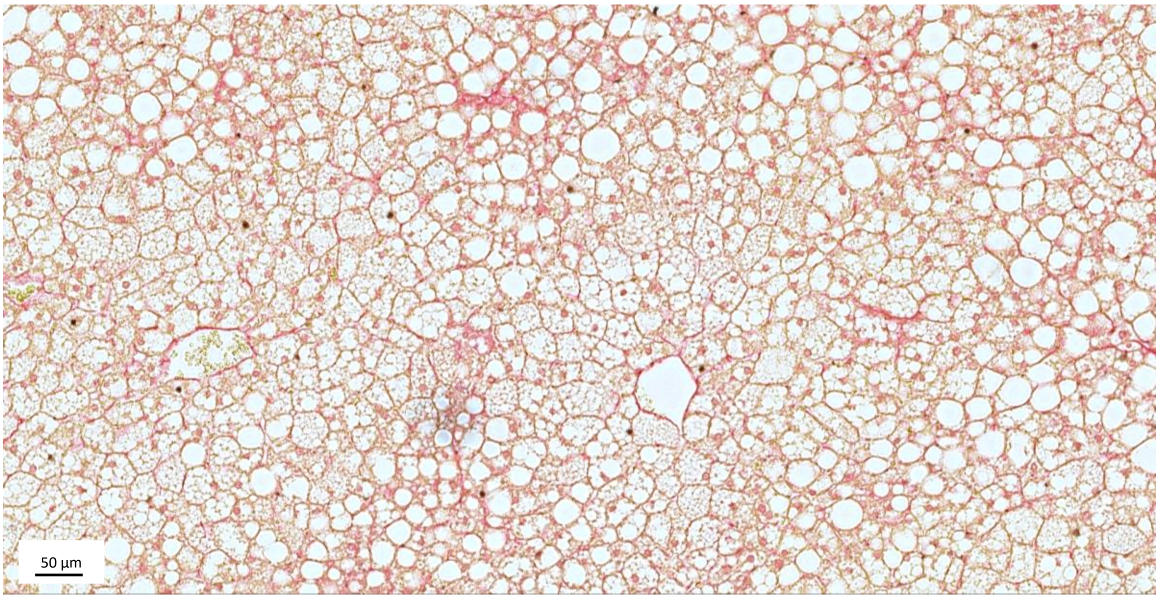

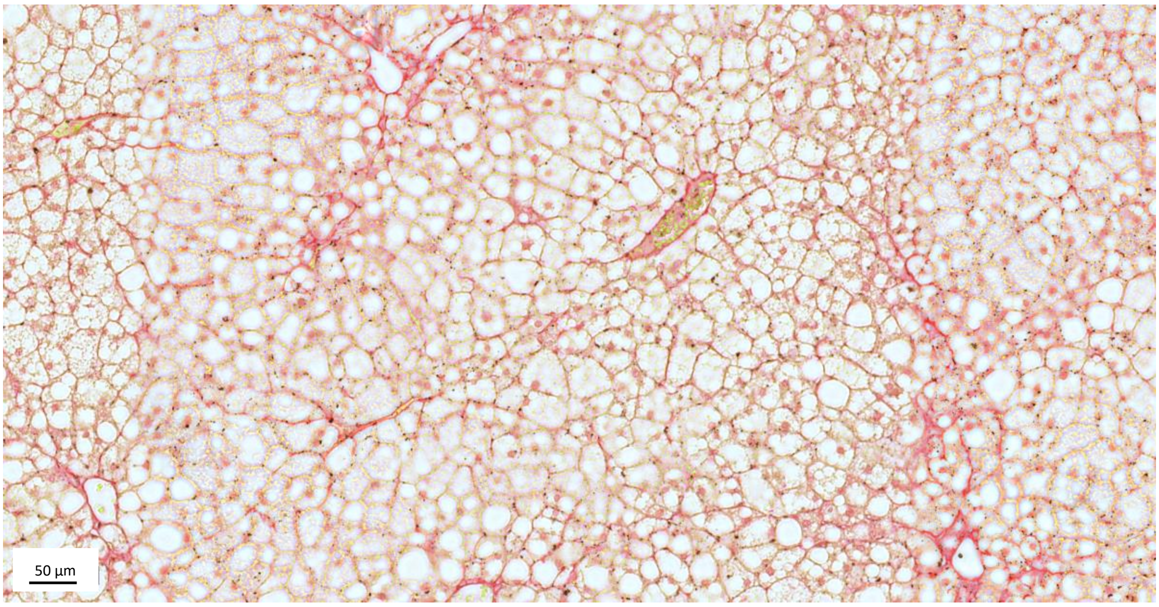

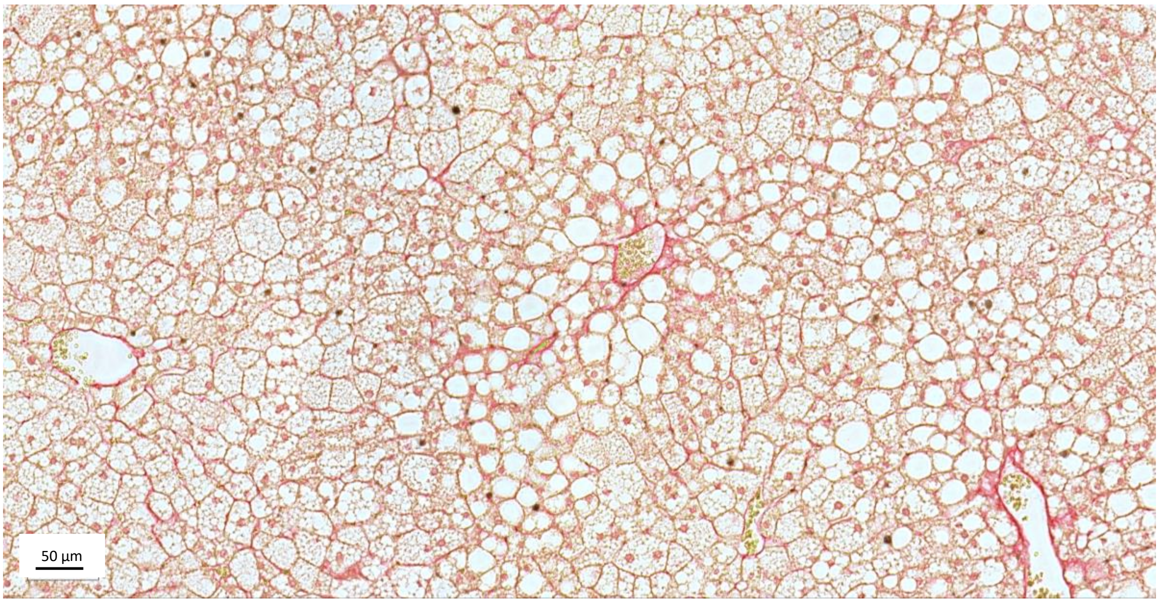

HFHCD-I-4

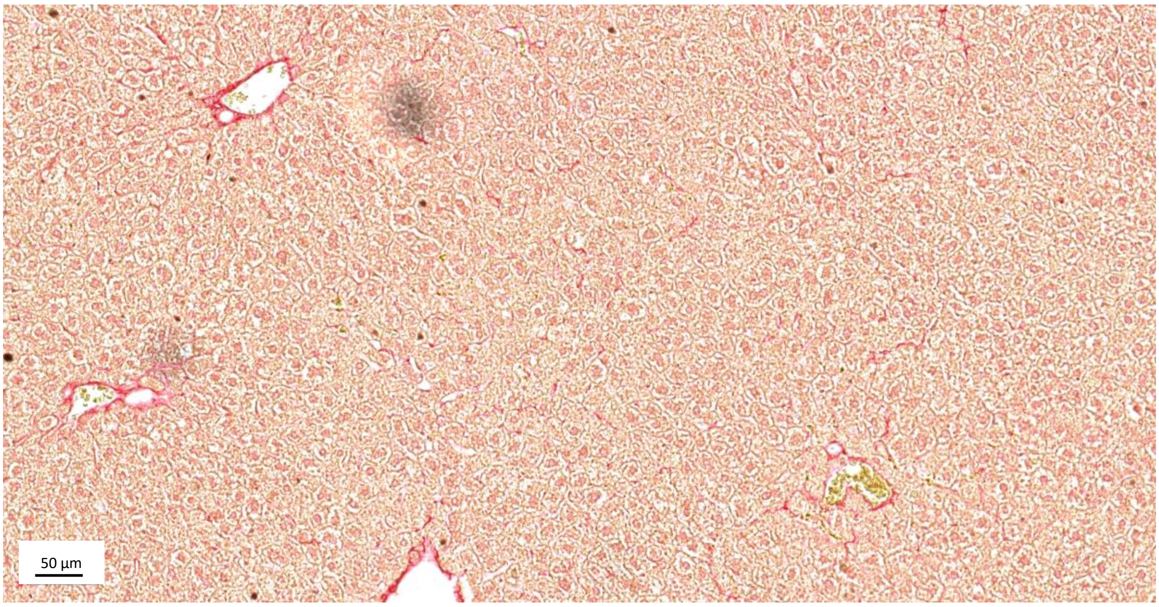

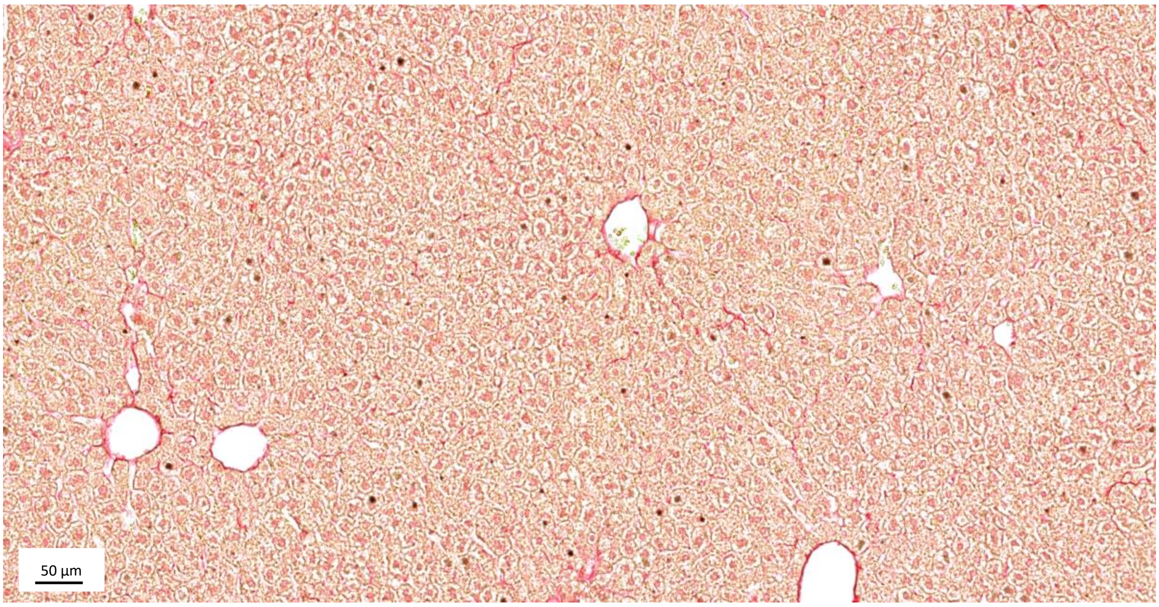

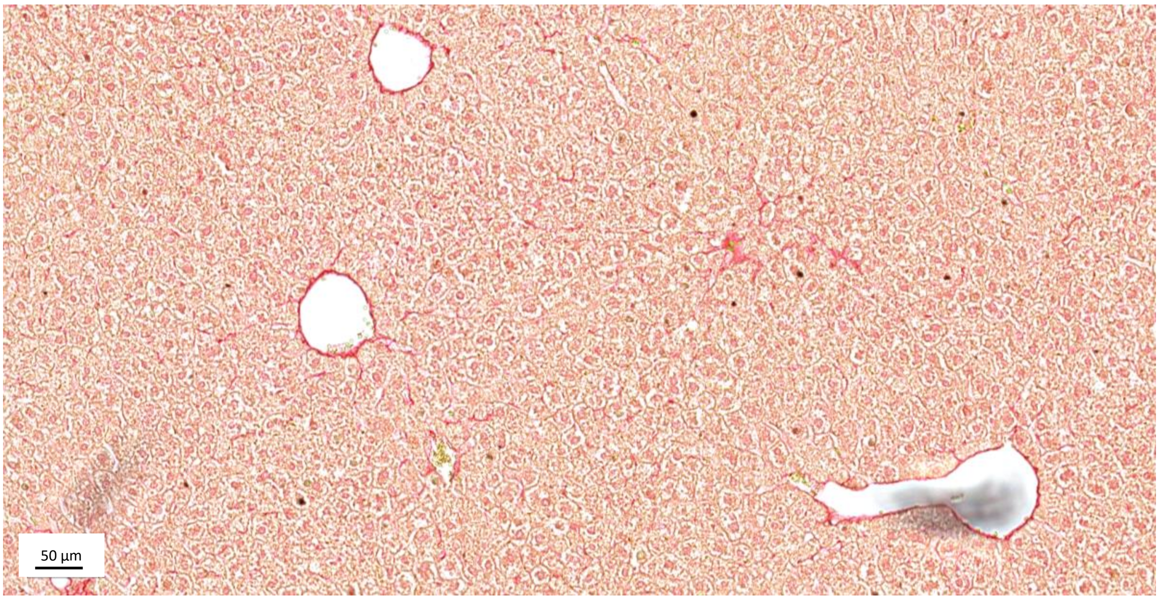

HFHCD-I-5

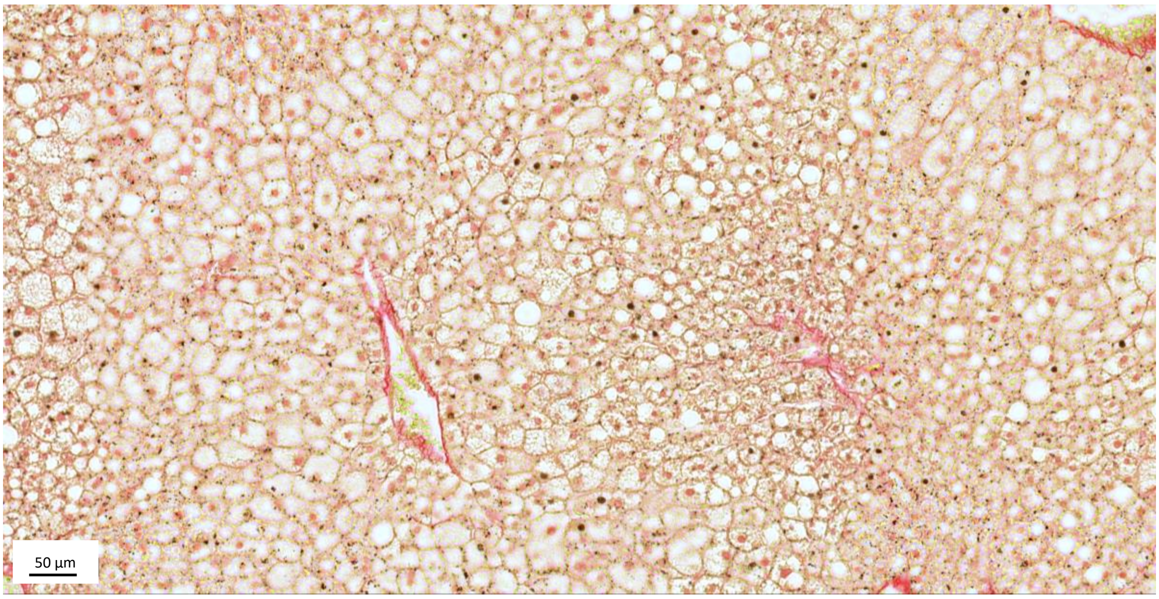

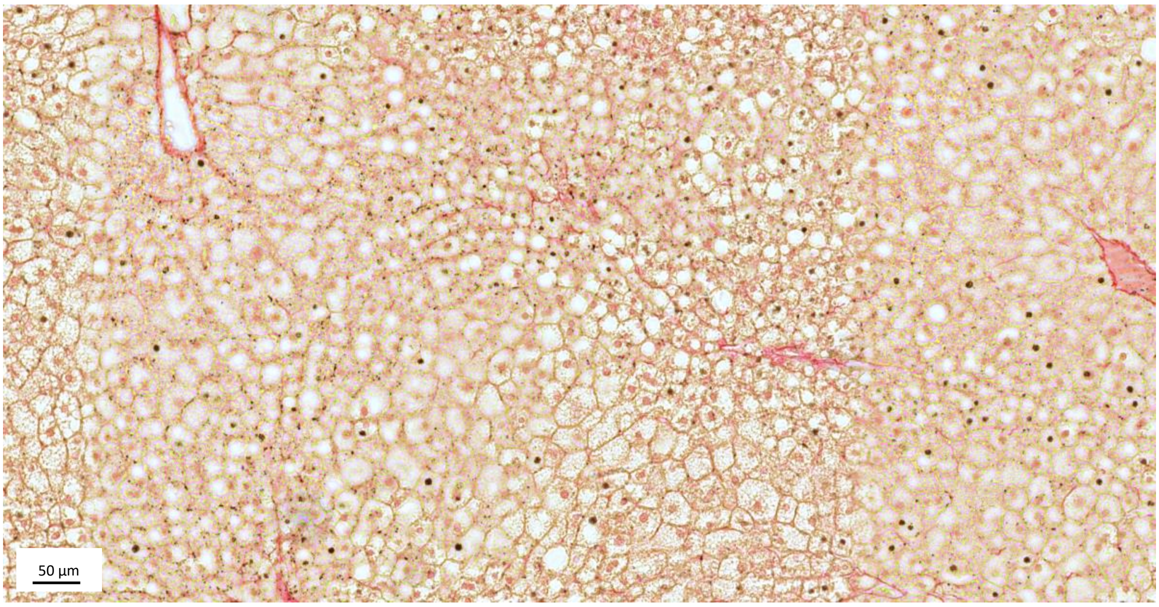

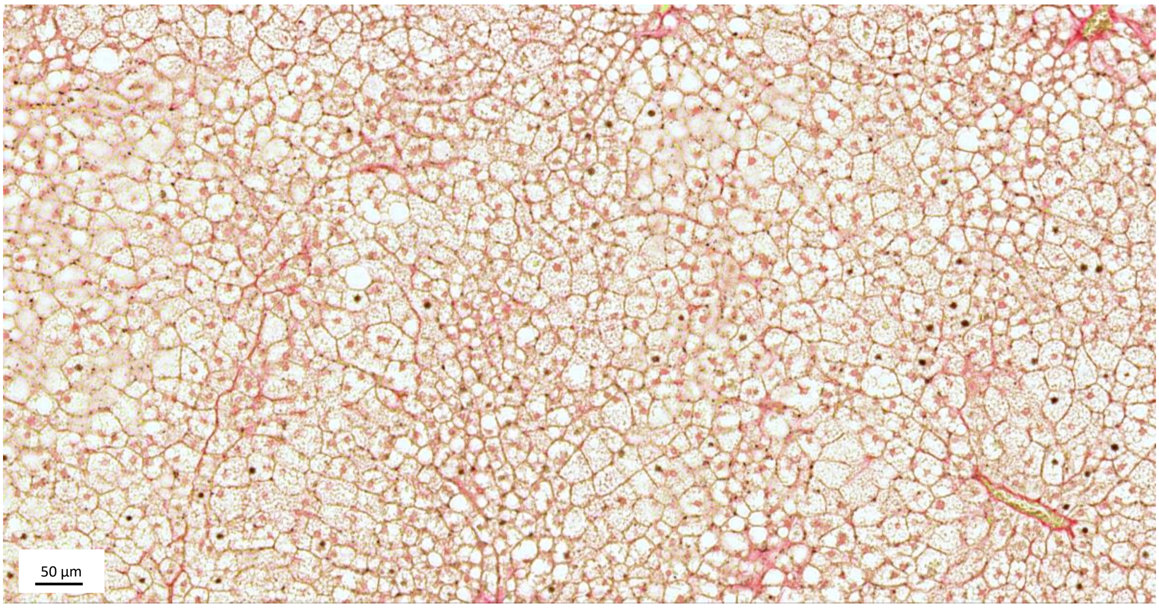

HFHCD-I-6

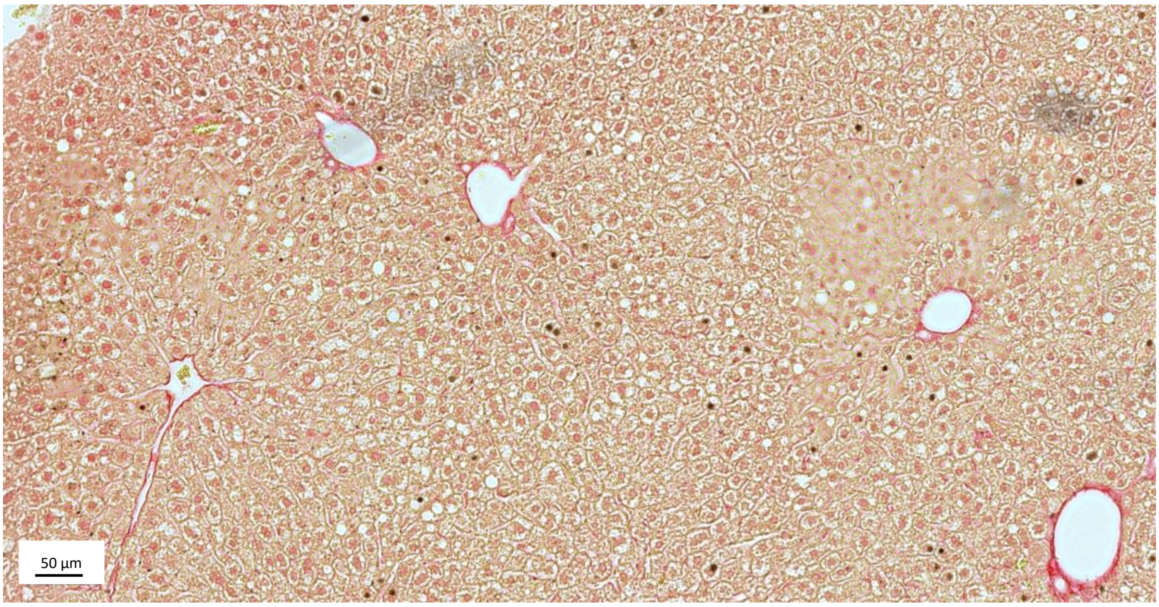

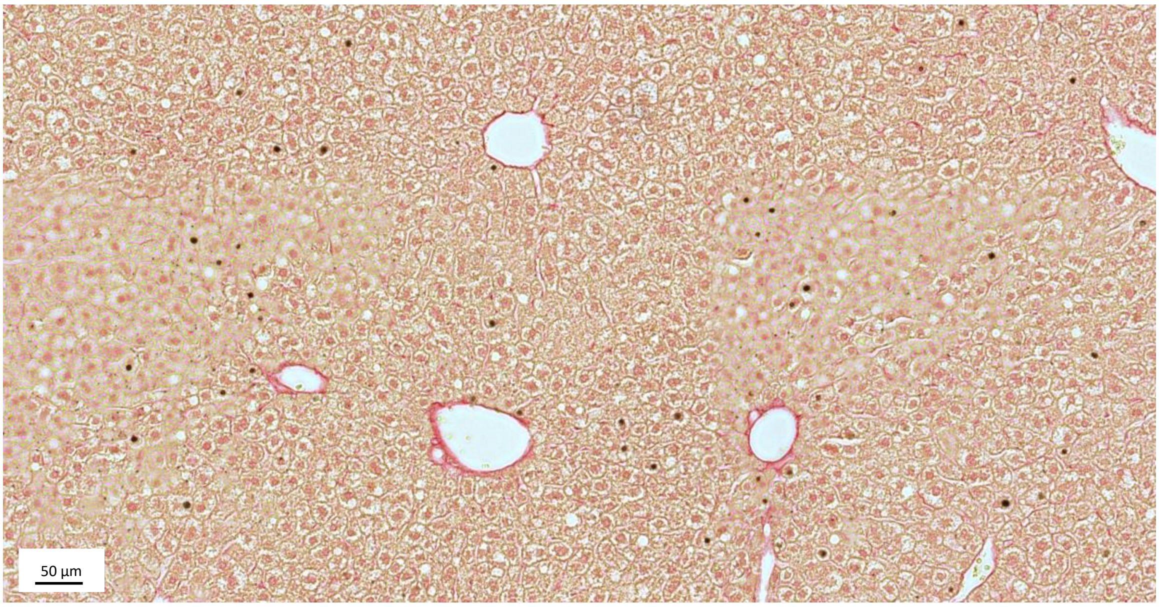

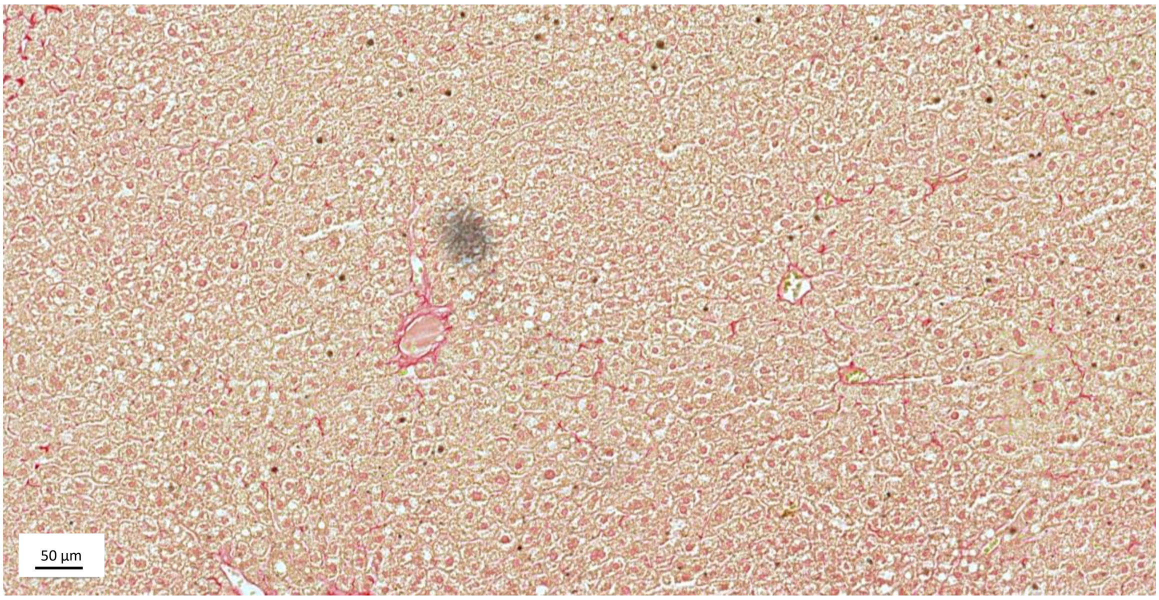

HFHCD-I-7

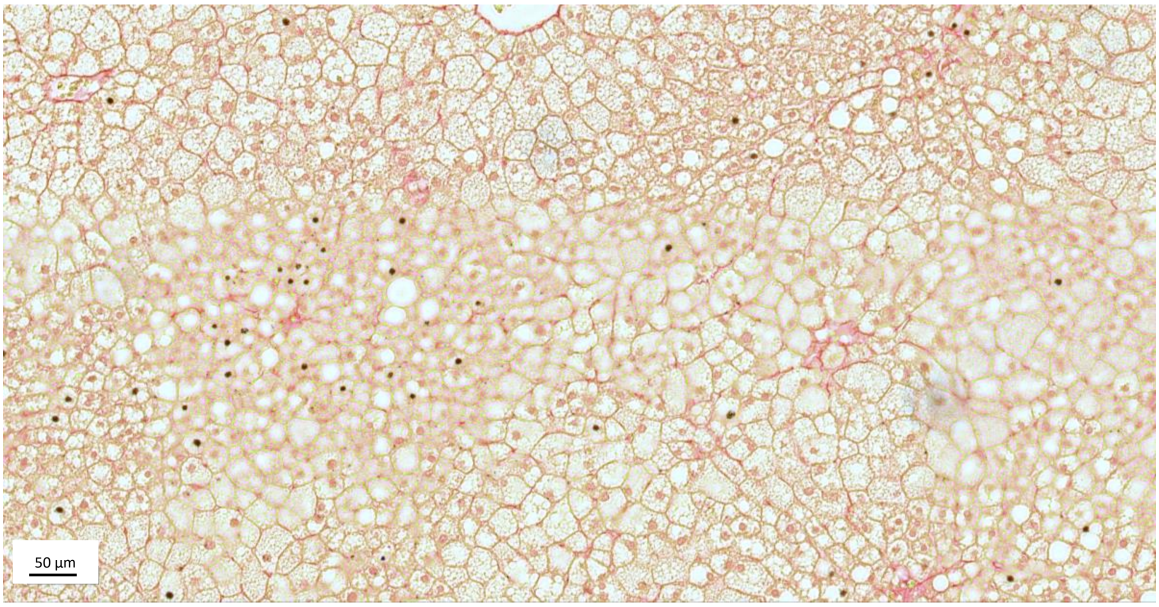

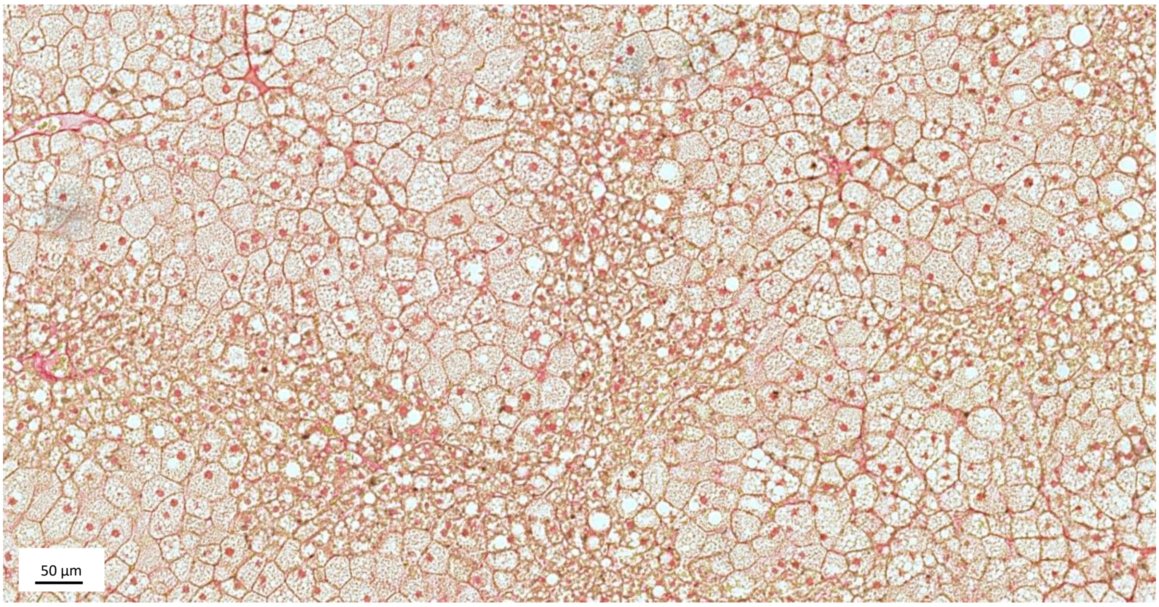

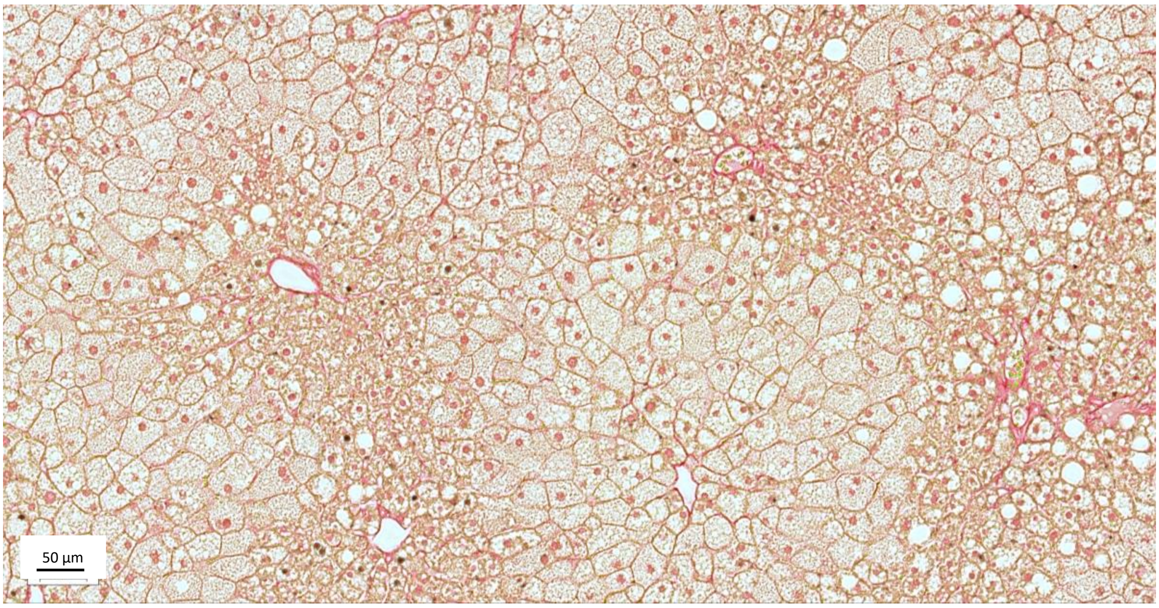

HFHCD-I-8

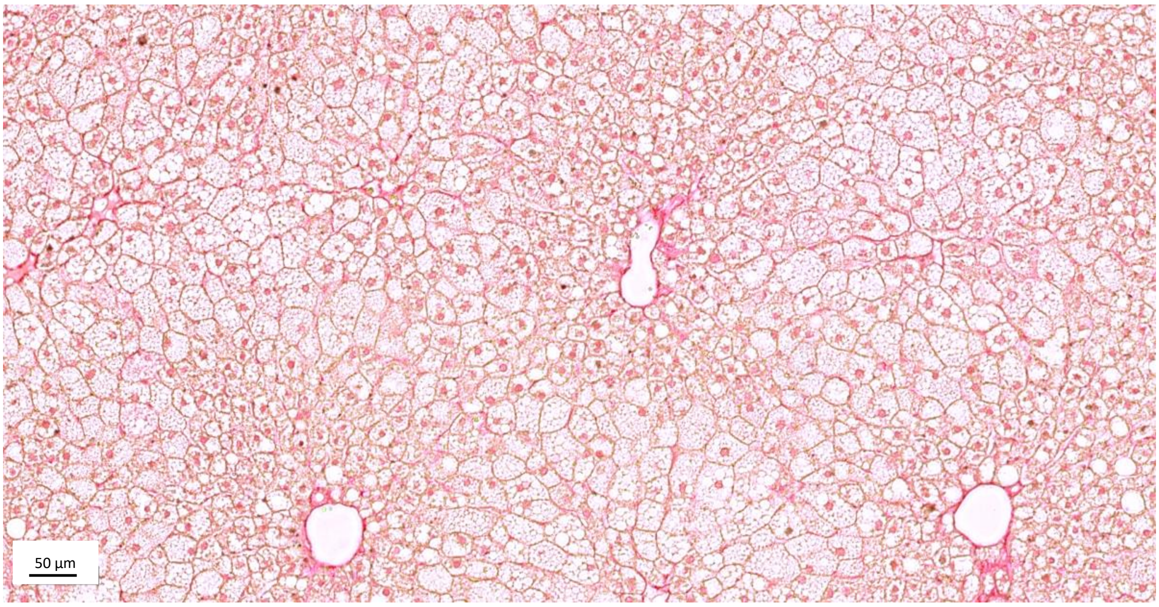

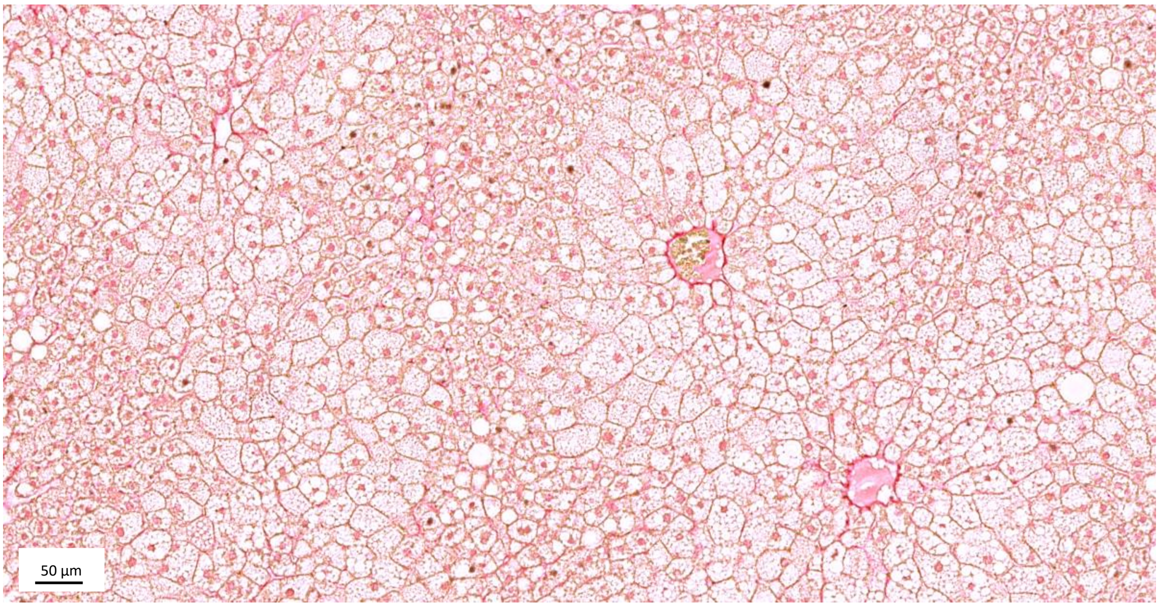

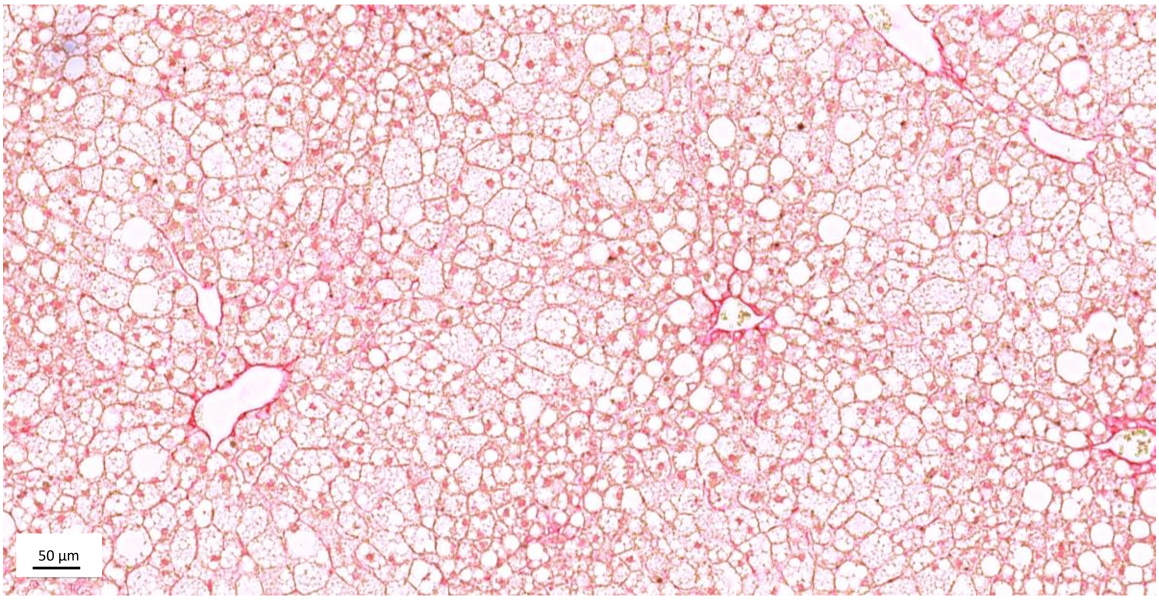

HFHCD-I-9

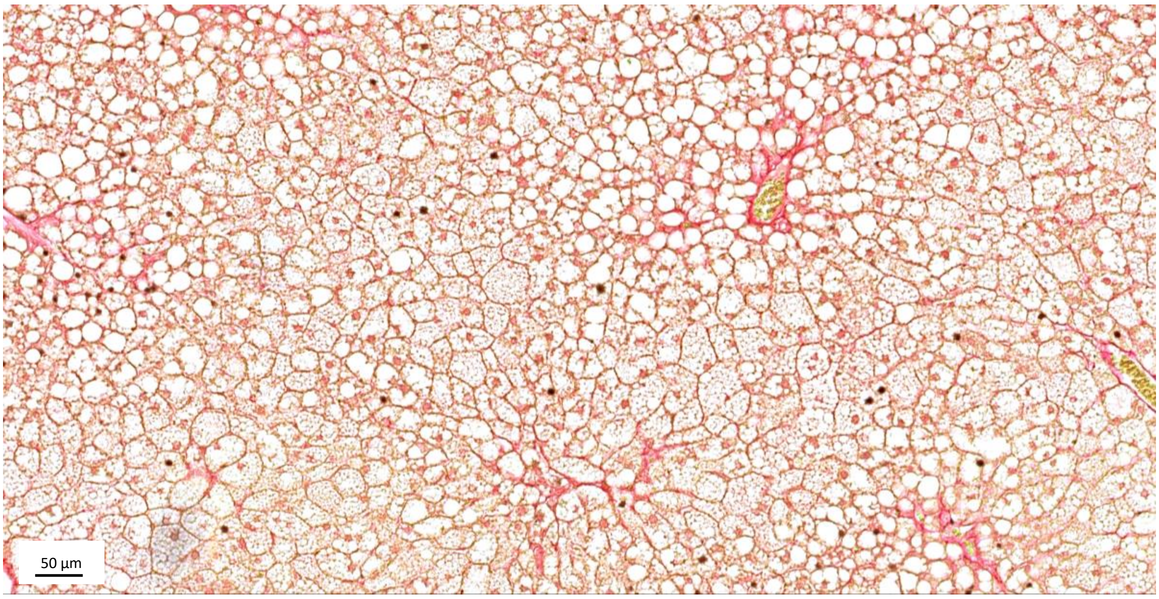

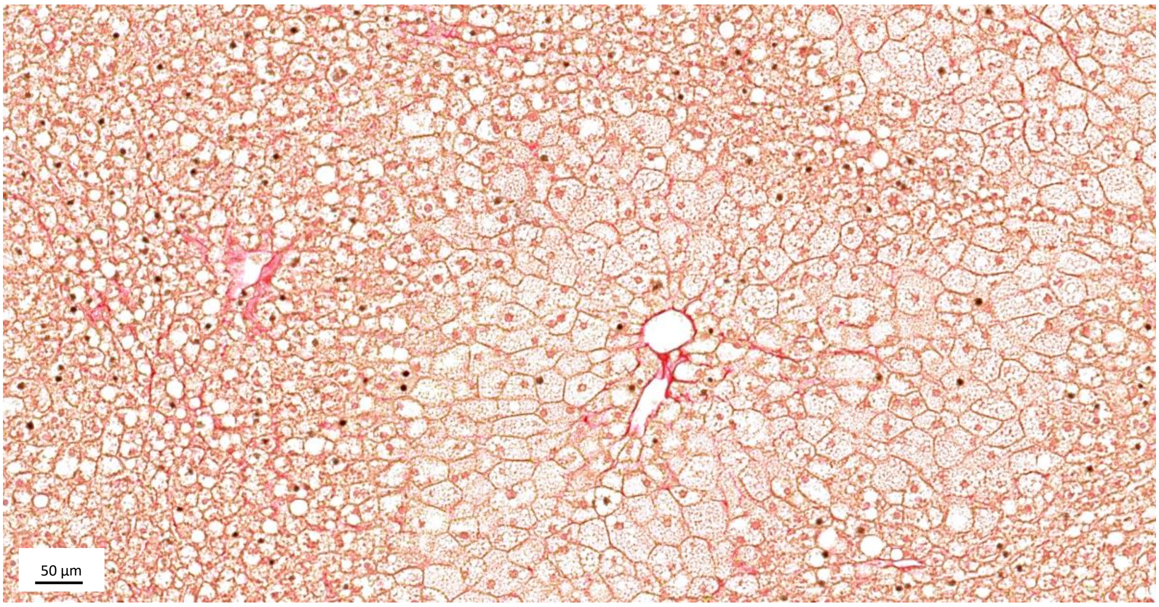

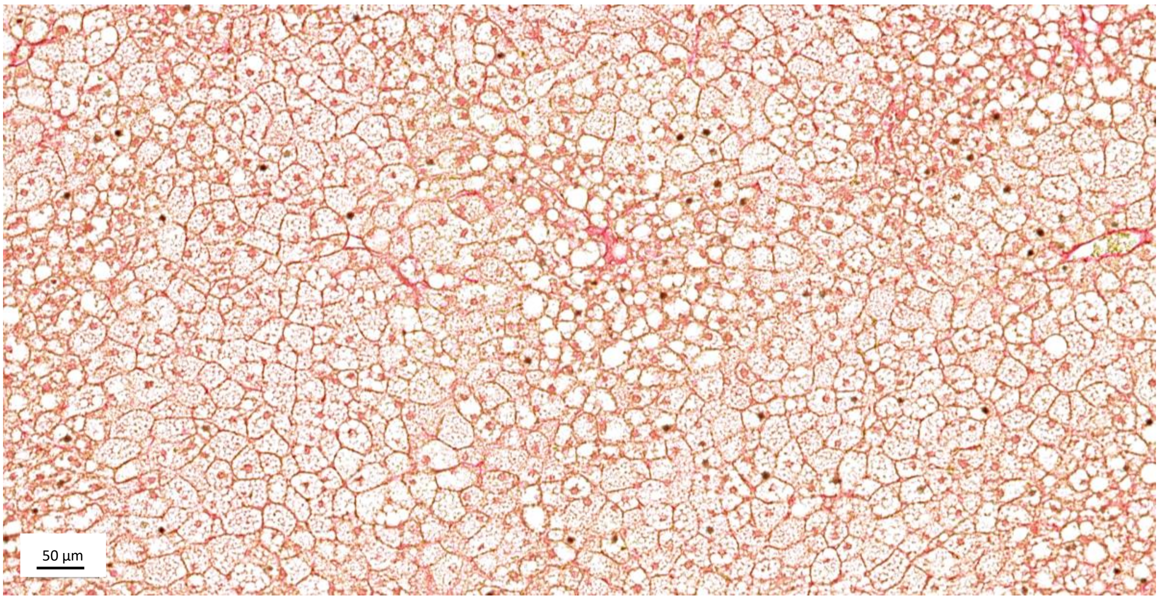

HFHCD-I-10

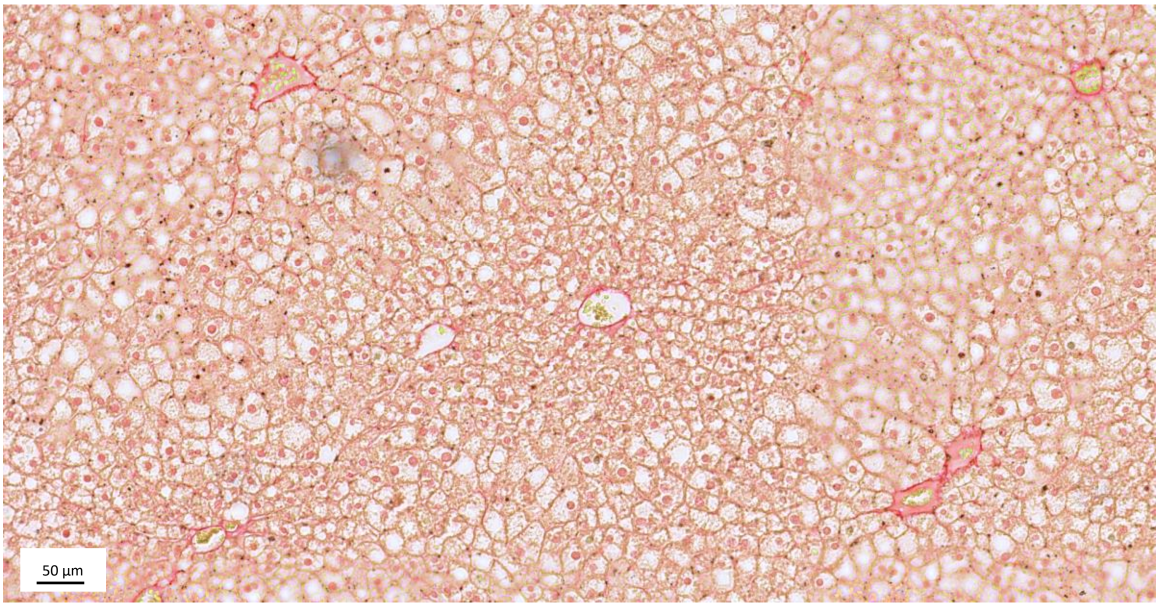

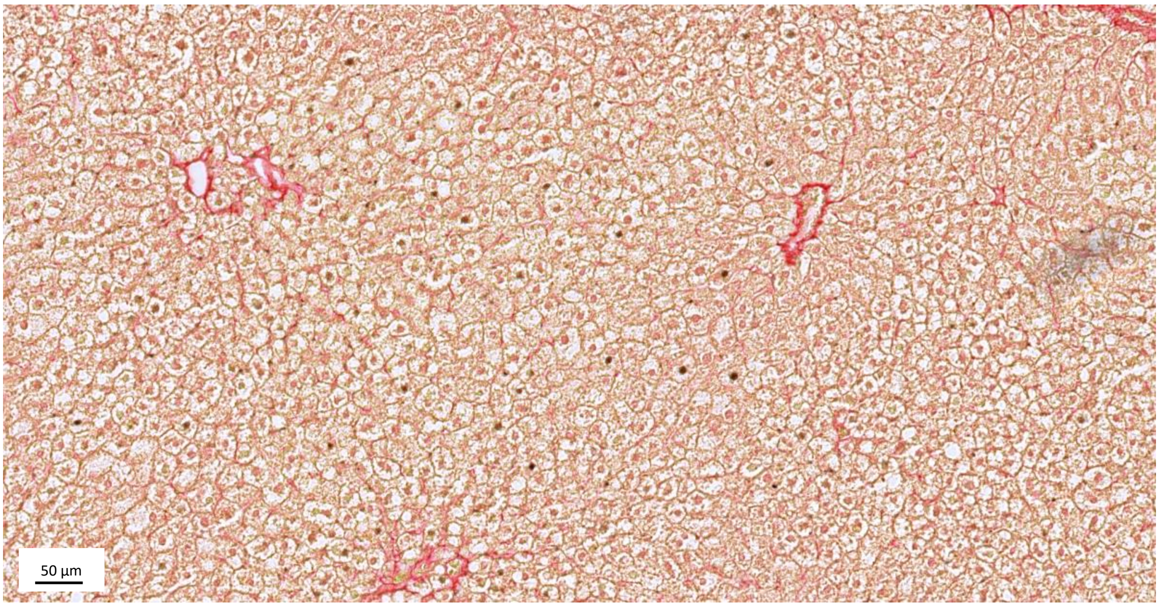

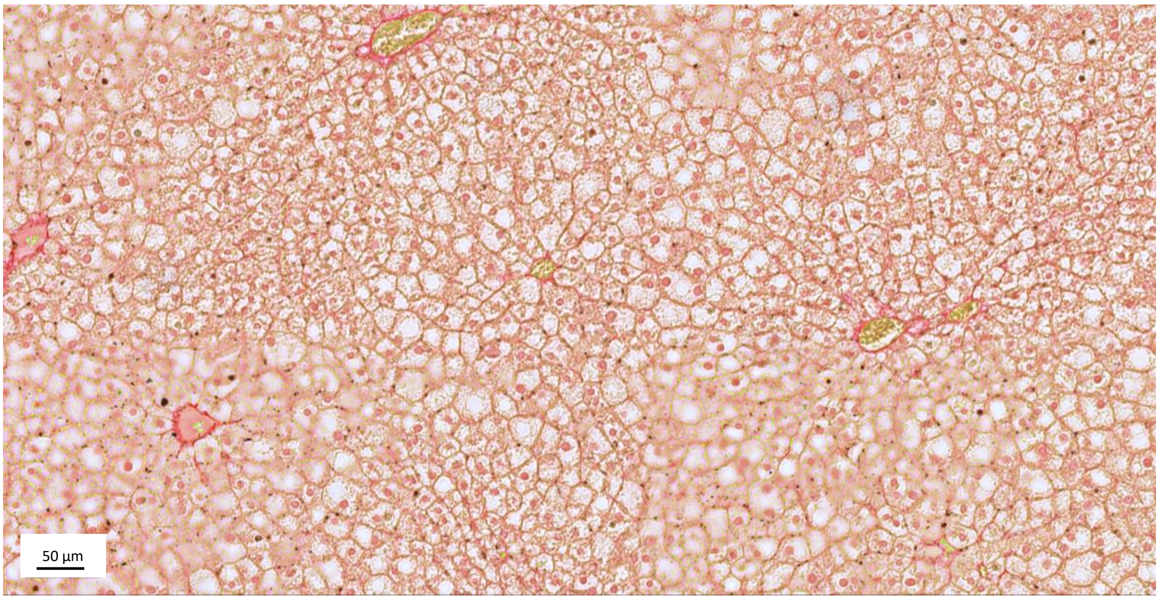

HFHCD-I-11

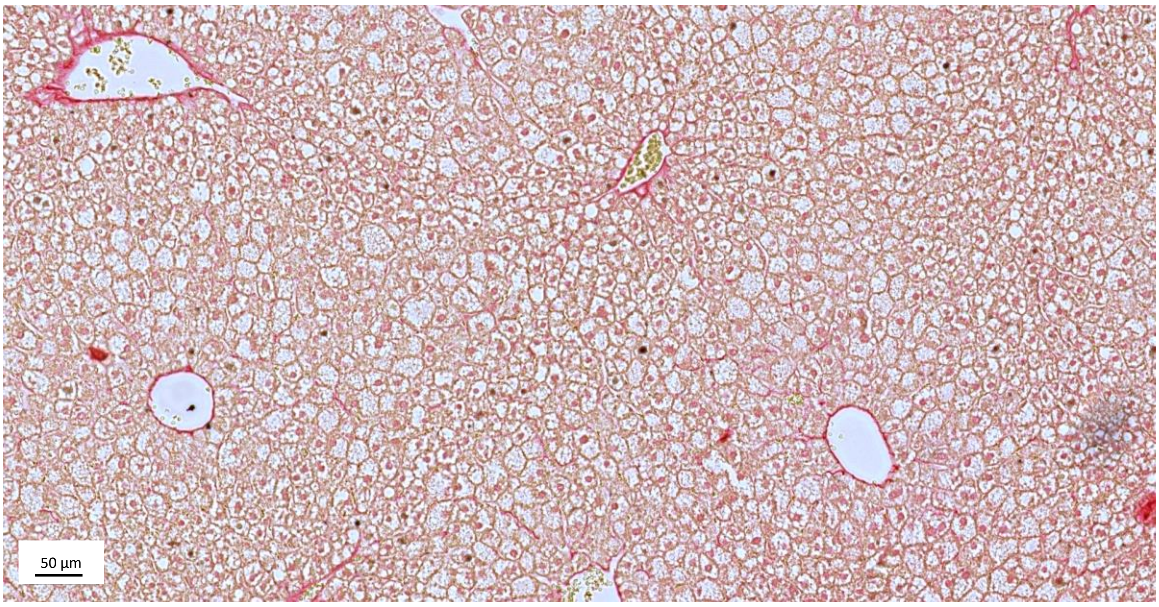

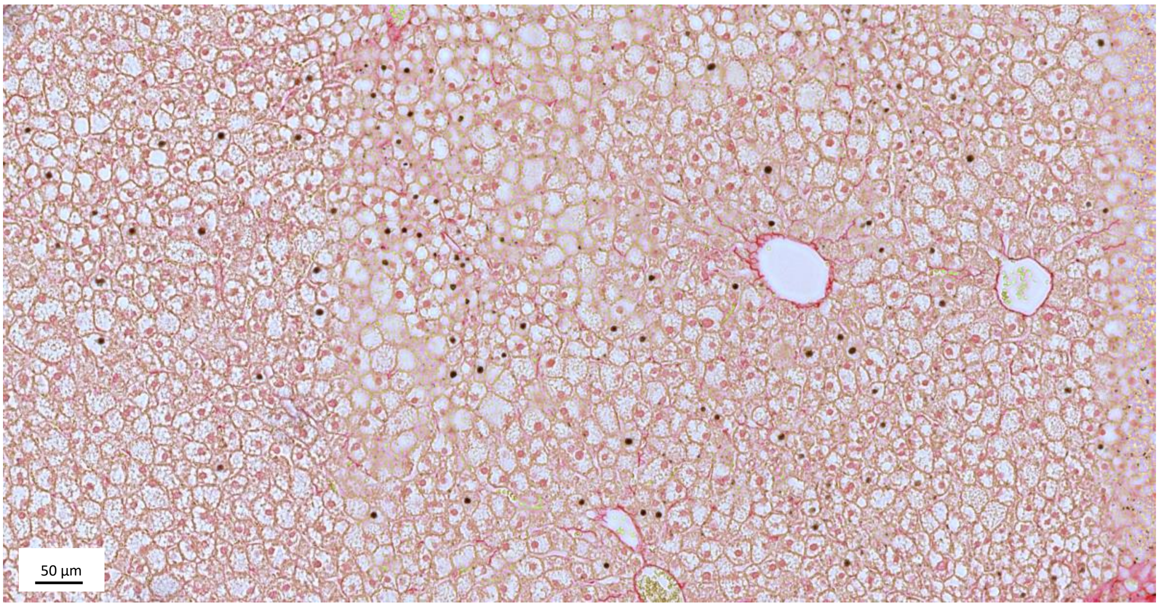

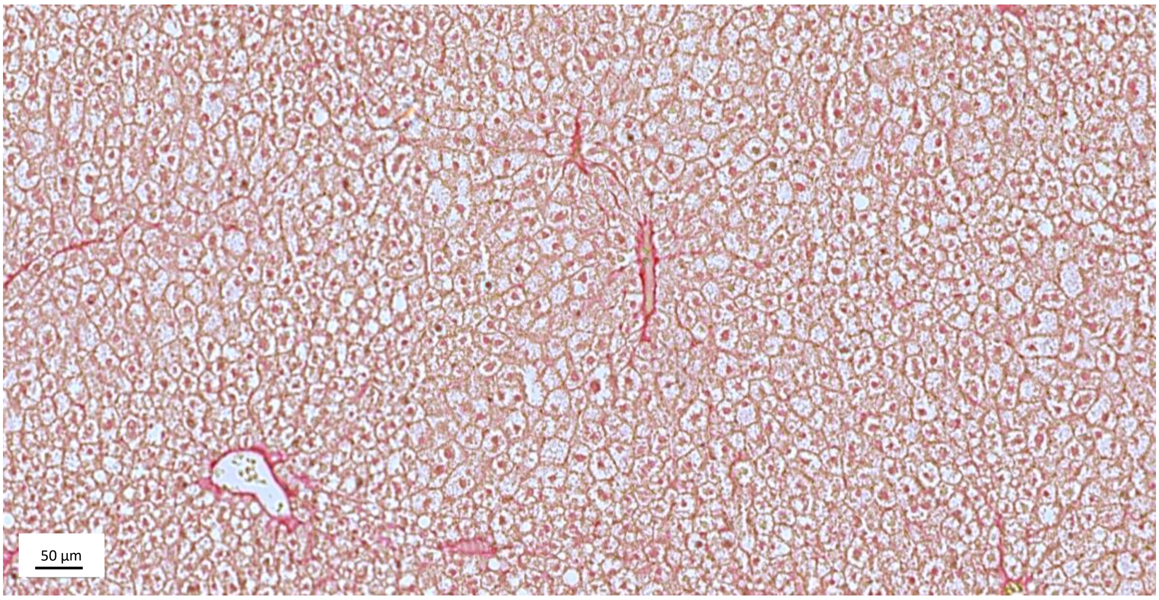

HFHCD-I-12

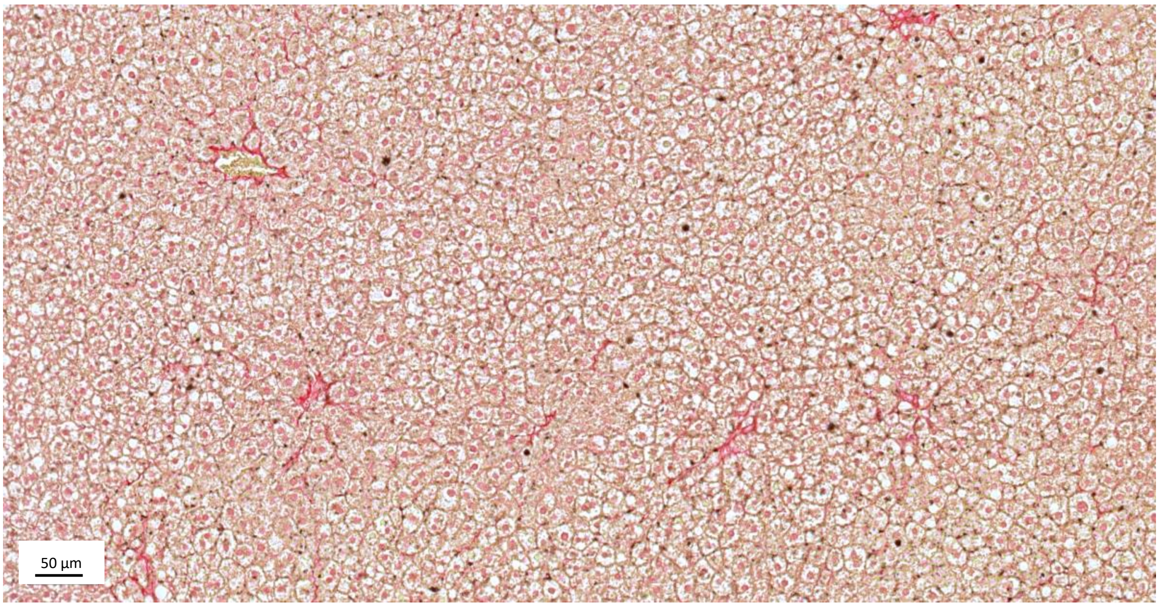

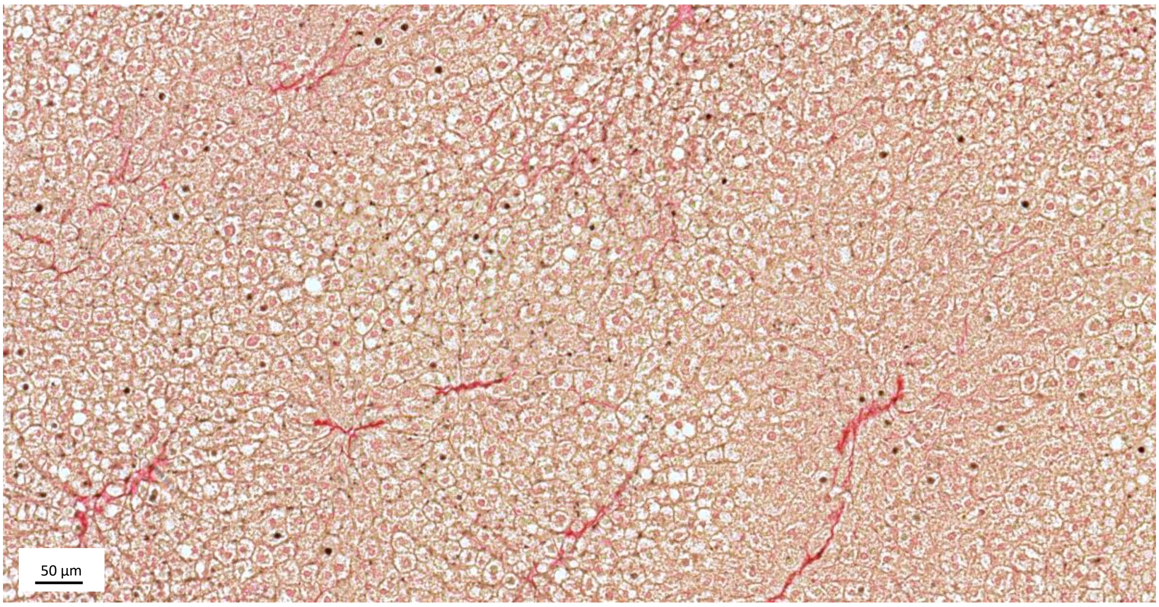

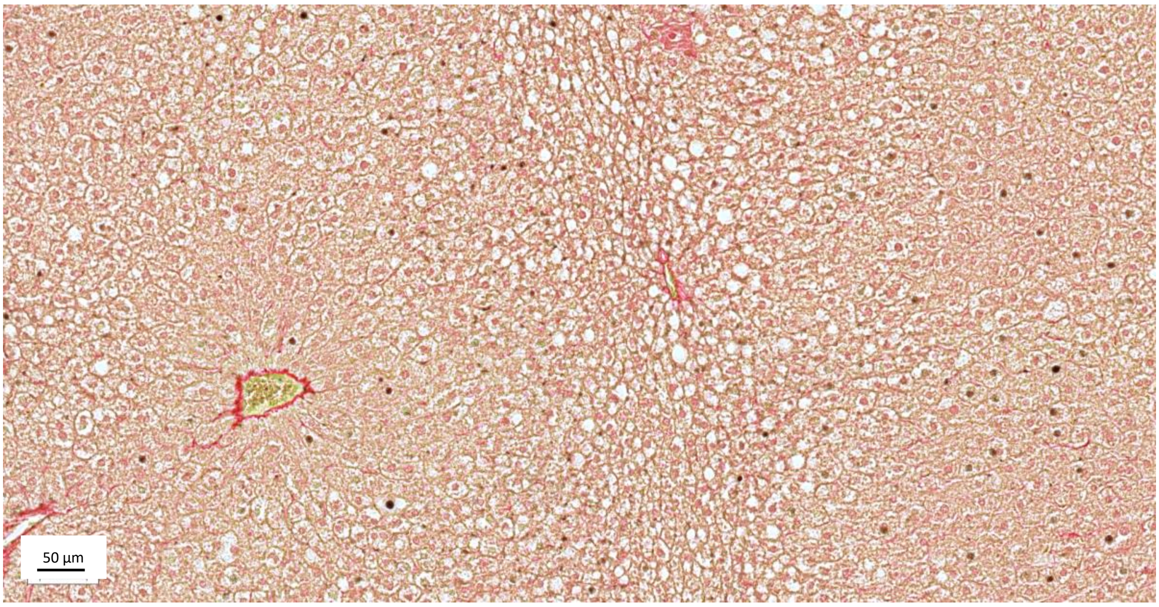

Sirius red Staining

HFHCD-C group

(12 mice were included)

HFHCD-C-1

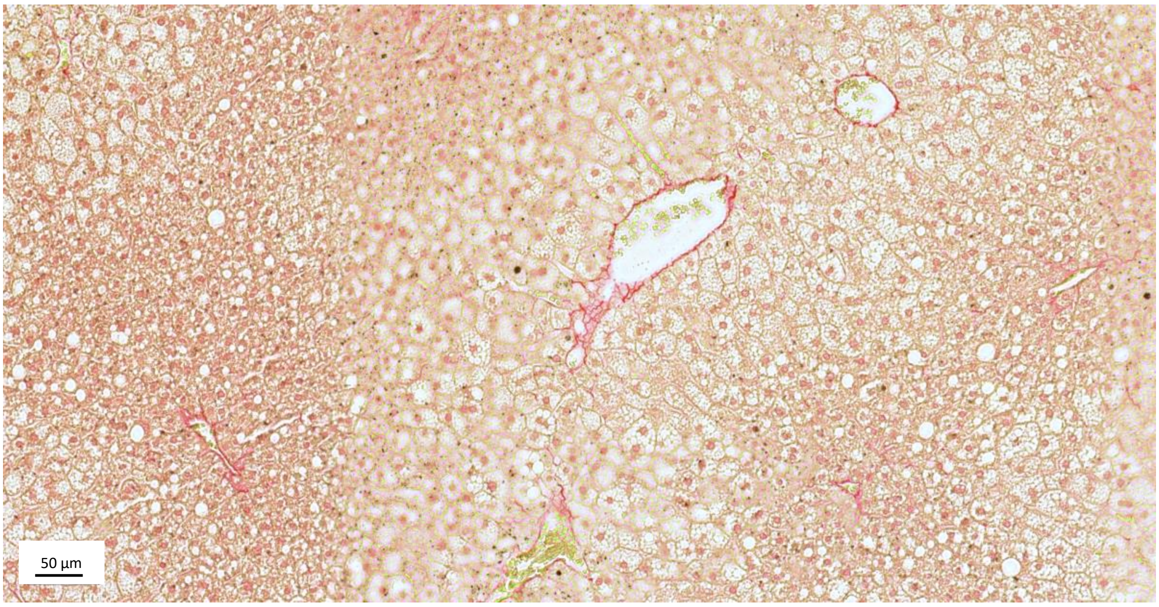

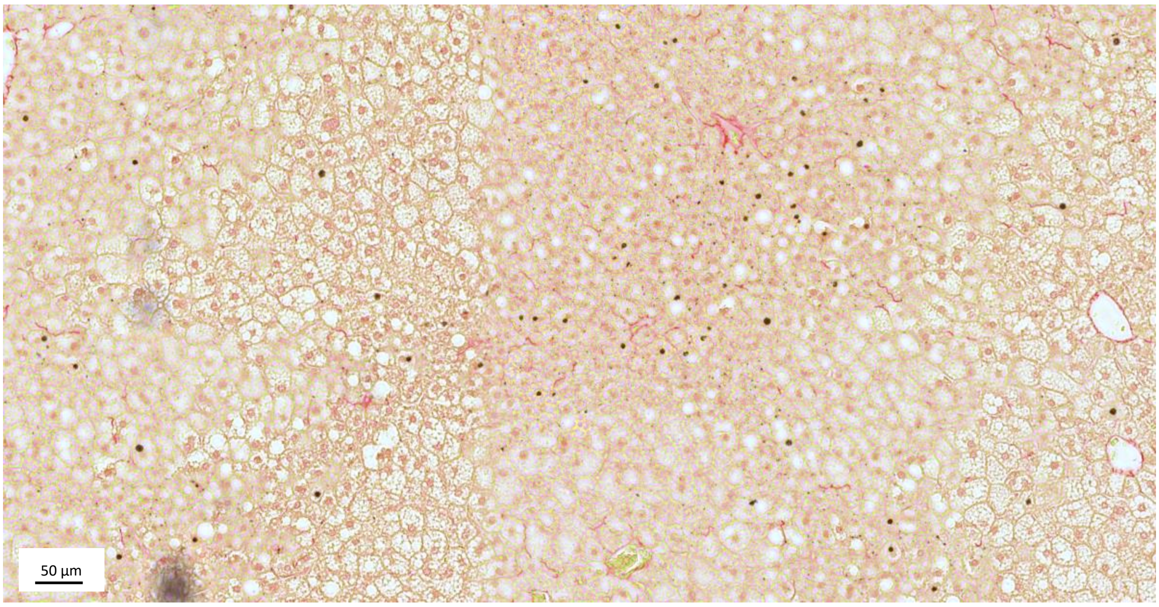

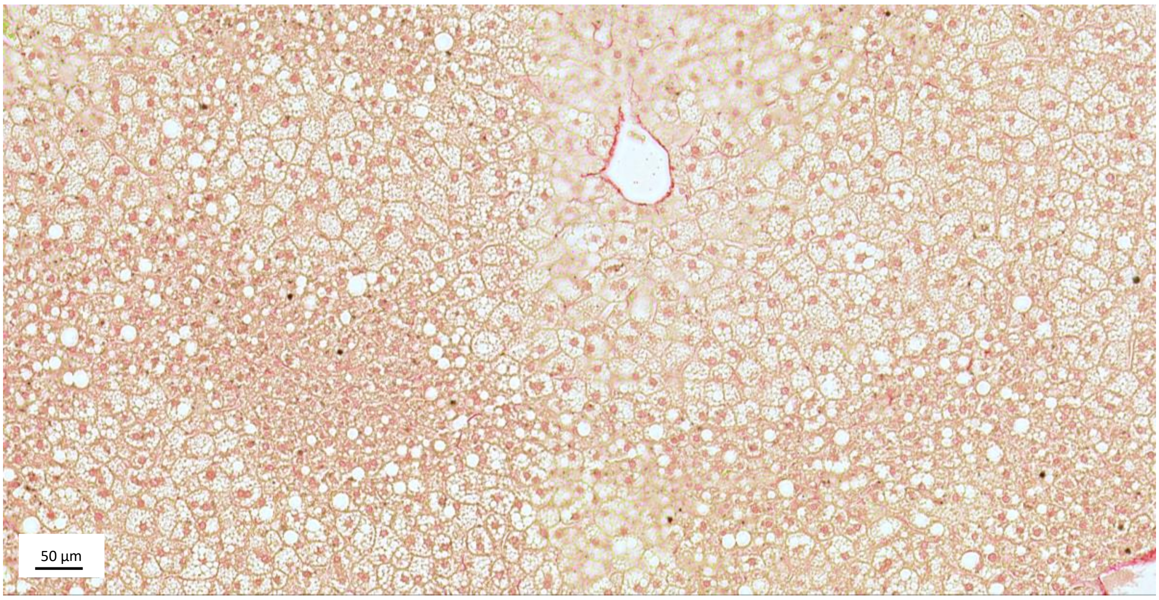

HFHCD-C-2

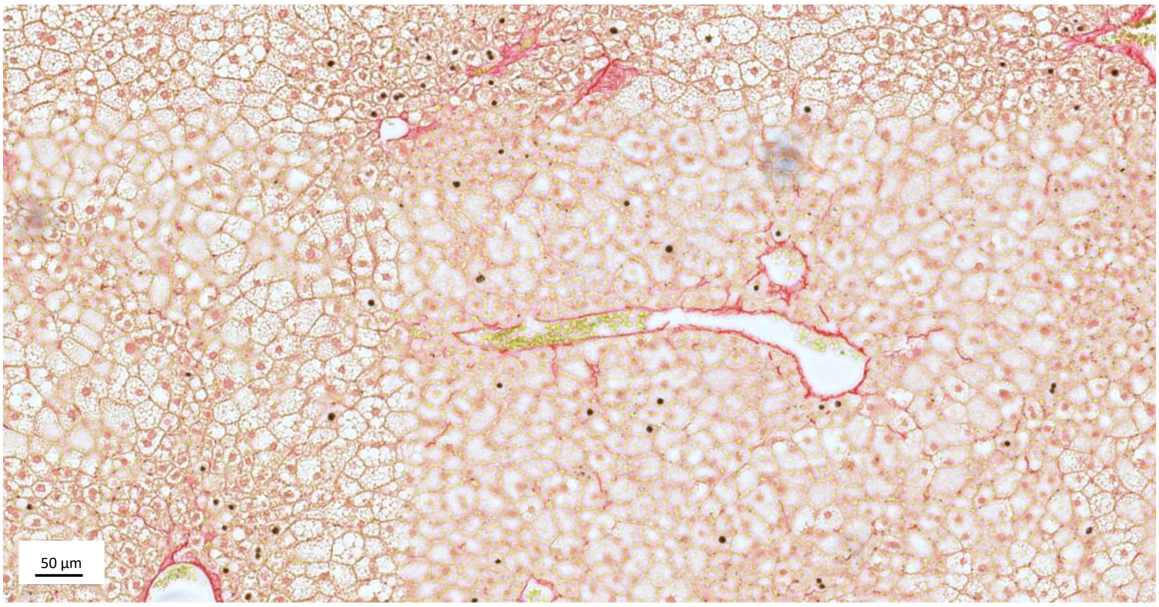

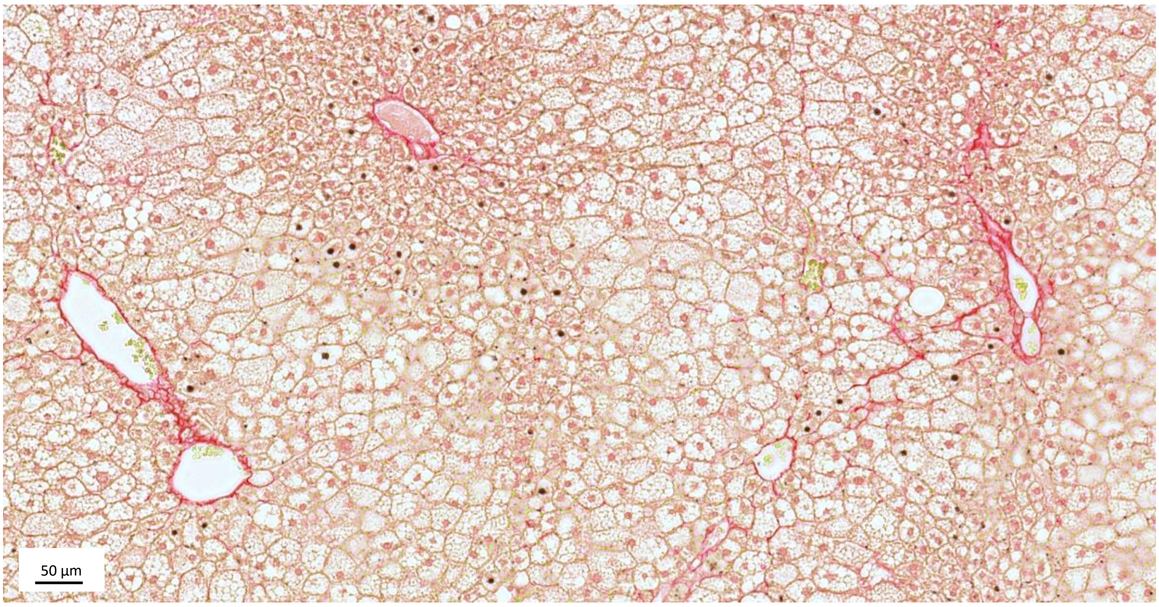

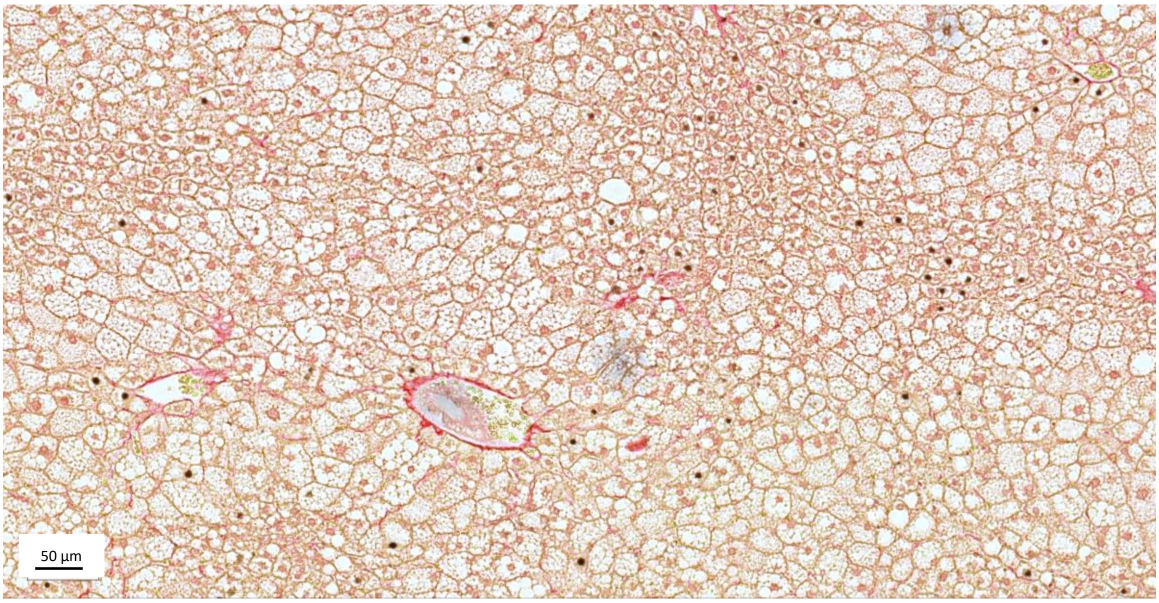

HFHCD-C-3

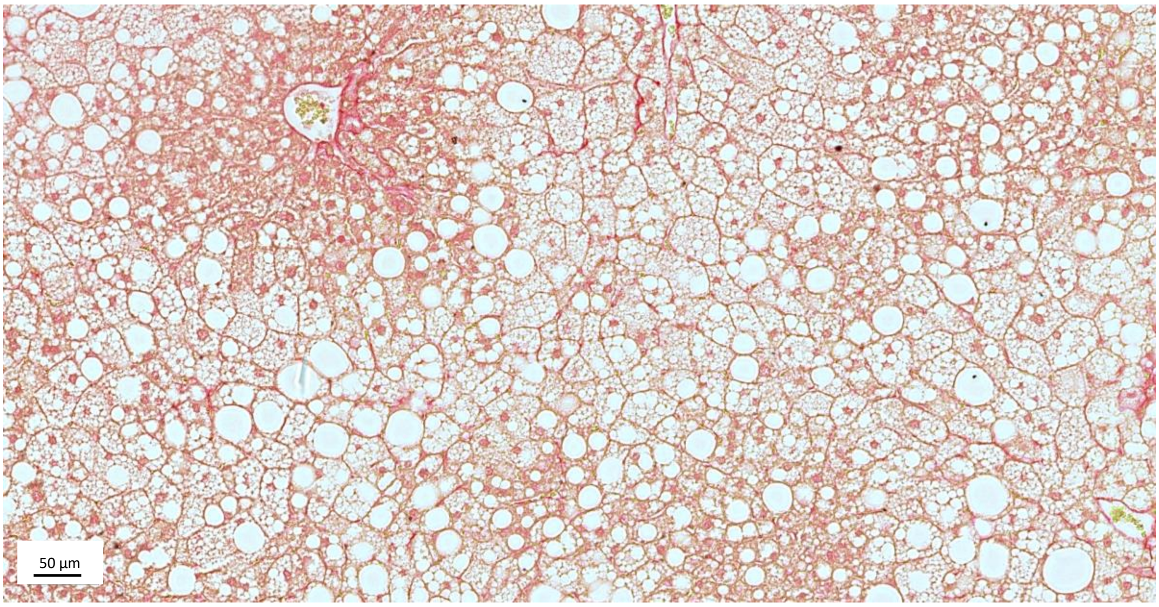

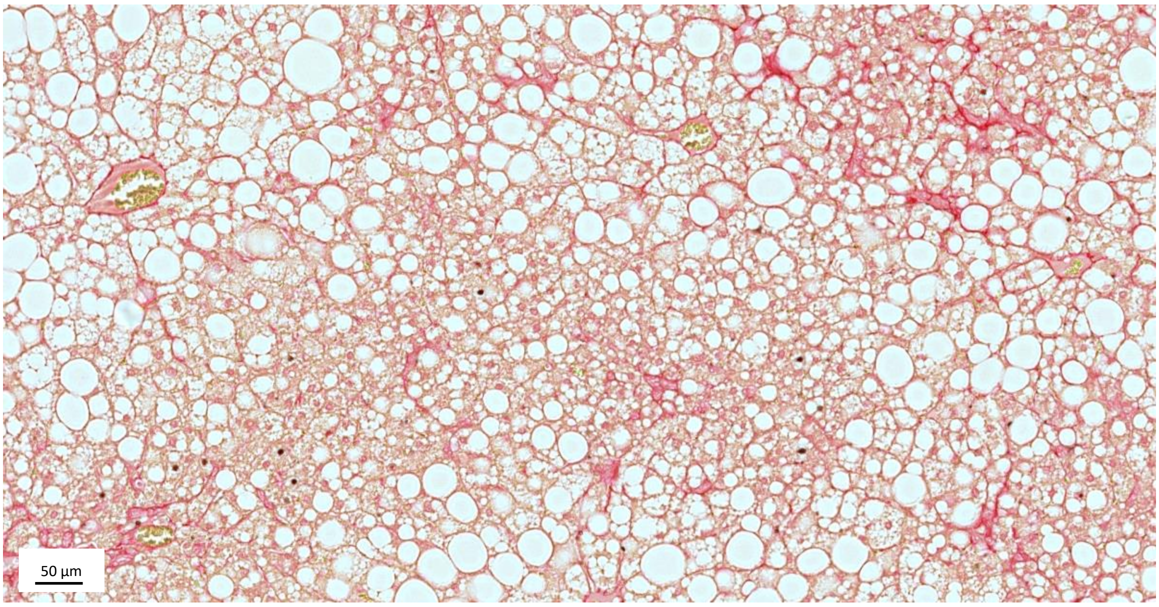

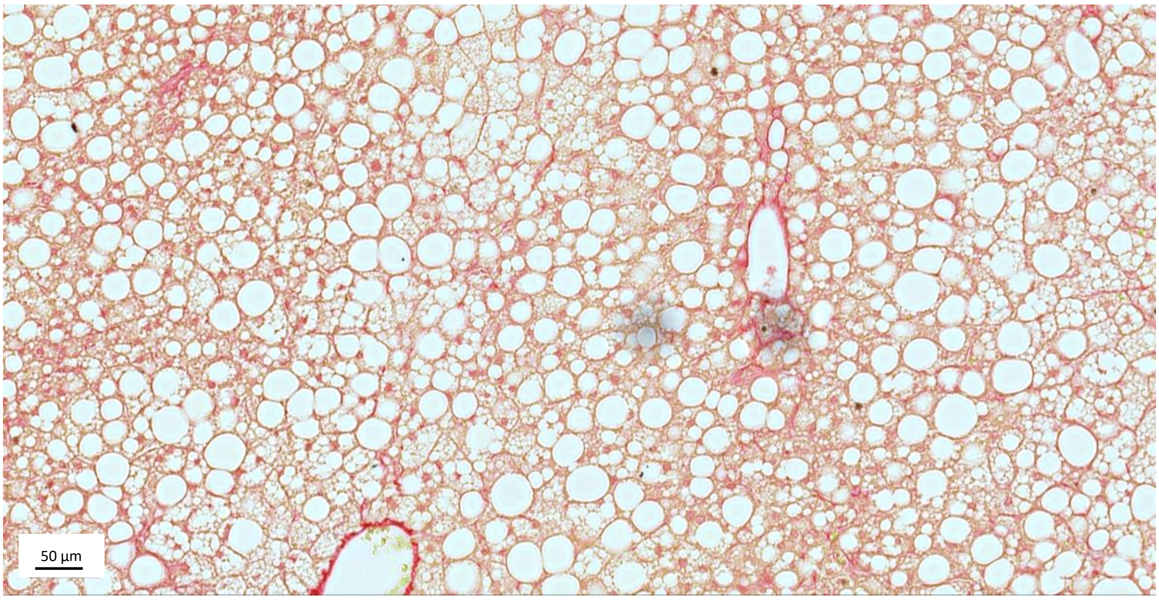

HFHCD-C-4

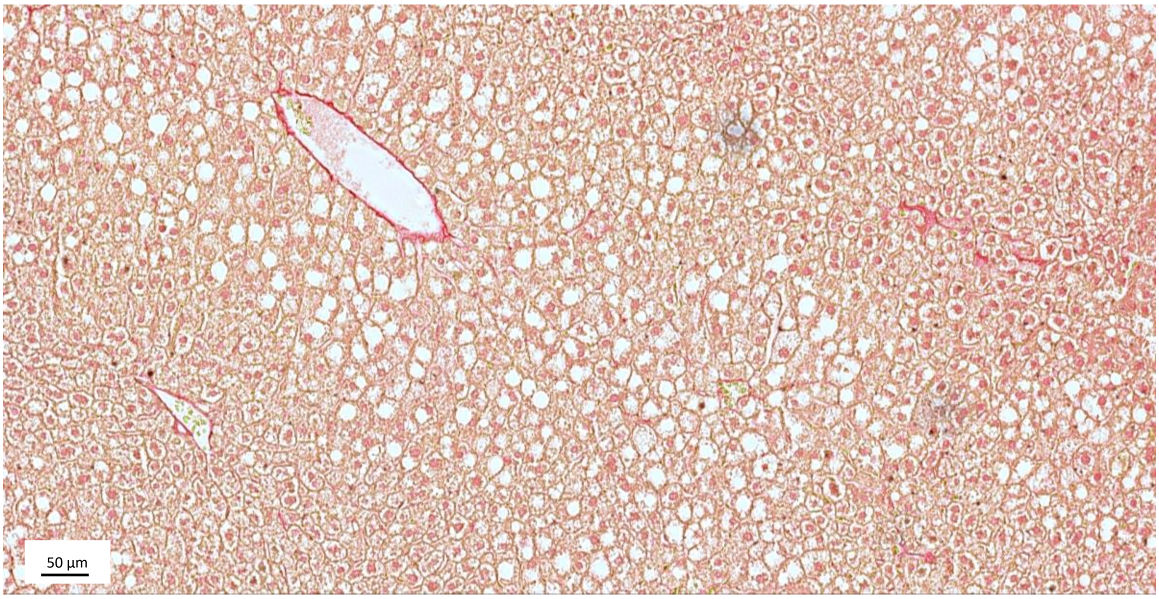

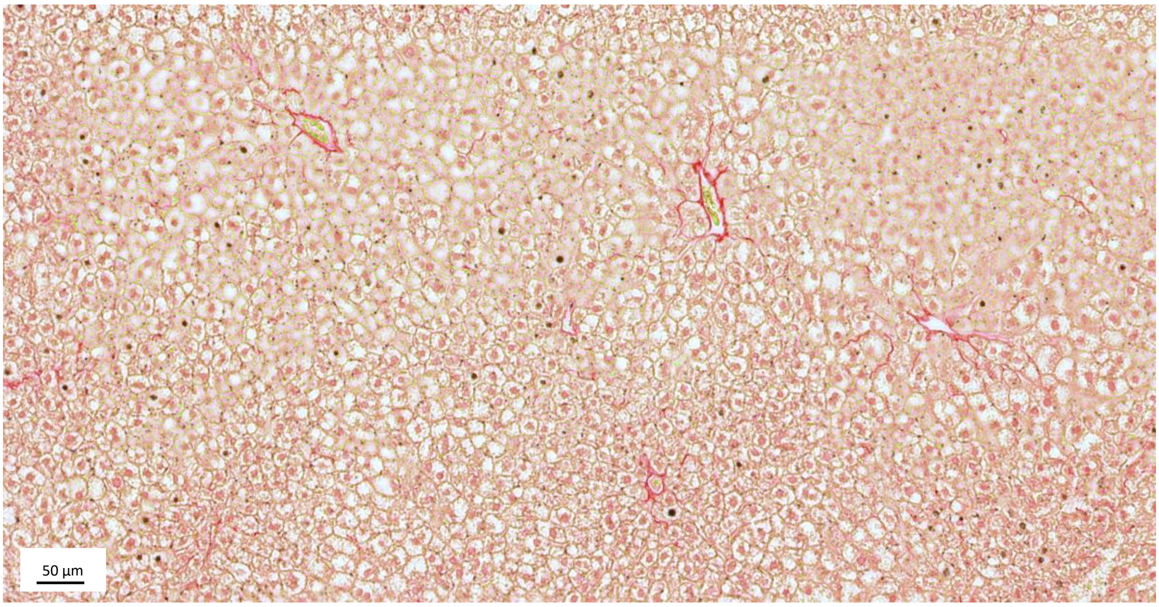

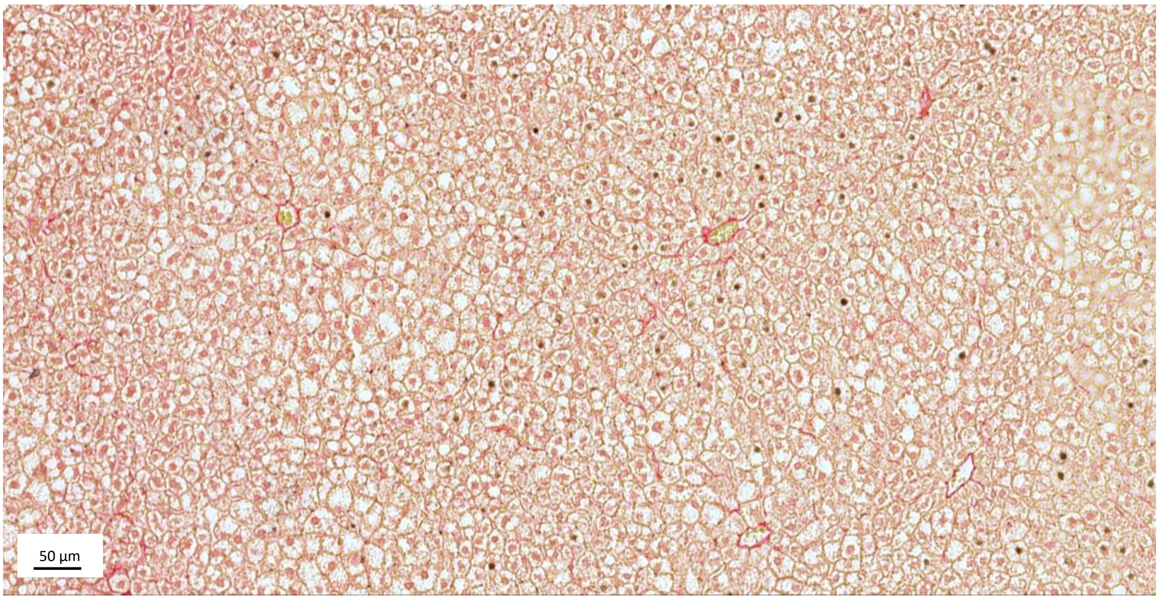

HFHCD-C-5

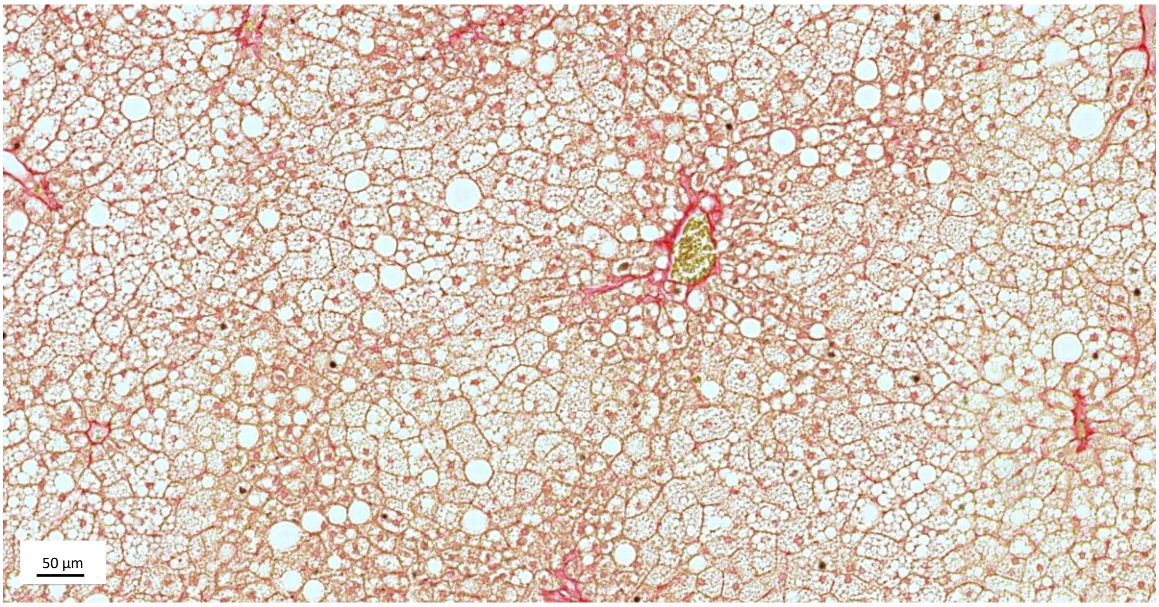

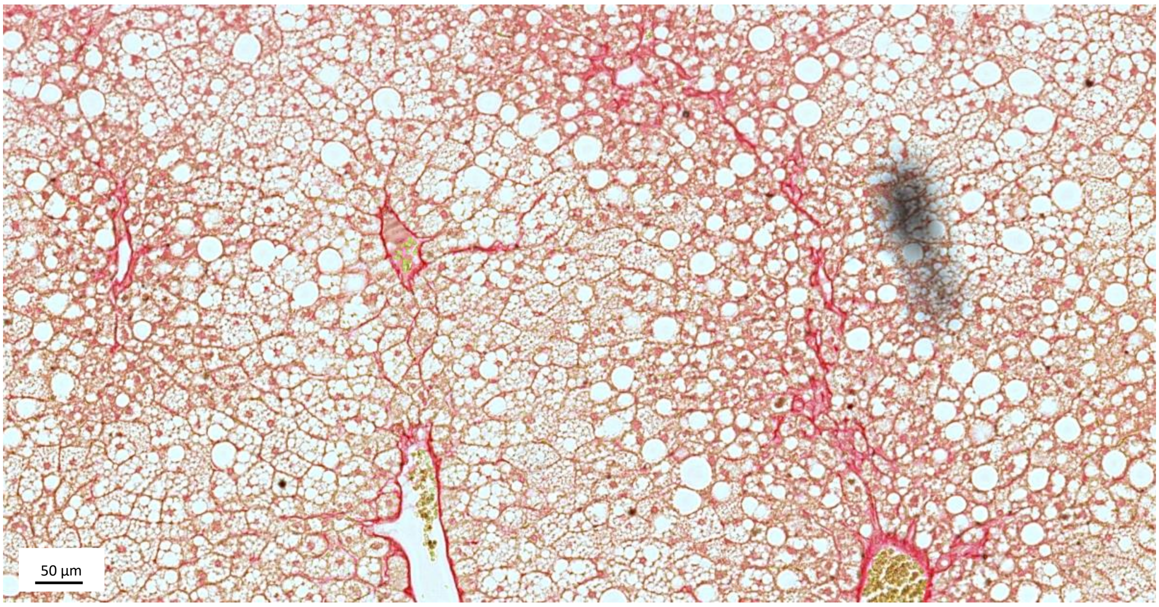

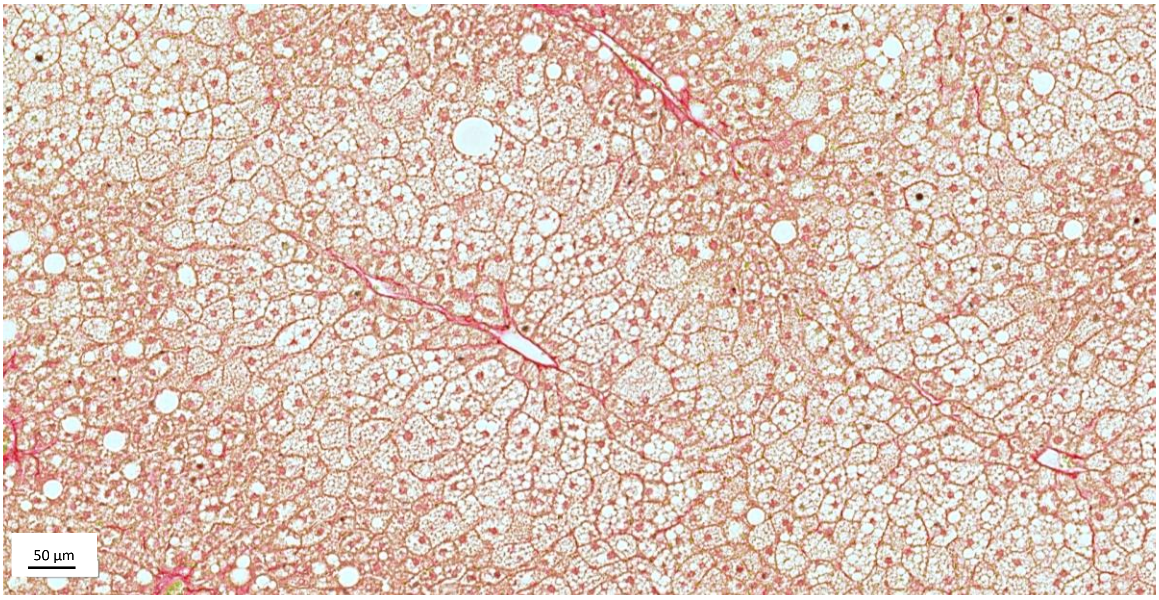

HFHCD-C-6

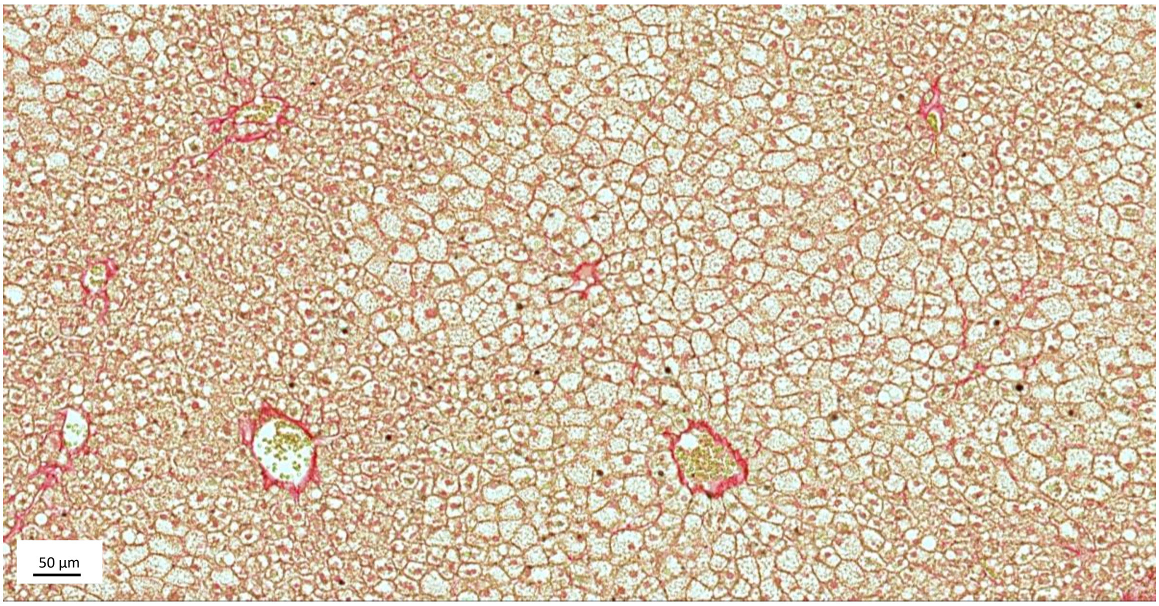

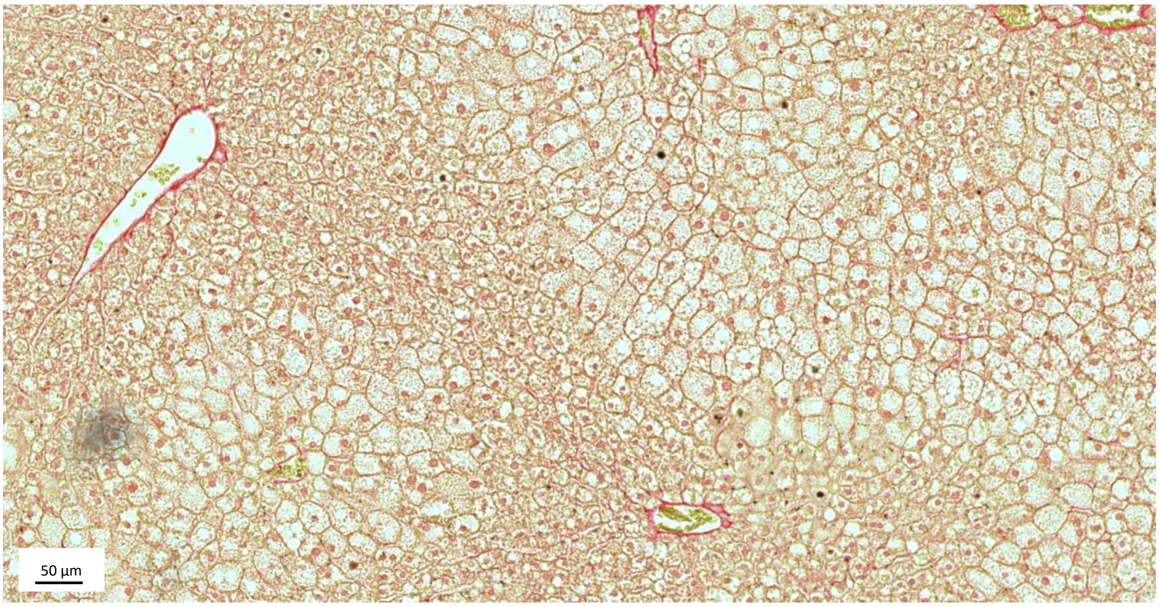

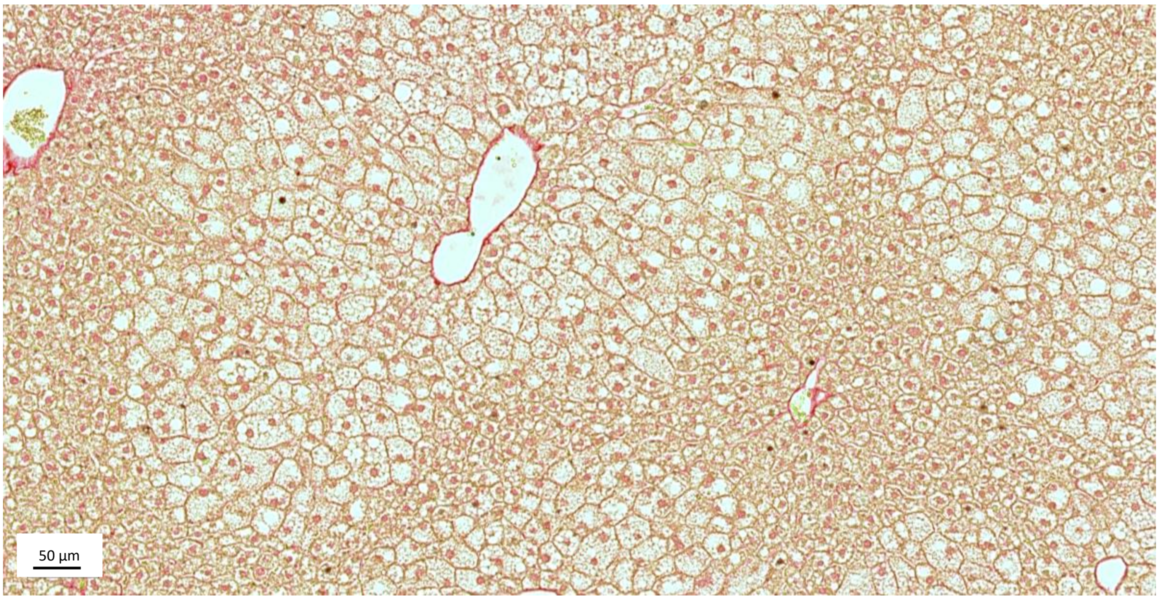

HFHCD-C-7

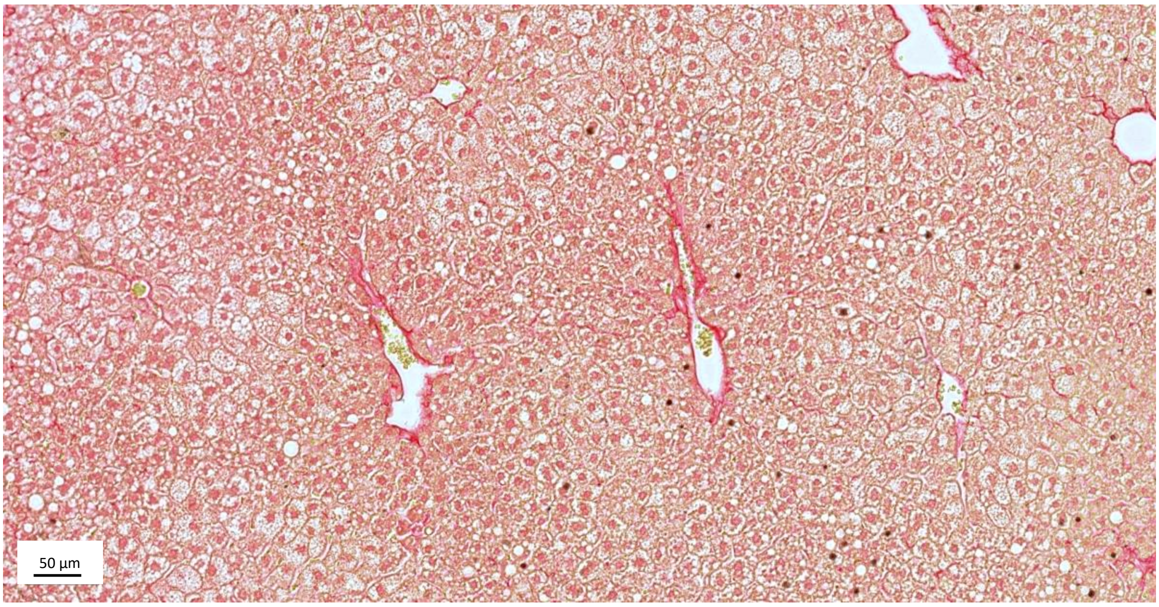

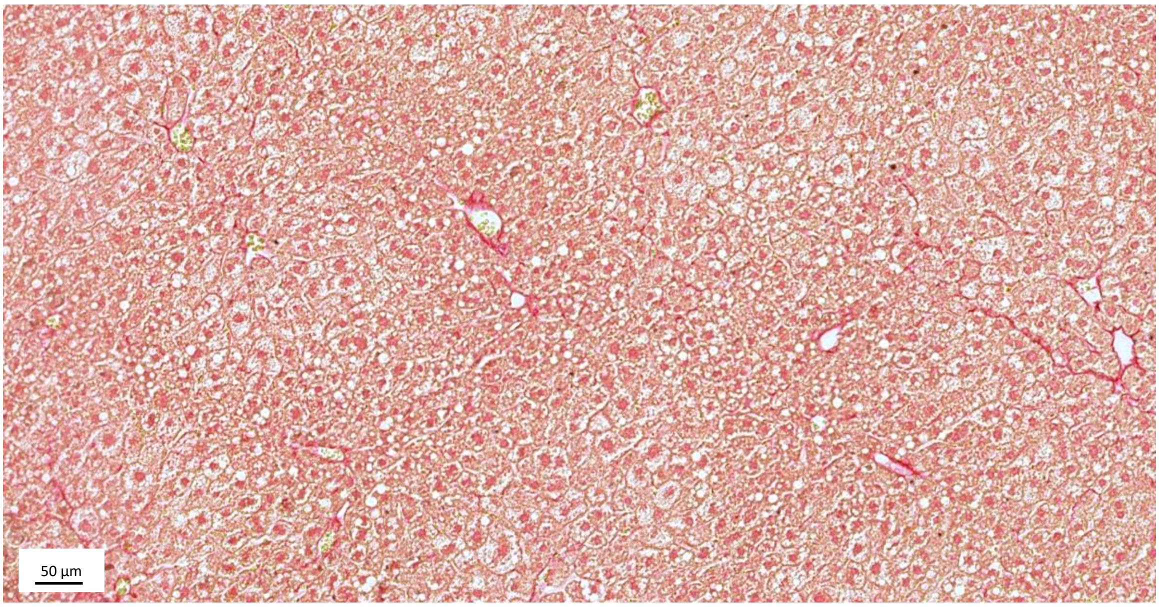

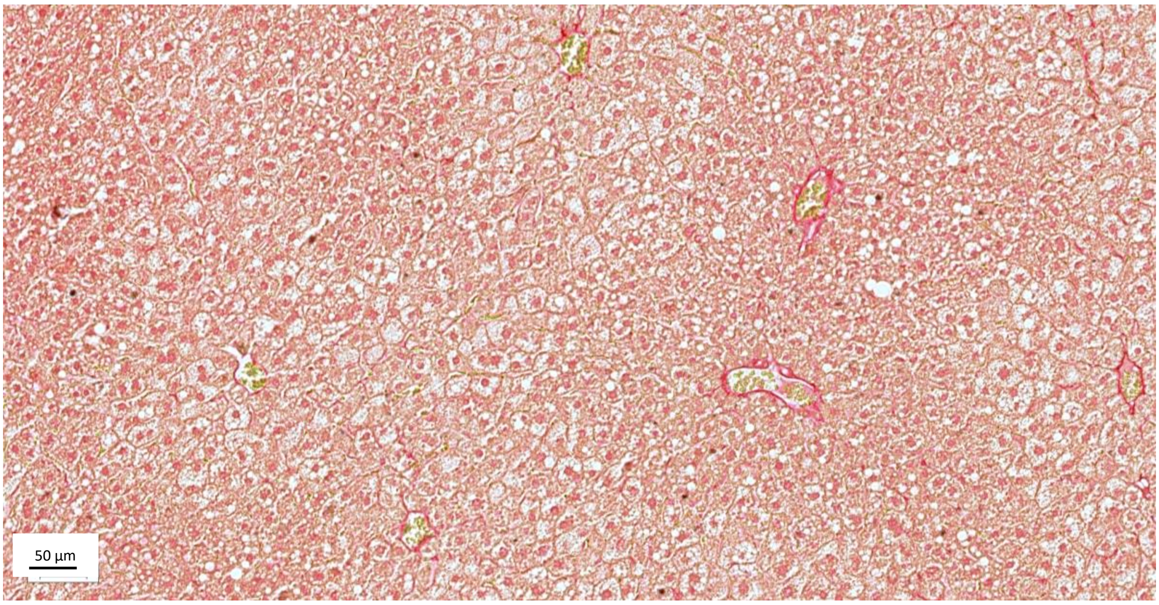

HFHCD-C-8

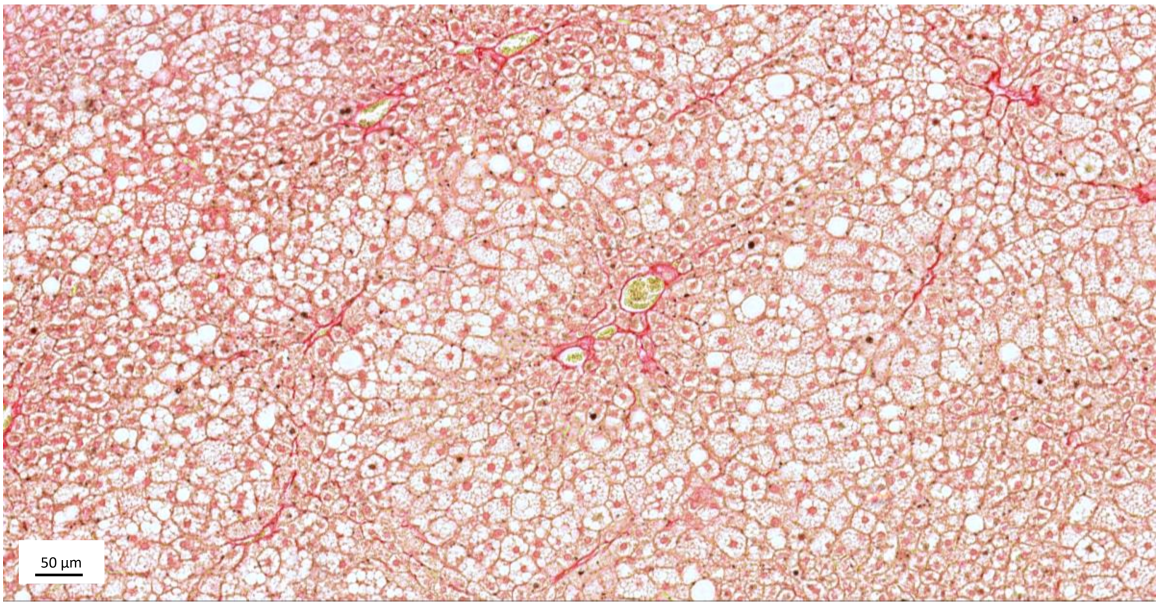

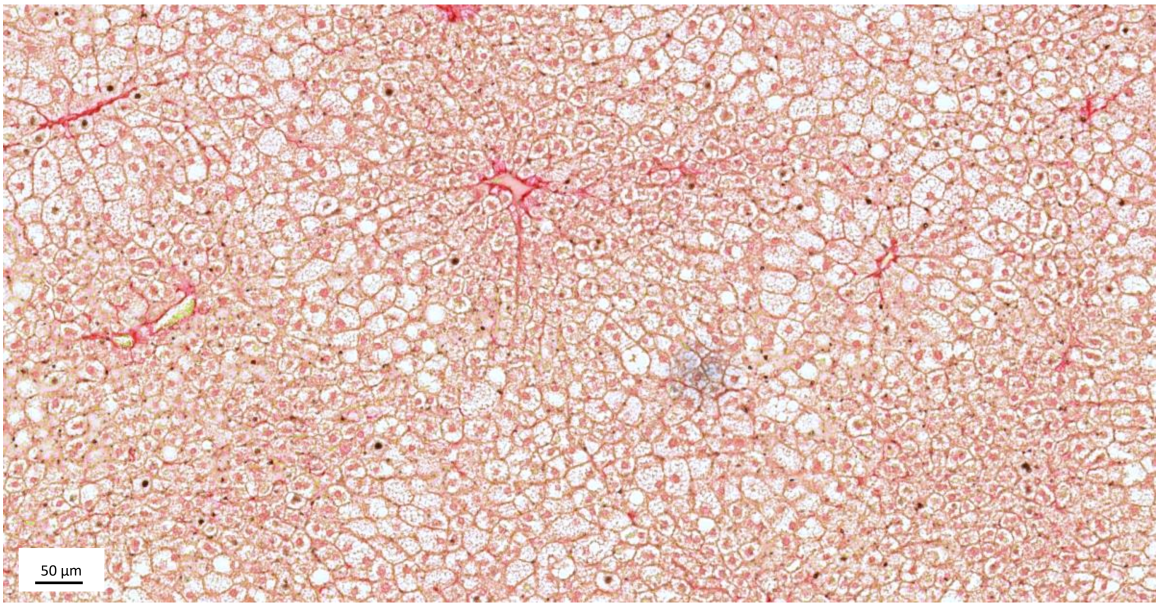

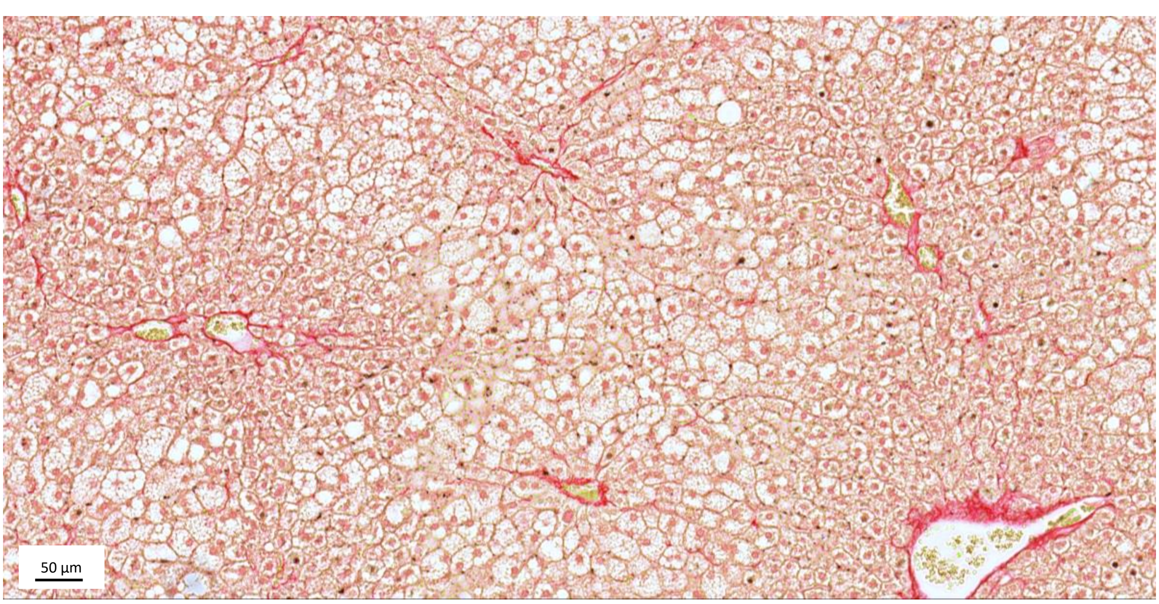

HFHCD-C-9

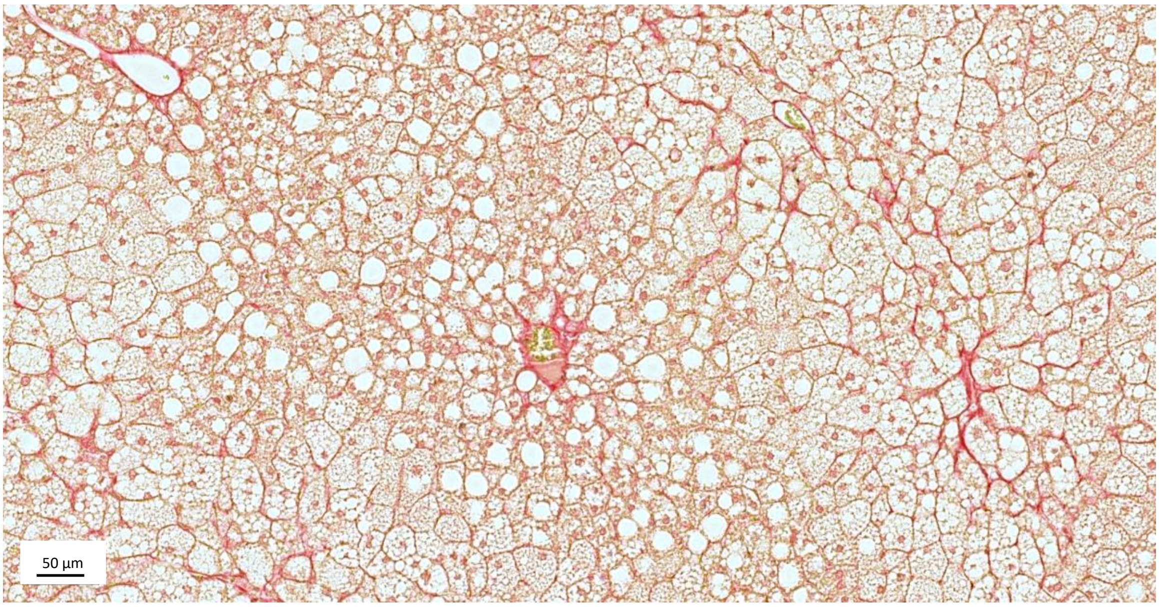

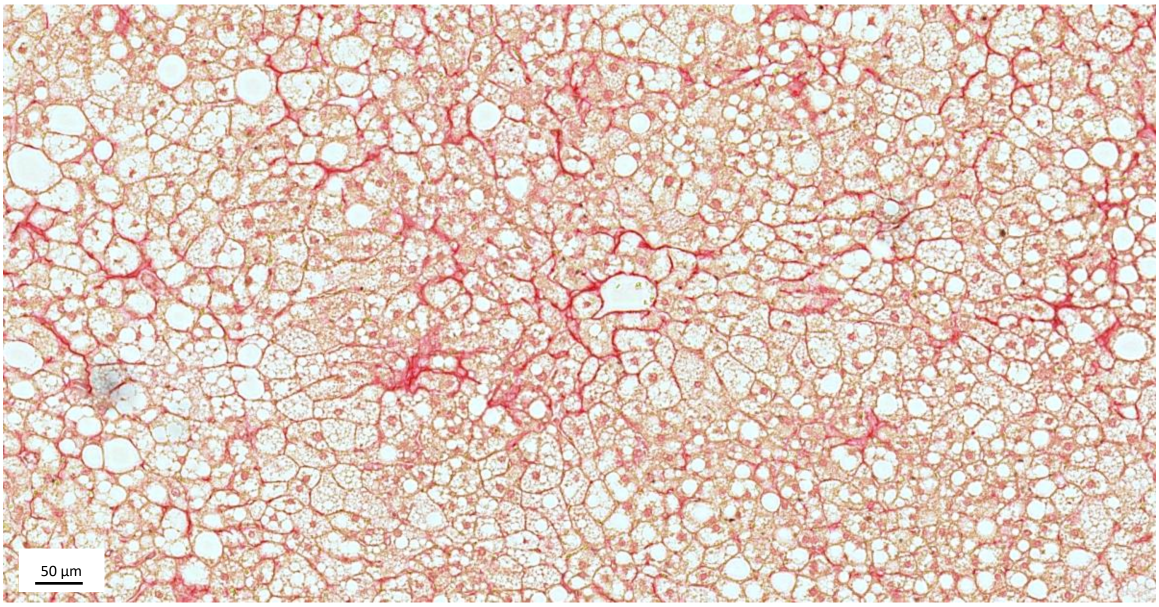

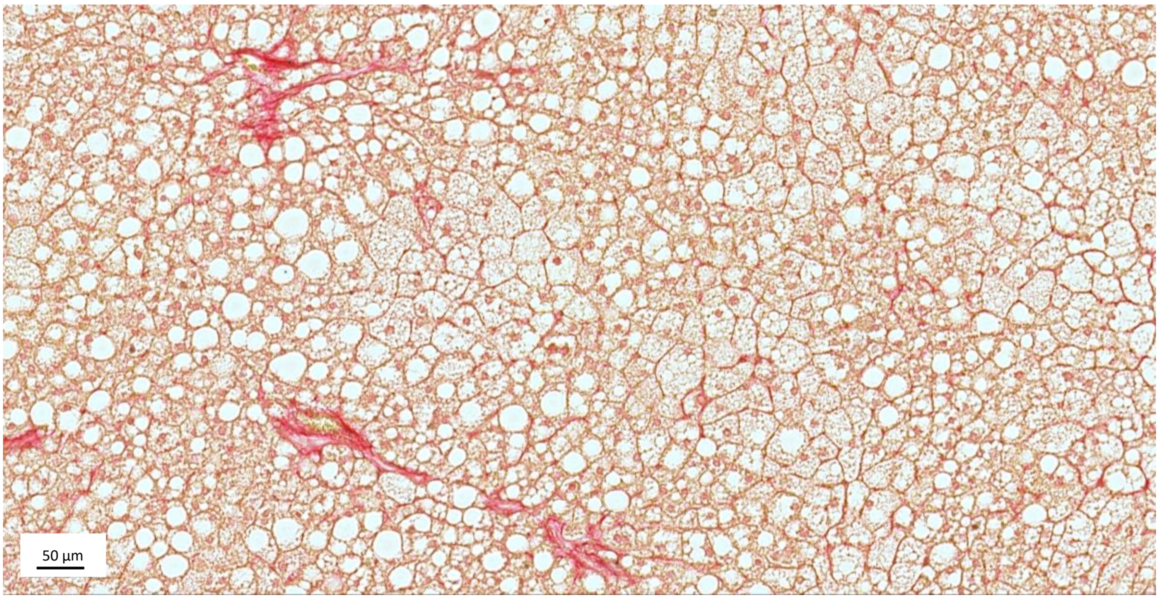

HFHCD-C-10

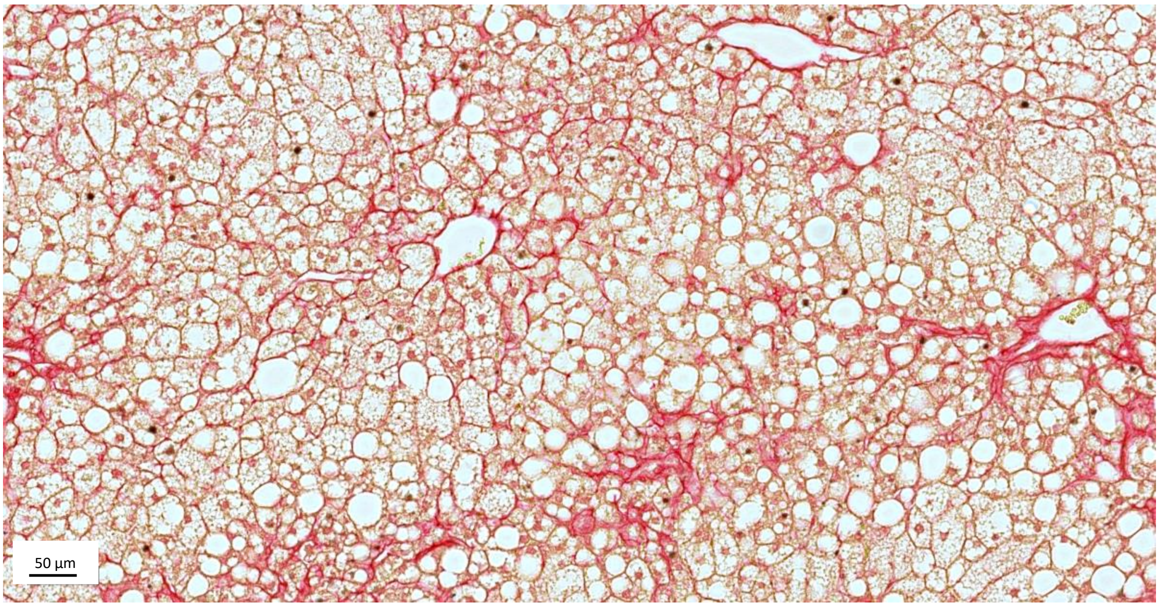

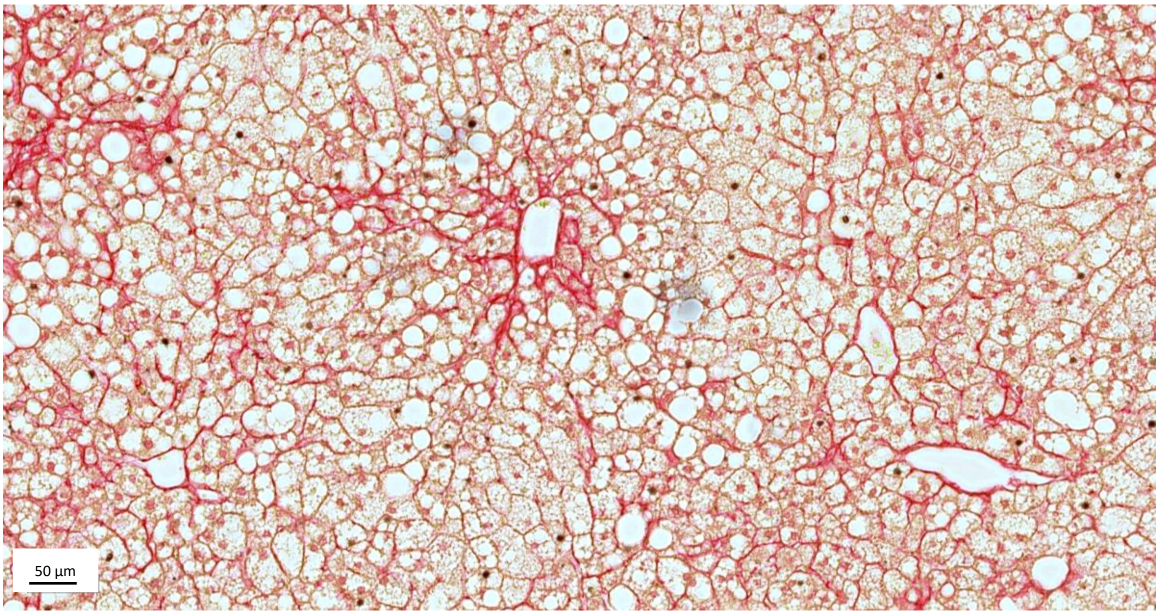

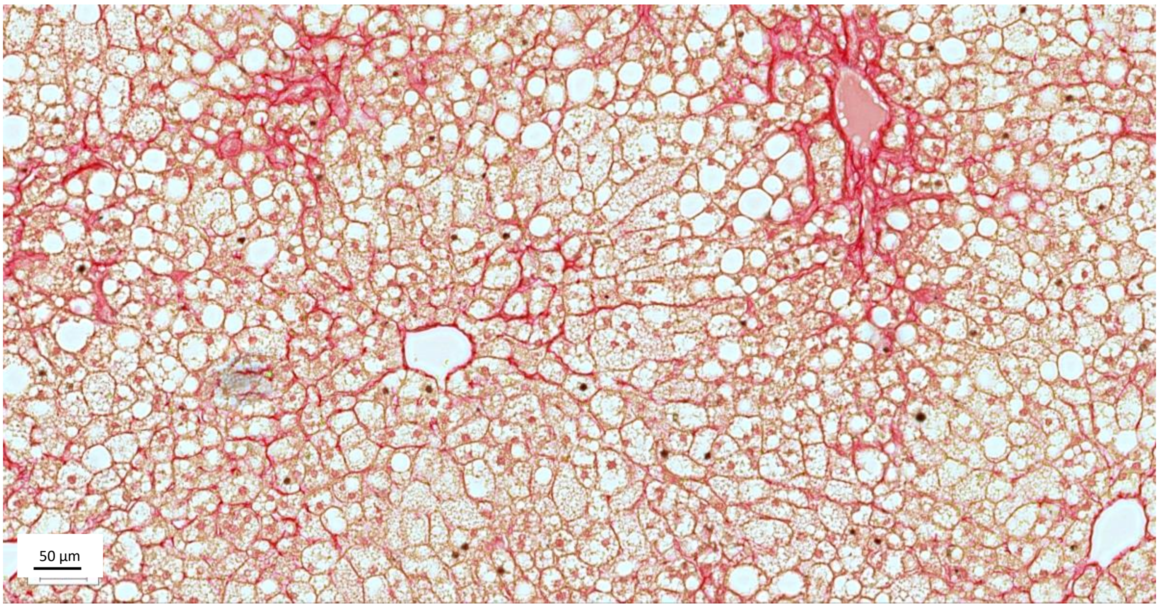

HFHCD-C-11

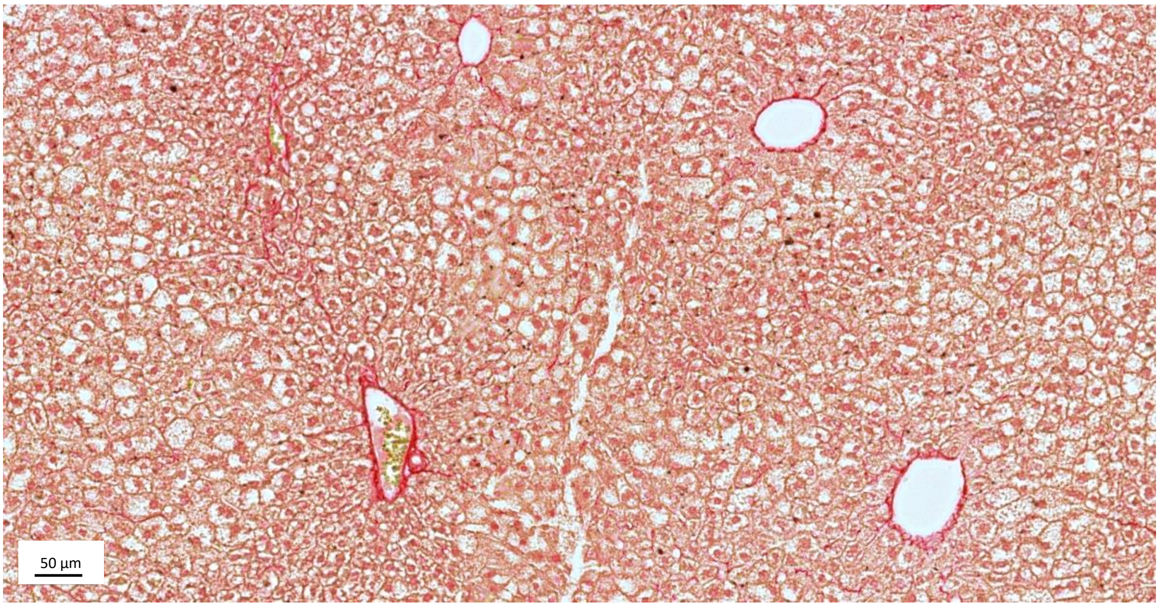

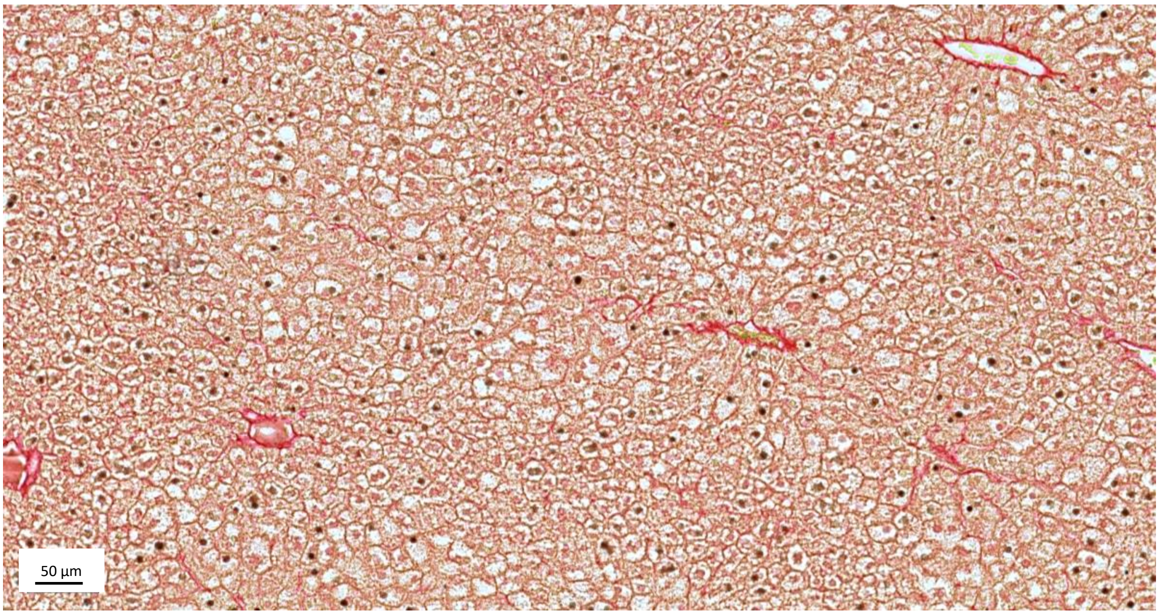

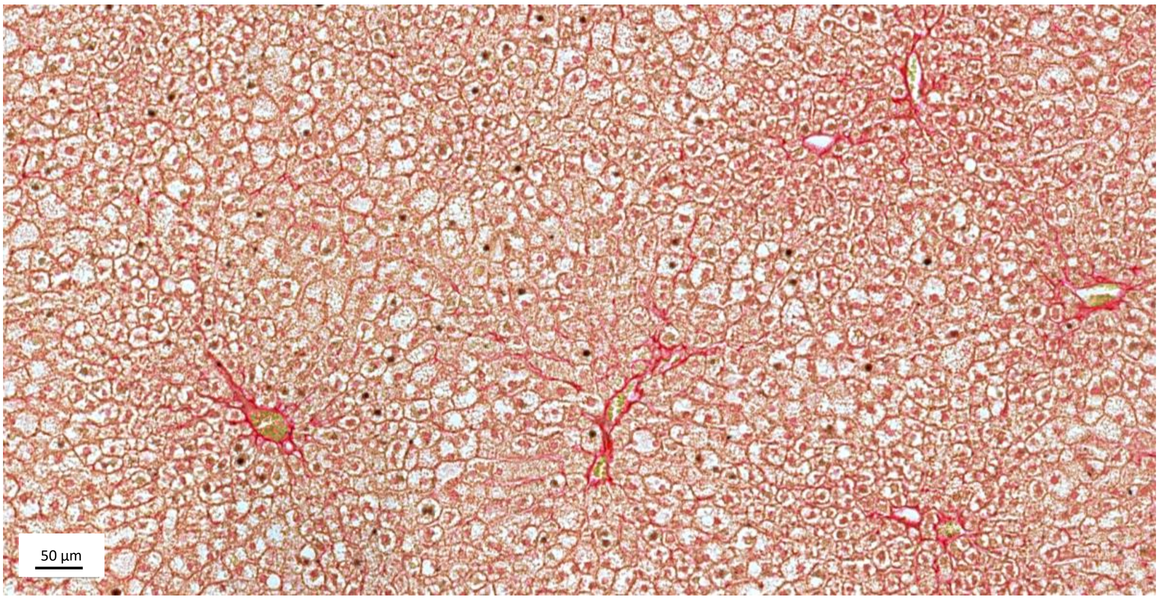

HFHCD-C-12

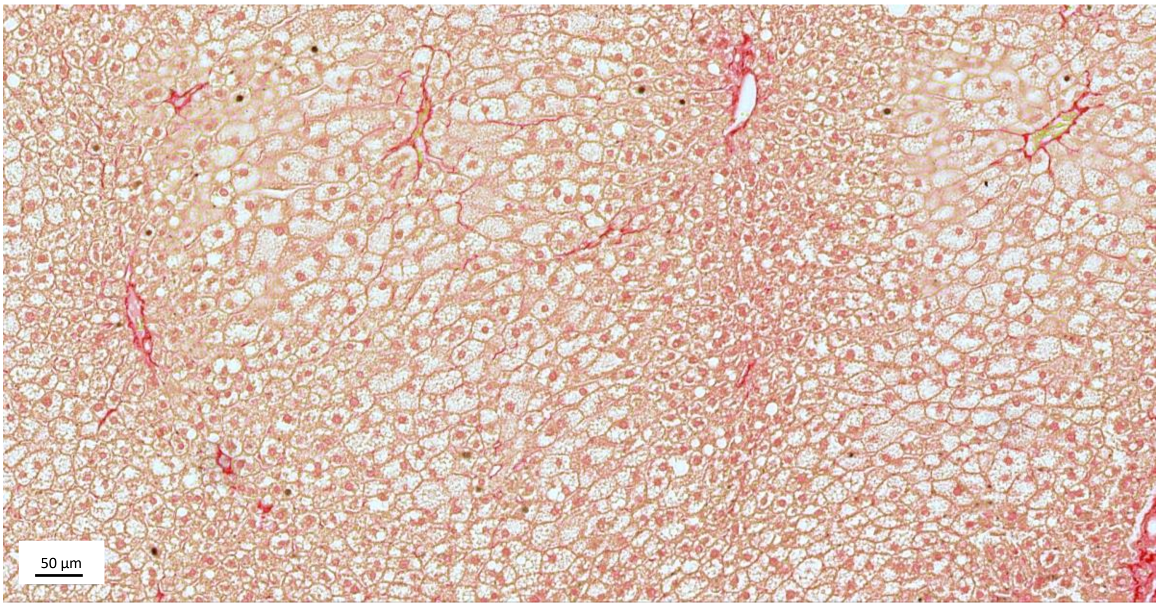

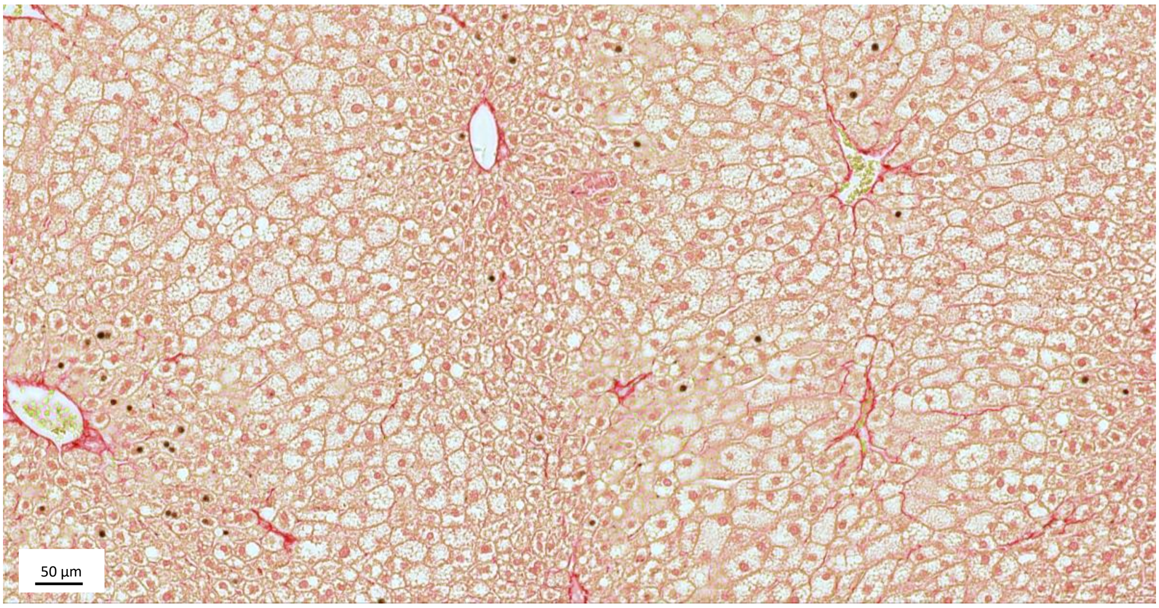

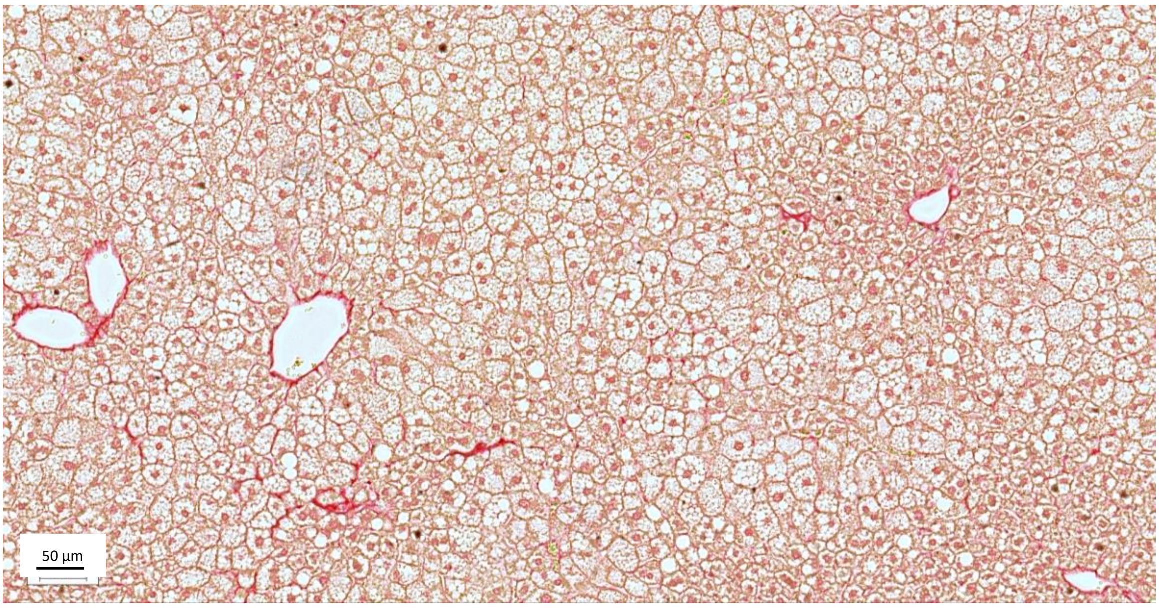

Supplement: Source Data Extended Data Fig. 1 — Replicate histological images. [file 41564_2023_1418_MOESM14_ESM.pdf]
